# Model for Vaccine Design by Prediction of B-Epitopes of IEDB Given Perturbations in Peptide Sequence, In Vivo Process, Experimental Techniques, and Source or Host Organisms

**DOI:** 10.1155/2014/768515

**Published:** 2014-01-12

**Authors:** Humberto González-Díaz, Lázaro G. Pérez-Montoto, Florencio M. Ubeira

**Affiliations:** ^1^Department of Organic Chemistry II, University of the Basque Country UPV/EHU, 48940 Bilbao, Spain; ^2^Ikerbasque, Basque Foundation for Science, 48011 Bilbao, Spain; ^3^Department of Microbiology and Parasitology, University of Santiago de Compostela (USC), 15782 Santiago de Compostela, Spain

## Abstract

Perturbation methods add variation terms to a known experimental solution of one problem to approach a solution for a related problem without known exact solution. One problem of this type in immunology is the prediction of the possible action of epitope of one peptide after a perturbation or variation in the structure of a known peptide and/or other boundary conditions (host organism, biological process, and experimental assay). However, to the best of our knowledge, there are no reports of general-purpose perturbation models to solve this problem. In a recent work, we introduced a new quantitative structure-property relationship theory for the study of perturbations in complex biomolecular systems. In this work, we developed the first model able to classify more than 200,000 cases of perturbations with accuracy, sensitivity, and specificity >90% both in training and validation series. The perturbations include structural changes in >50000 peptides determined in experimental assays with boundary conditions involving >500 source organisms, >50 host organisms, >10 biological process, and >30 experimental techniques. The model may be useful for the prediction of new epitopes or the optimization of known peptides towards computational vaccine design.

## 1. Introduction

National Institute of Allergy and Infectious Diseases (NIAID) supported the launch, in 2004, of the Immune Epitope Database (IEDB), http://www.iedb.org/ [1–4]. The IEDB system withdrew information from approximately 99% of all papers published to date that describe immune epitopes. In doing so, IEDB system analyses over 22 million PubMed abstracts and subsequently curated *≈*13 K references, including *≈*7 K manuscripts about infectious diseases, *≈*1 K about allergy topics, *≈*4 K about autoimmunity, and 1 K about transplant/alloantigen topics [5]. IEDB lists a huge amount of information about the molecular structure as well as the experimental conditions (*c*_*ij*_) in which different *i*th molecules were determined to be immune epitopes or not. This explosion of information makes necessary both query/display functions for retrieval of known data from IEDB as well predictive tools for new epitopes. Salimi et al. [5] reviewed advances in epitope analysis and predictive tools available in the IEDB. In fact, IEDB analysis resource (IEDB-AR: http://tools.iedb.org/) is a collection of tools for prediction of molecular targets of T- and B-cell immune responses (i.e., epitopes) [6, 7].

On the other hand, Quantitative Structure-Activity/Property Relationships (QSAR/QSPR) techniques are useful tool to predict new drugs, RNA, drug-protein complexes, and protein-protein complexes. In general, QSAR/QSPR-like methods transform molecular structures into numeric molecular descriptors (*λ*_*i*_) in a first stage and later fit a model to predict the biological process. For example, DRAGON [8–10], CODESSA [11, 12], MOE [13], TOPS-MODE [14–17], TOMOCOMD [18, 19], and MARCH-INSIDE [20] are among the most used softwares to calculate molecular descriptors based on quantum mechanics (QM) and/or graph theory [21–27]. The software STATISTICA [28] and WEKA [29] are often used to perform multivariate statistics and/or machine learning (ML) analysis in order to preprocess data and later fit the final QSAR/QSPR model using techniques like principal component analysis (PCA), linear discriminant analysis (LDA), support vector machine (SVM), or artificial neural networks (ANN) [28].

QSAR/QSPR models are also important in immunoinformatics to predict the propensity of different molecular structures to play different roles in immunological processes. They include skin vaccine adjuvants and sensitizers [30–38], drugs and their activity/toxicity protein targets in the immune system [39], and epitopes [40–49]. Moreover, Reche and Reinherz [50] implemented PEPVAC (promiscuous epitope-based vaccine), a web server for the formulation of multiepitope vaccines that predict peptides binding to five distinct HLA class I supertypes (A2, A3, B7, A24, and B15). PEPVAC can also identify conserved MHC ligands, as well as those with a C-terminus resulting from proteasomal cleavage. The Dana-Farber Cancer Institute hosted the PEPVAC server at the site http://immunax.dfci.harvard.edu/PEPVAC/. To close with a last example, Lafuente and Reche [51] reviewed the available methods for predicting MHC-peptide binding and discussed their most relevant advantages and drawbacks.

In many complex QSPR-like problems in immunoinformatics, like in other areas, we know the exact experimental result (known solution) of the problem, but we are interested in the possible result obtained after a change (perturbation) on one or multiple values of the initial conditions of the experiment (new solution). For instance, we often know, for large collections of *i*th molecules (*m*_*i*_), organic compounds, drugs, xenobiotics, and/or peptide sequences, the efficiency of the compound *ε*(*c*_*ij*_) as adjuvant, action as epitope, immunotoxicity, and/or the interaction (affinity, inhibition, etc.) with immunological targets. In addition, we often known for each molecule the exact conditions (*c*_*ij*_) of assay for the initial experiment including structure of the molecule *m*_*i*_ (drug, adjuvant, and sequence of the peptide), source organism (so), host organism (ho), immunological process (ip), experimental technique (tq), concentration, temperature, time, solvents, and coadjuvants. This is the case of big data retrieved from very large databases like IEDB [1–4] and CHEMBL [52]. However, we do not know the possible result of the experiment if we change at least one of these conditions (perturbation). We refer to small changes or perturbations in both structure and condition for input or output variables. It means that we include changes in ho, so, ip, and tq, changes of the compound by one analogue compound with similar structure, changes in the sequence of the epitope (artificial by organic synthesis or natural mutations), and polarity of the solvent or coadjuvants. In these cases, we could use a perturbation theory model to solve the QSAR/QSPR problem. Perturbation theory includes methods that add “small” terms to a known solution of a problem in order to approach a solution to a related problem without known solution. Perturbation models have been widely used in all branches of science from QM to astronomy and life sciences including chaos or “butterfly effect,” Bohr's atomic theory, Heisenberg's mechanics, Zeeman's and Stark's effects, and other models with applications in like protein spectroscopy and others [53–57]. In a very recent work Gonzalez-Diaz et al. [58] formulated a general-purpose perturbation theory or model for multiple-boundary QSPR/QSAR problems. However, there is not report in the immunoinformatics literature of a general QSPR perturbation model for IEDB B-epitopes. Here we report the first example of QSPR-perturbation model for B-epitopes reported in IEDB able to predict the probability of occurrence of an epitope after a perturbation in the sequence, the experimental technique, the exposition process, and/or the source or host organisms.

## 2. Materials and Methods

### 2.1. Molecular Descriptors for Peptides

We calculated the molecular descriptors of the structure of peptides using the software MARCH-INSIDE (MI) based on the algorithm with the same name [59]. The MI approach uses a Markov Chain method to calculate the *k*th mean values of different physicochemical molecular properties *λ*(*m*_*i*_) for *i*th molecules (*m*). These *λ*(*m*_*i*_) values are calculated as an average of ^*k*^ *λ*(*m*_*i*_) values for all atoms placed at topological distance *d* ≤ *k*; which are in turn the means of atomic properties (*λ*_*j*_) for all atoms in the molecule and its neighbors placed at *d* = *k*. For instance, it is possible to derive average estimations of molecular refractivities ^*k*^MR(*m*_*i*_), partition coefficients ^*k*^ *P*(*m*_*i*_), and hardness ^*k*^ *η*(*m*_*i*_) for atoms placed at different topological distances *d* ≤ *k*. In this first work, we calculated only one type of *λ*(*m*_*i*_) values. We calculated for all peptides the average value *χ*(*m*_*i*_) of all the atomic electronegativities *χ*_*i*_ for all *δ*_*i*_ atoms connected to the *i*th atom (*i* → *j*) and their neighbors placed at a distance *d* ≤ 5 [59]:

(1) χ(mi)=16∑k=05χjk=16∑k=05∑i→jδipk(χj)·χj.

We calculate the probabilities ^*k*^ *p*(*λ*_*j*_) for any atomic property including ^*k*^ *p*(*χ*_*j*_) using a Markov Chain model for the gradual effects of the neighboring atoms at different distances in the molecular backbone. This method has been explained in detail in many previous works so we omit the details here [59].

### 2.2. Electronegativity Perturbation Model for Prediction of B-Epitopes

Very recently Gonzalez-Diaz et al. [58] formulated a general-purpose perturbation theory or model for multiple-boundary QSPR/QSAR problems. We adapted here this new theory or modeling method to approach to the peptide prediction problem from the point of view of perturbation theory. Let be a set of *i*th peptide molecules denoted as *m*_*i*_ with a value of efficiency *ε*_*ij*_ as epitopes experimentally determined under a set of boundary conditions *c*_*j*_ ≡ (*c*_0_, *c*_1_, *c*_2_, *c*_3_,…, *c*_*n*_). We put the main emphasis here on peptides reported in the database IEDB. In this sense, the boundary conditions *c*_*j*_ used here are the same reported in this database, *c*_0_ = is the specific peptide, *c*_1_ = so_*j*_, *c*_2_ = ho_*j*_, *c*_3_ = ip_*j*_, and *c*_4_ = tq_*j*_. In general, so is the organism that expresses the peptide (but it can include also artificial peptides, cellular lines, etc.), ho is the host organism exposed to the peptide by means of the bp detected with tq. As our analysis, based on the data reported by IEDB we are unable to work with continuous values of epitope activity *ε*_*ij*_. Consequently, we have to predict the discrete function of B-epitope efficiency *λ*(*ε*_*ij*_) = 1 for epitopes reported in the conditions *c*_*j*_ and *λ*(*ε*_*ij*_) = 0, otherwise. Our main aim is to predict the shift or change in a function of the output efficiency Δ*λ*(*ε*_*ij*_) = *λ*(*ε*_*ij*_)_ref_ − *λ*(*ε*_*ij*_)_new_ that takes place after a change, variation, or perturbation (Δ*V*) in the structure and/or boundary conditions of a peptide of reference. But we know the efficiency of the process of reference *λ*(*ε*_*ij*_)_ref_ in addition to the molecular structure and the set of conditions *c*_*j*_ for initial (reference) and final processes (new). Consequently, to predict Δ*λ*(*ε*_*ij*_) we have to predict only *λ*(*ε*_*ij*_)_new_ the efficiency function of the new state obtained by a change in the structure of the peptide and/or the boundary conditions. Let Δ*V* be a perturbation in a function *λ*; we can define *V*_*ij*_ as the state information function for the reference and new states. According to our recent model [58], we can write *V*_*ij*_ as a function of the conditions and structure of the peptide *m*_*i*_ as follows. In fact, the variational state functions *V*_*ij*_ have to be written in pairs in order to describe the initial (reference) and final (new) states of a perturbation, as follow:

(2) $$\begin{array}{c} V_{ij}=\lambda {\left(\varepsilon_{ij}\right)}_{\text{new}}-\sum_{j=1}^{4}\left(\lambda \left(m_{i}\right)-\lambda {\left(c_{ij}\right)}_{\text{avg}}\right),\\ V_{qr}=\lambda {\left(\varepsilon_{qr}\right)}_{\text{ref}}-\sum_{r=1}^{4}\left(\lambda \left(m_{q}\right)-\lambda {\left(c_{qr}\right)}_{\text{avg}}\right). \end{array}$$

The state function ^*n*^ *V*_*ij*_ is for the *i*th peptide measured under a set of *c*_*ij*_ boundary conditions in output, final, or new state. The conjugated state function ^*r*^ *V*_*qr*_ is for the *q*th peptide measured under a set of *c*_*qr*_ boundary conditions for the input, initial, or reference state. The difference Δ*V* between the new (output) state and the reference (input) state is the additive perturbation [58]. Consider

(2) ΔV=Vij−Vqr=[λ(εij)new−∑j=14(λ(mi)−λ(cij)avg)]−[λ(εqr)ref−∑r=14(λ(mq)−λ(cqr)avg)].

Equation (3) described before opens the door to test different hypothesis. A simple hypotheses is H_0_: existence of one small and constant value of the perturbation function Δ*V* = *e*_0_ for all the pairs of peptides and a linear relationship between perturbations of input/output boundary conditions with coefficients *a*_*ij*_, *b*_*ij*_, *c*_*qr*_, and *d*_*ij*_. Consider

(2) e0=ΔV=[aij·λ(εij)new−∑j=14bij·(λ(mi)−λ(cj)avg)]−[cqr·λ(εqr)ref−∑r=14dqr·(λ(mq)−λ(cr)avg)].

We can use elemental algebraic operations to obtain from these equations an expression for efficiency as epitope of the peptide *λ*(*ε*_*ij*_)_new_. In this case, considering *b*_*ij*_ ≈ *d*_*qr*_, we can obtain the different expressions; the last may be very useful to solve the QSRR problem for the large datasets formed by IEDB B-epitopes. Consider

(2) λ(εij)new=(cqraij)·λ(εqr)ref+[∑j=14(bqraij)·(λ(mi)−λ(cj)avg)new]−[∑r=14(dqraij)·(λ(mq)−λ(cr)avg)ref]+(e0aij),λ(εij)new=c0′· λ(εqr)ref+∑j=14dij′· Δ(λ(mi)−λ(cj)avg)+e0′,λ(εij)new=c0′· λ(εqr)ref+∑j=14dij′· ΔΔλijqr+e0′,λ(εij)new=c0′· λ(εqr)ref+∑j=14dij′· ΔΔχijqr+e0′.

## 3. Results and Discussion

We propose herein, for the first time, a QSRR-perturbation model able to predict variations in the propensity of a peptide to act as B-epitope taking into consideration the propensity of a peptide of reference and the changes in peptide sequence, immunological process, host organism, source organisms, and the experimental technique used. The best QSPR-perturbation model found here with LDA was

(6) λ(εij)new=4.979·λ(εij)ref−221.642·Δχseq+8.770·ΔΔχho+63.572·ΔΔχso−55.387·ΔΔχip+201.919·ΔΔχtq−2.149,N=155169,  Rc=0.92,U=0.15,  p<0.01.

The first input term is the value *λ*(*ε*_*ij*_)_ref_ is the scoring function *λ* of the efficiency of the initial process *ε*_*ij*_ (known solution). The function *λ*(*ε*_*ij*_)_ref_ = 1 if the *i*th peptide could experimentally be demonstrated to be a B-epitope in the assay of reference (reference) carried out in the conditions *c*_*j*_, *λ*(*ε*_*ij*_)_ref_ = 0 otherwise. The variational-perturbation terms ΔΔ*χ*_*cj*_ are at the same time terms typical of perturbation theory and moving average (MA) functions used in Box-Jenkin models in time series [60]. These new types of terms account both for the deviation of the electronegativity of all amino acids in the sequence of the new peptide with respect to the peptide of reference and with respect to all boundary conditions. In Table 2, we give the overall classification results obtained with this model. Speck-Planche et al. [61–63] introduced different multitarget/multiplexing QSAR models that incorporate this type of information based on MAs. The results obtained with the present model are excellent compared with other similar models in the literature useful for other problems including moving average models [64, 65] or perturbation models [58]. Notably, this is also the first model combining both perturbation theory and MAs in a QSPR context.

The other input terms are the following. The first Δ*χ*_seq_ = *χ*(*m*_*q*_)_ref_ − *χ*(*m*_*i*_)_new_ is the perturbation term for the variation or in the mean value of electronegativity for all amino acids in the sequence of the peptide of reference. The remnant input variables of the model ΔΔ*χ*_*cj*_ = Δ*χ*_*cj*-ref_ − Δ*χ*_*cj*-new_ = [*χ*(*m*_*q*_)_ref_ − *χ(*c*_*qr*_)_ref_]−[*χ*(*m*_*i*_)_new_ − *χ(*c*_*ij*_)_new_] quantify values of the conditions of the new assay *cj*-new that represent perturbations with respect to the initial conditions *c*_*ij*_-ref of the assay of reference. The quantities *χ(*c*_*ij*_) and *χ(*c*_*qr*_) are the average values of the mean electronegativity values *χ*(*m*_*i*_) and *χ*(*m*_*q*_) for all new and reference peptides in IEDB that are epitopes under the *j*th or *r*th boundary condition. The values of these terms have been tabulated for >500 source organisms, >50 host organisms, >10 biological process, and >30 experimental techniques. We must substitute the values of *χ*(*m*_*i*_) and *χ*(*m*_*q*_) of the new and reference peptides and the tabulated values of *χ(*c*_*ij*_) and *χ(*c*_*qr*_) for all combinations of boundary conditions to predict the perturbations of the action as epitope of peptides. In doing so we can found the optimal sequence and boundary conditions towards the use of the peptide in the development of a vaccine. In Table 2 we give some of these values of *χ(*c*_*ij*_) and *χ(*c*_*qr*_).

In Table 3 we depict the sequences and input-output boundary conditions for top perturbations present in IEDB. All these perturbations have observed value of *λ*(*ε*_*ij*_)_new_ = 1 and predicted value also equal to 1 with a high probability. See Supplementary Material available online at http://dx.doi.org/10.1155/2014/768515 file contains a full list of >200,000 cases of perturbations.

## 4. Conclusions

It is possible to develop general models for vaccine design able to predict the results of multiple input-output perturbations in peptide sequence and experimental assay boundary conditions using ideas of QSPR analysis, perturbation theory, and Box and Jenkins MA operators. The electronegativity values calculated with MARCH-INSIDE seem to be good molecular descriptors for this type of QSPR-perturbation models.

## Conflict of Interests

The authors declare that there is no conflict of interests regarding the publication of this paper.

## Supplementary Material

Supplementary Material includes the sequences of peptides obtained from IEDB, boudary conditions (source organisms, host organisms, techniques, biological process) as well as the values of the input/output variables of the models calculated for all the cases present in the dataset used. These values have been obtained for all the input-output boundary conditions for the perturbations.

## Figures and Tables

**Table 1:** Results of QSPR-perturbation model for IEDB B-Epitopes.

Data	Stat.	Pred.	Predicted epitope perturbations	
subset	param.	%	*λ*(*ε*_*ij*_) = 1	*λ*(*ε*_*ij*_) = 0
*λ*(*ε*_*ij*_) = 1	Sp	97.0	**84607**	2660
*λ*(*ε*_*ij*_) = 0	Sn	93.6	4354	**63548**

Total train	Ac	95.5		

*λ*(*ε*_*ij*_) = 1	Sp	97.1	**28060**	840
*λ*(*ε*_*ij*_) = 0	Sn	93.3	1485	**20641**

Total cv	Ac	95.4		

Bold font is used to highlight the number of cases correctly classified by the model.

**Table 2:** Average values and count of input-output cases for different organisms, process, and techniques.

Source organism (so)	*N*_in_	*N*_out_	*χ
*Homo sapiens *	38920	39274	2.685
*Plasmodium falciparum *	10669	9446	2.704
*Hepatitis C virus *	9935	10239	2.683
*Bos taurus *	5671	5780	2.690
*Canine parvovirus *	5655	5637	2.693
*Foot-mouth disease virus *	4062	4176	2.676
*Triticum aestivum *	3769	3887	2.703
*Bacillus anthracis *	3602	3600	2.699
*Human papillomavirus *	3316	3414	2.693
*Human herpesvirus *	3026	3132	2.684
*Gallus gallus *	2850	2829	2.689
*Arachis hypogaea *	2648	2670	2.687
*Mycobacterium tuberculosis *	2637	2593	2.688
*Clostridium botulinum *	2588	2722	2.685
*SARS coronavirus *	2550	2704	2.686
*Mus musculus *	2334	2287	2.682
*Hepatitis B virus *	2007	2066	2.680
*Helicobacter pylori *	1958	1796	2.695
*Hevea brasiliensis *	1938	1958	2.697
*Hepatitis E virus *	1928	1941	2.685
*Shigella flexneri *	1878	1701	2.699
*Dengue virus 2 *	1767	1828	2.679
*Staphylococcus aureus *	1757	1661	2.694
*Treponema pallidum *	1739	1755	2.691
*Escherichia coli *	1721	1678	2.689
*Murine hepatitis virus *	1575	1603	2.692
*Haemophilus influenzae *	1545	1587	2.695
*Streptococcus mutans *	1523	1537	2.697
*Puumala virus (strain) *	1505	1574	2.689
*Chlamydia trachomatis *	1402	1546	2.704
*Human respiratory virus *	1347	1398	2.682
*Borrelia burgdorferi *	1228	1237	2.698
*Hepatitis delta virus *	1182	1199	2.690
*Streptococcus pyogenes *	1181	1251	2.697
*Porphyromonas gingivalis *	1143	1085	2.688
*Human enterovirus *	1106	1132	2.689
*Influenza A virus *	1085	1086	2.687
*Mycoplasma hyopneumoniae *	1044	1024	2.695
*Rattus norvegicus *	1025	1039	2.689
*Bordetella pertussis *	1011	960	2.685
*Human T-lymphotropic virus *	996	1031	2.680
*Anaplasma marginale *	977	857	2.707
*Measles virus strain *	804	810	2.688
*Fasciola hepatica *	803	857	2.685
*Neisseria meningitidis *	789	853	2.696
*Human poliovirus *	766	780	2.690
*Tityus serrulatus *	764	775	2.680
*Torpedo californica *	752	788	2.687
*Cryptomeria japonica *	719	794	2.680
*Mycobacterium bovis *	717	733	2.688
*Trypanosoma cruzi *	691	777	2.704
*Andes virus CHI-7913 *	679	687	2.690
*Bovine papillomavirus *	672	665	2.692
*Human hepatitis *	670	696	2.688
*Leishmania infantum *	659	735	2.688
*Human parvovirus *	649	691	2.683
*Poa pratensis *	648	664	2.692
*Aspergillus fumigatus *	642	709	2.677
*Duck hepatitis *	587	603	2.688
*Olea europaea *	571	577	2.692
*Porcine reproductive *	515	514	2.681
*Fagopyrum esculentum *	509	497	2.685
*Juniperus ashei *	505	568	2.672
*Mycobacterium leprae *	489	542	2.690
*Glycine max *	477	509	2.685
*D. pteronyssinus *	455	464	2.680
*Plasmodium vivax *	453	446	2.690
*Chlamydophila pneumoniae *	446	462	2.690
*Pseudomonas aeruginosa *	443	454	2.691
*Vibrio cholera *	427	426	2.694
*Streptococcus sp. *	426	425	2.691
*Mycobacterium avium *	425	415	2.689
*Dermatophagoides farinae *	410	390	2.693
*Human coxsackievirus *	406	392	2.694
*Equine infectious virus *	404	419	2.688
*Babesia equi *	383	371	2.696
*Prunus dulcis *	383	379	2.708
*Human adenovirus *	375	405	2.686
*Theileria parva *	366	371	2.713
*Candida albicans *	365	370	2.690
*Porcine endogenous *	355	351	2.692
*Ovis aries *	352	350	2.683
*Chironomus thummi *	347	338	2.691
*Sus scrofa *	343	362	2.686
*Bovine leukemia virus *	333	329	2.676
*Ricinus communis *	329	314	2.692
*Androctonus australis *	322	357	2.685
*Renibacterium salmoninarum *	319	350	2.690
*Orientia tsutsugamushi *	309	372	2.705
*Anacardium occidentale *	293	306	2.693
*Conus geographus *	289	295	2.660

Host organism (ho)	*N*_in_	*N*_out_	*χ

*Homo sapiens *	257293	91093	2.6856
*Mus musculus *	107867	51466	2.6873
*Oryctolagus cuniculus *	65053	31433	2.6900
*Bos taurus *	15333	2072	2.6909
*Rattus norvegicus *	9450	3562	2.6876
*Aotus sp. *	9044	3933	2.6879
*Sus scrofa *	7725	3464	2.6873
*Gallus gallus *	7507	997	2.6790
*Canis lupus *	6604	3334	2.6906
*Macaca mulatta *	5261	2569	2.6993
*Ovis aries *	3953	1653	2.6836
*Equus caballus *	3943	2099	2.6842
*Cavia porcellus *	3458	1688	2.6833
*Capra hircus *	2182	1127	2.6830
*Aotus nancymaae *	1659	852	2.6837
*Pan troglodytes *	1614	732	2.6757
*Marmota monax *	1100	509	2.7011
*Felis catus *	901	279	2.6838
*Myodes glareolus *	814	388	2.6863
*Anas platyrhynchos *	688	342	2.6880
*Homo sapiens (human) *	508	270	2.6851
*Trichosurus vulpecula *	456	126	2.6921
*Mesocricetus auratus *	438	104	2.6909
*Macaca cyclopis *	382	193	2.6871
*O. tshawytscha *	333	159	2.6929
*Macaca fuscata *	188	100	2.6667
*Cricetulus migratorius *	171	142	2.7008
*Camelus dromedarius *	171	89	2.6886
*Dicentrarchus labrax *	121	55	2.6759
*Macaca fascicularis *	96	52	2.6793
*Saimiri sciureus *	92	44	2.6900
*Canis familiaris *	77	42	2.6850
*Rattus rattus *	72	31	2.6760
*Callithrix pygmaea *	67	30	2.6920
*Chinchilla lanigera *	41	24	2.6729
*Aotus lemurinus *	30	19	2.6860
*Papio cynocephalus *	27	13	2.7267
*Aotus griseimembra *	26	12	2.7000
*Mustela vison *	18	10	2.7000
*Chlorocebus aethiops *	15	10	2.6875
*Bos indicus *	13	4	2.6925
*Oncorhynchus mykiss *	10	4	2.6700
*M. macquariensis *	9	6	2.6600
*Cricetulus griseus *	8	4	2.6900
*Aotus trivirgatus *	7	4	2.7000

Process type (pt)	*N*_in_	*N*_out_	*χ

AID	111197	108536	2.6876
OOID	32419	32617	2.6868
OAID	19210	18954	2.6801
OOA	15863	16303	2.6902
NI	13430	15206	2.6845
EWEIR	4818	4864	2.6843
EEE	3113	3546	2.6906
OOD	2806	2799	2.6887
AICD	1077	1095	2.6812
EWED	696	686	2.6879
DEWED	280	337	2.6804
TT	260	215	2.6806
OOC	153	137	2.6800

Technique (tq)	*N*_in_	*N*_out_	*χ

ELISA	133458	135109	2.6871
WI	33627	33292	2.6887
ACAbB	7780	9068	2.6862
PhDIP	7450	4496	2.6771
RIA	5241	5218	2.6858
IFAIH	4454	4581	2.6879
NIAA	4222	4316	2.6892
FIA	2255	2276	2.6897
PAC	1312	1219	2.6837
IP	1127	1089	2.6886
SPR	758	639	2.6860
FACS	608	647	2.6907
Other	502	495	2.6813
SAC	484	393	2.6878
ELISPOT	396	412	2.6979
RDAT	366	323	2.6859
EDAT	284	330	2.6800
XRC	231	227	2.6880
MS	209	179	2.6849
PFF	171	153	2.6820
AbDPO	162	295	2.6968
CdC	146	205	2.6895
IAbBA	144	183	2.6940
IOT	124	106	2.6835
HAGGI	115	122	2.6834
IgMHR	89	90	2.6929
EAAA	84	139	2.6922
HS	82	67	2.6791
AbdCC	73	118	2.6897
AGG	50	60	2.6980
CM	50	57	2.6863

The ^*∗*^indicates that quantities like ^*∗*^ *χ* is the average value of the mean electronegativity (*m*_*i*_) for all the peptides in IEDB that are epitopes for the same boundary condition.

**Table 3:** Top100 values of p1 for positive perturbations in training series.

New experiment						Experiment of reference						Input perturbation terms				
IDE	Sequence	ho	so	ip	tq	IDE	Sequence	ho	so	ip	tq	Δ*χ*	ΔΔ*χ*_ho_	ΔΔ*χ*_so_	ΔΔ*χ*_ip_	ΔΔ*χ*_tq_
115153	MKGVVC TRIYEKV	*Homo sapiens *	*Homo sapiens *	OAID	ELISA	115155	NNQRK KAKNTP FNMLKRERN	*Mus musculus *	*Dengue virus 2 *	AID	WI	0.01	0.012	0.004	0.017	0.012
52124	QQQPP	*Homo sapiens *	*Triticum aestivum *	OOA	ELISA	52128	QQQQGG SQSQKGKQQ	*Homo sapiens *	*Glycine max *	OOA	WI	0	0	−0.018	0	0.002
3639	APLGVT	*Homo sapiens *	*Hepatitis E virus *	EWEIR	ELISA	3652	APLTRG SCRKRN RSPER	*Homo sapiens *	*Human herpesvirus *	OOID	ELISA	0.04	0.04	0.039	0.043	0.04
135959	LTRAYA KDVKFG	*Homo sapiens *	*Homo sapiens *	OAID	ELISA	135963	NGQEEK AGVVS TGLIGGG	*Mus musculus *	MD	AID	ELISA	−0.04	−0.038	−0.047	−0.033	−0.04
108075	PREPQVY	*Homo sapiens *	*Homo sapiens *	OAID	ELISA	108078	PTSPSGV EEWIVTQ VVPGVA	*Oryctolagus cuniculus *	*Homo sapiens *	AID	ACAbB	−0.01	−0.006	−0.01	−0.003	−0.011
25113	HVVDLP	*Homo sapiens *	*Hepatitis E virus *	EWEIR	ELISA	25126	HWGNH SKSHPQR	*Mus musculus *	MD	AID	ELISA	0.02	0.022	0.013	0.023	0.02
48780	PPFSPQ	*Homo sapiens *	*Hepatitis delta virus *	OOID	ELISA	48782	PPFTSAV GGVDHRS	*Mus musculus *	MD	AID	SAC	0.02	0.022	0.008	0.021	0.021
40988	LYVVAYQA	*Mus musculus *	*Viscum album *	AID	ELISA	41004	MAARLCC QLDPARDV	*Homo sapiens *	*Hepatitis B virus *	OOID	ELISA	0.02	0.018	0.006	0.019	0.02
50439	QDAYNAAG	*Mus musculus *	*Mycobacterium scrofulaceum *	AID	ELISA	50445	QDCNCSI YPGHAS GHRMAWD	*Homo sapiens *	*Hepatitis C virus *	OOID	ELISA	0.04	0.038	0.028	0.039	0.04
98849	KIPAVFKIDA	*Homo sapiens *	*Bos taurus *	DEWED	WI	98850	KKGSEEE GDITNPIN	*Homo sapiens *	*Arachis hypogaea *	OOA	IFAIH	−0.05	−0.05	−0.053	−0.04	−0.051
116171	TQDQDP BBHFFK NIVTPR	*Homo sapiens *	*Homo sapiens *	OAID	ACAbB	116286	CGKGLS ATVTGG QKGRGSR	*Oryctolagus cuniculus *	*Mus musculus *	AID	MS	0.01	0.014	0.008	0.017	0.009
123442	LLKDLRKN	*Homo sapiens *	*Borna disease virus *	EWEIR	WI	123443	LLTEHR MTWDPA QPPRDLTE	*Homo sapiens *	*Homo sapiens *	OOID	ELISA	−0.02	−0.02	−0.025	−0.017	−0.022
47858	PHVVDL	*Homo sapiens *	*Hepatitis E virus *	EWEIR	ELISA	47860	PHWIK KPNRQG LGYYS	*Capra hircus *	*Human T-lymphotropic virus *	AID	ACAbB	0.01	0.007	0.005	0.013	0.009
61783	STNKAVVSLS	*Bos taurus *	*Bovine respiratory *	AID	ELISA	61792	STNPKP QRKTKRN TNRRPQD	*Homo sapiens *	*Hepatitis C virus *	OOID	ELISA	0.03	0.025	0.019	0.029	0.03
118210	VMLYQISEE	*Homo sapiens *	*Homo sapiens *	OAID	WI	118217	VTKYITK GWKEVH	*Oryctolagus cuniculus *	*Homo sapiens *	AID	ELISA	0.05	0.054	0.05	0.057	0.048
130944	LFKHS	*Oryctolagus cuniculus *	*Rattus norvegicus *	AID	ELISA	130956	LPPRVTP KWSLDA WSTWR	*Homo sapiens *	MD	OOD	WI	0.01	0.006	−0.002	0.011	0.012
23028	GVKYA	*Homo sapiens *	MD	OAID	WI	23032	GVLAKD VRFSQV	*Homo sapiens *	MD	OOID	ELISA	0	0	0	0.007	−0.002
51199	QKKAIE	*Oryctolagus cuniculus *	*Vibrio cholerae *	AID	ELISA	51204	QKKNK RNTNRR PQDV	*Homo sapiens *	*Hepatitis C virus *	OOID	ELISA	0.03	0.026	0.018	0.029	0.03
144783	SHVVT MLDNF	*Homo sapiens *	*Homo sapiens *	NI	ELISA	144786	SMNRGRG THPSLIWM	*Mus musculus *	MD	AID	ACAbB	0.03	0.032	0.023	0.033	0.029
134343	DLYIK	*Mus musculus *	*Human papillomavirus *	AID	NIAA	134344	DMAQV TVGPGLL GVSTL	*Mus musculus *	*Homo sapiens *	AID	WI	0	0	−0.009	0	0
38321	LNQLAGRM	*Anas platyrhynchos *	*Duck hepatitis *	AID	ELISA	38323	LNQTAR AFPDCAI CWEPSPP	*Oryctolagus cuniculus *	*Bovine leukemia virus *	AID	ACAbB	−0.01	−0.008	−0.022	−0.01	−0.011
144657	GQITVD MMYG	*Homo sapiens *	*Homo sapiens *	OAID	ELISA	144661	GREGYP ADGGCA WPACYC	*Oryctolagus cuniculus *	MD	AID	WI	0.02	0.024	0.013	0.027	0.022
21084	GLQN	*Mus musculus *	*Chlamydia trachomatis *	AID	ELISA	21093	GLRAQD DFSGWDI NTPAFEW	*Mus musculus *	*Mycobacterium tuberculosis *	AID	WI	0.03	0.03	0.013	0.03	0.032
98453	SGFSGSVQFV	*Oryctolagus cuniculus *	*Neisseria meningitidis *	AID	ELISA	98456	SICSNN PTCWAIC KRIPNKK	*Mus musculus *	*Human respiratory virus *	AID	IFAIH	0.04	0.037	0.027	0.04	0.041
98453	SGFSGSVQFV	*Mus musculus *	*Neisseria meningitidis *	AID	ELISA	98456	SICSNN PTCWAIC KRIPNKK	*Mus musculus *	*Human respiratory virus *	AID	IFAIH	0.04	0.04	0.027	0.04	0.041
107107	EAIQP	*Rattus norvegicus *	*Homo sapiens *	AID	ELISA	107110	EKERRP SPIGTATLL	*Homo sapiens *	MD	OOA	ELISA	0.05	0.048	0.043	0.053	0.05
110857	FTGEAY SYWSAK	*Homo sapiens *	*Mycoplasma penetrans *	EWEIR	ELISA	110859	GEESRIS LPLPNF SSLNLRE	*Mus musculus *	*Homo sapiens *	AID	FIA	0	0.002	−0.017	0.003	0.003
36315	LGSAYP	*Mus musculus *	*Mycobacterium leprae *	MD	ELISA	36317	LGSGAFG TIYKG	*Mus musculus *	*Avian erythroblastosis virus *	AID	ACAbB	0.01	0.01	0	0.011	0.009
122034	WNPAD	*Rattus norvegicus *	*Torpedo californica *	AID	ELISA	122035	WNPAD YGGIKWN PADYGGIK	*Rattus norvegicus *	MD	AID	RIA	0.01	0.01	0.001	0.01	0.009
25013	HVADIDKLID	*Mus musculus *	*Puumala virus Kazan *	AID	ELISA	25021	HVAPTH YVTESDA SQRVTQL	*Homo sapiens *	*Hepatitis C virus *	OOID	ELISA	0	−0.002	−0.014	−0.001	0
36162	LGIHE	*Oryctolagus cuniculus *	*Candida albicans *	AID	ELISA	36166	LGIMGE YRGTPRN QDLYDAA	*Mus musculus *	*Human respiratory virus *	AID	RIA	0	−0.003	−0.007	0	−0.001
67253	TWEVLH	*Mus musculus *	*Plasmodium vivax *	AID	ELISA	67257	TWGEN ETDVLLL NNTRPPQ	*Homo sapiens *	*Hepatitis C virus *	OOID	ACAbB	−0.02	−0.022	−0.027	−0.021	−0.021
50990	QGYRVSSYLP	*Homo sapiens *	*Hevea brasiliensis *	OOA	WI	50998	QHEQDR PTPSPAP SRPFSVL	*Homo sapiens *	*Hepatitis E virus *	OOID	ELISA	0.01	0.01	−0.002	0.007	0.008
100458	RDVLQLYAPE	*Mus musculus *	*Bacillus anthracis *	AID	ELISA	100462	RFSTRY GNQNGRI RVLQRFD	*Homo sapiens *	*Arachis hypogaea *	EWED	ELISA	0.03	0.028	0.018	0.03	0.03
111036	TESTFT GEAYSV	*Homo sapiens *	*Mycoplasma penetrans *	EWEIR	ELISA	111039	TGVPID PAVPDSS IVPLLES	*Bos taurus *	*Bovine papillomavirus *	AID	ELISA	0.03	0.035	0.02	0.033	0.03
117919	IFIEME	*Homo sapiens *	*Homo sapiens *	OAID	WI	117921	IGIIDLIE KRKFNQ	*Mus musculus *	*Homo sapiens *	AID	WI	0.03	0.032	0.03	0.037	0.03
7127	CTDTDKLF	*Oryctolagus cuniculus *	*Shigella flexneri *	AID	ELISA	7128	CTDVST AIHADQL TPAW	*Homo sapiens *	*SARS coronavirus *	OOID	ELISA	0.01	0.006	−0.003	0.009	0.01
112253	PGQSPKL	*Homo sapiens *	*Homo sapiens *	OAID	ELISA	112255	PIRALV GDEVELP CRISPGK	*Mus musculus *	*Homo sapiens *	AID	ELISA	0.01	0.012	0.01	0.017	0.01
122034	WNPAD	*Rattus norvegicus *	*Torpedo californica *	AID	ELISA	122038	WNPDDY GGVKWNP DDYGGVK	*Rattus norvegicus *	MD	AID	RIA	0	0	−0.009	0	−0.001
131878	FLMLVG GSTL	*Homo sapiens *	*Homo sapiens *	OAID	ACAbB	131879	FLVAHT RARAPSA GERARRS	*Mus musculus *	*Mus musculus *	AID	NIAA	0.03	0.032	0.028	0.037	0.033
70664	VQVVYDYQ	*Homo sapiens *	*Treponema pallidum *	OOID	ELISA	70667	VQWMNR LIAFAFAG NHVSP	*Homo sapiens *	*Hepatitis C virus *	OOID	ELISA	0.05	0.05	0.041	0.05	0.05
71545	VTV	*Homo sapiens *	*Helicobacter pylori *	OOID	ELISA	71559	VTVRGGL RILSPDRK	*Homo sapiens *	*Arachis hypogaea *	OOA	WI	0.04	0.04	0.032	0.043	0.042
127856	TDVRYKD	*Mus musculus *	*Mus musculus *	AID	ACAbB	127857	TDVRYK DDMYHFF CPAIQAQ	*Mus musculus *	*Mus musculus *	AID	PFF	0.01	0.01	0.01	0.01	0.006
112149	GVGWIRQ	*Homo sapiens *	*Homo sapiens *	OAID	ELISA	112152	HHPART AHYGSLP QKSHGRT	*Homo sapiens *	*Homo sapiens *	AID	ELISA	0	0	0	0.007	0
119581	FSCSVMHE	*Homo sapiens *	*Homo sapiens *	OAID	ELISA	119582	GLQLIQL INVDEVNQI	*Mus musculus *	*Homo sapiens *	AID	RIA	−0.01	−0.008	−0.01	−0.003	−0.011
144657	GQITVD MMYG	*Homo sapiens *	*Homo sapiens *	OAID	ELISA	144659	GREGYP ADGGAA GYCNTE	*Oryctolagus cuniculus *	MD	AID	WI	−0.01	−0.006	−0.017	−0.003	−0.008
25013	HVADI DKLID	*Mus musculus *	*Puumala virus Kazan *	AID	ELISA	25022	HVAPTH YVVESDA SQRVTQV	*Homo sapiens *	*Hepatitis C virus *	OOID	ELISA	0	−0.002	−0.014	−0.001	0
144652	GMRGM KGLVY	*Homo sapiens *	*Homo sapiens *	OAID	ELISA	144654	GPHPTLE VVPMGRGS	*Mus musculus *	MD	AID	ELISA	−0.02	−0.018	−0.027	−0.013	−0.02
104515	HDCRPKKI	*Mus musculus *	*La Crosse virus *	AID	IFAIH	104521	IGTLKKIL DETVKD KIAKEQ	*Rattus norvegicus *	*Streptococcus pyogenes *	AID	ELISA	−0.04	−0.04	−0.053	−0.04	−0.041
7367	CYGDWA	*Homo sapiens *	*Triticum aestivum *	OOA	ELISA	7374	CYGLPDS EPTKTNGK	*Mus musculus *	*Tityus serrulatus *	AID	WI	−0.02	−0.018	−0.043	−0.023	−0.018
7367	CYGDWA	*Homo sapiens *	*Triticum aestivum *	OOA	ELISA	7374	CYGLPDS EPTKTNGK	*Mus musculus *	*Tityus serrulatus *	AID	WI	−0.02	−0.018	−0.043	−0.023	−0.018
144610	DFFTYK	*Mus musculus *	*Porcine transmissible *	AID	WI	144611	DFNGSF DMNGTITA	*Oryctolagus cuniculus *	*Escherichia coli *	AID	ELISA	−0.01	−0.007	−0.018	−0.01	−0.012
112047	ASTRESG	*Homo sapiens *	*Homo sapiens *	OAID	ELISA	112048	ATASTM DHARHGF LPRHRDT	*Homo sapiens *	*Homo sapiens *	AID	ELISA	0.05	0.05	0.05	0.057	0.05
36136	LGGVFT	*Homo sapiens *	*Dengue virus 2 *	OOID	ELISA	36137	LGGWKLQ SDPRAYAL	*Homo sapiens *	*Ambrosia artemisiifolia *	OOA	RIA	0.01	0.01	0.007	0.013	0.009
115256	FRELKD LKGY	*Homo sapiens *	*Bos taurus *	DEWED	WI	115261	GDLEILL QKWENG ECAQKKI	*Homo sapiens *	*Bos taurus *	OOA	FIA	−0.01	−0.01	−0.01	0	−0.009
129024	KADQLYK	*Homo sapiens *	*Homo sapiens *	OAID	ELISA	129026	KAKKP AAAAGA KKAKS	*Oryctolagus cuniculus *	*Homo sapiens *	AID	ELISA	0.03	0.034	0.03	0.037	0.03
148481	YTRDLVYK	*Rattus norvegicus *	*Homo sapiens *	AID	WI	148483	YVPIVT FYSEISM HSSRAIP	*Oryctolagus cuniculus *	MD	AID	ELISA	0	0.002	−0.007	0	−0.002
150850	GY	*Mus musculus *	*Human papillomavirus *	AID	ELISA	150853	HIGGLSI LDPIFGVL	*Homo sapiens *	*Dermatophagoides farinae *	OOA	ACAbB	0.04	0.038	0.04	0.043	0.039
107366	FPPKPKD	*Homo sapiens *	*Homo sapiens *	OAID	ELISA	107376	GDRSGYS SPGSPG	*Mus musculus *	*Homo sapiens *	AID	ACAbB	−0.03	−0.028	−0.03	−0.023	−0.031
114859	ICGTD GVTYT	*Homo sapiens *	*Gallus gallus *	OOA	WI	114865	IVERETR GQSENPL WHALRR	*Rattus norvegicus *	*Human herpesvirus *	AID	ELISA	0.04	0.042	0.035	0.037	0.038
62149	SVHLF	*Homo sapiens *	MD	OAID	WI	62150	SVIALGS QEGALHQ ALAGAI	*Equus caballus *	*West Nile virus *	AID	IFAIH	−0.02	−0.021	−0.012	−0.013	−0.021
98455	SGSVQFVPIQ	*Mus musculus *	*Neisseria meningitidis *	AID	ELISA	98456	SICSNNP TCWAICK RIPNKK	*Mus musculus *	*Human respiratory virus *	AID	IFAIH	0.04	0.04	0.027	0.04	0.041
61783	STNKAV VSLS	*Bos taurus *	*Bovine respiratory *	AID	ELISA	61791	STNPKPQ RKTKRNT NRRPQ	*Homo sapiens *	*Hepatitis C virus *	EWEIR	ACAbB	0.04	0.035	0.029	0.037	0.039
100318	NAPKT FQFIN	*Mus musculus *	*Bacillus anthracis *	AID	ELISA	100319	NASSELH LLGFGIN AENNHR	*Homo sapiens *	*Arachis hypogaea *	EWED	ELISA	0	−0.002	−0.012	0	0
118947	PFSAPPPA	*Homo sapiens *	*Homo sapiens *	OAID	ELISA	118948	PGAIEQG PADDPGE GPSTGP	*Homo sapiens *	*Human herpesvirus *	NI	ACAbB	−0.04	−0.04	−0.041	−0.036	−0.041
78323	YSFRD	*Mus musculus *	*Bluetongue virus 1 *	AID	ELISA	78341	AALTAEN TAIKKRN ADAKA	*Homo sapiens *	*Streptococcus mutans *	EEE	ELISA	0.01	0.008	0.007	0.013	0.01
145831	IPLGTRP	*Mus musculus *	*Human papillomavirus *	AID	ELISA	145841	KEDFRY AISSTNEI GLLGA	*Sus scrofa *	*Classical swine *	AID	PAC	−0.04	−0.04	−0.045	−0.04	−0.043
119592	HTFPAVLQ	*Homo sapiens *	*Homo sapiens *	OAID	ELISA	119596	IHIPSEKI WRPDLVLY	*Mus musculus *	*Homo sapiens *	AID	RIA	0.01	0.012	0.01	0.017	0.009
58780	SKAANLSIIK	MD	*Beet necrotic *	MD	WI	58783	SKAFSN CYPYDVP DYASL	*Oryctolagus cuniculus *	*Influenza A virus *	AID	RIA	−0.01	−0.004	−0.014	−0.009	−0.013
115153	MKGVVC TRIYEKV	*Homo sapiens *	*Homo sapiens *	NI	ELISA	115155	NNQRKK AKNTPFN MLKRERN	*Mus musculus *	*Dengue virus 2 *	AID	ELISA	0.01	0.012	0.004	0.013	0.01
133629	LPLRF	*Oryctolagus cuniculus *	*Gallus gallus *	AID	ACAbB	133630	LPPGLHV FPLASNRS	*Mus musculus *	MD	AID	SPR	−0.01	−0.013	−0.021	−0.01	−0.01
96215	EEEEAE DKED	*Homo sapiens *	*Homo sapiens *	OAID	ELISA	96216	EEEGLLK KSADTLW NMQK	*Mus musculus *	*Mus musculus *	AID	ELISA	0.08	0.082	0.078	0.087	0.08
39782	LTAASV	*Homo sapiens *	*Triticum aestivum *	OOA	ELISA	39788	LTAELKI YSVIQAEI NKHL	*Oryctolagus cuniculus *	*Yersinia pestis *	AID	ACAbB	0.01	0.014	−0.001	0.007	0.009
107479	KFNWYVD	*Homo sapiens *	*Homo sapiens *	OAID	ELISA	107482	KGEPGL PGRGFP GFP	*Mus musculus *	*Homo sapiens *	AID	ACAbB	0	0.002	0	0.007	−0.001
63569	TETVNSDI	*Macaca mulatta *	*Shigella flexneri *	AID	ELISA	63573	TEVELKER KHRIEDAV RNAK	*Homo sapiens *	*Mycobacterium leprae *	OOID	ELISA	0.05	0.036	0.041	0.049	0.05
134028	DDTIS	*Mus musculus *	*Homo sapiens *	AID	NIAA	134029	DEDENQS PRSFQKKTR	*Oryctolagus cuniculus *	*Homo sapiens *	AID	ELISA	0.05	0.053	0.05	0.05	0.048
23028	GVKYA	*Homo sapiens *	MD	OAID	WI	23032	GVLAKDV RFSQV	*Homo sapiens *	MD	EWEIR	ACAbB	0	0	0	0.004	−0.003
115293	IMCVKK ILDK	*Homo sapiens *	*Bos taurus *	DEWED	WI	115295	INPSKEN LCSTFCK EVVRNA	*Homo sapiens *	*Bos taurus *	OOA	FIA	−0.03	−0.03	−0.03	−0.02	−0.029
65105	TLTPENTL	*Mus musculus *	*Shigella flexneri *	AID	ELISA	65110	TLTSGSD LDRCTT FDDV	*Oryctolagus cuniculus *	*SARS coronavirus *	AID	ELISA	0.01	0.013	−0.003	0.01	0.01
134471	PKPEQ	*Mus musculus *	*Streptococcus pneumoniae *	AID	FACS	134472	PLLPGT STTSTG PCKT	*Homo sapiens *	*Hepatitis B virus *	AID	ELISA	0.02	0.018	0.019	0.02	0.016
142228	LYCYEQ LNDSSE	*Homo sapiens *	*Human papillomavirus *	NI	ELISA	142250	NWGDEP SKRRDRS NSRGRKN	*Felis catus *	*Feline infectious *	AID	ELISA	0.07	0.068	0.071	0.073	0.07
21549	GNYDFW YQS	*Homo sapiens *	*Staphylococcus aureus *	OOID	ELISA	21553	GNYNYKY RYLRHGK LRPFER	*Mus musculus *	*SARS coronavirus *	AID	ELISA	0.03	0.032	0.021	0.031	0.03
21549	GNYDFWYQS	*Homo sapiens *	*Staphylococcus aureus *	OOID	ELISA	21553	GNYNYKY RYLRHGK LRPFER	*Oryctolagus cuniculus *	*SARS coronavirus *	AID	ELISA	0.03	0.034	0.021	0.031	0.03
34908	LAPLGE	*Homo sapiens *	*Hepatitis E virus *	EWEIR	ELISA	34914	LAPSTL RSLRKR RLSSP	*Homo sapiens *	*Human herpesvirus *	OOID	ELISA	0.06	0.06	0.059	0.063	0.06
130454	CLFPNNSYC	*Mus musculus *	MD	AID	NIAA	130456	CRPQVN NPKEWS CAAC	*Homo sapiens *	MD	OOD	ACAbB	0.01	0.008	0.01	0.011	0.007
19644	GFVPSM	*Homo sapiens *	*Hepatitis delta virus *	OOID	ELISA	19647	GFVSASI FGFQAEV GPNNTR	*Oryctolagus cuniculus *	*Vaccinia virus WR *	AID	ELISA	−0.01	−0.006	−0.013	−0.009	−0.01
20678	GKRPE	*Mus musculus *	*Streptococcus pyogenes *	AID	WI	20680	GKSKRD AKNNAAK LAVDKLL	*Mus musculus *	*Vaccinia virus WR *	AID	WI	0.01	0.01	0.001	0.01	0.01
123278	GYLKDLPTT	*Ovis aries *	*Fasciola hepatica *	AID	ELISA	123282	HACQKK LLKFEAL QQEEGEE	*Rattus norvegicus *	*Gallus gallus *	AID	PFF	−0.02	−0.016	−0.016	−0.02	−0.025
141067	PLSLEPDP	*Mus musculus *	*Homo sapiens *	AID	FACS	141073	REGVRW RVMAIQ	*Mus musculus *	*Homo sapiens *	AID	ELISA	0.06	0.06	0.06	0.06	0.056
139248	SFAGTVIE	*Mus musculus *	*Classical swine *	AID	WI	139305	TAAQITQ RKWEAA REAEQRR	*Oryctolagus cuniculus *	*Homo sapiens *	AID	IP	0.03	0.033	0.026	0.03	0.03
104515	HDCRPKKI	*Mus musculus *	*La Crosse virus *	AID	IFAIH	104520	IAKEQE NKETIGT LKKILDE	*Rattus norvegicus *	*Streptococcus pyogenes *	AID	ELISA	−0.06	−0.06	−0.073	−0.06	−0.061
113517	HLYADGLTD	*Mus musculus *	*Human papillomavirus *	AID	IFAIH	113518	HNKIQA IELEDLLR YSKLYR	*Mus musculus *	*Homo sapiens *	AID	ELISA	0.02	0.02	0.011	0.02	0.019
156970	VERHQ	*Homo sapiens *	*Homo sapiens *	OAID	ELISA	156975	WSSTV LRVSPT RTVP	*Mus musculus *	MD	AID	ELISA	0.01	0.012	0.003	0.017	0.01
78252	PVQNLT	*Mus musculus *	*Porphyromonas gingivalis *	AID	WI	78253	QGGCGR GWAFSA TGAIEA	*Mus musculus *	*Glycine max *	AID	ELISA	0.02	0.02	0.017	0.02	0.018
6068	CCPDKNKS	*Mus musculus *	*Human herpesvirus *	AID	WI	6074	CCRHKQ KDVGDVK QTLPPS	*Ovis aries *	MD	AID	ELISA	0.01	0.006	0.004	0.01	0.008
53109	RAGVCY	*Homo sapiens *	*Triticum aestivum *	OOA	ELISA	53116	RAILTA FSPAQDI WGTS	*Oryctolagus cuniculus *	*SARS coronavirus *	AID	ELISA	−0.02	−0.016	−0.037	−0.023	−0.02
147041	IPEQ	*Homo sapiens *	*Triticum aestivum *	OOA	WI	147064	KHQGA QYVWN RTA	*Homo sapiens *	*Bos taurus *	OOID	WI	0.06	0.06	0.047	0.057	0.06
70664	VQVVYDYQ	*Oryctolagus cuniculus *	*Treponema pallidum *	AID	ELISA	70667	VQWMN RLIAFAF AGNHVSP	*Homo sapiens *	*Hepatitis C virus *	OOID	ELISA	0.05	0.046	0.041	0.049	0.05
134343	DLYIK	*Mus musculus *	*Human papillomavirus *	AID	PhDIP	134344	DMAQV TVGPGLL GVSTL	*Mus musculus *	*Homo sapiens *	AID	PhDIP	0	0	−0.009	0	0
