# Supplementary material for: Model for Vaccine Design by Prediction of B-Epitopes of IEDB Given Perturbations in Peptide Sequence, In Vivo Process, Experimental Techniques, and Source or Host Organisms
Source: J Immunol Res. 2014 Jan 12;2014:768515. doi: 10.1155/2014/768515 (PMC3987976; doi:10.1155/2014/768515)
Supplement: Supplementary file 1 — Supplementary Material includes the sequences of peptides obtained from IEDB, boudary conditions (source organisms, host organisms, techniques, biological process) as well as the values of the input/output variables of the models calculated for all the cases present in the dataset used. These values have been obtained for all the input-output boundary conditions for the perturbations. [file 768515.f1.pdf]

Pred		Obs	INPUT	VARS						NEW	STATE	
λ(εij) new	p1	λ(εij) new	λ(εij) ref	Δχseq	ΔΔχho	ΔΔχso	ΔΔχpt	ΔΔχtq	CV	IDEi_new	Sequence	ho
1	1.00	1	1	0.01	0.012	0.004	0.017	0.012	t	115153	MKGVVCT RIYEKV	Homo sapiens
1	1.00	1	1	0	0	-0.018	0	0.002	t	52124	QQQPP	Homo sapiens
1	1.00	1	1	0.04	0.04	0.039	0.043	0.04	t	3639	APLGVT	Homo sapiens
1	1.00	1	1	-0.04	-0.038	-0.047	-0.033	-0.04	t	135959	LTRAYAKD VKFG	Homo sapiens
1	1.00	1	1	-0.01	-0.006	-0.01	-0.003	-0.011	t	108075	PREPQVY	Homo sapiens
1	1.00	1	1	0.02	0.022	0.013	0.023	0.02	t	25113	HVVDLP	Homo sapiens
1	1.00	1	1	0.02	0.022	0.008	0.021	0.021	t	48780	PPFSPQ	Homo sapiens
1	1.00	1	1	0.02	0.018	0.006	0.019	0.02	t	40988	LYVVAYQA	Mus musculu s
1	1.00	1	1	0.04	0.038	0.028	0.039	0.04	t	50439	QDAYNAA G	Mus musculu s
1	1.00	1	1	-0.05	-0.05	-0.053	-0.04	-0.051	t	98849	KIPAVFKID A	Homo sapiens
1	1.00	1	1	0.01	0.014	0.008	0.017	0.009	t	116171	TQDQDPB BHFFKNIV TPR	Homo sapiens
1	1.00	1	1	-0.02	-0.02	-0.025	-0.017	-0.022	t	123442	LLKDLRKN	Homo sapiens
1	1.00	1	1	0.01	0.007	0.005	0.013	0.009	t	47858	PHVVDL	Homo sapiens
1	1.00	1	1	0.03	0.025	0.019	0.029	0.03	t	61783	STNKAVVS LS	Bos taurus

1	1.00	1	1	0.05	0.054	0.05	0.057	0.048	t	118210	VMLYQISE E	Homo sapiens
1	1.00	1	1	0.01	0.006	-0.002	0.011	0.012	t	130944	LFKHS	Oryctola gus cuniculu s
1	1.00	1	1	0	0	0	0.007	-0.002	t	23028	GVKYA	Homo sapiens
1	1.00	1	1	0.03	0.026	0.018	0.029	0.03	t	51199	QKKAIE	Oryctola gus cuniculu s
1	1.00	1	1	0.03	0.032	0.023	0.033	0.029	t	144783	SHVVTML DNF	Homo sapiens
1	1.00	1	1	0	0	-0.009	0	0	t	134343	DLYIK	Mus musculu s
1	1.00	1	1	-0.01	-0.008	-0.022	-0.01	-0.011	t	38321	LNQLAGR M	Anas platyrhy nchos
1	1.00	1	1	0.02	0.024	0.013	0.027	0.022	t	144657	GQITVDM MYG	Homo sapiens
1	1.00	1	1	0.03	0.03	0.013	0.03	0.032	t	21084	GLQN	Mus musculu s
1	1.00	1	1	0.04	0.037	0.027	0.04	0.041	t	98453	SGFSGSVQ FV	Oryctola gus cuniculu s
1	1.00	1	1	0.04	0.04	0.027	0.04	0.041	t	98453	SGFSGSVQ FV	Mus musculu s
1	1.00	1	1	0.05	0.048	0.043	0.053	0.05	t	107107	EAIQP	Rattus norvegic us
1	1.00	1	1	0	0.002	-0.017	0.003	0.003	t	110857	FTGEAYSY WSAK	Homo sapiens
1	1.00	1	1	0.01	0.01	0	0.011	0.009	t	36315	LGSAYP	Mus musculu s

1	1.00	1	1	0.01	0.01	0.001	0.01	0.009	t	122034	WNPAD	Rattus norvegicus
1	1.00	1	1	0	-0.002	-0.014	-0.001	0	t	25013	HVADIDKLID	Mus musculus
1	1.00	1	1	0	-0.003	-0.007	0	-0.001	t	36162	LGIHE	Oryctolagus cuniculus
1	1.00	1	1	-0.02	-0.022	-0.027	-0.021	-0.021	t	67253	TWEVLH	Mus musculus
1	1.00	1	1	-0.02	-0.022	-0.027	-0.021	-0.021	t	67253	TWEVLH	Mus musculus
1	1.00	1	1	0.01	0.01	-0.002	0.007	0.008	t	50990	QGYRVSSYLP	Homo sapiens
1	1.00	1	1	0.03	0.028	0.018	0.03	0.03	t	100458	RDVLQLYAPE	Mus musculus
1	1.00	1	1	0.03	0.035	0.02	0.033	0.03	t	111036	TESTFTGEAYSV	Homo sapiens
1	1.00	1	1	0.03	0.032	0.03	0.037	0.03	t	117919	IFIEME	Homo sapiens
1	1.00	1	1	0.01	0.006	-0.003	0.009	0.01	t	7127	CTDTDKLF	Oryctolagus cuniculus
1	1.00	1	1	0.01	0.012	0.01	0.017	0.01	t	112253	PGQSPKL	Homo sapiens
1	1.00	1	1	0	0	-0.009	0	-0.001	t	122034	WNPAD	Rattus norvegicus
1	1.00	1	1	0.03	0.032	0.028	0.037	0.033	t	131878	FLMLVGGSTL	Homo sapiens
1	1.00	1	1	0.05	0.05	0.041	0.05	0.05	t	70664	VQVVYDYQ	Homo sapiens
1	1.00	1	1	0.04	0.04	0.032	0.043	0.042	t	71545	VTV	Homo sapiens

1	1.00	1	1	0.01	0.01	0.01	0.01	0.006	t	127856	TDVRYKD	Mus musculus
1	1.00	1	1	0	0	0	0.007	0	t	112149	GVGWIRQ	Homo sapiens
1	1.00	1	1	-0.01	-0.008	-0.01	-0.003	-0.011	t	119581	FSCSVMHE	Homo sapiens
1	1.00	1	1	-0.01	-0.006	-0.017	-0.003	-0.008	t	144657	GQITVDMMYG	Homo sapiens
1	1.00	1	1	0	-0.002	-0.014	-0.001	0	t	25013	HVADIDKLID	Mus musculus
1	1.00	1	1	-0.02	-0.018	-0.027	-0.013	-0.02	t	144652	GMRGMKGLVY	Homo sapiens
1	1.00	1	1	-0.04	-0.04	-0.053	-0.04	-0.041	t	104515	HDCRPKKI	Mus musculus
1	1.00	1	1	-0.02	-0.018	-0.043	-0.023	-0.018	t	7367	CYGDWA	Homo sapiens
1	1.00	1	1	-0.02	-0.018	-0.043	-0.023	-0.018	t	7367	CYGDWA	Homo sapiens
1	1.00	1	1	-0.01	-0.007	-0.018	-0.01	-0.012	t	144610	DFFTYK	Mus musculus
1	1.00	1	1	0.05	0.05	0.05	0.057	0.05	t	112047	ASTRESG	Homo sapiens
1	1.00	1	1	0.01	0.01	0.007	0.013	0.009	t	36136	LGGVFT	Homo sapiens
1	1.00	1	1	-0.01	-0.01	-0.01	0	-0.009	t	115256	FRELKDLKGY	Homo sapiens
1	1.00	1	1	0.03	0.034	0.03	0.037	0.03	t	129024	KADQLYK	Homo sapiens
1	1.00	1	1	0	0.002	-0.007	0	-0.002	t	148481	YTRDLVYK	Rattus norvegicus

1	1.00	1	1	0.04	0.038	0.04	0.043	0.039	t	150850	GY	Mus musculus
1	1.00	1	1	-0.03	-0.028	-0.03	-0.023	-0.031	t	107366	FPPKPKD	Homo sapiens
1	1.00	1	1	0.04	0.042	0.035	0.037	0.038	t	114859	ICGTDGVTYT	Homo sapiens
1	1.00	1	1	-0.02	-0.021	-0.012	-0.013	-0.021	t	62149	SVHLF	Homo sapiens
1	1.00	1	1	0.04	0.04	0.027	0.04	0.041	t	98455	SGSVQFVPIQ	Mus musculus
1	1.00	1	1	0.04	0.035	0.029	0.037	0.039	t	61783	STNKAVVSLS	Bos taurus
1	1.00	1	1	0	-0.002	-0.012	0	0	t	100318	NAPKTFQFIN	Mus musculus
1	1.00	1	1	-0.04	-0.04	-0.041	-0.036	-0.041	t	118947	PFSAPPPA	Homo sapiens
1	1.00	1	1	0.01	0.008	0.007	0.013	0.01	t	78323	YSFRD	Mus musculus
1	1.00	1	1	-0.04	-0.04	-0.045	-0.04	-0.043	t	145831	IPLGTRP	Mus musculus
1	1.00	1	1	0.01	0.012	0.01	0.017	0.009	t	119592	HTFPAVLQ	Homo sapiens
1	1.00	1	1	-0.01	-0.004	-0.014	-0.009	-0.013	t	58780	SKAANLSIIK	MD
1	1.00	1	1	0.01	0.012	0.004	0.013	0.01	t	115153	MKGVVCTRIYEKV	Homo sapiens
1	1.00	1	1	-0.01	-0.013	-0.021	-0.01	-0.01	t	133629	LPLRF	Oryctolagus cuniculus
1	1.00	1	1	0.08	0.082	0.078	0.087	0.08	t	96215	EEEEAEDKED	Homo sapiens

1	1.00	1	1	0.01	0.014	-0.001	0.007	0.009	t	39782	LTAASV	Homo sapiens
1	1.00	1	1	0	0.002	0	0.007	-0.001	t	107479	KFNWYVD	Homo sapiens
1	1.00	1	1	0.05	0.036	0.041	0.049	0.05	t	63569	TETVNSDI	Macaca mulatta
1	1.00	1	1	0.05	0.053	0.05	0.05	0.048	t	134028	DDTIS	Mus musculu s
1	1.00	1	1	0	0	0	0.004	-0.003	t	23028	GVKYA	Homo sapiens
1	1.00	1	1	-0.03	-0.03	-0.03	-0.02	-0.029	t	115293	IMCVKKIL DK	Homo sapiens
1	1.00	1	1	0.01	0.013	-0.003	0.01	0.01	t	65105	TLTPENTL	Mus musculu s
1	1.00	1	1	0.02	0.018	0.019	0.02	0.016	t	134471	PKPEQ	Mus musculu s
1	1.00	1	1	0.07	0.068	0.071	0.073	0.07	t	142228	LYCYEQLN DSSE	Homo sapiens
1	1.00	1	1	0.03	0.032	0.021	0.031	0.03	t	21549	GNYDFWY QS	Homo sapiens
1	1.00	1	1	0.03	0.034	0.021	0.031	0.03	t	21549	GNYDFWY QS	Homo sapiens
1	1.00	1	1	0.06	0.06	0.059	0.063	0.06	t	34908	LAPLGE	Homo sapiens
1	1.00	1	1	0.01	0.008	0.01	0.011	0.007	t	130454	CLFPNNSY C	Mus musculu s
1	1.00	1	1	-0.01	-0.006	-0.013	-0.009	-0.01	t	19644	GFVPSM	Homo sapiens

1	1.00	1	1	0.01	0.01	0.001	0.01	0.01	t	20678	GKRPE	Mus musculus
1	1.00	1	1	-0.02	-0.016	-0.016	-0.02	-0.025	t	123278	GYLKDLPTT	Ovis aries
1	1.00	1	1	0.06	0.06	0.06	0.06	0.056	t	141067	PLSLEPDP	Mus musculus
1	1.00	1	1	0.03	0.033	0.026	0.03	0.03	t	139248	SFAGTVIE	Mus musculus
1	1.00	1	1	-0.06	-0.06	-0.073	-0.06	-0.061	t	104515	HDCRPKKI	Mus musculus
1	1.00	1	1	0.02	0.02	0.011	0.02	0.019	t	113517	HLYADGLTD	Mus musculus
1	1.00	1	1	0.01	0.012	0.003	0.017	0.01	t	156970	VERHQ	Homo sapiens
1	1.00	1	1	0.02	0.02	0.017	0.02	0.018	t	78252	PVQNLT	Mus musculus
1	1.00	1	1	0.01	0.006	0.004	0.01	0.008	t	6068	CCPDKNKS	Mus musculus
1	1.00	1	1	-0.02	-0.016	-0.037	-0.023	-0.02	t	53109	RAGVCY	Homo sapiens
1	1.00	1	1	0.06	0.06	0.047	0.057	0.06	t	147041	IPEQ	Homo sapiens
1	1.00	1	1	0.05	0.046	0.041	0.049	0.05	t	70664	VQVVYDYQ	Oryctolagus cuniculus
1	1.00	1	1	0	0	-0.009	0	0	t	134343	DLYIK	Mus musculus
1	1.00	1	1	0.03	0.03	0.03	0.03	0.03	t	131365	ATLVCLI	Homo sapiens
1	1.00	1	1	0	0.002	-0.002	0.007	0.001	t	112119	FYNTVLK	Homo sapiens

1	1.00	1	1	0.02	0.02	0.025	0.02	0.015	t	107378	GELP	Rattus norvegicus
1	1.00	1	1	-0.01	-0.01	-0.01	-0.01	-0.013	t	119461	GVQGGMV	Homo sapiens
1	1.00	1	1	0.03	0.03	0.03	0.04	0.03	t	114757	CLLCAYSIEF	Homo sapiens
1	1.00	1	1	-0.05	-0.048	-0.051	-0.043	-0.051	t	117526	HGIIKECT	Homo sapiens
1	1.00	1	1	0	0.002	-0.007	0.007	0	t	121117	ADGKKIDG	Homo sapiens
1	1.00	1	1	0.05	0.048	0.038	0.05	0.05	t	100451	QYTHQDEIYE	Mus musculus
1	1.00	1	1	0	0.002	-0.017	0.003	0.002	t	96967	TKEKPTPPVK	Homo sapiens(human)
1	1.00	1	1	0.05	0.05	0.048	0.051	0.047	t	116085	EPANPSEK	Mus musculus
1	1.00	1	1	0.01	0.01	0.005	0.012	0.008	t	117973	LCG	Homo sapiens
1	1.00	1	1	-0.01	-0.014	-0.015	-0.007	-0.01	t	97169	ADPVATKPL	Oryctolagus cuniculus
1	1.00	1	1	-0.04	-0.038	-0.047	-0.037	-0.041	t	115658	VGWYRPPFSRVV	Homo sapiens
1	1.00	1	1	0	0	-0.007	0	-0.002	t	127196	TEVPFRLM	Rattus norvegicus
1	1.00	1	1	-0.01	-0.01	-0.01	-0.004	-0.011	t	157292	GAIIGLM	Homo sapiens
1	1.00	1	1	0	0.004	-0.014	0.003	0.002	t	64769	TLEEFSAKL	Homo sapiens
1	1.00	1	1	0.03	0.027	0.023	0.03	0.029	t	138040	SLGQNVLS	Oryctolagus cuniculus

1	1.00	1	1	0.03	0.016	0.023	0.03	0.029	t	138040	SLGQNVLS	Oryctolagus cuniculus
1	1.00	1	1	-0.02	-0.022	-0.028	-0.021	-0.02	t	27416	ILYAVFK	Mus musculus
1	1.00	1	1	0.06	0.06	0.047	0.057	0.06	t	147041	IPEQ	Homo sapiens
1	1.00	1	1	0.04	0.042	0.028	0.037	0.04	t	115060	GYLEQL	Homo sapiens
1	1.00	1	1	0.02	0.02	0.017	0.03	0.02	t	98740	EALEKFDKAL	Homo sapiens
1	1.00	1	1	0	0.002	-0.007	0.003	0	t	144783	SHVVTMLDNF	Homo sapiens
1	1.00	1	1	-0.04	-0.038	-0.04	-0.039	-0.043	t	111055	VYDFAFQDL	Homo sapiens
1	1.00	1	1	-0.04	-0.038	-0.04	-0.039	-0.043	t	111055	VYDFAFQDL	Homo sapiens
1	1.00	1	1	-0.02	-0.022	-0.029	-0.019	-0.021	t	73889	YFRTLEDAAH	Mus musculus
1	1.00	1	1	-0.02	-0.022	-0.029	-0.019	-0.021	t	73889	YFRTLEDAAH	Mus musculus
1	1.00	1	1	0.11	0.113	0.103	0.11	0.11	t	140014	DSDVGE	Mus musculus
1	1.00	1	1	0.02	0.024	0.028	0.027	0.017	t	124205	GPLQLGM	Homo sapiens
1	1.00	1	1	0.08	0.078	0.066	0.079	0.081	t	46777	PAEGEK	Mus musculus
1	1.00	1	1	0.03	0.032	0.023	0.037	0.03	t	141673	IDEYCVQQLK	Homo sapiens
1	1.00	1	1	0.02	0.022	0.013	0.021	0.02	t	22669	GTGP	Homo sapiens

1	1.00	1	1	0.01	0.014	0.01	0.017	0.008	t	126903	QVINVREG	Homo sapiens
1	1.00	1	1	0.02	0.02	0.008	0.02	0.02	t	30799	KFNPDLAPG	Homo sapiens
1	1.00	1	1	-0.03	-0.027	-0.04	-0.03	-0.031	t	156553	YSTCYFIM	Mus musculus
1	1.00	1	1	-0.02	-0.02	-0.023	-0.01	-0.018	t	116732	GLGFYG	Homo sapiens
1	1.00	1	1	-0.04	-0.04	-0.04	-0.03	-0.039	t	115197	CVKKILDKVG	Homo sapiens
1	1.00	1	1	0	0.002	0	0.007	-0.003	t	134783	RKLQKSTHLLIRKLP	Homo sapiens
1	1.00	1	1	0.06	0.06	0.06	0.06	0.057	t	120082	LPTDSGEK	Rattus norvegicus
1	1.00	1	1	-0.01	-0.006	-0.01	-0.003	-0.011	t	131166	YSCRHGYELQ	Homo sapiens
1	1.00	1	1	0	0	0	0.007	0.001	t	112283	RDGAWFA	Homo sapiens
1	1.00	1	1	0.03	0.032	0.035	0.037	0.029	t	112414	YLSSFTF	Homo sapiens
1	1.00	1	1	0.01	0.014	0.007	0.011	0.007	t	103297	IWKLIKDMLILPYLD	Homo sapiens
1	1.00	1	1	0.06	0.062	0.05	0.061	0.062	t	30265	KDTTSDYE	Homo sapiens
1	1.00	1	1	-0.01	-0.012	-0.014	-0.01	-0.012	t	20453	GITDVF	Mus musculus
1	1.00	1	1	0.04	0.04	0.04	0.04	0.038	t	139524	ISSQT	Mus musculus

1	1.00	1	1	-0.03	-0.03	-0.041	-0.03	-0.03	t	60039	SPFART	Homo sapiens
1	1.00	1	1	0.02	0.032	0.019	0.019	0.018	t	41126	MASLIYRQLL	Gallus gallus
1	1.00	1	1	0.04	0.038	0.022	0.041	0.042	t	60798	SRSESSIENF	Homo sapiens
1	1.00	1	1	0.01	0.015	-0.005	0.013	0.012	t	97065	VPKVTKEKPT	Homo sapiens(human)
1	1.00	1	1	0.07	0.07	0.058	0.07	0.071	t	122569	SFQDENFVFEEF	Rattus norvegicus
1	1.00	1	1	0.03	0.034	0.028	0.03	0.027	t	1448	AGAPDRARGQYP	Mus musculus
1	1.00	1	1	0	0.002	-0.015	-0.003	0.002	t	39217	LRSVYQ	Homo sapiens
1	1.00	1	1	0.02	0.022	0.02	0.027	0.019	t	119632	QYNSTYRV	Homo sapiens
1	1.00	1	1	0.01	0.012	0.01	0.013	0.007	t	94685	SICSNNPTCWAI	Homo sapiens
1	1.00	1	1	-0.02	-0.018	-0.025	-0.019	-0.02	t	19644	GFVPSM	Homo sapiens
1	1.00	1	1	0	0	-0.018	0	0.003	t	52124	QQQPP	Homo sapiens
1	1.00	1	1	0.03	0.03	0.024	0.03	0.028	t	48876	PPPQAPYQGY	Mus musculus
1	1.00	1	1	0.04	0.042	0.03	0.041	0.041	t	58963	SKVPEDTL	Homo sapiens

1	1.00	1	1	-0.02	-0.023	-0.033	-0.02	-0.02	t	41982	MLKSLDNL	Oryctolagus cuniculus
1	1.00	1	1	-0.02	-0.02	-0.019	-0.013	-0.022	t	40340	LVFKT	Homo sapiens
1	1.00	1	1	-0.01	-0.008	-0.017	-0.007	-0.011	t	115661	VVHLYRNGKDQD	Homo sapiens
1	1.00	1	1	-0.01	-0.008	-0.027	-0.007	-0.007	t	110932	LNLKIDGLKISV	Homo sapiens
1	1.00	1	1	0.03	0.03	0.023	0.03	0.029	t	107702	YVDGVQV	Homo sapiens
1	1.00	1	1	0.01	0.01	0.003	0.01	0.01	t	14702	EVGKVA	Homo sapiens
1	1.00	1	1	0	-0.001	-0.006	0.001	0	t	71545	VTV	Bos taurus
1	1.00	1	1	-0.01	-0.006	-0.01	-0.003	-0.008	t	107421	GPSVFLF	Homo sapiens
1	1.00	1	1	-0.06	-0.057	-0.071	-0.06	-0.062	t	102784	RWWS	Mus musculus
1	1.00	1	1	0.02	0.022	0.02	0.027	0.019	t	119593	HYTQKSLS	Homo sapiens
1	1.00	1	1	0	0.002	-0.013	0.001	0	t	34082	KVKVEYQKTMGSM	Homo sapiens
1	1.00	1	1	0.01	0.012	-0.007	0.007	0.01	t	33936	KTYSQYKV	Homo sapiens
1	1.00	1	1	0.03	0.03	0.022	0.033	0.03	t	110964	PNLKLNNGTDYK	Homo sapiens
1	1.00	1	1	-0.01	-0.005	-0.02	-0.007	-0.01	t	110839	ETTSQMIVKDIK	Homo sapiens

1	1.00	1	1	0.01	0.01	-0.002	0.01	0.01	t	48620	PNDPPP	Mus musculus
1	1.00	1	1	0.01	0.01	-0.002	0.01	0.01	t	48620	PNDPPP	Mus musculus
1	1.00	1	1	0	0	0	0	-0.003	t	101053	MASPGS	Homo sapiens
1	1.00	1	1	0.04	0.038	0.036	0.039	0.038	t	3698	APPQA	Mus musculus
1	1.00	1	1	-0.03	-0.028	-0.039	-0.027	-0.03	t	77929	KFHRVIKDF	Homo sapiens
1	1.00	1	1	-0.02	-0.023	-0.027	-0.02	-0.022	t	137425	KFP	Oryctolagus cuniculus
1	1.00	1	1	0.03	0.03	0.03	0.03	0.031	t	107784	DGSFFLY	Homo sapiens
1	1.00	1	1	0.03	0.032	0.03	0.037	0.029	t	119576	EYKCKVSN	Homo sapiens
1	1.00	1	1	0.03	0.032	0.013	0.031	0.032	t	96940	TAPTKPTYET	Homo sapiens(human)
1	1.00	1	1	-0.03	-0.03	-0.045	-0.027	-0.028	t	53548	REKMITEF	Homo sapiens
1	1.00	1	1	0.01	0.012	0.01	0.017	0.009	t	119619	NNYKTTPP	Homo sapiens
1	1.00	1	1	0.08	0.084	0.08	0.087	0.078	t	129058	KEESEESDEDMGFGL	Homo sapiens
1	1.00	1	1	-0.01	-0.01	-0.026	-0.012	-0.01	t	45679	NRGFDART	Homo sapiens
1	1.00	1	1	-0.01	-0.007	-0.017	-0.01	-0.013	t	75222	YNSSRSYWT	Mus musculus

1	1.00	1	1	0.04	0.04	0.04	0.04	0.036	t	141067	PLSLEPDP	Mus musculus
1	1.00	1	1	-0.01	-0.008	-0.012	-0.007	-0.011	t	131580	PDRFSGS	Homo sapiens
1	1.00	1	1	0.01	0.011	0.003	0.009	0.01	t	73973	YFYVGLDY	Equus caballus
1	1.00	1	1	0.01	0.008	0.003	0.009	0.01	t	73973	YFYVGLDY	Mus musculus
1	1.00	1	1	0.14	0.14	0.125	0.14	0.143	t	144880	EE	Mus musculus
1	1.00	1	1	0.07	0.067	0.064	0.07	0.068	t	50396	QAYQQGVTVD	Oryctolagus cuniculus
1	1.00	1	1	0.04	0.042	0.035	0.037	0.038	t	115901	PQSVLSLSQS	Homo sapiens
1	1.00	1	1	0	0.002	-0.011	0.003	0	t	159063	RKAADTFNFKTFFHT	Homo sapiens
1	1.00	1	1	-0.07	-0.072	-0.09	-0.069	-0.068	t	96386	IFDVTT	Mus musculus
1	1.00	1	1	0.02	0.023	0.014	0.02	0.018	t	9591	DPAKAAFD	Mus musculus
1	1.00	1	1	0.02	0.02	0.012	0.02	0.02	t	63832	TGGAGYFY	Mus musculus
1	1.00	1	1	0.08	0.08	0.075	0.083	0.08	t	65604	TPIDTT	Homo sapiens
1	1.00	1	1	-0.02	-0.02	-0.02	-0.013	-0.023	t	158587	EFGTFLW	Homo sapiens
1	1.00	1	1	0.05	0.046	0.033	0.043	0.051	t	110768	ANPENYFT	Oryctolagus cuniculus

1	1.00	1	1	0.05	0.046	0.033	0.043	0.051	t	110768	ANPENYFT	Oryctolagus cuniculus
1	1.00	1	1	0.01	0.008	0.008	0.013	0.01	t	97167	AANYTTAV	Mus musculus
1	1.00	1	1	0	0	0	0.006	-0.001	t	157178	EASNCF	Homo sapiens
1	1.00	1	1	0.03	0.03	0.018	0.03	0.03	t	133552	ECAYCLTI	Mus musculus
1	1.00	1	1	0.02	0.018	0.02	0.021	0.017	t	130457	CRQPGNNYC	Mus musculus
1	1.00	1	1	0.01	0.008	0.003	0.009	0.008	t	43330	NAVFHFDQNG	Mus musculus
1	1.00	1	1	-0.05	-0.052	-0.06	-0.051	-0.052	t	12992	ELDLQARR	Mus musculus
1	1.00	1	1	-0.01	-0.008	-0.015	-0.01	-0.012	t	127196	TEVPFRLM	Rattus norvegicus
1	1.00	1	1	0.03	0.032	0.035	0.037	0.029	t	112058	AYWGQGT	Homo sapiens
1	1.00	1	1	0.01	0.012	-0.011	0.007	0.012	t	10164	DSLKEKLQ	Homo sapiens
1	1.00	1	1	0.04	0.046	0.029	0.04	0.04	t	92873	GVDTIYGNDG	Ovis aries
1	1.00	1	1	-0.02	-0.018	-0.018	-0.013	-0.023	t	120612	IFNLFSTKDSSAAW	Homo sapiens
1	1.00	1	1	0	0.004	-0.011	0.001	0	t	9982	DRHFLN	Homo sapiens
1	1.00	1	1	0.03	0.03	0.028	0.033	0.03	t	3685	APPLPP	Homo sapiens

1	1.00	1	1	0.02	0.018	0.011	0.023	0.02	t	8143	DEREECKC	Mus musculus
1	1.00	1	1	-0.01	-0.007	-0.018	-0.01	-0.011	t	144610	DFFTYK	Mus musculus
1	1.00	1	1	0	-0.009	-0.013	0	0.002	t	64561	TKFLGKNK	Macaca mulatta
1	1.00	1	1	-0.04	-0.04	-0.043	-0.03	-0.041	t	98885	LPMHIRLSFN	Homo sapiens
1	1.00	1	1	0.01	0.012	0.001	0.007	0.008	t	10794	DVYSFSLA	Homo sapiens
1	1.00	1	1	0.02	0.023	0.002	0.02	0.022	t	66303	TSLLSNS	Mus musculus
1	1.00	1	1	0.03	0.032	0.03	0.037	0.03	t	122564	RTAGFDIKLIDT	Homo sapiens
1	1.00	1	1	0	0.004	-0.006	0.001	-0.002	t	17998	FTKWTWETKGDL	Homo sapiens
1	1.00	1	1	-0.04	-0.04	-0.049	-0.04	-0.041	t	118764	CYGSFARPTN	Mus musculus
1	1.00	1	1	0.04	0.038	0.029	0.039	0.04	t	96854	SAPSISTE	Mus musculus
1	1.00	1	1	-0.02	-0.017	-0.025	-0.02	-0.022	t	126828	PKYA	Mus musculus
1	1.00	1	1	0.01	0.007	-0.003	0.01	0.01	t	1445	AGAKSAIL	Oryctolagus cuniculus
1	1.00	1	1	0	0	0	0.007	0.001	t	112314	SGVPDRF	Homo sapiens
1	1.00	1	1	0	0.002	-0.007	-0.003	-0.003	t	116039	YPSYGLNYYQ	Homo sapiens

1	1.00	1	1	0	0.002	-0.007	-0.003	-0.003	t	116039	YPSYGLNYYQ	Homo sapiens
1	1.00	1	1	0.02	0.022	0.013	0.021	0.019	t	138057	TPGTEYTV	Homo sapiens
1	1.00	1	1	0.02	0.01	0.013	0.021	0.019	t	138057	TPGTEYTV	Homo sapiens
1	1.00	1	1	0.02	0.018	0.02	0.023	0.02	t	49295	PSAPPL	Homo sapiens
1	1.00	1	1	0.02	0.018	0.02	0.023	0.02	t	49295	PSAPPL	Homo sapiens
1	1.00	1	1	0	0.002	0.005	0.007	0	t	112814	PEQPPIH	Homo sapiens
1	1.00	1	1	0.05	0.047	0.045	0.05	0.048	t	49408	PSISEGPSYEFS	Oryctolagus cuniculus
1	1.00	1	1	0.04	0.04	0.033	0.04	0.04	t	95692	PTTSDV	Mus musculus
1	1.00	1	1	-0.01	-0.01	-0.017	-0.004	-0.01	t	96762	PVKPTAPTKP	Homo sapiens(human)
1	1.00	1	1	0.01	0.012	0.005	0.007	0.008	t	115900	PQRDMPIQAF	Homo sapiens
1	1.00	1	1	0.03	0.033	0.034	0.03	0.025	t	122917	TKVYSTDIFFILEITDNPY	Mus musculus
1	1.00	1	1	-0.03	-0.026	-0.03	-0.023	-0.033	t	134707	KLPFSRLAREICVKF	Homo sapiens
1	1.00	1	1	0.01	0.01	0.01	0.02	0.01	t	114757	CLLCAYSIEF	Homo sapiens
1	1.00	1	1	0	0.002	-0.011	0.003	0	t	159063	RKAADTFNFKTFFHT	Homo sapiens
1	1.00	1	1	0.01	0.008	-0.007	0.007	0.01	t	45052	NNAYNMSI	Mus musculus

1	1.00	1	1	0.02	0.024	0.02	0.021	0.017	t	48778	PPFSLSPV PTL	Homo sapiens
1	1.00	1	1	-0.02	-0.024	-0.021	-0.019	-0.023	t	75686	YRYDSRPP	Oryctola gus cuniculu s
1	1.00	1	1	-0.02	-0.022	-0.021	-0.019	-0.023	t	75686	YRYDSRPP	Mus musculu s
1	1.00	1	1	0.02	0.02	0.01	0.02	0.02	t	137759	NQEDAEAI THLF	Mus musculu s
1	1.00	1	1	0.02	0.02	0.01	0.02	0.02	t	137759	NQEDAEAI THLF	Mus musculu s
1	1.00	1	1	0.02	0.02	0.012	0.02	0.02	t	45629	NQSTVK	Mus musculu s
1	1.00	1	1	-0.01	-0.01	-0.01	-0.003	-0.01	t	112151	GWIRQRS	Homo sapiens
1	1.00	1	1	-0.01	-0.01	-0.019	-0.01	-0.012	t	67524	VAALNRRI QLLEEDL	Homo sapiens
1	1.00	1	1	-0.02	-0.024	-0.033	-0.021	-0.02	t	41982	MLKSLDNL	Oryctola gus cuniculu s
1	1.00	1	1	-0.01	-0.014	-0.014	-0.009	-0.012	t	30294	KEAIVTK	Bos taurus
1	1.00	1	1	0.03	0.034	0.016	0.027	0.03	t	147041	IPEQ	Homo sapiens
1	1.00	1	1	-0.02	-0.022	-0.029	-0.02	-0.021	t	73889	YFRTLEDA AH	Mus musculu s
1	1.00	1	1	-0.02	-0.02	-0.029	-0.02	-0.021	t	73889	YFRTLEDA AH	Mus musculu s

1	1.00	1	1	0.01	0.012	0.003	0.017	0.01	t	144589	ALKHLPNDPM	Homo sapiens
1	1.00	1	1	0.08	0.084	0.073	0.087	0.083	t	140020	ENTNIT	Homo sapiens
1	1.00	1	1	0.04	0.044	0.035	0.047	0.041	t	122282	AYLVGLFEDTNLCAI	Homo sapiens
1	1.00	1	1	0.02	0.022	0.013	0.02	0.019	t	126982	RREFEFYG	Homo sapiens
1	1.00	1	1	-0.05	-0.046	-0.057	-0.043	-0.051	t	115633	PPFSRVVHLYRN	Homo sapiens
1	1.00	1	1	0.04	0.042	0.029	0.047	0.042	t	133921	QLELTEGMRFDKGYI	Homo sapiens
1	1.00	1	1	0.06	0.059	0.05	0.06	0.06	t	21504	GNQSLLET	Anas platyrhynchos
1	1.00	1	1	0.01	0.01	0.003	0.01	0.009	t	130919	KFPEDFES	Homo sapiens
1	1.00	1	1	0.02	0.032	0.019	0.019	0.018	t	41126	MASLIYRQLL	Gallus gallus
1	1.00	1	1	0.05	0.047	0.044	0.05	0.049	t	105979	NIEADVNT	Oryctolagus cuniculus
1	1.00	1	1	0.01	0.007	0.01	0.01	0.007	t	135566	KCSRLYTACVY	Oryctolagus cuniculus
1	1.00	1	1	0.03	0.03	0.023	0.03	0.028	t	132766	YRAYATEP	Mus musculus
1	1.00	1	1	0.01	0.01	-0.001	0.01	0.009	t	113800	QYTPTTQPYPRVT	Mus musculus
1	1.00	1	1	0.05	0.052	0.055	0.057	0.048	t	122607	TEAFLAEMEK	Homo sapiens

1	1.00	1	1	0.01	0.013	0.004	0.01	0.007	t	43548	NDYEDRYYRE	Mus musculus
1	1.00	1	1	0.03	0.03	0.022	0.03	0.03	t	6205	CETEFSKLY	Mus musculus
1	1.00	1	1	0.03	0.03	0.022	0.03	0.03	t	6205	CETEFSKLY	Mus musculus
1	1.00	1	1	0.03	0.026	0.021	0.029	0.03	t	70665	VQVVYDYQHK	Oryctolagus cuniculus
1	1.00	1	1	0	0.002	0	0.007	-0.001	t	119639	SCSVMHEA	Homo sapiens
1	1.00	1	1	-0.02	-0.02	-0.032	-0.02	-0.02	t	49975	PVSSLTML	Oryctolagus cuniculus
1	1.00	1	1	0.04	0.038	0.04	0.039	0.036	t	62289	SVPSSSS	Mus musculus
1	1.00	1	1	0.01	0.01	-0.003	0.007	0.01	t	147225	QLQPFP	Homo sapiens
1	1.00	1	1	-0.01	-0.008	-0.017	-0.007	-0.011	t	115661	VVHLYRNGKDQD	Homo sapiens
1	1.00	1	1	0.03	0.03	0.018	0.03	0.03	t	58275	SGV	Homo sapiens
1	1.00	1	1	0.03	0.03	0.017	0.024	0.028	t	70439	VPPVIKQVSA	Homo sapiens
1	1.00	1	1	0.01	0.008	0.004	0.007	0.008	t	50666	QEPIVA	Mus musculus
1	1.00	1	1	0.03	0.03	0.022	0.032	0.03	t	110964	PNLKLNNGTDYK	Homo sapiens
1	1.00	1	1	0.02	0.018	0.019	0.02	0.017	t	134471	PKPEQ	Mus musculus

1	1.00	1	1	0.02	0.023	0.015	0.019	0.017	t	157702	NKFAVETL	Chlorocebus aethiops
1	1.00	1	1	-0.01	-0.01	-0.025	-0.013	-0.01	t	54340	RKEVMK	Homo sapiens
1	1.00	1	1	0.02	0.02	0.005	0.018	0.021	t	98780	FPQPQLPYSQPQ	Homo sapiens
1	1.00	1	1	-0.02	-0.014	-0.02	-0.021	-0.025	t	131294	DWGLPIPSPSP	Homo sapiens
1	1.00	1	1	0.02	0.022	0.011	0.017	0.018	t	114823	FNPVCGTDGV	Homo sapiens
1	1.00	1	1	-0.02	-0.02	-0.025	-0.014	-0.021	t	107218	PAGGPGFP	Rattus norvegicus
1	1.00	1	1	0.01	0.014	0.01	0.017	0.012	t	107296	APIEKTI	Homo sapiens
1	1.00	1	1	0.02	0.02	0.011	0.023	0.022	t	70038	VMYASSG	Homo sapiens
1	1.00	1	1	0.01	0.01	-0.002	0.01	0.01	t	120839	NLTEFQHAFS	Mus musculus
1	1.00	1	1	0.03	0.03	0.014	0.03	0.031	t	120867	PFDQDLSKKH	Mus musculus
1	1.00	1	1	0.02	0.02	0.011	0.024	0.02	t	135900	ATCRFYTLDSIK	Homo sapiens
1	1.00	1	1	0.04	0.038	0.035	0.037	0.037	t	115827	KKNQDKTEIP	Homo sapiens
1	1.00	1	1	0.04	0.044	0.035	0.037	0.037	t	115827	KKNQDKTEIP	Homo sapiens
1	1.00	1	1	0.02	0.024	0.008	0.02	0.02	t	61945	STTNKDK	Cavia porcellus

1	1.00	1	1	0.02	0.02	0.008	0.02	0.02	t	61945	STTNKDK	Mus musculus
1	1.00	1	1	-0.02	-0.02	-0.02	-0.01	-0.019	t	115197	CVKKILDKVG	Homo sapiens
1	1.00	1	1	0.07	0.068	0.07	0.069	0.07	t	61741	STLPETTVV	Mus musculus
1	1.00	1	1	0.01	0.014	0	0.011	0.01	t	155473	PSTP	Homo sapiens
1	1.00	1	1	-0.01	-0.012	-0.019	-0.013	-0.011	t	109349	IPVDLL	Mus musculus
1	1.00	1	1	0	0	0	0.011	-0.002	t	18588	GAGGGAGGAGAGGGAGGAGA	Homo sapiens
1	1.00	1	1	0.04	0.028	0.034	0.04	0.038	t	63252	TDVFSS	Mus musculus
1	1.00	1	1	-0.03	-0.026	-0.032	-0.029	-0.032	t	29755	KAANLSIIK	MD
1	1.00	1	1	-0.03	-0.026	-0.032	-0.029	-0.032	t	29755	KAANLSIIK	MD
1	1.00	1	1	0.07	0.07	0.07	0.07	0.066	t	141004	EPDP	Mus musculus
1	1.00	1	1	0.04	0.043	0.039	0.04	0.038	t	122113	IIASTEKLP	Mus musculus
1	1.00	1	1	-0.04	-0.042	-0.048	-0.041	-0.04	t	27416	ILYAVFK	Mus musculus
1	1.00	1	1	-0.04	-0.042	-0.048	-0.041	-0.04	t	27416	ILYAVFK	Mus musculus
1	1.00	1	1	0.02	0.018	0.013	0.023	0.019	t	107166	GPPGPQG	Mus musculus
1	1.00	1	1	0.04	0.042	0.035	0.041	0.039	t	14173	ESIYQC	Homo sapiens
1	1.00	1	1	0.01	0.013	0.01	0.01	0.007	t	134702	KEKKYS	Mus musculus

1	1.00	1	1	0.14	0.138	0.125	0.139	0.143	t	144880	EE	Mus musculus
1	1.00	1	1	-0.02	-0.02	-0.026	-0.02	-0.022	t	56075	RTHRQLY	Mus musculus
1	1.00	1	1	0	-0.004	-0.014	-0.001	0	t	10580	DVENSMLQAN	Oryctolagus cuniculus
1	1.00	1	1	0.04	0.04	0.039	0.04	0.037	t	122913	TGEPVELK	Oryctolagus cuniculus
1	1.00	1	1	0.04	0.026	0.031	0.039	0.04	t	65098	TLTKTSLE	Macaca mulatta
1	1.00	1	1	0	0.002	0	0.007	0	t	107479	KFNWYVD	Homo sapiens
1	1.00	1	1	0.02	0.022	0.003	0.02	0.023	t	110951	NDGYFWEDGTNG	Homo sapiens
1	1.00	1	1	-0.02	-0.027	-0.02	-0.017	-0.023	t	48839	PPNKPNNDFHFE	Homo sapiens
1	1.00	1	1	0	0	0	0	-0.003	t	107368	FSSAP	Rattus norvegicus
1	1.00	1	1	-0.01	-0.01	-0.011	0	-0.01	t	106429	FVLPWRI	Homo sapiens
1	1.00	1	1	0.03	0.037	0.026	0.033	0.028	t	48308	PLENRVLT	MD
1	1.00	1	1	0.03	0.037	0.026	0.033	0.028	t	48308	PLENRVLT	MD
1	1.00	1	1	0.03	0.03	0.034	0.03	0.025	t	122932	VCTPKSDEFNPCEDIMGYK	Mus musculus

1	1.00	1	1	0.01	0.013	-0.003	0.01	0.011	t	65105	TLTPENTL	Mus musculus
1	1.00	1	1	0.03	0.026	0.023	0.029	0.029	t	138040	SLGQNVLS	Oryctolagus cuniculus
1	1.00	1	1	0.03	0.03	0.019	0.027	0.03	t	53270	RCFDST	Homo sapiens
1	1.00	1	1	0	0.004	0.002	0.007	-0.001	t	119648	SWNSGALT	Homo sapiens
1	1.00	1	1	-0.05	-0.048	-0.051	-0.043	-0.05	t	117526	HGIIKECT	Homo sapiens
1	1.00	1	1	0.06	0.065	0.051	0.061	0.06	t	3572	APGITTAIEQ	Homo sapiens
1	1.00	1	1	-0.02	-0.018	-0.018	-0.013	-0.022	t	120464	EDSILAVRKYFQRI	Homo sapiens
1	1.00	1	1	0	-0.004	-0.01	-0.001	0	t	8599	DIDFDSVPQI	Oryctolagus cuniculus
1	1.00	1	1	0.03	0.026	0.023	0.03	0.03	t	12459	EIIEQLDVTT	Oryctolagus cuniculus
1	1.00	1	1	0.03	0.03	0.022	0.027	0.03	t	53984	RGSQGF	Homo sapiens
1	1.00	1	1	0	0.004	0	0	-0.001	t	139840	PAAFAFL	Ovis aries
1	1.00	1	1	0.03	0.036	0.018	0.031	0.03	t	30140	KDEPNGRASDT	Equus caballus
1	1.00	1	1	0.03	0.026	0.019	0.03	0.03	t	25013	HVADIDKLID	Mus musculus

1	1.00	1	1	0.02	0.02	0.014	0.02	0.018	t	79524	CHTTATIPSSAVTC	Mus musculus
1	1.00	1	1	0.02	0.02	0.014	0.02	0.018	t	79524	CHTTATIPSSAVTC	Mus musculus
1	1.00	1	1	0	0.002	0.002	0.007	0	t	119671	WLNGKEYK	Homo sapiens
1	1.00	1	1	-0.02	-0.017	-0.02	-0.02	-0.022	t	79215	FNPVYP	Mus musculus
1	1.00	1	1	0.02	0.022	0.02	0.02	0.019	t	104014	KPQ	Homo sapiens
1	1.00	1	1	-0.01	-0.008	-0.011	-0.007	-0.01	t	24722	HSAPLG	Homo sapiens
1	1.00	1	1	-0.01	-0.012	-0.011	-0.007	-0.01	t	24722	HSAPLG	Homo sapiens
1	1.00	1	1	0.02	0.026	0.009	0.02	0.02	t	92864	GSKFNDAFNG	Ovis aries
1	1.00	1	1	0	-0.002	-0.014	-0.001	0	t	133543	DKAFAV	Mus musculus
1	1.00	1	1	0.02	0.018	0	0.017	0.022	t	17261	FPAYGGMQQILA	Sus scrofa
1	1.00	1	1	0.04	0.037	0.025	0.04	0.042	t	38606	LPNVGITQ	Oryctolagus cuniculus
1	1.00	1	1	0.04	0.034	0.025	0.04	0.042	t	38606	LPNVGITQ	Oryctolagus cuniculus
1	1.00	1	1	0	-0.003	-0.003	0	-0.002	t	94484	DDYRQK	Oryctolagus cuniculus
1	1.00	1	1	0.01	0.014	0.001	0.007	0.008	t	6314	CGLRAPQRCDLDVES	Homo sapiens

1	1.00	1	1	-0.01	-0.01	-0.01	-0.003	-0.01	t	112038	ARMRDGA	Homo sapiens
1	1.00	1	1	0.03	0.032	0.028	0.033	0.032	t	131878	FLMLVGG STL	Homo sapiens
1	1.00	1	1	0.02	0.02	0.012	0.02	0.02	t	45630	NQSTVKT	Mus musculu s
1	1.00	1	1	-0.01	-0.012	-0.01	-0.009	-0.013	t	130454	CLFPNNSY C	Mus musculu s
1	1.00	1	1	-0.01	-0.012	-0.027	-0.017	-0.01	t	67605	VAGLEK	Mus musculu s
1	1.00	1	1	0.02	0.02	0.013	0.02	0.02	t	35341	LDYSPAFS	Mus musculu s
1	1.00	1	1	0.02	0.023	0.013	0.02	0.02	t	35341	LDYSPAFS	Equus caballus
1	1.00	1	1	0	0	-0.012	0	0	t	120942	SGVATKGL NE	Mus musculu s
1	1.00	1	1	0	-0.002	-0.009	-0.001	-0.002	t	7768	DDEIYGND GHP	Mus musculu s
1	1.00	1	1	-0.01	-0.008	-0.01	-0.014	-0.016	t	23174	GVTRPS	Homo sapiens
1	1.00	1	1	0.01	0.01	0.015	0.017	0.009	t	125027	EHIEL	Homo sapiens
1	1.00	1	1	0.02	0.02	0.02	0.03	0.02	t	114885	LCAYSIEFG T	Homo sapiens
1	1.00	1	1	0	0.002	-0.005	0.001	-0.001	t	38662	LPQVVM	Homo sapiens
1	1.00	1	1	0.02	0.023	0.022	0.02	0.016	t	146067	VPSTSLSG Y	Mus musculu s

1	1.00	1	1	0.01	0.008	0.01	0.017	0.01	t	123259	GQPPYMPP	Homo sapiens
1	1.00	1	1	0.01	0.01	0.01	0.02	0.01	t	114752	AYSIEFGTNI	Homo sapiens
1	1.00	1	1	0	-0.003	-0.007	0	0	t	36162	LGIHE	Oryctolagus cuniculus
1	1.00	1	1	0.04	0.038	0.026	0.043	0.042	t	25277	IAGAGD	Mus musculus
1	1.00	1	1	0.03	0.03	0.03	0.03	0.027	t	9588	DPAFSL	Mus musculus
1	1.00	1	1	-0.03	-0.027	-0.04	-0.03	-0.03	t	156553	YSTCYFIM	Mus musculus
1	1.00	1	1	0.04	0.042	0.03	0.041	0.04	t	19202	GEAGPGVA	Homo sapiens
1	1.00	1	1	0.03	0.03	0.03	0.03	0.031	t	131365	ATLVCLI	Homo sapiens
1	1.00	1	1	0.04	0.042	0.04	0.043	0.039	t	114207	FLGELTSSEVATEV	Homo sapiens
1	1.00	1	1	0.01	0.01	0.01	0.02	0.01	t	108595	PVSSTRGNM	Homo sapiens
1	1.00	1	1	-0.03	-0.03	-0.035	-0.024	-0.03	t	109310	IAGAPGFP	Rattus norvegicus
1	1.00	1	1	-0.03	-0.034	-0.034	-0.03	-0.033	t	47859	PHWA	Mus musculus
1	1.00	1	1	0.02	0.006	0.013	0.02	0.019	t	138057	TPGTEYTV	Oryctolagus cuniculus
1	1.00	1	1	0.02	0.017	0.013	0.02	0.019	t	138057	TPGTEYTV	Oryctolagus cuniculus

1	1.00	1	1	0.01	0.012	0	0.013	0.01	t	4849	ASYKVMT RSSHQSLV	Homo sapiens
1	1.00	1	1	0	0	-0.007	0.006	0	t	112841	SSKRGRQT PVLYAML DH	Rattus norvegic us
1	1.00	1	1	-0.01	-0.008	-0.027	-0.01	-0.007	t	110954	NNKYTVTL KATP	Homo sapiens
1	1.00	1	1	0.07	0.07	0.07	0.07	0.068	t	159283	EGP	Oryctola gus cuniculu s
1	1.00	1	1	0	0	-0.013	-0.003	0	t	146918	FFQP	Homo sapiens
1	1.00	1	1	0.1	0.1	0.098	0.1	0.1	t	117195	DT	Mus musculu s
1	1.00	1	1	-0.01	-0.01	-0.021	-0.007	-0.01	t	6906	CRAAKL	Homo sapiens
1	1.00	1	1	-0.05	-0.05	-0.05	-0.04	-0.049	t	115209	DKVGINY WLA	Homo sapiens
1	1.00	1	1	0.06	0.058	0.057	0.065	0.06	t	10692	DVPDYASL	Mus musculu s
1	1.00	1	1	0	0.002	-0.007	0.007	0.002	t	108944	FFLYSKL	Homo sapiens
1	1.00	1	1	0.05	0.05	0.049	0.06	0.052	t	106428	FVEIYSP	Homo sapiens
1	1.00	1	1	0	0.002	0.007	0.007	0	t	112769	EPLYGPY	Homo sapiens
1	1.00	1	1	0.02	0.024	0.022	0.027	0.019	t	119561	DWLNGKE Y	Homo sapiens
1	1.00	1	1	0.02	0.022	0.022	0.027	0.019	t	119561	DWLNGKE Y	Homo sapiens
1	1.00	1	1	-0.02	-0.032	-0.035	-0.02	-0.018	t	34895	LAPDNISL	Macaca mulatta

1	1.00	1	1	-0.02	-0.029	-0.035	-0.02	-0.018	t	34895	LAPDNISL	Macaca mulatta
1	1.00	1	1	-0.02	-0.029	-0.035	-0.02	-0.018	t	34895	LAPDNISL	Macaca mulatta
1	1.00	1	1	0.04	0.044	0.04	0.047	0.039	t	119660	VEWESNG Q	Homo sapiens
1	1.00	1	1	0.02	0.02	0.01	0.02	0.02	t	26046	IFDT	Mus musculu s
1	1.00	1	1	0.03	0.032	0.03	0.037	0.03	t	122579	SLIFVTISSE WG	Homo sapiens
1	1.00	1	1	0.08	0.08	0.08	0.087	0.08	t	133692	SG	Homo sapiens
1	1.00	1	1	0.02	0.02	0.012	0.02	0.019	t	76633	YYVNKQE GKSLYVKG	Mus musculu s
1	1.00	1	1	-0.01	-0.008	-0.028	-0.011	-0.009	t	98697	AMCNVYI PPYCT	Homo sapiens
1	1.00	1	1	0.02	0.02	0.02	0.02	0.017	t	48778	PPFSLSPV PTL	Oryctola gus cuniculu s
1	1.00	1	1	-0.01	-0.013	-0.021	-0.01	-0.009	t	133629	LPLRF	Oryctola gus cuniculu s
1	1.00	1	1	0.05	0.052	0.04	0.051	0.05	t	19202	GEAGPGV A	Homo sapiens
1	1.00	1	1	0.03	0.034	0.019	0.03	0.029	t	113504	GYIPANTTI PFGGAYNI PLV	Cavia porcellu s
1	1.00	1	1	0.03	0.03	0.028	0.03	0.028	t	107378	GELP	Rattus norvegic us
1	1.00	1	1	-0.02	-0.018	-0.018	-0.013	-0.022	t	127927	AKPAPPKP EPKPKKA	Homo sapiens

1	1.00	1	1	0.04	0.04	0.03	0.04	0.04	t	71091	VSQKNGGY	Oryctolagus cuniculus
1	1.00	1	1	0.03	0.03	0.016	0.03	0.032	t	45625	NQSFEEDTEK	Oryctolagus cuniculus
1	1.00	1	1	0.04	0.037	0.036	0.04	0.038	t	94484	DDYRQK	Oryctolagus cuniculus
1	1.00	1	1	-0.01	-0.01	-0.017	-0.01	-0.011	t	107700	YRVVSVL	Homo sapiens
1	1.00	1	1	-0.01	-0.012	-0.017	-0.007	-0.011	t	10529	DTYRYI	Mus musculus
1	1.00	1	1	0	0	0	0.01	0.001	t	115256	FRELKDLKGY	Homo sapiens
1	1.00	1	1	0	0.002	-0.005	0.003	0	t	3382	ANPPDH	Homo sapiens
1	1.00	1	1	0.03	0.034	0.015	0.027	0.03	t	13084	ELGVRE	Homo sapiens
1	1.00	1	1	-0.03	-0.028	-0.037	-0.029	-0.031	t	119275	MPMMGPPPPGMMPVGP	Homo sapiens
1	1.00	1	1	0.01	0.008	0.01	0.017	0.01	t	123259	GQPPYMPP	Homo sapiens
1	1.00	1	1	0.02	0.02	0.005	0.018	0.021	t	98783	FQQPLQQYPLGQ	Homo sapiens
1	1.00	1	1	-0.02	-0.022	-0.028	-0.019	-0.02	t	1373	AFPL	Mus musculus
1	1.00	1	1	-0.03	-0.028	-0.035	-0.027	-0.031	t	48828	PPLPPV	Homo sapiens
1	1.00	1	1	0.02	0.022	0.01	0.021	0.02	t	136749	WMLRAAEEYSMNLGF	Homo sapiens

1	1.00	1	1	0	0.002	-0.012	0.003	0.001	t	1477	AGDR	Homo sapiens
1	1.00	1	1	-0.01	-0.011	-0.008	-0.003	-0.012	t	3985	AQKKIIAEKT	Homo sapiens
1	1.00	1	1	0	0.002	-0.016	0.003	0.002	t	27379	ILTGGKSKQTEGLLLL	Homo sapiens
1	1.00	1	1	0.01	0.014	0.007	0.011	0.009	t	36136	LGGVFT	Homo sapiens
1	1.00	1	1	-0.06	-0.062	-0.079	-0.057	-0.058	t	96062	ADTIQKGFYY	Mus musculus
1	1.00	1	1	0	0.002	-0.013	0.001	0	t	48676	PNM	Homo sapiens
1	1.00	1	1	0	0	0	0.004	-0.003	t	119272	MGPPIGLPPAR	Homo sapiens
1	1.00	1	1	0.02	0.022	0.015	0.02	0.02	t	14398	ETHPLYQS	Anas platyrhynchos
1	1.00	1	1	0.04	0.04	0.04	0.04	0.039	t	153865	PETTDLYCY	Cavia porcellus
1	1.00	1	1	0.03	0.033	0.02	0.03	0.03	t	132754	YER	Mus musculus
1	1.00	1	1	0	0.002	0	0.007	-0.002	t	134783	RKLQKSTHLLIRKLP	Homo sapiens
1	1.00	1	1	0.03	0.03	0.018	0.03	0.03	t	97539	NYTTAVDR	Mus musculus
1	1.00	1	1	0.04	0.04	0.04	0.04	0.039	t	139524	ISSQT	Mus musculus
1	1.00	1	1	0.04	0.038	0.028	0.039	0.04	t	66143	TSAEDK	Mus musculus

1	1.00	1	1	0.04	0.042	0.033	0.047	0.042	t	109983	TLPPSRE	Homo sapiens
1	1.00	1	1	-0.02	-0.02	-0.02	-0.02	-0.024	t	48819	PPLGNWF G	Rattus norvegic us
1	1.00	1	1	0.04	0.037	0.028	0.04	0.04	t	25831	IEHYADTY EEVL	Oryctola gus cuniculu s
1	1.00	1	1	0.02	0.016	0.011	0.023	0.022	t	70038	VMYASSG	Oryctola gus cuniculu s
1	1.00	1	1	0	0	0.011	0.01	-0.003	t	69265	VKLSG	Homo sapiens
1	1.00	1	1	-0.03	-0.026	-0.03	-0.023	-0.032	t	126922	REWIKFAA ACRE	Homo sapiens
1	1.00	1	1	0.06	0.06	0.051	0.06	0.06	t	81983	FDTTTL	Mus musculu s
1	1.00	1	1	0.06	0.058	0.051	0.06	0.06	t	81983	FDTTTL	Mus musculu s
1	1.00	1	1	-0.01	-0.017	-0.011	-0.007	-0.013	t	48844	PPNRQDP RFTDTLVD	Homo sapiens
1	1.00	1	1	-0.02	-0.022	-0.032	-0.017	-0.02	t	107219	PAGGPGF PG	Rattus norvegic us
1	1.00	1	1	0.02	0.024	0.018	0.027	0.018	t	131955	LPHGNALV RYSSKEE	Homo sapiens
1	1.00	1	1	-0.02	-0.023	-0.035	-0.02	-0.02	t	98575	VGKPGSD VYY	Oryctola gus cuniculu s
1	1.00	1	1	0	-0.002	-0.012	0	0	t	100385	PKTFQFIN DQ	Mus musculu s
1	1.00	1	1	0	0	-0.008	0	0	t	22460	GSQGFP	Homo sapiens
1	1.00	1	1	0.01	0.008	-0.005	0.009	0.012	t	49119	PQRPQQP FPQ	Mus musculu s

1	1.00	1	1	0.02	0.02	0.003	0.02	0.022	t	15984	FGGHNSVDFE	Oryctolagus cuniculus
1	1.00	1	1	0.01	0.007	0.001	0.01	0.01	t	113346	DTSTTPVPSV	Oryctolagus cuniculus
1	1.00	1	1	0.01	0.008	0.001	0.009	0.009	t	10588	DVFGANYYYQ	Mus musculus
1	1.00	1	1	-0.02	-0.018	-0.022	-0.016	-0.021	t	118548	PAPGMRPP	Homo sapiens
1	1.00	1	1	0.04	0.04	0.043	0.046	0.04	t	142150	EYMLDLQPETTD	Homo sapiens
1	1.00	1	1	0.03	0.028	0.029	0.031	0.029	t	78972	FGGDTA	Mus musculus
1	1.00	1	1	0.03	0.032	0.023	0.027	0.027	t	61155	SSLFQLWKSE	Homo sapiens
1	1.00	1	1	0	0.002	0	0.007	-0.002	t	119633	QYTGHITWTPPAIFKS	Homo sapiens
1	1.00	1	1	0	0	-0.012	0	0.001	t	35852	LFIG	Mus musculus
1	1.00	1	1	-0.03	-0.025	-0.03	-0.023	-0.031	t	144311	GNLTAG	Homo sapiens
1	1.00	1	1	-0.02	-0.018	-0.023	-0.019	-0.02	t	33888	KTTLLK	Homo sapiens
1	1.00	1	1	0.01	0.012	0.012	0.017	0.009	t	119676	WQQGNVFS	Homo sapiens
1	1.00	1	1	-0.01	-0.01	-0.01	-0.004	-0.011	t	157292	GAIIGLM	Homo sapiens
1	1.00	1	1	0	-0.003	0.008	0	-0.005	t	125211	QHWSYGLRPG	Canis lupus

1	1.00	1	1	0.02	0.023	0.012	0.02	0.018	t	52249	QRQYGDVFKGD	Mus musculus
1	1.00	1	1	0.03	0.028	0.027	0.031	0.029	t	11862	EERPPETT	Anas platyrhynchos
1	1.00	1	1	-0.02	-0.02	-0.025	-0.017	-0.02	t	123442	LLKDLRKN	Homo sapiens
1	1.00	1	1	0.02	0.022	0.023	0.021	0.019	t	45033	NMTVM	Homo sapiens
1	1.00	1	1	0.03	0.03	0.017	0.027	0.03	t	147218	QGSFQP	Homo sapiens
1	1.00	1	1	0	0.002	-0.012	0.001	0	t	142381	AAEFATEVKKAT	Homo sapiens
1	1.00	1	1	0	0	0	0.01	0.001	t	115317	KKILDKVGIN	Homo sapiens
1	1.00	1	1	0.03	0.032	0.028	0.037	0.03	t	104756	DTTFEYQDLC	Homo sapiens
1	1.00	1	1	0	0.002	-0.02	0.001	0.002	t	43582	NEDQRVGI	Homo sapiens
1	1.00	1	1	-0.05	-0.049	-0.051	-0.047	-0.051	t	99204	EISKFL	Equus caballus
1	1.00	1	1	0.01	0.008	-0.003	0.009	0.011	t	65105	TLTPENTL	Mus musculus
1	1.00	1	1	0.03	0.033	0.018	0.03	0.03	t	12550	EIPQPQWT	Anas platyrhynchos
1	1.00	1	1	-0.01	-0.008	-0.016	-0.003	-0.01	t	144823	YWEGSGIGAI	Homo sapiens
1	1.00	1	1	0.03	0.028	0.018	0.03	0.03	t	100477	RNKTQEEHLK	Mus musculus

1	1.00	1	1	0.01	0.013	-0.004	0.011	0.011	t	58683	SIQTKFAP	Sus scrofa
1	1.00	1	1	-0.02	-0.02	-0.021	-0.02	-0.023	t	75686	YRYDSRPP	Mus musculus
1	1.00	1	1	-0.02	-0.023	-0.021	-0.02	-0.023	t	75686	YRYDSRPP	Mus musculus
1	1.00	1	1	-0.02	-0.023	-0.021	-0.02	-0.023	t	75686	YRYDSRPP	Oryctolagus cuniculus
1	1.00	1	1	-0.02	-0.022	-0.021	-0.02	-0.023	t	75686	YRYDSRPP	Mus musculus
1	1.00	1	1	0.02	0.02	0.013	0.02	0.02	t	68763	VGPQSVGPL	Mus musculus
1	1.00	1	1	-0.01	-0.009	-0.02	-0.01	-0.009	t	111038	TGALSKIY	Oryctolagus cuniculus
1	1.00	1	1	0.01	0.01	-0.006	0.01	0.012	t	46087	NTFINNAY	Mus musculus
1	1.00	1	1	0	-0.007	-0.017	0	0.003	t	110857	FTGEAYSYWSAK	Macaca
1	1.00	1	1	0.01	0.013	0.004	0.01	0.007	t	43549	NDYEDRYYREN	Mus musculus
1	1.00	1	1	0.03	0.034	0.03	0.03	0.03	t	137402	FIDSYICQV	Homo sapiens
1	1.00	1	1	0	0	-0.009	0	0	t	120694	KLLLDYTGKI	Rattus norvegicus
1	1.00	1	1	0.04	0.043	0.039	0.04	0.038	t	122113	IIASTEKLP	Mus musculus

1	1.00	1	1	0.02	0.022	0.02	0.027	0.02	t	133371	SPSPTPTP GP	Homo sapiens
1	1.00	1	1	-0.02	-0.018	-0.02	-0.013	-0.022	t	128494	EDKINALIK AAGVNV	Homo sapiens
1	1.00	1	1	0	0.003	0.002	0	-0.004	t	146066	VPKVSGLQ Y	Mus musculu s
1	1.00	1	1	0	0.004	-0.003	0.001	-0.004	t	85032	LCVYGKLQ KN	Homo sapiens
1	1.00	1	1	0.03	0.028	0.016	0.033	0.032	t	60527	SQPQQPF PQQ	Mus musculu s
1	1.00	1	1	0.04	0.04	0.04	0.043	0.04	t	67990	VDIVLE	Homo sapiens
1	1.00	1	1	0.01	0.01	0.005	0.013	0.01	t	59112	SLFTPGP	Homo sapiens
1	1.00	1	1	0.01	0.013	0.008	0.01	0.008	t	74008	YGGL	Mus musculu s
1	1.00	1	1	0.01	0.006	0.008	0.01	0.008	t	74008	YGGL	Mus musculu s
1	1.00	1	1	0.01	0.005	0.008	0.01	0.008	t	74008	YGGL	Mus musculu s
1	1.00	1	1	-0.03	-0.025	-0.037	-0.027	-0.03	t	60780	SRRFAPAL	Homo sapiens
1	1.00	1	1	-0.01	-0.01	-0.01	-0.01	-0.013	t	134769	QIFNL	Mus musculu s
1	1.00	1	1	0.02	0.022	0.014	0.023	0.019	t	53240	RAVVAPD KEVLYE	Homo sapiens
1	1.00	1	1	-0.03	-0.03	-0.03	-0.02	-0.029	t	115239	EVFRELKD LK	Homo sapiens

1	1.00	1	1	0.01	0.01	0.01	0.01	0.007	t	50104	PYIEQGMQL	Homo sapiens
1	1.00	1	1	0.02	0.022	0	0.017	0.022	t	75541	YQQYPQQ	Homo sapiens
1	1.00	1	1	-0.02	-0.018	-0.027	-0.023	-0.023	t	116037	YIPIQYVLSR	Homo sapiens
1	1.00	1	1	-0.02	-0.021	-0.032	-0.023	-0.02	t	130621	TFYTK	Oryctolagus cuniculus
1	1.00	1	1	-0.02	-0.019	-0.032	-0.023	-0.02	t	130621	TFYTK	Rattus norvegicus
1	1.00	1	1	0.04	0.04	0.038	0.04	0.038	t	130741	ATGFVKKDQ	Mus musculus
1	1.00	1	1	0.03	0.032	0.021	0.031	0.029	t	20722	GKVTNGS	Homo sapiens
1	1.00	1	1	0.03	0.034	0.021	0.031	0.029	t	20722	GKVTNGS	Homo sapiens
1	1.00	1	1	0	0.002	-0.015	-0.003	0	t	27693	INNEWC	Homo sapiens
1	1.00	1	1	0.02	0.011	0.007	0.02	0.021	t	34576	KYQAWNAG	Macaca mulatta
1	1.00	1	1	-0.01	-0.01	-0.02	-0.01	-0.01	t	2179	AKEVLKGY	Oryctolagus cuniculus
1	1.00	1	1	-0.05	-0.046	-0.069	-0.053	-0.05	t	47240	PEAVLR	Homo sapiens
1	1.00	1	1	-0.03	-0.027	-0.04	-0.03	-0.03	t	156553	YSTCYFIM	Mus musculus
1	1.00	1	1	-0.01	-0.008	-0.018	-0.007	-0.01	t	110971	PVSDLNLKIDGL	Homo sapiens

1	1.00	1	1	-0.03	-0.031	-0.042	-0.033	-0.03	t	130620	TFYLK	Oryctolagus cuniculus
1	1.00	1	1	0.01	0.012	0.003	0.017	0.012	t	108966	FLYSKLT	Homo sapiens
1	1.00	1	1	0	0	0.007	0	-0.003	t	3242	ANDPPP	Mus musculus
1	1.00	1	1	0.01	0.006	-0.004	0.009	0.01	t	37001	LKQPGDGN	Oryctolagus cuniculus
1	1.00	1	1	0.04	0.042	0.038	0.041	0.039	t	36955	LKLYFEP	Homo sapiens
1	1.00	1	1	0.01	0.007	0.001	0.01	0.01	t	113734	PVAPSDPSIV	Oryctolagus cuniculus
1	1.00	1	1	0.02	0.02	0.013	0.02	0.019	t	65653	TPLQCTM	Mus musculus
1	1.00	1	1	-0.01	-0.006	-0.01	-0.003	-0.01	t	122943	WDPAQPPRD	Homo sapiens
1	1.00	1	1	0.03	0.028	0.019	0.029	0.03	t	120406	APDMFEYMNK	Mus musculus
1	1.00	1	1	0	0.002	0	0.001	-0.002	t	38522	LPHSNL	Homo sapiens
1	1.00	1	1	0	0.002	0	0.007	0	t	117208	GLSLSRFSWGA	Homo sapiens
1	1.00	1	1	-0.04	-0.04	-0.043	-0.03	-0.04	t	98885	LPMHIRLSFN	Homo sapiens
1	1.00	1	1	-0.03	-0.028	-0.03	-0.023	-0.031	t	119661	VFSCSVMH	Homo sapiens
1	1.00	1	1	0.05	0.046	0.033	0.043	0.051	t	110767	AKEEISFVATFS	Oryctolagus cuniculus

1	1.00	1	1	0.02	0.006	0.011	0.019	0.02	t	54982	RNEYPINK	Macaca mulatta
1	1.00	1	1	0.01	0.013	0	0.01	0.01	t	121081	YIPFDQDLSK	Mus musculus
1	1.00	1	1	-0.01	-0.01	-0.015	-0.01	-0.011	t	133549	DVNGKLFL	Mus musculus
1	1.00	1	1	-0.01	-0.008	-0.017	-0.003	-0.009	t	117333	MVTSTITEKLLKNLVKI	Homo sapiens
1	1.00	1	1	0	0.004	-0.014	0.001	0.002	t	64769	TLEEFSAKL	Homo sapiens
1	1.00	1	1	0.01	-0.002	0.005	0.01	0.008	t	65027	TLQNQGAWP	Mus musculus
1	1.00	1	1	0.03	0.032	0.023	0.033	0.031	t	144783	SHVVTMLDNF	Homo sapiens
1	1.00	1	1	0.02	0.022	0.018	0.021	0.02	t	58883	SKLLTL	Homo sapiens
1	1.00	1	1	0.04	0.04	0.04	0.04	0.037	t	140593	PYQFFTASE	Mus musculus
1	1.00	1	1	0	0	0	0.007	0	t	135893	AFLQDVMNILLQ	Homo sapiens
1	1.00	1	1	0.01	0.012	0.012	0.017	0.009	t	119675	WNSGALTS	Homo sapiens
1	1.00	1	1	-0.01	-0.01	-0.02	-0.01	-0.011	t	112898	AAVKQSYAAT	Mus musculus
1	1.00	1	1	0.03	0.033	0.02	0.03	0.03	t	120919	RLNEAVKYTG	Mus musculus
1	1.00	1	1	0.01	0.012	0.015	0.017	0.01	t	134058	GDKVSFF	Homo sapiens
1	1.00	1	1	0.01	0.012	0.015	0.017	0.01	t	134058	GDKVSFF	Homo sapiens

1	1.00	1	1	0.01	0.01	0.025	0.01	0.001	t	55115	RPAPPKPT PQP	Mus musculu s
1	1.00	1	1	-0.01	-0.01	-0.025	-0.01	-0.01	t	41461	MEVTPSG TWLTYHG AIK	Homo sapiens
1	1.00	1	1	0.01	0.005	0	0.01	0.009	t	127847	SYNSTADE WLPV	Trichosu rus vulpecul a
1	1.00	1	1	0	0.002	-0.013	0.001	0.001	t	45589	NQMHML SNKS	Homo sapiens
1	1.00	1	1	0.01	0.01	0.001	0.01	0.01	t	25217	IAADQLPN	Oryctola gus cuniculu s
1	1.00	1	1	-0.02	-0.018	-0.027	-0.013	-0.02	t	144600	CQREFALK HL	Homo sapiens
1	1.00	1	1	0.01	0.014	-0.003	0.007	0.009	t	65983	TQYTDAPS FS	Homo sapiens
1	1.00	1	1	0.02	0.022	0.003	0.023	0.023	t	110926	KSEKDSTN NNK	Homo sapiens
1	1.00	1	1	-0.01	-0.01	-0.018	-0.008	-0.01	t	110932	LNLKIDGL KISV	Homo sapiens
1	1.00	1	1	0	0	0	0.004	-0.001	t	119621	NVFSCSV M	Homo sapiens
1	1.00	1	1	0.02	0.024	0.035	0.027	0.019	t	103843	DF	Homo sapiens
1	1.00	1	1	0	0	0	0	-0.003	t	50104	PYIEQGM QL	Homo sapiens
1	1.00	1	1	0.02	0.02	0.015	0.02	0.018	t	35611	LEN	Mus musculu s
1	1.00	1	1	0.03	0.028	0.023	0.029	0.028	t	21475	GNMVETE DTKEPGVL	Mus musculu s

1	1.00	1	1	0.01	0.008	-0.004	0.007	0.009	t	49450	PSNQDSF QWQ	Mus musculu s
1	1.00	1	1	0.02	0.02	0.02	0.02	0.019	t	117344	PPGMRPP	Homo sapiens
1	1.00	1	1	0	0	-0.01	0	0	t	69942	VMENFVA F	Homo sapiens
1	1.00	1	1	0.03	0.032	0.025	0.037	0.03	t	141604	APPDDGP P	Homo sapiens
1	1.00	1	1	0.05	0.05	0.052	0.06	0.05	t	105993	PAEGT	Homo sapiens
1	1.00	1	1	0.08	0.084	0.073	0.083	0.08	t	61994	STVSEEAS AEDVVC	Homo sapiens
1	1.00	1	1	0.04	0.036	0.033	0.039	0.038	t	104235	SDIDFLIEEI ERLGQDL	Oryctola gus cuniculu s
1	1.00	1	1	-0.05	-0.046	-0.046	-0.05	-0.055	t	123280	GYWKIRGL Q	Ovis aries
1	1.00	1	1	0.02	0.02	0.016	0.02	0.018	t	106690	NSDHFLQP DPPL	Mus musculu s
1	1.00	1	1	0.02	0.022	0.018	0.027	0.021	t	112130	GKGLEWL	Homo sapiens
1	1.00	1	1	0	0.002	0.002	0.007	-0.003	t	122525	PRFLRLLDL AQEGLK	Homo sapiens
1	1.00	1	1	-0.01	-0.008	-0.016	-0.003	-0.01	t	144823	YWEGSGI GAI	Homo sapiens
1	1.00	1	1	-0.01	-0.011	-0.02	-0.006	-0.01	t	30041	KCAFATTP VCQF	Myodes glareolu s

1	1.00	1	1	0	0.004	-0.011	0.001	0	t	9983	DRHFLNNVC	Homo sapiens
1	1.00	1	1	0.02	0.02	0.01	0.02	0.02	t	136749	WMLRAAEEYSMNLGF	Mus musculus
1	1.00	1	1	0.02	0.02	0.01	0.02	0.02	t	136749	WMLRAAEEYSMNLGF	Mus musculus
1	1.00	1	1	0.03	0.03	0.024	0.03	0.028	t	19375	GENK	Mus musculus
1	1.00	1	1	0.04	0.042	0.031	0.037	0.038	t	114814	ESNGTLTLSH	Homo sapiens
1	1.00	1	1	0.03	0.023	0.025	0.03	0.03	t	65	AAAVLNLT	Oryctolagus cuniculus
1	1.00	1	1	0.11	0.11	0.11	0.11	0.107	t	143682	EEIEKE	Mus musculus
1	1.00	1	1	0.04	0.044	0.042	0.047	0.039	t	119646	SNGQPENN	Homo sapiens
1	1.00	1	1	0.01	0.008	-0.005	0.009	0.012	t	49118	PQRPFAQQPE	Mus musculus
1	1.00	1	1	-0.03	-0.03	-0.037	-0.02	-0.03	t	107087	ARGLTGRPGDA	Homo sapiens
1	1.00	1	1	0	0.004	0	0.007	0.002	t	107396	GKEYKCK	Homo sapiens
1	1.00	1	1	-0.01	-0.01	-0.031	-0.01	-0.008	t	65909	TQP	Mus musculus
1	1.00	1	1	-0.06	-0.054	-0.056	-0.06	-0.065	t	123281	GYWKLRGLA	Ovis aries
1	1.00	1	1	0.01	0.008	-0.001	0.007	0.008	t	47969	PIQPQQPFPQ	Mus musculus

1	1.00	1	1	0.05	0.054	0.034	0.051	0.052	t	67562	VAEEHVE	Homo sapiens
1	1.00	1	1	0.03	0.033	0.025	0.037	0.03	t	117543	KFKGKATL TSDK	Mus musculu s
1	1.00	1	1	0.03	0.03	0.025	0.037	0.03	t	117543	KFKGKATL TSDK	Mus musculu s
1	1.00	1	1	0.01	0.01	0.01	0.021	0.01	t	104115	MILSLQM	Homo sapiens
1	1.00	1	1	0.01	0.01	0.001	0.014	0.01	t	135900	ATCRFYTL DSIK	Homo sapiens
1	1.00	1	1	-0.05	-0.048	-0.063	-0.049	-0.051	t	55639	RRLGAALL	Homo sapiens
1	1.00	1	1	0.02	0.017	0.013	0.02	0.018	t	1022	AEKQKAAE ATKVAE	Oryctola gus cuniculu s
1	1.00	1	1	0	0.002	0	0.007	-0.001	t	119671	WLNGKEY K	Homo sapiens
1	1.00	1	1	0	-0.002	0.002	0.003	-0.003	t	69946	VMFDFAS G	Mus musculu s
1	1.00	1	1	-0.02	-0.022	-0.02	-0.021	-0.024	t	48819	PPLGNWF G	Rattus norvegic us
1	1.00	1	1	0.05	0.052	0.043	0.057	0.052	t	110029	VEWGSND	Homo sapiens
1	1.00	1	1	0.02	0.017	0.008	0.02	0.021	t	61945	STTNKDK	Oryctola gus cuniculu s
1	1.00	1	1	0.02	0.024	0.008	0.02	0.021	t	61945	STTNKDK	Cavia porcellu s
1	1.00	1	1	0.02	0.02	0.008	0.02	0.021	t	61945	STTNKDK	Mus musculu s
1	1.00	1	1	0.07	0.066	0.068	0.073	0.071	t	99274	GGFSEV	Oryctola gus cuniculu s

1	1.00	1	1	0.05	0.05	0.05	0.05	0.047	t	67323	TYDNE	Homo sapiens
1	1.00	1	1	0.03	0.03	0.019	0.031	0.03	t	25538	IDANDCLVTPSVK	Homo sapiens
1	1.00	1	1	0.06	0.058	0.05	0.059	0.06	t	44163	NHPQNTSD	Mus musculus
1	1.00	1	1	0.03	0.027	0.016	0.027	0.03	t	47316	PEKAFRELAD	Oryctolagus cuniculus
1	1.00	1	1	0.02	0.02	0.02	0.029	0.02	t	107814	EGLHNHY	Homo sapiens
1	1.00	1	1	0.01	0.008	0.009	0.011	0.01	t	36315	LGSAYP	Mus musculus
1	1.00	1	1	-0.04	-0.036	-0.04	-0.033	-0.043	t	137456	TAIASRSV	Homo sapiens
1	1.00	1	1	0.01	0.013	0	0.01	0.01	t	121079	YFTDIDLVEH	Mus musculus
1	1.00	1	1	0	-0.002	-0.005	-0.001	-0.001	t	109841	RPGEAGLP	Rattus norvegicus
1	1.00	1	1	0.01	0.01	0.006	0.01	0.009	t	48279	PLATQPPVLAL	Mus musculus
1	1.00	1	1	0.01	0.007	0	0.01	0.012	t	95111	KTVDH	Oryctolagus cuniculus
1	1.00	1	1	0.04	0.042	0.04	0.047	0.04	t	119573	EWEDTYGIVL	Homo sapiens
1	1.00	1	1	-0.01	-0.01	-0.01	-0.008	-0.012	t	69828	VLVLFGFFTLSPWY	Homo sapiens
1	1.00	1	1	-0.02	-0.018	-0.028	-0.02	-0.021	t	48926	PPSSPPQW	Anas platyrhynchos

1	1.00	1	1	0.02	0.02	0.012	0.02	0.02	t	76268	YVFDKSELTN	Mus musculus
1	1.00	1	1	0.02	0.023	0.012	0.02	0.02	t	76268	YVFDKSELTN	Mus musculus
1	1.00	1	1	-0.01	-0.013	-0.024	-0.01	-0.01	t	47751	PGSQHI	Oryctolagus cuniculus
1	1.00	1	1	0.07	0.074	0.07	0.077	0.07	t	125610	YYVFFEEQEDEIIGFGQELK	Homo sapiens
1	1.00	1	1	-0.04	-0.04	-0.049	-0.04	-0.04	t	118764	CYGSFARPTN	Mus musculus
1	1.00	1	1	-0.04	-0.04	-0.049	-0.04	-0.04	t	118764	CYGSFARPTN	Mus musculus
1	1.00	1	1	0	0	0	0.007	-0.002	t	113321	DENPVVHFFKNIVTPRT	Homo sapiens
1	1.00	1	1	0.03	0.026	0.021	0.029	0.03	t	70665	VQVVYDYQHK	Oryctolagus cuniculus
1	1.00	1	1	-0.04	-0.038	-0.045	-0.037	-0.042	t	134183	PLIFRNV	Homo sapiens
1	1.00	1	1	0.01	0.008	0.002	0.009	0.01	t	34132	KVNDA	Mus musculus
1	1.00	1	1	0.03	0.028	0.022	0.029	0.028	t	10336	DTFNPVYPYDTE	Mus musculus
1	1.00	1	1	0.02	0	0.017	0.021	0.018	t	111523	LTADEVK	Cricetinae
1	1.00	1	1	0.03	0.034	0.03	0.031	0.027	t	134696	ITLYLTEK	MD
1	1.00	1	1	-0.01	-0.008	-0.015	-0.003	-0.009	t	115256	FRELKDLKGY	Homo sapiens

1	1.00	1	1	0.02	0.017	0.011	0.02	0.02	t	41449	METMKL MMEKVD	Oryctolagus cuniculus
1	1.00	1	1	0.02	0.02	0.011	0.02	0.02	t	41449	METMKL MMEKVD	Rattus norvegicus
1	1.00	1	1	0.03	0.027	0.016	0.03	0.032	t	47316	PEKAFREL AD	Oryctolagus cuniculus
1	1.00	1	1	0.01	-0.004	0	0.007	0.01	t	33770	KTLTKTSL	Macaca mulatta
1	1.00	1	1	0.04	0.042	0.042	0.047	0.04	t	119670	WESNGQP E	Homo sapiens
1	1.00	1	1	0	0.003	-0.019	0	0.002	t	4032	AQPKSA	Mus musculus
1	1.00	1	1	0.06	0.06	0.058	0.06	0.059	t	137209	ITANVTDE SYLY	Sus scrofa
1	1.00	1	1	0.03	0.03	0.015	0.03	0.032	t	43506	NDPN	Mus musculus
1	1.00	1	1	0	-0.004	-0.005	0	0	t	16569	FLEGAAR	Mus musculus
1	1.00	1	1	0.01	0.012	0.004	0.013	0.012	t	115153	MKGVVCT RIYEKV	Homo sapiens
1	1.00	1	1	0	-0.001	0.001	0.004	-0.003	t	30041	KCAFATTP VCQF	Myodes glareolus
1	1.00	1	1	0.04	0.042	0.037	0.047	0.04	t	107800	DSRGNPT VE	Homo sapiens
1	1.00	1	1	0.01	0.01	-0.005	0.007	0.01	t	52023	QQFHQQ Q	Homo sapiens
1	1.00	1	1	0.03	0.03	0.014	0.03	0.032	t	120867	PFDQDLSK KH	Mus musculus
1	1.00	1	1	-0.01	-0.01	-0.017	-0.01	-0.01	t	131249	LVVGLC	Homo sapiens

1	1.00	1	1	0.02	0.018	0.019	0.019	0.019	t	56788	SAFVFPTKD	Mus musculus
1	1.00	1	1	-0.01	-0.01	-0.025	-0.01	-0.01	t	98321	PAYYTKNTNN	Mus musculus
1	1.00	1	1	0.01	0.007	0.009	0.01	0.009	t	105828	GKDLESIK	Oryctolagus cuniculus
1	1.00	1	1	0.05	0.052	0.05	0.057	0.049	t	119574	EWESNGQP	Homo sapiens
1	1.00	1	1	0	0	-0.007	0	-0.002	t	117689	VSMNTHLKAVKMT	Homo sapiens
1	1.00	1	1	-0.04	-0.038	-0.047	-0.037	-0.04	t	135959	LTRAYAKDVKFG	Homo sapiens
1	1.00	1	1	-0.03	-0.028	-0.03	-0.023	-0.032	t	129249	LKARVDELERIRRSIL	Homo sapiens
1	1.00	1	1	0.01	0.012	0.012	0.017	0.009	t	119676	WQQGNVFS	Homo sapiens
1	1.00	1	1	-0.01	-0.01	-0.024	-0.01	-0.01	t	120532	FKDSINNLVK	Mus musculus
1	1.00	1	1	0.05	0.052	0.04	0.047	0.05	t	13894	EQNVPD	Cavia porcellus
1	1.00	1	1	-0.01	-0.008	-0.01	-0.003	-0.011	t	119602	KGQPREPQ	Homo sapiens
1	1.00	1	1	0.02	0.022	0.018	0.021	0.02	t	62329	SVSLVL	Homo sapiens
1	1.00	1	1	0.04	0.04	0.04	0.04	0.037	t	143362	ETPLMKE	Mus musculus
1	1.00	1	1	0.01	0.01	-0.005	0.007	0.01	t	7698	DCCQQL	Homo sapiens
1	1.00	1	1	0	0	0	0	-0.003	t	50104	PYIEQGMQL	Homo sapiens
1	1.00	1	1	-0.02	-0.02	-0.023	-0.01	-0.02	t	98858	LACQCLVRTP	Homo sapiens

1	1.00	1	1	0.03	0.03	0.025	0.031	0.03	t	22044	GRGGIYRF VTPGER	Homo sapiens
1	1.00	1	1	-0.01	-0.014	-0.011	-0.011	-0.012	t	78711	KQYDPDR P	Oryctola gus cuniculu s
1	1.00	1	1	0	0	0	0	-0.003	t	115376	LTKCEVFR EL	Homo sapiens
1	1.00	1	1	0	0.002	-0.012	0.001	0	t	39626	LSREM	Homo sapiens
1	1.00	1	1	0	-0.002	-0.02	0.003	0.003	t	141689	KMDKKYS AHKMKCT EV	Rattus norvegic us
1	1.00	1	1	0	-0.004	-0.02	0.003	0.003	t	141689	KMDKKYS AHKMKCT EV	Oryctola gus cuniculu s
1	1.00	1	1	0.04	0.042	0.042	0.047	0.039	t	96828	RLKEEEED KK	Homo sapiens
1	1.00	1	1	-0.01	-0.008	-0.017	-0.007	-0.01	t	48827	PPLPHV	Homo sapiens
1	1.00	1	1	0.02	0.022	0.015	0.023	0.02	t	15246	FAPALPV W	Homo sapiens
1	1.00	1	1	0.01	0.01	0.003	0.014	0.009	t	130448	AYAKDVKF GADA	Homo sapiens
1	1.00	1	1	0.01	0.008	0.01	0.01	0.01	t	112672	TAEFQMT FHLFI	Oryctola gus cuniculu s
1	1.00	1	1	0.01	0.012	0.003	0.013	0.009	t	135899	ASLYHVYE VNLV	Homo sapiens
1	1.00	1	1	0.02	0.028	0.009	0.02	0.02	t	99226	ERQLKSA	Gallus gallus
1	1.00	1	1	-0.01	-0.01	-0.013	0	-0.01	t	98973	QSLACQCL VR	Homo sapiens
1	1.00	1	1	0	0.002	0	0.007	-0.002	t	117512	GRGRGRG RGGPRR	Homo sapiens

1	1.00	1	1	0	0	0	0.01	0	t	114885	LCAYSIEFGT	Homo sapiens
1	1.00	1	1	0.02	0.021	0.014	0.02	0.02	t	41644	MGVIGFSFFVKDWS	Myodes glareolus
1	1.00	1	1	-0.03	-0.03	-0.042	-0.03	-0.03	t	23641	HDRHFLNNV	Homo sapiens
1	1.00	1	1	0.02	0.018	0.013	0.02	0.02	t	45303	NPASTTNKDK	Mus musculus
1	1.00	1	1	0.04	0.04	0.04	0.05	0.04	t	114965	TSEDGKVMVL	Homo sapiens
1	1.00	1	1	0.05	0.052	0.037	0.05	0.05	t	142218	LDLQPETTDLYC	Homo sapiens
1	1.00	1	1	0.01	0.01	0.003	0.01	0.008	t	126069	FVFEEFARQNLK	Mus musculus
1	1.00	1	1	0.02	0.018	0.019	0.02	0.019	t	95146	SESPNK	Oryctolagus cuniculus
1	1.00	1	1	0.02	0.018	0.016	0.023	0.018	t	19906	GGLEPINFQT	Mus musculus
1	1.00	1	1	0.01	0.008	0.001	0.007	0.01	t	108900	EIPVDLL	Mus musculus
1	1.00	1	1	0	0	-0.009	0	-0.001	t	94553	IFD	Mus musculus
1	1.00	1	1	-0.01	-0.013	-0.012	-0.01	-0.011	t	55263	RPPPAPCL	Oryctolagus cuniculus
1	1.00	1	1	-0.03	-0.024	-0.03	-0.023	-0.031	t	131293	AGRAREYV	Homo sapiens
1	1.00	1	1	0.01	0.012	-0.001	0.007	0.008	t	114790	EGKDVLVCNK	Homo sapiens
1	1.00	1	1	0	0.004	-0.003	0.001	-0.001	t	20724	GKVVLP	Homo sapiens

1	1.00	1	1	0	0.002	-0.003	0.001	-0.001	t	20724	GKVVLP	Homo sapiens
1	1.00	1	1	0	0.002	-0.003	0.001	-0.001	t	20724	GKVVLP	Homo sapiens
1	1.00	1	1	-0.02	-0.022	-0.015	-0.017	-0.024	t	141726	PTAPPLLP HS	Rattus norvegic us
1	1.00	1	1	0.02	0.018	0.018	0.027	0.021	t	3516	APDTRP	Mus musculu s
1	1.00	1	1	0.02	0.023	0.011	0.02	0.02	t	41449	METMKL MMEKVD	Equus caballus
1	1.00	1	1	0.03	0.03	0.027	0.037	0.03	t	120167	RFQSSAV MALQEAC EAYL	Mus musculu s
1	1.00	1	1	0.01	0.003	0.003	0.017	0.012	t	156970	VERHQ	Homo sapiens
1	1.00	1	1	0.02	0.02	0.017	0.02	0.02	t	78252	PVQNLT	Mus musculu s
1	1.00	1	1	0.05	0.05	0.048	0.05	0.048	t	98725	DDEALEKF DK	Homo sapiens
1	1.00	1	1	0.05	0.048	0.047	0.049	0.05	t	50512	QDPV	Mus musculu s
1	1.00	1	1	0.02	0.018	0.013	0.023	0.02	t	107166	GPPGPQG	Mus musculu s
1	1.00	1	1	-0.01	-0.012	-0.01	-0.003	-0.01	t	123787	TPATKKAV	Homo sapiens
1	1.00	1	1	-0.04	-0.038	-0.039	-0.033	-0.042	t	40340	LVFKT	Homo sapiens
1	1.00	1	1	0.02	0.016	0.011	0.019	0.02	t	39465	LSKSFLEA GGVSK	Oryctola gus cuniculu s

1	1.00	1	1	0	0.002	0.002	0.007	0	t	119669	VSWNSGA L	Homo sapiens
1	1.00	1	1	0.01	0.006	0.004	0.01	0.01	t	6068	CCPDKNKS	Mus musculu s
1	1.00	1	1	0.01	0.012	0.01	0.017	0.01	t	119673	WNPDDYG GVK	Homo sapiens
1	1.00	1	1	0	0.002	0.005	0.007	0	t	112813	PEFAPWA	Homo sapiens
1	1.00	1	1	-0.02	-0.022	-0.025	-0.023	-0.023	t	116025	VYPFPGPI PN	Homo sapiens
1	1.00	1	1	0	-0.002	0	0.001	-0.003	t	130449	CFFPNLSY C	Mus musculu s
1	1.00	1	1	0.02	0.02	0.012	0.023	0.02	t	55469	RQQEPE	Homo sapiens
1	1.00	1	1	0.04	0.038	0.028	0.037	0.039	t	144641	FFENKD	Mus musculu s
1	1.00	1	1	0.01	0.013	0.01	0.01	0.008	t	137401	FEDVW	Mus musculu s
1	1.00	1	1	-0.01	-0.01	-0.01	-0.01	-0.013	t	134769	QIFNL	Mus musculu s
1	1.00	1	1	0	0	0	0.007	-0.002	t	113321	DENPVVH FFKNIVTP RT	Homo sapiens
1	1.00	1	1	0.04	0.044	0.04	0.047	0.04	t	138230	QQFNSTF	Homo sapiens
1	1.00	1	1	0.05	0.052	0.045	0.051	0.05	t	66295	TSLFTPG	Homo sapiens
1	1.00	1	1	0.05	0.052	0.045	0.051	0.05	t	66295	TSLFTPG	Homo sapiens
1	1.00	1	1	0.04	0.036	0.032	0.04	0.04	t	48073	PKGTLTWS	Anas platyrhy nchos

1	1.00	1	1	0.04	0.044	0.037	0.041	0.041	t	19965	GGPLTN	Homo sapiens
1	1.00	1	1	0.04	0.038	0.034	0.04	0.042	t	25080	HVQTMEEI	Anas platyrhynchos
1	1.00	1	1	0.02	0.018	0.011	0.023	0.022	t	57654	SETQKSQNY	Mus musculus
1	1.00	1	1	0.03	0.034	0.03	0.037	0.028	t	126903	QVINVREG	Homo sapiens
1	1.00	1	1	0.04	0.04	0.04	0.04	0.04	t	127899	YFKEQFLDGD	Homo sapiens
1	1.00	1	1	0.04	0.042	0.038	0.04	0.039	t	36955	LKLYFEP	Homo sapiens
1	1.00	1	1	0.02	0.016	0.013	0.019	0.019	t	138057	TPGTEYTV	Oryctolagus cuniculus
1	1.00	1	1	-0.05	-0.048	-0.055	-0.047	-0.051	t	72326	WDQMWKCLI	Homo sapiens
1	1.00	1	1	0.02	0.017	0.007	0.02	0.02	t	15462	FDSSKQSF	Oryctolagus cuniculus
1	1.00	1	1	0.02	0.017	0.007	0.02	0.02	t	15462	FDSSKQSF	Oryctolagus cuniculus
1	1.00	1	1	0.04	0.044	0.034	0.04	0.038	t	57766	SFFSYGEI	MD
1	1.00	1	1	0.05	0.042	0.033	0.043	0.05	t	110769	ANPENYFTNGEA	Macaca
1	1.00	1	1	0.05	0.042	0.033	0.043	0.05	t	110769	ANPENYFTNGEA	Macaca

1	1.00	1	1	0.05	0.042	0.033	0.043	0.05	t	110769	ANPENYFTNGEA	Macaca
1	1.00	1	1	0.03	0.034	0.022	0.031	0.03	t	33478	KSQDADYH	Homo sapiens
1	1.00	1	1	0.03	0.032	0.025	0.027	0.028	t	114854	HKVEQGASVD	Homo sapiens
1	1.00	1	1	0.04	0.04	0.025	0.04	0.042	t	28268	IRDFMEKESP	Mus musculus
1	1.00	1	1	0.05	0.05	0.05	0.05	0.048	t	159282	EENW	Oryctolagus cuniculus
1	1.00	1	1	0.01	0.012	0.013	0.013	0.01	t	48452	PLPPVA	Homo sapiens
1	1.00	1	1	0.04	0.041	0.033	0.04	0.039	t	139689	APDPSVITLENS	Oryctolagus cuniculus
1	1.00	1	1	0.04	0.042	0.038	0.04	0.039	t	36955	LKLYFEP	Homo sapiens
1	1.00	1	1	0.04	0.042	0.038	0.04	0.039	t	36955	LKLYFEP	Homo sapiens
1	1.00	1	1	0	0.002	-0.006	0.007	0	t	118824	GRRPFFHPVGEADYFEYHQE	Homo sapiens
1	1.00	1	1	0.03	0.029	0.027	0.03	0.029	t	11862	EERPPETT	Anas platyrhynchos
1	1.00	1	1	0.02	0.022	0.016	0.021	0.02	t	112837	SGYIPANTTIPF	Homo sapiens
1	1.00	1	1	0.04	0.04	0.039	0.04	0.038	t	122913	TGEPVELK	Oryctolagus cuniculus
1	1.00	1	1	-0.01	-0.01	-0.026	-0.01	-0.009	t	109325	IDGVNH	Mus musculus

1	1.00	1	1	0	0	-0.024	0.006	0.006	t	113013	GSLPQKSQRSQDEN	Rattus norvegicus
1	1.00	1	1	0.04	0.042	0.033	0.047	0.042	t	109227	GQPREPQ	Homo sapiens
1	1.00	1	1	0	0.002	0	0.003	-0.001	t	8473	DGQPAGDR	Homo sapiens
1	1.00	1	1	-0.01	-0.008	-0.017	-0.013	-0.013	t	116040	YQQKPVALIN	Homo sapiens
1	1.00	1	1	-0.07	-0.068	-0.07	-0.063	-0.073	t	134650	CRYTPRRNIP	Homo sapiens
1	1.00	1	1	0	0.002	-0.002	0.007	0.001	t	112129	GIGVGWI	Homo sapiens
1	1.00	1	1	0.04	0.04	0.034	0.04	0.038	t	155744	SAHSTPS	Homo sapiens
1	1.00	1	1	0.03	0.031	0.024	0.031	0.028	t	6721	CNPPDCPGVGTG	Myodes glareolus
1	1.00	1	1	0	0	-0.013	0	0.001	t	45589	NQMHMLSNKS	Mus musculus
1	1.00	1	1	0.01	0.012	0.008	0.013	0.01	t	29342	IVLGGGCALL	Homo sapiens
1	1.00	1	1	0.01	0.006	0.001	0.009	0.01	t	25217	IAADQLPN	Oryctolagus cuniculus
1	1.00	1	1	-0.01	-0.004	-0.021	-0.01	-0.011	t	94196	VVDNYELLKH	Ovis aries
1	1.00	1	1	-0.03	-0.026	-0.053	-0.033	-0.029	t	67859	VCKVPI	Homo sapiens
1	1.00	1	1	-0.01	-0.014	-0.012	-0.007	-0.013	t	14899	EVVLQNRRGLDLL	Oryctolagus cuniculus
1	1.00	1	1	-0.01	-0.014	-0.012	-0.007	-0.013	t	14899	EVVLQNRRGLDLL	Oryctolagus cuniculus

1	1.00	1	1	0.01	0.013	-0.006	0.01	0.012	t	98061	GFSGSVQFVP	Mus musculus
1	1.00	1	1	-0.01	-0.01	-0.027	-0.014	-0.01	t	10106	DSEPFPRQKHKKVD	Homo sapiens
1	1.00	1	1	0.01	0.008	0.005	0.007	0.007	t	115828	KPAAVRSPAQ	Homo sapiens
1	1.00	1	1	0.01	0.014	0.005	0.007	0.007	t	115828	KPAAVRSPAQ	Homo sapiens
1	1.00	1	1	0.02	0.022	0.008	0.021	0.02	t	142408	DQVTKKEKKTIKKA	Homo sapiens
1	1.00	1	1	-0.02	-0.021	-0.032	-0.023	-0.02	t	130621	TFYTK	Oryctolagus cuniculus
1	1.00	1	1	-0.02	-0.019	-0.032	-0.023	-0.02	t	130621	TFYTK	Rattus norvegicus
1	1.00	1	1	0.04	0.04	0.03	0.04	0.04	t	9980	DRGYISQY	Homo sapiens
1	1.00	1	1	0.02	0.022	0.02	0.027	0.02	t	101258	SQNALPK	Homo sapiens
1	1.00	1	1	0.01	0.014	0.008	0.017	0.011	t	116171	TQDQDPBBHFFKNIVTPR	Homo sapiens
1	1.00	1	1	0.02	0.018	0.009	0.019	0.02	t	8510	DGVFNNPI	Mus musculus
1	1.00	1	1	0	0.003	-0.01	0	-0.002	t	48715	PNVDPN	Mus musculus
1	1.00	1	1	0.01	0.01	0.01	0.02	0.01	t	115293	IMCVKKILDK	Homo sapiens
1	1.00	1	1	0.01	0.01	-0.001	0.01	0.009	t	117238	TKDTNNNL	Mus musculus

1	1.00	1	1	0.02	0.023	0.018	0.02	0.018	t	45303	NPASTTNKDK	Mus musculus
1	1.00	1	1	-0.02	-0.016	-0.036	-0.017	-0.019	t	119301	PEVVVLGSQEGAMHT	Homo sapiens
1	1.00	1	1	0.02	0.02	0.02	0.02	0.017	t	4330	ASARGDLAHLTTT	Mus musculus
1	1.00	1	1	0.02	0.02	0.013	0.02	0.019	t	138257	VLTVLHQ	Homo sapiens
1	1.00	1	1	0.04	0.042	0.028	0.041	0.04	t	142383	AEQIQAELQKVKTA	Homo sapiens
1	1.00	1	1	-0.01	-0.013	-0.025	-0.01	-0.01	t	141763	TGKGIDDANKKACCAI	Oryctolagus cuniculus
1	1.00	1	1	0.02	0.022	0.003	0.017	0.021	t	2777	ALNELNSFGDLINP	Homo sapiens
1	1.00	1	1	0.06	0.06	0.06	0.063	0.06	t	149108	SLPNLTQ	Sus scrofa
1	1.00	1	1	-0.01	-0.01	-0.009	-0.003	-0.01	t	42633	MSPVNKSFEIEVTCS	Homo sapiens
1	1.00	1	1	0.01	0.012	0.012	0.017	0.009	t	119675	WNSGALTS	Homo sapiens
1	1.00	1	1	0	0.004	0	0	0	t	139840	PAAFAFL	Ovis aries
1	1.00	1	1	0	-0.003	0	0	-0.003	t	7849	DDPPATVYRYD	Oryctolagus cuniculus
1	1.00	1	1	0	-0.003	0	0	-0.003	t	7849	DDPPATVYRYD	Oryctolagus cuniculus

1	1.00	1	1	0.01	0.01	0.01	0.01	0.008	t	117456	DGQFCH	Homo sapiens
1	1.00	1	1	0	0	-0.014	0	0	t	120951	SLDPEFLNLI	Mus musculus
1	1.00	1	1	0.01	0.006	-0.006	0.009	0.012	t	35241	LDNLGGNG	Oryctolagus cuniculus
1	1.00	1	1	-0.01	-0.008	-0.027	-0.01	-0.007	t	110795	CDLIPNLKLNNG	Homo sapiens
1	1.00	1	1	0.03	0.032	0.023	0.031	0.029	t	76192	YTTSEYPSQPPS	Homo sapiens
1	1.00	1	1	0.03	0.032	0.023	0.031	0.029	t	76192	YTTSEYPSQPPS	Homo sapiens
1	1.00	1	1	0.01	0.012	0.013	0.017	0.008	t	127923	AIQVGTSWKQVDQFLGVPYA	Homo sapiens
1	1.00	1	1	-0.01	-0.013	-0.022	-0.01	-0.01	t	42251	MNYLLFCLFFAF	Oryctolagus cuniculus
1	1.00	1	1	0.03	0.03	0.036	0.032	0.029	t	8259	DFELIK	Homo sapiens
1	1.00	1	1	0.03	0.03	0.023	0.037	0.03	t	140681	GPEYWEQQTRIAKEWEQ	Rattus norvegicus
1	1.00	1	1	0	0.002	-0.01	0.007	0.003	t	131874	EVFISAPR	Homo sapiens
1	1.00	1	1	0.03	0.03	0.024	0.03	0.029	t	48876	PPPQAPYQGY	Mus musculus
1	1.00	1	1	0	0.015	0	0.007	-0.002	t	125478	LHQEYEENLGDSIVGYKEKS	Homo sapiens
1	1.00	1	1	-0.01	-0.008	-0.02	-0.009	-0.01	t	49993	PVVAESPKKP	Homo sapiens

1	1.00	1	1	0.03	0.03	0.018	0.03	0.031	t	97539	NYTTAVDR	Mus musculus
1	1.00	1	1	-0.03	-0.028	-0.035	-0.03	-0.031	t	114223	PFSRVVHL	Rattus norvegicus
1	1.00	1	1	-0.03	-0.03	-0.031	-0.026	-0.031	t	118822	GPPRPGMMPA	Homo sapiens
1	1.00	1	1	0	0	-0.01	0	0	t	53186	RAQEGAFLTG	Homo sapiens
1	1.00	1	1	0	0.002	-0.007	0.001	0	t	34297	KWGVTTSLS	Homo sapiens
1	1.00	1	1	-0.05	-0.053	-0.063	-0.05	-0.051	t	55639	RRLGAALL	Oryctolagus cuniculus
1	1.00	1	1	0.03	0.024	0.028	0.03	0.028	t	10277	DSYGRDPYSPSQDPY	Oryctolagus cuniculus
1	1.00	1	1	-0.03	-0.028	-0.049	-0.029	-0.028	t	49459	PSPFGQAAAGDK	Homo sapiens
1	1.00	1	1	0.03	0.032	0.03	0.033	0.03	t	117919	IFIEME	Homo sapiens
1	1.00	1	1	0	0.004	-0.005	0.001	0	t	70839	VRVCEK	Homo sapiens
1	1.00	1	1	0	0.004	-0.005	0.001	0	t	70839	VRVCEK	Homo sapiens
1	1.00	1	1	0	-0.003	-0.007	0	0.001	t	36162	LGIHE	Oryctolagus cuniculus
1	1.00	1	1	-0.03	-0.026	-0.032	-0.023	-0.031	t	119554	CNVNHKPS	Homo sapiens
1	1.00	1	1	0.03	0.027	0.019	0.033	0.031	t	142150	EYMLDLQPETTD	Homo sapiens

1	1.00	1	1	0.05	0.05	0.054	0.05	0.05	t	23207	GVVTLY	Homo sapiens
1	1.00	1	1	0	-0.001	0	0.004	-0.003	t	15293	FATTPVCQ FDGN	Myodes glareolu s
1	1.00	1	1	0.01	0.01	0.003	0.01	0.01	t	130919	KFPEDFES	Homo sapiens
1	1.00	1	1	0.06	0.062	0.059	0.067	0.06	t	144320	GSEN	Homo sapiens
1	1.00	1	1	0.05	0.052	0.043	0.051	0.05	t	150971	CDNTCME SVKNGTYD	Homo sapiens
1	1.00	1	1	0	0.004	-0.009	0	0	t	93522	NVTNSLDK LI	Ovis aries
1	1.00	1	1	-0.01	-0.012	-0.017	-0.011	-0.01	t	27014	ILDTSNPV K	Mus musculu s
1	1.00	1	1	0	0.004	-0.011	0	-0.001	t	93328	LSKVVDNY EL	Ovis aries
1	1.00	1	1	0.03	0.026	0.02	0.033	0.03	t	51077	QIHTL	Oryctola gus cuniculu s
1	1.00	1	1	0	0.004	-0.02	0.001	0.002	t	66666	TTIVHDKN	Homo sapiens
1	1.00	1	1	0.01	0.007	0	0.01	0.009	t	59972	SPAFTKGE KVDLN	Oryctola gus cuniculu s
1	1.00	1	1	0	0.004	-0.007	0.003	0	t	13728	EPPVVHG C	Homo sapiens
1	1.00	1	1	0	0.002	0	0.007	-0.001	t	108074	PQVYTLP	Homo sapiens
1	1.00	1	1	0.03	0.03	0.019	0.03	0.03	t	25538	IDANDCLV TPSVK	Homo sapiens

1	1.00	1	1	0	-0.004	0	0.003	-0.001	t	115397	NLLSICKTAEF	Oryctolagus cuniculus
1	1.00	1	1	0.04	0.04	0.024	0.042	0.043	t	99057	WQIPEQSQCQAI	Homo sapiens
1	1.00	1	1	-0.02	-0.012	-0.018	-0.02	-0.025	t	156974	WGTHTSYPWRLS	Gallus gallus
1	1.00	1	1	0.01	0.01	0.001	0.01	0.01	t	800	ADYHRVMY	Homo sapiens
1	1.00	1	1	0.01	0.008	0.006	0.009	0.009	t	48279	PLATQPPVLAL	Mus musculus
1	1.00	1	1	0.07	0.07	0.07	0.07	0.069	t	159283	EGP	Oryctolagus cuniculus
1	1.00	1	1	0.03	0.031	0.024	0.031	0.028	t	6721	CNPPDCPGVGTG	Myodes glareolus
1	1.00	1	1	0.04	0.04	0.033	0.04	0.04	t	48115	PKPKVASEAFMST	Homo sapiens
1	1.00	1	1	-0.03	-0.028	-0.031	-0.023	-0.032	t	134803	THLLIRKLPFSRLAR	Homo sapiens
1	1.00	1	1	0.03	0.032	0.02	0.031	0.029	t	44613	NKVDAAVA	Homo sapiens
1	1.00	1	1	0	0	-0.008	0	-0.001	t	79111	VNKGYTGY	Mus musculus
1	1.00	1	1	0.01	0.01	0.015	0.017	0.01	t	125027	EHIEL	Homo sapiens
1	1.00	1	1	0.03	0.03	0.03	0.03	0.028	t	140462	FNTKHDMQGDTS	Mus musculus
1	1.00	1	1	-0.01	-0.007	-0.014	-0.01	-0.013	t	50177	PYTSTLLP	Mus musculus

1	1.00	1	1	0	-0.004	-0.004	-0.001	-0.001	t	43106	MYYLKNTNF	Oryctolagus cuniculus
1	1.00	1	1	-0.03	-0.03	-0.03	-0.03	-0.033	t	115530	VAGTWYSLAM	Homo sapiens
1	1.00	1	1	-0.05	-0.052	-0.065	-0.051	-0.05	t	50350	QAQYNQMHML	Mus musculus
1	1.00	1	1	0.03	0.03	0.027	0.037	0.03	t	120167	RFQSSAVMALQEACEAYL	Mus musculus
1	1.00	1	1	-0.01	-0.01	-0.019	-0.01	-0.012	t	60267	SPSQDPYSPSQDPDR	Homo sapiens
1	1.00	1	1	0	0.004	-0.007	0.007	0	t	128038	KSPKKAKAAKPKKAP	Homo sapiens
1	1.00	1	1	0.04	0.04	0.033	0.04	0.038	t	102816	SPVPQD	Mus musculus
1	1.00	1	1	0	0.001	-0.001	0.003	-0.001	t	99260	FVDRLL	Equus caballus
1	1.00	1	1	0.05	0.048	0.043	0.05	0.05	t	50871	QGGNIVDIDF	Oryctolagus cuniculus
1	1.00	1	1	0.01	0.007	0.001	0.01	0.01	t	62907	TALDTSNK	Oryctolagus cuniculus
1	1.00	1	1	0.03	0.032	0.023	0.037	0.03	t	108080	QGKVGPSGAPG	Homo sapiens
1	1.00	1	1	-0.01	-0.008	-0.017	-0.007	-0.008	t	78024	SYTRLFLIL	Homo sapiens
1	1.00	1	1	0.01	0.01	-0.002	0.01	0.01	t	78365	DSWDHEQA	Mus musculus
1	1.00	1	1	0.02	0.02	0.012	0.02	0.019	t	30180	KDKDKDNTDE	Mus musculus

1	1.00	1	1	0.04	0.037	0.033	0.04	0.04	t	37325	LLGEATAL	Oryctolagus cuniculus
1	1.00	1	1	0.01	0.014	-0.001	0.01	0.009	t	93326	LSFSDSNLKD	Ovis aries
1	1.00	1	1	0.01	0.014	0.007	0.011	0.006	t	89854	VAKLTVMVYN	Homo sapiens
1	1.00	1	1	0.04	0.037	0.033	0.047	0.042	t	136704	VEALAFPDTDFPGLITLTIS	Homo sapiens
1	1.00	1	1	0.03	0.03	0.024	0.03	0.028	t	19375	GENK	Mus musculus
1	1.00	1	1	-0.02	-0.018	-0.027	-0.017	-0.02	t	144652	GMRGMKGLVY	Homo sapiens
1	1.00	1	1	0.04	0.04	0.04	0.04	0.036	t	6457	CITQYQRESQAYYQRG	Mus musculus
1	1.00	1	1	0.03	0.03	0.03	0.034	0.029	t	119576	EYKCKVSN	Homo sapiens
1	1.00	1	1	0.01	0.01	0	0.01	0.009	t	78461	LYVCLNET	Mus musculus
1	1.00	1	1	0.05	0.052	0.04	0.057	0.052	t	125229	RDSEGDLVAEKLGPI	Homo sapiens
1	1.00	1	1	0.02	0.022	0.013	0.027	0.022	t	109530	LTKNQVS	Homo sapiens
1	1.00	1	1	0.02	0.02	0.015	0.02	0.02	t	7058	CSQAAPYIE	Homo sapiens
1	1.00	1	1	-0.02	-0.018	-0.028	-0.02	-0.021	t	48926	PPSSPPQW	Anas platyrhynchos
1	1.00	1	1	0	0.027	-0.013	0	-0.002	t	61827	STPPLPWPWSPAAL	Mustela

1	1.00	1	1	0	0.027	-0.013	0	-0.002	t	61827	STPPLPWP WSPAAL	Mustela
1	1.00	1	1	0.06	0.062	0.056	0.061	0.06	t	144925	NETIYNTTL K	Homo sapiens
1	1.00	1	1	0.01	0.01	0.018	0.01	0.005	t	59975	SPAIFQSS M	Mus musculu s
1	1.00	1	1	0.02	0.02	0.019	0.03	0.022	t	106235	AGFNVPW	Homo sapiens
1	1.00	1	1	0	0.002	-0.007	0.001	0	t	98438	SAVGKTVK VY	Homo sapiens
1	1.00	1	1	0.01	0.012	0.01	0.011	0.01	t	64004	TGYGTV	Homo sapiens
1	1.00	1	1	-0.05	-0.048	-0.05	-0.043	-0.049	t	118864	KIPPTPFS	Homo sapiens
1	1.00	1	1	0.01	0.01	0.01	0.01	0.007	t	134200	PVVNSLDP PLLTRY	Homo sapiens
1	1.00	1	1	0.02	0.022	0.01	0.023	0.02	t	1595	AGLFLTTE AV	Homo sapiens
1	1.00	1	1	0.02	0.02	0.018	0.027	0.02	t	135953	LLQYVVKS FDRS	Homo sapiens
1	1.00	1	1	0.03	0.034	0.03	0.031	0.027	t	134696	ITLYLTEK	MD
1	1.00	1	1	0	-0.002	0	0.007	0	t	123314	IGTFKAFD	Homo sapiens
1	1.00	1	1	0.02	0.016	0.014	0.02	0.018	t	29567	IYDKLTVQ TTG	Oryctola gus cuniculu s
1	1.00	1	1	0.04	0.036	0.028	0.04	0.04	t	144538	VVDAEVV DDDRESK	Bos taurus
1	1.00	1	1	0.04	0.039	0.03	0.04	0.04	t	9669	DPLLGNQS	Anas platyrhy nchos

1	1.00	1	1	0.01	0.014	0.005	0.013	0.009	t	119466	IRGERA	Homo sapiens
1	1.00	1	1	0.05	0.05	0.047	0.05	0.05	t	61997	STVTEN	Homo sapiens
1	1.00	1	1	0.03	0.028	0.024	0.03	0.028	t	30561	KEPGVLM GGQSESVE	Mus musculu s
1	1.00	1	1	0.02	0.018	0.018	0.027	0.021	t	3517	APDTRPAP	Mus musculu s
1	1.00	1	1	-0.03	-0.032	-0.042	-0.033	-0.032	t	25664	IDRSLPPIV K	Homo sapiens
1	1.00	1	1	0.01	0.014	-0.002	0.009	0.01	t	141559	SDEALPLG SPR	Mus musculu s
1	1.00	1	1	-0.01	-0.006	-0.017	-0.003	-0.008	t	144700	KEWAKRA ALP	Homo sapiens
1	1.00	1	1	0.04	0.04	0.04	0.044	0.04	t	157130	CIIEEN	Homo sapiens
1	1.00	1	1	0	0	-0.017	0	0.004	t	32802	KPNDKSLY	Mus musculu s
1	1.00	1	1	-0.03	-0.03	-0.03	-0.023	-0.03	t	112295	RMRDGA W	Homo sapiens
1	1.00	1	1	-0.01	-0.017	-0.027	-0.01	-0.007	t	110932	LNLKIDGL KISV	Macaca
1	1.00	1	1	0.05	0.046	0.048	0.049	0.05	t	65193	TMEEINTL	Anas platyrhy nchos
1	1.00	1	1	-0.02	-0.02	-0.025	-0.02	-0.02	t	48409	PLLRTQF	Mus musculu s
1	1.00	1	1	-0.02	-0.023	-0.025	-0.02	-0.02	t	48409	PLLRTQF	Oryctola gus cuniculu s

1	1.00	1	1	0.01	0.006	0.004	0.01	0.01	t	6068	CCPDKNKS	Mus musculus
1	1.00	1	1	0.01	0.008	-0.004	0.009	0.01	t	39251	LRYGNVLDVN	Mus musculus
1	1.00	1	1	0.07	0.07	0.07	0.073	0.07	t	149106	SITNPTQ	Sus scrofa
1	1.00	1	1	0.07	0.066	0.066	0.073	0.072	t	105721	DQISTVQA	Oryctolagus cuniculus
1	1.00	1	1	0.03	0.034	0.023	0.037	0.03	t	20097	GGYGDGGAHGGGYGG	Homo sapiens
1	1.00	1	1	0.06	0.064	0.06	0.067	0.06	t	118373	GIEADDDRLNKVISE	Homo sapiens
1	1.00	1	1	0.04	0.043	0.038	0.04	0.039	t	89661	TQLTSSYEDYML	Mus musculus
1	1.00	1	1	0.01	0.01	0.01	0.013	0.01	t	3639	APLGVT	Homo sapiens
1	1.00	1	1	0.01	0.01	0	0.01	0.01	t	21566	GPAFHER	Mus musculus
1	1.00	1	1	0.07	0.073	0.058	0.07	0.07	t	97667	STTDSFSD	Mus musculus
1	1.00	1	1	0.03	0.032	0.028	0.031	0.03	t	63805	TGDLRAY	Homo sapiens
1	1.00	1	1	0.03	0.032	0.028	0.031	0.03	t	63805	TGDLRAY	Homo sapiens
1	1.00	1	1	0.03	0.03	0.021	0.03	0.029	t	110941	MHGDTPTLHEYMLDLQPE	Mus musculus
1	1.00	1	1	0.01	0.013	0.008	0.01	0.007	t	32089	KLPTPIELPLK	Oryctolagus cuniculus

1	1.00	1	1	-0.01	-0.008	-0.015	-0.009	-0.01	t	20082	GGVYLLPR	Homo sapiens
1	1.00	1	1	0	-0.002	0	-0.001	-0.004	t	59374	SLNTGWL AGLFY	Mus musculu s
1	1.00	1	1	0	-0.002	-0.009	0.003	-0.001	t	47014	PAYYTKDT NNNLTL	Mus musculu s
1	1.00	1	1	0.02	0.022	0.015	0.021	0.02	t	38404	LPALSTGL	Homo sapiens
1	1.00	1	1	0.01	0.01	0.005	0.01	0.01	t	78433	KLYVCLNE	Mus musculu s
1	1.00	1	1	0.01	0.01	0.005	0.01	0.01	t	78433	KLYVCLNE	Mus musculu s
1	1.00	1	1	-0.02	-0.022	-0.02	-0.021	-0.022	t	40922	LYREFD	Mus musculu s
1	1.00	1	1	0.02	0.022	0.025	0.027	0.02	t	112801	LATPNPS	Homo sapiens
1	1.00	1	1	0.03	0.022	0.022	0.029	0.03	t	110964	PNLKLNN GTDYK	Macaca
1	1.00	1	1	-0.01	-0.01	-0.023	-0.01	-0.01	t	96849	SAETIF	Mus musculu s
1	1.00	1	1	-0.01	-0.01	-0.023	-0.01	-0.01	t	96849	SAETIF	Mus musculu s
1	1.00	1	1	0.03	0.03	0.03	0.037	0.03	t	135974	PSNSASVP ALTA	Homo sapiens
1	1.00	1	1	-0.01	-0.01	-0.01	-0.01	-0.012	t	117600	PKKAK	Homo sapiens
1	1.00	1	1	-0.02	-0.018	-0.028	-0.017	-0.02	t	110913	KDIKANPE NYFT	Homo sapiens
1	1.00	1	1	0.01	0.012	0.01	0.01	0.01	t	128955	ILFSVFD	MD

1	1.00	1	1	0.02	0.017	0.003	0.02	0.022	t	141763	TGKGIDDA NKKACCAI	Oryctola gus cuniculu s
1	1.00	1	1	0.02	0.02	0.003	0.02	0.022	t	141763	TGKGIDDA NKKACCAI	Rattus norvegic us
1	1.00	1	1	0.03	0.034	0.03	0.031	0.027	t	134696	ITLYLTEK	MD
1	1.00	1	1	-0.02	-0.022	-0.028	-0.019	-0.019	t	1373	AFPL	Mus musculu s
1	1.00	1	1	-0.01	-0.006	-0.01	-0.003	-0.008	t	108075	PREPQVY	Homo sapiens
1	1.00	1	1	-0.04	-0.04	-0.047	-0.034	-0.041	t	10987	EAAGIGILT V	Homo sapiens
1	1.00	1	1	0	0	0	0.007	-0.002	t	125552	RRFEYDDP RFLRLLDLA QEG	Homo sapiens
1	1.00	1	1	0.01	0.01	0.007	0.012	0.01	t	383	AAPYIEQA QVIAHQ	Homo sapiens
1	1.00	1	1	0.01	0.012	0.003	0.007	0.008	t	73334	YAATVAA APEVKY	Homo sapiens
1	1.00	1	1	0.02	0.02	0.02	0.027	0.019	t	125284	TYICEVED QKEE	Mus musculu s
1	1.00	1	1	-0.01	-0.013	-0.02	-0.01	-0.01	t	110839	ETTSQMIV KDIK	Macaca
1	1.00	1	1	0.03	0.028	0.02	0.033	0.033	t	78256	QYPALTA	Mus musculu s
1	1.00	1	1	0.01	0.01	0.008	0.01	0.008	t	75370	YPNQV	Mus musculu s
1	1.00	1	1	0.01	0.01	0.008	0.01	0.008	t	75370	YPNQV	Mus musculu s

1	1.00	1	1	0.05	0.048	0.033	0.049	0.051	t	78148	ENQANPTTAET	Mus musculus
1	1.00	1	1	0	0	-0.005	0	-0.001	t	26339	IGVKGSSI	Oryctolagus cuniculus
1	1.00	1	1	0.02	0.022	0.018	0.027	0.02	t	126227	HNNAHYYVFFEEQED	Homo sapiens
1	1.00	1	1	0	-0.003	-0.017	-0.003	0.003	t	111038	TGALSKIY	Oryctolagus cuniculus
1	1.00	1	1	0.01	0.013	0.01	0.01	0.006	t	21407	GNDYEDRYYREN	Mus musculus
1	1.00	1	1	0.06	0.056	0.059	0.057	0.058	t	118991	RAEDNADTLALVFEA	Oryctolagus cuniculus
1	1.00	1	1	0	0	-0.012	0	0	t	8972	DKPSYQFGGH	Oryctolagus cuniculus
1	1.00	1	1	0.03	0.032	0.023	0.031	0.032	t	40649	LVYQTEKF	Homo sapiens
1	1.00	1	1	-0.03	-0.025	-0.037	-0.027	-0.03	t	72236	WAKHMWNFI	Homo sapiens
1	1.00	1	1	-0.02	-0.018	-0.028	-0.023	-0.023	t	30115	KDASIQVVSA	Homo sapiens
1	1.00	1	1	0.02	0.02	0.008	0.016	0.02	t	30799	KFNPDLAPG	Homo sapiens
1	1.00	1	1	0.01	0.012	0.009	0.011	0.01	t	47426	PESPFA	Homo sapiens
1	1.00	1	1	-0.01	-0.012	-0.019	-0.007	-0.009	t	115483	SFFDIGGSVSAGFSN	Mus musculus

1	1.00	1	1	0.03	0.033	0.02	0.03	0.031	t	132754	YER	Mus musculus
1	1.00	1	1	0.01	0.005	0	0.01	0.01	t	127782	QAAELTLGPSAC	Trichosurus vulpecula
1	1.00	1	1	-0.03	-0.037	-0.037	-0.023	-0.028	t	156885	GYRAR	Homo sapiens
1	1.00	1	1	0.01	0.014	0.01	0.01	0.008	t	22357	GSGVRGDFG	Cavia porcellus
1	1.00	1	1	0.04	0.038	0.028	0.043	0.042	t	95723	QNDPTT	Mus musculus
1	1.00	1	1	0	0.002	-0.017	0	0.001	t	68833	VHASFFDIG	Homo sapiens
1	1.00	1	1	0	0	-0.017	0	0.001	t	68833	VHASFFDIG	Mus musculus
1	1.00	1	1	0	-0.004	-0.005	0	-0.002	t	99914	CGQGIDCGPIQP	Oryctolagus cuniculus
1	1.00	1	1	0.03	0.032	0.025	0.033	0.032	t	71203	VSVPAEIL	Homo sapiens
1	1.00	1	1	-0.01	-0.01	-0.01	0	-0.01	t	33732	KTKIPAVFKI	Homo sapiens
1	1.00	1	1	0	0.002	0	0.007	-0.001	t	119622	NVNHKPSN	Homo sapiens
1	1.00	1	1	0	0	0	0	-0.004	t	10124	DSGCVVSWK	Homo sapiens
1	1.00	1	1	0.01	0.01	0	0.016	0.012	t	8473	DGQPAGDR	Homo sapiens
1	1.00	1	1	0.02	0.02	0.018	0.023	0.02	t	47674	PGHLAP	Homo sapiens

1	1.00	1	1	-0.03	-0.032	-0.027	-0.031	-0.032	t	122138	LKKDV	Mus musculus
1	1.00	1	1	0.01	0.008	0.012	0.009	0.009	t	15807	FFFDIDLLL	Mus musculus
1	1.00	1	1	0.06	0.06	0.06	0.06	0.058	t	156756	SGPSYTT	Mus musculus
1	1.00	1	1	-0.02	-0.022	-0.025	-0.021	-0.022	t	74476	YKLPDHVRT	Mus musculus
1	1.00	1	1	-0.03	-0.025	-0.027	-0.028	-0.033	t	157292	GAIIGLM	Homo sapiens
1	1.00	1	1	-0.02	-0.02	-0.028	-0.017	-0.021	t	47983	PISLERLDVGTNLGN	Homo sapiens
1	1.00	1	1	-0.04	-0.04	-0.047	-0.043	-0.04	t	46987	PAVFK	Homo sapiens
1	1.00	1	1	0	0	-0.006	0	-0.001	t	30661	KEVPALTAVETGAT	Homo sapiens
1	1.00	1	1	-0.04	-0.034	-0.043	-0.04	-0.04	t	139898	SLPWPFP	Ovis aries
1	1.00	1	1	0.02	0.018	0.019	0.02	0.019	t	134471	PKPEQ	Mus musculus
1	1.00	1	1	0	0	-0.01	0	0	t	21422	GNFERISGDLKTQID	Homo sapiens
1	1.00	1	1	-0.01	-0.006	-0.017	-0.003	-0.008	t	144816	WSHNFTNMLG	Homo sapiens
1	1.00	1	1	-0.02	-0.018	-0.027	-0.013	-0.02	t	117281	DVVYALKRQGRTLYGFGG	Homo sapiens

1	1.00	1	1	-0.02	-0.018	-0.027	-0.013	-0.02	t	117281	DVVYALKRQGRTLYGFGG	Homo sapiens
1	1.00	1	1	-0.02	-0.018	-0.022	-0.016	-0.02	t	118548	PAPGMRPP	Homo sapiens
1	1.00	1	1	-0.02	-0.027	-0.02	-0.019	-0.024	t	50965	QGTDYKHW	Homo sapiens
1	1.00	1	1	0	-0.002	-0.007	-0.001	-0.002	t	33244	KRVVIN	Mus musculus
1	1.00	1	1	-0.02	-0.018	-0.026	-0.02	-0.021	t	27085	ILGWPLAERT	Homo sapiens
1	1.00	1	1	0.02	0.018	0.013	0.02	0.02	t	65852	TQGIVEFY	Oryctolagus cuniculus
1	1.00	1	1	0.01	0.01	0.006	0.008	0.008	t	96598	LPPKKIKDPD	Homo sapiens
1	1.00	1	1	0.03	0.032	0.035	0.037	0.028	t	121347	GPLTVAQKKAQNIME	Homo sapiens
1	1.00	1	1	0.03	0.032	0.035	0.037	0.028	t	121347	GPLTVAQKKAQNIME	Homo sapiens
1	1.00	1	1	-0.02	0.004	-0.029	-0.02	-0.02	t	113597	LCLPLAPQGLL	Rattus
1	1.00	1	1	0.05	0.046	0.047	0.049	0.05	t	4771	ASSGIGPEKA	Oryctolagus cuniculus
1	1.00	1	1	0	0.002	-0.007	-0.003	-0.002	t	115690	AQTQSLVYPF	Homo sapiens
1	1.00	1	1	0.09	0.09	0.09	0.097	0.091	t	133692	SG	Homo sapiens
1	1.00	1	1	-0.04	-0.038	-0.047	-0.043	-0.041	t	51431	QLLRLKKYKV	Homo sapiens
1	1.00	1	1	-0.01	-0.008	-0.016	-0.003	-0.009	t	144823	YWEGSGIGAI	Homo sapiens

1	1.00	1	1	0.03	0.032	0.022	0.031	0.03	t	61824	STPLLYPSLA	Homo sapiens
1	1.00	1	1	0.03	0.032	0.022	0.031	0.03	t	61824	STPLLYPSLA	Homo sapiens
1	1.00	1	1	-0.02	-0.019	-0.021	-0.017	-0.021	t	99260	FVDRLL	Equus caballus
1	1.00	1	1	0.03	0.03	0.023	0.04	0.031	t	117383	TLHKAFKGSIFVVFDSIESA	Homo sapiens
1	1.00	1	1	0.03	0.03	0.02	0.033	0.03	t	30877	KFVKEQRE	Homo sapiens
1	1.00	1	1	0	-0.003	-0.004	0	-0.002	t	19532	GFGHIGVTVDDVHKA	Oryctolagus cuniculus
1	1.00	1	1	0	-0.003	-0.004	0	-0.002	t	19532	GFGHIGVTVDDVHKA	Oryctolagus cuniculus
1	1.00	1	1	0	-0.003	-0.004	0	-0.002	t	19532	GFGHIGVTVDDVHKA	Oryctolagus cuniculus
1	1.00	1	1	0.03	0.03	0.02	0.03	0.03	t	155334	PADSS	Oryctolagus cuniculus
1	1.00	1	1	0.02	0.024	0.018	0.027	0.019	t	119855	FAESRIRRETIAAED	Homo sapiens
1	1.00	1	1	0.02	0.024	0.018	0.027	0.019	t	119855	FAESRIRRETIAAED	Homo sapiens
1	1.00	1	1	0.01	0.007	0.004	0.011	0.009	t	72616	WIGNGYRY	Homo sapiens
1	1.00	1	1	0.02	0.02	0.02	0.02	0.018	t	140461	EVSLHDMNLATH	Mus musculus

1	1.00	1	1	0.02	0.016	0.013	0.023	0.02	t	107247	TFGSGTRLTV	Oryctolagus cuniculus
1	1.00	1	1	0.01	0.009	0.003	0.01	0.008	t	56768	SAEYEKSKVNKYLKTIMPF	Equus caballus
1	1.00	1	1	0	0.004	0	0.007	0	t	136063	FIRFDKRIEAEE	Homo sapiens
1	1.00	1	1	0.07	0.072	0.069	0.071	0.069	t	69624	VLLQME	Homo sapiens
1	1.00	1	1	0.01	0.008	0.01	0.011	0.009	t	130454	CLFPNNSYC	Mus musculus
1	1.00	1	1	-0.01	-0.012	-0.019	-0.011	-0.01	t	39085	LRFGSK	Mus musculus
1	1.00	1	1	0.03	0.03	0.024	0.03	0.03	t	42984	MVQVVYD	Homo sapiens
1	1.00	1	1	-0.01	-0.01	-0.016	-0.01	-0.01	t	31787	KLDLYGKVH	Sus scrofa
1	1.00	1	1	0.07	0.07	0.07	0.073	0.07	t	149106	SITNPTQ	Sus scrofa
1	1.00	1	1	0	-0.004	-0.006	-0.001	-0.002	t	40159	LTSAAILGLDGTVW	Oryctolagus cuniculus
1	1.00	1	1	-0.04	-0.038	-0.047	-0.033	-0.041	t	136007	VKEKGMAALPRL	Homo sapiens
1	1.00	1	1	-0.03	-0.022	-0.038	-0.033	-0.032	t	14380	ETFTNKLKEKHTD	Homo sapiens
1	1.00	1	1	0.01	0.014	0.01	0.017	0.01	t	123612	PPGMIPPP	Homo sapiens
1	1.00	1	1	0.07	0.072	0.061	0.073	0.072	t	34908	LAPLGE	Homo sapiens

1	1.00	1	1	0.06	0.057	0.05	0.061	0.061	t	89745	TTELTAENQE	Homo sapiens
1	1.00	1	1	0.02	0.017	0.003	0.017	0.023	t	110952	NDLQTVLG	Oryctolagus cuniculus
1	1.00	1	1	0	-0.004	-0.02	0.003	0.003	t	141618	DLMNEVLNKMDKKYSA	Oryctolagus cuniculus
1	1.00	1	1	0	-0.002	-0.02	0.003	0.003	t	141618	DLMNEVLNKMDKKYSA	Rattus norvegicus
1	1.00	1	1	-0.01	-0.008	-0.011	-0.009	-0.01	t	72413	WFLDLP	Homo sapiens
1	1.00	1	1	0.03	0.03	0.021	0.034	0.03	t	135908	DINTVTTVAQSR	Homo sapiens
1	1.00	1	1	0.04	0.036	0.034	0.039	0.038	t	155744	SAHSTPS	Oryctolagus cuniculus
1	1.00	1	1	0.01	0.01	0.01	0.02	0.01	t	3985	AQKKIIAEKT	Homo sapiens
1	1.00	1	1	0.07	0.067	0.064	0.07	0.07	t	50396	QAYQQGVTVD	Oryctolagus cuniculus
1	1.00	1	1	0.01	0.006	-0.006	0.013	0.012	t	105412	NQTKV	Oryctolagus cuniculus
1	1.00	1	1	0.04	0.04	0.027	0.04	0.042	t	75479	YQELGV	Homo sapiens
1	1.00	1	1	0	0.001	-0.001	0.003	-0.001	t	99779	YACRDI	Equus caballus
1	1.00	1	1	0.03	0.03	0.023	0.03	0.031	t	107702	YVDGVQV	Homo sapiens
1	1.00	1	1	0.05	0.047	0.04	0.051	0.051	t	89745	TTELTAENQE	Homo sapiens
1	1.00	1	1	0.01	0.014	-0.002	0.009	0.01	t	141560	SDEALPPGSPR	Mus musculus

1	1.00	1	1	0.01	0.01	0.01	0.01	0.01	t	135506	VTFPGA	Oryctolagus cuniculus
1	1.00	1	1	-0.03	-0.027	-0.04	-0.03	-0.029	t	156553	YSTCYFIM	Mus musculus
1	1.00	1	1	0.04	0.042	0.04	0.047	0.039	t	119670	WESNGQPE	Homo sapiens
1	1.00	1	1	-0.02	-0.02	-0.02	-0.01	-0.02	t	114948	SIEFGTNISK	Homo sapiens
1	1.00	1	1	0	-0.003	-0.002	0.001	-0.003	t	103143	EMIEAVGYRSWPRYF	Homo sapiens
1	1.00	1	1	0.06	0.058	0.053	0.059	0.06	t	96067	AETIFD	Mus musculus
1	1.00	1	1	0.06	0.055	0.053	0.059	0.06	t	96067	AETIFD	Oryctolagus cuniculus
1	1.00	1	1	0.06	0.058	0.053	0.059	0.06	t	96067	AETIFD	Mus musculus
1	1.00	1	1	0.01	0.007	0	0.01	0.01	t	26047	IFDVTTL	Oryctolagus cuniculus
1	1.00	1	1	0.01	0.01	-0.001	0.01	0.01	t	38700	LPSAVK	Mus musculus
1	1.00	1	1	0.05	0.048	0.041	0.043	0.05	t	110845	EYMLD	Mus musculus
1	1.00	1	1	0.05	0.048	0.041	0.043	0.05	t	110845	EYMLD	Mus musculus
1	1.00	1	1	0.05	0.048	0.041	0.043	0.05	t	110845	EYMLD	Mus musculus
1	1.00	1	1	0.05	0.048	0.041	0.043	0.05	t	110845	EYMLD	Mus musculus
1	1.00	1	1	0.05	0.048	0.041	0.043	0.05	t	110845	EYMLD	Mus musculus

1	1.00	1	1	0.01	0.012	-0.002	0.011	0.01	t	142383	AEQIQAEL QKVKTA	Homo sapiens
1	1.00	1	1	0.04	0.04	0.041	0.037	0.039	t	19607	GFQYSP	Homo sapiens
1	1.00	1	1	-0.03	-0.03	-0.04	-0.027	-0.031	t	20663	GKPVVQTI LA	Homo sapiens
1	1.00	1	1	0.02	0.018	0.014	0.02	0.02	t	9591	DPAKAAF D	Mus musculu s
1	1.00	1	1	0.05	0.05	0.05	0.051	0.051	t	130643	YSLLVEPYT FDP	Homo sapiens
1	1.00	1	1	0.01	0.01	0.01	0.01	0.007	t	45004	NMMKYIS PPIFL	Oryctola gus cuniculu s
1	1.00	1	1	-0.01	-0.012	-0.03	-0.007	-0.007	t	141771	VAEREAYY LWKQYNP	Rattus norvegic us
1	1.00	1	1	-0.01	-0.012	-0.01	-0.009	-0.013	t	130457	CRQPGNN YC	Mus musculu s
1	1.00	1	1	0.02	0.016	0.022	0.02	0.018	t	99985	EFRHDSGY EV	Oryctola gus cuniculu s
1	1.00	1	1	0	0.002	0	0.001	0	t	29126	ITTYV	Homo sapiens
1	1.00	1	1	-0.01	-0.01	-0.01	-0.008	-0.011	t	157292	GAIIGLM	Homo sapiens
1	1.00	1	1	0.01	0.012	0.012	0.011	0.009	t	52273	QRVEFL	Homo sapiens
1	1.00	1	1	0	0	-0.024	0.006	0.006	t	113013	GSLPQKSQ RSQDEN	Rattus norvegic us
1	1.00	1	1	0	0.002	0.017	0.007	-0.001	t	109251	GSFFLYS	Homo sapiens

1	1.00	1	1	-0.05	-0.048	-0.064	-0.047	-0.05	t	19108	GDRRKAMFEDIA	Homo sapiens
1	1.00	1	1	-0.01	-0.01	-0.01	-0.01	-0.011	t	76586	YYR	Mus musculus
1	1.00	1	1	-0.07	-0.068	-0.07	-0.063	-0.073	t	134650	CRYTPRRNIP	Homo sapiens
1	1.00	1	1	0.03	0.032	0.029	0.03	0.029	t	21631	GPGEVWVT	Homo sapiens
1	1.00	1	1	0.01	0.01	0.01	0.01	0.007	t	50929	QGPGAP	Mus musculus
1	1.00	1	1	0.01	0.01	0.01	0.01	0.007	t	50929	QGPGAP	Mus musculus
1	1.00	1	1	0.01	0.008	0.01	0.013	0.01	t	3382	ANPPDH	Homo sapiens
1	1.00	1	1	0.04	0.037	0.034	0.04	0.038	t	133540	DALVAKAQEVS	Oryctolagus cuniculus
1	1.00	1	1	0.07	0.072	0.07	0.077	0.07	t	117547	KLEVQATDREENKQIALGTS	Homo sapiens
1	1.00	1	1	0	-0.004	-0.005	0.001	-0.002	t	79026	MSDGAVQPDGGQPAV	Oryctolagus cuniculus
1	1.00	1	1	-0.01	-0.008	-0.017	-0.003	-0.01	t	144594	ARGISRTKYW	Homo sapiens
1	1.00	1	1	0.03	0.03	0.03	0.033	0.03	t	38631	LPPVAD	Homo sapiens
1	1.00	1	1	0.04	0.036	0.034	0.041	0.039	t	59869	SNNPE	Bos taurus
1	1.00	1	1	-0.01	-0.014	-0.01	-0.007	-0.01	t	105717	DPSIMAKF	Oryctolagus cuniculus
1	1.00	1	1	0.03	0.034	0.011	0.027	0.031	t	39789	LTAENTAI	Homo sapiens

1	1.00	1	1	-0.01	-0.008	-0.019	-0.013	-0.012	t	67524	VAALNRRIQLLEEDL	Homo sapiens
1	1.00	1	1	-0.03	-0.03	-0.03	-0.02	-0.03	t	98845	KFDKALKALP	Homo sapiens
1	1.00	1	1	-0.05	-0.049	-0.067	-0.05	-0.05	t	66075	TRPHGR	Mus musculus
1	1.00	1	1	0.01	0.008	-0.001	0.009	0.01	t	120419	AVKYTGYTGG	Mus musculus
1	1.00	1	1	0	0.002	-0.007	-0.003	-0.003	t	27650	INHFVLLVGY	Homo sapiens
1	1.00	1	1	-0.01	-0.01	-0.02	-0.01	-0.01	t	49993	PVVAESPKKP	Mus musculus
1	1.00	1	1	0.03	0.035	0.021	0.031	0.03	t	47483	PFCGESTLYT	Homo sapiens
1	1.00	1	1	0.02	0.02	0.02	0.02	0.018	t	73384	YALADASLKMAD	Oryctolagus cuniculus
1	1.00	1	1	0.01	0.01	-0.002	0.01	0.01	t	25622	IDLTNKNAA	Homo sapiens
1	1.00	1	1	-0.03	-0.03	-0.042	-0.03	-0.03	t	78452	LNVAHQLA	Mus musculus
1	1.00	1	1	0.01	0.01	-0.002	0.01	0.01	t	25623	IDLTNKNAAK	Homo sapiens
1	1.00	1	1	0.04	0.04	0.033	0.04	0.04	t	119478	KNDK	Homo sapiens
1	1.00	1	1	-0.03	-0.032	-0.042	-0.03	-0.03	t	100250	LDVRKQYKRD	Mus musculus
1	1.00	1	1	0.04	0.04	0.04	0.04	0.039	t	118038	NLNEKIKKD	Homo sapiens
1	1.00	1	1	0.05	0.048	0.05	0.043	0.045	t	134669	FGFPQE	Mus musculus

1	1.00	1	1	0.03	0.032	0.027	0.03	0.03	t	48990	PQEIPQPQ	Anas platyrhynchos
1	1.00	1	1	0.03	0.032	0.013	0.031	0.032	t	68745	VGNYDFWYQ	Homo sapiens
1	1.00	1	1	-0.01	-0.008	-0.018	-0.009	-0.01	t	49993	PVVAESPKKP	Homo sapiens
1	1.00	1	1	0.03	0.028	0.025	0.031	0.03	t	38956	LQQATRQAQ	Homo sapiens
1	1.00	1	1	0.03	0.028	0.025	0.031	0.03	t	38970	LQRATQQQA	Homo sapiens
1	1.00	1	1	0.01	0.01	-0.005	0.013	0.012	t	39626	LSREM	Homo sapiens
1	1.00	1	1	0	0.004	0	0.007	0	t	131818	AKEERRY	Homo sapiens
1	1.00	1	1	0.01	0.012	0.01	0.017	0.01	t	134781	RGDGPFRLGG	Homo sapiens
1	1.00	1	1	0.04	0.038	0.04	0.039	0.04	t	110962	PIDVVDP	Mus musculus
1	1.00	1	1	0.03	0.032	0.016	0.03	0.03	t	140080	YEKEPTPPTRTPDQ	Rattus norvegicus
1	1.00	1	1	0.03	0.032	0.016	0.03	0.03	t	140080	YEKEPTPPTRTPDQ	Rattus norvegicus
1	1.00	1	1	0.07	0.078	0.07	0.07	0.067	t	136365	IDSNHVYKDFLT	Gallus gallus
1	1.00	1	1	-0.01	-0.01	-0.017	-0.004	-0.01	t	109241	GRTGPAGA	Rattus norvegicus
1	1.00	1	1	0.05	0.053	0.045	0.057	0.051	t	119749	ALQEACEAYLVGLF	Mus musculus

1	1.00	1	1	0	0.004	-0.005	0.003	0	t	4024	AQPGYPWPLYGNEG	Homo sapiens
1	1.00	1	1	0.06	0.058	0.057	0.059	0.06	t	61727	STLLPPELSETPNAT	Mus musculus
1	1.00	1	1	0.06	0.06	0.053	0.06	0.058	t	121775	RAPVSQAAKFDPSTK	Homo sapiens
1	1.00	1	1	0.02	0.02	0.01	0.023	0.02	t	52010	QQEGAQQPAPATA	Homo sapiens
1	1.00	1	1	0.02	0.023	0.016	0.02	0.02	t	102818	SPYTDRYKF	Mus musculus
1	1.00	1	1	-0.03	-0.026	-0.045	-0.027	-0.028	t	136080	RAGTDEGFG	Homo sapiens
1	1.00	1	1	0.04	0.042	0.038	0.047	0.04	t	125201	PPQTEHIEL	Homo sapiens
1	1.00	1	1	0.03	0.03	0.03	0.026	0.03	t	131365	ATLVCLI	Homo sapiens
1	1.00	1	1	0.02	0.02	0.01	0.02	0.019	t	10496	DTTIP	Mus musculus
1	1.00	1	1	0	0.002	-0.016	0.001	0.002	t	27379	ILTGGKSKQTEGLLLL	Homo sapiens
1	1.00	1	1	-0.01	-0.006	-0.01	-0.003	-0.01	t	107659	VKFNWYV	Homo sapiens
1	1.00	1	1	0.01	0.006	0.001	0.009	0.01	t	800	ADYHRVMY	Oryctolagus cuniculus
1	1.00	1	1	0.03	0.028	0.023	0.033	0.03	t	125565	STDDYASFSRAL	Mus musculus
1	1.00	1	1	0.04	0.037	0.035	0.04	0.04	t	47096	PDFYGDPLE	Mus musculus
1	1.00	1	1	0.03	0.042	0.032	0.03	0.027	t	4702	ASQKIQLV	Pan troglodytes

1	1.00	1	1	0.05	0.05	0.048	0.05	0.05	t	10320	DTDSGYD VPDY	Mus musculus
1	1.00	1	1	-0.01	-0.012	-0.026	-0.007	-0.008	t	38946	LQPGKTS	Mus musculus
1	1.00	1	1	0.01	0.014	0.01	0.017	0.007	t	122892	RSILPYGDS MDRIE	Homo sapiens
1	1.00	1	1	0.03	0.028	0.022	0.033	0.032	t	111661	QAEP	Mus musculus
1	1.00	1	1	0.06	0.06	0.063	0.066	0.06	t	142225	LNDSSEEE DEID	Homo sapiens
1	1.00	1	1	0	-0.002	0.001	-0.001	-0.001	t	56788	SAFVFPTK D	Mus musculus
1	1.00	1	1	0	0	-0.002	0	-0.003	t	57648	SETLKS	Mus musculus
1	1.00	1	1	0.02	0.02	0.009	0.02	0.02	t	49703	PTLLGEAT	Oryctolagus cuniculus
1	1.00	1	1	0.02	0.018	0.013	0.02	0.019	t	95047	VEVPGSQ HIDSQ	Oryctolagus cuniculus
1	1.00	1	1	0.02	0.018	0.013	0.02	0.019	t	95047	VEVPGSQ HIDSQ	Oryctolagus cuniculus
1	1.00	1	1	0	0	-0.005	-0.002	-0.002	t	100085	GSWCVPK PGVSD	Homo sapiens
1	1.00	1	1	0	-0.002	0.003	-0.001	-0.002	t	122138	LKKDV	Mus musculus
1	1.00	1	1	0.01	0.01	0.01	0.01	0.01	t	127906	YVKLFPNS LD	Homo sapiens
1	1.00	1	1	0.01	0.014	0.002	0.01	0.009	t	140594	RELVLAGTI	Cavia porcellus

1	1.00	1	1	0	-0.002	-0.017	-0.007	0	t	67606	VAGLQNDPT	Mus musculus
1	1.00	1	1	0.01	0.006	0.001	0.009	0.01	t	62907	TALDTSNK	Oryctolagus cuniculus
1	1.00	1	1	0.04	0.042	0.033	0.043	0.04	t	62458	SWGSPKVTKD	Homo sapiens
1	1.00	1	1	0.02	0.018	0.02	0.023	0.022	t	49295	PSAPPL	Homo sapiens
1	1.00	1	1	0.04	0.04	0.033	0.04	0.041	t	78276	TEVPVEKT	Homo sapiens
1	1.00	1	1	-0.02	-0.016	-0.02	-0.013	-0.02	t	121875	SRERSKDK	Homo sapiens
1	1.00	1	1	0.04	0.04	0.045	0.04	0.037	t	28250	IQWDGYARQ	Mus musculus
1	1.00	1	1	0.01	0.006	0.001	0.013	0.012	t	70039	VMYASSGIGP	Oryctolagus cuniculus
1	1.00	1	1	0.01	0.008	-0.002	0.013	0.011	t	107126	GAPGTPGPQG	Mus musculus
1	1.00	1	1	0	0.002	0	0.007	-0.001	t	119669	VSWNSGAL	Homo sapiens
1	1.00	1	1	0.02	0.02	0.02	0.019	0.02	t	35994	LFTGHL	Homo sapiens
1	1.00	1	1	-0.03	-0.038	-0.03	-0.03	-0.029	t	110126	YIK	Mus musculus
1	1.00	1	1	-0.03	-0.027	-0.043	-0.03	-0.031	t	65779	TPTAWKPN	Mus musculus
1	1.00	1	1	0.07	0.069	0.069	0.071	0.07	t	66316	TSLSVS	Homo sapiens
1	1.00	1	1	-0.02	-0.018	-0.027	-0.013	-0.019	t	144600	CQREFALKHL	Homo sapiens

1	1.00	1	1	0.03	0.032	0.029	0.027	0.028	t	75761	YSIEF	Homo sapiens
1	1.00	1	1	0.07	0.072	0.065	0.071	0.07	t	19057	GDN	Homo sapiens
1	1.00	1	1	-0.01	-0.006	-0.017	-0.007	-0.011	t	115631	NVRFSDEG GFTC	Homo sapiens
1	1.00	1	1	0.02	0.022	0.011	0.02	0.019	t	110733	VGENVPD DLYIKGSG	Homo sapiens
1	1.00	1	1	0.02	0.02	0.017	0.02	0.02	t	11882	EESPLGFS PE	Mus musculu s
1	1.00	1	1	0.01	0.012	-0.002	0.013	0.01	t	80018	RQTDR	Homo sapiens
1	1.00	1	1	0.01	0.012	-0.002	0.013	0.01	t	80018	RQTDR	Homo sapiens
1	1.00	1	1	-0.01	-0.007	-0.014	-0.01	-0.013	t	50177	PYTSTLLP	Mus musculu s
1	1.00	1	1	-0.01	-0.01	-0.01	-0.007	-0.01	t	25094	HVSPTHYV P	Homo sapiens
1	1.00	1	1	0.01	0.012	0.01	0.013	0.007	t	47045	PCSICSNN PTCWAICK	Homo sapiens
1	1.00	1	1	0.01	0.014	0.025	0.017	0.01	t	103843	DF	Homo sapiens
1	1.00	1	1	0.03	0.028	0.014	0.027	0.031	t	915	AEGDDPA KA	Mus musculu s
1	1.00	1	1	0	0.004	0	0.007	-0.002	t	130289	VSAAPGSA APAAGSA	Homo sapiens

1	1.00	1	1	-0.01	-0.014	-0.025	-0.011	-0.01	t	48912	PPSDIKGK YVKEVEVK	Oryctolagus cuniculus
1	1.00	1	1	0.03	0.033	0.033	0.03	0.029	t	49878	PVGTPE	Mus musculus
1	1.00	1	1	0.03	0.026	0.028	0.029	0.028	t	9819	DPYSPSQD PDRRDPY	Oryctolagus cuniculus
1	1.00	1	1	0.06	0.06	0.056	0.06	0.06	t	144925	NETIYNTTL K	Homo sapiens
1	1.00	1	1	-0.01	-0.013	-0.013	-0.01	-0.012	t	66668	TTKAPTQA AK	Oryctolagus cuniculus
1	1.00	1	1	0.01	0.014	-0.006	0.009	0.011	t	99057	WQIPEQS QCQAI	Homo sapiens
1	1.00	1	1	-0.01	-0.014	-0.015	-0.01	-0.012	t	100032	FEPNTVKA HAAY	Oryctolagus cuniculus
1	1.00	1	1	0.03	0.029	0.017	0.027	0.03	t	25525	ICSISPSQG CQF	Homo sapiens
1	1.00	1	1	0.03	0.034	0.017	0.027	0.03	t	25525	ICSISPSQG CQF	Homo sapiens
1	1.00	1	1	0.03	0.032	0.017	0.027	0.03	t	25525	ICSISPSQG CQF	Homo sapiens
1	1.00	1	1	0.04	0.036	0.04	0.033	0.037	t	122872	PLTLEL	Oryctolagus cuniculus
1	1.00	1	1	0.02	0.018	0.02	0.023	0.022	t	49295	PSAPPL	Homo sapiens
1	1.00	1	1	0.02	0.018	0.02	0.023	0.022	t	49295	PSAPPL	Homo sapiens

1	1.00	1	1	0	0	-0.001	0.011	0	t	30661	KEVPALTAVETGAT	Homo sapiens
1	1.00	1	1	0.05	0.048	0.043	0.049	0.05	t	96286	FDVTTL	Mus musculus
1	1.00	1	1	0.05	0.048	0.043	0.049	0.05	t	96286	FDVTTL	Mus musculus
1	1.00	1	1	0.04	0.038	0.028	0.043	0.042	t	95416	GLQNDP	Mus musculus
1	1.00	1	1	0.02	0.022	0.013	0.023	0.019	t	144682	HLVGSALSDP	Homo sapiens
1	1.00	1	1	0.01	0.01	0.01	0.01	0.008	t	70387	VPNLRGDLQV	Cavia porcellus
1	1.00	1	1	0.03	0.03	0.018	0.03	0.03	t	72544	WHLKSIGQLVIE	Mus musculus
1	1.00	1	1	0.03	0.027	0.018	0.03	0.03	t	72544	WHLKSIGQLVIE	Oryctolagus cuniculus
1	1.00	1	1	0.03	0.032	0.023	0.033	0.03	t	62458	SWGSPKVTKD	Homo sapiens
1	1.00	1	1	-0.02	-0.02	-0.023	-0.017	-0.02	t	50016	PVWARPDY	Homo sapiens
1	1.00	1	1	0.01	0.01	-0.004	0.01	0.01	t	102943	YGCYCGLGG	Mus musculus
1	1.00	1	1	-0.03	-0.032	-0.046	-0.029	-0.029	t	23455	GYSDRVRSITLGNS	Homo sapiens
1	1.00	1	1	0.08	0.08	0.08	0.08	0.078	t	135570	LFDASDSSS	Oryctolagus cuniculus
1	1.00	1	1	0	0	-0.012	0	0	t	1477	AGDR	Mus musculus

1	1.00	1	1	0.04	0.038	0.03	0.039	0.04	t	9669	DPLLGNQS	Anas platyrhy nchos
1	1.00	1	1	0	0.004	0	0.001	-0.003	t	134694	ISPFSCLK	MD
1	1.00	1	1	0.03	0.034	0.025	0.037	0.03	t	122956	YYVFFEEQ EDEIIGFG	Homo sapiens
1	1.00	1	1	0.04	0.043	0.039	0.04	0.039	t	122113	IIASTEKLP	Mus musculu s
1	1.00	1	1	-0.01	-0.01	-0.02	-0.01	-0.011	t	112897	AATGAAT AAA	Mus musculu s
1	1.00	1	1	-0.01	-0.007	-0.019	-0.007	-0.01	t	66101	TRSQSGLT FA	Capra hircus
1	1.00	1	1	0.02	0.025	0.005	0.021	0.022	t	97069	VQPQVNK EIR	Homo sapiens(human)
1	1.00	1	1	0.02	0.018	0.012	0.023	0.022	t	111176	CTSICKYPD YIKMVS	Mus musculu s
1	1.00	1	1	-0.01	-0.007	-0.017	-0.01	-0.01	t	63832	TGGAGYFY	Equus caballus
1	1.00	1	1	0.04	0.043	0.031	0.04	0.04	t	99934	DFLSTEEKE F	Mus musculu s
1	1.00	1	1	0.01	0.012	0.005	0.011	0.008	t	30697	KFAENVG	Homo sapiens
1	1.00	1	1	0.01	0.012	0.004	0.011	0.01	t	57215	SDIEKKVN	Homo sapiens
1	1.00	1	1	-0.01	-0.008	-0.016	-0.003	-0.009	t	144823	YWEGSGI GAI	Homo sapiens

1	1.00	1	1	0.01	0.009	0	0.01	0.009	t	3863	APTIEAITRPIQVA	Anas platyrhynchos
1	1.00	1	1	0.02	0.024	0.02	0.027	0.02	t	123618	PPPGLAPG	Homo sapiens
1	1.00	1	1	0.01	0.013	0.01	0.01	0.009	t	137401	FEDVW	Mus musculus
1	1.00	1	1	0.06	0.062	0.053	0.06	0.058	t	142486	YNQYTEEEK	Homo sapiens
1	1.00	1	1	0.02	0.022	0.02	0.021	0.019	t	58129	SGLTSLF	Homo sapiens
1	1.00	1	1	0	0.002	-0.005	0.003	0	t	159063	RKAADTFNFKTFFHT	Homo sapiens
1	1.00	1	1	0.03	0.025	0.02	0.03	0.03	t	127786	QEPGNPSEFEAD	Trichosurus vulpecula
1	1.00	1	1	0.02	0.018	0.015	0.023	0.02	t	6067	CCNLEPEARKVISS	Homo sapiens
1	1.00	1	1	0.03	0.034	0.034	0.037	0.03	t	122932	VCTPKSDEFNPCEDIMGYK	Homo sapiens
1	1.00	1	1	0	0.002	0	0	-0.005	t	105206	GAIIGLMVGGVV	Homo sapiens
1	1.00	1	1	0.03	0.022	0.019	0.03	0.03	t	53011	QYNQLSTGLQEA	Mus musculus
1	1.00	1	1	0.01	0.012	0.003	0.017	0.01	t	156799	VYATRSSAVRLRSSV	Homo sapiens
1	1.00	1	1	0.02	0.022	0.008	0.021	0.02	t	142382	AEDAAEEAKEAAKK	Homo sapiens
1	1.00	1	1	0	0.002	0	0.007	0	t	118535	NGNLQLRNKEVT	Homo sapiens

1	1.00	1	1	0	-0.002	-0.008	-0.001	-0.001	t	21883	GQHPAKSM	Anas platyrhynchos
1	1.00	1	1	0	0.004	-0.005	0.007	0	t	122956	YYVFFEEQEDEIIGFG	Homo sapiens
1	1.00	1	1	0	0.002	-0.005	0.007	0	t	106634	LVGPRGERGFP	Homo sapiens
1	1.00	1	1	0.02	0.022	0.013	0.027	0.021	t	122328	EEYHLYKDGHLPISEFALLV	Homo sapiens
1	1.00	1	1	0	0.002	0	0.007	0	t	118536	NITDHGSLIYSFSECAF	Homo sapiens
1	1.00	1	1	0.04	0.042	0.034	0.041	0.039	t	133540	DALVAKAQEVS	Homo sapiens
1	1.00	1	1	0.05	0.046	0.048	0.053	0.051	t	99737	TVKVGD	Oryctolagus cuniculus
1	1.00	1	1	0	-0.003	-0.01	0.001	0.002	t	81048	DIKLTITGPG	Homo sapiens
1	1.00	1	1	-0.01	-0.01	-0.02	0	-0.008	t	52184	QRAKEALDKYELENH	Homo sapiens
1	1.00	1	1	0.02	0.022	0.009	0.021	0.02	t	68272	VELKIKIASGFGPLI	Homo sapiens
1	1.00	1	1	0.01	0.009	0.003	0.01	0.008	t	56768	SAEYEKSKVNKYLKTIMPF	Equus caballus
1	1.00	1	1	0.01	0.008	-0.004	0.007	0.01	t	49450	PSNQDSFQWQ	Mus musculus
1	1.00	1	1	-0.05	-0.05	-0.057	-0.053	-0.05	t	46987	PAVFK	Homo sapiens

1	1.00	1	1	0.02	0.019	0.023	0.021	0.02	t	25694	IDTTIM	Homo sapiens
1	1.00	1	1	0.01	0.013	-0.002	0.016	0.012	t	125209	QFQIHGPR	Mus musculus
1	1.00	1	1	0.02	0.018	0.02	0.02	0.02	t	117344	PPGMRPP	Mus musculus
1	1.00	1	1	0.02	0.02	0.02	0.02	0.02	t	117344	PPGMRPP	Homo sapiens
1	1.00	1	1	0.01	0.012	-0.002	0.011	0.01	t	142391	DAANIAHQKWLKAT	Homo sapiens
1	1.00	1	1	0	0	0	0	-0.002	t	74415	YIWPRNDYDG	Mus musculus
1	1.00	1	1	-0.01	-0.01	-0.01	-0.01	-0.011	t	131314	TFLTVTPN	Homo sapiens
1	1.00	1	1	0.05	0.054	0.045	0.053	0.048	t	118133	SESQTLDKVYQM	Homo sapiens
1	1.00	1	1	0.02	0.016	0.011	0.017	0.018	t	79622	NYARTGGQVTAG	Oryctolagus cuniculus
1	1.00	1	1	0.01	0.008	-0.004	0.009	0.01	t	133546	DSARAAY	Mus musculus
1	1.00	1	1	0.02	0.024	0.027	0.021	0.014	t	5249	AVAEAEGG	Homo sapiens
1	1.00	1	1	-0.02	-0.02	-0.027	-0.02	-0.02	t	121823	RTVLVSMNY	Mus musculus
1	1.00	1	1	0.03	0.029	0.036	0.031	0.029	t	8259	DFELIK	Homo sapiens
1	1.00	1	1	0.03	0.03	0.036	0.031	0.029	t	8259	DFELIK	Homo sapiens
1	1.00	1	1	0	-0.003	-0.002	0	0	t	36834	LKEKYKDK	Oryctolagus cuniculus

1	1.00	1	1	0.07	0.07	0.07	0.07	0.069	t	107107	EAIQP	Rattus norvegicus
1	1.00	1	1	0	0.002	-0.002	0.003	0	t	131580	PDRFSGS	Homo sapiens
1	1.00	1	1	0.03	0.022	0.022	0.029	0.03	t	110964	PNLKLNNGTDYK	Macaca
1	1.00	1	1	0.03	0.03	0.025	0.036	0.029	t	13986	EREVSVPA	Homo sapiens
1	1.00	1	1	-0.04	-0.04	-0.04	-0.036	-0.041	t	119607	KTHTCPPC	Homo sapiens
1	1.00	1	1	-0.01	-0.012	-0.021	-0.011	-0.01	t	58402	SHRELWAK	Mus musculus
1	1.00	1	1	-0.01	-0.024	-0.021	-0.011	-0.01	t	58402	SHRELWAK	Macaca mulatta
1	1.00	1	1	0	0	-0.012	0.01	0.003	t	106738	QKRAAYDQYGHAAFE	Homo sapiens
1	1.00	1	1	0	0.008	0	0	-0.002	t	156946	QLLYGS	Gallus gallus
1	1.00	1	1	-0.01	-0.016	-0.008	-0.01	-0.013	t	5109	ATPYHPPATP	Oryctolagus cuniculus
1	1.00	1	1	0.04	0.04	0.04	0.044	0.04	t	135959	LTRAYAKDVKFG	Homo sapiens
1	1.00	1	1	-0.03	-0.028	-0.032	-0.029	-0.032	t	46713	NYTIFKIRM	Homo sapiens
1	1.00	1	1	0	0	-0.007	0	-0.002	t	133664	PPPPPPPPPP	Mus musculus
1	1.00	1	1	0.02	0.024	0.007	0.017	0.021	t	75541	YQQYPQQ	Homo sapiens
1	1.00	1	1	-0.02	-0.021	-0.024	-0.019	-0.022	t	30294	KEAIVTK	Bos taurus

1	1.00	1	1	0	0.004	-0.005	0.003	0	t	36463	LIANCASILCKCYTT	Homo sapiens
1	1.00	1	1	0.01	0.006	0.01	0.013	0.01	t	105937	LPNIEADV	Oryctolagus cuniculus
1	1.00	1	1	0.01	0.01	0.003	0.01	0.01	t	153425	MQIFVKTLTGKTITL	Mus musculus
1	1.00	1	1	0.03	0.028	0.017	0.029	0.03	t	63089	TCPDKKSTA	Mus musculus
1	1.00	1	1	0.02	0.02	0.01	0.02	0.02	t	63238	TDSQDS	Oryctolagus cuniculus
1	1.00	1	1	0.01	0.008	0.008	0.009	0.01	t	97167	AANYTTAV	Mus musculus
1	1.00	1	1	0.01	0.008	0.008	0.009	0.01	t	97167	AANYTTAV	Mus musculus
1	1.00	1	1	-0.06	-0.058	-0.067	-0.053	-0.061	t	107828	ERGLKGHRGFT	Homo sapiens
1	1.00	1	1	0	0	0	0.006	-0.002	t	868	AEEAFDLWNECAKACV	Homo sapiens
1	1.00	1	1	0.03	0.03	0.021	0.03	0.03	t	8646	DIGDIVRGKDLY	Homo sapiens
1	1.00	1	1	-0.01	-0.008	-0.022	-0.009	-0.01	t	12422	EIDLTNKNAAKLRGD	Homo sapiens
1	1.00	1	1	0.01	0.013	0.006	0.01	0.01	t	29103	ITTATGY	Felis catus
1	1.00	1	1	-0.01	-0.014	-0.02	-0.007	-0.01	t	11313	EDERTLTK	Oryctolagus cuniculus
1	1.00	1	1	0	-0.002	0	0.007	0	t	123570	PATKKAVQ	Homo sapiens

1	1.00	1	1	0.03	0.032	0.03	0.037	0.03	t	107315	DGKEYKC	Homo sapiens
1	1.00	1	1	-0.02	-0.02	-0.019	-0.016	-0.021	t	55577	RRGPRLGV	Homo sapiens
1	1.00	1	1	0.03	0.033	0.025	0.037	0.031	t	117543	KFKGKATL TSDK	Mus musculu s
1	1.00	1	1	0.02	0.017	0.01	0.021	0.021	t	88952	SNYTPKNK IE	Homo sapiens
1	1.00	1	1	-0.01	-0.012	-0.015	-0.007	-0.01	t	38836	LQFGYTSR SM	Mus musculu s
1	1.00	1	1	0.03	0.038	0.03	0.03	0.027	t	136648	SPLNNFYK TQLR	Gallus gallus
1	1.00	1	1	0.01	0.01	0.002	0.01	0.01	t	43212	NALSFTNK	Mus musculu s
1	1.00	1	1	0.02	0.022	0.02	0.027	0.019	t	119660	VEWESNG Q	Homo sapiens
1	1.00	1	1	0.01	0.014	0.015	0.017	0.009	t	115608	GRTELLKD AIGE	Homo sapiens
1	1.00	1	1	0.01	0.014	0.015	0.017	0.009	t	115608	GRTELLKD AIGE	Homo sapiens
1	1.00	1	1	0.02	0.016	0.02	0.023	0.02	t	105850	GTAPFEIH	Oryctola gus cuniculu s
1	1.00	1	1	0	0.004	-0.016	0.003	0.001	t	119379	VGNETQG VTAEITPQ	Homo sapiens
1	1.00	1	1	0.02	0.02	0.017	0.02	0.019	t	46786	PAEVTQLK GLS	Mus musculu s
1	1.00	1	1	0.01	0.012	0.006	0.017	0.01	t	141916	HSLRYNLT VLSW	Homo sapiens

1	1.00	1	1	0.01	0.01	0.01	0.019	0.01	t	122116	IPDSRLK	Homo sapiens
1	1.00	1	1	0.01	0.01	0.01	0.01	0.007	t	134200	PVVNSLDPPLLTRY	Homo sapiens
1	1.00	1	1	0.02	0.02	0.008	0.02	0.02	t	81782	EQVEYPLKPI	Oryctolagus cuniculus
1	1.00	1	1	0	0.006	-0.014	0.001	0	t	120928	RRSNVIWPVSKDQLLR	Equus caballus
1	1.00	1	1	0.03	0.034	0.03	0.031	0.027	t	134696	ITLYLTEK	MD
1	1.00	1	1	0.01	0.007	0.005	0.01	0.01	t	52476	QTASGLVSMF	Oryctolagus cuniculus
1	1.00	1	1	0.01	0.013	0.011	0.01	0.008	t	30863	KFTEPVKDIM	Mus musculus
1	1.00	1	1	-0.01	-0.01	-0.027	-0.014	-0.01	t	111025	SKIYDTKTGTDR	Homo sapiens
1	1.00	1	1	-0.01	-0.01	-0.027	-0.014	-0.01	t	111025	SKIYDTKTGTDR	Homo sapiens
1	1.00	1	1	0	-0.002	0	-0.001	-0.004	t	10124	DSGCVVSWK	Mus musculus
1	1.00	1	1	0.05	0.053	0.039	0.05	0.05	t	111309	GDTPTLHEYMLDLQ	Mus musculus
1	1.00	1	1	0.03	0.03	0.021	0.03	0.03	t	8475	DGQPAGDRADGQPAGDRA	Mus musculus
1	1.00	1	1	0.02	0.02	0.02	0.02	0.02	t	78400	GLLNDY	Mus musculus
1	1.00	1	1	-0.01	-0.006	-0.008	-0.003	-0.011	t	119647	SRWQQGNV	Homo sapiens

1	1.00	1	1	0.02	0.02	0.02	0.029	0.02	t	133795	DFTYDQV AFNGLPQF	Homo sapiens
1	1.00	1	1	0.01	0.01	0.009	0.01	0.01	t	49825	PVADLP	Homo sapiens
1	1.00	1	1	-0.01	-0.008	-0.021	-0.009	-0.009	t	66410	TSRVEHAV VYYVYSP	Homo sapiens
1	1.00	1	1	0	0.002	0.011	0.007	-0.003	t	69265	VKLSG	Homo sapiens
1	1.00	1	1	-0.03	-0.028	-0.04	-0.029	-0.03	t	36890	LKIKIASGF GPLITH	Homo sapiens
1	1.00	1	1	0.02	0.02	0.02	0.029	0.021	t	107814	EGLHNHY	Homo sapiens
1	1.00	1	1	0	0.002	0	0.007	-0.001	t	108874	DPISGHVS LF	Homo sapiens
1	1.00	1	1	0.03	0.029	0.03	0.033	0.028	t	156965	SVQERYYA FWST	Bos taurus
1	1.00	1	1	0.03	0.026	0.03	0.033	0.028	t	156965	SVQERYYA FWST	Bos taurus
1	1.00	1	1	-0.01	-0.01	-0.024	-0.01	-0.01	t	63730	TFPQPQQ TYP	Mus musculu s
1	1.00	1	1	0.01	0.008	0.001	0.009	0.01	t	80508	ATAIFD	Mus musculu s
1	1.00	1	1	0.01	0.008	0.001	0.009	0.01	t	80508	ATAIFD	Mus musculu s
1	1.00	1	1	0.05	0.052	0.05	0.057	0.049	t	117613	QEKLQVAL GE	Homo sapiens
1	1.00	1	1	0.02	0.01	0.013	0.021	0.019	t	137891	HETHYGYA TLSY	Homo sapiens
1	1.00	1	1	0.02	0.022	0.013	0.021	0.019	t	137891	HETHYGYA TLSY	Homo sapiens
1	1.00	1	1	0.03	0.026	0.023	0.029	0.028	t	88388	SAWNSDS EKPFDDHL	Oryctola gus cuniculu s

1	1.00	1	1	0.03	0.032	0.023	0.029	0.028	t	88388	SAWNSDSEKPFDDHL	MD
1	1.00	1	1	-0.03	-0.028	-0.032	-0.023	-0.03	t	127643	ERPLELRVRVLDI	Homo sapiens
1	1.00	1	1	0.02	0.013	0.013	0.027	0.022	t	156970	VERHQ	Homo sapiens
1	1.00	1	1	0.08	0.08	0.067	0.08	0.082	t	141957	MEKIAEEDI	Mus musculus
1	1.00	1	1	-0.04	-0.04	-0.052	-0.036	-0.038	t	96487	KPTAPTKPTY	Homo sapiens(human)
1	1.00	1	1	0.03	0.029	0.02	0.03	0.03	t	51943	QPQWTPEE	Anas platyrhynchos
1	1.00	1	1	0.02	0.024	0.014	0.021	0.019	t	53240	RAVVAPDKEVLYE	Homo sapiens
1	1.00	1	1	0.06	0.059	0.053	0.06	0.062	t	14576	ETTTIPPS	Anas platyrhynchos
1	1.00	1	1	0.03	0.026	0.03	0.033	0.03	t	105715	DLESIKGT	Oryctolagus cuniculus
1	1.00	1	1	0.02	0.024	0.02	0.027	0.019	t	131700	TGVTTQGVKSLLTSMYVKEF	Homo sapiens
1	1.00	1	1	-0.02	-0.016	-0.039	-0.023	-0.018	t	47350	PELPYPQP	Homo sapiens
1	1.00	1	1	-0.02	-0.016	-0.028	-0.019	-0.02	t	53112	RAHYNIVTF	Homo sapiens
1	1.00	1	1	0.04	0.038	0.03	0.037	0.04	t	64062	THPLYQSE	Anas platyrhynchos
1	1.00	1	1	0.01	0.012	-0.007	0.01	0.013	t	110819	DTKTGTDRETTS	Homo sapiens
1	1.00	1	1	-0.01	-0.014	-0.019	-0.011	-0.011	t	29803	KAFRELADAL	Oryctolagus cuniculus

1	1.00	1	1	-0.04	-0.04	-0.049	-0.04	-0.039	t	118764	CYGSFARP TN	Mus musculu s
1	1.00	1	1	-0.04	-0.036	-0.056	-0.043	-0.04	t	43791	NEWCRC	Homo sapiens
1	1.00	1	1	-0.02	-0.016	-0.016	-0.013	-0.021	t	144700	KEWAKRA ALP	Homo sapiens
1	1.00	1	1	0.08	0.082	0.078	0.079	0.078	t	96215	EEEEAEDK ED	Homo sapiens
1	1.00	1	1	0	-0.006	-0.016	0	0.001	t	41169	MAVPAFS DIAKSAND LLNKD	Oryctola gus cuniculu s
1	1.00	1	1	-0.01	-0.012	-0.01	-0.009	-0.013	t	130461	CWNYCLP	Mus musculu s
1	1.00	1	1	0	0	-0.013	0	0	t	94517	FNENMAY	Oryctola gus cuniculu s
1	1.00	1	1	0	0	0	0.011	0	t	18588	GAGGGAG GAGAGGG AGGAGA	Homo sapiens
1	1.00	1	1	0.02	0.02	0.011	0.023	0.02	t	2610	ALHQSML NSQAIDNL	Homo sapiens
1	1.00	1	1	-0.03	-0.03	-0.042	-0.03	-0.031	t	30062	KCKNDGW AGYWR	Oryctola gus cuniculu s
1	1.00	1	1	-0.03	-0.027	-0.042	-0.03	-0.031	t	30062	KCKNDGW AGYWR	Mus musculu s
1	1.00	1	1	0.02	0.024	0.02	0.027	0.019	t	116681	CFEGQEKA HNQDLGL	Homo sapiens
1	1.00	1	1	0	0.004	-0.014	0.001	0	t	21353	GMTFRAQ EGAFLTG	Homo sapiens

1	1.00	1	1	-0.01	-0.01	-0.011	0	-0.008	t	106235	AGFNVPW	Homo sapiens
1	1.00	1	1	0.03	0.03	0.03	0.03	0.03	t	78400	GLLNDY	Mus musculu s
1	1.00	1	1	-0.01	-0.01	-0.008	0.001	-0.01	t	118432	ISIGYHYPP ICLGRA	Homo sapiens
1	1.00	1	1	-0.01	-0.006	-0.008	-0.009	-0.011	t	74217	YIGGPL	Homo sapiens
1	1.00	1	1	0.04	0.03	0.033	0.041	0.039	t	137867	GEIKFEAV EGAV	Homo sapiens
1	1.00	1	1	0.04	0.042	0.033	0.041	0.039	t	137867	GEIKFEAV EGAV	Homo sapiens
1	1.00	1	1	0.03	0.032	0.03	0.037	0.03	t	107451	ISKAKGQ	Homo sapiens
1	1.00	1	1	0	0.002	0.002	0.003	-0.001	t	46951	PASSHTILV	Homo sapiens
1	1.00	1	1	0	0.002	0.002	0.003	-0.001	t	46951	PASSHTILV	Homo sapiens
1	1.00	1	1	0.05	0.048	0.039	0.053	0.051	t	70502	VPVPDFSQ	Mus musculu s
1	1.00	1	1	0.01	0.012	0.015	0.017	0.01	t	112814	PEQPPIH	Homo sapiens
1	1.00	1	1	-0.03	-0.031	-0.039	-0.029	-0.03	t	58346	SHDRHFLN N	Homo sapiens
1	1.00	1	1	0.05	0.05	0.037	0.05	0.052	t	87834	REGVEEEK TSEE	Oryctola gus cuniculu s
1	1.00	1	1	0	0.002	-0.018	-0.001	0.001	t	98697	AMCNVYI PPYCT	Homo sapiens
1	1.00	1	1	0.03	0.032	0.023	0.033	0.031	t	142474	SSTTGVA M	Homo sapiens

1	1.00	1	1	-0.01	-0.018	-0.014	-0.01	-0.011	t	29160	ITYGACPR YVK	Mus musculu s
1	1.00	1	1	-0.01	-0.018	-0.014	-0.01	-0.011	t	29160	ITYGACPR YVK	Mus musculu s
1	1.00	1	1	-0.01	-0.018	-0.014	-0.01	-0.011	t	29160	ITYGACPR YVK	Mus musculu s
1	1.00	1	1	-0.07	-0.068	-0.074	-0.063	-0.072	t	127779	PSRRRRRR KATLAPA	Homo sapiens
1	1.00	1	1	-0.01	-0.012	-0.022	-0.013	-0.01	t	75445	YPYLKDGK	Mus musculu s
1	1.00	1	1	0.01	0.008	0.008	0.009	0.006	t	74794	YLLPAIVHI	Mus musculu s
1	1.00	1	1	0.01	0.014	0.007	0.011	0.007	t	103550	RIFYKDAF AKHQELF	Homo sapiens
1	1.00	1	1	0	0.004	0.002	0.007	0	t	119641	SEHETRLV AKLFKDYS	Homo sapiens
1	1.00	1	1	0.02	0.02	0.006	0.02	0.022	t	109349	IPVDLL	Mus musculu s
1	1.00	1	1	0.01	0.012	0.003	0.013	0.01	t	986	AEIERMVR EAAKYEA	Homo sapiens
1	1.00	1	1	0.04	0.04	0.04	0.043	0.04	t	149116	SYAAVAS	Sus scrofa
1	1.00	1	1	0.04	0.036	0.028	0.039	0.04	t	65	AAAVLNLT	Oryctola gus cuniculu s
1	1.00	1	1	0.03	0.032	0.017	0.027	0.03	t	115060	GYLEQL	Homo sapiens
1	1.00	1	1	0.02	0.02	0.019	0.02	0.02	t	32494	KNFNFEVE	Homo sapiens
1	1.00	1	1	-0.02	-0.018	-0.02	-0.013	-0.02	t	131358	APSVTLF	Homo sapiens

1	1.00	1	1	0	-0.002	-0.006	-0.001	-0.002	t	70134	VNNKV	Mus musculus
1	1.00	1	1	0.02	0.022	0.018	0.02	0.02	t	71449	VTPLGPPAAEY	Homo sapiens
1	1.00	1	1	0.04	0.042	0.029	0.041	0.04	t	133687	SAEALLRLQA	Homo sapiens
1	1.00	1	1	0	-0.001	-0.012	0	0	t	64644	TKPTDPTGIE	Mus musculus
1	1.00	1	1	0.01	0.006	-0.003	0.009	0.01	t	43039	MYASSGIG	Oryctolagus cuniculus
1	1.00	1	1	0	0.004	-0.005	0.001	0	t	65428	TNSKGQ	Homo sapiens
1	1.00	1	1	-0.01	-0.011	-0.02	-0.01	-0.011	t	73074	WSGKFPTL	Anas platyrhynchos
1	1.00	1	1	0	0	0	0	-0.002	t	61394	SSTKNLIY	Mus musculus
1	1.00	1	1	0	0.002	0	0.006	-0.002	t	3274	ANETIYNTTLKYGDV	MD
1	1.00	1	1	0.02	0.02	0.003	0.02	0.022	t	120414	ATNLIKSGVA	Mus musculus
1	1.00	1	1	0.01	0.01	0.003	0.01	0.009	t	138258	VMHEALH	Homo sapiens
1	1.00	1	1	-0.05	-0.05	-0.05	-0.04	-0.05	t	98681	AEKTKIPAVF	Homo sapiens
1	1.00	1	1	0.02	0.022	0.004	0.017	0.02	t	74475	YKLNFYFDLLRAKL	Homo sapiens
1	1.00	1	1	0	0.002	0	0.007	0.002	t	107479	KFNWYVD	Homo sapiens
1	1.00	1	1	0.04	0.042	0.04	0.043	0.04	t	68017	VDLPQL	Homo sapiens

1	1.00	1	1	0.01	0.012	0.005	0.01	0.01	t	49745	PTPQEIPQ	Anas platyrhynchos
1	1.00	1	1	-0.02	-0.018	-0.028	-0.019	-0.02	t	60626	SRCEQIRE	Homo sapiens
1	1.00	1	1	0.01	0.012	0.015	0.017	0.007	t	57618	SERVQLLHSQNTSLINQK	Homo sapiens
1	1.00	1	1	-0.02	-0.018	-0.027	-0.013	-0.019	t	144600	CQREFALKHL	Homo sapiens
1	1.00	1	1	0.03	0.03	0.03	0.03	0.028	t	79528	CPSKKPYEEVTC	Mus musculus
1	1.00	1	1	0.03	0.03	0.03	0.03	0.028	t	79528	CPSKKPYEEVTC	Mus musculus
1	1.00	1	1	0.03	0.03	0.03	0.03	0.028	t	79528	CPSKKPYEEVTC	Mus musculus
1	1.00	1	1	-0.01	-0.01	-0.025	-0.01	-0.009	t	133533	CKCGKCNT	Mus musculus
1	1.00	1	1	-0.05	-0.05	-0.057	-0.053	-0.05	t	46987	PAVFK	Homo sapiens
1	1.00	1	1	0.03	0.027	0.03	0.03	0.028	t	95717	QLDTDGKGYE	Oryctolagus cuniculus
1	1.00	1	1	0.04	0.04	0.04	0.049	0.04	t	119506	PIVET	Homo sapiens
1	1.00	1	1	0	0.002	0	0	0	t	29126	ITTYV	Homo sapiens
1	1.00	1	1	0.01	0.012	0.003	0.017	0.01	t	133787	CQALVRMLAKKPGWK	Homo sapiens
1	1.00	1	1	-0.01	-0.01	-0.01	-0.001	-0.011	t	131293	AGRAREYV	Homo sapiens

1	1.00	1	1	-0.03	-0.032	-0.044	-0.031	-0.03	t	42576	MSIRRSMA	Mus musculus
1	1.00	1	1	0.05	0.049	0.039	0.049	0.05	t	69289	VKPGTPAQEI	Myodes glareolus
1	1.00	1	1	0.02	0.022	0.02	0.027	0.02	t	114196	CKPISGHNSLFQWYRQT	Homo sapiens
1	1.00	1	1	-0.02	-0.022	-0.019	-0.017	-0.02	t	73741	YEPPVVHG	Homo sapiens
1	1.00	1	1	-0.02	-0.018	-0.019	-0.017	-0.02	t	73741	YEPPVVHG	Homo sapiens
1	1.00	1	1	0.02	0.022	0.02	0.027	0.02	t	107758	AWNCCVKQTP	Homo sapiens
1	1.00	1	1	0.02	0.022	0.02	0.027	0.02	t	107758	AWNCCVKQTP	Homo sapiens
1	1.00	1	1	0.01	0.012	0.01	0.017	0.009	t	107878	GWVAMHPQGDL	Homo sapiens
1	1.00	1	1	0.03	0.03	0.03	0.03	0.027	t	22620	GTDGV	Homo sapiens
1	1.00	1	1	0.02	0.02	0.02	0.027	0.02	t	136003	TRKDINTVTTVA	Homo sapiens
1	1.00	1	1	-0.02	-0.023	-0.027	-0.02	-0.02	t	137425	KFP	Oryctolagus cuniculus
1	1.00	1	1	0.02	0.024	0.011	0.021	0.02	t	46867	PAN	MD
1	1.00	1	1	0	0	0.006	0	-0.006	t	15968	FGDSYI	Homo sapiens
1	1.00	1	1	0.02	0.022	0.018	0.021	0.02	t	55967	RSSSGCVT	Homo sapiens
1	1.00	1	1	0.04	0.043	0.029	0.04	0.04	t	43157	NAGTSL	Equus caballus

1	1.00	1	1	0.06	0.06	0.045	0.053	0.061	t	102880	VDELD	Mus musculus
1	1.00	1	1	0.02	0.022	0.016	0.023	0.02	t	76350	YVLLSEKKIS	Homo sapiens
1	1.00	1	1	-0.01	-0.014	-0.005	-0.007	-0.012	t	95979	VPQPPVSQFHIQ	Oryctolagus cuniculus
1	1.00	1	1	0.02	0.018	0.02	0.019	0.019	t	69883	VLYQEFDE	Mus musculus
1	1.00	1	1	0.01	0.008	0.01	0.01	0.011	t	112672	TAEFQMTFHLFI	Oryctolagus cuniculus
1	1.00	1	1	-0.02	-0.02	-0.02	-0.01	-0.02	t	114752	AYSIEFGTNI	Homo sapiens
1	1.00	1	1	0.04	0.042	0.026	0.037	0.04	t	62354	SVTEEVVEEEGPVDEE	Homo sapiens
1	1.00	1	1	0	0.002	0.005	0.003	-0.003	t	47033	PCNTKRTA	Homo sapiens
1	1.00	1	1	-0.01	-0.01	-0.02	-0.01	-0.01	t	112898	AAVKQSYAAT	Mus musculus
1	1.00	1	1	0.04	0.042	0.034	0.041	0.039	t	133540	DALVAKAQEVS	Homo sapiens
1	1.00	1	1	0.02	0.017	0.014	0.02	0.02	t	105954	MAKFTQFA	Oryctolagus cuniculus
1	1.00	1	1	0.06	0.06	0.05	0.06	0.06	t	34138	KVNIKPLEDK	Mus musculus
1	1.00	1	1	-0.01	-0.012	-0.017	-0.007	-0.01	t	10529	DTYRYI	Mus musculus
1	1.00	1	1	0.01	0.006	0.008	0.01	0.009	t	74008	YGGL	Mus musculus

1	1.00	1	1	0.01	0.013	0.008	0.01	0.009	t	74008	YGGL	Mus musculus
1	1.00	1	1	0.01	0.005	0.008	0.01	0.009	t	74008	YGGL	Mus musculus
1	1.00	1	1	0.01	0.006	-0.002	0.009	0.011	t	133683	RQRY	Oryctolagus cuniculus
1	1.00	1	1	0.02	0.026	0.017	0.021	0.019	t	20725	GKVVLPEN	MD
1	1.00	1	1	0.02	0.024	0.017	0.021	0.019	t	20725	GKVVLPEN	MD
1	1.00	1	1	0.02	0.024	0.017	0.021	0.019	t	20725	GKVVLPEN	MD
1	1.00	1	1	-0.01	-0.006	-0.017	-0.009	-0.011	t	138027	SADFTPGT	Homo sapiens
1	1.00	1	1	0.01	0.012	-0.002	0.011	0.01	t	142385	AKTAATNAEKKKTK	Homo sapiens
1	1.00	1	1	0.01	0.012	0.003	0.007	0.008	t	115700	CEKDERFFSD	Homo sapiens
1	1.00	1	1	0.01	0.014	0.003	0.013	0.012	t	111049	TVTLKATPNDGY	Homo sapiens
1	1.00	1	1	0	0	0	0.002	-0.001	t	157178	EASNCF	Homo sapiens
1	1.00	1	1	0.01	0.008	-0.006	0.003	0.01	t	98081	GSVQFVPIQN	Mus musculus
1	1.00	1	1	0	0.003	-0.017	0	0.001	t	45325	NPDPNPN	Mus musculus

1	1.00	1	1	0	-0.004	-0.005	0	-0.002	t	100538	SWNCDFSQTATL	Oryctolagus cuniculus
1	1.00	1	1	0.03	0.03	0.025	0.027	0.028	t	50901	QGLRGEEMEEMV	Homo sapiens
1	1.00	1	1	0.02	0.02	0.02	0.016	0.018	t	117695	YITTIAGVM	Homo sapiens
1	1.00	1	1	0	0	-0.018	0	0.001	t	56599	RYNLVFSGDSDRICSNRA	Mus musculus
1	1.00	1	1	0	0	0	0	-0.002	t	65555	TPEMDIPGQV	Mus musculus
1	1.00	1	1	0.02	0.018	0.025	0.019	0.02	t	104312	TNMWDF	Mus musculus
1	1.00	1	1	0.02	0.02	0.002	0.02	0.022	t	1187	AETHLFTG	Mus musculus
1	1.00	1	1	0.05	0.052	0.043	0.057	0.052	t	142448	NKYRGTAGNALMDGASQL	Homo sapiens
1	1.00	1	1	0	0.002	-0.017	-0.002	0	t	45009	NMNLVDWLR	Homo sapiens
1	1.00	1	1	0.02	0.02	0.006	0.02	0.022	t	109348	IPVDL	Mus musculus
1	1.00	1	1	0.01	0.01	0	0.008	0.01	t	63420	TEKFVKEQ	Homo sapiens
1	1.00	1	1	-0.02	-0.014	-0.03	-0.02	-0.021	t	66743	TTNRFAKY	Equus caballus
1	1.00	1	1	0.05	0.05	0.038	0.048	0.05	t	28907	ITGEYVQILDAHAAY	Homo sapiens

1	1.00	1	1	0.02	0.02	0.001	0.02	0.022	t	30289	KEAESVCAPIMSKIYQ	Homo sapiens
1	1.00	1	1	-0.01	-0.008	-0.01	-0.003	-0.01	t	118189	TYYIKEQKLGL	Homo sapiens
1	1.00	1	1	0.01	0.01	0.003	0.02	0.011	t	117383	TLHKAFKGSIFVVFDSIESA	Homo sapiens
1	1.00	1	1	0.03	0.027	0.027	0.03	0.029	t	74760	YLL	Oryctolagus cuniculus
1	1.00	1	1	0.05	0.05	0.041	0.054	0.05	t	10991	EAAKYEAEDKDQVRQ	Homo sapiens
1	1.00	1	1	0.01	0.01	0.005	0.01	0.012	t	133570	FLPKYALS	Mus musculus
1	1.00	1	1	0.05	0.051	0.049	0.053	0.049	t	99176	EDTLEEKMYACRDIG	Equus caballus
1	1.00	1	1	0.02	0.023	0.008	0.02	0.02	t	134794	SEATVAESDSF	Mus musculus
1	1.00	1	1	-0.02	-0.02	-0.024	-0.02	-0.02	t	60792	SRRYPFVKF	Homo sapiens
1	1.00	1	1	0	0	-0.005	0.003	-0.001	t	64093	THVTGGA	Homo sapiens
1	1.00	1	1	0	0.004	0	0.003	0	t	5380	AVILLMCAVHPTLV	Homo sapiens
1	1.00	1	1	0.01	0.01	-0.002	0.01	0.01	t	133537	CLTINTTVCA	Mus musculus
1	1.00	1	1	-0.05	-0.05	-0.057	-0.053	-0.05	t	46987	PAVFK	Homo sapiens
1	1.00	1	1	0.01	0.002	0.006	0.01	0.008	t	29159	ITYGACPKYVKQNTLK	Mus musculus
1	1.00	1	1	-0.02	-0.02	-0.016	-0.014	-0.022	t	33915	KTWGQYWQV	Homo sapiens

1	1.00	1	1	-0.02	-0.023	-0.028	-0.02	-0.02	t	68182	VEFPLDITAG	Oryctolagus cuniculus
1	1.00	1	1	-0.02	-0.02	-0.028	-0.02	-0.02	t	68182	VEFPLDITAG	Mus musculus
1	1.00	1	1	0.01	0.006	0.002	0.009	0.008	t	51463	QLPFPQQPQQ	Mus musculus
1	1.00	1	1	0.04	0.04	0.029	0.04	0.04	t	66707	TTLPKGFYAEGSRGG	Macaca cyclopis
1	1.00	1	1	0.04	0.04	0.029	0.04	0.04	t	66707	TTLPKGFYAEGSRGG	Mus musculus
1	1.00	1	1	0.01	0	0.003	0.011	0.009	t	137867	GEIKFEAVEGAV	Homo sapiens
1	1.00	1	1	0.03	0.033	0.023	0.03	0.031	t	138862	KFPED	Mus musculus
1	1.00	1	1	0.02	0.024	0.02	0.027	0.02	t	113091	MESALDQLKQFTTVV	Homo sapiens
1	1.00	1	1	0.03	0.03	0.02	0.03	0.032	t	22639	GTEKF	Mus musculus
1	1.00	1	1	0	0	-0.009	0	0	t	94553	IFD	Mus musculus
1	1.00	1	1	0	0.003	-0.01	0	0	t	48714	PNVDP	Mus musculus
1	1.00	1	1	0.05	0.05	0.05	0.05	0.05	t	15355	FCVQPE	Homo sapiens
1	1.00	1	1	0	0.002	-0.021	-0.001	0.002	t	153174	YTVDVKNKRTFLSP	Homo sapiens
1	1.00	1	1	0.03	0.028	0.018	0.033	0.031	t	107242	SQTTANPSCPEGT	Rattus norvegicus
1	1.00	1	1	0.04	0.04	0.029	0.04	0.04	t	94473	CNPACGPKYS	Oryctolagus cuniculus

1	1.00	1	1	0	0	-0.002	0.003	0.002	t	38654	LPQLGP	Homo sapiens
1	1.00	1	1	-0.01	-0.01	-0.012	-0.01	-0.014	t	159204	VYNDVEKWKSDYKYFP	Mus musculus
1	1.00	1	1	0.02	0.018	0.013	0.023	0.021	t	107166	GPPGPQG	Mus musculus
1	1.00	1	1	0.02	0.018	0.015	0.023	0.018	t	55048	RNQE	Mus musculus
1	1.00	1	1	0.03	0.027	0.023	0.03	0.028	t	122906	SRPSESNG	Oryctolagus cuniculus
1	1.00	1	1	0.04	0.044	0.033	0.047	0.041	t	104569	MASPGSGFWSFGSEDGS	Homo sapiens
1	1.00	1	1	-0.02	-0.018	-0.027	-0.017	-0.021	t	138807	RLQGISPKI	Homo sapiens
1	1.00	1	1	0.03	0.03	0.03	0.033	0.03	t	32683	KPDYEPPV	Homo sapiens
1	1.00	1	1	0	0.004	-0.005	0.001	0	t	58742	SIVTAPFQVK	Homo sapiens
1	1.00	1	1	0	0.008	-0.007	0	-0.002	t	125246	RRQPHVMS	Macaca fascicularis
1	1.00	1	1	0.03	0.032	0.028	0.037	0.03	t	126227	HNNAHYYVFFEEQED	Homo sapiens
1	1.00	1	1	0.04	0.04	0.04	0.05	0.04	t	114987	YANTTSEDGK	Homo sapiens
1	1.00	1	1	-0.01	-0.01	-0.026	-0.008	-0.007	t	98930	PFRPQQPYPQPQ	Homo sapiens
1	1.00	1	1	0.05	0.052	0.043	0.051	0.05	t	150964	AIDEITNKVNSVIEK	Homo sapiens
1	1.00	1	1	0.05	0.05	0.05	0.05	0.049	t	159282	EENW	Oryctolagus cuniculus
1	1.00	1	1	0.04	0.042	0.04	0.039	0.04	t	123441	LLEYLGEEY	Ovis aries

1	1.00	1	1	0.04	0.039	0.033	0.04	0.039	t	55248	RPPETTTI	Anas platyrhynchos
1	1.00	1	1	-0.02	-0.02	-0.02	-0.02	-0.022	t	48819	PPLGNWFG	Rattus norvegicus
1	1.00	1	1	-0.02	-0.02	-0.02	-0.02	-0.022	t	48819	PPLGNWFG	Mus musculus
1	1.00	1	1	0.03	0.028	0.02	0.029	0.029	t	78687	IRVRKRKGSNKKAEDTLED	Mus musculus
1	1.00	1	1	0	-0.004	-0.002	0.003	0.001	t	99137	DDTFTV	Oryctolagus cuniculus
1	1.00	1	1	0.04	0.04	0.04	0.04	0.04	t	153865	PETTDLYCY	Cavia porcellus
1	1.00	1	1	0.01	0.01	0.008	0.014	0.009	t	47593	PFVQWFVGL	Homo sapiens
1	1.00	1	1	0.02	0.02	0.02	0.023	0.02	t	72087	VYLLPR	Homo sapiens
1	1.00	1	1	-0.01	-0.01	-0.017	0	-0.009	t	117383	TLHKAFKGSIFVVFDSIESA	Homo sapiens
1	1.00	1	1	0.01	0.006	0.01	0.01	0.008	t	70388	VPNLRGDLQVLA	Mus musculus
1	1.00	1	1	0.04	0.042	0.028	0.041	0.04	t	142415	EVAKAEVLNAEVK	Homo sapiens
1	1.00	1	1	-0.01	-0.01	-0.017	-0.01	-0.01	t	121966	VFRFSFVPV	Mus musculus
1	1.00	1	1	-0.01	-0.008	-0.017	-0.007	-0.01	t	117333	MVTSTITEKLLKNLVKI	Homo sapiens
1	1.00	1	1	0.03	0.03	0.021	0.032	0.03	t	17531	FQSFPSGEPSNSKLSGFFQS	Homo sapiens
1	1.00	1	1	-0.01	-0.008	-0.01	-0.003	-0.011	t	107758	AWNCCVKQTP	Homo sapiens

1	1.00	1	1	-0.01	-0.012	-0.014	-0.011	-0.01	t	8880	DKHIEQYLKK	Mus musculus
1	1.00	1	1	0.04	0.039	0.03	0.04	0.04	t	55248	RPPETTTI	Anas platyrhynchos
1	1.00	1	1	0.01	0.006	-0.002	0.01	0.01	t	95663	PKYVKQNT	Mus musculus
1	1.00	1	1	0.01	0.008	0.006	0.013	0.009	t	158947	LDIDIDPKVKVATLS	Mus musculus
1	1.00	1	1	0.02	0.018	0.011	0.023	0.023	t	57654	SETQKSQNY	Mus musculus
1	1.00	1	1	0.01	0.012	0.012	0.017	0.009	t	96808	QVKSGTIFDN	Homo sapiens
1	1.00	1	1	0.02	0.02	0.013	0.02	0.02	t	138257	VLTVLHQ	Homo sapiens
1	1.00	1	1	0.04	0.038	0.03	0.039	0.04	t	14358	ETEFSKLY	Mus musculus
1	1.00	1	1	0	-0.002	-0.007	0.003	0.001	t	107062	AEFRHD	Mus musculus
1	1.00	1	1	0.02	0.022	0.013	0.023	0.022	t	77841	AYDFLYNYL	Homo sapiens
1	1.00	1	1	0.02	0.02	0.02	0.02	0.019	t	68399	VEVPGS	Oryctolagus cuniculus
1	1.00	1	1	0.01	0.008	0.002	0.009	0.011	t	56699	SAAFYYRV	Mus musculus
1	1.00	1	1	0.02	0.024	0.007	0.021	0.02	t	67167	TVSSNGDR	Homo sapiens
1	1.00	1	1	0.01	0.006	0.01	0.013	0.007	t	111966	YKSNNIARMRLYDPN	Oryctolagus cuniculus
1	1.00	1	1	0	0	0	0.007	-0.001	t	58388	SHLVEALYLVCGERG	Mus musculus

1	1.00	1	1	-0.01	-0.008	-0.017	-0.003	-0.01	t	156799	VYATRSSA VRLRSSV	Homo sapiens
1	1.00	1	1	0.02	0.02	0.011	0.02	0.02	t	132715	VEEFSPSIA REIYEMY	Mus musculu s
1	1.00	1	1	-0.02	-0.024	-0.037	-0.023	-0.02	t	55935	RSQRQLLQ W	Oryctola gus cuniculu s
1	1.00	1	1	0.02	0.016	0.01	0.019	0.02	t	7817	DDLDDKC KNDGW	Oryctola gus cuniculu s
1	1.00	1	1	0.01	0.008	0.009	0.008	0.01	t	36315	LGSAYP	Mus musculu s
1	1.00	1	1	0.02	0.024	0.018	0.027	0.02	t	131955	LPHGNALV RYSSKEE	Homo sapiens
1	1.00	1	1	-0.03	-0.03	-0.02	-0.019	-0.032	t	14013	ERKRR	Homo sapiens
1	1.00	1	1	0.03	0.03	0.03	0.033	0.03	t	149116	SYAAVAS	Sus scrofa
1	1.00	1	1	0.02	0.024	0.02	0.021	0.02	t	18039	FTPGPSQ	Homo sapiens
1	1.00	1	1	0	0	-0.012	0	0	t	25624	IDLTNKNA AKL	Homo sapiens
1	1.00	1	1	0.02	0.018	0.013	0.02	0.019	t	95047	VEVPGSQ HIDSQ	Oryctola gus cuniculu s
1	1.00	1	1	0.02	0.018	0.013	0.02	0.019	t	95047	VEVPGSQ HIDSQ	Oryctola gus cuniculu s
1	1.00	1	1	0.03	0.035	0.035	0.03	0.03	t	41573	MGFSYD	Homo sapiens

1	1.00	1	1	0.03	0.03	0.035	0.03	0.03	t	41573	MGFSYD	Homo sapiens
1	1.00	1	1	0	0.002	-0.013	0.001	0	t	21606	GPEKAFRE	Homo sapiens
1	1.00	1	1	-0.02	-0.016	-0.032	-0.023	-0.022	t	105044	YQFVDCG DSF	Homo sapiens
1	1.00	1	1	0.05	0.048	0.038	0.049	0.05	t	9108	DLGTTL	Mus musculu s
1	1.00	1	1	0.01	0.012	-0.002	0.011	0.01	t	142407	DKTIAAAK KAKKARE	Homo sapiens
1	1.00	1	1	0.02	0.018	0.02	0.021	0.019	t	130457	CRQPGNN YC	Mus musculu s
1	1.00	1	1	0.04	0.04	0.03	0.04	0.04	t	119575	EWESTYSIL G	Homo sapiens
1	1.00	1	1	0.03	0.028	0.026	0.037	0.03	t	123851	WTEVSEV ATEV	Homo sapiens
1	1.00	1	1	0.02	0.02	0.012	0.02	0.02	t	31521	KKEFLNSY EDEFSSE	Mus musculu s
1	1.00	1	1	-0.02	-0.018	-0.027	-0.017	-0.021	t	144600	CQREFALK HL	Homo sapiens
1	1.00	1	1	-0.01	-0.01	-0.01	-0.01	-0.012	t	69828	VLVLFGFF TLSPWY	Homo sapiens
1	1.00	1	1	-0.01	-0.018	-0.018	-0.011	-0.01	t	110932	LNLKIDGL KISV	Macaca
1	1.00	1	1	0	0	-0.009	0.002	0	t	135941	ITTQECAN VVVG	Homo sapiens
1	1.00	1	1	-0.05	-0.05	-0.065	-0.052	-0.05	t	50350	QAQYNQ MHML	Homo sapiens
1	1.00	1	1	0.03	0.028	0.018	0.029	0.03	t	72544	WHLKSIG QLVIE	Mus musculu s

1	1.00	1	1	0.02	0.022	0.008	0.021	0.02	t	142385	AKTAATNAEKKKTK	Homo sapiens
1	1.00	1	1	-0.01	-0.008	-0.01	-0.003	-0.01	t	104254	SPFLKAF	Homo sapiens
1	1.00	1	1	0.02	0.02	0.008	0.02	0.02	t	150200	FGEHKQEKDLEYGAC	Mus musculus
1	1.00	1	1	0.01	0.012	0.001	0.011	0.01	t	67444	TYRATYQD	Homo sapiens
1	1.00	1	1	0.03	0.027	0.018	0.03	0.03	t	144611	DFNGSFDMNGTITA	Oryctolagus cuniculus
1	1.00	1	1	-0.01	-0.01	-0.021	-0.013	-0.01	t	53152	RALLGLAVCLFLMALCT	Homo sapiens
1	1.00	1	1	0.05	0.048	0.039	0.053	0.053	t	45571	NQITSALSTQ	Mus musculus
1	1.00	1	1	0.02	0.02	0.02	0.02	0.02	t	75202	YNPLLVET	Homo sapiens
1	1.00	1	1	0.07	0.07	0.07	0.07	0.07	t	159283	EGP	Oryctolagus cuniculus
1	1.00	1	1	0.03	0.027	0.02	0.03	0.03	t	13354	EMDKID	Oryctolagus cuniculus
1	1.00	1	1	0.02	0.02	0.032	0.02	0.015	t	19264	GEFMFDFDGDEIF	Mus musculus
1	1.00	1	1	0.01	0.008	0.002	0.007	0.01	t	34132	KVNDA	Mus musculus
1	1.00	1	1	0.01	0.013	0	0.01	0.01	t	120402	ALKASGLVPE	Mus musculus
1	1.00	1	1	0.01	0.01	0	0.01	0.01	t	120402	ALKASGLVPE	Mus musculus
1	1.00	1	1	-0.01	-0.01	-0.01	-0.006	-0.012	t	135970	PPGMRPPMGPMGIPP	Homo sapiens

1	1.00	1	1	0.04	0.04	0.03	0.04	0.04	t	9980	DRGYISQY	Homo sapiens
1	1.00	1	1	0.01	0.01	0.01	0.01	0.007	t	62830	TAEGRAIN	Mus musculu s
1	1.00	1	1	0	0	-0.016	0.002	0.003	t	98930	PFRPQQPY PQPQ	Homo sapiens
1	1.00	1	1	0.07	0.072	0.075	0.077	0.068	t	118289	DEKKEESE ESDDDMG	Homo sapiens
1	1.00	1	1	0.03	0.03	0.016	0.024	0.029	t	38294	LNPDLQ	Mus musculu s
1	1.00	1	1	-0.01	-0.006	-0.019	-0.01	-0.01	t	113993	VHTGFGA MDFTTLQ	Cavia porcellu s
1	1.00	1	1	-0.01	-0.006	-0.019	-0.01	-0.01	t	113993	VHTGFGA MDFTTLQ	Cavia porcellu s
1	1.00	1	1	-0.01	-0.013	-0.019	-0.01	-0.01	t	113993	VHTGFGA MDFTTLQ	Oryctola gus cuniculu s
1	1.00	1	1	-0.02	-0.02	-0.022	-0.02	-0.022	t	79748	AIFTKPKA	Mus musculu s
1	1.00	1	1	-0.02	-0.02	-0.022	-0.02	-0.022	t	79748	AIFTKPKA	Mus musculu s
1	1.00	1	1	-0.01	-0.008	-0.019	-0.013	-0.012	t	50833	QGASVDK R	Homo sapiens
1	1.00	1	1	-0.02	-0.016	-0.016	-0.013	-0.021	t	144700	KEWAKRA ALP	Homo sapiens
1	1.00	1	1	0.02	0.023	0.012	0.02	0.022	t	48774	PPFFLVQE KGI	Mus musculu s
1	1.00	1	1	-0.01	-0.01	-0.019	-0.01	-0.01	t	29802	KAFRELAD	Homo sapiens

1	1.00	1	1	0.04	0.04	0.04	0.04	0.04	t	11481	EDYPSP	Homo sapiens
1	1.00	1	1	0.03	0.031	0.029	0.033	0.029	t	99264	GAKEPY	Equus caballus
1	1.00	1	1	-0.01	-0.01	-0.018	-0.01	-0.01	t	30704	KFDFDKSK VKENSY	Mus musculu s
1	1.00	1	1	0.01	0.008	0.003	0.013	0.011	t	107109	EFRHDS	Mus musculu s
1	1.00	1	1	0.02	0.02	0.02	0.02	0.02	t	27421	ILYEAFDE M	Homo sapiens
1	1.00	1	1	0.03	0.026	0.02	0.033	0.03	t	51078	QIHTLN	Oryctola gus cuniculu s
1	1.00	1	1	0.02	0.018	0.02	0.019	0.019	t	69883	VLYQEFDE	Mus musculu s
1	1.00	1	1	0.02	0.022	0.003	0.023	0.022	t	96537	LDQAAKD AKSAGVN VVQDA	Homo sapiens(human)
1	1.00	1	1	0.02	0.022	0.011	0.021	0.021	t	110808	DLYCYEQL NDSSEE	Homo sapiens
1	1.00	1	1	0.01	0.01	0.01	0.01	0.01	t	23221	GVYLLPRR	Homo sapiens
1	1.00	1	1	-0.02	-0.02	-0.015	-0.02	-0.024	t	108382	GGVVIA	Mus musculu s
1	1.00	1	1	0.04	0.043	0.031	0.04	0.04	t	100555	THQDEIYE QV	Mus musculu s
1	1.00	1	1	0.03	0.03	0.025	0.033	0.03	t	39472	LSKYTINP	Homo sapiens
1	1.00	1	1	0.01	0.014	0.008	0.01	0.009	t	105805	GAGIGWG ID	Mus musculu s
1	1.00	1	1	-0.02	-0.02	-0.035	-0.02	-0.02	t	102912	VPKVTKEK PTPPVKPT APTK	Mus musculu s

1	1.00	1	1	-0.01	-0.013	-0.018	-0.01	-0.012	t	153392	GNTLQCIMLVYCFLGYCC	Oryctolagus cuniculus
1	1.00	1	1	0.04	0.04	0.045	0.041	0.038	t	106633	LVFFAED	Mus musculus
1	1.00	1	1	0.04	0.04	0.045	0.041	0.038	t	106633	LVFFAED	Mus musculus
1	1.00	1	1	0.02	0.02	0.013	0.02	0.02	t	138257	VLTVLHQ	Homo sapiens
1	1.00	1	1	0.03	0.03	0.028	0.03	0.03	t	107378	GELP	Rattus norvegicus
1	1.00	1	1	-0.02	-0.02	-0.035	-0.02	-0.02	t	53864	RGFLGRMLF	Homo sapiens
1	1.00	1	1	0.03	0.028	0.03	0.029	0.028	t	22494	GSRYPGQGSPG	Mus musculus
1	1.00	1	1	0.01	0.012	0.008	0.013	0.01	t	117653	TLESLPAPETSVYFCA	Homo sapiens
1	1.00	1	1	0.02	0.02	0.02	0.023	0.021	t	69883	VLYQEFDE	Homo sapiens
1	1.00	1	1	0	0.002	0	0.007	-0.001	t	110996	RARPAKAAATQKKVERKA	Homo sapiens
1	1.00	1	1	0.05	0.05	0.034	0.052	0.053	t	99042	VLQQSTYQLLQE	Homo sapiens
1	1.00	1	1	-0.02	-0.018	-0.018	-0.017	-0.023	t	120612	IFNLFSTKDSSAAW	Homo sapiens
1	1.00	1	1	-0.03	-0.03	-0.037	-0.02	-0.028	t	96211	EDKKRKEEEE	Homo sapiens
1	1.00	1	1	0.03	0.03	0.03	0.03	0.03	t	105855	GVV	Mus musculus
1	1.00	1	1	0.05	0.05	0.045	0.052	0.048	t	118133	SESQTLDKVYQM	Homo sapiens

1	1.00	1	1	0.03	0.028	0.01	0.023	0.03	t	65421	TNSGINNS	Mus musculus
1	1.00	1	1	0	0	0	0.006	-0.002	t	11819	EENVEHDA	Homo sapiens
1	1.00	1	1	0.02	0.02	0.008	0.02	0.02	t	105078	AFNHFGIQLVQR	Homo sapiens
1	1.00	1	1	-0.01	-0.01	-0.024	-0.01	-0.01	t	111722	RGLPRYFNVTLRKRWV	Oryctolagus cuniculus
1	1.00	1	1	0.03	0.03	0.016	0.027	0.03	t	30212	KDLVFTKENTIT	Homo sapiens
1	1.00	1	1	0.05	0.054	0.038	0.051	0.05	t	142440	LDISPESADNITSK	Homo sapiens
1	1.00	1	1	-0.03	-0.028	-0.033	-0.033	-0.032	t	111212	DQIKVSTAIDLT	Homo sapiens
1	1.00	1	1	-0.03	-0.028	-0.033	-0.033	-0.032	t	111212	DQIKVSTAIDLT	Homo sapiens
1	1.00	1	1	0	0	0	0.003	-0.003	t	45013	NMPASMKRVRIE	Rattus norvegicus
1	1.00	1	1	0	0	0	0.003	-0.003	t	33215	KRRLRPSRVRIE	Rattus norvegicus
1	1.00	1	1	0.02	0.022	0.013	0.018	0.018	t	106595	LDGETKTGPQRV	Homo sapiens
1	1.00	1	1	0	0.001	-0.009	0.001	0	t	7355	CYDLSCNQTVCQ	Myodes glareolus
1	1.00	1	1	0	0.001	-0.009	0.001	0	t	7355	CYDLSCNQTVCQ	Myodes glareolus
1	1.00	1	1	-0.03	-0.004	-0.026	-0.021	-0.031	t	106906	THWQGVYWVGATPKA	Maccullochella macquariensis
1	1.00	1	1	0.06	0.06	0.027	0.06	0.067	t	102093	STGSSDV	Mus musculus

1	1.00	1	1	0	0	-0.005	0	0	t	58742	SIVTAPFQ VK	Homo sapiens
1	1.00	1	1	-0.05	-0.048	-0.05	-0.043	-0.048	t	118864	KIPPTPFS	Homo sapiens
1	1.00	1	1	0	0	-0.014	0	0	t	51942	QPQRPFG QQP	Mus musculu s
1	1.00	1	1	0.04	0.042	0.04	0.047	0.041	t	131762	YAASSYL	Homo sapiens
1	1.00	1	1	0.02	0.016	0.022	0.02	0.018	t	100034	FFAEDVGS NK	Oryctola gus cuniculu s
1	1.00	1	1	0.02	0.02	0.02	0.02	0.02	t	146019	TCPPDIIP	Mus musculu s
1	1.00	1	1	-0.01	-0.006	-0.023	-0.013	-0.01	t	31638	KKMSQFID KKFIPLVFY TLK	Homo sapiens
1	1.00	1	1	0.05	0.05	0.034	0.052	0.053	t	98941	PQYSQPQ QPISQ	Homo sapiens
1	1.00	1	1	0	0	-0.007	-0.003	-0.002	t	14109	ESAFNKAL NECT	Homo sapiens
1	1.00	1	1	0.02	0.018	0.017	0.019	0.02	t	125400	ELLKLLIEA	Mus musculu s
1	1.00	1	1	0	-0.002	-0.01	0.003	0	t	6073	CCRGRCN KKGEQVG M	Homo sapiens
1	1.00	1	1	0.01	0.012	0.008	0.017	0.01	t	115619	IRALVGDE VELP	Homo sapiens
1	1.00	1	1	-0.01	-0.006	-0.02	-0.009	-0.01	t	30041	KCAFATTP VCQF	Myodes glareolu s
1	1.00	1	1	-0.01	-0.01	-0.02	-0.01	-0.01	t	63236	TDSPGG	Oryctola gus cuniculu s
1	1.00	1	1	0.05	0.05	0.041	0.05	0.05	t	135990	SASVPALT AVET	Homo sapiens

1	1.00	1	1	0.02	0.018	0	0.013	0.02	t	128146	WSVCVGLTTYVNDYVLPYLA	Mus musculus
1	1.00	1	1	0.05	0.05	0.048	0.049	0.048	t	42277	MPHKLEKADLLKAIQEQLIA	Homo sapiens
1	1.00	1	1	0.03	0.03	0.024	0.03	0.03	t	48876	PPPQAPYQGY	Mus musculus
1	1.00	1	1	-0.01	-0.006	-0.01	-0.003	-0.01	t	123612	PPGMIPPP	Homo sapiens
1	1.00	1	1	-0.01	-0.008	-0.022	-0.01	-0.01	t	37626	LLNQLAGR	Anas platyrhynchos
1	1.00	1	1	0.04	0.036	0.028	0.037	0.04	t	82932	GTNVELGGRLSI	Oryctolagus cuniculus
1	1.00	1	1	0.01	0.014	0.005	0.013	0.012	t	134185	PLQKTY	Homo sapiens
1	1.00	1	1	0.03	0.028	0.025	0.033	0.03	t	72466	WGENDTDVFVLNNTR	Mus musculus
1	1.00	1	1	-0.01	-0.01	-0.01	-0.01	-0.012	t	159294	QPLWLL	Oryctolagus cuniculus
1	1.00	1	1	0	0.002	-0.004	-0.003	-0.002	t	106691	NTFWASDLMH	Homo sapiens
1	1.00	1	1	0.03	0.027	0.039	0.03	0.024	t	46084	NTEPSQLPPTA	Oryctolagus cuniculus
1	1.00	1	1	0.04	0.04	0.046	0.041	0.039	t	8329	DFVEGV	Homo sapiens

1	1.00	1	1	-0.05	-0.053	-0.063	-0.05	-0.05	t	55639	RRLGAALL	Oryctolagus cuniculus
1	1.00	1	1	-0.03	-0.026	-0.037	-0.023	-0.028	t	144594	ARGISRTKYW	Homo sapiens
1	1.00	1	1	0.02	0.005	0.008	0.017	0.02	t	42655	MSRSER	Marmota monax
1	1.00	1	1	0.04	0.043	0.039	0.04	0.04	t	122113	IIASTEKLP	Mus musculus
1	1.00	1	1	0.05	0.05	0.05	0.061	0.05	t	118289	DEKKEESEESDDDMG	Homo sapiens
1	1.00	1	1	-0.03	-0.032	-0.042	-0.03	-0.03	t	100667	YMKPKHVRVWCPRP	Homo sapiens
1	1.00	1	1	0.02	0.02	0.012	0.02	0.02	t	30180	KDKDKDNTDE	Mus musculus
1	1.00	1	1	0.01	0.007	-0.007	0.01	0.013	t	97020	TVTRGTISDP	Oryctolagus cuniculus
1	1.00	1	1	0	0	-0.011	0	0	t	26328	IGTLKKILDETVKDKLAK	Mus musculus
1	1.00	1	1	0.01	0.008	0.011	0.013	0.01	t	30863	KFTEPVKDIM	Mus musculus
1	1.00	1	1	0	0.004	0	0.007	0	t	108074	PQVYTLP	Homo sapiens
1	1.00	1	1	0	0.002	0	0.007	0	t	108074	PQVYTLP	Homo sapiens
1	1.00	1	1	0	0.002	0	0.007	0	t	108074	PQVYTLP	Homo sapiens
1	1.00	1	1	0.06	0.06	0.053	0.06	0.06	t	119524	SKEQV	Homo sapiens

1	1.00	1	1	0.01	0.014	-0.002	0.007	0.009	t	67707	VAPFPEVFGK	Homo sapiens
1	1.00	1	1	-0.03	-0.028	-0.041	-0.027	-0.03	t	110468	YYVLHLCLAATKYPLLKLLG	Homo sapiens
1	1.00	1	1	0.03	0.03	0.03	0.03	0.03	t	153865	PETTDLYCY	Cavia porcellus
1	1.00	1	1	0.01	0.012	0.003	0.013	0.012	t	48940	PPVADL	Homo sapiens
1	1.00	1	1	0.02	0.022	0.013	0.021	0.02	t	1022	AEKQKAAEATKVAE	Homo sapiens
1	1.00	1	1	-0.02	-0.016	-0.018	-0.013	-0.023	t	134679	GGSGPQRRGG	Homo sapiens
1	1.00	1	1	-0.03	-0.028	-0.035	-0.027	-0.032	t	134076	GWLGFNKERGHLQ	Homo sapiens
1	1.00	1	1	-0.05	-0.05	-0.062	-0.046	-0.048	t	96239	EKPTPPVKPT	Homo sapiens(human)
1	1.00	1	1	0.04	0.037	0.028	0.04	0.041	t	132838	HSDLKIQE	Oryctolagus cuniculus
1	1.00	1	1	-0.01	-0.012	-0.02	-0.007	-0.01	t	6073	CCRGRCNKKGEQVGM	Homo sapiens
1	1.00	1	1	0.02	0.022	0.015	0.021	0.019	t	17557	FQYSPG	Homo sapiens
1	1.00	1	1	0.05	0.054	0.05	0.047	0.047	t	67323	TYDNE	Homo sapiens
1	1.00	1	1	0.03	0.026	0.03	0.033	0.03	t	105853	GVTHDQLN	Oryctolagus cuniculus
1	1.00	1	1	-0.01	-0.013	-0.02	-0.01	-0.01	t	98434	RYMDQPSRD	Oryctolagus cuniculus

1	1.00	1	1	-0.01	-0.01	-0.02	-0.01	-0.01	t	98434	RYMDQPSRD	Mus musculus
1	1.00	1	1	-0.02	-0.02	-0.02	-0.02	-0.024	t	141760	TAPPLLPH	Rattus norvegicus
1	1.00	1	1	0.03	0.033	0.021	0.03	0.03	t	55802	RSASAAAILEEDDSK	Mus musculus
1	1.00	1	1	0.06	0.06	0.06	0.06	0.06	t	120082	LPTDSGEK	Rattus norvegicus
1	1.00	1	1	0.06	0.062	0.06	0.06	0.06	t	120082	LPTDSGEK	Rattus norvegicus
1	1.00	1	1	0	0.003	-0.009	0	0	t	100407	QDLLFTNQLK	Mus musculus
1	1.00	1	1	0.02	0.015	0.016	0.02	0.02	t	71568	VTVVTTSGS	Bos taurus
1	1.00	1	1	0.03	0.032	0.02	0.031	0.03	t	44613	NKVDAAVA	Homo sapiens
1	1.00	1	1	0.03	0.032	0.02	0.031	0.03	t	44613	NKVDAAVA	Homo sapiens
1	1.00	1	1	0.01	0.014	0.01	0.017	0.009	t	107478	KEYKCKV	Homo sapiens
1	1.00	1	1	0.01	0.012	0.01	0.017	0.009	t	107478	KEYKCKV	Homo sapiens
1	1.00	1	1	-0.07	-0.033	-0.08	-0.07	-0.074	t	12792	EKLCVWNNKTPHAIA	Oryctolagus cuniculus
1	1.00	1	1	0	0.002	-0.007	0.001	0	t	150965	AKSFYKNLIWLVKKG	Homo sapiens

1	1.00	1	1	0.01	-0.003	0.005	0.01	0.009	t	19096	GDRADGQPA	Aotus griseimembra
1	1.00	1	1	-0.05	-0.045	-0.067	-0.05	-0.05	t	21334	GMRFDRGY	Homo sapiens
1	1.00	1	1	0.03	0.03	0.013	0.03	0.031	t	114142	PQKSQRSQDEN	Rattus norvegicus
1	1.00	1	1	0.04	0.04	0.028	0.04	0.04	t	140674	ESQPDPKPDELHKS	Mus musculus
1	1.00	1	1	-0.01	-0.012	-0.022	-0.007	-0.01	t	107092	AVDAVIAELKKQSKP	Rattus norvegicus
1	1.00	1	1	0.05	0.05	0.05	0.053	0.05	t	149054	GSPNFTQ	Sus scrofa
1	1.00	1	1	0.06	0.06	0.053	0.057	0.06	t	42678	MSTLQELQENITA	Homo sapiens
1	1.00	1	1	0.05	0.05	0.037	0.047	0.05	t	5206	ATVSSQSF	Homo sapiens
1	1.00	1	1	-0.01	-0.008	-0.01	-0.003	-0.01	t	104343	VSGGPFI	Homo sapiens
1	1.00	1	1	0.01	0.01	-0.006	0.01	0.012	t	10801	DWEDRYYRE	Mus musculus
1	1.00	1	1	-0.01	-0.01	-0.01	-0.01	-0.015	t	133536	CKPVENRAC	Mus musculus
1	1.00	1	1	0.02	0.02	0.015	0.02	0.02	t	114224	PQDENPVVHF	Mus musculus
1	1.00	1	1	0.02	0.023	0.015	0.02	0.02	t	114224	PQDENPVVHF	Mus musculus
1	1.00	1	1	-0.03	-0.032	-0.039	-0.027	-0.029	t	115483	SFFDIGGSVSAGFSN	Mus musculus
1	1.00	1	1	0	0	-0.015	-0.003	0	t	61760	STLTITVNSKKT	Homo sapiens
1	1.00	1	1	0.03	0.03	0.023	0.03	0.03	t	119508	QFIGYPI	Homo sapiens

1	1.00	1	1	-0.04	-0.038	-0.04	-0.033	-0.041	t	134764	PTPGPSRRGP	Homo sapiens
1	1.00	1	1	0.02	0.022	0.013	0.023	0.02	t	53281	RCIPALDSLT	Homo sapiens
1	1.00	1	1	-0.01	-0.01	-0.017	-0.01	-0.01	t	122944	WEGVGVVPDV	Rattus norvegicus
1	1.00	1	1	0.02	0.026	0.009	0.02	0.02	t	94178	VSAFTSSNDS	Ovis aries
1	1.00	1	1	0.01	0.01	-0.005	0.013	0.013	t	56142	RTNPSDNSSDSNIKT	Homo sapiens
1	1.00	1	1	0.06	0.06	0.053	0.06	0.058	t	102309	ANQTEV	Mus musculus
1	1.00	1	1	0	0.002	0.011	0.007	-0.003	t	69265	VKLSG	Homo sapiens
1	1.00	1	1	0.02	0.02	0.015	0.023	0.02	t	4091	AQVIAEHQF	Homo sapiens
1	1.00	1	1	0	0.002	-0.011	0.003	0	t	158834	IGFASKSADDVKKAF	Homo sapiens
1	1.00	1	1	0	0.004	-0.013	0.001	0	t	19014	GDKPSLFGQAAAGDKPSLF	Homo sapiens
1	1.00	1	1	0.01	0.014	0.007	0.011	0.009	t	103297	IWKLIKDMLILPYLD	Homo sapiens
1	1.00	1	1	0.02	0.02	0.02	0.03	0.02	t	114910	NTTSEDGKVM	Homo sapiens
1	1.00	1	1	0.03	0.028	0.03	0.033	0.028	t	51262	QKTTS	Mus musculus
1	1.00	1	1	-0.04	-0.038	-0.04	-0.033	-0.04	t	117229	RHGFLPRHRD	Homo sapiens
1	1.00	1	1	0.03	0.032	0.013	0.027	0.032	t	71199	VSVNDPFIDLEYM	Homo sapiens
1	1.00	1	1	-0.01	-0.01	-0.021	-0.01	-0.011	t	14210	ESMAGKREMV	Mus musculus

1	1.00	1	1	-0.03	-0.03	-0.05	-0.036	-0.03	t	49372	PSGDRA	Homo sapiens
1	1.00	1	1	0.04	0.026	0.033	0.04	0.039	t	137867	GEIKFEAV EGAV	Oryctola gus cuniculu s
1	1.00	1	1	0.03	0.033	0.024	0.03	0.028	t	9592	DPAKAAF DSLQASAT	Mus musculu s
1	1.00	1	1	0.01	0.008	0.01	0.013	0.01	t	3394	ANQPGH	Homo sapiens
1	1.00	1	1	0.02	0.018	0.004	0.013	0.02	t	98122	IQNSKSAY TP	Mus musculu s
1	1.00	1	1	0.02	0.016	0.004	0.013	0.02	t	98122	IQNSKSAY TP	Oryctola gus cuniculu s
1	1.00	1	1	0.03	0.033	0.021	0.03	0.03	t	100406	QDEIYEQV HS	Mus musculu s
1	1.00	1	1	-0.03	-0.03	-0.034	-0.03	-0.03	t	60792	SRRYPFVK F	Homo sapiens
1	1.00	1	1	-0.01	-0.01	-0.016	-0.011	-0.01	t	31787	KLDLYGKV H	Sus scrofa
1	1.00	1	1	0	0	-0.012	0.004	0.002	t	96450	KEKPTPPV KP	Homo sapiens(human)
1	1.00	1	1	-0.02	-0.017	-0.029	-0.02	-0.018	t	120790	LVPEHADA FK	Mus musculu s
1	1.00	1	1	-0.02	-0.034	-0.029	-0.017	-0.02	t	34476	KYKDKPLY	Macaca mulatta
1	1.00	1	1	-0.02	-0.022	-0.029	-0.017	-0.02	t	66792	TTQTKPKS AF	Mus musculu s
1	1.00	1	1	-0.06	-0.058	-0.067	-0.053	-0.06	t	121983	VKGWRPR R	Homo sapiens

1	1.00	1	1	0.04	0.042	0.033	0.041	0.04	t	62975	TAQSTMKAEEITP	Homo sapiens
1	1.00	1	1	-0.01	-0.013	-0.022	-0.009	-0.009	t	88446	SDTVQTRHVVQHRSR	Homo sapiens
1	1.00	1	1	0.03	0.028	0.021	0.029	0.03	t	49450	PSNQDSFQWQ	Mus musculus
1	1.00	1	1	0.05	0.052	0.04	0.057	0.053	t	125229	RDSEGDLVAEKLGPI	Homo sapiens
1	1.00	1	1	0	0	0	0.006	-0.002	t	868	AEEAFDLWNECAKACV	Homo sapiens
1	1.00	1	1	0	-0.003	-0.009	0	0	t	112442	ACQKH	Oryctolagus cuniculus
1	1.00	1	1	0.04	0.042	0.025	0.043	0.042	t	119313	QGVTAEITPQASTTE	Homo sapiens
1	1.00	1	1	0	0	-0.011	0.006	0.002	t	16031	FGPLWILQASLLKV	Homo sapiens
1	1.00	1	1	0.04	0.042	0.038	0.04	0.04	t	36955	LKLYFEP	Homo sapiens
1	1.00	1	1	0.01	0.007	0.01	0.01	0.009	t	135566	KCSRLYTACVY	Oryctolagus cuniculus
1	1.00	1	1	0.04	0.043	0.03	0.04	0.04	t	57506	SEKSKCEENTMFQPY	Equus caballus
1	1.00	1	1	0	0	-0.008	0.003	0	t	110964	PNLKLNNGTDYK	Homo sapiens
1	1.00	1	1	0.05	0.05	0.05	0.05	0.052	t	127818	SDFGKFV	Homo sapiens
1	1.00	1	1	-0.02	-0.02	-0.025	-0.024	-0.022	t	123442	LLKDLRKN	Homo sapiens
1	1.00	1	1	-0.02	-0.018	-0.02	-0.019	-0.021	t	51428	QLLRIPQAIMDM	Homo sapiens

1	1.00	1	1	-0.01	-0.012	-0.017	-0.01	-0.011	t	120146	QGARVYA YVF	Oryctolagus cuniculus
1	1.00	1	1	-0.01	-0.01	-0.017	-0.01	-0.011	t	120146	QGARVYA YVF	Mus musculus
1	1.00	1	1	0	0	0	0.004	-0.003	t	133372	SPSPTPTP GPSRRG	Homo sapiens
1	1.00	1	1	-0.03	-0.028	-0.037	-0.023	-0.03	t	121667	NTHLKAVK MTLKNR	Homo sapiens
1	1.00	1	1	0.04	0.042	0.033	0.047	0.041	t	136547	PGGKHNM DFYVEALA FPDTD	Homo sapiens
1	1.00	1	1	-0.02	-0.02	-0.028	-0.02	-0.021	t	119654	TSGVHTFP	Homo sapiens
1	1.00	1	1	0.06	0.062	0.055	0.061	0.06	t	19621	GFSYDT	Homo sapiens
1	1.00	1	1	-0.01	-0.008	-0.01	-0.003	-0.01	t	112753	ARWELNL	Homo sapiens
1	1.00	1	1	0.01	0.01	-0.01	0.01	0.012	t	74475	YKLNFYFD LLRAKL	Mus musculus
1	1.00	1	1	0	0.004	0	0.007	-0.002	t	126922	REWIKFAA ACRE	Homo sapiens
1	1.00	1	1	0.02	0.018	0.008	0.021	0.022	t	70384	VPNLQGV PESPFSRT	Homo sapiens
1	1.00	1	1	0.02	0.018	0.017	0.019	0.018	t	67645	VAKETSDK Q	Mus musculus
1	1.00	1	1	0.01	0.014	0.014	0.017	0.009	t	144746	PTEEQVS WLS	Homo sapiens
1	1.00	1	1	0.02	0.016	0.006	0.019	0.023	t	50815	QFQTQQ	Oryctolagus cuniculus

1	1.00	1	1	0	0.002	0.004	0.007	0	t	145657	YRSYNDSVDP	Homo sapiens
1	1.00	1	1	0	0.003	0.002	0.007	0.001	t	104895	LVEALYLVCGERG	Mus musculus
1	1.00	1	1	0.05	0.048	0.05	0.051	0.052	t	130643	YSLLVEPYTFDP	Mus musculus
1	1.00	1	1	0.05	0.05	0.05	0.051	0.052	t	130643	YSLLVEPYTFDP	Homo sapiens
1	1.00	1	1	0.02	0.022	0.025	0.027	0.021	t	112801	LATPNPS	Homo sapiens
1	1.00	1	1	-0.03	-0.034	-0.044	-0.027	-0.028	t	105	AAEFTINKPK	Oryctolagus cuniculus
1	1.00	1	1	-0.05	-0.048	-0.055	-0.049	-0.051	t	72326	WDQMWKCLI	Homo sapiens
1	1.00	1	1	-0.03	-0.031	-0.045	-0.033	-0.03	t	41862	MKLTAA	Homo sapiens
1	1.00	1	1	0.01	0.012	0.01	0.011	0.007	t	37310	LLGACSFS	Homo sapiens
1	1.00	1	1	0	0	-0.007	0	-0.002	t	76606	YYSPAF	Mus musculus
1	1.00	1	1	0.02	0.02	0.02	0.023	0.021	t	69883	VLYQEFDE	Homo sapiens
1	1.00	1	1	0.04	0.04	0.04	0.04	0.038	t	140507	TKTWQSM	Mus musculus
1	1.00	1	1	0.03	0.023	0.03	0.03	0.03	t	114008	VSLVEETSFI	Oryctolagus cuniculus
1	1.00	1	1	-0.01	-0.014	-0.018	-0.007	-0.01	t	6106	CDICTNTMDVIKKML	Oryctolagus cuniculus
1	1.00	1	1	0.04	0.04	0.04	0.044	0.04	t	135975	PTLAFLQDVMNI	Homo sapiens

1	1.00	1	1	0.01	0.012	0.004	0.011	0.01	t	39254	LRYLA	Homo sapiens
1	1.00	1	1	0.01	0.012	0.004	0.011	0.01	t	39254	LRYLA	Homo sapiens
1	1.00	1	1	0.05	0.052	0.035	0.047	0.05	t	51279	QLADIN	Homo sapiens
1	1.00	1	1	0.05	0.05	0.05	0.059	0.052	t	130919	KFPEDFES	Homo sapiens
1	1.00	1	1	0.04	0.04	0.04	0.04	0.04	t	65846	TQGCNCSIYP	Homo sapiens
1	1.00	1	1	-0.04	-0.037	-0.04	-0.04	-0.042	t	134017	CFHQGKEYAPG	Mus musculus
1	1.00	1	1	0	0	-0.017	0	0.002	t	7939	DEDKRDGN	Oryctolagus cuniculus
1	1.00	1	1	0.02	0.022	-0.003	0.017	0.022	t	62277	SVPEVC	Homo sapiens
1	1.00	1	1	0.01	0.014	-0.002	0.009	0.011	t	141559	SDEALPLGSPR	Mus musculus
1	1.00	1	1	-0.02	-0.023	-0.022	-0.02	-0.021	t	95034	PKPAKSP	Oryctolagus cuniculus
1	1.00	1	1	-0.02	-0.02	-0.022	-0.02	-0.021	t	95034	PKPAKSP	Mus musculus
1	1.00	1	1	-0.05	-0.046	-0.057	-0.047	-0.051	t	115633	PPFSRVVHLYRN	Homo sapiens
1	1.00	1	1	-0.05	-0.036	-0.057	-0.047	-0.051	t	115633	PPFSRVVHLYRN	Homo sapiens
1	1.00	1	1	0	0	0	0.006	0	t	107064	AGAPGFPG	Rattus norvegicus
1	1.00	1	1	-0.01	-0.01	-0.026	-0.011	-0.007	t	98976	QYPLGQGSFRPS	Homo sapiens

1	1.00	1	1	-0.03	-0.028	-0.025	-0.023	-0.033	t	126757	NKPLITVTNSKY	Homo sapiens
1	1.00	1	1	0.01	0.01	0.003	0.01	0.011	t	107698	YNSTYRV	Homo sapiens
1	1.00	1	1	0	0.004	0.002	0.007	0	t	119641	SEHETRLVAKLFKDYS	Homo sapiens
1	1.00	1	1	0.01	0.01	0.008	0.01	0.009	t	75370	YPNQV	Mus musculus
1	1.00	1	1	0.01	0.006	0.009	0.009	0.01	t	78711	KQYDPDRP	Oryctolagus cuniculus
1	1.00	1	1	-0.01	-0.012	-0.016	-0.011	-0.012	t	70134	VNNKV	Mus musculus
1	1.00	1	1	-0.01	-0.006	-0.01	-0.003	-0.011	t	116682	CHGRGTCNYYANAYS	Homo sapiens
1	1.00	1	1	0.04	0.043	0.04	0.04	0.04	t	57504	SEKSEEIN	Mus musculus
1	1.00	1	1	0.04	0.043	0.04	0.04	0.04	t	57504	SEKSEEIN	Mus musculus
1	1.00	1	1	-0.01	-0.006	-0.01	-0.003	-0.011	t	107660	VLHQNWL	Homo sapiens
1	1.00	1	1	-0.02	-0.016	-0.031	-0.02	-0.021	t	93332	LSSLQFARAA	Ovis aries
1	1.00	1	1	0.06	0.06	0.06	0.063	0.06	t	149106	SITNPTQ	Sus scrofa
1	1.00	1	1	0.05	0.053	0.038	0.05	0.051	t	101363	VEPSYEAEPT	Mus musculus

1	1.00	1	1	0.05	0.056	0.057	0.057	0.048	t	127754	NTPEEKD	Homo sapiens
1	1.00	1	1	0.06	0.056	0.059	0.057	0.059	t	118991	RAEDNAD TLALVFEA	Oryctola gus cuniculu s
1	1.00	1	1	-0.01	-0.016	-0.008	-0.01	-0.012	t	5109	ATPYHPPA TP	Oryctola gus cuniculu s
1	1.00	1	1	0.03	0.036	0.03	0.03	0.03	t	58743	SIVYEAA	MD
1	1.00	1	1	-0.02	-0.02	-0.019	-0.017	-0.02	t	73741	YEPPVVHG	Homo sapiens
1	1.00	1	1	0.01	0.005	0	0.01	0.01	t	132827	FSQILPSFD DETMGR	Trichosu rus vulpecul a
1	1.00	1	1	-0.01	-0.01	-0.01	-0.01	-0.014	t	16755	FLLTRILTI	Mus musculu s
1	1.00	1	1	0.03	0.034	0.023	0.037	0.031	t	20097	GGYGDGG AHGGGYG G	Homo sapiens
1	1.00	1	1	-0.01	-0.014	-0.015	-0.007	-0.01	t	50887	QGIVEFYT	Oryctola gus cuniculu s
1	1.00	1	1	0.04	0.04	0.033	0.04	0.041	t	58728	SITTPSQ	Oryctola gus cuniculu s
1	1.00	1	1	0	0	-0.014	0	0	t	51942	QPQRPFG QQP	Mus musculu s
1	1.00	1	1	-0.03	-0.026	-0.03	-0.023	-0.03	t	123626	PPYMPPP G	Homo sapiens
1	1.00	1	1	0.01	0.01	-0.001	0.01	0.01	t	79898	LDPLVTAL LGAFD	Mus musculu s

1	1.00	1	1	0.01	0.01	0.006	0.014	0.009	t	120060	LGSSESRRALQDSQR	Homo sapiens
1	1.00	1	1	0.04	0.04	0.03	0.04	0.041	t	57542	SELSQLS	Mus musculus
1	1.00	1	1	0	0	0	0.008	-0.001	t	136080	RAGTDEGFG	Homo sapiens
1	1.00	1	1	0	0	0	0.008	-0.001	t	124604	QIERDTERDRFLSAPEAV	Homo sapiens
1	1.00	1	1	0.02	0.02	0.022	0.02	0.018	t	72911	WNSTTFHQTLQDP	Mus musculus
1	1.00	1	1	0.02	0.02	0.022	0.02	0.018	t	72911	WNSTTFHQTLQDP	Mus musculus
1	1.00	1	1	0.01	0.01	0.01	0.01	0.008	t	50823	QFYQKFPQYL	Homo sapiens
1	1.00	1	1	0.06	0.058	0.049	0.059	0.06	t	66084	TRQELANKQQESKENEKAL	Mus musculus
1	1.00	1	1	0.04	0.04	0.04	0.04	0.04	t	117509	GQIPPGAM	Homo sapiens
1	1.00	1	1	0.01	0.01	0	0.013	0.013	t	58582	SIKEDVQF	Homo sapiens
1	1.00	1	1	0.04	0.043	0.028	0.039	0.04	t	30939	KGGKGTEEK	Mus musculus
1	1.00	1	1	0.01	0.012	0.01	0.017	0.01	t	134773	QKSTHLLIRKLPFSR	Homo sapiens
1	1.00	1	1	0.01	0.012	0.018	0.009	0.008	t	2133	AIYGAA	Homo sapiens
1	1.00	1	1	0.01	0.01	0.01	0.02	0.013	t	141638	EIHSKTKS	Homo sapiens
1	1.00	1	1	0	-0.002	-0.004	-0.003	-0.002	t	18955	GDDPLLGNQSLL	Mus musculus

1	1.00	1	1	0	-0.003	-0.01	0	0	t	131874	EVFISAPR	Oryctolagus cuniculus
1	1.00	1	1	0.06	0.06	0.053	0.06	0.058	t	102310	ANQTEVEM	Mus musculus
1	1.00	1	1	0.02	0.022	0.013	0.021	0.02	t	34278	KVYVGNYDF	Homo sapiens
1	1.00	1	1	0.05	0.05	0.053	0.05	0.047	t	78938	CAPGQNLCY	Mus musculus
1	1.00	1	1	0.01	0.01	0.01	0.013	0.01	t	149049	FTPQMVR	Sus scrofa
1	1.00	1	1	-0.08	-0.076	-0.084	-0.077	-0.08	t	134143	LMPPKY	Homo sapiens
1	1.00	1	1	0.02	0.022	0.008	0.021	0.02	t	142413	EIAAEVAKAKVAK	Homo sapiens
1	1.00	1	1	0.05	0.05	0.047	0.05	0.051	t	102880	VDELD	Mus musculus
1	1.00	1	1	0.02	0.02	0.02	0.02	0.018	t	137407	GPEYWDGETRKVKA	Oryctolagus cuniculus
1	1.00	1	1	0	0.001	-0.007	-0.003	-0.001	t	130464	DFRVT	Mus musculus
1	1.00	1	1	0.02	0.02	0.009	0.02	0.02	t	125176	NNSTGDFEL	Mus musculus
1	1.00	1	1	0.03	0.032	0.028	0.03	0.029	t	122876	PSPDSKPT	Rattus norvegicus
1	1.00	1	1	-0.01	-0.008	-0.017	-0.013	-0.012	t	116040	YQQKPVALIN	Homo sapiens
1	1.00	1	1	-0.03	-0.028	-0.037	-0.023	-0.031	t	121267	ERMERKRREK	Homo sapiens
1	1.00	1	1	0.05	0.054	0.043	0.053	0.05	t	131657	SGLQAEDEADYYCSSY	Homo sapiens

1	1.00	1	1	0.03	0.028	0.024	0.029	0.03	t	55048	RNQE	Mus musculus
1	1.00	1	1	-0.03	-0.028	-0.027	-0.023	-0.03	t	119654	TSGVHTFP	Homo sapiens
1	1.00	1	1	-0.01	-0.01	-0.017	-0.01	-0.011	t	138027	SADFTPGT	Oryctolagus cuniculus
1	1.00	1	1	0.03	0.028	0.031	0.033	0.03	t	11377	EDLDGA	Mus musculus
1	1.00	1	1	0	-0.002	-0.013	0.003	0.002	t	75392	YPQPQPFRPQ	Mus musculus
1	1.00	1	1	0	0	0	0	-0.004	t	143676	DRVGLRNLRGYY	Mus musculus
1	1.00	1	1	0.06	0.06	0.06	0.06	0.059	t	143362	ETPLMKE	Mus musculus
1	1.00	1	1	0.06	0.06	0.06	0.06	0.059	t	143362	ETPLMKE	Mus musculus
1	1.00	1	1	-0.01	-0.006	-0.01	-0.003	-0.007	t	108075	PREPQVY	Homo sapiens
1	1.00	1	1	-0.04	-0.04	-0.047	-0.034	-0.04	t	10987	EAAGIGILTV	Homo sapiens
1	1.00	1	1	0.05	0.054	0.043	0.051	0.05	t	137858	FTPGTEYT	Homo sapiens
1	1.00	1	1	-0.01	-0.01	-0.009	-0.01	-0.012	t	76606	YYSPAF	Mus musculus
1	1.00	1	1	-0.01	-0.007	-0.009	-0.01	-0.012	t	76606	YYSPAF	Mus musculus
1	1.00	1	1	0	-0.003	-0.012	0	0	t	141792	YYLWKQYNPTGKGIDD	Oryctolagus cuniculus

1	1.00	1	1	0	-0.005	-0.007	-0.006	-0.002	t	148481	YTRDLVYK	Rattus norvegicus
1	1.00	1	1	-0.01	-0.013	-0.027	-0.01	-0.009	t	69233	VKGSSIAA	Oryctolagus cuniculus
1	1.00	1	1	0.03	0.03	0.03	0.03	0.027	t	138088	WLDLSKT	Mus musculus
1	1.00	1	1	0.04	0.038	0.045	0.043	0.039	t	138765	EEIKMDISKLNA	Mus musculus
1	1.00	1	1	0.09	0.086	0.087	0.09	0.09	t	141004	EPDP	Mus musculus
1	1.00	1	1	0.02	0.022	0.01	0.017	0.018	t	11190	EASQAADESERMRKV	Homo sapiens
1	1.00	1	1	0.01	0.01	0.003	0.02	0.011	t	117383	TLHKAFKGSIFVVFDSIESA	Homo sapiens
1	1.00	1	1	0.01	0	0.008	0.01	0.01	t	42655	MSRSER	Marmota monax
1	1.00	1	1	0	-0.003	-0.005	0	0	t	26339	IGVKGSSI	Oryctolagus cuniculus
1	1.00	1	1	0.03	0.034	0.023	0.027	0.028	t	50677	QEQIKGEEVREL	Homo sapiens
1	1.00	1	1	0	0	0	0.009	0.001	t	132098	TLLLEGVMAA	Homo sapiens
1	1.00	1	1	0.02	0.022	0.013	0.023	0.02	t	141638	EIHSKTKS	Homo sapiens
1	1.00	1	1	0	-0.003	-0.006	0	-0.001	t	105891	KADPSIMA	Oryctolagus cuniculus
1	1.00	1	1	0.01	0.01	-0.004	0.01	0.01	t	59376	SLNVLFDGRD	Mus musculus
1	1.00	1	1	0.01	0.013	0.01	0.01	0.009	t	134702	KEKKYS	Mus musculus

1	1.00	1	1	0.02	0.024	0.018	0.027	0.018	t	132044	RLRKWTT VDSISVNT	Homo sapiens
1	1.00	1	1	-0.02	-0.018	-0.027	-0.023	-0.022	t	115694	AVPYPQR DMP	Homo sapiens
1	1.00	1	1	0.01	0.012	0.004	0.011	0.008	t	59222	SLKLESSCN FDL	Homo sapiens
1	1.00	1	1	0	0.004	0	0.007	0	t	119678	YKCKVSNK	Homo sapiens
1	1.00	1	1	0.03	0.032	0.025	0.031	0.029	t	97598	RDLLKKGV DA	Homo sapiens
1	1.00	1	1	0.03	0.032	0.025	0.031	0.029	t	97598	RDLLKKGV DA	Homo sapiens
1	1.00	1	1	-0.02	-0.023	-0.03	-0.02	-0.021	t	6762	CPFIKPEVK PGTPA	Oryctola gus cuniculu s
1	1.00	1	1	0.03	0.03	0.021	0.03	0.029	t	69312	VKSASPHG ESNSSEE	Mus musculu s
1	1.00	1	1	-0.01	-0.017	-0.018	-0.01	-0.01	t	110971	PVSDLNLKI DGL	Macaca
1	1.00	1	1	0.02	0.005	0.008	0.017	0.02	t	42655	MSRSER	Marmot a monax
1	1.00	1	1	0.01	0.008	-0.002	0.01	0.01	t	100244	LANAYSHF YDGFAK	Homo sapiens
1	1.00	1	1	0	0.002	0	0.007	-0.001	t	9278	DLSLLGKA RKVVVTKD	Homo sapiens
1	1.00	1	1	0.02	0.02	0.02	0.02	0.018	t	95934	TTVRIVDQ CS	Homo sapiens
1	1.00	1	1	0	0	0	0	-0.004	t	135912	EALVLDTN GLMS	Mus musculu s

1	1.00	1	1	-0.03	-0.03	-0.042	-0.026	-0.028	t	96239	EKPTPPVKPT	Homo sapiens(human)
1	1.00	1	1	0.01	0.008	0.011	0.011	0.01	t	61473	SSWPKLEQF	Homo sapiens
1	1.00	1	1	0	0	0	0	0	t	71343	VTGSVVC	Mus musculus
1	1.00	1	1	-0.03	-0.032	-0.05	-0.027	-0.027	t	141713	NGTDRKTIRQLVWDAM	Rattus norvegicus
1	1.00	1	1	-0.02	-0.023	-0.028	-0.02	-0.022	t	2215	AKKPAGKGSPSTLQTPG	Oryctolagus cuniculus
1	1.00	1	1	0.01	0.012	0.006	0.011	0.009	t	110872	GSGSTANLASSNYFP	Homo sapiens
1	1.00	1	1	0.02	0.02	0.017	0.027	0.02	t	127935	ARLSAKPAPPKPEPK	Homo sapiens
1	1.00	1	1	-0.02	-0.022	-0.02	-0.021	-0.022	t	48819	PPLGNWFG	Mus musculus
1	1.00	1	1	0.05	0.053	0.05	0.05	0.051	t	134028	DDTIS	Mus musculus
1	1.00	1	1	0.03	0.032	0.028	0.037	0.029	t	51852	QPHDLGKVGEVIVTKD	Homo sapiens
1	1.00	1	1	0	0.002	-0.002	0.007	0.001	t	114013	VVHFFKNIVT	Homo sapiens
1	1.00	1	1	0.02	0.018	0	0.013	0.02	t	128152	YIFSEEAQVKDVPVVT	Mus musculus
1	1.00	1	1	0	-0.002	0.005	-0.001	-0.006	t	54707	RLPRYMQEV	Mus musculus
1	1.00	1	1	0.02	0.02	0.014	0.02	0.02	t	79524	CHTTATIPSSAVTC	Mus musculus
1	1.00	1	1	0.02	0.02	0.014	0.02	0.02	t	79524	CHTTATIPSSAVTC	Mus musculus

1	1.00	1	1	0.02	0.02	0.014	0.02	0.02	t	79524	CHTTATIPSSAVTC	Mus musculus
1	1.00	1	1	0.02	0.02	0.014	0.02	0.02	t	79524	CHTTATIPSSAVTC	Mus musculus
1	1.00	1	1	0	-0.002	-0.006	-0.001	0	t	133725	TVCTYRDFMY	Mus musculus
1	1.00	1	1	0.02	0.02	0.017	0.02	0.019	t	46786	PAEVTQLKGLS	Mus musculus
1	1.00	1	1	-0.01	-0.013	-0.017	-0.01	-0.011	t	135506	VTFPGA	Oryctolagus cuniculus
1	1.00	1	1	0.05	0.046	0.046	0.053	0.052	t	105753	ELPNIEAD	Oryctolagus cuniculus
1	1.00	1	1	0.04	0.04	0.04	0.044	0.04	t	115671	AAMEL	Homo sapiens
1	1.00	1	1	0.03	0.03	0.03	0.03	0.03	t	141895	FKYPEIEKYEVDG	Mus musculus
1	1.00	1	1	0.03	0.03	0.03	0.03	0.03	t	141895	FKYPEIEKYEVDG	Mus musculus
1	1.00	1	1	0.04	0.04	0.04	0.04	0.04	t	134257	VKSTP	Homo sapiens
1	1.00	1	1	0.03	0.03	0.03	0.03	0.029	t	64640	TKPTDGN	Oryctolagus cuniculus
1	1.00	1	1	0.03	0.03	0.03	0.03	0.029	t	64640	TKPTDGN	Oryctolagus cuniculus
1	1.00	1	1	0	0.003	-0.005	0	0	t	6317	CGMFTGLFNQGAQQKL	Mus musculus
1	1.00	1	1	0.01	0.014	0.01	0.01	0.009	t	137402	FIDSYICQV	Homo sapiens

1	1.00	1	1	0	0	0.003	0	-0.004	t	133697	SITTANPSSFNKY	Rattus norvegicus
1	1.00	1	1	-0.02	-0.02	-0.028	-0.017	-0.02	t	47983	PISLERLDVGTNLGN	Homo sapiens
1	1.00	1	1	-0.01	-0.008	-0.017	-0.007	-0.011	t	135905	CLFDRRLCSHLE	Homo sapiens
1	1.00	1	1	0.02	0.024	0.015	0.027	0.02	t	136087	VETDKATVGF	Homo sapiens
1	1.00	1	1	0.04	0.046	0.047	0.047	0.04	t	127646	EYNIMFGPDI	Homo sapiens
1	1.00	1	1	-0.02	-0.02	-0.024	-0.023	-0.022	t	114997	YTNDCLLCAY	Homo sapiens
1	1.00	1	1	0.06	0.062	0.059	0.063	0.058	t	47267	PEEFEGAGDGEPPEDDDSAT	Homo sapiens
1	1.00	1	1	-0.01	-0.008	-0.014	-0.009	-0.011	t	12625	EKAFREL	Homo sapiens
1	1.00	1	1	0.02	0.02	0.02	0.02	0.019	t	138623	SKFGGFDPQG	Oryctolagus cuniculus
1	1.00	1	1	0	0	0	0	-0.003	t	21990	GRAIN	Mus musculus
1	1.00	1	1	-0.02	-0.02	-0.023	-0.02	-0.02	t	67272	TWKSKK	Homo sapiens
1	1.00	1	1	0.01	0.016	0.011	0.011	0.009	t	99208	EKMYAC	Equus caballus
1	1.00	1	1	-0.02	-0.012	-0.013	-0.02	-0.025	t	123014	AIMRYIADK	Ovis aries

1	1.00	1	1	0.01	0.012	0.008	0.011	0.01	t	49405	PSIPSRAATL	Homo sapiens
1	1.00	1	1	0.01	0.014	0.003	0.01	0.01	t	21720	GPPAAEY	Homo sapiens
1	1.00	1	1	0.03	0.034	0.018	0.031	0.03	t	142472	SATAAETAKNNAVS	Homo sapiens
1	1.00	1	1	0	0.002	-0.01	-0.003	-0.002	t	50696	QESLLKANIQLVEKD	Homo sapiens
1	1.00	1	1	0.02	0.022	0.02	0.027	0.02	t	107520	LTVLHQN	Homo sapiens
1	1.00	1	1	0.01	0.009	0	0.01	0.01	t	37354	LLGNQSLL	Anas platyrhynchos
1	1.00	1	1	-0.01	-0.008	-0.015	-0.003	-0.01	t	121034	VHPDTGISSKAMGIMN	Homo sapiens
1	1.00	1	1	0.03	0.03	0.029	0.04	0.032	t	106647	MHLTTNI	Homo sapiens
1	1.00	1	1	0.03	0.032	0.018	0.031	0.03	t	70384	VPNLQGVPESPFSRT	Homo sapiens
1	1.00	1	1	0.04	0.039	0.033	0.04	0.039	t	55248	RPPETTTI	Anas platyrhynchos
1	1.00	1	1	0.01	0.008	0.004	0.013	0.01	t	63694	TFKSGATFQV	Mus musculus
1	1.00	1	1	0.01	0.01	0.001	0.008	0.01	t	800	ADYHRVMY	Homo sapiens
1	1.00	1	1	0.04	0.04	0.043	0.042	0.04	t	142150	EYMLDLQPETTD	Homo sapiens
1	1.00	1	1	-0.03	-0.028	-0.03	-0.023	-0.032	t	134782	RGRGRGGPRR	Homo sapiens
1	1.00	1	1	0.01	0.01	-0.004	0.01	0.01	t	75532	YQPQQPFPQP	Mus musculus

1	1.00	1	1	0.02	0.018	0.015	0.021	0.021	t	29939	KASAAC	Homo sapiens
1	1.00	1	1	-0.02	-0.018	-0.018	-0.013	-0.021	t	122392	HARLRDSG RAY	Homo sapiens
1	1.00	1	1	0.01	0.012	0.006	0.007	0.008	t	106898	TFWASDL MHR	Homo sapiens
1	1.00	1	1	0.03	0.026	0.029	0.03	0.03	t	8800	DITKD	Oryctola gus cuniculu s
1	1.00	1	1	0.04	0.04	0.04	0.041	0.038	t	106941	TVKQLYTL YPAA	Mus musculu s
1	1.00	1	1	-0.01	-0.01	-0.009	-0.006	-0.011	t	30945	KGGRKP	Homo sapiens
1	1.00	1	1	0.01	0.006	0.005	0.01	0.011	t	41713	MIHFGND	Mus musculu s
1	1.00	1	1	-0.01	-0.013	-0.008	-0.01	-0.012	t	122320	DVTVGPLI FL	Oryctola gus cuniculu s
1	1.00	1	1	0.04	0.04	0.04	0.04	0.04	t	58769	SIYQCC	Homo sapiens
1	1.00	1	1	0	-0.003	-0.013	0	0	t	21606	GPEKAFRE	Oryctola gus cuniculu s
1	1.00	1	1	0	-0.003	-0.013	0	0	t	21606	GPEKAFRE	Oryctola gus cuniculu s
1	1.00	1	1	0.01	0.008	-0.004	0.009	0.01	t	48107	PKNTLTPL EELYPT	Mus musculu s
1	1.00	1	1	0.01	0.012	-0.004	0.009	0.01	t	48107	PKNTLTPL EELYPT	Aotus nancyma ae
1	1.00	1	1	0.01	0.007	-0.003	0.009	0.012	t	1445	AGAKSAIL	Oryctola gus cuniculu s

1	1.00	1	1	0.01	0.014	0.005	0.011	0.009	t	3127	AMKPDLQNTLMAVPR	Homo sapiens
1	1.00	1	1	0.01	-0.001	-0.005	0.01	0.012	t	95142	RIKNTDGSIS	Oryctolagus cuniculus
1	1.00	1	1	0.01	0.007	-0.005	0.01	0.012	t	43509	NDPNRNVD	Oryctolagus cuniculus
1	1.00	1	1	-0.05	-0.052	-0.065	-0.053	-0.05	t	50350	QAQYNQMHML	Mus musculus
1	1.00	1	1	0.04	0.043	0.029	0.04	0.04	t	78937	AYSDDFFVGRV	Mus musculus
1	1.00	1	1	0.02	0.02	0.02	0.02	0.02	t	105850	GTAPFEIH	Oryctolagus cuniculus
1	1.00	1	1	0.06	0.06	0.06	0.063	0.06	t	149113	STPNVTQ	Sus scrofa
1	1.00	1	1	-0.02	-0.022	-0.037	-0.02	-0.018	t	58906	SKNLAPI	Mus musculus
1	1.00	1	1	0.02	0.02	0.015	0.02	0.019	t	3881	APTYSWGE	Mus musculus
1	1.00	1	1	0.01	0.007	-0.005	0.003	0.008	t	104051	LPSDPIGGKSNMHFMNTL	Oryctolagus cuniculus
1	1.00	1	1	0.01	0.012	0.005	0.011	0.01	t	66004	TRCFDS	Homo sapiens
1	1.00	1	1	-0.02	-0.016	-0.016	-0.013	-0.022	t	124061	ELERIRRSILPYGD	Homo sapiens
1	1.00	1	1	0.01	0.013	0.018	0.01	0.006	t	141053	LTPDTQYEFQVR	Mus musculus
1	1.00	1	1	-0.02	-0.017	-0.02	-0.02	-0.02	t	79215	FNPVYP	Mus musculus

1	1.00	1	1	-0.07	-0.072	-0.079	-0.067	-0.07	t	115540	VKNKRTFL SPWISNI	Mus musculu s
1	1.00	1	1	0.01	-0.002	0.005	0.01	0.01	t	65026	TLQNQGA W	Anas platyrhy nchos
1	1.00	1	1	0	0	0	0.007	-0.001	t	118060	PPPGMRP P	Mus musculu s
1	1.00	1	1	0	0.004	0	0.007	-0.001	t	117245	VTWEVLE GEVEKEAL KKI	Homo sapiens
1	1.00	1	1	0	0	0	0.007	-0.001	t	117302	GSGKGKV QFQGKKT KFASDD	Homo sapiens
1	1.00	1	1	0	0	0	0.007	-0.001	t	117300	GSGKGKV QFQGKKT KF	Homo sapiens
1	1.00	1	1	-0.02	-0.022	-0.03	-0.017	-0.02	t	52107	QQPQRPF AQQ	Mus musculu s
1	1.00	1	1	0.06	0.064	0.05	0.061	0.06	t	80885	DADIGNEY DQVV	Homo sapiens
1	1.00	1	1	-0.01	-0.012	-0.02	-0.007	-0.01	t	6073	CCRGRCN KKGEQVG M	Homo sapiens
1	1.00	1	1	0	-0.005	-0.01	0	0	t	132826	FPEDLGNK SWQAYVV	Trichosu rus vulpecul a
1	1.00	1	1	0.01	0.01	-0.006	0.012	0.012	t	98956	QGSVQPQ QLPQF	Homo sapiens
1	1.00	1	1	0.02	0.022	0.02	0.027	0.02	t	110104	WQEGNVF	Homo sapiens
1	1.00	1	1	-0.01	-0.01	-0.017	-0.01	-0.011	t	120146	QGARVYA YVF	Mus musculu s
1	1.00	1	1	-0.01	-0.012	-0.017	-0.01	-0.011	t	120146	QGARVYA YVF	Oryctola gus cuniculu s
1	1.00	1	1	0.01	0.01	0.01	0.02	0.01	t	115291	ILDKVGINY W	Homo sapiens

1	1.00	1	1	0.02	0.024	0.015	0.027	0.02	t	136087	VETDKATVGF	Homo sapiens
1	1.00	1	1	-0.02	-0.022	-0.029	-0.017	-0.02	t	137132	DINCFRPHGVSA	Sus scrofa
1	1.00	1	1	0.04	0.037	0.04	0.04	0.039	t	43575	NEDNEKLR	Oryctolagus cuniculus
1	1.00	1	1	-0.01	-0.008	-0.017	-0.003	-0.009	t	144594	ARGISRTKYW	Homo sapiens
1	1.00	1	1	-0.05	-0.052	-0.052	-0.047	-0.051	t	7559	DAKFRPN	Mus musculus
1	1.00	1	1	0.02	0.02	0.02	0.02	0.02	t	117614	QGFPFYDKPMRI	Homo sapiens
1	1.00	1	1	0.01	0.01	0.01	0.017	0.01	t	2039	AINPESDQPDLSNF	Homo sapiens
1	1.00	1	1	-0.04	-0.04	-0.04	-0.03	-0.04	t	115943	RINKKIEKFQ	Homo sapiens
1	1.00	1	1	0.01	0.014	0.007	0.011	0.008	t	34639	KYVTGIN	Homo sapiens
1	1.00	1	1	0.03	0.028	0.016	0.033	0.032	t	95611	NDPTTN	Mus musculus
1	1.00	1	1	-0.01	-0.012	-0.013	-0.011	-0.012	t	151073	YEDVNGIRK	Mus musculus
1	1.00	1	1	0.04	0.04	0.03	0.04	0.04	t	23630	HDKNNSDI	Homo sapiens
1	1.00	1	1	0.01	0.008	0.012	0.009	0.008	t	122094	FLALDVPTIGLC	Mus musculus
1	1.00	1	1	0.07	0.07	0.068	0.073	0.071	t	123731	SEESAEVATEEV	Homo sapiens

1	1.00	1	1	0.02	0.017	0.009	0.02	0.02	t	20562	GKFLEGA	Oryctolagus cuniculus
1	1.00	1	1	0.02	0.02	0.009	0.02	0.02	t	20562	GKFLEGA	Mus musculus
1	1.00	1	1	0.05	0.053	0.044	0.05	0.05	t	54504	RLDGTTLEV	Mus musculus
1	1.00	1	1	0.02	0.018	0.011	0.02	0.02	t	90069	VKASAE	Mus musculus
1	1.00	1	1	0.02	0.023	0.011	0.019	0.02	t	41143	MATAGVAAVVKRKEEN	Mus musculus
1	1.00	1	1	0.01	0.014	-0.002	0.009	0.011	t	141560	SDEALPPGSPR	Mus musculus
1	1.00	1	1	-0.03	-0.027	-0.044	-0.03	-0.029	t	94513	FHPDK	Mus musculus
1	1.00	1	1	-0.01	-0.007	-0.022	-0.007	-0.01	t	47710	PGNKPRIAEM	Capra hircus
1	1.00	1	1	0.01	0.007	0.005	0.01	0.008	t	65238	TMPFSPDFSNVD	Oryctolagus cuniculus
1	1.00	1	1	0.01	0.01	0.002	0.013	0.009	t	63165	TDHAGSNDNIALLVQ	Homo sapiens
1	1.00	1	1	0.04	0.042	0.036	0.037	0.037	t	25997	IETWNPNNQEFECAG	Homo sapiens
1	1.00	1	1	-0.04	-0.042	-0.045	-0.037	-0.04	t	108976	FPGARGPP	Rattus norvegicus
1	1.00	1	1	-0.01	-0.006	-0.012	-0.009	-0.011	t	38404	LPALSTGL	Homo sapiens
1	1.00	1	1	-0.01	-0.01	-0.017	-0.01	-0.01	t	130806	EMDVVRFDD	Homo sapiens

1	1.00	1	1	-0.04	-0.039	-0.041	-0.037	-0.041	t	99389	KMYACR	Equus caballus
1	1.00	1	1	-0.01	-0.008	-0.009	-0.003	-0.012	t	124198	GLLFGLIALAEEVRKLKAR	Homo sapiens
1	1.00	1	1	0.02	0.022	0.013	0.023	0.022	t	77842	AYIDFEMKI	Homo sapiens
1	1.00	1	1	-0.03	-0.027	-0.039	-0.03	-0.03	t	25414	IARLTDKK	Mus musculus
1	1.00	1	1	-0.03	-0.03	-0.039	-0.03	-0.03	t	25414	IARLTDKK	Mus musculus
1	1.00	1	1	0.06	0.06	0.06	0.06	0.057	t	118373	GIEADDDRLNKVISE	Homo sapiens
1	1.00	1	1	0.02	0.02	0.016	0.017	0.018	t	23562	HASARQQWEL	Homo sapiens
1	1.00	1	1	0.01	0.014	0.01	0.017	0.01	t	123522	MTQAPRIM	Homo sapiens
1	1.00	1	1	-0.01	-0.013	-0.012	-0.01	-0.013	t	14899	EVVLQNRRGLDLL	Oryctolagus cuniculus
1	1.00	1	1	-0.01	-0.01	-0.012	-0.01	-0.013	t	14899	EVVLQNRRGLDLL	Oryctolagus cuniculus
1	1.00	1	1	-0.01	-0.01	-0.012	-0.01	-0.013	t	14899	EVVLQNRRGLDLL	Oryctolagus cuniculus
1	1.00	1	1	-0.02	-0.02	-0.02	-0.013	-0.02	t	117300	GSGKGKVQFQGKKTKF	Homo sapiens
1	1.00	1	1	-0.03	-0.03	-0.04	-0.027	-0.03	t	20663	GKPVVQTILA	Homo sapiens
1	1.00	1	1	0.02	0.023	0.014	0.02	0.021	t	9591	DPAKAAFD	Mus musculus

1	1.00	1	1	0.04	0.038	0.028	0.033	0.038	t	144641	FFENKD	Mus musculus
1	1.00	1	1	0	0	-0.007	0.004	0	t	35006	LAVLLGAC	Homo sapiens
1	1.00	1	1	0.01	0.01	-0.002	0.01	0.012	t	120646	ITEHEGEIGK	Mus musculus
1	1.00	1	1	-0.02	-0.02	-0.035	-0.02	-0.02	t	52186	QRANLGAFQN	Homo sapiens
1	1.00	1	1	-0.02	-0.02	-0.018	-0.02	-0.021	t	120173	RLMRYWANFA	Mus musculus
1	1.00	1	1	-0.01	-0.01	-0.01	-0.01	-0.013	t	131100	TDSEMDVVRFD	Mus musculus
1	1.00	1	1	0.02	0.016	0.018	0.02	0.018	t	29567	IYDKLTVQTTG	Oryctolagus cuniculus
1	1.00	1	1	-0.02	-0.014	-0.023	-0.02	-0.02	t	139855	PSAFHFL	Ovis aries
1	1.00	1	1	0.01	0.01	0.01	0.01	0.01	t	135507	VVGAGP	Oryctolagus cuniculus
1	1.00	1	1	0.03	0.03	0.019	0.03	0.03	t	94621	NPACGPKYSC	Capra hircus
1	1.00	1	1	0.03	0.028	0.022	0.029	0.03	t	22783	GTPLDIDTASESS	Mus musculus
1	1.00	1	1	0.01	0.012	0.01	0.013	0.01	t	134418	KGAIIGLMVGGVV	Homo sapiens
1	1.00	1	1	0	0	0	0	-0.004	t	138194	IIPKSSWS	Homo sapiens
1	1.00	1	1	0	0	0	0	-0.004	t	99922	DAEFRHDSGY	Mus musculus

1	1.00	1	1	0	0	0	0	-0.004	t	105127	DAEFRHD	Mus musculus
1	1.00	1	1	0	0	0	0	-0.004	t	115652	SSRISIYWTIVKP	Homo sapiens
1	1.00	1	1	0	0	0	0	-0.004	t	115630	NFDKLYIWG	Homo sapiens
1	1.00	1	1	0	0	0	0	-0.004	t	99922	DAEFRHDSGY	Mus musculus
1	1.00	1	1	0.01	0.014	0.01	0.017	0.01	t	113193	TGEIKALAGCDFLTI	Homo sapiens
1	1.00	1	1	0	0.002	0	0.003	0	t	8473	DGQPAGDR	Homo sapiens
1	1.00	1	1	0	-0.004	0	0.003	-0.003	t	34965	LASLRKAFADTVPVRPPSNY	Oryctolagus cuniculus
1	1.00	1	1	0	-0.004	0	0.003	-0.003	t	28075	IPSTAFTDIAWVRLPNHY	Oryctolagus cuniculus
1	1.00	1	1	0.01	0.013	-0.001	0.01	0.009	t	123282	HACQKKLLKFEALQQEEGEE	Mus musculus
1	1.00	1	1	0.02	0.018	0.019	0.019	0.02	t	56788	SAFVFPTKD	Mus musculus
1	1.00	1	1	0.06	0.06	0.06	0.063	0.06	t	149103	SGPNPTQ	Sus scrofa
1	1.00	1	1	0	-0.004	-0.006	0.003	0	t	95092	GYQKTVDHTKVNSK	Oryctolagus cuniculus
1	1.00	1	1	-0.01	-0.012	-0.01	-0.009	-0.011	t	130454	CLFPNNSYC	Mus musculus
1	1.00	1	1	0	0	-0.005	0.006	0	t	109175	GPEGAQGP	Rattus norvegicus
1	1.00	1	1	0.03	0.032	0.023	0.037	0.031	t	136546	PFGPVINGRCCLEEKVCSLL	Homo sapiens

1	1.00	1	1	0.01	0.01	0.005	0.01	0.009	t	97331	GFLKLNIG D	Oryctolagus cuniculus
1	1.00	1	1	0.02	0.02	0.008	0.02	0.02	t	105076	AFNHFGE GLMQR	Homo sapiens
1	1.00	1	1	0.03	0.028	0.031	0.033	0.03	t	142316	TTDLYCYE QLND	Homo sapiens
1	1.00	1	1	0.04	0.042	0.046	0.047	0.037	t	2039	AINPESDQ PDLSNF	Homo sapiens
1	1.00	1	1	-0.02	-0.02	-0.02	-0.02	-0.022	t	133156	IRLR	Oryctolagus cuniculus
1	1.00	1	1	-0.01	-0.008	-0.01	-0.007	-0.011	t	11819	EENVEHD A	Homo sapiens
1	1.00	1	1	0.01	0.01	0.01	0.01	0.008	t	50929	QGPGAP	Mus musculus
1	1.00	1	1	0.05	0.048	0.056	0.053	0.05	t	67967	VDGSDP	Mus musculus
1	1.00	1	1	0.03	0.027	0.013	0.03	0.032	t	97023	VATRSQTD TSEKDC	Oryctolagus cuniculus
1	1.00	1	1	0	0	0	0	-0.004	t	151763	WYRATHQ E	Mus musculus
1	1.00	1	1	0	0	0	0	-0.004	t	143676	DRVGLRNL RGYY	Mus musculus
1	1.00	1	1	0	0	0	0	-0.004	t	140701	LNEDLRS WTA	Mus musculus
1	1.00	1	1	0	0	0	0	-0.004	t	140730	QFAYDGK DY	Mus musculus
1	1.00	1	1	0	0	0	0	-0.004	t	143687	FRVNLRTL RRYY	Mus musculus

1	1.00	1	1	0	0	0	0	-0.004	t	137866	GDTRPRFL	Oryctolagus cuniculus
1	1.00	1	1	0	0	0	0	-0.004	t	140665	DTAAQI	Mus musculus
1	1.00	1	1	0	0	0	0	-0.004	t	137962	RDSPEDFV	Oryctolagus cuniculus
1	1.00	1	1	0	0	0	0	-0.004	t	133622	KPGDPSALHVVRCWIC	Mus musculus
1	1.00	1	1	0	0	0	0	-0.004	t	122377	GLPRLETPADLSTAIASRS	Oryctolagus cuniculus
1	1.00	1	1	0	0	0	0	-0.004	t	133528	CADQKPGEC	Mus musculus
1	1.00	1	1	0	0	0	0	-0.004	t	125015	DNESSYSRGFDMTYTE	Mus musculus
1	1.00	1	1	0	0	0	0	-0.004	t	133535	CKPGEVQQC	Mus musculus
1	1.00	1	1	0	0	0	0	-0.004	t	133531	CDTLKPGEC	Mus musculus
1	1.00	1	1	0	0	0	0	-0.004	t	133554	EGLYSPWWPRSLPVL	Mus musculus
1	1.00	1	1	0	0	0	0	-0.004	t	133580	GFLSSCNFRFCELVR	Mus musculus
1	1.00	1	1	0	0	0	0	-0.004	t	133642	MCPEMSFVAVHSCAR	Mus musculus
1	1.00	1	1	0	0	0	0	-0.004	t	133542	DGRRDVVVRSATFYL	Mus musculus
1	1.00	1	1	0	0	0	0	-0.004	t	122749	EIIGFGQELKNPQEE	Oryctolagus cuniculus
1	1.00	1	1	0	0	0	0	-0.004	t	133536	CKPVENRAC	Mus musculus

1	1.00	1	1	0	0	0	0	-0.004	t	133538	CNKPGERT C	Mus musculu s
1	1.00	1	1	0	0	0	0	-0.004	t	117520	GSFSDADL AD	Mus musculu s
1	1.00	1	1	0	0	0	0	-0.004	t	122746	EEQEDEIIG FGQELKN	Oryctola gus cuniculu s
1	1.00	1	1	0	0	0	0	-0.004	t	117506	GGAHFSVS SLAEG	Mus musculu s
1	1.00	1	1	0	0	0	0	-0.004	t	125136	LGSISESRR ALQDSQR	Oryctola gus cuniculu s
1	1.00	1	1	0	0	0	0	-0.004	t	122780	GQELKNP QEETLQAF DSHYD	Oryctola gus cuniculu s
1	1.00	1	1	0	0	0	0	-0.004	t	133623	KPGEMR	Mus musculu s
1	1.00	1	1	0	0	0	0	-0.004	t	133665	PVLLFCFLA GRCVSV	Mus musculu s
1	1.00	1	1	0	0	0	0	-0.004	t	97287	ETPLTETAL KWH	Mus musculu s
1	1.00	1	1	0	0	0	0	-0.004	t	97252	DTPLTTAA LRLV	Mus musculu s
1	1.00	1	1	0	0	0	0	-0.004	t	97594	QTPLTETA LKWH	Mus musculu s
1	1.00	1	1	0	0	0	0	-0.004	t	97595	QTPLTMA ALELF	Mus musculu s
1	1.00	1	1	0	0	0	0	-0.004	t	57542	SELSQLS	Mus musculu s
1	1.00	1	1	0	0	0	0	-0.004	t	38522	LPHSNL	Rattus norvegic us
1	1.00	1	1	0	0	0	0	-0.004	t	57542	SELSQLS	Mus musculu s

1	1.00	1	1	0	0	0	0	-0.004	t	30173	KDILEDERAAVDTYC	Oryctolagus cuniculus
1	1.00	1	1	0	0	0	0	-0.004	t	30200	KDLLEQKRAAVDTYC	Oryctolagus cuniculus
1	1.00	1	1	0	0	0	0	-0.004	t	28589	ISLNFENYV	Rattus norvegicus
1	1.00	1	1	0	0	0	0	-0.004	t	10433	DTNNN	Mus musculus
1	1.00	1	1	0	0	0	0	-0.004	t	10908	DYKDDDDK	Mus musculus
1	1.00	1	1	0	-0.003	0	0	-0.004	t	23975	HHAEMQNPVYLIPETVPYIK	Oryctolagus cuniculus
1	1.00	1	1	0	0	0	0	-0.004	t	4429	ASGQ	Mus musculus
1	1.00	1	1	0.01	0.01	0.007	0.013	0.01	t	78839	SRNLGKVIDTLTCGFA	Homo sapiens
1	1.00	1	1	0.04	0.042	0.041	0.047	0.04	t	133822	ERPLKQIAFNGGLEP	Homo sapiens
1	1.00	1	1	0.04	0.042	0.038	0.047	0.041	t	125201	PPQTEHIEL	Homo sapiens
1	1.00	1	1	0.02	0.02	0.01	0.02	0.02	t	10496	DTTIP	Mus musculus
1	1.00	1	1	0.02	0.02	0.02	0.026	0.02	t	41948	MLGNAPSVVPNTTLGM	Homo sapiens
1	1.00	1	1	0.02	0.023	0.014	0.02	0.02	t	84184	KASAEG	Mus musculus

1	1.00	1	1	0.02	0.023	0.014	0.02	0.02	t	84184	KASAEG	Mus musculus
1	1.00	1	1	0	0	0	0	-0.001	t	9611	DPDKDVDIMV	Mus musculus
1	1.00	1	1	0.02	0.024	0.02	0.021	0.02	t	48778	PPFSLSPVPTL	Homo sapiens
1	1.00	1	1	0.01	0.01	-0.004	0.01	0.01	t	133952	WIFQNF	Mus musculus
1	1.00	1	1	-0.01	-0.014	-0.015	-0.01	-0.009	t	41665	MHLPATIGDY	Mus musculus
1	1.00	1	1	0	0	0.002	0	-0.005	t	25140	HWSYGLRPG	Mus musculus
1	1.00	1	1	-0.03	-0.032	-0.024	-0.029	-0.032	t	79042	PKSKPFA	Mus musculus
1	1.00	1	1	0.03	0.031	0.029	0.033	0.029	t	99279	GHPQEI	Equus caballus
1	1.00	1	1	-0.04	-0.043	-0.048	-0.04	-0.042	t	111052	VKSEKDSTNNNKYTVTLK	Oryctolagus cuniculus
1	1.00	1	1	-0.04	-0.043	-0.048	-0.04	-0.042	t	111052	VKSEKDSTNNNKYTVTLK	Oryctolagus cuniculus
1	1.00	1	1	0.03	0.026	0.022	0.029	0.03	t	39708	LSTLSYLG	Oryctolagus cuniculus
1	1.00	1	1	0.08	0.078	0.068	0.073	0.08	t	134794	SEATVAESDSF	Mus musculus
1	1.00	1	1	-0.01	-0.013	-0.017	-0.01	-0.011	t	55263	RPPPAPCL	Oryctolagus cuniculus

1	1.00	1	1	0.09	0.092	0.083	0.097	0.091	t	136499	LVNCDRDNLESSAMDCEDDE	Homo sapiens
1	1.00	1	1	0.03	0.032	0.023	0.033	0.029	t	130608	SIDLKDKYKNIGA	Homo sapiens
1	1.00	1	1	-0.03	-0.026	-0.045	-0.03	-0.029	t	136080	RAGTDEGFG	Homo sapiens
1	1.00	1	1	-0.02	-0.02	-0.023	-0.01	-0.02	t	98869	LEKFDKALKA	Homo sapiens
1	1.00	1	1	-0.01	-0.007	-0.017	-0.01	-0.008	t	88382	SATAIF	Mus musculus
1	1.00	1	1	0.01	0.014	0.01	0.013	0.008	t	136081	RTTLQSDQEEIDY	Homo sapiens
1	1.00	1	1	0.04	0.04	0.04	0.04	0.04	t	107702	YVDGVQV	Homo sapiens
1	1.00	1	1	0.04	0.036	0.033	0.043	0.04	t	71548	VTVDDVHKACERFEQ	Oryctolagus cuniculus
1	1.00	1	1	-0.01	-0.01	-0.01	-0.01	-0.013	t	122465	LKRDMKSAHLLPEH	Homo sapiens
1	1.00	1	1	-0.01	-0.01	-0.009	-0.004	-0.011	t	55581	RRGPRLGVRATRKTS	Homo sapiens
1	1.00	1	1	-0.01	-0.01	-0.009	-0.004	-0.011	t	55581	RRGPRLGVRATRKTS	Homo sapiens
1	1.00	1	1	0.03	0.03	0.019	0.027	0.03	t	25538	IDANDCLVTPSVK	Homo sapiens
1	1.00	1	1	0.03	0.032	0.027	0.03	0.03	t	48990	PQEIPQPQ	Anas platyrhynchos
1	1.00	1	1	0	0.003	0	0	0	t	131772	YPGAVTV	Mus musculus

1	1.00	1	1	0.02	0.017	0.013	0.02	0.019	t	138081	VTENTNGLKTML	Oryctolagus cuniculus
1	1.00	1	1	0.02	0.006	0.013	0.02	0.019	t	138081	VTENTNGLKTML	Oryctolagus cuniculus
1	1.00	1	1	0.02	0.02	0.009	0.02	0.02	t	94472	CNPACGPKY	Capra hircus
1	1.00	1	1	0.02	0.02	0.009	0.02	0.02	t	94472	CNPACGPKY	Oryctolagus cuniculus
1	1.00	1	1	0.02	0.027	0.009	0.02	0.02	t	94472	CNPACGPKY	Capra hircus
1	1.00	1	1	-0.01	-0.01	-0.012	-0.01	-0.01	t	40123	LTPRPIIHAV	Homo sapiens
1	1.00	1	1	0.02	0.02	0.02	0.02	0.018	t	118783	EIIVTHFPFDEQNCSMK	Rattus norvegicus
1	1.00	1	1	0.03	0.028	0.021	0.029	0.029	t	61007	SSFSSYGEI	Mus musculus
1	1.00	1	1	0.02	0.018	0.013	0.023	0.02	t	125565	STDDYASFSRAL	Mus musculus
1	1.00	1	1	-0.01	-0.01	-0.025	-0.01	-0.01	t	65252	TMQYVDMI	Homo sapiens
1	1.00	1	1	0.01	0.006	-0.007	0.007	0.011	t	117451	DDGFFYIPKD	Oryctolagus cuniculus
1	1.00	1	1	0.02	0.016	0.012	0.019	0.02	t	57292	SDSSFFSYGEIPFG	Oryctolagus cuniculus
1	1.00	1	1	0.01	0.008	-0.001	0.007	0.01	t	47969	PIQPQQPFPQ	Mus musculus
1	1.00	1	1	-0.01	-0.008	-0.01	-0.003	-0.01	t	119678	YKCKVSNK	Homo sapiens

1	1.00	1	1	-0.01	-0.006	-0.01	-0.003	-0.01	t	119678	YKCKVSNK	Homo sapiens
1	1.00	1	1	0.06	0.06	0.06	0.06	0.06	t	104771	FA	Mus musculus
1	1.00	1	1	0.03	0.03	0.022	0.03	0.03	t	57766	SFFSYGEI	Mus musculus
1	1.00	1	1	0	0.004	-0.01	0.001	0	t	155744	SAHSTPS	Homo sapiens
1	1.00	1	1	0.05	0.046	0.048	0.053	0.052	t	99737	TVKVGD	Oryctolagus cuniculus
1	1.00	1	1	0.01	0.012	0.003	0.017	0.01	t	133787	CQALVRMLAKKPGWK	Homo sapiens
1	1.00	1	1	0	-0.002	-0.011	-0.001	0	t	26328	IGTLKKILDETVKDKLAK	Mus musculus
1	1.00	1	1	0	0	0	0	-0.001	t	13525	ENMYRYPNQVYY	Mus musculus
1	1.00	1	1	0.02	0.006	0.003	0.017	0.021	t	115640	QKSQRSQDEN	Macaca mulatta
1	1.00	1	1	0.02	0.016	0.003	0.017	0.021	t	115640	QKSQRSQDEN	Oryctolagus cuniculus
1	1.00	1	1	0.01	0.012	0.008	0.017	0.01	t	126201	GYKEKSKFQDTHNNA	Homo sapiens
1	1.00	1	1	0.04	0.042	0.033	0.041	0.04	t	61157	SSLGNGV	Homo sapiens
1	1.00	1	1	0	0.014	0.005	0.007	0	t	108921	EPQVYTL	Homo sapiens
1	1.00	1	1	0.03	0.03	0.022	0.027	0.032	t	623	ADALLEKG	Homo sapiens
1	1.00	1	1	-0.01	-0.009	-0.011	-0.009	-0.012	t	73770	YETGWGCNPPDC	Myodes glareolus

1	1.00	1	1	-0.02	-0.02	-0.02	-0.017	-0.02	t	25094	HVSPTHYVP	Homo sapiens
1	1.00	1	1	0.04	0.04	0.033	0.041	0.04	t	49214	PRLLYDEL	Homo sapiens
1	1.00	1	1	0.06	0.06	0.056	0.06	0.06	t	11927	EEVVGEEKLVSEEIVT	Mus musculus
1	1.00	1	1	-0.02	-0.02	-0.015	-0.02	-0.022	t	108227	YIHEGLK	Mus musculus
1	1.00	1	1	-0.02	-0.02	-0.02	-0.02	-0.024	t	28589	ISLNFENYV	Rattus norvegicus
1	1.00	1	1	0.05	0.05	0.045	0.054	0.05	t	133776	AGDLSKVDAKQPGDY	Homo sapiens
1	1.00	1	1	-0.01	-0.006	-0.01	-0.003	-0.01	t	136070	KTNLIVNYLP	Homo sapiens
1	1.00	1	1	-0.02	-0.018	-0.019	-0.014	-0.02	t	109751	QGPRGEPG	Rattus norvegicus
1	1.00	1	1	0.03	0.03	0.03	0.029	0.027	t	9585	DPAF	Mus musculus
1	1.00	1	1	0	0.002	0	0.007	-0.001	t	108104	RRLPFGSQM	Homo sapiens
1	1.00	1	1	0.03	0.033	0.033	0.03	0.03	t	49878	PVGTPE	Mus musculus
1	1.00	1	1	0.03	0.03	0.033	0.03	0.03	t	49878	PVGTPE	Mus musculus
1	1.00	1	1	0.05	0.05	0.048	0.05	0.051	t	10320	DTDSGYDVPDY	Mus musculus
1	1.00	1	1	0.03	0.026	0.03	0.023	0.03	t	107784	DGSFFLY	Oryctolagus cuniculus
1	1.00	1	1	0.02	0.015	0.016	0.019	0.02	t	71568	VTVVTTSGS	Bos taurus

1	1.00	1	1	0	-0.004	-0.006	0.003	0	t	95087	GPIKNKMS ESPNKT	Oryctola gus cuniculu s
1	1.00	1	1	0.05	0.048	0.041	0.05	0.05	t	100635	VTNYLVDG NG	Mus musculu s
1	1.00	1	1	-0.03	-0.025	-0.037	-0.029	-0.03	t	72236	WAKHMW NFI	Homo sapiens
1	1.00	1	1	0	0	-0.007	0	-0.001	t	39980	LTIVSGGP TLFS	Mus musculu s
1	1.00	1	1	0	0	-0.007	0	-0.001	t	39980	LTIVSGGP TLFS	Mus musculu s
1	1.00	1	1	0.03	0.027	0.019	0.033	0.031	t	142141	EIDGPAGQ AEPD	Homo sapiens
1	1.00	1	1	0	0.002	-0.001	-0.003	-0.002	t	102271	YYNSLGFN IK	Homo sapiens
1	1.00	1	1	-0.01	-0.01	-0.024	-0.01	-0.01	t	78424	IPRSRQIDL QIG	Mus musculu s
1	1.00	1	1	0.05	0.048	0.043	0.05	0.05	t	50872	QGGNIVDI DFDSVP	Oryctola gus cuniculu s
1	1.00	1	1	0.01	0.006	0.003	0.01	0.008	t	153904	VVEQLGH ETQIHIQIP	Mus musculu s
1	1.00	1	1	0.03	0.028	0.032	0.031	0.028	t	70946	VSGDPRG RYEDI	Mus musculu s
1	1.00	1	1	0.03	0.028	0.027	0.034	0.03	t	120167	RFQSSAV MALQEAC EAYL	Mus musculu s
1	1.00	1	1	-0.01	-0.011	-0.022	-0.009	-0.01	t	1022	AEKQKAAE ATKVAE	Homo sapiens
1	1.00	1	1	-0.01	-0.01	-0.01	-0.004	-0.01	t	118384	GMKFDRG YISPYF	Homo sapiens

1	1.00	1	1	0.03	0.03	0.012	0.024	0.03	t	49046	PQPELPYP	Homo sapiens
1	1.00	1	1	0.04	0.037	0.036	0.04	0.04	t	94484	DDYRQK	Oryctolagus cuniculus
1	1.00	1	1	-0.01	-0.008	-0.014	-0.007	-0.01	t	60752	SRPGLKPDLTGTSKS	Homo sapiens
1	1.00	1	1	0.07	0.07	0.07	0.077	0.07	t	122746	EEQEDEIIGFGQELKN	Homo sapiens
1	1.00	1	1	0.02	0.019	0.012	0.019	0.02	t	57771	SFFVKDWSERIREF	Myodes glareolus
1	1.00	1	1	0.02	0.019	0.012	0.019	0.02	t	57771	SFFVKDWSERIREF	Myodes glareolus
1	1.00	1	1	0.01	0.01	0.001	0.01	0.009	t	56432	RVTTRSQAQDAAGLKEKAD	Mus musculus
1	1.00	1	1	0.01	0.014	0.002	0.01	0.01	t	140594	RELVLAGTI	Cavia porcellus
1	1.00	1	1	-0.01	-0.01	-0.02	-0.01	-0.01	t	25627	IDLTNKNAAKLRGD	Homo sapiens
1	1.00	1	1	-0.02	-0.016	-0.02	-0.013	-0.018	t	131686	SYLSLTP	Homo sapiens
1	1.00	1	1	0.07	0.07	0.062	0.07	0.07	t	106378	EEVKTTEQKR	Homo sapiens
1	1.00	1	1	-0.02	-0.02	-0.02	-0.02	-0.023	t	64552	TKEPGVLMGGQSESV	Mus musculus
1	1.00	1	1	-0.01	-0.01	-0.01	-0.001	-0.008	t	131041	RLVGQQVMQ	Homo sapiens
1	1.00	1	1	-0.03	-0.03	-0.035	-0.03	-0.032	t	55232	RPMIHF	Mus musculus

1	1.00	1	1	0.03	0.032	0.028	0.037	0.029	t	51852	QPHDLGKVGEVIVTKD	Homo sapiens
1	1.00	1	1	-0.02	-0.022	-0.033	-0.021	-0.021	t	144798	TKYTMM	Mus musculus
1	1.00	1	1	0.01	0.008	-0.005	0.009	0.01	t	71163	VSTQSAKNPPGATVP	Mus musculus
1	1.00	1	1	0.04	0.04	0.04	0.044	0.04	t	135975	PTLAFLQDVMNI	Homo sapiens
1	1.00	1	1	0.03	0.032	0.03	0.037	0.03	t	131769	YDSYAIA	Homo sapiens
1	1.00	1	1	-0.03	-0.03	-0.03	-0.03	-0.03	t	78504	RLFLHV	Mus musculus
1	1.00	1	1	0	0	0	0.006	-0.001	t	20064	GGVATY	Homo sapiens
1	1.00	1	1	0.05	0.054	0.039	0.047	0.05	t	78104	APPASEQET	Homo sapiens
1	1.00	1	1	-0.01	-0.013	-0.017	-0.01	-0.011	t	55263	RPPPAPCL	Oryctolagus cuniculus
1	1.00	1	1	0.01	0.005	0	0.01	0.011	t	127847	SYNSTADEWLPV	Trichosurus vulpecula
1	1.00	1	1	0	0	-0.019	0	0.002	t	7756	DDDMGFGLFD	Homo sapiens
1	1.00	1	1	-0.02	-0.018	-0.026	-0.019	-0.022	t	141580	YIGVAPRKAIPA	Homo sapiens
1	1.00	1	1	0.08	0.08	0.073	0.08	0.08	t	105803	GAE	Mus musculus
1	1.00	1	1	-0.01	-0.013	-0.014	-0.01	-0.01	t	141953	LTACKV	Oryctolagus cuniculus
1	1.00	1	1	0.01	0.012	0.008	0.017	0.011	t	132683	SRYDERPGPS	Homo sapiens

1	1.00	1	1	0.01	0.008	0.008	0.013	0.009	t	1199	AETRLNPDLQ	Mus musculus
1	1.00	1	1	-0.01	-0.01	-0.02	-0.01	-0.01	t	104161	NVVLKTITESATSPPT	Oryctolagus cuniculus
1	1.00	1	1	0.04	0.04	0.033	0.039	0.039	t	137867	GEIKFEAVEGAV	Homo sapiens
1	1.00	1	1	0	-0.012	0.002	0	0	t	40755	LYFP	Mus musculus
1	1.00	1	1	0.03	0.032	0.029	0.027	0.028	t	102265	YSCGENSFMD	Homo sapiens
1	1.00	1	1	-0.03	-0.026	-0.035	-0.033	-0.033	t	116029	YAKPAAVRSP	Homo sapiens
1	1.00	1	1	-0.03	-0.032	-0.035	-0.033	-0.033	t	116029	YAKPAAVRSP	Homo sapiens
1	1.00	1	1	0.03	0.028	0.01	0.023	0.03	t	127933	APDDSFKKLEDRFTNLKF	Mus musculus
1	1.00	1	1	0.05	0.05	0.047	0.047	0.05	t	61997	STVTEN	Homo sapiens
1	1.00	1	1	0.01	0.01	0.016	0.01	0.01	t	17271	FPDLGV	Homo sapiens
1	1.00	1	1	0.01	0.012	0.01	0.011	0.01	t	98938	PQP	Homo sapiens
1	1.00	1	1	0	-0.002	-0.007	-0.003	-0.002	t	33244	KRVVIN	Mus musculus
1	1.00	1	1	0.02	0.021	0.019	0.023	0.019	t	99279	GHPQEI	Equus caballus
1	1.00	1	1	-0.03	-0.037	-0.037	-0.023	-0.028	t	156885	GYRAR	Homo sapiens
1	1.00	1	1	0.05	0.05	0.055	0.06	0.053	t	115912	QAPEY	Homo sapiens
1	1.00	1	1	-0.01	-0.01	-0.01	-0.01	-0.012	t	133379	SRPYMA	Homo sapiens

1	1.00	1	1	0.05	0.048	0.04	0.047	0.05	t	52572	QTMEEINT	Anas platyrhynchos
1	1.00	1	1	0.03	0.03	0.03	0.03	0.028	t	7673	DAVTE	Mus musculus
1	1.00	1	1	0.04	0.036	0.04	0.033	0.038	t	122872	PLTLEL	Oryctolagus cuniculus
1	1.00	1	1	0.04	0.04	0.04	0.044	0.04	t	135893	AFLQDVMNILLQ	Homo sapiens
1	1.00	1	1	0.02	0.023	0.015	0.02	0.02	t	114224	PQDENPVVHF	Mus musculus
1	1.00	1	1	0.01	0.01	0.011	0.01	0.008	t	49	AAALPGKCGV	Homo sapiens
1	1.00	1	1	0.02	0.024	0.028	0.027	0.02	t	124205	GPLQLGM	Homo sapiens
1	1.00	1	1	0	0.004	0.009	0.007	-0.001	t	119452	FRKTSKGGS	Homo sapiens
1	1.00	1	1	0.02	0.022	0.013	0.023	0.02	t	144682	HLVGSALSDP	Homo sapiens
1	1.00	1	1	0.01	0.01	0.005	0.01	0.01	t	67278	TWLVNASNVD	Homo sapiens
1	1.00	1	1	-0.04	-0.035	-0.054	-0.037	-0.04	t	39196	LRRLRTACERAKRTL	Homo sapiens
1	1.00	1	1	-0.05	-0.05	-0.055	-0.05	-0.05	t	129244	LINRAKAVK	Mus musculus
1	1.00	1	1	0.04	0.04	0.044	0.04	0.038	t	102279	AAANQT	Mus musculus
1	1.00	1	1	-0.04	-0.04	-0.047	-0.03	-0.037	t	113577	KNIVTPRTPPPSQGKG	Homo sapiens
1	1.00	1	1	0.01	0.008	0.012	0.009	0.01	t	1666	AGRMIPKG	Anas platyrhynchos

1	1.00	1	1	-0.01	-0.008	-0.021	-0.007	-0.01	t	110602	GLKAKPKFTLGKRKATPTT	Homo sapiens
1	1.00	1	1	-0.02	-0.02	-0.02	-0.02	-0.021	t	122033	WKKKLGASP	Mus musculus
1	1.00	1	1	0.04	0.04	0.035	0.037	0.04	t	9822	DQAMEDIKQM	Homo sapiens
1	1.00	1	1	0.03	0.028	0.024	0.027	0.027	t	151711	RATHQE	Mus musculus
1	1.00	1	1	0.01	0.01	0.01	0.01	0.006	t	42757	MTDVIRYRYSNNYEAESHIS	Aotus nancymaae
1	1.00	1	1	0	-0.004	-0.009	0	0	t	43545	NDYCLRECKQQGYKG	Mus musculus
1	1.00	1	1	0.07	0.07	0.063	0.07	0.07	t	105802	GAD	Mus musculus
1	1.00	1	1	0.03	0.034	0.023	0.033	0.03	t	124186	GFTPGGGGS	Homo sapiens
1	1.00	1	1	0.02	0.025	0.013	0.023	0.02	t	48548	PMEKKVIVWGAETA	Homo sapiens
1	1.00	1	1	0	0	0	0	-0.004	t	97771	WYGYHHSN	Mus musculus
1	1.00	1	1	0.04	0.04	0.04	0.04	0.039	t	99029	TPGQFEDFFP	Homo sapiens
1	1.00	1	1	0	-0.002	0.008	-0.001	-0.006	t	30986	KGIYPLSYE	Mus musculus
1	1.00	1	1	0.05	0.055	0.032	0.05	0.052	t	78603	AELGEYEKLLNSVLEPI	Canis familiaris
1	1.00	1	1	-0.03	-0.033	-0.043	-0.03	-0.03	t	55640	RRLGAALLQS	Oryctolagus cuniculus
1	1.00	1	1	0	0	0	0	-0.004	t	14435	ETIYNTTLKY	Homo sapiens

1	1.00	1	1	0.01	0.012	0.004	0.01	0.01	t	39254	LRYLA	Homo sapiens
1	1.00	1	1	0	-0.002	-0.01	0.003	-0.001	t	58036	SGFSETVTIVEDTRP	Mus musculus
1	1.00	1	1	0.04	0.041	0.028	0.04	0.04	t	12551	EIPQPQWTPEEDQK	Oryctolagus cuniculus
1	1.00	1	1	0.04	0.039	0.039	0.041	0.04	t	66316	TSLSVS	Homo sapiens
1	1.00	1	1	0.01	0.006	0.007	0.01	0.01	t	100249	LDPLSEKGLTTY	Oryctolagus cuniculus
1	1.00	1	1	0.01	0.01	-0.006	0.013	0.012	t	57910	SFSSIPNG	Homo sapiens
1	1.00	1	1	0.01	0.012	0.01	0.011	0.009	t	252	AAKLQD	Homo sapiens
1	1.00	1	1	0.01	0.01	-0.004	0.01	0.01	t	133952	WIFQNF	Mus musculus
1	1.00	1	1	0	0	0	0.006	0	t	112841	SSKRGRQTPVLYAMLDH	Rattus norvegicus
1	1.00	1	1	-0.02	-0.022	-0.027	-0.02	-0.018	t	20674	GKREMVIITF	Mus musculus
1	1.00	1	1	-0.02	-0.018	-0.025	-0.019	-0.018	t	25328	IAKTTLLKI	Homo sapiens
1	1.00	1	1	0.03	0.028	0.027	0.033	0.029	t	67163	TVSRPVTPLSEPIPV	Sus scrofa
1	1.00	1	1	0	-0.003	-0.014	0	0	t	21715	GPNNSEAIPALTAAE	Rattus norvegicus
1	1.00	1	1	0.01	0.012	0.003	0.011	0.009	t	99009	SNALSKRL	Homo sapiens

1	1.00	1	1	-0.01	-0.01	-0.02	-0.01	-0.01	t	52720	QVKARNII SPVMG	Mus
1	1.00	1	1	0.03	0.036	0.037	0.037	0.03	t	127650	FEPFSNKG	Homo sapiens
1	1.00	1	1	-0.01	-0.006	-0.017	-0.01	-0.01	t	48827	PPLPHV	Cavia porcellu s
1	1.00	1	1	-0.01	-0.013	-0.02	-0.01	-0.01	t	140836	DEAAYSKN RRAV	Oryctola gus cuniculu s
1	1.00	1	1	0.03	0.034	0.03	0.037	0.029	t	134209	RDLASGLI GPLLICYKE SVD	Homo sapiens
1	1.00	1	1	-0.02	-0.018	-0.02	-0.017	-0.021	t	40724	LYCGGPM FNSKGAQ	Homo sapiens
1	1.00	1	1	0.01	0.008	0.002	0.007	0.01	t	22656	GTGFSLSD LP	Mus musculu s
1	1.00	1	1	0.01	0.012	0.002	0.007	0.008	t	13252	ELRNLPQQ CGLRAPQ	Homo sapiens
1	1.00	1	1	0.01	0.012	0.002	0.007	0.008	t	13252	ELRNLPQQ CGLRAPQ	Homo sapiens
1	1.00	1	1	-0.01	-0.012	-0.017	-0.01	-0.011	t	120146	QGARVYA YVF	Oryctola gus cuniculu s
1	1.00	1	1	0	0	0	0	-0.004	t	28299	IRGDTFAT R	Mus musculu s
1	1.00	1	1	0	0	0	0	-0.004	t	7492	DAEFRHDS GYEVHHQ KL	Mus musculu s
1	1.00	1	1	0	0	0	0	-0.004	t	7492	DAEFRHDS GYEVHHQ KL	Mus musculu s
1	1.00	1	1	-0.05	-0.048	-0.057	-0.043	-0.049	t	106114	SLNPERMF RKP	Homo sapiens
1	1.00	1	1	0	-0.003	-0.013	0	0	t	105874	IEADVNTF	Oryctola gus cuniculu s

1	1.00	1	1	0	0	-0.002	0.006	0	t	116096	GFGYGGRASDYKSAHK	Rattus norvegicus
1	1.00	1	1	0	0	-0.007	0	-0.001	t	105763	EVHHQK	Mus musculus
1	1.00	1	1	0.03	0.028	0.025	0.031	0.031	t	28413	IRVEES	Homo sapiens
1	1.00	1	1	0.02	0.018	0.008	0.019	0.02	t	72690	WKNAYFDK	Mus musculus
1	1.00	1	1	0.01	0.012	0.01	0.017	0.01	t	96455	KGKNVLINKD	Homo sapiens
1	1.00	1	1	0	0	0	0	-0.004	t	151813	CGADSYEMEEDGVRKC	Mus musculus
1	1.00	1	1	0	0	0	0	-0.004	t	151731	SAQSQRAPDR	Mus musculus
1	1.00	1	1	-0.02	-0.02	-0.035	-0.02	-0.02	t	64454	TISADSKKTKDLVFL	Mus musculus
1	1.00	1	1	0.04	0.036	0.033	0.039	0.041	t	58728	SITTPSQ	Oryctolagus cuniculus
1	1.00	1	1	-0.02	-0.02	-0.027	-0.02	-0.017	t	96548	LFLLAAH	Mus musculus
1	1.00	1	1	-0.03	-0.029	-0.031	-0.027	-0.031	t	99172	ECRNAM	Equus caballus
1	1.00	1	1	0.04	0.036	0.033	0.043	0.04	t	51868	QPLEQIKLSES	Oryctolagus cuniculus
1	1.00	1	1	-0.01	-0.005	-0.024	-0.013	-0.01	t	95498	KIPAVF	Homo sapiens
1	1.00	1	1	0.02	0.024	0.02	0.021	0.02	t	48778	PPFSLSPVPTL	Homo sapiens

1	1.00	1	1	0	0.002	0	0.007	0	t	108302	CNFASRNDYSYWLSTPEPMP	Homo sapiens
1	1.00	1	1	0	0	0.001	0	-0.004	t	145746	DPEIVMHSF	Mus musculus
1	1.00	1	1	0	-0.002	0	0.005	-0.002	t	40110	LTPLEELY	Mus musculus
1	1.00	1	1	0.01	0.008	0.01	0.009	0.01	t	69860	VLYEAFDE	Mus musculus
1	1.00	1	1	0.01	0.008	0.01	0.009	0.01	t	69860	VLYEAFDE	Mus musculus
1	1.00	1	1	0	0	-0.002	0	0	t	17504	FQNEKWDL	Mus musculus
1	1.00	1	1	-0.01	-0.01	-0.017	-0.01	-0.01	t	130806	EMDVVRFDD	Homo sapiens
1	1.00	1	1	0.02	0.016	0.015	0.023	0.022	t	8800	DITKD	Oryctolagus cuniculus
1	1.00	1	1	0.02	0.022	0.013	0.017	0.018	t	106595	LDGETKTGPQRV	Homo sapiens
1	1.00	1	1	-0.04	-0.037	-0.046	-0.04	-0.04	t	65429	TNSKRK	Mus musculus
1	1.00	1	1	-0.04	-0.037	-0.046	-0.04	-0.04	t	65429	TNSKRK	Mus musculus
1	1.00	1	1	0.01	0.006	0.001	0.009	0.01	t	801	ADYHRVMYAS	Oryctolagus cuniculus
1	1.00	1	1	0.05	0.048	0.033	0.047	0.051	t	78148	ENQANPTTAET	Mus musculus
1	1.00	1	1	0.01	0.014	0.01	0.007	0.007	t	50823	QFYQKFPQYL	Homo sapiens

1	1.00	1	1	0.02	0.022	0.013	0.021	0.02	t	98340	PNPATAAGKI	Homo sapiens
1	1.00	1	1	-0.02	-0.023	-0.022	-0.02	-0.021	t	95034	PKPAKSP	Oryctolagus cuniculus
1	1.00	1	1	-0.02	-0.023	-0.022	-0.02	-0.021	t	95034	PKPAKSP	Oryctolagus cuniculus
1	1.00	1	1	-0.02	-0.02	-0.022	-0.02	-0.021	t	95034	PKPAKSP	Mus musculus
1	1.00	1	1	-0.02	-0.02	-0.022	-0.02	-0.021	t	95034	PKPAKSP	Mus musculus
1	1.00	1	1	0.04	0.041	0.039	0.043	0.039	t	99158	DRLLSQIKSEGHPQE	Equus caballus
1	1.00	1	1	0	0	0	0	-0.003	t	119272	MGPPIGLPPAR	Homo sapiens
1	1.00	1	1	-0.01	-0.008	-0.017	-0.003	-0.009	t	144594	ARGISRTKYW	Homo sapiens
1	1.00	1	1	0.02	0.022	0.02	0.027	0.02	t	103960	GTYFLRS	Homo sapiens
1	1.00	1	1	0	0	0	0	-0.004	t	39183	LRRDLDASREAKKQVEKALE	Homo sapiens
1	1.00	1	1	0.01	0.012	0.01	0.011	0.01	t	36071	LGDDTGIHVI	Homo sapiens
1	1.00	1	1	0.01	0.008	0.01	0.011	0.01	t	36071	LGDDTGIHVI	Homo sapiens
1	1.00	1	1	-0.01	-0.01	-0.01	-0.009	-0.013	t	105163	EFRHD	Mus musculus
1	1.00	1	1	0.01	0.006	0.012	0.01	0.008	t	100034	FFAEDVGSNK	Oryctolagus cuniculus
1	1.00	1	1	-0.03	-0.028	-0.023	-0.023	-0.032	t	113371	EPARSRSR	Homo sapiens

1	1.00	1	1	-0.02	-0.023	-0.033	-0.02	-0.02	t	105889	IVGFFSKI	Oryctolagus cuniculus
1	1.00	1	1	0.05	0.048	0.056	0.049	0.048	t	67967	VDGSDP	Mus musculus
1	1.00	1	1	0.02	0.014	0.008	0.02	0.02	t	57120	SCNATQYNQLST	Oryctolagus cuniculus
1	1.00	1	1	0.02	0.027	0.009	0.02	0.02	t	94452	CCNPACGPKY	Capra hircus
1	1.00	1	1	0.02	0.02	0.009	0.02	0.02	t	94452	CCNPACGPKY	Capra hircus
1	1.00	1	1	0.03	0.022	0.03	0.03	0.029	t	125055	FVYGGCQGNN	Mus musculus
1	1.00	1	1	0	-0.012	-0.016	0	0.002	t	66873	TTTSSHPV	Macaca mulatta
1	1.00	1	1	0.03	0.03	0.025	0.036	0.03	t	13986	EREVSVPA	Homo sapiens
1	1.00	1	1	0.06	0.062	0.057	0.057	0.058	t	114859	ICGTDGVTYT	Homo sapiens
1	1.00	1	1	0.07	0.066	0.066	0.069	0.07	t	65840	TQETGDTTPSAYSMS	Oryctolagus cuniculus
1	1.00	1	1	0.03	0.03	0.03	0.032	0.03	t	60364	SQAEAALENL	Homo sapiens
1	1.00	1	1	0.04	0.042	0.045	0.047	0.04	t	109530	LTKNQVS	Homo sapiens
1	1.00	1	1	-0.04	-0.043	-0.052	-0.04	-0.041	t	111051	VGPLEVIPEAVDPAGSSIV	Oryctolagus cuniculus
1	1.00	1	1	0	0.004	0	0.007	0	t	123610	PPGAMPPQ	Homo sapiens

1	1.00	1	1	0.06	0.062	0.05	0.061	0.061	t	34154	KVPEDTLN	Homo sapiens
1	1.00	1	1	-0.03	-0.026	-0.037	-0.027	-0.031	t	115591	APEYRGRT ELLK	Homo sapiens
1	1.00	1	1	0	0	-0.007	0	-0.001	t	133664	PPPPPPPP PP	Mus musculu s
1	1.00	1	1	0.03	0.03	0.015	0.033	0.032	t	76307	YVKELPTE VQKLKEK	Homo sapiens
1	1.00	1	1	-0.03	-0.031	-0.038	-0.03	-0.03	t	50836	QGAWPA GA	Anas platyrhy nchos
1	1.00	1	1	-0.03	-0.028	-0.034	-0.029	-0.03	t	53883	RGIDKSVK SV	Homo sapiens
1	1.00	1	1	-0.02	-0.024	-0.017	-0.02	-0.022	t	41544	MFTKYNG NLNTI	Oryctola gus cuniculu s
1	1.00	1	1	0.02	0.022	0.015	0.021	0.02	t	56079	RTIGGSQA QT	Homo sapiens
1	1.00	1	1	0.04	0.038	0.033	0.041	0.041	t	131217	EDISAYLL	Mus musculu s
1	1.00	1	1	-0.03	-0.03	-0.037	-0.027	-0.03	t	96071	AGIRPKGA FQ	Homo sapiens(human)
1	1.00	1	1	-0.01	-0.014	-0.014	-0.007	-0.011	t	63195	TDLLAGGK DDVK	Oryctola gus cuniculu s
1	1.00	1	1	0	-0.003	-0.01	0	0	t	140936	YLAGKGV DAGKL	Oryctola gus cuniculu s
1	1.00	1	1	0.03	0.033	0.029	0.03	0.027	t	133611	IVSYLCDLA PEAPPPTL PP	Mus musculu s
1	1.00	1	1	0	0.002	0.005	0.007	0	t	112408	YGIGVGW	Homo sapiens

1	1.00	1	1	0.04	0.042	0.043	0.041	0.04	t	61485	SSYGFQ	Homo sapiens
1	1.00	1	1	-0.02	-0.016	-0.02	-0.019	-0.022	t	96102	APPLLPH	Homo sapiens
1	1.00	1	1	0.02	0.02	0.026	0.02	0.018	t	46443	NVPEKQT	Mus musculus
1	1.00	1	1	0.02	0.02	0.026	0.02	0.018	t	46443	NVPEKQT	Mus musculus
1	1.00	1	1	0.04	0.04	0.037	0.04	0.037	t	114559	RAESEGFEWVAFKTN	Homo sapiens
1	1.00	1	1	-0.03	-0.028	-0.036	-0.027	-0.031	t	72335	WDVRPLTETRGDLFS	Homo sapiens
1	1.00	1	1	0.01	0.007	0.001	0.01	0.01	t	67445	TYRATYQDFD	Oryctolagus cuniculus
1	1.00	1	1	-0.02	-0.015	-0.024	-0.017	-0.021	t	77801	SVKKCLFEKC	Homo sapiens
1	1.00	1	1	0	0.002	-0.001	0.001	-0.001	t	19893	GGIVTC	Homo sapiens
1	1.00	1	1	0.02	0.022	0.017	0.021	0.019	t	103301	KAMLFDGRSGEPFPY	Homo sapiens
1	1.00	1	1	0.03	0.029	0.03	0.031	0.03	t	8355	DGAVQPDGG	Canis lupus
1	1.00	1	1	0.02	0.023	0.008	0.02	0.02	t	153425	MQIFVKTLTGKTITL	Mus musculus
1	1.00	1	1	0.02	0.02	0.008	0.02	0.02	t	153425	MQIFVKTLTGKTITL	Mus musculus
1	1.00	1	1	0.03	0.03	0.029	0.04	0.032	t	106648	MHNPSMT	Homo sapiens

1	1.00	1	1	0.03	0.023	0.019	0.03	0.03	t	94473	CNPACGPKYS	Oryctolagus cuniculus
1	1.00	1	1	0.03	0.03	0.019	0.03	0.03	t	94473	CNPACGPKYS	Capra hircus
1	1.00	1	1	0.03	0.03	0.019	0.03	0.03	t	94473	CNPACGPKYS	Oryctolagus cuniculus
1	1.00	1	1	0.05	0.05	0.05	0.045	0.046	t	116085	EPANPSEK	Mus musculus
1	1.00	1	1	-0.06	-0.056	-0.049	-0.06	-0.065	t	123279	GYWKIRGLA	Ovis aries
1	1.00	1	1	0.01	0.01	-0.003	0.007	0.01	t	147200	PQQLPQ	Homo sapiens
1	1.00	1	1	0.02	0.025	0.016	0.021	0.02	t	26308	IGQMFETTMR	Homo sapiens
1	1.00	1	1	-0.02	-0.018	-0.02	-0.013	-0.018	t	133638	LTSRPA	Homo sapiens
1	1.00	1	1	0.01	0.013	0.004	0.01	0.01	t	43548	NDYEDRYYRE	Mus musculus
1	1.00	1	1	0.06	0.062	0.053	0.059	0.059	t	135805	YVNSTEAE	Homo sapiens
1	1.00	1	1	-0.05	-0.052	-0.044	-0.049	-0.052	t	79042	PKSKPFA	Mus musculus
1	1.00	1	1	0.01	0.01	-0.003	0.01	0.012	t	7274	CVKKQCPE	Mus musculus
1	1.00	1	1	0.01	0.012	0.005	0.011	0.01	t	19096	GDRADGQPA	Homo sapiens
1	1.00	1	1	0.06	0.06	0.06	0.063	0.06	t	149035	EGSGTTK	Sus scrofa

1	1.00	1	1	0.05	0.054	0.043	0.053	0.05	t	131657	SGLQAEDEADYYCSSY	Homo sapiens
1	1.00	1	1	-0.02	-0.018	-0.022	-0.02	-0.021	t	118548	PAPGMRPP	Homo sapiens
1	1.00	1	1	0.03	0.03	0.03	0.03	0.03	t	105813	GELPNIEA	Oryctolagus cuniculus
1	1.00	1	1	0.03	0.028	0.031	0.029	0.03	t	41350	MEEINTLQ	Anas platyrhynchos
1	1.00	1	1	-0.01	-0.008	-0.01	-0.01	-0.014	t	141749	SNLDHI	Rattus norvegicus
1	1.00	1	1	0.05	0.05	0.043	0.059	0.052	t	136590	RDYFEEYGKIDTIEIIT	Homo sapiens
1	1.00	1	1	-0.01	-0.012	-0.017	-0.007	-0.011	t	108323	DTPPPCPRCP	Mus musculus
1	1.00	1	1	0	-0.007	0	0	-0.001	t	153841	KCLKFYSKISEYRHY	Oryctolagus cuniculus
1	1.00	1	1	0.03	0.03	0.03	0.03	0.03	t	78399	GLLNDS	Mus musculus
1	1.00	1	1	0	0	0	0.004	-0.002	t	125411	FQGLCNETLTLKLYNNGFTS	Homo sapiens
1	1.00	1	1	-0.04	-0.038	-0.047	-0.033	-0.039	t	121692	PAPPKPEPKPKKAPA	Homo sapiens
1	1.00	1	1	0.02	0.016	0.015	0.023	0.021	t	100320	NCDFSQTATLTN	Oryctolagus cuniculus
1	1.00	1	1	0.02	0.013	0.009	0.02	0.02	t	94452	CCNPACGPKY	Oryctolagus cuniculus
1	1.00	1	1	0.02	0.027	0.009	0.02	0.02	t	94452	CCNPACGPKY	Capra hircus

1	1.00	1	1	0	-0.003	-0.01	0	0	t	755	ADQLPNVG	Oryctolagus cuniculus
1	1.00	1	1	0	0	-0.01	0	0	t	755	ADQLPNVG	Oryctolagus cuniculus
1	1.00	1	1	-0.03	-0.028	-0.03	-0.023	-0.032	t	134765	PTPGPSRRGPSLGAS	Homo sapiens
1	1.00	1	1	0.02	0.02	0.02	0.027	0.02	t	136006	VETGHTSQVTPS	Homo sapiens
1	1.00	1	1	-0.02	-0.024	-0.028	-0.021	-0.022	t	2215	AKKPAGKGSPSTLQTPG	Oryctolagus cuniculus
1	1.00	1	1	0.01	0.011	0	0.011	0.01	t	72182	VYVGNYDFW	Homo sapiens
1	1.00	1	1	0.05	0.05	0.048	0.047	0.048	t	98725	DDEALEKFDK	Homo sapiens
1	1.00	1	1	0.02	0.02	0.02	0.03	0.023	t	141707	MPEEVHHGEEEV	Homo sapiens
1	1.00	1	1	0.02	0.022	0.02	0.027	0.02	t	138219	PTPETGPLQR	Homo sapiens
1	1.00	1	1	-0.02	-0.016	-0.02	-0.013	-0.02	t	123579	PGMIPPPG	Homo sapiens
1	1.00	1	1	-0.04	-0.041	-0.05	-0.04	-0.04	t	34081	KVKTTVVYGRRRSK	Anas platyrhynchos
1	1.00	1	1	-0.02	-0.018	-0.003	-0.013	-0.021	t	109251	GSFFLYS	Homo sapiens
1	1.00	1	1	0	0.004	-0.005	-0.003	-0.002	t	5385	AVIRGKKGSG	Homo sapiens
1	1.00	1	1	0.02	0.026	0.017	0.02	0.02	t	139896	SLNSSAQ	Ovis aries

1	1.00	1	1	0.01	0.013	0.001	0.01	0.01	t	13735	EPQVYTLPPSR	Mus musculus
1	1.00	1	1	-0.01	-0.006	-0.022	-0.011	-0.01	t	141560	SDEALPPGSPR	Mus musculus
1	1.00	1	1	0.01	0.008	0.001	0.01	0.01	t	82594	GDVKAS	Mus musculus
1	1.00	1	1	0	0	-0.005	0	0	t	18363	FWRGDLVFDFQV	Homo sapiens
1	1.00	1	1	0.02	0.018	0.013	0.013	0.016	t	128612	FDSHY	Mus musculus
1	1.00	1	1	0.01	0.01	0.008	0.014	0.01	t	47591	PFVQWFMGL	Homo sapiens
1	1.00	1	1	0.01	0.01	0.003	0.01	0.01	t	138258	VMHEALH	Homo sapiens
1	1.00	1	1	0.01	0.012	0.015	0.017	0.011	t	112814	PEQPPIH	Homo sapiens
1	1.00	1	1	0	0	0	0	-0.003	t	50931	QGPGAPQGPGAPQGPGAP	Mus musculus
1	1.00	1	1	0	0	0	0	-0.003	t	50931	QGPGAPQGPGAPQGPGAP	Mus musculus
1	1.00	1	1	0.03	0.034	0.023	0.033	0.032	t	142474	SSTTGVAM	Homo sapiens
1	1.00	1	1	-0.02	-0.022	-0.029	-0.017	-0.02	t	115540	VKNKRTFLSPWISNI	Mus musculus
1	1.00	1	1	0	0.002	0	0.003	-0.002	t	119633	QYTGHITWTPPAIFKS	Homo sapiens
1	1.00	1	1	-0.01	-0.01	-0.014	-0.01	-0.011	t	110873	GSGTGGRTGYIPL	Mus musculus
1	1.00	1	1	0	0	-0.005	0	-0.001	t	3880	APTYSWGA	Mus musculus
1	1.00	1	1	0.02	0.02	0.02	0.031	0.02	t	118645	VAVSAAPGSAAPAAG	Homo sapiens

1	1.00	1	1	0	0.005	-0.012	-0.001	0.002	t	99226	ERQLKSA	Gallus gallus
1	1.00	1	1	0	0.003	0	0	0	t	31373	KINSLS	Mus musculus
1	1.00	1	1	0	0.002	-0.005	-0.003	-0.002	t	98740	EALEKFDKAL	Homo sapiens
1	1.00	1	1	0.01	0.01	-0.001	0.01	0.01	t	94620	NPACGPKY	Capra hircus
1	1.00	1	1	0.01	0.017	-0.001	0.01	0.01	t	94620	NPACGPKY	Capra hircus
1	1.00	1	1	0	-0.002	0	0	-0.004	t	137401	FEDVW	Mus musculus
1	1.00	1	1	0.05	0.05	0.042	0.05	0.05	t	59430	SLRECCQELQEV	Homo sapiens
1	1.00	1	1	0.02	0.02	0.006	0.02	0.023	t	109349	IPVDLL	Mus musculus
1	1.00	1	1	-0.02	-0.02	-0.021	-0.02	-0.02	t	48762	PPDHSA	Homo sapiens
1	1.00	1	1	-0.03	-0.028	-0.032	-0.023	-0.029	t	127643	ERPLELRVRVLDI	Homo sapiens
1	1.00	1	1	0.02	0.02	0.02	0.02	0.019	t	115060	GYLEQL	Homo sapiens
1	1.00	1	1	0	0	0	0	-0.004	t	143676	DRVGLRNLRGYY	Mus musculus
1	1.00	1	1	0.01	0.008	0.008	0.009	0.008	t	25002	HTVTTTTK	Mus musculus
1	1.00	1	1	0.01	0.007	0.01	0.01	0.009	t	135562	IHAMDIPPPTG	Oryctolagus cuniculus
1	1.00	1	1	0.02	0.02	0.015	0.023	0.02	t	38764	LPVWARPD	Homo sapiens

1	1.00	1	1	-0.03	-0.028	-0.032	-0.023	-0.03	t	112403	WWNDNKF	Homo sapiens
1	1.00	1	1	0.01	0.015	0.013	0.01	0.01	t	48452	PLPPVA	Cavia porcellus
1	1.00	1	1	0	0	-0.005	-0.003	-0.002	t	42159	MMNWSPT	Homo sapiens
1	1.00	1	1	0	-0.003	0	0	-0.004	t	134078	GYYYPSDPNTFYAPPYQ	Oryctolagus cuniculus
1	1.00	1	1	0	-0.003	0	0	-0.004	t	134188	PMSTPSATSVR	Oryctolagus cuniculus
1	1.00	1	1	0	-0.003	0	0	-0.004	t	59922	SNTFINNA	Oryctolagus cuniculus
1	1.00	1	1	0	-0.004	0	0	-0.004	t	58975	SKYSNTFNINAYNMVIRRSM	Aotus sp.
1	1.00	1	1	0	-0.004	0	0	-0.004	t	51125	QIPYNLKIRAGGLDGGKKLV	Aotus sp.
1	1.00	1	1	0	-0.004	0	0	-0.004	t	42761	MTDVIRYRYSNNYEASDHIS	Aotus sp.
1	1.00	1	1	0	-0.005	0	0	-0.004	t	58975	SKYSNTFNINAYNMVIRRSM	Aotus sp.
1	1.00	1	1	0	0	0	0	-0.004	t	42780	MTDVNRYRYSNNYEEQPHIS	Aotus nancymaae
1	1.00	1	1	-0.02	-0.02	-0.035	-0.02	-0.02	t	51971	QPSSRRYPF	Homo sapiens
1	1.00	1	1	-0.01	-0.006	-0.017	-0.007	-0.011	t	115645	RNGKDQDGDQAP	Homo sapiens
1	1.00	1	1	-0.01	0.004	-0.017	-0.007	-0.011	t	115645	RNGKDQDGDQAP	Homo sapiens
1	1.00	1	1	-0.03	-0.032	-0.03	-0.027	-0.03	t	45816	NSGCFRHL	Mus musculus

1	1.00	1	1	0.01	0.01	0.01	0.01	0.011	t	115566	YKKYLLFCME	Homo sapiens
1	1.00	1	1	0.02	0.017	0.014	0.02	0.018	t	7864	DDQPSSHQPLFY	Oryctolagus cuniculus
1	1.00	1	1	-0.01	-0.01	-0.02	-0.01	-0.01	t	104161	NVVLKTITESATSPPT	Oryctolagus cuniculus
1	1.00	1	1	0.04	0.036	0.032	0.039	0.04	t	98061	GFSGSVQFVP	Oryctolagus cuniculus
1	1.00	1	1	0.04	0.038	0.032	0.039	0.04	t	98061	GFSGSVQFVP	Mus musculus
1	1.00	1	1	-0.01	-0.01	-0.015	-0.01	-0.012	t	125055	FVYGGCQGNN	Mus musculus
1	1.00	1	1	-0.01	-0.008	-0.01	-0.01	-0.01	t	107368	FSSAP	Rattus norvegicus
1	1.00	1	1	0.02	0.024	0.02	0.027	0.02	t	131700	TGVTTQGVKSLLTSMYVKEF	Homo sapiens
1	1.00	1	1	0.02	0.02	0.022	0.02	0.018	t	72911	WNSTTFHQTLQDP	Mus musculus
1	1.00	1	1	0.01	0.01	0.008	0.01	0.006	t	97769	WNPSLLGN	Mus musculus
1	1.00	1	1	0.01	0.01	0.008	0.01	0.006	t	97769	WNPSLLGN	Mus musculus
1	1.00	1	1	0.01	0.01	0.005	0.007	0.008	t	38630	LPPTVMFPPQ	Homo sapiens
1	1.00	1	1	0.03	0.033	0.022	0.03	0.032	t	65974	TQYERE	Mus musculus
1	1.00	1	1	0	0	0	0	0	t	27421	ILYEAFDEM	Homo sapiens

1	1.00	1	1	0.03	0.03	0.022	0.03	0.029	t	26375	IHFGSDYE DRYY	Mus musculu s
1	1.00	1	1	0.05	0.05	0.048	0.05	0.051	t	10320	DTDSGYD VPDY	Mus musculu s
1	1.00	1	1	0.06	0.064	0.052	0.06	0.062	t	1450	AGAPSAE QRER	Mus musculu s
1	1.00	1	1	0.01	0.006	0.001	0.007	0.008	t	95334	EDVPQPP VSQFH	Oryctola gus cuniculu s
1	1.00	1	1	0	0	0	0	-0.004	t	7502	DAEVAGT QWFTPSG KTPVFG	Aotus nancyma ae
1	1.00	1	1	0.04	0.04	0.04	0.04	0.04	t	78399	GLLNDS	Mus musculu s
1	1.00	1	1	-0.01	-0.01	-0.01	0	-0.01	t	98973	QSLACQCL VR	Homo sapiens
1	1.00	1	1	-0.01	-0.01	-0.01	0	-0.01	t	115197	CVKKILDK VG	Homo sapiens
1	1.00	1	1	-0.01	-0.014	-0.019	-0.011	-0.01	t	29803	KAFRELAD AL	Oryctola gus cuniculu s
1	1.00	1	1	-0.01	-0.008	-0.009	-0.004	-0.01	t	109030	GAQGPRG E	Rattus norvegic us
1	1.00	1	1	-0.05	-0.05	-0.047	-0.043	-0.049	t	105966	MKKRH	Homo sapiens
1	1.00	1	1	-0.01	-0.013	-0.01	-0.01	-0.011	t	105982	NKGAIIGL MVG	Oryctola gus cuniculu s
1	1.00	1	1	-0.02	-0.016	-0.016	-0.013	-0.02	t	144700	KEWAKRA ALP	Homo sapiens
1	1.00	1	1	0	-0.006	-0.016	0	0.002	t	41169	MAVPAFS DIAKSAND LLNKD	Oryctola gus cuniculu s

1	1.00	1	1	0	0.001	-0.007	-0.003	0	t	130464	DFRVT	Mus musculus
1	1.00	1	1	0.01	0.008	0.006	0.009	0.01	t	48279	PLATQPPVLAL	Mus musculus
1	1.00	1	1	0.03	0.03	0.03	0.033	0.03	t	149114	STPRLPD	Sus scrofa
1	1.00	1	1	0.02	0.023	0.013	0.02	0.018	t	134654	DQQNQFQKAET	Mus musculus
1	1.00	1	1	-0.06	-0.062	-0.06	-0.053	-0.06	t	123244	GMIPPPGL	Homo sapiens
1	1.00	1	1	0.02	0.024	0.005	0.017	0.02	t	111588	NLIQHVKGGTPKRPN	Homo sapiens
1	1.00	1	1	0.04	0.038	0.032	0.043	0.042	t	95933	TTSDVA	Mus musculus
1	1.00	1	1	0.04	0.041	0.044	0.039	0.038	t	41131	MASRPAASSPVE	Oryctolagus cuniculus
1	1.00	1	1	0.01	0.011	-0.006	0.007	0.01	t	78835	SPDKLLTFIASDTCPLV	Canis familiaris
1	1.00	1	1	0.03	0.032	0.029	0.031	0.03	t	2133	AIYGAA	Homo sapiens
1	1.00	1	1	0.02	0.021	0.019	0.023	0.019	t	99631	RQGAKEPYPEFVDRL	Equus caballus
1	1.00	1	1	-0.01	-0.01	-0.017	-0.001	-0.01	t	130971	MILHPDVQRRVQQEIDDVIG	Homo sapiens
1	1.00	1	1	0	0.004	0.009	0.007	-0.001	t	119452	FRKTSKGGS	Homo sapiens
1	1.00	1	1	0.01	0.01	0.01	0.02	0.01	t	114987	YANTTSEDGK	Homo sapiens
1	1.00	1	1	-0.01	-0.008	-0.016	-0.007	-0.01	t	144823	YWEGSGIGAI	Homo sapiens

1	1.00	1	1	-0.01	-0.006	-0.008	-0.009	-0.01	t	74217	YIGGPL	Homo sapiens
1	1.00	1	1	0.04	0.036	0.03	0.04	0.04	t	64905	TLLGEATA	Oryctolagus cuniculus
1	1.00	1	1	-0.01	-0.01	-0.015	-0.012	-0.012	t	100398	PTTPATPTPTPK	Homo sapiens
1	1.00	1	1	-0.03	-0.028	-0.038	-0.027	-0.029	t	114450	LEIIEEFFKNKSGLK	Homo sapiens
1	1.00	1	1	0	0.003	-0.001	-0.001	0	t	41043	MAGKREMVII	Mus musculus
1	1.00	1	1	-0.01	-0.01	-0.01	0.001	-0.008	t	118585	RNPLIAGK	Homo sapiens
1	1.00	1	1	0.05	0.05	0.047	0.05	0.05	t	94606	LTGGFYEPL	Mus musculus
1	1.00	1	1	-0.02	-0.024	-0.02	-0.017	-0.022	t	156860	DRWNSMWKESLL	Bos taurus
1	1.00	1	1	0	0	0	0	-0.003	t	45407	NPNANPNA	Mus musculus
1	1.00	1	1	0.01	0.01	-0.006	0.008	0.01	t	67745	VASGGSGNSRRTNPS	Homo sapiens
1	1.00	1	1	-0.01	-0.01	-0.022	-0.01	-0.011	t	72544	WHLKSIGQLVIE	Mus musculus
1	1.00	1	1	-0.01	-0.012	-0.022	-0.01	-0.011	t	72544	WHLKSIGQLVIE	Oryctolagus cuniculus
1	1.00	1	1	0.01	0.01	0.01	0.01	0.009	t	21601	GPEET	Mus musculus
1	1.00	1	1	0.01	0.013	0.01	0.01	0.009	t	21601	GPEET	Mus musculus

1	1.00	1	1	0	0	-0.014	0	0.001	t	120522	FALAPSLTEI	Mus musculus
1	1.00	1	1	-0.01	-0.01	-0.01	-0.01	-0.011	t	119581	FSCSVMHE	Homo sapiens
1	1.00	1	1	-0.01	-0.01	-0.01	0	-0.01	t	114965	TSEDGKVMVL	Homo sapiens
1	1.00	1	1	0	0	0	0	-0.004	t	25140	HWSYGLRPG	Rattus norvegicus
1	1.00	1	1	0.02	0.02	0.016	0.02	0.02	t	58648	SIP	Homo sapiens
1	1.00	1	1	0	0	0	0	-0.003	t	108622	SHQESTRGRSRGRS	Homo sapiens
1	1.00	1	1	0.05	0.05	0.05	0.05	0.05	t	159282	EENW	Oryctolagus cuniculus
1	1.00	1	1	-0.01	-0.008	-0.01	-0.007	-0.01	t	69828	VLVLFGFFTLSPWY	Homo sapiens
1	1.00	1	1	-0.04	-0.04	-0.04	-0.033	-0.04	t	119475	KKIK	Homo sapiens
1	1.00	1	1	-0.02	-0.016	-0.02	-0.014	-0.021	t	107503	LFPPKPK	Homo sapiens
1	1.00	1	1	0.03	0.032	0.03	0.037	0.028	t	121879	SSQFDPNKQTPSGKK	Homo sapiens
1	1.00	1	1	0.03	0.032	0.027	0.033	0.03	t	49920	PVNPSTAPAPAPTPT	Homo sapiens
1	1.00	1	1	0.03	0.03	0.035	0.03	0.03	t	137748	DTQLTLEALKLV	Mus musculus
1	1.00	1	1	-0.02	-0.017	-0.036	-0.02	-0.019	t	119301	PEVVVLGSQEGAMHT	Mus musculus
1	1.00	1	1	0.03	0.03	0.017	0.03	0.03	t	52494	QTDEIKNDNIQTDEIKNDNI	Oryctolagus cuniculus

1	1.00	1	1	0.03	0.033	0.017	0.03	0.03	t	52494	QTDEIKNDNIQTDEIKNDNI	Mus musculus
1	1.00	1	1	0.01	0.007	0.004	0.01	0.008	t	133618	KKLLVRGLYRISFTTYK	Oryctolagus cuniculus
1	1.00	1	1	0.03	0.03	0.023	0.03	0.031	t	78136	EEPKHETKETE	Homo sapiens
1	1.00	1	1	0.04	0.042	0.031	0.037	0.04	t	64998	TLPDTEKQI	Homo sapiens
1	1.00	1	1	0.04	0.042	0.031	0.037	0.04	t	64998	TLPDTEKQI	Homo sapiens
1	1.00	1	1	-0.01	-0.008	-0.017	-0.003	-0.01	t	132304	FMIHQLMGNGVWHFGET	Homo sapiens
1	1.00	1	1	0.02	0.018	0.02	0.019	0.02	t	69883	VLYQEFDE	Mus musculus
1	1.00	1	1	0.05	0.053	0.045	0.05	0.048	t	78703	KKKKTDEAQP	Mus musculus
1	1.00	1	1	0.04	0.04	0.04	0.047	0.04	t	119506	PIVET	Homo sapiens
1	1.00	1	1	0.04	0.036	0.035	0.037	0.04	t	115135	YSYDVNSTEKG	Oryctolagus cuniculus
1	1.00	1	1	0.04	0.036	0.035	0.037	0.04	t	115135	YSYDVNSTEKG	Oryctolagus cuniculus
1	1.00	1	1	0.07	0.072	0.069	0.071	0.07	t	69624	VLLQME	Homo sapiens
1	1.00	1	1	0.03	0.032	0.025	0.027	0.028	t	13509	ENLCSTFCKE	Homo sapiens
1	1.00	1	1	0.01	0.006	0.008	0.013	0.011	t	99662	SEVIFR	Oryctolagus cuniculus
1	1.00	1	1	0	0	0.001	0	0	t	15334	FCLFFAFSVAAP	Oryctolagus cuniculus

1	1.00	1	1	-0.01	-0.008	-0.001	-0.003	-0.011	t	119513	RGLKSKIAM	Homo sapiens
1	1.00	1	1	-0.01	-0.006	-0.001	-0.003	-0.011	t	119513	RGLKSKIAM	Homo sapiens
1	1.00	1	1	0.01	0.01	0.01	0.01	0.01	t	146020	TCPPDIIPK	Mus musculus
1	1.00	1	1	0.02	0.024	0.014	0.017	0.018	t	30115	KDASIQVVSA	Homo sapiens
1	1.00	1	1	0.04	0.038	0.036	0.037	0.04	t	1391	AFRRYQEE	Anas platyrhynchos
1	1.00	1	1	-0.01	-0.011	-0.02	-0.01	-0.01	t	73074	WSGKFPTL	Anas platyrhynchos
1	1.00	1	1	0.03	0.028	0.027	0.027	0.028	t	122167	PPPITQGECKQL	Mus musculus
1	1.00	1	1	0.01	0.012	0.022	0.017	0.01	t	130859	GLLAQQMDY	Homo sapiens
1	1.00	1	1	0.02	0.023	0.012	0.02	0.019	t	102334	CETDKWKYENPCKKMCTVSD	Mus musculus
1	1.00	1	1	0.02	0.02	0.012	0.02	0.019	t	102334	CETDKWKYENPCKKMCTVSD	Mus musculus
1	1.00	1	1	0.02	0.02	0.012	0.02	0.019	t	102334	CETDKWKYENPCKKMCTVSD	Mus musculus
1	1.00	1	1	0.01	0.01	0.003	0.01	0.01	t	138258	VMHEALH	Homo sapiens
1	1.00	1	1	0	0.002	0	0.007	0.003	t	107479	KFNWYVD	Homo sapiens
1	1.00	1	1	0.04	0.04	0.04	0.042	0.04	t	71321	VTGGAAA	Homo sapiens
1	1.00	1	1	0.01	0.012	0.005	0.007	0.008	t	98696	AMAASDISLL	Homo sapiens

1	1.00	1	1	-0.03	-0.025	-0.037	-0.027	-0.028	t	60780	SRRFAPAL	Homo sapiens
1	1.00	1	1	-0.01	-0.008	-0.017	-0.007	-0.011	t	144594	ARGISRTKYW	Homo sapiens
1	1.00	1	1	-0.02	-0.011	-0.028	-0.02	-0.02	t	45115	NNGTWSKHKAEVKKFL	Gallus gallus
1	1.00	1	1	0	0.002	0	0.001	0	t	69125	VITGTI	Homo sapiens
1	1.00	1	1	0.01	0.01	0	0.01	0.009	t	139730	EQLAGNLRENIE	Mus musculus
1	1.00	1	1	-0.02	-0.02	-0.023	-0.018	-0.02	t	48343	PLGLKRGIEK	Homo sapiens
1	1.00	1	1	0.02	0.02	0.025	0.02	0.02	t	137749	DTQLTYAGLKLV	Mus musculus
1	1.00	1	1	0	-0.004	-0.007	0.003	0	t	35006	LAVLLGAC	Oryctolagus cuniculus
1	1.00	1	1	0.01	0.01	0.01	0.01	0.009	t	119995	KNEELRLYHHLFNNY	Rattus norvegicus
1	1.00	1	1	-0.01	-0.008	-0.017	-0.007	-0.01	t	121161	AVEKVKRTKDE	Homo sapiens
1	1.00	1	1	0	0	-0.018	0	0.002	t	56599	RYNLVFSGDSDRICSNRA	Mus musculus
1	1.00	1	1	-0.03	-0.034	-0.036	-0.031	-0.032	t	20796	GLDGTVWAQSAKFP	Oryctolagus cuniculus
1	1.00	1	1	-0.01	-0.017	-0.027	-0.013	-0.007	t	110795	CDLIPNLKLNNG	Macaca

1	1.00	1	1	-0.01	-0.012	-0.019	-0.011	-0.01	t	41043	MAGKREMVII	Mus musculus
1	1.00	1	1	0	0.003	-0.006	0	0	t	54504	RLDGTTLEV	Mus musculus
1	1.00	1	1	-0.02	-0.02	-0.025	-0.013	-0.02	t	118144	SLCQSLRER	Homo sapiens
1	1.00	1	1	0.02	0.02	0.006	0.02	0.023	t	109348	IPVDL	Mus musculus
1	1.00	1	1	-0.02	-0.02	-0.018	-0.019	-0.021	t	64377	TIMAKN	Homo sapiens
1	1.00	1	1	0.02	0.022	0.002	0.019	0.021	t	98686	AHGRSQVLQQST	Homo sapiens
1	1.00	1	1	0.01	0.01	0.01	0.01	0.007	t	61778	STNDNIKDLLD	Oryctolagus cuniculus
1	1.00	1	1	0.01	0.012	0.01	0.017	0.01	t	119579	FKDYSSVVRPVEDHRQ	Homo sapiens
1	1.00	1	1	0	0.002	0.007	0	-0.005	t	123013	AIFSQFGQILDILV	Oryctolagus cuniculus
1	1.00	1	1	-0.02	-0.016	-0.019	-0.013	-0.023	t	134682	GPSRRGPSLGASSHQ	Homo sapiens
1	1.00	1	1	0.04	0.04	0.029	0.037	0.042	t	63196	TDLNILERFNYEEAQT	Homo sapiens
1	1.00	1	1	-0.01	-0.008	-0.018	-0.007	-0.008	t	110912	KATPNDGYFWED	Homo sapiens
1	1.00	1	1	0	-0.004	-0.007	0.003	0	t	125441	HPLTCDDPRFQ	Oryctolagus cuniculus

1	1.00	1	1	0.01	0.008	0	0.009	0.012	t	25060	HVLDHVQT	Anas platyrhynchos
1	1.00	1	1	-0.01	-0.01	-0.019	-0.01	-0.01	t	97020	TVTRGTISDP	Oryctolagus cuniculus
1	1.00	1	1	0.01	0.012	0.012	0.011	0.008	t	40611	LVTKDIDFD	Homo sapiens
1	1.00	1	1	0.03	0.034	0.032	0.031	0.029	t	30199	KDLLED	Homo sapiens
1	1.00	1	1	0.01	0.008	0.01	0.009	0.01	t	46969	PATIGDYTDF	Mus musculus
1	1.00	1	1	0	-0.002	-0.02	-0.007	0	t	128152	YIFSEEAQVKDVPVVT	Mus musculus
1	1.00	1	1	-0.05	-0.052	-0.052	-0.047	-0.05	t	7559	DAKFRPN	Mus musculus
1	1.00	1	1	0.03	0.033	0.03	0.03	0.03	t	143682	EEIEKE	Mus musculus
1	1.00	1	1	0	0.003	-0.01	0	0	t	48715	PNVDPN	Mus musculus
1	1.00	1	1	0	0.002	-0.007	0.007	0.001	t	114196	CKPISGHNSLFQWYRQT	Homo sapiens
1	1.00	1	1	-0.01	-0.01	-0.02	-0.013	-0.01	t	49993	PVVAESPKKP	Homo sapiens
1	1.00	1	1	0	0	-0.002	-0.003	-0.002	t	98904	MKGLDIQKVA	Homo sapiens
1	1.00	1	1	0	0.003	-0.009	0	0	t	71300	VTFGDMR	Mus musculus
1	1.00	1	1	0	-0.01	0	0.001	-0.002	t	24694	HRMAWDMMM	Homo sapiens

1	1.00	1	1	0.02	0.024	0.02	0.021	0.02	t	14405	ETHVTGG	Homo sapiens
1	1.00	1	1	-0.01	-0.01	-0.01	-0.007	-0.011	t	11819	EENVEHDA	Homo sapiens
1	1.00	1	1	0.05	0.047	0.043	0.05	0.05	t	108185	VEHSDLSFS	Oryctolagus cuniculus
1	1.00	1	1	0.01	0.014	0.001	0.01	0.009	t	113539	IIPKVEGKTIAEQILQYGSM	Cavia porcellus
1	1.00	1	1	0.01	0.014	0.001	0.01	0.009	t	113539	IIPKVEGKTIAEQILQYGSM	Cavia porcellus
1	1.00	1	1	0.02	0.022	0.02	0.027	0.021	t	107758	AWNCCVKQTP	Homo sapiens
1	1.00	1	1	0.04	0.043	0.029	0.04	0.042	t	111725	RHGEEYDLQFIFQLC	Mus musculus
1	1.00	1	1	0	0	0	0	-0.004	t	78939	CINHRGYWVC	Mus musculus
1	1.00	1	1	0.01	0.012	0.01	0.017	0.01	t	107878	GWVAMHPQGDL	Homo sapiens
1	1.00	1	1	0.02	0.022	0.02	0.021	0.02	t	58129	SGLTSLF	Homo sapiens
1	1.00	1	1	0.03	0.032	0.023	0.033	0.032	t	77858	DYPSLSATDI	Homo sapiens
1	1.00	1	1	0	0.002	-0.011	-0.003	0	t	159063	RKAADTFNFKTFFHT	Homo sapiens
1	1.00	1	1	0	0.004	0	0.007	0	t	108844	DAGVIQSPRHEVTEMG	Homo sapiens
1	1.00	1	1	0	-0.002	0	0.009	0	t	38304	LNPKTNKWEDK	Mus musculus

1	1.00	1	1	0	-0.002	0	0.009	0	t	27728	INQQLNPKTNKWEDK	Mus musculus
1	1.00	1	1	0	-0.002	0	0.009	0	t	27727	INQQLNPK	Mus musculus
1	1.00	1	1	-0.01	-0.01	-0.004	-0.007	-0.01	t	48414	PLLVETWK	Homo sapiens
1	1.00	1	1	0.01	0.008	-0.004	0.003	0.009	t	121100	YYFAPDHRTV	Mus musculus
1	1.00	1	1	0.01	0.007	0.01	0.01	0.007	t	12951	ELAPEDPEDS	Oryctolagus cuniculus
1	1.00	1	1	0.03	0.041	0.03	0.03	0.028	t	156886	HGLYGS	Gallus gallus
1	1.00	1	1	0.03	0.028	0.025	0.031	0.03	t	76192	YTTSEYPSQPPS	Homo sapiens
1	1.00	1	1	0.01	0.012	0.01	0.014	0.009	t	111028	SRALAREVDLKDYEDQQK	Homo sapiens
1	1.00	1	1	-0.01	-0.01	-0.002	-0.003	-0.014	t	122512	PEPAKSAPAPKKGSK	Mus musculus
1	1.00	1	1	0.03	0.028	0.024	0.029	0.03	t	27681	INLLDDSDF	Sus scrofa
1	1.00	1	1	0.01	0.007	-0.002	0.01	0.01	t	136832	EVVRAEIMRS	Oryctolagus cuniculus
1	1.00	1	1	-0.01	-0.01	-0.01	-0.01	-0.007	t	107784	DGSFFLY	Homo sapiens
1	1.00	1	1	0.01	0.005	0.01	0.011	0.008	t	100297	LVFFAEDVGS	Oryctolagus cuniculus
1	1.00	1	1	0.02	0.02	0.009	0.02	0.02	t	56059	RTGEGL	Homo sapiens

1	1.00	1	1	-0.01	-0.01	-0.02	-0.01	-0.01	t	112897	AATGAATAAA	Mus musculus
1	1.00	1	1	0.02	0.02	0.02	0.02	0.02	t	48778	PPFSLSPVPTL	Oryctolagus cuniculus
1	1.00	1	1	0.01	0.01	-0.003	0.007	0.01	t	147197	PLVQEQQF	Homo sapiens
1	1.00	1	1	0.05	0.047	0.052	0.05	0.05	t	78806	QSQIESR	Oryctolagus cuniculus
1	1.00	1	1	0.03	0.032	0.028	0.027	0.028	t	50863	QGFSRNTLEAAFNAE	Homo sapiens
1	1.00	1	1	0.02	0.024	0.017	0.021	0.019	t	103301	KAMLFDGRSGEPFPY	Homo sapiens
1	1.00	1	1	-0.03	-0.032	-0.046	-0.027	-0.028	t	33436	KSMDVRRI	Anas platyrhynchos
1	1.00	1	1	-0.03	-0.032	-0.046	-0.027	-0.028	t	33436	KSMDVRRI	Anas platyrhynchos
1	1.00	1	1	-0.02	-0.022	-0.02	-0.02	-0.02	t	117931	IMAPPPGMRP	Mus musculus
1	1.00	1	1	0	0.003	-0.002	0	0	t	30716	KFDWNTPD	Equus caballus
1	1.00	1	1	0.04	0.044	0.033	0.047	0.042	t	104569	MASPGSGFWSFGSEDGS	Homo sapiens
1	1.00	1	1	0.01	0.012	0.01	0.013	0.008	t	136092	YKSQYLNNGPQRIGRKYKK	Homo sapiens
1	1.00	1	1	0	-0.002	-0.003	-0.001	-0.002	t	58872	SKKSVTV	Mus musculus
1	1.00	1	1	0.04	0.042	0.034	0.041	0.04	t	133540	DALVAKAQEVS	Homo sapiens

1	1.00	1	1	-0.01	-0.01	-0.01	-0.01	-0.01	t	105717	DPSIMAKF	Oryctolagus cuniculus
1	1.00	1	1	0	0.008	-0.007	0	-0.001	t	125246	RRQPHVMS	Macaca fascicularis
1	1.00	1	1	0.01	0.01	0.01	0.013	0.01	t	149099	RVLTSMD	Sus scrofa
1	1.00	1	1	0	0	0.005	0.001	-0.001	t	138238	SMLDTPT	Mus musculus
1	1.00	1	1	0.01	0.006	-0.004	0.009	0.01	t	33424	KSLSTLSY	Oryctolagus cuniculus
1	1.00	1	1	0.01	0.01	0.01	0.01	0.01	t	23223	GVYLLPRRGP	Homo sapiens
1	1.00	1	1	-0.01	-0.01	-0.014	-0.01	-0.011	t	120700	KNAIEGIK	Rattus norvegicus
1	1.00	1	1	0	0.002	0	0.007	0.001	t	121287	EYFIRK	Homo sapiens
1	1.00	1	1	-0.01	-0.012	-0.017	-0.007	-0.01	t	37316	LLGCIGSTCA	Mus musculus
1	1.00	1	1	0.03	0.028	0.02	0.029	0.03	t	78687	IRVRKRKGSNKKAEDTLED	Mus musculus
1	1.00	1	1	0.07	0.075	0.066	0.071	0.07	t	38201	LNEEQD	Homo sapiens
1	1.00	1	1	0	0.002	-0.002	0.007	0	t	122904	SNTRVFPNPNNKQALY	Homo sapiens
1	1.00	1	1	0.01	0.01	0.006	0.01	0.007	t	22792	GTQETGDTTPSAYSMS	Oryctolagus cuniculus
1	1.00	1	1	0	0	0	0	0	t	50102	PYIEQGMML	Homo sapiens

1	1.00	1	1	0	0	-0.008	0	0	t	106301	CDIKTTLPPI	Homo sapiens
1	1.00	1	1	0.04	0.043	0.038	0.04	0.04	t	89661	TQLTSSYEDYML	Mus musculus
1	1.00	1	1	0.05	0.05	0.05	0.046	0.048	t	118014	MDC	Homo sapiens
1	1.00	1	1	0.01	0.012	0.006	0.013	0.009	t	19517	GFEKISKGAN	Homo sapiens
1	1.00	1	1	-0.01	-0.006	-0.019	-0.01	-0.01	t	23947	HGRVGIYFGMK	MD
1	1.00	1	1	0	0.002	-0.011	-0.003	-0.002	t	68001	VDKRHD	Homo sapiens
1	1.00	1	1	0.01	0.013	-0.002	0.01	0.01	t	97654	SMGAKPTG	Mus musculus
1	1.00	1	1	-0.02	-0.017	-0.03	-0.02	-0.02	t	66743	TTNRFAKY	Mus musculus
1	1.00	1	1	-0.02	-0.014	-0.03	-0.02	-0.02	t	66743	TTNRFAKY	Equus caballus
1	1.00	1	1	-0.03	-0.03	-0.039	-0.026	-0.03	t	136003	TRKDINTVTTVA	Homo sapiens
1	1.00	1	1	0	0.002	0	0.001	0	t	38522	LPHSNL	Homo sapiens
1	1.00	1	1	0	0.002	0	0.001	0	t	38522	LPHSNL	Homo sapiens
1	1.00	1	1	-0.02	-0.02	-0.02	-0.01	-0.02	t	98903	MHIRLSFNPT	Homo sapiens

1	1.00	1	1	0.05	0.048	0.043	0.047	0.049	t	12185	EGHTDSVGTDAYNQK	Mus musculus
1	1.00	1	1	0.01	0.01	0.007	0.01	0.01	t	11411	EDPASR	Mus musculus
1	1.00	1	1	-0.02	-0.02	-0.037	-0.026	-0.02	t	8730	DINKRKYD	Homo sapiens
1	1.00	1	1	-0.01	-0.01	-0.01	-0.006	-0.012	t	13688	EPLEPFPTQIHKDYK	Homo sapiens
1	1.00	1	1	0.03	0.03	0.023	0.03	0.03	t	138245	SVMHEAL	Homo sapiens
1	1.00	1	1	0.03	0.027	0.016	0.03	0.033	t	64045	THKVGTSFTFES	Oryctolagus cuniculus
1	1.00	1	1	0.03	0.027	0.024	0.031	0.029	t	105647	ADVNTFVA	Oryctolagus cuniculus
1	1.00	1	1	0.05	0.05	0.05	0.045	0.046	t	116085	EPANPSEK	Mus musculus
1	1.00	1	1	0.01	0.008	-0.002	0.013	0.011	t	107149	GIAGFKGEQGPKGET	Mus musculus
1	1.00	1	1	-0.01	-0.006	-0.01	-0.003	-0.009	t	107520	LTVLHQN	Homo sapiens
1	1.00	1	1	-0.02	-0.02	-0.027	-0.014	-0.019	t	117336	NGWSHKDLLR	Homo sapiens
1	1.00	1	1	0.01	0.016	0	0.01	0.01	t	91344	FYETVLFKGFESI	Equus caballus
1	1.00	1	1	-0.01	-0.01	-0.01	-0.01	-0.013	t	4530	ASLKMADPNRFR	Mus musculus
1	1.00	1	1	0	0	-0.005	0.003	0	t	64093	THVTGGA	Homo sapiens
1	1.00	1	1	0	0	0	0	-0.002	t	99037	VFKIDALNEN	Homo sapiens

1	1.00	1	1	-0.01	-0.012	-0.008	-0.003	-0.01	t	130752	CLAVLWV<br>VKSTPAS	Mus<br>musculu<br>s
1	1.00	1	1	0.02	0.018	0.003	0.013	0.021	t	122543	QRSQDEN	Rattus<br>norvegic<br>us
1	1.00	1	1	0.01	0.012	0.003	0.017	0.01	t	139494	ERGSAGH<br>WTSESSVS	Homo<br>sapiens
1	1.00	1	1	0.01	0.01	0.005	0.01	0.008	t	140674	ESQPDPKP<br>DELHKS	Mus<br>musculu<br>s
1	1.00	1	1	0.02	0.02	0.015	0.022	0.02	t	48384	PLKQIAFN<br>SG	Homo<br>sapiens
1	1.00	1	1	0	0	-0.007	0.004	0	t	55838	RSFQNKKS<br>LVAFKIMP<br>LEDM	Homo<br>sapiens
1	1.00	1	1	0.02	0.02	0.02	0.02	0.02	t	30808	KFPGGGQI	Homo<br>sapiens
1	1.00	1	1	-0.03	-0.03	-0.037	-0.033	-0.032	t	14100	ERYLGYLE<br>QLLRLK	Homo<br>sapiens
1	1.00	1	1	0	0	-0.015	0.003	0.002	t	60664	SRGAPGG<br>GFVPSMQ	Homo<br>sapiens
1	1.00	1	1	0.04	0.038	0.033	0.041	0.041	t	131217	EDISAYLL	Mus<br>musculu<br>s
1	1.00	1	1	0	0	0	0	-0.004	t	63122	TDAYNQKL<br>SERRAN	Mus<br>musculu<br>s
1	1.00	1	1	0	0	0	0	-0.004	t	63038	TAVSPTTL<br>RTEVVK	Sus<br>scrofa
1	1.00	1	1	0	0	0	0	-0.004	t	43317	NATAEGR<br>AINRRVE	Mus<br>musculu<br>s
1	1.00	1	1	0	0	0	0	-0.004	t	43317	NATAEGR<br>AINRRVE	Mus<br>musculu<br>s
1	1.00	1	1	0	0	0	0	-0.004	t	48858	PPPPNPN<br>DPPPPNP<br>ND	Mus<br>musculu<br>s
1	1.00	1	1	0	0	0	0	-0.004	t	46444	NVPEKQT<br>RGIFGAIA<br>GFIE	Mus<br>musculu<br>s

1	1.00	1	1	0	0	0	0	-0.004	t	48858	PPPPNPNDPPPPNPND	Mus musculus
1	1.00	1	1	0	0	0	0	-0.004	t	46841	PAKLLKERGFFGAIAGFLE	Mus musculus
1	1.00	1	1	0	0	0	0	-0.004	t	43317	NATAEGRAINRRVE	Mus musculus
1	1.00	1	1	0	0	0	0	-0.004	t	43317	NATAEGRAINRRVE	Mus musculus
1	1.00	1	1	0	0	0	0	-0.004	t	27725	INQDPDKILTY	Mus musculus
1	1.00	1	1	0	0.002	-0.013	0.001	0.004	t	45589	NQMHMLSNKS	Homo sapiens
1	1.00	1	1	-0.03	-0.028	-0.03	-0.023	-0.032	t	120952	SLGSRRTLMLLAQM	Homo sapiens
1	1.00	1	1	-0.06	-0.058	-0.067	-0.053	-0.06	t	107828	ERGLKGHRGFT	Homo sapiens
1	1.00	1	1	0.02	0.022	0.018	0.023	0.019	t	10932	DYNPLLVE	Homo sapiens
1	1.00	1	1	0.02	0.022	0.012	0.021	0.019	t	41177	MAYRFNGIGVTQNVLYE	Homo sapiens
1	1.00	1	1	0.06	0.06	0.06	0.063	0.06	t	48518	PLVAEEDE	Homo sapiens
1	1.00	1	1	0.03	0.028	0.017	0.027	0.03	t	63089	TCPDKKSTA	Mus musculus
1	1.00	1	1	0.02	0.02	0.006	0.02	0.022	t	58906	SKNLAPI	Mus musculus
1	1.00	1	1	0.01	0.008	0.012	0.009	0.008	t	122094	FLALDVPTIGLC	Mus musculus
1	1.00	1	1	-0.02	-0.023	-0.03	-0.02	-0.02	t	98434	RYMDQPSRD	Oryctolagus cuniculus
1	1.00	1	1	0.01	0.01	0.004	0.01	0.012	t	56788	SAFVFPTKD	Mus musculus

1	1.00	1	1	0.03	0.026	0.023	0.033	0.03	t	95558	LNAASAYC ST	Oryctola gus cuniculu s
1	1.00	1	1	0	0.004	-0.005	0.007	0.002	t	37659	LLPHSNLD HILEPSIPW K	Homo sapiens
1	1.00	1	1	0	0	-0.007	0.004	0	t	117688	VSLVCEKL CNEKLLKK ARIH	Homo sapiens
1	1.00	1	1	-0.01	-0.012	-0.01	-0.007	-0.01	t	95824	SDVAGL	Mus musculu s
1	1.00	1	1	0.03	0.034	0.03	0.037	0.03	t	118497	LNGKNIED VIAQGIG	Homo sapiens
1	1.00	1	1	0.02	0.02	0.013	0.02	0.02	t	78375	EPGITTNY DT	Mus musculu s
1	1.00	1	1	0.03	0.023	0.023	0.03	0.03	t	159284	FSLRLMEE NWNAEKR S	Oryctola gus cuniculu s
1	1.00	1	1	0.03	0.023	0.023	0.03	0.03	t	159284	FSLRLMEE NWNAEKR S	Oryctola gus cuniculu s
1	1.00	1	1	-0.02	-0.014	-0.02	-0.019	-0.023	t	134830	YSPCAWE VVR	MD
1	1.00	1	1	0.01	0.01	0.001	0.01	0.01	t	125400	ELLKLLIEA	Mus musculu s
1	1.00	1	1	0.01	0.01	0.001	0.01	0.01	t	125400	ELLKLLIEA	Mus musculu s
1	1.00	1	1	0	0	0	0	-0.004	t	23464	GYSLFQKE MKTHNEG TSGTA	Aotus nancyma ae
1	1.00	1	1	0.02	0.016	0	0.013	0.02	t	127980	FPVIPGINI EQKNACS	Oryctola gus cuniculu s

1	1.00	1	1	0.02	0.018	0	0.013	0.02	t	127980	FPVIPGINI EQKNACS	Mus musculu s
1	1.00	1	1	-0.01	-0.014	-0.01	-0.01	-0.012	t	70396	VPNLRGD RQALA	Mus musculu s
1	1.00	1	1	0.01	0.01	0.008	0.014	0.01	t	47593	PFVQWFV GL	Homo sapiens
1	1.00	1	1	0.03	0.034	0.03	0.037	0.03	t	116681	CFEGQEKA HNQDLGL	Homo sapiens
1	1.00	1	1	0.02	0.016	0.018	0.02	0.018	t	29567	IYDKLTVQ TTG	Oryctola gus cuniculu s
1	1.00	1	1	0.02	0.02	0.02	0.023	0.021	t	72087	VYLLPR	Homo sapiens
1	1.00	1	1	0.02	0.02	0.02	0.023	0.02	t	149114	STPRLPD	Sus scrofa
1	1.00	1	1	0.02	0.02	0	0.02	0.022	t	151601	ADLSLEKA ANVQWD E	Homo sapiens
1	1.00	1	1	0.01	0.012	0.003	0.013	0.009	t	135914	EEIAQVATI SAN	Homo sapiens
1	1.00	1	1	0.02	0.022	0.02	0.023	0.019	t	133995	AEVETDKA TVGFE	Homo sapiens
1	1.00	1	1	0	-0.004	-0.001	-0.001	-0.004	t	1257	AFASNRTF DITVSGLG TAPK	Oryctola gus cuniculu s
1	1.00	1	1	-0.02	-0.022	-0.029	-0.02	-0.02	t	100052	GALMLPH FNSKAMVI VVVNK	Homo sapiens
1	1.00	1	1	-0.03	-0.03	-0.031	-0.026	-0.03	t	14261	ESRRAL	Homo sapiens
1	1.00	1	1	-0.03	-0.03	-0.031	-0.026	-0.03	t	14261	ESRRAL	Homo sapiens
1	1.00	1	1	-0.03	-0.03	-0.031	-0.026	-0.03	t	14261	ESRRAL	Homo sapiens
1	1.00	1	1	0.03	0.03	0.02	0.027	0.028	t	38962	LQQQEQIK GEEV	Homo sapiens

1	1.00	1	1	0.01	0.01	0.01	0.01	0.01	t	1771	AHHAAD	Oryctolagus cuniculus
1	1.00	1	1	0.06	0.064	0.06	0.067	0.058	t	131981	NGWKSTEEEDQGSAT	Homo sapiens
1	1.00	1	1	0	0	0	0.004	-0.001	t	119622	NVNHKPSN	Homo sapiens
1	1.00	1	1	0.05	0.05	0.05	0.05	0.05	t	140378	TAEETKQ	Oryctolagus cuniculus
1	1.00	1	1	0.02	0.02	0.012	0.02	0.02	t	9886	DQPKQYEQHLTDYEKIKEG	Mus musculus
1	1.00	1	1	0.02	0.017	0.012	0.02	0.02	t	9886	DQPKQYEQHLTDYEKIKEG	Oryctolagus cuniculus
1	1.00	1	1	0	0.008	0	0	-0.002	t	156864	EPNPTRL	Gallus gallus
1	1.00	1	1	-0.03	-0.03	-0.03	-0.022	-0.032	t	137399	FCGLPRLE	Homo sapiens
1	1.00	1	1	0	0	-0.008	0	0.001	t	79111	VNKGYTGY	Mus musculus
1	1.00	1	1	0.06	0.06	0.052	0.066	0.062	t	105319	LVFFAEDVGSNK	Homo sapiens
1	1.00	1	1	-0.01	-0.008	-0.01	-0.003	-0.01	t	107758	AWNCCVKQTP	Homo sapiens
1	1.00	1	1	-0.01	-0.008	-0.01	-0.003	-0.01	t	107758	AWNCCVKQTP	Homo sapiens
1	1.00	1	1	0.05	0.043	0.043	0.057	0.052	t	136724	VLDSEDLQDMSLMTLSTKTP	Homo sapiens
1	1.00	1	1	0	0	0	0	0.001	t	115342	LDTDYKKYLL	Homo sapiens
1	1.00	1	1	0.01	0.01	0.008	0.017	0.01	t	120548	GAGAPVYLAAVLEYLTAE	Homo sapiens

1	1.00	1	1	-0.04	-0.038	-0.045	-0.037	-0.041	t	45054	NNDFHFE VFNFV	Homo sapiens
1	1.00	1	1	-0.01	-0.01	-0.01	-0.007	-0.013	t	146901	ETHGGED VAVFAR	Homo sapiens
1	1.00	1	1	0.05	0.048	0.05	0.047	0.05	t	134028	DDTIS	Mus musculu s
1	1.00	1	1	0.04	0.04	0.045	0.05	0.043	t	115671	AAMEL	Homo sapiens
1	1.00	1	1	0.04	0.042	0.034	0.043	0.042	t	40076	LTPCD	Homo sapiens
1	1.00	1	1	0.03	0.028	0.025	0.029	0.03	t	70812	VRRIEGGE	Anas platyrhy nchos
1	1.00	1	1	0.08	0.082	0.071	0.077	0.08	t	11210	EAVESTVA TLED	Homo sapiens
1	1.00	1	1	0.08	0.084	0.071	0.077	0.08	t	11210	EAVESTVA TLED	Homo sapiens
1	1.00	1	1	0.02	0.02	0.015	0.02	0.02	t	35611	LEN	Mus musculu s
1	1.00	1	1	0.02	0.024	0.02	0.021	0.02	t	21173	GLTSLFT	Homo sapiens
1	1.00	1	1	0.06	0.06	0.06	0.06	0.06	t	79403	SVGDYG	Mus musculu s
1	1.00	1	1	0.02	0.022	0.02	0.027	0.02	t	107446	IEKTISK	Homo sapiens
1	1.00	1	1	0.04	0.04	0.036	0.04	0.04	t	5670	AWITEDCP VP	Homo sapiens
1	1.00	1	1	0.05	0.052	0.038	0.05	0.05	t	136896	VLHDDLLE A	Homo sapiens
1	1.00	1	1	0.05	0.047	0.043	0.05	0.05	t	79158	ASPYGNA	Oryctola gus cuniculu s

1	1.00	1	1	0.05	0.047	0.043	0.05	0.05	t	79158	ASPYGNA	Oryctolagus cuniculus
1	1.00	1	1	-0.01	-0.012	-0.01	-0.009	-0.014	t	96457	KGTVNLTWSR	Mus musculus
1	1.00	1	1	-0.02	-0.017	-0.024	-0.02	-0.022	t	139198	QARNRPTT	Mus musculus
1	1.00	1	1	0.02	0.02	0.013	0.02	0.021	t	78143	EKIEKTEEPAP	Homo sapiens
1	1.00	1	1	0.01	0.008	0.012	0.009	0.01	t	15807	FFFDIDLLL	Mus musculus
1	1.00	1	1	0.03	0.03	0.03	0.03	0.03	t	9588	DPAFSL	Mus musculus
1	1.00	1	1	0.03	0.03	0.03	0.03	0.03	t	9588	DPAFSL	Mus musculus
1	1.00	1	1	0.05	0.052	0.05	0.057	0.05	t	117613	QEKLQVALGE	Homo sapiens
1	1.00	1	1	0.05	0.052	0.05	0.057	0.05	t	117613	QEKLQVALGE	Homo sapiens
1	1.00	1	1	-0.02	-0.017	-0.025	-0.013	-0.019	t	131994	PAKSAPAPKK	Mus musculus
1	1.00	1	1	0.01	-0.004	0.015	0.01	0.009	t	21990	GRAIN	Mus musculus
1	1.00	1	1	-0.05	-0.05	-0.05	-0.041	-0.05	t	135642	CKNKEKKC	Homo sapiens
1	1.00	1	1	0.08	0.08	0.075	0.08	0.08	t	115035	ASSDSSNTE	Rattus norvegicus
1	1.00	1	1	0	0.002	-0.004	-0.003	-0.002	t	51872	QPLPPTVMFPPQ	Homo sapiens
1	1.00	1	1	0.02	0.016	0.009	0.019	0.02	t	61006	SSFSPNNT	Oryctolagus cuniculus

1	1.00	1	1	0.04	0.038	0.028	0.043	0.042	t	11092	EAKKQVEKALEE	Mus musculus
1	1.00	1	1	0	0	0	0.002	0	t	66311	TSLNLQKDEPN	Equus caballus
1	1.00	1	1	0.04	0.036	0.033	0.039	0.039	t	137867	GEIKFEAVEGAV	Oryctolagus cuniculus
1	1.00	1	1	-0.01	-0.01	-0.017	-0.01	-0.012	t	122486	MILHPDVQRRVQQEIDDVI	Homo sapiens
1	1.00	1	1	0	-0.004	-0.01	-0.001	0	t	26244	IGILGGK	Oryctolagus cuniculus
1	1.00	1	1	-0.01	-0.012	-0.015	-0.006	-0.011	t	13992	ERFSFPR	Mus musculus
1	1.00	1	1	0.03	0.032	0.025	0.037	0.032	t	141604	APPDDGPP	Homo sapiens
1	1.00	1	1	0.01	0.01	-0.005	0.007	0.011	t	52043	QQIPQQQ	Homo sapiens
1	1.00	1	1	0.02	0.018	0.02	0.019	0.02	t	69883	VLYQEFDE	Mus musculus
1	1.00	1	1	-0.01	-0.012	-0.02	-0.011	-0.012	t	5429	AVLFFGRCVS	Mus musculus
1	1.00	1	1	0	0	-0.007	0	-0.001	t	119496	MFPEVKEKGMAAL	Homo sapiens
1	1.00	1	1	0	-0.002	0.002	-0.001	0	t	40755	LYFP	Mus musculus
1	1.00	1	1	0.02	0.02	0.018	0.02	0.02	t	62286	SVPNP	Mus musculus
1	1.00	1	1	0.04	0.042	0.029	0.043	0.041	t	111585	NKSEVPLDICTSICKYPDYI	Cavia porcellus
1	1.00	1	1	0.02	0.02	0.02	0.02	0.018	t	51296	QLCQLL	Mus musculus

1	1.00	1	1	-0.01	-0.01	-0.019	-0.01	-0.01	t	66101	TRSQSGLTFA	Oryctolagus cuniculus
1	1.00	1	1	0.01	0.01	0.01	0.01	0.01	t	98938	PQP	Mus musculus
1	1.00	1	1	0.03	0.028	0.021	0.029	0.03	t	98408	QYEADLAAVK	Mus musculus
1	1.00	1	1	-0.06	-0.062	-0.06	-0.053	-0.06	t	123244	GMIPPPGL	Homo sapiens
1	1.00	1	1	0.01	0.008	0.015	0.009	0.008	t	25002	HTVTTTTK	Mus musculus
1	1.00	1	1	-0.03	-0.034	-0.034	-0.03	-0.03	t	47859	PHWA	Mus musculus
1	1.00	1	1	-0.02	-0.018	-0.026	-0.019	-0.02	t	71687	VVHGCPLPPTKAPP	Homo sapiens
1	1.00	1	1	-0.02	-0.018	-0.026	-0.019	-0.02	t	71687	VVHGCPLPPTKAPP	Homo sapiens
1	1.00	1	1	-0.01	-0.006	-0.01	-0.003	-0.01	t	144101	TMMQTMSFLRGF	Homo sapiens
1	1.00	1	1	-0.02	-0.018	-0.031	-0.017	-0.02	t	110602	GLKAKPKFTLGKRKATPTT	Homo sapiens
1	1.00	1	1	-0.02	-0.016	-0.025	-0.017	-0.022	t	118144	SLCQSLRER	Homo sapiens
1	1.00	1	1	0	0	0	0	-0.002	t	133722	TSKSKYPLVV	Mus musculus
1	1.00	1	1	-0.02	-0.02	-0.034	-0.02	-0.02	t	9523	DNNYGKTIINNDFNF	Mus musculus

1	1.00	1	1	-0.02	-0.018	-0.022	-0.013	-0.02	t	132078	SQGWGDPPKSNQSLG	Homo sapiens
1	1.00	1	1	0.01	0.01	0.012	0.01	0.007	t	7230	CTTPAQGNSMFPSC	Mus musculus
1	1.00	1	1	0.01	0.012	0.004	0.011	0.009	t	133618	KKLLVRGLYRISFTTYK	Homo sapiens
1	1.00	1	1	0.09	0.09	0.09	0.09	0.09	t	139771	GYSFSTS	Ovis aries
1	1.00	1	1	0.01	0.008	-0.004	0.009	0.01	t	48108	PKNTLTPLEELYPTN	Mus musculus
1	1.00	1	1	0.02	0.023	0.013	0.02	0.02	t	150858	ISSLYNLVKSS	Mus musculus
1	1.00	1	1	-0.01	-0.008	-0.003	-0.003	-0.01	t	141969	NGTYQTW	Homo sapiens
1	1.00	1	1	0.01	0.014	0.012	0.011	0.007	t	103170	FGDVDAHGAMIRAQA	Homo sapiens
1	1.00	1	1	0.06	0.06	0.053	0.06	0.061	t	78255	QTTPEEKPAEP	Homo sapiens
1	1.00	1	1	0.06	0.06	0.06	0.06	0.06	t	141067	PLSLEPDP	Mus musculus
1	1.00	1	1	0.03	0.03	0.03	0.03	0.03	t	78637	EKSLTSLSE	Oryctolagus cuniculus
1	1.00	1	1	0.01	0.01	0.01	0.01	0.009	t	117232	SAPDTRPAP	Mus musculus
1	1.00	1	1	0.03	0.034	0.026	0.03	0.03	t	112486	DTYRF	Cavia porcellus
1	1.00	1	1	0.03	0.028	0.026	0.03	0.03	t	112486	DTYRF	Oryctolagus cuniculus
1	1.00	1	1	-0.01	-0.01	-0.024	-0.01	-0.01	t	133952	WIFQNF	Mus musculus

1	1.00	1	1	0.01	0.012	0.003	0.007	0.01	t	58882	SKLIKIFQDH	Homo sapiens
1	1.00	1	1	0.02	0.02	0.015	0.026	0.021	t	107064	AGAPGFPG	Rattus norvegicus
1	1.00	1	1	0.03	0.03	0.023	0.036	0.03	t	125357	AEIETDKATIGFEVQEEG	Homo sapiens
1	1.00	1	1	0	0	0	0.006	-0.001	t	53195	RARGRGRGRGEKRP	Homo sapiens
1	1.00	1	1	0	0	0	0.006	-0.001	t	55585	RRGRGRERARGGSRE	Homo sapiens
1	1.00	1	1	0.01	0.012	0.003	0.007	0.007	t	106977	VQGKEKEPSK	Homo sapiens
1	1.00	1	1	0.04	0.043	0.028	0.04	0.042	t	95416	GLQNDP	Mus musculus
1	1.00	1	1	0.01	0.012	0.01	0.01	0.008	t	3274	ANETIYNTTLKYGDV	MD
1	1.00	1	1	0.01	0.014	0.005	0.01	0.009	t	24602	HQQILLQKYQVIKV	Capra hircus
1	1.00	1	1	0.05	0.045	0.042	0.053	0.052	t	157743	PLATQSYGFGSD	Canis lupus
1	1.00	1	1	0.02	0.02	0.02	0.02	0.02	t	50098	PYIEQAQVI	Homo sapiens
1	1.00	1	1	0.04	0.042	0.039	0.043	0.039	t	47858	PHVVDL	Homo sapiens
1	1.00	1	1	0	-0.003	0	0	-0.002	t	73489	YCDKLCKDKKADSGY	Oryctolagus cuniculus
1	1.00	1	1	0	-0.004	-0.009	-0.001	0	t	596	ACSFSSIPNG	Oryctolagus cuniculus
1	1.00	1	1	0.02	0.021	0.019	0.023	0.019	t	99579	QNIRQGAKEPYPEFV	Equus caballus

1	1.00	1	1	0	-0.003	-0.013	0	0	t	61827	STPPLPWP WSPAAL	Oryctolagus cuniculus
1	1.00	1	1	0	-0.003	-0.013	0	0	t	61827	STPPLPWP WSPAAL	Oryctolagus cuniculus
1	1.00	1	1	0.01	0.01	0.004	0.009	0.01	t	39254	LRYLA	Homo sapiens
1	1.00	1	1	-0.03	-0.033	-0.032	-0.029	-0.033	t	103503	PVIAENRA ELMILIA	Homo sapiens
1	1.00	1	1	0	0	0	0.004	0	t	136063	FIRFDKRIE AEE	Homo sapiens
1	1.00	1	1	0.05	0.046	0.049	0.05	0.048	t	123849	WQDE	Mus musculus
1	1.00	1	1	0.06	0.058	0.05	0.053	0.06	t	118029	NAGG	Mus musculus
1	1.00	1	1	-0.01	-0.01	-0.015	-0.01	-0.01	t	125791	CSFWSKYI QTLK	Mus musculus
1	1.00	1	1	-0.01	-0.008	-0.012	-0.003	-0.012	t	122341	ESGFLREV LNAVPVLL	Homo sapiens
1	1.00	1	1	0.03	0.032	0.025	0.033	0.03	t	50272	QAGQEMI LAVQGVQ D	Homo sapiens
1	1.00	1	1	0.02	0.022	0.013	0.021	0.02	t	69908	VLYSPNVS VPSSSS	Homo sapiens
1	1.00	1	1	-0.04	-0.04	-0.04	-0.04	-0.044	t	26372	IHFG	Mus musculus
1	1.00	1	1	-0.03	-0.027	-0.035	-0.03	-0.032	t	126921	RERRGIAL D	Mus musculus
1	1.00	1	1	0	0	0	0	0	t	47119	PDKEILYEA	Homo sapiens

1	1.00	1	1	0.03	0.032	0.025	0.033	0.03	t	141604	APPDDGPP	Homo sapiens
1	1.00	1	1	-0.01	-0.01	-0.025	-0.007	-0.008	t	53823	RGAPGG	Homo sapiens
1	1.00	1	1	0	0.002	-0.007	0.003	-0.001	t	121589	LRKFKNISYFPGKLR	Homo sapiens
1	1.00	1	1	-0.02	-0.016	-0.016	-0.013	-0.02	t	144700	KEWAKRAALP	Homo sapiens
1	1.00	1	1	0	0	-0.005	-0.002	0	t	62065	SVCGPVYCFT	Homo sapiens
1	1.00	1	1	0.02	0.022	0.016	0.017	0.02	t	112837	SGYIPANTTIPF	Homo sapiens
1	1.00	1	1	0	0	0	0	-0.004	t	56771	SAFDDNLTAANAMGLILNKR	Aotus nancymaae
1	1.00	1	1	0	0	-0.005	0	0	t	60663	SRGAPGGGFVPSLQGVP	Homo sapiens
1	1.00	1	1	-0.02	-0.02	-0.02	-0.011	-0.02	t	135642	CKNKEKKC	Homo sapiens
1	1.00	1	1	0.02	0.02	0.022	0.031	0.02	t	118587	RPRVNYLQDFSYQRS	Homo sapiens
1	1.00	1	1	-0.01	-0.01	-0.017	-0.014	-0.01	t	131249	LVVGLC	Homo sapiens
1	1.00	1	1	0.04	0.04	0.044	0.04	0.038	t	102280	AAANQTEV	Mus musculus
1	1.00	1	1	-0.01	-0.013	-0.021	-0.01	-0.009	t	9472	DNIDSPNAPWVFAC	Myodes glareolus
1	1.00	1	1	-0.03	-0.028	-0.03	-0.023	-0.031	t	120699	KMFPEVKEKGMAALPRL	Homo sapiens
1	1.00	1	1	0.01	0.01	0.002	0.007	0.008	t	13252	ELRNLPQQCGLRAPQ	Homo sapiens
1	1.00	1	1	0.01	0.01	-0.006	0.01	0.013	t	10801	DWEDRYYRE	Mus musculus

1	1.00	1	1	0.01	0.007	-0.006	0.01	0.013	t	10801	DWEDRYYRE	Oryctolagus cuniculus
1	1.00	1	1	0	0.002	0	0.007	0	t	11571	EEEDDDMGFGLFD	Homo sapiens
1	1.00	1	1	0	-0.002	-0.012	-0.003	0	t	95253	AQKKII	Homo sapiens
1	1.00	1	1	-0.01	-0.01	-0.01	-0.01	-0.01	t	79118	WKSLPF	Mus musculus
1	1.00	1	1	0.01	0.012	-0.001	0.011	0.01	t	110589	GDICNTMHYTNWTHIYICEE	Homo sapiens
1	1.00	1	1	0.01	0.01	0.019	0.014	0.01	t	75560	YQTEKFVK	Homo sapiens
1	1.00	1	1	-0.04	-0.04	-0.045	-0.04	-0.041	t	26372	IHFG	Mus musculus
1	1.00	1	1	0.06	0.06	0.06	0.06	0.062	t	127822	SIYAYDNFGV	Homo sapiens
1	1.00	1	1	-0.01	-0.01	-0.01	-0.01	-0.011	t	159294	QPLWLL	Oryctolagus cuniculus
1	1.00	1	1	0.03	0.034	0.031	0.037	0.03	t	127961	ELLADVQFALGLPFYPAYEG	Homo sapiens
1	1.00	1	1	0.01	0.008	0.002	0.009	0.01	t	62831	TAEGRAINRRVE	Mus musculus
1	1.00	1	1	-0.01	-0.007	-0.019	-0.01	-0.011	t	63757	TFTNKLKSKHTDLGK	Mus musculus
1	1.00	1	1	0.01	0.01	-0.003	0.004	0.008	t	95674	PNNLCCSQWG	Homo sapiens
1	1.00	1	1	-0.02	-0.02	-0.015	-0.02	-0.023	t	108382	GGVVIA	Mus musculus

1	1.00	1	1	-0.02	-0.02	-0.015	-0.02	-0.023	t	108382	GGVVIA	Mus musculus
1	1.00	1	1	0.03	0.03	0.03	0.029	0.027	t	9585	DPAF	Mus musculus
1	1.00	1	1	-0.01	-0.008	-0.016	-0.009	-0.012	t	36893	LKILPQSGPIGGIKSMGITT	Homo sapiens
1	1.00	1	1	-0.01	-0.012	-0.011	-0.011	-0.01	t	73301	WYWGPSLYSI	Mus musculus
1	1.00	1	1	-0.02	-0.027	-0.028	-0.02	-0.02	t	110913	KDIKANPENYFT	Macaca
1	1.00	1	1	0	0.001	-0.016	-0.003	0	t	78843	TAAQITAGIALHQSNLN	Canis familiaris
1	1.00	1	1	-0.02	-0.016	-0.02	-0.013	-0.02	t	123512	MPPPGMIP	Homo sapiens
1	1.00	1	1	-0.03	-0.028	-0.025	-0.023	-0.03	t	121892	TALLRNLG	Homo sapiens
1	1.00	1	1	0	0	0	0.003	0	t	15438	FDPLVAEE	Homo sapiens
1	1.00	1	1	0.02	0.02	0.02	0.02	0.02	t	48778	PPFSLSPVPTL	Oryctolagus cuniculus
1	1.00	1	1	0	0.003	0	0	0	t	135457	FGF	Mus musculus
1	1.00	1	1	0.02	0.02	0.007	0.02	0.02	t	67444	TYRATYQD	Homo sapiens
1	1.00	1	1	0.02	0.02	0.019	0.018	0.02	t	32494	KNFNFEVE	Homo sapiens
1	1.00	1	1	0	0.003	-0.008	0	0	t	64644	TKPTDPTGIE	Mus musculus

1	1.00	1	1	0	-0.001	-0.008	0	0	t	64644	TKPTDPTGIE	Mus musculus
1	1.00	1	1	0.01	0.01	-0.003	0.009	0.011	t	146390	KFTFLFCGSMMATG	Homo sapiens
1	1.00	1	1	0.02	0.02	0.016	0.02	0.022	t	150850	GY	Mus musculus
1	1.00	1	1	0	-0.001	0.005	0.001	0	t	73991	YGFQYS	Homo sapiens
1	1.00	1	1	0.01	0.01	0.006	0.01	0.01	t	6093	CDCDRTAA	Mus musculus
1	1.00	1	1	-0.01	-0.013	-0.018	-0.01	-0.011	t	153392	GNTLQCIMLVYCFLGYCC	Oryctolagus cuniculus
1	1.00	1	1	0	0	-0.002	0	-0.002	t	57648	SETLKS	Mus musculus
1	1.00	1	1	0.03	0.026	0.033	0.029	0.028	t	15529	FEFPPPPTDE	Oryctolagus cuniculus
1	1.00	1	1	0.01	0.012	0.01	0.011	0.011	t	39663	LSSKAV	Homo sapiens
1	1.00	1	1	0	0	0	0.004	-0.002	t	117660	TVETQNLEGLHH	Homo sapiens
1	1.00	1	1	-0.03	-0.03	-0.037	-0.024	-0.03	t	107087	ARGLTGRPGDA	Homo sapiens
1	1.00	1	1	0.04	0.038	0.028	0.033	0.039	t	144641	FFENKD	Mus musculus
1	1.00	1	1	0.02	0.02	0.012	0.02	0.02	t	32138	KLRPFERDI	Mus musculus
1	1.00	1	1	0.02	0.018	0.018	0.021	0.018	t	21480	GNNLEDPASRDL	Mus musculus
1	1.00	1	1	0.01	0.013	0.001	0.01	0.01	t	57288	SDSQKECT	Mus musculus

1	1.00	1	1	0.01	0.01	0.01	0.01	0.006	t	19636	GFTTNEERYNVFAE	Mus musculus
1	1.00	1	1	-0.01	-0.006	-0.015	-0.007	-0.01	t	70241	VPAEILRK	Homo sapiens
1	1.00	1	1	0.05	0.048	0.041	0.05	0.05	t	33424	KSLSTLSY	Oryctolagus cuniculus
1	1.00	1	1	0.03	0.026	0.018	0.029	0.03	t	12248	EGQKSAVTEY	Oryctolagus cuniculus
1	1.00	1	1	0.02	0.018	0.02	0.023	0.02	t	75151	YNGEEYLILS	Mus musculus
1	1.00	1	1	0.08	0.08	0.08	0.08	0.08	t	141893	FEEETESSASSES	Mus musculus
1	1.00	1	1	-0.02	-0.018	-0.018	-0.017	-0.022	t	120612	IFNLFSTKDSSAAW	Homo sapiens
1	1.00	1	1	0.02	0.024	0.015	0.021	0.02	t	52814	QVVYDYQH	Homo sapiens
1	1.00	1	1	0.01	0.008	0.011	0.013	0.01	t	142307	TLHEYMLDLQPE	Homo sapiens
1	1.00	1	1	0.01	0.01	0.008	0.01	0.008	t	97282	ESNNL	Oryctolagus cuniculus
1	1.00	1	1	0.01	0.008	0.009	0.01	0.009	t	95092	GYQKTVDHTKVNSK	Oryctolagus cuniculus
1	1.00	1	1	0	0.004	-0.011	0	0.001	t	93328	LSKVVDNYEL	Ovis aries
1	1.00	1	1	0.02	0.02	0.02	0.026	0.02	t	108790	AQGPRGEP	Rattus norvegicus

1	1.00	1	1	0.01	0.007	0.01	0.01	0.007	t	73131	WTGVAQD	Oryctolagus cuniculus
1	1.00	1	1	0.01	0.007	0.01	0.01	0.007	t	73131	WTGVAQD	Oryctolagus cuniculus
1	1.00	1	1	0	0	0	0.006	-0.001	t	11813	EENVEENV	Homo sapiens
1	1.00	1	1	0	-0.004	-0.009	-0.001	0	t	51474	QLPNVGITQGIV	Bos taurus
1	1.00	1	1	0.01	0.014	0.01	0.017	0.01	t	119676	WQQGNVFS	Homo sapiens
1	1.00	1	1	-0.02	-0.022	-0.025	-0.023	-0.023	t	115828	KPAAVRSPAQ	Homo sapiens
1	1.00	1	1	-0.02	-0.02	-0.02	-0.02	-0.023	t	151729	RYSPSLNTRL	Mus musculus
1	1.00	1	1	0.01	0.013	0.001	0.01	0.01	t	96874	SIAPDLSLDFEKLNV	Mus musculus
1	1.00	1	1	-0.02	-0.013	-0.019	-0.021	-0.021	t	55193	RPKPG	Gallus gallus
1	1.00	1	1	0.01	0.014	0.025	0.017	0.009	t	104014	KPQ	Homo sapiens
1	1.00	1	1	-0.08	-0.08	-0.099	-0.077	-0.078	t	96059	AANNAKNAAL	Homo sapiens(human)
1	1.00	1	1	0.02	0.02	0.007	0.019	0.021	t	146516	RAVPYYGPGVDYRN	Homo sapiens
1	1.00	1	1	-0.04	-0.044	-0.046	-0.04	-0.042	t	94979	YNKRFPGFVSP	Oryctolagus cuniculus
1	1.00	1	1	0	0	0	0	0	t	54291	RISNLND	Homo sapiens

1	1.00	1	1	0	0	-0.016	0.002	0.003	t	98976	QYPLGQG SFRPS	Homo sapiens
1	1.00	1	1	0.05	0.05	0.05	0.05	0.05	t	38662	LPQVVM	Homo sapiens
1	1.00	1	1	-0.04	-0.042	-0.035	-0.037	-0.042	t	26360	IH	Mus musculu s
1	1.00	1	1	-0.02	-0.016	-0.013	-0.013	-0.021	t	106491	GWGQLFQ	Homo sapiens
1	1.00	1	1	-0.02	-0.018	-0.025	-0.023	-0.02	t	115090	NLLRFFVA PFPE	Homo sapiens
1	1.00	1	1	0.02	0.021	0.021	0.023	0.02	t	57506	SEKSKCEE NTMFQPY	Equus caballus
1	1.00	1	1	0.01	0.01	0.016	0.01	0.004	t	50959	QGSNWI	Homo sapiens
1	1.00	1	1	0.03	0.03	0.044	0.03	0.026	t	146012	SSPSTPLLY	Mus musculu s
1	1.00	1	1	-0.01	-0.006	-0.005	-0.003	-0.01	t	138205	MHEALHN	Homo sapiens
1	1.00	1	1	0.05	0.054	0.039	0.047	0.05	t	78104	APPASEQE T	Homo sapiens
1	1.00	1	1	-0.01	-0.013	-0.014	-0.01	-0.012	t	45357	NPGISGSR LLGT	Oryctola gus cuniculu s
1	1.00	1	1	0	0	0	0	-0.004	t	79303	LVAAGVD YEDERISF QDWPK	Rattus norvegic us
1	1.00	1	1	0.01	0.012	-0.009	0.007	0.012	t	52028	QQFPQQQ	Homo sapiens
1	1.00	1	1	0.02	0.022	0.02	0.017	0.018	t	106562	KKCDIKTTL P	Homo sapiens
1	1.00	1	1	0.02	0.022	0.02	0.017	0.018	t	106562	KKCDIKTTL P	Homo sapiens

1	1.00	1	1	0.01	0.014	0.001	0.01	0.01	t	113666	NNGLYDIYADDFITDTSTTP	Cavia porcellus
1	1.00	1	1	0.01	0.014	0.001	0.01	0.01	t	113666	NNGLYDIYADDFITDTSTTP	Cavia porcellus
1	1.00	1	1	0	0	-0.012	0	0	t	10126	DSGDGRQTITNP	Mus musculus
1	1.00	1	1	0.02	0.023	0.012	0.02	0.022	t	76268	YVFDKSELTN	Mus musculus
1	1.00	1	1	0.02	0.022	0.015	0.021	0.02	t	142008	SEATGLSSEKITKL	Homo sapiens
1	1.00	1	1	-0.02	-0.022	-0.02	-0.021	-0.02	t	40922	LYREFD	Mus musculus
1	1.00	1	1	-0.01	-0.01	-0.015	-0.011	-0.012	t	31075	KGTMDSGQTKR	Mus musculus
1	1.00	1	1	0.02	0.017	0.015	0.02	0.02	t	56079	RTIGGSQAQT	Oryctolagus cuniculus
1	1.00	1	1	0	0	-0.02	0	0.002	t	11269	ECTGLAWEWWR	Mus musculus
1	1.00	1	1	0.03	0.028	0.021	0.03	0.03	t	100629	VRKQYKRDIQ	Mus musculus
1	1.00	1	1	0.02	0.022	0.007	0.021	0.022	t	110437	TLHEYMLDLQ	Homo sapiens
1	1.00	1	1	0.02	0.017	0.011	0.02	0.019	t	115265	GKDITADNK	Oryctolagus cuniculus
1	1.00	1	1	0	0	-0.005	0	0	t	23185	GVTTSLSYF	Homo sapiens
1	1.00	1	1	-0.01	-0.013	-0.023	-0.01	-0.008	t	96849	SAETIF	Oryctolagus cuniculus

1	1.00	1	1	-0.01	-0.01	-0.023	-0.01	-0.008	t	96849	SAETIF	Mus musculus
1	1.00	1	1	-0.01	-0.01	-0.023	-0.01	-0.008	t	96849	SAETIF	Mus musculus
1	1.00	1	1	0.01	0.01	0.01	0.01	0.01	t	105937	LPNIEADV	Oryctolagus cuniculus
1	1.00	1	1	0.07	0.07	0.065	0.072	0.07	t	28505	ISGDLKTQIDQVEST	Homo sapiens
1	1.00	1	1	-0.02	-0.018	-0.028	-0.017	-0.018	t	110913	KDIKANPENYFT	Homo sapiens
1	1.00	1	1	-0.02	-0.017	-0.029	-0.02	-0.02	t	100385	PKTFQFINDQ	Mus musculus
1	1.00	1	1	0.02	0.022	0.016	0.02	0.019	t	65638	TPLGPPDAEYW	Homo sapiens
1	1.00	1	1	0.01	0.008	0.008	0.013	0.011	t	99643	RRVEIEVK	Mus musculus
1	1.00	1	1	0.03	0.033	0.03	0.03	0.03	t	8355	DGAVQPDGG	Mus musculus
1	1.00	1	1	0.03	0.03	0.03	0.03	0.03	t	8355	DGAVQPDGG	Oryctolagus cuniculus
1	1.00	1	1	0.04	0.026	0.039	0.039	0.04	t	9802	DPVIDLLQ	Macaca mulatta
1	1.00	1	1	-0.01	-0.008	-0.01	-0.003	-0.01	t	151795	APDDRSYKSQY	Homo sapiens
1	1.00	1	1	0.02	0.022	0.018	0.027	0.019	t	118335	EQLRILGEKEAKLAQQSQA	Homo sapiens
1	1.00	1	1	0.04	0.042	0.04	0.047	0.042	t	131048	RQSDGQYQM	Homo sapiens

1	1.00	1	1	0.05	0.047	0.041	0.05	0.051	t	10336	DTFNPVYPYDTE	Mus musculus
1	1.00	1	1	0	0	0	0	-0.003	t	110833	EQFNWVSRLANLTQGEDQYY	Mus musculus
1	1.00	1	1	0.02	0.02	0.02	0.02	0.02	t	60358	SQAAPYIEQ	Homo sapiens
1	1.00	1	1	0.02	0.02	0.02	0.023	0.02	t	149026	ANITQSS	Sus scrofa
1	1.00	1	1	0.01	-0.003	0.005	0.01	0.01	t	19096	GDRADGQPA	Aotus griseimembra
1	1.00	1	1	0.01	-0.003	0.005	0.01	0.01	t	19096	GDRADGQPA	Aotus griseimembra
1	1.00	1	1	0.01	-0.003	0.005	0.01	0.01	t	19096	GDRADGQPA	Aotus griseimembra
1	1.00	1	1	-0.01	-0.013	-0.013	-0.01	-0.013	t	7208	CTSPYDGKYWSMYSRL	Oryctolagus cuniculus
1	1.00	1	1	-0.05	-0.048	-0.058	-0.049	-0.05	t	8216	DEWWKVIKKDVWNVISWVF	Homo sapiens
1	1.00	1	1	-0.02	-0.02	-0.02	-0.02	-0.022	t	78628	DQKPPSR	Oryctolagus cuniculus
1	1.00	1	1	0	-0.002	-0.009	0.001	0	t	155856	SSWSDH	Mus musculus
1	1.00	1	1	0.03	0.034	0.024	0.03	0.03	t	92786	GDGNDIITDS	Ovis aries
1	1.00	1	1	-0.01	-0.013	-0.013	-0.01	-0.012	t	39916	LTGISICNPGSSLC	Oryctolagus cuniculus
1	1.00	1	1	-0.01	-0.013	-0.013	-0.01	-0.012	t	39916	LTGISICNPGSSLC	Oryctolagus cuniculus
1	1.00	1	1	0.02	0.02	0.015	0.02	0.018	t	156903	KNRGIIKTFSN	Mus musculus

1	1.00	1	1	0.01	0.008	0.005	0.011	0.011	t	29939	KASAAC	Homo sapiens
1	1.00	1	1	-0.02	-0.018	-0.027	-0.017	-0.021	t	135902	AVSAHGCLFDRR	Homo sapiens
1	1.00	1	1	-0.02	-0.017	-0.029	-0.02	-0.02	t	99892	ALLHEHYVYA	Mus musculus
1	1.00	1	1	0.01	0.012	0.01	0.013	0.01	t	94685	SICSNNPTCWAI	Homo sapiens
1	1.00	1	1	0	-0.002	-0.01	0.001	0.001	t	9474	DNIGNANIGFGN	Homo sapiens
1	1.00	1	1	0.01	0.01	0.01	0.01	0.008	t	21407	GNDYEDRYYREN	Mus musculus
1	1.00	1	1	0.01	0.013	0.01	0.01	0.008	t	21407	GNDYEDRYYREN	Mus musculus
1	1.00	1	1	0.03	0.033	0.038	0.03	0.027	t	79891	KSADVESDV	Mus musculus
1	1.00	1	1	0.04	0.04	0.04	0.04	0.038	t	107107	EAIQP	Rattus norvegicus
1	1.00	1	1	0.03	0.026	0.02	0.033	0.03	t	48176	PKYEQGGNIV	Oryctolagus cuniculus
1	1.00	1	1	0.02	0.027	0.009	0.02	0.02	t	94621	NPACGPKYSC	Capra hircus
1	1.00	1	1	0.03	0.028	0.031	0.029	0.03	t	35983	LFSLNAPGL	Rattus norvegicus
1	1.00	1	1	-0.04	-0.04	-0.045	-0.04	-0.041	t	26372	IHFG	Mus musculus
1	1.00	1	1	0.02	0.018	0.029	0.019	0.016	t	13675	EPIVGAETF	Mus musculus

1	1.00	1	1	0	-0.007	-0.006	0.007	0.002	t	133798	DKIRGEG MRYIIILD	Homo sapiens
1	1.00	1	1	-0.01	-0.008	-0.007	-0.009	-0.01	t	34278	KVYVGNY DF	Homo sapiens
1	1.00	1	1	0.01	0.012	0.003	0.011	0.009	t	19637	GFTTNEPR YNVFAE	Homo sapiens
1	1.00	1	1	0.01	0.012	0.009	0.011	0.012	t	47426	PESPFA	Homo sapiens
1	1.00	1	1	-0.02	-0.02	-0.007	-0.012	-0.023	t	98824	IIAEKTKIP A	Homo sapiens
1	1.00	1	1	-0.02	-0.02	-0.025	-0.02	-0.021	t	14447	ETKRKDGD	Mus musculu s
1	1.00	1	1	-0.02	-0.017	-0.025	-0.02	-0.021	t	14447	ETKRKDGD	Equus caballus
1	1.00	1	1	0	0	-0.005	-0.003	-0.002	t	5385	AVIRGKKG SG	Homo sapiens
1	1.00	1	1	0.01	0.01	0.01	0.01	0.01	t	135504	VPGVGGL G	Oryctola gus cuniculu s
1	1.00	1	1	0.01	0.007	0.002	0.01	0.008	t	65492	TPADPAAS SSERG	Oryctola gus cuniculu s
1	1.00	1	1	0.01	0.01	0.002	0.01	0.008	t	65492	TPADPAAS SSERG	Mus musculu s
1	1.00	1	1	0	-0.002	-0.004	-0.003	0	t	18953	GDDPLLG N	Anas platyrhy nchos
1	1.00	1	1	0.02	0.018	0.012	0.019	0.02	t	5311	AVEVDPD DVN	Mus musculu s
1	1.00	1	1	0.03	0.03	0.031	0.03	0.03	t	41629	MGSSYG	Homo sapiens

1	1.00	1	1	0.01	0.01	0.006	0.016	0.009	t	67069	TVLGCALP PTPQTP	Homo sapiens
1	1.00	1	1	0	0.008	0.011	0	-0.008	t	24215	HLGLMAM DHLPW	Gallus gallus
1	1.00	1	1	0.01	0.014	-0.001	0.01	0.011	t	93326	LSFSDSNL KD	Ovis aries
1	1.00	1	1	0	0.004	0	0.007	0.001	t	108074	PQVYTLP	Homo sapiens
1	1.00	1	1	0	0.002	0	0.007	0.001	t	108074	PQVYTLP	Homo sapiens
1	1.00	1	1	-0.01	-0.011	-0.012	-0.01	-0.01	t	26222	IGFK	Mus musculu s
1	1.00	1	1	0	0	0	0	0	t	50102	PYIEQGM ML	Homo sapiens
1	1.00	1	1	-0.01	-0.018	-0.022	-0.01	-0.01	t	47131	PDKILTYIA ADHC	Mus musculu s
1	1.00	1	1	0.03	0.032	0.028	0.027	0.028	t	43392	NCSSYA	Homo sapiens
1	1.00	1	1	-0.04	-0.04	-0.047	-0.04	-0.041	t	95041	SLAGKRE MA	Oryctola gus cuniculu s
1	1.00	1	1	-0.04	-0.042	-0.047	-0.04	-0.041	t	95041	SLAGKRE MA	Oryctola gus cuniculu s
1	1.00	1	1	0.02	0.022	0.013	0.021	0.021	t	1022	AEKQKAAE ATKVAE	Homo sapiens
1	1.00	1	1	0.02	0.02	0.025	0.02	0.02	t	127585	ASFEPFSN	Homo sapiens
1	1.00	1	1	0.02	0.022	0.02	0.023	0.018	t	151609	DETPMRG ETRKV	Homo sapiens

1	1.00	1	1	0	-0.002	-0.005	0.001	0	t	123380	KLPGKF	Homo sapiens
1	1.00	1	1	-0.06	-0.06	-0.07	-0.06	-0.061	t	63237	TDSPRNPV TR	Oryctolagus cuniculus
1	1.00	1	1	-0.01	-0.01	-0.01	-0.007	-0.011	t	24105	HITHYFVV PSVS	Homo sapiens
1	1.00	1	1	0.01	0	0.003	0.007	0.01	t	57990	SGAWYYV PLG	Homo sapiens
1	1.00	1	1	-0.01	-0.013	-0.013	-0.01	-0.01	t	48409	PLLRTQF	Oryctolagus cuniculus
1	1.00	1	1	0.04	0.038	0.04	0.037	0.037	t	135864	VNDIFERI	Mus musculus
1	1.00	1	1	-0.01	-0.006	-0.01	-0.003	-0.01	t	103830	CHAVKVG NPSC	Homo sapiens
1	1.00	1	1	0.01	0.006	-0.006	0.013	0.012	t	57911	SFSSIPNGT Y	Oryctolagus cuniculus
1	1.00	1	1	0.02	0.019	0.008	0.021	0.022	t	7901	DEADRYFT YEEP	Bos taurus
1	1.00	1	1	0.08	0.078	0.081	0.083	0.08	t	142335	YEQLNDSS EEED	Homo sapiens
1	1.00	1	1	0	-0.002	-0.011	-0.001	0	t	12216	EGLKEVIN DRYDMLN	Mus musculus
1	1.00	1	1	-0.05	-0.052	-0.06	-0.047	-0.05	t	72924	WPAGAGR R	Anas platyrhynchos
1	1.00	1	1	-0.03	-0.027	-0.034	-0.03	-0.03	t	65653	TPLQCTM	Mus musculus
1	1.00	1	1	0	0	0	0	-0.004	t	106221	AAVTPEER	Mus musculus

1	1.00	1	1	0	0	0	0	-0.004	t	116286	CGKGLSATVTGGQKGRGSR	Oryctolagus cuniculus
1	1.00	1	1	0	0	0	0	-0.004	t	28299	IRGDTFATR	Mus musculus
1	1.00	1	1	-0.01	-0.008	0.005	-0.013	-0.019	t	45770	NSARVGTSSCRI	Homo sapiens
1	1.00	1	1	0	0.004	0	0.001	0	t	5380	AVILLMCAVHPTLV	Homo sapiens
1	1.00	1	1	0	0	0	0.009	0	t	119474	KIRY	Homo sapiens
1	1.00	1	1	-0.02	-0.02	-0.036	-0.018	-0.017	t	98976	QYPLGQGSFRPS	Homo sapiens
1	1.00	1	1	-0.03	-0.032	-0.027	-0.031	-0.03	t	122138	LKKDV	Mus musculus
1	1.00	1	1	0	-0.004	-0.004	0.003	0.002	t	106146	TQFAGKDL	Oryctolagus cuniculus
1	1.00	1	1	0.06	0.052	0.06	0.06	0.06	t	156756	SGPSYTT	Mus musculus
1	1.00	1	1	0	0	0	0	-0.002	t	140247	KAAESEIEKLTTK	Oryctolagus cuniculus
1	1.00	1	1	-0.02	-0.016	-0.02	-0.013	-0.02	t	123244	GMIPPPGL	Homo sapiens
1	1.00	1	1	0.06	0.06	0.06	0.06	0.06	t	144898	GQSRGQSRG	Homo sapiens
1	1.00	1	1	-0.02	-0.018	-0.02	-0.017	-0.02	t	73298	WYTTVPKYVATQGYL	Homo sapiens
1	1.00	1	1	0	0	-0.003	0.003	0	t	32809	KPNNDFHFEVFN	Homo sapiens

1	1.00	1	1	0.03	0.027	0.023	0.03	0.028	t	116974	FKEQFLDGDGW	Oryctolagus cuniculus
1	1.00	1	1	-0.01	-0.024	-0.021	-0.013	-0.01	t	28607	ISMDVNKS	Macaca mulatta
1	1.00	1	1	0	0	-0.008	0.006	0.002	t	72259	WARPDYNP	Homo sapiens
1	1.00	1	1	-0.01	-0.003	-0.011	-0.011	-0.012	t	27720	INPSPFAQTR	Gallus gallus
1	1.00	1	1	0.01	0.012	0.01	0.017	0.01	t	107478	KEYKCKV	Homo sapiens
1	1.00	1	1	0.01	0.014	0.01	0.017	0.01	t	107478	KEYKCKV	Homo sapiens
1	1.00	1	1	0.04	0.04	0.04	0.04	0.039	t	143362	ETPLMKE	Mus musculus
1	1.00	1	1	0.04	0.04	0.04	0.049	0.042	t	134253	VFFSGYTFKHKMVYEDTLTL	Homo sapiens
1	1.00	1	1	0.11	0.112	0.105	0.107	0.11	t	96171	DEEDKEEDEE	Homo sapiens
1	1.00	1	1	0.03	0.038	0.023	0.03	0.028	t	106495	GYEVHHQKLVF	Gallus gallus
1	1.00	1	1	-0.01	-0.014	-0.005	-0.007	-0.012	t	95752	RAGFITELSEFI	Oryctolagus cuniculus
1	1.00	1	1	0.04	0.04	0.026	0.037	0.04	t	52043	QQIPQQQ	Homo sapiens
1	1.00	1	1	0.03	0.03	0.022	0.026	0.03	t	110964	PNLKLNNGTDYK	Homo sapiens
1	1.00	1	1	0.02	0.02	0.02	0.02	0.02	t	140916	VSYGEEKPAVLG	Oryctolagus cuniculus

1	1.00	1	1	0.02	0.02	0.003	0.02	0.022	t	114125	HYGSLPQKSQRSQDENPV	Rattus norvegicus
1	1.00	1	1	0.03	0.03	0.017	0.03	0.03	t	5609	AVVYNNEGTNVELG	Mus musculus
1	1.00	1	1	0.02	0.02	0.021	0.02	0.02	t	53145	RALEDGINFA	Homo sapiens
1	1.00	1	1	-0.03	-0.03	-0.035	-0.03	-0.03	t	117601	PKKKRKV	Homo sapiens
1	1.00	1	1	-0.03	-0.03	-0.035	-0.03	-0.03	t	117601	PKKKRKV	Homo sapiens
1	1.00	1	1	0.03	0.03	0.03	0.03	0.03	t	105715	DLESIKGT	Oryctolagus cuniculus
1	1.00	1	1	0.03	0.023	0.03	0.03	0.031	t	114008	VSLVEETSFI	Oryctolagus cuniculus
1	1.00	1	1	-0.02	-0.018	-0.027	-0.017	-0.02	t	138807	RLQGISPKI	Homo sapiens
1	1.00	1	1	0.04	0.042	0.037	0.043	0.04	t	107800	DSRGNPTVE	Homo sapiens
1	1.00	1	1	0.01	0.008	0	0.009	0.01	t	37354	LLGNQSLL	Anas platyrhynchos
1	1.00	1	1	0.02	0.016	0.014	0.019	0.018	t	57184	SDEVRKHTYNLTIA	Oryctolagus cuniculus
1	1.00	1	1	0	-0.002	0	-0.001	0	t	64418	TIPPSSPP	Anas platyrhynchos
1	1.00	1	1	0.02	0.02	0.015	0.02	0.021	t	114224	PQDENPVVHF	Mus musculus
1	1.00	1	1	0.02	0.022	0.016	0.027	0.02	t	11035	EAESISSSEEIVPNSVEQKH	Homo sapiens
1	1.00	1	1	0.02	0.018	0.014	0.019	0.02	t	29703	IYTINDKILS	Mus musculus

1	1.00	1	1	0	0.002	0.005	0.007	0	t	134073	GRTCPKPDDLP	Homo sapiens
1	1.00	1	1	-0.04	-0.038	-0.04	-0.033	-0.04	t	134764	PTPGPSRRGP	Homo sapiens
1	1.00	1	1	0.01	0.003	0.003	0.01	0.01	t	159296	SFSKPSNSWFPVEGP	Oryctolagus cuniculus
1	1.00	1	1	-0.01	-0.012	-0.014	-0.013	-0.01	t	1118	AEPLLSC	Mus musculus
1	1.00	1	1	-0.01	-0.01	-0.01	-0.01	-0.012	t	140460	EKLKHSM	Mus musculus
1	1.00	1	1	0.03	0.03	0.03	0.03	0.03	t	32683	KPDYEPPV	Homo sapiens
1	1.00	1	1	-0.02	-0.018	-0.031	-0.017	-0.02	t	110602	GLKAKPKFTLGKRKATPTT	Homo sapiens
1	1.00	1	1	-0.03	-0.032	-0.03	-0.023	-0.03	t	123643	PYMPPPGM	Homo sapiens
1	1.00	1	1	0.07	0.068	0.056	0.067	0.07	t	53957	RGQSATATYTNLQNSYYNG	Mus musculus
1	1.00	1	1	0	0.003	0	0.007	0	t	118059	PPPGMRP	Mus musculus
1	1.00	1	1	0	-0.002	0	0.003	-0.003	t	137395	EESEDEKRRWGQRDN	Mus musculus
1	1.00	1	1	0	-0.004	0	0.003	-0.003	t	111682	QNFGLFYPNKQPVYQ	Oryctolagus cuniculus
1	1.00	1	1	0	-0.004	0	0.003	-0.003	t	111970	YNQNLIRHVGGGTPR	Oryctolagus cuniculus
1	1.00	1	1	0	-0.004	0	0.003	-0.003	t	111593	NNIARMRLYDPNQAA	Oryctolagus cuniculus

1	1.00	1	1	0	-0.004	0	0.003	-0.003	t	94838	GTSSCRLVP	Oryctolagus cuniculus
1	1.00	1	1	0	-0.002	0	0.003	-0.003	t	56807	SAHGTSTGVRGP	Mus musculus
1	1.00	1	1	0	-0.002	0	0.003	-0.003	t	1367	AFNQFGPNAGQR	Mus musculus
1	1.00	1	1	0.02	0.02	0.02	0.02	0.018	t	122569	SFQDENFVFEEF	Rattus norvegicus
1	1.00	1	1	0.02	0.018	0.015	0.023	0.02	t	125565	STDDYASFSRAL	Mus musculus
1	1.00	1	1	0	-0.002	0.001	-0.001	0	t	56788	SAFVFPTKD	Mus musculus
1	1.00	1	1	-0.02	-0.02	-0.033	-0.023	-0.02	t	70490	VPTAVYFSEK	Homo sapiens
1	1.00	1	1	-0.01	-0.01	-0.021	-0.01	-0.01	t	14210	ESMAGKREMV	Mus musculus
1	1.00	1	1	0.02	0.018	0.022	0.02	0.019	t	112123	GERGAAGIAGPK	Mus musculus
1	1.00	1	1	0.01	0.01	-0.002	0.01	0.01	t	120839	NLTEFQHAFS	Mus musculus
1	1.00	1	1	0.03	0.033	0.023	0.03	0.032	t	113139	QSYKFIPALE	Mus musculus
1	1.00	1	1	-0.06	-0.059	-0.071	-0.06	-0.061	t	11370	EDKLADYKRRMADA	Myodes glareolus
1	1.00	1	1	0.04	0.025	0.032	0.039	0.04	t	12125	EGAPPA	Marmota monax
1	1.00	1	1	0.01	0.014	-0.003	0.01	0.01	t	10551	DVDGINYWTKAITGGGEFTD	Ovis aries
1	1.00	1	1	0.01	0.007	-0.003	0.01	0.01	t	10551	DVDGINYWTKAITGGGEFTD	Oryctolagus cuniculus

1	1.00	1	1	-0.01	-0.006	-0.019	-0.01	-0.011	t	113660	NIPLVSGPDIPINITDQAPS	Cavia porcellus
1	1.00	1	1	-0.01	-0.008	-0.015	-0.007	-0.01	t	48828	PPLPPV	Homo sapiens
1	1.00	1	1	0.03	0.033	0.03	0.03	0.03	t	131487	IPDRFSG	Mus musculus
1	1.00	1	1	0	-0.002	0	0.001	0	t	49296	PSAPPLPHVV	Homo sapiens
1	1.00	1	1	-0.02	-0.018	-0.027	-0.017	-0.02	t	138807	RLQGISPKI	Homo sapiens
1	1.00	1	1	-0.01	-0.01	-0.014	-0.013	-0.012	t	14609	ETYKFIPSLEAA	Homo sapiens
1	1.00	1	1	0.03	0.029	0.022	0.031	0.03	t	62967	TAQAAPVQEG	Homo sapiens
1	1.00	1	1	0	0	0	0.004	-0.002	t	118185	TSQFTGLKC	Homo sapiens
1	1.00	1	1	0.01	0.01	0.01	0.01	0.008	t	21868	GQGGSHSQWNKP	Oryctolagus cuniculus
1	1.00	1	1	0.01	0.01	0	0.01	0.011	t	50609	QEGVQQEGA	Mus musculus
1	1.00	1	1	0	-0.005	-0.006	0	-0.002	t	71545	VTV	Bos taurus
1	1.00	1	1	0	0	0	0.003	0	t	54431	RKTKRNTN	Homo sapiens
1	1.00	1	1	0.03	0.03	0.021	0.03	0.03	t	17531	FQSFPSGEPSNSKLSGFFQS	Homo sapiens
1	1.00	1	1	0	0.002	0	0.006	-0.001	t	3274	ANETIYNTTLKYGDV	MD
1	1.00	1	1	0.02	0.02	0.023	0.02	0.015	t	19617	GFSGNMVETEDTKEP	Mus musculus

1	1.00	1	1	0.02	0.02	0.023	0.02	0.015	t	19617	GFSGNMV ETEDTKEP	Mus musculu s
1	1.00	1	1	0	0	-0.013	0	0	t	158923	KSCTWPET HTLWGDA V	Mus musculu s
1	1.00	1	1	-0.02	-0.014	-0.026	-0.02	-0.02	t	123437	LKMWSDF LG	Ovis aries
1	1.00	1	1	0.02	0.017	0.008	0.02	0.02	t	32	AAAETGV GVIKSIAP	Oryctola gus cuniculu s
1	1.00	1	1	0.03	0.032	0.032	0.03	0.027	t	118577	RCPAKPEE EGMMINI	Homo sapiens
1	1.00	1	1	0	0	0	0	-0.002	t	15205	FAKY	Mus musculu s
1	1.00	1	1	-0.02	-0.034	-0.026	-0.021	-0.02	t	33661	KTFESYRV	Macaca mulatta
1	1.00	1	1	-0.02	-0.02	-0.02	-0.013	-0.02	t	135993	SGGLRLLL CLLL	Homo sapiens
1	1.00	1	1	0.07	0.07	0.07	0.073	0.07	t	149035	EGSGTTK	Sus scrofa
1	1.00	1	1	-0.02	-0.016	-0.03	-0.019	-0.02	t	12388	EIAKTTLLK	Homo sapiens
1	1.00	1	1	0	-0.003	0.002	0	-0.001	t	38747	LPVCPL	Oryctola gus cuniculu s
1	1.00	1	1	-0.01	-0.014	-0.011	-0.011	-0.014	t	1257	AFASNRTF DITVSGLG TAPK	Oryctola gus cuniculu s
1	1.00	1	1	-0.02	-0.018	-0.02	-0.019	-0.022	t	96102	APPLLPH	Homo sapiens
1	1.00	1	1	-0.01	-0.012	-0.02	-0.007	-0.009	t	69183	VKAVTTLL KA	Mus musculu s

1	1.00	1	1	-0.02	-0.024	-0.026	-0.017	-0.021	t	7558	DAKDGKEI DTKHLPST C	Oryctola gus cuniculu s
1	1.00	1	1	0	0.004	0	0	-0.003	t	25645	IDPRGKM V	MD
1	1.00	1	1	0.02	0.018	0.02	0.017	0.018	t	69025	VILFVVIVG	Mus musculu s
1	1.00	1	1	0.04	0.04	0.033	0.04	0.039	t	57747	SFEEDTEK DK	Oryctola gus cuniculu s
1	1.00	1	1	0.04	0.04	0.033	0.04	0.039	t	57747	SFEEDTEK DK	Oryctola gus cuniculu s
1	1.00	1	1	-0.01	-0.007	-0.019	-0.01	-0.01	t	100288	LSKINQPY QK	Mus musculu s
1	1.00	1	1	-0.02	-0.013	-0.009	-0.02	-0.024	t	141052	LRDIFAWE P	Ovis aries
1	1.00	1	1	0.04	0.04	0.031	0.04	0.04	t	135941	ITTQECAN VVVG	Homo sapiens
1	1.00	1	1	0.02	0.018	0.013	0.02	0.022	t	45303	NPASTTNK DK	Mus musculu s
1	1.00	1	1	0.01	0.01	0.01	0.013	0.012	t	45392	NPLLVET W	Homo sapiens
1	1.00	1	1	0	0	0	0.008	0	t	38810	LQAMRKY SPFRNGY MEPTLG	Homo sapiens
1	1.00	1	1	0	0	0	0.008	0	t	47984	PISLLFNEK EADDNDH EPQI	Homo sapiens
1	1.00	1	1	0	0	0	0.008	0	t	60062	SPGGLEPP SEKHFRET EV	Homo sapiens

1	1.00	1	1	0	0	0	0.008	0	t	23722	HEPQISPGGLEPPSEKHFRE	Homo sapiens
1	1.00	1	1	0.02	0.018	0.008	0.021	0.023	t	70384	VPNLQGVPESPFSRT	Homo sapiens
1	1.00	1	1	-0.03	-0.026	-0.041	-0.027	-0.03	t	18053	FTQCSMNVVSKTLFRKSPIY	Homo sapiens
1	1.00	1	1	0.02	0.02	0.02	0.02	0.018	t	78703	KKKKTDEAQP	Mus musculus
1	1.00	1	1	0.01	0.01	0.005	0.01	0.01	t	134087	IGTLCADAR	Oryctolagus cuniculus
1	1.00	1	1	0.01	0.01	0.01	0.01	0.008	t	6455	CITQYERESQAYY	Mus musculus
1	1.00	1	1	0.02	0.02	0.03	0.02	0.017	t	26024	IEYKVQFATK	Mus musculus
1	1.00	1	1	0.02	0.02	0.012	0.02	0.02	t	19269	GEGAPPAKRARTDQME	Homo sapiens
1	1.00	1	1	-0.01	-0.008	-0.007	-0.009	-0.01	t	59112	SLFTPGP	Homo sapiens
1	1.00	1	1	0.03	0.03	0.022	0.03	0.03	t	106302	CDIKTTLPPITA	Homo sapiens
1	1.00	1	1	-0.02	-0.024	-0.019	-0.021	-0.022	t	44276	NILISLTDLEIW	Oryctolagus cuniculus
1	1.00	1	1	0.02	0.022	0.013	0.021	0.019	t	137828	DVLLAGNAEADR	Homo sapiens
1	1.00	1	1	0.02	0.01	0.013	0.021	0.019	t	137828	DVLLAGNAEADR	Homo sapiens
1	1.00	1	1	0.02	0.02	0.009	0.02	0.021	t	125176	NNSTGDFEL	Mus musculus

1	1.00	1	1	-0.01	-0.008	-0.014	-0.002	-0.011	t	1219	AEVLKDAIKDLVMTKPAPTC	Homo sapiens
1	1.00	1	1	0.05	0.05	0.05	0.056	0.05	t	118533	NEEAGDGTTTATV	Homo sapiens
1	1.00	1	1	0	-0.002	0	-0.001	0	t	46969	PATIGDYTDF	Mus musculus
1	1.00	1	1	-0.01	-0.008	-0.015	-0.01	-0.008	t	21055	GLPAA	Homo sapiens
1	1.00	1	1	0	-0.002	0	0.001	0	t	49296	PSAPPLPHVV	Homo sapiens
1	1.00	1	1	0.06	0.06	0.06	0.063	0.06	t	149102	SGPNATQ	Sus scrofa
1	1.00	1	1	-0.01	-0.008	-0.017	-0.011	-0.01	t	135804	YTNIFLKF	Homo sapiens
1	1.00	1	1	0.04	0.033	0.04	0.04	0.04	t	113894	STIDPAEEIE	Oryctolagus cuniculus
1	1.00	1	1	-0.02	-0.02	-0.02	-0.02	-0.023	t	106976	VPTQLYNLYPPL	Mus musculus
1	1.00	1	1	0.02	0.018	0.02	0.019	0.02	t	46353	NVDPN	Mus musculus
1	1.00	1	1	0	0	0	0.004	-0.002	t	118381	GKTCPKEIPKGSKNT	Homo sapiens
1	1.00	1	1	0	0	0	0.004	-0.002	t	117246	VTWEVLEGEVEKEALKKI	Homo sapiens
1	1.00	1	1	0	0.002	0	0	0	t	69125	VITGTI	Homo sapiens
1	1.00	1	1	0	-0.007	0	0	0	t	69125	VITGTI	Homo sapiens
1	1.00	1	1	0.01	0.01	0.01	0.013	0.009	t	42696	MSTNPKPQRKTKRNT	Homo sapiens

1	1.00	1	1	0	0.002	-0.007	-0.003	-0.002	t	118387	GPKGRTVII EQSW	Homo sapiens
1	1.00	1	1	-0.06	-0.056	-0.06	-0.053	-0.062	t	122748	EHRMTW DPAQPPR	Homo sapiens
1	1.00	1	1	0.02	0.022	0.023	0.021	0.022	t	45033	NMTVM	Homo sapiens
1	1.00	1	1	-0.01	-0.01	-0.01	-0.01	-0.013	t	3399	ANRAVKP TGSAAIGL	Homo sapiens
1	1.00	1	1	0.02	0.018	0.02	0.027	0.02	t	122996	AAFQQGKI PP	Homo sapiens
1	1.00	1	1	0	-0.01	-0.007	0.001	-0.001	t	137892	HGHGAFR QQFQY	Homo sapiens
1	1.00	1	1	0	0.002	-0.007	0.001	-0.001	t	137892	HGHGAFR QQFQY	Homo sapiens
1	1.00	1	1	0.01	0.009	0.01	0.008	0.008	t	65796	TPVCQFD GNTIS	Myodes glareolu s
1	1.00	1	1	0.02	0.02	0.015	0.027	0.021	t	119749	ALQEACEA YLVGLF	Mus musculu s
1	1.00	1	1	0.03	0.032	0.032	0.037	0.03	t	125320	YDGPR	Homo sapiens
1	1.00	1	1	0	0.005	-0.004	0.003	0	t	77801	SVKKCLFE KC	Homo sapiens
1	1.00	1	1	0.02	0.02	0.02	0.02	0.02	t	60358	SQAAPYIE Q	Homo sapiens
1	1.00	1	1	0.01	0.01	0.01	0.01	0.007	t	70389	VPNLRGDL QVLAQKV A	Cavia porcellu s
1	1.00	1	1	0.01	0.01	0.01	0.01	0.007	t	70389	VPNLRGDL QVLAQKV A	Cavia porcellu s

1	1.00	1	1	0	0.004	-0.012	0.001	0	t	55706	RRRAGRCP	Homo sapiens
1	1.00	1	1	-0.01	-0.008	-0.004	-0.009	-0.01	t	744	ADPPFS	Homo sapiens
1	1.00	1	1	-0.06	-0.063	-0.065	-0.06	-0.063	t	132846	KAKRLHLS PVRLAE	Oryctolagus cuniculus
1	1.00	1	1	0.04	0.038	0.045	0.043	0.04	t	138765	EEIKMDIS KLNA	Mus musculus
1	1.00	1	1	0	0	0	0	-0.004	t	125212	QHWSYGL RPGQHWS YGLRPG	Sus scrofa
1	1.00	1	1	0.08	0.08	0.08	0.077	0.078	t	135570	LFDASDSS S	Oryctolagus cuniculus
1	1.00	1	1	0.03	0.03	0.03	0.03	0.031	t	115342	LDTDYKKY LL	Homo sapiens
1	1.00	1	1	0	0.004	-0.009	0	0	t	112443	ACQKHTP PAP	Cavia porcellus
1	1.00	1	1	0	0.004	-0.009	0	0	t	112443	ACQKHTP PAP	Cavia porcellus
1	1.00	1	1	0.04	0.038	0.038	0.037	0.04	t	38463	LPET	Mus musculus
1	1.00	1	1	0	0	0	0	-0.004	t	21408	GNDYEDR YYRENMY RYPNQ	Mus musculus
1	1.00	1	1	0.02	0.022	0.013	0.021	0.02	t	151032	LYQNADTY VFVGSSR	Homo sapiens
1	1.00	1	1	0	-0.002	-0.013	-0.001	0	t	65514	TPAYYTKD TNNNLTLV	Mus musculus
1	1.00	1	1	0.06	0.062	0.055	0.057	0.058	t	98682	AEPEQSLA CQ	Homo sapiens
1	1.00	1	1	0.01	0.01	0.01	0.013	0.01	t	149028	AVTNATQ	Sus scrofa

1	1.00	1	1	0.05	0.054	0.042	0.051	0.05	t	3893	APVQEGVQQE	Homo sapiens
1	1.00	1	1	0.05	0.052	0.042	0.051	0.05	t	3893	APVQEGVQQE	Homo sapiens
1	1.00	1	1	0.04	0.04	0.04	0.04	0.041	t	57504	SEKSEEIN	Oryctolagus cuniculus
1	1.00	1	1	-0.01	-0.008	-0.01	-0.003	-0.01	t	107660	VLHQNWL	Homo sapiens
1	1.00	1	1	0	0.002	-0.014	0.001	0	t	18025	FTNKLKEKHTDLG	Homo sapiens
1	1.00	1	1	-0.01	-0.012	-0.012	-0.007	-0.009	t	99774	VVLGYTDR	Mus musculus
1	1.00	1	1	0.01	0.012	0	0.013	0.01	t	6702	CNKKGEQVGMSRPGL	Homo sapiens
1	1.00	1	1	0.04	0.037	0.035	0.04	0.041	t	50872	QGGNIVDIDFDSVP	Oryctolagus cuniculus
1	1.00	1	1	0	0.004	-0.005	0	-0.002	t	57766	SFFSYGEI	MD
1	1.00	1	1	-0.01	-0.012	-0.02	-0.013	-0.01	t	49993	PVVAESPKKP	Mus musculus
1	1.00	1	1	0.02	0.024	0.013	0.027	0.021	t	102441	FRERQSSKNLLSCENSDRDA	Homo sapiens
1	1.00	1	1	-0.02	-0.02	-0.02	-0.02	-0.023	t	151729	RYSPSLNTRL	Mus musculus
1	1.00	1	1	0.01	0.007	0.01	0.01	0.008	t	30299	KEALDKYELENHD	Oryctolagus cuniculus
1	1.00	1	1	-0.03	-0.027	-0.038	-0.03	-0.03	t	38434	LPDKFNAYLANKWV	Mus musculus

1	1.00	1	1	0.03	0.021	0.027	0.03	0.03	t	45628	NQSLLETH	Anas platyrhynchos
1	1.00	1	1	0.06	0.053	0.058	0.06	0.06	t	18105	FTYEEPNDGEER	Bos taurus
1	1.00	1	1	0.06	0.061	0.058	0.06	0.06	t	18105	FTYEEPNDGEER	Capra hircus
1	1.00	1	1	-0.02	-0.02	-0.025	-0.02	-0.021	t	112507	FAVLKLMGRGTK	Rattus norvegicus
1	1.00	1	1	0.02	0.02	0.02	0.02	0.018	t	78479	PETLPYL	Mus musculus
1	1.00	1	1	-0.02	-0.022	-0.04	-0.027	-0.02	t	128043	LDAMPAGNPAFPVIPG	Mus musculus
1	1.00	1	1	0.03	0.018	0.028	0.03	0.029	t	137827	DVKSAIEG	Rattus norvegicus
1	1.00	1	1	-0.02	-0.02	-0.025	-0.02	-0.023	t	58465	SICSNNPTCWAICKR	Mus musculus
1	1.00	1	1	0	-0.002	0	0.001	-0.001	t	130449	CFFPNLSYC	Mus musculus
1	1.00	1	1	0.02	0.022	0.013	0.021	0.019	t	18743	GAQLNAMKPDLQNTL	Homo sapiens
1	1.00	1	1	0.03	0.03	0.026	0.04	0.033	t	106738	QKRAAYDQYGHAAFE	Homo sapiens
1	1.00	1	1	0	0	0	0.008	0	t	117773	CNSRQTDREDELI	Homo sapiens
1	1.00	1	1	-0.02	-0.02	-0.02	-0.016	-0.02	t	71192	VSVK	Homo sapiens
1	1.00	1	1	0.01	0.01	0.007	0.01	0.009	t	98834	IRLSFNPTQL	Homo sapiens
1	1.00	1	1	0.09	0.09	0.088	0.09	0.09	t	120412	ASKEKQENKIFADDIDISD	Mus musculus

1	1.00	1	1	0	-0.002	0	-0.001	0	t	46969	PATIGDYTDF	Mus musculus
1	1.00	1	1	0.01	0.012	0.006	0.011	0.012	t	44740	NLIDTS	Homo sapiens
1	1.00	1	1	0.03	0.034	0.03	0.027	0.027	t	22620	GTDGV	Homo sapiens
1	1.00	1	1	0	0.004	0	0.008	0.002	t	131818	AKEERRY	Homo sapiens
1	1.00	1	1	0.02	0.027	0.014	0.02	0.02	t	48452	PLPPVA	Cavia porcellus
1	1.00	1	1	0	0.002	0.005	0.007	0	t	109589	MHEGLHN	Homo sapiens
1	1.00	1	1	0	0.002	0.005	0.007	0	t	109589	MHEGLHN	Homo sapiens
1	1.00	1	1	0.01	0.008	0.015	0.009	0.01	t	104238	SDWWDF	Mus musculus
1	1.00	1	1	-0.01	-0.007	-0.019	-0.01	-0.011	t	63756	TFTNKLKEKHTDLGK	Mus musculus
1	1.00	1	1	-0.02	-0.018	-0.02	-0.013	-0.02	t	152051	PVVNSLDPPLL	Homo sapiens
1	1.00	1	1	0.01	0.012	-0.007	0.011	0.012	t	32686	KPEASSSD	Homo sapiens
1	1.00	1	1	0.03	0.028	0.01	0.023	0.03	t	128146	WSVCVGLTTYVNDYVLPYLA	Mus musculus
1	1.00	1	1	0.05	0.046	0.049	0.049	0.05	t	94746	YREDMEY	Oryctolagus cuniculus
1	1.00	1	1	0.02	0.02	0.02	0.02	0.02	t	60472	SQKIQLI	Homo sapiens

1	1.00	1	1	-0.01	-0.008	-0.023	-0.01	-0.009	t	68846	VHFFKNIV TPRTP	Rattus norvegic us
1	1.00	1	1	-0.02	-0.018	-0.015	-0.013	-0.022	t	104586	NRVAPNV RQ	Homo sapiens
1	1.00	1	1	0.01	0.012	0.014	0.011	0.01	t	10278	DSYIII	Homo sapiens
1	1.00	1	1	0.05	0.05	0.045	0.05	0.049	t	131023	QTTANPSC PE	Rattus norvegic us
1	1.00	1	1	-0.02	-0.022	-0.022	-0.018	-0.02	t	48732	PPA	Mus musculu s
1	1.00	1	1	-0.02	-0.023	-0.032	-0.02	-0.02	t	65401	TNQALKKE	Oryctola gus cuniculu s
1	1.00	1	1	-0.01	-0.01	-0.005	-0.01	-0.012	t	95979	VPQPPVS QFHIQ	Homo sapiens
1	1.00	1	1	0	-0.002	0	0.003	-0.001	t	70459	VPQYLRW FEEWAE	Rattus norvegic us
1	1.00	1	1	-0.05	-0.048	-0.048	-0.043	-0.05	t	127963	EPKPKKAP AKKGEKV	Homo sapiens
1	1.00	1	1	0.03	0.032	0.028	0.03	0.03	t	122876	PSPDSKPT	Rattus norvegic us
1	1.00	1	1	0.01	0.01	0.01	0.01	0.01	t	66542	TTASPISSIF	Mus musculu s
1	1.00	1	1	0.01	0.01	-0.006	0.012	0.013	t	98954	QEQVPLV QQQQF	Homo sapiens
1	1.00	1	1	0.01	0.01	-0.006	0.012	0.013	t	98956	QGSVQPQ QLPQF	Homo sapiens
1	1.00	1	1	0	0.002	-0.007	0.007	0	t	144801	TPPSVVGG LGVTMVH GNLT	Homo sapiens
1	1.00	1	1	-0.04	-0.044	-0.04	-0.043	-0.043	t	5109	ATPYHPPA TP	Oryctola gus cuniculu s

1	1.00	1	1	-0.01	-0.012	-0.013	-0.011	-0.012	t	151073	YEDVNGIRK	Mus musculus
1	1.00	1	1	0.01	0.013	0.004	0.01	0.01	t	43549	NDYEDRYYREN	Mus musculus
1	1.00	1	1	0.03	0.026	0.022	0.033	0.032	t	111177	CVQSTHVDIRTLEDLL	Oryctolagus cuniculus
1	1.00	1	1	-0.02	-0.023	-0.024	-0.02	-0.022	t	45357	NPGISGSRLLGT	Oryctolagus cuniculus
1	1.00	1	1	0.03	0.028	0.021	0.03	0.029	t	44471	NKDARELL	Oryctolagus cuniculus
1	1.00	1	1	0.01	0.01	0.01	0.01	0.009	t	108499	KKGKKKDP	Homo sapiens
1	1.00	1	1	0.03	0.028	0.01	0.023	0.03	t	128136	VFIADSDGKLNFKEWSVCVG	Mus musculus
1	1.00	1	1	0	0	-0.008	0	0.002	t	133546	DSARAAY	Mus musculus
1	1.00	1	1	0.01	0.012	0.01	0.011	0.007	t	19636	GFTTNEERYNVFAE	Homo sapiens
1	1.00	1	1	-0.02	-0.016	-0.02	-0.013	-0.02	t	143932	HNIWSAPRLIFN	Homo sapiens
1	1.00	1	1	-0.03	-0.032	-0.03	-0.027	-0.03	t	45816	NSGCFRHL	Mus musculus
1	1.00	1	1	0.02	0.02	0.02	0.02	0.017	t	134704	KFFNSNKKKRDDFEKL	Oryctolagus cuniculus
1	1.00	1	1	0	0.004	-0.002	0.003	0	t	25030	HVDTESYFIVLSIAY	Homo sapiens

1	1.00	1	1	0.02	0.024	0.02	0.027	0.019	t	131969	LSQLNQLS QISQLQR	Homo sapiens
1	1.00	1	1	0	0	0	0.002	-0.002	t	70953	VSGHRMA WD	Homo sapiens
1	1.00	1	1	0.02	0.017	0.022	0.02	0.019	t	40448	LVLLDYQG MLPVCPL	Oryctolagus cuniculus
1	1.00	1	1	0.03	0.033	0.038	0.03	0.028	t	79891	KSADVESD V	Mus musculus
1	1.00	1	1	-0.01	-0.01	-0.01	-0.007	-0.01	t	55135	RPDYNPLL	Homo sapiens
1	1.00	1	1	0.02	0.017	0.021	0.02	0.02	t	11199	EATALDTS	Oryctolagus cuniculus
1	1.00	1	1	0.01	0.01	0.006	0.012	0.009	t	19517	GFEKISKG AN	Homo sapiens
1	1.00	1	1	0.03	0.03	0.02	0.03	0.03	t	31406	KIQRDEDS YGRDPYS	Oryctolagus cuniculus
1	1.00	1	1	0.01	0.014	0.01	0.017	0.01	t	112935	DFLTISPKL LGELLQ	Homo sapiens
1	1.00	1	1	0	0.002	0.002	0.007	0.002	t	119648	SWNSGAL T	Homo sapiens
1	1.00	1	1	0	0.004	-0.013	0.001	0	t	67167	TVSSNGDR	Homo sapiens
1	1.00	1	1	0.02	0.023	0.021	0.023	0.02	t	78270	SIPIGS	Equus caballus
1	1.00	1	1	0.03	0.042	0.037	0.03	0.028	t	4702	ASQKIQLV	Pan troglodytes
1	1.00	1	1	0	0.002	-0.002	0.007	0	t	131936	KPVSSPD WNKQQDI V	Homo sapiens

1	1.00	1	1	-0.03	-0.03	-0.037	-0.03	-0.032	t	49645	PTGTAAA NYKTP	Mus musculu s
1	1.00	1	1	0.04	0.04	0.024	0.042	0.043	t	99046	VQQQQFL GQQQP	Homo sapiens
1	1.00	1	1	0.04	0.037	0.034	0.04	0.04	t	57766	SFFSYGEI	Oryctola gus cuniculu s
1	1.00	1	1	0.04	0.044	0.034	0.04	0.04	t	57766	SFFSYGEI	MD
1	1.00	1	1	0.01	0.01	0.019	0.01	0.007	t	19256	GEEYLILSA R	Mus musculu s
1	1.00	1	1	0	0.004	0	0.007	0	t	134217	RNVILFSVF DENRSWY LTEN	Homo sapiens
1	1.00	1	1	0.02	0.02	0.005	0.012	0.021	t	98781	FPSQQPYL QLQP	Homo sapiens
1	1.00	1	1	0.05	0.05	0.05	0.053	0.05	t	149103	SGPNPTQ	Sus scrofa
1	1.00	1	1	0.01	0.01	0.01	0.01	0.01	t	127906	YVKLFPNS LD	Homo sapiens
1	1.00	1	1	-0.01	-0.012	-0.011	-0.009	-0.009	t	69106	VIRVQY	Homo sapiens
1	1.00	1	1	0.01	0.01	0.01	0.01	0.007	t	137432	NHNYPPLS LLTF	Mus musculu s
1	1.00	1	1	-0.02	-0.02	-0.028	-0.02	-0.021	t	119656	TVDKSRW Q	Homo sapiens
1	1.00	1	1	0.01	0.013	0.01	0.01	0.01	t	134702	KEKKYS	Mus musculu s

1	1.00	1	1	-0.02	-0.02	-0.02	-0.02	-0.021	t	9759	DPRVRGLY LPA	Mus musculus
1	1.00	1	1	0.01	0.007	0.015	0.01	0.007	t	70249	VPANIKSM ALSG	Oryctolagus cuniculus
1	1.00	1	1	0	-0.002	-0.007	0.003	0.002	t	100432	QLYAPEAF NY	Mus musculus
1	1.00	1	1	-0.02	-0.02	-0.022	-0.023	-0.022	t	98851	KKIIAEKTKI	Homo sapiens
1	1.00	1	1	0.04	0.038	0.028	0.033	0.039	t	144641	FFENKD	Mus musculus
1	1.00	1	1	-0.05	-0.048	-0.05	-0.043	-0.05	t	119596	IHIPSEKIW RPDLVLY	Homo sapiens
1	1.00	1	1	-0.01	-0.01	-0.01	-0.006	-0.012	t	118614	SSPSAKDIK KILDSV	Homo sapiens
1	1.00	1	1	0.05	0.046	0.046	0.053	0.052	t	106001	PNIEADVN	Oryctolagus cuniculus
1	1.00	1	1	0.05	0.05	0.038	0.05	0.051	t	17629	FRPSQQN	Oryctolagus cuniculus
1	1.00	1	1	0.03	0.024	0.03	0.033	0.028	t	123565	NVKDYYQ KWM	Oryctolagus cuniculus
1	1.00	1	1	0.05	0.052	0.05	0.057	0.05	t	107501	LDGKEYK	Homo sapiens
1	1.00	1	1	0.01	0.008	0.01	0.013	0.01	t	58371	SHITSYPAY FW	Mus musculus
1	1.00	1	1	-0.04	-0.044	-0.046	-0.041	-0.041	t	27553	IMSALAM VYLGAK	Oryctolagus cuniculus
1	1.00	1	1	0	-0.002	-0.007	-0.001	-0.002	t	57259	SDPPLVAN QVVTCPD KKSTA	Rattus norvegicus
1	1.00	1	1	0.03	0.018	0.025	0.03	0.03	t	27748	INTLQNQG	Anas platyrhynchos

1	1.00	1	1	-0.02	-0.023	-0.027	-0.02	-0.023	t	108164	TLVCLATG FFPDHVEL	Oryctolagus cuniculus
1	1.00	1	1	0.01	0.009	0.002	0.01	0.01	t	4227	ARQQTVS ALE	Mus musculus
1	1.00	1	1	0.04	0.038	0.036	0.039	0.038	t	30001	KAVYNFAT C	Mus musculus
1	1.00	1	1	0.02	0.023	0.015	0.02	0.021	t	65982	TQYQKES QAYY	Mus musculus
1	1.00	1	1	0.01	0.008	0.001	0.01	0.01	t	100427	QKNAPKTF QF	Mus musculus
1	1.00	1	1	0.05	0.048	0.03	0.043	0.053	t	136118	AEFPLDIT	Mus musculus
1	1.00	1	1	0.05	0.05	0.043	0.05	0.05	t	138765	EEIKMDIS KLNA	Mus musculus
1	1.00	1	1	0.02	0.022	0.009	0.017	0.02	t	159060	RELTDAET KAFLKAG	Homo sapiens
1	1.00	1	1	0	0	-0.003	0	0	t	384	AAQAALG LLQTASR	Homo sapiens
1	1.00	1	1	0.05	0.05	0.052	0.05	0.052	t	70654	VQTMEEI N	Anas platyrhynchos
1	1.00	1	1	-0.02	-0.016	-0.02	-0.013	-0.02	t	116685	CPAGWISL WKGFS	Homo sapiens
1	1.00	1	1	0	0.002	-0.004	-0.003	-0.002	t	114946	SEYPKPDC TA	Homo sapiens
1	1.00	1	1	-0.02	-0.016	-0.015	-0.013	-0.021	t	115591	APEYRGRT ELLK	Homo sapiens

1	1.00	1	1	-0.02	-0.006	-0.015	-0.013	-0.021	t	115591	APEYRGRTELLK	Homo sapiens
1	1.00	1	1	-0.02	-0.018	-0.015	-0.013	-0.021	t	115591	APEYRGRTELLK	Homo sapiens
1	1.00	1	1	0.06	0.06	0.064	0.06	0.06	t	78806	QSQIESR	Oryctolagus cuniculus
1	1.00	1	1	-0.01	-0.008	-0.01	-0.003	-0.009	t	10594	DVFQNGFTAWGNND	Homo sapiens
1	1.00	1	1	0.02	0.02	0.02	0.02	0.02	t	72087	VYLLPR	Homo sapiens
1	1.00	1	1	0	0.004	0	0.007	0.002	t	131818	AKEERRY	Homo sapiens
1	1.00	1	1	0.04	0.038	0.04	0.037	0.039	t	51147	QIVGGVYLL	Mus musculus
1	1.00	1	1	0.02	0.02	0.012	0.02	0.019	t	41177	MAYRFNGIGVTQNVLYE	Mus musculus
1	1.00	1	1	0.01	0.01	-0.003	0.01	0.01	t	5205	ATVSSNGD	Homo sapiens
1	1.00	1	1	0	0	0	0.003	-0.002	t	62353	SVTEEIAEEDKSVTEEIAV	Homo sapiens
1	1.00	1	1	0	0	0	0.003	-0.002	t	51372	QLINTNGSWHINSTA	Homo sapiens
1	1.00	1	1	-0.01	-0.01	-0.017	-0.01	-0.01	t	46859	PALTAVETGATNPL	Homo sapiens
1	1.00	1	1	0.02	0.02	0.02	0.02	0.019	t	121690	PAIALREY	Homo sapiens
1	1.00	1	1	0.01	0.01	0.002	0.01	0.01	t	106302	CDIKTTLPPITA	Homo sapiens
1	1.00	1	1	0.07	0.07	0.07	0.07	0.069	t	136365	IDSNHVYKDFLT	Mus musculus

1	1.00	1	1	0	0.002	0	0	-0.001	t	117208	GLSLSRFS WGA	Homo sapiens
1	1.00	1	1	-0.01	-0.008	-0.01	-0.003	-0.01	t	119618	NHYTQKSL	Homo sapiens
1	1.00	1	1	0	0.003	-0.008	0	-0.002	t	51463	QLPFPQQ PQQ	Mus musculu s
1	1.00	1	1	-0.01	-0.007	-0.019	-0.01	-0.011	t	63756	TFTNKLKE KHTDLGK	Mus musculu s
1	1.00	1	1	0	0	-0.007	0.003	0	t	125791	CSFWSKYI QTLK	Mus musculu s
1	1.00	1	1	0	0.002	-0.002	0.003	-0.001	t	75937	YTAADCP GLLYLCP	Homo sapiens
1	1.00	1	1	0.02	0.023	0.016	0.021	0.02	t	41868	MKNNPVY SESL	Equus caballus
1	1.00	1	1	0.02	0.023	0.016	0.021	0.02	t	41868	MKNNPVY SESL	Equus caballus
1	1.00	1	1	0	-0.012	-0.006	0	-0.002	t	19266	GEFPRHVK LKNEIENLL DQV	Mus musculu s
1	1.00	1	1	0.02	0.022	0.009	0.021	0.02	t	110571	EKTGILTVT YHSETQRT K	Homo sapiens
1	1.00	1	1	0.03	0.03	0.021	0.03	0.03	t	69312	VKSASPHG ESNSSEE	Mus musculu s
1	1.00	1	1	0.03	0.03	0.017	0.028	0.03	t	12931	ELADALLE	Homo sapiens
1	1.00	1	1	0	0.002	0	0.007	0	t	9278	DLSLLGKA RKVVVTKD	Homo sapiens
1	1.00	1	1	0.05	0.048	0.05	0.043	0.046	t	117520	GSFSDADL AD	Mus musculu s

1	1.00	1	1	0	0	-0.006	0	0	t	42603	MSLSLLDLYLGRGYN	Homo sapiens
1	1.00	1	1	-0.01	-0.008	-0.015	-0.009	-0.012	t	29287	IVGGVYLLPR	Homo sapiens
1	1.00	1	1	-0.03	-0.025	-0.039	-0.029	-0.03	t	4467	ASICQMFCFWRGDLVFDFQV	Homo sapiens
1	1.00	1	1	0.01	0.013	0.002	0.01	0.01	t	51463	QLPFPQQPQQ	Mus musculus
1	1.00	1	1	0.01	0.01	0	0.01	0.01	t	98737	DPIELINLCTNALG	Oryctolagus cuniculus
1	1.00	1	1	0.03	0.03	0.03	0.033	0.03	t	149026	ANITQSS	Sus scrofa
1	1.00	1	1	0	-0.003	0.002	0	-0.003	t	55719	RRRLRGTWV	Oryctolagus cuniculus
1	1.00	1	1	0	0	-0.007	0	-0.001	t	105763	EVHHQK	Mus musculus
1	1.00	1	1	-0.01	-0.014	-0.01	-0.007	-0.012	t	156871	FQNIASMPHLYV	Bos taurus
1	1.00	1	1	0.03	0.034	0.022	0.031	0.03	t	62967	TAQAAPVQEG	Homo sapiens
1	1.00	1	1	0.03	0.032	0.022	0.031	0.03	t	62967	TAQAAPVQEG	Homo sapiens
1	1.00	1	1	-0.03	-0.026	-0.029	-0.023	-0.03	t	128073	PKKSAKKTPKKAKKP	Homo sapiens
1	1.00	1	1	0.02	0.023	0.023	0.02	0.02	t	15395	FDGTNP	Mus musculus
1	1.00	1	1	0.01	0.014	0.01	0.017	0.01	t	123576	PGAMPPQQ	Homo sapiens

1	1.00	1	1	-0.03	-0.025	-0.034	-0.027	-0.031	t	77793	PKSKLSVKKC	Homo sapiens
1	1.00	1	1	-0.04	-0.036	-0.035	-0.033	-0.041	t	115607	GPRHPIRALVGD	Homo sapiens
1	1.00	1	1	0.01	0.013	0.01	0.01	0.008	t	78623	DMDDFSRQLQ	Mus musculus
1	1.00	1	1	0.01	0.007	0.01	0.01	0.008	t	78623	DMDDFSRQLQ	Mus musculus
1	1.00	1	1	0.01	0.01	0.01	0.01	0.008	t	78623	DMDDFSRQLQ	Mus musculus
1	1.00	1	1	0	-0.002	-0.002	-0.001	0	t	97181	AFPLPTDA	Mus musculus
1	1.00	1	1	0	-0.002	-0.002	-0.001	0	t	97181	AFPLPTDA	Mus musculus
1	1.00	1	1	0.02	0.024	0.009	0.017	0.018	t	38582	LPLPLLQSWM	Homo sapiens
1	1.00	1	1	-0.02	-0.018	-0.019	-0.019	-0.023	t	65694	TPPPRRRRTVGLSE	Homo sapiens
1	1.00	1	1	0.02	-0.005	0.02	0.02	0.02	t	19268	GEGAPP	Marmota monax
1	1.00	1	1	0.02	0.009	0.02	0.02	0.02	t	19268	GEGAPP	Marmota monax
1	1.00	1	1	0.01	0.012	0.015	0.017	0.008	t	118288	DDRLNKVISELNGKN	Homo sapiens
1	1.00	1	1	0.03	0.03	0.03	0.034	0.031	t	110104	WQEGNVF	Homo sapiens
1	1.00	1	1	0.03	0.032	0.028	0.029	0.033	t	131878	FLMLVGGSTL	Homo sapiens

1	1.00	1	1	0	0	-0.013	0	0	t	105340	MPRCRHGY	Homo sapiens
1	1.00	1	1	0.04	0.036	0.04	0.033	0.038	t	134037	DYDDTIS	Oryctolagus cuniculus
1	1.00	1	1	0	-0.001	0	0.004	0	t	6721	CNPPDCPGVGTG	Myodes glareolus
1	1.00	1	1	0.02	0.02	0.017	0.02	0.02	t	46786	PAEVTQLKGLS	Mus musculus
1	1.00	1	1	-0.01	-0.01	-0.01	-0.01	-0.013	t	75949	YTASARGDLAHL	Mus musculus
1	1.00	1	1	0.02	0.02	0.02	0.02	0.018	t	104132	MSENVEQHNPINAGT	Oryctolagus cuniculus
1	1.00	1	1	-0.03	-0.032	-0.039	-0.031	-0.03	t	74478	YKLTRPYMARC	Mus musculus
1	1.00	1	1	-0.01	-0.01	-0.014	-0.007	-0.008	t	5286	AVCLGGLIGIPALIC	Homo sapiens
1	1.00	1	1	0.04	0.04	0.03	0.037	0.04	t	47907	PIGSENSEKT	Homo sapiens
1	1.00	1	1	0.01	0.01	0.02	0.01	0.007	t	95161	TYGKFCIKP	Mus musculus
1	1.00	1	1	0	0	-0.003	0	0	t	21845	GQAAWKEARA	Mus musculus
1	1.00	1	1	0	0	-0.003	0	0	t	21845	GQAAWKEARA	Mus musculus
1	1.00	1	1	0.04	0.04	0.038	0.04	0.041	t	130741	ATGFVKKDQ	Mus musculus

1	1.00	1	1	0	0.002	-0.007	-0.003	0	t	116039	YPSYGLNYYQ	Homo sapiens
1	1.00	1	1	-0.01	-0.006	-0.013	-0.009	-0.013	t	103549	RHGLSSLGWNLCRIF	Homo sapiens
1	1.00	1	1	0	0.003	-0.008	0	0	t	27935	IPLNVGK	Mus musculus
1	1.00	1	1	0.04	0.028	0.034	0.04	0.04	t	28898	ITFKSGATFQ	Mus musculus
1	1.00	1	1	0	0	0	0.008	0	t	110869	GQAEPDRAHYNIVTFCCKCD	Mus musculus
1	1.00	1	1	0	0	0	0.008	0	t	110828	EIDGPAGQAEPDRAHYNI	Mus musculus
1	1.00	1	1	0	0	0	0.008	0	t	110974	QDIVLHLEPQNEIPVDLL	Mus musculus
1	1.00	1	1	0	0	0	0.008	0	t	110869	GQAEPDRAHYNIVTFCCKCD	Mus musculus
1	1.00	1	1	0	0	0	0.008	0	t	41660	MHGDTPTLHEYMLDLQPETT	Mus musculus
1	1.00	1	1	0	0	0	0.008	0	t	41660	MHGDTPTLHEYMLDLQPETT	Mus musculus
1	1.00	1	1	0	0	0	0.008	0	t	23178	GVTSAPDTRPAPGSTA	Mus musculus
1	1.00	1	1	0.03	0.03	0.026	0.03	0.028	t	60535	SQQPQQPFPQ	Mus musculus
1	1.00	1	1	0.01	0.01	0.001	0.01	0.01	t	47315	PEKAFREL	Homo sapiens
1	1.00	1	1	-0.01	-0.014	-0.022	-0.011	-0.01	t	60445	SQHI	Oryctolagus cuniculus
1	1.00	1	1	0.02	0.016	0.005	0.019	0.021	t	21816	GPVEESVERAMVRVA	Oryctolagus cuniculus

1	1.00	1	1	0	-0.004	0	0.003	-0.002	t	71809	VVRFDKLPGFGDSIE	Oryctolagus cuniculus
1	1.00	1	1	0	-0.004	0	0.003	-0.002	t	94838	GTSSCRLVP	Oryctolagus cuniculus
1	1.00	1	1	0	-0.004	0	0.003	-0.002	t	76332	YVLDGDKLLPKVAPQAL	Oryctolagus cuniculus
1	1.00	1	1	0	-0.004	0	0.003	-0.002	t	27553	IMSALAMVYLGAK	Oryctolagus cuniculus
1	1.00	1	1	0	-0.004	0	0.003	-0.002	t	10254	DSTRTQINK	Oryctolagus cuniculus
1	1.00	1	1	-0.01	-0.012	-0.03	-0.017	-0.01	t	128043	LDAMPAGNPAFPVIPG	Mus musculus
1	1.00	1	1	-0.04	-0.038	-0.049	-0.04	-0.038	t	34792	LAGRMIPK	Anas platyrhynchos
1	1.00	1	1	-0.02	-0.017	-0.02	-0.02	-0.023	t	20836	GLFGAIAGF	Mus musculus
1	1.00	1	1	0.01	0.012	0.003	0.013	0.012	t	78068	VYDYNCHVDL	Homo sapiens
1	1.00	1	1	-0.01	-0.012	-0.01	-0.009	-0.011	t	130449	CFFPNLSYC	Mus musculus
1	1.00	1	1	0.01	0.003	0.003	0.009	0.008	t	52414	QSQLERANLRPCEQH	Oryctolagus cuniculus
1	1.00	1	1	0	0	0	0	0	t	8697	DILFPA	Homo sapiens
1	1.00	1	1	0	0.002	0	0.009	0	t	122445	KQYSIEAALRCRRISKEEYH	Homo sapiens
1	1.00	1	1	0	0	0	0.009	0	t	122445	KQYSIEAALRCRRISKEEYH	Homo sapiens

1	1.00	1	1	0.02	0.02	0.023	0.026	0.019	t	23162	GVSYNIGS QEWYTTV	Homo sapiens
1	1.00	1	1	-0.01	-0.008	-0.014	-0.009	-0.01	t	12625	EKAFREL	Homo sapiens
1	1.00	1	1	-0.01	-0.006	-0.022	-0.009	-0.01	t	7099	CSVTCGVG VRVRRRV NAANK	Homo sapiens
1	1.00	1	1	-0.01	-0.006	-0.022	-0.009	-0.01	t	7099	CSVTCGVG VRVRRRV NAANK	Homo sapiens
1	1.00	1	1	0.05	0.05	0.039	0.05	0.052	t	111309	GDTPTLHE YMLDLQ	Oryctola gus cuniculu s
1	1.00	1	1	0	0.002	-0.006	0.003	0	t	118824	GRRPFFHP VGEADYFE YHQE	Homo sapiens
1	1.00	1	1	0.02	0.016	0.019	0.019	0.02	t	78609	ANPSPHQ VYN	Oryctola gus cuniculu s
1	1.00	1	1	-0.01	-0.012	-0.01	-0.007	-0.01	t	73785	YEVRNVSG IYHVT	Homo sapiens
1	1.00	1	1	-0.01	-0.01	-0.012	-0.003	-0.01	t	125462	KLPLSLSFL HLTRADLS YPS	Homo sapiens
1	1.00	1	1	-0.01	-0.013	-0.012	-0.01	-0.011	t	56107	RTLKIPAM TI	Oryctola gus cuniculu s
1	1.00	1	1	0	0	0.013	0.008	-0.003	t	98923	PAVFKIDA LN	Homo sapiens
1	1.00	1	1	0.05	0.05	0.05	0.05	0.05	t	159291	MQVTDD	Oryctola gus cuniculu s
1	1.00	1	1	0	0.004	0	0.007	0.001	t	118904	MERKRRE	Homo sapiens

1	1.00	1	1	0.02	0.014	0.011	0.02	0.019	t	113861	SISTTNFTNP	Oryctolagus cuniculus
1	1.00	1	1	-0.01	-0.012	-0.026	-0.011	-0.009	t	6210	CEYNVFHNKTFELPRA	Mus musculus
1	1.00	1	1	-0.01	-0.013	-0.017	-0.01	-0.011	t	135507	VVGAGP	Oryctolagus cuniculus
1	1.00	1	1	0	0	-0.007	0	0.002	t	12105	EFVVEFDLPGIKA	Homo sapiens
1	1.00	1	1	-0.04	-0.038	-0.047	-0.039	-0.04	t	55781	RRYPF	Homo sapiens
1	1.00	1	1	0	0	0	0	0	t	112853	TPTFMYF	Homo sapiens
1	1.00	1	1	0.05	0.055	0.042	0.053	0.05	t	95428	GSGGDDDDPHGPVQLSYYD	Homo sapiens
1	1.00	1	1	0	-0.007	-0.005	0.001	0	t	147284	SIFGLAPSKALDS	Homo sapiens
1	1.00	1	1	-0.03	-0.028	-0.037	-0.023	-0.03	t	116927	AVCRTSMCSIQSAPP	Homo sapiens
1	1.00	1	1	0	-0.002	-0.008	-0.002	-0.001	t	25118	HVVVNNKVATH	Mus musculus
1	1.00	1	1	0	0.002	-0.011	-0.003	-0.002	t	114977	VMVLCNRAFN	Homo sapiens
1	1.00	1	1	0.02	0.02	0.009	0.02	0.02	t	43157	NAGTSL	Mus musculus
1	1.00	1	1	0.01	0.01	0.008	0.013	0.01	t	12570	EIRPSA	Homo sapiens
1	1.00	1	1	0.01	0.01	0.002	0.013	0.01	t	63165	TDHAGSNDNIALLVQ	Homo sapiens

1	1.00	1	1	0.02	0.024	-0.001	0.017	0.023	t	153886	SEEKANQR EEAYKEQ	Homo sapiens
1	1.00	1	1	0.01	0.013	0.011	0.01	0.008	t	59757	SMSYTWT GA	Mus musculu s
1	1.00	1	1	-0.02	-0.018	-0.02	-0.013	-0.02	t	121975	VHPAIALR	Homo sapiens
1	1.00	1	1	0.03	0.026	0.03	0.027	0.028	t	64638	TKPSDGNC T	Oryctola gus cuniculu s
1	1.00	1	1	0.03	0.03	0.03	0.03	0.031	t	115194	CNISCDKF LD	Homo sapiens
1	1.00	1	1	0.02	0.02	0.01	0.02	0.02	t	68764	VGPQSVG PLSVGPQS VGPLS	Mus musculu s
1	1.00	1	1	0.01	0.01	0.001	0.013	0.01	t	22708	GTIINQDP DKILTYI	Homo sapiens
1	1.00	1	1	0.01	0.01	0.01	0.01	0.01	t	127856	TDVRYKD	Mus musculu s
1	1.00	1	1	0.01	0.01	0.01	0.01	0.01	t	127856	TDVRYKD	Mus musculu s
1	1.00	1	1	-0.06	-0.062	-0.078	-0.059	-0.058	t	99842	RPQQPYP	Mus musculu s
1	1.00	1	1	0.04	0.039	0.033	0.04	0.04	t	55248	RPPETTTI	Anas platyrhy nchos
1	1.00	1	1	0.01	0.01	0.01	0.01	0.009	t	119998	KQQWVDY NLKWNPD DY	Rattus norvegic us
1	1.00	1	1	0.01	0.008	0.01	0.01	0.009	t	119998	KQQWVDY NLKWNPD DY	Oryctola gus cuniculu s
1	1.00	1	1	0.01	0.01	0.01	0.01	0.009	t	119998	KQQWVDY NLKWNPD DY	Mus musculu s
1	1.00	1	1	0.03	0.03	0.03	0.03	0.028	t	7673	DAVTE	Mus musculu s

1	1.00	1	1	-0.05	-0.05	-0.05	-0.043	-0.051	t	119823	EDDDMGF GLFD	Homo sapiens
1	1.00	1	1	0.01	0.008	0.006	0.009	0.008	t	59757	SMSYTWT GA	Mus musculu s
1	1.00	1	1	0	-0.002	0.004	0.003	-0.002	t	34169	KVPTL	Mus musculu s
1	1.00	1	1	0.01	0.01	0.01	0.01	0.01	t	96735	PKVTKEKP TP	Homo sapiens(human)
1	1.00	1	1	-0.02	-0.017	-0.034	-0.02	-0.018	t	99982	EFLKKLKLD I	Mus musculu s
1	1.00	1	1	-0.02	-0.022	-0.027	-0.017	-0.019	t	106873	SSTGSIDM VD	Mus musculu s
1	1.00	1	1	0.01	0.007	0.01	0.01	0.007	t	37311	LLGACSFSS I	Oryctola gus cuniculu s
1	1.00	1	1	-0.02	-0.02	-0.02	-0.016	-0.02	t	135955	LRLLLCLLL LSS	Homo sapiens
1	1.00	1	1	-0.01	-0.01	-0.009	-0.006	-0.01	t	30945	KGGRKP	Homo sapiens
1	1.00	1	1	0.02	0.032	0.02	0.026	0.02	t	109736	PTGASGPL	Rattus norvegic us
1	1.00	1	1	0.02	0.023	0.02	0.02	0.018	t	101953	PDPLSPGE	Mus musculu s
1	1.00	1	1	0.01	0.01	0.01	0.01	0.008	t	119658	TYGIVLLSG V	Homo sapiens
1	1.00	1	1	0.01	0.002	-0.005	0.01	0.013	t	95142	RIKNTDGSI S	Mus musculu s
1	1.00	1	1	0.03	0.03	0.03	0.03	0.029	t	108897	EIDGPAGQ AEP	Mus musculu s
1	1.00	1	1	0	0.004	0	0.007	0	t	134707	KLPFSRLA REICVKF	Homo sapiens

1	1.00	1	1	0.04	0.04	0.021	0.04	0.043	t	71328	VTGKVEYT KYNDDD	Mus musculu s
1	1.00	1	1	0.05	0.047	0.043	0.05	0.05	t	130908	ITRTTLQSD QEEIDY	Oryctola gus cuniculu s
1	1.00	1	1	0.02	0.022	0.02	0.023	0.02	t	25795	IEEVALSTT GEIPF	Homo sapiens
1	1.00	1	1	0	-0.004	0	0.003	-0.002	t	71353	VTHESYQE LVKKLEALE DA	Oryctola gus cuniculu s
1	1.00	1	1	0	-0.002	0	0.003	-0.002	t	62779	TAADTPTA TESISPSPP	Mus musculu s
1	1.00	1	1	0.03	0.03	0.018	0.03	0.032	t	81782	EQVEYPLK PI	Oryctola gus cuniculu s
1	1.00	1	1	0	0	-0.005	0	0.002	t	54157	RIGYFGHQ	Homo sapiens
1	1.00	1	1	0	0	0.002	0	-0.002	t	121773	QYVAMVA DRLFLY	Rattus norvegic us
1	1.00	1	1	0	-0.004	-0.007	0.003	0	t	35007	LAVLLGAC SF	Oryctola gus cuniculu s
1	1.00	1	1	0.01	0.01	0.006	0.01	0.007	t	22792	GTQETGD TTPSAYSM S	Oryctola gus cuniculu s
1	1.00	1	1	0	0.004	0	0.003	-0.001	t	118568	QKIGIEIIKR TLK	Homo sapiens
1	1.00	1	1	0.05	0.05	0.043	0.05	0.049	t	57746	SFEEDTEE DK	Oryctola gus cuniculu s
1	1.00	1	1	0.05	0.05	0.043	0.05	0.049	t	57746	SFEEDTEE DK	Oryctola gus cuniculu s

1	1.00	1	1	-0.01	-0.013	-0.022	-0.01	-0.008	t	42251	MNYLLFCLFFAF	Oryctolagus cuniculus
1	1.00	1	1	-0.01	-0.01	-0.022	-0.01	-0.008	t	42251	MNYLLFCLFFAF	Mus musculus
1	1.00	1	1	0.02	0.02	0.02	0.02	0.019	t	119632	QYNSTYRV	Homo sapiens
1	1.00	1	1	0.02	0.016	0.011	0.023	0.022	t	30516	KELSQKTL	Oryctolagus cuniculus
1	1.00	1	1	0.02	0.02	0.021	0.021	0.02	t	13266	ELRWDLTDIYNL	Mus musculus
1	1.00	1	1	0.02	0.02	0.021	0.021	0.02	t	13266	ELRWDLTDIYNL	Mus musculus
1	1.00	1	1	0.04	0.04	0.035	0.04	0.039	t	36953	LKLYDDKNASL	Mus musculus
1	1.00	1	1	0.01	0.01	0.006	0.01	0.008	t	49108	PQQQPFPQPQ	Mus musculus
1	1.00	1	1	0.02	0.02	0.017	0.02	0.02	t	47140	PDLIPLL	Mus musculus
1	1.00	1	1	0.02	0.017	0.017	0.02	0.02	t	47140	PDLIPLL	Oryctolagus cuniculus
1	1.00	1	1	-0.03	-0.03	-0.039	-0.023	-0.03	t	20065	GGVAVIKAGA	Homo sapiens
1	1.00	1	1	-0.05	-0.05	-0.063	-0.053	-0.05	t	72280	WCRCGD	Homo sapiens
1	1.00	1	1	0.03	0.03	0.03	0.036	0.03	t	94481	CSNNPTCWAICK	Homo sapiens
1	1.00	1	1	0	0	0	0.004	-0.001	t	122947	WTQEPQPLEEKWQHRVVE	Homo sapiens
1	1.00	1	1	0.06	0.06	0.06	0.063	0.06	t	149035	EGSGTTK	Sus scrofa
1	1.00	1	1	-0.02	-0.02	-0.02	-0.02	-0.02	t	118556	PPPGMIPP	Homo sapiens

1	1.00	1	1	0.02	0.022	0.004	0.02	0.022	t	134684	GSLPQKSQRDEN	Rattus norvegicus
1	1.00	1	1	0	-0.004	-0.013	-0.001	0	t	65794	TPVAPTQEVK	Oryctolagus cuniculus
1	1.00	1	1	0.04	0.04	0.033	0.04	0.039	t	137444	SDEALPLGSPRCD	Mus musculus
1	1.00	1	1	0	0	0	0.007	0	t	138812	SSVLYGGPPSAA	Rattus norvegicus
1	1.00	1	1	-0.02	-0.018	-0.022	-0.013	-0.02	t	132079	SQLQRLLAQQQRAQS	Homo sapiens
1	1.00	1	1	0.01	0.01	0.01	0.01	0.011	t	115190	CDKFLDDDLT	Homo sapiens
1	1.00	1	1	0.03	0.03	0.023	0.03	0.03	t	78375	EPGITTNYDT	Mus musculus
1	1.00	1	1	0	0.004	0	0.007	0	t	136419	KIHAREIFDSRGNPTVE	Homo sapiens
1	1.00	1	1	0	0.004	0	0.007	0	t	136418	KIHAREIFDSRGNPTVE	Homo sapiens
1	1.00	1	1	0	0.015	0	0.007	0	t	125610	YYVFFEEQEDEIIGFGQELK	Homo sapiens
1	1.00	1	1	0	0.015	0	0.007	0	t	125478	LHQEYEENLGDSIVGYKEKS	Homo sapiens
1	1.00	1	1	0	0	0	0.007	0	t	118060	PPPGMRPP	Mus musculus
1	1.00	1	1	0	0.015	0	0.007	0	t	125540	QSLRQRKSVNALNSPLHQEY	Homo sapiens
1	1.00	1	1	0	0.015	0	0.007	0	t	122780	GQELKNPQEETLQAFDSHYD	Homo sapiens

1	1.00	1	1	0	0.004	0	0.007	0	t	123511	MPPMTQ AP	Homo sapiens
1	1.00	1	1	0	0.002	0	0.007	0	t	117550	KREAVAG RGRGRGR G	Homo sapiens
1	1.00	1	1	0	0	0	0.007	0	t	117380	TKPAIRRL ARRGGVK RIS	Mus musculu s
1	1.00	1	1	0	0.002	0	0.007	0	t	108629	SHQESTRG RSRGRSGR SGS	Homo sapiens
1	1.00	1	1	0	0	0	0.007	0	t	117300	GSGKGKV QFQGKKT KF	Homo sapiens
1	1.00	1	1	0	0	0	0.007	0	t	105967	MKRGLTV AVAGAAIL VAGLS	Homo sapiens
1	1.00	1	1	0	0.004	0	0.007	0	t	117245	VTWEVLE GEVEKEAL KKI	Homo sapiens
1	1.00	1	1	0	0.002	0	0.007	0	t	108629	SHQESTRG RSRGRSGR SGS	Homo sapiens
1	1.00	1	1	0	0	0	0.007	0	t	117295	GITKPAIRR LARRGG	Mus musculu s
1	1.00	1	1	0	0	0	0.007	0	t	67607	VAGLSGCS SNKSTTGS GETT	Homo sapiens
1	1.00	1	1	0	0	0	0.007	0	t	42633	MSPVNKS FEIEVTCS	Homo sapiens
1	1.00	1	1	0	0	0	0.007	0	t	58388	SHLVEALY LVCGERG	Mus musculu s
1	1.00	1	1	0	0	0	0.007	0	t	58388	SHLVEALY LVCGERG	Mus musculu s
1	1.00	1	1	0	0	0	0.007	0	t	30661	KEVPALTA VETGAT	Homo sapiens
1	1.00	1	1	0	0	0	0.007	0	t	25315	IAIGGAAT GIAAVLTD GNPP	Homo sapiens

1	1.00	1	1	0	0	0	0.007	0	t	4334	ASATKDGSHYKITGTATGVD	Homo sapiens
1	1.00	1	1	0	0	0	0.007	0	t	433	AASGPKVVIDGKDQNVTGSV	Homo sapiens
1	1.00	1	1	0	0	0	0.007	0	t	4990	ATGVDMANPMSPVNKSFEIE	Homo sapiens
1	1.00	1	1	0.03	0.032	0.03	0.037	0.031	t	124590	PSPRSQT	Homo sapiens
1	1.00	1	1	0.01	0.01	0.005	0.01	0.01	t	22303	GSDYEDRYYRENM	Mus musculus
1	1.00	1	1	0.01	0.01	0.005	0.01	0.01	t	22303	GSDYEDRYYRENM	Mus musculus
1	1.00	1	1	0.01	0.01	0.005	0.01	0.01	t	22303	GSDYEDRYYRENM	Mus musculus
1	1.00	1	1	-0.01	-0.01	-0.01	-0.01	-0.013	t	115531	VAPFPEVFGKEKVNELSKDI	Homo sapiens
1	1.00	1	1	0.02	0.018	0.025	0.019	0.018	t	40938	LYRPSDSSLAGP	Mus musculus
1	1.00	1	1	-0.01	-0.01	-0.01	-0.01	-0.01	t	46860	PALVPDKEV	Homo sapiens
1	1.00	1	1	0.03	0.03	0.03	0.033	0.03	t	149118	TFGNRTQ	Sus scrofa
1	1.00	1	1	-0.01	-0.01	-0.022	-0.01	-0.011	t	78635	EKIERNNKLKQPPPPPNPN	Mus musculus
1	1.00	1	1	-0.02	-0.017	-0.02	-0.02	-0.02	t	135457	FGF	Mus musculus
1	1.00	1	1	0	0	0	0	0	t	132411	KMKGTFVE	Homo sapiens
1	1.00	1	1	0.03	0.018	0.03	0.03	0.03	t	61391	SSTISSCT	Macaca mulatta

1	1.00	1	1	0.02	0.024	0.006	0.02	0.021	t	45931	NSNNKEYT	Cavia porcellus
1	1.00	1	1	-0.02	-0.02	-0.029	-0.017	-0.02	t	6983	CSETFTNKLKEKH	Homo sapiens
1	1.00	1	1	0.05	0.053	0.038	0.05	0.05	t	102524	IDDLD	Mus musculus
1	1.00	1	1	-0.01	-0.008	-0.018	-0.009	-0.01	t	70481	VPSPDPNNSPARP	Homo sapiens
1	1.00	1	1	0.03	0.03	0.025	0.033	0.03	t	50272	QAGQEMILAVQGVQD	Homo sapiens
1	1.00	1	1	-0.03	-0.03	-0.032	-0.03	-0.032	t	55232	RPMIHF	Mus musculus
1	1.00	1	1	0.04	0.037	0.04	0.04	0.04	t	43575	NEDNEKLR	Oryctolagus cuniculus
1	1.00	1	1	-0.01	-0.012	-0.003	-0.009	-0.01	t	79011	LDPAFG	Mus musculus
1	1.00	1	1	-0.05	-0.048	-0.055	-0.049	-0.051	t	50494	QDMRNTIMASKTVG	Homo sapiens
1	1.00	1	1	0.01	0.012	0.019	0.017	0.012	t	108895	EGNVFSC	Homo sapiens
1	1.00	1	1	0	0	-0.011	0	0	t	111497	LMGTLGIVCPICSQKP	Oryctolagus cuniculus
1	1.00	1	1	0.02	0.02	0.025	0.02	0.02	t	9026	DLAPEA	Homo sapiens
1	1.00	1	1	-0.01	-0.012	-0.02	-0.011	-0.01	t	26244	IGILGGK	Mus musculus
1	1.00	1	1	-0.01	-0.014	-0.02	-0.011	-0.01	t	26244	IGILGGK	Oryctolagus cuniculus
1	1.00	1	1	0.03	0.03	0.03	0.03	0.031	t	115340	LDDDLTDDIM	Homo sapiens

1	1.00	1	1	0.06	0.06	0.053	0.061	0.059	t	64600	TKKNYSELDDEEPMELDYP	Homo sapiens
1	1.00	1	1	-0.01	-0.011	-0.002	-0.01	-0.012	t	25649	IDPY	Mus musculus
1	1.00	1	1	0.02	0.022	0.017	0.021	0.019	t	103304	KGLMNIALAISAQQV	Homo sapiens
1	1.00	1	1	0.06	0.058	0.064	0.063	0.06	t	8119	DEPAGE	Mus musculus
1	1.00	1	1	-0.02	-0.018	-0.029	-0.023	-0.023	t	105997	PFKFFVPPSEQSLRA	Homo sapiens
1	1.00	1	1	0.05	0.054	0.038	0.05	0.05	t	34137	KVNGGNLFDVSRVTF	Capra hircus
1	1.00	1	1	0.07	0.068	0.058	0.063	0.07	t	134794	SEATVAESDSF	Mus musculus
1	1.00	1	1	0.02	0.018	0.006	0.013	0.019	t	120584	HKTEKNKTEK	Mus musculus
1	1.00	1	1	-0.01	-0.012	-0.011	-0.009	-0.009	t	69106	VIRVQY	Homo sapiens
1	1.00	1	1	-0.03	-0.026	-0.03	-0.023	-0.031	t	134039	EDWDYAPLVLAPDDRSYKSQ	Homo sapiens
1	1.00	1	1	-0.01	-0.012	-0.01	-0.009	-0.01	t	98383	QPFPQPQLPYP	Mus musculus
1	1.00	1	1	-0.02	-0.018	-0.02	-0.013	-0.021	t	108104	RRLPFGSQM	Homo sapiens
1	1.00	1	1	0.01	0.01	0.01	0.01	0.01	t	6059	CCDLAP	Homo sapiens
1	1.00	1	1	0	0	-0.003	0	0	t	38273	LNLTTWNPTLLG	Oryctolagus cuniculus

1	1.00	1	1	0.01	0.01	0.01	0.006	0.008	t	117456	DGQFCH	Homo sapiens
1	1.00	1	1	0	0.003	0.001	0.003	0	t	78292	VPTKYH	Equus caballus
1	1.00	1	1	0.07	0.07	0.061	0.07	0.07	t	133698	SLLTEVETPIRNEWG	Mus musculus
1	1.00	1	1	0.01	0.014	0.006	0.011	0.01	t	71838	VVSTLP	Homo sapiens
1	1.00	1	1	0	0	-0.012	0	0	t	105341	MPRCRRGY	Homo sapiens
1	1.00	1	1	-0.02	-0.022	-0.029	-0.017	-0.02	t	115351	LKQKSSNSRKKRSTS	Mus musculus
1	1.00	1	1	0	-0.01	0	0.003	0	t	24694	HRMAWDMMM	Homo sapiens
1	1.00	1	1	0.08	0.08	0.078	0.074	0.078	t	7959	DEEEEYDEDEYEYDE	Homo sapiens
1	1.00	1	1	-0.01	-0.008	-0.01	-0.009	-0.013	t	146963	GGEDVAVFARGPQ	Homo sapiens
1	1.00	1	1	-0.01	-0.012	-0.019	-0.01	-0.01	t	100629	VRKQYKRDIQ	Mus musculus
1	1.00	1	1	0.02	0.017	0.011	0.02	0.022	t	20473	GIVEFYTD	Oryctolagus cuniculus
1	1.00	1	1	0.02	0.023	0.018	0.02	0.017	t	68858	VHILCYCYGLPDSEP	Equus caballus
1	1.00	1	1	-0.04	-0.04	-0.055	-0.04	-0.04	t	25900	IENAIRMISD	Homo sapiens
1	1.00	1	1	-0.03	-0.03	-0.035	-0.03	-0.031	t	55232	RPMIHF	Mus musculus
1	1.00	1	1	0.01	0.012	0.004	0.011	0.012	t	57215	SDIEKKVN	Homo sapiens

1	1.00	1	1	-0.01	-0.013	-0.02	-0.01	-0.01	t	140836	DEAAYSKNRRAV	Oryctolagus cuniculus
1	1.00	1	1	0.04	0.042	0.04	0.043	0.04	t	131762	YAASSYL	Homo sapiens
1	1.00	1	1	0.02	0.02	0.02	0.02	0.02	t	134752	MGPKRRQLTF	Homo sapiens
1	1.00	1	1	0.03	0.03	0.027	0.03	0.03	t	61824	STPLLYPSLA	Mus musculus
1	1.00	1	1	0	-0.004	-0.012	0.003	0	t	106779	RKPLSPGMTGR	Oryctolagus cuniculus
1	1.00	1	1	-0.03	-0.026	-0.039	-0.033	-0.033	t	117396	VKNLHNQARS	Homo sapiens
1	1.00	1	1	-0.01	-0.01	-0.015	-0.01	-0.011	t	46806	PAGGAYSMYTNWEQD	Homo sapiens
1	1.00	1	1	-0.02	-0.02	-0.027	-0.02	-0.021	t	130543	LLKGKGDKAQIEKRI	Homo sapiens
1	1.00	1	1	0	-0.002	-0.008	-0.001	0	t	64644	TKPTDPTGIE	Mus musculus
1	1.00	1	1	0.04	0.044	0.04	0.043	0.038	t	21738	GPPVDPTSG	Homo sapiens
1	1.00	1	1	-0.04	-0.04	-0.046	-0.04	-0.042	t	33548	KSVIIGCTGGSPEKHHC	Rattus norvegicus
1	1.00	1	1	0.03	0.03	0.03	0.03	0.03	t	74727	YLKASA	Homo sapiens
1	1.00	1	1	-0.01	-0.008	-0.013	-0.007	-0.01	t	52898	QYENLKYTVIITVHT	Homo sapiens
1	1.00	1	1	0.05	0.047	0.042	0.05	0.049	t	153445	QMTKLATTEELPDEFVVVTA	Oryctolagus cuniculus

1	1.00	1	1	-0.01	-0.01	-0.018	-0.007	-0.01	t	46473	NVSAIFMA VLLTLQT	Homo sapiens
1	1.00	1	1	-0.01	-0.01	-0.01	-0.01	-0.012	t	131100	TDSEMDV VRFD	Mus musculu s
1	1.00	1	1	-0.02	-0.02	-0.02	-0.02	-0.023	t	115396	NLLRFFVA PFPEVFGK EKVN	Homo sapiens
1	1.00	1	1	0.05	0.048	0.05	0.047	0.048	t	110877	GTVGENV PDDLYIKG	Mus musculu s
1	1.00	1	1	0.04	0.043	0.04	0.04	0.037	t	105702	CVYDQYG TEFEDYSY ES	Mus musculu s
1	1.00	1	1	-0.03	-0.03	-0.03	-0.026	-0.031	t	119605	KSRWQQG N	Homo sapiens
1	1.00	1	1	0.01	0.006	0	0.013	0.01	t	67941	VDFEEDTL PK	Oryctola gus cuniculu s
1	1.00	1	1	-0.02	-0.016	-0.022	-0.023	-0.02	t	139939	VLPWPWE	Ovis aries
1	1.00	1	1	0.03	0.033	0.021	0.03	0.03	t	99945	DIRDSLSEE E	Mus musculu s
1	1.00	1	1	0.02	0.02	0.029	0.027	0.02	t	135975	PTLAFLQD VMNI	Homo sapiens
1	1.00	1	1	0.05	0.052	0.062	0.057	0.05	t	130859	GLLAQQM DY	Homo sapiens
1	1.00	1	1	0.01	0.014	-0.003	0.007	0.012	t	65983	TQYTDAPS FS	Homo sapiens
1	1.00	1	1	0.01	0.003	0.003	0.017	0.012	t	156799	VYATRSSA VRLRSSV	Homo sapiens

1	1.00	1	1	-0.02	-0.016	-0.035	-0.02	-0.02	t	43796	NEYGVEG GRVNAVG	Mus musculu s
1	1.00	1	1	-0.02	-0.016	-0.035	-0.02	-0.02	t	43796	NEYGVEG GRVNAVG	Mus musculu s
1	1.00	1	1	-0.02	-0.02	-0.024	-0.02	-0.023	t	18524	GAARQPD	Mus musculu s
1	1.00	1	1	0.04	0.04	0.04	0.046	0.04	t	118567	QIEKRIQEII EQL	Homo sapiens
1	1.00	1	1	-0.01	-0.01	-0.01	-0.007	-0.01	t	125791	CSFWSKYI QTLK	Mus musculu s
1	1.00	1	1	0.03	0.032	0.028	0.027	0.027	t	116740	GRDPYSPS QDPYSPS	Homo sapiens
1	1.00	1	1	0	0.002	-0.012	0.001	0.002	t	96453	KFKEAFLR SV	Homo sapiens(human)
1	1.00	1	1	-0.02	-0.016	-0.02	-0.023	-0.023	t	70443	VPQKAVP YPQRD	Homo sapiens
1	1.00	1	1	0	-0.002	0	0.003	-0.002	t	43233	NANP	Mus musculu s
1	1.00	1	1	0	-0.004	0	0.003	-0.002	t	34965	LASLRKAF ADTVPVRP PSNY	Oryctola gus cuniculu s
1	1.00	1	1	0	-0.004	0	0.003	-0.002	t	28075	IPSTAFTDI AWVRLPN HY	Oryctola gus cuniculu s
1	1.00	1	1	0	-0.002	0	0.003	-0.002	t	6402	CHPSDGK CN	Mus musculu s
1	1.00	1	1	0	-0.002	0	0.003	-0.002	t	10069	DRYIPYSP	Mus musculu s

1	1.00	1	1	0.01	0.01	-0.001	0.01	0.01	t	123282	HACQKKLLKFEALQQEEGEE	Mus musculus
1	1.00	1	1	0.01	0.008	-0.001	0.01	0.01	t	123282	HACQKKLLKFEALQQEEGEE	Oryctolagus cuniculus
1	1.00	1	1	0.01	0.013	-0.001	0.01	0.01	t	123282	HACQKKLLKFEALQQEEGEE	Mus musculus
1	1.00	1	1	0	0	0	0	-0.003	t	9754	DPRVRGLYF	Mus musculus
1	1.00	1	1	0.01	0.012	-0.001	0.011	0.01	t	110571	EKTGILTVTYHSETQRTK	Homo sapiens
1	1.00	1	1	-0.01	-0.008	-0.005	-0.003	-0.013	t	49763	PTRAETREERMERKRREKIE	Homo sapiens
1	1.00	1	1	0.01	0.01	-0.002	0.01	0.01	t	153441	QKESTLHLVLRLRGG	Mus musculus
1	1.00	1	1	0.07	0.078	0.07	0.07	0.069	t	136365	IDSNHVYKDFLT	Gallus gallus
1	1.00	1	1	-0.03	-0.028	-0.023	-0.027	-0.034	t	142007	SDWRFLRGYHQYA	Homo sapiens
1	1.00	1	1	-0.01	-0.014	-0.013	-0.011	-0.012	t	111820	TEAVPGDPDVNRF	Oryctolagus cuniculus
1	1.00	1	1	0	0.003	0.006	0	-0.005	t	122912	TDVRYKDDMYHFFCPAIQA	Mus musculus
1	1.00	1	1	0	0	0	0.004	0	t	135953	LLQYVVKSFDRS	Homo sapiens
1	1.00	1	1	0	-0.005	-0.009	-0.002	0	t	51474	QLPNVGITQGIV	Bos taurus
1	1.00	1	1	0.02	0.02	0.014	0.02	0.02	t	22063	GRGYNVSSIVTMTSQ	Homo sapiens
1	1.00	1	1	-0.03	-0.03	-0.031	-0.03	-0.031	t	118822	GPPRPGMMPA	Homo sapiens
1	1.00	1	1	0.01	0.012	0.006	0.007	0.008	t	303	AALTKAITAMTQA	Homo sapiens

1	1.00	1	1	0.01	0.013	0.003	0.01	0.01	t	105704	DEAFRHDSGY	Mus musculus
1	1.00	1	1	-0.03	-0.028	-0.031	-0.023	-0.03	t	134803	THLLIRKLPFSRLAR	Homo sapiens
1	1.00	1	1	0.02	0.022	0.013	0.021	0.02	t	98059	GEWAGKDKIE	Homo sapiens
1	1.00	1	1	0.02	0.023	0.02	0.02	0.02	t	7532	DAHHAAD	Mus musculus
1	1.00	1	1	-0.02	-0.032	-0.02	-0.02	-0.022	t	9756	DPRVRGLYFPAGG	Mus musculus
1	1.00	1	1	-0.02	-0.032	-0.02	-0.02	-0.022	t	9756	DPRVRGLYFPAGG	Mus musculus
1	1.00	1	1	-0.02	-0.032	-0.02	-0.02	-0.022	t	9756	DPRVRGLYFPAGG	Mus musculus
1	1.00	1	1	-0.03	-0.027	-0.039	-0.03	-0.03	t	99946	DIYGKDALLH	Mus musculus
1	1.00	1	1	-0.02	-0.02	-0.032	-0.02	-0.02	t	134656	DSDMGRMGPI	Oryctolagus cuniculus
1	1.00	1	1	0.01	0.01	0.01	0.01	0.01	t	19735	GGFVQFVTSTRNA	Oryctolagus cuniculus
1	1.00	1	1	0.01	0.01	0.01	0.01	0.01	t	19735	GGFVQFVTSTRNA	Oryctolagus cuniculus
1	1.00	1	1	0.02	0.022	0.009	0.017	0.018	t	6985	CSEYPKPDCTAE	Homo sapiens
1	1.00	1	1	0.03	0.032	0.035	0.037	0.03	t	126903	QVINVREG	Homo sapiens
1	1.00	1	1	0.02	0.019	0.011	0.02	0.02	t	49690	PTLDHVLD	Anas platyrhynchos

1	1.00	1	1	0.06	0.06	0.06	0.063	0.059	t	78872	VIPDREVLYQEFDEMEECAS	Homo sapiens
1	1.00	1	1	0.04	0.042	0.04	0.043	0.041	t	68017	VDLPQL	Homo sapiens
1	1.00	1	1	0.01	0.01	0.01	0.01	0.007	t	140605	TLYDALGAV	Mus musculus
1	1.00	1	1	0.06	0.06	0.06	0.06	0.06	t	79403	SVGDYG	Mus musculus
1	1.00	1	1	0.01	0.012	-0.002	0.001	0.008	t	115795	IPTINTIASG	Homo sapiens
1	1.00	1	1	0.05	0.048	0.056	0.053	0.051	t	67967	VDGSDP	Mus musculus
1	1.00	1	1	0.03	0.03	0.03	0.03	0.03	t	140462	FNTKHDMQGDTS	Mus musculus
1	1.00	1	1	0.03	0.027	0.013	0.03	0.033	t	97023	VATRSQTDTSEKDC	Oryctolagus cuniculus
1	1.00	1	1	0	0	0	0	-0.004	t	141726	PTAPPLLPHS	Rattus norvegicus
1	1.00	1	1	0	0	0	0	-0.004	t	141067	PLSLEPDP	Mus musculus
1	1.00	1	1	0	0	0	0	-0.004	t	139935	VIAIALFGTIATANAAD	Homo sapiens
1	1.00	1	1	0	0	0	0	-0.004	t	137962	RDSPEDFV	Oryctolagus cuniculus
1	1.00	1	1	0	0	0	0	-0.004	t	139830	MKKISSVIAIALFGTIA	Homo sapiens
1	1.00	1	1	0	0	0	0	-0.004	t	137401	FEDVW	Mus musculus
1	1.00	1	1	0	0	0	0	-0.004	t	144364	ITDAVITVPAYFND	Mus musculus

1	1.00	1	1	0	0.004	0	0	-0.004	t	144364	ITDAVITVP AYFND	Mus musculu s
1	1.00	1	1	0	0	0	0	-0.004	t	141004	EPDP	Mus musculu s
1	1.00	1	1	0	0	0	0	-0.004	t	141760	TAPPLLPH	Rattus norvegic us
1	1.00	1	1	0	0	0	0	-0.004	t	141761	TAPPLLPH SNLD	Rattus norvegic us
1	1.00	1	1	0	0	0	0	-0.004	t	142006	SDSAQGS DVSLTA	Oryctola gus cuniculu s
1	1.00	1	1	0	0	0	0	-0.004	t	151327	PDGAAAG GG	Mus musculu s
1	1.00	1	1	0	0	0	0	-0.004	t	151763	WYRATHQ E	Mus musculu s
1	1.00	1	1	0	0	0	0	-0.004	t	137866	GDTRPRFL	Oryctola gus cuniculu s
1	1.00	1	1	0	0	0	0	-0.004	t	141605	APPLLPHS NL	Mus musculu s
1	1.00	1	1	0	0	0	0	-0.004	t	141859	ASSNSAQ GSDESLIA	Oryctola gus cuniculu s
1	1.00	1	1	0	0	0	0	-0.004	t	122749	EIIGFGQEL KNPQEE	Oryctola gus cuniculu s
1	1.00	1	1	0	0	0	0	-0.004	t	133642	MCPEMSF VAVHSCA R	Mus musculu s
1	1.00	1	1	0	0	0	0	-0.004	t	133531	CDTLKPGE C	Mus musculu s

1	1.00	1	1	0	0	0	0	-0.004	t	133705	SSKTRSFG VHLVGPY	Mus musculu s
1	1.00	1	1	0	0	0	0	-0.004	t	131956	LPKSAKPV SQMRMA TPLL	Oryctola gus cuniculu s
1	1.00	1	1	0	-0.003	0	0	-0.004	t	122780	GQELKNP QEETLQAF DSHYD	Oryctola gus cuniculu s
1	1.00	1	1	0	0	0	0	-0.004	t	133632	LRCYSHCT FYFASST	Mus musculu s
1	1.00	1	1	0	0	0	0	-0.004	t	133528	CADQKPG EC	Mus musculu s
1	1.00	1	1	0	0	0	0	-0.004	t	133554	EGLYSPW WPRSLPVL	Mus musculu s
1	1.00	1	1	0	0	0	0	-0.004	t	133630	LPPGLHVF PLASNRS	Mus musculu s
1	1.00	1	1	0	0	0	0	-0.004	t	125478	LHQEYEEN LGDSIVGY KEKS	Cricetulu s migrator ius
1	1.00	1	1	0	0	0	0	-0.004	t	125478	LHQEYEEN LGDSIVGY KEKS	Mus musculu s
1	1.00	1	1	0	0	0	0	-0.004	t	132045	RMATPLL MRPMSM	Oryctola gus cuniculu s
1	1.00	1	1	0	0	0	0	-0.004	t	122780	GQELKNP QEETLQAF DSHYD	Oryctola gus cuniculu s
1	1.00	1	1	0	0	0	0	-0.004	t	133580	GFLSSCNF RFCELVR	Mus musculu s
1	1.00	1	1	0	0	0	0	-0.004	t	133624	KPGEMRG AA	Mus musculu s
1	1.00	1	1	0	0	0	0	-0.004	t	133539	CYKPGEW AC	Mus musculu s
1	1.00	1	1	0	0	0	0	-0.004	t	133698	SLLTEVETP IRNEWG	Mus musculu s

1	1.00	1	1	0	0	0	0	-0.004	t	133542	DGRRDVV VRSATFYL	Mus musculu s
1	1.00	1	1	0	0	0	0	-0.004	t	122861	NALNSPLH QEYEENL	Oryctola gus cuniculu s
1	1.00	1	1	0	0	0	0	-0.004	t	122377	GLPRLETP ADLSTAIA SRS	Oryctola gus cuniculu s
1	1.00	1	1	0	0	0	0	-0.004	t	133622	KPGDPSAL HVVRCWI C	Mus musculu s
1	1.00	1	1	0	0	0	0	-0.004	t	125015	DNESSYSR GFDMTYT E	Mus musculu s
1	1.00	1	1	0	-0.003	0	0	-0.004	t	122780	GQELKNP QEETLQAF DSHYD	Oryctola gus cuniculu s
1	1.00	1	1	0	0	0	0	-0.004	t	133536	CKPVENRA C	Mus musculu s
1	1.00	1	1	0	0	0	0	-0.004	t	122392	HARLRDSG RAY	Homo sapiens
1	1.00	1	1	0	0	0	0	-0.004	t	122377	GLPRLETP ADLSTAIA SRS	Oryctola gus cuniculu s
1	1.00	1	1	0	0	0	0	-0.004	t	108600	QLINTNGS	Capra hircus
1	1.00	1	1	0	0	0	0	-0.004	t	100508	SGYEVHH QKL	Mus musculu s
1	1.00	1	1	0	0	0	0	-0.004	t	104695	YLLYYTEFT PTEK	Mus musculu s
1	1.00	1	1	0	0	0	0	-0.004	t	115035	ASSDSSNT E	Rattus norvegic us
1	1.00	1	1	0	0	0	0	-0.004	t	113628	LRLIHYSYD VN	Oryctola gus cuniculu s
1	1.00	1	1	0	0	0	0	-0.004	t	97594	QTPLTETA LKWH	Mus musculu s

1	1.00	1	1	0	0	0	0	-0.004	t	76275	YVFYSGNGPKASGGDYCIQS	Oryctolagus cuniculus
1	1.00	1	1	0	0	0	0	-0.004	t	62003	STWHVLYSPNVSVPSSSSTP	Homo sapiens
1	1.00	1	1	0	0	0	0	-0.004	t	75441	YPYDVPDYA	Mus musculus
1	1.00	1	1	0	0	0	0	-0.004	t	91100	AFQTPVHS	Mus musculus
1	1.00	1	1	0	0	0	0	-0.004	t	97595	QTPLTMAALELF	Mus musculus
1	1.00	1	1	0	0	0	0	-0.004	t	97726	VETPIRN	Mus musculus
1	1.00	1	1	0	0	0	0	-0.004	t	62289	SVPSSSS	Mus musculus
1	1.00	1	1	0	0	0	0	-0.004	t	61824	STPLLYPSLA	Mus musculus
1	1.00	1	1	0	0	0	0	-0.004	t	97777	YKNMILGTSVNIYEYIGKLQ	Oryctolagus cuniculus
1	1.00	1	1	0	0	0	0	-0.004	t	62859	TAGLVGLLTPGA	Mus musculus
1	1.00	1	1	0	0	0	0	-0.004	t	75802	YSLRLTPRPVSRFLGNNSIL	Oryctolagus cuniculus
1	1.00	1	1	0	0	0	0	-0.004	t	91109	AHFVQQTP	Mus musculus
1	1.00	1	1	0	0	0	0	-0.004	t	74798	YLLPRRGPRL	Homo sapiens
1	1.00	1	1	0	0	0	0	-0.004	t	97287	ETPLTETALKWH	Mus musculus

1	1.00	1	1	0	0	0	0	-0.004	t	97605	RIVRGPANLRNLKEFKE	Oryctolagus cuniculus
1	1.00	1	1	0	0	0	0	-0.004	t	97288	ETQLTTAGLRLL	Mus musculus
1	1.00	1	1	0	0	0	0	-0.004	t	75441	YPYDVPDYA	Mus musculus
1	1.00	1	1	0	0	0	0	-0.004	t	66600	TTETGKGV	Mus musculus
1	1.00	1	1	0	0	0	0	-0.004	t	92794	GENKRPNDKAGEV	Mus musculus
1	1.00	1	1	0	0	0	0	-0.004	t	66841	TTTCFRDTDTGK	Oryctolagus cuniculus
1	1.00	1	1	0	0	0	0	-0.004	t	75441	YPYDVPDYA	Mus musculus
1	1.00	1	1	0	0	0	0	-0.004	t	76491	YYDGRRS	Mus musculus
1	1.00	1	1	0	0	0	0	-0.004	t	76491	YYDGRRS	Mus musculus
1	1.00	1	1	0	0	0	0	-0.004	t	74798	YLLPRRGPRL	Homo sapiens
1	1.00	1	1	0	0	0	0	-0.004	t	97650	SLLTEVET	Mus musculus
1	1.00	1	1	0	0	0	0	-0.004	t	41826	MKHM	Mus musculus
1	1.00	1	1	0	0	0	0	-0.004	t	57812	SFKVTALNVVSRRYLASLHK	Sus scrofa
1	1.00	1	1	0	0	0	0	-0.004	t	38522	LPHSNL	Rattus norvegicus
1	1.00	1	1	0	0	0	0	-0.004	t	52792	QVSLESVDVYFQDVFGTMWC	Oryctolagus cuniculus

1	1.00	1	1	0	0	0	0	-0.004	t	38628	LPPTAPPLL	Mus musculus
1	1.00	1	1	0	0	0	0	-0.004	t	48557	PMIHFGNDWEDRYYREN	Mus musculus
1	1.00	1	1	0	-0.003	0	0	-0.004	t	49539	PSTSSKLRPRWTFTSPPVTT	Oryctolagus cuniculus
1	1.00	1	1	0	0	0	0	-0.004	t	57542	SELSQLS	Mus musculus
1	1.00	1	1	0	0	0	0	-0.004	t	37793	LLSHSNLDHI	Mus musculus
1	1.00	1	1	0	0.004	0	0	-0.004	t	46371	NVESQPGGQPNT	Mus musculus
1	1.00	1	1	0	0	0	0	-0.004	t	45597	NQNNFVHDCVNITIKQHTVT	Mus musculus
1	1.00	1	1	0	0	0	0	-0.004	t	59374	SLNTGWLAGLFY	Mus musculus
1	1.00	1	1	0	0	0	0	-0.004	t	37660	LLPHSNLDHILEPSIPWKSK	Mus musculus
1	1.00	1	1	0	0	0	0	-0.004	t	37656	LLPHSNLD	Mus musculus
1	1.00	1	1	0	0	0	0	-0.004	t	40938	LYRPSDSSLAGP	Mus musculus
1	1.00	1	1	0	0	0	0	-0.004	t	37660	LLPHSNLDHILEPSIPWKSK	Mus musculus
1	1.00	1	1	0	0	0	0	-0.004	t	46371	NVESQPGGQPNT	Mus musculus
1	1.00	1	1	0	0	0	0	-0.004	t	37660	LLPHSNLDHILEPSIPWKSK	Mus musculus

1	1.00	1	1	0	0	0	0	-0.004	t	37660	LLPHSNLDHILEPSIPWKSK	Mus musculus
1	1.00	1	1	0	0	0	0	-0.004	t	44920	NLVPMVATV	Homo sapiens
1	1.00	1	1	0	0.002	0	0	-0.004	t	38522	LPHSNL	Rattus norvegicus
1	1.00	1	1	0	0	0	0	-0.004	t	59374	SLNTGWLAGLFY	Mus musculus
1	1.00	1	1	0	0	0	0	-0.004	t	33206	KRPNDKAG	Mus musculus
1	1.00	1	1	0	0	0	0	-0.004	t	23975	HHAEMQNPVYLIPETVPYIK	Oryctolagus cuniculus
1	1.00	1	1	0	0	0	0	-0.004	t	21407	GNDYEDRYYREN	Mus musculus
1	1.00	1	1	0	0	0	0	-0.004	t	20089	GGWNTGGSR	Mus musculus
1	1.00	1	1	0	0	0	0	-0.004	t	23254	GWGQPHGGGWGQPHG	Mus musculus
1	1.00	1	1	0	0	0	0	-0.004	t	15509	FEDVKGTGGESRDLPSGQG	Mus musculus
1	1.00	1	1	0	0	0	0	-0.004	t	10888	DYGGDSTTPRPRYNATTYLP	Oryctolagus cuniculus
1	1.00	1	1	0	0	0	0	-0.004	t	10987	EAAGIGILTV	Homo sapiens
1	1.00	1	1	0	0	0	0	-0.004	t	14686	EVETPIRN	Mus musculus
1	1.00	1	1	0	0	0	0	-0.004	t	14686	EVETPIRN	Mus musculus
1	1.00	1	1	0	0	0	0	-0.004	t	24402	HNQWNKPSK	Mus musculus

1	1.00	1	1	0	0	0	0	-0.004	t	30200	KDLLEQKRAAVDTYC	Oryctolagus cuniculus
1	1.00	1	1	0	0	0	0	-0.004	t	14435	ETIYNTTLKY	Homo sapiens
1	1.00	1	1	0	0	0	0	-0.004	t	14686	EVETPIRN	Mus musculus
1	1.00	1	1	0	0	0	0	-0.004	t	10815	DWIWNQMHIDEEKNE	Rattus norvegicus
1	1.00	1	1	0	0	0	0	-0.004	t	10908	DYKDDDDK	Mus musculus
1	1.00	1	1	0	0	0	0	-0.004	t	11571	EEEDDDMGFGLFD	Mus musculus
1	1.00	1	1	0	-0.003	0	0	-0.004	t	23975	HHAEMQNPVYLIPETVPYIK	Oryctolagus cuniculus
1	1.00	1	1	0	0	0	0	-0.004	t	11571	EEEDDDMGFGLFD	Mus musculus
1	1.00	1	1	0	0	0	0	-0.004	t	12941	ELAGIGILTV	Homo sapiens
1	1.00	1	1	0	0	0	0	-0.004	t	22280	GSAMSRPIIHFGSDYEDRYY	Mus musculus
1	1.00	1	1	0	0	0	0	-0.004	t	6479	CKGKIQALCENPEWA	Mus musculus
1	1.00	1	1	0	0	0	0	-0.004	t	1165	AESPKKP	Homo sapiens
1	1.00	1	1	0	0	0	0	-0.004	t	6479	CKGKIQALCENPEWA	Mus musculus
1	1.00	1	1	0	0	0	0	-0.004	t	4429	ASGQ	Mus musculus
1	1.00	1	1	0	0	0	0	-0.004	t	1257	AFASNRTFDITVSGLGTAPK	Oryctolagus cuniculus
1	1.00	1	1	0	0	0	0	-0.004	t	6457	CITQYQRESQAYYQRG	Mus musculus

1	1.00	1	1	0	0	0	0	-0.004	t	4397	ASFEAQGALANIAVDKA	Mus musculus
1	1.00	1	1	0	0	-0.007	0	0	t	145508	SARIPCFLAG	Homo sapiens
1	1.00	1	1	-0.03	-0.031	-0.04	-0.029	-0.03	t	42618	MSPARR	Homo sapiens
1	1.00	1	1	0.03	0.027	0.019	0.03	0.03	t	144776	SEISTGLVGITSVAS	Oryctolagus cuniculus
1	1.00	1	1	0.01	0.014	0.01	0.017	0.01	t	119925	HFPFDEQNGSMKLGTWT	Homo sapiens
1	1.00	1	1	0.01	0.012	0.01	0.017	0.01	t	119925	HFPFDEQNGSMKLGTWT	Homo sapiens
1	1.00	1	1	-0.01	-0.01	-0.01	-0.01	-0.013	t	134704	KFFNSNKKKRDDFEKL	Oryctolagus cuniculus
1	1.00	1	1	0.01	0.01	0.009	0.013	0.01	t	62500	SWVDIV	Homo sapiens
1	1.00	1	1	0.01	0.007	-0.001	0.01	0.009	t	36043	LGAALLQSAR	Oryctolagus cuniculus
1	1.00	1	1	-0.01	-0.01	-0.02	-0.013	-0.012	t	47897	PIFKSGRGCGSCFEI	Homo sapiens
1	1.00	1	1	0.01	0.01	0	0.007	0.01	t	96085	ALCSEKLDQWLCEKL	Homo sapiens
1	1.00	1	1	0.02	0.018	0.013	0.021	0.02	t	3592	APHLPYIEQGMQLAEQFKQK	Homo sapiens
1	1.00	1	1	0	0	0	0	0	t	74415	YIWPRNDYDG	Mus musculus
1	1.00	1	1	0	0	0	0	0	t	9611	DPDKDVDIMV	Mus musculus

1	1.00	1	1	0.02	0.02	0.02	0.023	0.02	t	149028	AVTNATQ	Sus scrofa
1	1.00	1	1	0.03	0.028	0.019	0.029	0.03	t	47136	PDLERYAG	Mus musculus
1	1.00	1	1	0.02	0.018	0.011	0.017	0.02	t	79622	NYARTGGQVTAG	Mus musculus
1	1.00	1	1	0.01	0.01	0.014	0.01	0.01	t	73974	YGAAFS	Homo sapiens
1	1.00	1	1	-0.02	-0.015	-0.024	-0.017	-0.021	t	77808	YGCWKGLVGR	Homo sapiens
1	1.00	1	1	-0.01	-0.013	-0.01	-0.007	-0.012	t	134189	PPPSLPGPPRPG	Oryctolagus cuniculus
1	1.00	1	1	0.03	0.028	0.03	0.031	0.03	t	86716	NFNSNNYTYD	Homo sapiens
1	1.00	1	1	0	0	-0.015	0	0	t	71354	VTHESYQELVKKLEALEDAV	Mus musculus
1	1.00	1	1	0	0.003	-0.015	0	0	t	71354	VTHESYQELVKKLEALEDAV	Mus musculus
1	1.00	1	1	-0.02	-0.014	-0.013	-0.013	-0.02	t	127676	GTIFDNFLIT	Homo sapiens
1	1.00	1	1	0.01	0.008	0.001	0.01	0.01	t	81325	DVKASA	Mus musculus
1	1.00	1	1	-0.01	-0.008	-0.017	-0.007	-0.01	t	41604	MGLKVNVSAIFMAVL	Homo sapiens
1	1.00	1	1	0	-0.003	0	0	-0.004	t	64628	TKNTNNNLTL	Oryctolagus cuniculus
1	1.00	1	1	-0.02	-0.012	-0.02	-0.02	-0.022	t	156945	QLHTSI	Gallus gallus

1	1.00	1	1	0	0.003	-0.007	0	-0.002	t	41430	MERVVEQ MCITQYQ RESQAY	Mus musculu s
1	1.00	1	1	0.05	0.052	0.057	0.057	0.05	t	138217	PREQQFN	Homo sapiens
1	1.00	1	1	0.03	0.032	0.023	0.033	0.03	t	130608	SIDLKDKYK NIGA	Homo sapiens
1	1.00	1	1	0.01	0.01	-0.001	0.01	0.011	t	79898	LDPLVTAL LGAFD	Mus musculu s
1	1.00	1	1	0.01	0.01	-0.001	0.01	0.011	t	79898	LDPLVTAL LGAFD	Mus musculu s
1	1.00	1	1	0	-0.002	-0.01	0.003	0	t	58036	SGFSETVTI VEDTRP	Mus musculu s
1	1.00	1	1	0.03	0.032	0.013	0.031	0.032	t	151070	WTLVEPG DKITFEAT	Homo sapiens
1	1.00	1	1	0.01	0.012	-0.004	0.007	0.011	t	78412	GYKDVDK APFNGMT GCG	Homo sapiens
1	1.00	1	1	-0.03	-0.03	-0.048	-0.033	-0.03	t	63798	TGCRAM	Homo sapiens
1	1.00	1	1	0	-0.002	0	0.001	-0.003	t	131101	TDSEMDV VRFDDDN S	Mus musculu s
1	1.00	1	1	-0.03	-0.03	-0.03	-0.029	-0.03	t	61553	STDKNRGF	Homo sapiens
1	1.00	1	1	0.02	0.022	0.013	0.021	0.019	t	18743	GAQLNAM KPDLQNTL	Homo sapiens
1	1.00	1	1	0.02	0.018	0.01	0.023	0.02	t	69171	VIYSKYGG TE	Mus musculu s
1	1.00	1	1	0	0.004	-0.003	0.001	-0.003	t	103548	RGLRAAAE AGVRLAF	Homo sapiens

1	1.00	1	1	0.04	0.042	0.045	0.041	0.04	t	17557	FQYSPG	Homo sapiens
1	1.00	1	1	0	0	-0.005	-0.003	-0.002	t	42162	MMNWSP TTC	Homo sapiens
1	1.00	1	1	0.01	0.007	0.008	0.01	0.008	t	141622	DPNGWTG TDNNFSI	Oryctola gus cuniculu s
1	1.00	1	1	0.01	0.014	0.002	0.01	0.01	t	140594	RELVLAGTI	Cavia porcellu s
1	1.00	1	1	0.01	0.014	-0.004	0.01	0.01	t	21956	GQSFQNT WEGQAAS A	Mus musculu s
1	1.00	1	1	0.01	0.011	-0.004	0.01	0.01	t	21956	GQSFQNT WEGQAAS A	Oryctola gus cuniculu s
1	1.00	1	1	-0.01	-0.013	-0.005	-0.01	-0.012	t	95979	VPQPPVS QFHIQ	Oryctola gus cuniculu s
1	1.00	1	1	0.01	0.008	0.009	0.01	0.009	t	95092	GYQKTVD HTKVNSK	Oryctola gus cuniculu s
1	1.00	1	1	-0.01	-0.01	-0.01	-0.01	-0.012	t	122465	LKRDMKS AHLLPEH	Homo sapiens
1	1.00	1	1	0	-0.003	-0.01	0	0.002	t	72903	WNPTLLG E	Oryctola gus cuniculu s
1	1.00	1	1	0.01	0.007	-0.005	0.009	0.01	t	68584	VFTKENTI	Oryctola gus cuniculu s
1	1.00	1	1	-0.04	-0.042	-0.044	-0.037	-0.037	t	78186	KMDKLELK G	Mus musculu s
1	1.00	1	1	-0.01	-0.01	-0.01	-0.01	-0.01	t	46860	PALVPDKE V	Homo sapiens

1	1.00	1	1	0.02	0.017	0.016	0.02	0.02	t	76350	YVLLSEKKIS	Oryctolagus cuniculus
1	1.00	1	1	0.03	0.026	0.023	0.033	0.032	t	20464	GITQGIVE	Oryctolagus cuniculus
1	1.00	1	1	0.03	0.03	0.021	0.03	0.03	t	119599	KAEEYILKKPRSELMFEE	Mus musculus
1	1.00	1	1	0.02	0.02	0.02	0.02	0.019	t	119593	HYTQKSLS	Homo sapiens
1	1.00	1	1	0.02	0.018	0.028	0.021	0.018	t	138930	WDPEEGQIVP	Mus musculus
1	1.00	1	1	0.05	0.049	0.04	0.05	0.05	t	12435	EIEDTEGLKEVIN	Mus musculus
1	1.00	1	1	0.03	0.041	0.03	0.03	0.028	t	156965	SVQERYYAFWST	Gallus gallus
1	1.00	1	1	-0.01	-0.008	-0.009	-0.003	-0.01	t	98435	RYPHYSSLIKPESSL	Homo sapiens
1	1.00	1	1	0	-0.003	0	0	-0.004	t	135921	EVGWYRSPF	Oryctolagus cuniculus
1	1.00	1	1	0.01	0.01	0.01	0.017	0.01	t	118266	APAAGSAPAAAEEKK	Homo sapiens
1	1.00	1	1	0.01	0.007	-0.004	0.01	0.012	t	72903	WNPTLLGE	Oryctolagus cuniculus
1	1.00	1	1	0.03	0.032	0.03	0.037	0.032	t	107315	DGKEYKC	Homo sapiens
1	1.00	1	1	0	0	-0.002	0.007	0	t	120420	AVTYTEHAKRKTVTAM	Homo sapiens

1	1.00	1	1	0	0.002	0	0	0	t	69124	VITGSI	Homo sapiens
1	1.00	1	1	0.01	0.013	0.012	0.01	0.01	t	126328	ISHG	Mus musculus
1	1.00	1	1	0	0	0	0	-0.002	t	48597	PNANPN	Mus musculus
1	1.00	1	1	0.02	0.02	0.005	0.016	0.02	t	71162	VSTQSAKNPPGATV	Homo sapiens
1	1.00	1	1	0.03	0.03	0.03	0.03	0.03	t	43746	NESKYSNT	Mus musculus
1	1.00	1	1	0.05	0.05	0.039	0.05	0.052	t	111790	SLVEETSFIDAGAPTS	Oryctolagus cuniculus
1	1.00	1	1	0.01	0.014	0.019	0.017	0.009	t	119549	YRKTAKEGT	Homo sapiens
1	1.00	1	1	0.01	0.012	0.019	0.017	0.009	t	119549	YRKTAKEGT	Homo sapiens
1	1.00	1	1	0.01	0.012	0.019	0.017	0.009	t	119549	YRKTAKEGT	Homo sapiens
1	1.00	1	1	0.03	0.032	0.039	0.031	0.027	t	82611	GEQQEAIKSA	Homo sapiens
1	1.00	1	1	0.01	0.01	0.003	0.01	0.009	t	19637	GFTTNEPRYNVFAE	Mus musculus
1	1.00	1	1	0.01	0.01	0.003	0.01	0.009	t	19637	GFTTNEPRYNVFAE	Homo sapiens
1	1.00	1	1	0	-0.003	0.005	0.003	0	t	74374	YISPYFINTS	Homo sapiens

1	1.00	1	1	0.03	0.033	0.019	0.03	0.032	t	111742	RRETQ	Mus musculus
1	1.00	1	1	0	-0.003	-0.011	0	0	t	144748	QAVNPNAGN	Oryctolagus cuniculus
1	1.00	1	1	0.06	0.058	0.053	0.057	0.062	t	14576	ETTTIPPS	Anas platyrhynchos
1	1.00	1	1	0.03	0.03	0.03	0.03	0.029	t	136648	SPLNNFYKTQLR	Mus musculus
1	1.00	1	1	0.03	0.032	0.03	0.037	0.03	t	106223	ADGGAQGTA	Homo sapiens
1	1.00	1	1	0	-0.006	-0.012	0	0	t	12375	EHWSYGLRPG	Canis lupus
1	1.00	1	1	0	-0.002	-0.012	0	0	t	12375	EHWSYGLRPG	Mus musculus
1	1.00	1	1	0.03	0.032	0.022	0.031	0.03	t	13460	ENDVLNQETEEEMEK	Homo sapiens
1	1.00	1	1	0	0.001	-0.011	-0.001	0	t	73973	YFYVGLDY	Equus caballus
1	1.00	1	1	0.03	0.03	0.013	0.03	0.032	t	30181	KDKIAKEQENKETIGTLK	Mus musculus
1	1.00	1	1	0	0	0	0.004	0	t	136063	FIRFDKRIEAEE	Homo sapiens
1	1.00	1	1	0	-0.002	-0.01	-0.001	0	t	51168	QKAREAFR	Anas platyrhynchos
1	1.00	1	1	0	-0.002	-0.008	0.003	0	t	45556	NQGAWPAG	Anas platyrhynchos
1	1.00	1	1	0.03	0.03	0.03	0.03	0.031	t	131107	TGVEAAA	Homo sapiens

1	1.00	1	1	0.01	0.013	0.01	0.01	0.008	t	79285	LDPEYWNFRNGDLT	Mus musculus
1	1.00	1	1	0.02	0.022	0.009	0.023	0.02	t	57508	SEKTIWSLIAWQRSNAEFEQ	Homo sapiens
1	1.00	1	1	-0.03	-0.032	-0.031	-0.03	-0.03	t	73745	YEQGGNIVDI	Oryctolagus cuniculus
1	1.00	1	1	-0.03	-0.033	-0.031	-0.03	-0.03	t	73745	YEQGGNIVDI	Oryctolagus cuniculus
1	1.00	1	1	-0.03	-0.033	-0.031	-0.03	-0.03	t	73745	YEQGGNIVDI	Oryctolagus cuniculus
1	1.00	1	1	-0.02	-0.019	-0.036	-0.021	-0.018	t	15289	FATNREKM	Homo sapiens
1	1.00	1	1	-0.01	-0.013	-0.014	-0.01	-0.009	t	141953	LTACKV	Oryctolagus cuniculus
1	1.00	1	1	0	0.002	-0.01	0.003	0	t	2981	ALVREGLRNV	Homo sapiens
1	1.00	1	1	0	0.002	-0.007	0	0	t	140601	SLYSFPEPEA	Homo sapiens
1	1.00	1	1	0	0	0	0	0	t	117621	QVKGFV	Homo sapiens
1	1.00	1	1	-0.02	-0.017	-0.025	-0.02	-0.019	t	126828	PKYA	Mus musculus
1	1.00	1	1	0.01	0.012	0.005	0.011	0.01	t	141967	NGDKSTQKYNELKT	Homo sapiens
1	1.00	1	1	0.04	0.04	0.04	0.04	0.04	t	141067	PLSLEPDP	Mus musculus
1	1.00	1	1	-0.04	-0.04	-0.038	-0.038	-0.04	t	7830	DDMPGCYPMCTLNK	Homo sapiens

1	1.00	1	1	0.04	0.04	0.033	0.04	0.04	t	133611	IVSYLCDLAPEAPPPTLPP	Mus musculus
1	1.00	1	1	0.03	0.028	0.009	0.027	0.032	t	153828	ERAEERAETGESKI	Homo sapiens
1	1.00	1	1	0.01	0.013	0.011	0.01	0.01	t	30863	KFTEPVKDIM	Mus musculus
1	1.00	1	1	0.04	0.038	0.052	0.043	0.039	t	78403	GLQNDF	Mus musculus
1	1.00	1	1	-0.02	-0.023	-0.022	-0.02	-0.021	t	131316	TLMVMVSK	Oryctolagus cuniculus
1	1.00	1	1	0	0	0	0.001	-0.003	t	103838	DAEFRHDSGYEV	Mus musculus
1	1.00	1	1	0.04	0.04	0.038	0.04	0.04	t	56711	SAAQEKISF	Mus musculus
1	1.00	1	1	-0.04	-0.038	-0.04	-0.033	-0.041	t	108152	TCPPCPAPELLGG	Homo sapiens
1	1.00	1	1	0.02	0.028	0.02	0.02	0.018	t	156961	SLLASTT	Gallus gallus
1	1.00	1	1	0.04	0.036	0.038	0.043	0.041	t	99265	GDKELF	Oryctolagus cuniculus
1	1.00	1	1	0.02	0.02	0.018	0.017	0.018	t	16955	FLVLLLFSLL	Homo sapiens
1	1.00	1	1	0.02	0.019	0.008	0.02	0.022	t	7901	DEADRYFTYEEP	Bos taurus
1	1.00	1	1	0.02	0.02	0.011	0.02	0.019	t	137937	NKIFADDI	Rattus norvegicus
1	1.00	1	1	0.01	0.012	0.016	0.011	0.009	t	60810	SRSTSL	Homo sapiens
1	1.00	1	1	0.01	0.012	0.016	0.011	0.009	t	60810	SRSTSL	Homo sapiens

1	1.00	1	1	0	0	0	0	0	t	48753	PPAYEK	Mus musculus
1	1.00	1	1	0	0	0.002	0.011	0	t	118566	QDFSYQRSLKFRPKG	Homo sapiens
1	1.00	1	1	0.02	0.022	0.019	0.021	0.019	t	65479	TNVVIRACNFELCDNPF	Homo sapiens
1	1.00	1	1	-0.01	-0.01	-0.015	-0.009	-0.01	t	122442	KPMSNFRFGENHA	Rattus norvegicus
1	1.00	1	1	-0.03	-0.028	-0.028	-0.03	-0.031	t	114223	PFSRVVHL	Rattus norvegicus
1	1.00	1	1	0.03	0.032	0.025	0.031	0.03	t	2979	ALVPDKEVL	Homo sapiens
1	1.00	1	1	0.03	0.03	0.021	0.03	0.032	t	8475	DGQPAGDRADGQPAGDRA	Mus musculus
1	1.00	1	1	0	0.002	0	0.007	0	t	108104	RRLPFGSQM	Homo sapiens
1	1.00	1	1	0.01	0.014	0.009	0.011	0.012	t	9719	DPPFSP	Homo sapiens
1	1.00	1	1	0.03	0.03	0.03	0.03	0.03	t	105853	GVTHDQLN	Oryctolagus cuniculus
1	1.00	1	1	0.02	0.023	0.01	0.02	0.022	t	120919	RLNEAVKYTG	Mus musculus
1	1.00	1	1	0.04	0.04	0.04	0.04	0.04	t	111690	QTATLTY	Homo sapiens
1	1.00	1	1	-0.01	-0.007	-0.01	-0.007	-0.01	t	11819	EENVEHDA	Aotus
1	1.00	1	1	0.03	0.026	0.03	0.023	0.031	t	107784	DGSFFLY	Oryctolagus cuniculus

1	1.00	1	1	0.05	0.046	0.052	0.053	0.051	t	99359	IVDDVNC	Oryctolagus cuniculus
1	1.00	1	1	0	-0.002	0	-0.001	0.002	t	68548	VFPTKDVAL	Mus musculus
1	1.00	1	1	-0.01	-0.007	-0.025	-0.01	-0.008	t	104298	TEATAIRPAQVGYNT	Sus scrofa
1	1.00	1	1	0.03	0.026	0.03	0.033	0.028	t	156843	AHSEMGTRATMT	Bos taurus
1	1.00	1	1	0.01	0.008	0.004	0.007	0.009	t	49377	PSGLVIP	Mus musculus
1	1.00	1	1	-0.01	-0.012	-0.02	-0.007	-0.008	t	39980	LTIVSGGPTLFS	Mus musculus
1	1.00	1	1	0.05	0.048	0.041	0.05	0.051	t	100635	VTNYLVDGNG	Mus musculus
1	1.00	1	1	0	-0.002	-0.008	0.003	0.002	t	95323	DPTTNV	Mus musculus
1	1.00	1	1	0.01	0.007	0.015	0.01	0.006	t	6385	CHGIFTLTWEIWGY	Oryctolagus cuniculus
1	1.00	1	1	0.03	0.026	0.023	0.029	0.028	t	116974	FKEQFLDGDGW	Oryctolagus cuniculus
1	1.00	1	1	0.02	0.02	0.011	0.02	0.019	t	48858	PPPPNPNDPPPPNPND	Mus musculus
1	1.00	1	1	0.04	0.04	0.04	0.04	0.041	t	115308	KFLDDDLTDD	Homo sapiens
1	1.00	1	1	-0.01	-0.012	-0.018	-0.011	-0.012	t	22784	GTPNPNPNPNPNPNPGTT	Mus musculus
1	1.00	1	1	0	0	-0.004	-0.004	0.001	t	134143	LMPPKY	Homo sapiens

1	1.00	1	1	0.02	0.022	0.013	0.023	0.021	t	144682	HLVGSALSDP	Homo sapiens
1	1.00	1	1	0.01	0.01	-0.002	0.008	0.01	t	54923	RMMATKDSFQSFN	Homo sapiens
1	1.00	1	1	-0.01	-0.01	-0.008	-0.01	-0.014	t	125052	FSLRLMEENW	Mus musculus
1	1.00	1	1	0	0.005	-0.002	0.003	0	t	33748	KTKRSAS	Homo sapiens
1	1.00	1	1	-0.02	-0.018	-0.029	-0.021	-0.02	t	53693	RFDRGYIS	Homo sapiens
1	1.00	1	1	0.03	0.03	0.03	0.03	0.029	t	119576	EYKCKVSN	Homo sapiens
1	1.00	1	1	0.03	0.032	0.025	0.031	0.03	t	97598	RDLLKKGVDA	Homo sapiens
1	1.00	1	1	0.01	0.01	0.003	0.01	0.009	t	130585	QKKIRGILE	Mus musculus
1	1.00	1	1	0.01	0.006	-0.002	0.009	0.01	t	17545	FQVEVP	Oryctolagus cuniculus
1	1.00	1	1	-0.01	-0.01	-0.024	-0.01	-0.009	t	78424	IPRSRQIDLQIG	Mus musculus
1	1.00	1	1	0.06	0.06	0.05	0.06	0.06	t	49646	PTGTPLDIDTASE	Mus musculus
1	1.00	1	1	0.05	0.052	0.04	0.053	0.052	t	125229	RDSEGDLVAEKLGPI	Homo sapiens
1	1.00	1	1	0.01	0.012	-0.002	0.001	0.008	t	115700	CEKDERFFSD	Homo sapiens
1	1.00	1	1	0.03	0.028	0.025	0.029	0.03	t	4448	ASHLEQYGTNEYS	Mus musculus
1	1.00	1	1	-0.01	-0.011	-0.022	-0.009	-0.009	t	1022	AEKQKAAEATKVAE	Homo sapiens

1	1.00	1	1	0.03	0.028	0.023	0.029	0.03	t	88388	SAWNSDSEKPFDDHL	Mus musculus
1	1.00	1	1	-0.02	-0.02	-0.02	-0.014	-0.021	t	19737	GGGAGAGGAGAGGGGR	Homo sapiens
1	1.00	1	1	0.04	0.04	0.032	0.04	0.04	t	6953	CRTNYGYS	Oryctolagus cuniculus
1	1.00	1	1	-0.02	-0.016	-0.02	-0.017	-0.02	t	41659	MHGDTPTLHEYM	Homo sapiens
1	1.00	1	1	-0.03	-0.025	-0.027	-0.032	-0.033	t	157292	GAIIGLM	Homo sapiens
1	1.00	1	1	0.06	0.06	0.063	0.062	0.06	t	142225	LNDSSEEEDEID	Homo sapiens
1	1.00	1	1	-0.05	-0.052	-0.054	-0.051	-0.05	t	145934	PDIIPK	Mus musculus
1	1.00	1	1	0.04	0.036	0.042	0.039	0.04	t	32116	KLQPGDDPLLG	Oryctolagus cuniculus
1	1.00	1	1	0	0	0	0.004	-0.002	t	110284	HKSAIVTLTYDSEWQRDQ	Homo sapiens
1	1.00	1	1	0	0.003	-0.003	0.003	0	t	73254	WWYEGLPQQHYIGLVA	Equus caballus
1	1.00	1	1	0	0	0	0	-0.003	t	130567	NPCEDI	Mus musculus
1	1.00	1	1	0	0	0	0	-0.003	t	130559	NAHYYVFFEEQE	Mus musculus
1	1.00	1	1	0	0	0	0	-0.003	t	130581	QAFDSH	Mus musculus
1	1.00	1	1	0	0	0	0	-0.003	t	130625	TVIDKDAFG	Mus musculus

1	1.00	1	1	0	0	0	0	-0.003	t	130916	KEDFDIYD EDE	Mus musculu s
1	1.00	1	1	0	0	0	0	-0.003	t	133372	SPSPTPTP GPSRRG	Homo sapiens
1	1.00	1	1	0	0	0	0	-0.003	t	122562	RRQPHVV SQW	Mus musculu s
1	1.00	1	1	0	0	0	0	-0.003	t	130585	QKKIRGILE	Mus musculu s
1	1.00	1	1	0	0	0	0	-0.003	t	110760	ACKDSDW PFCSDED WDWPFC	Mus musculu s
1	1.00	1	1	0	0	0	0	-0.003	t	115399	NMAINPS KENLCSTF CKEVV	Homo sapiens
1	1.00	1	1	0	0	0	0	-0.003	t	113731	PTSPQQSG QGQQPG QGQQ	Homo sapiens
1	1.00	1	1	0	0	0	0	-0.003	t	110946	MSTRSVSS SSYRRMFG GPGT	Mus musculu s
1	1.00	1	1	0	0	0	0	-0.003	t	110833	EQFNWVS RLANLTQG EDQYY	Mus musculu s
1	1.00	1	1	0	0	0	0	-0.003	t	115379	MAIPPKKN QDKTEIPTI NTI	Homo sapiens
1	1.00	1	1	0	0	0	0	-0.003	t	110821	DVRQQYE SVAAKNL QEAEEW	Mus musculu s
1	1.00	1	1	0	0	0	0	-0.003	t	110794	CDDDDDN DGVPDSR DNCRLV	Mus musculu s
1	1.00	1	1	0	0	0	0	-0.003	t	109917	SHQESTRG RSRGRSGR SGS	Mus musculu s
1	1.00	1	1	0	0	0	0	-0.003	t	110946	MSTRSVSS SSYRRMFG GPGT	Mus musculu s
1	1.00	1	1	0	0	0	0	-0.003	t	111042	TIHAHPGS RRGGRHG YHH	Mus musculu s
1	1.00	1	1	0	0	0	0	-0.003	t	110820	DVRQQYE SVAAKNL QEAEEW	Mus musculu s
1	1.00	1	1	0	0	0	0	-0.003	t	110798	CPTGCQL QEALLQQE RPIRN	Mus musculu s

1	1.00	1	1	0	0	0	0	-0.003	t	110997	RCHAANPNGRYYWGGQYTWD	Mus musculus
1	1.00	1	1	0	0	0	0	-0.003	t	115532	VATEEVKITVDDKHYQKALN	Homo sapiens
1	1.00	1	1	0	0	0	0	-0.003	t	110834	EQLKGQGKSRLGDLYEEEMR	Mus musculus
1	1.00	1	1	0	0	0	0	-0.003	t	110999	REEAENTLQSFRQDVNTLQS	Mus musculus
1	1.00	1	1	0	0	0	0	-0.003	t	111033	STYDRDNDGWLTSDPRKQCS	Mus musculus
1	1.00	1	1	0	0	0	0	-0.003	t	110820	DVRQQYESVAAKNLQEAEEW	Mus musculus
1	1.00	1	1	0	0	0	0	-0.003	t	110955	NQNPGSPRPGSTGTWNPGSS	Mus musculus
1	1.00	1	1	0	0	0	0	-0.003	t	113580	KPQQQSPQQQFPQQQIPQQQ	Homo sapiens
1	1.00	1	1	0	0	0	0	-0.003	t	115437	QKALNEINQFYQKFPQYLQY	Homo sapiens
1	1.00	1	1	0	0	0	0	-0.003	t	78939	CINHRGYWVC	Mus musculus
1	1.00	1	1	0	0	0	0	-0.003	t	51827	QPFPQPQLPYPQPQ	Mus musculus
1	1.00	1	1	0	0	0	0	-0.003	t	8756	DIPNPIGSENSEKTTMPLW	Homo sapiens
1	1.00	1	1	0	0	0	0.001	0	t	66311	TSLNLQKDEPN	Equus caballus
1	1.00	1	1	0.01	0.003	0.01	0.01	0.009	t	153838	HQKRTAMFQDPQER	Oryctolagus cuniculus
1	1.00	1	1	-0.04	-0.04	-0.047	-0.04	-0.041	t	95041	SLAGKREMA	Oryctolagus cuniculus

1	1.00	1	1	0	0	-0.007	0	0	t	112841	SSKRGRQTPVLYAMLDH	Rattus norvegicus
1	1.00	1	1	0.08	0.08	0.08	0.08	0.078	t	129058	KEESEESDEDMGFGL	Homo sapiens
1	1.00	1	1	0.01	0.005	-0.005	0.013	0.012	t	95277	CGTTDSTATTKISEVFTE	Bos taurus
1	1.00	1	1	0.01	0.014	0.011	0.007	0.008	t	30141	KDERFFSDKI	Homo sapiens
1	1.00	1	1	0.04	0.038	0.033	0.043	0.04	t	13984	ERESQAY	Mus musculus
1	1.00	1	1	0	0	-0.013	0	0	t	159083	RYKYLPETPKQLAKVV	Mus musculus
1	1.00	1	1	-0.02	-0.018	-0.031	-0.019	-0.02	t	110468	YYVLHLCLAATKYPLLKLLG	Homo sapiens
1	1.00	1	1	0	0	-0.007	0	0	t	70439	VPPVIKQVSA	Homo sapiens
1	1.00	1	1	0.01	0.013	0.001	0.01	0.01	t	96874	SIAPDLSLDFEKLNV	Mus musculus
1	1.00	1	1	0.01	0.013	0.001	0.01	0.01	t	96874	SIAPDLSLDFEKLNV	Mus musculus
1	1.00	1	1	0	0	0	0	-0.001	t	133539	CYKPGEWAC	Mus musculus
1	1.00	1	1	0	0	0	0.006	0	t	43242	NANPNANPNANP	Homo sapiens
1	1.00	1	1	0	0	0	0.006	0	t	43242	NANPNANPNANP	Homo sapiens
1	1.00	1	1	-0.03	-0.032	-0.034	-0.031	-0.031	t	31834	KLFAVWKITYKDT	Mus musculus

1	1.00	1	1	0.01	0.01	0.01	0.01	0.01	t	23223	GVYLLPRRGP	Homo sapiens
1	1.00	1	1	0	0	-0.019	0	0.003	t	7756	DDDMGFGLFD	Homo sapiens
1	1.00	1	1	0.01	0.013	0.01	0.01	0.008	t	135501	SQWNKP	Mus musculus
1	1.00	1	1	0	-0.02	-0.003	0.001	-0.002	t	111240	EGTEAVPGDPDVNRF	Cricetinae
1	1.00	1	1	0.02	0.016	0.014	0.019	0.02	t	42985	MVQVVYDYQH	Oryctolagus cuniculus
1	1.00	1	1	0	0.001	-0.001	0.003	-0.001	t	99579	QNIRQGAKEPYPEFV	Equus caballus
1	1.00	1	1	0.01	0.01	0.005	0.004	0.007	t	115675	AFLLYQEPVL	Homo sapiens
1	1.00	1	1	0.03	0.027	0.022	0.03	0.03	t	62967	TAQAAPVQEG	Mus musculus
1	1.00	1	1	-0.01	-0.006	-0.002	-0.003	-0.01	t	121473	KKAKAKAK	Homo sapiens
1	1.00	1	1	-0.01	-0.01	-0.01	-0.006	-0.01	t	119578	FAIVKFTKVLLQYTGH	Homo sapiens
1	1.00	1	1	0.06	0.069	0.054	0.06	0.06	t	64557	TKEYEDIV	Oryctolagus cuniculus
1	1.00	1	1	0	0.002	0	-0.003	-0.002	t	25445	IAT	Homo sapiens
1	1.00	1	1	0.02	0.018	0.016	0.017	0.019	t	52420	QSRGEAR	Mus musculus
1	1.00	1	1	0.03	0.028	0.02	0.027	0.031	t	72065	VYKLDISEA	Mus musculus
1	1.00	1	1	0.01	0.01	0.01	0.021	0.012	t	118441	IWLWNKQKYEAGTYPQT	Homo sapiens

1	1.00	1	1	0.01	0.012	0.005	0.013	0.011	t	114318	ELLHQRFFKNVESTP	Homo sapiens
1	1.00	1	1	0	-0.003	-0.001	0	0	t	30145	KDFLEVTF	Oryctolagus cuniculus
1	1.00	1	1	-0.01	-0.01	-0.01	-0.01	-0.01	t	47124	PDKEVLYQQ	Homo sapiens
1	1.00	1	1	0.03	0.028	0.022	0.029	0.031	t	10336	DTFNPVYPYDTE	Mus musculus
1	1.00	1	1	0.03	0.03	0.026	0.03	0.032	t	66385	TSQGMYGGTYLVEKP	Homo sapiens
1	1.00	1	1	-0.01	-0.006	-0.017	-0.007	-0.008	t	144700	KEWAKRAALP	Homo sapiens
1	1.00	1	1	-0.05	-0.05	-0.059	-0.046	-0.05	t	136002	TKQMVQMRRKLE	Homo sapiens
1	1.00	1	1	-0.01	-0.014	-0.006	-0.007	-0.01	t	97207	APAFADVVI	Oryctolagus cuniculus
1	1.00	1	1	0	0.002	-0.017	0.001	0.002	t	32687	KPEASSTN	Homo sapiens
1	1.00	1	1	-0.01	-0.01	-0.01	-0.01	-0.012	t	63037	TAVSPTTLR	Mus musculus
1	1.00	1	1	0	0	0	0.011	0.002	t	118601	SGKGKVQFQGKKTKF	Homo sapiens
1	1.00	1	1	0	-0.003	-0.009	0	0	t	27959	IPNGTYRA	Oryctolagus cuniculus
1	1.00	1	1	0	0	0	0	-0.002	t	43704	NENLLRFFVAPFPE	Homo sapiens
1	1.00	1	1	-0.01	-0.01	-0.023	-0.01	-0.008	t	96849	SAETIF	Mus musculus

1	1.00	1	1	-0.01	-0.01	-0.023	-0.01	-0.008	t	96849	SAETIF	Mus musculus
1	1.00	1	1	-0.01	-0.013	-0.023	-0.01	-0.008	t	96849	SAETIF	Oryctolagus cuniculus
1	1.00	1	1	0.02	0.017	0.007	0.02	0.022	t	82932	GTNVELGGRLSI	Oryctolagus cuniculus
1	1.00	1	1	-0.01	-0.014	-0.011	-0.011	-0.012	t	78615	CPGHANRKQCGG	Oryctolagus cuniculus
1	1.00	1	1	0.02	0.02	0.02	0.02	0.02	t	18808	GAVDSI	Homo sapiens
1	1.00	1	1	0.04	0.038	0.025	0.043	0.042	t	33717	KTIINNDFNFDDYNY	Mus musculus
1	1.00	1	1	-0.01	-0.01	-0.005	-0.003	-0.012	t	29265	IVFKT	Homo sapiens
1	1.00	1	1	0.01	0.006	-0.008	0.01	0.012	t	49636	PTGPTIAAGGEQPPS	Bos taurus
1	1.00	1	1	-0.01	-0.006	-0.01	-0.007	-0.011	t	118430	ISDAMKKVGRKGV	Homo sapiens
1	1.00	1	1	0.01	0.008	0.006	0.013	0.01	t	158947	LDIDIDPKVKVATLS	Mus musculus
1	1.00	1	1	0.03	0.026	0.023	0.027	0.03	t	49703	PTLLGEAT	Oryctolagus cuniculus
1	1.00	1	1	0	0	0	0.004	-0.001	t	117315	KALSVETEKLLKYLEAV	Homo sapiens
1	1.00	1	1	0	0	0	0.004	-0.001	t	117336	NGWSHKDLLR	Homo sapiens
1	1.00	1	1	0	0	0	0	0	t	6059	CCDLAP	Homo sapiens
1	1.00	1	1	-0.03	-0.03	-0.035	-0.03	-0.03	t	107126	GAPGTPGPQG	Mus musculus

1	1.00	1	1	-0.03	-0.028	-0.027	-0.029	-0.032	t	41171	MAWDMMMNW	Homo sapiens
1	1.00	1	1	0	0	-0.002	0	-0.002	t	53481	REEAYKEQIKTLTNK	Homo sapiens
1	1.00	1	1	-0.01	-0.01	-0.023	-0.003	-0.008	t	14426	ETIGTLKKILDETVK	Homo sapiens
1	1.00	1	1	0.01	0.008	0.007	0.013	0.01	t	52825	QVYYRPVDQ	Mus musculus
1	1.00	1	1	0.02	0.02	0.02	0.017	0.017	t	111030	SRGSEGFFSPMLGEFVSETE	Mus musculus
1	1.00	1	1	0.01	0.008	0.012	0.013	0.011	t	98980	RELSA	Mus musculus
1	1.00	1	1	0	0	0	0.007	0	t	125552	RRFEYDDPRFLRLLDLAQEG	Homo sapiens
1	1.00	1	1	0	0	-0.002	0	0.001	t	17504	FQNEKWDL	Mus musculus
1	1.00	1	1	-0.03	-0.034	-0.03	-0.03	-0.032	t	70395	VPNLRGDRHALA	Mus musculus
1	1.00	1	1	0.03	0.026	0.02	0.033	0.03	t	11090	EAKESPANNPGLSTV	Oryctolagus cuniculus
1	1.00	1	1	0.02	0.02	0.015	0.02	0.021	t	114224	PQDENPVVHF	Mus musculus
1	1.00	1	1	0.03	0.034	0.023	0.033	0.033	t	142474	SSTTGVAM	Homo sapiens
1	1.00	1	1	0	0.002	-0.005	0.001	0	t	44144	NHIHSV	Homo sapiens
1	1.00	1	1	0.06	0.06	0.058	0.06	0.062	t	137209	ITANVTDESYLY	Sus scrofa
1	1.00	1	1	0.01	0.01	0.01	0.01	0.01	t	96715	NVPKVTKEKP	Homo sapiens(human)

1	1.00	1	1	-0.01	-0.01	-0.01	-0.01	-0.01	t	35247	LDPAFR	Mus musculus
1	1.00	1	1	0.03	0.03	0.018	0.03	0.034	t	133552	ECAYCLTI	Mus musculus
1	1.00	1	1	0.04	0.038	0.035	0.037	0.037	t	116000	TPTTEAVEST	Homo sapiens
1	1.00	1	1	0	-0.002	-0.02	-0.007	0	t	127950	DVPVVTSVTTAGVGPSPDIT	Mus musculus
1	1.00	1	1	0	0.004	0.002	0.007	-0.001	t	119453	FSNHGEIKWIDFVRGAKEGI	Homo sapiens
1	1.00	1	1	-0.01	-0.019	-0.02	-0.01	-0.008	t	60478	SQKNGGYV	Macaca mulatta
1	1.00	1	1	-0.01	-0.01	-0.007	-0.01	-0.01	t	59112	SLFTPGP	Homo sapiens
1	1.00	1	1	0.02	0.017	0.013	0.02	0.019	t	137828	DVLLAGNAEADR	Oryctolagus cuniculus
1	1.00	1	1	0.02	0.006	0.013	0.02	0.019	t	137828	DVLLAGNAEADR	Oryctolagus cuniculus
1	1.00	1	1	0.01	0.008	0.002	0.009	0.011	t	11744	EELFKEYK	Mus musculus
1	1.00	1	1	-0.02	-0.02	-0.025	-0.02	-0.021	t	12539	EIPALTAVE	Mus musculus
1	1.00	1	1	0.05	0.046	0.041	0.05	0.05	t	20046	GGTEESVVTASRS	Bos taurus
1	1.00	1	1	0.03	0.03	0.023	0.03	0.03	t	63662	TFGLQLELT	Rattus norvegicus
1	1.00	1	1	0.01	0.008	0	0.007	0.01	t	32787	KPLEDKILVQ	Mus musculus
1	1.00	1	1	0	0	0	0.004	0	t	117140	DWEYSVWLSN	Homo sapiens

1	1.00	1	1	0.02	0.02	0.008	0.017	0.02	t	75991	YTDAPSFS DIPNPI	Homo sapiens
1	1.00	1	1	-0.03	-0.028	-0.037	-0.029	-0.03	t	98354	PVVRNNIY HI	Homo sapiens
1	1.00	1	1	0	-0.002	-0.004	-0.003	0	t	67297	TWSGKFP T	Anas platyrhy nchos
1	1.00	1	1	0.01	0.01	0.003	0.01	0.009	t	159050	PKRLTATT EKLEIGWK	Mus musculu s
1	1.00	1	1	-0.01	-0.01	-0.022	-0.008	-0.008	t	96453	KFKEAFLR SV	Homo sapiens(human)
1	1.00	1	1	0	-0.004	-0.012	-0.007	-0.003	t	131015	QDRRLDE N	Oryctola gus cuniculu s
1	1.00	1	1	-0.03	-0.027	-0.037	-0.03	-0.031	t	33794	KTNMKH M	Mus musculu s
1	1.00	1	1	0.02	0.015	0.022	0.02	0.02	t	45462	NPQVNLV D	Bos taurus
1	1.00	1	1	0.02	0.015	0.022	0.02	0.02	t	45462	NPQVNLV D	Bos taurus
1	1.00	1	1	-0.01	-0.008	-0.025	-0.013	-0.01	t	38937	LQPFPQPE	Homo sapiens
1	1.00	1	1	0.01	0.012	0.009	0.011	0.012	t	47426	PESPFA	Homo sapiens
1	1.00	1	1	-0.01	-0.007	-0.01	-0.01	-0.011	t	78344	AEIRGERK	Mus musculu s
1	1.00	1	1	0.01	0.012	0.012	0.011	0.009	t	40611	LVTKDIDF D	Homo sapiens
1	1.00	1	1	0.03	0.032	0.026	0.027	0.027	t	114761	DCSEYPKP DC	Homo sapiens
1	1.00	1	1	0.02	0.017	0.013	0.02	0.018	t	101309	TPIESHQV EKR	Oryctola gus cuniculu s

1	1.00	1	1	-0.03	-0.028	-0.041	-0.03	-0.03	t	54095	RHRQPRG WEQ	Homo sapiens
1	1.00	1	1	-0.03	-0.028	-0.041	-0.03	-0.03	t	54095	RHRQPRG WEQ	Homo sapiens
1	1.00	1	1	-0.04	-0.043	-0.052	-0.04	-0.04	t	111051	VGPLEVIP EAVDPAG SSIV	Oryctola gus cuniculu s
1	1.00	1	1	-0.04	-0.043	-0.052	-0.04	-0.04	t	111051	VGPLEVIP EAVDPAG SSIV	Oryctola gus cuniculu s
1	1.00	1	1	0	-0.003	-0.008	0	0	t	84250	KEVAREAS KKK	Oryctola gus cuniculu s
1	1.00	1	1	-0.01	-0.008	-0.018	-0.013	-0.012	t	13911	EQQRFRN CQRYV	Homo sapiens
1	1.00	1	1	0	-0.002	0	0.006	-0.001	t	3236	ANCASILC KCYTTGT	Mus musculu s
1	1.00	1	1	0	-0.002	0	0.006	-0.001	t	3236	ANCASILC KCYTTGT	Mus musculu s
1	1.00	1	1	-0.02	-0.02	-0.028	-0.02	-0.021	t	63967	TGSQDFFV RSIG	Mus musculu s
1	1.00	1	1	-0.03	-0.03	-0.025	-0.023	-0.031	t	140672	EQIYRVDL RTLR	Rattus norvegic us
1	1.00	1	1	0.02	0.022	0.013	0.027	0.021	t	121475	KKGEKVPK GKKGKAD	Homo sapiens
1	1.00	1	1	0	0.002	0.007	0.001	-0.003	t	49764	PTRTIGGS QA	Homo sapiens
1	1.00	1	1	0.02	0.02	0.016	0.02	0.02	t	76350	YVLLSEKKI S	Homo sapiens
1	1.00	1	1	0.03	0.03	0.017	0.023	0.03	t	141931	ITKLTPEEL ENLAK	Homo sapiens
1	1.00	1	1	0.07	0.07	0.07	0.07	0.07	t	142449	NNQNLSV VQALL	Mus musculu s

1	1.00	1	1	0	0.003	-0.005	0	0.001	t	141567	TQDGLYHNKN	Mus musculus
1	1.00	1	1	-0.01	-0.012	-0.019	-0.007	-0.007	t	115483	SFFDIGGSVSAGFSN	Mus musculus
1	1.00	1	1	0	0.002	-0.017	0	0.003	t	68833	VHASFFDIG	Homo sapiens
1	1.00	1	1	0	0	-0.017	0	0.003	t	68833	VHASFFDIG	Mus musculus
1	1.00	1	1	0.05	0.053	0.042	0.05	0.05	t	3893	APVQEGVQQE	Mus musculus
1	1.00	1	1	0.05	0.05	0.042	0.05	0.05	t	3893	APVQEGVQQE	Mus musculus
1	1.00	1	1	0	0.006	-0.01	0	0	t	91345	FYQEVLFKGFEAV	Equus caballus
1	1.00	1	1	0	0	-0.014	0	0	t	18025	FTNKLKEKHTDLG	Mus musculus
1	1.00	1	1	0.06	0.06	0.06	0.064	0.06	t	144812	VTGCIPTEEQ	Homo sapiens
1	1.00	1	1	0.01	0.008	-0.006	0.003	0.01	t	98364	QFVPIQNSKS	Mus musculus
1	1.00	1	1	0.04	0.042	0.042	0.039	0.037	t	96828	RLKEEEEDKK	Homo sapiens
1	1.00	1	1	0	-0.003	0	-0.003	-0.005	t	99922	DAEFRHDSGY	Oryctolagus cuniculus
1	1.00	1	1	0.01	0.01	0.007	0.01	0.009	t	98763	ENKVLVLDTD	Homo sapiens
1	1.00	1	1	-0.02	-0.024	-0.026	-0.017	-0.021	t	7558	DAKDGKEIDTKHLPSTC	Oryctolagus cuniculus

1	1.00	1	1	0.02	0.017	0.006	0.019	0.02	t	14227	ESPANNPGLSTVRDE	Oryctolagus cuniculus
1	1.00	1	1	-0.02	-0.018	-0.02	-0.019	-0.02	t	51428	QLLRIPQAIMDM	Homo sapiens
1	1.00	1	1	-0.04	-0.043	-0.043	-0.04	-0.039	t	124708	SFERFEIF	Oryctolagus cuniculus
1	1.00	1	1	0.01	0.014	0.017	0.011	0.01	t	43693	NEMVLL	Homo sapiens
1	1.00	1	1	0.04	0.04	0.033	0.04	0.039	t	57747	SFEEDTEKDK	Oryctolagus cuniculus
1	1.00	1	1	-0.03	-0.027	-0.039	-0.03	-0.03	t	100318	NAPKTFQFIN	Mus musculus
1	1.00	1	1	-0.02	-0.016	-0.038	-0.023	-0.02	t	20683	GKSMANTWPYFGYYMGGNML	Homo sapiens
1	1.00	1	1	0.04	0.042	0.04	0.043	0.041	t	68017	VDLPQL	Homo sapiens
1	1.00	1	1	0	-0.003	0	0	-0.003	t	11815	EENVEENVEENVEENV	Oryctolagus cuniculus
1	1.00	1	1	0.01	0.013	0.004	0.01	0.013	t	94564	KIAKEQ	Mus musculus
1	1.00	1	1	0.01	0.008	-0.01	0.003	0.01	t	128090	QYYRLPMNAYRDFTSEPLNS	Mus musculus
1	1.00	1	1	0.01	0.008	0.01	0.017	0.01	t	122996	AAFQQGKIPP	Homo sapiens
1	1.00	1	1	0.01	0.014	0.007	0.01	0.01	t	123117	EEVKGDYLK	Ovis aries
1	1.00	1	1	0.05	0.052	0.045	0.051	0.05	t	16144	FHSTSSESN	Homo sapiens

1	1.00	1	1	-0.01	-0.012	-0.01	-0.011	-0.01	t	46969	PATIGDYTDF	Mus musculus
1	1.00	1	1	0.01	0.012	0.01	0.011	0.01	t	60709	SRKAYDHNSP	Homo sapiens
1	1.00	1	1	0.01	0.008	0.01	0.011	0.01	t	60709	SRKAYDHNSP	Homo sapiens
1	1.00	1	1	0.02	0.02	0.016	0.02	0.02	t	152053	PYCLGSPSQDGY	Sus scrofa
1	1.00	1	1	0.01	0.008	0.01	0.013	0.01	t	51929	QPQQPQQSFP	Mus musculus
1	1.00	1	1	0.01	0.008	-0.006	0.003	0.01	t	98378	QNSKSAYTPA	Mus musculus
1	1.00	1	1	0	0.003	0.002	0.007	0.002	t	104895	LVEALYLVCGERG	Mus musculus
1	1.00	1	1	-0.01	-0.012	-0.01	-0.013	-0.013	t	131656	SGKLEEFVQ	Mus musculus
1	1.00	1	1	0.03	0.027	0.03	0.03	0.027	t	106764	QYTSIHHGVVE	Oryctolagus cuniculus
1	1.00	1	1	0.03	0.038	0.03	0.03	0.029	t	136648	SPLNNFYKTQLR	Gallus gallus
1	1.00	1	1	0.01	0.012	0.003	0.017	0.011	t	121474	KKAPAKKGEKVPKGK	Homo sapiens
1	1.00	1	1	-0.01	-0.01	-0.015	-0.007	-0.011	t	56804	SAGRSTGSLVRLFAPG	Homo sapiens
1	1.00	1	1	-0.03	-0.033	-0.041	-0.03	-0.03	t	6540	CLAVLLGA	Oryctolagus cuniculus
1	1.00	1	1	0.05	0.05	0.044	0.044	0.049	t	12187	EGIHAQQKEP	Homo sapiens
1	1.00	1	1	0.04	0.04	0.04	0.04	0.04	t	132268	ENGYLMEAAP	Homo sapiens

1	1.00	1	1	0	0.006	-0.018	0	0.002	t	92556	EIIGQNGERI	Ovis aries
1	1.00	1	1	0.02	0.02	0.026	0.021	0.02	t	34238	KVVLPE	Homo sapiens
1	1.00	1	1	-0.02	-0.022	-0.025	-0.021	-0.02	t	74476	YKLPDHVRT	Mus musculus
1	1.00	1	1	0.05	0.05	0.041	0.048	0.05	t	135974	PSNSASVPALTA	Homo sapiens
1	1.00	1	1	0.05	0.048	0.05	0.049	0.05	t	48900	PPQWKLQP	Anas platyrhynchos
1	1.00	1	1	0.01	0.006	-0.002	0.009	0.01	t	60446	SQHIDS	Oryctolagus cuniculus
1	1.00	1	1	-0.01	-0.01	-0.01	-0.01	-0.012	t	67605	VAGLEK	Mus musculus
1	1.00	1	1	0	-0.004	-0.005	0.003	0	t	95414	GLDLDVNVFR	Oryctolagus cuniculus
1	1.00	1	1	0.04	0.04	0.04	0.04	0.04	t	124015	DKTEDVDIEE	Homo sapiens
1	1.00	1	1	0	0	0	0.007	0	t	138812	SSVLYGGPPSAA	Rattus norvegicus
1	1.00	1	1	0	0	0	0.007	0	t	140718	NKWERARYAER	Rattus norvegicus
1	1.00	1	1	0	0	0	0.007	0	t	140672	EQIYRVDLRTLR	Rattus norvegicus
1	1.00	1	1	0	0	0	0.007	0	t	140681	GPEYWEQQTRIAKEWEQ	Rattus norvegicus
1	1.00	1	1	0	0	0	0.011	0	t	103892	ERYWLRS	Homo sapiens

1	1.00	1	1	0.02	0.022	0.009	0.021	0.02	t	110571	EKTGILTVTYHSETQRTK	Homo sapiens
1	1.00	1	1	0.02	0.017	0.017	0.02	0.02	t	62830	TAEGRAIN	Mus musculus
1	1.00	1	1	0	0	0	0.007	0	t	18441	FYNQKFKGKATL	Mus musculus
1	1.00	1	1	0.02	0.018	0.022	0.017	0.019	t	73312	YAADITNGL	Mus musculus
1	1.00	1	1	0.02	0.02	0.025	0.02	0.016	t	25139	HWSISKPQ	Mus musculus
1	1.00	1	1	-0.01	-0.008	-0.008	-0.009	-0.01	t	20082	GGVYLLPR	Homo sapiens
1	1.00	1	1	-0.02	-0.02	-0.027	-0.02	-0.022	t	95294	CSNGGLDLDV	Homo sapiens
1	1.00	1	1	0	0	0	0.004	0	t	132098	TLLLEGVMAA	Homo sapiens
1	1.00	1	1	-0.03	-0.028	-0.03	-0.029	-0.03	t	55382	RPYCWHYPP	Homo sapiens
1	1.00	1	1	0.01	0.014	0.004	0.017	0.012	t	55838	RSFQNKKSLVAFKIMPLEDM	Homo sapiens
1	1.00	1	1	-0.01	-0.01	-0.017	-0.01	-0.009	t	130806	EMDVVRFDD	Homo sapiens
1	1.00	1	1	0.08	0.08	0.08	0.08	0.081	t	117235	SS	Mus musculus
1	1.00	1	1	0.01	0.008	0.01	0.013	0.01	t	51925	QPQQPFPQSQ	Mus musculus
1	1.00	1	1	0	0	-0.005	0.001	0	t	50168	PYSASFT	Mus musculus
1	1.00	1	1	0.01	0.007	0.003	0.01	0.011	t	114740	AQGHRPQDE	Oryctolagus cuniculus

1	1.00	1	1	0.01	0.007	0.003	0.01	0.011	t	114740	AQGHRPQDE	Oryctolagus cuniculus
1	1.00	1	1	0.01	-0.002	0.003	0.01	0.011	t	114740	AQGHRPQDE	Macaca mulatta
1	1.00	1	1	0.01	0.013	0.01	0.01	0.01	t	21601	GPEET	Mus musculus
1	1.00	1	1	0.01	0.01	0.01	0.01	0.01	t	21601	GPEET	Mus musculus
1	1.00	1	1	0.01	0.013	0.01	0.01	0.01	t	21601	GPEET	Mus musculus
1	1.00	1	1	0	-0.003	-0.01	0.001	0	t	38369	LNVSREVLQESRILRVIKKK	Canis lupus
1	1.00	1	1	-0.02	-0.02	-0.02	-0.013	-0.023	t	119455	GGQFNFDEGTP	Homo sapiens
1	1.00	1	1	-0.03	-0.03	-0.037	-0.03	-0.03	t	119475	KKIK	Homo sapiens
1	1.00	1	1	0.02	0.018	0.02	0.014	0.019	t	119888	GARVYAYVFE	Oryctolagus cuniculus
1	1.00	1	1	0	0	0	0	0	t	4486	ASINKFVV	Mus musculus
1	1.00	1	1	0	0	0	0	0	t	25254	IAEHQFKEK	Homo sapiens
1	1.00	1	1	-0.02	-0.023	-0.014	-0.02	-0.022	t	139062	GIWPEKIC	Mus musculus
1	1.00	1	1	0.01	0.006	0.012	0.011	0.01	t	43110	NAAFS	Bos taurus
1	1.00	1	1	-0.02	-0.018	-0.026	-0.019	-0.022	t	141580	YIGVAPRKAIPA	Homo sapiens
1	1.00	1	1	0.01	0.01	0.003	0.014	0.009	t	139494	ERGSAGHWTSESSVS	Homo sapiens

1	1.00	1	1	0	0	0	0	0	t	131852	DVDEPLEV FISA	Homo sapiens
1	1.00	1	1	0.03	0.03	0.03	0.03	0.031	t	115308	KFLDDDLT DD	Homo sapiens
1	1.00	1	1	0.01	0.01	0.01	0.01	0.011	t	115201	DDIMCVK KIL	Homo sapiens
1	1.00	1	1	0	-0.002	0	0.003	-0.002	t	60722	SRLGRSSA WV	Mus musculu s
1	1.00	1	1	-0.02	-0.016	-0.02	-0.013	-0.02	t	123579	PGMIPPP G	Homo sapiens
1	1.00	1	1	-0.04	-0.04	-0.041	-0.044	-0.041	t	118947	PFSAPPPA	Homo sapiens
1	1.00	1	1	0.05	0.05	0.05	0.05	0.05	t	106598	LEAYAFDI AA	Homo sapiens
1	1.00	1	1	0.03	0.028	0.024	0.027	0.03	t	54302	RITYLTETKI	Mus musculu s
1	1.00	1	1	-0.03	-0.028	-0.03	-0.023	-0.032	t	137399	FCGLPRLE	Homo sapiens
1	1.00	1	1	0	0.004	0	0.001	-0.003	t	36959	LKMADPN RFRGKD	MD
1	1.00	1	1	-0.02	-0.021	-0.035	-0.02	-0.02	t	13897	EQPFPSRL GPLVTLE	Bos taurus
1	1.00	1	1	0	-0.02	-0.003	0.001	-0.002	t	111106	AKIQLPTL NEDLT	Cricetina e
1	1.00	1	1	0.02	0.018	0.016	0.012	0.016	t	144324	GVSTITAD EIR	Mus musculu s
1	1.00	1	1	0	0.002	0.009	0.006	-0.001	t	65050	TLRVYK	Homo sapiens
1	1.00	1	1	0	0.004	-0.001	0.001	0.002	t	71545	VTV	Homo sapiens

1	1.00	1	1	0.01	0.01	-0.002	0.01	0.01	t	104520	IAKEQENKETIGTLKKILDE	Rattus norvegicus
1	1.00	1	1	0.06	0.058	0.072	0.063	0.06	t	138763	DWEYSEGL	Mus musculus
1	1.00	1	1	0.02	0.022	0.02	0.023	0.02	t	156603	AEVLRDGDCP	Homo sapiens
1	1.00	1	1	0	0	0	0	0	t	146337	IPEEIKQL	Mus musculus
1	1.00	1	1	0.04	0.042	0.033	0.038	0.038	t	106501	HCGIVDSKLP	Homo sapiens
1	1.00	1	1	0	-0.004	0.007	0	-0.002	t	10853	DYAWDQTHQ	Mus musculus
1	1.00	1	1	0.02	0.022	0.02	0.027	0.02	t	119617	NGQPENNY	Homo sapiens
1	1.00	1	1	0.03	0.03	0.028	0.03	0.03	t	56700	SAAGLEVRYV	Homo sapiens
1	1.00	1	1	0.01	0.013	0	0.01	0.011	t	93373	MDKVLNREESL	Mus musculus
1	1.00	1	1	0.02	0.02	0.005	0.017	0.021	t	100	AAEAACFK	Homo sapiens
1	1.00	1	1	0.01	0.014	0.01	0.017	0.012	t	130407	YSSIAWL	MD
1	1.00	1	1	0	0	-0.016	0.002	0.002	t	98686	AHGRSQVLQQST	Homo sapiens
1	1.00	1	1	0.05	0.046	0.048	0.053	0.051	t	99274	GGFSEV	Oryctolagus cuniculus
1	1.00	1	1	-0.02	-0.027	-0.022	-0.023	-0.02	t	46722	NYVLMVPK	Homo sapiens
1	1.00	1	1	-0.01	-0.01	-0.01	-0.008	-0.01	t	16443	FKQKALGLLQTATK	Homo sapiens

1	1.00	1	1	0.05	0.052	0.042	0.051	0.05	t	7383	CYGVSATK LNDLCFSN V	Homo sapiens
1	1.00	1	1	0.01	0.01	0.01	0.01	0.01	t	77781	GLVGRPKS KL	Homo sapiens
1	1.00	1	1	0.05	0.052	0.051	0.051	0.049	t	56386	RVQYEG	Homo sapiens
1	1.00	1	1	0.05	0.05	0.05	0.05	0.049	t	134421	KQIPECEN VKTTQCVF	Mus musculu s
1	1.00	1	1	-0.02	-0.018	-0.027	-0.019	-0.021	t	137837	EGAVALA DRASS	Homo sapiens
1	1.00	1	1	0.02	0.02	0.02	0.024	0.02	t	135956	LRQLTPEQ LSTL	Homo sapiens
1	1.00	1	1	0.01	0.01	-0.007	0.01	0.011	t	49325	PSDKHIEQ YLKKIQNSL STE	Homo sapiens
1	1.00	1	1	0.01	0.01	0.006	0.01	0.008	t	51922	QPQQPFP QPQ	Mus musculu s
1	1.00	1	1	0.05	0.038	0.045	0.051	0.05	t	50156	PYQGSGK GVS	Mus musculu s
1	1.00	1	1	0.02	0.02	0.02	0.02	0.02	t	140461	EVSLHDM NLATH	Mus musculu s
1	1.00	1	1	-0.03	-0.032	-0.042	-0.027	-0.028	t	95920	TNVARP	Mus musculu s
1	1.00	1	1	-0.04	-0.037	-0.039	-0.037	-0.04	t	78220	NKTWYL	Equus caballus
1	1.00	1	1	0.02	0.024	0.017	0.021	0.019	t	103457	PAEFLENF VRSSNLK	Homo sapiens
1	1.00	1	1	0.05	0.046	0.038	0.049	0.051	t	17629	FRPSQQN	Oryctola gus cuniculu s
1	1.00	1	1	0	-0.003	0	0	-0.003	t	110385	QLYKTCKQ AGTCPPDII PKV	Oryctola gus cuniculu s

1	1.00	1	1	0	-0.003	0	0	-0.003	t	116677	ASATQLYKTCKQAGTCPPD	Oryctolagus cuniculus
1	1.00	1	1	0	0.008	0	0	-0.003	t	70390	VPNLRGDLQVLAQKVART	Cavia porcellus
1	1.00	1	1	0	-0.003	0	0	-0.003	t	95040	SHRPREAK	Oryctolagus cuniculus
1	1.00	1	1	0	-0.004	0	0	-0.003	t	70391	VPNLRGDLQVLAQKVARTLP	Mus musculus
1	1.00	1	1	0	-0.017	0	0	-0.003	t	75571	YQWLTRVGKYRPQDKPNT	Oryctolagus cuniculus
1	1.00	1	1	0	-0.003	0	0	-0.003	t	95032	PAGTGDRL	Oryctolagus cuniculus
1	1.00	1	1	0	-0.003	0	0	-0.003	t	64396	TINKPKGYVGKE	Oryctolagus cuniculus
1	1.00	1	1	0	-0.004	0	0	-0.003	t	70391	VPNLRGDLQVLAQKVARTLP	Mus musculus
1	1.00	1	1	0	0.004	0	0	-0.003	t	91165	CCELCCNPACAGCY	Sus scrofa
1	1.00	1	1	0	0.003	0	0	-0.003	t	63038	TAVSPTTLRTEVVK	Sus scrofa
1	1.00	1	1	0	-0.004	0	0	-0.003	t	70391	VPNLRGDLQVLAQKVARTLP	Mus musculus
1	1.00	1	1	0	0.003	0	0	-0.003	t	63038	TAVSPTTLRTEVVK	Sus scrofa

1	1.00	1	1	0	0.008	0	0	-0.003	t	70390	VPNLRGDLQVLAQKVART	Cavia porcellus
1	1.00	1	1	0	-0.003	0	0	-0.003	t	59922	SNTFINNA	Oryctolagus cuniculus
1	1.00	1	1	0	0.008	0	0	-0.003	t	54058	RHKQKIVAPVKQTL	Cavia porcellus
1	1.00	1	1	0	-0.007	0	0	-0.003	t	54058	RHKQKIVAPVKQTL	Oryctolagus cuniculus
1	1.00	1	1	0	0.001	0	0	-0.003	t	46094	NTFYCCELCCNPACAGCY	Oryctolagus cuniculus
1	1.00	1	1	0	0.001	0	0	-0.003	t	46094	NTFYCCELCCNPACAGCY	Oryctolagus cuniculus
1	1.00	1	1	0	-0.003	0	0	-0.003	t	58783	SKAFSNCYPYDVPDYASL	Oryctolagus cuniculus
1	1.00	1	1	0	0.008	0	0	-0.003	t	54058	RHKQKIVAPVKQTL	Cavia porcellus
1	1.00	1	1	0	-0.003	0	0	-0.003	t	49006	PQGQGKVT	Oryctolagus cuniculus
1	1.00	1	1	0	-0.017	0	0	-0.003	t	55818	RSDYKFYEDANGTRDHKKG	Oryctolagus cuniculus
1	1.00	1	1	0	-0.007	0	0	-0.003	t	54058	RHKQKIVAPVKQTL	Oryctolagus cuniculus

1	1.00	1	1	0	-0.004	0	0	-0.003	t	22360	GSGVRGDFGSLAPRVARQL	Mus musculus
1	1.00	1	1	0	-0.003	0	0	-0.003	t	9516	DNNLVSGP	Oryctolagus cuniculus
1	1.00	1	1	0	-0.003	0	0	-0.003	t	1330	AFKGGNGAKKG	Oryctolagus cuniculus
1	1.00	1	1	0	0	0	0	0	t	45407	NPNANPNA	Mus musculus
1	1.00	1	1	0	0.002	-0.001	0.001	0	t	19893	GGIVTC	Homo sapiens
1	1.00	1	1	0	0	0	0.009	0	t	131874	EVFISAPR	Homo sapiens
1	1.00	1	1	-0.02	-0.017	-0.027	-0.02	-0.022	t	33795	KTNMKHMAGA	Mus musculus
1	1.00	1	1	0	-0.003	0.006	0	-0.002	t	139191	NYTCCKLQ	Mus musculus
1	1.00	1	1	0.04	0.042	0.044	0.041	0.039	t	45659	NRDFVE	Homo sapiens
1	1.00	1	1	0	0	0	0	0	t	65555	TPEMDIPGQV	Mus musculus
1	1.00	1	1	0	0	0	0	0	t	65555	TPEMDIPGQV	Mus musculus
1	1.00	1	1	0.04	0.044	0.04	0.043	0.04	t	118567	QIEKRIQEIIEQL	Homo sapiens
1	1.00	1	1	0.01	0.013	0.01	0.01	0.008	t	140892	QILDAHAAYLNATPAAKVLV	Mus musculus
1	1.00	1	1	-0.01	-0.012	-0.02	-0.011	-0.01	t	34435	KYGGTEIKYN	Mus musculus

1	1.00	1	1	0.01	0.012	-0.002	0.007	0.009	t	257	AAKVLVEGNTDERGT	Homo sapiens
1	1.00	1	1	0.01	0.012	0.006	0.007	0.008	t	114920	PVCGTDGVTY	Homo sapiens
1	1.00	1	1	0.01	0.007	-0.002	0.01	0.011	t	36543	LIGVKGSS	Oryctolagus cuniculus
1	1.00	1	1	-0.01	-0.01	-0.015	-0.01	-0.011	t	125055	FVYGGCQGNN	Mus musculus
1	1.00	1	1	-0.03	-0.03	-0.032	-0.027	-0.03	t	7055	CSPHHTALRQ	Homo sapiens
1	1.00	1	1	0.01	0.01	0.01	0.01	0.01	t	60449	SQHLPYIEQ	Homo sapiens
1	1.00	1	1	0.01	0.01	0.01	0.01	0.01	t	60449	SQHLPYIEQ	Homo sapiens
1	1.00	1	1	0.06	0.062	0.053	0.059	0.06	t	135805	YVNSTEAE	Homo sapiens
1	1.00	1	1	0.06	0.062	0.053	0.059	0.06	t	135805	YVNSTEAE	Homo sapiens
1	1.00	1	1	0.02	0.02	0.02	0.02	0.018	t	50505	QDPR	Mus musculus
1	1.00	1	1	0.02	0.02	0.025	0.024	0.021	t	122194	STVQDCV	Homo sapiens
1	1.00	1	1	0.05	0.05	0.045	0.054	0.05	t	76307	YVKELPTEVQKLKEK	Homo sapiens
1	1.00	1	1	-0.02	-0.018	-0.02	-0.017	-0.021	t	7046	CSNKSKR	Homo sapiens
1	1.00	1	1	-0.01	-0.018	-0.018	-0.01	-0.011	t	32138	KLRPFERDI	Mus musculus
1	1.00	1	1	-0.01	-0.006	-0.017	-0.007	-0.008	t	144816	WSHNFTNMLG	Homo sapiens
1	1.00	1	1	0.03	0.03	0.03	0.038	0.03	t	110909	IVTFCCKCDSTLRLCVQST	Mus musculus

1	1.00	1	1	-0.01	-0.01	-0.01	-0.01	-0.01	t	26619	IIPDREVLY	Homo sapiens
1	1.00	1	1	-0.01	-0.014	-0.017	-0.011	-0.01	t	46859	PALTAVETGATNPL	Oryctolagus cuniculus
1	1.00	1	1	0.03	0.032	0.023	0.027	0.028	t	58037	SGFTLEFLEHAFSVD	Homo sapiens
1	1.00	1	1	0.01	0.012	0.011	0.011	0.01	t	66539	TTASGKLIT	Homo sapiens
1	1.00	1	1	-0.03	-0.03	-0.03	-0.023	-0.028	t	118061	PPPGMRPPMG	Mus musculus
1	1.00	1	1	-0.02	-0.018	-0.026	-0.02	-0.02	t	122843	LNAGNQLS	Rattus norvegicus
1	1.00	1	1	-0.02	-0.02	-0.023	-0.023	-0.023	t	115630	NFDKLYIWG	Homo sapiens
1	1.00	1	1	0	-0.004	-0.011	-0.007	-0.002	t	98434	RYMDQPSRD	Oryctolagus cuniculus
1	1.00	1	1	-0.03	-0.024	-0.036	-0.03	-0.03	t	123438	LKMWSNFLG	Ovis aries
1	1.00	1	1	0.04	0.037	0.033	0.04	0.04	t	79158	ASPYGNA	Oryctolagus cuniculus
1	1.00	1	1	-0.02	-0.02	-0.022	-0.02	-0.02	t	118548	PAPGMRPP	Homo sapiens
1	1.00	1	1	-0.02	-0.018	-0.022	-0.02	-0.02	t	118548	PAPGMRPP	Homo sapiens
1	1.00	1	1	0.03	0.03	0.026	0.03	0.029	t	14841	EVPGSQ	Oryctolagus cuniculus
1	1.00	1	1	0.03	0.03	0.022	0.03	0.03	t	26375	IHFGSDYEDRYY	Mus musculus

1	1.00	1	1	0.03	0.03	0.022	0.03	0.03	t	26375	IHFGSDYE DRYY	Mus musculu s
1	1.00	1	1	0	0	0	0	-0.003	t	135939	IPVLDENG LFAP	Mus musculu s
1	1.00	1	1	0	0	0	0	-0.003	t	139846	PEWWLEE PPFISSGK	Mus musculu s
1	1.00	1	1	0	0	0	0	-0.003	t	141683	KDWG	Mus musculu s
1	1.00	1	1	0	0	0	0	-0.003	t	141693	KTWG	Mus musculu s
1	1.00	1	1	0	0	0	0	-0.003	t	139841	PAGRQAR RPATVPEG	Mus musculu s
1	1.00	1	1	0	0	0	0	-0.003	t	95051	YSQFEKSY	Oryctola gus cuniculu s
1	1.00	1	1	0	0	0	0	-0.003	t	95032	PAGTGDRL	Oryctola gus cuniculu s
1	1.00	1	1	0	0	0	0	-0.003	t	30262	KDTNNN	Mus musculu s
1	1.00	1	1	0	0	0	0	-0.003	t	7674	DAVTELDK N	Mus musculu s
1	1.00	1	1	0	0	0	0	-0.003	t	4429	ASGQ	Mus musculu s
1	1.00	1	1	0	0	0	0	-0.003	t	7673	DAVTE	Mus musculu s
1	1.00	1	1	-0.02	-0.02	-0.02	-0.014	-0.02	t	158670	ADTFNFKT FFHTIGF	Homo sapiens
1	1.00	1	1	0	0.003	0	0	-0.002	t	49270	PRVRGLY	Mus musculu s

1	1.00	1	1	0	0	-0.011	0	0	t	774	ADSVGGKD	Mus musculus
1	1.00	1	1	-0.02	-0.017	-0.015	-0.02	-0.024	t	138227	QKRAA	Mus musculus
1	1.00	1	1	-0.05	-0.048	-0.05	-0.043	-0.05	t	118361	FRFLALKLYFSFTRP	Homo sapiens
1	1.00	1	1	-0.02	-0.02	-0.02	-0.02	-0.02	t	118779	EEAKKFLAPK	Mus musculus
1	1.00	1	1	-0.01	-0.012	-0.017	-0.007	-0.01	t	108323	DTPPPCPRCP	Mus musculus
1	1.00	1	1	0.03	0.028	0.022	0.03	0.03	t	48396	PLLGNQSL	Anas platyrhynchos
1	1.00	1	1	0.03	0.03	0.025	0.03	0.03	t	72466	WGENDTDVFVLNNTR	Mus musculus
1	1.00	1	1	0.02	0.02	0.018	0.02	0.02	t	122799	IKYIAEQLES	Rattus norvegicus
1	1.00	1	1	0.04	0.04	0.032	0.04	0.04	t	141748	SLLTEVETP	Mus musculus
1	1.00	1	1	0.02	0.02	0.005	0.012	0.021	t	98783	FQQPLQQYPLGQ	Homo sapiens
1	1.00	1	1	0.03	0.032	0.035	0.033	0.028	t	121347	GPLTVAQKKAQNIME	Homo sapiens
1	1.00	1	1	0.03	0.032	0.035	0.033	0.028	t	121347	GPLTVAQKKAQNIME	Homo sapiens
1	1.00	1	1	0.03	0.03	0.021	0.026	0.03	t	20064	GGVATY	Homo sapiens
1	1.00	1	1	-0.01	-0.01	-0.01	-0.01	-0.01	t	47119	PDKEILYEA	Homo sapiens
1	1.00	1	1	0.01	0.012	0.008	0.007	0.007	t	105977	NHEQEFLRYQQQSRR	Homo sapiens

1	1.00	1	1	-0.01	-0.014	-0.017	-0.011	-0.01	t	52694	QVEVPG	Oryctolagus cuniculus
1	1.00	1	1	-0.01	-0.008	-0.011	-0.009	-0.01	t	40213	LTTTAATRFM	Homo sapiens
1	1.00	1	1	-0.02	-0.02	-0.028	-0.02	-0.02	t	14844	EVPLGVAADANK	Oryctolagus cuniculus
1	1.00	1	1	0.01	0.01	-0.002	0.01	0.01	t	120396	AGYIPFDQDL	Mus musculus
1	1.00	1	1	-0.01	-0.01	-0.01	-0.01	-0.013	t	140458	EHNDFPMYTWRP	Mus musculus
1	1.00	1	1	0.02	0.02	0.024	0.019	0.02	t	32418	KMVYATN	Homo sapiens
1	1.00	1	1	0.01	0.01	0.022	0.02	0.012	t	138553	PVPHGTQ	Homo sapiens
1	1.00	1	1	-0.02	-0.02	-0.02	-0.02	-0.023	t	106976	VPTQLYNLYPPL	Mus musculus
1	1.00	1	1	0.02	0.018	0.022	0.019	0.02	t	73312	YAADITNGL	Mus musculus
1	1.00	1	1	0.01	0.006	0.002	0.009	0.01	t	51463	QLPFPQQPQQ	Mus musculus
1	1.00	1	1	0.01	0.01	-0.003	0.01	0.01	t	159172	TTDSGKLVTDWCCRSC	Mus musculus
1	1.00	1	1	-0.02	-0.022	-0.025	-0.021	-0.021	t	56699	SAAFYYRV	Mus musculus
1	1.00	1	1	0.02	0.02	0.013	0.026	0.02	t	57632	SESTEGDAMEDIKQMEAESI	Homo sapiens
1	1.00	1	1	0.03	0.03	0.022	0.03	0.03	t	62967	TAQAAPVQEG	Mus musculus

1	1.00	1	1	-0.06	-0.058	-0.07	-0.059	-0.062	t	103143	EMIEAVGY RSWPRYF	Homo sapiens
1	1.00	1	1	0	0	-0.009	0	0.002	t	94553	IFD	Mus musculu s
1	1.00	1	1	-0.03	-0.026	-0.029	-0.023	-0.03	t	113211	VPVLAFDA ARLRLLE	Homo sapiens
1	1.00	1	1	-0.01	-0.008	-0.013	-0.009	-0.01	t	67168	TVSSQSFR	Homo sapiens
1	1.00	1	1	-0.01	-0.008	-0.017	-0.007	-0.011	t	135935	IEQGWGS PKVTK	Homo sapiens
1	1.00	1	1	0.06	0.058	0.055	0.057	0.06	t	12507	EILLNQLA	Anas platyrhy nchos
1	1.00	1	1	-0.01	-0.006	-0.022	-0.011	-0.009	t	141560	SDEALPPG SPR	Mus musculu s
1	1.00	1	1	0	0	-0.005	0	0.001	t	18363	FWRGDLV FDFQV	Homo sapiens
1	1.00	1	1	-0.02	-0.02	-0.018	-0.02	-0.02	t	139500	FHTHISV	Homo sapiens
1	1.00	1	1	-0.01	-0.008	-0.016	-0.007	-0.009	t	144823	YWEGSGI GAI	Homo sapiens
1	1.00	1	1	-0.01	-0.007	-0.018	-0.01	-0.01	t	45156	NNKTPNSI AA	Mus musculu s
1	1.00	1	1	0.02	0.02	0.02	0.019	0.02	t	19268	GEGAPP	Marmot a monax
1	1.00	1	1	-0.01	-0.01	-0.008	-0.007	-0.012	t	13728	EPPVVHG C	Homo sapiens
1	1.00	1	1	0.03	0.028	0.025	0.029	0.03	t	4448	ASHLEQYG TNEYS	Mus musculu s
1	1.00	1	1	-0.03	-0.031	-0.04	-0.03	-0.03	t	53449	REAFRRYQ	Anas platyrhy nchos

1	1.00	1	1	0	0.004	0	0	0.002	t	139840	PAAFAFL	Ovis aries
1	1.00	1	1	0.02	0.023	0.023	0.02	0.019	t	125127	LDPENLTL	Mus musculus
1	1.00	1	1	-0.03	-0.03	-0.031	-0.03	-0.03	t	122715	AKHDLDIKPT	Rattus norvegicus
1	1.00	1	1	0.01	0.013	0.01	0.01	0.01	t	134702	KEKKYS	Mus musculus
1	1.00	1	1	0.01	0.01	0.008	0.01	0.009	t	97282	ESNNL	Oryctolagus cuniculus
1	1.00	1	1	-0.05	-0.048	-0.053	-0.053	-0.052	t	111135	AWLAQFVLP	Homo sapiens
1	1.00	1	1	0.05	0.05	0.05	0.05	0.049	t	119519	RKVVQTSLE	Homo sapiens
1	1.00	1	1	0.05	0.05	0.038	0.05	0.051	t	17629	FRPSQQN	Oryctolagus cuniculus
1	1.00	1	1	0.02	0.024	0.018	0.023	0.019	t	119855	FAESRIRRETIAAED	Homo sapiens
1	1.00	1	1	0.02	0.022	0.018	0.023	0.019	t	119855	FAESRIRRETIAAED	Homo sapiens
1	1.00	1	1	0	0	0	0	0.001	t	115335	KYLLFCMENS	Homo sapiens
1	1.00	1	1	0.02	0.018	0.015	0.023	0.02	t	55048	RNQE	Mus musculus
1	1.00	1	1	0.02	0.016	0.007	0.019	0.02	t	67445	TYRATYQDFD	Oryctolagus cuniculus
1	1.00	1	1	0.01	0.01	0.01	0.01	0.008	t	78479	PETLPYL	Mus musculus

1	1.00	1	1	0	-0.002	0	0.007	0	t	110817	DSTLRLCVQSTHVDIRTLE	Mus musculus
1	1.00	1	1	0	-0.002	0	0.007	0	t	110869	GQAEPDRAHYNIVTFCCKCD	Mus musculus
1	1.00	1	1	0.01	0.006	0.001	0.009	0.01	t	47315	PEKAFREL	Oryctolagus cuniculus
1	1.00	1	1	0.01	0.014	0.01	0.007	0.008	t	106330	DFDCSKIQST	Homo sapiens
1	1.00	1	1	0.01	0.01	0.01	0.01	0.009	t	5562	AVTKGTINDPEA	Oryctolagus cuniculus
1	1.00	1	1	0.01	0.01	0.004	0.01	0.01	t	42951	MVIITFKSGA	Mus musculus
1	1.00	1	1	-0.01	-0.008	-0.019	-0.007	-0.01	t	18053	FTQCSMNVVSKTLFRKSPIY	Homo sapiens
1	1.00	1	1	0.01	0.006	0.005	0.011	0.008	t	133775	AETNRAPFDLAEAE	Oryctolagus cuniculus
1	1.00	1	1	0	-0.002	0.006	-0.001	-0.002	t	14009	ERKIKV	Mus musculus
1	1.00	1	1	0.04	0.038	0.028	0.039	0.04	t	66183	TSEEFQVKETSSGT	Mus musculus
1	1.00	1	1	-0.02	-0.02	-0.036	-0.018	-0.017	t	99014	SQCQAIHNVVHA	Homo sapiens
1	1.00	1	1	0.02	0.02	0.011	0.02	0.02	t	12258	EGRFKSQD	Homo sapiens
1	1.00	1	1	0.04	0.04	0.039	0.051	0.043	t	132014	PQIVPEPM	Homo sapiens
1	1.00	1	1	0.02	0.027	0.016	0.023	0.02	t	7996	DEGESPTPAT	Capra hircus

1	1.00	1	1	-0.02	-0.023	-0.028	-0.02	-0.022	t	6953	CRTNYGYS	Oryctolagus cuniculus
1	1.00	1	1	0.03	0.03	0.03	0.034	0.03	t	115605	GDEVELPCRISP	Homo sapiens
1	1.00	1	1	0.04	0.036	0.033	0.043	0.04	t	107254	TQSPGTLSLSPGERAT	Oryctolagus cuniculus
1	1.00	1	1	-0.04	-0.038	-0.047	-0.043	-0.039	t	51431	QLLRLKKYKV	Homo sapiens
1	1.00	1	1	-0.01	-0.01	-0.017	-0.014	-0.013	t	131038	RKIAKDEGAK	Homo sapiens
1	1.00	1	1	-0.01	-0.013	-0.014	-0.01	-0.01	t	12625	EKAFREL	Oryctolagus cuniculus
1	1.00	1	1	-0.01	-0.013	-0.014	-0.01	-0.01	t	12625	EKAFREL	Oryctolagus cuniculus
1	1.00	1	1	0.02	0.017	0.02	0.02	0.02	t	119923	GYISKAQEYFN	Oryctolagus cuniculus
1	1.00	1	1	-0.04	-0.04	-0.038	-0.04	-0.04	t	119411	YKYFPEVKTKGMA	Homo sapiens
1	1.00	1	1	-0.01	-0.014	-0.012	-0.011	-0.012	t	120889	QISYSCNVLIYPS	Oryctolagus cuniculus
1	1.00	1	1	0.03	0.026	0.023	0.031	0.033	t	131117	TRDL	Oryctolagus cuniculus
1	1.00	1	1	-0.01	-0.012	-0.007	-0.011	-0.014	t	46488	NVSVPSPSSTPLLY	Mus musculus
1	1.00	1	1	0	0	-0.002	0	-0.002	t	78028	TCRNNDTCRAPD	Mus musculus
1	1.00	1	1	0	0.002	0	0.003	-0.003	t	134088	IHGIKTQGARQKFSS	Homo sapiens

1	1.00	1	1	-0.02	-0.02	-0.02	-0.02	-0.022	t	117565	LKAVKMTLKNREP	Homo sapiens
1	1.00	1	1	0.02	0.02	0.02	0.02	0.021	t	115234	EQLTKCEVFR	Homo sapiens
1	1.00	1	1	0	0	-0.015	0	0.001	t	122048	YCEIIV	Oryctolagus cuniculus
1	1.00	1	1	-0.01	-0.013	-0.018	-0.01	-0.01	t	12417	EIDKGIYQTSNFRVVPS	Oryctolagus cuniculus
1	1.00	1	1	0.01	0.006	0.015	0.009	0.008	t	53894	RGKDLPVLDQLTD	Oryctolagus cuniculus
1	1.00	1	1	0.04	0.04	0.04	0.04	0.04	t	117464	DVEPKVKSKKREAVA	Homo sapiens
1	1.00	1	1	0.04	0.04	0.04	0.04	0.04	t	117465	DVEPKVKSKKREAVA	Homo sapiens
1	1.00	1	1	-0.02	-0.017	-0.031	-0.02	-0.018	t	111382	GTLGIVCPICSQKP	Mus musculus
1	1.00	1	1	0.04	0.042	0.031	0.037	0.041	t	64998	TLPDTEKQI	Homo sapiens
1	1.00	1	1	-0.02	-0.018	-0.028	-0.017	-0.02	t	27244	ILPGGGTALIRC	Homo sapiens
1	1.00	1	1	0	0.003	-0.006	0	0.002	t	133725	TVCTYRDFMY	Mus musculus
1	1.00	1	1	0.01	0.008	0.001	0.013	0.01	t	125575	THDALKAVKQLIKT	Mus musculus
1	1.00	1	1	0.02	0.022	0.022	0.027	0.02	t	119560	DLVLYNNADGDFAIVK	Homo sapiens
1	1.00	1	1	0.02	0.024	0.022	0.027	0.02	t	119560	DLVLYNNADGDFAIVK	Homo sapiens

1	1.00	1	1	-0.03	-0.03	-0.037	-0.023	-0.03	t	116927	AVCRTSM CSIQSAPP	Homo sapiens
1	1.00	1	1	0.02	0.02	0.02	0.023	0.02	t	23174	GVTRPS	Homo sapiens
1	1.00	1	1	-0.01	-0.007	-0.019	-0.01	-0.01	t	63756	TFTNKLKE KHTDLGK	Mus musculu s
1	1.00	1	1	-0.01	-0.01	-0.015	-0.011	-0.011	t	31075	KGTMDSG QTKR	Mus musculu s
1	1.00	1	1	0.04	0.04	0.037	0.04	0.04	t	61996	STVSSEAN AEDVVC	Homo sapiens
1	1.00	1	1	-0.06	-0.058	-0.043	-0.059	-0.07	t	948	AEGGTWR I	Homo sapiens
1	1.00	1	1	0.05	0.048	0.05	0.047	0.047	t	135854	IQQSIERL	Mus musculu s
1	1.00	1	1	0.02	0.024	0.02	0.027	0.02	t	123511	MPPMTQ AP	Homo sapiens
1	1.00	1	1	0	0	-0.012	0	0	t	30799	KFNPDLAP G	Homo sapiens
1	1.00	1	1	0.04	0.04	0.033	0.04	0.04	t	123278	GYLKDLPT T	Ovis aries
1	1.00	1	1	0.03	0.028	0.025	0.029	0.03	t	4448	ASHLEQYG TNEYS	Mus musculu s
1	1.00	1	1	0	-0.004	-0.001	-0.001	0	t	78836	SPKRIGTG DR	Oryctola gus cuniculu s
1	1.00	1	1	0	0.002	0	0.003	0.001	t	52420	QSRGEAR	Homo sapiens
1	1.00	1	1	0.05	0.046	0.047	0.05	0.05	t	141004	EPDP	Mus musculu s
1	1.00	1	1	-0.06	-0.058	-0.07	-0.059	-0.061	t	96133	AYGIKSNV VR	Homo sapiens(human)

1	1.00	1	1	-0.01	-0.008	-0.01	-0.003	-0.009	t	107479	KFNWYVD	Homo sapiens
1	1.00	1	1	0.01	0.01	0.01	0.01	0.01	t	156886	HGLYGS	Gallus gallus
1	1.00	1	1	-0.02	-0.017	-0.018	-0.02	-0.021	t	120077	LMRYWANFAR	Mus musculus
1	1.00	1	1	-0.01	-0.01	-0.008	-0.01	-0.013	t	156975	WSSTVLRVSPTRTVP	Mus musculus
1	1.00	1	1	-0.02	-0.018	-0.025	-0.019	-0.02	t	21497	GNPNRPDGGILT	Homo sapiens
1	1.00	1	1	0	0.002	-0.009	-0.003	-0.002	t	60019	SPDRKRRADEEEEYD	Homo sapiens
1	1.00	1	1	-0.02	-0.02	-0.02	-0.02	-0.021	t	9132	DLIPLLR	Oryctolagus cuniculus
1	1.00	1	1	-0.05	-0.048	-0.05	-0.043	-0.05	t	107607	RVVSVLT	Homo sapiens
1	1.00	1	1	-0.02	-0.018	-0.033	-0.019	-0.02	t	137946	PGYYRLTLIANG	Homo sapiens
1	1.00	1	1	0.01	0.007	0.004	0.01	0.01	t	54908	RMKDTL	Oryctolagus cuniculus
1	1.00	1	1	-0.03	-0.028	-0.03	-0.037	-0.036	t	23176	GVTRPSAPPLPHVV	Homo sapiens
1	1.00	1	1	0.01	0.01	0	0.01	0.01	t	15523	FEEVNGIRKP	Mus musculus
1	1.00	1	1	0.01	0.01	0	0.01	0.01	t	15517	FEEINGIRKP	Mus musculus
1	1.00	1	1	0.05	0.05	0.05	0.05	0.05	t	141894	FEQEPSSEEPKDSKL	Mus musculus
1	1.00	1	1	0.05	0.05	0.05	0.05	0.05	t	141894	FEQEPSSEEPKDSKL	Mus musculus

1	1.00	1	1	0.01	0.014	0.001	0.011	0.011	t	13735	EPQVYTLPPSR	Homo sapiens
1	1.00	1	1	-0.01	-0.006	-0.01	-0.003	-0.011	t	117298	GPSRRGPSLGASSH	Homo sapiens
1	1.00	1	1	-0.01	-0.008	-0.01	-0.003	-0.011	t	117298	GPSRRGPSLGASSH	Homo sapiens
1	1.00	1	1	0.05	0.05	0.05	0.05	0.05	t	137888	GTEYTVEV	Homo sapiens
1	1.00	1	1	0	0	-0.007	0	-0.001	t	141523	ALEPPHNLPVP	Mus musculus
1	1.00	1	1	-0.01	-0.014	-0.016	-0.009	-0.01	t	22835	GTSMDDEADRYF	Bos taurus
1	1.00	1	1	0	0	-0.002	0	0	t	78379	FEPSKSISGVSHYLL	Mus musculus
1	1.00	1	1	0.03	0.032	0.021	0.033	0.033	t	110733	VGENVPDDLYIKGSG	Homo sapiens
1	1.00	1	1	0	-0.002	-0.017	-0.003	0	t	60098	SPKGGAISIKDS	Mus musculus
1	1.00	1	1	0	0	0.005	0.001	0	t	138238	SMLDTPT	Mus musculus
1	1.00	1	1	-0.01	-0.008	-0.01	-0.004	-0.011	t	138190	HNAKTKL	Homo sapiens
1	1.00	1	1	-0.02	-0.018	-0.02	-0.017	-0.02	t	51976	QPTLTFGAGVNVPAG	Homo sapiens
1	1.00	1	1	-0.02	-0.022	-0.02	-0.017	-0.02	t	51976	QPTLTFGAGVNVPAG	Homo sapiens
1	1.00	1	1	0	0	0	0.004	-0.001	t	135449	CATLRVYKGG	Homo sapiens
1	1.00	1	1	0.02	0.017	0.012	0.02	0.019	t	10326	DTEEDKPKYE	Oryctolagus cuniculus

1	1.00	1	1	0.03	0.035	0.035	0.034	0.03	t	720	ADLPQP	Homo sapiens
1	1.00	1	1	-0.01	-0.017	-0.017	-0.003	-0.008	t	156799	VYATRSSA VRLRSSV	Homo sapiens
1	1.00	1	1	0.03	0.028	0.025	0.029	0.03	t	4448	ASHLEQYG TNEYS	Mus musculu s
1	1.00	1	1	0.01	0.01	0.012	0.01	0.01	t	53924	RGLYFPAG	Mus musculu s
1	1.00	1	1	-0.01	-0.01	-0.01	-0.01	-0.01	t	47119	PDKEILYEA	Homo sapiens
1	1.00	1	1	0.01	0.015	0.003	0.011	0.01	t	14074	ERSDLEPSI PSEYM	Homo sapiens
1	1.00	1	1	-0.05	-0.05	-0.05	-0.05	-0.052	t	145831	IPLGTRP	Mus musculu s
1	1.00	1	1	0.05	0.05	0.045	0.05	0.05	t	51072	QIGNTVTP TVTFTMD GDTMT	Mus musculu s
1	1.00	1	1	0.05	0.05	0.045	0.05	0.05	t	51072	QIGNTVTP TVTFTMD GDTMT	Mus musculu s
1	1.00	1	1	0.05	0.05	0.045	0.05	0.05	t	51072	QIGNTVTP TVTFTMD GDTMT	Mus musculu s
1	1.00	1	1	0.02	0.02	0.019	0.02	0.02	t	32331	KMITEFEN	Homo sapiens
1	1.00	1	1	0.01	0.01	0.01	0.01	0.008	t	42984	MVQVVYD	Oryctola gus cuniculu s
1	1.00	1	1	0.01	0.01	0.001	0.01	0.01	t	135990	SASVPALT AVET	Homo sapiens
1	1.00	1	1	0.01	0.009	0.012	0.01	0.009	t	95521	KQTLPPSV	Bos taurus

1	1.00	1	1	0.04	0.04	0.04	0.04	0.04	t	153865	PETTDLYCY	Cavia porcellus
1	1.00	1	1	0.01	0.008	0.004	0.013	0.01	t	95676	PPRPTRPTTLPPTPLLT	Homo sapiens
1	1.00	1	1	-0.03	-0.026	-0.03	-0.023	-0.03	t	113001	GIRKFAADAVKLERM	Homo sapiens
1	1.00	1	1	0	0.008	-0.002	0	-0.003	t	146808	CTPKSDEFNPCEDIMGYKFL	Gallus gallus
1	1.00	1	1	-0.02	-0.02	-0.019	-0.02	-0.023	t	20523	GKCGVSIPYK	Homo sapiens
1	1.00	1	1	0	0	-0.008	0	0.002	t	133546	DSARAAY	Mus musculus
1	1.00	1	1	0.02	0.017	0.024	0.02	0.018	t	100074	GNINYACGQGID	Oryctolagus cuniculus
1	1.00	1	1	0	0.002	0	0.001	0	t	23689	HEHHGVYNQG	Homo sapiens
1	1.00	1	1	0.02	0.022	0.01	0.021	0.02	t	103641	TDVIPETDGAEKGPT	Homo sapiens
1	1.00	1	1	-0.01	-0.022	-0.022	-0.01	-0.01	t	115610	GSLPQKAQGHRPQDEN	Macaca mulatta
1	1.00	1	1	0.02	0.018	0.021	0.021	0.02	t	13266	ELRWDLTDIYNL	Mus musculus
1	1.00	1	1	-0.02	-0.02	-0.027	-0.02	-0.019	t	150972	CKLRGVAPLHLGKC	Mus musculus
1	1.00	1	1	0.05	0.05	0.05	0.05	0.05	t	111573	MSSQFYQ	Homo sapiens
1	1.00	1	1	0.01	0.01	0.011	0.007	0.01	t	55293	RPQDVKFP	Homo sapiens
1	1.00	1	1	0.01	0.008	0.012	0.009	0.009	t	122094	FLALDVPTIGLC	Mus musculus

1	1.00	1	1	0.03	0.028	0.019	0.029	0.03	t	54004	RGTYETGN KKVHGN	Mus musculu s
1	1.00	1	1	0.03	0.03	0.022	0.03	0.03	t	21675	GPKLSTDLI KNQCVNF NF	Mus musculu s
1	1.00	1	1	0.02	0.02	0.02	0.02	0.02	t	109756	QKSLSLS	Homo sapiens
1	1.00	1	1	0.01	0.014	0.01	0.013	0.01	t	117329	MAVPETR PNHT	Homo sapiens
1	1.00	1	1	-0.01	-0.012	-0.021	-0.007	-0.008	t	111745	RSSRTR	Mus musculu s
1	1.00	1	1	0	0.002	0	0.007	0	t	119410	YKMFPEVK EKGMA	Homo sapiens
1	1.00	1	1	0	0.004	0	0.007	0	t	119410	YKMFPEVK EKGMA	Homo sapiens
1	1.00	1	1	0.05	0.05	0.047	0.05	0.051	t	94606	LTGGFYEP L	Mus musculu s
1	1.00	1	1	-0.02	-0.02	-0.02	-0.017	-0.021	t	117300	GSGKGKV QFQGKKT KF	Homo sapiens
1	1.00	1	1	-0.01	-0.01	-0.01	-0.01	-0.012	t	117809	EALTKHFQ D	Homo sapiens
1	1.00	1	1	0	-0.006	-0.005	0	0	t	139764	GNYDRLE QLAGN	Oryctola gus cuniculu s
1	1.00	1	1	0.02	0.025	0.002	0.02	0.022	t	78835	SPDKLLTFI ASDTCPLV	Canis familiari s
1	1.00	1	1	0.01	0.01	0.007	0.01	0.01	t	13775	EPTPNAYY EGVFCSSS S	Mus musculu s
1	1.00	1	1	0.01	0.01	0.007	0.01	0.01	t	13775	EPTPNAYY EGVFCSSS S	Mus musculu s
1	1.00	1	1	0.01	0.01	0.007	0.01	0.01	t	13775	EPTPNAYY EGVFCSSS S	Mus musculu s

1	1.00	1	1	-0.03	-0.028	-0.037	-0.029	-0.03	t	55782	RRYPFVKFT	Homo sapiens
1	1.00	1	1	0.01	0.01	0.01	0.01	0.008	t	125869	EEEEE	Mus musculus
1	1.00	1	1	0.05	0.05	0.041	0.05	0.05	t	136006	VETGHTSQVTPS	Homo sapiens
1	1.00	1	1	0.02	0.02	0.02	0.031	0.022	t	118621	TGEVYRGKLIEAED	Homo sapiens
1	1.00	1	1	0	0	-0.008	0.003	0.002	t	72255	WAQPGYPW	Homo sapiens
1	1.00	1	1	-0.03	-0.03	-0.039	-0.03	-0.03	t	135923	FRIPVTINAPLQ	Homo sapiens
1	1.00	1	1	0.01	0.014	0.01	0.01	0.01	t	136436	KPVVGMPIVEVW	Gallus gallus
1	1.00	1	1	0.01	0.014	0.001	0.01	0.01	t	113539	IIPKVEGKTIAEQILQYGSM	Cavia porcellus
1	1.00	1	1	0.01	0.014	0.001	0.01	0.01	t	113539	IIPKVEGKTIAEQILQYGSM	Cavia porcellus
1	1.00	1	1	-0.03	-0.03	-0.03	-0.03	-0.03	t	136077	PPPTPPP	Oryctolagus cuniculus
1	1.00	1	1	-0.03	-0.028	-0.03	-0.023	-0.03	t	107534	NWYVDGV	Homo sapiens
1	1.00	1	1	0.02	0.022	0.013	0.021	0.02	t	34297	KWGVTTSLS	Homo sapiens
1	1.00	1	1	0.01	0.01	0.003	0.01	0.01	t	130585	QKKIRGILE	Mus musculus
1	1.00	1	1	0.02	0.02	0.02	0.02	0.02	t	73384	YALADASLKMAD	Oryctolagus cuniculus
1	1.00	1	1	0.02	0.02	0.02	0.02	0.02	t	73384	YALADASLKMAD	Oryctolagus cuniculus
1	1.00	1	1	0.01	0.008	0.005	0.009	0.01	t	9614	DPDKILTYIAA	Mus musculus

1	1.00	1	1	-0.04	-0.04	-0.042	-0.04	-0.04	t	73033	WRIGYFGH	Homo sapiens
1	1.00	1	1	-0.02	-0.022	-0.029	-0.017	-0.02	t	6983	CSETFTNKLKEKH	Mus musculus
1	1.00	1	1	0	0	0.002	0	-0.004	t	6479	CKGKIQALCENPEWA	Mus musculus
1	1.00	1	1	0.07	0.068	0.061	0.066	0.07	t	110884	HEYMLDLQPETTDLYCYE	Mus musculus
1	1.00	1	1	0.07	0.068	0.061	0.066	0.07	t	110884	HEYMLDLQPETTDLYCYE	Mus musculus
1	1.00	1	1	0.01	0.01	-0.001	0.011	0.01	t	134859	AGENLNTSKFKKNEF	Sus scrofa
1	1.00	1	1	-0.01	-0.01	-0.014	-0.01	-0.009	t	41529	MFNPV	Mus musculus
1	1.00	1	1	0	0.004	0.009	0.007	-0.001	t	119548	YRKKSSAEL	Homo sapiens
1	1.00	1	1	0	0.002	0.009	0.007	-0.001	t	119548	YRKKSSAEL	Homo sapiens
1	1.00	1	1	-0.02	-0.02	-0.02	-0.02	-0.02	t	39167	LRPTFDTRLM	Mus musculus
1	1.00	1	1	-0.01	-0.01	-0.012	-0.013	-0.012	t	44035	NGNATPQLTKNA	Homo sapiens
1	1.00	1	1	0.01	0.01	0.01	0.01	0.007	t	136753	YHKYTFYKYD	Homo sapiens
1	1.00	1	1	0.07	0.064	0.069	0.069	0.072	t	99136	DDDTFT	Oryctolagus cuniculus
1	1.00	1	1	0.04	0.04	0.028	0.04	0.043	t	27068	ILGGVATYGA	Homo sapiens

1	1.00	1	1	-0.01	-0.009	-0.021	-0.01	-0.011	t	22037	GRFRTIVC GLFPTQ	Myodes glareolu s
1	1.00	1	1	0.02	0.024	0.02	0.027	0.02	t	123610	PPGAMPP Q	Homo sapiens
1	1.00	1	1	0.04	0.042	0.023	0.041	0.042	t	151071	YAADLKST QNAIDEI	Homo sapiens
1	1.00	1	1	0.02	0.02	-0.013	0.02	0.027	t	102172	VDQQQQP TQGDT	Mus musculu s
1	1.00	1	1	0.02	0.018	0.02	0.019	0.018	t	55825	RSELSQLS	Mus musculu s
1	1.00	1	1	-0.02	-0.02	-0.02	-0.013	-0.02	t	136003	TRKDINTV TTVA	Homo sapiens
1	1.00	1	1	0.02	0.024	0.02	0.027	0.022	t	132064	SLLGQLSG QV	Homo sapiens
1	1.00	1	1	-0.01	-0.01	-0.013	-0.01	-0.01	t	98851	KKIIAEKTKI	Homo sapiens
1	1.00	1	1	0.01	0.015	0.002	0.013	0.01	t	75203	YNPLYGSR LGVPDTLG GDPK	Homo sapiens
1	1.00	1	1	0.03	0.026	0.021	0.029	0.03	t	10785	DVVTGAT V	Oryctola gus cuniculu s
1	1.00	1	1	-0.02	-0.015	-0.02	-0.019	-0.021	t	97018	TVTFKATA AT	Homo sapiens(human)
1	1.00	1	1	-0.04	-0.04	-0.043	-0.04	-0.042	t	136074	LVKK	Mus musculu s
1	1.00	1	1	0.05	0.048	0.048	0.049	0.05	t	15323	FCETNGLE YI	Mus musculu s
1	1.00	1	1	0.05	0.05	0.05	0.05	0.049	t	112282	QYLSSFT	Homo sapiens
1	1.00	1	1	0	0.002	-0.007	0.001	-0.001	t	138082	VVGCGSS HHETH	Homo sapiens

1	1.00	1	1	0	-0.01	-0.007	0.001	-0.001	t	138082	VVGCGSS HHETH	Homo sapiens
1	1.00	1	1	0.01	0.01	0.008	0.01	0.008	t	20052	GGTHSQW NKPSKP	Mus musculu s
1	1.00	1	1	-0.02	-0.02	-0.029	-0.02	-0.02	t	66792	TTQTKPKS AF	Mus musculu s
1	1.00	1	1	0.02	0.02	0.016	0.023	0.018	t	58377	SHLKLFTIK YVDVNTN ELLK	Homo sapiens
1	1.00	1	1	0.05	0.05	0.052	0.05	0.052	t	70654	VQTMEEI N	Anas platyrhy nchos
1	1.00	1	1	-0.05	-0.048	-0.056	-0.049	-0.051	t	78370	EFMKEAD KEAY	Homo sapiens
1	1.00	1	1	0.01	0.013	0.009	0.01	0.009	t	24841	HSQWNKP SKP	Mus musculu s
1	1.00	1	1	0.03	0.028	0.023	0.029	0.03	t	1794	AHLKAYAK INEESL	Anas platyrhy nchos
1	1.00	1	1	0	0.006	-0.014	0	0	t	142324	VELAILIDN INNTLVNL EWL	Felis catus
1	1.00	1	1	-0.01	-0.01	-0.012	-0.01	-0.013	t	151652	HWWRAE SDEARRCY NDPKC	Mus musculu s
1	1.00	1	1	0.05	0.047	0.043	0.05	0.05	t	130908	ITRTTLQSD QEEIDY	Oryctola gus cuniculu s
1	1.00	1	1	0	0	-0.005	0	-0.002	t	124925	YYVFFEEQ EDEIIGF	Oryctola gus cuniculu s
1	1.00	1	1	0	0.002	0	0.001	0	t	59922	SNTFINNA	Homo sapiens
1	1.00	1	1	0.02	0.022	0.015	0.019	0.02	t	96964	TKAAEKQ MKD	Homo sapiens

1	1.00	1	1	-0.02	-0.02	-0.02	-0.02	-0.022	t	151729	RYSPSLNTRL	Mus musculus
1	1.00	1	1	0.03	0.032	0.016	0.03	0.032	t	140080	YEKEPTPPTRTPDQ	Rattus norvegicus
1	1.00	1	1	0.03	0.032	0.016	0.03	0.032	t	140080	YEKEPTPPTRTPDQ	Rattus norvegicus
1	1.00	1	1	0	-0.004	-0.002	-0.001	0	t	47363	PEMVDVVT	Oryctolagus cuniculus
1	1.00	1	1	0.02	0.018	0.012	0.023	0.022	t	111253	ENDEIDGVNHQHLPA	Mus musculus
1	1.00	1	1	0.01	0.01	0.008	0.007	0.007	t	29810	KAGNEGLEWVEL	Homo sapiens
1	1.00	1	1	0.02	0.02	0.015	0.02	0.02	t	158924	KSCTWPETHTLWGDGI	Mus musculus
1	1.00	1	1	0.02	0.02	0.014	0.02	0.02	t	122293	CTYVLDPENLTLKAPYEA	Mus musculus
1	1.00	1	1	0.01	0.004	0.008	0.01	0.011	t	74008	YGGL	Oryctolagus cuniculus
1	1.00	1	1	0.01	0.01	0.008	0.01	0.011	t	74008	YGGL	Oryctolagus cuniculus
1	1.00	1	1	-0.01	-0.013	-0.003	-0.006	-0.012	t	130622	TGLVAQGTDSIG	Camelus dromedarius
1	1.00	1	1	0.05	0.048	0.043	0.051	0.05	t	131128	VEMKKEDFDIYDEDE	Mus musculus
1	1.00	1	1	0.05	0.046	0.043	0.051	0.05	t	131128	VEMKKEDFDIYDEDE	Oryctolagus cuniculus
1	1.00	1	1	-0.01	-0.017	-0.024	-0.01	-0.01	t	149980	TLKIPSVHFGSPSGS	Oryctolagus cuniculus

1	1.00	1	1	-0.01	-0.008	-0.017	-0.007	-0.01	t	144594	ARGISRTKYW	Homo sapiens
1	1.00	1	1	0.02	0.02	0.02	0.02	0.02	t	156858	DMSYYYALPTYT	Gallus gallus
1	1.00	1	1	0.01	0.012	0.01	0.017	0.013	t	144325	GVTTV	Homo sapiens
1	1.00	1	1	-0.03	-0.03	-0.045	-0.03	-0.028	t	104295	TAIRPAQVGYNTPYMNF	Oryctolagus cuniculus
1	1.00	1	1	-0.01	-0.01	-0.01	-0.01	-0.01	t	134769	QIFNL	Mus musculus
1	1.00	1	1	0.02	0.02	0.02	0.02	0.018	t	100153	INDQIKFIIN	Mus musculus
1	1.00	1	1	0.01	0.014	0.01	0.017	0.01	t	112931	DATTNPSLILAAAQM	Homo sapiens
1	1.00	1	1	0.03	0.03	0.024	0.03	0.03	t	84461	KLEFFTYLRFDLELT	Homo sapiens
1	1.00	1	1	0.01	0.013	0.005	0.01	0.008	t	78687	IRVRKRKGSNKKAEDTLED	Mus musculus
1	1.00	1	1	-0.05	-0.05	-0.053	-0.05	-0.051	t	98849	KIPAVFKIDA	Homo sapiens
1	1.00	1	1	-0.02	-0.022	-0.034	-0.021	-0.02	t	14772	EVLTRRQSQDPKYVTQRIS	Mus musculus
1	1.00	1	1	0.01	0.012	-0.01	0.007	0.012	t	74475	YKLNFYFDLLRAKL	Homo sapiens
1	1.00	1	1	-0.02	-0.016	-0.015	-0.013	-0.022	t	134815	VKDEPQRRSA	Homo sapiens
1	1.00	1	1	-0.01	-0.012	-0.022	-0.01	-0.01	t	114041	YGSLPQKAQGHRPQDEN	Oryctolagus cuniculus

1	1.00	1	1	0.04	0.038	0.04	0.039	0.04	t	62289	SVPSSSS	Mus musculus
1	1.00	1	1	0.02	0.023	0.019	0.02	0.02	t	6455	CITQYERESQAYY	Mus musculus
1	1.00	1	1	0	0.002	-0.014	0.003	0.002	t	7705	DCHLLGTEELSGIPPPPRGV	Homo sapiens
1	1.00	1	1	0	0	0	0.002	0	t	32635	KNVVTGEVQVLYTA	Homo sapiens
1	1.00	1	1	0.03	0.033	0.021	0.03	0.03	t	100477	RNKTQEEHLK	Mus musculus
1	1.00	1	1	0.04	0.043	0.035	0.04	0.038	t	138316	DALGNVTS	Mus musculus
1	1.00	1	1	0	0	-0.009	0	0	t	135923	FRIPVTINAPLQ	Homo sapiens
1	1.00	1	1	-0.04	-0.044	-0.04	-0.043	-0.042	t	5109	ATPYHPPATP	Oryctolagus cuniculus
1	1.00	1	1	0.01	0.013	-0.002	0.01	0.011	t	125209	QFQIHGPR	Mus musculus
1	1.00	1	1	0.02	0.022	0.014	0.023	0.022	t	115148	KAVSEKKAKGLGESRKDKK	Homo sapiens
1	1.00	1	1	0	0	0.005	0	0	t	115047	ELQFPFLQD	Mus musculus
1	1.00	1	1	-0.01	-0.01	-0.01	-0.01	-0.01	t	131413	ERILTIS	Homo sapiens
1	1.00	1	1	0.01	0.013	0.011	0.01	0.008	t	140610	TQWQGFSVA	Mus musculus
1	1.00	1	1	-0.02	-0.022	-0.023	-0.021	-0.022	t	151073	YEDVNGIRK	Mus musculus

1	1.00	1	1	0.04	0.04	0.033	0.04	0.039	t	137859	FTPGTEYTVEVR	Oryctolagus cuniculus
1	1.00	1	1	-0.04	-0.035	-0.054	-0.039	-0.04	t	39196	LRRLRTACERAKRTL	Homo sapiens
1	1.00	1	1	0.04	0.035	0.032	0.043	0.042	t	157364	GYPKNPEEAYAD	Canis lupus
1	1.00	1	1	0.03	0.033	0.032	0.03	0.03	t	127253	TTYGNA	Mus musculus
1	1.00	1	1	0.01	0.012	0.006	0.011	0.01	t	38814	LQDCTM	Homo sapiens
1	1.00	1	1	-0.02	-0.018	-0.005	-0.023	-0.029	t	45768	NSARARADSCRI	Homo sapiens
1	1.00	1	1	-0.01	-0.008	-0.021	-0.009	-0.01	t	110602	GLKAKPKFTLGKRKATPTT	Homo sapiens
1	1.00	1	1	0.02	0.018	0.012	0.023	0.022	t	95198	AAPTTS	Mus musculus
1	1.00	1	1	0.02	0.016	0.008	0.013	0.019	t	30299	KEALDKYELENHD	Oryctolagus cuniculus
1	1.00	1	1	0	0	0	0	-0.003	t	51371	QLINTNGSWHIN	Rattus norvegicus
1	1.00	1	1	0.02	0.02	0.02	0.02	0.02	t	112395	VYFCHQY	Homo sapiens
1	1.00	1	1	-0.01	-0.01	-0.019	-0.01	-0.009	t	97020	TVTRGTISDP	Oryctolagus cuniculus
1	1.00	1	1	0.01	0.01	0.003	0.01	0.009	t	72503	WGQGG	Mus musculus
1	1.00	1	1	0.02	0.018	0.013	0.024	0.02	t	31076	KGTPTAENPEYLGLDVPV	Mus musculus
1	1.00	1	1	-0.01	-0.01	-0.01	-0.01	-0.012	t	66060	TRLNPD	Mus musculus

1	1.00	1	1	0.02	0.016	0.019	0.02	0.018	t	123838	VWQDE	Mus musculus
1	1.00	1	1	0.01	0.008	0.013	0.014	0.009	t	10692	DVPDYASL	Mus musculus
1	1.00	1	1	-0.02	-0.022	-0.02	-0.021	-0.022	t	46868	PANCPKS	Mus musculus
1	1.00	1	1	0.03	0.034	0.032	0.031	0.03	t	30199	KDLLED	Homo sapiens
1	1.00	1	1	0.01	0.01	0.012	0.012	0.009	t	21887	GQICYYGRDAYLCFL	Homo sapiens
1	1.00	1	1	0.03	0.032	0.023	0.037	0.03	t	117015	KEQFLDGDGWTSRWIESK	Homo sapiens
1	1.00	1	1	0.01	0.01	0.006	0.007	0.008	t	303	AALTKAITAMTQA	Homo sapiens
1	1.00	1	1	-0.02	-0.02	-0.02	-0.02	-0.02	t	96521	KVTKEKPTPP	Homo sapiens(human)
1	1.00	1	1	0.03	0.042	0.032	0.03	0.029	t	4702	ASQKIQLV	Pan troglodytes
1	1.00	1	1	-0.01	-0.012	-0.019	-0.01	-0.009	t	33143	KRGPGSG	Mus musculus
1	1.00	1	1	-0.01	-0.007	-0.013	-0.01	-0.011	t	136074	LVKK	Mus musculus
1	1.00	1	1	0	0	0	0	-0.003	t	33148	KRHDGGCRKE	Homo sapiens
1	1.00	1	1	0	0	-0.007	0	-0.002	t	147559	CTVRGLG	Rattus norvegicus
1	1.00	1	1	-0.01	-0.01	-0.019	-0.01	-0.011	t	45252	NNSTNSGI	Mus musculus

1	1.00	1	1	0.02	0.02	0.02	0.023	0.02	t	71636	VVDLPQ	Homo sapiens
1	1.00	1	1	-0.01	-0.01	-0.01	-0.01	-0.01	t	46825	PAIIPDREV	Homo sapiens
1	1.00	1	1	-0.01	-0.01	-0.023	-0.008	-0.007	t	98832	IPPYCTIAP FGI	Homo sapiens
1	1.00	1	1	0	0.004	0	-0.001	-0.004	t	40938	LYRPSDSSL AGP	Mus musculu s
1	1.00	1	1	0	0.004	0	-0.001	-0.004	t	46371	NVESQPG GQPNT	Mus musculu s
1	1.00	1	1	0.01	0.012	0.01	0.017	0.011	t	107878	GWVAMH PQGDL	Homo sapiens
1	1.00	1	1	-0.01	-0.01	-0.021	-0.004	-0.008	t	114463	LTSRPAHG	Homo sapiens
1	1.00	1	1	-0.04	-0.04	-0.043	-0.04	-0.042	t	136074	LVKK	Mus musculu s
1	1.00	1	1	-0.02	-0.022	-0.019	-0.021	-0.02	t	44268	NIKPLEDKI L	Mus musculu s
1	1.00	1	1	0.03	0.03	0.03	0.03	0.03	t	131107	TGVEAAA	Homo sapiens
1	1.00	1	1	0	-0.002	-0.004	-0.003	0	t	18954	GDDPLLG NQ	Anas platyrhy nchos
1	1.00	1	1	-0.01	-0.01	-0.016	-0.011	-0.01	t	81337	DVTLYGTI KAGV	Homo sapiens
1	1.00	1	1	-0.03	-0.03	-0.037	-0.03	-0.03	t	119475	KKIK	Homo sapiens
1	1.00	1	1	0	-0.002	0	0.003	-0.002	t	21254	GMDGTQL DRW	Mus musculu s
1	1.00	1	1	-0.02	-0.02	-0.02	-0.02	-0.02	t	158610	SFGTFLW	Homo sapiens
1	1.00	1	1	0	-0.002	0	0.009	0.001	t	38304	LNPKTNK WEDK	Mus musculu s

1	1.00	1	1	0	-0.002	0	0.009	0.001	t	6986	CSEYREPLLC	Mus musculus
1	1.00	1	1	0.01	0.008	-0.004	0.003	0.01	t	121100	YYFAPDHRTV	Mus musculus
1	1.00	1	1	0	0	0	0	-0.001	t	153381	ETPLTIAGLKLK	Mus musculus
1	1.00	1	1	0.03	0.032	0.03	0.037	0.029	t	107345	EQRRAA	Homo sapiens
1	1.00	1	1	0.01	0.012	0.002	0.013	0.012	t	110876	GTDRETTSQMIV	Homo sapiens
1	1.00	1	1	-0.08	-0.082	-0.094	-0.077	-0.08	t	95920	TNVARP	Mus musculus
1	1.00	1	1	0.05	0.044	0.049	0.049	0.052	t	99137	DDTFTV	Oryctolagus cuniculus
1	1.00	1	1	0.06	0.062	0.055	0.061	0.059	t	19723	GGESSEELSESSFFNLIT	Homo sapiens
1	1.00	1	1	-0.01	-0.012	-0.01	-0.01	-0.01	t	98383	QPFPQPQLPYP	Mus musculus
1	1.00	1	1	0.03	0.03	0.023	0.03	0.03	t	127650	FEPFSNKG	Homo sapiens
1	1.00	1	1	0	0	-0.004	0	-0.002	t	52158	QQTFPQQPQL	Mus musculus
1	1.00	1	1	0.03	0.032	0.024	0.031	0.03	t	25404	IAQVATVS	Homo sapiens
1	1.00	1	1	0	-0.007	-0.005	0.001	0	t	147284	SIFGLAPSKALDS	Homo sapiens
1	1.00	1	1	0	0	0	0	-0.003	t	59971	SPAFTKGEKVD	Oryctolagus cuniculus
1	1.00	1	1	0.01	0.012	0.01	0.017	0.011	t	132690	TCSADGGTLFYNLCL	Homo sapiens

1	1.00	1	1	0.04	0.038	0.041	0.039	0.04	t	121054	VTGEVIFQTPL	Rattus norvegicus
1	1.00	1	1	0.04	0.036	0.041	0.039	0.04	t	121054	VTGEVIFQTPL	Oryctolagus cuniculus
1	1.00	1	1	0.04	0.038	0.039	0.039	0.042	t	56711	SAAQEKISF	Mus musculus
1	1.00	1	1	-0.02	-0.018	-0.02	-0.019	-0.021	t	40724	LYCGGPMFNSKGAQ	Homo sapiens
1	1.00	1	1	0.01	0.008	0.008	0.013	0.011	t	99419	LGYTDRIG	Mus musculus
1	1.00	1	1	0.01	0.01	0.01	0.01	0.01	t	113778	QQPGQ	Homo sapiens
1	1.00	1	1	0.02	0.021	0.013	0.019	0.022	t	19118	GDSSNAGT	Equus caballus
1	1.00	1	1	0.02	0.018	0.013	0.019	0.022	t	19118	GDSSNAGT	Mus musculus
1	1.00	1	1	0.01	0.01	0.01	0.01	0.01	t	140841	EEKPAVLGHDEA	Oryctolagus cuniculus
1	1.00	1	1	-0.05	-0.05	-0.05	-0.05	-0.053	t	117557	KVQLLRNWH	Homo sapiens
1	1.00	1	1	0.01	0.012	0.01	0.013	0.01	t	43567	NEDEQIGARIVL	Homo sapiens
1	1.00	1	1	0	0	0.002	0	-0.004	t	32807	KPNLSSKRSELSQLS	Mus musculus
1	1.00	1	1	-0.01	-0.01	-0.023	-0.01	-0.01	t	158836	IHNDVEAWIDRYKYLP	Mus musculus
1	1.00	1	1	0.01	0.012	0.005	0.007	0.008	t	115760	GLNYYQQKPV	Homo sapiens

1	1.00	1	1	0	-0.007	0.009	0.007	0	t	109286	HEGLHNH	Homo sapiens
1	1.00	1	1	0.01	0.01	0.017	0.019	0.009	t	107744	APVDSRTYTRVADHI	Homo sapiens
1	1.00	1	1	0.02	0.02	0.012	0.021	0.02	t	39216	LRSSSGCV	Homo sapiens
1	1.00	1	1	0	0	-0.007	0	0	t	125246	RRQPHVMS	Mus musculus
1	1.00	1	1	0	0.008	-0.007	0	0	t	125246	RRQPHVMS	Macaca fascicularis
1	1.00	1	1	0	0	0.005	0.001	0	t	138238	SMLDTPT	Mus musculus
1	1.00	1	1	-0.03	-0.03	-0.03	-0.03	-0.032	t	103940	GLTLPGYKYLGPGNSLD	Oryctolagus cuniculus
1	1.00	1	1	-0.01	-0.01	-0.018	-0.014	-0.01	t	110932	LNLKIDGLKISV	Homo sapiens
1	1.00	1	1	0.01	0.012	0.004	0.011	0.012	t	47324	PEKGGR	Homo sapiens
1	1.00	1	1	0.03	0.03	0.03	0.03	0.029	t	143362	ETPLMKE	Mus musculus
1	1.00	1	1	0.03	0.03	0.03	0.03	0.029	t	143362	ETPLMKE	Mus musculus
1	1.00	1	1	0.01	0.014	0.01	0.017	0.009	t	116682	CHGRGTCNYYANAYS	Homo sapiens
1	1.00	1	1	0.02	0.02	0.015	0.02	0.019	t	156903	KNRGIIKTFSN	Mus musculus
1	1.00	1	1	0.01	0.012	0.01	0.009	0.007	t	136566	PYKYTFYKYD	Homo sapiens
1	1.00	1	1	0	0	-0.014	0	0.001	t	51942	QPQRPFGQQP	Mus musculus
1	1.00	1	1	0.04	0.04	0.045	0.04	0.04	t	17557	FQYSPG	Homo sapiens

1	1.00	1	1	-0.03	-0.028	-0.046	-0.023	-0.026	t	135911	DRVRSITL GNST	Homo sapiens
1	1.00	1	1	-0.01	-0.01	-0.01	-0.004	-0.01	t	158695	ASKSADDV KKAFKVI	Homo sapiens
1	1.00	1	1	0.05	0.05	0.05	0.05	0.049	t	120342	YNAEEVEF TFAVDND	Rattus norvegic us
1	1.00	1	1	0.05	0.05	0.05	0.05	0.049	t	120342	YNAEEVEF TFAVDND	Rattus norvegic us
1	1.00	1	1	-0.02	-0.018	-0.025	-0.019	-0.02	t	54862	RMAWDM MMN	Homo sapiens
1	1.00	1	1	0.04	0.04	0.033	0.04	0.04	t	132793	DLQEAKYE TFS	Mus musculu s
1	1.00	1	1	0.01	0.012	0.017	0.013	0.006	t	14435	ETIYNTTLK Y	Homo sapiens
1	1.00	1	1	-0.05	-0.046	-0.057	-0.043	-0.048	t	133231	MGPRRRS RKP	Homo sapiens
1	1.00	1	1	0.03	0.033	0.04	0.03	0.026	t	61824	STPLLYPSL A	Mus musculu s
1	1.00	1	1	-0.01	-0.008	-0.01	-0.003	-0.01	t	104343	VSGGPFI	Homo sapiens
1	1.00	1	1	-0.03	-0.028	-0.037	-0.023	-0.029	t	118348	FHSGKHY WEVDVTG KE	Homo sapiens
1	1.00	1	1	0	0	0	0.006	0	t	77841	AYDFLYNY L	Homo sapiens
1	1.00	1	1	0.01	0.011	0.006	0.009	0.01	t	41449	METMKL MMEKVD	Equus caballus
1	1.00	1	1	0.03	0.03	0.03	0.034	0.03	t	109425	KNQVSLT	Homo sapiens
1	1.00	1	1	0	0	0	0	0	t	98939	PQPGP	Mus musculu s
1	1.00	1	1	0.02	0.021	0.006	0.023	0.022	t	30140	KDEPNGR ASDT	Equus caballus

1	1.00	1	1	0.01	0.012	-0.004	0.013	0.012	t	15519	FEELCGELFRGTLQPV	Homo sapiens
1	1.00	1	1	0	0.002	0	0.001	0.001	t	38522	LPHSNL	Homo sapiens
1	1.00	1	1	0	0.002	0	0.001	0.001	t	38522	LPHSNL	Homo sapiens
1	1.00	1	1	0.01	0.016	0.011	0.011	0.01	t	99208	EKMYAC	Equus caballus
1	1.00	1	1	0	0.004	0	0.007	0	t	127926	AKKGEKVPKGKKGKA	Homo sapiens
1	1.00	1	1	0.01	0.012	0.01	0.011	0.008	t	58076	SGHRMAWDM	Homo sapiens
1	1.00	1	1	0.01	0.01	0.007	0.01	0.011	t	11411	EDPASR	Mus musculus
1	1.00	1	1	-0.02	-0.018	-0.02	-0.013	-0.019	t	113514	HHPARTAHYGSLPQK	Homo sapiens
1	1.00	1	1	-0.02	-0.018	-0.02	-0.013	-0.019	t	113514	HHPARTAHYGSLPQK	Homo sapiens
1	1.00	1	1	0	0	0	0.001	-0.002	t	46841	PAKLLKERGFFGAIAGFLE	Mus musculus
1	1.00	1	1	0.01	0.01	0.002	0.01	0.01	t	88060	RMAFTEIKVQ	Homo sapiens
1	1.00	1	1	0.03	0.026	0.03	0.028	0.031	t	142443	LSTTEVAM	Oryctolagus cuniculus
1	1.00	1	1	-0.01	-0.01	-0.01	-0.01	-0.01	t	159294	QPLWLL	Oryctolagus cuniculus
1	1.00	1	1	0.01	0.008	0.003	0.009	0.009	t	19637	GFTTNEPRYNVFAE	Mus musculus

1	1.00	1	1	0	0	0	0.004	0	t	118610	SPGFNDGGKNTAPLTLC	Homo sapiens
1	1.00	1	1	-0.02	-0.018	-0.017	-0.013	-0.023	t	30302	KEALISSLTRGKLTYTQQ	Homo sapiens
1	1.00	1	1	0.04	0.04	0.04	0.04	0.04	t	112392	VTMTCKS	Homo sapiens
1	1.00	1	1	0.03	0.027	0.022	0.03	0.03	t	13460	ENDVLNQETEEEMEK	Oryctolagus cuniculus
1	1.00	1	1	0	0	0	0	-0.003	t	48597	PNANPN	Mus musculus
1	1.00	1	1	-0.03	-0.024	-0.023	-0.026	-0.03	t	123878	YMPPPGMI	Homo sapiens
1	1.00	1	1	-0.02	-0.02	-0.022	-0.02	-0.019	t	78457	LRLPPAP	Mus musculus
1	1.00	1	1	0.02	0.024	-0.001	0.017	0.022	t	75645	YRNILYFPNHLPEELRKQTK	Homo sapiens
1	1.00	1	1	0	0.002	-0.005	0.001	0	t	142047	TYFKGVSTELKDKK	Homo sapiens
1	1.00	1	1	0.02	0.024	0.016	0.021	0.02	t	23048	GVLSVDLSLTVELKI	Homo sapiens
1	1.00	1	1	-0.01	-0.007	-0.01	-0.01	-0.011	t	48911	PPSDIKGK	Mus musculus
1	1.00	1	1	0.01	0.012	0.003	0.017	0.01	t	116171	TQDQDPBBHFFKNIVTPR	Homo sapiens
1	1.00	1	1	0.03	0.033	0.048	0.03	0.02	t	54348	RKGRGGANPVFRPTGGTEVF	Mus musculus
1	1.00	1	1	0	0.002	-0.003	0.003	0.002	t	61124	SSIVYEAADMIMHT	Homo sapiens

1	1.00	1	1	0.04	0.04	0.04	0.04	0.04	t	79403	SVGDYG	Mus musculus
1	1.00	1	1	0.02	0.02	0.02	0.02	0.02	t	140830	AVLGHDEAAYSK	Oryctolagus cuniculus
1	1.00	1	1	0.03	0.03	0.03	0.03	0.03	t	117546	KIRRSPSKPLPEVT	Homo sapiens
1	1.00	1	1	0.02	0.02	0.015	0.02	0.02	t	73636	YEDRYYRE	Mus musculus
1	1.00	1	1	0	0	-0.016	0	0.002	t	64057	THNQWNKPSKPKTNLK	Oryctolagus cuniculus
1	1.00	1	1	0	0	-0.016	0	0.002	t	64057	THNQWNKPSKPKTNLK	Oryctolagus cuniculus
1	1.00	1	1	0.02	0.02	0.02	0.02	0.02	t	78552	VERLGDYLIDQGL	Homo sapiens
1	1.00	1	1	-0.01	-0.01	-0.001	-0.01	-0.01	t	48418	PLLYPS	Homo sapiens
1	1.00	1	1	0.03	0.028	0.031	0.033	0.032	t	11377	EDLDGA	Mus musculus
1	1.00	1	1	0	0	-0.004	0	-0.002	t	52085	QQPFPQPQQQ	Mus musculus
1	1.00	1	1	0	0	-0.004	0	-0.002	t	51929	QPQQPQQSFP	Mus musculus
1	1.00	1	1	0	0	-0.004	0	-0.002	t	52089	QQPFPQSQQP	Mus musculus
1	1.00	1	1	0.05	0.05	0.05	0.05	0.05	t	40380	LVGVVT	Homo sapiens
1	1.00	1	1	0.01	0.008	0.006	0.009	0.008	t	65698	TPPRPAGDNATVAAGHAT	Mus musculus

1	1.00	1	1	0.02	0.018	0.015	0.019	0.02	t	74708	YLILSARDVL	Mus musculus
1	1.00	1	1	0.03	0.03	0.03	0.036	0.03	t	158753	FIEVEELKLFLQNFC	Homo sapiens
1	1.00	1	1	0	-0.004	-0.009	-0.001	0.001	t	51473	QLPNVGIT	Oryctolagus cuniculus
1	1.00	1	1	-0.02	-0.022	-0.027	-0.021	-0.02	t	19093	GDRAAGQPAGDRAAGQPA	Mus musculus
1	1.00	1	1	-0.02	-0.022	-0.027	-0.021	-0.02	t	19093	GDRAAGQPAGDRAAGQPA	Mus musculus
1	1.00	1	1	-0.02	-0.022	-0.027	-0.021	-0.02	t	19093	GDRAAGQPAGDRAAGQPA	Mus musculus
1	1.00	1	1	-0.02	-0.022	-0.027	-0.021	-0.02	t	19093	GDRAAGQPAGDRAAGQPA	Mus musculus
1	1.00	1	1	0.01	0.012	0.005	0.011	0.01	t	66082	TRQAQDIQP	Homo sapiens
1	1.00	1	1	0.03	0.03	0.03	0.03	0.03	t	60364	SQAEAALENL	Homo sapiens
1	1.00	1	1	0.03	0.026	0.033	0.029	0.029	t	15529	FEFPPPPTDE	Oryctolagus cuniculus
1	1.00	1	1	0.06	0.062	0.059	0.067	0.063	t	144320	GSEN	Homo sapiens
1	1.00	1	1	-0.01	-0.01	-0.01	-0.016	-0.012	t	67916	VDCSRFPNA	Homo sapiens
1	1.00	1	1	0	0.004	0.007	-0.001	-0.003	t	131129	VFQSYFSMPTPAHL	Homo sapiens
1	1.00	1	1	0.03	0.03	0.029	0.03	0.029	t	10550	DVCSDSDNDGVCDNV	Mus musculus
1	1.00	1	1	0.02	0.02	0.02	0.02	0.019	t	119620	NPDDYGGVK	Rattus norvegicus

1	1.00	1	1	0.02	0.02	0.02	0.02	0.019	t	24035	HIDSQK	Oryctolagus cuniculus
1	1.00	1	1	-0.01	-0.012	-0.026	-0.017	-0.01	t	97916	AVVGKPGSDV	Mus musculus
1	1.00	1	1	-0.01	-0.014	-0.026	-0.017	-0.01	t	97916	AVVGKPGSDV	Oryctolagus cuniculus
1	1.00	1	1	0.03	0.03	0.021	0.027	0.03	t	71902	VVYDYQHK	Homo sapiens
1	1.00	1	1	0.01	0.006	0.005	0.011	0.009	t	133775	AETNRAPFDLAEAE	Oryctolagus cuniculus
1	1.00	1	1	-0.01	-0.008	-0.01	-0.011	-0.013	t	28911	ITGHRMAWDMMMNWS	Homo sapiens
1	1.00	1	1	0.02	0.022	0.018	0.023	0.02	t	10932	DYNPLLVE	Homo sapiens
1	1.00	1	1	0	0	0	0	-0.002	t	48001	PITNDQKK	Mus musculus
1	1.00	1	1	0.02	0.022	0.012	0.021	0.02	t	41177	MAYRFNGIGVTQNVLYE	Homo sapiens
1	1.00	1	1	0	0	-0.005	-0.002	0	t	17271	FPDLGV	Homo sapiens
1	1.00	1	1	-0.04	-0.04	-0.04	-0.04	-0.043	t	110789	ARLKSWFEPLVEDMQRQWAG	Mus musculus
1	1.00	1	1	-0.02	-0.02	-0.028	-0.02	-0.02	t	21424	GNFGDQDLIRQGTDYKHW	Homo sapiens
1	1.00	1	1	0.04	0.038	0.038	0.039	0.04	t	56711	SAAQEKISF	Mus musculus
1	1.00	1	1	0.01	0.01	0.002	0.013	0.012	t	111663	QDKILTHYENDSTD	Homo sapiens

1	1.00	1	1	-0.02	-0.014	-0.032	-0.019	-0.02	t	15461	FDSSGSFL GHI	Equus caballus
1	1.00	1	1	0	-0.004	-0.003	0	0	t	100454	RAVPYYGP GVDYKN	Oryctola gus cuniculu s
1	1.00	1	1	-0.01	-0.013	-0.01	-0.01	-0.009	t	113527	HYGSLPQK	Oryctola gus cuniculu s
1	1.00	1	1	-0.04	-0.036	-0.047	-0.039	-0.041	t	138028	SAGALQLL VVGC	Homo sapiens
1	1.00	1	1	0.03	0.032	0.026	0.027	0.028	t	106904	THEGGQL HCT	Homo sapiens
1	1.00	1	1	0.01	0.009	0.003	0.007	0.01	t	65006	TLPIILKALY MLST	Myodes glareolu s
1	1.00	1	1	0.03	0.027	0.022	0.03	0.03	t	4534	ASLLTTAE VV	Oryctola gus cuniculu s
1	1.00	1	1	0.05	0.052	0.047	0.051	0.05	t	8371	DGEGAP	Homo sapiens
1	1.00	1	1	0.03	0.03	0.03	0.03	0.03	t	135465	GSDYEDRY YREN	Mus musculu s
1	1.00	1	1	0.05	0.052	0.038	0.051	0.052	t	22398	GSKVPEDT	Homo sapiens
1	1.00	1	1	0.01	0.01	0.01	0.01	0.008	t	124560	NGVELRD	Mus musculu s
1	1.00	1	1	0	0	0	0.007	0	t	135908	DINTVTTV AQSR	Homo sapiens
1	1.00	1	1	0.01	0.014	0.003	0.011	0.012	t	42580	MSKAKQY NICCKFKE	Homo sapiens

1	1.00	1	1	0.02	0.024	0.013	0.021	0.02	t	79926	MASMTG GQQMG	MD
1	1.00	1	1	0.01	0.01	-0.003	0.01	0.01	t	110198	CPPDIIPKV EGKTIA	Mus musculu s
1	1.00	1	1	0.01	0.007	-0.003	0.01	0.01	t	110198	CPPDIIPKV EGKTIA	Oryctola gus cuniculu s
1	1.00	1	1	0	0	-0.002	0	-0.003	t	119052	WNPADYG GIK	Rattus norvegic us
1	1.00	1	1	0.01	0.01	0.001	0.01	0.01	t	135987	RSITLGNST ITT	Homo sapiens
1	1.00	1	1	0	0.002	-0.007	0.003	0.001	t	144772	SAHTSHLV GS	Homo sapiens
1	1.00	1	1	0.02	0.024	0.01	0.02	0.022	t	20769	GLAQALID QKVKEI	Myodes glareolu s
1	1.00	1	1	0	0.004	-0.007	0.007	0	t	139601	SCSRALAR EVDLKDY	Homo sapiens
1	1.00	1	1	0.04	0.04	0.047	0.049	0.042	t	130850	GGIYQDLV S	Homo sapiens
1	1.00	1	1	0.05	0.052	0.05	0.051	0.05	t	98786	GASEL	Homo sapiens
1	1.00	1	1	-0.02	-0.018	-0.03	-0.017	-0.021	t	61412	SSTSIVYILI AVCLG	Homo sapiens
1	1.00	1	1	-0.02	-0.02	-0.025	-0.017	-0.02	t	52680	QVCGPVY CFT	Homo sapiens
1	1.00	1	1	0	0	0	0.004	-0.001	t	43242	NANPNAN PNANP	Homo sapiens
1	1.00	1	1	0	0	0	0.004	-0.001	t	43242	NANPNAN PNANP	Homo sapiens
1	1.00	1	1	0	0	0	0.004	-0.001	t	43242	NANPNAN PNANP	Homo sapiens

1	1.00	1	1	0	0	0	0.004	-0.001	t	43242	NANPNANPNANP	Homo sapiens
1	1.00	1	1	0	0	0	0.004	-0.001	t	43242	NANPNANPNANP	Homo sapiens
1	1.00	1	1	0.01	0.013	0.01	0.01	0.012	t	110229	FTEPSVLH	Mus musculus
1	1.00	1	1	0.01	0.016	0.017	0.017	0.01	t	127663	GGYVKLFPNS	Homo sapiens
1	1.00	1	1	0	0.002	-0.011	0.001	-0.001	t	57296	SDSVGQQIKVIPVDPYFFQM	Homo sapiens
1	1.00	1	1	-0.03	-0.03	-0.03	-0.03	-0.033	t	95387	FLKKISQRYQ	Homo sapiens
1	1.00	1	1	-0.01	-0.01	-0.022	-0.01	-0.01	t	104521	IGTLKKILDETVKDKIAKEQ	Rattus norvegicus
1	1.00	1	1	0.03	0.03	0.021	0.023	0.031	t	110982	QYNKPLCDLL	Homo sapiens
1	1.00	1	1	0.03	0.03	0.021	0.023	0.031	t	110982	QYNKPLCDLL	Homo sapiens
1	1.00	1	1	-0.01	-0.008	-0.007	-0.01	-0.012	t	132885	PVRLAE	Oryctolagus cuniculus
1	1.00	1	1	0.03	0.026	0.029	0.029	0.028	t	110862	GGPTLVSLHELPAETP	Oryctolagus cuniculus
1	1.00	1	1	0.01	0.01	-0.01	0.003	0.01	t	107824	ELWDKRYGGRSNI	Homo sapiens
1	1.00	1	1	0.03	0.028	0.025	0.033	0.029	t	16396	FKLEKEVAE	Mus musculus
1	1.00	1	1	-0.03	-0.032	-0.03	-0.03	-0.032	t	111211	DPVGPSDPSIVSLVEETSF	Oryctolagus cuniculus

1	1.00	1	1	0	0.002	0	0.007	-0.002	t	121925	TPFLPKIFIKPNAQK	Homo sapiens
1	1.00	1	1	0.02	0.022	0.009	0.021	0.02	t	110589	GDICNTMHYTNWTHIYICEE	Homo sapiens
1	1.00	1	1	0	0	-0.007	0	-0.002	t	122430	KGLRRDLDASREAK	Mus musculus
1	1.00	1	1	-0.01	-0.01	-0.01	-0.01	-0.009	t	115342	LDTDYKKYLL	Homo sapiens
1	1.00	1	1	0.03	0.03	0.036	0.03	0.03	t	14992	EYEDLLKA	Mus musculus
1	1.00	1	1	0.02	0.022	0.014	0.021	0.02	t	58903	SKNFNFEV	Homo sapiens
1	1.00	1	1	-0.01	-0.01	-0.01	-0.01	-0.01	t	46825	PAIIPDREV	Homo sapiens
1	1.00	1	1	-0.05	-0.05	-0.051	-0.047	-0.05	t	35252	LDPHAFHLLL	Homo sapiens
1	1.00	1	1	0	-0.002	-0.009	0.003	0.002	t	47014	PAYYTKDTNNNLTL	Mus musculus
1	1.00	1	1	0.01	0.01	0.003	0.01	0.01	t	39174	LRQLDFVEEVLRKHPDKVE	Homo sapiens
1	1.00	1	1	-0.01	-0.013	-0.026	-0.01	-0.008	t	31067	KGTAPFGVAFGNS	Oryctolagus cuniculus
1	1.00	1	1	-0.01	-0.008	-0.003	-0.003	-0.01	t	108007	LLPWLDGHPM	Homo sapiens
1	1.00	1	1	0.01	0.012	0.003	0.017	0.011	t	105886	IPSTVKAGELEKI	Homo sapiens
1	1.00	1	1	0.02	0.024	0.018	0.027	0.02	t	132044	RLRKWTTVDSISVNT	Homo sapiens
1	1.00	1	1	-0.01	-0.008	-0.005	-0.003	-0.012	t	122943	WDPAQPPRD	Homo sapiens

1	1.00	1	1	0.06	0.06	0.053	0.06	0.06	t	121130	AICSQCSD	Homo sapiens
1	1.00	1	1	0.01	0.01	0.01	0.01	0.01	t	105068	AEFDLD	Oryctolagus cuniculus
1	1.00	1	1	0	0	-0.007	0	-0.001	t	122375	GLINRAKAVKQFEESQG	Mus musculus
1	1.00	1	1	0	-0.002	0	-0.001	-0.003	t	11571	EEEDDDMGFGLFD	Mus musculus
1	1.00	1	1	-0.01	-0.01	-0.02	-0.01	-0.008	t	60959	SSDSQTITNPAY	Oryctolagus cuniculus
1	1.00	1	1	0.04	0.04	0.04	0.04	0.04	t	119753	AQGARVYAYV	Mus musculus
1	1.00	1	1	0.04	0.043	0.04	0.04	0.04	t	119753	AQGARVYAYV	Mus musculus
1	1.00	1	1	0.04	0.04	0.04	0.04	0.04	t	119753	AQGARVYAYV	Oryctolagus cuniculus
1	1.00	1	1	0.01	0.01	0.01	0.01	0.01	t	62907	TALDTSNK	Oryctolagus cuniculus
1	1.00	1	1	-0.01	-0.016	-0.024	-0.01	-0.01	t	43153	NAGKEGAIIFQQ	Oryctolagus cuniculus
1	1.00	1	1	0	-0.004	-0.002	0.003	0.001	t	99761	VKVGDK	Oryctolagus cuniculus
1	1.00	1	1	0.02	0.018	0.028	0.019	0.018	t	79040	PGQNLCYTK	Mus musculus
1	1.00	1	1	0	-0.004	0	0.003	-0.002	t	71199	VSVNDPFIDLEYM	Oryctolagus cuniculus
1	1.00	1	1	0	-0.002	0	0.003	-0.002	t	43233	NANP	Mus musculus

1	1.00	1	1	0	-0.004	0	0.003	-0.002	t	46895	PAPQAPYQGYQEPPAPQAPY	Oryctolagus cuniculus
1	1.00	1	1	0	-0.004	0	0.003	-0.002	t	34045	KVIHDKFEIVE	Oryctolagus cuniculus
1	1.00	1	1	0	-0.004	0	0.003	-0.002	t	8942	DKNEKGQHEIVEVEEILPE	Oryctolagus cuniculus
1	1.00	1	1	0.04	0.04	0.031	0.04	0.04	t	135951	LLESQIATIEQT	Homo sapiens
1	1.00	1	1	-0.01	-0.006	-0.016	-0.009	-0.011	t	47662	PGGGQIVGGVYLLPRR	Homo sapiens
1	1.00	1	1	-0.01	-0.008	-0.016	-0.009	-0.011	t	47662	PGGGQIVGGVYLLPRR	Homo sapiens
1	1.00	1	1	-0.01	-0.008	-0.016	-0.009	-0.011	t	47662	PGGGQIVGGVYLLPRR	Homo sapiens
1	1.00	1	1	0.03	0.03	0.03	0.041	0.032	t	118624	TLDPDTANPWLILSE	Homo sapiens
1	1.00	1	1	0.01	0.01	0.003	0.01	0.01	t	72503	WGQGG	Mus musculus
1	1.00	1	1	0	0	-0.001	0.006	0	t	30661	KEVPALTAVETGAT	Homo sapiens
1	1.00	1	1	0.04	0.038	0.031	0.037	0.04	t	13657	EPGVLMGGQSESVEFT	Mus musculus
1	1.00	1	1	-0.04	-0.04	-0.052	-0.038	-0.038	t	96320	GKKPNIWYSL	Homo sapiens(human)
1	1.00	1	1	0.01	0.007	-0.001	0.009	0.01	t	41117	MASEAKESPANNPGL	Oryctolagus cuniculus
1	1.00	1	1	0.02	0.02	0.02	0.02	0.022	t	136088	VPPTPQ	Oryctolagus cuniculus

1	1.00	1	1	0.02	0.02	0.02	0.023	0.02	t	49295	PSAPPL	Homo sapiens
1	1.00	1	1	0.02	0.02	0.02	0.023	0.02	t	49295	PSAPPL	Homo sapiens
1	1.00	1	1	0	0.004	0.001	0.003	0	t	34215	KVTKDGVTVA	Homo sapiens
1	1.00	1	1	0	0.002	0.001	0.003	0	t	34215	KVTKDGVTVA	Homo sapiens
1	1.00	1	1	0.01	0.01	0.005	0.01	0.01	t	145838	IVSLVEETSFIDAGAP	Mus musculus
1	1.00	1	1	0	0.004	0	0.003	-0.001	t	118434	ISKGANPVEIRRG	Homo sapiens
1	1.00	1	1	0.02	0.023	0.011	0.02	0.02	t	100501	SEEEKELLNR	Mus musculus
1	1.00	1	1	-0.02	-0.021	-0.027	-0.02	-0.02	t	41609	MGQHPAKS	Anas platyrhynchos
1	1.00	1	1	0	0	-0.003	0	0	t	22217	GRTPFII	Homo sapiens
1	1.00	1	1	-0.04	-0.04	-0.038	-0.04	-0.04	t	120249	TPVLVWIYGG	Mus musculus
1	1.00	1	1	-0.04	-0.043	-0.038	-0.04	-0.04	t	120249	TPVLVWIYGG	Oryctolagus cuniculus
1	1.00	1	1	-0.01	-0.013	-0.015	-0.01	-0.012	t	54975	RNCKKLGPRENVA	Oryctolagus cuniculus
1	1.00	1	1	0.06	0.06	0.06	0.06	0.062	t	127610	AVYFKEQFLD	Homo sapiens
1	1.00	1	1	0.01	0.012	-0.01	0.007	0.012	t	75541	YQQYPQQ	Homo sapiens
1	1.00	1	1	0.03	0.028	0.028	0.029	0.03	t	45496	NPTPQEIP	Anas platyrhynchos

1	1.00	1	1	0.04	0.038	0.031	0.039	0.04	t	25060	HVLDHVQT	Anas platyrhynchos
1	1.00	1	1	0.02	0.02	0.02	0.023	0.02	t	19310	GEIRPS	Homo sapiens
1	1.00	1	1	-0.01	-0.008	-0.021	-0.013	-0.009	t	6219	CFDVFKELK	Homo sapiens
1	1.00	1	1	-0.03	-0.026	-0.03	-0.033	-0.032	t	106340	DIMPCLHFVKGE	Homo sapiens
1	1.00	1	1	0.07	0.07	0.074	0.07	0.072	t	136059	EGY	Rattus norvegicus
1	1.00	1	1	0.02	0.02	0.013	0.02	0.018	t	72908	WNSQKDILEQARA	Mus musculus
1	1.00	1	1	0.01	0.01	0.002	0.01	0.009	t	102333	CCKRNDEVLFRLENHAETLR	Mus musculus
1	1.00	1	1	0.01	0.013	0.002	0.01	0.009	t	102333	CCKRNDEVLFRLENHAETLR	Mus musculus
1	1.00	1	1	0.01	0.01	0.002	0.01	0.009	t	102333	CCKRNDEVLFRLENHAETLR	Mus musculus
1	1.00	1	1	0.05	0.05	0.05	0.054	0.05	t	135914	EEIAQVATISAN	Homo sapiens
1	1.00	1	1	0.05	0.05	0.043	0.047	0.05	t	42678	MSTLQELQENITA	Homo sapiens
1	1.00	1	1	0	0	0	0.006	0	t	118490	LKDKYKNIGAKLV	Homo sapiens
1	1.00	1	1	0.01	0.007	-0.006	0.01	0.014	t	10801	DWEDRYYRE	Oryctolagus cuniculus
1	1.00	1	1	-0.01	-0.01	-0.01	-0.004	-0.01	t	77842	AYIDFEMKI	Homo sapiens
1	1.00	1	1	-0.03	-0.032	-0.039	-0.027	-0.027	t	115483	SFFDIGGSVSAGFSN	Mus musculus
1	1.00	1	1	0.01	0.013	0.008	0.01	0.01	t	53906	RGLAQSLIDQ	Mus musculus

1	1.00	1	1	0	0	-0.005	0	0	t	133870	LAKLAGGVAVICAGA	Homo sapiens
1	1.00	1	1	0	0	-0.005	0	0	t	133870	LAKLAGGVAVICAGA	Homo sapiens
1	1.00	1	1	0	-0.002	-0.005	-0.001	0	t	27896	IPKGTLTW	Anas platyrhynchos
1	1.00	1	1	-0.01	-0.01	-0.003	-0.01	-0.012	t	95750	QYLYQGPIVL	Homo sapiens
1	1.00	1	1	-0.01	-0.012	-0.02	-0.007	-0.008	t	69183	VKAVTTLLKA	Mus musculus
1	1.00	1	1	0.01	0.01	0.01	0.01	0.01	t	140934	YGEEKPAVLGHD	Oryctolagus cuniculus
1	1.00	1	1	0.01	0.01	0.01	0.01	0.01	t	78517	SGPISKY	Mus musculus
1	1.00	1	1	0	-0.004	-0.009	-0.007	-0.002	t	133880	LKLLLKEYV	Oryctolagus cuniculus
1	1.00	1	1	0	0.002	0	0.001	0	t	47289	PEGRTWAQ	Homo sapiens
1	1.00	1	1	0	0	0	-0.001	-0.004	t	105127	DAEFRHD	Mus musculus
1	1.00	1	1	0.04	0.04	0.04	0.04	0.038	t	78001	SEGLKEET	Mus musculus
1	1.00	1	1	-0.04	-0.04	-0.045	-0.04	-0.04	t	26372	IHFG	Mus musculus
1	1.00	1	1	-0.04	-0.04	-0.045	-0.04	-0.04	t	26372	IHFG	Mus musculus
1	1.00	1	1	-0.02	-0.018	-0.032	-0.029	-0.022	t	115694	AVPYPQRDMP	Homo sapiens
1	1.00	1	1	0	0.002	-0.008	0.001	0	t	1460	AGCLIGAEHVDTSYECD	Homo sapiens

1	1.00	1	1	0.05	0.05	0.045	0.05	0.05	t	145838	IVSLVEETS FIDAGAP	Mus musculu s
1	1.00	1	1	0	0	-0.008	0	0	t	86504	MSAAYDN IYT	Homo sapiens
1	1.00	1	1	0	0.005	-0.017	0	0.002	t	32687	KPEASSTN	Homo sapiens
1	1.00	1	1	-0.02	-0.02	-0.032	-0.014	-0.018	t	106634	LVGPRGER GFP	Homo sapiens
1	1.00	1	1	-0.02	-0.022	-0.027	-0.027	-0.021	t	108064	PGPPGPP	Mus musculu s
1	1.00	1	1	0	0	0	-0.006	-0.006	t	125209	QFQIHGPR	Mus musculu s
1	1.00	1	1	0	0	0	-0.006	-0.006	t	117140	DWEYSVW LSN	Mus musculu s
1	1.00	1	1	0	0	0	-0.006	-0.006	t	117223	NTWTTCD SIAFPSK	Mus musculu s
1	1.00	1	1	0	0	0	-0.006	-0.006	t	113879	SQRSQDE N	Rattus norvegic us
1	1.00	1	1	0	0	0	-0.006	-0.006	t	113966	TTHYGSLP QKAQGHR PQDE	Oryctola gus cuniculu s
1	1.00	1	1	0	0	0	-0.006	-0.006	t	113013	GSLPQKSQ RSQDEN	Rattus norvegic us
1	1.00	1	1	0	0	0	-0.006	-0.006	t	107242	SQTTANPS CPEGT	Rattus norvegic us
1	1.00	1	1	0	0	0	-0.006	-0.006	t	113966	TTHYGSLP QKAQGHR PQDE	Oryctola gus cuniculu s
1	1.00	1	1	0	0	0	-0.006	-0.006	t	107951	KTTICGKG LSATVT	Mus musculu s
1	1.00	1	1	0	0	0	-0.006	-0.006	t	108200	VSLGKWL GHPDKF	Mus musculu s

1	1.00	1	1	0	0	0	-0.006	-0.006	t	18441	FYNQKFKG KATL	Mus musculu s
1	1.00	1	1	0.01	0.01	0.003	0.013	0.01	t	26839	IKLKDCP	Homo sapiens
1	1.00	1	1	0.01	0.01	0.01	0.021	0.012	t	118281	CPLNIGSQ GSTDY	Homo sapiens
1	1.00	1	1	-0.01	-0.01	-0.005	-0.01	-0.013	t	122766	FQGLCNET LTLKLYN	Mus musculu s
1	1.00	1	1	-0.01	-0.011	-0.011	-0.01	-0.01	t	49377	PSGLVIP	Mus musculu s
1	1.00	1	1	0	-0.002	-0.001	0	0	t	48401	PLLPIF	Mus musculu s
1	1.00	1	1	0.02	0.018	0.022	0.023	0.02	t	75151	YNGEEYLIL S	Mus musculu s
1	1.00	1	1	0.02	0.018	0.013	0.02	0.019	t	120108	NFMESGE WVIKESRG W	Oryctola gus cuniculu s
1	1.00	1	1	0.02	0.02	0.013	0.02	0.019	t	120108	NFMESGE WVIKESRG W	Rattus norvegic us
1	1.00	1	1	0.02	0.02	0.013	0.02	0.019	t	120108	NFMESGE WVIKESRG W	Mus musculu s
1	1.00	1	1	0.03	0.032	0.028	0.027	0.028	t	14638	EVDDEALE KF	Homo sapiens
1	1.00	1	1	0.02	0.02	0.015	0.02	0.018	t	59873	SNNVQIVR QQSYSI	Mus musculu s
1	1.00	1	1	-0.01	-0.006	-0.003	-0.007	-0.01	t	30710	KFDRGYIS PY	Homo sapiens
1	1.00	1	1	-0.02	-0.009	-0.026	-0.02	-0.02	t	41126	MASLIYRQ LL	Gallus gallus
1	1.00	1	1	0.03	0.03	0.025	0.03	0.03	t	35172	LDGRPLST TQ	Homo sapiens

1	1.00	1	1	0.05	0.05	0.035	0.047	0.05	t	44339	NISGHCSD CTFLGEE	Homo sapiens
1	1.00	1	1	0	-0.001	0	0.003	-0.002	t	130612	SPNTAHIT SKSL	Camelus dromed arius
1	1.00	1	1	-0.01	-0.004	-0.003	-0.003	-0.012	t	127727	LQEGLRRA	Homo sapiens
1	1.00	1	1	0	0	-0.005	0	-0.002	t	51439	QLLTAIDL ADNT	Oryctola gus cuniculu s
1	1.00	1	1	0.01	0.014	0.01	0.007	0.009	t	74687	YLGYLEQL LR	Homo sapiens
1	1.00	1	1	-0.02	-0.02	-0.025	-0.02	-0.021	t	107219	PAGGPGF PG	Rattus norvegic us
1	1.00	1	1	0.02	0.02	0.01	0.02	0.02	t	32787	KPLEDKILV Q	Mus musculu s
1	1.00	1	1	-0.03	-0.03	-0.02	-0.023	-0.032	t	14013	ERKRR	Homo sapiens
1	1.00	1	1	0.03	0.03	0.03	0.034	0.031	t	108360	FEDMAKS DKARYDRE	Homo sapiens
1	1.00	1	1	-0.02	-0.018	-0.027	-0.019	-0.02	t	44911	NLVDWLR QY	Homo sapiens
1	1.00	1	1	-0.01	-0.01	-0.014	-0.01	-0.01	t	106349	DPLRPQRE GC	Oryctola gus cuniculu s
1	1.00	1	1	-0.03	-0.028	-0.039	-0.022	-0.027	t	110965	PNNNKILV PKVSGLQY RVFR	Homo sapiens
1	1.00	1	1	0.05	0.05	0.041	0.048	0.05	t	135974	PSNSASVP ALTA	Homo sapiens
1	1.00	1	1	0.01	0.008	-0.01	0.003	0.01	t	128010	GRWGSQR AVNVSGG FQF	Mus musculu s

1	1.00	1	1	0.02	0.02	0.02	0.02	0.02	t	11583	EEEIAQVA	Homo sapiens
1	1.00	1	1	0	0.002	-0.005	0.003	0	t	106634	LVGPRGERGFP	Homo sapiens
1	1.00	1	1	-0.02	-0.017	-0.03	-0.02	-0.021	t	64899	TLLDKNKKIEE	Mus musculus
1	1.00	1	1	0.02	0.018	0.018	0.023	0.021	t	99637	RRAQSVVD	Mus musculus
1	1.00	1	1	0.01	0.012	0.01	0.013	0.009	t	96455	KGKNVLINKD	Homo sapiens
1	1.00	1	1	0.03	0.028	0.023	0.027	0.031	t	42042	MLQGVD	Mus musculus
1	1.00	1	1	0.04	0.042	0.04	0.039	0.037	t	134166	MPKFYCDYCDTYLTHDS	Homo sapiens
1	1.00	1	1	0	0	0	0.006	0	t	118324	EIIKRTLKIPAMT	Homo sapiens
1	1.00	1	1	0	0	0	0.006	0	t	118440	IVLGGGCALLRCI	Homo sapiens
1	1.00	1	1	0	0	0	0.006	0	t	118510	LVLNRLKVGLQVV	Homo sapiens
1	1.00	1	1	0	0	0	0.006	0	t	118451	KGIIDPTKVVRTA	Homo sapiens
1	1.00	1	1	0	0	0	0.006	0	t	118491	LKVGLQVVAVKAP	Homo sapiens
1	1.00	1	1	0	0	0	0.006	0	t	118275	ATVLARSIAKEGF	Homo sapiens
1	1.00	1	1	0	0	0	0.006	0	t	118443	KAPGFGDNRKNQL	Homo sapiens
1	1.00	1	1	0	0	0	0.006	0	t	118540	NPVEIRRGVMLAV	Homo sapiens
1	1.00	1	1	0	0	0	0.006	0	t	118487	LEIANAHRKPLVI	Homo sapiens
1	1.00	1	1	0	0	0	0.006	0	t	118459	KKVGRKGVITVKD	Homo sapiens
1	1.00	1	1	0	0	0	0.006	0	t	118490	LKDKYKNIGAKLV	Homo sapiens
1	1.00	1	1	0	0	0	0.006	0	t	118313	EALSTLVLNRLKV	Homo sapiens
1	1.00	1	1	0	0	0	0.006	0	t	118430	ISDAMKKVGRKGV	Homo sapiens
1	1.00	1	1	0	0	0	0.006	0	t	118568	QKIGIEIIKRTLK	Homo sapiens

1	1.00	1	1	0	0	0	0.006	0	t	118284	DAYVLLSE KKISS	Homo sapiens
1	1.00	1	1	0	0	0	0.006	0	t	94481	CSNNPTC WAICK	Homo sapiens
1	1.00	1	1	0	0	0	0.006	0	t	94651	PTCWAICK RIPN	Homo sapiens
1	1.00	1	1	0	0	0	0.006	0	t	66933	TVAEEHVE EPTVAEE	Homo sapiens
1	1.00	1	1	0	0	0	0.006	0	t	94613	NNPTCWA ICKRI	Homo sapiens
1	1.00	1	1	0	0	0	0.006	0	t	94695	SNNPTCW AICKR	Homo sapiens
1	1.00	1	1	0	0	0	0.006	0	t	48681	PNNDFHF EVFNF	Homo sapiens
1	1.00	1	1	0	0	0	0.006	0	t	54214	RIPNKKPG KKTT	Homo sapiens
1	1.00	1	1	0	0	0	0.006	0	t	45054	NNDFHFE VFNFV	Homo sapiens
1	1.00	1	1	0	0	0	0.006	0	t	42416	MQTLWDE IMDINKRK	Homo sapiens
1	1.00	1	1	0	0	0	0.006	0	t	43242	NANPNAN PNANP	Homo sapiens
1	1.00	1	1	0	0	0	0.006	0	t	51729	QNKPPNK PNNDF	Homo sapiens
1	1.00	1	1	0	0	0	0.006	0	t	44571	NKPPNKP NNDFH	Homo sapiens
1	1.00	1	1	0	0	0	0.006	0	t	46895	PAPQAPY QGYQEPP APQAPY	Homo sapiens
1	1.00	1	1	0	0	0	0.006	0	t	44569	NKPNNDF HFEVF	Homo sapiens
1	1.00	1	1	0	0	0	0.006	0	t	49137	PQTTKPKE VPTT	Homo sapiens

1	1.00	1	1	0	0	0	0.006	0	t	16103	FHFEVFNF VPCS	Homo sapiens
1	1.00	1	1	0	0	0	0.006	0	t	29965	KATYEAAV AANNAKN AALT	Homo sapiens(human)
1	1.00	1	1	0	0	0	0.006	0	t	18588	GAGGGAG GAGAGGG AGGAGA	Homo sapiens
1	1.00	1	1	0	0	0	0.006	0	t	23770	HFEVFNFV PCSI	Homo sapiens
1	1.00	1	1	0	0	0	0.006	0	t	27463	IMDINKRK YDSLK	Homo sapiens
1	1.00	1	1	0	0	0	0.006	0	t	32809	KPNNDFH FEVFN	Homo sapiens
1	1.00	1	1	0	0	0	0.006	0	t	32827	KPPNKPN NDFHF	Homo sapiens
1	1.00	1	1	0	0	0	0.006	0	t	1433	AGAGGGA GGAGAGG GAGGAG	Homo sapiens
1	1.00	1	1	0	0	0	0.006	0	t	1433	AGAGGGA GGAGAGG GAGGAG	Homo sapiens
1	1.00	1	1	0	0	0	0.006	0	t	8275	DFHFEVFN FVPC	Homo sapiens
1	1.00	1	1	0.07	0.07	0.063	0.07	0.07	t	119480	KQQKTEN GAGDQ	Homo sapiens
1	1.00	1	1	-0.02	-0.018	-0.027	-0.023	-0.022	t	95999	WDLNAAS AYC	Homo sapiens
1	1.00	1	1	0.02	0.018	0.013	0.019	0.021	t	67325	TYEAALKQ YEADL	Mus musculu s
1	1.00	1	1	0	-0.004	-0.008	-0.001	0	t	97963	DVYYAGL NYK	Oryctola gus cuniculu s
1	1.00	1	1	0	-0.002	-0.008	-0.001	0	t	97963	DVYYAGL NYK	Mus musculu s
1	1.00	1	1	0.04	0.042	0.03	0.037	0.04	t	159145	TDAETKAF LKAGDAD	Homo sapiens

1	1.00	1	1	-0.03	-0.026	-0.032	-0.033	-0.03	t	139943	VPTWYRA	Ovis aries
1	1.00	1	1	-0.01	-0.01	-0.017	-0.01	-0.01	t	108998	FVCGDRG FYFNK	Mus musculu s
1	1.00	1	1	-0.01	-0.01	-0.02	-0.004	-0.008	t	52184	QRAKEALD KYELENH	Homo sapiens
1	1.00	1	1	-0.02	-0.013	-0.019	-0.021	-0.02	t	55193	RPKPG	Gallus gallus
1	1.00	1	1	0.04	0.041	0.039	0.043	0.04	t	99096	AGQKQILL DAIDKIA	Equus caballus
1	1.00	1	1	0.03	0.032	0.019	0.031	0.032	t	36042	LGAALLQS	Homo sapiens
1	1.00	1	1	0.06	0.06	0.06	0.06	0.06	t	104181	PPSLASSS ASQLSAPS LKA	Homo sapiens
1	1.00	1	1	0.03	0.032	0.026	0.027	0.028	t	58038	SGFTPEFLE QAFQVD	Homo sapiens
1	1.00	1	1	-0.02	-0.02	-0.025	-0.024	-0.02	t	123442	LLKDLRKN	Homo sapiens
1	1.00	1	1	0.04	0.038	0.053	0.043	0.039	t	138862	KFPED	Mus musculu s
1	1.00	1	1	0.02	0.02	0.013	0.019	0.019	t	137828	DVLLAGN AEADR	Homo sapiens
1	1.00	1	1	0	-0.003	0	0.006	0	t	118970	PPPGRRP	Oryctola gus cuniculu s
1	1.00	1	1	0	0.012	0	0.006	0	t	109030	GAQGPRG E	Rattus norvegic us
1	1.00	1	1	0	0.012	0	0.006	0	t	108790	AQGPRGE P	Rattus norvegic us
1	1.00	1	1	-0.01	-0.011	-0.007	-0.01	-0.01	t	39697	LSTGLIHL	Homo sapiens

1	1.00	1	1	0.02	0.018	0.022	0.019	0.02	t	79011	LDPAFG	Mus musculus
1	1.00	1	1	0	-0.001	-0.003	-0.001	0	t	38273	LNLTTWNPTLLG	Bos taurus
1	1.00	1	1	-0.05	-0.05	-0.05	-0.05	-0.052	t	25086	HVSGHRMAW	Homo sapiens
1	1.00	1	1	0.06	0.06	0.04	0.053	0.062	t	14006	ERKDRVED	Homo sapiens
1	1.00	1	1	0.04	0.042	0.034	0.043	0.042	t	40077	LTPCDN	Homo sapiens
1	1.00	1	1	-0.01	-0.01	-0.008	-0.01	-0.012	t	79760	ASSPDAHVPFCF	Mus musculus
1	1.00	1	1	0.05	0.046	0.044	0.047	0.05	t	48606	PNATQPEL	Oryctolagus cuniculus
1	1.00	1	1	0	0.007	0	0.001	0	t	66105	TRTFTSL	Homo sapiens
1	1.00	1	1	0.03	0.028	0.03	0.029	0.03	t	29758	KAAQIYSEP	Mus musculus
1	1.00	1	1	0.03	0.028	0.03	0.029	0.03	t	29758	KAAQIYSEP	Mus musculus
1	1.00	1	1	-0.01	-0.012	-0.002	-0.011	-0.012	t	74674	YLGLTPM	Mus musculus
1	1.00	1	1	0.01	0.01	0.007	0.014	0.01	t	51263	QKTYMVVDVT	Homo sapiens
1	1.00	1	1	-0.01	-0.014	-0.017	-0.011	-0.01	t	35312	LDTSNKFA	Oryctolagus cuniculus
1	1.00	1	1	0.01	0.01	0.002	0.01	0.012	t	43212	NALSFTNK	Mus musculus

1	1.00	1	1	0.02	0.02	0.02	0.02	0.022	t	136089	VPPTPQP	Oryctolagus cuniculus
1	1.00	1	1	0	-0.003	0.007	0	-0.003	t	49764	PTRTIGGSQA	Oryctolagus cuniculus
1	1.00	1	1	-0.01	-0.01	-0.021	-0.016	-0.01	t	53152	RALLGLAVCLFLMALCT	Homo sapiens
1	1.00	1	1	0.01	0.013	0.008	0.01	0.01	t	12539	EIPALTAVE	Mus musculus
1	1.00	1	1	-0.05	-0.076	-0.054	-0.053	-0.052	t	106524	HWR	Homo sapiens
1	1.00	1	1	-0.05	-0.048	-0.054	-0.053	-0.052	t	106524	HWR	Homo sapiens
1	1.00	1	1	0.05	0.053	0.052	0.05	0.049	t	73601	YDVPDYAS	Mus musculus
1	1.00	1	1	-0.01	-0.01	-0.01	-0.01	-0.014	t	130872	GSFLTKGPSKLNDRA	Mus musculus
1	1.00	1	1	0.02	0.02	0.018	0.02	0.019	t	144791	STIDYFQPNNKR	Sus scrofa
1	1.00	1	1	0	-0.002	-0.005	-0.001	0.001	t	141567	TQDGLYHNKN	Mus musculus
1	1.00	1	1	0.01	0.01	0.005	0.01	0.01	t	23184	GVTTSLS	Homo sapiens
1	1.00	1	1	0	-0.002	-0.002	-0.001	0	t	89292	SYHAGFNYKNGG	Mus musculus
1	1.00	1	1	0	0	-0.002	-0.001	0	t	89292	SYHAGFNYKNGG	Homo sapiens
1	1.00	1	1	0.03	0.03	0.03	0.03	0.03	t	125880	EEMTMQQAK	Homo sapiens

1	1.00	1	1	0	0	-0.007	-0.001	-0.001	t	137892	HGHGAFR QQFQY	Homo sapiens
1	1.00	1	1	-0.02	-0.02	-0.02	-0.011	-0.019	t	135642	CKNKEKKC	Homo sapiens
1	1.00	1	1	-0.04	-0.034	-0.058	-0.04	-0.038	t	92565	EKVTIQN WFR	Ovis aries
1	1.00	1	1	0	-0.002	-0.005	0.001	0	t	17128	FNASDSVG QQIKVIP	Mus musculu s
1	1.00	1	1	0.03	0.028	0.009	0.027	0.032	t	153829	ERSVQKLQ KEVDRLED E	Homo sapiens
1	1.00	1	1	0	0.002	-0.005	-0.003	0	t	17964	FTGIQIICL A	Homo sapiens
1	1.00	1	1	0.01	0.01	0.005	0.017	0.01	t	118220	WKFLEHK GPVFAPPY EPLPE	Homo sapiens
1	1.00	1	1	-0.01	-0.01	-0.022	-0.012	-0.01	t	96453	KFKEAFLR SV	Homo sapiens(human)
1	1.00	1	1	0.01	0.008	0.017	0.011	0.01	t	78923	AFGANS	Mus musculu s
1	1.00	1	1	0.02	0.018	0.019	0.02	0.019	t	95146	SESPNK	Oryctola gus cuniculu s
1	1.00	1	1	0	0.002	0	-0.003	-0.002	t	59922	SNTFINNA	Homo sapiens
1	1.00	1	1	0.04	0.042	0.041	0.041	0.039	t	56386	RVQYEG	Homo sapiens
1	1.00	1	1	-0.01	-0.01	-0.01	-0.01	-0.013	t	72249	WAP	Mus musculu s
1	1.00	1	1	0	0.003	-0.018	0	0.002	t	41537	MFSGGGG PLSPGGKS	Mus musculu s

1	1.00	1	1	0.02	0.02	0.02	0.02	0.017	t	131032	RDEIESVKELKDTGKDAVN	Oryctolagus cuniculus
1	1.00	1	1	-0.01	-0.012	-0.01	-0.011	-0.014	t	46488	NVSVPSPSSTPLLY	Mus musculus
1	1.00	1	1	0.03	0.03	0.023	0.036	0.032	t	106990	VYATRSSAVRLRSSVP	Homo sapiens
1	1.00	1	1	0	-0.004	-0.005	-0.003	-0.002	t	130537	LCENIANHLK	Oryctolagus cuniculus
1	1.00	1	1	0	-0.002	-0.005	-0.003	-0.002	t	130537	LCENIANHLK	Rattus norvegicus
1	1.00	1	1	0	0.003	-0.006	0	0	t	53587	REMVIITFKS	Mus musculus
1	1.00	1	1	0	0	0	0.004	-0.001	t	151101	AAA	Homo sapiens
1	1.00	1	1	0	0	0	0.004	-0.001	t	151101	AAA	Homo sapiens
1	1.00	1	1	0	0	0	0.004	-0.001	t	131504	KATLVCLISDFYPGAV	Homo sapiens
1	1.00	1	1	0	0	0	0.004	-0.001	t	122892	RSILPYGDSMDRIE	Homo sapiens
1	1.00	1	1	0	0	0	0.004	-0.001	t	121542	LFKPPKYHPDVPYVKR	Homo sapiens
1	1.00	1	1	0	0	0	0.004	-0.001	t	117742	AKAQTDREDLRTLLRY	Homo sapiens
1	1.00	1	1	0	0	0	0.004	-0.001	t	131628	RGYDSYA	Homo sapiens
1	1.00	1	1	0	0	0	0.004	-0.001	t	117773	CNSRQTDREDELI	Homo sapiens
1	1.00	1	1	0	0	0	0.004	-0.001	t	131457	GSHTRGD	Homo sapiens

1	1.00	1	1	0	0	0	0.004	-0.001	t	131657	SGLQAEDEADYYCSSY	Homo sapiens
1	1.00	1	1	0	0	0	0.004	-0.001	t	108834	CKPISGHNSLFWYRQT	Homo sapiens
1	1.00	1	1	-0.01	-0.008	-0.01	-0.009	-0.01	t	64659	TKRNTNR	Homo sapiens
1	1.00	1	1	0.06	0.06	0.051	0.06	0.06	t	135951	LLESQIATIEQT	Homo sapiens
1	1.00	1	1	-0.01	-0.016	-0.012	-0.01	-0.013	t	57119	SCMVCVDRSSLSSW	Oryctolagus cuniculus
1	1.00	1	1	-0.05	-0.046	-0.062	-0.049	-0.05	t	9804	DPVIDRLYLSSHRGV	Homo sapiens
1	1.00	1	1	0	-0.002	-0.003	0.003	0.002	t	45573	NQLAGRMIP	Anas platyrhynchos
1	1.00	1	1	0.02	0.02	0.013	0.02	0.02	t	78630	DYGEILTEKVEDLYKGVLL	Mus musculus
1	1.00	1	1	0.03	0.03	0.019	0.03	0.032	t	111772	SGFSITTSTDTTPA	Oryctolagus cuniculus
1	1.00	1	1	-0.02	-0.02	-0.02	-0.014	-0.02	t	118275	ATVLARSIAKEGF	Homo sapiens
1	1.00	1	1	-0.05	-0.057	-0.041	-0.043	-0.05	t	108867	DKSRWQE	Homo sapiens
1	1.00	1	1	-0.04	-0.042	-0.052	-0.043	-0.04	t	95564	LPMHIR	Homo sapiens
1	1.00	1	1	-0.04	-0.04	-0.04	-0.04	-0.041	t	119607	KTHTCPPC	Homo sapiens
1	1.00	1	1	-0.04	-0.037	-0.04	-0.04	-0.04	t	134017	CFHQGKEYAPG	Mus musculus
1	1.00	1	1	0.01	0.012	0.005	0.013	0.01	t	159048	PKARELTDAETKAFL	Homo sapiens

1	1.00	1	1	0	0	0	0.004	0.002	t	121713	PLTALLRN	Homo sapiens
1	1.00	1	1	0.04	0.04	0.04	0.04	0.04	t	135959	LTRAYAKD VKFG	Homo sapiens
1	1.00	1	1	0.03	0.03	0.025	0.023	0.028	t	142383	AEQIQAEL QKVKTA	Homo sapiens
1	1.00	1	1	-0.02	-0.02	-0.02	-0.014	-0.02	t	158775	FNFKTFFH TIGFASK	Homo sapiens
1	1.00	1	1	-0.01	-0.012	-0.008	-0.007	-0.011	t	25180	HYLVQSPP W	Mus musculu s
1	1.00	1	1	-0.01	-0.012	-0.01	-0.013	-0.011	t	96526	LAANNPA	Mus musculu s
1	1.00	1	1	0.01	0.013	0.007	0.01	0.01	t	46235	NTQIYTIN DK	Mus musculu s
1	1.00	1	1	0	0.002	-0.002	0.001	0.001	t	23685	HEGKAYFP REGV	Homo sapiens
1	1.00	1	1	0.01	-0.003	0.005	0.01	0.011	t	19096	GDRADGQ PA	Aotus griseime mbra
1	1.00	1	1	0.02	0.026	0.008	0.021	0.02	t	48643	PNGRASD TAGQ	Equus caballus
1	1.00	1	1	0.02	0.02	0.026	0.02	0.02	t	34238	KVVLPE	Homo sapiens
1	1.00	1	1	-0.01	-0.012	-0.019	-0.011	-0.01	t	133590	GMVDGW YG	Mus musculu s
1	1.00	1	1	-0.01	-0.01	-0.008	-0.01	-0.012	t	79755	AMKPYEV TPTRM	Mus musculu s
1	1.00	1	1	0.06	0.06	0.06	0.061	0.06	t	78748	MISGPCPK DGQPSLVL QSSS	Homo sapiens

1	1.00	1	1	-0.04	-0.038	-0.048	-0.039	-0.04	t	12531	EINHVQDHALPKENI	Homo sapiens
1	1.00	1	1	0.01	0.012	0.008	0.011	0.006	t	9511	DNNDSGHSTGADTG	Homo sapiens
1	1.00	1	1	-0.01	-0.007	-0.014	-0.01	-0.008	t	39085	LRFGSK	Mus musculus
1	1.00	1	1	0.02	0.013	0.013	0.027	0.022	t	136153	CLEEKVCSLLEPLGLQCTFI	Homo sapiens
1	1.00	1	1	0.01	0.01	0.004	0.01	0.01	t	22063	GRGYNVSSIVTMTSQ	Homo sapiens
1	1.00	1	1	0.02	0.027	0.027	0.021	0.018	t	136302	GALIYTPEKYTI	Gallus gallus
1	1.00	1	1	0.01	0.013	0.011	0.01	0.009	t	59757	SMSYTWTGA	Mus musculus
1	1.00	1	1	0.02	0.016	0.013	0.017	0.019	t	135445	AKAAQFG	Oryctolagus cuniculus
1	1.00	1	1	-0.01	-0.01	-0.01	-0.014	-0.01	t	139511	GPM	Homo sapiens
1	1.00	1	1	-0.03	-0.03	-0.03	-0.03	-0.03	t	55382	RPYCWHYPP	Homo sapiens
1	1.00	1	1	-0.02	-0.02	-0.027	-0.01	-0.018	t	106990	VYATRSSAVRLRSSVP	Homo sapiens
1	1.00	1	1	0.04	0.046	0.043	0.04	0.04	t	141046	KEVTVVTTS	Ovis aries
1	1.00	1	1	-0.01	-0.01	-0.017	-0.01	-0.01	t	122900	SEVATEVPFRLMH	Rattus norvegicus
1	1.00	1	1	-0.03	-0.025	-0.043	-0.033	-0.032	t	157808	QQDRHQKIRHFR	Homo sapiens

1	1.00	1	1	-0.01	-0.01	-0.013	-0.01	-0.01	t	60914	SSATK	Homo sapiens
1	1.00	1	1	0.03	0.028	0.033	0.033	0.028	t	33222	KRTISIWGT	Mus musculus
1	1.00	1	1	0.03	0.033	0.03	0.03	0.031	t	14951	EWSPCSVTCGK	Mus musculus
1	1.00	1	1	0.05	0.053	0.053	0.05	0.048	t	139732	ERETQKAKG	Mus musculus
1	1.00	1	1	0	-0.004	-0.003	0.001	0	t	22718	GTK	Bos taurus
1	1.00	1	1	0.04	0.04	0.04	0.04	0.039	t	107107	EAIQP	Rattus norvegicus
1	1.00	1	1	0.02	0.02	0.02	0.02	0.019	t	105566	VFFAE	Mus musculus
1	1.00	1	1	0.02	0.02	0.02	0.02	0.02	t	123091	DLPNLPYYI	Ovis aries
1	1.00	1	1	0.01	0.008	0.006	0.009	0.009	t	59757	SMSYTWTGA	Mus musculus
1	1.00	1	1	0.02	0.022	0.004	0.026	0.026	t	134684	GSLPQKSQRDEN	Rattus norvegicus
1	1.00	1	1	-0.01	-0.01	-0.01	-0.01	-0.011	t	60795	SRSERR	Homo sapiens
1	1.00	1	1	-0.04	-0.04	-0.04	-0.04	-0.042	t	101716	HQPTPAA	Mus musculus
1	1.00	1	1	0	0	0	0	0	t	47189	PDREVLYRE	Homo sapiens
1	1.00	1	1	-0.01	-0.014	-0.019	-0.007	-0.01	t	71653	VVERLGDYLLEQGM	Oryctolagus cuniculus
1	1.00	1	1	0.04	0.042	0.032	0.041	0.04	t	7383	CYGVSATKLNDLCFSNV	Homo sapiens

1	1.00	1	1	0.04	0.042	0.032	0.041	0.04	t	7383	CYGVSATKLNDLCFSNV	Homo sapiens
1	1.00	1	1	-0.04	-0.036	-0.04	-0.043	-0.043	t	115574	YWLAHKALCS	Homo sapiens
1	1.00	1	1	0.01	0.01	0.01	0.01	0.009	t	99209	ELFTNR	Oryctolagus cuniculus
1	1.00	1	1	0.01	0.012	0.003	0.007	0.007	t	141640	ETTKQVQRYRARLSP	Homo sapiens
1	1.00	1	1	0.06	0.063	0.062	0.06	0.058	t	121024	VDEVNQIVET	Mus musculus
1	1.00	1	1	0	0	0	0	0	t	78825	SFPPPSPGPSCPT	Homo sapiens
1	1.00	1	1	0	-0.004	-0.004	0.003	-0.001	t	108327	DTYDALHMQTLAPR	Oryctolagus cuniculus
1	1.00	1	1	0.07	0.07	0.07	0.07	0.07	t	138854	GAGAGAGA	Mus musculus
1	1.00	1	1	0.01	0.01	0.009	0.01	0.012	t	105828	GKDLESIK	Oryctolagus cuniculus
1	1.00	1	1	-0.02	-0.023	-0.026	-0.02	-0.022	t	141580	YIGVAPRKAIPA	Oryctolagus cuniculus
1	1.00	1	1	0.01	0.014	0.015	0.013	0.009	t	115608	GRTELLKDAIGE	Homo sapiens
1	1.00	1	1	0.01	0.024	0.015	0.013	0.009	t	115608	GRTELLKDAIGE	Homo sapiens
1	1.00	1	1	0.03	0.03	0.02	0.03	0.03	t	57542	SELSQLS	Mus musculus
1	1.00	1	1	0.03	0.03	0.02	0.03	0.03	t	57542	SELSQLS	Mus musculus

1	1.00	1	1	0.02	0.018	0.02	0.019	0.02	t	46424	NVLQNFS VF	Aotus sp.
1	1.00	1	1	-0.04	-0.038	-0.04	-0.033	-0.042	t	134763	PSLGASSH QHSRRRQ	Homo sapiens
1	1.00	1	1	0.01	0.01	0.01	0.01	0.01	t	30333	KEDVPSER YL	Homo sapiens
1	1.00	1	1	0	0.003	0.002	0	-0.001	t	120014	LAAQGAR VYA	Mus musculu s
1	1.00	1	1	0.03	0.03	0.03	0.03	0.03	t	78403	GLQNDF	Mus musculu s
1	1.00	1	1	0.01	0.013	0.01	0.01	0.01	t	41233	MDIDPYKE FGA	Mus musculu s
1	1.00	1	1	0.02	0.02	0.02	0.024	0.02	t	115626	LKDAIGEG KVTL	Homo sapiens
1	1.00	1	1	0.03	0.032	0.016	0.027	0.03	t	138844	EVVETDGK KKECSS	Homo sapiens
1	1.00	1	1	0.04	0.042	0.04	0.043	0.039	t	117948	KFVETPGQ KYKETDL	Homo sapiens
1	1.00	1	1	0.03	0.03	0.028	0.03	0.028	t	99935	DFSQTATL TNTN	Oryctola gus cuniculu s
1	1.00	1	1	0	0.006	-0.005	0	0	t	35282	LDRLVESA KREMAQK LA	Equus caballus
1	1.00	1	1	0.1	0.1	0.1	0.1	0.1	t	116005	TTEAVEST VA	Homo sapiens
1	1.00	1	1	-0.02	-0.018	-0.02	-0.017	-0.02	t	40724	LYCGGPM FNSKGAQ	Homo sapiens
1	1.00	1	1	0.01	0.01	0.003	0.01	0.011	t	36406	LHNNEAG RTTVFS	Mus musculu s
1	1.00	1	1	0.04	0.038	0.035	0.037	0.04	t	70812	VRRIEGGE	Anas platyrhy nchos

1	1.00	1	1	0.01	0.013	0.005	0.01	0.01	t	120527	FEYMNKLE KG	Mus musculu s
1	1.00	1	1	0.05	0.052	0.052	0.057	0.05	t	122551	RGQYLQL QELECDW EEVTTS	Homo sapiens
1	1.00	1	1	-0.03	-0.028	-0.03	-0.023	-0.03	t	131075	SQSYPEPA RGSVPMP	Homo sapiens
1	1.00	1	1	0	0	0	0	0	t	120862	PDVKSAIE GV	Rattus norvegic us
1	1.00	1	1	0.02	0.021	0.019	0.023	0.019	t	99341	ILSVDCTSE EMNAFL	Equus caballus
1	1.00	1	1	0	0	0	0	-0.003	t	142913	MVLNEGT SGTA	Oryctola gus cuniculu s
1	1.00	1	1	0	0	0	0	-0.003	t	134767	QDPYVKE AENLKKYF NAG	Mus musculu s
1	1.00	1	1	0	0	0	0	-0.003	t	134654	DQQNQFQ KAET	Mus musculu s
1	1.00	1	1	0	0	0	0	-0.003	t	134699	KDRHDFG F	Mus musculu s
1	1.00	1	1	0	0	0	0	-0.003	t	139029	DLQVLT	Mus musculu s
1	1.00	1	1	0	0	0	0	-0.003	t	134702	KEKKYS	Mus musculu s
1	1.00	1	1	0	0	0	0	-0.003	t	146337	IPEEIKQL	Mus musculu s
1	1.00	1	1	0	0	0	0	-0.003	t	151713	RGEARK	Homo sapiens
1	1.00	1	1	0	0	0	0	-0.003	t	140459	EHWSHD MFSPGD	Mus musculu s

1	1.00	1	1	0	0	0	0	-0.003	t	134148	LQSDQEEIDY	Oryctolagus cuniculus
1	1.00	1	1	0	0	0	0	-0.003	t	136353	HGNAEVHASFFDIGGS	Oryctolagus cuniculus
1	1.00	1	1	0	0	0	0	-0.003	t	134804	TIFILFCCSKEKRKKKQAAT	Mus musculus
1	1.00	1	1	0	0	0	0	-0.003	t	150200	FGEHKQEKDLEYGAC	Mus musculus
1	1.00	1	1	0	0	0	0	-0.003	t	137432	NHNYPPLSLLTF	Mus musculus
1	1.00	1	1	0	0	0	0	-0.003	t	140460	EKLKHSM	Mus musculus
1	1.00	1	1	0	0	0	0	-0.003	t	134669	FGFPQE	Mus musculus
1	1.00	1	1	0	0	0	0	-0.003	t	134770	QIFNLFSTKDSSAA	Mus musculus
1	1.00	1	1	0	0	0	0	-0.003	t	138162	EKKYSPCAWEVVRAE	Mus musculus
1	1.00	1	1	0	0	0	0	-0.003	t	134037	DYDDTIS	Oryctolagus cuniculus
1	1.00	1	1	0	0	0	0	-0.003	t	118877	LDVEQLGIPEQEYSC	Mus musculus
1	1.00	1	1	0	0	0	0	-0.003	t	128955	ILFSVFD	MD
1	1.00	1	1	0	0	0	0	-0.003	t	133611	IVSYLCDLAPEAPPPTLPP	Mus musculus
1	1.00	1	1	0	0	0	0	-0.003	t	130757	CQEPGGLVVPPTDAP	Mus musculus
1	1.00	1	1	0	0	0	0	-0.003	t	130518	ITQSTQSFNDFTRVV	Mus musculus

1	1.00	1	1	0	0	0	0	-0.003	t	122780	GQELKNPQEETLQAFDSHYD	Oryctolagus cuniculus
1	1.00	1	1	0	0	0	0	-0.003	t	130605	SCRLFGKTYLCH	Mus musculus
1	1.00	1	1	0	0	0	0	-0.003	t	119187	GNAEVHASFFDIGG	Oryctolagus cuniculus
1	1.00	1	1	0	0	0	0	-0.003	t	130583	QCQTFGKTMLCT	Mus musculus
1	1.00	1	1	0	0	0	0	-0.003	t	134028	DDTIS	Mus musculus
1	1.00	1	1	0	0	0	0	-0.003	t	131100	TDSEMDVVRFD	Mus musculus
1	1.00	1	1	0	0	0	0	-0.003	t	130473	ECIVYGKTALCT	Mus musculus
1	1.00	1	1	0	0	0	0	-0.003	t	125610	YYVFFEEQEDEIIGFGQELK	Oryctolagus cuniculus
1	1.00	1	1	0	0	0	0	-0.003	t	120109	NIHPKAPIWGIITSNF	Rattus norvegicus
1	1.00	1	1	0	0	0	0	-0.003	t	131967	LRLDCRHEN	Mus musculus
1	1.00	1	1	0	0	0	0	-0.003	t	125441	HPLTCDDPRFQ	Oryctolagus cuniculus
1	1.00	1	1	0	0	0	0	-0.003	t	133707	STRKEEKQRNGT	Mus musculus
1	1.00	1	1	0	0	0	0	-0.003	t	125127	LDPENLTL	Mus musculus
1	1.00	1	1	0	0	0	0	-0.003	t	134017	CFHQGKEYAPG	Mus musculus
1	1.00	1	1	0	0	0	0	-0.003	t	129973	SPRSFQKKT	Oryctolagus cuniculus

1	1.00	1	1	0	0	0	0	-0.003	t	124713	SGEQPSDEQPSGEHG	Mus musculus
1	1.00	1	1	0	0	0	0	-0.003	t	125004	DAPDTDWCDSIP	Mus musculus
1	1.00	1	1	0	0	0	0	-0.003	t	131128	VEMKKEDFDIYDEDE	Mus musculus
1	1.00	1	1	0	0	0	0	-0.003	t	130787	EDFDIYDEDE	Mus musculus
1	1.00	1	1	0	0	0	0	-0.003	t	125015	DNESSYSRGFDMTYTE	Mus musculus
1	1.00	1	1	0	0	0	0	-0.003	t	125021	DWC	Mus musculus
1	1.00	1	1	0	0	0	0	-0.003	t	124925	YYVFFEEQEDEIIGF	Oryctolagus cuniculus
1	1.00	1	1	0	0	0	0	-0.003	t	126201	GYKEKSKFQDTHNNA	Oryctolagus cuniculus
1	1.00	1	1	0	0	0	0	-0.003	t	124588	PQEETLQAFDSHYDYT	Oryctolagus cuniculus
1	1.00	1	1	0	-0.003	0	0	-0.003	t	131128	VEMKKEDFDIYDEDE	Oryctolagus cuniculus
1	1.00	1	1	0	0	0	0	-0.003	t	130912	KADILPEMNSMRADRM	Mus musculus
1	1.00	1	1	0	0	0	0	-0.003	t	125415	FSPDGKPCTPPALNCYW	Mus musculus
1	1.00	1	1	0	0	0	0	-0.003	t	133551	EAPPPTLPPDMAQVTVGPG	Mus musculus
1	1.00	1	1	0	0	0	0	-0.003	t	129978	SQFIIMY	MD
1	1.00	1	1	0	0	0	0	-0.003	t	130916	KEDFDIYDEDE	Mus musculus

1	1.00	1	1	0	0	0	0	-0.003	t	119538	VEDGECYIQF	Oryctolagus cuniculus
1	1.00	1	1	0	0	0	0	-0.003	t	122842	LLDLPRDLGGMGCSSPPCE	Oryctolagus cuniculus
1	1.00	1	1	0	0	0	0	-0.003	t	123849	WQDE	Mus musculus
1	1.00	1	1	0	0	0	0	-0.003	t	131217	EDISAYLL	Mus musculus
1	1.00	1	1	0	0	0	0	-0.003	t	130590	RCKTFGKTTLCS	Mus musculus
1	1.00	1	1	0	0	0	0	-0.003	t	119322	QRVFKEKVDTKAPEPP	Mus musculus
1	1.00	1	1	0	0	0	0	-0.003	t	130610	SMCMHGGAYCFP	Mus musculus
1	1.00	1	1	0	0	0	0	-0.003	t	113365	EHPVTGCGERTEGRCLHYTV	Mus musculus
1	1.00	1	1	0	0	0	0	-0.003	t	110733	VGENVPDDLYIKGSG	Mus musculus
1	1.00	1	1	0	0	0	0	-0.003	t	113365	EHPVTGCGERTEGRCLHYTV	Mus musculus
1	1.00	1	1	0	0	0	0	-0.003	t	117235	SS	Mus musculus
1	1.00	1	1	0	0	0	0	-0.003	t	99849	VNSTEAETI	Homo sapiens
1	1.00	1	1	0	0	0	0	-0.003	t	99975	EEKELLKRIQ	Mus musculus
1	1.00	1	1	0	0	0	0	-0.003	t	110385	QLYKTCKQAGTCPPDIIPKV	Oryctolagus cuniculus
1	1.00	1	1	0	0	0	0	-0.003	t	116677	ASATQLYKTCKQAGTCPPD	Mus musculus
1	1.00	1	1	0	0	0	0	-0.003	t	103998	IVKWDRDM	Mus musculus

1	1.00	1	1	0	0	0	0	-0.003	t	99945	DIRDSLSEE E	Mus musculu s
1	1.00	1	1	0	0	0	0	-0.003	t	104443	DAEFRHDS GYE	Homo sapiens
1	1.00	1	1	0	0	0	0	-0.003	t	100153	INDQIKFII N	Mus musculu s
1	1.00	1	1	0	0	0	0	-0.003	t	99210	ELFTNRW NLQSLL	Oryctola gus cuniculu s
1	1.00	1	1	0	0	0	0	-0.003	t	102510	HHQKLVFF AEDVGSN K	Mus musculu s
1	1.00	1	1	0	0	0	0	-0.003	t	117195	DT	Mus musculu s
1	1.00	1	1	0	0	0	0	-0.003	t	106322	DAEFRHDS G	Oryctola gus cuniculu s
1	1.00	1	1	0	0	0	0	-0.003	t	98822	IEAGAIGFK IHEDWG	Mus musculu s
1	1.00	1	1	0	0	0	0	-0.003	t	100777	DIDFLIEEIE RLGQDL	Oryctola gus cuniculu s
1	1.00	1	1	0	0	0	0	-0.003	t	61394	SSTKNLIY	Mus musculu s
1	1.00	1	1	0	0	0	0	-0.003	t	61317	SSRGKVVE	Mus musculu s
1	1.00	1	1	0	0	0	0	-0.003	t	68524	VFMVPQY GYL	Mus musculu s
1	1.00	1	1	0	0	0	0	-0.003	t	71798	VVQAMYV PPGAPNP KE	Oryctola gus cuniculu s

1	1.00	1	1	0	0	0	0	-0.003	t	96367	HSNLDHI	Homo sapiens
1	1.00	1	1	0	0	0	0	-0.003	t	95044	SSRGK	Mus musculus
1	1.00	1	1	0	0	0	0	-0.003	t	66108	TRTNPWPALAEGEF	Oryctolagus cuniculus
1	1.00	1	1	0	0	0	0	-0.003	t	72065	VYKLDISEA	Mus musculus
1	1.00	1	1	0	-0.003	0	0	-0.003	t	95142	RIKNTDGSIS	Oryctolagus cuniculus
1	1.00	1	1	0	0	0	0	-0.003	t	63736	TFQVEVPGSQHIDS	Oryctolagus cuniculus
1	1.00	1	1	0	0	0	0	-0.003	t	80014	RNYPTGAEFLGEG	Oryctolagus cuniculus
1	1.00	1	1	0	0	0	0	-0.003	t	74497	YKQKIIAP	Mus musculus
1	1.00	1	1	0	0	0	0	-0.003	t	80002	RKADLEKALEGAM	Oryctolagus cuniculus
1	1.00	1	1	0	0	0	0	-0.003	t	94483	CYKKVWRDHRGTIIERGC	Mus musculus
1	1.00	1	1	0	0	0	0	-0.003	t	69178	VKAETRLNPDLQPTE	Mus musculus
1	1.00	1	1	0	0	0	0	-0.003	t	70656	VQTRHVVQHRSRSESSIESF	Oryctolagus cuniculus
1	1.00	1	1	0	0	0	0	-0.003	t	95524	KRQIYTDIEMNRLGK	Mus musculus

1	1.00	1	1	0	0	0	0	-0.003	t	66304	TSLNDRQ GNATK	Oryctolagus cuniculus
1	1.00	1	1	0	0	0	0	-0.003	t	66976	TVDNPAST TNKDKLFA VWK	Rattus norvegicus
1	1.00	1	1	0	0	0	0	-0.003	t	73487	YCCELCCN PACAGCY	Mus musculus
1	1.00	1	1	0	0	0	0	-0.003	t	97348	GLFGAIAG FIEGGW	Oryctolagus cuniculus
1	1.00	1	1	0	0	0	0	-0.003	t	95524	KRQIYTDIE MNRLGK	Oryctolagus cuniculus
1	1.00	1	1	0	0	0	0	-0.003	t	76290	YVHDNC	Mus musculus
1	1.00	1	1	0	0	0	0	-0.003	t	71328	VTGKVEYT KYNDDD	Oryctolagus cuniculus
1	1.00	1	1	0	0	0	0	-0.003	t	70390	VPNLRGDL QVLAQKV ART	Ovis aries
1	1.00	1	1	0	0	0	0	-0.003	t	74009	YGGPANK KNAG	Mus musculus
1	1.00	1	1	0	0	0	0	-0.003	t	78424	IPRSRQIDL QIG	Mus musculus
1	1.00	1	1	0	0	0	0	-0.003	t	75949	YTASARGD LAHL	Mus musculus
1	1.00	1	1	0	0	0	0	-0.003	t	63037	TAVSPTTL R	Oryctolagus cuniculus
1	1.00	1	1	0	0	0	0	-0.003	t	69175	VKAETRLN	Mus musculus

1	1.00	1	1	0	0	0	0	-0.003	t	61394	SSTKNLIY	Mus musculus
1	1.00	1	1	0	0	0	0	-0.003	t	70656	VQTRHVVQHRSRSESIESF	Rattus norvegicus
1	1.00	1	1	0	0	0	0	-0.003	t	58511	SIFYTYGTAPARISVPYVGI	Oryctolagus cuniculus
1	1.00	1	1	0	0	0	0	-0.003	t	56373	RVNQVAEVLQL	Mus musculus
1	1.00	1	1	0	0	0	0	-0.003	t	38522	LPHSNL	Homo sapiens
1	1.00	1	1	0	0	0	0	-0.003	t	51371	QLINTNGSWHIN	Rattus norvegicus
1	1.00	1	1	0	0	0	0	-0.003	t	54057	RHKQKIVAPVK	Oryctolagus cuniculus
1	1.00	1	1	0	0	0	0	-0.003	t	43242	NANPNANPNANP	Oryctolagus cuniculus
1	1.00	1	1	0	0	0	0	-0.003	t	57726	SFANTNTNTNSK	Mus musculus
1	1.00	1	1	0	0	0	0	-0.003	t	57542	SELSQLS	Mus musculus
1	1.00	1	1	0	0.01	0	0	-0.003	t	59967	SNYYSDNEIEQHTAKQPKL	Oryctolagus cuniculus
1	1.00	1	1	0	0	0	0	-0.003	t	45932	NSNNPDWDF	Mus musculus

1	1.00	1	1	0	0	0	0	-0.003	t	43730	NEREDERTLTKEYEDIVLK	Oryctolagus cuniculus
1	1.00	1	1	0	-0.003	0	0	-0.003	t	39092	LRGDLQVLAQKVARTL	Oryctolagus cuniculus
1	1.00	1	1	0	0	0	0	-0.003	t	49629	PTGDRHKQPLVAPAKQLL	Mus musculus
1	1.00	1	1	0	0	0	0	-0.003	t	39092	LRGDLQVLAQKVARTL	Mus musculus
1	1.00	1	1	0	0	0	0	-0.003	t	56734	SADNNNSEYSWT	Mus musculus
1	1.00	1	1	0	0.002	0	0	-0.003	t	58511	SIFYTYGTAPARISVPYVGI	Rattus norvegicus
1	1.00	1	1	0	0	0	0	-0.003	t	51775	QNTRNIVPVSIVSR	Mus musculus
1	1.00	1	1	0	0.002	0	0	-0.003	t	44474	NKDKLFAVWK	Rattus norvegicus
1	1.00	1	1	0	0	0	0	-0.003	t	58511	SIFYTYGTAPARISVPYVGI	Rattus norvegicus
1	1.00	1	1	0	0	0	0	-0.003	t	37698	LLQAHRQLEER	Mus musculus
1	1.00	1	1	0	0	0	0	-0.003	t	56699	SAAFYYRV	Mus musculus
1	1.00	1	1	0	0	0	0	-0.003	t	43576	NEDNEKLRKPKHKKLKQPG	Mus musculus
1	1.00	1	1	0	0	0	0	-0.003	t	43191	NAKATYEAALKQYEADLAA	Mus musculus

1	1.00	1	1	0	0	0	0	-0.003	t	41608	MGPNLVL PDQKPPS	Mus musculu s
1	1.00	1	1	0	0	0	0	-0.003	t	59810	SNEFAPVQ NLTGSSVG	Mus musculu s
1	1.00	1	1	0	-0.003	0	0	-0.003	t	46960	PASTTNKD KL	Oryctola gus cuniculu s
1	1.00	1	1	0	0	0	0	-0.003	t	38522	LPHSNL	Rattus norvegic us
1	1.00	1	1	0	0	0	0	-0.003	t	32808	KPNLSSKR SELSQLSM YRVF	Mus musculu s
1	1.00	1	1	0	0	0	0	-0.003	t	11463	EDVNGIRK PK	Mus musculu s
1	1.00	1	1	0	0	0	0	-0.003	t	23975	HHAEMQ NPVYLIPET VPYIK	Mus musculu s
1	1.00	1	1	0	0	0	0	-0.003	t	10433	DTNNN	Mus musculu s
1	1.00	1	1	0	0	0	0	-0.003	t	21856	GQDLTAH	Mus musculu s
1	1.00	1	1	0	0	0	0	-0.003	t	28277	IRECTGLA WEWWRT V	Mus musculu s
1	1.00	1	1	0	0	0	0	-0.003	t	34537	KYNDDDT FTVKVGD	Oryctola gus cuniculu s
1	1.00	1	1	0	0	0	0	-0.003	t	34965	LASLRKAF ADTVPVRP PSNY	Homo sapiens
1	1.00	1	1	0	0	0	0	-0.003	t	21763	GPRLQPY	Mus musculu s
1	1.00	1	1	0	0	0	0	-0.003	t	13290	ELTIPIFAT NSDCELIV KA	Oryctola gus cuniculu s

1	1.00	1	1	0	0	0	0	-0.003	t	20710	GKVEYTKYNDDDTFTVKVGD	Oryctolagus cuniculus
1	1.00	1	1	0	0	0	0	-0.003	t	25146	HWYISKPQ	Mus musculus
1	1.00	1	1	0	0	0	0	-0.003	t	31916	KLILAYTPPGARGPQD	Oryctolagus cuniculus
1	1.00	1	1	0	0	0	0	-0.003	t	31105	KHFRETEV	Mus musculus
1	1.00	1	1	0	0	0	0	-0.003	t	14808	EVLYLKPLAGVYRSLKKQLE	Mus musculus
1	1.00	1	1	0	0	0	0	-0.003	t	22401	GSLAAR	Mus musculus
1	1.00	1	1	0	0	0	0	-0.003	t	11270	ECTGLAWEWWRT	Mus musculus
1	1.00	1	1	0	0	0	0	-0.003	t	25108	HVTSTLTSLF	Mus musculus
1	1.00	1	1	0	0	0	0	-0.003	t	14808	EVLYLKPLAGVYRSLKKQLE	Mus musculus
1	1.00	1	1	0	0	0	0	-0.003	t	24970	HTRVTGGVQG	Mus musculus
1	1.00	1	1	0	0	0	0	-0.003	t	21763	GPRLQPY	Mus musculus
1	1.00	1	1	0	0	0	0	-0.003	t	14809	EVLYLKPLAGVYRSLKKQLE	Mus musculus
1	1.00	1	1	0	0	0	0	-0.003	t	35852	LFIG	Mus musculus

1	1.00	1	1	0	0	0	0	-0.003	t	25139	HWSISKPQ	Mus musculus
1	1.00	1	1	0	0	0	0	-0.003	t	34912	LAPRVAR	Mus musculus
1	1.00	1	1	0	0	0	0	-0.003	t	25807	IEFQQKNNRLLEITREF	Mus musculus
1	1.00	1	1	0	0	0	0	-0.003	t	22360	GSGVRGDFGSLAPRVARQL	Cavia porcellus
1	1.00	1	1	0	0	0	0	-0.003	t	11208	EAVEAERAGRGTG	Mus musculus
1	1.00	1	1	0	0	0	0	-0.003	t	10579	DVELSLRSSDIA	Homo sapiens
1	1.00	1	1	0	0.004	0	0	-0.003	t	25807	IEFQQKNNRLLEITREF	Mus musculus
1	1.00	1	1	0	0	0	0	-0.003	t	6226	CFGNSEY	Mus musculus
1	1.00	1	1	0	0	0	0	-0.003	t	6093	CDCDRTAA	Mus musculus
1	1.00	1	1	0	-0.003	0	0	-0.003	t	9520	DNNQTSP	Oryctolagus cuniculus
1	1.00	1	1	0	0	0	0	-0.003	t	3274	ANETIYNTTLKYGDV	MD
1	1.00	1	1	0	0	0	0	-0.003	t	9520	DNNQTSP	Mus musculus
1	1.00	1	1	0	0	0	0	-0.003	t	1190	AETIHELLVRM	Mus musculus

1	1.00	1	1	0	0	0	0	-0.003	t	6832	CPRPPRAV AYYGPGV DYK	Oryctola gus cuniculu s
1	1.00	1	1	0	0	0	0	-0.003	t	9723	DPPPPNP NDPPPPN PND	Mus musculu s
1	1.00	1	1	0	0	0	0	-0.003	t	4149	ARGSLAVT I	Mus musculu s
1	1.00	1	1	0	0.002	0	0	-0.003	t	279	AALGDSLY GAASLNDF GILA	Rattus norvegic us
1	1.00	1	1	0	0	0	0	-0.003	t	279	AALGDSLY GAASLNDF GILA	Rattus norvegic us
1	1.00	1	1	0	0	0	0	-0.003	t	7313	CVTIMTVD NPASTTNK DK	Oryctola gus cuniculu s
1	1.00	1	1	0	0	0	0	-0.003	t	6139	CDSSDRPR VYTIT	Mus musculu s
1	1.00	1	1	0	0	0	0	-0.003	t	9524	DNPASTT NKDK	Mus musculu s
1	1.00	1	1	0	0	0	0	-0.003	t	4331	ASARGDLA HLTTTHAR HLP	Mus musculu s
1	1.00	1	1	0	0	0	0	-0.003	t	6139	CDSSDRPR VYTIT	Mus musculu s
1	1.00	1	1	0.01	0.01	0.01	0.01	0.009	t	6455	CITQYERES QAYY	Mus musculu s
1	1.00	1	1	0	-0.002	0	-0.003	-0.004	t	16101	FHENWPS	Mus musculu s
1	1.00	1	1	0	0.004	0	0.007	0	t	112972	FHAIDEYK PQDATTN	Homo sapiens
1	1.00	1	1	0.01	0.013	0.01	0.01	0.008	t	135501	SQWNKP	Mus musculu s

1	1.00	1	1	0.01	0.007	0.014	0.01	0.008	t	100074	GNINYACGQGID	Oryctolagus cuniculus
1	1.00	1	1	0	0	0.005	0	-0.002	t	130474	ECLLLSKYCMPS	Mus musculus
1	1.00	1	1	0.04	0.04	0.04	0.04	0.04	t	111444	KDQTNYP	Homo sapiens
1	1.00	1	1	-0.01	-0.01	-0.022	-0.01	-0.01	t	62845	TAFSKN	Homo sapiens
1	1.00	1	1	-0.02	-0.018	-0.016	-0.013	-0.021	t	119400	WPGAGPPPPGRVLYGG	Homo sapiens
1	1.00	1	1	0	0.003	0.002	0.007	0	t	117331	MKLVRFLMKLSHETVTIELK	Mus musculus
1	1.00	1	1	0	0	0.002	0.007	0	t	117331	MKLVRFLMKLSHETVTIELK	Mus musculus
1	1.00	1	1	0.01	0.014	0.014	0.01	0.008	t	134335	CTKPSDGNCT	Homo sapiens
1	1.00	1	1	0.01	0.006	0.001	0.009	0.01	t	63038	TAVSPTTLRTEVVK	Oryctolagus cuniculus
1	1.00	1	1	-0.04	-0.037	-0.045	-0.033	-0.04	t	117380	TKPAIRRLARRGGVKRIS	Mus musculus
1	1.00	1	1	0	0.002	-0.013	0.001	0.004	t	23249	GWGNGC	Homo sapiens
1	1.00	1	1	0.01	0.012	0.008	0.007	0.007	t	105746	EETICTASFKKNIGR	Homo sapiens
1	1.00	1	1	0.02	0.02	0.02	0.02	0.018	t	145827	IIPKVEGK	Mus musculus
1	1.00	1	1	0.05	0.047	0.042	0.05	0.05	t	7383	CYGVSATKLNDLCFSNV	Oryctolagus cuniculus
1	1.00	1	1	-0.01	-0.01	-0.013	-0.004	-0.01	t	48343	PLGLKRGIEK	Homo sapiens

1	1.00	1	1	0	0	-0.003	0.003	-0.001	t	16006	FGLQLELT EGMRFDK	Homo sapiens
1	1.00	1	1	0	0	0	0.004	-0.001	t	122947	WTQEPQP LEEKWQH RVVE	Homo sapiens
1	1.00	1	1	0	0	0	0.004	-0.001	t	65050	TLRVYK	Homo sapiens
1	1.00	1	1	-0.01	-0.012	-0.01	-0.013	-0.011	t	72251	WAPSV	Mus musculu s
1	1.00	1	1	-0.01	-0.015	-0.018	-0.007	-0.008	t	158290	SQQAPAV PPLPQ	Canis lupus
1	1.00	1	1	-0.01	-0.012	-0.01	-0.009	-0.011	t	130461	CWNYCLP	Mus musculu s
1	1.00	1	1	0	0	-0.014	0	0.002	t	28350	IRNVPHPF VALGLKQ	Mus musculu s
1	1.00	1	1	0	0	-0.014	0	0.002	t	28350	IRNVPHPF VALGLKQ	Mus musculu s
1	1.00	1	1	0	0	0	0	0	t	63043	TAWDFG	Homo sapiens
1	1.00	1	1	0.03	0.03	0.03	0.03	0.03	t	79528	CPSKKPYE EVTC	Mus musculu s
1	1.00	1	1	0.04	0.038	0.035	0.041	0.04	t	39888	LTEVETPIR NEWGC	Mus musculu s
1	1.00	1	1	0.02	0.023	0.016	0.02	0.02	t	41866	MKNNPIYS EGSL	Equus caballus
1	1.00	1	1	0.02	0.023	0.016	0.02	0.02	t	41866	MKNNPIYS EGSL	Equus caballus
1	1.00	1	1	0.02	0.02	0.02	0.024	0.02	t	133795	DFTYDQV AFNGLPQF	Homo sapiens
1	1.00	1	1	0	-0.002	0	-0.003	-0.004	t	141282	GETRK	Mus musculu s
1	1.00	1	1	0	-0.002	0	-0.003	-0.004	t	140665	DTAAQI	Mus musculu s

1	1.00	1	1	-0.01	-0.006	-0.003	-0.009	-0.017	t	23226	GVYLLPRRGPRLGVRATRKT	Homo sapiens
1	1.00	1	1	-0.01	-0.01	-0.01	-0.006	-0.01	t	135975	PTLAFLQDVMNI	Homo sapiens
1	1.00	1	1	0.02	0.022	0.02	0.023	0.018	t	104443	DAEFRHDSGYE	Homo sapiens
1	1.00	1	1	-0.01	-0.012	-0.012	-0.011	-0.01	t	97377	GYKGVAFP	Mus musculus
1	1.00	1	1	0.02	0.022	0.018	0.027	0.02	t	118335	EQLRILGEKEAKLAQQSQA	Homo sapiens
1	1.00	1	1	-0.01	-0.01	-0.024	-0.01	-0.01	t	78937	AYSDDFFVGRV	Mus musculus
1	1.00	1	1	0.01	0.01	-0.001	0.01	0.01	t	111746	RSSRTRRETQ	Oryctolagus cuniculus
1	1.00	1	1	0.01	0.006	-0.008	0.01	0.012	t	49636	PTGPTIAAGGEQPPS	Bos taurus
1	1.00	1	1	-0.01	-0.013	-0.023	-0.01	-0.009	t	571	ACKSTQDPMFTPKGCDN	Oryctolagus cuniculus
1	1.00	1	1	-0.03	-0.03	-0.03	-0.03	-0.03	t	145934	PDIIPK	Mus musculus
1	1.00	1	1	0	0.002	0	0.003	-0.001	t	108874	DPISGHVSLF	Homo sapiens
1	1.00	1	1	-0.02	-0.018	-0.034	-0.019	-0.02	t	31005	KGLRRDLDASREAKKQ	Homo sapiens
1	1.00	1	1	-0.02	-0.024	-0.016	-0.019	-0.022	t	65618	TPKGP	Bos taurus
1	1.00	1	1	-0.01	-0.012	-0.01	-0.007	-0.01	t	67222	TVYFGFDKYD	Mus musculus
1	1.00	1	1	0.03	0.032	0.03	0.037	0.03	t	107497	KVSNKAL	Homo sapiens

1	1.00	1	1	-0.01	-0.013	-0.013	-0.01	-0.012	t	7208	CTSPYDGKYWSMYSRL	Oryctolagus cuniculus
1	1.00	1	1	-0.01	-0.01	-0.014	-0.01	-0.012	t	47545	PFPQPQQQFP	Mus musculus
1	1.00	1	1	-0.01	-0.012	-0.024	-0.011	-0.01	t	28107	IPVSDAQR	Mus musculus
1	1.00	1	1	0.02	0.02	0.02	0.02	0.02	t	106042	QLKNNNPFKFFVPPS	Homo sapiens
1	1.00	1	1	-0.01	-0.014	-0.019	-0.007	-0.01	t	115058	GNAEVHASFFDIGGS	Oryctolagus cuniculus
1	1.00	1	1	0.02	0.02	0.026	0.021	0.02	t	34238	KVVLPE	Homo sapiens
1	1.00	1	1	0	-0.003	0	0	-0.001	t	24009	HHPSTDKEQTNLY	Oryctolagus cuniculus
1	1.00	1	1	0	-0.003	0	0	-0.001	t	24009	HHPSTDKEQTNLY	Oryctolagus cuniculus
1	1.00	1	1	-0.01	-0.008	-0.017	-0.003	-0.006	t	135897	APKRPGPAGELL	Homo sapiens
1	1.00	1	1	0.06	0.062	0.056	0.057	0.058	t	114848	GVTYDNECLL	Homo sapiens
1	1.00	1	1	-0.02	-0.018	-0.02	-0.019	-0.02	t	9268	DLRSSSGC	Homo sapiens
1	1.00	1	1	0.01	0.014	0.017	0.007	0.006	t	106762	QYFALEAYAF	Homo sapiens
1	1.00	1	1	0.01	0.006	0.008	0.009	0.01	t	45693	NRKNQLKDMA	Oryctolagus cuniculus

1	1.00	1	1	0.03	0.028	0.03	0.03	0.03	t	107086	ANYGYTFGSG	Oryctolagus cuniculus
1	1.00	1	1	0.02	0.024	0.013	0.021	0.02	t	79926	MASMTGGQQMG	MD
1	1.00	1	1	-0.01	-0.012	-0.012	-0.007	-0.008	t	99774	VVLGYTDR	Mus musculus
1	1.00	1	1	0.01	0.01	0.003	0.019	0.012	t	131053	RRVQQEIDDVIGQVRRPEMG	Homo sapiens
1	1.00	1	1	0.03	0.03	0.03	0.03	0.03	t	112371	TYYCARM	Homo sapiens
1	1.00	1	1	-0.05	-0.05	-0.055	-0.048	-0.05	t	67569	VAEKVRNLPA	Homo sapiens
1	1.00	1	1	0.03	0.03	0.03	0.032	0.032	t	70270	VPDKEVLYQQYDEM	Homo sapiens
1	1.00	1	1	0.03	0.027	0.029	0.03	0.03	t	63818	TGFADPNTTTG	Oryctolagus cuniculus
1	1.00	1	1	0.02	0.018	0.01	0.017	0.02	t	59880	SNPTPQEI	Anas platyrhynchos
1	1.00	1	1	0.02	0.019	0.01	0.017	0.02	t	59880	SNPTPQEI	Anas platyrhynchos
1	1.00	1	1	0.01	0.006	0.001	0.009	0.01	t	20284	GIGPEKAFRE	Oryctolagus cuniculus
1	1.00	1	1	-0.03	-0.03	-0.03	-0.03	-0.03	t	56133	RTMQYVDM	Homo sapiens
1	1.00	1	1	-0.04	-0.04	-0.029	-0.039	-0.04	t	41541	MFTCKK	Homo sapiens
1	1.00	1	1	0.03	0.032	0.031	0.031	0.03	t	9929	DQYCADVAAEELMNA	Homo sapiens
1	1.00	1	1	-0.03	-0.03	-0.03	-0.03	-0.03	t	60751	SRNWLPGPCY	Mus musculus

1	1.00	1	1	0.01	0.003	0.003	0.01	0.011	t	159296	SFSKPSNS WFPVEGP	Oryctola gus cuniculu s
1	1.00	1	1	0	-0.002	0	-0.001	0.001	t	48598	PNANPNA	Mus musculu s
1	1.00	1	1	0.01	0.007	-0.005	0.003	0.01	t	104036	LHSDIMFY TIENAVPIH LL	Oryctola gus cuniculu s
1	1.00	1	1	0.02	0.016	0.018	0.02	0.02	t	22835	GTSMDDE ADRYF	Bos taurus
1	1.00	1	1	-0.02	-0.02	-0.032	-0.023	-0.02	t	71192	VSVK	Homo sapiens
1	1.00	1	1	0.01	0.012	-0.007	0.011	0.012	t	151068	VGSSRYSK KFKPEIA	Homo sapiens
1	1.00	1	1	0.02	0.02	0.02	0.02	0.02	t	139191	NYTCCKLQ	Mus musculu s
1	1.00	1	1	0	0	0	0	0	t	107655	VFLFPPK	Homo sapiens
1	1.00	1	1	0.04	0.042	0.035	0.041	0.04	t	16143	FHSTSSEG N	Homo sapiens
1	1.00	1	1	-0.01	-0.013	-0.011	-0.01	-0.014	t	30200	KDLLEQKR AAVDTYC	Oryctola gus cuniculu s
1	1.00	1	1	0.02	0.018	0.01	0.023	0.02	t	30774	KFLNPDRE YDFRDLT	Mus musculu s
1	1.00	1	1	0.01	0.013	0.01	0.01	0.008	t	64541	TKDTNNN L	Mus musculu s
1	1.00	1	1	-0.02	-0.016	-0.026	-0.021	-0.023	t	50814	QFQGQQ QPFP	Homo sapiens
1	1.00	1	1	-0.01	-0.008	-0.023	-0.009	-0.01	t	21277	GMGVSCT VTREDGT NRR	Homo sapiens

1	1.00	1	1	0.02	0.017	0.018	0.02	0.02	t	57680	SEWRSPADTSPRPLP	Oryctolagus cuniculus
1	1.00	1	1	0.02	0.016	0.018	0.02	0.02	t	57680	SEWRSPADTSPRPLP	Oryctolagus cuniculus
1	1.00	1	1	-0.01	-0.01	-0.01	-0.01	-0.012	t	72910	WNSTTFHQTL	Mus musculus
1	1.00	1	1	0	0.002	0	0.007	0.001	t	140547	CSIGEALSHGC	Homo sapiens
1	1.00	1	1	-0.03	-0.032	-0.033	-0.029	-0.03	t	133749	YNLLMHLSSML	Mus musculus
1	1.00	1	1	0.01	0.01	0.016	0.01	0.01	t	50536	QDVVVLGS	Homo sapiens
1	1.00	1	1	0	0	-0.011	0	0.001	t	17467	FQDMLNIS	Mus musculus
1	1.00	1	1	-0.01	-0.008	-0.014	-0.012	-0.013	t	105490	QSRATLGI	Homo sapiens
1	1.00	1	1	0.01	0.01	0.01	0.014	0.01	t	135969	PPEPRPRDRSGL	Homo sapiens
1	1.00	1	1	0.01	0.01	0.001	0.01	0.01	t	70945	VSGCPMRLNMASLEEKGRHF	Homo sapiens
1	1.00	1	1	0	0.003	-0.006	0	0.002	t	66061	TRLNPDLQ	Mus musculus
1	1.00	1	1	-0.02	-0.005	-0.024	-0.019	-0.02	t	60792	SRRYPFVKF	Homo sapiens
1	1.00	1	1	-0.02	-0.024	-0.018	-0.021	-0.02	t	139500	FHTHISV	Oryctolagus cuniculus
1	1.00	1	1	0.01	0.012	0.003	0.008	0.008	t	106595	LDGETKTGPQRV	Homo sapiens
1	1.00	1	1	0	0	0	0.007	0	t	119474	KIRY	Homo sapiens

1	1.00	1	1	0.01	0.006	0.003	0.013	0.01	t	96017	YHLYNSQDHG	Oryctolagus cuniculus
1	1.00	1	1	0.01	0.01	0.006	0.016	0.01	t	67069	TVLGCALPPTPQTP	Homo sapiens
1	1.00	1	1	0.05	0.048	0.045	0.047	0.049	t	134131	LEEAKTQGPYDVVVL	Mus musculus
1	1.00	1	1	-0.03	-0.027	-0.037	-0.03	-0.028	t	113049	KLDAAYKLAY	Mus musculus
1	1.00	1	1	0.02	0.022	0.029	0.027	0.022	t	109780	QVYTLPP	Homo sapiens
1	1.00	1	1	-0.01	-0.01	-0.01	-0.01	-0.01	t	128024	IKKVATPKKASPKK	Homo sapiens
1	1.00	1	1	0	0	-0.001	0	-0.002	t	137944	PGTEYT	Oryctolagus cuniculus
1	1.00	1	1	0.02	0.022	0.022	0.027	0.02	t	119598	INPESDQPDLSNFMESG	Homo sapiens
1	1.00	1	1	0.02	0.02	0.02	0.02	0.019	t	118783	EIIVTHFPFDEQNCSMK	Rattus norvegicus
1	1.00	1	1	0	0	0	0	0	t	56246	RVCEKM	Homo sapiens
1	1.00	1	1	0	0	-0.01	-0.001	0	t	57875	SFQSFNLTEPHIT	Homo sapiens
1	1.00	1	1	-0.01	-0.008	-0.007	-0.003	-0.01	t	128101	SFSRALENATRDYFIICPII	Homo sapiens
1	1.00	1	1	0.06	0.06	0.053	0.06	0.063	t	96170	DEDSYERDP	Mus musculus
1	1.00	1	1	0.02	0.016	0.015	0.023	0.02	t	95263	ATYHLYNSQD	Oryctolagus cuniculus

1	1.00	1	1	-0.04	-0.038	-0.034	-0.039	-0.047	t	61516	STAIHADQ LTPAWRIY	Homo sapiens
1	1.00	1	1	0	0	-0.004	-0.003	0	t	39136	LRLKKYKV PQ	Homo sapiens
1	1.00	1	1	0	0	0	0.006	0	t	117773	CNSRQTD REDELI	Homo sapiens
1	1.00	1	1	0	0	0	0.006	0	t	43248	NANPNAN PNANPNA NPNANP	Homo sapiens
1	1.00	1	1	0.01	0.008	-0.004	0.007	0.01	t	75532	YQPQQPF PQP	Mus musculu s
1	1.00	1	1	0	0.002	-0.002	0.003	0	t	118196	VEPTGKRF L	Homo sapiens
1	1.00	1	1	0.02	0.023	0.02	0.02	0.02	t	7532	DAHHAAD	Mus musculu s
1	1.00	1	1	0.04	0.042	0.038	0.043	0.04	t	78478	PDSDPQI	Homo sapiens
1	1.00	1	1	0.04	0.042	0.038	0.043	0.04	t	78478	PDSDPQI	Homo sapiens
1	1.00	1	1	0	-0.009	-0.016	0	0.002	t	65218	TMKNNLQ T	Macaca mulatta
1	1.00	1	1	0.03	0.034	0.035	0.031	0.029	t	58779	SKAALIEEG	Homo sapiens
1	1.00	1	1	0	0	0	0	0.001	t	59024	SLAYGLR	Oryctola gus cuniculu s
1	1.00	1	1	-0.01	-0.01	-0.01	-0.01	-0.01	t	47189	PDREVLYR E	Homo sapiens

1	1.00	1	1	-0.04	-0.038	-0.04	-0.037	-0.041	t	134418	KGAIIGLM VGGVV	Homo sapiens
1	1.00	1	1	0.04	0.044	0.04	0.041	0.04	t	18511	GAALLQSA	Homo sapiens
1	1.00	1	1	-0.02	-0.02	-0.011	-0.02	-0.025	t	134492	QIQRRPS M	Mus musculu s
1	1.00	1	1	0.01	0.003	0.004	0.007	0.009	t	44920	NLVPMVA TV	Homo sapiens
1	1.00	1	1	0.01	0.006	0.009	0.009	0.01	t	58950	SKTNIFWA GDLL	Oryctola gus cuniculu s
1	1.00	1	1	0.01	0.008	0.009	0.009	0.01	t	58950	SKTNIFWA GDLL	Mus musculu s
1	1.00	1	1	0.05	0.048	0.045	0.051	0.05	t	39888	LTEVETPIR NEWGC	Mus musculu s
1	1.00	1	1	0.01	0.004	0.01	0.01	0.01	t	123259	GQPPYMP P	Oryctola gus cuniculu s
1	1.00	1	1	0.05	0.054	0.061	0.051	0.049	t	44699	NLEYTI	Homo sapiens
1	1.00	1	1	-0.01	-0.01	-0.023	-0.01	-0.01	t	159137	STFVVDGP ETAECPNS	Mus musculu s
1	1.00	1	1	0	0	0	0	-0.003	t	137407	GPEYWDG ETRKVKA	Oryctola gus cuniculu s
1	1.00	1	1	0	0	0	0	-0.003	t	122748	EHRMTW DPAQPPR	Homo sapiens
1	1.00	1	1	0	-0.003	0	0	-0.003	t	105830	GKSGDRS GYSSPGSP GTPG	Oryctola gus cuniculu s
1	1.00	1	1	0	0	0	0	-0.003	t	102344	DAEFR	Mus musculu s

1	1.00	1	1	0	-0.003	0	0	-0.003	t	78940	CRRHNYGFWVC	Oryctolagus cuniculus
1	1.00	1	1	0	0	0	0	-0.003	t	94580	LDNKYAGKGY	Mus musculus
1	1.00	1	1	0	0	0	0	-0.003	t	76577	YYQQTDEANK	Oryctolagus cuniculus
1	1.00	1	1	0	0	0	0	-0.003	t	63604	TEYYLNHGKWPEN	Oryctolagus cuniculus
1	1.00	1	1	0	0	0	0	-0.003	t	79303	LVAAGVDYEDERISFQDWPK	Rattus norvegicus
1	1.00	1	1	0	-0.003	0	0	-0.003	t	78939	CINHRGYWVC	Oryctolagus cuniculus
1	1.00	1	1	0	0	0	0	-0.003	t	63811	TGEDPGFFNV	Oryctolagus cuniculus
1	1.00	1	1	0	0	0	0	-0.003	t	48093	PKKPNENKLKQPNE	Oryctolagus cuniculus
1	1.00	1	1	0	0	0	0	-0.003	t	48912	PPSDIKGKYVKEVEVK	Oryctolagus cuniculus
1	1.00	1	1	0	0	0	0	-0.003	t	38422	LPAYQDYTARAQVSE	Oryctolagus cuniculus
1	1.00	1	1	0	0	0	0	-0.003	t	40194	LTTDATTFEEVATAEKLV	Oryctolagus cuniculus
1	1.00	1	1	0	0	0	0	-0.003	t	48093	PKKPNENKLKQPNE	Mus musculus

1	1.00	1	1	0	0	0	0	-0.003	t	48093	PKKPNENKLKQPNE	Oryctolagus cuniculus
1	1.00	1	1	0	-0.003	0	0	-0.003	t	48670	PNLRGDLQVLAQKVARTLP	Oryctolagus cuniculus
1	1.00	1	1	0	0	0	0	-0.003	t	54975	RNCKKLGPRENVA	Oryctolagus cuniculus
1	1.00	1	1	0	0	0	0	-0.003	t	55621	RRKAHAGNKKAEDLTMDDLE	Mus musculus
1	1.00	1	1	0	0	0	0	-0.003	t	55621	RRKAHAGNKKAEDLTMDDLE	Oryctolagus cuniculus
1	1.00	1	1	0	0	0	0	-0.003	t	55621	RRKAHAGNKKAEDLTMDDLE	Oryctolagus cuniculus
1	1.00	1	1	0	0	0	0	-0.003	t	43242	NANPNANPNANP	Oryctolagus cuniculus
1	1.00	1	1	0	0	0	0	-0.003	t	48912	PPSDIKGKYVKEVEVK	Oryctolagus cuniculus
1	1.00	1	1	0	0	0	0	-0.003	t	53326	RDALPNTEASGPTHSKE	Rattus norvegicus
1	1.00	1	1	0	0	0	0	-0.003	t	41610	MGQHPAKSMDVRR	Oryctolagus cuniculus
1	1.00	1	1	0	0	0	0	-0.003	t	50180	PYVEPTAPQVL	Homo sapiens
1	1.00	1	1	0	0	0	0	-0.003	t	43242	NANPNANPNANP	Homo sapiens

1	1.00	1	1	0	0	0	0	-0.003	t	50180	PYVEPTAP QVL	Oryctolagus cuniculus
1	1.00	1	1	0	0	0	0	-0.003	t	55621	RRKAHAG NKKAEDLT MDDLE	Oryctolagus cuniculus
1	1.00	1	1	0	0	0	0	-0.003	t	43242	NANPNAN PNANP	Mus musculus
1	1.00	1	1	0	-0.003	0	0	-0.003	t	48670	PNLRGDL QVLAQKV ARTLP	Oryctolagus cuniculus
1	1.00	1	1	0	0	0	0	-0.003	t	10639	DVKFPGG GQIVGGV YLLPRR	Mus musculus
1	1.00	1	1	0	-0.003	0	0	-0.003	t	32844	KPQEEKEK ITKEILNGK	Oryctolagus cuniculus
1	1.00	1	1	0	-0.003	0	0	-0.003	t	19636	GFTTNEER YNVFAE	Oryctolagus cuniculus
1	1.00	1	1	0	0	0	0	-0.003	t	11815	EENVEENV EENVEENV	Mus musculus
1	1.00	1	1	0	0	0	0	-0.003	t	27965	IPNPLLGL D	Mus musculus
1	1.00	1	1	0	-0.003	0	0	-0.003	t	19636	GFTTNEER YNVFAE	Oryctolagus cuniculus
1	1.00	1	1	0	-0.003	0	0	-0.003	t	9722	DPPPPNP NDPPPPN PN	Oryctolagus cuniculus
1	1.00	1	1	0	0	0	0	-0.003	t	5728	AYANSTQE ETF	Oryctolagus cuniculus
1	1.00	1	1	0	0	0	0	-0.003	t	8475	DGQPAGD RADGQPA GDRA	Mus musculus

1	1.00	1	1	0	0	0	0	-0.003	t	9753	DPRVRGLY	Mus musculus
1	1.00	1	1	0	0	0	0	-0.003	t	9753	DPRVRGLY	Mus musculus
1	1.00	1	1	0	0	0	0	-0.003	t	7558	DAKDGKEIDTKHLPSTC	Oryctolagus cuniculus
1	1.00	1	1	0	0	0	0	-0.003	t	6338	CGQPVTRTDDDTVA	Oryctolagus cuniculus
1	1.00	1	1	0.06	0.06	0.06	0.06	0.06	t	78746	MDTWNPPLGSTSQPCLFQTP	Homo sapiens
1	1.00	1	1	0	0	0	0.006	0	t	110938	LYIKGSGSTANLASSNYFPT	Homo sapiens
1	1.00	1	1	0	0	0	0.006	0	t	110965	PNNNKILVPKVSGLQYRVFR	Homo sapiens
1	1.00	1	1	0.02	0.02	0.013	0.02	0.02	t	7005	CSGPTTIRGQFS	Mus musculus
1	1.00	1	1	0.02	0.02	0.013	0.02	0.02	t	7005	CSGPTTIRGQFS	Mus musculus
1	1.00	1	1	0	0.002	0	0.001	-0.002	t	54860	RMAWDMM	Homo sapiens
1	1.00	1	1	0.04	0.048	0.04	0.04	0.038	t	156866	FGSSSAG	Gallus gallus
1	1.00	1	1	0.07	0.07	0.07	0.07	0.07	t	125799	DDDRYSDL	Oryctolagus cuniculus
1	1.00	1	1	0.08	0.082	0.069	0.081	0.08	t	60441	SQGISDDDNDSAVAEFFQSF	Homo sapiens
1	1.00	1	1	0.01	0.01	0.003	0.02	0.012	t	106990	VYATRSSAVRLRSSVP	Homo sapiens
1	1.00	1	1	0.01	0.008	0.005	0.011	0.01	t	123144	ENPTMAGYR	Homo sapiens

1	1.00	1	1	0.01	0.012	0.01	0.011	0.009	t	51483	QLPQGTTLPKGFYAE	Homo sapiens
1	1.00	1	1	-0.04	-0.04	-0.044	-0.037	-0.04	t	48864	PPPPPPPPPPPPPPPPPPP	Homo sapiens
1	1.00	1	1	0	0	-0.008	0	0	t	106715	PSKECCSGTKKL	Homo sapiens
1	1.00	1	1	-0.02	-0.018	-0.02	-0.02	-0.022	t	122886	RGTDEYFIRKPP	Rattus norvegicus
1	1.00	1	1	-0.02	-0.018	-0.03	-0.017	-0.02	t	73298	WYTTVPKYVATQGYL	Homo sapiens
1	1.00	1	1	0	0	0	0	0	t	13525	ENMYRYPNQVYY	Mus musculus
1	1.00	1	1	0	0	0	0	0	t	13525	ENMYRYPNQVYY	Mus musculus
1	1.00	1	1	0.03	0.032	0.024	0.031	0.031	t	144925	NETIYNTTLK	Homo sapiens
1	1.00	1	1	-0.03	-0.034	-0.019	-0.031	-0.032	t	26926	IKVFG	Oryctolagus cuniculus
1	1.00	1	1	0.04	0.036	0.04	0.039	0.04	t	46348	NVDENANA	Oryctolagus cuniculus
1	1.00	1	1	0.06	0.062	0.056	0.057	0.06	t	11927	EEVVGEEKLVSEEIVT	Homo sapiens
1	1.00	1	1	0.02	0.021	0.02	0.019	0.02	t	57147	SCTVTREDGT	Oryctolagus cuniculus
1	1.00	1	1	-0.04	-0.036	-0.036	-0.033	-0.04	t	144614	DMMYGGMRGM	Homo sapiens
1	1.00	1	1	-0.01	-0.008	-0.005	-0.009	-0.011	t	39883	LTERLY	Homo sapiens

1	1.00	1	1	0.02	0.023	0.015	0.02	0.021	t	141567	TQDGLYHNKN	Mus musculus
1	1.00	1	1	-0.01	-0.01	-0.01	-0.001	-0.012	t	131815	AHELWKVPLPPKNIT	Homo sapiens
1	1.00	1	1	0.06	0.06	0.06	0.06	0.06	t	48518	PLVAEEDE	Homo sapiens
1	1.00	1	1	-0.01	-0.008	-0.01	-0.003	-0.01	t	130737	ASAISVAR	Homo sapiens
1	1.00	1	1	0	0	-0.01	-0.003	-0.002	t	69619	VLLPDE	Homo sapiens
1	1.00	1	1	0.03	0.032	0.019	0.031	0.03	t	110589	GDICNTMHYTNWTHIYICEE	Homo sapiens
1	1.00	1	1	0	-0.001	0.005	-0.003	0	t	73991	YGFQYS	Homo sapiens
1	1.00	1	1	0.05	0.05	0.039	0.053	0.052	t	50746	QFDEMEECSRHIPYLAEGQQ	Homo sapiens
1	1.00	1	1	-0.01	-0.013	-0.021	-0.01	-0.006	t	133629	LPLRF	Oryctolagus cuniculus
1	1.00	1	1	0.01	0.01	0.01	0.01	0.01	t	42694	MSTNPKPQRKTK	Homo sapiens
1	1.00	1	1	0	-0.003	-0.005	0	-0.001	t	138157	DPNAPPPPADPNA	Oryctolagus cuniculus
1	1.00	1	1	0.03	0.026	0.029	0.029	0.029	t	110862	GGPTLVSLHELPAETP	Oryctolagus cuniculus
1	1.00	1	1	0.01	0.01	0.006	0.015	0.009	t	55670	RRPFFHPVGEADYFEYHQEG	Homo sapiens
1	1.00	1	1	-0.02	-0.016	-0.011	-0.013	-0.021	t	119547	YKKGARPIQ	Homo sapiens
1	1.00	1	1	0	0	0	0	0	t	43703	NENLLRFFVA	Homo sapiens
1	1.00	1	1	-0.03	-0.032	-0.025	-0.031	-0.03	t	104139	MWNDFW	Mus musculus

1	1.00	1	1	-0.01	-0.01	-0.01	-0.01	-0.011	t	55071	RNTNRRPQDVKF	Homo sapiens
1	1.00	1	1	0.02	0.024	0.006	0.02	0.022	t	45931	NSNNKEYT	Cavia porcellus
1	1.00	1	1	0	0	0	0	0	t	51505	QLQPFPQP	Homo sapiens
1	1.00	1	1	0.05	0.046	0.05	0.049	0.05	t	137888	GTEYTVEV	Oryctolagus cuniculus
1	1.00	1	1	-0.01	-0.006	-0.016	-0.01	-0.01	t	22835	GTSMDDEADRYF	Capra hircus
1	1.00	1	1	-0.02	-0.017	-0.023	-0.02	-0.022	t	66841	TTTCFRDTDTGK	Mus musculus
1	1.00	1	1	0.01	0.01	0.004	0.01	0.01	t	55751	RRTQSMGIQL	Mus musculus
1	1.00	1	1	0.03	0.028	0.023	0.023	0.027	t	108202	VVSHFND	Mus musculus
1	1.00	1	1	0	0.002	-0.007	-0.003	-0.001	t	118387	GPKGRTVIIEQSW	Homo sapiens
1	1.00	1	1	-0.03	-0.028	-0.029	-0.029	-0.031	t	63082	TCKKNM	Homo sapiens
1	1.00	1	1	-0.02	-0.018	-0.02	-0.017	-0.02	t	131358	APSVTLF	Homo sapiens
1	1.00	1	1	0.01	0.01	0.008	0.01	0.008	t	97282	ESNNL	Oryctolagus cuniculus
1	1.00	1	1	0.05	0.048	0.048	0.049	0.053	t	103117	DTDY	Mus musculus
1	1.00	1	1	0.02	0.008	0.011	0.02	0.02	t	66741	TTNQALKK	Macaca mulatta

1	1.00	1	1	0.02	0.005	0.011	0.02	0.02	t	66741	TTNQALKK	Macaca mulatta
1	1.00	1	1	-0.01	-0.008	-0.01	-0.002	-0.01	t	110938	LYIKGSGSTANLASSNYFPT	Homo sapiens
1	1.00	1	1	-0.01	-0.008	-0.01	-0.002	-0.01	t	110938	LYIKGSGSTANLASSNYFPT	Homo sapiens
1	1.00	1	1	0	0.002	-0.005	0.003	0	t	20391	GIPDGWP	Felis catus
1	1.00	1	1	0.03	0.028	0.041	0.033	0.028	t	111486	LIEESAIINAGAP	Mus musculus
1	1.00	1	1	-0.02	-0.016	-0.016	-0.013	-0.02	t	144609	DEKLRDYIWN	Homo sapiens
1	1.00	1	1	0.01	0.01	0.001	0.004	0.008	t	95334	EDVPQPPVSQFH	Homo sapiens
1	1.00	1	1	0.02	0.018	0.013	0.023	0.021	t	106947	VATWLNPDPSQK	Mus musculus
1	1.00	1	1	-0.02	-0.017	-0.03	-0.02	-0.02	t	45492	NPTIAG	Mus musculus
1	1.00	1	1	-0.02	-0.017	-0.03	-0.02	-0.02	t	45492	NPTIAG	Mus musculus
1	1.00	1	1	-0.02	-0.017	-0.03	-0.02	-0.02	t	45492	NPTIAG	Mus musculus
1	1.00	1	1	-0.01	-0.01	-0.01	-0.01	-0.01	t	96239	EKPTPPVKPT	Homo sapiens(human)
1	1.00	1	1	0.01	0.008	0.014	0.007	0.008	t	25002	HTVTTTTK	Mus musculus
1	1.00	1	1	-0.08	-0.078	-0.087	-0.077	-0.08	t	135961	MRRPRRPGGLGG	Homo sapiens

1	1.00	1	1	0.05	0.048	0.045	0.049	0.05	t	51072	QIGNTVTP TVTFTMD GDTMT	Mus musculu s
1	1.00	1	1	0.05	0.048	0.045	0.049	0.05	t	51072	QIGNTVTP TVTFTMD GDTMT	Mus musculu s
1	1.00	1	1	0.01	0.014	0.01	0.013	0.01	t	117736	AEMKSLEE KIGCLLK	Homo sapiens
1	1.00	1	1	-0.01	-0.01	-0.01	-0.01	-0.01	t	121708	PKPEPKPK	Homo sapiens
1	1.00	1	1	0.01	0.01	0.01	0.01	0.009	t	11814	EENVEENV EENV	Oryctola gus cuniculu s
1	1.00	1	1	0.01	0.013	0.01	0.01	0.008	t	140933	YDITGEYV QILDAHAA YLNA	Mus musculu s
1	1.00	1	1	-0.05	-0.048	-0.05	-0.043	-0.051	t	117229	RHGFLPRH RD	Homo sapiens
1	1.00	1	1	0	-0.004	0	0.003	-0.002	t	53334	RDEDSYER DPYSPSQ	Oryctola gus cuniculu s
1	1.00	1	1	0	-0.002	0	0.003	-0.002	t	31885	KLGKFGAA RV	Mus musculu s
1	1.00	1	1	-0.04	-0.038	-0.047	-0.037	-0.039	t	144652	GMRGMK GLVY	Homo sapiens
1	1.00	1	1	0.01	0.012	0.003	0.009	0.01	t	73590	YDTADII	Felis catus
1	1.00	1	1	0.01	0.014	0.01	0.017	0.01	t	132036	QVNTNKG GGVWESG A	Homo sapiens
1	1.00	1	1	0.01	0.012	0.003	0.011	0.009	t	39732	LSVDLSLTV ELKIKI	Homo sapiens
1	1.00	1	1	0.01	0.01	0.01	0.01	0.008	t	32807	KPNLSSKR SELSQLS	Mus musculu s

1	1.00	1	1	-0.02	-0.02	-0.032	-0.02	-0.02	t	78452	LNVAHQLA	Mus musculus
1	1.00	1	1	0.01	0.008	0.011	0.007	0.01	t	55293	RPQDVKFP	Mus musculus
1	1.00	1	1	-0.01	-0.01	-0.009	-0.01	-0.01	t	76606	YYSPAF	Mus musculus
1	1.00	1	1	0.02	0.016	0.009	0.017	0.02	t	20562	GKFLEGA	Oryctolagus cuniculus
1	1.00	1	1	0.02	0.018	0.009	0.017	0.02	t	20562	GKFLEGA	Mus musculus
1	1.00	1	1	-0.01	-0.01	-0.018	-0.01	-0.01	t	86551	MTAEPKTIVL	Homo sapiens
1	1.00	1	1	0.02	0.017	0.02	0.02	0.02	t	119923	GYISKAQEYFN	Oryctolagus cuniculus
1	1.00	1	1	0.01	0.01	0.01	0.01	0.009	t	153474	TLITHIAPYIQT	Mus musculus
1	1.00	1	1	-0.01	-0.006	-0.013	-0.003	-0.01	t	124068	ELMALHTGQSLEQIER	Homo sapiens
1	1.00	1	1	0.05	0.05	0.047	0.05	0.05	t	20047	GGTEIKYNGE	Mus musculus
1	1.00	1	1	0	0.004	-0.011	0	0	t	110562	DIILECVYCKQQLLRREVYD	Cavia porcellus
1	1.00	1	1	-0.02	-0.016	-0.031	-0.02	-0.019	t	93332	LSSLQFARAA	Ovis aries
1	1.00	1	1	0.04	0.038	0.04	0.039	0.04	t	110877	GTVGENVPDDLYIKG	Mus musculus
1	1.00	1	1	0	0.002	-0.009	-0.002	-0.001	t	110828	EIDGPAGQAEPDRAHYNI	Homo sapiens

1	1.00	1	1	0.01	0.01	0.01	0.01	0.01	t	51505	QLQPFPQP	Homo sapiens
1	1.00	1	1	-0.03	-0.031	-0.03	-0.03	-0.031	t	22753	GTLTWSGK	Anas platyrhynchos
1	1.00	1	1	-0.02	-0.018	-0.022	-0.013	-0.02	t	133871	LAKLGGAGQAARAGT	Homo sapiens
1	1.00	1	1	-0.02	-0.021	-0.029	-0.021	-0.021	t	52891	QYDSNGTKLE	Mus musculus
1	1.00	1	1	-0.03	-0.032	-0.038	-0.031	-0.03	t	27416	ILYAVFK	Mus musculus
1	1.00	1	1	0	0	-0.016	0	0.001	t	140573	LCRSACVIYIKYSSA	Mus musculus
1	1.00	1	1	-0.03	-0.028	-0.03	-0.023	-0.029	t	113321	DENPVVHFFKNIVTPRT	Homo sapiens
1	1.00	1	1	-0.03	-0.028	-0.03	-0.023	-0.029	t	113321	DENPVVHFFKNIVTPRT	Homo sapiens
1	1.00	1	1	-0.03	-0.033	-0.037	-0.03	-0.032	t	41610	MGQHPAKSMDVRR	Oryctolagus cuniculus
1	1.00	1	1	-0.05	-0.053	-0.066	-0.05	-0.048	t	65905	TQNRQIDR	Oryctolagus cuniculus
1	1.00	1	1	-0.02	-0.017	-0.02	-0.02	-0.02	t	6276	CGAVIDDC	Mus musculus
1	1.00	1	1	-0.02	-0.017	-0.02	-0.02	-0.02	t	6276	CGAVIDDC	Mus musculus
1	1.00	1	1	0.06	0.06	0.06	0.06	0.059	t	112278	QSVLYSS	Homo sapiens
1	1.00	1	1	0.01	0.012	0.003	0.013	0.01	t	133787	CQALVRMLAKKPGWK	Homo sapiens
1	1.00	1	1	-0.01	-0.013	-0.018	-0.01	-0.011	t	94945	TLGASSGYLKGN	Oryctolagus cuniculus

1	1.00	1	1	-0.01	-0.01	-0.017	-0.01	-0.013	t	133577	FVVDMMERLRISQKWVRVA	Mus musculus
1	1.00	1	1	0	0	0	0.006	0	t	108125	SHQESTRGRSRGRSGRSGS	Homo sapiens
1	1.00	1	1	0	0	0	0.006	0	t	65423	TNSHVPILQERPPLE	Homo sapiens
1	1.00	1	1	-0.01	-0.008	-0.012	-0.003	-0.01	t	48779	PPFSLSPVPTLGSRSRR	Homo sapiens
1	1.00	1	1	-0.01	-0.01	-0.015	-0.003	-0.01	t	117972	LAVTKYKQRNGWSHK	Homo sapiens
1	1.00	1	1	0.02	0.017	0.015	0.02	0.02	t	25587	IDIDLCDICTNTMDV	Oryctolagus cuniculus
1	1.00	1	1	0.02	0.02	0.015	0.02	0.02	t	25587	IDIDLCDICTNTMDV	Mus musculus
1	1.00	1	1	-0.04	-0.038	-0.04	-0.033	-0.04	t	144619	DYIWNTLNSG	Homo sapiens
1	1.00	1	1	0.01	0.007	-0.005	0.003	0.01	t	104051	LPSDPIGGKSNMHFMNTL	Oryctolagus cuniculus
1	1.00	1	1	0	0.003	0.007	0	-0.002	t	78695	KDTNNNLYNGK	Mus musculus
1	1.00	1	1	0.01	0.012	0.01	0.007	0.01	t	43255	NANPNVDP	Homo sapiens
1	1.00	1	1	0.05	0.05	0.041	0.05	0.05	t	135920	ETSLSAAGNSII	Homo sapiens
1	1.00	1	1	0	0	-0.015	0.001	0.001	t	78896	YTKDTNNNLTL	Mus musculus

1	1.00	1	1	0.05	0.052	0.05	0.051	0.05	t	98786	GASEL	Homo sapiens
1	1.00	1	1	0.02	0.02	0.018	0.02	0.02	t	66343	TSNPLGFF PDHQLDP	Mus musculu s
1	1.00	1	1	0.04	0.04	0.04	0.04	0.04	t	134781	RGDGPFRL GG	Homo sapiens
1	1.00	1	1	0.01	0.012	0.015	0.011	0.01	t	67450	TYRGFTSL	Homo sapiens
1	1.00	1	1	0	0	-0.009	0	0	t	73611	YDYQHKE G	Homo sapiens
1	1.00	1	1	0	0	0	0	-0.003	t	115434	QEKNMAI NPSKENLC STFCK	Homo sapiens
1	1.00	1	1	-0.04	-0.036	-0.039	-0.033	-0.042	t	123985	CPCGSCCS WWKWLL GLLL	Homo sapiens
1	1.00	1	1	0.02	0.02	0.013	0.021	0.021	t	106357	DTWEPF	Mus musculu s
1	1.00	1	1	0	0	-0.003	0	0.002	t	137770	TLARSFIICI QM	Mus musculu s
1	1.00	1	1	0.02	0.022	0.021	0.017	0.02	t	7713	DCLLCAYSI EF	Homo sapiens
1	1.00	1	1	0.04	0.04	0.031	0.04	0.04	t	135958	LTAVETGH TSQV	Homo sapiens
1	1.00	1	1	-0.02	-0.022	-0.03	-0.017	-0.018	t	52107	QQPQRPF AQQ	Mus musculu s
1	1.00	1	1	0.04	0.04	0.031	0.043	0.04	t	22708	GTIINQDP DKILTYI	Homo sapiens
1	1.00	1	1	-0.03	-0.036	-0.033	-0.03	-0.03	t	31743	KLAAAVLN	Oryctola gus cuniculu s
1	1.00	1	1	-0.04	-0.036	-0.058	-0.039	-0.038	t	22809	GTSAPSGS GTSPSSR	Homo sapiens
1	1.00	1	1	0	0	0	0.003	-0.002	t	36124	LGGKPAIV PDKEVLYQ QYDE	Homo sapiens

1	1.00	1	1	0.01	0.01	0.01	0.01	0.011	t	115278	GYGGVSLP EW	Homo sapiens
1	1.00	1	1	0	0	0	0	0	t	135893	AFLQDVM NILLQ	Homo sapiens
1	1.00	1	1	0.02	0.02	-0.001	0.014	0.023	t	47747	PGSANKPK DELDYEND	Homo sapiens
1	1.00	1	1	0.02	0.022	0.015	0.021	0.02	t	141926	ILAALSEAA NKIEN	Homo sapiens
1	1.00	1	1	0.02	0.022	0.015	0.021	0.02	t	141926	ILAALSEAA NKIEN	Homo sapiens
1	1.00	1	1	-0.02	-0.018	-0.026	-0.019	-0.02	t	47239	PEARQA	Homo sapiens
1	1.00	1	1	0.02	0.02	0.02	0.02	0.02	t	153380	ETELTIAAL KYV	Mus musculu s
1	1.00	1	1	0	0.002	-0.007	0.003	0	t	116927	AVCRTSM CSIQSAPP	Homo sapiens
1	1.00	1	1	0	-0.002	-0.002	-0.001	0	t	10835	DWVKGFP IDSSC	Mus musculu s
1	1.00	1	1	-0.02	-0.018	-0.017	-0.017	-0.02	t	94626	NPTCWAI CKRIP	Homo sapiens
1	1.00	1	1	0.02	0.02	0.022	0.02	0.019	t	4701	ASQKIQLI	Mus musculu s
1	1.00	1	1	-0.05	-0.048	-0.05	-0.043	-0.05	t	117602	PKVKSKKR EAVAGRG	Homo sapiens
1	1.00	1	1	-0.05	-0.048	-0.05	-0.043	-0.05	t	117603	PKVKSKKR EAVAGRG	Homo sapiens
1	1.00	1	1	0.04	0.038	0.04	0.041	0.04	t	149435	QPQYSQP QQP	Mus musculu s
1	1.00	1	1	0.01	0.01	0.01	0.01	0.009	t	139616	SYNRGDST FESKSYK	Homo sapiens
1	1.00	1	1	0.02	0.02	0.02	0.02	0.021	t	115278	GYGGVSLP EW	Homo sapiens

1	1.00	1	1	0	0.004	0.002	0.007	0	t	433	AASGPKVVIDGKDQNVTGSV	Homo sapiens
1	1.00	1	1	-0.01	-0.01	-0.014	-0.01	-0.012	t	49070	PQPQQQFPQP	Mus musculus
1	1.00	1	1	0	0	0.009	0.007	0	t	65050	TLRVYK	Homo sapiens
1	1.00	1	1	0.01	0.008	0.015	0.007	0.008	t	25002	HTVTTTTK	Mus musculus
1	1.00	1	1	-0.02	-0.02	-0.02	-0.02	-0.02	t	46825	PAIIPDREV	Homo sapiens
1	1.00	1	1	0	-0.002	0	-0.002	-0.004	t	138166	FHEFWPT	Mus musculus
1	1.00	1	1	0	-0.002	0	-0.002	-0.004	t	138238	SMLDTPT	Mus musculus
1	1.00	1	1	0	-0.002	0	-0.002	-0.004	t	138189	HILGSTA	Mus musculus
1	1.00	1	1	0	-0.002	0	-0.002	-0.004	t	138202	LPTITNT	Mus musculus
1	1.00	1	1	0	0.002	0	-0.002	-0.004	t	138191	HPTWSSL	MD
1	1.00	1	1	0	-0.002	0	-0.002	-0.004	t	138192	HQTPLST	Mus musculus
1	1.00	1	1	0.01	0.01	0.01	0.013	0.01	t	42696	MSTNPKPQRKTKRNT	Homo sapiens
1	1.00	1	1	0	0	0	0.009	-0.002	t	131889	GKTFTNGWKSTEEED	Homo sapiens
1	1.00	1	1	0.01	0.01	0.012	0.01	0.008	t	7230	CTTPAQGNSMFPSC	Mus musculus
1	1.00	1	1	-0.02	-0.018	-0.031	-0.019	-0.02	t	110602	GLKAKPKFTLGKRKATPTT	Homo sapiens
1	1.00	1	1	-0.02	-0.016	-0.009	-0.013	-0.02	t	138171	FRVVSVL	Homo sapiens
1	1.00	1	1	-0.01	-0.01	-0.017	-0.01	-0.009	t	46859	PALTAVETGATNPL	Homo sapiens

1	1.00	1	1	0.01	0.012	0.004	0.011	0.01	t	133618	KKLLVRGL YRISFTTYK	Homo sapiens
1	1.00	1	1	0.05	0.05	0.046	0.05	0.05	t	36805	LKASAA	Homo sapiens
1	1.00	1	1	0	0	0	0	-0.003	t	150874	NWFDISK WLWYI	Homo sapiens
1	1.00	1	1	-0.02	-0.023	-0.018	-0.02	-0.022	t	127927	AKPAPPKP EPKPKKA	Oryctola gus cuniculu s
1	1.00	1	1	0	-0.002	-0.003	0.003	0	t	18955	GDDPLLG NQSLL	Mus musculu s
1	1.00	1	1	0.01	0.014	0.007	0.011	0.01	t	34639	KYVTGIN	Homo sapiens
1	1.00	1	1	0.02	0.018	0.022	0.023	0.018	t	55785	RRYQKSTE L	Mus musculu s
1	1.00	1	1	0.02	0.02	0.02	0.02	0.02	t	156919	MLHTSM	Gallus gallus
1	1.00	1	1	-0.02	-0.022	-0.04	-0.027	-0.018	t	44555	NKNDNKN D	Mus musculu s
1	1.00	1	1	0	0	0	0	-0.002	t	73785	YEVRNVSG IYHVT	Homo sapiens
1	1.00	1	1	0	0.004	0	0	-0.003	t	70387	VPNLRGDL QV	Cavia porcellu s
1	1.00	1	1	0.02	0.019	0.028	0.021	0.02	t	67345	TYGLASL	Homo sapiens
1	1.00	1	1	-0.04	-0.04	-0.035	-0.037	-0.04	t	46817	PAHYANYL P	Homo sapiens
1	1.00	1	1	-0.01	-0.015	-0.006	-0.006	-0.012	t	34170	KVPTLK	Bos taurus

1	1.00	1	1	0.05	0.052	0.048	0.051	0.048	t	22755	GTLVKTITDDQIEV	Homo sapiens
1	1.00	1	1	0.02	0.023	0.02	0.02	0.019	t	107517	LSTNEVAM	Mus musculus
1	1.00	1	1	-0.02	-0.023	-0.026	-0.02	-0.022	t	100351	NQILKPKLISYTLPVVG	Oryctolagus cuniculus
1	1.00	1	1	-0.05	-0.048	-0.045	-0.043	-0.05	t	117169	RYHRFKELSTLRR	Homo sapiens
1	1.00	1	1	0	0	0	0	-0.001	t	121287	EYFIRK	Rattus norvegicus
1	1.00	1	1	0	0	0.003	0.001	0	t	64697	TKVTLDGRPL	Homo sapiens
1	1.00	1	1	0	-0.002	-0.005	-0.001	0	t	78708	KLVEYCDFLTTFVHAKKKE	Mus musculus
1	1.00	1	1	0	-0.004	-0.005	-0.001	0	t	78708	KLVEYCDFLTTFVHAKKKE	Oryctolagus cuniculus
1	1.00	1	1	0	0	0	-0.006	-0.006	t	113879	SQRSQDEN	Rattus norvegicus
1	1.00	1	1	0	0	-0.013	-0.007	0	t	141932	KDKKDDKYTKILAA	Homo sapiens
1	1.00	1	1	0.06	0.06	0.06	0.06	0.06	t	135981	QYVISQEMERIP	Homo sapiens
1	1.00	1	1	0.01	0.01	0.01	0.01	0.009	t	43729	NEREDERT	Oryctolagus cuniculus
1	1.00	1	1	0	0.004	0	0.007	-0.001	t	132031	QPPAQPLSSSQPNLR	Homo sapiens

1	1.00	1	1	0.03	0.032	0.025	0.031	0.029	t	75851	YSPGQR	Homo sapiens
1	1.00	1	1	-0.01	-0.01	-0.01	-0.004	-0.01	t	118451	KGIIDPTKVVRTA	Homo sapiens
1	1.00	1	1	0.02	0.024	0.02	0.017	0.018	t	106457	GIVDSKLPPIDV	Homo sapiens
1	1.00	1	1	0.02	0.019	0.011	0.02	0.021	t	49690	PTLDHVLD	Anas platyrhynchos
1	1.00	1	1	0.05	0.05	0.035	0.05	0.052	t	17333	FPLQPQQPFP	Mus musculus
1	1.00	1	1	-0.01	-0.01	-0.005	-0.001	-0.01	t	133798	DKIRGEGMRYIIILD	Homo sapiens
1	1.00	1	1	-0.01	-0.01	-0.01	-0.003	-0.01	t	125416	FYNLSKVTHIEIRNTRNLTY	Homo sapiens
1	1.00	1	1	-0.03	-0.03	-0.03	-0.03	-0.032	t	119658	TYGIVLLSGV	Homo sapiens
1	1.00	1	1	0.05	0.05	0.05	0.053	0.05	t	111041	TIGRLQDEIQNMKEEMARHL	Mus musculus
1	1.00	1	1	0.05	0.05	0.05	0.053	0.05	t	111041	TIGRLQDEIQNMKEEMARHL	Mus musculus
1	1.00	1	1	-0.04	-0.038	-0.033	-0.033	-0.04	t	142058	VLGNKTW	Homo sapiens
1	1.00	1	1	0.03	0.033	0.014	0.036	0.037	t	136062	FGISETQAEAILE	Mus musculus
1	1.00	1	1	0.01	0.01	0.01	0.01	0.01	t	129066	KFKSV	Homo sapiens
1	1.00	1	1	0.03	0.034	0.029	0.03	0.029	t	56019	RTAPGPGE	Homo sapiens
1	1.00	1	1	0	0.004	0.007	0.003	-0.005	t	135449	CATLRVYKGG	Homo sapiens
1	1.00	1	1	0	0	0	0.002	0	t	67492	TYTWGENEKDVFLL	Homo sapiens

1	1.00	1	1	0.03	0.03	0.023	0.037	0.03	t	117015	KEQFLDGDGWTSRWIESK	Homo sapiens
1	1.00	1	1	0	0.014	0	0.003	0	t	42795	MTEQQWNFAGIEAAAS	Homo sapiens
1	1.00	1	1	0.03	0.03	0.028	0.03	0.029	t	66344	TSNQRGVGSTVVILD	Sus scrofa
1	1.00	1	1	0.02	0.016	0.018	0.023	0.021	t	99259	FTVKVGDKELF	Oryctolagus cuniculus
1	1.00	1	1	0.04	0.04	0.035	0.033	0.038	t	142382	AEDAAEEAKEAAKK	Homo sapiens
1	1.00	1	1	0.05	0.046	0.035	0.05	0.052	t	73649	YEEPNDGEERQS	Bos taurus
1	1.00	1	1	0.05	0.054	0.035	0.05	0.052	t	73649	YEEPNDGEERQS	Capra hircus
1	1.00	1	1	0	0.002	-0.007	-0.003	-0.003	t	106513	HKARLERPQI	Homo sapiens
1	1.00	1	1	-0.01	-0.013	-0.01	-0.01	-0.01	t	72484	WGNGCGLFG	Mus musculus
1	1.00	1	1	-0.03	-0.03	-0.037	-0.036	-0.032	t	49368	PSFSDIPNPI	Homo sapiens
1	1.00	1	1	0	-0.003	-0.01	0	0	t	140908	TVYFGFDKYDITGEYVQILD	Oryctolagus cuniculus
1	1.00	1	1	0.01	0.013	-0.001	0.01	0.011	t	43191	NAKATYEAALKQYEADLAA	Mus musculus
1	1.00	1	1	0.04	0.038	0.033	0.039	0.041	t	19998	GGRGDSG	Mus musculus
1	1.00	1	1	-0.01	-0.01	-0.025	-0.01	-0.01	t	43226	NANANPN	Mus musculus
1	1.00	1	1	0.01	0.004	0.01	0.01	0.01	t	123259	GQPPYMPP	Oryctolagus cuniculus

1	1.00	1	1	0.01	0.01	0.01	0.01	0.01	t	73432	YARFMDTDPD	Mus musculus
1	1.00	1	1	0	0.002	-0.009	-0.003	-0.002	t	60019	SPDRKRRADEEEEYD	Homo sapiens
1	1.00	1	1	0.02	0.02	0.02	0.02	0.02	t	134050	FKPPKY	Homo sapiens
1	1.00	1	1	0.05	0.052	0.043	0.053	0.052	t	142448	NKYRGTAGNALMDGASQL	Homo sapiens
1	1.00	1	1	-0.03	-0.03	-0.03	-0.03	-0.03	t	79219	FWLAVR	Mus musculus
1	1.00	1	1	-0.01	-0.01	-0.017	-0.01	-0.012	t	106375	EDHAGTYGLGDRK	Mus musculus
1	1.00	1	1	0	-0.004	-0.003	-0.001	-0.003	t	150860	IYLKNVLKPDEKIH	Oryctolagus cuniculus
1	1.00	1	1	0.02	0.02	0.02	0.02	0.02	t	149048	FSTQKSI	Sus scrofa
1	1.00	1	1	0.03	0.03	0.03	0.03	0.03	t	44881	NLSSKA	Homo sapiens
1	1.00	1	1	-0.02	-0.02	-0.012	-0.02	-0.02	t	115096	PPSLLFLP	Mus musculus
1	1.00	1	1	-0.02	-0.02	-0.025	-0.02	-0.02	t	112507	FAVLKLMGRGTK	Rattus norvegicus
1	1.00	1	1	0.01	0.012	0.002	0.011	0.009	t	14208	ESLTTTSTALGKLQDVV	Homo sapiens
1	1.00	1	1	0.02	0.018	0.022	0.023	0.018	t	26076	IFGVDANTGNVL	Rattus norvegicus
1	1.00	1	1	0.01	0.01	0.001	0.01	0.012	t	68182	VEFPLDITAG	Mus musculus
1	1.00	1	1	0	0	0.002	0	-0.001	t	121773	QYVAMVADRLFLY	Rattus norvegicus
1	1.00	1	1	-0.01	-0.006	-0.025	-0.013	-0.01	t	111588	NLIQHVKGGTPKRPN	Homo sapiens

1	1.00	1	1	-0.01	-0.01	-0.015	-0.01	-0.012	t	131015	QDRRLDEN	Oryctolagus cuniculus
1	1.00	1	1	0	0.004	0	0.003	0	t	118568	QKIGIEIIKRTLK	Homo sapiens
1	1.00	1	1	0	0.002	-0.002	0.001	-0.001	t	76170	YTRTWSEVDA	Homo sapiens
1	1.00	1	1	0.05	0.056	0.057	0.053	0.052	t	78154	FEDETW	Equus caballus
1	1.00	1	1	0	0.002	0	0.003	0.002	t	8473	DGQPAGDR	Homo sapiens
1	1.00	1	1	0	0	0	0.008	0.001	t	117742	AKAQTDREDLRTLLRY	Homo sapiens
1	1.00	1	1	0	0	0	0.008	0.001	t	117773	CNSRQTDREDELI	Homo sapiens
1	1.00	1	1	0	-0.012	-0.002	0	0	t	64308	TIGKVSQK	Macaca mulatta
1	1.00	1	1	0	-0.012	-0.002	0	0	t	64308	TIGKVSQK	Macaca mulatta
1	1.00	1	1	0.01	0.014	0.005	0.007	0.01	t	48026	PKAFDEKLFT	Homo sapiens
1	1.00	1	1	-0.01	-0.01	0	-0.01	-0.012	t	95161	TYGKFCIKP	Mus musculus
1	1.00	1	1	-0.01	-0.008	-0.01	-0.007	-0.01	t	118189	TYYIKEQKLGL	Homo sapiens
1	1.00	1	1	-0.01	-0.012	-0.021	-0.011	-0.01	t	41840	MKIRLHTLLAVLTAAPLLLA	Mus musculus

1	1.00	1	1	-0.01	-0.012	-0.014	-0.011	-0.01	t	17136	FNCLGMSSRDFIEGA	Mus musculus
1	1.00	1	1	-0.01	-0.012	-0.014	-0.011	-0.01	t	17136	FNCLGMSSRDFIEGA	Mus musculus
1	1.00	1	1	-0.01	-0.007	-0.014	-0.011	-0.01	t	17136	FNCLGMSSRDFIEGA	Mus musculus
1	1.00	1	1	0.02	0.018	0.013	0.013	0.016	t	128612	FDSHY	Mus musculus
1	1.00	1	1	-0.01	-0.01	-0.01	-0.01	-0.01	t	134769	QIFNL	Mus musculus
1	1.00	1	1	-0.01	-0.01	-0.01	-0.01	-0.01	t	134769	QIFNL	Mus musculus
1	1.00	1	1	-0.01	-0.01	-0.01	-0.01	-0.01	t	134769	QIFNL	Mus musculus
1	1.00	1	1	-0.01	-0.012	-0.02	-0.013	-0.01	t	48858	PPPPNPNDPPPPNPND	Mus musculus
1	1.00	1	1	0.01	0.01	0.01	0.012	0.01	t	63540	TESTLSTALAELAT	Homo sapiens
1	1.00	1	1	-0.01	-0.012	-0.022	-0.01	-0.01	t	100559	TKVTSKVRIYMKPK	Homo sapiens
1	1.00	1	1	0	-0.002	0	0.006	0	t	21254	GMDGTQLDRW	Mus musculus
1	1.00	1	1	0	-0.002	0	0.006	0	t	21254	GMDGTQLDRW	Mus musculus
1	1.00	1	1	-0.01	-0.008	-0.012	-0.003	-0.009	t	119741	AIFKSYGEIIVTHFPFD	Homo sapiens
1	1.00	1	1	-0.01	-0.006	-0.012	-0.003	-0.009	t	119741	AIFKSYGEIIVTHFPFD	Homo sapiens
1	1.00	1	1	-0.01	-0.008	-0.012	-0.003	-0.009	t	119741	AIFKSYGEIIVTHFPFD	Homo sapiens

1	1.00	1	1	0.03	0.03	0.03	0.03	0.029	t	49533	PSTPGDNPLV	Homo sapiens
1	1.00	1	1	0.02	0.02	0.013	0.02	0.018	t	124720	SILPYGDSMDRIEK	Homo sapiens
1	1.00	1	1	-0.01	-0.007	-0.019	-0.01	-0.01	t	63756	TFTNKLKEKHTDLGK	Mus musculus
1	1.00	1	1	0	0	0	0.004	0	t	135959	LTRAYAKDVKFG	Homo sapiens
1	1.00	1	1	0	0	0	0.004	0	t	135922	FFRMVISNPAAT	Homo sapiens
1	1.00	1	1	0.02	0.024	0.018	0.027	0.022	t	131955	LPHGNALVRYSSKEE	Homo sapiens
1	1.00	1	1	-0.01	-0.01	-0.01	-0.01	-0.012	t	51084	QIISQQPFPL	Mus musculus
1	1.00	1	1	-0.01	-0.01	-0.022	-0.01	-0.01	t	114041	YGSLPQKAQGHRPQDEN	Rattus norvegicus
1	1.00	1	1	-0.01	-0.01	-0.022	-0.01	-0.01	t	114041	YGSLPQKAQGHRPQDEN	Rattus norvegicus
1	1.00	1	1	-0.01	-0.012	-0.022	-0.01	-0.01	t	114041	YGSLPQKAQGHRPQDEN	Oryctolagus cuniculus
1	1.00	1	1	0	-0.002	-0.005	-0.001	0	t	20137	GHGSETAWGT	Mus musculus
1	1.00	1	1	0.01	0.01	0.001	0.008	0.01	t	135936	IERAVVHVADTM	Homo sapiens
1	1.00	1	1	-0.01	-0.007	-0.003	-0.011	-0.012	t	33222	KRTISIWGT	Mus musculus
1	1.00	1	1	-0.01	-0.01	-0.01	-0.007	-0.01	t	76114	YTLLDR	Homo sapiens
1	1.00	1	1	-0.02	-0.02	-0.02	-0.02	-0.02	t	21758	GPRLGVRATR	Homo sapiens
1	1.00	1	1	-0.06	-0.056	-0.06	-0.053	-0.06	t	145639	WTPGVKRNGF	Homo sapiens

1	1.00	1	1	0.01	0.01	0.005	0.01	0.011	t	22303	GSDYEDRYYRENM	Mus musculus
1	1.00	1	1	0	0	-0.007	0	0	t	141523	ALEPPHNLPVP	Mus musculus
1	1.00	1	1	-0.01	-0.01	-0.024	-0.01	-0.009	t	78937	AYSDDFFVGRV	Mus musculus
1	1.00	1	1	-0.03	-0.032	-0.032	-0.027	-0.032	t	72922	WPACYCYGLPNWVKV	Mus musculus
1	1.00	1	1	-0.04	-0.038	-0.035	-0.047	-0.049	t	131022	QSGRKGADIM	Homo sapiens
1	1.00	1	1	-0.04	-0.038	-0.037	-0.037	-0.038	t	24694	HRMAWDMMM	Homo sapiens
1	1.00	1	1	0	-0.002	0	0.003	0	t	70459	VPQYLRWFEEWAE	Rattus norvegicus
1	1.00	1	1	0	0.003	-0.011	0	0	t	79867	INNLIVELIRGT	Mus musculus
1	1.00	1	1	0.02	0.01	0.014	0.021	0.02	t	55066	RNTIMASKTVGTAE	Homo sapiens
1	1.00	1	1	0.01	0.014	0.003	0.007	0.007	t	50172	PYSPSQDPYSPSQDP	Homo sapiens
1	1.00	1	1	0.03	0.03	0.03	0.03	0.03	t	112158	HQYLSSF	Homo sapiens
1	1.00	1	1	0.03	0.03	0.014	0.02	0.028	t	116691	DNAMDRADTLEQQNK	Homo sapiens
1	1.00	1	1	0.04	0.043	0.04	0.04	0.04	t	10757	DVTTLN	Mus musculus
1	1.00	1	1	-0.01	-0.008	-0.012	-0.009	-0.01	t	38632	LPPVADLPQPGLRR	Homo sapiens
1	1.00	1	1	-0.01	-0.008	-0.012	-0.009	-0.01	t	38632	LPPVADLPQPGLRR	Homo sapiens

1	1.00	1	1	0	0	-0.007	0	0	t	105763	EVHHQK	Mus musculus
1	1.00	1	1	-0.01	-0.01	-0.007	-0.01	-0.01	t	746	ADPSIMAK	Oryctolagus cuniculus
1	1.00	1	1	0	-0.001	-0.008	0	0	t	11353	EDINGIRRPK	Mus musculus
1	1.00	1	1	0.02	0.025	0.012	0.021	0.018	t	48307	PLENPSSLFDLVARIKNNLK	Homo sapiens
1	1.00	1	1	0.02	0.025	0.012	0.021	0.018	t	48307	PLENPSSLFDLVARIKNNLK	Homo sapiens
1	1.00	1	1	0.03	0.038	0.03	0.03	0.029	t	156965	SVQERYYAFWST	Gallus gallus
1	1.00	1	1	-0.02	-0.02	-0.031	-0.02	-0.02	t	37165	LLDKIYTSPLC	Mus musculus
1	1.00	1	1	-0.02	-0.018	-0.02	-0.019	-0.02	t	25101	HVTGGAA	Homo sapiens
1	1.00	1	1	0	0	-0.018	0	0.002	t	76248	YVDISLDAGQYVLVMKANSS	Oryctolagus cuniculus
1	1.00	1	1	0	0	0	0.001	-0.002	t	60116	SPLLGCIGSTCAE	Mus musculus
1	1.00	1	1	0	0	0	0.001	-0.002	t	40110	LTPLEELY	Mus musculus
1	1.00	1	1	0.01	0.01	0.007	0.01	0.01	t	22332	GSGASDLR	Homo sapiens
1	1.00	1	1	0.02	0.018	0	0.013	0.02	t	128090	QYYRLPMNAYRDFTSEPLNS	Mus musculus
1	1.00	1	1	-0.02	-0.022	-0.025	-0.021	-0.02	t	57941	SFVTRRYLSQLC	Mus musculus

1	1.00	1	1	0	0.002	0	-0.001	-0.003	t	136568	PYYRVFYKYD	Homo sapiens
1	1.00	1	1	0.02	0.02	0.02	0.02	0.018	t	145827	IIPKVEGK	Mus musculus
1	1.00	1	1	0.03	0.03	0.03	0.03	0.03	t	133398	TKEQTTFPP	Homo sapiens
1	1.00	1	1	0.01	0.01	0.017	0.01	0.008	t	106225	ADIWLTPDPTTP	Mus musculus
1	1.00	1	1	0.01	0.01	0.017	0.01	0.008	t	106225	ADIWLTPDPTTP	Mus musculus
1	1.00	1	1	0	0.002	-0.011	0	0.002	t	87725	QTEVAATLAYRF	Homo sapiens
1	1.00	1	1	-0.01	-0.01	-0.01	-0.01	-0.011	t	15981	FGGETFTPDWMMEV	Mus musculus
1	1.00	1	1	0.02	0.02	0.02	0.02	0.022	t	45637	NQVYYRP	Mus musculus
1	1.00	1	1	0.01	0.012	0.002	0.011	0.009	t	62872	TAGWTFGAGAALQIPFA	Homo sapiens
1	1.00	1	1	-0.01	-0.01	-0.017	-0.01	-0.012	t	106374	EDHAGTYGLGDRK	Mus musculus
1	1.00	1	1	0	0.004	-0.002	0.001	0	t	60614	SRAATLTPAA	Homo sapiens
1	1.00	1	1	0.04	0.036	0.044	0.043	0.04	t	97437	LEIQGEGQF	Oryctolagus cuniculus
1	1.00	1	1	0.03	0.026	0.033	0.027	0.028	t	15529	FEFPPPPTDE	Oryctolagus cuniculus
1	1.00	1	1	-0.02	-0.02	-0.02	-0.02	-0.02	t	140936	YLAGKGVDAGKL	Oryctolagus cuniculus
1	1.00	1	1	-0.02	-0.02	-0.027	-0.02	-0.02	t	121977	VHWKNQFDHY	Mus musculus
1	1.00	1	1	0.03	0.03	0.03	0.03	0.03	t	156858	DMSYYYALPTYT	Gallus gallus

1	1.00	1	1	-0.02	-0.018	-0.019	-0.013	-0.02	t	98435	RYPHYSSLIKPESSL	Homo sapiens
1	1.00	1	1	-0.02	-0.018	-0.026	-0.013	-0.02	t	118809	GGRPGAPGGSGSGPRHRDGV	Homo sapiens
1	1.00	1	1	0	0	0	0	-0.003	t	149298	ELDKWAS	Homo sapiens
1	1.00	1	1	0.03	0.034	0.03	0.037	0.03	t	117363	RPDAEYWNSQKDLLEQKRGR	Homo sapiens
1	1.00	1	1	0	-0.002	0.005	-0.001	0	t	4488	ASINKV	Mus musculus
1	1.00	1	1	-0.04	-0.037	-0.038	-0.04	-0.041	t	120189	RVYAYVFEHR	Mus musculus
1	1.00	1	1	-0.04	-0.04	-0.038	-0.04	-0.041	t	120189	RVYAYVFEHR	Mus musculus
1	1.00	1	1	-0.04	-0.04	-0.038	-0.04	-0.041	t	120189	RVYAYVFEHR	Oryctolagus cuniculus
1	1.00	1	1	-0.04	-0.04	-0.038	-0.04	-0.041	t	120189	RVYAYVFEHR	Mus musculus
1	1.00	1	1	-0.04	-0.043	-0.038	-0.04	-0.041	t	120189	RVYAYVFEHR	Oryctolagus cuniculus
1	1.00	1	1	-0.04	-0.04	-0.035	-0.037	-0.04	t	46817	PAHYANYLP	Homo sapiens
1	1.00	1	1	0.01	0.013	0.005	0.01	0.009	t	57062	SAYTPAYYTKDTNNNLTL	Mus musculus
1	1.00	1	1	0.01	0.014	0.005	0.01	0.009	t	57062	SAYTPAYYTKDTNNNLTL	Homo sapiens

1	1.00	1	1	-0.03	-0.026	-0.045	-0.03	-0.027	t	136080	RAGTDEGFG	Homo sapiens
1	1.00	1	1	0.02	0.016	0.011	0.019	0.02	t	47316	PEKAFRELAD	Oryctolagus cuniculus
1	1.00	1	1	0.01	0.01	0.01	0.01	0.008	t	117642	SKKREAVAGRGRG	Homo sapiens
1	1.00	1	1	0.01	0.012	0.012	0.011	0.01	t	66457	TSTFTSL	Homo sapiens
1	1.00	1	1	0	0	-0.011	0	0.002	t	17467	FQDMLNIS	Mus musculus
1	1.00	1	1	0.01	0.014	0.01	0.013	0.01	t	136081	RTTLQSDQEEIDY	Homo sapiens
1	1.00	1	1	0.08	0.076	0.08	0.077	0.077	t	134148	LQSDQEEIDY	Oryctolagus cuniculus
1	1.00	1	1	0.03	0.03	0.03	0.034	0.03	t	132260	EKIERRQQEV	Homo sapiens
1	1.00	1	1	-0.04	-0.042	-0.045	-0.039	-0.04	t	133836	GKKRKRSRKE	Mus musculus
1	1.00	1	1	-0.03	-0.03	-0.037	-0.03	-0.032	t	32233	KLVLSLCFLFSGCF	Homo sapiens
1	1.00	1	1	0.05	0.048	0.05	0.05	0.05	t	117835	EQEAKQKLEEDAEMK	Mus musculus
1	1.00	1	1	-0.01	-0.01	-0.012	-0.01	-0.013	t	151652	HWWRAESDEARRCYNDPKC	Mus musculus
1	1.00	1	1	0.01	0.006	0.002	0.007	0.01	t	70795	VRPATTLLADG	Oryctolagus cuniculus
1	1.00	1	1	0.01	0.012	0.004	0.011	0.01	t	56895	SAMILAAYHPQQFIYAGSLS	Homo sapiens
1	1.00	1	1	0.01	0.012	0.004	0.011	0.01	t	56895	SAMILAAYHPQQFIYAGSLS	Homo sapiens
1	1.00	1	1	-0.01	-0.01	-0.01	-0.006	-0.01	t	115603	ELKVEDPFYWVS	Homo sapiens

1	1.00	1	1	0.12	0.12	0.12	0.12	0.12	t	104496	GDD	Mus musculus
1	1.00	1	1	0.02	0.018	0.023	0.023	0.019	t	10691	DVPDYAS	Mus musculus
1	1.00	1	1	0.01	0.008	0.01	0.013	0.011	t	58371	SHITSYPAYFW	Mus musculus
1	1.00	1	1	0.05	0.048	0.043	0.053	0.051	t	106790	RSFTLASSETGVG	Mus musculus
1	1.00	1	1	-0.03	-0.034	-0.045	-0.027	-0.029	t	42274	MPGGMPGGMPGGMPGG	Oryctolagus cuniculus
1	1.00	1	1	-0.04	-0.037	-0.041	-0.04	-0.042	t	56075	RTHRQLY	Mus musculus
1	1.00	1	1	0.01	0.01	-0.001	0.011	0.01	t	134859	AGENLNTSKFKKNEF	Sus scrofa
1	1.00	1	1	-0.02	-0.02	-0.017	-0.02	-0.022	t	14202	ESLNTGWLAGLF	Rattus norvegicus
1	1.00	1	1	-0.02	-0.024	-0.026	-0.021	-0.02	t	12900	EKVSRRSNFEF	Oryctolagus cuniculus
1	1.00	1	1	0.02	0.021	0.021	0.02	0.02	t	57506	SEKSKCEENTMFQPY	Equus caballus
1	1.00	1	1	0	0	0	0.001	-0.002	t	24591	HQLDPAFGANSTNPD	Mus musculus
1	1.00	1	1	-0.02	-0.023	-0.027	-0.02	-0.022	t	108164	TLVCLATGFFPDHVEL	Oryctolagus cuniculus
1	1.00	1	1	0.04	0.045	0.038	0.037	0.038	t	157929	SFEDQGRR	Homo sapiens
1	1.00	1	1	0	0	-0.004	-0.001	0	t	106522	HVTPISKLMWFN	Mus musculus

1	1.00	1	1	-0.01	-0.008	-0.01	-0.007	-0.01	t	119578	FAIVKFTKVLLQYTGH	Homo sapiens
1	1.00	1	1	0.03	0.026	0.021	0.027	0.03	t	71902	VVYDYQHK	Oryctolagus cuniculus
1	1.00	1	1	-0.01	-0.02	-0.013	-0.009	-0.01	t	22217	GRTPFII	Homo sapiens
1	1.00	1	1	0.03	0.029	0.026	0.03	0.03	t	48900	PPQWKLQP	Anas platyrhynchos
1	1.00	1	1	0.01	0.012	0.002	0.007	0.01	t	36840	LKEPTNEDILPNHNP	Homo sapiens
1	1.00	1	1	0	0	-0.015	-0.001	0	t	1480	AGDSSTKNLIYTSTLCELNV	Mus musculus
1	1.00	1	1	0.03	0.03	0.035	0.033	0.03	t	720	ADLPQP	Homo sapiens
1	1.00	1	1	-0.02	-0.02	-0.02	-0.02	-0.02	t	6524	CKTC	Mus musculus
1	1.00	1	1	0	0	0	0.002	0	t	22123	GRLGTYRYVSSGER	Homo sapiens
1	1.00	1	1	0.01	0.01	0.005	0.01	0.01	t	114581	RNIGYIFFKNSTIDI	Homo sapiens
1	1.00	1	1	0.03	0.032	0.018	0.027	0.032	t	49858	PVESKVT	Homo sapiens
1	1.00	1	1	0.02	0.023	0.006	0.02	0.022	t	96674	NGDGNPREVIED	Mus musculus
1	1.00	1	1	0.02	0.02	0.012	0.02	0.02	t	41177	MAYRFNGIGVTQNVLYE	Mus musculus
1	1.00	1	1	-0.01	-0.009	-0.01	-0.007	-0.01	t	78244	PMWPME	Equus caballus

1	1.00	1	1	0	0.002	0	0.007	0	t	156675	GPRVVERHQSACKDS	Homo sapiens
1	1.00	1	1	-0.01	-0.012	-0.016	-0.011	-0.011	t	39182	LRRDLDASREAKKQVEKAL	Mus musculus
1	1.00	1	1	-0.02	-0.02	-0.025	-0.02	-0.02	t	58103	SGKGVSFQLVKLGVW	Homo sapiens
1	1.00	1	1	-0.03	-0.032	-0.036	-0.027	-0.031	t	58942	SKSSRC	Mus musculus
1	1.00	1	1	-0.02	-0.022	-0.02	-0.017	-0.02	t	22881	GTVVAVGPGR	Mus musculus
1	1.00	1	1	-0.01	-0.01	-0.017	-0.01	-0.009	t	46859	PALTAVETGATNPL	Homo sapiens
1	1.00	1	1	0.02	0.02	0.016	0.02	0.02	t	71298	VTFDGGKMVQ	Oryctolagus cuniculus
1	1.00	1	1	0	0	-0.016	0	0.002	t	76491	YYDGRRS	Mus musculus
1	1.00	1	1	0.02	0.02	0.02	0.02	0.022	t	75202	YNPLLVET	Homo sapiens
1	1.00	1	1	0.01	0.008	0.01	0.011	0.01	t	86748	NHIENDPLKI	Homo sapiens
1	1.00	1	1	0.05	0.05	0.05	0.05	0.049	t	120342	YNAEEVEFTFAVDND	Rattus norvegicus
1	1.00	1	1	-0.02	-0.02	-0.02	-0.02	-0.022	t	79213	FGMLPVPQQPMGPQ	Oryctolagus cuniculus
1	1.00	1	1	0.05	0.036	0.047	0.05	0.05	t	8371	DGEGAP	Marmota monax
1	1.00	1	1	0.02	0.018	0.02	0.019	0.018	t	47096	PDFYGDPLE	Mus musculus

1	1.00	1	1	0.01	0.014	0.009	0.017	0.012	t	130283	VRGVGGPS	Homo sapiens
1	1.00	1	1	-0.01	-0.01	-0.01	-0.01	-0.013	t	106941	TVKQLYTLYPAA	Mus musculus
1	1.00	1	1	0	0.003	0.003	0	-0.002	t	14749	EVKKKSDS	Mus musculus
1	1.00	1	1	0.01	0.01	0.01	0.01	0.01	t	105068	AEFDLD	Oryctolagus cuniculus
1	1.00	1	1	-0.03	-0.03	-0.03	-0.03	-0.03	t	145947	PPDIIPK	Mus musculus
1	1.00	1	1	-0.01	-0.01	-0.01	-0.006	-0.01	t	157127	CFNLFLNSQEKHY	Homo sapiens
1	1.00	1	1	0.02	0.02	0.02	0.02	0.018	t	100549	TEEKEFLKKL	Mus musculus
1	1.00	1	1	0.01	0.012	0.003	0.011	0.009	t	56796	SAGGQLFYSRPVVSANGE	Homo sapiens
1	1.00	1	1	0.04	0.042	0.049	0.047	0.04	t	112348	TEDLAVY	Homo sapiens
1	1.00	1	1	0	0	0	0	-0.003	t	132860	LKKLKPGLEKDF	Rattus norvegicus
1	1.00	1	1	0	0	0	0	-0.003	t	104461	EFRHDS	Mus musculus
1	1.00	1	1	0	0	0	0	-0.003	t	102344	DAEFR	Mus musculus
1	1.00	1	1	0	0	0	0	-0.003	t	106535	IFPSFLRPDPPL	Mus musculus
1	1.00	1	1	0	0	0	0	-0.003	t	106487	GTSTEVWLAPDP	Mus musculus
1	1.00	1	1	0	0	0	0	-0.003	t	105163	EFRHD	Mus musculus
1	1.00	1	1	0	0	0	0	-0.003	t	106515	HLPSFLRPDPPL	Mus musculus

1	1.00	1	1	0	0	0	0	-0.003	t	105090	AIIGLMVG GVVIA	Mus musculu s
1	1.00	1	1	0	0	0	0	-0.003	t	106225	ADIWLTPD PTTP	Mus musculu s
1	1.00	1	1	0	0	0	0	-0.003	t	64587	TKINADNP VPILELE	Mus musculu s
1	1.00	1	1	0	0	0	0	-0.003	t	64587	TKINADNP VPILELE	Mus musculu s
1	1.00	1	1	0	0	0	0	-0.003	t	64587	TKINADNP VPILELE	Mus musculu s
1	1.00	1	1	0	0	0	0	-0.003	t	43411	NDDASVD FVAEPVK	Mus musculu s
1	1.00	1	1	0	0	0	0	-0.003	t	56469	RWAPTGA PADVTDQ L	Mus musculu s
1	1.00	1	1	0	0	0	0	-0.003	t	43411	NDDASVD FVAEPVK	Mus musculu s
1	1.00	1	1	0	0	0	0	-0.003	t	7491	DAEFRHDS GYEVHHQ K	Mus musculu s
1	1.00	1	1	0	0	0	0	-0.003	t	7491	DAEFRHDS GYEVHHQ K	Mus musculu s
1	1.00	1	1	-0.01	-0.008	-0.01	-0.01	-0.012	t	141749	SNLDHI	Rattus norvegic us
1	1.00	1	1	0.03	0.03	0.03	0.03	0.03	t	108897	EIDGPAGQ AEP	Mus musculu s

1	1.00	1	1	0.03	0.03	0.03	0.03	0.03	t	108897	EIDGPAGQAEP	Mus musculus
1	1.00	1	1	0.02	0.02	0.02	0.02	0.02	t	144800	TNMLGYTDHQ	Homo sapiens
1	1.00	1	1	0	0	-0.007	0.007	0	t	139601	SCSRALAREVDLKDY	Homo sapiens
1	1.00	1	1	-0.03	-0.028	-0.03	-0.023	-0.03	t	131075	SQSYPEPARGSVPMP	Homo sapiens
1	1.00	1	1	0.05	0.048	0.05	0.043	0.046	t	117520	GSFSDADLAD	Mus musculus
1	1.00	1	1	0	-0.002	0	0.003	-0.001	t	63843	TGGVYHFVKKHVHES	Rattus norvegicus
1	1.00	1	1	0	-0.004	0	0.003	-0.001	t	71353	VTHESYQELVKKLEALEDA	Oryctolagus cuniculus
1	1.00	1	1	0	-0.002	0	0.003	-0.001	t	62779	TAADTPTATESISPSPP	Mus musculus
1	1.00	1	1	0	-0.008	0	0.003	-0.001	t	20064	GGVATY	Macaca
1	1.00	1	1	-0.01	-0.013	-0.003	-0.006	-0.012	t	130622	TGLVAQGTDSIG	Camelus dromedarius
1	1.00	1	1	-0.03	-0.033	-0.022	-0.03	-0.037	t	45317	NPDKARELLNK	Oryctolagus cuniculus
1	1.00	1	1	0	0	0	0	-0.003	t	130590	RCKTFGKTTLCS	Mus musculus
1	1.00	1	1	0	-0.003	0.002	0	-0.002	t	55719	RRRLRGTWV	Oryctolagus cuniculus
1	1.00	1	1	0.03	0.028	0.025	0.027	0.03	t	68725	VGLSNPTP	Anas platyrhynchos
1	1.00	1	1	-0.01	-0.013	-0.015	-0.01	-0.01	t	59985	SPAQDIWGTSAAAYFVG	Oryctolagus cuniculus

1	1.00	1	1	0	-0.003	-0.011	0	0	t	58511	SIFYTYGTAPARISVPYVGI	Oryctolagus cuniculus
1	1.00	1	1	-0.01	-0.009	-0.011	-0.009	-0.01	t	73770	YETGWGCNPPDC	Myodes glareolus
1	1.00	1	1	-0.03	-0.025	-0.034	-0.027	-0.03	t	77793	PKSKLSVKKC	Homo sapiens
1	1.00	1	1	0.08	0.078	0.075	0.079	0.082	t	25080	HVQTMEEI	Anas platyrhynchos
1	1.00	1	1	0.02	0.02	0.021	0.027	0.02	t	52184	QRAKEALDKYELENH	Homo sapiens
1	1.00	1	1	-0.02	-0.017	-0.018	-0.02	-0.021	t	119861	FHRWSSYMVH	Mus musculus
1	1.00	1	1	-0.02	-0.02	-0.018	-0.02	-0.021	t	119861	FHRWSSYMVH	Mus musculus
1	1.00	1	1	-0.02	-0.02	-0.018	-0.02	-0.021	t	119861	FHRWSSYMVH	Oryctolagus cuniculus
1	1.00	1	1	-0.02	-0.02	-0.018	-0.02	-0.021	t	119861	FHRWSSYMVH	Mus musculus
1	1.00	1	1	-0.02	-0.02	-0.018	-0.02	-0.021	t	119861	FHRWSSYMVH	Mus musculus
1	1.00	1	1	-0.02	-0.02	-0.018	-0.02	-0.021	t	119861	FHRWSSYMVH	Mus musculus
1	1.00	1	1	-0.01	-0.01	-0.013	-0.006	-0.01	t	25315	IAIGGAATGIAAVLTDGNPP	Homo sapiens
1	1.00	1	1	0	0	-0.003	-0.001	0	t	22217	GRTPFII	Homo sapiens

1	1.00	1	1	0.01	0.01	0.01	0.01	0.008	t	117666	VAGRGRGRGRGRG	Homo sapiens
1	1.00	1	1	0.02	0.018	0.012	0.02	0.02	t	7846	DDPLLGNQ	Anas platyrhynchos
1	1.00	1	1	0.02	0.024	0.021	0.021	0.017	t	53429	RDTLPQSPGPAFP	Homo sapiens
1	1.00	1	1	-0.04	-0.044	-0.04	-0.043	-0.042	t	5109	ATPYHPPATP	Oryctolagus cuniculus
1	1.00	1	1	0.04	0.043	0.034	0.04	0.04	t	84184	KASAEG	Mus musculus
1	1.00	1	1	0.04	0.043	0.034	0.04	0.04	t	84184	KASAEG	Mus musculus
1	1.00	1	1	0	0	0	0.004	-0.001	t	122445	KQYSIEAALRCRRISKEEYH	Homo sapiens
1	1.00	1	1	0	-0.002	-0.001	0	-0.001	t	95092	GYQKTVDHTKVNSK	Oryctolagus cuniculus
1	1.00	1	1	0	0	-0.001	0	0	t	39885	LTETKIDKLC	Mus musculus
1	1.00	1	1	0.04	0.026	0.035	0.04	0.041	t	134021	CQSIAAPSKTS	Oryctolagus cuniculus
1	1.00	1	1	-0.03	-0.033	-0.039	-0.03	-0.032	t	15973	FGEWRERVLLNR	Oryctolagus cuniculus
1	1.00	1	1	0.04	0.038	0.037	0.043	0.041	t	105072	AEPRQEFEV	Mus musculus
1	1.00	1	1	0.01	0.01	0.01	0.01	0.01	t	79284	LDAVPL	Mus musculus
1	1.00	1	1	0	0.002	-0.011	-0.003	-0.002	t	33148	KRHDGGCRKE	Homo sapiens

1	1.00	1	1	0.01	0.01	0.01	0.01	0.011	t	9941	DRADGQPAG	Mus musculus
1	1.00	1	1	0	-0.003	0	0	0	t	156862	DVGDPLR	Oryctolagus cuniculus
1	1.00	1	1	0	0	0	0	-0.002	t	10800	DWEDRYY	Mus musculus
1	1.00	1	1	0.03	0.03	0.036	0.03	0.03	t	18039	FTPGPSQ	Homo sapiens
1	1.00	1	1	0	0	0	0	0	t	140834	DAVKGYLAGKGV	Oryctolagus cuniculus
1	1.00	1	1	0.01	0.01	0.005	0.01	0.011	t	22303	GSDYEDRYYRENM	Mus musculus
1	1.00	1	1	-0.01	-0.01	-0.022	-0.01	-0.01	t	78635	EKIERNNKLKQPPPPPNPN	Mus musculus
1	1.00	1	1	-0.01	0.004	-0.016	-0.01	-0.011	t	47662	PGGGQIVGGVYLLPRR	Pan troglodytes
1	1.00	1	1	-0.01	0.002	-0.016	-0.01	-0.011	t	47662	PGGGQIVGGVYLLPRR	Pan troglodytes
1	1.00	1	1	0	0	0	0	-0.003	t	140725	PDELHK	Mus musculus
1	1.00	1	1	0	0	0	0	-0.003	t	137743	DTPLTIAGLKLV	Mus musculus
1	1.00	1	1	0	0	0	0	-0.003	t	137770	TLARSFIICIQM	Mus musculus
1	1.00	1	1	0	0	0	0	-0.003	t	138862	KFPED	Mus musculus
1	1.00	1	1	0	0	0	0	-0.003	t	150858	ISSLYNLVKSS	Mus musculus
1	1.00	1	1	0	0	0	0	-0.003	t	135569	KTNMK	Mus musculus

1	1.00	1	1	0	0	0	0	-0.003	t	140674	ESQPDPKPDELHKS	Mus musculus
1	1.00	1	1	0	0	0	0	-0.003	t	151813	CGADSYEMEEDGVRKC	Mus musculus
1	1.00	1	1	0	0	0	0	-0.003	t	117495	ERDRKREKRKPKS	Mus musculus
1	1.00	1	1	0	0	0	0	-0.003	t	131217	EDISAYLL	Mus musculus
1	1.00	1	1	0	0	0	0	-0.003	t	118060	PPPGMRPP	Homo sapiens
1	1.00	1	1	0	0	0	0	-0.003	t	117614	QGFPFYDKPMRI	Mus musculus
1	1.00	1	1	0	0	0	0	-0.003	t	108382	GGVVIA	Mus musculus
1	1.00	1	1	0	0	0	0	-0.003	t	105163	EFRHD	Mus musculus
1	1.00	1	1	0	0	0	0	-0.003	t	102423	FEYQD	Mus musculus
1	1.00	1	1	0	0	0	0	-0.003	t	102931	WQALCAYQAE	Mus musculus
1	1.00	1	1	0	0	0	0	-0.003	t	67967	VDGSDP	Mus musculus
1	1.00	1	1	0	0	0	0	-0.003	t	75441	YPYDVPDYA	Mus musculus
1	1.00	1	1	0	0	0	0	-0.003	t	60917	SSAVVDNKLKI	Mus musculus
1	1.00	1	1	0	0	0	0	-0.003	t	63839	TGGSTDGKQG	Mus musculus
1	1.00	1	1	0	0	0	0	-0.003	t	43242	NANPNANPNANP	Mus musculus
1	1.00	1	1	0	0	0	0	-0.003	t	45932	NSNNPDWDF	Mus musculus

1	1.00	1	1	0	0	0	0	-0.003	t	40332	LVFFAEDV	Mus musculus
1	1.00	1	1	0	0	0	0	-0.003	t	40332	LVFFAEDV	Mus musculus
1	1.00	1	1	0	0	0	0	-0.003	t	50637	QELDCWHYQHYCVTSTKS	MD
1	1.00	1	1	0	0	0	0	-0.003	t	46054	NTANPDW	Mus musculus
1	1.00	1	1	0	0	0	0	-0.003	t	38304	LNPKTNKWEDK	Homo sapiens
1	1.00	1	1	0	0	0	0	-0.003	t	51853	QPHGGGW	Mus musculus
1	1.00	1	1	0	0	0	0	-0.003	t	30093	KCTSDQDEQFIPKGCSK	Oryctolagus cuniculus
1	1.00	1	1	0	0	0	0	-0.003	t	11571	EEEDDDMGFGLFD	Mus musculus
1	1.00	1	1	0	0	0	0	-0.003	t	17262	FPCDRLSGYWERGIPSPCVR	Homo sapiens
1	1.00	1	1	0	0	0	0	-0.003	t	11571	EEEDDDMGFGLFD	Mus musculus
1	1.00	1	1	0	0	0	0	-0.003	t	11571	EEEDDDMGFGLFD	Homo sapiens
1	1.00	1	1	0	0	0	0	-0.003	t	32808	KPNLSSKRSELSQLSMYRVF	Mus musculus
1	1.00	1	1	0	0	0	0	-0.003	t	22586	GSYNRSSFESSSGLV	Mus musculus
1	1.00	1	1	0	0	0	0	-0.003	t	28299	IRGDTFATR	Mus musculus
1	1.00	1	1	0	0	0	0	-0.003	t	7492	DAEFRHDSGYEVHHQKL	Mus musculus

1	1.00	1	1	0	0	0	0	-0.003	t	4401	ASFFD	Mus musculus
1	1.00	1	1	0	0	0	0	-0.003	t	7491	DAEFRHDSGYEVHHQK	Mus musculus
1	1.00	1	1	0	0	0	0	-0.003	t	4907	ATCDKLFGYYERGIPLPCAL	Homo sapiens
1	1.00	1	1	0	0	0	0	-0.003	t	3952	AQEPPRQ	Mus musculus
1	1.00	1	1	0	0	0	0	-0.003	t	7491	DAEFRHDSGYEVHHQK	Mus musculus
1	1.00	1	1	0.05	0.05	0.051	0.05	0.05	t	63542	TESTVSSALAELAT	Homo sapiens
1	1.00	1	1	0.04	0.04	0.039	0.04	0.04	t	48849	PPPAPDPSPT	Homo sapiens
1	1.00	1	1	0	0	0	0	0	t	153841	KCLKFYSKISEYRHY	Cavia porcellus
1	1.00	1	1	-0.02	-0.016	-0.026	-0.019	-0.021	t	12247	EGQDRGYSY	Homo sapiens
1	1.00	1	1	0.03	0.028	0.026	0.033	0.03	t	123851	WTEVSEVATEV	Homo sapiens
1	1.00	1	1	0.02	0.02	0.02	0.02	0.02	t	138080	VSLGQNVL	Homo sapiens
1	1.00	1	1	-0.03	-0.032	-0.037	-0.027	-0.031	t	53876	RGGIPSLLKKGKDIKV	Mus musculus
1	1.00	1	1	0	0	0	0	-0.002	t	45405	NPNANP	Mus musculus

1	1.00	1	1	0.01	0.013	0.005	0.01	0.01	t	37656	LLPHSNLD	Mus musculus
1	1.00	1	1	-0.04	-0.038	-0.045	-0.037	-0.042	t	134076	GWLGFNKERGHLQ	Homo sapiens
1	1.00	1	1	-0.02	-0.02	-0.02	-0.02	-0.018	t	135457	FGF	Mus musculus
1	1.00	1	1	0.01	0.01	0.003	0.01	0.01	t	55162	RPGLPVEYLQVPSEA	Homo sapiens
1	1.00	1	1	0	0.002	0	0.009	0.001	t	122445	KQYSIEAALRCRRISKEEYH	Homo sapiens
1	1.00	1	1	0	0	0	0.009	0.001	t	122445	KQYSIEAALRCRRISKEEYH	Homo sapiens
1	1.00	1	1	0	-0.002	0	0	-0.002	t	137401	FEDVW	Mus musculus
1	1.00	1	1	0.03	0.032	0.03	0.031	0.032	t	10636	DVKFPGGGQI	Homo sapiens
1	1.00	1	1	0	0.002	0.005	0.007	-0.003	t	131897	GQMPNNQSINSKVSG	Homo sapiens
1	1.00	1	1	0.01	0.006	0.005	0.009	0.01	t	97404	ISPNDNTFV	Oryctolagus cuniculus
1	1.00	1	1	-0.04	-0.036	-0.047	-0.039	-0.041	t	138028	SAGALQLLVVGC	Homo sapiens
1	1.00	1	1	0.05	0.053	0.039	0.05	0.053	t	111309	GDTPTLHEYMLDLQ	Mus musculus
1	1.00	1	1	-0.04	-0.038	-0.049	-0.037	-0.04	t	63098	TCRRMLAFLKDKENR	Homo sapiens
1	1.00	1	1	0	0.002	-0.006	0.003	0.001	t	118824	GRRPFFHPVGEADYFEYHQE	Homo sapiens
1	1.00	1	1	-0.02	-0.017	-0.029	-0.027	-0.022	t	134509	RHFFNRSGTV	Mus musculus

1	1.00	1	1	0.05	0.051	0.043	0.05	0.05	t	45571	NQITSALSTQ	Mus musculus
1	1.00	1	1	-0.01	-0.007	-0.019	-0.01	-0.01	t	100502	SEEGRGLLKK	Mus musculus
1	1.00	1	1	0.02	0.018	0.022	0.019	0.021	t	62641	SYLPQLG	Mus musculus
1	1.00	1	1	0.08	0.082	0.075	0.081	0.08	t	20174	GHQVGDGE	Homo sapiens
1	1.00	1	1	-0.01	-0.008	-0.018	-0.009	-0.01	t	30304	KEAPTTKPTEKP	Homo sapiens
1	1.00	1	1	0.01	0.016	0	0.01	0.01	t	139100	ITGRLAALNAYVSQTLTQY	Felis catus
1	1.00	1	1	-0.02	-0.02	-0.021	-0.02	-0.02	t	7057	CSPRTG	Homo sapiens
1	1.00	1	1	0.01	0.01	0.006	0.013	0.009	t	67069	TVLGCALPPTPQTP	Homo sapiens
1	1.00	1	1	0.07	0.07	0.077	0.079	0.072	t	131048	RQSDGQYQM	Homo sapiens
1	1.00	1	1	0.02	0.018	0.015	0.023	0.019	t	23694	HEIAWFTERSDKSYEHQT	Mus musculus
1	1.00	1	1	0.02	0.018	0.015	0.023	0.019	t	23694	HEIAWFTERSDKSYEHQT	Mus musculus
1	1.00	1	1	0.02	0.016	0.016	0.023	0.022	t	106073	RGVTHDQL	Oryctolagus cuniculus
1	1.00	1	1	-0.06	-0.06	-0.063	-0.06	-0.063	t	110390	QWGRL	Mus musculus

1	1.00	1	1	0.02	0.02	0.02	0.02	0.021	t	73384	YALADASLKMAD	Oryctolagus cuniculus
1	1.00	1	1	-0.02	-0.02	-0.02	-0.02	-0.02	t	156909	LAHPFTP	Gallus gallus
1	1.00	1	1	0	-0.004	-0.005	0.003	0	t	76396	YVRGGGAVP	Oryctolagus cuniculus
1	1.00	1	1	0	-0.004	-0.005	0.003	0	t	70153	VNPNNAAAL	Oryctolagus cuniculus
1	1.00	1	1	0.01	0.008	0.002	0.009	0.01	t	2220	AKKQVEKALEEANSKLAALE	Mus musculus
1	1.00	1	1	0.06	0.062	0.055	0.061	0.06	t	19723	GGESSEELSESSFFNLIT	Homo sapiens
1	1.00	1	1	0.02	0.018	-0.001	0.017	0.022	t	153829	ERSVQKLQKEVDRLEDE	Homo sapiens
1	1.00	1	1	-0.01	-0.012	-0.012	-0.007	-0.01	t	51529	QLRRKLEFFTYVRFD	Mus musculus
1	1.00	1	1	0.02	0.016	0.018	0.023	0.02	t	95480	ITELSEFIPGAS	Oryctolagus cuniculus
1	1.00	1	1	0.01	0.006	-0.002	0.009	0.01	t	24878	HSWYNDLLVDFDSKDIVDKY	Oryctolagus cuniculus
1	1.00	1	1	0	0	0	0	-0.002	t	119633	QYTGHITWTPPAIFKS	Mus musculus
1	1.00	1	1	0	-0.002	0	0.003	-0.001	t	157702	NKFAVETL	Chlorocebus aethiops
1	1.00	1	1	0.01	0.012	0.012	0.011	0.009	t	103185	FRMELLSLPQDEWAG	Homo sapiens
1	1.00	1	1	0.03	0.028	0.03	0.029	0.03	t	22494	GSRYPGQGSPG	Mus musculus

1	1.00	1	1	0	-0.005	-0.008	0.003	0.002	t	157772	QGMMSPGRSEEK	Canis lupus
1	1.00	1	1	0.03	0.034	0.03	0.037	0.03	t	117364	RPDAEYWNSQKDLLEQRRAA	Homo sapiens
1	1.00	1	1	0.01	0.01	0.007	0.014	0.01	t	23171	GVTLLQAAPT	Homo sapiens
1	1.00	1	1	0.01	0.006	0.006	0.013	0.009	t	105684	ASFDKVKGDPVGILYAVFKA	Oryctolagus cuniculus
1	1.00	1	1	-0.03	-0.028	-0.039	-0.029	-0.03	t	110427	STWPTTPPRPIPKPSPWAPK	Homo sapiens
1	1.00	1	1	-0.01	-0.008	-0.014	-0.007	-0.008	t	60752	SRPGLKPDLTGTSKS	Homo sapiens
1	1.00	1	1	0.02	0.023	0.013	0.02	0.021	t	150858	ISSLYNLVKSS	Mus musculus
1	1.00	1	1	0.01	0.013	0.01	0.01	0.008	t	26046	IFDT	Mus musculus
1	1.00	1	1	0.04	0.038	0.04	0.037	0.04	t	51147	QIVGGVYLL	Mus musculus
1	1.00	1	1	0	0.01	0	-0.001	-0.001	t	55070	RNTNRRPQDV	Pan troglodytes
1	1.00	1	1	0.06	0.062	0.059	0.057	0.058	t	55861	RSILDLDLTK	Homo sapiens
1	1.00	1	1	0.01	0.01	0.005	0.01	0.01	t	153440	PRLKNPPKKPKDDY	Mus musculus
1	1.00	1	1	-0.04	-0.038	-0.037	-0.033	-0.04	t	119609	LHQDWLNG	Homo sapiens
1	1.00	1	1	0	0	-0.006	0.003	0.002	t	12258	EGRFKSQD	Homo sapiens

1	1.00	1	1	0.03	0.03	0.03	0.034	0.03	t	34935	LAQMYQANDNSLEDV	Homo sapiens
1	1.00	1	1	-0.01	-0.01	-0.01	-0.01	-0.01	t	54430	RKTKRNT	Homo sapiens
1	1.00	1	1	0.07	0.07	0.07	0.077	0.072	t	122746	EEQEDEIIGFGQELKN	Homo sapiens
1	1.00	1	1	0.04	0.041	0.039	0.043	0.04	t	99450	LRPEDTLEEKMYACR	Equus caballus
1	1.00	1	1	-0.03	-0.033	-0.037	-0.03	-0.031	t	41610	MGQHPAKSMDVRR	Oryctolagus cuniculus
1	1.00	1	1	0.01	0.01	0.008	0.01	0.008	t	38702	LPSDFFPSVRDL	Mus musculus
1	1.00	1	1	-0.02	-0.02	-0.02	-0.02	-0.019	t	115349	LKGYGGVSLP	Homo sapiens
1	1.00	1	1	0	0	0	0.004	-0.001	t	43242	NANPNANPNANP	Homo sapiens
1	1.00	1	1	0	0	0	0.004	-0.001	t	11819	EENVEHDA	Homo sapiens
1	1.00	1	1	0	0	0	0.004	-0.001	t	11819	EENVEHDA	Homo sapiens
1	1.00	1	1	-0.01	-0.01	-0.022	-0.01	-0.01	t	116971	FAVLKLMGRGTKF	Rattus norvegicus
1	1.00	1	1	0.03	0.026	0.024	0.023	0.029	t	105884	IMAKFTQF	Oryctolagus cuniculus
1	1.00	1	1	0.01	0.01	0.005	0.007	0.008	t	98734	DLEILLQKWE	Homo sapiens
1	1.00	1	1	0.01	0.012	0.012	0.017	0.011	t	122730	DPAQPPRD	Homo sapiens

1	1.00	1	1	0	0.004	-0.003	0.007	0	t	121051	VSEGTKAV TKYTSSK	Homo sapiens
1	1.00	1	1	0	0.002	-0.003	0.007	0	t	121051	VSEGTKAV TKYTSSK	Homo sapiens
1	1.00	1	1	0.01	0.01	0.006	0.01	0.01	t	46858	PALTAAET G	Homo sapiens
1	1.00	1	1	0.03	0.029	0.025	0.03	0.03	t	19301	GEILLNQL	Anas platyrhy nchos
1	1.00	1	1	-0.03	-0.034	-0.043	-0.033	-0.03	t	55640	RRLGAALL QS	Oryctola gus cuniculu s
1	1.00	1	1	-0.03	-0.028	-0.03	-0.023	-0.029	t	113321	DENPVVH FFKNIVTP RT	Homo sapiens
1	1.00	1	1	-0.03	-0.028	-0.03	-0.023	-0.029	t	113321	DENPVVH FFKNIVTP RT	Homo sapiens
1	1.00	1	1	-0.04	-0.04	-0.054	-0.04	-0.04	t	39196	LRRLRTAC ERAKRTL	Homo sapiens
1	1.00	1	1	-0.01	-0.008	-0.019	-0.01	-0.01	t	51474	QLPNVGIT QGIV	Ovis aries
1	1.00	1	1	-0.05	-0.05	-0.05	-0.043	-0.05	t	119823	EDDDMGF GLFD	Homo sapiens
1	1.00	1	1	0.01	0.01	0	0.009	0.012	t	24449	HPFVALGL KQPKEIK	Mus musculu s
1	1.00	1	1	0.03	0.028	0.026	0.033	0.03	t	58212	SGQTTPQ VETEDTKE	Mus musculu s
1	1.00	1	1	-0.04	-0.037	-0.05	-0.04	-0.04	t	47157	PDPLKPTK R	Mus musculu s
1	1.00	1	1	0.03	0.03	0.028	0.027	0.028	t	14638	EVDDEALE KF	Homo sapiens
1	1.00	1	1	-0.01	-0.008	-0.01	-0.007	-0.011	t	918	AEGEFATA PPSHYSW DPAK	Homo sapiens

1	1.00	1	1	0.01	0.003	0.004	0.01	0.01	t	86742	NGVWVMMDGNEQVEYPLK	Oryctolagus cuniculus
1	1.00	1	1	-0.04	-0.038	-0.047	-0.037	-0.038	t	77826	AFLRHAAL	Homo sapiens
1	1.00	1	1	0	0	0.002	0	-0.001	t	112134	GLVGPRGERGF	Mus musculus
1	1.00	1	1	0	-0.014	-0.011	-0.001	0	t	51259	QKTLTKTS	Macaca mulatta
1	1.00	1	1	0.01	0.01	0.004	0.007	0.01	t	44172	NHVADIDKLIDYAA	Homo sapiens
1	1.00	1	1	0.03	0.03	0.022	0.033	0.032	t	34942	LARGSPPSLASSSASQLSA	Homo sapiens
1	1.00	1	1	0	0.004	-0.003	-0.003	-0.002	t	115300	ISCDKFLDDD	Homo sapiens
1	1.00	1	1	-0.01	-0.01	-0.012	-0.01	-0.013	t	35060	LCKDKKADSGYCYWV	Mus musculus
1	1.00	1	1	-0.01	-0.01	-0.012	-0.01	-0.013	t	35060	LCKDKKADSGYCYWV	Mus musculus
1	1.00	1	1	0.01	0.014	0.01	0.017	0.009	t	122874	PPARALKRSLWVEVDLLIGS	Homo sapiens
1	1.00	1	1	0.05	0.046	0.049	0.05	0.049	t	123849	WQDE	Mus musculus
1	1.00	1	1	-0.01	-0.012	-0.019	-0.011	-0.008	t	63967	TGSQDFFVRSIG	Mus musculus
1	1.00	1	1	0.01	0.012	0.004	0.011	0.009	t	36950	LKLSIPVAERAKPRK	Homo sapiens
1	1.00	1	1	0.04	0.04	0.04	0.044	0.04	t	115631	NVRFSDEGGFTC	Homo sapiens
1	1.00	1	1	0.01	0.008	0.006	0.009	0.012	t	60917	SSAVVDNKLKI	Mus musculus

1	1.00	1	1	0.02	0.022	0.015	0.017	0.018	t	98696	AMAASDISLL	Homo sapiens
1	1.00	1	1	0	0.002	-0.012	0.001	0.001	t	63217	TDQMEVDSGPRKRPLRGG	Homo sapiens
1	1.00	1	1	-0.03	-0.03	-0.03	-0.03	-0.032	t	94741	WEQWW	Mus musculus
1	1.00	1	1	0.01	0.014	-0.001	0.01	0.01	t	110562	DIILECVYCKQQLLRREVYD	Cavia porcellus
1	1.00	1	1	-0.01	-0.01	-0.024	-0.01	-0.008	t	8267	DFFVRSIGSKGGKL	Mus musculus
1	1.00	1	1	-0.02	-0.02	-0.027	-0.02	-0.02	t	44911	NLVDWLRQY	Homo sapiens
1	1.00	1	1	-0.03	-0.032	-0.03	-0.026	-0.03	t	117885	GMRPPMGPPI	Mus musculus
1	1.00	1	1	0.02	0.02	0.025	0.022	0.018	t	96081	AKIDDPTDSK	Homo sapiens
1	1.00	1	1	-0.03	-0.028	-0.032	-0.027	-0.03	t	51361	QLGPRR	Homo sapiens
1	1.00	1	1	-0.03	-0.028	-0.032	-0.027	-0.03	t	51361	QLGPRR	Homo sapiens
1	1.00	1	1	0	0	0	0	0	t	42493	MRQLDTNVER	Homo sapiens
1	1.00	1	1	-0.03	-0.029	-0.03	-0.027	-0.03	t	78281	TKLPAA	Equus caballus
1	1.00	1	1	-0.01	-0.01	-0.02	-0.01	-0.009	t	104161	NVVLKTITESATSPPT	Oryctolagus cuniculus
1	1.00	1	1	-0.01	-0.01	-0.014	-0.01	-0.012	t	73021	WRCVVYPGNGFVSASIFGFQ	Oryctolagus cuniculus
1	1.00	1	1	0	-0.003	0	0	-0.003	t	105127	DAEFRHD	Oryctolagus cuniculus
1	1.00	1	1	0.03	0.026	0.032	0.029	0.03	t	78637	EKSLTSLSE	Oryctolagus cuniculus

1	1.00	1	1	0.07	0.068	0.065	0.067	0.07	t	12498	EIKYNGEEYL	Mus musculus
1	1.00	1	1	0.02	0.022	0.014	0.023	0.022	t	133795	DFTYDQVAFNGLPQF	Homo sapiens
1	1.00	1	1	0.04	0.037	0.032	0.04	0.04	t	34217	KVTLADAGFMKQYGECL	Oryctolagus cuniculus
1	1.00	1	1	0.04	0.04	0.032	0.04	0.04	t	34217	KVTLADAGFMKQYGECL	Mus musculus
1	1.00	1	1	-0.01	-0.007	-0.014	-0.01	-0.01	t	50177	PYTSTLLP	Mus musculus
1	1.00	1	1	0.02	0.024	0.02	0.027	0.021	t	131700	TGVTTQGVKSLLTSMYVKEF	Homo sapiens
1	1.00	1	1	-0.02	-0.018	-0.03	-0.017	-0.018	t	47643	PGFGDRRKAM	Homo sapiens
1	1.00	1	1	0.02	0.022	0.023	0.021	0.02	t	51454	QLNPKDNDVEL	Homo sapiens
1	1.00	1	1	0.03	0.028	0.038	0.029	0.03	t	30986	KGIYPLSYE	Mus musculus
1	1.00	1	1	0	0.002	-0.008	0.001	-0.001	t	62872	TAGWTFGAGAALQIPFA	Homo sapiens
1	1.00	1	1	-0.01	-0.014	-0.01	-0.007	-0.012	t	156911	LGMSTNPGPRIL	Bos taurus
1	1.00	1	1	0.01	0.01	0.01	0.01	0.01	t	96126	AVNVPKVTKE	Homo sapiens(human)
1	1.00	1	1	-0.04	-0.038	-0.041	-0.039	-0.041	t	120410	ARLVVLASGTGSLLR	Homo sapiens
1	1.00	1	1	-0.01	-0.01	-0.013	-0.01	-0.012	t	60992	SSFFSYGEIP	Oryctolagus cuniculus

1	1.00	1	1	0.01	0.012	0.003	0.017	0.012	t	142386	ANKYQISVNKYRGTAGNAL	Homo sapiens
1	1.00	1	1	0.03	0.028	0.022	0.029	0.03	t	57196	SDFTDSVRDPKTS	Mus musculus
1	1.00	1	1	0.02	0.016	0.007	0.02	0.02	t	149731	GVSDYHKNLINN	Mus musculus
1	1.00	1	1	0.06	0.06	0.052	0.063	0.062	t	26235	IGGSQAQTAS	Homo sapiens
1	1.00	1	1	0.02	0.016	0.013	0.017	0.02	t	135448	AKYGAA	Oryctolagus cuniculus
1	1.00	1	1	0.06	0.06	0.06	0.06	0.06	t	79652	SSFSPST	Oryctolagus cuniculus
1	1.00	1	1	0.03	0.027	0.028	0.03	0.03	t	127185	TCDDPRFQDSS	Oryctolagus cuniculus
1	1.00	1	1	0.03	0.03	0.031	0.033	0.028	t	59983	SPAPPPPEHRGGPEEFEGAG	Homo sapiens
1	1.00	1	1	-0.01	-0.01	-0.01	-0.01	-0.014	t	133630	LPPGLHVFPLASNRS	Mus musculus
1	1.00	1	1	-0.01	-0.001	-0.022	-0.01	-0.01	t	115593	AQGHRPQDEN	Oryctolagus cuniculus
1	1.00	1	1	-0.01	0.002	-0.022	-0.01	-0.01	t	115593	AQGHRPQDEN	Rattus norvegicus
1	1.00	1	1	-0.01	-0.019	-0.022	-0.01	-0.01	t	115593	AQGHRPQDEN	Macaca mulatta
1	1.00	1	1	-0.01	-0.01	-0.022	-0.01	-0.01	t	115593	AQGHRPQDEN	Macaca mulatta
1	1.00	1	1	-0.01	-0.01	-0.022	-0.01	-0.01	t	115593	AQGHRPQDEN	Oryctolagus cuniculus

1	1.00	1	1	0.04	0.04	0.033	0.04	0.041	t	130567	NPCEDI	Mus musculus
1	1.00	1	1	-0.01	-0.01	-0.02	-0.013	-0.012	t	32975	KQEVQRGGRYNQ	Homo sapiens
1	1.00	1	1	0.03	0.027	0.019	0.03	0.032	t	36042	LGAALLQS	Oryctolagus cuniculus
1	1.00	1	1	0.03	0.03	0.019	0.03	0.032	t	36042	LGAALLQS	Oryctolagus cuniculus
1	1.00	1	1	0.01	0.01	-0.001	0.01	0.01	t	95100	IMPATSDSVPLIAQYFASTM	Rattus norvegicus
1	1.00	1	1	0.03	0.026	0.013	0.03	0.032	t	35098	LCSGGYTVSKSK	Mus musculus
1	1.00	1	1	0.03	0.023	0.013	0.03	0.032	t	35098	LCSGGYTVSKSK	Oryctolagus cuniculus
1	1.00	1	1	0	0.002	0	0.007	0.001	t	108629	SHQESTRGRSRGRSGRSGS	Homo sapiens
1	1.00	1	1	0	0.002	0	0.007	0.001	t	108629	SHQESTRGRSRGRSGRSGS	Homo sapiens
1	1.00	1	1	0	0.002	0	0.001	0	t	20850	GLFGKG	Homo sapiens
1	1.00	1	1	0.02	0.016	0.013	0.019	0.019	t	137828	DVLLAGNAEADR	Oryctolagus cuniculus
1	1.00	1	1	0.01	0.007	-0.004	0.01	0.011	t	21807	GPTEESVERAMGRVA	Oryctolagus cuniculus
1	1.00	1	1	-0.02	-0.02	-0.02	-0.02	-0.02	t	133156	IRLR	Oryctolagus cuniculus
1	1.00	1	1	0	0	-0.019	0	0.002	t	45162	NNLGLLCEYQAIGSA	Homo sapiens

1	1.00	1	1	-0.01	-0.01	-0.008	-0.01	-0.011	t	119827	EFHRWSSYMV	Mus musculus
1	1.00	1	1	-0.01	-0.01	-0.008	-0.01	-0.011	t	119827	EFHRWSSYMV	Oryctolagus cuniculus
1	1.00	1	1	-0.01	-0.01	-0.008	-0.01	-0.011	t	119827	EFHRWSSYMV	Mus musculus
1	1.00	1	1	0.03	0.032	0.03	0.031	0.03	t	111693	QVILCPTSVFSSNEV	Homo sapiens
1	1.00	1	1	0.01	0.012	0.003	0.017	0.011	t	139493	EQSADSSRHSGSGH	Homo sapiens
1	1.00	1	1	0	0	0	0	0	t	129025	KAFDKHMN	Homo sapiens
1	1.00	1	1	-0.04	-0.036	-0.036	-0.033	-0.04	t	144614	DMMYGGMRGM	Homo sapiens
1	1.00	1	1	0	0.01	0	-0.001	0	t	48496	PLTDFDQGWG	Pan troglodytes
1	1.00	1	1	0.03	0.03	0.03	0.03	0.029	t	7673	DAVTE	Mus musculus
1	1.00	1	1	0.03	0.03	0.03	0.03	0.031	t	115308	KFLDDDLTDD	Homo sapiens
1	1.00	1	1	0	0.004	0	0.007	0	t	123048	ATKKAVQG	Homo sapiens
1	1.00	1	1	0.05	0.05	0.05	0.05	0.05	t	139770	GWYSFQH	Ovis aries
1	1.00	1	1	0.02	0.022	0.016	0.017	0.02	t	112837	SGYIPANTTIPF	Homo sapiens
1	1.00	1	1	-0.06	-0.059	-0.067	-0.06	-0.061	t	11370	EDKLADYKRRMADA	Myodes glareolus
1	1.00	1	1	0.01	0.01	0.005	0.016	0.016	t	125209	QFQIHGPR	Mus musculus

1	1.00	1	1	-0.01	-0.008	-0.012	-0.007	-0.01	t	44175	NHVSPTHYVPESDAAAR	Homo sapiens
1	1.00	1	1	0.01	0.01	0.009	0.01	0.008	t	72503	WGQGG	Mus musculus
1	1.00	1	1	0.01	0.006	0.008	0.013	0.01	t	41544	MFTKYNGNLNTI	Oryctolagus cuniculus
1	1.00	1	1	0	0.002	-0.005	0.001	-0.001	t	59944	SNVPFSPDGKPCTPPAL	Homo sapiens
1	1.00	1	1	-0.02	-0.022	-0.027	-0.017	-0.019	t	107124	GAPGLRGGAG	Mus musculus
1	1.00	1	1	0.03	0.027	0.019	0.03	0.031	t	144776	SEISTGLVGITSVAS	Oryctolagus cuniculus
1	1.00	1	1	0	0	-0.012	0	0	t	153370	DKEGIPPDQQRLIFA	Mus musculus
1	1.00	1	1	-0.02	-0.02	-0.02	-0.02	-0.023	t	95739	QRYQKFALPQ	Homo sapiens
1	1.00	1	1	0.03	0.021	0.025	0.03	0.03	t	29972	KAVEKVTETLLKGAK	Rattus norvegicus
1	1.00	1	1	-0.02	-0.016	-0.015	-0.017	-0.022	t	65476	TNVGLKIRQLLW	Homo sapiens
1	1.00	1	1	-0.02	-0.018	-0.015	-0.017	-0.022	t	65476	TNVGLKIRQLLW	Homo sapiens
1	1.00	1	1	0.01	0.007	0.007	0.01	0.008	t	111820	TEAVPGDPDVNRF	Oryctolagus cuniculus
1	1.00	1	1	0	0	0	0	-0.003	t	48597	PNANPN	Mus musculus
1	1.00	1	1	-0.01	-0.01	-0.01	-0.01	-0.013	t	97348	GLFGAIAGFIEGGW	Oryctolagus cuniculus

1	1.00	1	1	0	0	0	0	-0.001	t	156907	KVGDPLW	Mus musculus
1	1.00	1	1	0.01	0.01	0.016	0.011	0.01	t	29463	IVTCAM	Homo sapiens
1	1.00	1	1	0.01	0.01	0.01	0.012	0.01	t	24881	HSYSPGEINRVASC	Homo sapiens
1	1.00	1	1	-0.02	-0.017	-0.022	-0.019	-0.019	t	48732	PPA	Mus musculus
1	1.00	1	1	0	0	0	0	-0.002	t	10538	DVAGLEKDPVA	Mus musculus
1	1.00	1	1	-0.03	-0.032	-0.042	-0.031	-0.03	t	133647	MMHVERKEC	Mus musculus
1	1.00	1	1	0.02	0.024	0.016	0.017	0.018	t	50863	QGFSRNTLEAAFNAE	Homo sapiens
1	1.00	1	1	0.01	0.01	0.01	0.01	0.01	t	44554	NKND	Mus musculus
1	1.00	1	1	0	0	0	0	-0.001	t	120681	KILPEVKEKH	Oryctolagus cuniculus
1	1.00	1	1	0.02	0.016	0.023	0.023	0.02	t	115265	GKDITADNK	Oryctolagus cuniculus
1	1.00	1	1	0	0	0	0	-0.001	t	101983	PVYQQQP	Mus musculus
1	1.00	1	1	-0.01	-0.013	-0.016	-0.01	-0.01	t	29824	KAIERM	Oryctolagus cuniculus
1	1.00	1	1	-0.02	-0.02	-0.02	-0.02	-0.02	t	112853	TPTFMYF	Homo sapiens
1	1.00	1	1	0	0	0	-0.006	-0.006	t	113966	TTHYGSLPQKAQGHRPQDE	Oryctolagus cuniculus
1	1.00	1	1	0	0	0	-0.006	-0.006	t	113879	SQRSQDEN	Rattus norvegicus

1	1.00	1	1	0	0	0	-0.006	-0.006	t	113966	TTHYGSLPQKAQGHRPQDE	Oryctolagus cuniculus
1	1.00	1	1	0.04	0.04	0.04	0.036	0.04	t	131688	SYSCQVT	Homo sapiens
1	1.00	1	1	0.02	0.02	0.021	0.023	0.02	t	35139	LDEGKQSLTKLAAAWG	Homo sapiens
1	1.00	1	1	0.06	0.05	0.06	0.067	0.06	t	134265	VQLEDPEFQASNIMHSINGY	Homo sapiens
1	1.00	1	1	0	0.002	0.005	0.001	-0.002	t	72321	WDMMMNWSP	Homo sapiens
1	1.00	1	1	0.01	0.022	-0.006	0.01	0.01	t	21373	GNAEKYDKMDEPQHYGKS	Mus musculus
1	1.00	1	1	0	0	0	0.001	-0.002	t	40332	LVFFAEDV	Mus musculus
1	1.00	1	1	0	0	-0.001	0	0	t	126328	ISHG	Mus musculus
1	1.00	1	1	-0.02	-0.02	-0.02	-0.02	-0.02	t	32648	KPAIIPDRE	Homo sapiens
1	1.00	1	1	0.03	0.027	0.029	0.03	0.031	t	63811	TGEDPGFFNV	Oryctolagus cuniculus
1	1.00	1	1	-0.05	-0.048	-0.054	-0.053	-0.052	t	114454	LIYIERGRITGVLF	Homo sapiens
1	1.00	1	1	0.03	0.027	0.019	0.03	0.03	t	16120	FHLGDDMDPELRGL	Oryctolagus cuniculus
1	1.00	1	1	0.03	0.031	0.019	0.03	0.03	t	16120	FHLGDDMDPELRGL	Myodes glareolus
1	1.00	1	1	0.01	0.008	0.001	0.013	0.01	t	11148	EANNREGVEG	Mus musculus
1	1.00	1	1	0	0.003	-0.011	0	0	t	42382	MQKGFVSINDNKHYFDDSG	Mus musculus

1	1.00	1	1	0.06	0.057	0.064	0.06	0.06	t	78806	QSQIESR	Oryctolagus cuniculus
1	1.00	1	1	0.06	0.06	0.064	0.06	0.06	t	78806	QSQIESR	Oryctolagus cuniculus
1	1.00	1	1	0	0	-0.007	0	-0.001	t	158938	KTQNQGPWDEGRVEID	Mus musculus
1	1.00	1	1	0.04	0.04	0.033	0.04	0.041	t	96788	QQEQQFK	Mus musculus
1	1.00	1	1	-0.02	-0.02	-0.02	-0.02	-0.02	t	128088	QVYYRPV	Ovis aries
1	1.00	1	1	-0.02	-0.018	-0.02	-0.013	-0.02	t	108104	RRLPFGSQM	Homo sapiens
1	1.00	1	1	0.05	0.054	0.047	0.047	0.05	t	13587	ENSEKTTMPL	Homo sapiens
1	1.00	1	1	0.01	0.01	-0.003	0.013	0.012	t	73036	WRIYSTGNNVFQTQAGC	Homo sapiens
1	1.00	1	1	0.01	0.01	-0.003	0.013	0.012	t	73036	WRIYSTGNNVFQTQAGC	Homo sapiens
1	1.00	1	1	0.03	0.033	0.024	0.031	0.03	t	41868	MKNNPVYSESL	Equus caballus
1	1.00	1	1	-0.01	-0.008	-0.014	-0.007	-0.008	t	20152	GHLAPL	Homo sapiens
1	1.00	1	1	0	-0.003	0	0	-0.003	t	6884	CQKAGGVAL	Oryctolagus cuniculus
1	1.00	1	1	0.02	0.02	0.02	0.02	0.02	t	21720	GPPAAEY	Homo sapiens
1	1.00	1	1	0	-0.009	0.001	0	0	t	109750	QGPKGEPG	Macaca mulatta

1	1.00	1	1	0	0	-0.005	-0.003	-0.002	t	76131	YTNGPQEIYIQQGNG	Homo sapiens
1	1.00	1	1	0	0	0	0.004	-0.001	t	71284	VTEEI	Homo sapiens
1	1.00	1	1	0.02	0.02	0.031	0.02	0.02	t	71855	VVTLYL	Homo sapiens
1	1.00	1	1	0.03	0.03	0.023	0.03	0.029	t	131114	TQLDELLTEHRMTWDPAQPP	Homo sapiens
1	1.00	1	1	0	0	-0.004	0.003	0.002	t	38496	LPGCSFSIFLLA	Homo sapiens
1	1.00	1	1	-0.03	-0.028	-0.031	-0.023	-0.03	t	144759	RAALPSHVVT	Homo sapiens
1	1.00	1	1	0.01	0.01	0.01	0.01	0.01	t	79462	VTSTYH	Mus musculus
1	1.00	1	1	0.05	0.046	0.047	0.05	0.05	t	141067	PLSLEPDP	Mus musculus
1	1.00	1	1	0.02	0.02	0.02	0.02	0.02	t	99276	GGKGPLLSILKAFN	Homo sapiens
1	1.00	1	1	-0.02	-0.02	-0.02	-0.02	-0.019	t	115202	DDLTDDIMCV	Homo sapiens
1	1.00	1	1	-0.03	-0.03	-0.036	-0.033	-0.03	t	9633	DPFFPWPRTTFD	Homo sapiens
1	1.00	1	1	0.07	0.066	0.07	0.069	0.068	t	122746	EEQEDEIIGFGQELKN	Oryctolagus cuniculus
1	1.00	1	1	0.03	0.03	0.03	0.03	0.03	t	135465	GSDYEDRYYREN	Mus musculus
1	1.00	1	1	0.02	0.018	0.009	0.023	0.02	t	46072	NTDGSTDYGILQINSR	Mus musculus
1	1.00	1	1	0.01	0.008	0.005	0.011	0.011	t	30629	KETLHSTSPEGN	Homo sapiens
1	1.00	1	1	-0.01	-0.008	-0.011	-0.003	-0.01	t	134799	SRKPEAPRRRSPSPT	Homo sapiens

1	1.00	1	1	0.02	0.017	0.022	0.02	0.02	t	57289	SDSQTITN PAYD	Oryctolagus cuniculus
1	1.00	1	1	-0.03	-0.03	-0.033	-0.03	-0.03	t	29825	KAIERMKD TL	Mus musculus
1	1.00	1	1	0.01	0.014	0.019	0.01	0.006	t	140730	QFAYDGK DY	Mus musculus
1	1.00	1	1	0	0	0	0	0	t	77781	GLVGRPKS KL	Homo sapiens
1	1.00	1	1	0	0	0	0	0	t	3288	ANGAGNQ PG	Homo sapiens
1	1.00	1	1	0	0	0	0	0	t	3288	ANGAGNQ PG	Homo sapiens
1	1.00	1	1	0	0	0	0	0	t	3288	ANGAGNQ PG	Homo sapiens
1	1.00	1	1	0.02	0.02	0.02	0.02	0.018	t	61842	STPVAPTQ EV	Oryctolagus cuniculus
1	1.00	1	1	0.01	0.01	0.01	0.01	0.007	t	46774	PAEEEK	Mus musculus
1	1.00	1	1	0.01	0.014	0.01	0.013	0.01	t	131978	MLVGGST LCV	Homo sapiens
1	1.00	1	1	0.02	0.02	0.014	0.02	0.018	t	64032	THGQWN KPSKPKTN MK	Oryctolagus cuniculus
1	1.00	1	1	-0.03	-0.026	-0.039	-0.033	-0.032	t	117396	VKNLHNQ ARS	Homo sapiens
1	1.00	1	1	-0.02	-0.02	-0.033	-0.023	-0.02	t	147033	IILHQQHH	Homo sapiens
1	1.00	1	1	-0.01	-0.008	-0.005	-0.007	-0.011	t	102563	KKRPKPG	Homo sapiens
1	1.00	1	1	0.02	0.02	0.029	0.02	0.02	t	39936	LTGYGT	Homo sapiens

1	1.00	1	1	0.04	0.038	0.04	0.033	0.038	t	117509	GQIPPGAM	Mus musculus
1	1.00	1	1	0.03	0.032	0.039	0.037	0.032	t	109779	QVSLTCL	Homo sapiens
1	1.00	1	1	0.03	0.03	0.03	0.03	0.03	t	112854	TSPSVAY	Homo sapiens
1	1.00	1	1	0.03	0.03	0.03	0.033	0.03	t	149093	QGRNPTQ	Sus scrofa
1	1.00	1	1	0.01	0.012	0.008	0.013	0.01	t	115619	IRALVGDEVELP	Homo sapiens
1	1.00	1	1	0.02	0.016	0.018	0.019	0.018	t	72507	WGQGGTHGQWNKPSK	Oryctolagus cuniculus
1	1.00	1	1	0.02	0.016	0.018	0.019	0.018	t	72507	WGQGGTHGQWNKPSK	Oryctolagus cuniculus
1	1.00	1	1	-0.01	-0.022	-0.008	-0.01	-0.013	t	146185	DAEFGH	Macaca mulatta
1	1.00	1	1	-0.04	-0.042	-0.044	-0.043	-0.04	t	65114	TLTWSGKF	Anas platyrhynchos
1	1.00	1	1	-0.03	-0.026	-0.041	-0.029	-0.03	t	18053	FTQCSMNVVSKTLFRKSPIY	Homo sapiens
1	1.00	1	1	-0.01	-0.011	-0.007	-0.007	-0.01	t	29744	KAAASKVKANLLSV	Homo sapiens
1	1.00	1	1	0.02	0.022	0.013	0.017	0.017	t	141751	SQIQRPVKELAFPGS	Homo sapiens
1	1.00	1	1	0.05	0.05	0.041	0.046	0.05	t	135990	SASVPALTAVET	Homo sapiens
1	1.00	1	1	0.01	0.01	0.008	0.01	0.01	t	117551	KREAVAGRGRGRGRG	Mus musculus
1	1.00	1	1	0.05	0.051	0.049	0.05	0.05	t	63979	TGTIINQDPDKILTY	Mus musculus
1	1.00	1	1	-0.02	-0.02	-0.02	-0.02	-0.019	t	115347	LIVTQTMKGL	Homo sapiens

1	1.00	1	1	0	0.004	0.007	0.007	0.001	t	135546	DWEYS	Homo sapiens
1	1.00	1	1	-0.01	-0.01	-0.013	-0.011	-0.01	t	60914	SSATK	Homo sapiens
1	1.00	1	1	0.04	0.04	0.04	0.04	0.04	t	99185	EENEGGNI FSGFTPE	Homo sapiens
1	1.00	1	1	0.04	0.044	0.033	0.041	0.04	t	137859	FTPGTEYT VEVR	Homo sapiens
1	1.00	1	1	0.05	0.05	0.05	0.05	0.049	t	53923	RGLTSLFS AFSAQ	Homo sapiens
1	1.00	1	1	0.01	0.014	0.01	0.011	0.01	t	155473	PSTP	Homo sapiens
1	1.00	1	1	0.01	0.01	0.01	0.01	0.007	t	149298	ELDKWAS	Homo sapiens
1	1.00	1	1	0.03	0.026	0.021	0.029	0.03	t	12259	EGRFKSQD AD	Oryctolagus cuniculus
1	1.00	1	1	0	0.003	0	0	-0.002	t	44554	NKND	Mus musculus
1	1.00	1	1	-0.02	-0.024	-0.019	-0.023	-0.022	t	44276	NILISLTDL EIW	Oryctolagus cuniculus
1	1.00	1	1	0.03	0.032	0.028	0.027	0.028	t	116740	GRDPYSPS QDPYSPS	Homo sapiens
1	1.00	1	1	0.02	0.02	0.02	0.02	0.018	t	42425	MQWNST	Mus musculus
1	1.00	1	1	0.03	0.026	0.023	0.033	0.03	t	106789	RSFFSFLGE AFDGARD M	Oryctolagus cuniculus
1	1.00	1	1	-0.03	-0.03	-0.03	-0.023	-0.029	t	110385	QLYKTCKQ AGTCPPDII PKV	Homo sapiens

1	1.00	1	1	-0.02	-0.018	-0.02	-0.023	-0.022	t	70443	VPQKAVP YPQRD	Homo sapiens
1	1.00	1	1	0.01	0.008	-0.001	0.01	0.011	t	123282	HACQKKLL KFEALQQE EGEE	Oryctola gus cuniculu s
1	1.00	1	1	0.01	0.01	-0.001	0.01	0.011	t	123282	HACQKKLL KFEALQQE EGEE	Oryctola gus cuniculu s
1	1.00	1	1	0	0	-0.008	0	0.002	t	38700	LPSAVK	Mus musculu s
1	1.00	1	1	0.04	0.037	0.032	0.04	0.04	t	7383	CYGVSATK LNDLCFSN V	Oryctola gus cuniculu s
1	1.00	1	1	0.04	0.037	0.032	0.04	0.04	t	7383	CYGVSATK LNDLCFSN V	Oryctola gus cuniculu s
1	1.00	1	1	0	0.002	-0.005	0.003	0	t	44569	NKPNNDF HFEVF	Homo sapiens
1	1.00	1	1	0	0	0	0	-0.002	t	137734	DTALTKAA LKLV	Mus musculu s
1	1.00	1	1	-0.01	-0.01	-0.01	-0.01	-0.011	t	112816	PGKVCGS NLLSICKTA EF	Mus musculu s
1	1.00	1	1	0.04	0.04	0.042	0.04	0.04	t	139029	DLQVLT	Mus musculu s
1	1.00	1	1	-0.01	-0.01	-0.026	-0.014	-0.008	t	59212	SLKEKLQK	Homo sapiens
1	1.00	1	1	-0.01	-0.006	-0.016	-0.003	-0.008	t	8930	DKLKQQR DTLSTQKE T	Homo sapiens
1	1.00	1	1	-0.02	-0.02	-0.02	-0.02	-0.023	t	111589	NLIRHVGG GTPR	Homo sapiens

1	1.00	1	1	-0.01	-0.01	-0.008	-0.01	-0.013	t	70391	VPNLRGDLQVLAQKVARTLP	Cavia porcellus
1	1.00	1	1	0.01	0.017	0.01	0.01	0.01	t	7171	CTKPTDGNC	Cavia porcellus
1	1.00	1	1	0.01	0.017	0.01	0.01	0.01	t	7171	CTKPTDGNC	Capra hircus
1	1.00	1	1	0	-0.003	-0.011	0	0.001	t	144748	QAVNPNAGN	Oryctolagus cuniculus
1	1.00	1	1	0	-0.002	-0.002	-0.001	0	t	10835	DWVKGFPIDSSC	Mus musculus
1	1.00	1	1	0	-0.004	-0.002	-0.001	0	t	10835	DWVKGFPIDSSC	Oryctolagus cuniculus
1	1.00	1	1	-0.02	-0.016	-0.025	-0.019	-0.02	t	53763	RFQEALKKSLKKP	Homo sapiens
1	1.00	1	1	0.07	0.067	0.055	0.063	0.07	t	104057	LTEPTTEGDQHPGTLPAANT	Oryctolagus cuniculus
1	1.00	1	1	-0.01	-0.006	-0.01	-0.007	-0.01	t	118430	ISDAMKKVGRKGV	Homo sapiens
1	1.00	1	1	0	0	0.002	0	0	t	79428	TKLKGESGY	Oryctolagus cuniculus
1	1.00	1	1	0.01	0.008	0	0.013	0.01	t	8475	DGQPAGDRADGQPAGDRA	Mus musculus
1	1.00	1	1	0.05	0.05	0.038	0.046	0.05	t	28907	ITGEYVQILDAHAAY	Homo sapiens

1	1.00	1	1	0.05	0.05	0.05	0.05	0.048	t	153460	SFEVDPID ENSG	Mus musculu s
1	1.00	1	1	0.04	0.042	0.04	0.047	0.04	t	139616	SYNRGDST FESKSYK	Homo sapiens
1	1.00	1	1	0.01	0.01	0.011	0.01	0.008	t	60594	SQWNKPS K	Mus musculu s
1	1.00	1	1	0	0	0.003	0	0	t	68763	VGPQSVG PL	Mus musculu s
1	1.00	1	1	-0.01	-0.009	-0.014	-0.009	-0.01	t	52058	QQLIVARQ KL	Homo sapiens
1	1.00	1	1	0.01	0.01	0.01	0.014	0.01	t	119579	FKDYSSVV RPVEDHR Q	Homo sapiens
1	1.00	1	1	0.01	0.008	0.006	0.007	0.01	t	55761	RRVGLSNP T	Anas platyrhy nchos
1	1.00	1	1	0.03	0.026	0.029	0.029	0.028	t	65944	TQTADVPT EAL	Oryctola gus cuniculu s
1	1.00	1	1	0	-0.002	0	-0.001	-0.003	t	58409	SHSAFNM FDFGVGPP	Mus musculu s
1	1.00	1	1	0	-0.002	0	-0.001	-0.003	t	58416	SHSQLGPP VGGPP	Mus musculu s
1	1.00	1	1	0	-0.002	0	-0.001	-0.003	t	21026	GLLRYHTY ARFG	Mus musculu s
1	1.00	1	1	0	-0.002	0	-0.001	-0.003	t	18363	FWRGDLV FDFQV	Mus musculu s
1	1.00	1	1	0.03	0.027	0.03	0.03	0.028	t	125414	FSEEKLVFS LRLMEEN	Oryctola gus cuniculu s
1	1.00	1	1	-0.03	-0.03	-0.031	-0.027	-0.03	t	51841	QPGLRR	Homo sapiens
1	1.00	1	1	-0.02	-0.02	-0.015	-0.02	-0.022	t	140488	MSLLGHK	Mus musculu s
1	1.00	1	1	-0.04	-0.062	-0.047	-0.033	-0.04	t	127733	LWRCALR WAERQVG A	Homo sapiens

1	1.00	1	1	0.02	0.02	0.02	0.02	0.02	t	133371	SPSPTPTP GP	Homo sapiens
1	1.00	1	1	0.02	0.02	0.015	0.02	0.02	t	12688	EKFVKEQR	Homo sapiens
1	1.00	1	1	-0.02	-0.02	-0.02	-0.016	-0.02	t	70975	VSHDRHFL N	Homo sapiens
1	1.00	1	1	-0.03	-0.026	-0.037	-0.027	-0.028	t	144594	ARGISRTK YW	Homo sapiens
1	1.00	1	1	-0.01	-0.014	-0.022	-0.011	-0.01	t	62845	TAFSKN	Oryctola gus cuniculu s
1	1.00	1	1	0.01	0.013	0.003	0.01	0.012	t	113194	TGGAYQSY KF	Mus musculu s
1	1.00	1	1	0	-0.002	-0.013	-0.003	0	t	65514	TPAYYTKD TNNNLTLV	Mus musculu s
1	1.00	1	1	0.03	0.028	0.03	0.033	0.03	t	6281	CGELGVDC	Mus musculu s
1	1.00	1	1	0.02	0.02	0.009	0.02	0.02	t	138229	QPAPNAQ PAPGAPA	Oryctola gus cuniculu s
1	1.00	1	1	0.01	0.01	0	0.007	0.011	t	96085	ALCSEKLD QWLCEKL	Homo sapiens
1	1.00	1	1	0	0	0	0	0.001	t	9611	DPDKDVDI MV	Mus musculu s
1	1.00	1	1	0	-0.003	0	0	-0.001	t	24652	HQTLQDP RVRGL	Oryctola gus cuniculu s
1	1.00	1	1	0.03	0.03	0.024	0.03	0.03	t	55066	RNTIMASK TVGTAE	Homo sapiens
1	1.00	1	1	-0.02	-0.018	-0.03	-0.019	-0.02	t	50442	QDAYNAA GGHNAVF NFPPNG	Homo sapiens

1	1.00	1	1	0.01	0.01	0.01	0.01	0.008	t	120920	RLVEHLLD PSRYNKLIR	Oryctola gus cuniculu s
1	1.00	1	1	0	0.002	0.005	0.007	0.002	t	129718	RATPPPGI	Homo sapiens
1	1.00	1	1	0	-0.003	-0.005	0	0	t	57680	SEWRSPA DTSPRPLP	Oryctola gus cuniculu s
1	1.00	1	1	-0.02	-0.015	-0.024	-0.017	-0.02	t	77808	YGCWKGL VGR	Homo sapiens
1	1.00	1	1	0.02	0.02	0.02	0.02	0.019	t	118196	VEPTGKRF L	Homo sapiens
1	1.00	1	1	-0.03	-0.028	-0.03	-0.023	-0.03	t	107366	FPPKPKD	Homo sapiens
1	1.00	1	1	-0.05	-0.046	-0.059	-0.047	-0.05	t	132763	YMASLQM RGNPGSH F	Homo sapiens
1	1.00	1	1	-0.02	-0.02	-0.02	-0.02	-0.021	t	136059	EGY	Rattus norvegic us
1	1.00	1	1	0.05	0.048	0.056	0.049	0.05	t	67967	VDGSDP	Mus musculu s
1	1.00	1	1	0.02	0.022	0.016	0.017	0.018	t	114948	SIEFGTNIS K	Homo sapiens
1	1.00	1	1	0.04	0.04	0.044	0.046	0.04	t	109620	NDGQPGP A	Rattus norvegic us
1	1.00	1	1	0.02	0.02	0.02	0.02	0.02	t	7058	CSQAAPYI E	Homo sapiens
1	1.00	1	1	0	0	-0.008	0	0	t	102942	YGCYCGK GG	Mus musculu s
1	1.00	1	1	0	0	-0.008	0	0	t	102942	YGCYCGK GG	Mus musculu s

1	1.00	1	1	-0.05	-0.048	-0.045	-0.043	-0.05	t	117169	RYHRFKEL STLRR	Homo sapiens
1	1.00	1	1	0	-0.003	0	0.001	-0.002	t	7491	DAEFRHDS GYEVHHQ K	Oryctolagus cuniculus
1	1.00	1	1	0.02	0.02	0.011	0.02	0.02	t	48858	PPPPNPN DPPPPNP ND	Mus musculus
1	1.00	1	1	0.02	0.02	0.011	0.02	0.02	t	48858	PPPPNPN DPPPPNP ND	Mus musculus
1	1.00	1	1	-0.01	-0.011	-0.019	-0.01	-0.01	t	64752	TLDHVLDH	Anas platyrhynchos
1	1.00	1	1	0.03	0.03	0.027	0.03	0.027	t	114558	QVVRPERS RQEWERQ	Homo sapiens
1	1.00	1	1	-0.02	-0.027	-0.027	-0.013	-0.02	t	136137	APDRKGFR LLLASPRSC YKL	Homo sapiens
1	1.00	1	1	-0.04	-0.04	-0.043	-0.04	-0.041	t	136074	LVKK	Mus musculus
1	1.00	1	1	0	0	0	0	-0.001	t	98981	REREEDW RQP	Homo sapiens
1	1.00	1	1	-0.02	-0.009	-0.026	-0.02	-0.02	t	41126	MASLIYRQ LL	Gallus gallus
1	1.00	1	1	-0.02	-0.02	-0.034	-0.02	-0.02	t	31005	KGLRRDLD ASREAKKQ	Mus musculus
1	1.00	1	1	0.01	0.01	0.01	0.01	0.01	t	33834	KTQYSKF	Mus musculus
1	1.00	1	1	0	0.004	-0.012	-0.003	-0.001	t	104996	TAFCGPVG AH	Homo sapiens
1	1.00	1	1	-0.02	-0.02	-0.02	-0.02	-0.021	t	48837	PPNAPIL	Mus musculus

1	1.00	1	1	0.05	0.036	0.045	0.05	0.05	t	50156	PYQGSGKGVS	Oryctolagus cuniculus
1	1.00	1	1	0	-0.002	-0.005	-0.001	0	t	20137	GHGSETAWGT	Mus musculus
1	1.00	1	1	0	0.003	-0.011	0	0.002	t	111510	LPSEATVYLPPVPVS	Mus musculus
1	1.00	1	1	0.02	0.02	0.02	0.02	0.017	t	115435	QEQNQEQPIRCEKDERFFSD	Homo sapiens
1	1.00	1	1	-0.02	-0.018	-0.029	-0.021	-0.019	t	53693	RFDRGYIS	Homo sapiens
1	1.00	1	1	0.02	0.02	0.026	0.02	0.02	t	34238	KVVLPE	Homo sapiens
1	1.00	1	1	0	-0.004	0	-0.001	-0.001	t	24009	HHPSTDKEQTNLY	Oryctolagus cuniculus
1	1.00	1	1	-0.06	-0.057	-0.065	-0.06	-0.062	t	131967	LRLDCRHEN	Mus musculus
1	1.00	1	1	0.01	0.01	0.01	0.01	0.008	t	146624	WNWFDITN	Homo sapiens
1	1.00	1	1	0	0	-0.006	0	0	t	102943	YGCYCGLGG	Mus musculus
1	1.00	1	1	-0.03	-0.024	-0.039	-0.031	-0.031	t	11802	EENKKPAE	Ovis aries
1	1.00	1	1	-0.03	-0.028	-0.037	-0.023	-0.03	t	131053	RRVQQEIDDVIGQVRRPEMG	Homo sapiens
1	1.00	1	1	-0.06	-0.064	-0.069	-0.061	-0.06	t	74135	YHRVMYAS	Oryctolagus cuniculus

1	1.00	1	1	0	0.004	-0.003	0.001	0	t	85032	LCVYGKLQKN	Homo sapiens
1	1.00	1	1	0.01	0.01	0.004	0.01	0.01	t	158910	KNLDIDPKLQEYLGK	Mus musculus
1	1.00	1	1	-0.01	-0.01	-0.01	-0.01	-0.01	t	158610	SFGTFLW	Homo sapiens
1	1.00	1	1	-0.04	-0.04	-0.052	-0.042	-0.04	t	96028	YSWRSKYGWT	Homo sapiens
1	1.00	1	1	0.02	0.016	0.007	0.021	0.021	t	68054	VDREGRDNVRSKGV	Oryctolagus cuniculus
1	1.00	1	1	0.01	0.01	0.007	0.01	0.01	t	98763	ENKVLVLDTD	Homo sapiens
1	1.00	1	1	0.06	0.06	0.054	0.06	0.06	t	105979	NIEADVNT	Oryctolagus cuniculus
1	1.00	1	1	-0.03	-0.028	-0.023	-0.027	-0.033	t	142007	SDWRFLRGYHQYA	Homo sapiens
1	1.00	1	1	0	0.002	0.001	0.007	0	t	98387	QPPCPNLVSYSSYHATYS	Homo sapiens
1	1.00	1	1	0.02	0.02	0.02	0.019	0.018	t	50505	QDPR	Mus musculus
1	1.00	1	1	0.03	0.028	0.029	0.029	0.03	t	12953	ELAPEDPEDSALLED	Mus musculus
1	1.00	1	1	0	0	0	0	0.001	t	9934	DRAAGQPAG	Mus musculus
1	1.00	1	1	0	0	0	0	0.001	t	9934	DRAAGQPAG	Mus musculus
1	1.00	1	1	0.01	0.01	0.01	0.01	0.01	t	113778	QQPGQ	Homo sapiens
1	1.00	1	1	0.04	0.04	0.045	0.04	0.038	t	120804	MRSFSLSTNLQESL	Homo sapiens

1	1.00	1	1	-0.01	-0.007	-0.015	-0.01	-0.01	t	105228	GLMVGGV V	Mus musculu s
1	1.00	1	1	-0.01	-0.01	-0.015	-0.01	-0.01	t	105228	GLMVGGV V	Mus musculu s
1	1.00	1	1	-0.01	-0.01	-0.01	-0.01	-0.011	t	24105	HITHYFVV PSVS	Homo sapiens
1	1.00	1	1	0.01	0.01	-0.001	0.01	0.01	t	153469	STETGNQ HHYQTRV VSNANK	Mus musculu s
1	1.00	1	1	-0.03	-0.026	-0.031	-0.029	-0.031	t	71262	VTCAMF	Homo sapiens
1	1.00	1	1	-0.02	-0.022	-0.04	-0.027	-0.02	t	127950	DVPVVTSV TTAGVGPS PDIT	Mus musculu s
1	1.00	1	1	0.02	0.02	0.02	0.024	0.02	t	135926	GADIKKT MEGPV	Homo sapiens
1	1.00	1	1	0.01	0.009	0.002	0.007	0.01	t	37997	LMCDIDG NRL	Homo sapiens
1	1.00	1	1	-0.04	-0.04	-0.052	-0.04	-0.039	t	53864	RGFLGRM LF	Homo sapiens
1	1.00	1	1	-0.03	-0.028	-0.021	-0.023	-0.03	t	102643	MASPGSG FWSFG	Homo sapiens
1	1.00	1	1	0	0	0	0	0	t	112849	TIKNTFF	Homo sapiens
1	1.00	1	1	-0.03	-0.026	-0.03	-0.029	-0.03	t	26234	IGGPLT	Homo sapiens
1	1.00	1	1	0.01	0.01	0.01	0.01	0.008	t	140507	TKTWQSM	Mus musculu s
1	1.00	1	1	-0.02	-0.018	-0.015	-0.013	-0.019	t	122175	RLGKRDS	Homo sapiens
1	1.00	1	1	0.01	0.01	0.01	0.006	0.008	t	134006	ALRMEVL GCEAQDLY	Homo sapiens
1	1.00	1	1	-0.01	-0.01	-0.017	-0.01	-0.01	t	96014	YGWTAFC GPV	Homo sapiens

1	1.00	1	1	-0.01	-0.01	-0.01	-0.012	-0.01	t	40793	LYIGGP	Homo sapiens
1	1.00	1	1	0.01	0.01	0.01	0.014	0.01	t	118615	SVLTQSVK NNTQ	Homo sapiens
1	1.00	1	1	-0.02	-0.022	-0.026	-0.021	-0.02	t	28389	IRRPKHLY VS	Mus musculu s
1	1.00	1	1	0.05	0.045	0.043	0.046	0.05	t	42678	MSTLQEL QENITA	Mus
1	1.00	1	1	0.03	0.03	0.018	0.03	0.031	t	9598	DPAPPNA NDPAPPN AND	Mus musculu s
1	1.00	1	1	0.01	0.012	0.009	0.007	0.01	t	8383	DGFKVSNS SA	Homo sapiens
1	1.00	1	1	-0.01	-0.013	-0.023	-0.01	-0.01	t	21277	GMGVSCT VTREDGT NRR	Oryctola gus cuniculu s
1	1.00	1	1	0.01	0.013	-0.003	0.01	0.01	t	7649	DASREAKK QVEKALEE ANSK	Mus musculu s
1	1.00	1	1	0.01	0.01	0.001	0.016	0.012	t	72252	WAPTAPL PGDMNPA N	Homo sapiens
1	1.00	1	1	-0.01	-0.008	-0.012	-0.003	-0.009	t	122732	DRKASPPS GLWSPAY	Homo sapiens
1	1.00	1	1	-0.01	-0.01	-0.01	-0.01	-0.013	t	156938	PPEKIHV	Gallus gallus
1	1.00	1	1	0.02	0.022	0.013	0.027	0.022	t	142386	ANKYQISV NKYRGTA GNAL	Homo sapiens
1	1.00	1	1	0	0	0	0	0	t	140934	YGEEKPAV LGHD	Oryctola gus cuniculu s
1	1.00	1	1	0.03	0.027	0.016	0.03	0.034	t	97247	DNTFVTTS L	Oryctola gus cuniculu s

1	1.00	1	1	0	0	-0.005	0	-0.002	t	62961	TAPTDPTERTVWTFG	Oryctolagus cuniculus
1	1.00	1	1	0.01	0.013	0.004	0.01	0.012	t	133727	VERKECAY	Mus musculus
1	1.00	1	1	0	-0.003	-0.011	0	0.001	t	144748	QAVNPNAGN	Oryctolagus cuniculus
1	1.00	1	1	0	-0.002	0	-0.001	0	t	48599	PNANPNANPNA	Mus musculus
1	1.00	1	1	-0.04	-0.036	-0.047	-0.033	-0.038	t	133232	MGPRRRSRKPEA	Homo sapiens
1	1.00	1	1	-0.01	-0.013	-0.018	-0.01	-0.008	t	68182	VEFPLDITAG	Oryctolagus cuniculus
1	1.00	1	1	-0.01	-0.01	-0.018	-0.01	-0.008	t	68182	VEFPLDITAG	Mus musculus
1	1.00	1	1	0	0	-0.012	0	0.001	t	12375	EHWSYGLRPG	Canis familiaris
1	1.00	1	1	0	0.002	-0.005	0	0	t	107246	TEIPKEEKDPGMGAM	Rattus norvegicus
1	1.00	1	1	-0.02	-0.02	-0.024	-0.02	-0.02	t	60792	SRRYPFVKF	Homo sapiens
1	1.00	1	1	0	0.002	0	0.007	0.002	t	121287	EYFIRK	Homo sapiens
1	1.00	1	1	0	0.002	-0.017	-0.003	0	t	32970	KQEGDKCVENPNPTCNENN	Homo sapiens
1	1.00	1	1	0	0.003	0.005	0	0.001	t	116284	CAYSVAHLC	Mus musculus

1	1.00	1	1	0.06	0.062	0.055	0.061	0.062	t	19621	GFSYDT	Homo sapiens
1	1.00	1	1	0	0	0.001	0	-0.001	t	6561	CLFYASEM SEKGFKVIF	Oryctolagus cuniculus
1	1.00	1	1	0.03	0.028	0.023	0.023	0.029	t	108131	SKDWSFY	Mus musculus
1	1.00	1	1	-0.02	-0.02	-0.02	-0.02	-0.02	t	99266	GDLIAVPT GVAFWLY	Homo sapiens
1	1.00	1	1	0.01	0.012	0.003	0.011	0.01	t	54206	RIPADVID AFHAAT	Homo sapiens
1	1.00	1	1	0.01	0.005	0.008	0.01	0.01	t	7761	DDEADRYF TYEE	Bos taurus
1	1.00	1	1	0.04	0.042	0.04	0.047	0.042	t	117948	KFVETPGQ KYKETDL	Homo sapiens
1	1.00	1	1	0.04	0.04	0.04	0.047	0.042	t	117948	KFVETPGQ KYKETDL	Mus musculus
1	1.00	1	1	0.01	0.01	0.01	0.01	0.01	t	111089	ADVMTYI HSMNSTIL	Mus musculus
1	1.00	1	1	0.01	0.01	0.01	0.01	0.01	t	111089	ADVMTYI HSMNSTIL	Mus musculus
1	1.00	1	1	0.01	0.01	0.01	0.01	0.01	t	111089	ADVMTYI HSMNSTIL	Mus musculus
1	1.00	1	1	0	0	0	0	0	t	52420	QSRGEAR	Mus musculus
1	1.00	1	1	0.04	0.044	0.039	0.047	0.04	t	124367	KLKARVDE LERIRRS	Homo sapiens
1	1.00	1	1	0	-0.004	-0.001	-0.001	-0.002	t	5904	AYQQGVT VDSIGMLP RFIPE	Oryctolagus cuniculus
1	1.00	1	1	0	0.002	0.004	0.007	0.002	t	145657	YRSYNDSV DP	Homo sapiens

1	1.00	1	1	0.01	0.014	0.007	0.013	0.01	t	67059	TVLAQALVRE	Homo sapiens
1	1.00	1	1	-0.03	-0.033	-0.035	-0.03	-0.027	t	133683	RQRY	Oryctolagus cuniculus
1	1.00	1	1	-0.01	-0.013	-0.005	-0.01	-0.012	t	7143	CTGDARHC	Oryctolagus cuniculus
1	1.00	1	1	-0.01	-0.01	-0.01	-0.01	-0.011	t	66060	TRLNPD	Mus musculus
1	1.00	1	1	0.02	0.024	0.011	0.02	0.02	t	139880	RDEYDHVLSAKLAEQYDT	Ovis aries
1	1.00	1	1	0.01	0.013	0.01	0.01	0.011	t	21601	GPEET	Mus musculus
1	1.00	1	1	0.04	0.037	0.036	0.041	0.04	t	55627	RRKLTNPANKEES	Homo sapiens
1	1.00	1	1	0	0	-0.004	0	0	t	106862	SPEFRRD	Mus musculus
1	1.00	1	1	0	0.002	0.007	0.007	0	t	141969	NGTYQTW	Homo sapiens
1	1.00	1	1	0	0.002	0.007	0.007	0	t	141969	NGTYQTW	Homo sapiens
1	1.00	1	1	0.03	0.034	0.03	0.031	0.03	t	134696	ITLYLTEK	MD
1	1.00	1	1	-0.01	-0.01	-0.01	-0.01	-0.009	t	115304	KCEVFRELKD	Homo sapiens
1	1.00	1	1	0.01	0.008	0.01	0.009	0.011	t	9934	DRAAGQPAG	Mus musculus
1	1.00	1	1	0	0	0	0	-0.001	t	133722	TSKSKYPLVV	Mus musculus
1	1.00	1	1	-0.06	-0.06	-0.06	-0.06	-0.06	t	78504	RLFLHV	Mus musculus

1	1.00	1	1	0.01	0.014	0.01	0.017	0.012	t	125600	WESYPFRVGTSL	Homo sapiens
1	1.00	1	1	0.07	0.07	0.07	0.07	0.07	t	145172	EPPDDDDS	Mus musculus
1	1.00	1	1	-0.01	-0.008	-0.008	-0.003	-0.01	t	119674	WNPDDYGGVKKIHIPS	Homo sapiens
1	1.00	1	1	-0.01	-0.006	-0.013	-0.009	-0.011	t	103162	FCGRIHTRYSSAYEL	Homo sapiens
1	1.00	1	1	0.02	0.02	0.02	0.02	0.02	t	121828	RVYA	Mus musculus
1	1.00	1	1	-0.01	-0.01	-0.01	-0.01	-0.011	t	153393	GPDAPRIPLSKM	Mus musculus
1	1.00	1	1	0.05	0.046	0.047	0.05	0.05	t	141004	EPDP	Mus musculus
1	1.00	1	1	0	0	-0.006	0	0	t	91966	SLMVALDSNNTMPFT	Oryctolagus cuniculus
1	1.00	1	1	-0.02	-0.018	-0.016	-0.013	-0.02	t	144697	IYRNLYWEGS	Homo sapiens
1	1.00	1	1	-0.02	-0.018	-0.02	-0.013	-0.02	t	107364	FNWYVDG	Homo sapiens
1	1.00	1	1	0.02	0.022	0.017	0.023	0.02	t	49954	PVSATAMAPSVDPSAEPTAP	Homo sapiens
1	1.00	1	1	0	0	-0.007	0	0	t	76606	YYSPAF	Mus musculus
1	1.00	1	1	0.02	0.018	0.021	0.019	0.02	t	122156	NLHIDPTLI	Mus musculus
1	1.00	1	1	0.04	0.04	0.04	0.04	0.04	t	140507	TKTWQSM	Mus musculus

1	1.00	1	1	0	-0.004	-0.012	0.003	0	t	106398	EQLAEVAVSAKR	Oryctolagus cuniculus
1	1.00	1	1	0.02	0.023	0.019	0.019	0.02	t	76014	YTESMAGKRE	Mus musculus
1	1.00	1	1	-0.01	-0.014	-0.015	-0.007	-0.01	t	60959	SSDSQTITNPAY	Oryctolagus cuniculus
1	1.00	1	1	0.02	0.02	0.013	0.02	0.02	t	96210	EDKEEDEEED	Homo sapiens
1	1.00	1	1	0.02	0.022	0.013	0.023	0.02	t	117015	KEQFLDGDGWTSRWIESK	Homo sapiens
1	1.00	1	1	-0.01	-0.01	-0.018	-0.01	-0.012	t	89660	TQKIPKANAADADTDTT	Mus musculus
1	1.00	1	1	-0.04	-0.04	-0.04	-0.031	-0.038	t	96808	QVKSGTIFDN	Homo sapiens
1	1.00	1	1	0.04	0.04	0.04	0.04	0.04	t	18511	GAALLQSA	Oryctolagus cuniculus
1	1.00	1	1	0.01	0.012	0.006	0.007	0.008	t	10277	DSYGRDPYSPSQDPY	Homo sapiens
1	1.00	1	1	0.02	0.02	0.02	0.023	0.02	t	49023	PQLGPR	Homo sapiens
1	1.00	1	1	0.02	0.02	0.02	0.02	0.021	t	115519	TPEVDDEALE	Homo sapiens
1	1.00	1	1	0.02	0.02	0.017	0.024	0.02	t	78845	TATKQAEAAAPVVESKWRA	Homo sapiens
1	1.00	1	1	-0.04	-0.04	-0.043	-0.04	-0.041	t	136074	LVKK	Mus musculus
1	1.00	1	1	0.01	0.006	0.002	0.009	0.01	t	15179	FAGKDLESIKGTAPFEIHAN	Oryctolagus cuniculus
1	1.00	1	1	0	0.002	0.005	0.007	-0.003	t	126786	NRYTGPYT	Homo sapiens

1	1.00	1	1	0.03	0.026	0.03	0.027	0.029	t	64640	TKPTDGN	Oryctolagus cuniculus
1	1.00	1	1	0.02	0.018	0.015	0.019	0.02	t	5350	AVGPGRWDED	Mus musculus
1	1.00	1	1	0.03	0.03	0.018	0.03	0.03	t	121732	QAPEYRGRTELLKESIGE	Rattus norvegicus
1	1.00	1	1	-0.01	-0.013	-0.017	-0.01	-0.012	t	7313	CVTIMTVDNPASTTNKDK	Oryctolagus cuniculus
1	1.00	1	1	0.02	0.016	0.024	0.023	0.02	t	97324	GDSQATYNY	Oryctolagus cuniculus
1	1.00	1	1	0.02	0.02	0.02	0.02	0.02	t	33857	KTSERSQPRG	Homo sapiens
1	1.00	1	1	0.01	0.01	0.01	0.01	0.009	t	51483	QLPQGTTLPKGFYAE	Homo sapiens
1	1.00	1	1	0.02	0.018	0.02	0.017	0.017	t	134028	DDTIS	Mus musculus
1	1.00	1	1	0.02	0.02	0.018	0.02	0.022	t	5344	AVGGYAISIS	Homo sapiens
1	1.00	1	1	0.03	0.032	0.025	0.031	0.029	t	22223	GRTWAQPGYPWP	Homo sapiens
1	1.00	1	1	0.01	0.012	-0.001	0.01	0.01	t	21807	GPTEESVERAMGRVA	Rattus norvegicus
1	1.00	1	1	0.02	0.021	0.017	0.019	0.021	t	14844	EVPLGVAADANK	Oryctolagus cuniculus
1	1.00	1	1	0.02	0.022	0.013	0.021	0.02	t	51572	QLVLGENLDTERILAS	Homo sapiens
1	1.00	1	1	0.02	0.022	0.013	0.021	0.02	t	51572	QLVLGENLDTERILAS	Homo sapiens
1	1.00	1	1	0	0	-0.007	0	0	t	121812	RRATQLAH	Mus musculus

1	1.00	1	1	0	0	0	0	-0.002	t	67160	TVSPISSIFSR	Mus musculus
1	1.00	1	1	0.03	0.03	0.03	0.03	0.03	t	9585	DPAF	Mus musculus
1	1.00	1	1	-0.03	-0.033	-0.03	-0.03	-0.03	t	135488	PIFPGG	Oryctolagus cuniculus
1	1.00	1	1	-0.04	-0.04	-0.04	-0.04	-0.039	t	115197	CVKKILDKVG	Homo sapiens
1	1.00	1	1	-0.01	-0.01	-0.01	-0.01	-0.01	t	156859	DPHVFKS	Gallus gallus
1	1.00	1	1	-0.04	-0.038	-0.049	-0.043	-0.043	t	105700	CPSTYEEPAQQGRRH	Homo sapiens
1	1.00	1	1	-0.01	-0.01	-0.013	-0.007	-0.01	t	78643	EQGMQLAEQFKQKALGL	Homo sapiens
1	1.00	1	1	-0.01	-0.007	-0.008	-0.01	-0.01	t	146020	TCPPDIIPK	Mus musculus
1	1.00	1	1	-0.04	-0.037	-0.039	-0.037	-0.04	t	78218	NKQWFT	Equus caballus
1	1.00	1	1	-0.01	-0.01	-0.01	-0.01	-0.01	t	95824	SDVAGL	Mus musculus
1	1.00	1	1	0.01	0.006	0.001	0.009	0.01	t	19901	GGKMVQVVYD	Oryctolagus cuniculus
1	1.00	1	1	-0.02	-0.018	-0.016	-0.019	-0.02	t	6309	CGLFGK	Homo sapiens
1	1.00	1	1	0.06	0.06	0.06	0.062	0.062	t	27557	IMSGEVPSTEDLVN	Homo sapiens
1	1.00	1	1	-0.02	-0.022	-0.021	-0.017	-0.02	t	67052	TVKQHTVTT	Mus musculus
1	1.00	1	1	0.01	0.02	0.01	0.009	0.01	t	42694	MSTNPKPQRKTK	Pan troglodytes

1	1.00	1	1	0.04	0.04	0.04	0.044	0.037	t	131929	KGGGVWESGAANSQS	Homo sapiens
1	1.00	1	1	0.05	0.048	0.045	0.043	0.048	t	132269	EPVDGVVLVDPE	Mus musculus
1	1.00	1	1	0	-0.003	0.008	0	-0.001	t	125211	QHWSYGLRPG	Canis lupus
1	1.00	1	1	0.05	0.046	0.041	0.05	0.051	t	20046	GGTEESVVTASRS	Bos taurus
1	1.00	1	1	0	0.002	0.002	0.003	0	t	45584	NQLSCDLIGQKLGLK	Homo sapiens
1	1.00	1	1	0	0.002	0.002	0.003	0	t	45584	NQLSCDLIGQKLGLK	Homo sapiens
1	1.00	1	1	0	0.002	0.002	0.003	0	t	45584	NQLSCDLIGQKLGLK	Homo sapiens
1	1.00	1	1	-0.02	-0.017	-0.025	-0.02	-0.02	t	14447	ETKRKDGD	Equus caballus
1	1.00	1	1	-0.02	-0.02	-0.025	-0.02	-0.02	t	14447	ETKRKDGD	Mus musculus
1	1.00	1	1	0.01	0.01	0.001	0.01	0.01	t	79280	LAPISGTVQSAHLIIRFD	Mus musculus
1	1.00	1	1	0	0	0	0	-0.002	t	106256	ANYGYTFGSGTRLTVV	Oryctolagus cuniculus
1	1.00	1	1	0	0	-0.009	0	0	t	136011	VPALTAVETGHT	Homo sapiens
1	1.00	1	1	-0.01	-0.014	-0.021	-0.011	-0.01	t	99018	SYSITTPSQFV	Oryctolagus cuniculus
1	1.00	1	1	-0.04	-0.032	-0.04	-0.04	-0.042	t	156943	QFTPDKHMLMLP	Gallus gallus

1	1.00	1	1	0.03	0.03	0.03	0.03	0.03	t	7841	DDPF	Mus musculus
1	1.00	1	1	0	0	0	0	-0.001	t	110085	VYTLPPS	Homo sapiens
1	1.00	1	1	0.01	0.012	0.003	0.011	0.009	t	3487	APARGEPAPG	Homo sapiens
1	1.00	1	1	0.02	0.02	0.02	0.02	0.02	t	21951	GQRVEF	Homo sapiens
1	1.00	1	1	0.01	0.01	0.01	0.01	0.008	t	78696	KEEGEDDTARQEIRKAWVK	Oryctolagus cuniculus
1	1.00	1	1	-0.03	-0.026	-0.032	-0.033	-0.03	t	55822	RSEFPIWMMF	Homo sapiens
1	1.00	1	1	0.01	0.01	0.01	0.014	0.01	t	135982	RDRSGLAPKRPG	Homo sapiens
1	1.00	1	1	0.01	0.01	0.01	0.01	0.01	t	62328	SVSLPYANLATH	Mus musculus
1	1.00	1	1	0.03	0.035	0.026	0.03	0.03	t	49883	PVIGSQAR	Homo sapiens
1	1.00	1	1	0.03	0.035	0.026	0.03	0.03	t	49883	PVIGSQAR	Homo sapiens
1	1.00	1	1	0.02	0.02	0.013	0.02	0.02	t	105076	AFNHFGEGLMQR	Homo sapiens
1	1.00	1	1	-0.02	-0.018	-0.031	-0.019	-0.02	t	65881	TQIQVRNIMSPVMG	Homo sapiens
1	1.00	1	1	0.01	0.01	0.007	0.01	0.01	t	99052	VTQTMKGLDI	Homo sapiens
1	1.00	1	1	-0.01	-0.01	-0.01	-0.01	-0.01	t	78503	RFPYWFVLPPIATVD	Mus musculus
1	1.00	1	1	0.01	0.013	0	0.01	0.009	t	111304	GAVGENVPDDLYIKG	Mus musculus

1	1.00	1	1	-0.04	-0.038	-0.035	-0.033	-0.041	t	112405	YCARMRD	Homo sapiens
1	1.00	1	1	0.03	0.032	0.024	0.026	0.032	t	24445	HPEYAVSVLL	Homo sapiens
1	1.00	1	1	-0.04	-0.036	-0.036	-0.033	-0.04	t	144644	FRQQHGKTVV	Homo sapiens
1	1.00	1	1	-0.01	-0.013	-0.02	-0.01	-0.01	t	140939	YNIALGQRRADA	Oryctolagus cuniculus
1	1.00	1	1	-0.01	-0.01	-0.005	-0.01	-0.012	t	95752	RAGFITELSEFI	Homo sapiens
1	1.00	1	1	-0.03	-0.028	-0.03	-0.023	-0.03	t	107031	YTQKHKHQA	Homo sapiens
1	1.00	1	1	0.01	0.01	0.006	0.016	0.01	t	55670	RRPFFHPVGEADYFEYHQEG	Homo sapiens
1	1.00	1	1	0	0	0	0	-0.003	t	140567	KEAWLDSTKAT	Mus musculus
1	1.00	1	1	0	0	0	0	-0.003	t	138304	ATVQLPQ	Oryctolagus cuniculus
1	1.00	1	1	0	0	0	0	-0.003	t	138608	RVGAHDP	Oryctolagus cuniculus
1	1.00	1	1	0	0	0	0	-0.003	t	132843	ITPWPHWLERSS	Mus musculus
1	1.00	1	1	0	0	0	0	-0.003	t	133372	SPSPTPTPGPSRRG	Homo sapiens
1	1.00	1	1	0	0	0	0	-0.003	t	110390	QWGRL	Mus musculus
1	1.00	1	1	0	0	0	0	-0.003	t	110332	LYRWR	Mus musculus
1	1.00	1	1	0	0	0	0	-0.003	t	106374	EDHAGTYGLGDRK	Mus musculus

1	1.00	1	1	0	0	0	0	-0.003	t	110213	EGHLIDLKRV	Mus musculus
1	1.00	1	1	0	0	0	0	-0.003	t	64541	TKDTNNNL	Mus musculus
1	1.00	1	1	0	0	0	0	-0.003	t	68322	VEQKHIQKEDVPSERYLGYL	Homo sapiens
1	1.00	1	1	0	0	0	0	-0.003	t	64541	TKDTNNNL	Mus musculus
1	1.00	1	1	0	0	0	0	-0.003	t	64541	TKDTNNNL	Mus musculus
1	1.00	1	1	0	0	0	0	-0.003	t	68473	VFGKEKVNELSKDIGSESTE	Homo sapiens
1	1.00	1	1	0	0	0	0	-0.003	t	64541	TKDTNNNL	Mus musculus
1	1.00	1	1	0	0	0	0	-0.003	t	68401	VEVPGSQHIDSQKKA	Mus musculus
1	1.00	1	1	0	0	0	0	-0.003	t	43242	NANPNANPNANP	Mus musculus
1	1.00	1	1	0	0	0	0	-0.003	t	43242	NANPNANPNANP	Mus musculus
1	1.00	1	1	0	0	0	0	-0.003	t	38207	LNENLLRFFVAPFPEVFGKE	Homo sapiens
1	1.00	1	1	0	0	0	0	-0.003	t	39183	LRRDLDASREAKKQVEKALE	Homo sapiens
1	1.00	1	1	0	0	0	0	-0.003	t	43242	NANPNANPNANP	Mus musculus
1	1.00	1	1	0	0	0	0	-0.003	t	39183	LRRDLDASREAKKQVEKALE	Homo sapiens

1	1.00	1	1	0	0	0	0	-0.003	t	39183	LRRDLDASREAKKQVEKALE	Homo sapiens
1	1.00	1	1	0	0	0	0	-0.003	t	24814	HSMKEGIHAQQKEPMIGVNQ	Homo sapiens
1	1.00	1	1	0	0	0	0	-0.003	t	23876	HFVQQTPKSQPTLV	Mus musculus
1	1.00	1	1	0	0	0	0	-0.003	t	11035	EAESISSSEEIVPNSVEQKH	Homo sapiens
1	1.00	1	1	0	-0.002	0	0	-0.003	t	28911	ITGHRMAWDMMMNWS	Mus musculus
1	1.00	1	1	0	0	0	0	-0.003	t	7491	DAEFRHDSGYEVHHQK	Mus musculus
1	1.00	1	1	0	0	0	0	-0.003	t	7491	DAEFRHDSGYEVHHQK	Mus musculus
1	1.00	1	1	0	0	0	0	-0.003	t	7491	DAEFRHDSGYEVHHQK	Mus musculus
1	1.00	1	1	0	0	0	0	-0.003	t	655	ADEEQQQALSSQMGF	Homo sapiens
1	1.00	1	1	0	0	0	0	-0.003	t	8756	DIPNPIGSENSEKTTMPLW	Homo sapiens
1	1.00	1	1	0	0	0	0	-0.002	t	66060	TRLNPD	Mus musculus
1	1.00	1	1	0.01	0.01	0.003	0.01	0.01	t	159050	PKRLTATTEKLEIGWK	Mus musculus
1	1.00	1	1	0	-0.003	0	0	-0.001	t	15205	FAKY	Mus musculus
1	1.00	1	1	-0.02	-0.02	-0.02	-0.011	-0.018	t	96808	QVKSGTIFDN	Homo sapiens
1	1.00	1	1	0.02	0.016	0.015	0.019	0.02	t	78876	VLLGRKWEDEVDDPESYF	Oryctolagus cuniculus

1	1.00	1	1	0.02	0.018	0.015	0.019	0.02	t	78876	VLLGRKWEDEVDDPESYF	Mus musculus
1	1.00	1	1	0.01	0.01	0.01	0.01	0.01	t	140916	VSYGEEKPAVLG	Oryctolagus cuniculus
1	1.00	1	1	0.01	0.01	0.009	0.009	0.01	t	9673	DPLLRTAP	Homo sapiens
1	1.00	1	1	0.04	0.04	0.038	0.04	0.04	t	116709	EVHHQKLVFFAEDVGSNK	Mus musculus
1	1.00	1	1	-0.03	-0.022	-0.03	-0.03	-0.032	t	156936	PMPRFF	Gallus gallus
1	1.00	1	1	0	0	0	0	-0.002	t	22360	GSGVRGDFGSLAPRVARQL	Cavia porcellus
1	1.00	1	1	0.02	0.02	0.017	0.02	0.02	t	157219	EQRCCNELNR	Homo sapiens
1	1.00	1	1	0.01	0.01	0.01	0.01	0.008	t	138892	REQAPNLVY	Mus musculus
1	1.00	1	1	0.02	0.02	0.02	0.02	0.02	t	149434	QPQPFLPQQP	Homo sapiens
1	1.00	1	1	0.02	0.02	0.02	0.02	0.02	t	149434	QPQPFLPQQP	Mus musculus
1	1.00	1	1	-0.01	-0.01	-0.02	-0.01	-0.008	t	104161	NVVLKTITESATSPPT	Oryctolagus cuniculus
1	1.00	1	1	0	0	0	0	0	t	112090	EDLAVYF	Homo sapiens
1	1.00	1	1	0.01	0.01	0.003	0.01	0.01	t	130585	QKKIRGILE	Mus musculus
1	1.00	1	1	0.01	0.008	0.007	0.009	0.008	t	28290	IRFKDDSSYEEVN	Mus musculus
1	1.00	1	1	0	0	0	0	0	t	133539	CYKPGEWAC	Mus musculus

1	1.00	1	1	0	-0.002	0	-0.001	-0.002	t	56686	RYYRE	Mus musculus
1	1.00	1	1	0.01	0.01	0.007	0.007	0.009	t	63164	TDGVTYTNDCL	Homo sapiens
1	1.00	1	1	-0.01	-0.008	-0.01	-0.01	-0.008	t	107368	FSSAP	Rattus norvegicus
1	1.00	1	1	-0.02	-0.02	-0.02	-0.02	-0.023	t	130605	SCRLFGKTYLCH	Mus musculus
1	1.00	1	1	-0.05	-0.048	-0.051	-0.049	-0.051	t	120408	APYNVRRLPPPVVEP	Homo sapiens
1	1.00	1	1	0	0	0	0	-0.002	t	134411	IPP	Mus musculus
1	1.00	1	1	-0.02	-0.02	-0.017	-0.024	-0.026	t	74129	YHPDVPYVKR	Homo sapiens
1	1.00	1	1	0	0	-0.006	0	0	t	2621	ALIDQKVKEI	Homo sapiens
1	1.00	1	1	0.02	0.02	0.022	0.02	0.02	t	72911	WNSTTFHQTLQDP	Mus musculus
1	1.00	1	1	0.02	0.02	0.022	0.02	0.02	t	72911	WNSTTFHQTLQDP	Mus musculus
1	1.00	1	1	0.02	0.017	0.023	0.02	0.02	t	127964	EQLIEKLIEDDE	Oryctolagus cuniculus
1	1.00	1	1	-0.01	-0.008	-0.01	-0.003	-0.008	t	107479	KFNWYVD	Homo sapiens
1	1.00	1	1	-0.01	-0.008	-0.014	-0.009	-0.011	t	110869	GQAEPDRAHYNIVTFCCKCD	Homo sapiens
1	1.00	1	1	-0.05	-0.048	-0.049	-0.043	-0.049	t	121527	LCNEKLLKKARIHPFH	Homo sapiens
1	1.00	1	1	0	0.002	-0.007	-0.003	-0.003	t	106568	KLPPIDVNMDCK	Homo sapiens

1	1.00	1	1	0.01	0.012	0.016	0.011	0.009	t	8857	DKEILYEAF	Homo sapiens
1	1.00	1	1	0.02	0.02	0.02	0.02	0.02	t	9609	DPDI	Mus musculus
1	1.00	1	1	0	0.002	0	0.003	0	t	40076	LTPCD	Homo sapiens
1	1.00	1	1	0.02	0.02	0.016	0.02	0.02	t	2237	AKLQDC	Homo sapiens
1	1.00	1	1	-0.01	-0.01	-0.01	-0.01	-0.011	t	122150	MSKTLHAQLGFKKT	Oryctolagus cuniculus
1	1.00	1	1	0.05	0.05	0.053	0.05	0.049	t	78938	CAPGQNLCY	Mus musculus
1	1.00	1	1	0	0	0	0	-0.002	t	135587	QESYSSKKL	Oryctolagus cuniculus
1	1.00	1	1	0	-0.002	-0.004	-0.003	0	t	55760	RRVGLSNP	Anas platyrhynchos
1	1.00	1	1	0	0	0	0	-0.003	t	138456	KEQFEFALTAVAEEV	Oryctolagus cuniculus
1	1.00	1	1	0	0	0	0	-0.003	t	144623	EEAEGTTDAQIDANRKRESE	Mus musculus
1	1.00	1	1	0	0	0	0	-0.003	t	137211	KARIHPFHVLIALETYRAGH	Mus musculus
1	1.00	1	1	0	0	0	0	-0.003	t	157811	QRQCQQRCER	Homo sapiens
1	1.00	1	1	0	0	0	0	-0.003	t	137734	DTALTKAALKLV	Mus musculus
1	1.00	1	1	0	0	0	0	-0.003	t	140546	CNLWEVMHGTC	Mus musculus
1	1.00	1	1	0	0	0	0	-0.003	t	137290	SEIEKLKNKYEVAGIP	Mus musculus
1	1.00	1	1	0	0	0	0	-0.003	t	141267	FEGWFN	Capra hircus

1	1.00	1	1	0	0	0	0	-0.003	t	134492	QIQRRPSM	Mus musculus
1	1.00	1	1	0	0	0	0	-0.003	t	145376	NCWKPQVLHC	Homo sapiens
1	1.00	1	1	0	0	0	0	-0.003	t	137743	DTPLTIAGLKLV	Mus musculus
1	1.00	1	1	0	0	0	0	-0.003	t	135569	KTNMK	Mus musculus
1	1.00	1	1	0	0	0	0	-0.003	t	150883	SDDEDLATS	Mus musculus
1	1.00	1	1	0	0	0	0	-0.003	t	140606	TMWDALEAP	Mus musculus
1	1.00	1	1	0	0	0	0	-0.003	t	145375	NCSWCHVLQRCS	Homo sapiens
1	1.00	1	1	0	0	0	0	-0.003	t	141296	GPQQLE	Capra hircus
1	1.00	1	1	0	0	0	0	-0.003	t	151023	LCKLRGVAPLHLGKC	Mus musculus
1	1.00	1	1	0	0	0	0	-0.003	t	137395	EESEDEKRRWGQRDN	Homo sapiens
1	1.00	1	1	0	0	0	0	-0.003	t	141534	DRMNLQTLRGYY	Mus musculus
1	1.00	1	1	0	-0.003	0	0	-0.003	t	142480	VAAVTEKLRAGT	Oryctolagus cuniculus
1	1.00	1	1	0	-0.003	0	0	-0.003	t	142441	LFSPWLRVQNHF	Oryctolagus cuniculus
1	1.00	1	1	0	0	0	0	-0.003	t	138862	KFPED	Mus musculus
1	1.00	1	1	0	0	0	0	-0.003	t	135492	RAEIM	Oryctolagus cuniculus

1	1.00	1	1	0	0	0	0	-0.003	t	131420	FDGYWGYS	Homo sapiens
1	1.00	1	1	0	0	0	0	-0.003	t	130063	TEVPFRL	Rattus norvegicus
1	1.00	1	1	0	0	0	0	-0.003	t	125230	RERRGIAL	Mus musculus
1	1.00	1	1	0	0	0	0	-0.003	t	133556	EGPEYWDRNT	Mus musculus
1	1.00	1	1	0	0	0	0	-0.003	t	133135	HGTQCLAMGWGRVGAH	Homo sapiens
1	1.00	1	1	0	0	0	0	-0.003	t	132014	PQIVPEPM	Homo sapiens
1	1.00	1	1	0	0	0	0	-0.003	t	131876	FAKANKQVCTDI	Mus musculus
1	1.00	1	1	0	0	0	0	-0.003	t	132737	WIRSTLRRVEAKGRP	Homo sapiens
1	1.00	1	1	0	0	0	0	-0.003	t	126068	FVFEEF	Rattus norvegicus
1	1.00	1	1	0	0	0	0	-0.003	t	117921	IGIIDLIEKRKFNQ	Mus musculus
1	1.00	1	1	0	0	0	0	-0.003	t	124008	DGVVLVD	Rattus norvegicus
1	1.00	1	1	0	0	0	0	-0.003	t	120436	DGTKVSIS	Rattus norvegicus
1	1.00	1	1	0	0	0	0	-0.003	t	131836	CAPGSCFPGV	Mus musculus
1	1.00	1	1	0	0	0	0	-0.003	t	131381	CTYVLDPENL	Mus musculus
1	1.00	1	1	0	0	0	0	-0.003	t	131967	LRLDCRHEN	Mus musculus
1	1.00	1	1	0	0	0	0	-0.003	t	132194	CATRLFPDFFTRVAL	Homo sapiens

1	1.00	1	1	0	-0.003	0	0	-0.003	t	118105	RKGHFLLS SKSGFWTI WL	Oryctolagus cuniculus
1	1.00	1	1	0	0	0	0	-0.003	t	124008	DGVVLVD	Mus musculus
1	1.00	1	1	0	0	0	0	-0.003	t	129661	PVDGVVL VD	Mus musculus
1	1.00	1	1	0	0	0	0	-0.003	t	121187	DDALETDE	Mus musculus
1	1.00	1	1	0	0	0	0	-0.003	t	130019	STIIKEGI	Rattus norvegicus
1	1.00	1	1	0	0	0	0	-0.003	t	133926	RSSYWVYS PWRFISR	Mus musculus
1	1.00	1	1	0	0	0	0	-0.003	t	118763	CYGSFARP MN	Mus musculus
1	1.00	1	1	0	0	0	0	-0.003	t	117940	KARIHPFHI LIALETYKT GH	Mus musculus
1	1.00	1	1	0	0	0	0	-0.003	t	122269	ARQNLKD AGE	Mus musculus
1	1.00	1	1	0	0	0	0	-0.003	t	125453	KDRLQGM APAAGAD	Homo sapiens
1	1.00	1	1	0	0	0	0	-0.003	t	119331	RTVLVSM NYR	Oryctolagus cuniculus
1	1.00	1	1	0	0	0	0	-0.003	t	117399	VMIKSLPA LNSPTVEE	Oryctolagus cuniculus
1	1.00	1	1	0	0	0	0	-0.003	t	119025	SWTDTDG TN	Mus musculus
1	1.00	1	1	0	0	0	0	-0.003	t	122892	RSILPYGDS MDRIE	Homo sapiens
1	1.00	1	1	0	0	0	0	-0.003	t	129301	LQELEKDE REQ	Homo sapiens

1	1.00	1	1	0	0	0	0	-0.003	t	122430	KGLRRDLDASREAK	Mus musculus
1	1.00	1	1	0	0	0	0	-0.003	t	119658	TYGIVLLSGV	Homo sapiens
1	1.00	1	1	0	0	0	0	-0.003	t	125127	LDPENLTL	Mus musculus
1	1.00	1	1	0	0	0	0	-0.003	t	117495	ERDRKREKRKPKS	Mus musculus
1	1.00	1	1	0	0	0	0	-0.003	t	118867	KLSQTSNVDKEEEAV	Mus musculus
1	1.00	1	1	0	0	0	0	-0.003	t	120869	PGYRSISGPDPRIRKT	Mus musculus
1	1.00	1	1	0	0	0	0	-0.003	t	122269	ARQNLKDAGE	Mus musculus
1	1.00	1	1	0	0	0	0	-0.003	t	129973	SPRSFQKKT	Oryctolagus cuniculus
1	1.00	1	1	0	0	0	0	-0.003	t	117949	KGHFLLSSKSGFWT	Mus musculus
1	1.00	1	1	0	0	0	0	-0.003	t	122741	DWRDAEKCDIC	Mus musculus
1	1.00	1	1	0	0	0	0	-0.003	t	120319	WTPPAIFKSYCEIIVTHFPF	Mus musculus
1	1.00	1	1	0	0	0	0	-0.003	t	119499	MTFSVPIS	Mus musculus
1	1.00	1	1	0	0	0	0	-0.003	t	132982	AHCLRDIPQRLVNV	Homo sapiens
1	1.00	1	1	0	0	0	0	-0.003	t	130916	KEDFDIYDEDE	Mus musculus
1	1.00	1	1	0	0	0	0	-0.003	t	133551	EAPPPTLPPDMAQVTVGPG	Mus musculus
1	1.00	1	1	0	0	0	0	-0.003	t	129732	RERRGIALDGKI	Mus musculus

1	1.00	1	1	0	0	0	0	-0.003	t	131874	EVFISAPR	Homo sapiens
1	1.00	1	1	0	0	0	0	-0.003	t	119573	EWEDTYGIVL	Homo sapiens
1	1.00	1	1	0	0	0	0	-0.003	t	122932	VCTPKSDEFNPCEDIMGYK	Oryctolagus cuniculus
1	1.00	1	1	0	0	0	0	-0.003	t	133668	QEGPEYWDRNT	Mus musculus
1	1.00	1	1	0	0	0	0	-0.003	t	130518	ITQSTQSFNDFTRVV	Mus musculus
1	1.00	1	1	0	0	0	0	-0.003	t	120082	LPTDSGEK	Rattus norvegicus
1	1.00	1	1	0	0	0	0	-0.003	t	122569	SFQDENFVFEEF	Rattus norvegicus
1	1.00	1	1	0	0	0	0	-0.003	t	118829	GVLAGQASQLNA	Mus musculus
1	1.00	1	1	0	0	0	0	-0.003	t	130591	RCTVYGKTVLCT	Mus musculus
1	1.00	1	1	0	0	0	0	-0.003	t	125385	DSMDRIEKDRLQGMAPAAG	Homo sapiens
1	1.00	1	1	0	0	0	0	-0.003	t	131217	EDISAYLL	Mus musculus
1	1.00	1	1	0	0	0	0	-0.003	t	121064	WKHWVYYTCCPDTPYLDITY	Mus musculus
1	1.00	1	1	0	0	0	0	-0.003	t	120677	KHPEVKSAIEGIKYI	Rattus norvegicus
1	1.00	1	1	0	0	0	0	-0.003	t	130741	ATGFVKKDQ	Mus musculus
1	1.00	1	1	0	0	0	0	-0.003	t	117383	TLHKAFKGSIFVVFDSIESA	Homo sapiens
1	1.00	1	1	0	0	0	0	-0.003	t	98822	IEAGAIGFKIHEDWG	Mus musculus

1	1.00	1	1	0	0	0	0	-0.003	t	104850	KEGILQYCQEVYPELQ	Mus musculus
1	1.00	1	1	0	0	0	0	-0.003	t	99922	DAEFRHDSGY	Oryctolagus cuniculus
1	1.00	1	1	0	0	0	0	-0.003	t	106196	YGLPREQARQLKNNN	Homo sapiens
1	1.00	1	1	0	0	0	0	-0.003	t	105702	CVYDQYGTEFEDYSYES	Mus musculus
1	1.00	1	1	0	0	0	0	-0.003	t	106057	REFSPRGQHGRRERA	Homo sapiens
1	1.00	1	1	0	0	0	0	-0.003	t	103998	IVKWDRDM	Mus musculus
1	1.00	1	1	0	-0.003	0	0	-0.003	t	103309	KKNGRILTLPRSNPS	Oryctolagus cuniculus
1	1.00	1	1	0	0	0	0	-0.003	t	115271	GQKGRGSRG	Oryctolagus cuniculus
1	1.00	1	1	0	0	0	0	-0.003	t	102423	FEYQD	Mus musculus
1	1.00	1	1	0	0	0	0	-0.003	t	103993	IPPSLRTLEDNEERMS	Homo sapiens
1	1.00	1	1	0	0	0	0	-0.003	t	106076	RILSPDRKRRQQYER	Homo sapiens
1	1.00	1	1	0	0	0	0	-0.003	t	117195	DT	Mus musculus
1	1.00	1	1	0	0	0	0	-0.003	t	99922	DAEFRHDSGY	Oryctolagus cuniculus
1	1.00	1	1	0	0	0	0	-0.003	t	103551	RILTLPRSNPS	Oryctolagus cuniculus
1	1.00	1	1	0	0	0	0	-0.003	t	106495	GYEVHHQKLVF	Gallus gallus

1	1.00	1	1	0	0	0	0	-0.003	t	106122	SRRRSLPYSPYSPQT	Homo sapiens
1	1.00	1	1	0	0	0	0	-0.003	t	108078	PTSPSGVEEWIVTQVVPGVA	Oryctolagus cuniculus
1	1.00	1	1	0	0	0	0	-0.003	t	103830	CHAVKVGNPSC	Oryctolagus cuniculus
1	1.00	1	1	0	0	0	0	-0.003	t	103309	KKNGRILTLPRSNPS	Oryctolagus cuniculus
1	1.00	1	1	0	0	0	0	-0.003	t	106779	RKPLSPGMTGR	Oryctolagus cuniculus
1	1.00	1	1	0	0.002	0	0	-0.003	t	116971	FAVLKLMGRGTKF	Rattus norvegicus
1	1.00	1	1	0	0	0	0	-0.003	t	106398	EQLAEVAVSAKR	Oryctolagus cuniculus
1	1.00	1	1	0	0	0	0	-0.003	t	106621	LRKRL	Gallus gallus
1	1.00	1	1	0	0	0	0	-0.003	t	105570	VGGVVIA	Mus musculus
1	1.00	1	1	0	0	0	0	-0.003	t	108622	SHQESTRGRSRGRS	Homo sapiens
1	1.00	1	1	0	0	0	0	-0.003	t	107482	KGEPGLPGRGFPGFP	Mus musculus
1	1.00	1	1	0	0	0	0	-0.003	t	107013	YGASSRATGIPDR	Oryctolagus cuniculus
1	1.00	1	1	0	0	0	0	-0.003	t	100992	KSLSRHDHIHHH	Ovis aries
1	1.00	1	1	0	0	0	0	-0.003	t	105685	ASFKKNIGRNRSPDI	Homo sapiens

1	1.00	1	1	0	0	0	0	-0.003	t	101412	WIRNNF	Mus musculus
1	1.00	1	1	0	0	0	0	-0.003	t	102861	TQSDIDFLIEEIERLGQDL	Oryctolagus cuniculus
1	1.00	1	1	0	0	0	0	-0.003	t	70656	VQTRHVVQHRSRSESSIESF	Oryctolagus cuniculus
1	1.00	1	1	0	0	0	0	-0.003	t	64455	TISANGDKDIG	Rattus norvegicus
1	1.00	1	1	0	0	0	0	-0.003	t	74436	YKDGTLTPLSTKDLTTY	Rattus norvegicus
1	1.00	1	1	0	0	0	0	-0.003	t	67967	VDGSDP	Mus musculus
1	1.00	1	1	0	0	0	0	-0.003	t	79858	IEELQSNHGV	Mus musculus
1	1.00	1	1	0	0	0	0	-0.003	t	79042	PKSKPFA	Mus musculus
1	1.00	1	1	0	0	0	0	-0.003	t	78373	EMDDDETEYE	Mus musculus
1	1.00	1	1	0	0	0	0	-0.003	t	96367	HSNLDHI	Homo sapiens
1	1.00	1	1	0	0	0	0	-0.003	t	64086	THTPLPRGIGY	Mus musculus
1	1.00	1	1	0	0	0	0	-0.003	t	78077	YPYSMWKNLWPS	Mus musculus
1	1.00	1	1	0	0	0	0	-0.003	t	61549	STDEPAGESA	Oryctolagus cuniculus

1	1.00	1	1	0	0	0	0	-0.003	t	95524	KRQIYTDIEMNRLGK	Mus musculus
1	1.00	1	1	0	0	0	0	-0.003	t	60526	SQPNTEQSPAIVLPF	Mus musculus
1	1.00	1	1	0	0	0	0	-0.003	t	66976	TVDNPASTTNKDKLFAVWK	Oryctolagus cuniculus
1	1.00	1	1	0	0	0	0	-0.003	t	61549	STDEPAGESA	Oryctolagus cuniculus
1	1.00	1	1	0	0.002	0	0	-0.003	t	66976	TVDNPASTTNKDKLFAVWK	Rattus norvegicus
1	1.00	1	1	0	0	0	0	-0.003	t	92586	ENPSQFYERL	Oryctolagus cuniculus
1	1.00	1	1	0	0	0	0	-0.003	t	70387	VPNLRGDLQV	Cavia porcellus
1	1.00	1	1	0	0	0	0	-0.003	t	65996	TRAPRREELEWDDEDH	Oryctolagus cuniculus
1	1.00	1	1	0	0	0	0	-0.003	t	80102	YDDAASESSDDDDIDALI	Mus musculus
1	1.00	1	1	0	0	0	0	-0.003	t	63037	TAVSPTTLR	Oryctolagus cuniculus
1	1.00	1	1	0	0	0	0	-0.003	t	76290	YVHDNC	Mus musculus
1	1.00	1	1	0	0	0	0	-0.003	t	97348	GLFGAIAGFIEGGW	Oryctolagus cuniculus

1	1.00	1	1	0	0	0	0	-0.003	t	71798	VVQAMYVPPGAPNPKE	Oryctolagus cuniculus
1	1.00	1	1	0	0	0	0	-0.003	t	63758	TFTNPVVSTTQWSAP	Oryctolagus cuniculus
1	1.00	1	1	0	0	0	0	-0.003	t	96457	KGTVNLTWSR	Mus musculus
1	1.00	1	1	0	0	0	0	-0.003	t	70387	VPNLRGDLQV	Cavia porcellus
1	1.00	1	1	0	0	0	0	-0.003	t	80063	VDDEDSD	Mus musculus
1	1.00	1	1	0	-0.003	0	0	-0.003	t	78940	CRRHNYGFWVC	Oryctolagus cuniculus
1	1.00	1	1	0	0	0	0	-0.003	t	65050	TLRVYK	Mus musculus
1	1.00	1	1	0	0	0	0	-0.003	t	63037	TAVSPTTLR	Mus musculus
1	1.00	1	1	0	0	0	0	-0.003	t	94979	YNKRFPGFVSP	Oryctolagus cuniculus
1	1.00	1	1	0	0.002	0	0	-0.003	t	74436	YKDGTLTPLSTKDLTTY	Rattus norvegicus
1	1.00	1	1	0	0	0	0	-0.003	t	97256	EGAKGENKRPNDKAGEV	Mus musculus
1	1.00	1	1	0	0	0	0	-0.003	t	76523	YYIPLEAVKF	Homo sapiens
1	1.00	1	1	0	0	0	0	-0.003	t	79926	MASMTGGQQMG	Oryctolagus cuniculus

1	1.00	1	1	0	0	0	0	-0.003	t	63037	TAVSPTTLR	Mus musculus
1	1.00	1	1	0	0	0	0	-0.003	t	79584	KGDKAQIEKRIQEIIEQLDV	Mus musculus
1	1.00	1	1	0	0	0	0	-0.003	t	74614	YLEAFY	Mus musculus
1	1.00	1	1	0	0	0	0	-0.003	t	94501	EKQISDASRQGLRRD	Mus musculus
1	1.00	1	1	0	0	0	0	-0.003	t	70656	VQTRHVVQHRSRSESSIESF	Rattus norvegicus
1	1.00	1	1	0	-0.003	0	0	-0.003	t	78939	CINHRGYWVC	Oryctolagus cuniculus
1	1.00	1	1	0	0	0	0	-0.003	t	65360	TNLEDPASRDLVVSY	Mus musculus
1	1.00	1	1	0	0	0	0	-0.003	t	64454	TISADSKKTKDLVFL	Mus musculus
1	1.00	1	1	0	0	0	0	-0.003	t	63391	TEHRDYHPQQRG	Mus musculus
1	1.00	1	1	0	0	0	0	-0.003	t	94564	KIAKEQ	Mus musculus
1	1.00	1	1	0	-0.008	0	0	-0.003	t	97231	CNTKCQTP	Mus musculus
1	1.00	1	1	0	0	0	0	-0.003	t	72249	WAP	Mus musculus
1	1.00	1	1	0	0	0	0	-0.003	t	74436	YKDGTLTPLSTKDLTTY	Oryctolagus cuniculus
1	1.00	1	1	0	0	0	0	-0.003	t	61686	STKAAKKPEAKREAI	Mus musculus
1	1.00	1	1	0	0	0	0	-0.003	t	79153	ALQREAAERSEDEIK	Oryctolagus cuniculus

1	1.00	1	1	0	0	0	0	-0.003	t	37172	LLDPSFDY FQLYTTS	Mus musculus
1	1.00	1	1	0	0	0	0	-0.003	t	44474	NKDKLFAV WK	Rattus norvegicus
1	1.00	1	1	0	0	0	0	-0.003	t	39164	LRPGPVSQ PYHHWDT DK	Mus musculus
1	1.00	1	1	0	0	0	0	-0.003	t	48595	PNAN	Mus musculus
1	1.00	1	1	0	0	0	0	-0.003	t	48933	PPTAPPLL PHSNL	Homo sapiens
1	1.00	1	1	0	0	0	0	-0.003	t	48996	PQFKPQ	Mus musculus
1	1.00	1	1	0	0	0	0	-0.003	t	39169	LRPTFDTR LMRLEDE MKEGR	Mus musculus
1	1.00	1	1	0	0	0	0	-0.003	t	47859	PHWA	Mus musculus
1	1.00	1	1	0	0	0	0	-0.003	t	38214	LNFGQTG DADSV	Mus musculus
1	1.00	1	1	0	0	0	0	-0.003	t	45033	NMTVM	Homo sapiens
1	1.00	1	1	0	0	0	0	-0.003	t	44498	NKGVNPD E	Mus musculus
1	1.00	1	1	0	0.002	0	0	-0.003	t	44474	NKDKLFAV WK	Rattus norvegicus
1	1.00	1	1	0	0	0	0	-0.003	t	50609	QEGVQQE GA	Mus musculus
1	1.00	1	1	0	0	0	0	-0.003	t	50901	QGLRGEE MEEMV	Homo sapiens

1	1.00	1	1	0	0	0	0	-0.003	t	56373	RVNQVAE VLQL	Mus musculus
1	1.00	1	1	0	0	0	0	-0.003	t	44559	NKNLQEEI SDLTEQLG SS	Homo sapiens
1	1.00	1	1	0	0.008	0	0	-0.003	t	44221	NIETVIEFQ QKNNRLLE	Cavia porcellus
1	1.00	1	1	0	0	0	0	-0.003	t	39070	LREGVTLG VGASFDT QKL	Oryctolagus cuniculus
1	1.00	1	1	0	-0.003	0	0	-0.003	t	51252	QKSKQNIG ALKQEL	Oryctolagus cuniculus
1	1.00	1	1	0	0	0	0	-0.003	t	56807	SAHGTSTG VRGP	Mus musculus
1	1.00	1	1	0	0	0	0	-0.003	t	51853	QPHGGG W	Mus musculus
1	1.00	1	1	0	0	0	0	-0.003	t	51854	QPHGGSW	Mus musculus
1	1.00	1	1	0	0	0	0	-0.003	t	43242	NANPNAN PNANP	Oryctolagus cuniculus
1	1.00	1	1	0	0	0	0	-0.003	t	43512	NDPPPPN PNDPAPP QG	Mus musculus
1	1.00	1	1	0	0	0	0	-0.003	t	45627	NQSLDLAE QELVDCAS QHGC	Oryctolagus cuniculus
1	1.00	1	1	0	0	0	0	-0.003	t	38304	LNPKTNK WEDK	Homo sapiens
1	1.00	1	1	0	0	0	0	-0.003	t	58511	SIFYTYGTA PARISVPY VGI	Rattus norvegicus
1	1.00	1	1	0	0	0	0	-0.003	t	58511	SIFYTYGTA PARISVPY VGI	Oryctolagus cuniculus

1	1.00	1	1	0	0	0	0	-0.003	t	53355	RDGQLTIKAERTEQKDFDGR	Mus musculus
1	1.00	1	1	0	0	0	0	-0.003	t	43481	NDLALPWKHEGAQNWNNAER	Mus musculus
1	1.00	1	1	0	0	0	0	-0.003	t	55115	RPAPPKPTPQP	Mus musculus
1	1.00	1	1	0	0	0	0	-0.003	t	41169	MAVPAFSDIAKSANDLLNKD	Oryctolagus cuniculus
1	1.00	1	1	0	0	0	0	-0.003	t	57726	SFANTNTNTNSK	Mus musculus
1	1.00	1	1	0	0	0	0	-0.003	t	45932	NSNNPDWDF	Mus musculus
1	1.00	1	1	0	0	0	0	-0.003	t	60013	SPDDPAFRFAPYFTP	Mus musculus
1	1.00	1	1	0	0	0	0	-0.003	t	50180	PYVEPTAPQVL	Oryctolagus cuniculus
1	1.00	1	1	0	0	0	0	-0.003	t	44035	NGNATPQLTKNA	Homo sapiens
1	1.00	1	1	0	0	0	0	-0.003	t	42717	MSWQAYV	Mus musculus
1	1.00	1	1	0	0	0	0	-0.003	t	44474	NKDKLFAVWK	Oryctolagus cuniculus
1	1.00	1	1	0	0	0	0	-0.003	t	43551	NEAALKELGEVAVHKDS	Oryctolagus cuniculus
1	1.00	1	1	0	0	0	0	-0.003	t	55671	RRPFNIGINNQQ	Homo sapiens

1	1.00	1	1	0	0	0	0	-0.003	t	57618	SERVQLLHSQNTSLINQK	Homo sapiens
1	1.00	1	1	0	0	0	0	-0.003	t	46777	PAEGEK	Mus musculus
1	1.00	1	1	0	0	0	0	-0.003	t	42400	MQQLENDLDQVQESLLK	Homo sapiens
1	1.00	1	1	0	0	0	0	-0.003	t	50180	PYVEPTAPQVL	Oryctolagus cuniculus
1	1.00	1	1	0	0	0	0	-0.003	t	56102	RTLDQQYKEVKRETDLSHVQ	Mus musculus
1	1.00	1	1	0	0	0	0	-0.003	t	47650	PGFMFSRPSDPAFAA	Mus musculus
1	1.00	1	1	0	0	0	0	-0.003	t	47651	PGFMRGSRPSDPAFA	Mus musculus
1	1.00	1	1	0	0	0	0	-0.003	t	41870	MKNYLSFGMFALL	Mus musculus
1	1.00	1	1	0	0	0	0	-0.003	t	38522	LPHSNL	Homo sapiens
1	1.00	1	1	0	0	0	0	-0.003	t	48906	PPRRVS	Mus musculus
1	1.00	1	1	0	0	0	0	-0.003	t	10336	DTFNPVYPYDTE	Mus musculus
1	1.00	1	1	0	0	0	0	-0.003	t	12632	EKASVPGGGDMGGMDF	Mus musculus
1	1.00	1	1	0	0	0	0	-0.003	t	12632	EKASVPGGGDMGGMDF	Mus musculus
1	1.00	1	1	0	0	0	0	-0.003	t	24168	HKYSWK	Mus musculus

1	1.00	1	1	0	0	0	0	-0.003	t	17350	FPPRP	Mus musculus
1	1.00	1	1	0	0	0	0	-0.003	t	17360	FPQFKPEE	Mus musculus
1	1.00	1	1	0	0	0	0	-0.003	t	25647	IDPSFYWAPCRFSHC	Mus musculus
1	1.00	1	1	0	0	0	0	-0.003	t	35852	LFIG	Mus musculus
1	1.00	1	1	0	0	0	0	-0.003	t	28277	IRECTGLAWEWWRTV	Mus musculus
1	1.00	1	1	0	0	0	0	-0.003	t	11571	EEEDDDMGFGLFD	Homo sapiens
1	1.00	1	1	0	0	0	0	-0.003	t	11570	EEEDDD	Mus musculus
1	1.00	1	1	0	0	0	0	-0.003	t	16496	FLAEEADRK	Homo sapiens
1	1.00	1	1	0	0	0	0	-0.003	t	22357	GSGVRGDFG	Cavia porcellus
1	1.00	1	1	0	-0.003	0	0	-0.003	t	36045	LGAARGYTFGAC	Oryctolagus cuniculus
1	1.00	1	1	0	0	0	0	-0.003	t	36353	LGWLWG	Mus musculus
1	1.00	1	1	0	0	0	0	-0.003	t	26330	IGTRYLTR	Mus musculus
1	1.00	1	1	0	0	0	0	-0.003	t	36045	LGAARGYTFGAC	Mus musculus
1	1.00	1	1	0	0	0	0	-0.003	t	27965	IPNPLLGLD	Mus musculus
1	1.00	1	1	0	0	0	0	-0.003	t	11532	EECQLPSLKIFIAGNSAY	Mus musculus

1	1.00	1	1	0	0.002	0	0	-0.003	t	21816	GPVEESVE RAMVRVA	Rattus norvegic us
1	1.00	1	1	0	0	0	0	-0.003	t	10614	DVIAILPVD ELKALFQE KLE	Mus musculu s
1	1.00	1	1	0	0	0	0	-0.003	t	11081	EAIPALTA VETGHTSQ V	Oryctola gus cuniculu s
1	1.00	1	1	0	0	0	0	-0.003	t	20129	GHETQI	Mus musculu s
1	1.00	1	1	0	0	0	0	-0.003	t	16101	FHENWPS	Mus musculu s
1	1.00	1	1	0	0	0	0	-0.003	t	31105	KHFRETEV	Mus musculu s
1	1.00	1	1	0	0	0	0	-0.003	t	32808	KPNLSSKR SELSQLSM YRVF	Mus musculu s
1	1.00	1	1	0	0	0	0	-0.003	t	11216	EAVRRHA RAYNATVI	Mus musculu s
1	1.00	1	1	0	0	0	0	-0.003	t	11815	EENVEENV EENVEENV	Mus musculu s
1	1.00	1	1	0	0	0	0	-0.003	t	17262	FPCDRLSG YWERGIPS PCVR	Homo sapiens
1	1.00	1	1	0	0	0	0	-0.003	t	30302	KEALISSLT RGKLTYTQ Q	Homo sapiens
1	1.00	1	1	0	0	0	0	-0.003	t	35787	LFAAFPSF AGLRPTFD TRLM	Mus musculu s
1	1.00	1	1	0	0	0	0	-0.003	t	34502	KYLGFVQD AA	Homo sapiens
1	1.00	1	1	0	0	0	0	-0.003	t	33234	KRVLEPLG L	Mus musculu s

1	1.00	1	1	0	0	0	0	-0.003	t	12307	EGVQQEG AQQPA	Homo sapiens
1	1.00	1	1	0	0	0	0	-0.003	t	23125	GVRLLSGV FSDPAFA	Mus musculu s
1	1.00	1	1	0	0	0	0	-0.003	t	18036	FTPFYYQS SRTR	Mus musculu s
1	1.00	1	1	0	0	0	0	-0.003	t	19728	GGFSSDPG FLLRPRG	Mus musculu s
1	1.00	1	1	0	0	0	0	-0.003	t	19729	GGFSSDPG FLLRPRV	Mus musculu s
1	1.00	1	1	0	0	0	0	-0.003	t	21856	GQDLTAH	Mus musculu s
1	1.00	1	1	0	0	0	0	-0.003	t	33222	KRTISIWG T	Mus musculu s
1	1.00	1	1	0	0	0	0	-0.003	t	14809	EVLYLKPLA GVYRSLKK QLE	Mus musculu s
1	1.00	1	1	0	0	0	0	-0.003	t	19737	GGGAGAG GAGAGGG GR	Oryctola gus cuniculu s
1	1.00	1	1	0	0	0	0	-0.003	t	22357	GSGVRGD FG	Cavia porcellu s
1	1.00	1	1	0	0	0	0	-0.003	t	31916	KLILAYTPP GARGPQD	Oryctola gus cuniculu s
1	1.00	1	1	0	0	0	0	-0.003	t	10040	DRRCQSQ LER	Homo sapiens
1	1.00	1	1	0	0	0	0	-0.003	t	9589	DPAFSLNS RAFPAPF	Mus musculu s
1	1.00	1	1	0	0.002	0	0	-0.003	t	6832	CPRPPRAV AYYGPGV DYK	Rattus norvegic us

1	1.00	1	1	0	0	0	0	-0.003	t	3886	APVDSVFDRAFSAYL	Mus musculus
1	1.00	1	1	0	0	0	0	-0.003	t	279	AALGDSLYGAASLNDFGILA	Oryctolagus cuniculus
1	1.00	1	1	0	0	0	0	-0.003	t	6832	CPRPPRAVAYYGPGVDYK	Rattus norvegicus
1	1.00	1	1	0	0	0	0	-0.003	t	3698	APPQA	Mus musculus
1	1.00	1	1	0	0	0	0	-0.003	t	6763	CPFLKQNEPEDIDCW	Mus musculus
1	1.00	1	1	0	0	0	0	-0.003	t	3693	APPPPEHRGGPEEFEGAGDG	Mus musculus
1	1.00	1	1	0	0	0	0	-0.003	t	6832	CPRPPRAVAYYGPGVDYK	Oryctolagus cuniculus
1	1.00	1	1	0	0	0	0	-0.003	t	9588	DPAFSL	Mus musculus
1	1.00	1	1	0	0	0	0	-0.003	t	915	AEGDDPAKA	Mus musculus
1	1.00	1	1	0	0	0	0	-0.003	t	1433	AGAGGGAGGAGAGGGAGGAG	Oryctolagus cuniculus
1	1.00	1	1	0	0	0	0	-0.003	t	6093	CDCDRTAA	Mus musculus
1	1.00	1	1	0	0	0	0	-0.003	t	6226	CFGNSEY	Mus musculus
1	1.00	1	1	0	0	0	0	-0.003	t	10227	DSSAHGTPSTPA	Mus musculus
1	1.00	1	1	0	0	0	0	-0.003	t	8132	DEQFIPK	Mus musculus

1	1.00	1	1	0	-0.003	0	0	-0.003	t	4225	ARQPYGFFLETEEVYQPG	Oryctolagus cuniculus
1	1.00	1	1	0	0	0	0	-0.003	t	9611	DPDKDVDIMV	Mus musculus
1	1.00	1	1	0	0	0	0	-0.003	t	7313	CVTIMTVDNPASTTNKDK	Oryctolagus cuniculus
1	1.00	1	1	0	0	0	0	-0.003	t	7313	CVTIMTVDNPASTTNKDK	Rattus norvegicus
1	1.00	1	1	0	0	0	0	-0.003	t	8273	DFGSLAPRVA	Cavia porcellus
1	1.00	1	1	0	0	0	0	-0.003	t	6632	CLTFGRETVIEY	Mus musculus
1	1.00	1	1	0	0	0	0	-0.003	t	6249	CFPYCYPSESA	Mus musculus
1	1.00	1	1	0	0	0	0	-0.003	t	9520	DNNQTSP	Mus musculus
1	1.00	1	1	0	0	0	0	-0.003	t	1367	AFNQFGPNAGQR	Homo sapiens
1	1.00	1	1	0	0	0	0	-0.003	t	9586	DPAFR	Mus musculus
1	1.00	1	1	0	0	0	0	-0.003	t	8271	DFGPLFSDPGFRSAS	Mus musculus
1	1.00	1	1	0	0	0	0	-0.003	t	3542	APETEK	Mus musculus
1	1.00	1	1	0	0	0	0	-0.003	t	5865	AYLSPIDLNQLAK	Oryctolagus cuniculus
1	1.00	1	1	0	0	0	0	-0.003	t	9591	DPAKAAFD	Mus musculus
1	1.00	1	1	0	0	0	0	-0.003	t	7673	DAVTE	Mus musculus

1	1.00	1	1	0	0	0	0	-0.003	t	4907	ATCDKLFGYYERGIPLPCAL	Homo sapiens
1	1.00	1	1	0.04	0.04	0.04	0.04	0.04	t	5206	ATVSSQSF	Oryctolagus cuniculus
1	1.00	1	1	0	0	-0.006	0	-0.001	t	149681	ELAEAKVLANRE	Mus musculus
1	1.00	1	1	0.01	0.006	0.002	0.013	0.01	t	70437	VPPTQSKKKNKNET	Oryctolagus cuniculus
1	1.00	1	1	0	-0.003	-0.005	0	-0.002	t	13233	ELQGDRRCQSQLERA	Oryctolagus cuniculus
1	1.00	1	1	0.01	0.01	0.01	0.01	0.01	t	57556	SEMPQLP	Mus musculus
1	1.00	1	1	0.01	0.01	0.008	0.01	0.008	t	100074	GNINYACGQGID	Oryctolagus cuniculus
1	1.00	1	1	-0.02	-0.018	-0.02	-0.019	-0.019	t	51428	QLLRIPQAIMDM	Homo sapiens
1	1.00	1	1	0.06	0.06	0.06	0.06	0.06	t	78748	MISGPCPKDGQPSLVLQSSS	Homo sapiens
1	1.00	1	1	0	-0.002	-0.008	0.003	0.002	t	111801	SSILEDWNFGVPPPPTTSLV	Mus musculus
1	1.00	1	1	-0.04	-0.043	-0.043	-0.04	-0.038	t	124708	SFERFEIF	Oryctolagus cuniculus
1	1.00	1	1	-0.01	-0.006	-0.016	-0.003	-0.009	t	134804	TIFILFCCSKEKRKKKQAAT	Homo sapiens
1	1.00	1	1	-0.03	-0.016	-0.036	-0.027	-0.03	t	58087	SGIKDFLPVLQQ	Homo sapiens

1	1.00	1	1	0.03	0.03	0.032	0.033	0.032	t	98786	GASEL	Homo sapiens
1	1.00	1	1	0.02	0.02	0.02	0.022	0.022	t	70273	VPDREVLY REFDEM	Homo sapiens
1	1.00	1	1	0.02	0.018	0.016	0.017	0.02	t	52420	QSRGEAR	Mus musculu s
1	1.00	1	1	0	0.002	-0.006	0.003	-0.001	t	24508	HPSVRYVC LPPERD	Homo sapiens
1	1.00	1	1	-0.03	-0.032	-0.03	-0.031	-0.031	t	22753	GTLTWSG K	Anas platyrhy nchos
1	1.00	1	1	0.02	0.027	0.015	0.019	0.018	t	22395	GSKPPSGS PETGAG	Gallus gallus
1	1.00	1	1	-0.04	-0.04	-0.04	-0.04	-0.04	t	100125	HVGLGTAF	Mus musculu s
1	1.00	1	1	-0.04	-0.038	-0.035	-0.039	-0.046	t	62334	SVSRARTR HLLLCL	Homo sapiens
1	1.00	1	1	-0.01	-0.008	-0.017	-0.013	-0.01	t	116040	YQQKPVA LIN	Homo sapiens
1	1.00	1	1	0	0.002	-0.018	0.001	0.002	t	58196	SGPSGTPS GTSGPSG	Homo sapiens
1	1.00	1	1	0	0	0	0	0	t	140894	QRRADAV KGYLA	Oryctola gus cuniculu s
1	1.00	1	1	-0.03	-0.03	-0.03	-0.026	-0.03	t	135997	SLEKKMSN YIQF	Homo sapiens
1	1.00	1	1	0	0	-0.005	0.006	0	t	45053	NNCTRVEI AEYRRLL	Homo sapiens
1	1.00	1	1	0	0.002	-0.007	0	0	t	107266	VNMVEKG IIDPTKVV	Rattus norvegic us
1	1.00	1	1	0.03	0.03	0.029	0.03	0.03	t	3650	APLTAVAP AHDTPPV PDV	Homo sapiens

1	1.00	1	1	0.01	0.013	0.015	0.01	0.008	t	49967	PVSLGGDVPNS	Mus musculus
1	1.00	1	1	0.01	0.01	0.015	0.01	0.008	t	49967	PVSLGGDVPNS	Mus musculus
1	1.00	1	1	0.03	0.032	0.025	0.037	0.034	t	141604	APPDDGPP	Homo sapiens
1	1.00	1	1	-0.01	-0.013	-0.012	-0.01	-0.01	t	56107	RTLKIPAMTI	Oryctolagus cuniculus
1	1.00	1	1	0.04	0.04	0.051	0.04	0.04	t	55508	RQWFLD	Homo sapiens
1	1.00	1	1	0.02	0.02	0.015	0.02	0.02	t	73636	YEDRYYRE	Mus musculus
1	1.00	1	1	-0.02	-0.023	-0.024	-0.02	-0.022	t	122734	DSVPKWDRLPC	Oryctolagus cuniculus
1	1.00	1	1	0	0.002	-0.004	-0.003	0	t	76352	YVLMVPKQ	Homo sapiens
1	1.00	1	1	0	0.004	-0.012	0.003	0	t	97605	RIVRGPANLRNLKEFKE	Homo sapiens
1	1.00	1	1	-0.02	-0.02	-0.026	-0.017	-0.018	t	29802	KAFRELAD	Homo sapiens
1	1.00	1	1	-0.01	-0.012	-0.01	-0.009	-0.01	t	73785	YEVRNVSGIYHVT	Homo sapiens
1	1.00	1	1	-0.01	-0.013	-0.017	-0.01	-0.01	t	135507	VVGAGP	Oryctolagus cuniculus
1	1.00	1	1	0	0	-0.002	-0.002	-0.002	t	130990	NVLRGMGGAF	Homo sapiens
1	1.00	1	1	0.01	0.008	0.006	0.013	0.011	t	158947	LDIDIDPKVKVATLS	Mus musculus

1	1.00	1	1	0	0	-0.002	0	-0.002	t	150855	HPGGSQP	Mus musculus
1	1.00	1	1	0	0.004	0.002	0.003	0	t	119641	SEHETRLVAKLFKDYS	Homo sapiens
1	1.00	1	1	0.01	0.01	0.011	0.01	0.008	t	31555	KKGSSGYS	Mus musculus
1	1.00	1	1	0.01	0.006	0.013	0.013	0.009	t	30036	KAYSNCYPYDVPDY	Oryctolagus cuniculus
1	1.00	1	1	0.02	0.02	0.014	0.02	0.022	t	79524	CHTTATIPSSAVTC	Mus musculus
1	1.00	1	1	0.01	0.012	0.01	0.01	0.013	t	128955	ILFSVFD	MD
1	1.00	1	1	0	0	0	0.004	0	t	117315	KALSVETEKLLKYLEAV	Homo sapiens
1	1.00	1	1	0	0	0	0.004	0	t	109850	RQHARQQDKERLAALGPEGA	Homo sapiens
1	1.00	1	1	-0.01	-0.007	-0.017	-0.01	-0.011	t	72889	WNK	Mus musculus
1	1.00	1	1	0.04	0.036	0.042	0.043	0.041	t	99422	LKGESGY	Oryctolagus cuniculus
1	1.00	1	1	0.06	0.06	0.053	0.06	0.06	t	133707	STRKEEKQRNGT	Mus musculus
1	1.00	1	1	0.04	0.037	0.038	0.04	0.038	t	35662	LEQPVSNDLS	Oryctolagus cuniculus
1	1.00	1	1	0.01	0.005	0.002	0.013	0.012	t	157357	GVAPPGWYDPPVQ	Canis lupus
1	1.00	1	1	-0.01	-0.01	-0.017	-0.01	-0.01	t	41143	MATAGVAAVVKRKEEN	Mus musculus

1	1.00	1	1	-0.01	-0.01	-0.017	-0.01	-0.01	t	41143	MATAGVAAVVKRKEEN	Mus musculus
1	1.00	1	1	-0.01	-0.01	-0.017	-0.01	-0.01	t	41143	MATAGVAAVVKRKEEN	Mus musculus
1	1.00	1	1	0.02	0.019	0.015	0.021	0.022	t	47101	PDGSKVFAGS	Homo sapiens
1	1.00	1	1	0.02	0.016	0.006	0.017	0.02	t	14227	ESPANNPGLSTVRDE	Oryctolagus cuniculus
1	1.00	1	1	0.02	0.016	0.022	0.023	0.021	t	99760	VKDGYIVD	Oryctolagus cuniculus
1	1.00	1	1	-0.02	-0.022	-0.022	-0.017	-0.019	t	99126	CLAPDRRV	Mus musculus
1	1.00	1	1	0.01	0.014	0.01	0.011	0.009	t	48382	PLKPTKRSFIEDLLF	Homo sapiens
1	1.00	1	1	0.01	0.01	0.01	0.01	0.009	t	11814	EENVEENVEENV	Oryctolagus cuniculus
1	1.00	1	1	-0.01	-0.012	-0.001	-0.003	-0.011	t	113659	NIIKNREGYEMVFDGKP	Homo sapiens
1	1.00	1	1	0	0	0	0	0	t	55698	RRQPIPKARRPE	Homo sapiens
1	1.00	1	1	-0.04	-0.044	-0.046	-0.043	-0.041	t	27553	IMSALAMVYLGAK	Oryctolagus cuniculus
1	1.00	1	1	-0.01	-0.007	-0.009	-0.01	-0.01	t	76606	YYSPAF	Mus musculus
1	1.00	1	1	-0.01	-0.01	-0.009	-0.01	-0.01	t	76606	YYSPAF	Mus musculus
1	1.00	1	1	-0.04	-0.042	-0.048	-0.041	-0.04	t	27416	ILYAVFK	Mus musculus

1	1.00	1	1	-0.04	-0.042	-0.048	-0.041	-0.04	t	27416	ILYAVFK	Mus musculus
1	1.00	1	1	-0.01	-0.01	-0.01	-0.01	-0.008	t	63882	TGLIHLHQ	Homo sapiens
1	1.00	1	1	0.06	0.056	0.059	0.053	0.059	t	118991	RAEDNADTLALVFEA	Oryctolagus cuniculus
1	1.00	1	1	-0.01	-0.01	-0.01	-0.01	-0.012	t	75949	YTASARGDLAHL	Mus musculus
1	1.00	1	1	-0.02	-0.017	-0.024	-0.02	-0.02	t	139198	QARNRPTT	Mus musculus
1	1.00	1	1	-0.01	-0.008	-0.018	-0.014	-0.01	t	110971	PVSDLNLKIDGL	Homo sapiens
1	1.00	1	1	0.03	0.028	0.029	0.029	0.028	t	133611	IVSYLCDLAPEAPPPTLPP	Mus musculus
1	1.00	1	1	-0.01	-0.01	-0.018	-0.01	-0.01	t	10821	DWPERIRDFM	Mus musculus
1	1.00	1	1	0.03	0.03	0.03	0.03	0.029	t	138088	WLDLSKT	Mus musculus
1	1.00	1	1	-0.01	-0.01	-0.004	-0.01	-0.011	t	150371	LLELDKWASLW	Homo sapiens
1	1.00	1	1	0.03	0.033	0.015	0.03	0.036	t	40029	LTLSIDLP	Mus musculus
1	1.00	1	1	-0.01	-0.006	0.001	-0.009	-0.011	t	26338	IGVIIT	Homo sapiens
1	1.00	1	1	0.01	0.008	-0.001	0.009	0.01	t	26306	IGQLVIEHYADT	Mus musculus

1	1.00	1	1	0.03	0.042	0.037	0.03	0.029	t	4702	ASQKIQLV	Pan troglodytes
1	1.00	1	1	0.02	0.022	0.022	0.023	0.016	t	46000	NSTGADAADSGGRT	Homo sapiens
1	1.00	1	1	0.01	0.01	0	0.01	0.01	t	67933	VDENANAN	Oryctolagus cuniculus
1	1.00	1	1	-0.02	-0.02	-0.025	-0.02	-0.021	t	70010	VMPVGGQSSFYSDWY	Homo sapiens
1	1.00	1	1	-0.01	-0.01	-0.022	-0.01	-0.008	t	71139	VSSQSFRRLG	Oryctolagus cuniculus
1	1.00	1	1	0.03	0.03	0.024	0.03	0.031	t	19375	GENK	Mus musculus
1	1.00	1	1	0	0.003	-0.014	0	0.002	t	99964	DVGMHVKEKE	Mus musculus
1	1.00	1	1	-0.02	-0.02	-0.025	-0.02	-0.02	t	12539	EIPALTAVE	Mus musculus
1	1.00	1	1	0	0	-0.007	-0.001	-0.001	t	138082	VVGCGSSHHETH	Homo sapiens
1	1.00	1	1	0.01	0.01	0.01	0.01	0.01	t	31322	KILIGV	Homo sapiens
1	1.00	1	1	0.01	0.014	0.007	0.011	0.009	t	103550	RIFYKDAFAKHQELF	Homo sapiens
1	1.00	1	1	0.02	0.02	0.02	0.02	0.02	t	103420	MSFVTIQPVVLAAAT	Homo sapiens
1	1.00	1	1	0	-0.002	0.001	-0.001	0	t	70101	VNIKPLEDKI	Mus musculus
1	1.00	1	1	0.01	0.01	0.01	0.01	0.01	t	107745	ARGAQGPPGATGFP	Homo sapiens

1	1.00	1	1	0.02	0.02	0.02	0.02	0.019	t	51296	QLCQLL	Mus musculus
1	1.00	1	1	0.02	0.022	0.02	0.021	0.017	t	118478	KVISELNGKNIEDVI	Homo sapiens
1	1.00	1	1	0.01	0.01	0.006	0.01	0.009	t	120060	LGSSESRRALQDSQR	Homo sapiens
1	1.00	1	1	0	0.003	-0.01	0	-0.001	t	139404	YIDINKTIADMLEQYNPNYT	Felis catus
1	1.00	1	1	-0.01	-0.007	-0.003	-0.01	-0.012	t	74940	YLTHMRD	Mus musculus
1	1.00	1	1	0.01	0.01	0.011	0.01	0.009	t	79166	CEGNLACLS	Mus musculus
1	1.00	1	1	-0.02	-0.022	-0.026	-0.021	-0.02	t	51799	QPDLIPL	Mus musculus
1	1.00	1	1	-0.02	-0.024	-0.026	-0.021	-0.02	t	51799	QPDLIPL	Oryctolagus cuniculus
1	1.00	1	1	-0.02	-0.02	-0.029	-0.02	-0.02	t	110424	SGTGGRTGYIPLGTRPPT	Mus musculus
1	1.00	1	1	-0.01	-0.01	-0.022	-0.013	-0.01	t	84698	KSDYLGVNKIAE	Homo sapiens
1	1.00	1	1	-0.02	-0.02	-0.022	-0.02	-0.022	t	145	AAGANPLGLKRGIEKAV	Mus musculus
1	1.00	1	1	0.02	0.018	0.013	0.02	0.021	t	95047	VEVPGSQHIDSQ	Oryctolagus cuniculus
1	1.00	1	1	0.02	0.018	0.013	0.02	0.021	t	95047	VEVPGSQHIDSQ	Oryctolagus cuniculus
1	1.00	1	1	0.02	0.017	0.013	0.02	0.021	t	95047	VEVPGSQHIDSQ	Oryctolagus cuniculus

1	1.00	1	1	0	0	0	0.003	-0.001	t	136080	RAGTDEGFG	Homo sapiens
1	1.00	1	1	-0.02	-0.016	-0.009	-0.013	-0.02	t	138171	FRVVSVL	Homo sapiens
1	1.00	1	1	0.01	0.012	-0.003	0.011	0.01	t	138094	YGYATLSYADYW	Homo sapiens
1	1.00	1	1	0.03	0.03	0.026	0.03	0.03	t	102722	PNKPVEPTYEVIPTPPTDPV	Mus musculus
1	1.00	1	1	-0.03	-0.03	-0.026	-0.03	-0.03	t	71877	VVVLGS	Homo sapiens
1	1.00	1	1	0.04	0.035	0.035	0.04	0.04	t	15176	FAGDSDSSSSADDAAGKATV	Canis lupus
1	1.00	1	1	0.04	0.04	0.04	0.036	0.04	t	117509	GQIPPGAM	Homo sapiens
1	1.00	1	1	-0.04	-0.038	-0.04	-0.033	-0.04	t	138152	CSVMHEA	Homo sapiens
1	1.00	1	1	0	0	-0.013	0	0	t	110248	GGLGIGTGSGTGGRTGYIPL	Mus musculus
1	1.00	1	1	-0.02	-0.02	-0.02	-0.02	-0.02	t	111762	SCFFGRL	Homo sapiens
1	1.00	1	1	0.01	0.01	0.01	0.016	0.013	t	141638	EIHSKTKS	Homo sapiens
1	1.00	1	1	0	-0.002	0	0.007	0	t	123760	SSYAKVSICLPMDTETPLAL	Homo sapiens
1	1.00	1	1	-0.02	-0.02	-0.023	-0.018	-0.02	t	6233	CFKKPPKYPPALPI	Homo sapiens
1	1.00	1	1	0.04	0.042	0.031	0.037	0.042	t	64998	TLPDTEKQI	Homo sapiens
1	1.00	1	1	-0.08	-0.077	-0.08	-0.08	-0.082	t	143687	FRVNLRTLRRYY	Mus musculus

1	1.00	1	1	0	0	0	0	-0.003	t	105127	DAEFRHD	Mus musculus
1	1.00	1	1	-0.02	-0.018	-0.018	-0.019	-0.02	t	51849	QPGYPWPL	Homo sapiens
1	1.00	1	1	0	0.002	0.003	0.001	0	t	54603	RLIVFP	Homo sapiens
1	1.00	1	1	0	0	0	0.009	0.002	t	96211	EDKKRKEEEE	Homo sapiens
1	1.00	1	1	0.02	0.022	0.02	0.027	0.019	t	130933	KVFQGNQDSFTPVVNSLDPP	Homo sapiens
1	1.00	1	1	0.01	0.008	0.003	0.013	0.012	t	106265	ARGLT	Mus musculus
1	1.00	1	1	0.01	0.01	0.01	0.01	0.01	t	52094	QQPP	Mus musculus
1	1.00	1	1	-0.03	-0.028	-0.039	-0.027	-0.03	t	110602	GLKAKPKFTLGKRKATPTT	Homo sapiens
1	1.00	1	1	0.03	0.032	0.028	0.033	0.03	t	78478	PDSDPQI	Homo sapiens
1	1.00	1	1	-0.01	-0.01	-0.014	-0.013	-0.011	t	44227	NIFSGFTPEFLEQAF	Homo sapiens
1	1.00	1	1	0.05	0.05	0.048	0.05	0.048	t	22755	GTLVKTITDDQIEV	Mus musculus
1	1.00	1	1	0.05	0.05	0.048	0.05	0.048	t	22755	GTLVKTITDDQIEV	Mus musculus
1	1.00	1	1	-0.01	-0.01	-0.024	-0.01	-0.01	t	120624	IKSGVATKGL	Mus musculus
1	1.00	1	1	-0.04	-0.04	-0.041	-0.04	-0.038	t	105954	MAKFTQFA	Oryctolagus cuniculus
1	1.00	1	1	0	0	-0.008	0	0	t	47072	PDDHLKERSS	Mus musculus

1	1.00	1	1	0	-0.001	-0.008	0	0	t	47078	PDDVNKNTLQ	Mus musculus
1	1.00	1	1	0	0	-0.008	0	0	t	11353	EDINGIRRPK	Mus musculus
1	1.00	1	1	0	0	-0.008	0	0	t	8918	DKLADYKRRM	Mus musculus
1	1.00	1	1	-0.05	-0.052	-0.042	-0.049	-0.054	t	149384	MSLPGRWKPK	Mus musculus
1	1.00	1	1	-0.03	-0.024	-0.025	-0.03	-0.03	t	54748	RLRVKN	Equus caballus
1	1.00	1	1	0.01	0.01	0.01	0.01	0.008	t	140548	CSLSDAFNGSC	Mus musculus
1	1.00	1	1	-0.01	-0.01	-0.022	-0.01	-0.01	t	115610	GSLPQKAQGHRPQDEN	Oryctolagus cuniculus
1	1.00	1	1	-0.01	-0.001	-0.022	-0.01	-0.01	t	115610	GSLPQKAQGHRPQDEN	Oryctolagus cuniculus
1	1.00	1	1	-0.01	-0.01	-0.022	-0.01	-0.01	t	115610	GSLPQKAQGHRPQDEN	Macaca mulatta
1	1.00	1	1	-0.01	-0.019	-0.022	-0.01	-0.01	t	115610	GSLPQKAQGHRPQDEN	Macaca mulatta
1	1.00	1	1	-0.01	-0.008	-0.022	-0.01	-0.01	t	115610	GSLPQKAQGHRPQDEN	Rattus norvegicus
1	1.00	1	1	-0.01	-0.01	-0.022	-0.01	-0.01	t	115610	GSLPQKAQGHRPQDEN	Oryctolagus cuniculus
1	1.00	1	1	0.02	0.02	0.029	0.02	0.02	t	139801	LGSYSFI	Ovis aries
1	1.00	1	1	0.02	0.026	0.029	0.02	0.02	t	139801	LGSYSFI	Ovis aries

1	1.00	1	1	0.03	0.03	0.022	0.03	0.03	t	84698	KSDYLGVNKIAE	Homo sapiens
1	1.00	1	1	0.02	0.02	0.018	0.021	0.02	t	144791	STIDYFQPNNKR	Sus scrofa
1	1.00	1	1	0	-0.002	0.005	0.003	-0.003	t	94837	GTCKELKKL	Mus musculus
1	1.00	1	1	0.03	0.028	0.027	0.027	0.03	t	122167	PPPITQGECKQL	Mus musculus
1	1.00	1	1	0	0.003	0.002	0	-0.001	t	135842	NTYGDFMVKSPN	Mus musculus
1	1.00	1	1	0	0.003	0.002	0	-0.001	t	135842	NTYGDFMVKSPN	Mus musculus
1	1.00	1	1	0.01	0.01	0.01	0.01	0.008	t	107856	GGQGSRHQQAR	Homo sapiens
1	1.00	1	1	-0.02	-0.018	-0.009	-0.019	-0.024	t	35352	LEAFWHKHM	Homo sapiens
1	1.00	1	1	0.02	0.028	0.027	0.02	0.018	t	136302	GALIYTPEKYTI	Gallus gallus
1	1.00	1	1	-0.02	-0.024	-0.026	-0.017	-0.02	t	7558	DAKDGKEIDTKHLPSTC	Oryctolagus cuniculus
1	1.00	1	1	-0.08	-0.084	-0.072	-0.077	-0.082	t	58956	SKVALAALPA	Oryctolagus cuniculus
1	1.00	1	1	0	0	0	0	0	t	105246	ILDVTT	Oryctolagus cuniculus
1	1.00	1	1	0	0.002	0	0.007	0	t	107875	GTYKAGNQHNHVKYG	Homo sapiens
1	1.00	1	1	-0.03	-0.028	-0.035	-0.029	-0.03	t	142055	VKDTPRLIKLIKNS	Homo sapiens

1	1.00	1	1	0.02	0.024	0.025	0.017	0.017	t	59576	SLVYPFPG PIPNSL	Homo sapiens
1	1.00	1	1	-0.01	-0.01	-0.01	-0.01	-0.012	t	33794	KTNMKH M	Mus musculu s
1	1.00	1	1	0.01	0.007	0.008	0.01	0.007	t	62953	TAPPLLPH SNLDHILE	Oryctola gus cuniculu s
1	1.00	1	1	0.07	0.07	0.07	0.07	0.07	t	99578	QNANEE	Equus caballus
1	1.00	1	1	0.03	0.029	0.029	0.03	0.03	t	432	AASGDPTS PDNIDS	Myodes glareolu s
1	1.00	1	1	-0.04	-0.04	-0.04	-0.04	-0.043	t	121643	NEMHLLIQ QAREMPL	Homo sapiens
1	1.00	1	1	0.06	0.057	0.055	0.06	0.058	t	105963	MDPGLKE SPLQTPAD DGSE	Oryctola gus cuniculu s
1	1.00	1	1	-0.02	-0.02	-0.016	-0.02	-0.02	t	41310	MEDKAW	Homo sapiens
1	1.00	1	1	0.01	0.012	0.01	0.013	0.011	t	18031	FTPDPYQV PF	Homo sapiens
1	1.00	1	1	0.01	0.01	0.01	0.01	0.01	t	100287	LSEKGLTTY	Oryctola gus cuniculu s
1	1.00	1	1	-0.02	-0.02	-0.024	-0.02	-0.018	t	129911	SHYKVG	Homo sapiens
1	1.00	1	1	-0.04	-0.035	-0.044	-0.037	-0.04	t	77800	RGCTFYGC WK	Homo sapiens
1	1.00	1	1	-0.01	-0.01	-0.015	-0.013	-0.01	t	125791	CSFWSKYI QTLK	Mus musculu s
1	1.00	1	1	0	0	0	-0.003	-0.004	t	37660	LLPHSNLD HILEPSIPW KSK	Homo sapiens

1	1.00	1	1	0	0	0	-0.003	-0.004	t	48779	PPFSLSPVPTLGSRSRR	Homo sapiens
1	1.00	1	1	0.02	0.016	0.017	0.02	0.02	t	100387	PLKTDANDQIGDSL	Oryctolagus cuniculus
1	1.00	1	1	0	0	0	0	0	t	62720	SYTESMAGKR	Mus musculus
1	1.00	1	1	0.05	0.05	0.046	0.054	0.05	t	46417	NVLDVNAIDIEEPS	Homo sapiens
1	1.00	1	1	0	0.002	-0.009	0.003	0	t	29965	KATYEAAVAANNAKNAALT	Homo sapiens(human)
1	1.00	1	1	0.03	0.03	0.022	0.029	0.03	t	80395	ALYASVAVQQQD	Homo sapiens
1	1.00	1	1	-0.01	-0.012	-0.01	-0.013	-0.012	t	131656	SGKLEEFVQ	Mus musculus
1	1.00	1	1	0	0	0	0	0	t	50067	PYCWHYAP	Homo sapiens
1	1.00	1	1	-0.01	-0.012	-0.03	-0.017	-0.01	t	127944	DLVNCNLNTNCVAVAFSLPD	Mus musculus
1	1.00	1	1	-0.01	-0.008	-0.017	-0.003	-0.01	t	125401	ELLTEHRMTWDPAQPPRDLT	Homo sapiens
1	1.00	1	1	-0.01	-0.008	-0.017	-0.003	-0.01	t	125401	ELLTEHRMTWDPAQPPRDLT	Homo sapiens
1	1.00	1	1	-0.01	-0.007	-0.021	-0.01	-0.01	t	29897	KANADAKAAYEAAVAANNA	Mus musculus
1	1.00	1	1	0.01	0.007	0.01	0.01	0.007	t	107375	GDDNLAPNTANVQMT	Oryctolagus cuniculus
1	1.00	1	1	0	0.002	-0.005	0.001	0	t	42248	MNWSPTTCL	Homo sapiens

1	1.00	1	1	0.02	0.02	0.003	0.014	0.02	t	39597	LSQDEIDRMVNDAEKY	Homo sapiens
1	1.00	1	1	0	-0.002	0.002	0.011	0.003	t	118599	SAPAPKKGSKKAV	Mus musculus
1	1.00	1	1	0	0	0	0	-0.002	t	47013	PAYYTKDTNNNLT	Mus musculus
1	1.00	1	1	-0.01	-0.005	-0.01	-0.01	-0.01	t	134510	RIIWRSTQ	Homo sapiens
1	1.00	1	1	0	0	0	0	-0.003	t	57631	SESTEDQAMEDIKQMEAESI	Homo sapiens
1	1.00	1	1	-0.03	-0.034	-0.03	-0.027	-0.032	t	156850	APHNKWPHLNPP	Bos taurus
1	1.00	1	1	0.01	0.01	0.008	0.01	0.009	t	32807	KPNLSSKRSELSQLS	Mus musculus
1	1.00	1	1	-0.01	-0.01	-0.01	-0.01	-0.011	t	48782	PPFTSAVGGVDHRS	Mus musculus
1	1.00	1	1	0.01	0.01	0.001	0.01	0.01	t	135986	RSGPSNSASVPA	Homo sapiens
1	1.00	1	1	0	0	0	0	-0.003	t	156939	PPEKSHL	Gallus gallus
1	1.00	1	1	0	0	0	0	-0.003	t	136595	RHYQQYFQHF	Homo sapiens
1	1.00	1	1	0.04	0.04	0.04	0.04	0.041	t	124015	DKTEDVDIEE	Homo sapiens
1	1.00	1	1	0.02	0.02	0.022	0.02	0.02	t	110613	GPPGPQGATGPLGPK	Mus musculus
1	1.00	1	1	0	0	0	0.007	0.001	t	138812	SSVLYGGPPSAA	Rattus norvegicus
1	1.00	1	1	0	0.002	-0.009	-0.002	-0.001	t	110828	EIDGPAGQAEPDRAHYNI	Homo sapiens
1	1.00	1	1	-0.03	-0.034	-0.03	-0.027	-0.033	t	111590	NLIRHVGGGTPRRPG	Oryctolagus cuniculus

1	1.00	1	1	0	0	0	0.007	0.001	t	117211	GYYWSWIRQPPGKGEEWIG	Mus musculus
1	1.00	1	1	0	0	0	0.007	0.001	t	18441	FYNQKFKGKATL	Mus musculus
1	1.00	1	1	-0.01	-0.013	-0.016	-0.01	-0.01	t	31491	KKAIER	Oryctolagus cuniculus
1	1.00	1	1	0.02	0.022	0.021	0.017	0.02	t	8478	DGRGAQVYIGNG	Homo sapiens
1	1.00	1	1	0.01	0.01	0.01	0.01	0.01	t	19005	GDINKVLEKLGYS	Mus musculus
1	1.00	1	1	0.01	0.01	0.01	0.01	0.01	t	19005	GDINKVLEKLGYS	Mus musculus
1	1.00	1	1	0.02	0.018	0.02	0.019	0.018	t	123856	YCDYCDTYLT	Homo sapiens
1	1.00	1	1	-0.05	-0.048	-0.05	-0.043	-0.049	t	118361	FRFLALKLYFSFTRP	Homo sapiens
1	1.00	1	1	0.03	0.03	0.023	0.03	0.029	t	131114	TQLDELLTEHRMTWDPAQPP	Homo sapiens
1	1.00	1	1	0.06	0.058	0.061	0.053	0.06	t	134786	RPVDQY	Mus musculus
1	1.00	1	1	0.02	0.024	0.015	0.017	0.017	t	115228	ELKDLKGYGG	Homo sapiens
1	1.00	1	1	0.02	0.018	0.012	0.017	0.019	t	73311	YAAANPTGPASVQGMSQDPV	Mus musculus
1	1.00	1	1	0.01	0.01	0.01	0.008	0.01	t	6059	CCDLAP	Homo sapiens
1	1.00	1	1	0	0.002	-0.005	-0.003	0	t	159063	RKAADTFNFKTFFHT	Homo sapiens
1	1.00	1	1	-0.01	-0.008	-0.01	-0.009	-0.012	t	146963	GGEDVAVFARGPQ	Homo sapiens
1	1.00	1	1	-0.02	-0.018	-0.025	-0.019	-0.02	t	20084	GGVYLLPRRGPRLGV	Homo sapiens

1	1.00	1	1	0.01	0.012	0.019	0.011	0.01	t	75560	YQTEKFVK	Homo sapiens
1	1.00	1	1	-0.01	-0.012	-0.018	-0.011	-0.012	t	2040	AINRRVE	Mus musculus
1	1.00	1	1	0	0.002	0	0.003	-0.001	t	35397	LEDPASRDLV	Homo sapiens
1	1.00	1	1	0.01	0.008	0.003	0.013	0.012	t	100477	RNKTQEEHLK	Mus musculus
1	1.00	1	1	0.04	0.037	0.046	0.04	0.039	t	12981	ELDGDQMI	Oryctolagus cuniculus
1	1.00	1	1	0	-0.01	0	0.001	0	t	24693	HRMAWDMM	Homo sapiens
1	1.00	1	1	0.05	0.048	0.043	0.053	0.053	t	106771	RERMS	Mus musculus
1	1.00	1	1	0	0.002	0.005	0.001	0	t	23128	GVRVCE	Homo sapiens
1	1.00	1	1	0.08	0.083	0.08	0.08	0.078	t	113679	PEVPEVEDE	Mus musculus
1	1.00	1	1	0	-0.01	0	0.001	0	t	24694	HRMAWDMMM	Homo sapiens
1	1.00	1	1	0.02	0.02	0.02	0.026	0.02	t	76350	YVLLSEKKIS	Homo sapiens
1	1.00	1	1	-0.03	-0.028	-0.03	-0.029	-0.03	t	48005	PIVATNWQK	Homo sapiens
1	1.00	1	1	0.02	0.02	0.02	0.02	0.018	t	75391	YPQPQPFPSQ	Mus musculus
1	1.00	1	1	0	0	0.003	0.003	-0.004	t	115628	LPCRISPGKNAT	Homo sapiens
1	1.00	1	1	0	0	-0.017	0	0.002	t	8920	DKLAKEQKSKQNIGALKQ	Mus musculus

1	1.00	1	1	0	0	-0.017	0	0.002	t	8920	DKLAKEQKSKQNIGALKQ	Mus musculus
1	1.00	1	1	-0.04	-0.038	-0.042	-0.036	-0.04	t	107305	CKVSNKA	Homo sapiens
1	1.00	1	1	0.04	0.042	0.033	0.037	0.037	t	106569	KLSEEVKTTEQK	Homo sapiens
1	1.00	1	1	0.01	0.007	-0.002	0.01	0.011	t	36543	LIGVKGSS	Oryctolagus cuniculus
1	1.00	1	1	0.03	0.032	0.024	0.03	0.032	t	122786	HESNEMQPT	Rattus norvegicus
1	1.00	1	1	0.06	0.06	0.062	0.06	0.059	t	114193	AEYYNKQYLEQTRAELDT	Mus musculus
1	1.00	1	1	0.07	0.072	0.062	0.071	0.07	t	66906	TTVSTEQNVPDPQVGITTMK	Homo sapiens
1	1.00	1	1	-0.02	-0.022	-0.025	-0.021	-0.02	t	56699	SAAFYYRV	Mus musculus
1	1.00	1	1	-0.02	-0.018	-0.011	-0.013	-0.02	t	108895	EGNVFSC	Homo sapiens
1	1.00	1	1	0	0	-0.007	0	0	t	67986	VDINPEL	Mus musculus
1	1.00	1	1	0	0	-0.007	0	0	t	53596	RENP	Mus musculus
1	1.00	1	1	0	0	-0.007	0	0	t	13532	ENP	Mus musculus
1	1.00	1	1	0	0	-0.007	0	0	t	29875	KALRENP	Mus musculus
1	1.00	1	1	0	0	-0.007	0	0	t	13058	ELFSEIQEIR	Mus musculus
1	1.00	1	1	0	0	-0.007	0	0	t	29875	KALRENP	Mus musculus
1	1.00	1	1	0	0	-0.007	0	0	t	29875	KALRENP	Mus musculus

1	1.00	1	1	-0.02	-0.02	-0.023	-0.02	-0.023	t	125238	RLPLPPNQKR	Mus musculus
1	1.00	1	1	0.02	0.024	0.006	0.02	0.022	t	45931	NSNNKEYT	Cavia porcellus
1	1.00	1	1	0	0	0	0	-0.002	t	62858	TAGLVGLLTPG	Rattus norvegicus
1	1.00	1	1	0.01	0.012	0.015	0.011	0.01	t	56273	RVEFLV	Homo sapiens
1	1.00	1	1	0	-0.002	-0.008	-0.001	0	t	11353	EDINGIRRPK	Mus musculus
1	1.00	1	1	0.01	0.01	0.008	0.017	0.01	t	127938	AWAKRARGNVFMYHAPENYG	Homo sapiens
1	1.00	1	1	0.02	0.02	0.02	0.024	0.02	t	135933	GVELRQLTPEQL	Homo sapiens
1	1.00	1	1	0.02	0.02	0.02	0.024	0.02	t	131437	GASVKVT	Homo sapiens
1	1.00	1	1	-0.01	-0.008	-0.01	-0.013	-0.013	t	78145	EKPAEEEKP	Homo sapiens
1	1.00	1	1	0.02	0.02	0.018	0.017	0.018	t	55861	RSILDLDLTK	Homo sapiens
1	1.00	1	1	0.08	0.08	0.08	0.08	0.08	t	99596	RADEEEEYDEDEYEY	Homo sapiens
1	1.00	1	1	0	-0.002	-0.006	-0.001	0	t	133724	TVCAGYCMTRDVN	Mus musculus
1	1.00	1	1	-0.01	-0.008	-0.005	-0.003	-0.01	t	132268	ENGYLMEAAP	Homo sapiens
1	1.00	1	1	0	0.002	0	0.003	0	t	36278	LGQGSFRPSQQN	Homo sapiens
1	1.00	1	1	0.01	0.008	-0.006	0.007	0.01	t	10876	DYEKQINKLKN	Mus musculus
1	1.00	1	1	0.06	0.06	0.051	0.063	0.06	t	68883	VHRLEGVSYNIGSQE	Homo sapiens
1	1.00	1	1	0	0	0	0.003	-0.001	t	44920	NLVPMVATV	Homo sapiens

1	1.00	1	1	0.01	0.007	0.002	0.01	0.009	t	62872	TAGWTFGAGAALQIPFA	Oryctolagus cuniculus
1	1.00	1	1	0.01	0.01	0.01	0.016	0.012	t	53281	RCIPALDSLT	Homo sapiens
1	1.00	1	1	-0.02	-0.024	-0.021	-0.019	-0.021	t	43825	NFELCDNPFFAVSKPMG	Oryctolagus cuniculus
1	1.00	1	1	-0.06	-0.06	-0.059	-0.06	-0.062	t	49	AAALPGKCGV	Homo sapiens
1	1.00	1	1	0	-0.002	0	-0.001	-0.003	t	39183	LRRDLDASREAKKQVEKALE	Mus musculus
1	1.00	1	1	0	-0.002	0	-0.001	-0.003	t	39183	LRRDLDASREAKKQVEKALE	Mus musculus
1	1.00	1	1	0	-0.002	0	-0.001	-0.003	t	39183	LRRDLDASREAKKQVEKALE	Mus musculus
1	1.00	1	1	0.02	0.024	0.011	0.017	0.018	t	113756	QGQQSGQGQQGYYS	Homo sapiens
1	1.00	1	1	-0.05	-0.046	-0.062	-0.053	-0.052	t	104993	SWRSKYGWTA	Homo sapiens
1	1.00	1	1	0	-0.003	0.002	0	-0.001	t	120314	WKAEFHRWSS	Oryctolagus cuniculus
1	1.00	1	1	0.02	0.022	0.013	0.021	0.02	t	51572	QLVLGENLDTERILAS	Homo sapiens
1	1.00	1	1	0	0.002	0.005	0.007	-0.002	t	122341	ESGFLREVLNAVPVLL	Homo sapiens
1	1.00	1	1	0.01	0.01	0.01	0.01	0.009	t	32807	KPNLSSKRSELSQLS	Mus musculus
1	1.00	1	1	0	0.003	-0.014	0	0	t	39788	LTAELKIYSVIQAEINKHL	Mus musculus

1	1.00	1	1	0.02	0.02	0.022	0.02	0.017	t	103544	RGEHRDE HTQDAGD K	Homo sapiens
1	1.00	1	1	0.03	0.03	0.03	0.03	0.028	t	106850	SLQQLYDE YPGP	Mus musculu s
1	1.00	1	1	0.05	0.05	0.057	0.05	0.05	t	130850	GGIYQDLV S	Homo sapiens
1	1.00	1	1	0.02	0.018	0.024	0.017	0.018	t	6068	CCPDKNKS	Mus musculu s
1	1.00	1	1	0	0	0	0.003	-0.001	t	62353	SVTEEIAEE DKSVTEEI AV	Homo sapiens
1	1.00	1	1	0	-0.003	0	0.003	-0.001	t	11819	EENVEHD A	Homo sapiens
1	1.00	1	1	0.02	0.019	0.016	0.023	0.02	t	73191	WVFACAP DRCPPTC	Myodes glareolu s
1	1.00	1	1	0	0.003	0	0	0.001	t	146337	IPEEIKQL	Mus musculu s
1	1.00	1	1	0	0	0	0	0.001	t	146337	IPEEIKQL	Mus musculu s
1	1.00	1	1	0	0	0	0	0.001	t	115376	LTKCEVFR EL	Homo sapiens
1	1.00	1	1	-0.01	-0.011	-0.003	-0.008	-0.012	t	130513	IGYQPSMP LDMP	Camelus dromed arius
1	1.00	1	1	-0.01	-0.01	-0.017	-0.01	-0.008	t	7489	DADYHRV MYA	Oryctola gus cuniculu s
1	1.00	1	1	0.04	0.038	0.038	0.041	0.04	t	142125	DLDASREA KKQLEAEQ QKLE	Homo sapiens
1	1.00	1	1	0	0.003	0	0	0	t	14436	ETKAVYPY	Equus caballus

1	1.00	1	1	0	0	0	0	0	t	14436	ETKAVYPY	Equus caballus
1	1.00	1	1	0	0	0	0	0	t	14436	ETKAVYPY	Mus musculus
1	1.00	1	1	0.04	0.045	0.045	0.04	0.04	t	31056	KGSDVESYSSMPPL	Homo sapiens
1	1.00	1	1	0.01	0.012	0.01	0.011	0.01	t	41015	MADNGTITVEELKQLL	Homo sapiens
1	1.00	1	1	-0.03	-0.032	-0.022	-0.029	-0.034	t	149384	MSLPGRWKPK	Mus musculus
1	1.00	1	1	0.01	0.014	0.01	0.007	0.009	t	106330	DFDCSKIQST	Homo sapiens
1	1.00	1	1	-0.03	-0.028	-0.03	-0.023	-0.03	t	134782	RGRGRGGPRR	Homo sapiens
1	1.00	1	1	0.02	0.025	0.013	0.02	0.02	t	138937	YIGEVLVSV	Homo sapiens
1	1.00	1	1	0.04	0.04	0.04	0.04	0.04	t	18494	GAAAAGAVVGGLG	Oryctolagus cuniculus
1	1.00	1	1	0	0.004	0	0.001	-0.002	t	61186	SSLRDHINVIVS	Homo sapiens
1	1.00	1	1	-0.02	-0.018	-0.022	-0.013	-0.019	t	118200	VGRATPPPGIMA	Homo sapiens
1	1.00	1	1	0.02	0.023	0.012	0.02	0.024	t	76268	YVFDKSELTN	Mus musculus
1	1.00	1	1	0	0	0	0	0	t	9896	DQRPYCWHYP	Homo sapiens
1	1.00	1	1	-0.02	-0.02	-0.02	-0.02	-0.02	t	34310	KWKYENPCKKM	Mus musculus

1	1.00	1	1	-0.03	-0.029	-0.03	-0.027	-0.03	t	78244	PMWPME	Equus caballus
1	1.00	1	1	-0.02	-0.02	-0.023	-0.02	-0.021	t	28371	IRQGTDYKHWPQIAQFA	Homo sapiens
1	1.00	1	1	0.06	0.06	0.052	0.06	0.06	t	106606	LHFVKGEEKEPS	Homo sapiens
1	1.00	1	1	0.03	0.034	0.02	0.027	0.03	t	31406	KIQRDEDSYGRDPYS	Homo sapiens
1	1.00	1	1	0.02	0.022	0.02	0.021	0.02	t	34963	LASLCNPKPSDAPVT	Homo sapiens
1	1.00	1	1	0.02	0.024	0.02	0.021	0.02	t	34963	LASLCNPKPSDAPVT	Homo sapiens
1	1.00	1	1	0.03	0.032	0.023	0.026	0.028	t	50504	QDPDRRDPYSPSPYD	Homo sapiens
1	1.00	1	1	0.05	0.05	0.05	0.05	0.05	t	134421	KQIPECENVKTTQCVF	Mus musculus
1	1.00	1	1	0.05	0.05	0.05	0.05	0.05	t	134421	KQIPECENVKTTQCVF	Mus musculus
1	1.00	1	1	-0.02	-0.018	-0.02	-0.017	-0.02	t	49707	PTLSEIKGVIVHRLE	Homo sapiens
1	1.00	1	1	0.01	0.021	0.01	0.01	0.008	t	156896	KHTSLAS	Gallus gallus
1	1.00	1	1	0	-0.002	-0.004	-0.001	0	t	10824	DWSERIREFM	Mus musculus
1	1.00	1	1	-0.01	-0.005	-0.017	-0.007	-0.01	t	33747	KTKRNTNRRPQDVKF	Homo sapiens
1	1.00	1	1	-0.02	-0.016	-0.016	-0.013	-0.02	t	144644	FRQQHGKTVV	Homo sapiens

1	1.00	1	1	0.01	0.024	0.01	0.01	0.009	t	134128	LASAHRPQGPAD	Rattus rattus
1	1.00	1	1	0.01	0.01	0.008	0.01	0.008	t	55936	RSQSRESQCKLG	Homo sapiens
1	1.00	1	1	0.01	0.008	0.006	0.007	0.008	t	79624	PEEQVPQLIPPPIIPRAG	Mus musculus
1	1.00	1	1	0	0	0	0	-0.002	t	70390	VPNLRGDLQVLAQKVART	Cavia porcellus
1	1.00	1	1	0	0	0	0	-0.002	t	70390	VPNLRGDLQVLAQKVART	Bos taurus
1	1.00	1	1	0	0	0	0	-0.002	t	70390	VPNLRGDLQVLAQKVART	Cavia porcellus
1	1.00	1	1	0	0	0	0	-0.002	t	70390	VPNLRGDLQVLAQKVART	Bos taurus
1	1.00	1	1	0	0	0	0	-0.002	t	54058	RHKQKIVAPVKQTL	Cavia porcellus
1	1.00	1	1	0	0	0	0	-0.002	t	54058	RHKQKIVAPVKQTL	Cavia porcellus
1	1.00	1	1	0	0	0	0	-0.002	t	135970	PPGMRPPMGPMGIPP	Homo sapiens
1	1.00	1	1	-0.03	-0.026	-0.03	-0.027	-0.03	t	136415	KFAGRNFRNPLAK	Homo sapiens
1	1.00	1	1	0	-0.003	0	0	-0.002	t	110385	QLYKTCKQAGTCPPDIIPKV	Oryctolagus cuniculus

1	1.00	1	1	0	0	0	0	-0.002	t	70390	VPNLRGDLQVLAQKVART	Ovis aries
1	1.00	1	1	0	-0.003	0	0	-0.002	t	95040	SHRPREAK	Oryctolagus cuniculus
1	1.00	1	1	0	-0.004	0	0	-0.002	t	70391	VPNLRGDLQVLAQKVARTLP	Mus musculus
1	1.00	1	1	0	0	0	0	-0.002	t	70390	VPNLRGDLQVLAQKVART	Ovis aries
1	1.00	1	1	0	-0.003	0	0	-0.002	t	95032	PAGTGDRL	Oryctolagus cuniculus
1	1.00	1	1	0	-0.003	0	0	-0.002	t	71328	VTGKVEYTKYNDDD	Oryctolagus cuniculus
1	1.00	1	1	0	0	0	0	-0.002	t	36958	LKMADPNRFRGKD	Mus musculus
1	1.00	1	1	0	-0.004	0	0	-0.002	t	54058	RHKQKIVAPVKQTL	Mus musculus
1	1.00	1	1	0	-0.003	0	0	-0.002	t	34537	KYNDDDTFTVKVGD	Oryctolagus cuniculus
1	1.00	1	1	0	-0.003	0	0	-0.002	t	20710	GKVEYTKYNDDDTFTVKVGD	Oryctolagus cuniculus
1	1.00	1	1	0.03	0.03	0.025	0.027	0.031	t	50901	QGLRGEEMEEMV	Homo sapiens
1	1.00	1	1	-0.01	-0.01	-0.01	-0.01	-0.01	t	78455	LPTNLTQ	Mus musculus

1	1.00	1	1	-0.04	-0.042	-0.041	-0.041	-0.042	t	56075	RTHRQLY	Mus musculus
1	1.00	1	1	0	0	-0.009	0	0	t	64534	TKDASIDYHE	Mus musculus
1	1.00	1	1	0.04	0.04	0.04	0.04	0.04	t	115671	AAMEL	Homo sapiens
1	1.00	1	1	0.04	0.04	0.04	0.04	0.04	t	115671	AAMEL	Homo sapiens
1	1.00	1	1	0.03	0.034	0.03	0.031	0.03	t	137882	GQNVLSAD	Homo sapiens
1	1.00	1	1	0.03	0.03	0.03	0.03	0.03	t	156923	NLHTSF	Gallus gallus
1	1.00	1	1	-0.02	-0.016	-0.02	-0.023	-0.022	t	106408	EVPKKCDIKTTL	Homo sapiens
1	1.00	1	1	-0.03	-0.028	-0.03	-0.023	-0.03	t	119580	FMESGEWVIKESRGWKH	Homo sapiens
1	1.00	1	1	0.03	0.03	0.03	0.03	0.028	t	4429	ASGQ	Mus musculus
1	1.00	1	1	0.07	0.07	0.066	0.07	0.07	t	157173	DSDDTEEGEDEG	Canis lupus
1	1.00	1	1	-0.02	-0.023	-0.027	-0.02	-0.021	t	61308	SSQSFRRL	Oryctolagus cuniculus
1	1.00	1	1	-0.03	-0.03	-0.04	-0.027	-0.028	t	52186	QRANLGAFQN	Homo sapiens
1	1.00	1	1	0.02	0.02	0.017	0.02	0.02	t	98677	AASDISLLDA	Homo sapiens
1	1.00	1	1	-0.01	-0.006	-0.003	-0.013	-0.012	t	106411	FALEAYAFDI	Homo sapiens
1	1.00	1	1	0.01	0.008	0.01	0.017	0.01	t	123701	RGVGGPSQ	Homo sapiens

1	1.00	1	1	0.02	0.024	0.008	0.021	0.022	t	7765	DDEHVEEPTVA	Homo sapiens
1	1.00	1	1	0.02	0.022	0.008	0.021	0.022	t	7765	DDEHVEEPTVA	Homo sapiens
1	1.00	1	1	0.01	0.01	0.005	0.01	0.008	t	151672	KLYTLVLTDPDAPSRK	Mus musculus
1	1.00	1	1	0	0	0	0.009	0	t	132099	TLNELPNRT	Homo sapiens
1	1.00	1	1	0.01	0.006	0.008	0.009	0.01	t	45693	NRKNQLKDMA	Oryctolagus cuniculus
1	1.00	1	1	0	0	0	0	0	t	117909	HKGFKGVD	Homo sapiens
1	1.00	1	1	0.03	0.03	0.03	0.034	0.03	t	135988	RTALLDAAGVAS	Homo sapiens
1	1.00	1	1	0.03	0.037	0.033	0.033	0.029	t	64573	TKGENFTETD	Gallus gallus
1	1.00	1	1	-0.01	-0.01	-0.01	-0.006	-0.01	t	135971	PRPRDRSGLAPK	Homo sapiens
1	1.00	1	1	0.01	0.01	0.01	0.01	0.009	t	33122	KRELRNLPQQ	Homo sapiens
1	1.00	1	1	-0.02	-0.02	-0.033	-0.02	-0.02	t	159035	NRFEHQMWESVRDELN	Mus musculus
1	1.00	1	1	-0.02	-0.024	-0.025	-0.021	-0.02	t	69666	VLNLTTWN	Oryctolagus cuniculus
1	1.00	1	1	0.01	0.01	0.019	0.01	0.01	t	139800	LGPYSFV	Ovis aries
1	1.00	1	1	0.02	0.018	0.022	0.023	0.021	t	99604	REDLR	Mus musculus
1	1.00	1	1	-0.07	-0.072	-0.079	-0.067	-0.067	t	115540	VKNKRTFLSPWISNI	Mus musculus

1	1.00	1	1	-0.08	-0.082	-0.079	-0.081	-0.082	t	59326	SLLTYMKM	Mus musculus
1	1.00	1	1	-0.01	-0.006	-0.02	-0.009	-0.01	t	58403	SHRGVIADNQAKWAV	Homo sapiens
1	1.00	1	1	-0.01	-0.008	-0.02	-0.009	-0.01	t	58403	SHRGVIADNQAKWAV	Homo sapiens
1	1.00	1	1	-0.01	-0.008	-0.02	-0.009	-0.01	t	58403	SHRGVIADNQAKWAV	Homo sapiens
1	1.00	1	1	0.01	0.01	0.003	0.01	0.01	t	72503	WGQGG	Mus musculus
1	1.00	1	1	0.03	0.03	0.026	0.03	0.03	t	14841	EVPGSQ	Oryctolagus cuniculus
1	1.00	1	1	0	-0.003	0	0	-0.002	t	64628	TKNTNNNLTL	Oryctolagus cuniculus
1	1.00	1	1	0	-0.003	0	0	-0.002	t	95051	YSQFEKSY	Oryctolagus cuniculus
1	1.00	1	1	0	-0.003	0	0	-0.002	t	64628	TKNTNNNLTL	Oryctolagus cuniculus
1	1.00	1	1	0	-0.003	0	0	-0.002	t	95032	PAGTGDRL	Oryctolagus cuniculus
1	1.00	1	1	0.03	0.034	0.03	0.031	0.028	t	118497	LNGKNIEDVIAQGIG	Homo sapiens
1	1.00	1	1	0.07	0.072	0.07	0.071	0.069	t	150971	CDNTCMESVKNGTYD	Homo sapiens
1	1.00	1	1	0.01	0.012	0.01	0.011	0.01	t	50215	QAAPYIEQA	Homo sapiens
1	1.00	1	1	0.04	0.04	0.04	0.037	0.04	t	62447	SWGANDTDVFVL	Homo sapiens

1	1.00	1	1	-0.03	-0.03	-0.026	-0.03	-0.032	t	78628	DQKPPSR	Oryctolagus cuniculus
1	1.00	1	1	-0.01	-0.012	-0.005	-0.01	-0.01	t	138440	IKRYNSTA	Oryctolagus cuniculus
1	1.00	1	1	-0.04	-0.044	-0.06	-0.047	-0.04	t	127986	FVGVGADYRHASCPIDYIIA	Oryctolagus cuniculus
1	1.00	1	1	-0.04	-0.042	-0.06	-0.047	-0.04	t	127986	FVGVGADYRHASCPIDYIIA	Mus musculus
1	1.00	1	1	0	0.003	-0.006	0	0	t	28537	ISIKYDPRKDSEVFA	Mus musculus
1	1.00	1	1	0	-0.002	0.003	-0.001	-0.002	t	14749	EVKKKSDS	Mus musculus
1	1.00	1	1	0.06	0.06	0.06	0.06	0.06	t	137551	PEEVLANAFQISRED	Homo sapiens
1	1.00	1	1	0.03	0.034	0.034	0.031	0.03	t	45659	NRDFVE	Homo sapiens
1	1.00	1	1	0.03	0.03	0.02	0.033	0.032	t	34942	LARGSPPSLASSSASQLSA	Homo sapiens
1	1.00	1	1	0	0.002	-0.002	0.003	0.003	t	9586	DPAFR	Homo sapiens
1	1.00	1	1	0.01	0.006	-0.002	0.009	0.01	t	10785	DVVTGATV	Oryctolagus cuniculus
1	1.00	1	1	0	0	-0.017	0	0.001	t	56102	RTLDQQYKEVKRETDLSHVQ	Mus musculus
1	1.00	1	1	-0.01	-0.01	-0.012	-0.01	-0.013	t	95728	QPPVSQFHIQGQ	Homo sapiens
1	1.00	1	1	-0.02	-0.02	-0.033	-0.018	-0.017	t	98697	AMCNVYIPPYCT	Homo sapiens

1	1.00	1	1	0.04	0.04	0.042	0.04	0.04	t	95008	EDVFQNG FTAW	Mus musculus
1	1.00	1	1	0.04	0.036	0.045	0.04	0.04	t	146471	NFDIPEEI	Oryctolagus cuniculus
1	1.00	1	1	0.01	0.014	-0.001	0.007	0.01	t	21184	GLVDCYIN L	Homo sapiens
1	1.00	1	1	-0.02	-0.02	-0.029	-0.02	-0.02	t	102535	INLVHWN KKKYSSYEE AKKH	Mus musculus
1	1.00	1	1	0	0	-0.004	0	-0.001	t	52082	QQPFP	Mus musculus
1	1.00	1	1	0.04	0.04	0.034	0.04	0.042	t	40076	LTPCD	Mus musculus
1	1.00	1	1	0.03	0.03	0.03	0.03	0.028	t	137284	RTNTTQG KVPSQ	Sus scrofa
1	1.00	1	1	-0.03	-0.027	-0.031	-0.03	-0.032	t	94513	FHPDK	Mus musculus
1	1.00	1	1	0.03	0.03	0.03	0.041	0.032	t	118439	IVLERSES WNLKDLDI	Homo sapiens
1	1.00	1	1	0.02	0.02	0.02	0.02	0.018	t	118762	CYGSFAKP TN	Mus musculus
1	1.00	1	1	-0.02	-0.024	-0.03	-0.02	-0.02	t	64752	TLDHVLDH	Anas platyrhynchos
1	1.00	1	1	0.02	0.022	0.025	0.021	0.02	t	41057	MAKNEV	Homo sapiens
1	1.00	1	1	0.03	0.026	0.011	0.027	0.032	t	427	AASDYFAD ATLGKDIS GLDE	Oryctolagus cuniculus
1	1.00	1	1	0.03	0.036	0.03	0.03	0.03	t	123594	PLNSWIAS F	Ovis aries

1	1.00	1	1	0.01	0.012	0.012	0.009	0.007	t	96808	QVKSGTIF DN	Homo sapiens
1	1.00	1	1	0.03	0.028	0.018	0.031	0.03	t	34927	LAQEAGN FERISGDL	Homo sapiens
1	1.00	1	1	-0.03	-0.03	-0.03	-0.03	-0.033	t	45005	NMMNYI MDPRTH	Oryctola gus cuniculu s
1	1.00	1	1	-0.03	-0.027	-0.03	-0.03	-0.033	t	45005	NMMNYI MDPRTH	Mus musculu s
1	1.00	1	1	0.02	0.018	0.012	0.019	0.02	t	73311	YAAANPT GPASVQG MSQDPV	Mus musculu s
1	1.00	1	1	-0.01	-0.008	-0.015	-0.007	-0.01	t	121034	VHPDTGIS SKAMGIM N	Homo sapiens
1	1.00	1	1	0.02	0.016	0.016	0.017	0.018	t	53409	RDPYSPSQ DPYSPSQ	Oryctola gus cuniculu s
1	1.00	1	1	0	0.004	0.002	0.001	0	t	62447	SWGANDT DVFVL	Homo sapiens
1	1.00	1	1	0	0	-0.01	-0.003	-0.001	t	24104	HITHSGME GMGVSCT	Homo sapiens
1	1.00	1	1	0.02	0.018	0.021	0.017	0.019	t	144364	ITDAVITVP AYFND	Mus musculu s
1	1.00	1	1	0.02	0.015	0.021	0.017	0.019	t	144364	ITDAVITVP AYFND	Bos taurus
1	1.00	1	1	0.01	0.015	0.017	0.01	0.008	t	132869	LSPLGEEM	Mus musculu s
1	1.00	1	1	-0.01	-0.01	-0.01	-0.01	-0.01	t	132885	PVRLAE	Oryctola gus cuniculu s

1	1.00	1	1	0	-0.01	-0.005	0.001	0	t	75863	YSPSRSFSYFYPFRL	Homo sapiens
1	1.00	1	1	0.02	0.018	0.013	0.02	0.019	t	38422	LPAYQDYTARAQVSE	Oryctolagus cuniculus
1	1.00	1	1	0.06	0.06	0.043	0.06	0.062	t	48898	PPQQPPQQPPQQPPQQ	Mus musculus
1	1.00	1	1	0	-0.004	-0.005	-0.001	-0.002	t	39087	LRFPRGQGVPINTNS	Oryctolagus cuniculus
1	1.00	1	1	0.01	0.008	0.01	0.009	0.008	t	66894	TTVNAHQILPKV	Mus musculus
1	1.00	1	1	0.03	0.032	0.03	0.033	0.03	t	62993	TASITSPLTTQNTL	Homo sapiens
1	1.00	1	1	0.03	0.032	0.03	0.037	0.03	t	107345	EQRRAA	Homo sapiens
1	1.00	1	1	0.03	0.032	0.03	0.037	0.03	t	107345	EQRRAA	Homo sapiens
1	1.00	1	1	0.03	0.026	0.032	0.033	0.032	t	8156	DERTLTKE	Oryctolagus cuniculus
1	1.00	1	1	0.02	0.018	0.017	0.013	0.019	t	125400	ELLKLLIEA	Mus musculus
1	1.00	1	1	0.02	0.02	0.019	0.02	0.019	t	21722	GPPAAEYWNSQ	Homo sapiens
1	1.00	1	1	0	0	0	0	-0.002	t	108202	VVSHFND	Mus musculus
1	1.00	1	1	0.01	0.01	0.003	0.014	0.009	t	44917	NLVLNRDVSFQDL	Homo sapiens
1	1.00	1	1	0.03	0.032	0.03	0.031	0.03	t	116947	DIDYSDYSTR	Homo sapiens
1	1.00	1	1	-0.02	-0.017	-0.02	-0.02	-0.022	t	72153	VYRYDSRP	Mus musculus

1	1.00	1	1	-0.02	-0.017	-0.02	-0.02	-0.022	t	72153	VYRYDSRP	Mus musculus
1	1.00	1	1	0	-0.002	0	-0.007	-0.006	t	125297	VFFEEQED EIIGFG	Mus musculus
1	1.00	1	1	0.05	0.054	0.05	0.053	0.05	t	117315	KALSVETE KLLKYLEAV	Homo sapiens
1	1.00	1	1	-0.02	-0.016	-0.025	-0.019	-0.018	t	26961	ILALLPQG VF	Homo sapiens
1	1.00	1	1	0.04	0.042	0.031	0.041	0.04	t	45603	NQPGVHI QVFEGERA MTKDC	Homo sapiens
1	1.00	1	1	0.04	0.037	0.031	0.041	0.04	t	45603	NQPGVHI QVFEGERA MTKDC	Canis lupus
1	1.00	1	1	0.02	0.018	0.025	0.027	0.021	t	66161	TSAPDTRP AP	Mus musculus
1	1.00	1	1	0.03	0.033	0.026	0.029	0.031	t	28537	ISIKYDPRK DSEVFA	Mus musculus
1	1.00	1	1	-0.03	-0.026	-0.03	-0.023	-0.03	t	122842	LLDLPRDL GGMGCSS PPCE	Homo sapiens
1	1.00	1	1	0.02	0.023	0.023	0.02	0.02	t	125127	LDPENLTL	Mus musculus
1	1.00	1	1	-0.01	-0.01	-0.01	-0.01	-0.013	t	136754	YHWKVFY KYD	Homo sapiens
1	1.00	1	1	0.02	0.016	0.023	0.023	0.02	t	111255	ENQKAGGI EQNFGLF	Oryctolagus cuniculus
1	1.00	1	1	0.03	0.028	0.028	0.031	0.03	t	20288	GIGVTQN VLYENQK QIA	Homo sapiens

1	1.00	1	1	0	0	0	0	0	t	140861	GTVSYGEE KPAV	Oryctola gus cuniculu s
1	1.00	1	1	-0.03	-0.03	-0.025	-0.03	-0.034	t	133542	DGRRDVV VRSATFYL	Mus musculu s
1	1.00	1	1	0.01	0.012	0.006	0.011	0.01	t	28340	IRMISDQR AN	Homo sapiens
1	1.00	1	1	0.01	0.007	0.005	0.01	0.01	t	65238	TMPFSPDF SNVD	Oryctola gus cuniculu s
1	1.00	1	1	0	0.003	0	0	0	t	141017	FTTWSPW SQPLA	Mus musculu s
1	1.00	1	1	0.01	0.01	0.01	0.014	0.01	t	107892	HSASQDG QDTIRGHP GSS	Homo sapiens
1	1.00	1	1	0.03	0.03	0.027	0.034	0.03	t	33831	KTQIDQVE STAGSLQ	Homo sapiens
1	1.00	1	1	0.05	0.047	0.043	0.05	0.049	t	137469	YPPEEETG ERV	Oryctola gus cuniculu s
1	1.00	1	1	0.03	0.032	0.031	0.031	0.03	t	68800	VGTAEEKL KKKSSF	Homo sapiens
1	1.00	1	1	0.02	0.02	0.02	0.02	0.02	t	79828	FLGPKQLT FEGK	Mus musculu s
1	1.00	1	1	0.02	0.018	0.027	0.021	0.02	t	78923	AFGANS	Mus musculu s
1	1.00	1	1	0.02	0.017	0.009	0.02	0.021	t	80002	RKADLEKA LEGAM	Oryctola gus cuniculu s
1	1.00	1	1	-0.02	-0.02	-0.032	-0.02	-0.02	t	34490	KYKVPQLE IV	Homo sapiens
1	1.00	1	1	0.03	0.027	0.024	0.03	0.03	t	105647	ADVNTFV A	Oryctola gus cuniculu s

1	1.00	1	1	-0.05	-0.048	-0.05	-0.043	-0.05	t	145149	DRQHLPLIK	Homo sapiens
1	1.00	1	1	-0.04	-0.042	-0.04	-0.037	-0.04	t	103912	FWLDFW	Mus musculus
1	1.00	1	1	0.07	0.07	0.055	0.07	0.072	t	33715	KTIENINELIEES	Homo sapiens
1	1.00	1	1	-0.01	-0.01	-0.01	-0.01	-0.012	t	12625	EKAFREL	Oryctolagus cuniculus
1	1.00	1	1	0.04	0.043	0.045	0.04	0.04	t	138228	QLDFEDV	Mus musculus
1	1.00	1	1	-0.02	-0.017	-0.027	-0.017	-0.02	t	76165	YTQQRRVIGEFR	Capra hircus
1	1.00	1	1	0.02	0.017	0.018	0.02	0.019	t	105867	HTDFAGAE	Oryctolagus cuniculus
1	1.00	1	1	0.02	0.02	0.022	0.02	0.02	t	110611	GPKGQAGEPGIAGFK	Mus musculus
1	1.00	1	1	0.01	0.01	0.01	0.01	0.01	t	5562	AVTKGTINDPEA	Oryctolagus cuniculus
1	1.00	1	1	0.01	0.01	0.01	0.01	0.01	t	5562	AVTKGTINDPEA	Oryctolagus cuniculus
1	1.00	1	1	-0.03	-0.018	-0.028	-0.027	-0.03	t	55761	RRVGLSNPT	Anas platyrhynchos
1	1.00	1	1	0.01	0.01	0.01	0.014	0.01	t	131038	RKIAKDEGAK	Homo sapiens
1	1.00	1	1	0.02	0.022	0.016	0.017	0.018	t	52106	QQPQQSFPQQ	Homo sapiens
1	1.00	1	1	-0.02	-0.018	-0.023	-0.013	-0.018	t	120805	MSGRGKQGGKARAKA	Homo sapiens

1	1.00	1	1	-0.01	-0.01	-0.01	-0.01	-0.012	t	105651	AEFRHDS	Mus musculus
1	1.00	1	1	0.01	0.009	0.007	0.011	0.012	t	36805	LKASAA	Homo sapiens
1	1.00	1	1	0.04	0.038	0.033	0.039	0.038	t	133684	RRIASQVKYAGSQVASTSE	Mus musculus
1	1.00	1	1	0.01	0.01	0.01	0.01	0.01	t	16960	FLVNTW	Homo sapiens
1	1.00	1	1	0.02	0.022	0.019	0.021	0.022	t	63878	TGLDFN	Homo sapiens
1	1.00	1	1	0	-0.002	0	0.001	-0.002	t	99921	DAEFRHDS	Mus musculus
1	1.00	1	1	0.03	0.032	0.032	0.031	0.029	t	103393	LTTYILLPSQDLPLL	Homo sapiens
1	1.00	1	1	0.02	0.02	0.016	0.017	0.02	t	74075	YGRDPYSPSQDPYSP	Homo sapiens
1	1.00	1	1	-0.03	-0.033	-0.041	-0.03	-0.03	t	6541	CLAVLLGACS	Oryctolagus cuniculus
1	1.00	1	1	-0.02	-0.016	-0.024	-0.02	-0.021	t	79054	QPDGGQPAVRNERAT	MD
1	1.00	1	1	-0.02	-0.022	-0.024	-0.02	-0.021	t	79054	QPDGGQPAVRNERAT	Oryctolagus cuniculus
1	1.00	1	1	0.04	0.04	0.031	0.04	0.04	t	22691	GTHPSSSAGLKNDLLEN	Mus musculus
1	1.00	1	1	0	-0.003	0.005	0	-0.003	t	29218	IVCIDRASLST	Oryctolagus cuniculus
1	1.00	1	1	0.02	0.016	0.02	0.023	0.023	t	115397	NLLSICKTAEF	Oryctolagus cuniculus
1	1.00	1	1	0.02	0.022	0.02	0.021	0.019	t	150965	AKSFYKNLIWLVKKG	Homo sapiens

1	1.00	1	1	-0.01	-0.014	-0.004	-0.007	-0.01	t	36834	LKEKYKDK	Oryctolagus cuniculus
1	1.00	1	1	0.03	0.034	0.025	0.027	0.028	t	71669	VVGDEGRSVFDD	Homo sapiens
1	1.00	1	1	0.01	0.007	-0.002	0.01	0.01	t	30993	KGKKVDLYGAYYGYQCAGGT	Oryctolagus cuniculus
1	1.00	1	1	0	-0.004	0.007	0.003	0	t	697	ADKSSILFLCDD	Oryctolagus cuniculus
1	1.00	1	1	-0.04	-0.04	-0.047	-0.04	-0.041	t	138028	SAGALQLLVVGC	Oryctolagus cuniculus
1	1.00	1	1	0	0	0	0	-0.002	t	117675	VEPKVKSKKREAV	Homo sapiens
1	1.00	1	1	0.07	0.07	0.07	0.07	0.07	t	99007	SLQPED	Homo sapiens
1	1.00	1	1	0.02	0.02	0.02	0.02	0.019	t	119516	RKLAHPGTS	Homo sapiens
1	1.00	1	1	0.02	0.018	0.01	0.019	0.02	t	26700	IITFKSGATF	Mus musculus
1	1.00	1	1	0.08	0.08	0.075	0.08	0.08	t	20174	GHQVGDGE	Homo sapiens
1	1.00	1	1	0	0	0	0	-0.002	t	158947	LDIDIDPKVKVATLS	Mus musculus
1	1.00	1	1	0	0	0	0	-0.002	t	139154	KWEAAREAEQRR	Oryctolagus cuniculus
1	1.00	1	1	0	0	0	0	-0.002	t	118877	LDVEQLGIPEQEYSC	Oryctolagus cuniculus

1	1.00	1	1	0	0	0	0	-0.002	t	124588	PQEETLQAFDSHYDYT	Oryctolagus cuniculus
1	1.00	1	1	0	0	0	0	-0.002	t	122842	LLDLPRDLGGMGCSSPPCE	Oryctolagus cuniculus
1	1.00	1	1	0	0	0	0	-0.002	t	117520	GSFSDADLAD	Mus musculus
1	1.00	1	1	0	0	0	0	-0.002	t	131406	EGEGSGFIREDPIGAGA	Oryctolagus cuniculus
1	1.00	1	1	0	0	0	0	-0.002	t	120172	RKIPKRPGSVHRTPSRQ	Oryctolagus cuniculus
1	1.00	1	1	0	0	0	0	-0.002	t	131717	TTLVNPNKKNSYPHY	Oryctolagus cuniculus
1	1.00	1	1	0	0	0	0	-0.002	t	122790	HQEEDFRVTCKDIQR	Oryctolagus cuniculus
1	1.00	1	1	0	0	0	0	-0.002	t	120769	LLVDVEPKVKSKKRE	Mus musculus
1	1.00	1	1	0	0	0	0	-0.002	t	122746	EEQEDEIIGFGQELKN	Oryctolagus cuniculus
1	1.00	1	1	0	0	0	0	-0.002	t	120705	KNQFDHYSRHESCAEL	Oryctolagus cuniculus
1	1.00	1	1	0	0	0	0	-0.002	t	119052	WNPADYGGIK	Rattus norvegicus
1	1.00	1	1	0	0	0	0	-0.002	t	126201	GYKEKSKFQDTHNNA	Oryctolagus cuniculus
1	1.00	1	1	0	0	0	0	-0.002	t	131701	THHINNMKPSFTRENT	Oryctolagus cuniculus

1	1.00	1	1	0	0	0	0	-0.002	t	127643	ERPLELRVRVLDI	Homo sapiens
1	1.00	1	1	0	0	0	0	-0.002	t	133775	AETNRAPFDLAEAE	Oryctolagus cuniculus
1	1.00	1	1	0	0	0	0	-0.002	t	124384	KRSRHF	Oryctolagus cuniculus
1	1.00	1	1	0	0	0	0	-0.002	t	132848	KKEDFDIYDEDENQSPR	Mus musculus
1	1.00	1	1	0	0	0	0	-0.002	t	117506	GGAHFSVSSLAEG	Mus musculus
1	1.00	1	1	0	0	0	0	-0.002	t	119382	VISCAKDAIEDEEGSC	Oryctolagus cuniculus
1	1.00	1	1	0	0	0	0	-0.002	t	122861	NALNSPLHQEYEENL	Oryctolagus cuniculus
1	1.00	1	1	0	0	0	0	-0.002	t	102384	EFRH	Mus musculus
1	1.00	1	1	0	0	0	0	-0.002	t	103309	KKNGRILTLPRSNPS	Mus musculus
1	1.00	1	1	0	0	0	0	-0.002	t	113129	QLELPAVTPEDDGE	Mus musculus
1	1.00	1	1	0	0	0	0	-0.002	t	111193	DGPAGQAEPDRAHYNI	Mus musculus
1	1.00	1	1	0	0	0	0	-0.002	t	100777	DIDFLIEEIERLGQDL	Oryctolagus cuniculus
1	1.00	1	1	0	0	0	0	-0.002	t	112958	ELRPELSWLGHE	Mus musculus
1	1.00	1	1	0	0	0	0	-0.002	t	106494	GYENPTYKFFEQMQN	Oryctolagus cuniculus
1	1.00	1	1	0	0	0	0	-0.002	t	108522	LHEYMLDLQPETTDL	Mus musculus

1	1.00	1	1	0	0	0	0	-0.002	t	106842	SKDEIDSAVKMLVSLKMSY	Mus musculus
1	1.00	1	1	0	0	0	0	-0.002	t	104569	MASPGSGFWSFGSEDGS	Mus musculus
1	1.00	1	1	0	0	0	0	-0.002	t	103496	PRGYPGQV	Mus musculus
1	1.00	1	1	0	0	0	0	-0.002	t	106349	DPLRPQREGC	Oryctolagus cuniculus
1	1.00	1	1	0	0	0	0	-0.002	t	97348	GLFGAIAGFIEGGW	Oryctolagus cuniculus
1	1.00	1	1	0	0	0	0	-0.002	t	94836	GSSK	Oryctolagus cuniculus
1	1.00	1	1	0	0	0	0	-0.002	t	69175	VKAETRLN	Mus musculus
1	1.00	1	1	0	0	0	0	-0.002	t	94979	YNKRFPGFVSP	Oryctolagus cuniculus
1	1.00	1	1	0	0	0	0	-0.002	t	60751	SRNWLPGPCY	Mus musculus
1	1.00	1	1	0	0	0	0	-0.002	t	94606	LTGGFYEPL	Mus musculus
1	1.00	1	1	0	0	0	0	-0.002	t	95048	VIFQTPLIKNPDVKSAIEG	Rattus norvegicus
1	1.00	1	1	0	0	0	0	-0.002	t	95004	DEESSNAAEEWKYVAMVIDH	Rattus norvegicus
1	1.00	1	1	0	0	0	0	-0.002	t	75278	YPEKAVLDEYDYRYNRPK	Mus musculus
1	1.00	1	1	0	0	0	0	-0.002	t	95524	KRQIYTDIEMNRLGK	Oryctolagus cuniculus

1	1.00	1	1	0	0	0	0	-0.002	t	94496	EEEEYMPMEDLYLDILPEE	Oryctolagus cuniculus
1	1.00	1	1	0	0	0	0	-0.002	t	43232	NANNPDWDF	Mus musculus
1	1.00	1	1	0	0	0	0	-0.002	t	37698	LLQAHRQLEER	Mus musculus
1	1.00	1	1	0	0	0	0	-0.002	t	48906	PPRRVS	Mus musculus
1	1.00	1	1	0	0	0	0	-0.002	t	48876	PPPQAPYQGY	Mus musculus
1	1.00	1	1	0	0	0	0	-0.002	t	46894	PAPQAPYQGY	Mus musculus
1	1.00	1	1	0	-0.003	0	0	-0.002	t	55389	RQAESSEAMAAWSERAGEA	Oryctolagus cuniculus
1	1.00	1	1	0	0	0	0	-0.002	t	41537	MFSGGGGPLSPGGKS	Mus musculus
1	1.00	1	1	0	0	0	0	-0.002	t	56734	SADNNNSEYSWT	Mus musculus
1	1.00	1	1	0	0.004	0	0	-0.002	t	49680	PTKEFFREA	Mus musculus
1	1.00	1	1	0	0	0	0	-0.002	t	48779	PPFSLSPVPTLGSRSRR	Oryctolagus cuniculus
1	1.00	1	1	0	0	0	0	-0.002	t	13837	EQHHRRTDN	Mus musculus
1	1.00	1	1	0	0	0	0	-0.002	t	28676	ISQAVHAAHAEINEAGR	Homo sapiens
1	1.00	1	1	0	-0.003	0	0	-0.002	t	28676	ISQAVHAAHAEINEAGR	Oryctolagus cuniculus

1	1.00	1	1	0	0	0	0	-0.002	t	19067	GDPEDL	Mus musculus
1	1.00	1	1	0	0	0	0	-0.002	t	30661	KEVPALTAVETGAT	Oryctolagus cuniculus
1	1.00	1	1	0	0	0	0	-0.002	t	36729	LITNTIAGV	Mus musculus
1	1.00	1	1	0	0	0	0	-0.002	t	9044	DLDGAA	Mus musculus
1	1.00	1	1	0	0	0	0	-0.002	t	9620	DPEDLD	Mus musculus
1	1.00	1	1	0.01	0.01	0.01	0.01	0.01	t	99267	GEPDLSNNFGKLFEV	Homo sapiens
1	1.00	1	1	0.01	0.01	0.003	0.01	0.013	t	116169	TPRTPPPSQGKG	Oryctolagus cuniculus
1	1.00	1	1	0.01	0.021	0.003	0.01	0.013	t	116169	TPRTPPPSQGKG	Gallus gallus
1	1.00	1	1	0	-0.003	0	0	0	t	7849	DDPPATVYRYD	Oryctolagus cuniculus
1	1.00	1	1	0.07	0.059	0.07	0.07	0.07	t	125610	YYVFFEEQEDEIIGFGQELK	Cricetulus migratorius
1	1.00	1	1	0	0	0	0	-0.002	t	151731	SAQSQRAPDR	Mus musculus
1	1.00	1	1	0	0	0	0	-0.002	t	120849	NSQPEILERTRAELD	Mus musculus
1	1.00	1	1	0	0	0	0	-0.002	t	123562	NSSSSQFQIHGPR	Mus musculus
1	1.00	1	1	0	0	0	0	-0.002	t	120849	NSQPEILERTRAELD	Mus musculus
1	1.00	1	1	0	0	0	0	-0.002	t	120849	NSQPEILERTRAELD	Mus musculus

1	1.00	1	1	-0.02	-0.02	-0.02	-0.02	-0.018	t	123294	HMPGQPPY	Oryctolagus cuniculus
1	1.00	1	1	0.01	0	0.01	0.017	0.01	t	134205	QRGNQIMSDKRNVILFSVFD	Homo sapiens
1	1.00	1	1	-0.03	-0.028	-0.037	-0.023	-0.028	t	118348	FHSGKHYWEVDVTGKE	Homo sapiens
1	1.00	1	1	0.02	0.022	0.016	0.017	0.017	t	114762	DCTAEDRPLC	Homo sapiens
1	1.00	1	1	-0.01	-0.002	-0.01	-0.01	-0.012	t	156974	WGTHTSYPWRLS	Gallus gallus
1	1.00	1	1	0	0	-0.009	-0.003	0	t	53109	RAGVCY	Homo sapiens
1	1.00	1	1	-0.01	-0.01	-0.024	-0.01	-0.01	t	6832	CPRPPRAVAYYGPGVDYK	Oryctolagus cuniculus
1	1.00	1	1	0	0.003	0	0	-0.002	t	9106	DLGSIA	Mus musculus
1	1.00	1	1	0.03	0.03	0.03	0.037	0.03	t	117364	RPDAEYWNSQKDLLEQRRAA	Homo sapiens
1	1.00	1	1	0.02	0.022	0.023	0.023	0.019	t	23162	GVSYNIGSQEWYTTV	Homo sapiens
1	1.00	1	1	0.01	0.007	0.01	0.01	0.01	t	76616	YYTKDTNNNL	Oryctolagus cuniculus
1	1.00	1	1	0.01	0.01	0.01	0.01	0.01	t	76616	YYTKDTNNNL	Mus musculus
1	1.00	1	1	0	0	0	0.003	-0.001	t	117300	GSGKGKVQFQGKKTKF	Homo sapiens
1	1.00	1	1	-0.03	-0.03	-0.034	-0.03	-0.03	t	106861	SPDWFRH	Mus musculus
1	1.00	1	1	0.02	0.022	0.01	0.017	0.02	t	37997	LMCDIDGNRL	Homo sapiens

1	1.00	1	1	0.05	0.048	0.057	0.049	0.05	t	78499	QVADKE	Mus musculus
1	1.00	1	1	0.02	0.02	0.02	0.02	0.02	t	149046	FQINPTQ	Sus scrofa
1	1.00	1	1	0	-0.004	-0.006	0.003	0.002	t	12258	EGRFKSQD	Oryctolagus cuniculus
1	1.00	1	1	0.03	0.023	0.025	0.03	0.03	t	136705	VEEERFHQQGAPRA	Oryctolagus cuniculus
1	1.00	1	1	-0.01	-0.006	-0.008	-0.009	-0.01	t	46297	NTTYILVKGK	Homo sapiens
1	1.00	1	1	-0.01	-0.011	-0.013	-0.01	-0.011	t	12537	EINTLQNQ	Anas platyrhynchos
1	1.00	1	1	-0.06	-0.058	-0.06	-0.053	-0.059	t	126137	GLKKMLRTCAVHITL	Homo sapiens
1	1.00	1	1	0	-0.004	0	0.003	-0.001	t	19738	GGGAGGAGAGGGAGGAG	Oryctolagus cuniculus
1	1.00	1	1	0	-0.004	0	0.003	-0.001	t	34965	LASLRKAFADTVPVRPPSNY	Oryctolagus cuniculus
1	1.00	1	1	0	-0.004	0	0.003	-0.001	t	28075	IPSTAFTDIAWVRLPNHY	Oryctolagus cuniculus
1	1.00	1	1	0.03	0.03	0.03	0.027	0.03	t	51148	QIVGGVYLLP	Homo sapiens
1	1.00	1	1	0.02	0.016	0.008	0.017	0.02	t	140865	GYLAGKGVDAGKLGTVSYGE	Oryctolagus cuniculus
1	1.00	1	1	0.02	0.023	0.02	0.017	0.019	t	135569	KTNMK	Mus musculus
1	1.00	1	1	0	0	0	0	0	t	156864	EPNPTRL	Gallus gallus

1	1.00	1	1	0	0.003	-0.009	0	0	t	78627	DPDPPDANDPAPPNAN	Mus musculus
1	1.00	1	1	-0.01	-0.006	0.001	-0.003	-0.009	t	122071	AALGSAI	Homo sapiens
1	1.00	1	1	0.01	-0.001	0.012	0.01	0.008	t	40074	LTPANLIRGDNA	Oryctolagus cuniculus
1	1.00	1	1	0.01	0.013	0.01	0.01	0.008	t	156905	KPGDPRQ	Mus musculus
1	1.00	1	1	-0.01	-0.01	-0.01	-0.006	-0.01	t	103830	CHAVKVGNPSC	Homo sapiens
1	1.00	1	1	-0.02	-0.02	-0.02	-0.02	-0.02	t	9132	DLIPLLR	Oryctolagus cuniculus
1	1.00	1	1	0.02	0.02	0.009	0.019	0.02	t	54095	RHRQPRGWEQ	Homo sapiens
1	1.00	1	1	0.07	0.07	0.07	0.07	0.07	t	118392	GVGGPSQQ	Homo sapiens
1	1.00	1	1	0.05	0.05	0.05	0.05	0.05	t	112090	EDLAVYF	Homo sapiens
1	1.00	1	1	0.03	0.03	0.03	0.03	0.03	t	117341	PGSALLFDIPEF	Homo sapiens
1	1.00	1	1	-0.01	-0.01	-0.018	-0.01	-0.01	t	57767	SFFVKDWPER	Mus musculus
1	1.00	1	1	0.03	0.03	0.031	0.033	0.03	t	50989	QGYQEPPPPQAPYQGYQEPP	Homo sapiens
1	1.00	1	1	-0.01	-0.014	-0.019	-0.011	-0.01	t	51474	QLPNVGITQGIV	Oryctolagus cuniculus
1	1.00	1	1	0	0.002	-0.002	0	0	t	10714	DVRRIEGG	Anas platyrhynchos
1	1.00	1	1	0.04	0.04	0.031	0.038	0.04	t	135931	GDVEEAIERAVV	Homo sapiens

1	1.00	1	1	0.01	0.013	0.022	0.01	0.007	t	110761	AENPTNY	Mus musculus
1	1.00	1	1	-0.01	-0.008	-0.017	-0.007	-0.009	t	144594	ARGISRTKYW	Homo sapiens
1	1.00	1	1	0.03	0.028	0.014	0.023	0.029	t	136062	FGISETQAEAILE	Mus musculus
1	1.00	1	1	0	0.001	0	0	-0.002	t	56564	RYKQKIIAPAKQ	Oryctolagus cuniculus
1	1.00	1	1	0.01	0.007	0.007	0.01	0.01	t	97485	LVSEGFLKL	Oryctolagus cuniculus
1	1.00	1	1	0.01	0.01	0.003	0.01	0.009	t	158723	DTGCAIDISRQELRCG	Mus musculus
1	1.00	1	1	0.01	0.014	0.001	0.011	0.012	t	13735	EPQVYTLPPSR	Homo sapiens
1	1.00	1	1	-0.01	-0.006	-0.01	-0.003	-0.01	t	117298	GPSRRGPSLGASSH	Homo sapiens
1	1.00	1	1	0	0.003	-0.006	0	0	t	32617	KNTLQARQQT	Mus musculus
1	1.00	1	1	0.02	0.024	0.009	0.021	0.022	t	111663	QDKILTHYENDSTD	Homo sapiens
1	1.00	1	1	-0.01	-0.012	-0.02	-0.013	-0.01	t	59650	SMDVRRIE	Anas platyrhynchos
1	1.00	1	1	0.03	0.03	0.03	0.03	0.028	t	129543	PAAAAAPAKVEAKEE	Homo sapiens
1	1.00	1	1	0.01	0.01	0.008	0.009	0.01	t	22119	GRLDA	Homo sapiens
1	1.00	1	1	0.06	0.062	0.06	0.059	0.06	t	151945	KFSSLYISQFI	Homo sapiens

1	1.00	1	1	-0.01	-0.012	-0.01	-0.004	-0.01	t	138190	HNAKTKL	Homo sapiens
1	1.00	1	1	0	0	-0.005	0.002	0	t	118217	VTKYITKG WKEVH	Homo sapiens
1	1.00	1	1	-0.03	-0.03	-0.034	-0.031	-0.03	t	106523	HWHHTIL	Mus musculu s
1	1.00	1	1	0	0	-0.006	-0.006	-0.001	t	30661	KEVPALTA VETGAT	Homo sapiens
1	1.00	1	1	-0.04	-0.042	-0.038	-0.04	-0.042	t	120598	IARFKMFP EVKEKGM A	Oryctola gus cuniculu s
1	1.00	1	1	0	0	0.005	0.01	0.003	t	116024	VTLRI	Homo sapiens
1	1.00	1	1	-0.01	-0.017	-0.024	-0.01	-0.01	t	149725	GSEKEAPS KDFYLKN	Oryctola gus cuniculu s
1	1.00	1	1	0	-0.002	-0.012	-0.007	-0.001	t	102439	FQLKTADL PAGRDETT SFVL	Mus musculu s
1	1.00	1	1	0	-0.002	-0.012	-0.007	-0.001	t	102439	FQLKTADL PAGRDETT SFVL	Mus musculu s
1	1.00	1	1	-0.01	-0.01	-0.023	-0.007	-0.008	t	66934	TVAELQRL KMKVGKT REL	Homo sapiens
1	1.00	1	1	-0.01	-0.01	-0.023	-0.007	-0.008	t	66934	TVAELQRL KMKVGKT REL	Homo sapiens
1	1.00	1	1	0	0	0	0	0	t	60795	SRSERR	Homo sapiens
1	1.00	1	1	0.05	0.05	0.043	0.05	0.049	t	136590	RDYFEEYG KIDTIEIIT	Mus musculu s
1	1.00	1	1	0.01	0.008	0.01	0.009	0.009	t	38434	LPDKFNAY LANKWV	Mus musculu s
1	1.00	1	1	0.02	0.02	0.02	0.02	0.019	t	135837	DMYGDIRL TGLI	Mus musculu s

1	1.00	1	1	-0.01	-0.01	-0.008	0	-0.007	t	106114	SLNPERMFRKP	Homo sapiens
1	1.00	1	1	0.04	0.04	0.04	0.04	0.041	t	114764	DENPVVHFFK	Mus musculus
1	1.00	1	1	0.01	0.01	0.01	0.01	0.01	t	127585	ASFEPFSN	Homo sapiens
1	1.00	1	1	0.01	0.008	0.008	0.009	0.009	t	74794	YLLPAIVHI	Mus musculus
1	1.00	1	1	0	0	0	0.001	-0.002	t	103838	DAEFRHDSGYEV	Mus musculus
1	1.00	1	1	0	0	0	0.001	-0.002	t	70621	VQPDGGQPAVRNERA	Mus musculus
1	1.00	1	1	0	0	0	0.001	-0.002	t	62050	SVARTRGLYDDG	Mus musculus
1	1.00	1	1	0	0	0	0.001	-0.002	t	47670	PGGRERGIYHIE	Mus musculus
1	1.00	1	1	0	0	0	0.001	-0.002	t	48395	PLLGCIGSTCAEDGN	Mus musculus
1	1.00	1	1	0	0	0	0.001	-0.002	t	53194	RARGIYETETRA	Mus musculus
1	1.00	1	1	0	0.003	0	0.001	-0.002	t	21838	GPWSPIDIHHLS	Mus musculus
1	1.00	1	1	0	0	0	0.001	-0.002	t	21838	GPWSPIDIHHLS	Mus musculus
1	1.00	1	1	0	0	0	0.001	-0.002	t	1448	AGAPDRARGQYP	Mus musculus
1	1.00	1	1	-0.03	-0.03	-0.03	-0.026	-0.03	t	115658	VGWYRPPFSRVV	Homo sapiens
1	1.00	1	1	-0.04	-0.04	-0.05	-0.04	-0.04	t	13589	ENSGCFRH	Mus musculus
1	1.00	1	1	0.03	0.03	0.03	0.03	0.03	t	99293	GQEEENEGGNIFSGF	Homo sapiens
1	1.00	1	1	-0.01	-0.008	-0.025	-0.011	-0.01	t	45589	NQMHMLSNKS	Homo sapiens

1	1.00	1	1	0.05	0.05	0.052	0.053	0.051	t	99007	SLQPED	Homo sapiens
1	1.00	1	1	0	0.004	-0.003	-0.003	-0.003	t	115403	NQELAYFY PELFRQFY QLDA	Homo sapiens
1	1.00	1	1	0.01	0.012	0.006	0.013	0.008	t	3676	APPGSPAP PPPEHRG GPEEF	Homo sapiens
1	1.00	1	1	-0.01	-0.012	-0.006	-0.011	-0.012	t	48359	PLIHFGND	Mus musculu s
1	1.00	1	1	0	0.008	-0.002	0	-0.002	t	146808	CTPKSDEF NPCEDIM GYKFL	Gallus gallus
1	1.00	1	1	0.05	0.05	0.05	0.052	0.052	t	27555	IMSGEKPT VEDVVN	Homo sapiens
1	1.00	1	1	-0.02	-0.02	-0.019	-0.02	-0.022	t	20523	GKCGVSIP YK	Homo sapiens
1	1.00	1	1	0.01	0.01	0.01	0.014	0.01	t	135930	GDKVSFFC KNKEKKC	Homo sapiens
1	1.00	1	1	0.02	0.024	0.02	0.021	0.02	t	21466	GNLLFT	Homo sapiens
1	1.00	1	1	-0.03	-0.03	-0.041	-0.03	-0.03	t	74478	YKLTRPYM ARC	Mus musculu s
1	1.00	1	1	0.01	0.008	0.015	0.009	0.009	t	53894	RGKDLPVL DQLTD	Mus musculu s
1	1.00	1	1	-0.01	-0.01	-0.01	-0.01	-0.012	t	68184	VEFTKDTQ TGMSGQT	Mus musculu s
1	1.00	1	1	-0.02	-0.02	-0.017	-0.02	-0.022	t	14204	ESLNTGW LAGLP	Rattus norvegic us
1	1.00	1	1	0.02	0.02	0.023	0.02	0.02	t	51454	QLNPKDN DVEL	Mus musculu s
1	1.00	1	1	0.02	0.02	0.023	0.02	0.02	t	51454	QLNPKDN DVEL	Homo sapiens

1	1.00	1	1	-0.02	-0.022	-0.011	-0.013	-0.02	t	112423	YYCARMR	Homo sapiens
1	1.00	1	1	0.01	0.008	-0.014	0.013	0.016	t	20521	GKAVTKEQL	Mus musculus
1	1.00	1	1	0	0.002	0.007	0.007	-0.001	t	65050	TLRVYK	Homo sapiens
1	1.00	1	1	-0.01	-0.008	-0.01	-0.013	-0.012	t	25445	IAT	Homo sapiens
1	1.00	1	1	0.04	0.04	0.04	0.04	0.04	t	149078	NITQEAF	Sus scrofa
1	1.00	1	1	0.04	0.037	0.033	0.037	0.04	t	57504	SEKSEEIN	Mus musculus
1	1.00	1	1	-0.01	-0.01	-0.01	-0.01	-0.013	t	51853	QPHGGGW	Mus musculus
1	1.00	1	1	-0.01	-0.01	-0.01	-0.01	-0.01	t	31667	KKPTFK	Mus musculus
1	1.00	1	1	0	-0.004	-0.006	-0.001	0	t	21911	GQLQAVNPNAGNRGQ	Oryctolagus cuniculus
1	1.00	1	1	0.01	0.01	0.01	0.01	0.01	t	99255	FSEVIF	Oryctolagus cuniculus
1	1.00	1	1	0.06	0.056	0.052	0.063	0.062	t	26235	IGGSQAQTAS	Oryctolagus cuniculus
1	1.00	1	1	-0.02	-0.015	-0.018	-0.017	-0.02	t	55625	RRKAMLQDMA	Homo sapiens
1	1.00	1	1	-0.04	-0.038	-0.042	-0.039	-0.04	t	71192	VSVK	Homo sapiens
1	1.00	1	1	0.04	0.04	0.04	0.04	0.039	t	75847	YSPELE	Mus musculus
1	1.00	1	1	0.02	0.017	0.011	0.02	0.02	t	113861	SISTTNFTNP	Oryctolagus cuniculus

1	1.00	1	1	0	0	-0.011	0	0.002	t	17467	FQDMLNIS	Mus musculus
1	1.00	1	1	-0.01	-0.01	-0.019	-0.01	-0.01	t	45252	NNSTNSGI	Mus musculus
1	1.00	1	1	-0.01	-0.01	-0.01	-0.004	-0.01	t	119278	MSKTEWNASQLPRLGGKEDR	Rattus norvegicus
1	1.00	1	1	0.01	0.01	0.01	0.01	0.01	t	112398	WFAYWGQ	Homo sapiens
1	1.00	1	1	0.07	0.073	0.065	0.07	0.07	t	52313	QSESVEFTKDTQTGMS	Mus musculus
1	1.00	1	1	-0.01	-0.007	-0.017	-0.011	-0.01	t	67583	VAFGNPGDTPVTGDW	Capra hircus
1	1.00	1	1	-0.01	-0.017	-0.017	-0.011	-0.01	t	67583	VAFGNPGDTPVTGDW	Oncorhynchus tshawytscha
1	1.00	1	1	0	-0.002	-0.001	-0.001	-0.001	t	72251	WAPSV	Mus musculus
1	1.00	1	1	0.03	0.026	0.028	0.03	0.03	t	142146	EQKLISEEDL	Mus musculus
1	1.00	1	1	-0.02	-0.022	-0.018	-0.021	-0.02	t	55272	RPPRYMVQS	Mus musculus
1	1.00	1	1	-0.02	-0.02	-0.02	-0.02	-0.019	t	39167	LRPTFDTRLM	Mus musculus
1	1.00	1	1	0.02	0.016	0.012	0.02	0.02	t	52602	QTQSQVQDNVKQQQD	Bos taurus
1	1.00	1	1	0.01	0.01	0.01	0.01	0.01	t	119480	KQQKTENGAGDQ	Homo sapiens
1	1.00	1	1	-0.03	-0.026	-0.026	-0.023	-0.03	t	144772	SAHTSHLVGS	Homo sapiens

1	1.00	1	1	-0.03	-0.028	-0.023	-0.023	-0.029	t	122127	KRSRLDV	Homo sapiens
1	1.00	1	1	0.05	0.062	0.044	0.05	0.05	t	66733	TTNDAEAS	Mus musculus
1	1.00	1	1	0	-0.002	-0.001	-0.001	-0.001	t	65000	TLPERVRPPDDWAT	Mus musculus
1	1.00	1	1	0.04	0.04	0.04	0.04	0.04	t	149435	QPQYSQPQQP	Mus musculus
1	1.00	1	1	0.04	0.04	0.04	0.04	0.04	t	149435	QPQYSQPQQP	Homo sapiens
1	1.00	1	1	-0.05	-0.049	-0.055	-0.05	-0.05	t	50494	QDMRNTIMASKTVG	Myodes glareolus
1	1.00	1	1	0.02	0.018	0.027	0.023	0.02	t	97205	ANYTTAVD	Mus musculus
1	1.00	1	1	0	-0.001	0.005	-0.003	0	t	73991	YGFQYS	Homo sapiens
1	1.00	1	1	0.02	0.018	0.02	0.012	0.017	t	144385	KPLEEVLNL	Mus musculus
1	1.00	1	1	0.03	0.026	0.028	0.03	0.03	t	55965	RSSRPLSEENGEREYN	Mesocricetus auratus
1	1.00	1	1	0.01	0.012	0.006	0.011	0.01	t	56200	RTTSGLT	Homo sapiens
1	1.00	1	1	0.01	0.013	0.011	0.01	0.009	t	75371	YPNQVYYRPVDQYSN	Mus musculus
1	1.00	1	1	0.01	0.012	0.01	0.017	0.01	t	108082	QKTTTRPYPNLQIDP	Homo sapiens
1	1.00	1	1	-0.02	-0.017	-0.021	-0.02	-0.022	t	22027	GREVKAETRLNP	Mus musculus

1	1.00	1	1	0	0	0.005	0	-0.002	t	72321	WDMMMNWSP	Homo sapiens
1	1.00	1	1	-0.04	-0.038	-0.049	-0.033	-0.037	t	63098	TCRRMLAFLKDKENR	Homo sapiens
1	1.00	1	1	0.05	0.05	0.05	0.05	0.049	t	119574	EWESNGQP	Homo sapiens
1	1.00	1	1	-0.02	-0.015	-0.02	-0.019	-0.02	t	97018	TVTFKATAAT	Homo sapiens(human)
1	1.00	1	1	0	0.002	-0.009	0.001	0.001	t	64534	TKDASIDYHE	Homo sapiens
1	1.00	1	1	0	0.002	-0.009	0.001	0.001	t	64534	TKDASIDYHE	Homo sapiens
1	1.00	1	1	0	0	0	0	0	t	108632	SKEENTPEVKA	Canis lupus
1	1.00	1	1	0	0	0	0	0	t	118258	AKSPEKAK	Homo sapiens
1	1.00	1	1	0.01	0.01	0.017	0.01	0.009	t	105890	IVYVGDGVC	Mus musculus
1	1.00	1	1	-0.01	-0.007	-0.011	-0.01	-0.012	t	53634	RESQAYYQR	Mus musculus
1	1.00	1	1	0	0.006	0	0	0	t	123833	VSHVDFMVY	Ovis aries
1	1.00	1	1	0.02	0.027	0.02	0.02	0.02	t	39091	LRGDLQVLAQ	Cavia porcellus
1	1.00	1	1	0.02	0.027	0.02	0.02	0.02	t	39091	LRGDLQVLAQ	Cavia porcellus
1	1.00	1	1	0.02	0.02	0.018	0.02	0.02	t	21422	GNFERISGDLKTQID	Homo sapiens

1	1.00	1	1	0	0	0	0.003	-0.001	t	123540	NIFEYEVDAEPLRPCELER	Mus musculus
1	1.00	1	1	0	0	0	0.003	-0.001	t	105207	GAIIGLMVGGVVIA	Mus musculus
1	1.00	1	1	0.02	0.02	0.008	0.02	0.022	t	150200	FGEHKQEKDLEYGAC	Mus musculus
1	1.00	1	1	-0.02	-0.018	-0.02	-0.017	-0.02	t	96487	KPTAPTKPTY	Homo sapiens(human)
1	1.00	1	1	0.01	0.014	0.016	0.013	0.01	t	1254	AFAQAPTHQLSL	Homo sapiens
1	1.00	1	1	0.02	0.02	0.015	0.02	0.018	t	151676	KVDFPQDQLATLTGRI	Mus musculus
1	1.00	1	1	-0.03	-0.03	-0.035	-0.03	-0.032	t	24970	HTRVTGGVQG	Mus musculus
1	1.00	1	1	0.06	0.063	0.05	0.06	0.06	t	37377	LLGYKKGEGNTCVENNNPTC	Mus musculus
1	1.00	1	1	0.06	0.06	0.05	0.06	0.06	t	37377	LLGYKKGEGNTCVENNNPTC	Mus musculus
1	1.00	1	1	0.06	0.063	0.05	0.06	0.06	t	37377	LLGYKKGEGNTCVENNNPTC	Mus musculus
1	1.00	1	1	0	-0.002	0	0.003	-0.001	t	48618	PNDPNRNVD	Mus musculus
1	1.00	1	1	0	-0.002	0	0.003	-0.001	t	6402	CHPSDGKCN	Mus musculus
1	1.00	1	1	0.04	0.04	0.036	0.04	0.04	t	80440	AQQPSTATSVDAP	Homo sapiens
1	1.00	1	1	0.04	0.04	0.04	0.044	0.04	t	115602	EEAAMELKVEDP	Homo sapiens
1	1.00	1	1	-0.02	-0.022	-0.021	-0.017	-0.02	t	106535	IFPSFLRPDPPL	Mus musculus

1	1.00	1	1	0.03	0.03	0.023	0.03	0.03	t	140681	GPEYWEQQTRIAKEWEQ	Rattus norvegicus
1	1.00	1	1	0.04	0.042	0.041	0.043	0.04	t	133822	ERPLKQIAFNGGLEP	Homo sapiens
1	1.00	1	1	-0.02	-0.016	-0.025	-0.019	-0.02	t	6365	CGWAGWLLSPRG	Homo sapiens
1	1.00	1	1	0.05	0.047	0.041	0.047	0.05	t	78177	HVTDLDKEFL	Mus musculus
1	1.00	1	1	0	0.002	-0.004	-0.003	0	t	52082	QQPFP	Homo sapiens
1	1.00	1	1	0	-0.002	-0.005	-0.001	0	t	17128	FNASDSVGQQIKVIP	Mus musculus
1	1.00	1	1	0.01	0.01	0.008	0.007	0.008	t	29810	KAGNEGLEWVEL	Homo sapiens
1	1.00	1	1	0.03	0.028	0.037	0.031	0.03	t	78971	FGANST	Mus musculus
1	1.00	1	1	-0.02	-0.02	-0.03	-0.022	-0.02	t	12388	EIAKTTLLK	Homo sapiens
1	1.00	1	1	0.06	0.065	0.061	0.06	0.06	t	29899	KANGNGSGDNGDTTW	Homo sapiens
1	1.00	1	1	0	0	0	0.002	0	t	28909	ITGHRMAWDM	Homo sapiens
1	1.00	1	1	-0.01	-0.01	-0.01	-0.01	-0.013	t	106941	TVKQLYTLYPAA	Mus musculus
1	1.00	1	1	0.06	0.06	0.06	0.06	0.059	t	78872	VIPDREVLYQEFDEMEECAS	Homo sapiens
1	1.00	1	1	0.03	0.026	0.028	0.029	0.03	t	57281	SDSGSDSGASST	Oryctolagus cuniculus
1	1.00	1	1	0	0	0	0	-0.001	t	130637	WSSCVFSVSYCL	Mus musculus

1	1.00	1	1	-0.02	-0.018	-0.025	-0.019	-0.02	t	65414	TNRRPQDVKFPGGGQ	Homo sapiens
1	1.00	1	1	0	0.004	0	0.003	0	t	118434	ISKGANPVEIRRG	Homo sapiens
1	1.00	1	1	0.11	0.108	0.096	0.103	0.112	t	125871	EEEEEEEEEE	Mus musculus
1	1.00	1	1	0.02	0.02	0.019	0.024	0.02	t	127987	FYQALQNSLGGEDSDARVEA	Homo sapiens
1	1.00	1	1	0.01	0.014	0	0.007	0.01	t	11731	EEKPAVLGHDEAAYS	Homo sapiens
1	1.00	1	1	0.02	0.02	0.02	0.02	0.018	t	52086	QQPFPQQPQQ	Mus musculus
1	1.00	1	1	-0.04	-0.04	-0.04	-0.04	-0.042	t	26372	IHFG	Mus musculus
1	1.00	1	1	-0.01	-0.006	-0.008	-0.009	-0.011	t	4885	ATALDTSNKFADFLQI	Ovis aries
1	1.00	1	1	0	0	0	-0.003	-0.004	t	62003	STWHVLYSPNVSVPSSSTP	Homo sapiens
1	1.00	1	1	0	0	0	-0.002	0	t	6059	CCDLAP	Homo sapiens
1	1.00	1	1	0.06	0.06	0.06	0.06	0.06	t	137945	PGTEYTVE	Homo sapiens
1	1.00	1	1	0.01	0.007	0.007	0.01	0.008	t	7216	CTTFDDVQAPNYTQHTSSMR	Oryctolagus cuniculus
1	1.00	1	1	0	0.004	0	0.001	0.002	t	67181	TVTPID	Homo sapiens
1	1.00	1	1	0.02	0.02	0.02	0.02	0.02	t	35973	LFSGDEDSDSSDGYP	Homo sapiens
1	1.00	1	1	0.01	0.01	0.006	0.007	0.008	t	195	AAHASARQQWELQGD	Homo sapiens

1	1.00	1	1	0	0	0	0	-0.001	t	49505	PSSKRFQP FQQFGRD	Homo sapiens
1	1.00	1	1	-0.02	-0.019	-0.031	-0.02	-0.018	t	56089	RTIVCGLFP TQIQV	Myodes glareolu s
1	1.00	1	1	0.03	0.032	0.029	0.031	0.032	t	44778	NLLFTG	Homo sapiens
1	1.00	1	1	-0.02	-0.019	-0.02	-0.017	-0.02	t	78243	PLFRPS	Equus caballus
1	1.00	1	1	0.01	0.01	0	0.01	0.01	t	25291	IAGKTSVL RAIP	Homo sapiens
1	1.00	1	1	0.01	0.012	0.003	0.013	0.01	t	73298	WYTTVPK YVATQGYL	Homo sapiens
1	1.00	1	1	0.01	0.013	0.005	0.01	0.013	t	123479	LYEPPASLP	Mus musculu s
1	1.00	1	1	-0.03	-0.034	-0.033	-0.031	-0.031	t	67169	TVSSQSFR RL	Oryctola gus cuniculu s
1	1.00	1	1	0	0	-0.009	0.005	0.003	t	110828	EIDGPAGQ AEPDRAH YNI	Mus musculu s
1	1.00	1	1	-0.01	-0.006	-0.016	-0.003	-0.009	t	134804	TIFILFCCSK EKRKKKQA AT	Homo sapiens
1	1.00	1	1	-0.01	-0.01	-0.01	-0.01	-0.01	t	9591	DPAKAAF D	Mus musculu s
1	1.00	1	1	0	0	0	0	0	t	112055	AWFAYW G	Homo sapiens
1	1.00	1	1	0.01	0.014	0.01	0.011	0.01	t	137858	FTPGTEYT	Homo sapiens
1	1.00	1	1	0.01	0.012	0.005	0.011	0.01	t	142047	TYFKGVST ELKDKK	Homo sapiens
1	1.00	1	1	-0.01	-0.014	-0.012	-0.007	-0.01	t	95973	VNPLGFFK KEAL	Oryctola gus cuniculu s

1	1.00	1	1	0	0	0.005	0	0.001	t	115047	ELQFPFLQD	Mus musculus
1	1.00	1	1	-0.01	-0.008	-0.022	-0.009	-0.01	t	35982	LFSLMPDVAHSLA	Homo sapiens
1	1.00	1	1	0.03	0.03	0.022	0.033	0.032	t	42308	MPPYPTGGPPPV	Homo sapiens
1	1.00	1	1	0.01	0.008	-0.001	0.009	0.011	t	140573	LCRSACVIYIKYSSA	Mus musculus
1	1.00	1	1	0	-0.003	-0.011	0	0	t	58511	SIFYTYGTAPARISVPYVGI	Oryctolagus cuniculus
1	1.00	1	1	0.01	0.008	0.004	0.013	0.01	t	32817	KPNYAPANCPKS	Mus musculus
1	1.00	1	1	0.02	0.02	0.02	0.02	0.02	t	78506	RSDVVGLQSLFCCEI	Mus musculus
1	1.00	1	1	0.02	0.02	0.016	0.017	0.02	t	21653	GPGVAGSG	Homo sapiens
1	1.00	1	1	0.04	0.04	0.033	0.04	0.04	t	137859	FTPGTEYTVEVR	Oryctolagus cuniculus
1	1.00	1	1	0.01	0.013	0.004	0.01	0.012	t	66062	TRLNPDLQPTE	Mus musculus
1	1.00	1	1	0	0	-0.006	0	0	t	83209	HQVQEGLNIEKY	Homo sapiens
1	1.00	1	1	0	0.002	0	0.007	0.002	t	11571	EEEDDDMGFGLFD	Homo sapiens
1	1.00	1	1	-0.02	-0.02	-0.02	-0.02	-0.022	t	10892	DYHRVMY	Oryctolagus cuniculus

1	1.00	1	1	0	-0.002	-0.005	-0.001	0	t	14398	ETHPLYQS	Anas platyrhynchos
1	1.00	1	1	0.01	0.01	0.003	0.01	0.01	t	96171	DEEDKEEDEE	Homo sapiens
1	1.00	1	1	0.02	0.02	0.012	0.02	0.02	t	106913	TLPPITADFDCS	Homo sapiens
1	1.00	1	1	0.01	0.012	0.01	0.011	0.009	t	58076	SGHRMAWDM	Homo sapiens
1	1.00	1	1	-0.01	-0.008	-0.017	-0.003	-0.009	t	139496	ESSRDGSRHPRSHD	Homo sapiens
1	1.00	1	1	0.02	0.02	0.009	0.017	0.018	t	38582	LPLPLLQSWM	Homo sapiens
1	1.00	1	1	0.05	0.048	0.043	0.047	0.048	t	48754	PPAYEKLSAEQSPPP	Mus musculus
1	1.00	1	1	0.03	0.019	0.028	0.03	0.03	t	27048	ILFPAD	Marmota monax
1	1.00	1	1	0.03	0.03	0.03	0.03	0.03	t	108420	HELGDIAIA	Homo sapiens
1	1.00	1	1	0.03	0.03	0.03	0.03	0.03	t	105901	KGTAPFEI	Oryctolagus cuniculus
1	1.00	1	1	0.02	0.02	0.02	0.02	0.02	t	10782	DVVSTL	Homo sapiens
1	1.00	1	1	-0.04	-0.04	-0.051	-0.04	-0.04	t	21026	GLLRYHTYARFG	Homo sapiens
1	1.00	1	1	-0.02	-0.022	-0.015	-0.017	-0.021	t	141726	PTAPPLLPHS	Rattus norvegicus
1	1.00	1	1	0	0	-0.01	-0.006	-0.002	t	60327	SPVTKRASLKIDSKK	Homo sapiens
1	1.00	1	1	-0.02	-0.014	-0.013	-0.017	-0.02	t	78220	NKTWYL	Equus caballus
1	1.00	1	1	0.01	0.01	0.008	0.01	0.009	t	97282	ESNNL	Oryctolagus cuniculus

1	1.00	1	1	0.01	0.012	0.01	0.011	0.01	t	51014	QHLPYIEQG	Homo sapiens
1	1.00	1	1	-0.02	-0.026	-0.029	-0.02	-0.021	t	113862	SITSGPMARP	Oryctolagus cuniculus
1	1.00	1	1	0.03	0.03	0.03	0.029	0.029	t	9585	DPAF	Mus musculus
1	1.00	1	1	0.01	0.009	0.005	0.007	0.01	t	53464	RECCNEIRDVK	Homo sapiens
1	1.00	1	1	0.03	0.03	0.03	0.03	0.029	t	112309	SGFSLTT	Homo sapiens
1	1.00	1	1	-0.01	-0.01	-0.01	-0.01	-0.01	t	79253	IARLIS	Mus musculus
1	1.00	1	1	0.03	0.032	0.023	0.037	0.031	t	117015	KEQFLDGDGWTSRWIESK	Homo sapiens
1	1.00	1	1	-0.02	-0.027	-0.028	-0.02	-0.018	t	110913	KDIKANPENYFT	Macaca
1	1.00	1	1	0.02	0.023	0.022	0.02	0.018	t	47338	PELAPEDPEDSALL	Mus musculus
1	1.00	1	1	0.02	0.02	0.02	0.02	0.02	t	127718	KSTPQFT	Homo sapiens
1	1.00	1	1	0.02	0.02	0.016	0.02	0.02	t	51939	QPQQTFPQPQ	Mus musculus
1	1.00	1	1	0.02	0.02	0.016	0.02	0.02	t	51939	QPQQTFPQPQ	Mus musculus
1	1.00	1	1	0	0	0	0	0	t	78207	MLPLKS	Equus caballus
1	1.00	1	1	-0.03	-0.03	-0.027	-0.03	-0.03	t	73739	YEPGRSLW	Mus musculus
1	1.00	1	1	-0.02	-0.02	-0.025	-0.02	-0.02	t	53763	RFQEALKKSLKKP	Homo sapiens
1	1.00	1	1	-0.01	-0.01	-0.012	-0.01	-0.01	t	49748	PTPSAPARGE	Homo sapiens

1	1.00	1	1	-0.03	-0.03	-0.041	-0.027	-0.03	t	74503	YKRRMADAVSRKKM	Homo sapiens
1	1.00	1	1	0	-0.002	-0.01	0.003	0.001	t	35956	LFREDGSACR	Mus musculus
1	1.00	1	1	-0.02	-0.019	-0.017	-0.021	-0.022	t	54763	RLSGVFLIELDKLII	Oryctolagus cuniculus
1	1.00	1	1	-0.02	-0.024	-0.017	-0.021	-0.022	t	54763	RLSGVFLIELDKLII	Oryctolagus cuniculus
1	1.00	1	1	0	0	0	0	0	t	9895	DQRPYCWHYA	Homo sapiens
1	1.00	1	1	-0.04	-0.028	-0.038	-0.037	-0.04	t	55760	RRVGLSNP	Anas platyrhynchos
1	1.00	1	1	0.03	0.032	0.03	0.032	0.032	t	156603	AEVLRDGDCP	Homo sapiens
1	1.00	1	1	0	0	-0.003	0	-0.002	t	91996	SSLDRRYLDGLEVC	Oryctolagus cuniculus
1	1.00	1	1	0.02	0.018	0.02	0.02	0.016	t	34068	KVKILPKDRWTQHTTTGG	Oryctolagus cuniculus
1	1.00	1	1	-0.03	-0.03	-0.025	-0.023	-0.03	t	140672	EQIYRVDLRTLR	Rattus norvegicus
1	1.00	1	1	-0.01	-0.01	-0.01	-0.01	-0.012	t	78560	VTSRGNV	Mus musculus
1	1.00	1	1	0.02	0.02	0.02	0.02	0.02	t	78396	GLANDV	Mus musculus
1	1.00	1	1	0.02	0.015	0.022	0.018	0.02	t	45462	NPQVNLVD	Bos taurus
1	1.00	1	1	0.02	0.016	0.018	0.019	0.02	t	69201	VKDYTEKLHEGVAK	Oryctolagus cuniculus
1	1.00	1	1	-0.05	-0.051	-0.045	-0.049	-0.05	t	50067	PYCWHYAP	Homo sapiens

1	1.00	1	1	0.01	0.01	0.01	0.01	0.011	t	115234	EQLTKCEV FR	Homo sapiens
1	1.00	1	1	0	0.005	0.008	0	-0.002	t	50180	PYVEPTAP QVL	Homo sapiens
1	1.00	1	1	-0.03	-0.026	-0.038	-0.03	-0.03	t	140594	RELVLAGTI	Cavia porcellu s
1	1.00	1	1	-0.02	-0.02	-0.02	-0.02	-0.018	t	135457	FGF	Mus musculu s
1	1.00	1	1	0	0	-0.012	0.002	0.002	t	96806	QTELARVQ KA	Homo sapiens(human)
1	1.00	1	1	-0.04	-0.035	-0.038	-0.04	-0.042	t	132861	LKRGIEKA V	Mus musculu s
1	1.00	1	1	-0.02	-0.02	-0.02	-0.017	-0.02	t	27290	ILRKSRRF	Homo sapiens
1	1.00	1	1	0	-0.003	-0.005	0	0	t	61818	STPGIVI	Oryctola gus cuniculu s
1	1.00	1	1	-0.02	-0.016	-0.026	-0.013	-0.019	t	134804	TIFILFCCSK EKRKKKQA AT	Homo sapiens
1	1.00	1	1	0	0	0	0.004	0	t	38693	LPRSNTSS GAIPPAD	Homo sapiens
1	1.00	1	1	0.02	0.02	0.02	0.02	0.02	t	79821	FEGKPALE LIRM	Mus musculu s
1	1.00	1	1	0.02	0.022	0.013	0.027	0.022	t	130448	AYAKDVKF GADA	Homo sapiens
1	1.00	1	1	0.01	0.006	-0.007	0.003	0.011	t	117451	DDGFFYIP KD	Oryctola gus cuniculu s
1	1.00	1	1	-0.01	-0.008	-0.012	-0.007	-0.011	t	27961	IPNKKPGK KTTT	Homo sapiens

1	1.00	1	1	-0.02	-0.016	-0.005	-0.013	-0.021	t	104286	SRL	Homo sapiens
1	1.00	1	1	-0.01	-0.013	-0.015	-0.01	-0.01	t	58417	SHSQWNKPSKPKTNMK	Oryctolagus cuniculus
1	1.00	1	1	0.02	0.02	0.02	0.02	0.02	t	144676	GTHPSLQFT	Mus musculus
1	1.00	1	1	0.02	0.02	0.02	0.02	0.02	t	144676	GTHPSLQFT	Mus musculus
1	1.00	1	1	0	0	0	0	0	t	153381	ETPLTIAGLKLK	Mus musculus
1	1.00	1	1	-0.01	-0.007	-0.019	-0.01	-0.008	t	63730	TFPQPQQTYP	Mus musculus
1	1.00	1	1	0.01	0.01	0.003	0.01	0.009	t	159173	TTESGKLITDWCCRSC	Mus musculus
1	1.00	1	1	0.04	0.04	0.039	0.04	0.04	t	132644	SAKEK	Mus musculus
1	1.00	1	1	0.03	0.03	0.03	0.03	0.029	t	119518	RKTALGTRQ	Homo sapiens
1	1.00	1	1	0.05	0.05	0.05	0.05	0.052	t	134673	FVAQNKIDSLNLD	Homo sapiens
1	1.00	1	1	-0.02	-0.02	-0.026	-0.02	-0.02	t	48996	PQFKPQ	Mus musculus
1	1.00	1	1	0.02	0.018	0	0.013	0.02	t	128079	PSPDITSTTKTRNFDLVNCN	Mus musculus
1	1.00	1	1	-0.03	-0.022	-0.03	-0.03	-0.032	t	156929	PGPRFF	Gallus gallus
1	1.00	1	1	-0.02	-0.02	-0.02	-0.016	-0.02	t	135926	GADIKKTMEGPV	Homo sapiens
1	1.00	1	1	0.02	0.02	0.02	0.02	0.02	t	100580	TVDDFGVLAVRVVN	Homo sapiens

1	1.00	1	1	0.02	0.02	0.016	0.02	0.02	t	51940	QPQQTFPQQP	Mus musculus
1	1.00	1	1	0.02	0.02	0.016	0.02	0.02	t	51940	QPQQTFPQQP	Mus musculus
1	1.00	1	1	0.03	0.034	0.02	0.027	0.03	t	31406	KIQRDEDSYGRDPYS	Homo sapiens
1	1.00	1	1	0.03	0.033	0.031	0.03	0.031	t	127958	EHP	Mus musculus
1	1.00	1	1	0.03	0.03	0.031	0.03	0.031	t	127958	EHP	Oryctolagus cuniculus
1	1.00	1	1	-0.01	-0.013	-0.02	-0.01	-0.01	t	140835	DAVKGYLAGKGVDAGKLGTV	Oryctolagus cuniculus
1	1.00	1	1	-0.01	-0.006	-0.008	-0.007	-0.01	t	120904	RALKRRNARTKAR	Homo sapiens
1	1.00	1	1	0.01	0.008	0.009	0.009	0.01	t	21763	GPRLQPY	Mus musculus
1	1.00	1	1	0	0	-0.01	0	0	t	54909	RMKDTLRITY	Mus musculus
1	1.00	1	1	-0.03	-0.034	-0.03	-0.033	-0.03	t	131316	TLMVMVSK	Oryctolagus cuniculus
1	1.00	1	1	0	0	-0.005	0	0	t	153483	TRDGKLPTTQLRR	Mus musculus
1	1.00	1	1	0.03	0.033	0.023	0.03	0.028	t	8567	DHSDLVAEKQRLEDLGQKFE	Mus musculus
1	1.00	1	1	0.02	0.02	0.01	0.021	0.019	t	135390	YYLVSLGPDLIKDSQ	Sus scrofa

1	1.00	1	1	-0.01	-0.01	-0.017	-0.01	-0.009	t	97628	SAAANYTT	Mus musculus
1	1.00	1	1	0	0	0.002	0.001	-0.002	t	153251	AKAFSYWTPFDGKSLYSST	Mus musculus
1	1.00	1	1	0.01	0.014	0.01	0.013	0.012	t	134116	KGDVTAQIALQ	Homo sapiens
1	1.00	1	1	0.01	0.008	-0.002	0.009	0.01	t	66025	TRFVNASPTGYD	Mus musculus
1	1.00	1	1	0.01	0.014	-0.001	0.007	0.01	t	43191	NAKATYEAALKQYEADLAA	Homo sapiens
1	1.00	1	1	-0.02	-0.026	-0.029	-0.02	-0.02	t	68855	VHHANENIFYCPIA	Oryctolagus cuniculus
1	1.00	1	1	0.01	0.013	0.01	0.01	0.012	t	137401	FEDVW	Mus musculus
1	1.00	1	1	0	0	0	0	-0.002	t	117512	GRGRGRGRGGPRR	Homo sapiens
1	1.00	1	1	0	-0.004	0	-0.001	-0.003	t	61589	STEQNVPDPQVG	Oryctolagus cuniculus
1	1.00	1	1	0	-0.003	0	-0.001	-0.003	t	7491	DAEFRHDSGYEVHHQK	Oryctolagus cuniculus
1	1.00	1	1	0.01	0.008	0.006	0.007	0.009	t	79624	PEEQVPQLIPPPIIPRAG	Mus musculus
1	1.00	1	1	-0.02	-0.02	-0.021	-0.019	-0.021	t	43825	NFELCDNPFFAVSKPMG	Homo sapiens
1	1.00	1	1	0.01	0.012	0.003	0.013	0.01	t	133787	CQALVRMLAKKPGWK	Homo sapiens

1	1.00	1	1	-0.02	-0.023	-0.025	-0.02	-0.021	t	60992	SSFFSYGEIP	Oryctolagus cuniculus
1	1.00	1	1	-0.05	-0.046	-0.06	-0.049	-0.051	t	111233	EEASVTVVEGQVDYY	Homo sapiens
1	1.00	1	1	0	0.002	0.005	0.007	-0.002	t	122930	TWDPAQPPRD	Homo sapiens
1	1.00	1	1	-0.05	-0.05	-0.05	-0.05	-0.052	t	117557	KVQLLRNWH	Homo sapiens
1	1.00	1	1	0	0	-0.005	-0.007	-0.002	t	142385	AKTAATNAEKKKTK	Homo sapiens
1	1.00	1	1	-0.02	-0.02	-0.027	-0.013	-0.018	t	127046	SAPATGGVKKPHRYRPGT	Mus musculus
1	1.00	1	1	-0.01	-0.012	-0.015	-0.007	-0.01	t	28721	ISSISART	Mus musculus
1	1.00	1	1	0.03	0.032	0.026	0.027	0.029	t	58038	SGFTPEFLEQAFQVD	Homo sapiens
1	1.00	1	1	0	-0.003	-0.005	0	0	t	79622	NYARTGGQVTAG	Oryctolagus cuniculus
1	1.00	1	1	0.02	0.006	0.003	0.013	0.021	t	115640	QKSQRSQDEN	Macaca mulatta
1	1.00	1	1	-0.01	-0.008	-0.003	-0.003	-0.01	t	108052	NWLSLITAAR	Homo sapiens
1	1.00	1	1	0	0	0.002	0.006	0	t	117331	MKLVRFLMKLSHETVTIELK	Mus musculus
1	1.00	1	1	0.02	0.026	0.023	0.02	0.02	t	141021	GHKEVTVVT	Ovis aries
1	1.00	1	1	0.01	0.007	0.01	0.01	0.009	t	131579	PDIITGFSS	Oryctolagus cuniculus

1	1.00	1	1	-0.02	-0.022	-0.018	-0.021	-0.02	t	22199	GRRVGLSN	Anas platyrhynchos
1	1.00	1	1	0.03	0.026	0.02	0.033	0.03	t	63736	TFQVEVPGSQHIDS	Oryctolagus cuniculus
1	1.00	1	1	0.01	0.012	0.003	0.007	0.008	t	53282	RCKANKIQDK	Homo sapiens
1	1.00	1	1	0	0	0	0	-0.002	t	145831	IPLGTRP	Mus musculus
1	1.00	1	1	0.01	0.012	0.005	0.011	0.01	t	50442	QDAYNAAGGHNAVFNFPPNG	Homo sapiens
1	1.00	1	1	-0.03	-0.03	-0.03	-0.03	-0.03	t	119478	KNDK	Homo sapiens
1	1.00	1	1	0.05	0.048	0.035	0.053	0.052	t	52357	QSKSTSAASTSDELSGSEGP	Mus musculus
1	1.00	1	1	-0.07	-0.068	-0.07	-0.063	-0.07	t	107858	GHAKSRPVRG	Homo sapiens
1	1.00	1	1	0.01	0.006	-0.007	0.007	0.013	t	117451	DDGFFYIPKD	Oryctolagus cuniculus
1	1.00	1	1	-0.01	-0.008	-0.017	-0.013	-0.011	t	28575	ISLIFIYETF	Homo sapiens
1	1.00	1	1	0.03	0.034	0.023	0.031	0.029	t	59016	SLAQVKYTSASSI	Homo sapiens
1	1.00	1	1	0.03	0.03	0.025	0.028	0.03	t	71786	VVPLATYSLTGLWS	Homo sapiens
1	1.00	1	1	0.01	0.01	0.008	0.01	0.009	t	32807	KPNLSSKRSELSQLS	Mus musculus
1	1.00	1	1	0.03	0.026	0.029	0.029	0.029	t	110862	GGPTLVSLHELPAETP	Oryctolagus cuniculus
1	1.00	1	1	0.01	0.012	0.01	0.017	0.01	t	108141	SPSDAKSHVRVADLI	Homo sapiens

1	1.00	1	1	0	0	0.005	0	-0.002	t	122745	EEKPTSAPS	Oryctolagus cuniculus
1	1.00	1	1	0.03	0.03	0.03	0.03	0.032	t	129695	QLYKQK	Homo sapiens
1	1.00	1	1	0.03	0.03	0.036	0.03	0.031	t	14992	EYEDLLKA	Mus musculus
1	1.00	1	1	-0.01	-0.01	-0.01	-0.01	-0.011	t	131903	GVMAARGQL	Homo sapiens
1	1.00	1	1	-0.01	-0.01	-0.007	-0.01	-0.013	t	114454	LIYIERGRGITGVLF	Homo sapiens
1	1.00	1	1	0	0.002	-0.017	0.001	0.002	t	151069	WTGMVDGWYGYHHQN	Homo sapiens
1	1.00	1	1	0.05	0.05	0.05	0.05	0.05	t	118806	GGPDGEPDVPPGAIEQGPAD	Homo sapiens
1	1.00	1	1	0.03	0.03	0.03	0.03	0.03	t	121843	SGLGLFPYSSFIWSC	Mus musculus
1	1.00	1	1	0.03	0.03	0.03	0.03	0.03	t	121843	SGLGLFPYSSFIWSC	Mus musculus
1	1.00	1	1	0.01	0.01	0.01	0.01	0.01	t	114920	PVCGTDGVTY	Homo sapiens
1	1.00	1	1	-0.01	-0.01	-0.02	-0.01	-0.01	t	140725	PDELHK	Mus musculus
1	1.00	1	1	-0.01	-0.006	-0.01	-0.013	-0.011	t	24812	HSMKEGIHAQ	Homo sapiens
1	1.00	1	1	0.06	0.062	0.061	0.061	0.061	t	11456	EDTVTP	Homo sapiens
1	1.00	1	1	0.02	0.017	0.02	0.02	0.02	t	42273	MPFVTHN	Oryctolagus cuniculus
1	1.00	1	1	0	0.003	-0.005	0	-0.002	t	126921	RERRGIALD	Mus musculus

1	1.00	1	1	0.01	0.011	0.01	0.01	0.009	t	99386	KLKGESG	Oryctolagus cuniculus
1	1.00	1	1	0.02	0.018	0.025	0.019	0.015	t	74762	YLLARGHDPAFVHMS	Mus musculus
1	1.00	1	1	-0.01	-0.008	-0.009	-0.007	-0.011	t	53995	RGTARTPPTDPKTHPHGPAD	Homo sapiens
1	1.00	1	1	0.01	0.01	0.005	0.008	0.008	t	96352	GVTKAAEKQM	Homo sapiens
1	1.00	1	1	0.07	0.07	0.07	0.07	0.069	t	112279	QTEDLAV	Homo sapiens
1	1.00	1	1	0.01	0.01	0.01	0.01	0.008	t	47778	PGVLMGGQSESVEFT	Mus musculus
1	1.00	1	1	0.01	0.01	0.01	0.01	0.008	t	54862	RMAWDMMMN	Homo sapiens
1	1.00	1	1	-0.01	-0.01	-0.015	-0.01	-0.01	t	112461	AVLKLMGRGTKF	Rattus norvegicus
1	1.00	1	1	-0.01	-0.008	-0.005	-0.013	-0.012	t	95979	VPQPPVSQFHIQ	Homo sapiens
1	1.00	1	1	0.04	0.04	0.046	0.04	0.037	t	114404	IESWDPNNQQFQCAG	Homo sapiens
1	1.00	1	1	-0.02	-0.02	-0.02	-0.02	-0.02	t	91876	RALGLPPFLNSLPQS	Oryctolagus cuniculus
1	1.00	1	1	-0.05	-0.05	-0.062	-0.054	-0.05	t	96239	EKPTPPVKPT	Homo sapiens(human)
1	1.00	1	1	0.06	0.06	0.065	0.06	0.059	t	97596	QTPLTMAALELL	Mus musculus
1	1.00	1	1	0.02	0.02	0.025	0.02	0.018	t	134188	PMSTPSATSVR	Oryctolagus cuniculus
1	1.00	1	1	0.03	0.03	0.03	0.03	0.03	t	131697	TGTSSDVGGYNYVSWY	Homo sapiens

1	1.00	1	1	0.03	0.032	0.017	0.031	0.032	t	137939	NVLSADFT	Homo sapiens
1	1.00	1	1	0	0	-0.011	0	0	t	106842	SKDEIDSA VKMLVSLK MSY	Mus musculu s
1	1.00	1	1	-0.05	-0.049	-0.057	-0.051	-0.05	t	59854	SNKFADFL	Oryctola gus cuniculu s
1	1.00	1	1	-0.03	-0.03	-0.03	-0.03	-0.03	t	26234	IGGPLT	Homo sapiens
1	1.00	1	1	0.01	0.01	0.009	0.02	0.012	t	107031	YTQKHKH QA	Homo sapiens
1	1.00	1	1	-0.02	-0.02	-0.022	-0.02	-0.022	t	21471	GNLNTIRT RVHE	Oryctola gus cuniculu s
1	1.00	1	1	-0.02	-0.02	-0.022	-0.02	-0.022	t	21471	GNLNTIRT RVHE	Oryctola gus cuniculu s
1	1.00	1	1	0.01	0.006	0.009	0.009	0.008	t	6953	CRTNYGYS	Oryctola gus cuniculu s
1	1.00	1	1	0.01	0.008	0.013	0.014	0.01	t	10692	DVPDYASL	Mus musculu s
1	1.00	1	1	0	0	0	0	-0.002	t	151731	SAQSQRA PDR	Mus musculu s
1	1.00	1	1	0	0	0	0	-0.002	t	68401	VEVPGSQ HIDSQKKA	Mus musculu s
1	1.00	1	1	0.02	0.02	0.02	0.02	0.02	t	78396	GLANDV	Mus musculu s
1	1.00	1	1	0.02	0.02	0.007	0.02	0.021	t	68054	VDREGRD NVRSKGV	Homo sapiens
1	1.00	1	1	-0.02	-0.02	-0.028	-0.02	-0.021	t	94573	KRIPNKKP GKKT	Mus musculu s
1	1.00	1	1	0.02	0.017	0.013	0.02	0.02	t	79926	MASMTG GQQMG	Oryctola gus cuniculu s

1	1.00	1	1	0.03	0.03	0.03	0.03	0.03	t	112150	GVPDRFT	Homo sapiens
1	1.00	1	1	0.02	0.016	0.018	0.019	0.02	t	120407	APGYKHEI KYNC	Oryctola gus cuniculu s
1	1.00	1	1	0.02	0.018	0.018	0.019	0.02	t	120407	APGYKHEI KYNC	Mus musculu s
1	1.00	1	1	0	-0.002	0	-0.001	-0.003	t	79303	LVAAGVD YEDERISF QDWPK	Rattus norvegic us
1	1.00	1	1	0	-0.004	0	-0.001	-0.003	t	73385	YALADASL KMADPNR FRGKD	Oryctola gus cuniculu s
1	1.00	1	1	0	-0.002	0	-0.001	-0.003	t	19636	GFTTNEER YNVFAE	Mus musculu s
1	1.00	1	1	0.03	0.03	0.03	0.03	0.031	t	127185	TCDDPRF QDSS	Oryctola gus cuniculu s
1	1.00	1	1	0.02	0.022	0.013	0.023	0.02	t	130448	AYAKDVKF GADA	Homo sapiens
1	1.00	1	1	0.04	0.038	0.033	0.037	0.041	t	131217	EDISAYLL	Mus musculu s
1	1.00	1	1	0	0	0	0	-0.002	t	14435	ETIYNTTLK Y	Homo sapiens
1	1.00	1	1	-0.03	-0.03	-0.03	-0.03	-0.03	t	45816	NSGCFRHL	Mus musculu s
1	1.00	1	1	-0.03	-0.027	-0.03	-0.03	-0.03	t	45816	NSGCFRHL	Mus musculu s
1	1.00	1	1	0	-0.003	-0.008	0	-0.001	t	153392	GNTLQCI MLVYCFLG YCC	Oryctola gus cuniculu s
1	1.00	1	1	-0.01	-0.01	-0.01	-0.008	-0.01	t	22123	GRLGTYRY VSSGER	Homo sapiens

1	1.00	1	1	0.01	0.012	0	0.011	0.01	t	53191	RARFEELCGDLFRSTIQPVE	Homo sapiens
1	1.00	1	1	0	0	0	0	-0.002	t	48597	PNANPN	Mus musculus
1	1.00	1	1	0	-0.002	-0.003	-0.001	0	t	32809	KPNNDFHFEVFN	Mus musculus
1	1.00	1	1	0	0	0	0	0	t	130453	CLFFQNGKVKCAAC	Homo sapiens
1	1.00	1	1	0.02	0.024	0.02	0.027	0.02	t	117888	GQILDILVSRSLK	Homo sapiens
1	1.00	1	1	-0.01	-0.013	-0.013	-0.01	-0.011	t	7208	CTSPYDGKYWSMYSRL	Oryctolagus cuniculus
1	1.00	1	1	0	-0.003	0	0	-0.001	t	24652	HQTLQDPRVRGL	Oryctolagus cuniculus
1	1.00	1	1	0.02	0.023	0.018	0.02	0.018	t	22015	GREGYPADSKGCKIT	Equus caballus
1	1.00	1	1	0	0	-0.017	-0.004	0	t	32970	KQEGDKCVENPNPTCNENN	Homo sapiens
1	1.00	1	1	-0.02	-0.02	-0.02	-0.016	-0.02	t	143932	HNIWSAPRLIFN	Homo sapiens
1	1.00	1	1	-0.02	-0.02	-0.015	-0.01	-0.021	t	115109	SKIFKLGGRDSRSGSPMARR	Homo sapiens
1	1.00	1	1	0.01	0.014	0.008	0.013	0.011	t	11286	EDAKELLESSDQILR	Homo sapiens
1	1.00	1	1	0	0	0	-0.001	-0.003	t	105863	HHQKLVFFAEDVGSN	Mus musculus
1	1.00	1	1	0.03	0.028	0.027	0.03	0.03	t	120167	RFQSSAVMALQEACEAYL	Mus musculus

1	1.00	1	1	0.02	0.02	0.02	0.02	0.02	t	106991	WASDLMHRLY	Homo sapiens
1	1.00	1	1	-0.01	-0.013	-0.013	-0.01	-0.01	t	39916	LTGISICNPGSSLC	Oryctolagus cuniculus
1	1.00	1	1	0.02	0.018	0.02	0.019	0.018	t	123361	KFYCDYCDTY	Homo sapiens
1	1.00	1	1	0.03	0.028	0.031	0.033	0.033	t	11377	EDLDGA	Mus musculus
1	1.00	1	1	0.02	0.024	0.028	0.027	0.022	t	124205	GPLQLGM	Homo sapiens
1	1.00	1	1	0.01	0.013	0.008	0.009	0.01	t	7761	DDEADRYFTYEE	Capra hircus
1	1.00	1	1	-0.03	-0.026	-0.03	-0.033	-0.031	t	106340	DIMPCLHFVKGE	Homo sapiens
1	1.00	1	1	0	0	0	0	-0.002	t	48006	PIVNKQSCSISNIETV	Mus musculus
1	1.00	1	1	-0.01	-0.012	-0.01	-0.011	-0.011	t	49191	PRGRRQPI	Mus musculus
1	1.00	1	1	0	0	-0.005	0	0.002	t	133608	INTTVCAGY	Mus musculus
1	1.00	1	1	0	0	0	0	-0.002	t	117515	GRGRGRGRGRGRG	Homo sapiens
1	1.00	1	1	-0.01	-0.01	-0.01	-0.01	-0.011	t	69175	VKAETRLN	Mus musculus
1	1.00	1	1	-0.05	-0.05	-0.05	-0.05	-0.05	t	54401	RKPARL	Homo sapiens
1	1.00	1	1	0.01	0.005	0.006	0.013	0.012	t	157466	KLDITGKPMV	Canis lupus

1	1.00	1	1	0.01	0.01	0.01	0.01	0.011	t	115202	DDLTDDIMCV	Homo sapiens
1	1.00	1	1	0.02	0.02	0.02	0.02	0.02	t	24035	HIDSQK	Oryctolagus cuniculus
1	1.00	1	1	-0.01	-0.01	-0.017	-0.01	-0.011	t	25140	HWSYGLRPG	Mus musculus
1	1.00	1	1	0.01	0.01	0.009	0.02	0.012	t	105976	NGVTLGYTSGTCQGNASATK	Homo sapiens
1	1.00	1	1	0.02	0.022	0.029	0.027	0.02	t	102706	PAESGGSQPPRA	Homo sapiens
1	1.00	1	1	0.03	0.03	0.031	0.03	0.028	t	31555	KKGSSGYS	Mus musculus
1	1.00	1	1	-0.03	-0.03	-0.03	-0.03	-0.033	t	117695	YITTIAGVM	Homo sapiens
1	1.00	1	1	0.01	0.01	0.01	0.01	0.01	t	115923	QPLPPTVMFP	Homo sapiens
1	1.00	1	1	0.08	0.08	0.068	0.08	0.082	t	20167	GHNSVDFEED	Oryctolagus cuniculus
1	1.00	1	1	-0.02	-0.021	-0.022	-0.02	-0.021	t	11273	ECTLKKGSSGYCAWP	Equus caballus
1	1.00	1	1	-0.02	-0.026	-0.022	-0.02	-0.021	t	11273	ECTLKKGSSGYCAWP	Oryctolagus cuniculus
1	1.00	1	1	0.01	0.018	0.01	0.01	0.008	t	156958	SLHSSVQ	Gallus gallus
1	1.00	1	1	0	0.003	0	0	-0.003	t	80000	RGTGSYNRSSFES	Mus musculus
1	1.00	1	1	0.01	0.013	0.01	0.011	0.009	t	103838	DAEFRHDSGYEV	Mus musculus
1	1.00	1	1	0.01	0.01	0.008	0.013	0.01	t	71921	VWARPDYN	Homo sapiens

1	1.00	1	1	-0.01	-0.01	-0.013	-0.01	-0.013	t	41601	MGLIAGAVGGSLLAALV	Mus musculus
1	1.00	1	1	-0.01	-0.012	-0.01	-0.011	-0.011	t	153393	GPDAPRIPLSKM	Mus musculus
1	1.00	1	1	0	0	0	0.007	0.002	t	125551	RNLTYIDPDALKELPLLKFL	Homo sapiens
1	1.00	1	1	0.04	0.04	0.044	0.04	0.04	t	23207	GVVTLY	Homo sapiens
1	1.00	1	1	0	-0.003	0.005	0	-0.002	t	146795	CCELCCNPQCAGCY	Oryctolagus cuniculus
1	1.00	1	1	-0.03	-0.029	-0.031	-0.027	-0.031	t	99622	RNFRPLTPRGYTTWV	Equus caballus
1	1.00	1	1	-0.01	-0.016	-0.01	-0.01	-0.01	t	123787	TPATKKAV	Oryctolagus cuniculus
1	1.00	1	1	0	0	0	0	-0.001	t	133623	KPGEMR	Mus musculus
1	1.00	1	1	-0.01	-0.008	-0.028	-0.009	-0.008	t	58197	SGPSGTSPSSRSNTL	Homo sapiens
1	1.00	1	1	-0.01	-0.01	-0.01	-0.01	-0.01	t	140864	GYLAGKGVDAGK	Oryctolagus cuniculus
1	1.00	1	1	0.01	0.01	0.007	0.016	0.01	t	21109	GLRNVAAGAN	Homo sapiens
1	1.00	1	1	0	0	0	0	0.001	t	61394	SSTKNLIY	Mus musculus
1	1.00	1	1	0	0	0	0	0.001	t	61394	SSTKNLIY	Mus musculus

1	1.00	1	1	0.02	0.018	0.001	0.013	0.02	t	127933	APDDSFKKLEDRFTNLKF	Mus musculus
1	1.00	1	1	0.01	0.019	0.004	0.01	0.01	t	65096	TLTKEYED	Oryctolagus cuniculus
1	1.00	1	1	-0.02	-0.02	-0.02	-0.02	-0.021	t	48837	PPNAPIL	Mus musculus
1	1.00	1	1	-0.03	-0.027	-0.046	-0.031	-0.03	t	8370	DGEERQSYWFEN	Capra hircus
1	1.00	1	1	0	-0.002	0.002	-0.001	0	t	3180	AMQWNSTAFH	Mus musculus
1	1.00	1	1	0.02	0.02	0.013	0.022	0.02	t	117015	KEQFLDGDGWTSRWIESK	Homo sapiens
1	1.00	1	1	0	0	0.009	0	-0.005	t	48085	PKINRSASEPSL	Oryctolagus cuniculus
1	1.00	1	1	0.01	0.01	0.01	0.01	0.009	t	133624	KPGEMRGAA	Mus musculus
1	1.00	1	1	0.01	0.01	0.005	0.01	0.01	t	137909	KPESDELTAEK	Homo sapiens
1	1.00	1	1	0	0	0	0	-0.002	t	151813	CGADSYEMEEDGVRKC	Mus musculus
1	1.00	1	1	0	0	0	0	-0.002	t	137770	TLARSFIICIQM	Mus musculus
1	1.00	1	1	0	0	0	0	-0.002	t	55818	RSDYKFYEDANGTRDHKKG	Chinchilla lanigera
1	1.00	1	1	0	0	0	0	-0.002	t	55818	RSDYKFYEDANGTRDHKKG	Chinchilla lanigera
1	1.00	1	1	0	0	0	0	-0.002	t	36959	LKMADPNRFRGKD	Mus musculus
1	1.00	1	1	0	0	0	0	-0.002	t	25139	HWSISKPQ	Mus musculus

1	1.00	1	1	0	0	0	0	-0.002	t	7492	DAEFRHDSGYEVHHQKL	Mus musculus
1	1.00	1	1	0	0	0	0	-0.002	t	7492	DAEFRHDSGYEVHHQKL	Mus musculus
1	1.00	1	1	-0.04	-0.04	-0.052	-0.04	-0.039	t	53864	RGFLGRMLF	Homo sapiens
1	1.00	1	1	0.01	0.01	0.01	0.01	0.01	t	119673	WNPDDYGGVK	Homo sapiens
1	1.00	1	1	-0.01	-0.022	-0.01	-0.007	-0.01	t	156911	LGMSTNPGPRIL	Bos taurus
1	1.00	1	1	0.03	0.03	0.03	0.03	0.028	t	120649	ITVGLQLIQLISVDEVNQIV	Mus musculus
1	1.00	1	1	-0.01	-0.008	-0.01	-0.003	-0.01	t	107357	EYKCKVS	Homo sapiens
1	1.00	1	1	-0.01	-0.008	-0.014	-0.009	-0.01	t	55293	RPQDVKFP	Homo sapiens
1	1.00	1	1	0.01	0.012	0.006	0.018	0.01	t	47984	PISLLFNEKEADDNDHEPQI	Homo sapiens
1	1.00	1	1	-0.04	-0.044	-0.04	-0.047	-0.042	t	122320	DVTVGPLIFL	Oryctolagus cuniculus
1	1.00	1	1	0.03	0.03	0.03	0.03	0.03	t	99839	QSPF	Mus musculus
1	1.00	1	1	-0.03	-0.03	-0.03	-0.027	-0.03	t	118491	LKVGLQVVAVKAP	Homo sapiens
1	1.00	1	1	0	0.002	0	0.001	-0.002	t	149304	ENTTTLKGDSFYYGK	Cavia porcellus
1	1.00	1	1	0	-0.002	0	0	-0.002	t	3245	ANDVDNSNPVVQAEQLNWL	Mus musculus
1	1.00	1	1	-0.02	-0.018	-0.027	-0.017	-0.02	t	135934	GWGSPKVTKDGV	Homo sapiens

1	1.00	1	1	0.01	0.012	-0.002	0.007	0.01	t	257	AAKVLVEG NTDERGT	Homo sapiens
1	1.00	1	1	0.01	0.006	-0.001	0.009	0.01	t	99018	SYSITTPSQ FV	Oryctola gus cuniculu s
1	1.00	1	1	0.05	0.05	0.05	0.05	0.05	t	79346	QCETESDL LIPSCP	Mus musculu s
1	1.00	1	1	0.03	0.03	0.026	0.03	0.03	t	49107	PQQQFPQ TQQ	Mus musculu s
1	1.00	1	1	-0.01	-0.01	-0.01	-0.01	-0.01	t	112414	YLSSFTF	Homo sapiens
1	1.00	1	1	0.02	0.018	0.013	0.019	0.019	t	27795	IPALTAVET GATNPL	Mus musculu s
1	1.00	1	1	-0.04	-0.041	-0.05	-0.04	-0.039	t	139752	FRYPRPKH CHTQVA	Canis lupus
1	1.00	1	1	0.03	0.028	0.028	0.033	0.032	t	105869	IAGFKGEQ GPK	Mus musculu s
1	1.00	1	1	0	-0.003	0	0	0	t	75044	YMDLGMK	Oryctola gus cuniculu s
1	1.00	1	1	0.04	0.04	0.035	0.04	0.04	t	33319	KSGATFQV EV	Mus musculu s
1	1.00	1	1	0	0	0	0	-0.002	t	151731	SAQSQRA PDR	Mus musculu s
1	1.00	1	1	0	0	0	0	-0.002	t	142810	KEWEQIYR	Rattus norvegic us
1	1.00	1	1	0	0	0	0	-0.002	t	119488	LGIWTYDG TKVSISPES	Mus musculu s
1	1.00	1	1	0	0	0	0	-0.002	t	119488	LGIWTYDG TKVSISPES	Mus musculu s

1	1.00	1	1	0	0	0	0	-0.002	t	119488	LGIWTYDG TKVSISPES	Mus musculu s
1	1.00	1	1	0	0	0	0	-0.002	t	120849	NSQPEILE RTRAELD	Mus musculu s
1	1.00	1	1	0	0	0	0	-0.002	t	119488	LGIWTYDG TKVSISPES	Mus musculu s
1	1.00	1	1	0	0	0	0	-0.002	t	120849	NSQPEILE RTRAELD	Mus musculu s
1	1.00	1	1	0	0	0	0	-0.002	t	119405	YAIVHMTK LLLDYTGKI	Rattus norvegic us
1	1.00	1	1	0	0	0	0	-0.002	t	45130	NNIMFKLD GCRDTHR YIWI	Mus musculu s
1	1.00	1	1	0	0	0	0	-0.002	t	45130	NNIMFKLD GCRDTHR YIWI	Mus musculu s
1	1.00	1	1	0	0	0	0	-0.002	t	34612	KYTMFHYL RAQEFEH GKSR	Mus musculu s
1	1.00	1	1	0	-0.003	0	0	-0.002	t	24786	HSLGKWL GHPDKF	Oryctola gus cuniculu s
1	1.00	1	1	0	0	0	0	-0.002	t	34612	KYTMFHYL RAQEFEH GKSR	Mus musculu s
1	1.00	1	1	0	0	0	0	-0.002	t	34612	KYTMFHYL RAQEFEH GKSR	Mus musculu s
1	1.00	1	1	0	0	0	0	-0.002	t	34612	KYTMFHYL RAQEFEH GKSR	Mus musculu s
1	1.00	1	1	-0.02	-0.02	-0.02	-0.02	-0.021	t	24590	HQLDPAF GAN	Mus musculu s

1	1.00	1	1	0.06	0.06	0.06	0.06	0.058	t	107892	HSASQDGQDTIRGHPGSS	Homo sapiens
1	1.00	1	1	0.03	0.03	0.032	0.03	0.031	t	153270	LVAQINLEM	Mus musculus
1	1.00	1	1	-0.04	-0.047	-0.047	-0.033	-0.038	t	156885	GYRAR	Homo sapiens
1	1.00	1	1	-0.03	-0.03	-0.03	-0.03	-0.029	t	60751	SRNWLPGPCY	Mus musculus
1	1.00	1	1	0	0.002	-0.011	0.001	0	t	57296	SDSVGQQIKVIPVDPYFFQM	Homo sapiens
1	1.00	1	1	0.04	0.04	0.043	0.04	0.04	t	48606	PNATQPEL	Oryctolagus cuniculus
1	1.00	1	1	0	-0.003	0.014	0	-0.001	t	60590	SQW	Oryctolagus cuniculus
1	1.00	1	1	0.01	0.008	-0.001	0.009	0.01	t	21807	GPTEESVERAMGRVA	Rattus norvegicus
1	1.00	1	1	-0.01	-0.005	-0.01	-0.01	-0.01	t	39354	LSFTLTGNPNRP	Homo sapiens
1	1.00	1	1	0.02	0.02	0.013	0.02	0.02	t	51572	QLVLGENLDTERILAS	Homo sapiens
1	1.00	1	1	0.04	0.04	0.036	0.04	0.04	t	102723	PPTPTPDQPEPNKPVEPTYE	Mus musculus
1	1.00	1	1	0.02	0.02	0.02	0.02	0.021	t	115245	EYGLFQINNK	Homo sapiens
1	1.00	1	1	-0.01	-0.01	-0.014	-0.01	-0.01	t	157683	MRTSTDMPSQHI	Canis lupus
1	1.00	1	1	0.02	0.02	0.02	0.02	0.019	t	98842	KDLAFPGSGE	Homo sapiens
1	1.00	1	1	-0.01	-0.012	-0.011	-0.007	-0.01	t	32817	KPNYAPANCPKS	Mus musculus

1	1.00	1	1	0.03	0.028	0.025	0.033	0.03	t	16396	FKLEKEVAE	Mus musculus
1	1.00	1	1	0.03	0.028	0.025	0.033	0.03	t	16396	FKLEKEVAE	Mus musculus
1	1.00	1	1	0.02	0.024	0.027	0.017	0.018	t	107011	YFALEAYAFD	Homo sapiens
1	1.00	1	1	0.02	0.018	0.015	0.02	0.021	t	117598	PEPAKSAPAPKKG	Mus musculus
1	1.00	1	1	0.04	0.04	0.036	0.037	0.04	t	54145	RIESEGGYIETWNPN	Homo sapiens
1	1.00	1	1	0.03	0.03	0.03	0.03	0.03	t	135454	DYEDRY	Mus musculus
1	1.00	1	1	0	0	0.005	0	-0.006	t	35398	LEDPASRDLVVSY	Homo sapiens
1	1.00	1	1	0.04	0.038	0.033	0.043	0.042	t	13657	EPGVLMGGQSESVEFT	Mus musculus
1	1.00	1	1	0.03	0.034	0.031	0.037	0.03	t	127957	EESRGRTSSKTAFYQALQNS	Homo sapiens
1	1.00	1	1	0.01	0.01	0.01	0.01	0.008	t	68865	VHLDSAFWFVRTDFD	Mus musculus
1	1.00	1	1	0.01	0.007	0.003	0.01	0.01	t	9305	DLTNITHVEKLC	Oryctolagus cuniculus
1	1.00	1	1	-0.01	-0.009	-0.01	-0.007	-0.01	t	78281	TKLPAA	Equus caballus
1	1.00	1	1	0.01	0.01	0.003	0.01	0.01	t	19637	GFTTNEPRYNVFAE	Mus musculus
1	1.00	1	1	-0.01	-0.01	-0.01	-0.01	-0.013	t	136594	RHYQNFQFHF	Homo sapiens
1	1.00	1	1	-0.01	-0.008	-0.003	-0.003	-0.009	t	108007	LLPWLDGHPM	Homo sapiens

1	1.00	1	1	-0.02	-0.016	-0.016	-0.013	-0.02	t	144644	FRQQHGK TVV	Homo sapiens
1	1.00	1	1	0.01	0.013	0.012	0.01	0.009	t	135845	WTEIHDW EAAME	Mus musculu s
1	1.00	1	1	0.01	0.013	0.012	0.01	0.009	t	135845	WTEIHDW EAAME	Mus musculu s
1	1.00	1	1	0	0	-0.002	0	-0.002	t	100320	NCDFSQT ATLTN	Oryctola gus cuniculu s
1	1.00	1	1	-0.03	-0.032	-0.03	-0.03	-0.03	t	2040	AINRRVE	Mus musculu s
1	1.00	1	1	0.03	0.03	0.03	0.03	0.03	t	131892	GNDTSSVS GWGDPKP	Homo sapiens
1	1.00	1	1	-0.02	-0.02	-0.034	-0.017	-0.018	t	71138	VSSQSFRR	Homo sapiens
1	1.00	1	1	0	0	0	0.004	0	t	32113	KLQPAEIET CMKTIT	Homo sapiens
1	1.00	1	1	0.02	0.022	0.014	0.019	0.02	t	48452	PLPPVA	Cavia porcellu s
1	1.00	1	1	0	0	-0.007	0	0	t	122375	GLINRAKA VKQFEESQ G	Mus musculu s
1	1.00	1	1	0	0	-0.007	0	0	t	125791	CSFWSKYI QTLK	Mus musculu s
1	1.00	1	1	0	0	-0.007	0	0	t	122375	GLINRAKA VKQFEESQ G	Mus musculu s
1	1.00	1	1	0	0	-0.007	0	0	t	122375	GLINRAKA VKQFEESQ G	Mus musculu s
1	1.00	1	1	0	0	-0.007	0	0	t	114223	PFSRVVHL	Rattus norvegic us

1	1.00	1	1	0	0	-0.007	0	0	t	114236	VGWYRSPFSR	Rattus norvegicus
1	1.00	1	1	0	0	-0.007	0	0	t	112842	SSKRGRQTPVLYAMLDHSRS	Mus musculus
1	1.00	1	1	0.01	0.012	0.01	0.01	0.01	t	3274	ANETIYNTTLKYGDV	MD
1	1.00	1	1	-0.02	-0.02	-0.028	-0.02	-0.021	t	3539	APENKGTG	Mus musculus
1	1.00	1	1	0	0	0	0	0	t	106780	RKYFG	Homo sapiens
1	1.00	1	1	-0.03	-0.03	-0.03	-0.03	-0.033	t	107156	GLAGAPGLR	Mus musculus
1	1.00	1	1	-0.02	-0.02	-0.02	-0.013	-0.02	t	135987	RSITLGNSTITT	Homo sapiens
1	1.00	1	1	0	0	0	0	-0.002	t	151731	SAQSQRAPDR	Mus musculus
1	1.00	1	1	0	0	0	0	-0.002	t	120109	NIHPKAPIWGIITSNF	Rattus norvegicus
1	1.00	1	1	0	0	0	0	-0.002	t	106487	GTSTEVWLAPDP	Mus musculus
1	1.00	1	1	0	0	0	0	-0.002	t	106690	NSDHFLQPDPPL	Mus musculus
1	1.00	1	1	0	0	0	0	-0.002	t	106363	DVYWLMPDPPTS	Mus musculus
1	1.00	1	1	0	0	0	0	-0.002	t	106225	ADIWLTPDPTTP	Mus musculus
1	1.00	1	1	0	0	0	0	-0.002	t	106535	IFPSFLRPDPPL	Mus musculus
1	1.00	1	1	-0.03	-0.028	-0.037	-0.033	-0.03	t	28575	ISLIFIYETF	Homo sapiens
1	1.00	1	1	0.05	0.05	0.055	0.056	0.053	t	115912	QAPEY	Homo sapiens

1	1.00	1	1	0.01	0.007	0.01	0.01	0.01	t	13974	EREDERTL	Oryctolagus cuniculus
1	1.00	1	1	0.01	0.01	0.01	0.01	0.01	t	13974	EREDERTL	Oryctolagus cuniculus
1	1.00	1	1	-0.04	-0.037	-0.038	-0.04	-0.041	t	133749	YNLLMHLSSML	Mus musculus
1	1.00	1	1	0.04	0.04	0.042	0.042	0.04	t	55834	RSFGSSSTSGITGD	Homo sapiens
1	1.00	1	1	0.01	0.012	0.01	0.017	0.012	t	120210	SQKDLLEQRRAAVDTY	Homo sapiens
1	1.00	1	1	0	0.004	0	0.001	0.002	t	5380	AVILLMCAVHPTLV	Homo sapiens
1	1.00	1	1	-0.01	-0.01	-0.01	-0.01	-0.012	t	133231	MGPRRRSRKP	Homo sapiens
1	1.00	1	1	0	0.004	0	0.007	0	t	116684	CNYYSNSYSFWLASLDPKR	Homo sapiens
1	1.00	1	1	0.01	0.01	0.01	0.01	0.01	t	33745	KTKRNTN	Homo sapiens
1	1.00	1	1	0.01	0.006	0.009	0.009	0.01	t	40718	LYATFDEFPPP	Oryctolagus cuniculus
1	1.00	1	1	0	0	-0.009	-0.003	0	t	27959	IPNGTYRA	Homo sapiens
1	1.00	1	1	0.04	0.04	0.04	0.04	0.04	t	158603	PVELQLY	Homo sapiens
1	1.00	1	1	0	0	0	0.007	0	t	136010	VMIKSLPALNSPTVEEC	Homo sapiens
1	1.00	1	1	-0.02	-0.017	-0.02	-0.02	-0.021	t	105701	CVVGCGGGV	Mus musculus
1	1.00	1	1	0.04	0.038	0.038	0.039	0.043	t	103117	DTDY	Mus musculus

1	1.00	1	1	0	0.002	-0.009	0.001	0.003	t	110967	PRKLPQLC TELQTTI	Homo sapiens
1	1.00	1	1	0.03	0.03	0.03	0.03	0.03	t	132813	FGENNNM	Homo sapiens
1	1.00	1	1	0.04	0.04	0.028	0.04	0.042	t	72143	VYQVNNL EEIC	Mus musculu s
1	1.00	1	1	0	-0.004	0.002	0.003	0.001	t	99367	KDGYIVD	Oryctola gus cuniculu s
1	1.00	1	1	0.02	0.021	0.02	0.021	0.02	t	4884	ATALDTSN	Oryctola gus cuniculu s
1	1.00	1	1	0.04	0.038	0.031	0.039	0.04	t	65403	TNQEQTSL YV	Mus musculu s
1	1.00	1	1	0.01	0.011	0	0.01	0.012	t	22037	GRFRTIVC GLFPTQ	Myodes glareolu s
1	1.00	1	1	0.02	0.02	0.02	0.02	0.019	t	112150	GVPDRFT	Homo sapiens
1	1.00	1	1	0	0	-0.007	0	0	t	20088	GGWGQP HGGGWG QG	Mus musculu s
1	1.00	1	1	0.02	0.02	0.017	0.026	0.022	t	74322	YINNELIPS MNQLSC	Homo sapiens
1	1.00	1	1	-0.01	-0.01	-0.017	-0.01	-0.01	t	4027	AQPHEPG SYTNVFDH LDWIK	Oryctola gus cuniculu s
1	1.00	1	1	0.02	0.016	0.013	0.023	0.02	t	107013	YGASSRAT GIPDR	Oryctola gus cuniculu s
1	1.00	1	1	0.06	0.064	0.067	0.061	0.06	t	73605	YDVVST	Homo sapiens
1	1.00	1	1	0.06	0.062	0.067	0.061	0.06	t	73605	YDVVST	Homo sapiens

1	1.00	1	1	0.02	0.02	0.014	0.017	0.021	t	67729	VAQYKALP VVLENA	Homo sapiens
1	1.00	1	1	0.01	0.012	0.005	0.013	0.01	t	60285	SPTHYVPE SDAAAR	Homo sapiens
1	1.00	1	1	0.01	0.012	0.005	0.013	0.01	t	60285	SPTHYVPE SDAAAR	Homo sapiens
1	1.00	1	1	0.01	0.012	0.005	0.013	0.01	t	60285	SPTHYVPE SDAAAR	Homo sapiens
1	1.00	1	1	0.01	0.012	0.005	0.013	0.01	t	60285	SPTHYVPE SDAAAR	Homo sapiens
1	1.00	1	1	-0.01	-0.006	0.019	-0.01	-0.018	t	31772	KLCGVL	Homo sapiens
1	1.00	1	1	0	0.004	-0.009	-0.003	-0.002	t	113857	SGYYPTSP QQPGQG	Homo sapiens
1	1.00	1	1	-0.01	-0.014	-0.007	-0.007	-0.012	t	29379	IVNTLNGF YRSL	Oryctola gus cuniculu s
1	1.00	1	1	0.02	0.022	0.013	0.017	0.018	t	106891	TADFDCSK IQST	Homo sapiens
1	1.00	1	1	-0.01	-0.013	-0.009	-0.01	-0.012	t	5924	AYSMSFS WDWSGH NY	Oryctola gus cuniculu s
1	1.00	1	1	-0.01	-0.013	-0.009	-0.01	-0.012	t	5924	AYSMSFS WDWSGH NY	Oryctola gus cuniculu s
1	1.00	1	1	0	0.003	0.002	0	-0.002	t	60991	SSFFSYGEI	Mus musculu s
1	1.00	1	1	-0.02	-0.02	-0.032	-0.026	-0.02	t	31141	KHPIKHQG LP	Homo sapiens
1	1.00	1	1	0	-0.002	0	0.005	0	t	40110	LTPLEELY	Mus musculu s

1	1.00	1	1	-0.02	-0.018	-0.031	-0.023	-0.02	t	158933	KTFFHTIGF ASKSADDV K	Homo sapiens
1	1.00	1	1	-0.01	-0.01	-0.018	-0.01	-0.01	t	88083	RNHKYDIK LT	Homo sapiens
1	1.00	1	1	-0.01	-0.007	-0.017	-0.01	-0.008	t	100414	QFINDQIK FI	Mus musculu s
1	1.00	1	1	0	-0.002	0	0.003	-0.001	t	125538	QQQPFP	Mus musculu s
1	1.00	1	1	0.02	0.024	0.021	0.021	0.02	t	40070	LTNSKG	Homo sapiens
1	1.00	1	1	-0.03	-0.03	-0.03	-0.03	-0.03	t	140840	EAAYSKNR RAVL	Oryctola gus cuniculu s
1	1.00	1	1	0	0	-0.002	0	0.002	t	17504	FQNEKWD L	Mus musculu s
1	1.00	1	1	0.01	0.012	0.017	0.017	0.01	t	138217	PREQQFN	Homo sapiens
1	1.00	1	1	-0.01	-0.012	-0.01	-0.009	-0.013	t	45597	NQNNFVH DCVNITIK QHTVT	Mus musculu s
1	1.00	1	1	0	-0.002	0	-0.001	-0.003	t	97231	CNTKCQTP	Mus musculu s
1	1.00	1	1	0	-0.002	0	-0.001	-0.003	t	68054	VDREGRD NVRSKGV	Mus musculu s
1	1.00	1	1	0	-0.002	0	-0.001	-0.003	t	58582	SIKEDVQF	Mus musculu s
1	1.00	1	1	0	-0.002	0	-0.001	-0.003	t	40922	LYREFD	Mus musculu s
1	1.00	1	1	0	-0.004	0	-0.001	-0.003	t	1022	AEKQKAAE ATKVAE	Oryctola gus cuniculu s

1	1.00	1	1	0.03	0.03	0.021	0.03	0.033	t	8475	DGQPAGDRADGQPAGDRA	Mus musculus
1	1.00	1	1	-0.01	-0.01	-0.01	-0.01	-0.01	t	27291	ILRKSRRFAQALPV	Homo sapiens
1	1.00	1	1	0.02	0.009	0.02	0.02	0.021	t	19268	GEGAPP	Marmota monax
1	1.00	1	1	0.05	0.05	0.052	0.05	0.049	t	73601	YDVPDYAS	Mus musculus
1	1.00	1	1	0.03	0.03	0.021	0.028	0.03	t	135931	GDVEEAIERAVV	Homo sapiens
1	1.00	1	1	0.01	-0.002	0.01	0.013	0.01	t	156843	AHSEMGTRATMT	Bos taurus
1	1.00	1	1	0.01	0.008	0	0.009	0.012	t	37354	LLGNQSLL	Anas platyrhynchos
1	1.00	1	1	-0.05	-0.05	-0.05	-0.05	-0.051	t	105697	CCAVLNGAY	Mus musculus
1	1.00	1	1	0.04	0.043	0.037	0.04	0.042	t	37029	LKTAAESA	Mus musculus
1	1.00	1	1	0	0	0	0.004	0.001	t	118374	GKALCWVCAQSRKHRD	Homo sapiens
1	1.00	1	1	0.01	0.012	0.01	0.011	0.009	t	41016	MADNGTITVEELKQLLEQ	Homo sapiens
1	1.00	1	1	0	0	-0.012	0	0	t	20088	GGWGQPHGGGWGQG	Mus musculus
1	1.00	1	1	-0.04	-0.04	-0.057	-0.04	-0.036	t	145729	CPPDIIPK	Mus musculus
1	1.00	1	1	0	0.002	0.005	0.007	0.002	t	134073	GRTCPKPDDLP	Homo sapiens
1	1.00	1	1	0.05	0.048	0.045	0.051	0.051	t	39887	LTEVETPIRNEWG	Mus musculus

1	1.00	1	1	-0.01	-0.014	-0.024	-0.007	-0.008	t	58664	SIPNGTYRAT	Oryctolagus cuniculus
1	1.00	1	1	-0.01	-0.008	-0.012	-0.003	-0.009	t	48779	PPFSLSPVPTLGSRSRR	Homo sapiens
1	1.00	1	1	0	0	-0.001	0	0	t	50823	QFYQKFPQYL	Homo sapiens
1	1.00	1	1	-0.04	-0.038	-0.04	-0.033	-0.04	t	104348	WHNAPNI	Homo sapiens
1	1.00	1	1	-0.04	-0.038	-0.04	-0.033	-0.04	t	104348	WHNAPNI	Homo sapiens
1	1.00	1	1	0	0	-0.006	0	0	t	84461	KLEFFTYLRFDLELT	Homo sapiens
1	1.00	1	1	0	0.002	0	0.001	0.001	t	47289	PEGRTWAQ	Homo sapiens
1	1.00	1	1	0	0	0	0	0	t	156919	MLHTSM	Gallus gallus
1	1.00	1	1	0.01	0.01	0.02	0.01	0.009	t	95161	TYGKFCIKP	Mus musculus
1	1.00	1	1	-0.01	-0.008	-0.019	-0.009	-0.01	t	18053	FTQCSMNVVSKTLFRKSPIY	Homo sapiens
1	1.00	1	1	-0.01	-0.008	-0.019	-0.009	-0.01	t	18053	FTQCSMNVVSKTLFRKSPIY	Homo sapiens
1	1.00	1	1	-0.04	-0.04	-0.045	-0.04	-0.038	t	145831	IPLGTRP	Mus musculus
1	1.00	1	1	0.01	0.014	0.006	0.011	0.011	t	46858	PALTAAETG	Homo sapiens
1	1.00	1	1	0	0	-0.006	0	0	t	72484	WGNGCGLFG	Mus musculus

1	1.00	1	1	0.01	0.01	0.005	0.008	0.01	t	78925	AGTYDFAIAAPQANAKIWIA	Homo sapiens
1	1.00	1	1	-0.01	-0.01	-0.01	-0.009	-0.011	t	105163	EFRHD	Mus musculus
1	1.00	1	1	0	0	0	-0.001	-0.003	t	67615	VAGRKKLEELERDLRKTK	Homo sapiens
1	1.00	1	1	0	0	0	-0.001	-0.003	t	63217	TDQMEVDSGPRKRPLRGG	Homo sapiens
1	1.00	1	1	0	0	0	-0.001	-0.003	t	29852	KALENKKKQLSAGGKNLS	Homo sapiens
1	1.00	1	1	-0.02	-0.022	-0.027	-0.027	-0.02	t	108064	PGPPGPP	Mus musculus
1	1.00	1	1	0.01	0.01	0.003	0.01	0.01	t	95371	EYCSPDHNCQ	Homo sapiens
1	1.00	1	1	-0.03	-0.03	-0.03	-0.03	-0.03	t	54402	RKPARLIVFPDL	Homo sapiens
1	1.00	1	1	0	0	-0.004	0	0	t	11286	EDAKELLESSDQILR	Homo sapiens
1	1.00	1	1	-0.01	-0.01	-0.01	-0.01	-0.01	t	140836	DEAAYSKNRRAV	Oryctolagus cuniculus
1	1.00	1	1	0	0.003	-0.006	0	0	t	84184	KASAEG	Mus musculus
1	1.00	1	1	0	0.003	-0.006	0	0	t	84184	KASAEG	Mus musculus
1	1.00	1	1	-0.01	-0.007	-0.01	-0.01	-0.01	t	78413	HFYSGTER	Mus musculus
1	1.00	1	1	0	-0.002	0	0.003	-0.001	t	146990	GPGTIKKISF	Mus musculus
1	1.00	1	1	0	-0.002	0	0.003	-0.001	t	146906	EVDHTNFKYNYSVIEG	Mus musculus

1	1.00	1	1	0	-0.002	0	0.003	-0.001	t	146967	GGPIGDTL EKISNEIK	Mus musculus
1	1.00	1	1	0	-0.002	0	0.003	-0.001	t	72178	VYVEELKP TPEGDLEIL LQK	Mus musculus
1	1.00	1	1	0	-0.002	0	0.003	-0.001	t	60722	SRLGRSSA WV	Mus musculus
1	1.00	1	1	0	-0.004	0	0.003	-0.001	t	72178	VYVEELKP TPEGDLEIL LQK	Oryctolagus cuniculus
1	1.00	1	1	0	-0.004	0	0.003	-0.001	t	45768	NSARARA DSCRI	Oryctolagus cuniculus
1	1.00	1	1	0	-0.002	0	0.003	-0.001	t	54786	RLTENTEP LL	Mus musculus
1	1.00	1	1	0	-0.002	0	0.003	-0.001	t	56807	SAHGTSTG VRGP	Mus musculus
1	1.00	1	1	0	-0.004	0	0.003	-0.001	t	56195	RTTPDRQ AA	Oryctolagus cuniculus
1	1.00	1	1	0	-0.004	0	0.003	-0.001	t	56185	RTTADRQT A	Oryctolagus cuniculus
1	1.00	1	1	0	-0.002	0	0.003	-0.001	t	39349	LSFNPTQL EEQCHI	Mus musculus
1	1.00	1	1	0	-0.004	0	0.003	-0.001	t	51847	QPGVDIIE GPVKNVA VPLY	Oryctolagus cuniculus
1	1.00	1	1	0	-0.004	0	0.003	-0.001	t	45768	NSARARA DSCRI	Oryctolagus cuniculus
1	1.00	1	1	0	-0.002	0	0.003	-0.001	t	41204	MCQCVQK YGTEFCKK RLA	Mus musculus
1	1.00	1	1	0	-0.002	0	0.003	-0.001	t	43159	NAGVLTCS LSK	Mus musculus

1	1.00	1	1	0	-0.004	0	0.003	-0.001	t	59387	SLPGQTKILAKVFYGEN	Oryctolagus cuniculus
1	1.00	1	1	0	-0.002	0	0.003	-0.001	t	28676	ISQAVHAAHAEINEAGR	Mus musculus
1	1.00	1	1	0	-0.002	0	0.003	-0.001	t	20097	GGYGDGGAHGGGYGG	Mus musculus
1	1.00	1	1	0	-0.002	0	0.003	-0.001	t	1367	AFNQFGPNAGQR	Mus musculus
1	1.00	1	1	0	-0.002	0	0.003	-0.001	t	8756	DIPNPIGSENSEKTTMPLW	Mus musculus
1	1.00	1	1	0.01	0.014	0.015	0.011	0.01	t	56888	SALYVGDLCG	Homo sapiens
1	1.00	1	1	-0.01	-0.013	-0.01	-0.01	-0.01	t	135489	QFPLGGV	Oryctolagus cuniculus
1	1.00	1	1	-0.01	-0.011	-0.02	-0.007	-0.008	t	52456	QSYLRRTQSMGIQL	Myodes glareolus
1	1.00	1	1	0.04	0.04	0.04	0.04	0.04	t	112058	AYWGQGT	Homo sapiens
1	1.00	1	1	0	-0.002	0	-0.007	-0.002	t	135496	RYPNQVY	Mus musculus
1	1.00	1	1	0.01	0.01	0.01	0.01	0.01	t	139865	QGAFASPIQLQR	Mus musculus
1	1.00	1	1	0.01	0.009	0	0.01	0.01	t	41284	MDVRRIEG	Anas platyrhynchos
1	1.00	1	1	0.03	0.022	0.03	0.03	0.031	t	125055	FVYGGCQGNN	Mus musculus
1	1.00	1	1	-0.05	-0.05	-0.05	-0.04	-0.048	t	108610	RKALRLSAS	Homo sapiens
1	1.00	1	1	0.01	0.01	0.01	0.01	0.007	t	119448	EQHLPDDDKTMPSERLDD	Homo sapiens
1	1.00	1	1	0.01	0.01	-0.004	0.01	0.01	t	346	AAPANDKFTVFEAAFNDAIK	Mus musculus

1	1.00	1	1	0.01	0.01	-0.004	0.01	0.01	t	346	AAPANDKFTVFEAAFNDAIK	Mus musculus
1	1.00	1	1	-0.03	-0.03	-0.038	-0.03	-0.03	t	82608	GENKFVATAASV	Homo sapiens
1	1.00	1	1	0.01	0.012	0.006	0.013	0.012	t	23080	GVNVPAGAGAAILTP	Homo sapiens
1	1.00	1	1	0.02	0.023	0.018	0.02	0.018	t	7381	CYGVPDHIKVWDYAT	Equus caballus
1	1.00	1	1	-0.02	-0.016	-0.02	-0.019	-0.02	t	37055	LKVIRK	Homo sapiens
1	1.00	1	1	-0.02	-0.016	-0.02	-0.019	-0.02	t	37055	LKVIRK	Homo sapiens
1	1.00	1	1	0.04	0.039	0.039	0.04	0.041	t	65193	TMEEINTL	Anas platyrhynchos
1	1.00	1	1	0.03	0.03	0.023	0.03	0.029	t	131652	SFDSSNPEYFDGYWGYS	Oryctolagus cuniculus
1	1.00	1	1	0.02	0.021	0.019	0.023	0.019	t	99425	LLDAIDKIADDWDNR	Equus caballus
1	1.00	1	1	0.01	0.006	0.015	0.007	0.008	t	53894	RGKDLPVLDQLTD	Oryctolagus cuniculus
1	1.00	1	1	0.06	0.064	0.048	0.057	0.062	t	65565	TPEVDDEALEK	Homo sapiens
1	1.00	1	1	0.02	0.02	0.02	0.02	0.02	t	113942	TQDENPVVHF	Homo sapiens
1	1.00	1	1	0.01	0.01	0.005	0.01	0.008	t	151671	KLVINGKPITIFQERD	Mus musculus
1	1.00	1	1	-0.05	-0.05	-0.046	-0.047	-0.051	t	22756	GTLVRMAPVIG	Homo sapiens

1	1.00	1	1	0	-0.002	0	-0.007	-0.006	t	119673	WNPDDYGGVK	Rattus norvegicus
1	1.00	1	1	0	-0.004	0	-0.007	-0.006	t	124925	YYVFFEEQEDEIIGF	Oryctolagus cuniculus
1	1.00	1	1	0	0	0	0	-0.002	t	68369	VETEDTKEPGVLMGG	Mus musculus
1	1.00	1	1	0.02	0.02	0.02	0.02	0.02	t	113783	QQSGQGQ	Homo sapiens
1	1.00	1	1	0.01	0.01	0.004	0.01	0.009	t	74942	YLTIGKNPDFKSTFAVLWEP	Oryctolagus cuniculus
1	1.00	1	1	0	0	-0.007	0	0	t	123037	AQPVRLFLE	Ovis aries
1	1.00	1	1	0.01	0.01	0.01	0.01	0.009	t	48382	PLKPTKRSFIEDLLF	Homo sapiens
1	1.00	1	1	-0.02	-0.022	-0.027	-0.019	-0.02	t	47054	PCVREGNASRCW	Homo sapiens
1	1.00	1	1	0.05	0.05	0.05	0.05	0.05	t	81191	DNIYTFVPEK	Homo sapiens
1	1.00	1	1	0.03	0.03	0.03	0.03	0.03	t	142449	NNQNLSVVQALL	Mus musculus
1	1.00	1	1	0.01	0.01	0.01	0.01	0.01	t	1660	AGRDLL	Equus caballus
1	1.00	1	1	0.02	0.018	0.02	0.019	0.02	t	72292	WDEDGEK	Mus musculus
1	1.00	1	1	-0.02	-0.023	-0.025	-0.02	-0.02	t	53763	RFQEALKKSLKKP	Mus
1	1.00	1	1	-0.02	-0.02	-0.025	-0.02	-0.02	t	53763	RFQEALKKSLKKP	Mus

1	1.00	1	1	0.02	0.023	0.025	0.02	0.02	t	138138	ALDLL	Mus musculus
1	1.00	1	1	-0.01	-0.006	-0.011	-0.013	-0.012	t	32708	KPFQCAGVALSRCTL	Homo sapiens
1	1.00	1	1	-0.01	-0.008	-0.011	-0.013	-0.012	t	32708	KPFQCAGVALSRCTL	Homo sapiens
1	1.00	1	1	0.02	0.02	0.02	0.02	0.02	t	105566	VFFAE	Mus musculus
1	1.00	1	1	-0.01	-0.01	-0.015	-0.008	-0.012	t	146852	DVAVFARGPQAHL	Homo sapiens
1	1.00	1	1	0.01	0.01	0.01	0.01	0.01	t	21868	GQGGSHSQWNKP	Oryctolagus cuniculus
1	1.00	1	1	-0.01	-0.01	-0.01	-0.01	-0.01	t	134152	LRRVLLP	Homo sapiens
1	1.00	1	1	-0.06	-0.057	-0.065	-0.06	-0.062	t	131967	LRLDCRHEN	Mus musculus
1	1.00	1	1	0.01	0.01	0.01	0.01	0.008	t	55477	RQQWELQGDRRCQSQ	Homo sapiens
1	1.00	1	1	0.06	0.06	0.06	0.06	0.06	t	79504	ADCTTPT	Oryctolagus cuniculus
1	1.00	1	1	0.04	0.038	0.033	0.043	0.044	t	78148	ENQANPTTAET	Mus musculus
1	1.00	1	1	0.04	0.04	0.047	0.046	0.039	t	55174	RPIDDFDQGWGPITY	Homo sapiens
1	1.00	1	1	0.01	0.011	0.01	0.011	0.01	t	51473	QLPNVGIT	Oryctolagus cuniculus
1	1.00	1	1	0.03	0.034	0.023	0.027	0.028	t	50504	QDPDRRDPYSPSPYD	Homo sapiens

1	1.00	1	1	0.01	0.008	0.01	0.007	0.008	t	74488	YKPLGALTH	Mus musculus
1	1.00	1	1	-0.02	-0.016	-0.026	-0.02	-0.018	t	123437	LKMWSDFLG	Ovis aries
1	1.00	1	1	0.01	0.01	0.01	0.01	0.01	t	130598	RQFECYTTCVG	Mus musculus
1	1.00	1	1	0.03	0.034	0.035	0.037	0.03	t	122145	LTLFPSRDTPLSSQTD	Homo sapiens
1	1.00	1	1	0.02	0.02	0.025	0.03	0.023	t	115832	KTGQFL	Homo sapiens
1	1.00	1	1	-0.02	-0.019	-0.021	-0.017	-0.021	t	99194	EFVDRLLSQIKSEGH	Equus caballus
1	1.00	1	1	-0.02	-0.018	-0.02	-0.019	-0.02	t	96102	APPLLPH	Homo sapiens
1	1.00	1	1	0.01	0.014	0.01	0.01	0.011	t	22357	GSGVRGDFG	Cavia porcellus
1	1.00	1	1	0.01	0.014	0.005	0.011	0.01	t	66107	TRTIGGSQAQ	Homo sapiens
1	1.00	1	1	0	0	0	-0.003	-0.003	t	71284	VTEEI	Homo sapiens
1	1.00	1	1	0	0	0	0	-0.002	t	38022	LMGGQSESVEFTKDT	Mus musculus
1	1.00	1	1	0	-0.003	-0.006	0	0	t	1584	AGKREM	Oryctolagus cuniculus
1	1.00	1	1	0.03	0.03	0.03	0.03	0.03	t	107109	EFRHDS	Mus musculus
1	1.00	1	1	0.02	0.02	0.015	0.02	0.018	t	32818	KPPFSGMTGCGNT	Homo sapiens

1	1.00	1	1	0.04	0.04	0.028	0.04	0.041	t	40194	LTTDATTF EEVATAEK LV	Oryctolagus cuniculus
1	1.00	1	1	0.01	0.012	0.016	0.011	0.009	t	8857	DKEILYEAF	Homo sapiens
1	1.00	1	1	0.03	0.032	0.021	0.031	0.033	t	110733	VGENVPD DLYIKGSG	Homo sapiens
1	1.00	1	1	0	-0.003	0	0	-0.002	t	105228	GLMVGGV V	Oryctolagus cuniculus
1	1.00	1	1	0	-0.003	0	0	-0.002	t	48093	PKKPNENK LKQPNE	Oryctolagus cuniculus
1	1.00	1	1	0	0.012	0	0	-0.002	t	9757	DPRVRGLY FPAGGSSS GTV	Pan troglodytes
1	1.00	1	1	-0.04	-0.04	-0.043	-0.04	-0.04	t	2271	AKQYNICC KFKELLD	Mus musculus
1	1.00	1	1	0.02	0.011	0.026	0.02	0.02	t	35231	LDNAKYQ A	Macaca mulatta
1	1.00	1	1	0	0	0	0	0	t	156859	DPHVFKS	Gallus gallus
1	1.00	1	1	0.01	0.01	0.009	0.01	0.01	t	10075	DRYYREN	Mus musculus
1	1.00	1	1	0.01	0.012	0.005	0.007	0.01	t	19503	GFAVVLKA GNEG	Homo sapiens
1	1.00	1	1	0.01	0.012	0.005	0.007	0.01	t	19503	GFAVVLKA GNEG	Homo sapiens
1	1.00	1	1	0.05	0.05	0.053	0.05	0.049	t	78938	CAPGQNL CY	Mus musculus
1	1.00	1	1	0.03	0.023	0.03	0.031	0.032	t	8355	DGAVQPD GG	Canis lupus

1	1.00	1	1	0.03	0.029	0.03	0.031	0.032	t	8355	DGAVQPDGG	Canis lupus
1	1.00	1	1	-0.02	-0.018	-0.02	-0.017	-0.019	t	55799	RSAPRITFG	Homo sapiens
1	1.00	1	1	0.02	0.018	0	0.013	0.02	t	128115	SSHRGNICIGATNYVADN	Mus musculus
1	1.00	1	1	0	0.003	0.007	0	-0.002	t	78695	KDTNNNLYNGK	Mus musculus
1	1.00	1	1	-0.01	-0.01	-0.01	-0.01	-0.011	t	107147	GIAGFKGEQGPK	Mus musculus
1	1.00	1	1	-0.01	-0.01	-0.01	-0.01	-0.011	t	107147	GIAGFKGEQGPK	Mus musculus
1	1.00	1	1	-0.02	-0.024	-0.027	-0.021	-0.02	t	141792	YYLWKQYNPTGKGIDD	Oryctolagus cuniculus
1	1.00	1	1	-0.03	-0.03	-0.03	-0.03	-0.033	t	125472	LDAVYLNKNKYLTVIDKDAF	Homo sapiens
1	1.00	1	1	0	0.015	0	0.007	0.001	t	125478	LHQEYEENLGDSIVGYKEKS	Homo sapiens
1	1.00	1	1	0	0.002	0	0.002	-0.001	t	7491	DAEFRHDSGYEVHHQK	Homo sapiens
1	1.00	1	1	-0.01	-0.002	-0.01	-0.01	-0.012	t	156861	DSQKPCKCHCTMTQ	Gallus gallus
1	1.00	1	1	0.03	0.028	0.028	0.029	0.03	t	94743	YGTDEWEQWW	Mus musculus
1	1.00	1	1	0.01	0.012	0.003	0.008	0.008	t	106821	SDPAHKARLE	Homo sapiens
1	1.00	1	1	0	0	0	0	-0.002	t	69175	VKAETRLN	Mus musculus
1	1.00	1	1	0	0	0.002	0.002	0	t	50477	QDIQPAVQASWPKV	Homo sapiens

1	1.00	1	1	-0.02	-0.018	-0.02	-0.017	-0.019	t	40724	LYCGGPM FNSKGAQ	Homo sapiens
1	1.00	1	1	-0.01	-0.014	-0.006	-0.007	-0.01	t	97760	VTLPSGGA V	Oryctolagus cuniculus
1	1.00	1	1	-0.03	-0.03	-0.03	-0.03	-0.03	t	79219	FWLAVR	Mus musculus
1	1.00	1	1	0	0.004	-0.003	0	0	t	123397	KSRIEDLPK	Ovis aries
1	1.00	1	1	0.04	0.04	0.04	0.04	0.041	t	115427	PTQLEEQC HI	Homo sapiens
1	1.00	1	1	0.01	0.01	0.01	0.013	0.011	t	72087	VYLLPR	Homo sapiens
1	1.00	1	1	0.02	0.02	0.016	0.02	0.02	t	51949	QPRGGQA	Mus musculus
1	1.00	1	1	-0.01	-0.012	-0.022	-0.011	-0.01	t	101164	QGEGGSR WTM	Mus musculus
1	1.00	1	1	-0.05	-0.043	-0.042	-0.051	-0.052	t	8130	DEQDYQIR	Gallus gallus
1	1.00	1	1	-0.05	-0.052	-0.042	-0.051	-0.052	t	8130	DEQDYQIR	Mus musculus
1	1.00	1	1	0.04	0.04	0.04	0.04	0.04	t	133373	SPTPTPGP SR	Homo sapiens
1	1.00	1	1	0.02	0.02	0.016	0.02	0.02	t	17368	FPQPQQT FPQ	Mus musculus
1	1.00	1	1	0.02	0.02	0.016	0.02	0.02	t	17368	FPQPQQT FPQ	Mus musculus
1	1.00	1	1	0.01	0.01	0.01	0.01	0.01	t	98931	PGDYDDD RRQ	Homo sapiens
1	1.00	1	1	0.01	0.01	0.01	0.01	0.01	t	108662	TPEVKAED LQPAVD	Canis lupus
1	1.00	1	1	0.01	0.01	0.001	0.008	0.01	t	135990	SASVPALT AVET	Homo sapiens

1	1.00	1	1	-0.01	-0.006	-0.003	-0.009	-0.009	t	18177	FVGLFTP	Homo sapiens
1	1.00	1	1	-0.02	-0.017	-0.02	-0.02	-0.022	t	113345	DTPTLH	Mus musculu s
1	1.00	1	1	0.01	0.01	0.01	0.01	0.01	t	6455	CITQYERES QAYY	Mus musculu s
1	1.00	1	1	0.03	0.03	0.03	0.03	0.028	t	137387	DFESCDSIT GM	Oryctola gus cuniculu s
1	1.00	1	1	0	-0.002	0	-0.003	-0.004	t	16101	FHENWPS	Mus musculu s
1	1.00	1	1	0.05	0.05	0.05	0.05	0.05	t	126897	QSQQFSVS ENLLKEA	Mus musculu s
1	1.00	1	1	0.01	0.01	0.011	0.01	0.009	t	158910	KNLDIDPK LQEYLGK	Mus musculu s
1	1.00	1	1	0	-0.004	-0.002	0.003	0	t	70153	VNPNNAA AL	Oryctola gus cuniculu s
1	1.00	1	1	0.02	0.02	0.018	0.02	0.02	t	144791	STIDYFQP NNKR	Sus scrofa
1	1.00	1	1	0.02	0.018	0.015	0.019	0.018	t	32585	KNPFLFGS NRFETLF	Sus scrofa
1	1.00	1	1	0.01	0.01	0.01	0.01	0.01	t	79245	HCDSFWIP PPPVCK	Mus musculu s
1	1.00	1	1	-0.02	-0.02	-0.02	-0.02	-0.02	t	107247	TFGSGTRL TV	Oryctola gus cuniculu s
1	1.00	1	1	-0.01	-0.007	-0.021	-0.01	-0.008	t	111176	CTSICKYPD YIKMVS	Mus musculu s
1	1.00	1	1	0.03	0.032	0.025	0.031	0.03	t	69905	VLYREFDE MEECSQH LPYIE	Homo sapiens

1	1.00	1	1	-0.01	-0.008	-0.01	-0.01	-0.011	t	141749	SNLDHI	Rattus norvegicus
1	1.00	1	1	-0.03	-0.033	-0.035	-0.03	-0.03	t	52472	QTAPKAPTEA	Oryctolagus cuniculus
1	1.00	1	1	0.04	0.042	0.041	0.041	0.04	t	56386	RVQYEG	Homo sapiens
1	1.00	1	1	-0.01	-0.01	-0.01	-0.01	-0.012	t	72249	WAP	Mus musculus
1	1.00	1	1	0	0.003	-0.018	0	0.003	t	41537	MFSGGGGPLSPGGKS	Mus musculus
1	1.00	1	1	0.09	0.088	0.09	0.092	0.09	t	110460	YEQLNDSSEEEDEIDG	Mus musculus
1	1.00	1	1	0	0	0	0.004	0	t	135945	KFGADARALMLQ	Homo sapiens
1	1.00	1	1	0	0	0	0.004	0	t	141604	APPDDGPP	Homo sapiens
1	1.00	1	1	0	0	0	0.004	0	t	144644	FRQQHGKTVV	Homo sapiens
1	1.00	1	1	0	0	0	0.004	0	t	142447	NKYRGTAGNALMDGASQL	Homo sapiens
1	1.00	1	1	0	0	0	0.004	0	t	142447	NKYRGTAGNALMDGASQL	Homo sapiens
1	1.00	1	1	0	0	0	0.004	0	t	142448	NKYRGTAGNALMDGASQL	Homo sapiens
1	1.00	1	1	0	0	0	0.004	0	t	141611	CETNTGEV	Homo sapiens
1	1.00	1	1	0	0	0	0.004	0	t	135449	CATLRVYKGG	Homo sapiens
1	1.00	1	1	0	0	0	0.004	0	t	135944	KEQFEFALTAVAEEVC	Homo sapiens
1	1.00	1	1	0	0	0	0.004	0	t	144616	DPRYTCQREF	Homo sapiens
1	1.00	1	1	0	0	0	0.004	0	t	135942	KDVKFGADARAL	Homo sapiens
1	1.00	1	1	0	0	0	0.004	0	t	144619	DYIWNTLNSG	Homo sapiens

1	1.00	1	1	0	0	0	0.004	0	t	137161	FTTYIIMNRWALGNLACDLW	Homo sapiens
1	1.00	1	1	0	0	0	0.004	0	t	134112	KDKATF	Homo sapiens
1	1.00	1	1	0	0	0	0.004	0	t	138304	ATVQLPQ	Homo sapiens
1	1.00	1	1	0	0	0	0.004	0	t	144639	FAQAYARGIS	Homo sapiens
1	1.00	1	1	0	0	0	0.004	0	t	135958	LTAVETGHTSQV	Homo sapiens
1	1.00	1	1	0	0	0	0.004	0	t	144700	KEWAKRAALP	Homo sapiens
1	1.00	1	1	0	0	0	0.004	0	t	136474	LLDTSNLELPEAVVFQDSVV	Homo sapiens
1	1.00	1	1	0	0	0	0.004	0	t	136003	TRKDINTVTTVA	Homo sapiens
1	1.00	1	1	0	0	0	0.004	0	t	144816	WSHNFTNMLG	Homo sapiens
1	1.00	1	1	0	0	0	0.004	0	t	135990	SASVPALTAVET	Homo sapiens
1	1.00	1	1	0	0	0	0.004	0	t	136134	ANGWGVMVSHRSGETEDT	Homo sapiens
1	1.00	1	1	0	0	0	0.004	0	t	135914	EEIAQVATISAN	Homo sapiens
1	1.00	1	1	0	0	0	0.004	0	t	135650	DKVSFFCKNKEKKC	Homo sapiens
1	1.00	1	1	0	0	0	0.004	0	t	136397	IQVVGDDLTVTNPKRIAKA	Homo sapiens
1	1.00	1	1	0	0	0	0.004	0	t	144811	VSWLSKEWAK	Homo sapiens
1	1.00	1	1	0	0	0	0.004	0	t	144759	RAALPSHVVT	Homo sapiens
1	1.00	1	1	0	0	0	0.004	0	t	135999	SQIATIEQTAPS	Homo sapiens
1	1.00	1	1	0	0	0	0.004	0	t	134136	LKTPRV	Homo sapiens
1	1.00	1	1	0	0	0	0.004	0	t	141780	WSKAHPPE	Homo sapiens
1	1.00	1	1	0	0	0	0.004	0	t	136080	RAGTDEGFG	Homo sapiens

1	1.00	1	1	0	0	0	0.004	0	t	136415	KFAGRNFRNPLAK	Homo sapiens
1	1.00	1	1	0	0	0	0.004	0	t	144600	CQREFALKHL	Homo sapiens
1	1.00	1	1	0	0	0	0.004	0	t	141629	EEEEDGEM	Homo sapiens
1	1.00	1	1	0	0	0	0.004	0	t	136524	NFREAMRIGAEVYHNLKN	Homo sapiens
1	1.00	1	1	0	0	0	0.004	0	t	144783	SHVVTMLDNF	Homo sapiens
1	1.00	1	1	0	0	0	0.004	0	t	136197	EKGVPLYRHIADLAGNSE	Homo sapiens
1	1.00	1	1	0	0	0	0.004	0	t	144823	YWEGSGIGAI	Homo sapiens
1	1.00	1	1	0	0	0	0.004	0	t	135897	APKRPGPAGELL	Homo sapiens
1	1.00	1	1	0	0	0	0.004	0	t	135982	RDRSGLAPKRPG	Homo sapiens
1	1.00	1	1	0	0	0	0.004	0	t	136203	EPLGLQCTFINDFFTYHIRH	Homo sapiens
1	1.00	1	1	0	0	0	0.004	0	t	136011	VPALTAVETGHT	Homo sapiens
1	1.00	1	1	0	0	0	0.004	0	t	144720	LMKFVDSKSG	Homo sapiens
1	1.00	1	1	0	0	0	0.004	0	t	136731	VRVFQATRGKLSSKCSVVLG	Homo sapiens
1	1.00	1	1	0	0	0	0.004	0	t	141638	EIHSKTKS	Homo sapiens
1	1.00	1	1	0	0	0	0.004	0	t	141722	PDGPDGPE	Homo sapiens
1	1.00	1	1	0	0	0	0.004	0	t	141719	NTEEYIDG	Homo sapiens
1	1.00	1	1	0	0	0	0.004	0	t	144614	DMMYGGMRGM	Homo sapiens
1	1.00	1	1	0	0	0	0.004	0	t	137313	VLVNTFCDSCIPKTFWNLGY	Homo sapiens
1	1.00	1	1	0	0	0	0.004	0	t	136421	KIHAREIFDSRGNPTVE	Homo sapiens
1	1.00	1	1	0	0	0	0.004	0	t	135711	LDETVKDKLAKEQKSKQNI	Homo sapiens

1	1.00	1	1	0	0	0	0.004	0	t	135710	LAKEQKSKQNIGALKQELAK	Homo sapiens
1	1.00	1	1	0	0	0	0.004	0	t	141634	EELQKREE	Homo sapiens
1	1.00	1	1	0	0	0	0.004	0	t	136419	KIHAREIFDSRGNPTVE	Homo sapiens
1	1.00	1	1	0	0	0	0.004	0	t	135983	RLRPPEPRPRDR	Homo sapiens
1	1.00	1	1	0	0	0	0.004	0	t	144652	GMRGMKGLVY	Homo sapiens
1	1.00	1	1	0	0	0	0.004	0	t	151101	AAA	Homo sapiens
1	1.00	1	1	0	0	0	0.004	0	t	121998	VPPALADFI	Homo sapiens
1	1.00	1	1	0	0	0	0.004	0	t	121527	LCNEKLLKKARIHPFH	Homo sapiens
1	1.00	1	1	0	0	0	0.004	0	t	117731	AAFYKTFKTV	Homo sapiens
1	1.00	1	1	0	0	0	0.004	0	t	133900	NEHPVFAYLKDKLP	Homo sapiens
1	1.00	1	1	0	0	0	0.004	0	t	118806	GGPDGEPDVPPGAIEQGPAD	Homo sapiens
1	1.00	1	1	0	0	0	0.004	0	t	117907	HKAFKGSI	Homo sapiens
1	1.00	1	1	0	0	0	0.004	0	t	118381	GKTCPKEIPKGSKNT	Homo sapiens
1	1.00	1	1	0	0	0	0.004	0	t	121879	SSQFDPNKQTPSGKK	Homo sapiens
1	1.00	1	1	0	0	0	0.004	0	t	133787	CQALVRMLAKKPGWK	Homo sapiens
1	1.00	1	1	0	0	0	0.004	0	t	125229	RDSEGDLVAEKLGPI	Homo sapiens
1	1.00	1	1	0	0	0	0.004	0	t	119855	FAESRIRRETIAAED	Homo sapiens
1	1.00	1	1	0	0	0	0.004	0	t	133841	GRGNGDPGGGMEKDG	Homo sapiens
1	1.00	1	1	0	0	0	0.004	0	t	133372	SPSPTPTPGPSRRG	Homo sapiens
1	1.00	1	1	0	0	0	0.004	0	t	118514	MASAARLTMMWEE	Homo sapiens

1	1.00	1	1	0	0	0	0.004	0	t	118014	MDC	Homo sapiens
1	1.00	1	1	0	0	0	0.004	0	t	122947	WTQEPQPLEEKWQHRVVE	Homo sapiens
1	1.00	1	1	0	0	0	0.004	0	t	132067	SLVLTLNEL	Homo sapiens
1	1.00	1	1	0	0	0	0.004	0	t	131697	TGTSSDVGGYNYVSWY	Homo sapiens
1	1.00	1	1	0	0	0	0.004	0	t	123731	SEESAEVATEEV	Homo sapiens
1	1.00	1	1	0	0	0	0.004	0	t	118105	RKGHFLLSSKSGFWTIWL	Homo sapiens
1	1.00	1	1	0	0	0	0.004	0	t	131437	GASVKVT	Homo sapiens
1	1.00	1	1	0	0	0	0.004	0	t	118348	FHSGKHYWEVDVTGKE	Homo sapiens
1	1.00	1	1	0	0	0	0.004	0	t	118333	EPVSIECGHSFCQECI	Homo sapiens
1	1.00	1	1	0	0	0	0.004	0	t	125118	KVAAEQSLITVEGDK	Homo sapiens
1	1.00	1	1	0	0	0	0.004	0	t	118549	PEPAKSAPAPKKGSKKAV	Mus musculus
1	1.00	1	1	0	0	0	0.004	0	t	117742	AKAQTDREDLRTLLRY	Homo sapiens
1	1.00	1	1	0	0	0	0.004	0	t	118994	RGRGRGRGRGGGRPGAPGGS	Homo sapiens
1	1.00	1	1	0	0	0	0.004	0	t	118809	GGRPGAPGGSGSGPRHRDGV	Homo sapiens
1	1.00	1	1	0	0	0	0.004	0	t	119608	LGTWTYDGSVVAINPES	Homo sapiens
1	1.00	1	1	0	0	0	0.004	0	t	117742	AKAQTDREDLRTLLRY	Homo sapiens
1	1.00	1	1	0	0	0	0.004	0	t	121400	IDNSNTPFLPKIFIK	Homo sapiens
1	1.00	1	1	0	0	0	0.004	0	t	120738	LAIRNDEELNKLLGKV	Homo sapiens
1	1.00	1	1	0	0	0	0.004	0	t	117888	GQILDILVSRSLK	Homo sapiens
1	1.00	1	1	0	0	0	0.004	0	t	117745	ALLRNLGKMTA	Homo sapiens

1	1.00	1	1	0	0	0	0.004	0	t	120066	LKQQWVDYNLKWNPDD	Homo sapiens
1	1.00	1	1	0	0	0	0.004	0	t	117746	ALREYRKKMDIPAK	Homo sapiens
1	1.00	1	1	0	0	0	0.004	0	t	118814	GMMPAPHM	Homo sapiens
1	1.00	1	1	0	0	0	0.004	0	t	118004	LRLSHLKPS	Homo sapiens
1	1.00	1	1	0	0	0	0.004	0	t	117695	YITTIAGVM	Homo sapiens
1	1.00	1	1	0	0	0	0.004	0	t	131545	LMKINSD	Homo sapiens
1	1.00	1	1	0	0	0	0.004	0	t	118144	SLCQSLRER	Homo sapiens
1	1.00	1	1	0	0	0	0.004	0	t	120641	IQTAVRLLLPGELAKHAVSE	Homo sapiens
1	1.00	1	1	0	0	0	0.004	0	t	118549	PEPAKSAPAPKKGSKKAV	Mus musculus
1	1.00	1	1	0	0	0	0.004	0	t	119580	FMESGEWVIKESRGWKH	Homo sapiens
1	1.00	1	1	0	0	0	0.004	0	t	117973	LCG	Homo sapiens
1	1.00	1	1	0	0	0	0.004	0	t	123608	PPAQPLSE	Homo sapiens
1	1.00	1	1	0	0	0	0.004	0	t	118164	STKQAAFKAV	Homo sapiens
1	1.00	1	1	0	0	0	0.004	0	t	119579	FKDYSSVVRPVEDHRQ	Homo sapiens
1	1.00	1	1	0	0	0	0.004	0	t	118947	PFSAPPPA	Homo sapiens
1	1.00	1	1	0	0	0	0.004	0	t	118827	GSGPRHRDGVRRPQKRPSCI	Homo sapiens
1	1.00	1	1	0	0	0	0.004	0	t	121719	PSGKKCIAAKKIKQS	Homo sapiens
1	1.00	1	1	0	0	0	0.004	0	t	133776	AGDLSKVDAKQPGDY	Homo sapiens
1	1.00	1	1	0	0	0	0.004	0	t	119633	QYTGHITWTPPAIFKS	Homo sapiens
1	1.00	1	1	0	0	0	0.004	0	t	123386	KPKSQETP	Homo sapiens
1	1.00	1	1	0	0	0	0.004	0	t	118616	SVSYDDWDYSLEARV	Homo sapiens

1	1.00	1	1	0	0	0	0.004	0	t	133995	AEVETDKATVGFE	Homo sapiens
1	1.00	1	1	0	0	0	0.004	0	t	120772	LMLLAQMRRISLFS	Homo sapiens
1	1.00	1	1	0	0	0	0.004	0	t	120727	KYFPEVKTKGMAAVPKL	Homo sapiens
1	1.00	1	1	0	0	0	0.004	0	t	131369	AWHQQQP	Homo sapiens
1	1.00	1	1	0	0	0	0.004	0	t	131413	ERILTIS	Homo sapiens
1	1.00	1	1	0	0	0	0.004	0	t	118021	MKCGMWGRA	Homo sapiens
1	1.00	1	1	0	0	0	0.004	0	t	122874	PPARALKRSLWVEVDLLIGS	Homo sapiens
1	1.00	1	1	0	0	0	0.004	0	t	124604	QIERDTERDRFLSAPEAV	Homo sapiens
1	1.00	1	1	0	0	0	0.004	0	t	131766	YCQTWGS	Homo sapiens
1	1.00	1	1	0	0	0	0.004	0	t	131688	SYSCQVT	Homo sapiens
1	1.00	1	1	0	0	0	0.004	0	t	118550	PEPAKSAPAPKKGSKKAV	Mus musculus
1	1.00	1	1	0	0	0	0.004	0	t	119563	EDHRQVVEVTVGLQLI	Homo sapiens
1	1.00	1	1	0	0	0	0.004	0	t	131686	SYLSLTP	Homo sapiens
1	1.00	1	1	0	0	0	0.004	0	t	118335	EQLRILGEKEAKLAQQSQA	Homo sapiens
1	1.00	1	1	0	0	0	0.004	0	t	132528	PHGTQCLAMGWGRVG	Homo sapiens
1	1.00	1	1	0	0	0	0.004	0	t	132112	VLLPAVDFSL	Homo sapiens
1	1.00	1	1	0	0	0	0.004	0	t	121503	KRLKISKKPKDPEPP	Homo sapiens
1	1.00	1	1	0	0	0	0.004	0	t	121754	QKQEKKRLKISKKPK	Homo sapiens
1	1.00	1	1	0	0	0	0.004	0	t	119598	INPESDQPDLSNFMESG	Homo sapiens
1	1.00	1	1	0	0	0	0.004	0	t	124068	ELMALHTGQSLEQIER	Homo sapiens

1	1.00	1	1	0	0	0	0.004	0	t	122497	NGSSESRRALQDSQR	Homo sapiens
1	1.00	1	1	0	0	0	0.004	0	t	125357	AEIETDKATIGFEVQEEG	Homo sapiens
1	1.00	1	1	0	0	0	0.004	0	t	121173	CIAAKKIKQSVGNKS	Homo sapiens
1	1.00	1	1	0	0	0	0.004	0	t	130448	AYAKDVKFGADA	Homo sapiens
1	1.00	1	1	0	0	0	0.004	0	t	119274	MNRWALGNLACDLW	Homo sapiens
1	1.00	1	1	0	0	0	0.004	0	t	121925	TPFLPKIFIKPNAQK	Homo sapiens
1	1.00	1	1	0	0	0	0.004	0	t	131504	KATLVCLISDFYPGAV	Homo sapiens
1	1.00	1	1	0	0	0	0.004	0	t	118554	PPGIRGP	Homo sapiens
1	1.00	1	1	0	0	0	0.004	0	t	117449	CVP	Homo sapiens
1	1.00	1	1	0	0	0	0.004	0	t	119011	SGPEGSGGSGPQRRGGDNHG	Homo sapiens
1	1.00	1	1	0	0	0	0.004	0	t	118648	VCAVCRQRFLLKNLR	Homo sapiens
1	1.00	1	1	0	0	0	0.004	0	t	132526	PGSHFCGGTLIHPSF	Homo sapiens
1	1.00	1	1	0	0	0	0.004	0	t	133870	LAKLAGGVAVICAGA	Homo sapiens
1	1.00	1	1	0	0	0	0.004	0	t	118185	TSQFTGLKC	Homo sapiens
1	1.00	1	1	0	0	0	0.004	0	t	119925	HFPFDEQNGSMKLGTWT	Homo sapiens
1	1.00	1	1	0	0	0	0.004	0	t	121542	LFKPPKYHPDVPYVKR	Homo sapiens
1	1.00	1	1	0	0	0	0.004	0	t	118374	GKALCWVCAQSRKHRD	Homo sapiens
1	1.00	1	1	0	0	0	0.004	0	t	133798	DKIRGEGMRYIIILD	Homo sapiens
1	1.00	1	1	0	0	0	0.004	0	t	125515	PDLTKVYSTDIFFILEITDN	Homo sapiens

1	1.00	1	1	0	0	0	0.004	0	t	120857	PATGGVKKPHRYRPGT	Homo sapiens
1	1.00	1	1	0	0	0	0.004	0	t	125413	FRVTCKDIQRIPSLPPSTQT	Homo sapiens
1	1.00	1	1	0	0	0	0.004	0	t	125386	DVSQTSVTALPSKGLEHLKE	Homo sapiens
1	1.00	1	1	0	0	0	0.004	0	t	118930	MSDEGPGTGPGNGLGEKGDT	Homo sapiens
1	1.00	1	1	0	0	0	0.004	0	t	122401	HSEAETGPP	Homo sapiens
1	1.00	1	1	0	0	0	0.004	0	t	131318	VPSTPPTPSPSTPPTPSPS	Homo sapiens
1	1.00	1	1	0	0	0	0.004	0	t	122145	LTLFPSRDTPLSSQTD	Homo sapiens
1	1.00	1	1	0	0	0	0.004	0	t	117540	KEEGSRSNLGWL	Homo sapiens
1	1.00	1	1	0	-0.002	0	0.004	0	t	117736	AEMKSLEEKIGCLLK	Mus musculus
1	1.00	1	1	0	0	0	0.004	0	t	121858	SKLIKIFQDHPLQKTYN	Homo sapiens
1	1.00	1	1	0	0	0	0.004	0	t	118824	GRRPFFHPVGEADYFEYHQE	Homo sapiens
1	1.00	1	1	0	0	0	0.004	0	t	117736	AEMKSLEEKIGCLLK	Homo sapiens
1	1.00	1	1	0	0	0	0.004	0	t	121357	GTQVHGTI	Homo sapiens
1	1.00	1	1	0	0	0	0.004	0	t	117594	NWHQLHGKKEAY	Homo sapiens
1	1.00	1	1	0	0	0	0.004	0	t	131412	ERGLRYL	Homo sapiens
1	1.00	1	1	0	0	0	0.004	0	t	130538	LDVPPALADFIHQQR	Homo sapiens
1	1.00	1	1	0	0	0	0.004	0	t	132729	VVTFFCRPHNICTFV	Homo sapiens
1	1.00	1	1	0	0	0	0.004	0	t	121034	VHPDTGISSKAMGIMN	Homo sapiens
1	1.00	1	1	0	0	0	0.004	0	t	118189	TYYIKEQKLGL	Homo sapiens
1	1.00	1	1	0	0	0	0.004	0	t	118217	VTKYITKGWKEVH	Homo sapiens

1	1.00	1	1	0	0	0	0.004	0	t	122135	LDSRRSYRVRESGLNGVVKH	Homo sapiens
1	1.00	1	1	0	0	0	0.004	0	t	117456	DGQFCH	Homo sapiens
1	1.00	1	1	0	0	0	0.004	0	t	121945	TYKTGHGLRGKLKWRP	Homo sapiens
1	1.00	1	1	0	0	0	0.004	0	t	133822	ERPLKQIAFNGGLEP	Homo sapiens
1	1.00	1	1	0	0	0	0.004	0	t	118948	PGAIEQGPADDPGEGPSTGP	Homo sapiens
1	1.00	1	1	0	0	0	0.004	0	t	133638	LTSRPA	Homo sapiens
1	1.00	1	1	0	0	0	0.004	0	t	117910	HLLTNHLKSKEVWKAL	Homo sapiens
1	1.00	1	1	0	0	0	0.004	0	t	132575	QMRGNPGSHFCGGTL	Homo sapiens
1	1.00	1	1	0	0	0	0.004	0	t	131010	PSTRWTELDDVKRLLKGSRS	Homo sapiens
1	1.00	1	1	0	0	0	0.004	0	t	132099	TLNELPNRT	Homo sapiens
1	1.00	1	1	0	0	0	0.004	0	t	117509	GQIPPGAM	Homo sapiens
1	1.00	1	1	0	0	0	0.004	0	t	133871	LAKLGGAGQAARAGT	Homo sapiens
1	1.00	1	1	0	0	0	0.004	0	t	125411	FQGLCNETLTLKLYNNGFTS	Homo sapiens
1	1.00	1	1	0	0	0	0.004	0	t	121589	LRKFKNISYFPGKLR	Homo sapiens
1	1.00	1	1	0	0	0	0.004	0	t	125540	QSLRQRKSVNALNSPLHQEY	Homo sapiens
1	1.00	1	1	0	0	0	0.004	0	t	131358	APSVTLF	Homo sapiens
1	1.00	1	1	0	0	0	0.004	0	t	122271	ASRETKELRQEERQP	Homo sapiens
1	1.00	1	1	0	0	0	0.004	0	t	120711	KQLATKAARKSAPAT	Homo sapiens
1	1.00	1	1	0	0	0	0.004	0	t	131365	ATLVCLI	Homo sapiens
1	1.00	1	1	0	0	0	0.004	0	t	131753	WKAPSSP	Homo sapiens

1	1.00	1	1	0	0	0	0.004	0	t	120930	RYRPGTVA LREIRRYQ	Homo sapiens
1	1.00	1	1	0	0	0	0.004	0	t	120612	IFNLFSTKD SSAAW	Homo sapiens
1	1.00	1	1	0	0	0	0.004	0	t	122825	KSTAKDCT FKPDFEMT VKE	Homo sapiens
1	1.00	1	1	0	0	0	0.004	0	t	118133	SESQTLDK VYQM	Homo sapiens
1	1.00	1	1	0	0	0	0.004	0	t	119741	AIFKSYGEII VTHFPFD	Homo sapiens
1	1.00	1	1	0	0	0	0.004	0	t	119641	SEHETRLV AKLFKDYS	Homo sapiens
1	1.00	1	1	0	0	0	0.004	0	t	121161	AVEKVKRT KDE	Homo sapiens
1	1.00	1	1	0	0	0	0.004	0	t	133795	DFTYDQV AFNGLPQF	Homo sapiens
1	1.00	1	1	0	0	0	0.004	0	t	123851	WTEVSEV ATEV	Homo sapiens
1	1.00	1	1	0	0	0	0.004	0	t	118817	GNGLGEK GDTSGPE GSGGSG	Homo sapiens
1	1.00	1	1	0	0	0	0.004	0	t	125395	EHLKELIAR NTWTLKKL PLS	Homo sapiens
1	1.00	1	1	0	0	0	0.004	0	t	121112	AACDPRH GRYLTVA	Homo sapiens
1	1.00	1	1	0	0	0	0.004	0	t	118770	DPGEGPST GPRGQGD GGRRK	Homo sapiens
1	1.00	1	1	0	0	0	0.004	0	t	118158	SRNLYDIG E	Homo sapiens
1	1.00	1	1	0	0	0	0.004	0	t	121136	ALAVTKYK QRNGWS HK	Homo sapiens
1	1.00	1	1	0	0	0	0.004	0	t	117773	CNSRQTD REDELI	Homo sapiens
1	1.00	1	1	0	0	0	0.004	0	t	131344	AIGDRSKT DRENSVSI G	Homo sapiens
1	1.00	1	1	0	0	0	0.004	0	t	128512	EGPPPKDT	Homo sapiens

1	1.00	1	1	0	0	0	0.004	0	t	120699	KMFPEVKEKGMAALPRL	Homo sapiens
1	1.00	1	1	0	0	0	0.004	0	t	131657	SGLQAEDEADYYCSSY	Homo sapiens
1	1.00	1	1	0	0	0	0.004	0	t	122198	TEADVNPKPIPSQMPTSP	Homo sapiens
1	1.00	1	1	0	0	0	0.004	0	t	122173	QSIQQSIERLWCRLWPLPFP	Homo sapiens
1	1.00	1	1	0	0	0	0.004	0	t	132262	ELNVTVVTFFCRPHN	Homo sapiens
1	1.00	1	1	0	0	0	0.004	0	t	119013	SGSPPRRPPPGRRPFFHPVG	Homo sapiens
1	1.00	1	1	0	0	0	0.004	0	t	122377	GLPRLETPADLSTAIASRS	Homo sapiens
1	1.00	1	1	0	0	0	0.004	0	t	119004	RRPQKRPSCIGCKGTHGGTG	Homo sapiens
1	1.00	1	1	0	-0.002	0	0.004	0	t	117948	KFVETPGQKYKETDL	Mus musculus
1	1.00	1	1	0	0	0	0.004	0	t	120168	RGWKHSVTYSGGPDTPY	Homo sapiens
1	1.00	1	1	0	0	0	0.004	0	t	121347	GPLTVAQKKAQNIME	Homo sapiens
1	1.00	1	1	0	0	0	0.004	0	t	120682	KILPEVKEKHEFLSRL	Homo sapiens
1	1.00	1	1	0	0	0	0.004	0	t	121444	KAKSETFRLLHAKNI	Homo sapiens
1	1.00	1	1	0	0	0	0.004	0	t	121499	KQKKHLNTQQLTAFQ	Homo sapiens
1	1.00	1	1	0	0	0	0.004	0	t	121715	PNKQTPSGKKCIAAK	Homo sapiens
1	1.00	1	1	0	0	0	0.004	0	t	117781	DCT	Homo sapiens
1	1.00	1	1	0	0	0	0.004	0	t	117779	CVSYRNPAE	Homo sapiens
1	1.00	1	1	0	0	0	0.004	0	t	119837	ELIPSTSSAVPLIGK	Homo sapiens

1	1.00	1	1	0	0	0	0.004	0	t	114207	FLGELTSSEVATEV	Homo sapiens
1	1.00	1	1	0	0	0	0.004	0	t	115631	NVRFSDEGGFTC	Homo sapiens
1	1.00	1	1	0	0	0	0.004	0	t	103834	CPRDLNGKSGC	Homo sapiens
1	1.00	1	1	0	0	0	0.004	0	t	107856	GGQGSRHQQAR	Homo sapiens
1	1.00	1	1	0	0	0	0.004	0	t	117140	DWEYSVWLSN	Homo sapiens
1	1.00	1	1	0	0	0	0.004	0	t	112319	SKIFKLGGRDSRSGSPMARR	Homo sapiens
1	1.00	1	1	0	0	0	0.004	0	t	115784	HRTFE	Homo sapiens
1	1.00	1	1	0	0	0	0.004	0	t	116927	AVCRTSMCSIQSAPP	Homo sapiens
1	1.00	1	1	0	0	0	0.004	0	t	113209	VIVEMNSSVEAIEGSHVSLL	Homo sapiens
1	1.00	1	1	0	0	0	0.004	0	t	104798	GERAKRS	Homo sapiens
1	1.00	1	1	0	0	0	0.004	0	t	114973	VHFFKNIVTPRTE	Homo sapiens
1	1.00	1	1	0	0	0	0.004	0	t	108874	DPISGHVSLF	Homo sapiens
1	1.00	1	1	0	0	0	0.004	0	t	109767	QPLKEQPALNDSRYCL	Homo sapiens
1	1.00	1	1	0	0	0	0.004	0	t	109865	RSLDFQATTMF	Homo sapiens
1	1.00	1	1	0	0	0	0.004	0	t	115601	DPFYWVSPGVLV	Homo sapiens
1	1.00	1	1	0	0	0	0.004	0	t	117315	KALSVETEKLLKYLEAV	Homo sapiens
1	1.00	1	1	0	0	0	0.004	0	t	115912	QAPEY	Homo sapiens
1	1.00	1	1	0	0	0	0.004	0	t	102316	AQVATISANGDK	Homo sapiens
1	1.00	1	1	0	0	0	0.004	0	t	115153	MKGVVCTRIYEKV	Homo sapiens
1	1.00	1	1	0	0	0	0.004	0	t	106990	VYATRSSAVRLRSSVP	Homo sapiens
1	1.00	1	1	0	0	0	0.004	0	t	109938	SPRSGDLSVY	Homo sapiens
1	1.00	1	1	0	0	0	0.004	0	t	117141	FQFFQRDAEELEKW	Homo sapiens

1	1.00	1	1	0	0	0	0.004	0	t	115603	ELKVEDPF YWVS	Homo sapiens
1	1.00	1	1	0	0	0	0.004	0	t	115653	SSYAGQFR VIGP	Homo sapiens
1	1.00	1	1	0	0	0	0.004	0	t	117246	VTWEVLE GEVEKEAL KKI	Homo sapiens
1	1.00	1	1	0	0	0	0.004	0	t	108498	KIHAREIFD SRGNPTV	Homo sapiens
1	1.00	1	1	0	0	0	0.004	0	t	115628	LPCRISPGK NAT	Homo sapiens
1	1.00	1	1	0	0	0	0.004	0	t	110082	VYFCASSF STCSANYG	Homo sapiens
1	1.00	1	1	0	0	0	0.004	0	t	109427	KPISGHNS LF	Homo sapiens
1	1.00	1	1	0	0	0	0.004	0	t	112945	DSYTLTEEL AYAEIRVK	Homo sapiens
1	1.00	1	1	0	0	0	0.004	0	t	117348	PTRAPSGP RPP	Homo sapiens
1	1.00	1	1	0	0	0	0.004	0	t	106634	LVGPRGER GFP	Homo sapiens
1	1.00	1	1	0	0	0	0.004	0	t	115607	GPRHPIRA LVGD	Homo sapiens
1	1.00	1	1	0	0	0	0.004	0	t	107896	HSTSQEG QDTIHGH RGS	Homo sapiens
1	1.00	1	1	0	0	0	0.004	0	t	107087	ARGLTGRP GDA	Homo sapiens
1	1.00	1	1	0	0	0	0.004	0	t	107892	HSASQDG QDTIRGHP GSS	Homo sapiens
1	1.00	1	1	0	0	0	0.004	0	t	115057	GLSLSRFS WGAEGQR PGFG	Homo sapiens
1	1.00	1	1	0	0	0	0.004	0	t	115639	QFRVIGPR HPIR	Homo sapiens
1	1.00	1	1	0	0	0	0.004	0	t	109917	SHQESTRG RSRGRSGR SGS	Homo sapiens
1	1.00	1	1	0	0	0	0.004	0	t	115598	ATGMEVG WYRPP	Homo sapiens
1	1.00	1	1	0	0	0	0.004	0	t	115626	LKDAIGEG KVTL	Homo sapiens
1	1.00	1	1	0	0	0	0.004	0	t	115832	KTGQFL	Homo sapiens

1	1.00	1	1	0	0	0	0.004	0	t	104926	PDGSRLV	Homo sapiens
1	1.00	1	1	0	0	0	0.004	0	t	117366	RRREGPDR SPR	Homo sapiens
1	1.00	1	1	0	0	0	0.004	0	t	115677	AHRET	Homo sapiens
1	1.00	1	1	0	0	0	0.004	0	t	104938	PSRLVNQ	Homo sapiens
1	1.00	1	1	0	0	0	0.004	0	t	106675	NEEGFFSA RGHRPLDK K	Homo sapiens
1	1.00	1	1	0	0	0	0.004	0	t	117315	KALSVETE KLLKYLEAV	Homo sapiens
1	1.00	1	1	0	0	0	0.004	0	t	108834	CKPISGHN SLFWYRQT	Homo sapiens
1	1.00	1	1	0	0	0	0.004	0	t	115619	IRALVGDE VELP	Homo sapiens
1	1.00	1	1	0	0	0	0.004	0	t	115649	SPGKNAT GMEVG	Homo sapiens
1	1.00	1	1	0	0	0	0.004	0	t	116024	VTLRI	Homo sapiens
1	1.00	1	1	0	0	0	0.004	0	t	115633	PPFSRVVH LYRN	Homo sapiens
1	1.00	1	1	0	0	0	0.004	0	t	117300	GSGKGKV QFQGKKT KF	Homo sapiens
1	1.00	1	1	0	0	0	0.004	0	t	107799	DSGHRGY SGSQASD NEGH	Homo sapiens
1	1.00	1	1	0	0	0	0.004	0	t	109948	SRYCLSSRL RVSATFW	Homo sapiens
1	1.00	1	1	0	0	0	0.004	0	t	108844	DAGVIQSP RHEVTEM G	Homo sapiens
1	1.00	1	1	0	0	0	0.004	0	t	114941	RQDENYEE AIVHLKVA ALGS	Homo sapiens
1	1.00	1	1	0	0	0	0.004	0	t	115645	RNGKDQD GDQAP	Homo sapiens
1	1.00	1	1	0	0	0	0.004	0	t	115605	GDEVELPC RISP	Homo sapiens
1	1.00	1	1	0	0	0	0.004	0	t	107800	DSRGNPT VE	Homo sapiens

1	1.00	1	1	0	0	0	0.004	0	t	113912	TCFFRDHS YQEE	Homo sapiens
1	1.00	1	1	0	0	0	0.004	0	t	114746	ASQKRPSQ RSKYLATA STMD	Homo sapiens
1	1.00	1	1	0	0	0	0.004	0	t	117329	MAVPETR PNHT	Homo sapiens
1	1.00	1	1	0	0	0	0.004	0	t	117331	MKLVRFL MKLSHETV TIELK	Homo sapiens
1	1.00	1	1	0	0	0	0.004	0	t	115785	HSYQE	Homo sapiens
1	1.00	1	1	0	0	0	0.004	0	t	105031	VRGRGSK	Homo sapiens
1	1.00	1	1	0	0	0	0.004	0	t	108363	FLAEGGGV RGPRVVER H	Homo sapiens
1	1.00	1	1	0	0	0	0.004	0	t	117333	MVTSTITE KLLKNLVKI	Homo sapiens
1	1.00	1	1	0	-0.002	0	0.004	0	t	108834	CKPISGHN SLFWYRQT	Mus musculu s
1	1.00	1	1	0	0	0	0.004	0	t	115674	ADARM	Homo sapiens
1	1.00	1	1	0	0	0	0.004	0	t	115591	APEYRGRT ELLK	Homo sapiens
1	1.00	1	1	0	0	0	0.004	0	t	115148	KAVSEKKA KGLGESRK DKK	Homo sapiens
1	1.00	1	1	0	0	0	0.004	0	t	108125	SHQESTRG RSRGRSGR SGS	Homo sapiens
1	1.00	1	1	0	0	0	0.004	0	t	113081	LSYSHSDY DLGKRPTK DSYT	Homo sapiens
1	1.00	1	1	0	0	0	0.004	0	t	115658	VGWYRPP FSRVV	Homo sapiens
1	1.00	1	1	0	0	0	0.004	0	t	115600	DHSYQEEA AMEL	Homo sapiens
1	1.00	1	1	0	0	0	0.004	0	t	115056	GLSLSRFS WGAEGQR PGFG	Homo sapiens
1	1.00	1	1	0	0	0	0.004	0	t	115657	TLRIRNVR FSDE	Homo sapiens
1	1.00	1	1	0	0	0	0.004	0	t	74871	YLPVGGLR RIGG	Homo sapiens
1	1.00	1	1	0	0	0	0.004	0	t	65050	TLRVYK	Homo sapiens

1	1.00	1	1	0	0	0	0.004	0	t	96211	EDKKRKEEEE	Homo sapiens
1	1.00	1	1	0	0	0	0.004	0	t	96455	KGKNVLINKD	Homo sapiens
1	1.00	1	1	0	0	0	0.004	0	t	63098	TCRRMLAFLKDKENR	Homo sapiens
1	1.00	1	1	0	0	0	0.004	0	t	30661	KEVPALTAVETGAT	Homo sapiens
1	1.00	1	1	0	0	0	0.004	0	t	32159	KLSEGDLLAEIETDK	Homo sapiens
1	1.00	1	1	0	0	0	0.004	0	t	34833	LAKLAGGVAV	Homo sapiens
1	1.00	1	1	0	0	0	0.004	0	t	29197	IVAGGGVTLL	Homo sapiens
1	1.00	1	1	0	0	0	0.004	0	t	29342	IVLGGGCALL	Homo sapiens
1	1.00	1	1	0	0	0	0.004	0	t	14426	ETIGTLKKILDETVK	Homo sapiens
1	1.00	1	1	0	0	0	0.004	0	t	34836	LAKLSDGVAV	Homo sapiens
1	1.00	1	1	0	0	0	0.004	0	t	32159	KLSEGDLLAEIETDK	Homo sapiens
1	1.00	1	1	0	0	0	0.004	0	t	11081	EAIPALTAVETGHTSQV	Homo sapiens
1	1.00	1	1	0	0	0	0.004	0	t	32159	KLSEGDLLAEIETDK	Homo sapiens
1	1.00	1	1	0	0	0	0.004	0	t	23171	GVTLLQAAPT	Homo sapiens
1	1.00	1	1	0	0	0	0.004	0	t	8930	DKLKQQRDTLSTQKET	Homo sapiens
1	1.00	1	1	0.02	0.018	0.006	0.013	0.019	t	121071	YAINSEQSKE	Mus musculus
1	1.00	1	1	0.02	0.022	0.02	0.023	0.019	t	104443	DAEFRHDSGYE	Homo sapiens
1	1.00	1	1	-0.01	-0.01	-0.01	-0.01	-0.012	t	136392	IPKSRKRRIPQR	Mus musculus

1	1.00	1	1	0.02	0.02	0.02	0.02	0.02	t	79383	SCDVFLTM FYPGCK	Mus musculu s
1	1.00	1	1	-0.01	-0.007	-0.017	-0.01	-0.01	t	153904	VVEQLGH ETQIHIQIP	Mus musculu s
1	1.00	1	1	0.03	0.034	0.03	0.031	0.03	t	134696	ITLYLTEK	MD
1	1.00	1	1	-0.03	-0.03	-0.036	-0.036	-0.03	t	35962	LFRQFYQL DA	Homo sapiens
1	1.00	1	1	-0.03	-0.03	-0.033	-0.03	-0.03	t	36991	LKPIFKLPL GINITNFR	Homo sapiens
1	1.00	1	1	-0.03	-0.026	-0.025	-0.027	-0.03	t	35193	LDKAFHLL L	Homo sapiens
1	1.00	1	1	-0.01	-0.01	-0.01	-0.01	-0.01	t	5780	AYFYPELF RQ	Homo sapiens
1	1.00	1	1	0.01	0.01	0.01	0.01	0.01	t	133737	VTPYFSYP VA	Mus musculu s
1	1.00	1	1	0	0.003	-0.003	0	0.002	t	74068	YGPGVDY	Mus musculu s
1	1.00	1	1	0.05	0.05	0.05	0.05	0.051	t	72534	WHETDST YFRLP	Rattus norvegic us
1	1.00	1	1	-0.02	-0.02	-0.021	-0.014	-0.02	t	65953	TQTPVNG NSPWAPT A	Homo sapiens
1	1.00	1	1	0.01	0.01	0.008	0.01	0.009	t	65974	TQYERE	Mus musculu s
1	1.00	1	1	0.06	0.056	0.058	0.063	0.058	t	95563	LNTVNGTT RTVN	Oryctola gus cuniculu s
1	1.00	1	1	0.01	0.01	0.01	0.01	0.009	t	10801	DWEDRYY RE	Mus musculu s
1	1.00	1	1	0.02	0.02	0.02	0.027	0.022	t	125284	TYICEVED QKEE	Mus musculu s

1	1.00	1	1	-0.04	-0.038	-0.04	-0.033	-0.04	t	119913	GSLDSLRREIEQMRR	Homo sapiens
1	1.00	1	1	-0.04	-0.036	-0.04	-0.033	-0.04	t	119913	GSLDSLRREIEQMRR	Homo sapiens
1	1.00	1	1	-0.04	-0.038	-0.04	-0.033	-0.04	t	119913	GSLDSLRREIEQMRR	Homo sapiens
1	1.00	1	1	0.03	0.03	0.022	0.033	0.032	t	68900	VIAEHQFKE	Homo sapiens
1	1.00	1	1	-0.01	-0.007	-0.01	-0.01	-0.011	t	53634	RESQAYYQR	Mus musculus
1	1.00	1	1	0.03	0.03	0.03	0.03	0.03	t	123225	GGATPVVG	Oryctolagus cuniculus
1	1.00	1	1	0.03	0.027	0.018	0.03	0.033	t	114142	PQKSQRSQDEN	Oryctolagus cuniculus
1	1.00	1	1	0.03	0.018	0.018	0.03	0.033	t	114142	PQKSQRSQDEN	Macaca mulatta
1	1.00	1	1	0.07	0.07	0.061	0.063	0.07	t	110884	HEYMLDLQPETTDLYCYE	Homo sapiens
1	1.00	1	1	0.07	0.07	0.061	0.063	0.07	t	110884	HEYMLDLQPETTDLYCYE	Homo sapiens
1	1.00	1	1	0.07	0.07	0.061	0.063	0.07	t	110884	HEYMLDLQPETTDLYCYE	Homo sapiens
1	1.00	1	1	0.01	0.007	0.01	0.01	0.009	t	122289	CSNSSSSQFQIHGP	Oryctolagus cuniculus
1	1.00	1	1	0.02	0.018	0.02	0.017	0.017	t	922	AEGEFDYAWDQTHQDPAK	Mus musculus
1	1.00	1	1	-0.02	-0.02	-0.002	-0.019	-0.023	t	57860	SFP	Mus musculus
1	1.00	1	1	-0.02	-0.02	-0.002	-0.019	-0.023	t	57860	SFP	Mus musculus

1	1.00	1	1	0.01	0.01	-0.01	0.01	0.012	t	20012	GGSGGSVASGGSVAS	Homo sapiens
1	1.00	1	1	0	-0.002	-0.005	0.001	0	t	107087	ARGLTGRPGDA	Mus musculus
1	1.00	1	1	-0.01	-0.012	-0.021	-0.011	-0.01	t	29897	KANADAKAAYEAAVAANNA	Mus musculus
1	1.00	1	1	0.02	0.02	0.02	0.02	0.02	t	111351	GLQPPPGGTLEDTYR	Mus musculus
1	1.00	1	1	0.02	0.02	0.02	0.02	0.02	t	111351	GLQPPPGGTLEDTYR	Mus musculus
1	1.00	1	1	0.01	0.012	0.002	0.011	0.01	t	1460	AGCLIGAEHVDTSYECD	Homo sapiens
1	1.00	1	1	0	0.002	0	0.003	0	t	28912	ITGHRMAWDMMMNWSPTT	Homo sapiens
1	1.00	1	1	-0.04	-0.04	-0.047	-0.049	-0.043	t	130922	KGMLPDPKNV	Homo sapiens
1	1.00	1	1	-0.01	-0.01	-0.009	-0.011	-0.01	t	42633	MSPVNKSFEIEVTCS	Homo sapiens
1	1.00	1	1	0	0.002	-0.003	0	0.001	t	50621	QEIPQPQW	Anas platyrhynchos
1	1.00	1	1	-0.02	-0.02	-0.008	-0.014	-0.021	t	109245	GSAGAPGI	Rattus norvegicus
1	1.00	1	1	0.04	0.038	0.035	0.039	0.04	t	78876	VLLGRKWEDEVDDPESYF	Mus musculus
1	1.00	1	1	0.04	0.036	0.035	0.039	0.04	t	78876	VLLGRKWEDEVDDPESYF	Oryctolagus cuniculus
1	1.00	1	1	0.03	0.032	0.039	0.037	0.031	t	122194	STVQDCV	Homo sapiens

1	1.00	1	1	-0.01	-0.013	-0.017	-0.01	-0.012	t	7313	CVTIMTVDNPASTTNKDK	Oryctolagus cuniculus
1	1.00	1	1	0.04	0.04	0.04	0.04	0.04	t	103426	MTLLELSDFVKKFEE	Homo sapiens
1	1.00	1	1	0.04	0.04	0.04	0.04	0.04	t	78177	HVTDLDKEFL	Mus musculus
1	1.00	1	1	0	0	0	0	-0.002	t	21408	GNDYEDRYYRENMYRYPNQ	Mus musculus
1	1.00	1	1	0	0.002	0.002	0.007	0.003	t	119641	SEHETRLVAKLFKDYS	Homo sapiens
1	1.00	1	1	0.03	0.034	0.027	0.027	0.027	t	115485	SGEPTSTPTTEAVESTVATL	Homo sapiens
1	1.00	1	1	0.01	0.01	0.01	0.01	0.01	t	113779	QQPGQGQQ	Homo sapiens
1	1.00	1	1	0.01	0.01	0.01	0.01	0.01	t	113779	QQPGQGQQ	Homo sapiens
1	1.00	1	1	0	0	-0.009	-0.003	-0.001	t	110828	EIDGPAGQAEPDRAHYNI	Mus musculus
1	1.00	1	1	-0.02	-0.02	-0.02	-0.02	-0.021	t	97020	TVTRGTISDP	Oryctolagus cuniculus
1	1.00	1	1	-0.02	-0.018	-0.015	-0.013	-0.023	t	131898	GRPLSVQQQMMQQSR	Homo sapiens
1	1.00	1	1	-0.02	-0.022	-0.029	-0.017	-0.017	t	115540	VKNKRTFLSPWISNI	Mus musculus
1	1.00	1	1	0.1	0.1	0.098	0.1	0.101	t	114234	TQDENP	Mus musculus
1	1.00	1	1	-0.02	-0.018	-0.015	-0.013	-0.021	t	129659	PTQYPPGR	Homo sapiens

1	1.00	1	1	0.02	0.022	0.013	0.023	0.02	t	69049	VINMSLGGPSGSAAL	Cavia porcellus
1	1.00	1	1	-0.03	-0.032	-0.033	-0.031	-0.03	t	75661	YRPPNAPIL	Mus musculus
1	1.00	1	1	0.02	0.02	0.017	0.02	0.02	t	50307	QALIDQKVKEISNQ	Homo sapiens
1	1.00	1	1	0.02	0.022	0.013	0.023	0.021	t	122377	GLPRLETPADLSTAIASRS	Homo sapiens
1	1.00	1	1	0.01	0.008	0.004	0.007	0.01	t	36444	LHYTAVVKKSSAV	Mus musculus
1	1.00	1	1	-0.01	-0.008	-0.015	-0.009	-0.011	t	56804	SAGRSTGSLVRLFAPG	Homo sapiens
1	1.00	1	1	-0.01	-0.01	-0.01	-0.01	-0.012	t	152777	LLNIDKDIRK	Homo sapiens
1	1.00	1	1	0.03	0.03	0.03	0.03	0.03	t	147290	SKSYTSILYGNGP	Homo sapiens
1	1.00	1	1	0.03	0.03	0.03	0.03	0.03	t	103687	VCAVDMPYLTVELIE	Homo sapiens
1	1.00	1	1	0.01	0.01	0.01	0.01	0.01	t	112336	SSQSVLY	Homo sapiens
1	1.00	1	1	-0.05	-0.052	-0.067	-0.053	-0.049	t	35956	LFREDGSACR	Mus musculus
1	1.00	1	1	0	0	-0.005	0	0	t	95414	GLDLDVNVFR	Oryctolagus cuniculus
1	1.00	1	1	-0.02	-0.018	-0.016	-0.013	-0.019	t	144697	IYRNLYWEGS	Homo sapiens
1	1.00	1	1	0	0	0	0.001	-0.001	t	60116	SPLLGCIGSTCAE	Mus musculus
1	1.00	1	1	0	0	0	0	0	t	153837	HGRLVTLKDIVLDLQP	Cavia porcellus

1	1.00	1	1	0	0	0	0	0	t	153837	HGRLVTLKDIVLDLQP	Cavia porcellus
1	1.00	1	1	0.03	0.03	0.032	0.03	0.029	t	119845	ESDQPDLSNFMESGEW	Oryctolagus cuniculus
1	1.00	1	1	0.03	0.027	0.032	0.03	0.029	t	119845	ESDQPDLSNFMESGEW	Oryctolagus cuniculus
1	1.00	1	1	0.03	0.03	0.032	0.03	0.029	t	119845	ESDQPDLSNFMESGEW	Mus musculus
1	1.00	1	1	0.03	0.032	0.032	0.03	0.029	t	119845	ESDQPDLSNFMESGEW	Rattus norvegicus
1	1.00	1	1	0.03	0.03	0.032	0.03	0.029	t	119845	ESDQPDLSNFMESGEW	Mus musculus
1	1.00	1	1	0.03	0.033	0.032	0.03	0.029	t	119845	ESDQPDLSNFMESGEW	Mus musculus
1	1.00	1	1	-0.01	-0.01	-0.01	-0.01	-0.01	t	60795	SRSERR	Homo sapiens
1	1.00	1	1	-0.02	-0.017	-0.015	-0.02	-0.022	t	19626	GFTDRAFWVCPGVVC	Mus musculus
1	1.00	1	1	0	-0.002	0.005	-0.001	0	t	70162	VNPTYGN	Mus musculus
1	1.00	1	1	-0.02	-0.02	-0.02	-0.023	-0.021	t	66932	TVAEEHV	Homo sapiens
1	1.00	1	1	-0.03	-0.028	-0.037	-0.027	-0.029	t	144816	WSHNFTNMLG	Homo sapiens
1	1.00	1	1	-0.03	-0.028	-0.025	-0.023	-0.031	t	126757	NKPLITVTNSKY	Homo sapiens
1	1.00	1	1	0.06	0.06	0.06	0.06	0.058	t	99945	DIRDSLSEEE	Mus musculus

1	1.00	1	1	0	-0.002	0	0.003	-0.001	t	122406	IEIGLEGKGFEPTLEALFGK	Mus musculus
1	1.00	1	1	0	0	0	0	0	t	102371	DSVFYLDNLLPSTLDYFTYL	Mus musculus
1	1.00	1	1	-0.02	-0.018	-0.02	-0.017	-0.018	t	133638	LTSRPA	Homo sapiens
1	1.00	1	1	0.01	0.01	0.012	0.013	0.011	t	139499	FGEFSFQYDCSSWGC	Homo sapiens
1	1.00	1	1	0.01	0.01	0.015	0.01	0.01	t	56273	RVEFLV	Homo sapiens
1	1.00	1	1	0.01	0.01	0.01	0.01	0.006	t	151731	SAQSQRAPDR	Mus musculus
1	1.00	1	1	-0.01	-0.01	-0.01	-0.01	-0.01	t	156909	LAHPFTP	Gallus gallus
1	1.00	1	1	-0.01	-0.01	-0.01	-0.01	-0.01	t	106780	RKYFG	Homo sapiens
1	1.00	1	1	0.04	0.033	0.036	0.04	0.04	t	55627	RRKLTNPANKEES	Mus
1	1.00	1	1	0.02	0.018	0.02	0.019	0.02	t	1109	AENSAPTA	Mus musculus
1	1.00	1	1	-0.01	-0.008	-0.015	-0.007	-0.01	t	20391	GIPDGWP	Felis catus
1	1.00	1	1	0.05	0.05	0.048	0.049	0.052	t	153265	ILETLPANPPPASTN	Mus musculus
1	1.00	1	1	-0.01	-0.008	-0.007	-0.007	-0.01	t	94613	NNPTCWAICKRI	Homo sapiens
1	1.00	1	1	-0.01	-0.01	-0.007	-0.01	-0.008	t	14907	EVWVTPVI	Homo sapiens

1	1.00	1	1	0.02	0.022	0.017	0.02	0.02	t	46165	NTLQNQGA	Anas platyrhynchos
1	1.00	1	1	0	-0.003	0	0	-0.001	t	32889	KPTDG	Oryctolagus cuniculus
1	1.00	1	1	0.03	0.03	0.03	0.03	0.029	t	95300	DALNEN	Homo sapiens
1	1.00	1	1	0.01	0.01	0.01	0.01	0.008	t	10853	DYAWDQTHQ	Mus musculus
1	1.00	1	1	0.03	0.032	0.024	0.027	0.028	t	6722	CNRAFNPVCGTDGVTYDN	Homo sapiens
1	1.00	1	1	0.01	0.013	0.01	0.01	0.011	t	41233	MDIDPYKEFGA	Mus musculus
1	1.00	1	1	0.02	0.02	0.02	0.02	0.02	t	156877	GLHSGIQ	Gallus gallus
1	1.00	1	1	0.03	0.03	0.03	0.03	0.03	t	118824	GRRPFFHPVGEADYFEYHQE	Homo sapiens
1	1.00	1	1	0.03	0.03	0.03	0.03	0.03	t	118824	GRRPFFHPVGEADYFEYHQE	Homo sapiens
1	1.00	1	1	0	0	-0.011	0	0	t	38753	LPVGQIRNVPHPFVA	Mus musculus
1	1.00	1	1	0	0.002	0	0.001	0	t	63831	TGGAAAR	Homo sapiens
1	1.00	1	1	-0.02	-0.022	-0.02	-0.023	-0.02	t	122138	LKKDV	Mus musculus
1	1.00	1	1	0.01	0.008	-0.009	0.013	0.013	t	33450	KSNAATSYINAIINSKSVSD	Rattus norvegicus
1	1.00	1	1	0.01	0.006	0.004	0.013	0.012	t	12259	EGRFKSQDAD	Oryctolagus cuniculus
1	1.00	1	1	0.02	0.02	0.017	0.023	0.02	t	78845	TATKQAEAAAPVVESKWRA	Homo sapiens

1	1.00	1	1	0.01	0.012	0.008	0.017	0.01	t	6918	CRFPNITN SHVPILQE	Homo sapiens
1	1.00	1	1	0	0.002	0	0.003	0	t	42696	MSTNPKP QRKTKRNT	Homo sapiens
1	1.00	1	1	0	-0.002	-0.012	-0.003	0	t	79867	INNLIVELI RGT	Mus musculu s
1	1.00	1	1	0.03	0.035	0.037	0.03	0.028	t	132869	LSPLGEEM	Mus musculu s
1	1.00	1	1	-0.03	-0.028	-0.03	-0.023	-0.029	t	131075	SQSYPEPA RGSVPMP	Homo sapiens
1	1.00	1	1	-0.02	-0.02	-0.032	-0.02	-0.02	t	159035	NRFEHQM WESVRDE LN	Mus musculu s
1	1.00	1	1	-0.01	-0.01	-0.015	-0.01	-0.01	t	125055	FVYGGCQ GNN	Mus musculu s
1	1.00	1	1	0.02	0.018	0.02	0.019	0.02	t	75151	YNGEEYLIL S	Mus musculu s
1	1.00	1	1	-0.01	-0.01	-0.01	-0.013	-0.01	t	26619	IIPDREVLY	Homo sapiens
1	1.00	1	1	0.01	0.009	0.003	0.01	0.01	t	25654	IDQLTADK AKADAELA A	Equus caballus
1	1.00	1	1	0	0	0	0	-0.002	t	136686	TLHDMGIF SITSSDS	Mus musculu s
1	1.00	1	1	0	0	0	0	-0.002	t	147083	LCSEYRNT QIYTIN	Oryctola gus cuniculu s
1	1.00	1	1	0	0	0	0	-0.002	t	134333	CIPIPSSW AFARFLW	Homo sapiens
1	1.00	1	1	0	0	0	0	-0.002	t	151816	CLTSQAAK QRLE	Sus scrofa
1	1.00	1	1	0	0	0	0	-0.002	t	150200	FGEHKQEK DLEYGAC	Mus musculu s

1	1.00	1	1	0	0	0	0	-0.002	t	140033	HDFR	Homo sapiens
1	1.00	1	1	0	0	0	0	-0.002	t	136686	TLHDMGIF SITSSDS	Mus musculu s
1	1.00	1	1	0	0	0	0	-0.002	t	151609	DETPMRG ETRKV	Homo sapiens
1	1.00	1	1	0	0	0	0	-0.002	t	152057	QAARQRL EPGRN	Sus scrofa
1	1.00	1	1	0	0	0	0	-0.002	t	140256	KYEQGGNI ID	Mus musculu s
1	1.00	1	1	0	0	0	0	-0.002	t	136686	TLHDMGIF SITSSDS	Mus musculu s
1	1.00	1	1	0	0	0	0	-0.002	t	140479	KHGLLSM	Mus musculu s
1	1.00	1	1	0	0	0	0	-0.002	t	151817	CLTSQAAS QRLE	Sus scrofa
1	1.00	1	1	0	0	0	0	-0.002	t	138840	DREQAPN LVYM	Mus musculu s
1	1.00	1	1	0	0	0	0	-0.002	t	134702	KEKKYS	Mus musculu s
1	1.00	1	1	0	0	0	0	-0.002	t	141963	MVDRGW G	Mus musculu s
1	1.00	1	1	0	0	0	0	-0.002	t	140026	FVFNLKD MFSRANR KRKA	Oryctola gus cuniculu s
1	1.00	1	1	0	0	0	0	-0.002	t	136315	GGGTGPA DGTNATTI	Mus musculu s
1	1.00	1	1	0	0	0	0	-0.002	t	140479	KHGLLSM	Mus musculu s
1	1.00	1	1	0	0	0	0	-0.002	t	141786	YGEESSRR GDLAALAR RV	Mus musculu s
1	1.00	1	1	0	0	0	0	-0.002	t	141726	PTAPPLLP HS	Rattus norvegic us

1	1.00	1	1	0	0	0	0	-0.002	t	134767	QDPYVKEAENLKKYFNAG	Mus musculus
1	1.00	1	1	0	0	0	0	-0.002	t	140258	KYEQGGNIVD	Mus musculus
1	1.00	1	1	0	0	0	0	-0.002	t	140488	MSLLGHK	Mus musculus
1	1.00	1	1	0	0	0	0	-0.002	t	150163	DGYPTFGEHKQEKDL	Mus musculus
1	1.00	1	1	0	0	0	0	-0.002	t	137759	NQEDAEAITHLF	Mus musculus
1	1.00	1	1	0	0	0	0	-0.002	t	136686	TLHDMGIFSITSSDS	Mus musculus
1	1.00	1	1	0	0	0	0	-0.002	t	152903	PYSDFEKVTGRIDKNV	Homo sapiens
1	1.00	1	1	0	0	0	0	-0.002	t	153251	AKAFSYWTPFDGKSLYSST	Mus musculus
1	1.00	1	1	0	0	0	0	-0.002	t	134685	GVSSYMKVSICLPMDVE	Oryctolagus cuniculus
1	1.00	1	1	0	0	0	0	-0.002	t	152917	QLPELKQKSSNSRKKRSTSA	Homo sapiens
1	1.00	1	1	0	0	0	0	-0.002	t	149403	PCKSNT	Mus musculus
1	1.00	1	1	0	0	0	0	-0.002	t	136353	HGNAEVHASFFDIGGS	Oryctolagus cuniculus
1	1.00	1	1	0	0	0	0	-0.002	t	145725	CKQAGTCPP	Mus musculus
1	1.00	1	1	0	0	0	0	-0.002	t	135576	LRYSTGTVSG	Oryctolagus cuniculus
1	1.00	1	1	0	0	0	0	-0.002	t	140258	KYEQGGNIVD	Mus musculus

1	1.00	1	1	0	0	0	0	-0.002	t	137180	GVSAAQEKISFG	Sus scrofa
1	1.00	1	1	0	0	0	0	-0.002	t	140489	MSQDGHT	Mus musculus
1	1.00	1	1	0	0	0	0	-0.002	t	146337	IPEEIKQL	Mus musculus
1	1.00	1	1	0	0	0	0	-0.002	t	140507	TKTWQSM	Mus musculus
1	1.00	1	1	0	0	0	0	-0.002	t	138047	SSKPVSY	Mus musculus
1	1.00	1	1	0	0	0	0	-0.002	t	141523	ALEPPHNLPVP	Mus musculus
1	1.00	1	1	0	0	0	0	-0.002	t	135586	PFMLAQFDG	Oryctolagus cuniculus
1	1.00	1	1	0	0	0	0	-0.002	t	136315	GGGTGPADGTNATTI	Mus musculus
1	1.00	1	1	0	0	0	0	-0.002	t	141622	DPNGWTGTDNNFSI	Oryctolagus cuniculus
1	1.00	1	1	0	0	0	0	-0.002	t	150204	FHSVVHTLLEWLMEKELMV	Mus musculus
1	1.00	1	1	0	0	0	0	-0.002	t	137284	RTNTTQGKVPSQ	Sus scrofa
1	1.00	1	1	0	0	0	0	-0.002	t	150858	ISSLYNLVKSS	Mus musculus
1	1.00	1	1	0	0	0	0	-0.002	t	145841	KEDFRYAISSTNEIGLLGA	Sus scrofa
1	1.00	1	1	0	0	0	0	-0.002	t	151601	ADLSLEKAANVQWDE	Homo sapiens
1	1.00	1	1	0	0	0	0	-0.002	t	143362	ETPLMKE	Mus musculus
1	1.00	1	1	0	0	0	0	-0.002	t	140254	KYEHGGNIID	Mus musculus

1	1.00	1	1	0	0	0	0	-0.002	t	134337	CYCQDPYVKEAENLKKYFNA	Oryctolagus cuniculus
1	1.00	1	1	0	0	0	0	-0.002	t	140510	TTTHFLATKFYK	Mus musculus
1	1.00	1	1	0	0	0	0	-0.002	t	136341	GTTMPLHAAPHS	Homo sapiens
1	1.00	1	1	0	0	0	0	-0.002	t	145704	AEGLTTTWKDYDHNLQ	Sus scrofa
1	1.00	1	1	0	0	0	0	-0.002	t	134418	KGAIIGLMVGGVV	Homo sapiens
1	1.00	1	1	0	0	0	0	-0.002	t	136315	GGGTGPADGTNATTI	Mus musculus
1	1.00	1	1	0	0	0	0	-0.002	t	143362	ETPLMKE	Mus musculus
1	1.00	1	1	0	0	0	0	-0.002	t	141620	DNPRPNDKTGS	Oryctolagus cuniculus
1	1.00	1	1	0	0	0	0	-0.002	t	135572	LLCEDGCLAL	Oryctolagus cuniculus
1	1.00	1	1	0	0	0	0	-0.002	t	140167	AYNNGQEINGFKA	Oryctolagus cuniculus
1	1.00	1	1	0	0	0	0	-0.002	t	145831	IPLGTRP	Mus musculus
1	1.00	1	1	0	0	0	0	-0.002	t	140461	EVSLHDMNLATH	Mus musculus
1	1.00	1	1	0	0	0	0	-0.002	t	152777	LLNIDKDIRK	Homo sapiens
1	1.00	1	1	0	0	0	0	-0.002	t	149989	TRNSSKPAKRKKIKA	Oryctolagus cuniculus
1	1.00	1	1	0	0	0	0	-0.002	t	140378	TAEETKQ	Oryctolagus cuniculus

1	1.00	1	1	0	0	0	0	-0.002	t	136749	WMLRAAEEYSMNLGF	Mus musculus
1	1.00	1	1	0	0	0	0	-0.002	t	136749	WMLRAAEEYSMNLGF	Mus musculus
1	1.00	1	1	0	0	0	0	-0.002	t	134860	AGPKAQVEAIQKY	Mus musculus
1	1.00	1	1	0	0	0	0	-0.002	t	134335	CTKPSDGNCT	Homo sapiens
1	1.00	1	1	0	0	0	0	-0.002	t	134478	PSCCCTKPSD	Homo sapiens
1	1.00	1	1	0	0	0	0	-0.002	t	141706	MLEPFQIL	Mus musculus
1	1.00	1	1	0	0	0	0	-0.002	t	139725	EMRTRIRYNRRS	Mus musculus
1	1.00	1	1	0	0	0	0	-0.002	t	145827	IIPKVEGK	Mus musculus
1	1.00	1	1	0	0	0	0	-0.002	t	134557	TCTIPAQGTSMFPSC	Homo sapiens
1	1.00	1	1	0	0	0	0	-0.002	t	140381	TKTVNENKQNVDAKVKA	Oryctolagus cuniculus
1	1.00	1	1	0	0	0	0	-0.002	t	136827	DFGSVGGVL	Mus musculus
1	1.00	1	1	0	0	0	0	-0.002	t	140247	KAAESEIEKLTTK	Oryctolagus cuniculus
1	1.00	1	1	0	0	0	0	-0.002	t	134337	CYCQDPYVKEAENLKKYFNA	Oryctolagus cuniculus
1	1.00	1	1	0	0	0	0	-0.002	t	150108	AGGTGTEDSHPPYKQ	Mus musculus
1	1.00	1	1	0	0	0	0	-0.002	t	137401	FEDVW	Mus musculus
1	1.00	1	1	0	0	0	0	-0.002	t	151895	GSPSQDGYWSFF	Sus scrofa

1	1.00	1	1	0	0	0	0	-0.002	t	141761	TAPPLLPH SNLD	Rattus norvegic us
1	1.00	1	1	0	0	0	0	-0.002	t	150850	GY	Mus musculu s
1	1.00	1	1	0	0	0	0	-0.002	t	136315	GGGTGPA DGTNATTI	Mus musculu s
1	1.00	1	1	0	0	0	0	-0.002	t	141760	TAPPLLPH	Rattus norvegic us
1	1.00	1	1	0	0	0	0	-0.002	t	151713	RGEARK	Homo sapiens
1	1.00	1	1	0	0	0	0	-0.002	t	149247	AEQQWAL YADGSIRP Q	Mus musculu s
1	1.00	1	1	0	0	0	0	-0.002	t	141758	SWRNNILR TQES	Oryctola gus cuniculu s
1	1.00	1	1	0	0	0	0	-0.002	t	135587	QESYSSKK L	Oryctola gus cuniculu s
1	1.00	1	1	0	0	0	0	-0.002	t	138097	YPYPLIS	Mus musculu s
1	1.00	1	1	0	0	0	0	-0.002	t	137180	GVSAAQE KISFG	Sus scrofa
1	1.00	1	1	0	0	0	0	-0.002	t	139358	VLKTDVNK E	Mus musculu s
1	1.00	1	1	0	0	0	0	-0.002	t	146624	WNWFDIT N	Homo sapiens
1	1.00	1	1	0	0	0	0	-0.002	t	140081	YFVPNLKD MFSRAVT MTAAS	Oryctola gus cuniculu s
1	1.00	1	1	0	0	0	0	-0.002	t	140459	EHWSHD MFSPGD	Mus musculu s

1	1.00	1	1	0	0	0	0	-0.002	t	136484	LPSSSPTPFLSG	Homo sapiens
1	1.00	1	1	0	0	0	0	-0.002	t	137432	NHNYPPLSLLTF	Mus musculus
1	1.00	1	1	0	0	0	0	-0.002	t	134804	TIFILFCCSKEKRKKKQAAT	Mus musculus
1	1.00	1	1	0	0	0	0	-0.002	t	134784	RKRSQMLFRGRRASQ	Mus musculus
1	1.00	1	1	0	0	0	0	-0.002	t	139324	TRLIDERAAH	Mus musculus
1	1.00	1	1	0	0	0	0	-0.002	t	137286	RVTAAQGRIYTR	Sus scrofa
1	1.00	1	1	0	0	0	0	-0.002	t	151944	KFPSESRPNVVK	Sus scrofa
1	1.00	1	1	0	0	0	0	-0.002	t	146795	CCELCCNPQCAGCY	Oryctolagus cuniculus
1	1.00	1	1	0	0	0	0	-0.002	t	141605	APPLLPHSNL	Mus musculus
1	1.00	1	1	0	0	0	0	-0.002	t	153294	TYNHDYKTGMVIYSPFMTYP	Mus musculus
1	1.00	1	1	0	0	0	0	-0.002	t	138194	IIPKSSWS	Homo sapiens
1	1.00	1	1	0	0	0	0	-0.002	t	140144	ADVEADDFKGLGLK	Oryctolagus cuniculus
1	1.00	1	1	0	0	0	0	-0.002	t	138892	REQAPNLVY	Mus musculus
1	1.00	1	1	0	0	0	0	-0.002	t	134472	PLLPGTSTTSTGPCKT	Homo sapiens
1	1.00	1	1	0	0	0	0	-0.002	t	134133	LGGAKQAGDV	Mus musculus

1	1.00	1	1	0	0	0	0	-0.002	t	141750	SNLDHILEPSI	Oryctolagus cuniculus
1	1.00	1	1	0	0	0	0	-0.002	t	141701	LNDKHSNGTIKDRSPYR	Oryctolagus cuniculus
1	1.00	1	1	0	0	0	0	-0.002	t	156905	KPGDPRQ	Mus musculus
1	1.00	1	1	0	0	0	0	-0.002	t	135562	IHAMDIPPPTG	Oryctolagus cuniculus
1	1.00	1	1	0	0	0	0	-0.002	t	140053	MEDDYNLKDMFSRAVTM	Oryctolagus cuniculus
1	1.00	1	1	0	0	0	0	-0.002	t	140458	EHNDFPMYTWRP	Mus musculus
1	1.00	1	1	0	0	0	0	-0.002	t	135560	IEQAIGRVTPIF	Oryctolagus cuniculus
1	1.00	1	1	0	0	0	0	-0.002	t	138089	WLRPPTS	Mus musculus
1	1.00	1	1	0	0	0	0	-0.002	t	153472	TGLRNGITNKVNSVIEKAA	Mus musculus
1	1.00	1	1	0	0	0	0	-0.002	t	151842	EHSGQAAWKQVV	Sus scrofa
1	1.00	1	1	0	0	0	0	-0.002	t	138088	WLDLSKT	Mus musculus
1	1.00	1	1	0	0	0	0	-0.002	t	140248	KAGETIYDIDEDGTITKKD	Oryctolagus cuniculus
1	1.00	1	1	0	0	0	0	-0.002	t	125411	FQGLCNETLTLKLYNNGFTS	Homo sapiens
1	1.00	1	1	0	0	0	0	-0.002	t	130637	WSSCVFSVSYCL	Mus musculus

1	1.00	1	1	0	0	0	0	-0.002	t	119143	EPTITFGTAI	Oryctolagus cuniculus
1	1.00	1	1	0	0	0	0	-0.002	t	123838	VWQDE	Mus musculus
1	1.00	1	1	0	0	0	0	-0.002	t	118963	PNYKVNVYAVTK	Mus musculus
1	1.00	1	1	0	0	0	0	-0.002	t	119187	GNAEVHASFFDIGG	Mus musculus
1	1.00	1	1	0	0	0	0	-0.002	t	125250	SDEFNPCEDIMGYK	Mus musculus
1	1.00	1	1	0	0	0	0	-0.002	t	125250	SDEFNPCEDIMGYK	Mus musculus
1	1.00	1	1	0	0	0	0	-0.002	t	131879	FLVAHTRARAPSAGERARRS	Mus musculus
1	1.00	1	1	0	0	0	0	-0.002	t	122083	DPNIKLVLDDCWATS	Oryctolagus cuniculus
1	1.00	1	1	0	0	0	0	-0.002	t	127986	FVGVGADYRHASCPIDYIIA	Mus musculus
1	1.00	1	1	0	0	0	0	-0.002	t	123849	WQDE	Mus musculus
1	1.00	1	1	0	0	0	0	-0.002	t	125055	FVYGGCQGNN	Mus musculus
1	1.00	1	1	0	0	0	0	-0.002	t	125321	YFLHWWYDKK	Mus musculus
1	1.00	1	1	0	0	0	0	-0.002	t	119601	KGNNVHGFQKQIIRSFY	Rattus norvegicus
1	1.00	1	1	0	0	0	0	-0.002	t	122842	LLDLPRDLGGMGCSSPPCE	Oryctolagus cuniculus
1	1.00	1	1	0	0	0	0	-0.002	t	120109	NIHPKAPIWGIITSNF	Rattus norvegicus

1	1.00	1	1	0	0	0	0	-0.002	t	130449	CFFPNLSYC	Mus musculus
1	1.00	1	1	0	0	0	0	-0.002	t	120109	NIHPKAPIWGIITSNF	Rattus norvegicus
1	1.00	1	1	0	0	0	0	-0.002	t	122780	GQELKNPQEETLQAFDSHYD	Oryctolagus cuniculus
1	1.00	1	1	0	-0.003	0	0	-0.002	t	122780	GQELKNPQEETLQAFDSHYD	Oryctolagus cuniculus
1	1.00	1	1	0	0	0	0	-0.002	t	130474	ECLLLSKYCMPS	Mus musculus
1	1.00	1	1	0	0	0	0	-0.002	t	124069	ELQIAKDERYGS	Oryctolagus cuniculus
1	1.00	1	1	0	0	0	0	-0.002	t	119187	GNAEVHASFFDIGG	Oryctolagus cuniculus
1	1.00	1	1	0	0	0	0	-0.002	t	124713	SGEQPSDEQPSGEHG	Mus musculus
1	1.00	1	1	0	0	0	0	-0.002	t	125415	FSPDGKPCTPPALNCYW	Mus musculus
1	1.00	1	1	0	0	0	0	-0.002	t	132039	QYFVGKRTVPPGECFIQFLS	Oryctolagus cuniculus
1	1.00	1	1	0	0	0	0	-0.002	t	127984	FTNLKFKVRKITSSHRGN	Mus musculus
1	1.00	1	1	0	0	0	0	-0.002	t	119038	TSTNGIKKILIF	Mus musculus
1	1.00	1	1	0	0	0	0	-0.002	t	133707	STRKEEKQRNGT	Mus musculus
1	1.00	1	1	0	0	0	0	-0.002	t	125608	YYKKHIS	Mus musculus
1	1.00	1	1	0	0	0	0	-0.002	t	130454	CLFPNNSYC	Mus musculus

1	1.00	1	1	0	0	0	0	-0.002	t	125127	LDPENLTL	Mus musculus
1	1.00	1	1	0	0	0	0	-0.002	t	119135	EGVKYVLNETTNWLWAFLAR	Mus musculus
1	1.00	1	1	0	0	0	0	-0.002	t	130568	NQYCIYAGRYC	Mus musculus
1	1.00	1	1	0	0	0	0	-0.002	t	127933	APDDSFKKLEDRFTNLKF	Mus musculus
1	1.00	1	1	0	0	0	0	-0.002	t	125454	KDYKTMVKFCN	Mus musculus
1	1.00	1	1	0	0	0	0	-0.002	t	125384	DSHYDYTICGDSEDMVCTPK	Homo sapiens
1	1.00	1	1	0	0	0	0	-0.002	t	130622	TGLVAQGTDSIG	Camelus dromedarius
1	1.00	1	1	0	0	0	0	-0.002	t	125015	DNESSYSRGFDMTYTE	Mus musculus
1	1.00	1	1	0	0	0	0	-0.002	t	130555	MHNRTQALEDSR	Camelus dromedarius
1	1.00	1	1	0	0	0	0	-0.002	t	127944	DLVNCNLNTNCVAVAFSLPD	Mus musculus
1	1.00	1	1	0	0	0	0	-0.002	t	122291	CTYILDPEKLTLRATYDN	Oryctolagus cuniculus
1	1.00	1	1	0	0	0	0	-0.002	t	125051	FQSKANCLNT	Mus musculus
1	1.00	1	1	0	0	0	0	-0.002	t	128135	VCTPKSDEFNPCEDIMGYKF	Homo sapiens
1	1.00	1	1	0	0	0	0	-0.002	t	133272	PFFSQPG	Mus musculus
1	1.00	1	1	0	0	0	0	-0.002	t	125318	WWYDKKDNTC	Mus musculus
1	1.00	1	1	0	0	0	0	-0.002	t	130457	CRQPGNNYC	Mus musculus

1	1.00	1	1	0	0	0	0	-0.002	t	133722	TSKSKYPLVV	Mus musculus
1	1.00	1	1	0	0	0	0	-0.002	t	125607	YVSIDVTLQQLESHSFYNLS	Homo sapiens
1	1.00	1	1	0	0	0	0	-0.002	t	122292	CTYILDPEKLTLRVPYKA	Oryctolagus cuniculus
1	1.00	1	1	0	-0.003	0	0	-0.002	t	125610	YYVFFEEQEDEIIGFGQELK	Oryctolagus cuniculus
1	1.00	1	1	0	-0.003	0	0	-0.002	t	125472	LDAVYLNKNKYLTVIDKDAF	Oryctolagus cuniculus
1	1.00	1	1	0	0	0	0	-0.002	t	128043	LDAMPAGNPAFPVIPG	Mus musculus
1	1.00	1	1	0	0	0	0	-0.002	t	130787	EDFDIYDEDE	Mus musculus
1	1.00	1	1	0	-0.002	0	0	-0.002	t	118963	PNYKVNVYAVTK	Mus musculus
1	1.00	1	1	0	0	0	0	-0.002	t	126201	GYKEKSKFQDTHNNA	Oryctolagus cuniculus
1	1.00	1	1	0	0	0	0	-0.002	t	125109	KKDNTCSMFV	Mus musculus
1	1.00	1	1	0	0	0	0	-0.002	t	124232	GSITRDSIFRLR	Oryctolagus cuniculus
1	1.00	1	1	0	0	0	0	-0.002	t	128084	QKNACSFDLCNSYDVL	Mus musculus
1	1.00	1	1	0	0	0	0	-0.002	t	119322	QRVFKEKVDTKAPEPP	Mus musculus
1	1.00	1	1	0	0	0	0	-0.002	t	133676	RIYMRMKHVR	Mus musculus

1	1.00	1	1	0	0	0	0	-0.002	t	123979	CGTAKRVTGNQAV	Oryctolagus cuniculus
1	1.00	1	1	0	0	0	0	-0.002	t	131710	TPDAPGETV	Mus musculus
1	1.00	1	1	0	0	0	0	-0.002	t	124925	YYVFFEEQEDEIIGF	Oryctolagus cuniculus
1	1.00	1	1	0	-0.003	0	0	-0.002	t	125610	YYVFFEEQEDEIIGFGQELK	Oryctolagus cuniculus
1	1.00	1	1	0	0	0	0	-0.002	t	125448	IRGILESLMCNESSMQSLRQ	Oryctolagus cuniculus
1	1.00	1	1	0	0	0	0	-0.002	t	130487	FCQPHLSCYILP	Mus musculus
1	1.00	1	1	0	0	0	0	-0.002	t	125472	LDAVYLNKNKYLTVIDKDAF	Oryctolagus cuniculus
1	1.00	1	1	0	0	0	0	-0.002	t	128146	WSVCVGLTTYVNDYVLPYLA	Mus musculus
1	1.00	1	1	0	0	0	0	-0.002	t	125441	HPLTCDDPRFQ	Oryctolagus cuniculus
1	1.00	1	1	0	0	0	0	-0.002	t	128136	VFIADSDGKLNFKEWSVCVG	Mus musculus
1	1.00	1	1	0	0	0	0	-0.002	t	122202	TSPPLLWDSVHL	Mus musculus
1	1.00	1	1	0	0	0	0	-0.002	t	125267	TCSMFVYGGC	Mus musculus
1	1.00	1	1	0	0	0	0	-0.002	t	131101	TDSEMDVVRFDDDNS	Mus musculus
1	1.00	1	1	0	0	0	0	-0.002	t	130461	CWNYCLP	Mus musculus

1	1.00	1	1	0	0	0	0	-0.002	t	130614	STLPYLPTY SKH	Camelus dromed arius
1	1.00	1	1	0	0	0	0	-0.002	t	130610	SMCMHG GAYCFP	Mus musculu s
1	1.00	1	1	0	0	0	0	-0.002	t	122790	HQEEDFR VTCKDIQR	Oryctola gus cuniculu s
1	1.00	1	1	0	0	0	0	-0.002	t	122857	MGCSSPP CECHQEE	Mus musculu s
1	1.00	1	1	0	0	0	0	-0.002	t	122857	MGCSSPP CECHQEE	Mus musculu s
1	1.00	1	1	0	0	0	0	-0.002	t	131582	PEEDDITW TSDQRHG VIGS	Mus musculu s
1	1.00	1	1	0	0	0	0	-0.002	t	125552	RRFEYDDP RFLRLLDLA QEG	Homo sapiens
1	1.00	1	1	0	0	0	0	-0.002	t	121292	FATFVYAK	Mus musculu s
1	1.00	1	1	0	0	0	0	-0.002	t	125552	RRFEYDDP RFLRLLDLA QEG	Homo sapiens
1	1.00	1	1	0	0	0	0	-0.002	t	125478	LHQEYEEN LGDSIVGY KEKS	Homo sapiens
1	1.00	1	1	0	0	0	0	-0.002	t	130469	DQPPTSRV PSLR	Camelus dromed arius
1	1.00	1	1	0	0	0	0	-0.002	t	125297	VFFEEQED EIIGFG	Mus musculu s
1	1.00	1	1	0	0	0	0	-0.002	t	130612	SPNTAHIT SKSL	Camelus dromed arius
1	1.00	1	1	0	0	0	0	-0.002	t	130513	IGYQPSMP LDMP	Camelus dromed arius
1	1.00	1	1	0	0	0	0	-0.002	t	108846	DALQFVC GDRGFY	Mus musculu s
1	1.00	1	1	0	0	0	0	-0.002	t	110116	YCAPLKPA KSA	Mus musculu s

1	1.00	1	1	0	0	0	0	-0.002	t	111486	LIEESAIINAGAP	Mus musculus
1	1.00	1	1	0	0	0	0	-0.002	t	102384	EFRH	Mus musculus
1	1.00	1	1	0	0	0	0	-0.002	t	100153	INDQIKFIIN	Mus musculus
1	1.00	1	1	0	0	0	0	-0.002	t	111772	SGFSITTSTDTTPA	Oryctolagus cuniculus
1	1.00	1	1	0	0	0	0	-0.002	t	99975	EEKELLKRIQ	Mus musculus
1	1.00	1	1	0	0	0	0	-0.002	t	102384	EFRH	Mus musculus
1	1.00	1	1	0	0	0	0	-0.002	t	107202	LKFVEQNE	Homo sapiens
1	1.00	1	1	0	0	0	0	-0.002	t	110198	CPPDIIPKVEGKTIA	Mus musculus
1	1.00	1	1	0	0	0	0	-0.002	t	99975	EEKELLKRIQ	Mus musculus
1	1.00	1	1	0	0	0	0	-0.002	t	98822	IEAGAIGFKIHEDWG	Mus musculus
1	1.00	1	1	0	0	0	0	-0.002	t	110198	CPPDIIPKVEGKTIA	Oryctolagus cuniculus
1	1.00	1	1	0	0	0	0	-0.002	t	115406	NRKVKPTTEGGVETE	Oryctolagus cuniculus
1	1.00	1	1	0	0	0	0	-0.002	t	106429	FVLPWRI	Homo sapiens
1	1.00	1	1	0	0	0	0	-0.002	t	102384	EFRH	Mus musculus
1	1.00	1	1	0	0	0	0	-0.002	t	100549	TEEKEFLKKL	Mus musculus
1	1.00	1	1	0	0	0	0	-0.002	t	105207	GAIIGLMVGGVVIA	Mus musculus

1	1.00	1	1	0	0	0	0	-0.002	t	115302	IVTAGEERAVTT	Oryctolagus cuniculus
1	1.00	1	1	0	0	0	0	-0.002	t	103998	IVKWDRDM	Mus musculus
1	1.00	1	1	0	0	0	0	-0.002	t	111486	LIEESAIINAGAP	Oryctolagus cuniculus
1	1.00	1	1	0	0	0	0	-0.002	t	110275	GPVGPSDPSIVSLVEETSFI	Mus musculus
1	1.00	1	1	0	0	0	0	-0.002	t	111486	LIEESAIINAGAP	Oryctolagus cuniculus
1	1.00	1	1	0	0	0	0	-0.002	t	105651	AEFRHDS	Mus musculus
1	1.00	1	1	0	-0.003	0	0	-0.002	t	110424	SGTGGRTGYIPLGTRPPT	Oryctolagus cuniculus
1	1.00	1	1	0	0	0	0	-0.002	t	115540	VKNKRTFLSPWISNI	Mus musculus
1	1.00	1	1	0	0	0	0	-0.002	t	111486	LIEESAIINAGAP	Mus musculus
1	1.00	1	1	0	0	0	0	-0.002	t	113913	TCKQAGTCPP	Mus musculus
1	1.00	1	1	0	0	0	0	-0.002	t	98434	RYMDQPSRD	Oryctolagus cuniculus
1	1.00	1	1	0	0	0	0	-0.002	t	104327	VEQHNPINAGTELSAT	Oryctolagus cuniculus
1	1.00	1	1	0	0	0	0	-0.002	t	111790	SLVEETSFIDAGAPTS	Oryctolagus cuniculus
1	1.00	1	1	0	0	0	0	-0.002	t	113365	EHPVTGCGERTEGRCLHYTV	Mus musculus

1	1.00	1	1	0	0	0	0	-0.002	t	113365	EHPVTGCGERTEGRCLHYTV	Mus musculus
1	1.00	1	1	0	0	0	0	-0.002	t	113365	EHPVTGCGERTEGRCLHYTV	Mus musculus
1	1.00	1	1	0	0	0	0	-0.002	t	115488	SKDNGWEKDDNVSKS	Oryctolagus cuniculus
1	1.00	1	1	0	0	0	0	-0.002	t	111486	LIEESAIINAGAP	Oryctolagus cuniculus
1	1.00	1	1	0	0	0	0	-0.002	t	115265	GKDITADNK	Oryctolagus cuniculus
1	1.00	1	1	0	0	0	0	-0.002	t	108832	CGDRGFYFNKPTG	Mus musculus
1	1.00	1	1	0	0	0	0	-0.002	t	107307	DAEFGHDSGF	Mus musculus
1	1.00	1	1	0	0	0	0	-0.002	t	115351	LKQKSSNSRKKRSTS	Mus musculus
1	1.00	1	1	0	0	0	0	-0.002	t	102510	HHQKLVFFAEDVGSNK	Mus musculus
1	1.00	1	1	0	0	0	0	-0.002	t	108412	GTTDHVGVPTYDWGK	Capra hircus
1	1.00	1	1	0	0	0	0	-0.002	t	104132	MSENVEQHNPINAGT	Oryctolagus cuniculus
1	1.00	1	1	0	0	0	0	-0.002	t	108482	IINPSENGDTSTNGIKK	Mus musculus
1	1.00	1	1	0	0	0	0	-0.002	t	103972	HNPINAGTELSATGN	Oryctolagus cuniculus
1	1.00	1	1	0	0	0	0	-0.002	t	111486	LIEESAIINAGAP	Mus musculus
1	1.00	1	1	0	0	0	0	-0.002	t	111486	LIEESAIINAGAP	Mus musculus

1	1.00	1	1	0	0	0	0	-0.002	t	104175	PINAGTELSATGNESG	Oryctolagus cuniculus
1	1.00	1	1	0	0	0	0	-0.002	t	102384	EFRH	Mus musculus
1	1.00	1	1	0	0	0	0	-0.002	t	104327	VEQHNPINAGTELSAT	Sus scrofa
1	1.00	1	1	0	0	0	0	-0.002	t	108998	FVCGDRGFYFNK	Mus musculus
1	1.00	1	1	0	0	0	0	-0.002	t	98093	HGFTEQNSG	Oryctolagus cuniculus
1	1.00	1	1	0	-0.004	0	0	-0.002	t	110980	QPLGVGISGHPLLNKLDDTE	Mus musculus
1	1.00	1	1	0	0	0	0	-0.002	t	102384	EFRH	Mus musculus
1	1.00	1	1	0	0	0	0	-0.002	t	100635	VTNYLVDGNG	Mus musculus
1	1.00	1	1	0	0	0	0	-0.002	t	103886	EQHNPINAGTELSAT	Oryctolagus cuniculus
1	1.00	1	1	0	0	0	0	-0.002	t	110424	SGTGGRTGYIPLGTRPPT	Oryctolagus cuniculus
1	1.00	1	1	0	0	0	0	-0.002	t	116080	ATGGTLQQLFYSITVGQV	Mus musculus
1	1.00	1	1	0	0	0	0	-0.002	t	107156	GLAGAPGLR	Mus musculus
1	1.00	1	1	0	0	0	0	-0.002	t	110980	QPLGVGISGHPLLNKLDDTE	Mus musculus
1	1.00	1	1	0	0	0	0	-0.002	t	111013	RTGYIPLGTRPPT	Oryctolagus cuniculus
1	1.00	1	1	0	0	0	0	-0.002	t	115058	GNAEVHASFFDIGGS	Oryctolagus cuniculus

1	1.00	1	1	0	0	0	0	-0.002	t	112316	SIKNPTNAK	Mus musculus
1	1.00	1	1	0	0	0	0	-0.002	t	106642	MHLNTPP	Homo sapiens
1	1.00	1	1	0	0	0	0	-0.002	t	102344	DAEFR	Mus musculus
1	1.00	1	1	0	-0.003	0	0	-0.002	t	111380	GTGSGTGGRTGYIPL	Oryctolagus cuniculus
1	1.00	1	1	0	0	0	0	-0.002	t	99760	VKDGYIVD	Oryctolagus cuniculus
1	1.00	1	1	0	0	0	0	-0.002	t	99945	DIRDSLSEEE	Mus musculus
1	1.00	1	1	0	-0.003	0	0	-0.002	t	110248	GGLGIGTGSGTGGRTGYIPL	Oryctolagus cuniculus
1	1.00	1	1	0	0	0	0	-0.002	t	112352	TGNKYPGYP	Mus musculus
1	1.00	1	1	0	0	0	0	-0.002	t	105319	LVFFAEDVGSNK	Homo sapiens
1	1.00	1	1	0	0	0	0	-0.002	t	109177	GPETLCGAELVD	Mus musculus
1	1.00	1	1	0	0	0	0	-0.002	t	108600	QLINTNGS	Capra hircus
1	1.00	1	1	0	0	0	0	-0.002	t	105163	EFRHD	Mus musculus
1	1.00	1	1	0	0	0	0	-0.002	t	106634	LVGPRGERGFP	Mus musculus
1	1.00	1	1	0	0	0	0	-0.002	t	110248	GGLGIGTGSGTGGRTGYIPL	Oryctolagus cuniculus
1	1.00	1	1	0	0	0	0	-0.002	t	111381	GTGSGTGGRTGYVPL	Oryctolagus cuniculus
1	1.00	1	1	0	0	0	0	-0.002	t	110812	DPVGPLDPSIVSLVEESSFI	Oryctolagus cuniculus

1	1.00	1	1	0	0	0	0	-0.002	t	102344	DAEFR	Mus musculus
1	1.00	1	1	0	0	0	0	-0.002	t	110733	VGENVPDDLYIKGSG	Mus musculus
1	1.00	1	1	0	-0.003	0	0	-0.002	t	98093	HGFTEQNSG	Oryctolagus cuniculus
1	1.00	1	1	0	0	0	0	-0.002	t	103643	TGALQERSK	Mus musculus
1	1.00	1	1	0	0	0	0	-0.002	t	110308	KTCKQAGTCPPDIIPKVEG	Mus musculus
1	1.00	1	1	0	-0.003	0	0	-0.002	t	111013	RTGYIPLGTRPPT	Oryctolagus cuniculus
1	1.00	1	1	0	0	0	0	-0.002	t	110248	GGLGIGTGSGTGGRTGYIPL	Mus musculus
1	1.00	1	1	0	0	0	0	-0.002	t	106646	MHLTQSP	Homo sapiens
1	1.00	1	1	0	0	0	0	-0.002	t	110385	QLYKTCKQAGTCPPDIIPKV	Mus musculus
1	1.00	1	1	0	0	0	0	-0.002	t	110385	QLYKTCKQAGTCPPDIIPKV	Oryctolagus cuniculus
1	1.00	1	1	0	0	0	0	-0.002	t	108551	NDSLNTGFLAFLFYTKK	Capra hircus
1	1.00	1	1	0	-0.003	0	0	-0.002	t	117220	LYKTCKQAGTCPPD	Oryctolagus cuniculus
1	1.00	1	1	0	0	0	0	-0.002	t	111211	DPVGPSDPSIVSLVEETSF	Oryctolagus cuniculus
1	1.00	1	1	0	0	0	0	-0.002	t	117220	LYKTCKQAGTCPPD	Oryctolagus cuniculus
1	1.00	1	1	0	0	0	0	-0.002	t	110385	QLYKTCKQAGTCPPDIIPKV	Oryctolagus cuniculus

1	1.00	1	1	0	0	0	0	-0.002	t	105021	VHHQKLVFFAEDVGSNK	Homo sapiens
1	1.00	1	1	0	0	0	0	-0.002	t	110812	DPVGPLDPSIVSLVEESSFI	Oryctolagus cuniculus
1	1.00	1	1	0	0	0	0	-0.002	t	67373	TYKADGETATEKT	Mus musculus
1	1.00	1	1	0	0	0	0	-0.002	t	75418	YPTFGEHKQEKDLEY	Mus musculus
1	1.00	1	1	0	0	0	0	-0.002	t	70391	VPNLRGDLQVLAQKVARTLP	Mus musculus
1	1.00	1	1	0	-0.004	0	0	-0.002	t	64698	TKWCPPGQLVNLHDFRSDEI	Oryctolagus cuniculus
1	1.00	1	1	0	0	0	0	-0.002	t	74430	YKAYVSYKKRKAQEK	Oryctolagus cuniculus
1	1.00	1	1	0	-0.002	0	0	-0.002	t	64698	TKWCPPGQLVNLHDFRSDEI	Mus musculus
1	1.00	1	1	0	0	0	0	-0.002	t	79292	LKTDLELKYKQTADFS	Oryctolagus cuniculus
1	1.00	1	1	0	0	0	0	-0.002	t	60722	SRLGRSSAWV	Mus musculus
1	1.00	1	1	0	0	0	0	-0.002	t	67253	TWEVLH	Mus musculus
1	1.00	1	1	0	0	0	0	-0.002	t	69175	VKAETRLN	Mus musculus
1	1.00	1	1	0	0	0	0	-0.002	t	70729	VRGLYFP	Mus musculus

1	1.00	1	1	0	0	0	0	-0.002	t	70391	VPNLRGDLQVLAQKVARTLP	Mus musculus
1	1.00	1	1	0	0	0	0	-0.002	t	62264	SVNPKMVR	Mus musculus
1	1.00	1	1	0	0	0	0	-0.002	t	75923	YSWKTWG	Mus musculus
1	1.00	1	1	0	0	0	0	-0.002	t	67253	TWEVLH	Mus musculus
1	1.00	1	1	0	0	0	0	-0.002	t	60917	SSAVVDNKLKI	Mus musculus
1	1.00	1	1	0	0	0	0	-0.002	t	94580	LDNKYAGKGY	Mus musculus
1	1.00	1	1	0	0	0	0	-0.002	t	74476	YKLPDHVRT	Mus musculus
1	1.00	1	1	0	0	0	0	-0.002	t	78615	CPGHANRKQCGG	Oryctolagus cuniculus
1	1.00	1	1	0	0	0	0	-0.002	t	70391	VPNLRGDLQVLAQKVARTLP	Mus musculus
1	1.00	1	1	0	0	0	0	-0.002	t	65421	TNSGINNS	Mus musculus
1	1.00	1	1	0	0	0	0	-0.002	t	70391	VPNLRGDLQVLAQKVARTLP	Mus musculus
1	1.00	1	1	0	0	0	0	-0.002	t	62077	SVDFEED	Mus musculus
1	1.00	1	1	0	0	0	0	-0.002	t	87356	PNCHGPDWASPVCQRHS	Mus musculus

1	1.00	1	1	0	0	0	0	-0.002	t	93007	INNGLPPIQNTRNI	Mus musculus
1	1.00	1	1	0	0	0	0	-0.002	t	71506	VTSTLTSLFRPGASQKIQL	Mus musculus
1	1.00	1	1	0	-0.002	0	0	-0.002	t	69178	VKAETRLNPDLQPTE	Homo sapiens
1	1.00	1	1	0	0	0	0	-0.002	t	67170	TVSTEQNVPDPQVGI	Mus musculus
1	1.00	1	1	0	0	0	0	-0.002	t	97484	LVEETSFIDAGAP	Mus musculus
1	1.00	1	1	0	0	0	0	-0.002	t	67170	TVSTEQNVPDPQVGI	Mus musculus
1	1.00	1	1	0	0	0	0	-0.002	t	79287	LEVTVMLNKRLPDSRTS	Oryctolagus cuniculus
1	1.00	1	1	0	0	0	0	-0.002	t	73433	YARFMDTDPDRYGILPSNF	Mus musculus
1	1.00	1	1	0	0	0	0	-0.002	t	73433	YARFMDTDPDRYGILPSNF	Mus musculus
1	1.00	1	1	0	0	0	0	-0.002	t	76606	YYSPAF	Mus musculus
1	1.00	1	1	0	0	0	0	-0.002	t	78627	DPDPPDANDPAPPNAN	Mus musculus
1	1.00	1	1	0	0	0	0	-0.002	t	95058	AGLQND	Mus musculus
1	1.00	1	1	0	0	0	0	-0.002	t	61801	STNSGINN	Mus musculus
1	1.00	1	1	0	0	0	0	-0.002	t	65238	TMPFSPDFSNVD	Oryctolagus cuniculus
1	1.00	1	1	0	0	0	0	-0.002	t	62730	SYTVTNLVQTQSQVQ	Mus musculus

1	1.00	1	1	0	0.004	0	0	-0.002	t	62730	SYTVTNLVQTQSQVQ	Mus musculus
1	1.00	1	1	0	0	0	0	-0.002	t	78629	DQKPPSRQSQIESR	Oryctolagus cuniculus
1	1.00	1	1	0	0	0	0	-0.002	t	70437	VPPTQSKKKNKNET	Oryctolagus cuniculus
1	1.00	1	1	0	0	0	0	-0.002	t	82891	GSPNCHGPDWASPVCQRHS	Mus musculus
1	1.00	1	1	0	0	0	0	-0.002	t	71693	VVIANNVDKFVSWGITDFEM	Rattus norvegicus
1	1.00	1	1	0	0	0	0	-0.002	t	70391	VPNLRGDLQVLAQKVARTLP	Cavia porcellus
1	1.00	1	1	0	0	0	0	-0.002	t	69178	VKAETRLNPDLQPTE	Mus musculus
1	1.00	1	1	0	0	0	0	-0.002	t	73021	WRCVVYPGNGFVSASIFGFQ	Oryctolagus cuniculus
1	1.00	1	1	0	0	0	0	-0.002	t	62150	SVIALGSQEGALHQALAGAI	Equus caballus
1	1.00	1	1	0	-0.006	0	0	-0.002	t	78843	TAAQITAGIALHQSNLN	Canis lupus
1	1.00	1	1	0	0.002	0	0	-0.002	t	78843	TAAQITAGIALHQSNLN	Cavia porcellus
1	1.00	1	1	0	0	0	0	-0.002	t	94513	FHPDK	Mus musculus
1	1.00	1	1	0	0	0	0	-0.002	t	72911	WNSTTFHQTLQDP	Mus musculus
1	1.00	1	1	0	0	0	0	-0.002	t	64985	TLNPTI	Mus musculus

1	1.00	1	1	0	0	0	0	-0.002	t	60623	SRALRGD MANLTNQ	Oryctola gus cuniculu s
1	1.00	1	1	0	0	0	0	-0.002	t	71490	VTSGSTKD	Homo sapiens
1	1.00	1	1	0	0	0	0	-0.002	t	78635	EKIERNNK LKQPPPPP NPN	Mus musculu s
1	1.00	1	1	0	0	0	0	-0.002	t	77827	AKALKVEV	Mus musculu s
1	1.00	1	1	0	0	0	0	-0.002	t	69178	VKAETRLN PDLQPTE	Oryctola gus cuniculu s
1	1.00	1	1	0	0	0	0	-0.002	t	70391	VPNLRGDL QVLAQKV ARTLP	Cavia porcellu s
1	1.00	1	1	0	0	0	0	-0.002	t	94491	DRGWGN HCGLFGK GSI	Mus musculu s
1	1.00	1	1	0	0	0	0	-0.002	t	78638	EKSLTSLSE VVLQNRR GLD	Oryctola gus cuniculu s
1	1.00	1	1	0	0	0	0	-0.002	t	72320	WDMMM NW	Homo sapiens
1	1.00	1	1	0	0	0	0	-0.002	t	74009	YGGPANK KNAG	Mus musculu s
1	1.00	1	1	0	0	0	0	-0.002	t	63038	TAVSPTTL RTEVVK	Sus scrofa
1	1.00	1	1	0	0	0	0	-0.002	t	64587	TKINADNP VPILELE	Mus musculu s
1	1.00	1	1	0	0	0	0	-0.002	t	63038	TAVSPTTL RTEVVK	Sus scrofa
1	1.00	1	1	0	0	0	0	-0.002	t	66060	TRLNPD	Mus musculu s

1	1.00	1	1	0	0	0	0	-0.002	t	64587	TKINADNPVPILELE	Mus musculus
1	1.00	1	1	0	0	0	0	-0.002	t	72910	WNSTTFHQTL	Mus musculus
1	1.00	1	1	0	0	0	0	-0.002	t	69183	VKAVTTLLKA	Oryctolagus cuniculus
1	1.00	1	1	0	0	0	0	-0.002	t	64985	TLNPTI	Mus musculus
1	1.00	1	1	0	0	0	0	-0.002	t	72911	WNSTTFHQTLQDP	Mus musculus
1	1.00	1	1	0	0	0	0	-0.002	t	94661	QPDFGGFWDA	Mus musculus
1	1.00	1	1	0	0	0	0	-0.002	t	71284	VTEEI	Oryctolagus cuniculus
1	1.00	1	1	0	0	0	0	-0.002	t	71284	VTEEI	Homo sapiens
1	1.00	1	1	0	-0.003	0	0	-0.002	t	69183	VKAVTTLLKA	Oryctolagus cuniculus
1	1.00	1	1	0	0	0	0	-0.002	t	67875	VCRQGVVDRGWGNGCGLFGK	Equus caballus
1	1.00	1	1	0	0	0	0	-0.002	t	82657	GGYEFLLANDVDNSNPVVQ	Rattus norvegicus
1	1.00	1	1	0	0	0	0	-0.002	t	70489	VPSYSFIRAHDSEVQDLIA	Rattus norvegicus
1	1.00	1	1	0	0	0	0	-0.002	t	61783	STNKAVVSLS	Bos taurus
1	1.00	1	1	0	0	0	0	-0.002	t	77970	PQTQQFIP	Mus musculus

1	1.00	1	1	0	0	0	0	-0.002	t	76290	YVHDNC	Mus musculus
1	1.00	1	1	0	0	0	0	-0.002	t	62353	SVTEEIAEEDKSVTEEIAV	Homo sapiens
1	1.00	1	1	0	0	0	0	-0.002	t	66841	TTTCFRDTDTGK	Mus musculus
1	1.00	1	1	0	0	0	0	-0.002	t	70489	VPSYSFIRAHDSEVQDLIA	Rattus norvegicus
1	1.00	1	1	0	-0.007	0	0	-0.002	t	61945	STTNKDK	Oryctolagus cuniculus
1	1.00	1	1	0	0	0	0	-0.002	t	75951	YTASARGDLAHLTTTHAR	Cavia porcellus
1	1.00	1	1	0	0	0	0	-0.002	t	96367	HSNLDHI	Homo sapiens
1	1.00	1	1	0	0	0	0	-0.002	t	64010	TGYRYDSRTGFFATN	Mus musculus
1	1.00	1	1	0	0	0	0	-0.002	t	61589	STEQNVPDPQVG	Oryctolagus cuniculus
1	1.00	1	1	0	0	0	0	-0.002	t	66304	TSLNDRQGNATK	Oryctolagus cuniculus
1	1.00	1	1	0	0	0	0	-0.002	t	70489	VPSYSFIRAHDSEVQDLIA	Rattus norvegicus
1	1.00	1	1	0	0	0	0	-0.002	t	93172	KTSLWKEMQYNRDI	Mus musculus
1	1.00	1	1	0	0	0	0	-0.002	t	79054	QPDGGQPAVRNERAT	Oryctolagus cuniculus

1	1.00	1	1	0	-0.006	0	0	-0.002	t	79054	QPDGGQP AVRNERAT	Oryctola gus cuniculu s
1	1.00	1	1	0	0	0	0	-0.002	t	70489	VPSYSFIRA HDSEVQD LIA	Rattus norvegic us
1	1.00	1	1	0	0	0	0	-0.002	t	78836	SPKRIGTG DR	Oryctola gus cuniculu s
1	1.00	1	1	0	0	0	0	-0.002	t	97650	SLLTEVET	Mus musculu s
1	1.00	1	1	0	0	0	0	-0.002	t	77974	PVKDSSKQ	Mus musculu s
1	1.00	1	1	0	0	0	0	-0.002	t	70391	VPNLRGDL QVLAQKV ARTLP	Oryctola gus cuniculu s
1	1.00	1	1	0	-0.003	0	0	-0.002	t	76275	YVFYSGNG PKASGGD YCIQS	Oryctola gus cuniculu s
1	1.00	1	1	0	0	0	0	-0.002	t	72065	VYKLDISEA	Mus musculu s
1	1.00	1	1	0	0	0	0	-0.002	t	78001	SEGLKEET	Mus musculu s
1	1.00	1	1	0	0	0	0	-0.002	t	63756	TFTNKLKE KHTDLGK	Mus musculu s
1	1.00	1	1	0	0	0	0	-0.002	t	70391	VPNLRGDL QVLAQKV ARTLP	Cavia porcellu s
1	1.00	1	1	0	0	0	0	-0.002	t	75923	YSWKTWG	Mus musculu s
1	1.00	1	1	0	0	0	0	-0.002	t	61589	STEQNVP DPQVG	Mus musculu s

1	1.00	1	1	0	0	0	0	-0.002	t	70391	VPNLRGDLQVLAQKVARTLP	Cavia porcellus
1	1.00	1	1	0	0	0	0	-0.002	t	61589	STEQNVPDPQVG	Cavia porcellus
1	1.00	1	1	0	0	0	0	-0.002	t	63562	TETPLRKHFNLTPV	Ovis aries
1	1.00	1	1	0	0	0	0	-0.002	t	79040	PGQNLCYTK	Mus musculus
1	1.00	1	1	0	0	0	0	-0.002	t	78424	IPRSRQIDLQIG	Mus musculus
1	1.00	1	1	0	0	0	0	-0.002	t	63562	TETPLRKHFNLTPV	Ovis aries
1	1.00	1	1	0	0	0	0	-0.002	t	73889	YFRTLEDAAH	Mus musculus
1	1.00	1	1	0	0	0	0	-0.002	t	73889	YFRTLEDAAH	Mus musculus
1	1.00	1	1	0	0	0	0	-0.002	t	73889	YFRTLEDAAH	Mus musculus
1	1.00	1	1	0	0	0	0	-0.002	t	68837	VHDCVNITIKQ	Mus musculus
1	1.00	1	1	0	0	0	0	-0.002	t	94483	CYKKVWRDHRGTIIERGC	Mus musculus
1	1.00	1	1	0	0	0	0	-0.002	t	66539	TTASGKLIT	Mus musculus
1	1.00	1	1	0	0	0	0	-0.002	t	78011	SPSTSASS	Mus musculus
1	1.00	1	1	0	0	0	0	-0.002	t	78115	DDPPATVYRYDSRPP	Oryctolagus cuniculus

1	1.00	1	1	0	0	0	0	-0.002	t	71693	VVIANNVDKFVSWGITDFEM	Rattus norvegicus
1	1.00	1	1	0	0	0	0	-0.002	t	63250	TDTRKDINTVTTVAQS	Oryctolagus cuniculus
1	1.00	1	1	0	0	0	0	-0.002	t	70391	VPNLRGDLQVLAQKVARTLP	Mus musculus
1	1.00	1	1	0	-0.003	0	0	-0.002	t	61589	STEQNVPDPQVG	Oryctolagus cuniculus
1	1.00	1	1	0	0	0	0	-0.002	t	61589	STEQNVPDPQVG	Oryctolagus cuniculus
1	1.00	1	1	0	0	0	0	-0.002	t	96854	SAPSISTE	Mus musculus
1	1.00	1	1	0	0	0	0	-0.002	t	97484	LVEETSFIDAGAP	Homo sapiens
1	1.00	1	1	0	0	0	0	-0.002	t	94743	YGTDEWEQWW	Mus musculus
1	1.00	1	1	0	0	0	0	-0.002	t	97484	LVEETSFIDAGAP	Oryctolagus cuniculus
1	1.00	1	1	0	0	0	0	-0.002	t	75696	YSAGGTGRRGDLGPLA	Mus musculus
1	1.00	1	1	0	0	0	0	-0.002	t	70050	VNATKP	Mus musculus
1	1.00	1	1	0	0	0	0	-0.002	t	78107	ATYQSEYLAHRRIPP	Oryctolagus cuniculus
1	1.00	1	1	0	0	0	0	-0.002	t	69178	VKAETRLNPDLQPTE	Oryctolagus cuniculus

1	1.00	1	1	0	0	0	0	-0.002	t	73385	YALADASLKMADPNRFRGKD	Oryctolagus cuniculus
1	1.00	1	1	0	0	0	0	-0.002	t	73385	YALADASLKMADPNRFRGKD	Oryctolagus cuniculus
1	1.00	1	1	0	0	0	0	-0.002	t	69178	VKAETRLNPDLQPTE	Cavia porcellus
1	1.00	1	1	0	0	0	0	-0.002	t	69178	VKAETRLNPDLQPTE	Mus musculus
1	1.00	1	1	0	0	0	0	-0.002	t	78707	KLRERLKQR	Oryctolagus cuniculus
1	1.00	1	1	0	-0.006	0	0	-0.002	t	79026	MSDGAVQPDGGQPAV	Oryctolagus cuniculus
1	1.00	1	1	0	0	0	0	-0.002	t	79026	MSDGAVQPDGGQPAV	Oryctolagus cuniculus
1	1.00	1	1	0	-0.007	0	0	-0.002	t	66649	TTIHELLVRMKRAELYCPRP	Oryctolagus cuniculus
1	1.00	1	1	0	0	0	0	-0.002	t	80002	RKADLEKALEGAM	Oryctolagus cuniculus
1	1.00	1	1	0	0	0	0	-0.002	t	63970	TGT	Mus musculus
1	1.00	1	1	0	0	0	0	-0.002	t	67214	TVWRNRETLMEFEEPHATKQ	Equus caballus
1	1.00	1	1	0	0	0	0	-0.002	t	60783	SRRGDLGSLATRVATQ	Bos taurus
1	1.00	1	1	0	0	0	0	-0.002	t	78159	GALATYQSEYLAHRRIPP	Oryctolagus cuniculus

1	1.00	1	1	0	0	0	0	-0.002	t	66649	TTIHELLVRMKRAELYCPRP	Oryctolagus cuniculus
1	1.00	1	1	0	-0.003	0	0	-0.002	t	66649	TTIHELLVRMKRAELYCPRP	Oryctolagus cuniculus
1	1.00	1	1	0	0	0	0	-0.002	t	61945	STTNKDK	Oryctolagus cuniculus
1	1.00	1	1	0	0	0	0	-0.002	t	64545	TKEEVPPADLSDQVP	Mus musculus
1	1.00	1	1	0	0	0	0	-0.002	t	66108	TRTNPWPALAEGEF	Oryctolagus cuniculus
1	1.00	1	1	0	0	0	0	-0.002	t	60722	SRLGRSSAWV	Mus musculus
1	1.00	1	1	0	0	0	0	-0.002	t	61945	STTNKDK	Cavia porcellus
1	1.00	1	1	0	0	0	0	-0.002	t	64985	TLNPTI	Mus musculus
1	1.00	1	1	0	0	0	0	-0.002	t	73131	WTGVAQD	Oryctolagus cuniculus
1	1.00	1	1	0	0	0	0	-0.002	t	96102	APPLLPH	Homo sapiens
1	1.00	1	1	0	0	0	0	-0.002	t	61945	STTNKDK	Oryctolagus cuniculus
1	1.00	1	1	0	0	0	0	-0.002	t	79009	KTWCDAWCG	Mus musculus
1	1.00	1	1	0	0	0	0	-0.002	t	78711	KQYDPDRP	Oryctolagus cuniculus

1	1.00	1	1	0	0	0	0	-0.002	t	74497	YKQKIIAP	Mus musculus
1	1.00	1	1	0	0	0	0	-0.002	t	70391	VPNLRGDLQVLAQKVARTLP	Mus musculus
1	1.00	1	1	0	0	0	0	-0.002	t	63697	TFLARG	Mus musculus
1	1.00	1	1	0	0	0	0	-0.002	t	78044	TTASSMSS	Mus musculus
1	1.00	1	1	0	0	0	0	-0.002	t	95142	RIKNTDGSIS	Oryctolagus cuniculus
1	1.00	1	1	0	0	0	0	-0.002	t	70391	VPNLRGDLQVLAQKVARTLP	Mus musculus
1	1.00	1	1	0	0	0	0	-0.002	t	63022	TATRLLSSTNSRLC	Mus musculus
1	1.00	1	1	0	0	0	0	-0.002	t	65684	TPPHSARSKFGYGA	Mus musculus
1	1.00	1	1	0	0	0	0	-0.002	t	73132	WTGVTQN	Mus musculus
1	1.00	1	1	0	0	0	0	-0.002	t	70391	VPNLRGDLQVLAQKVARTLP	Cavia porcellus
1	1.00	1	1	0	0	0	0	-0.002	t	70391	VPNLRGDLQVLAQKVARTLP	Cavia porcellus
1	1.00	1	1	0	0	0	0	-0.002	t	70391	VPNLRGDLQVLAQKVARTLP	Cavia porcellus

1	1.00	1	1	0	0	0	0	-0.002	t	73132	WTGVTQN	Mus musculus
1	1.00	1	1	0	0	0	0	-0.002	t	73132	WTGVTQN	Mus musculus
1	1.00	1	1	0	0	0	0	-0.002	t	73132	WTGVTQN	Mus musculus
1	1.00	1	1	0	0	0	0	-0.002	t	73132	WTGVTQN	Mus musculus
1	1.00	1	1	0	0	0	0	-0.002	t	64534	TKDASIDYHE	Mus musculus
1	1.00	1	1	0	0	0	0	-0.002	t	91165	CCELCCNPACAGCY	Sus scrofa
1	1.00	1	1	0	0	0	0	-0.002	t	97348	GLFGAIAGFIEGGW	Oryctolagus cuniculus
1	1.00	1	1	0	0	0	0	-0.002	t	63736	TFQVEVPGSQHIDS	Oryctolagus cuniculus
1	1.00	1	1	0	0	0	0	-0.002	t	96874	SIAPDLSLDFEKLNV	Mus musculus
1	1.00	1	1	0	0	0	0	-0.002	t	70391	VPNLRGDLQVLAQKVARTLP	Cavia porcellus
1	1.00	1	1	0	0	0	0	-0.002	t	70391	VPNLRGDLQVLAQKVARTLP	Mus musculus
1	1.00	1	1	0	0	0	0	-0.002	t	61779	STNDNIKDLLDWYSSG	Oryctolagus cuniculus
1	1.00	1	1	0	0	0	0	-0.002	t	70390	VPNLRGDLQVLAQKVART	Cavia porcellus

1	1.00	1	1	0	0	0	0	-0.002	t	79437	TTQATLITSPFTFSYIREDD	Oryctolagus cuniculus
1	1.00	1	1	0	0	0	0	-0.002	t	63037	TAVSPTTLR	Oryctolagus cuniculus
1	1.00	1	1	0	0	0	0	-0.002	t	67605	VAGLEK	Mus musculus
1	1.00	1	1	0	0	0	0	-0.002	t	62340	SVSVGMKPSPRP	Mus musculus
1	1.00	1	1	0	0	0	0	-0.002	t	78177	HVTDLDKEFL	Mus musculus
1	1.00	1	1	0	0	0	0	-0.002	t	79217	FTWSWESGKYTTETFATNS	Oryctolagus cuniculus
1	1.00	1	1	0	0	0	0	-0.002	t	94741	WEQWW	Mus musculus
1	1.00	1	1	0	0	0	0	-0.002	t	75776	YSKFN	Mus musculus
1	1.00	1	1	0	0	0	0	-0.002	t	70391	VPNLRGDLQVLAQKVARTLP	Cavia porcellus
1	1.00	1	1	0	0	0	0	-0.002	t	95058	AGLQND	Mus musculus
1	1.00	1	1	0	0	0	0	-0.002	t	65535	TPDNNQTSPARR	Oryctolagus cuniculus
1	1.00	1	1	0	0	0	0	-0.002	t	66850	TTTGNAVAPS	Oryctolagus cuniculus
1	1.00	1	1	0	0	0	0	-0.002	t	78946	DAWCGSRGK	Mus musculus

1	1.00	1	1	0	-0.007	0	0	-0.002	t	65535	TPDNNQTSPARR	Oryctolagus cuniculus
1	1.00	1	1	0	0	0	0	-0.002	t	91109	AHFVQQTP	Mus musculus
1	1.00	1	1	0	0	0	0	-0.002	t	72143	VYQVNNLEEIC	Mus musculus
1	1.00	1	1	0	0	0	0	-0.002	t	70391	VPNLRGDLQVLAQKVARTLP	Oryctolagus cuniculus
1	1.00	1	1	0	0	0	0	-0.002	t	62997	TASNKNRGIIKTFS	Mus musculus
1	1.00	1	1	0	0	0	0	-0.002	t	91100	AFQTPVHS	Mus musculus
1	1.00	1	1	0	0	0	0	-0.002	t	70390	VPNLRGDLQVLAQKVART	Bos taurus
1	1.00	1	1	0	0	0	0	-0.002	t	43152	NAGGNAGGNAGGNAGGNAGG	Mus musculus
1	1.00	1	1	0	0	0	0	-0.002	t	51772	QNTEEMENLSDRVAS	Mus musculus
1	1.00	1	1	0	0	0	0	-0.002	t	43730	NEREDERTLTKEYEDIVLK	Oryctolagus cuniculus
1	1.00	1	1	0	0	0	0	-0.002	t	57542	SELSQLS	Mus musculus
1	1.00	1	1	0	0	0	0	-0.002	t	49539	PSTSSKLRPRWTFTSPPVTT	Oryctolagus cuniculus

1	1.00	1	1	0	0	0	0	-0.002	t	49629	PTGDRHKQPLVAPAKQLL	Mus musculus
1	1.00	1	1	0	0	0	0	-0.002	t	48715	PNVDPN	Mus musculus
1	1.00	1	1	0	0	0	0	-0.002	t	51772	QNTEEMENLSDRVAS	Mus musculus
1	1.00	1	1	0	-0.007	0	0	-0.002	t	60415	SQDRHKQKIIAPAKQLL	Oryctolagus cuniculus
1	1.00	1	1	0	0	0	0	-0.002	t	46960	PASTTNKDKL	Oryctolagus cuniculus
1	1.00	1	1	0	0	0	0	-0.002	t	47108	PDHVRTKGPGRCH	Mus musculus
1	1.00	1	1	0	0	0	0	-0.002	t	56699	SAAFYYRV	Mus musculus
1	1.00	1	1	0	0	0	0	-0.002	t	48147	PKSGKELGETQ	Mus musculus
1	1.00	1	1	0	0	0	0	-0.002	t	38946	LQPGKTS	Mus musculus
1	1.00	1	1	0	0	0	0	-0.002	t	45416	NPNANPNANPNANPNENPNA	Homo sapiens
1	1.00	1	1	0	0	0	0	-0.002	t	39183	LRRDLDASREAKKQVEKALE	Homo sapiens
1	1.00	1	1	0	0	0	0	-0.002	t	55825	RSELSQLS	Mus musculus
1	1.00	1	1	0	0	0	0	-0.002	t	38919	LQNDPTTNVA	Mus musculus

1	1.00	1	1	0	0	0	0	-0.002	t	40739	LYDSYAYYG	Mus musculus
1	1.00	1	1	0	0	0	0	-0.002	t	45407	NPNANPNA	Mus musculus
1	1.00	1	1	0	0	0	0	-0.002	t	44978	NMGLIAGAVG	Oryctolagus cuniculus
1	1.00	1	1	0	0	0	0	-0.002	t	51371	QLINTNGSWHIN	Rattus norvegicus
1	1.00	1	1	0	0	0	0	-0.002	t	50759	QFGGHNSVDFEEDT	Oryctolagus cuniculus
1	1.00	1	1	0	0	0	0	-0.002	t	47063	PDCAICWEPSPPWAPE	Oryctolagus cuniculus
1	1.00	1	1	0	0	0	0	-0.002	t	39182	LRRDLDASREAKKQVEKAL	Mus musculus
1	1.00	1	1	0	0.012	0	0	-0.002	t	43233	NANP	Mus musculus
1	1.00	1	1	0	0	0	0	-0.002	t	38918	LQND	Mus musculus
1	1.00	1	1	0	0	0	0	-0.002	t	57569	SENHGNYSAQVGASQ	Mus musculus
1	1.00	1	1	0	0	0	0	-0.002	t	50931	QGPGAPQGPGAPQGPGAP	Mus musculus
1	1.00	1	1	0	0	0	0	-0.002	t	58802	SKDVKIEVYL	Homo sapiens
1	1.00	1	1	0	0	0	0	-0.002	t	50396	QAYQQGVTVD	Oryctolagus cuniculus

1	1.00	1	1	0	0	0	0	-0.002	t	43248	NANPNANPNANPNANPNANP	Mus musculus
1	1.00	1	1	0	0	0	0	-0.002	t	50931	QGPGAPQGPGAPQGPGAP	Mus musculus
1	1.00	1	1	0	0	0	0	-0.002	t	43248	NANPNANPNANPNANPNANP	Mus musculus
1	1.00	1	1	0	0	0	0	-0.002	t	50931	QGPGAPQGPGAPQGPGAP	Mus musculus
1	1.00	1	1	0	0	0	0	-0.002	t	57569	SENHGNYSAQVGASQ	Mus musculus
1	1.00	1	1	0	0	0	0	-0.002	t	40619	LVTVNPFVSVATANS	Mus musculus
1	1.00	1	1	0	0	0	0	-0.002	t	43212	NALSFTNK	Mus musculus
1	1.00	1	1	0	0	0	0	-0.002	t	58582	SIKEDVQF	Oryctolagus cuniculus
1	1.00	1	1	0	0	0	0	-0.002	t	45405	NPNANP	Mus musculus
1	1.00	1	1	0	0	0	0	-0.002	t	43248	NANPNANPNANPNANPNANP	Mus musculus
1	1.00	1	1	0	0	0	0	-0.002	t	60024	SPDVDLGDISGINAS	Mus musculus
1	1.00	1	1	0	0	0	0	-0.002	t	43191	NAKATYEAALKQYEADLAA	Mus musculus
1	1.00	1	1	0	0	0	0	-0.002	t	49629	PTGDRHKQPLVAPAKQLL	Mus musculus

1	1.00	1	1	0	0	0	0	-0.002	t	60024	SPDVDLG DISGINAS	Mus musculu s
1	1.00	1	1	0	0	0	0	-0.002	t	58783	SKAFSNCY PYDVPDYA SL	Oryctola gus cuniculu s
1	1.00	1	1	0	-0.004	0	0	-0.002	t	48670	PNLRGDL QVLAQKV ARTLP	Mus musculu s
1	1.00	1	1	0	0	0	0	-0.002	t	51371	QLINTNGS WHIN	Mus musculu s
1	1.00	1	1	0	0	0	0	-0.002	t	52425	QSRRRGD MST	Oryctola gus cuniculu s
1	1.00	1	1	0	0	0	0	-0.002	t	48912	PPSDIKGK YVKEVEVK	Oryctola gus cuniculu s
1	1.00	1	1	0	0	0	0	-0.002	t	48912	PPSDIKGK YVKEVEVK	Oryctola gus cuniculu s
1	1.00	1	1	0	0	0	0	-0.002	t	43242	NANPNAN PNANP	Oryctola gus cuniculu s
1	1.00	1	1	0	0	0	0	-0.002	t	48670	PNLRGDL QVLAQKV ARTLP	Cavia porcellu s
1	1.00	1	1	0	0	0	0	-0.002	t	43242	NANPNAN PNANP	Homo sapiens
1	1.00	1	1	0	0.004	0	0	-0.002	t	53839	RGDLQVL AQK	Mus musculu s
1	1.00	1	1	0	0	0	0	-0.002	t	43242	NANPNAN PNANP	Homo sapiens

1	1.00	1	1	0	0	0	0	-0.002	t	51371	QLINTNGS WHIN	Mus musculu s
1	1.00	1	1	0	-0.007	0	0	-0.002	t	45931	NSNNKEYT	Oryctola gus cuniculu s
1	1.00	1	1	0	0	0	0	-0.002	t	51371	QLINTNGS WHIN	Mus musculu s
1	1.00	1	1	0	0	0	0	-0.002	t	60415	SQDRHKQ KIIAPAKQL L	Oryctola gus cuniculu s
1	1.00	1	1	0	0	0	0	-0.002	t	51371	QLINTNGS WHIN	Mus musculu s
1	1.00	1	1	0	0	0	0	-0.002	t	43242	NANPNAN PNANP	Homo sapiens
1	1.00	1	1	0	0	0	0	-0.002	t	59374	SLNTGWL AGLFY	Mus musculu s
1	1.00	1	1	0	0	0	0	-0.002	t	39164	LRPGPVSQ PYHHWDT DK	Homo sapiens
1	1.00	1	1	0	0	0	0	-0.002	t	59374	SLNTGWL AGLFY	Rattus norvegic us
1	1.00	1	1	0	0	0	0	-0.002	t	51371	QLINTNGS WHIN	Mus musculu s
1	1.00	1	1	0	0	0	0	-0.002	t	56657	RYSRNAVP NV	Bos taurus
1	1.00	1	1	0	0	0	0	-0.002	t	48536	PLYISNPN Y	Macaca fascicula ris
1	1.00	1	1	0	0	0	0	-0.002	t	59374	SLNTGWL AGLFY	Mus musculu s
1	1.00	1	1	0	0	0	0	-0.002	t	51286	QLAKDKV NE	MD

1	1.00	1	1	0	0	0	0	-0.002	t	57812	SFKVTALNVVSRRYLASLHK	Sus scrofa
1	1.00	1	1	0	0	0	0	-0.002	t	38522	LPHSNL	Rattus norvegicus
1	1.00	1	1	0	0	0	0	-0.002	t	57608	SERQPSL	Mus musculus
1	1.00	1	1	0	0	0	0	-0.002	t	56885	SALTSAIAGFVFSC	Ovis aries
1	1.00	1	1	0	0	0	0	-0.002	t	49450	PSNQDSFQWQ	Mus musculus
1	1.00	1	1	0	0	0	0	-0.002	t	43246	NANPNANPNANPNANP	Mus musculus
1	1.00	1	1	0	0	0	0	-0.002	t	56885	SALTSAIAGFVFSC	Ovis aries
1	1.00	1	1	0	0	0	0	-0.002	t	44978	NMGLIAGAVG	Oryctolagus cuniculus
1	1.00	1	1	0	0	0	0	-0.002	t	39504	LSN	Mus musculus
1	1.00	1	1	0	0	0	0	-0.002	t	46094	NTFYCCELCCNPACAGCY	Oryctolagus cuniculus
1	1.00	1	1	0	0	0	0	-0.002	t	56657	RYSRNAVPNV	Mus musculus
1	1.00	1	1	0	0	0	0	-0.002	t	46094	NTFYCCELCCNPACAGCY	Oryctolagus cuniculus

1	1.00	1	1	0	0	0	0	-0.002	t	49629	PTGDRHKQPLVAPAKQLL	Mus musculus
1	1.00	1	1	0	0	0	0	-0.002	t	51393	QLKLNWFKKGSS	Oryctolagus cuniculus
1	1.00	1	1	0	0	0	0	-0.002	t	46094	NTFYCCELCCNPACAGCY	Oryctolagus cuniculus
1	1.00	1	1	0	0	0	0	-0.002	t	56657	RYSRNAVPNV	Mus musculus
1	1.00	1	1	0	0	0	0	-0.002	t	46094	NTFYCCELCCNPACAGCY	Bos taurus
1	1.00	1	1	0	0	0	0	-0.002	t	60024	SPDVDLGDISGINAS	Mus musculus
1	1.00	1	1	0	0	0	0	-0.002	t	49015	PQIPPNWHIP	Oryctolagus cuniculus
1	1.00	1	1	0	-0.005	0	0	-0.002	t	46094	NTFYCCELCCNPACAGCY	Bos taurus
1	1.00	1	1	0	0	0	0	-0.002	t	45202	NNNSTNSG	Mus musculus
1	1.00	1	1	0	0	0	0	-0.002	t	49015	PQIPPNWHIP	Oryctolagus cuniculus
1	1.00	1	1	0	0	0	0	-0.002	t	46094	NTFYCCELCCNPACAGCY	Sus scrofa
1	1.00	1	1	0	0	0	0	-0.002	t	45303	NPASTTNKDK	Mus musculus
1	1.00	1	1	0	0	0	0	-0.002	t	60024	SPDVDLGDISGINAS	Mus musculus

1	1.00	1	1	0	0	0	0	-0.002	t	44142	NHGNYSA QVGASQ	Mus musculu s
1	1.00	1	1	0	0	0	0	-0.002	t	59210	SLKAVNPS DPLVYKRQ TAKF	Ovis aries
1	1.00	1	1	0	0	0	0	-0.002	t	59210	SLKAVNPS DPLVYKRQ TAKF	Ovis aries
1	1.00	1	1	0	0	0	0	-0.002	t	46094	NTFYCCEL CCNPACA GCY	Oryctola gus cuniculu s
1	1.00	1	1	0	0	0	0	-0.002	t	44142	NHGNYSA QVGASQ	Mus musculu s
1	1.00	1	1	0	0	0	0	-0.002	t	37656	LLPHSNLD	Mus musculu s
1	1.00	1	1	0	0	0	0	-0.002	t	51775	QNTRNIVP VSIVSR	Oryctola gus cuniculu s
1	1.00	1	1	0	0	0	0	-0.002	t	50929	QGPGAP	Mus musculu s
1	1.00	1	1	0	0	0	0	-0.002	t	43246	NANPNAN PNANPNA NP	Mus musculu s
1	1.00	1	1	0	0	0	0	-0.002	t	50929	QGPGAP	Mus musculu s
1	1.00	1	1	0	0	0	0	-0.002	t	58409	SHSAFNM FDFGVGPP	Mus musculu s
1	1.00	1	1	0	0	0	0	-0.002	t	40110	LTPLEELY	Mus musculu s
1	1.00	1	1	0	0	0	0	-0.002	t	51775	QNTRNIVP VSIVSR	Mus musculu s

1	1.00	1	1	0	0	0	0	-0.002	t	49772	PTSGDKIDMTPRAGVLMLE	Mus musculus
1	1.00	1	1	0	0	0	0	-0.002	t	46095	NTFYCCELCCYPACAGCN	Homo sapiens
1	1.00	1	1	0	0	0	0	-0.002	t	58923	SKPTTKQRQNKPPSKP	Mus musculus
1	1.00	1	1	0	0	0	0	-0.002	t	40111	LTPLEELYP	Mus musculus
1	1.00	1	1	0	0	0	0	-0.002	t	38434	LPDKFNAYLANKWV	Mus musculus
1	1.00	1	1	0	0	0	0	-0.002	t	56564	RYKQKIIAPAKQ	Oryctolagus cuniculus
1	1.00	1	1	0	0	0	0	-0.002	t	50666	QEPIVA	Mus musculus
1	1.00	1	1	0	0	0	0	-0.002	t	60024	SPDVDLGDISGINAS	Mus musculus
1	1.00	1	1	0	0	0	0	-0.002	t	58416	SHSQLGPPVGGPP	Mus musculus
1	1.00	1	1	0	0	0	0	-0.002	t	59229	SLKMADPNRFRGKDLP	Mus musculus
1	1.00	1	1	0	0	0	0	-0.002	t	44865	NLSDAHKKNLYDHAL	Oryctolagus cuniculus
1	1.00	1	1	0	0	0	0	-0.002	t	46677	NYNWKR	Mus musculus
1	1.00	1	1	0	0	0	0	-0.002	t	43589	NEECTKLKGESGYCQ	Mus musculus
1	1.00	1	1	0	0	0	0	-0.002	t	51390	QLKLDWFKKGSS	Mus musculus
1	1.00	1	1	0	0	0	0	-0.002	t	57320	SDYEDRYYREN	Mus musculus

1	1.00	1	1	0	0	0	0	-0.002	t	57306	SDVEGLQNDP	Mus musculus
1	1.00	1	1	0	0	0	0	-0.002	t	48597	PNANPN	Mus musculus
1	1.00	1	1	0	0	0	0	-0.002	t	50884	QGILFLQWVRDIIDDF	Mus musculus
1	1.00	1	1	0	-0.007	0	0	-0.002	t	44865	NLSDAHKKNLYDHAL	Oryctolagus cuniculus
1	1.00	1	1	0	0	0	0	-0.002	t	56565	RYKQKIIAPAKQLL	Bos taurus
1	1.00	1	1	0	0	0	0	-0.002	t	43190	NAKATYEAALKQYEADL	Mus musculus
1	1.00	1	1	0	0	0	0	-0.002	t	57303	SDVAGLEKDPVAN	Mus musculus
1	1.00	1	1	0	0	0	0	-0.002	t	48027	PKALFTQQGGAYGRC	Oryctolagus cuniculus
1	1.00	1	1	0	0	0	0	-0.002	t	57292	SDSSFFSYGEIPFG	Oryctolagus cuniculus
1	1.00	1	1	0	0	0	0	-0.002	t	43576	NEDNEKLRKPKHKKLKQPG	Mus musculus
1	1.00	1	1	0	0	0	0	-0.002	t	39092	LRGDLQVLAQKVARTL	Oryctolagus cuniculus
1	1.00	1	1	0	0	0	0	-0.002	t	43246	NANPNANPNANPNANP	Mus musculus
1	1.00	1	1	0	0	0	0	-0.002	t	48620	PNDPPP	Mus musculus

1	1.00	1	1	0	0	0	0	-0.002	t	57754	SFFD	Mus musculus
1	1.00	1	1	0	0	0	0	-0.002	t	41019	MADPNRFRG	Mus musculus
1	1.00	1	1	0	0	0	0	-0.002	t	41019	MADPNRFRG	Mus musculus
1	1.00	1	1	0	0	0	0	-0.002	t	54003	RGTTPPAYG	Mus musculus
1	1.00	1	1	0	0	0	0	-0.002	t	44250	NIIRTKKQ	Mus musculus
1	1.00	1	1	0	0	0	0	-0.002	t	57864	SFPMNEESPLGFSPE	Mus musculus
1	1.00	1	1	0	0	0	0	-0.002	t	39092	LRGDLQVLAQKVARTL	Mus musculus
1	1.00	1	1	0	0	0	0	-0.002	t	39092	LRGDLQVLAQKVARTL	Oryctolagus cuniculus
1	1.00	1	1	0	-0.003	0	0	-0.002	t	39092	LRGDLQVLAQKVARTL	Oryctolagus cuniculus
1	1.00	1	1	0	-0.003	0	0	-0.002	t	39092	LRGDLQVLAQKVARTL	Oryctolagus cuniculus
1	1.00	1	1	0	0	0	0	-0.002	t	43190	NAKATYEAALKQYEADL	Mus musculus
1	1.00	1	1	0	0	0	0	-0.002	t	47427	PESRESLAWQTATNP	Mus musculus
1	1.00	1	1	0	0	0	0	-0.002	t	57766	SFFSYGEI	MD

1	1.00	1	1	0	0	0	0	-0.002	t	41608	MGPNLVL PDQKPPS	Mus musculu s
1	1.00	1	1	0	0	0	0	-0.002	t	56657	RYSRNAVP NV	Bos taurus
1	1.00	1	1	0	-0.004	0	0	-0.002	t	56602	RYNRNAV PNLRGDL QVLAQK	Sus scrofa
1	1.00	1	1	0	0	0	0	-0.002	t	56602	RYNRNAV PNLRGDL QVLAQK	Cavia porcellu s
1	1.00	1	1	0	-0.003	0	0	-0.002	t	55951	RSRSES	Oryctola gus cuniculu s
1	1.00	1	1	0	0	0	0	-0.002	t	55951	RSRSES	Oryctola gus cuniculu s
1	1.00	1	1	0	0	0	0	-0.002	t	57864	SFPMNEES PLGFSPE	Mus musculu s
1	1.00	1	1	0	-0.003	0	0	-0.002	t	55951	RSRSES	Oryctola gus cuniculu s
1	1.00	1	1	0	-0.007	0	0	-0.002	t	55951	RSRSES	Oryctola gus cuniculu s
1	1.00	1	1	0	0	0	0	-0.002	t	55951	RSRSES	Oryctola gus cuniculu s
1	1.00	1	1	0	0	0	0	-0.002	t	54058	RHKQKIVA PVKQTL	Cavia porcellu s
1	1.00	1	1	0	0	0	0	-0.002	t	56583	RYLQEKYN LYNSDVFD GKVQ	Oryctola gus cuniculu s

1	1.00	1	1	0	0	0	0	-0.002	t	54058	RHKQKIVA PVKQTL	Bos taurus
1	1.00	1	1	0	0	0	0	-0.002	t	56657	RYSRNAVP NV	Bos taurus
1	1.00	1	1	0	0	0	0	-0.002	t	54057	RHKQKIVA PVK	Mus musculu s
1	1.00	1	1	0	-0.003	0	0	-0.002	t	54057	RHKQKIVA PVK	Oryctola gus cuniculu s
1	1.00	1	1	0	0	0	0	-0.002	t	46006	NSTLGVKS AREF	Mus musculu s
1	1.00	1	1	0	0	0	0	-0.002	t	45420	NPNDPP	Mus musculu s
1	1.00	1	1	0	0	0	0	-0.002	t	41126	MASLIYRQ LL	Mus musculu s
1	1.00	1	1	0	0	0	0	-0.002	t	56599	RYNLVFSG DSDRICSN RA	Mus musculu s
1	1.00	1	1	0	-0.003	0	0	-0.002	t	55952	RSRSESSIE SF	Oryctola gus cuniculu s
1	1.00	1	1	0	0	0	0	-0.002	t	43177	NAINPRLT PWTYRN	Mus musculu s
1	1.00	1	1	0	0	0	0	-0.002	t	59757	SMSYTWT GA	Mus musculu s
1	1.00	1	1	0	0	0	0	-0.002	t	44251	NIISCNKN DKNQ	Oryctola gus cuniculu s
1	1.00	1	1	0	0	0	0	-0.002	t	43227	NANDPP	Mus musculu s

1	1.00	1	1	0	0	0	0	-0.002	t	53838	RGDLGPLAAR	Mus musculus
1	1.00	1	1	0	0	0	0	-0.002	t	42264	MPDHALRPQYSI	Mus musculus
1	1.00	1	1	0	0	0	0	-0.002	t	53122	RAISSWKQRNRWEWRPD	Felis catus
1	1.00	1	1	0	0	0	0	-0.002	t	60116	SPLLGCIGSTCAE	Mus musculus
1	1.00	1	1	0	0	0	0	-0.002	t	45630	NQSTVKT	Mus musculus
1	1.00	1	1	0	0	0	0	-0.002	t	58422	SHSVTKSLRVFGGPP	Mus musculus
1	1.00	1	1	0	0	0	0	-0.002	t	38522	LPHSNL	Homo sapiens
1	1.00	1	1	0	0	0	0	-0.002	t	50872	QGGNIVDIDFDSVP	Oryctolagus cuniculus
1	1.00	1	1	0	0	0	0	-0.002	t	43085	MYRYPNQVYYRPV	Mus musculus
1	1.00	1	1	0	0	0	0	-0.002	t	37657	LLPHSNLDHI	Oryctolagus cuniculus
1	1.00	1	1	0	0	0	0	-0.002	t	51372	QLINTNGSWHINSTA	Homo sapiens
1	1.00	1	1	0	0	0	0	-0.002	t	51372	QLINTNGSWHINSTA	Homo sapiens
1	1.00	1	1	0	0	0	0	-0.002	t	44904	NLTQHPSAPTMP	Oryctolagus cuniculus

1	1.00	1	1	0	0	0	0	-0.002	t	43764	NETIYNTTL KYGDV	Homo sapiens
1	1.00	1	1	0	0	0	0	-0.002	t	46008	NSTNSGIN	Mus musculu s
1	1.00	1	1	0	-0.003	0	0	-0.002	t	44904	NLTQHPSA PTMP	Oryctola gus cuniculu s
1	1.00	1	1	0	0	0	0	-0.002	t	51372	QLINTNGS WHINSTA	Homo sapiens
1	1.00	1	1	0	0	0	0	-0.002	t	51372	QLINTNGS WHINSTA	Homo sapiens
1	1.00	1	1	0	0	0	0	-0.002	t	11821	EENVEHD AEENVEH DA	Mus musculu s
1	1.00	1	1	0	0	0	0	-0.002	t	13912	EQQRYLN NFRFIELV	Mus musculu s
1	1.00	1	1	0	0	0	0	-0.002	t	23269	GWLAGLF YQHKF	Rattus norvegic us
1	1.00	1	1	0	0	0	0	-0.002	t	18707	GANTE	Mus musculu s
1	1.00	1	1	0	0	0	0	-0.002	t	17470	FQEPFWLE DG	Mus musculu s
1	1.00	1	1	0	0	0	0	-0.002	t	11821	EENVEHD AEENVEH DA	Mus musculu s
1	1.00	1	1	0	-0.003	0	0	-0.002	t	19983	GGQPAVR NER	Oryctola gus cuniculu s
1	1.00	1	1	0	0	0	0	-0.002	t	19983	GGQPAVR NER	Oryctola gus cuniculu s
1	1.00	1	1	0	0	0	0	-0.002	t	24006	HHPITDSD QTRLY	Oryctola gus cuniculu s

1	1.00	1	1	0	0	0	0	-0.002	t	22494	GSRYPGQ GSPG	Mus musculu s
1	1.00	1	1	0	0	0	0	-0.002	t	11208	EAVEAERA GRGTG	Mus musculu s
1	1.00	1	1	0	0	0	0	-0.002	t	17470	FQEPFWLE DG	Mus musculu s
1	1.00	1	1	0	0	0	0	-0.002	t	33780	KTN	Mus musculu s
1	1.00	1	1	0	0	0	0	-0.002	t	23975	HHAEMQ NPVYLIPET VPYIK	Oryctola gus cuniculu s
1	1.00	1	1	0	-0.007	0	0	-0.002	t	34933	LAQKVART LP	Oryctola gus cuniculu s
1	1.00	1	1	0	0	0	0	-0.002	t	34933	LAQKVART LP	Oryctola gus cuniculu s
1	1.00	1	1	0	0	0	0	-0.002	t	32570	KNNNSTN S	Mus musculu s
1	1.00	1	1	0	0	0	0	-0.002	t	23975	HHAEMQ NPVYLIPET VPYIK	Mus musculu s
1	1.00	1	1	0	0	0	0	-0.002	t	34341	KWYISKPQ	Mus musculu s
1	1.00	1	1	0	0	0	0	-0.002	t	11208	EAVEAERA GRGTG	Mus musculu s
1	1.00	1	1	0	-0.002	0	0	-0.002	t	11208	EAVEAERA GRGTG	Mus musculu s
1	1.00	1	1	0	0	0	0	-0.002	t	21763	GPRLQPY	Mus musculu s

1	1.00	1	1	0	0	0	0	-0.002	t	11821	EENVEHDAEENVEHDA	Mus musculus
1	1.00	1	1	0	0	0	0	-0.002	t	21763	GPRLQPY	Mus musculus
1	1.00	1	1	0	0	0	0	-0.002	t	29378	IVNTLNEIYRYLYVR	Mus musculus
1	1.00	1	1	0	0	0	0	-0.002	t	34965	LASLRKAFADTVPVRPPSNY	Homo sapiens
1	1.00	1	1	0	0	0	0	-0.002	t	23973	HGYEKPEELWTAKSRVHPL	Homo sapiens
1	1.00	1	1	0	0	0	0	-0.002	t	12219	EGLQND	Mus musculus
1	1.00	1	1	0	-0.003	0	0	-0.002	t	14670	EVDNEQPTTRAQKLFA	Oryctolagus cuniculus
1	1.00	1	1	0	0	0	0	-0.002	t	30349	KEEKEEKEEKEEKEKEKE	Oryctolagus cuniculus
1	1.00	1	1	0	0	0	0	-0.002	t	11819	EENVEHDA	Homo sapiens
1	1.00	1	1	0	0	0	0	-0.002	t	11819	EENVEHDA	Mus musculus
1	1.00	1	1	0	0	0	0	-0.002	t	11819	EENVEHDA	Oryctolagus cuniculus
1	1.00	1	1	0	0	0	0	-0.002	t	11819	EENVEHDA	Mus musculus
1	1.00	1	1	0	0	0	0	-0.002	t	17408	FPSPYYSPAFTKGEKVDL	Mus musculus

1	1.00	1	1	0	0	0	0	-0.002	t	23269	GWLAGLF YQHKF	Rattus norvegic us
1	1.00	1	1	0	0	0	0	-0.002	t	27113	ILIRGIHHV L	Mus musculu s
1	1.00	1	1	0	0	0	0	-0.002	t	12951	ELAPEDPE DS	Oryctola gus cuniculu s
1	1.00	1	1	0	-0.004	0	0	-0.002	t	14670	EVDNEQP TTRAQKLF A	Mus musculu s
1	1.00	1	1	0	0	0	0	-0.002	t	13482	ENHTKFTS	Mus musculu s
1	1.00	1	1	0	0	0	0	-0.002	t	14670	EVDNEQP TTRAQKLF A	Oryctola gus cuniculu s
1	1.00	1	1	0	0	0	0	-0.002	t	17642	FRRYQEER	Mus musculu s
1	1.00	1	1	0	0	0	0	-0.002	t	11819	EENVEHD A	Homo sapiens
1	1.00	1	1	0	0	0	0	-0.002	t	25807	IEFQQKNN RLLEITREF	Mus musculu s
1	1.00	1	1	0	0	0	0	-0.002	t	13460	ENDVLNQ ETEEEMEK	Oryctola gus cuniculu s
1	1.00	1	1	0	0	0	0	-0.002	t	12225	EGLTATTF EEDGVAA	Mus musculu s
1	1.00	1	1	0	0	0	0	-0.002	t	14749	EVKKKSDS	Mus musculu s
1	1.00	1	1	0	0	0	0	-0.002	t	18363	FWRGDLV FDFQV	Mus musculu s
1	1.00	1	1	0	0	0	0	-0.002	t	12951	ELAPEDPE DS	Oryctola gus cuniculu s

1	1.00	1	1	0	-0.003	0	0	-0.002	t	14670	EVDNEQPTTRAQKLFA	Oryctolagus cuniculus
1	1.00	1	1	0	0	0	0	-0.002	t	21553	GNYNYKYRYLRHGKLRPFER	Cavia porcellus
1	1.00	1	1	0	0	0	0	-0.002	t	17187	FNKTLME	Homo sapiens
1	1.00	1	1	0	0	0	0	-0.002	t	15961	FGDKLIIPDIEKYQS	Mus musculus
1	1.00	1	1	0	0	0	0	-0.002	t	10648	DVKTGAE	Mus musculus
1	1.00	1	1	0	0	0	0	-0.002	t	34965	LASLRKAFADTVPVRPPSNY	Oryctolagus cuniculus
1	1.00	1	1	0	0	0	0	-0.002	t	23934	HGPDWASP	Mus musculus
1	1.00	1	1	0	0	0	0	-0.002	t	31069	KGTFDPLQEPRT	Mus musculus
1	1.00	1	1	0	0	0	0	-0.002	t	31069	KGTFDPLQEPRT	Mus musculus
1	1.00	1	1	0	0	0	0	-0.002	t	14670	EVDNEQPTTRAQKLFA	Mus musculus
1	1.00	1	1	0	0	0	0	-0.002	t	10803	DWEDRYYRENMYR	Mus musculus
1	1.00	1	1	0	0	0	0	-0.002	t	29138	ITVNPIVTEKDSPVNIE	Mus musculus
1	1.00	1	1	0	0	0	0	-0.002	t	29138	ITVNPIVTEKDSPVNIE	Mus musculus
1	1.00	1	1	0	0	0	0	-0.002	t	32535	KNKLEEKANKAMININ	Mus musculus
1	1.00	1	1	0	0	0	0	-0.002	t	11814	EENVEENVEENV	Oryctolagus cuniculus

1	1.00	1	1	0	0	0	0	-0.002	t	12248	EGQKSAVTEY	Oryctolagus cuniculus
1	1.00	1	1	0	0	0	0	-0.002	t	28075	IPSTAFTDIAWVRLPNHY	Oryctolagus cuniculus
1	1.00	1	1	0	0	0	0	-0.002	t	11813	EENVEENV	Homo sapiens
1	1.00	1	1	0	0	0	0	-0.002	t	21763	GPRLQPY	Mus musculus
1	1.00	1	1	0	0	0	0	-0.002	t	22360	GSGVRGDFGSLAPRVARQL	Cavia porcellus
1	1.00	1	1	0	-0.002	0	0	-0.002	t	22756	GTLVRMAPVIG	Mus musculus
1	1.00	1	1	0	0	0	0	-0.002	t	30902	KGAV	Mus musculus
1	1.00	1	1	0	0	0	0	-0.002	t	22756	GTLVRMAPVIG	Mus musculus
1	1.00	1	1	0	0	0	0	-0.002	t	19723	GGESSEELSESSFFNLIT	Homo sapiens
1	1.00	1	1	0	0	0	0	-0.002	t	19623	GFSYYRPPWIL	Mus musculus
1	1.00	1	1	0	0	0	0	-0.002	t	28299	IRGDTFATR	Mus musculus
1	1.00	1	1	0	0	0	0	-0.002	t	22360	GSGVRGDFGSLAPRVARQL	Cavia porcellus
1	1.00	1	1	0	0	0	0	-0.002	t	21084	GLQN	Mus musculus

1	1.00	1	1	0	0	0	0	-0.002	t	17524	FQQFGRDVSDFTDSVRDPKT	Mus musculus
1	1.00	1	1	0	0	0	0	-0.002	t	28331	IRLRHENKDLKARLENAMEV	Mus musculus
1	1.00	1	1	0	0	0	0	-0.002	t	13688	EPLEPFPTQIHKDYK	Homo sapiens
1	1.00	1	1	0	0	0	0	-0.002	t	29899	KANGNGSGDNGDTTW	Oryctolagus cuniculus
1	1.00	1	1	0	-0.003	0	0	-0.002	t	32280	KMADPNRFRGKDLPV	Oryctolagus cuniculus
1	1.00	1	1	0	0	0	0	-0.002	t	28075	IPSTAFTDIAWVRLPNHY	Homo sapiens
1	1.00	1	1	0	0	0	0	-0.002	t	13688	EPLEPFPTQIHKDYK	Homo sapiens
1	1.00	1	1	0	0	0	0	-0.002	t	24564	HPYISKPQ	Mus musculus
1	1.00	1	1	0	0	0	0	-0.002	t	21928	GQPAVRNERATGS	Mus musculus
1	1.00	1	1	0	0	0	0	-0.002	t	36413	LHQPPPQIFLKILPQSGPIG	Homo sapiens
1	1.00	1	1	0	0	0	0	-0.002	t	11821	EENVEHDAEENVEHDA	Mus musculus
1	1.00	1	1	0	0	0	0	-0.002	t	22360	GSGVRGDFGSLAPRVARQL	Cavia porcellus
1	1.00	1	1	0	0	0	0	-0.002	t	23510	GYYYPSDPNTFFYAPPYSA	Sus scrofa
1	1.00	1	1	0	0	0	0	-0.002	t	23269	GWLAGLFYQHKF	Mus musculus

1	1.00	1	1	0	0	0	0	-0.002	t	21191	GLVGLLTP GAKQN	Oryctola gus cuniculu s
1	1.00	1	1	0	0	0	0	-0.002	t	22784	GTPNPNP NPNPNPN PGTT	Mus musculu s
1	1.00	1	1	0	0	0	0	-0.002	t	27997	IPQPQWT P	Mus musculu s
1	1.00	1	1	0	0	0	0	-0.002	t	24243	HLKYATYP PYPQ	Mus musculu s
1	1.00	1	1	0	0	0	0	-0.002	t	26849	IKMVSSLK TKIT	Oryctola gus cuniculu s
1	1.00	1	1	0	0	0	0	-0.002	t	11821	EENVEHD AEENVEH DA	Mus musculu s
1	1.00	1	1	0	0	0	0	-0.002	t	31772	KLCGVL	Homo sapiens
1	1.00	1	1	0	0	0	0	-0.002	t	25146	HWYISKPQ	Mus musculu s
1	1.00	1	1	0	0	0	0	-0.002	t	13899	EQPTTRA Q	Oryctola gus cuniculu s
1	1.00	1	1	0	0	0	0	-0.002	t	30036	KAYSNCYP YDVPDY	Oryctola gus cuniculu s
1	1.00	1	1	0	0	0	0	-0.002	t	23526	HAEL	Mus musculu s
1	1.00	1	1	0	0	0	0	-0.002	t	14592	ETVKTNKK NVTVQEL DLQAR	Oryctola gus cuniculu s
1	1.00	1	1	0	0	0	0	-0.002	t	22401	GSLAAR	Mus musculu s
1	1.00	1	1	0	0	0	0	-0.002	t	14592	ETVKTNKK NVTVQEL DLQAR	Oryctola gus cuniculu s

1	1.00	1	1	0	-0.007	0	0	-0.002	t	13900	EQPTTRAQKLFAMWRI	Oryctolagus cuniculus
1	1.00	1	1	0	0	0	0	-0.002	t	13900	EQPTTRAQKLFAMWRI	Oryctolagus cuniculus
1	1.00	1	1	0	0	0	0	-0.002	t	31018	KGNCSGPTTIR	Oryctolagus cuniculus
1	1.00	1	1	0	0	0	0	-0.002	t	33163	KRIPNKKPGKKTTT	Oryctolagus cuniculus
1	1.00	1	1	0	0	0	0	-0.002	t	14670	EVDNEQPTTRAQKLFA	Mus musculus
1	1.00	1	1	0	0	0	0	-0.002	t	19023	GDLGSIA	Oryctolagus cuniculus
1	1.00	1	1	0	0	0	0	-0.002	t	10770	DVVGVGRL	Mus musculus
1	1.00	1	1	0	0	0	0	-0.002	t	14435	ETIYNTTLKY	Homo sapiens
1	1.00	1	1	0	0	0	0	-0.002	t	14435	ETIYNTTLKY	Homo sapiens
1	1.00	1	1	0	0	0	0	-0.002	t	26024	IEYKVQFATK	Mus musculus
1	1.00	1	1	0	0	0	0	-0.002	t	19266	GEFPRHVKLKNEIENLLDQV	Mus musculus
1	1.00	1	1	0	0	0	0	-0.002	t	12992	ELDLQARR	Mus musculus
1	1.00	1	1	0	0	0	0	-0.002	t	19620	GFSPEEMEAVASKFR	Mus musculus

1	1.00	1	1	0	0	0	0	-0.002	t	22813	GTSESTETSEVSTYSMS	Oryctolagus cuniculus
1	1.00	1	1	0	0	0	0	-0.002	t	11723	EEKKVPINLWLDGKQNTVPL	Oryctolagus cuniculus
1	1.00	1	1	0	-0.003	0	0	-0.002	t	30716	KFDWNTPD	Mus musculus
1	1.00	1	1	0	0	0	0	-0.002	t	34290	KWEDSTSTLTISADSKKTKD	Mus musculus
1	1.00	1	1	0	0	0	0	-0.002	t	30716	KFDWNTPD	Mus musculus
1	1.00	1	1	0	-0.007	0	0	-0.002	t	13899	EQPTTRAQ	Oryctolagus cuniculus
1	1.00	1	1	0	-0.003	0	0	-0.002	t	20179	GHRMAWDMM	Oryctolagus cuniculus
1	1.00	1	1	0	0	0	0	-0.002	t	30299	KEALDKYELENHD	Oryctolagus cuniculus
1	1.00	1	1	0	0	0	0	-0.002	t	30716	KFDWNTPD	Mus musculus
1	1.00	1	1	0	0	0	0	-0.002	t	11723	EEKKVPINLWLDGKQNTVPL	Oryctolagus cuniculus
1	1.00	1	1	0	0	0	0	-0.002	t	11815	EENVEENVEENVEENV	Oryctolagus cuniculus
1	1.00	1	1	0	0.001	0	0	-0.002	t	27997	IPQPQWTP	Mus musculus

1	1.00	1	1	0	0	0	0	-0.002	t	11148	EANNREG VEG	Mus musculu s
1	1.00	1	1	0	0	0	0	-0.002	t	17135	FNCLGMS NRDFLEGV SGATW	Equus caballus
1	1.00	1	1	0	0	0	0	-0.002	t	11148	EANNREG VEG	Mus musculu s
1	1.00	1	1	0	0	0	0	-0.002	t	10801	DWEDRYY RE	Oryctola gus cuniculu s
1	1.00	1	1	0	0	0	0	-0.002	t	10539	DVAGLEK DPVAN	Mus musculu s
1	1.00	1	1	0	0	0	0	-0.002	t	32280	KMADPNR FRGKDLPV	Oryctola gus cuniculu s
1	1.00	1	1	0	0	0	0	-0.002	t	19481	GEYGEVTV DCEPRSGI DTNA	Equus caballus
1	1.00	1	1	0	0	0	0	-0.002	t	27997	IPQPQWT P	Mus musculu s
1	1.00	1	1	0	0	0	0	-0.002	t	28313	IRHQMVLL QL	Mus musculu s
1	1.00	1	1	0	0	0	0	-0.002	t	22282	GSAMSRP MIHF	Mus musculu s
1	1.00	1	1	0	0.001	0	0	-0.002	t	27997	IPQPQWT P	Mus musculu s
1	1.00	1	1	0	0	0	0	-0.002	t	27675	INKRKYDS LKEKL	Homo sapiens
1	1.00	1	1	0	0	0	0	-0.002	t	19983	GGQPAVR NER	Mus musculu s
1	1.00	1	1	0	0	0	0	-0.002	t	9524	DNPASTT NKDK	Oryctola gus cuniculu s
1	1.00	1	1	0	0	0	0	-0.002	t	4149	ARGSLAVT I	Mus musculu s

1	1.00	1	1	0	-0.008	0	0	-0.002	t	4149	ARGSLAVTI	Mus musculus
1	1.00	1	1	0	0	0	0	-0.002	t	9524	DNPASTTNKDK	Oryctolagus cuniculus
1	1.00	1	1	0	0	0	0	-0.002	t	9524	DNPASTTNKDK	Mus musculus
1	1.00	1	1	0	0	0	0	-0.002	t	9524	DNPASTTNKDK	Mus musculus
1	1.00	1	1	0	0	0	0	-0.002	t	9103	DLGPLAAR	Mus musculus
1	1.00	1	1	0	0	0	0	-0.002	t	3245	ANDVDNSNPVVQAEQLNWL	Mus musculus
1	1.00	1	1	0	0	0	0	-0.002	t	9524	DNPASTTNKDK	Mus musculus
1	1.00	1	1	0	0	0	0	-0.002	t	1822	AHTTSDPWHPPG	Mus musculus
1	1.00	1	1	0	0	0	0	-0.002	t	4702	ASQKIQLV	Pan troglodytes
1	1.00	1	1	0	0	0	0	-0.002	t	4149	ARGSLAVTI	Mus musculus
1	1.00	1	1	0	0	0	0	-0.002	t	4331	ASARGDLAHLTTTHARHLP	Mus musculus
1	1.00	1	1	0	0	0	0	-0.002	t	5507	AVPNVRGDLQ	Mus musculus
1	1.00	1	1	0	0	0	0	-0.002	t	7461	DAADL	Mus musculus
1	1.00	1	1	0	0	0	0	-0.002	t	9526	DNPASTTNKDKL	Oryctolagus cuniculus

1	1.00	1	1	0	0	0	0	-0.002	t	3236	ANCASILCKCYTTGT	Mus musculus
1	1.00	1	1	0	0	0	0	-0.002	t	1547	AGHATLREHLRDIKAENTDA	Mus musculus
1	1.00	1	1	0	0	0	0	-0.002	t	3236	ANCASILCKCYTTGT	Mus musculus
1	1.00	1	1	0	0	0	0	-0.002	t	428	AASEALDPHAFHLLLNTYGR	Homo sapiens
1	1.00	1	1	0	0	0	0	-0.002	t	9516	DNNLVSGP	Oryctolagus cuniculus
1	1.00	1	1	0	0	0	0	-0.002	t	3236	ANCASILCKCYTTGT	Mus musculus
1	1.00	1	1	0	0	0	0	-0.002	t	9723	DPPPPNPNDPPPPNPND	Mus musculus
1	1.00	1	1	0	0	0	0	-0.002	t	9621	DPEDSALL	Mus musculus
1	1.00	1	1	0	0	0	0	-0.002	t	8567	DHSDLVAEKQRLEDLGQKFE	Mus musculus
1	1.00	1	1	0	0	0	0	-0.002	t	3825	APSSPGTPGVAAATQAANGG	Mus musculus
1	1.00	1	1	0	0	0	0	-0.002	t	9106	DLGSIA	Mus musculus
1	1.00	1	1	0	0	0	0	-0.002	t	89	AADKPTLDIRMMNIEA	Mus musculus
1	1.00	1	1	0	-0.002	0	0	-0.002	t	7850	DDPPATVYRYDSRPPED	Mus musculus
1	1.00	1	1	0	0	0	0	-0.002	t	6015	CAPESIEFPVSEARVLED	Homo sapiens
1	1.00	1	1	0	-0.007	0	0	-0.002	t	9557	DNTVRET	Oryctolagus cuniculus

1	1.00	1	1	0	0	0	0	-0.002	t	4331	ASARGDLAHLTTTHARHLP	Mus musculus
1	1.00	1	1	0	0	0	0	-0.002	t	7850	DDPPATVYRYDSRPPED	Mus musculus
1	1.00	1	1	0	0	0	0	-0.002	t	4331	ASARGDLAHLTTTHARHLP	Mus musculus
1	1.00	1	1	0	0	0	0	-0.002	t	7492	DAEFRHDSGYEVHHQKL	Mus musculus
1	1.00	1	1	0	0	0	0	-0.002	t	10171	DSLSEEEKELLNRIQVDS	Mus musculus
1	1.00	1	1	0	-0.007	0	0	-0.002	t	9557	DNTVRET	Oryctolagus cuniculus
1	1.00	1	1	0	-0.003	0	0	-0.002	t	8355	DGAVQPDGG	Oryctolagus cuniculus
1	1.00	1	1	0	0	0	0	-0.002	t	8355	DGAVQPDGG	Oryctolagus cuniculus
1	1.00	1	1	0	0	0	0	-0.002	t	33	AAAGAVVGGLGGYMLG	Mus musculus
1	1.00	1	1	0	0	0	0	-0.002	t	5109	ATPYHPPATP	Oryctolagus cuniculus
1	1.00	1	1	0	0	0	0	-0.002	t	632	ADASLKMADPNRFRGKDLP	Mus musculus
1	1.00	1	1	0	0	0	0	-0.002	t	7629	DARSVNGEFPRHVKLKNEIE	Mus musculus
1	1.00	1	1	0	-0.007	0	0	-0.002	t	8356	DGAVQPDGGQPAVRNERA	Oryctolagus cuniculus

1	1.00	1	1	0	0	0	0	-0.002	t	9018	DLAHLTA	Oryctolagus cuniculus
1	1.00	1	1	0	0	0	0	-0.002	t	4331	ASARGDLAHLTTTHARHLP	Mus musculus
1	1.00	1	1	0	0	0	0	-0.002	t	6093	CDCDRTAA	Mus musculus
1	1.00	1	1	0	0	0	0	-0.002	t	8356	DGAVQPDGGQPAVRNERA	Cavia porcellus
1	1.00	1	1	0	0	0	0	-0.002	t	10069	DRYIPYSP	Mus musculus
1	1.00	1	1	0	0	0	0	-0.002	t	89	AADKPTLDIRMMNIEA	Mus musculus
1	1.00	1	1	0	0	0	0	-0.002	t	89	AADKPTLDIRMMNIEA	Mus musculus
1	1.00	1	1	0	0	0	0	-0.002	t	4401	ASFFD	Mus musculus
1	1.00	1	1	0	0	0	0	-0.002	t	3275	ANETIYNTTLKYGDVVGVNT	Homo sapiens
1	1.00	1	1	0	-0.008	0	0	-0.002	t	6139	CDSSDRPRVYTIT	Mus musculus
1	1.00	1	1	0	-0.008	0	0	-0.002	t	4149	ARGSLAVTI	Mus musculus
1	1.00	1	1	0	0	0	0	-0.002	t	9585	DPAF	Mus musculus
1	1.00	1	1	0	0	0	0	-0.002	t	6479	CKGKIQALCENPEWA	Mus musculus
1	1.00	1	1	0	0	0	0	-0.002	t	6226	CFGNSEY	Mus musculus

1	1.00	1	1	0	0	0	0	-0.002	t	6139	CDSSDRPR VYTIT	Mus musculu s
1	1.00	1	1	0	0	0	0	-0.002	t	6139	CDSSDRPR VYTIT	Mus musculu s
1	1.00	1	1	0	0	0	0	-0.002	t	4038	AQPVKIDN ASPAST	Oryctola gus cuniculu s
1	1.00	1	1	0	0	0	0	-0.002	t	9753	DPRVRGLY	Mus musculu s
1	1.00	1	1	0	0	0	0	-0.002	t	9524	DNPASTT NKDK	Cavia porcellu s
1	1.00	1	1	0	-0.003	-0.007	0	-0.002	t	103166	FEKVVNKE SLVISGLRH	Oryctola gus cuniculu s
1	1.00	1	1	-0.01	-0.01	-0.017	-0.01	-0.01	t	114149	YGSLPQKA QGHRPQD E	Rattus norvegic us
1	1.00	1	1	-0.01	-0.013	-0.017	-0.01	-0.01	t	114149	YGSLPQKA QGHRPQD E	Oryctola gus cuniculu s
1	1.00	1	1	-0.01	-0.002	-0.01	-0.01	-0.012	t	156935	PLSKMHI	Gallus gallus
1	1.00	1	1	0.05	0.05	0.054	0.052	0.05	t	70952	VSGGSWV DIVLE	Homo sapiens
1	1.00	1	1	0.02	0.02	0.02	0.02	0.02	t	78398	GLKNDSA VLVYLVGS	Mus musculu s
1	1.00	1	1	0	0	0	0	0	t	119621	NVFSCSV M	Homo sapiens
1	1.00	1	1	-0.01	-0.01	-0.01	-0.01	-0.01	t	125791	CSFWSKYI QTLK	Mus musculu s
1	1.00	1	1	0.03	0.033	0.03	0.03	0.028	t	138315	DAFGGVYS	Mus musculu s
1	1.00	1	1	-0.01	-0.008	-0.027	-0.013	-0.01	t	11439	EDSGSSRK KITCECTKP DS	Homo sapiens

1	1.00	1	1	0.01	0.008	0.008	0.009	0.01	t	120407	APGYKHEIKYNC	Mus musculus
1	1.00	1	1	-0.02	-0.018	-0.019	-0.019	-0.02	t	470	AATRFMKDLY	Homo sapiens
1	1.00	1	1	0.02	0.02	0.02	0.023	0.02	t	4210	ARPDYNPL	Homo sapiens
1	1.00	1	1	0	0	0	-0.003	-0.003	t	37660	LLPHSNLDHILEPSIPWKSK	Homo sapiens
1	1.00	1	1	0.02	0.02	0.009	0.02	0.022	t	111790	SLVEETSFIDAGAPTS	Oryctolagus cuniculus
1	1.00	1	1	-0.03	-0.028	-0.04	-0.029	-0.031	t	65863	TQGVVKERRWTLVNENRPIW	Homo sapiens
1	1.00	1	1	0.02	0.015	0.02	0.021	0.018	t	99874	AEDVGSNKGA	Oryctolagus cuniculus
1	1.00	1	1	0.02	0.023	0.02	0.02	0.021	t	135569	KTNMK	Mus musculus
1	1.00	1	1	0	0	0	0	-0.002	t	151731	SAQSQRAPDR	Mus musculus
1	1.00	1	1	0	0	0	0	-0.002	t	105163	EFRHD	Mus musculus
1	1.00	1	1	0	0	0	0	-0.002	t	104461	EFRHDS	Mus musculus
1	1.00	1	1	-0.03	-0.032	-0.03	-0.023	-0.03	t	123636	PVGPAPGMRP	Homo sapiens
1	1.00	1	1	0	0	-0.007	0	0	t	28742	ISTEPFPNQKHKDLQ	Homo sapiens
1	1.00	1	1	0	0	-0.007	0	0	t	28742	ISTEPFPNQKHKDLQ	Homo sapiens

1	1.00	1	1	0.01	0.006	0.002	0.013	0.01	t	70437	VPPTQSKKKNKNET	Oryctolagus cuniculus
1	1.00	1	1	0	0	0	0	0	t	27727	INQQLNPK	Mus musculus
1	1.00	1	1	0	0	0	0	0	t	27727	INQQLNPK	Homo sapiens
1	1.00	1	1	0	0	0	0	0	t	27727	INQQLNPK	Mus musculus
1	1.00	1	1	0.04	0.048	0.04	0.04	0.038	t	156939	PPEKSHL	Gallus gallus
1	1.00	1	1	0.05	0.047	0.043	0.05	0.05	t	125799	DDDRYSDL	Oryctolagus cuniculus
1	1.00	1	1	0.03	0.03	0.03	0.03	0.03	t	137556	QGEGMTGISYPGCPE	Homo sapiens
1	1.00	1	1	-0.01	-0.01	-0.01	-0.01	-0.01	t	117049	NYKSPLFAVPMT	Mus musculus
1	1.00	1	1	-0.01	-0.01	-0.01	-0.01	-0.01	t	117049	NYKSPLFAVPMT	Homo sapiens
1	1.00	1	1	0	0	0	0	0	t	7061	CSQHLPYIE	Homo sapiens
1	1.00	1	1	-0.02	-0.02	-0.031	-0.018	-0.019	t	67569	VAEKVRNLPA	Homo sapiens
1	1.00	1	1	0.06	0.056	0.053	0.06	0.063	t	111039	TGVPIDPAVPDSSIVPLLES	Bos taurus
1	1.00	1	1	0.03	0.032	0.022	0.03	0.032	t	48771	PPETTTIP	Anas platyrhynchos
1	1.00	1	1	0.01	0.01	0.001	0.004	0.01	t	30318	KECEKYYKEKKGRER	Homo sapiens

1	1.00	1	1	0.07	0.073	0.065	0.07	0.07	t	52313	QSESVEFTKDTQTGMS	Mus musculus
1	1.00	1	1	-0.01	-0.01	-0.008	-0.004	-0.008	t	48981	PQDVKFP	Homo sapiens
1	1.00	1	1	0.03	0.03	0.021	0.03	0.033	t	110941	MHGDTPTLHEYMLDLQPE	Mus musculus
1	1.00	1	1	-0.04	-0.038	-0.04	-0.039	-0.042	t	119910	GQYFAKPRNQGG	Homo sapiens
1	1.00	1	1	-0.01	-0.01	-0.01	-0.01	-0.012	t	97348	GLFGAIAGFIEGGW	Oryctolagus cuniculus
1	1.00	1	1	0	0	0	0.009	0.001	t	132064	SLLGQLSGQV	Homo sapiens
1	1.00	1	1	0.02	0.022	0.02	0.021	0.02	t	32470	KNEEEIAQ	Homo sapiens
1	1.00	1	1	0.04	0.038	0.027	0.037	0.041	t	3474	APAEPEAPAPETEKAEEVEK	Mus musculus
1	1.00	1	1	-0.02	-0.02	-0.027	-0.02	-0.02	t	74476	YKLPDHVRT	Mus musculus
1	1.00	1	1	0.03	0.03	0.024	0.03	0.03	t	91203	DNLAPMSDGAVQPDG	Oryctolagus cuniculus
1	1.00	1	1	0.05	0.054	0.053	0.057	0.05	t	124702	SDDNYDKTED	Homo sapiens
1	1.00	1	1	-0.02	-0.022	-0.019	-0.023	-0.02	t	44268	NIKPLEDKIL	Mus musculus
1	1.00	1	1	0.02	0.02	0.017	0.02	0.021	t	133531	CDTLKPGEC	Mus musculus
1	1.00	1	1	0.03	0.03	0.03	0.03	0.03	t	137882	GQNVLSAD	Homo sapiens
1	1.00	1	1	-0.02	-0.018	-0.026	-0.019	-0.02	t	63551	TETFLRGFL	Homo sapiens

1	1.00	1	1	0.04	0.04	0.04	0.04	0.038	t	145841	KEDFRYAISSTNEIGLLGA	Sus scrofa
1	1.00	1	1	0	0	0.006	-0.003	-0.001	t	66457	TSTFTSL	Homo sapiens
1	1.00	1	1	-0.02	-0.023	-0.018	-0.02	-0.023	t	9595	DPALLLMHELIHVLHGLYG	Oryctolagus cuniculus
1	1.00	1	1	-0.02	-0.023	-0.018	-0.02	-0.023	t	9595	DPALLLMHELIHVLHGLYG	Oryctolagus cuniculus
1	1.00	1	1	0.02	0.017	0.021	0.02	0.022	t	11199	EATALDTS	Oryctolagus cuniculus
1	1.00	1	1	0	0	-0.012	0	0	t	45464	NPRENKLKQPGDRADGQPAG	Homo sapiens
1	1.00	1	1	0.01	0.01	0.002	0.01	0.01	t	4268	ARTGEG	Homo sapiens
1	1.00	1	1	0.02	0.023	0.008	0.02	0.022	t	7765	DDEHVEEPTVA	Mus musculus
1	1.00	1	1	-0.05	-0.048	-0.05	-0.047	-0.052	t	120772	LMLLAQMRRISLFS	Homo sapiens
1	1.00	1	1	-0.03	-0.027	-0.042	-0.03	-0.028	t	95920	TNVARP	Mus musculus
1	1.00	1	1	0.03	0.03	0.03	0.03	0.03	t	108508	KSGPIDIIG	Homo sapiens
1	1.00	1	1	0.01	0.01	0.01	0.01	0.01	t	78208	MPTKSL	Equus caballus
1	1.00	1	1	0.02	0.02	0.008	0.02	0.021	t	40194	LTTDATTFEEVATAEKLV	Oryctolagus cuniculus
1	1.00	1	1	-0.02	-0.02	-0.02	-0.02	-0.02	t	143959	KFPHLAVGF	Oryctolagus cuniculus

1	1.00	1	1	0.01	0.014	0.01	0.007	0.01	t	60413	SQDPYSPSPY	Homo sapiens
1	1.00	1	1	0.01	0.008	0.001	0.013	0.01	t	11148	EANNREGVEG	Mus musculus
1	1.00	1	1	0	0	0	0.004	0	t	142201	KGLRRDLDASREAKKQVEKA	Homo sapiens
1	1.00	1	1	0.02	0.022	0.02	0.019	0.018	t	51830	QPFPSQQPYL	Homo sapiens
1	1.00	1	1	0.02	0.02	0.02	0.02	0.02	t	131865	EPLEVFISA	Homo sapiens
1	1.00	1	1	0.02	0.018	0.02	0.017	0.018	t	122129	KTVTVKEDQVMQQNPPKF	Mus musculus
1	1.00	1	1	0.01	0.013	0.017	0.01	0.009	t	75543	YQRE	Mus musculus
1	1.00	1	1	0.01	0.006	0.001	0.007	0.01	t	27960	IPNGTYRATY	Oryctolagus cuniculus
1	1.00	1	1	0	0	0.016	0.003	-0.003	t	39172	LRQAILCWGELM	Homo sapiens
1	1.00	1	1	-0.01	-0.007	-0.02	-0.01	-0.01	t	58403	SHRGVIADNQAKWAV	Mus musculus
1	1.00	1	1	-0.01	-0.01	-0.02	-0.01	-0.01	t	58403	SHRGVIADNQAKWAV	Mus musculus
1	1.00	1	1	0	-0.002	-0.009	-0.001	0	t	66382	TSQDGNNHQFT	Mus musculus
1	1.00	1	1	-0.01	-0.01	-0.002	-0.008	-0.013	t	149255	ALDKWD	Homo sapiens
1	1.00	1	1	0	0	0	0	-0.002	t	144820	YLEDWIKYNNQK	Mus musculus

1	1.00	1	1	0	0	0	0	-0.002	t	156903	KNRGIIKTFSN	Mus musculus
1	1.00	1	1	0	0	0	0	-0.002	t	151813	CGADSYEMEEDGVRKC	Mus musculus
1	1.00	1	1	0	0	0	0	-0.002	t	108155	TDHGAE	Mus musculus
1	1.00	1	1	0	0	0	0	-0.002	t	116085	EPANPSEK	Mus musculus
1	1.00	1	1	0	0	0	0	-0.002	t	99921	DAEFRHDS	Mus musculus
1	1.00	1	1	0	0	0	0	-0.002	t	103496	PRGYPGQV	Mus musculus
1	1.00	1	1	0	0	0	0	-0.002	t	107084	AKFRHD	Mus musculus
1	1.00	1	1	0	0	0	0	-0.002	t	107228	REEFRHEA	Mus musculus
1	1.00	1	1	0	0	0	0	-0.002	t	105163	EFRHD	Mus musculus
1	1.00	1	1	0	0	0	0	-0.002	t	75950	YTASARGDLAHLTTT	Mus musculus
1	1.00	1	1	0	0	0	0	-0.002	t	68320	VEQHHRRTD	Mus musculus
1	1.00	1	1	0	0	0	0	-0.002	t	64031	THGQWNKPSK	Mus musculus
1	1.00	1	1	0	0	0	0	-0.002	t	74117	YHLENEVARLKK	MD
1	1.00	1	1	0	0	0	0	-0.002	t	51147	QIVGGVYLL	Mus musculus
1	1.00	1	1	0	0	0	0	-0.002	t	45932	NSNNPDWDF	Mus musculus
1	1.00	1	1	0	0	0	0	-0.002	t	14435	ETIYNTTLKY	Homo sapiens

1	1.00	1	1	0	0	0	0	-0.002	t	24591	HQLDPAFGANSTNPD	Mus musculus
1	1.00	1	1	0	0	0	0	-0.002	t	11571	EEEDDDMGFGLFD	Mus musculus
1	1.00	1	1	0	-0.002	0	-0.001	-0.002	t	19636	GFTTNEERYNVFAE	Mus musculus
1	1.00	1	1	0.04	0.036	0.041	0.037	0.04	t	121054	VTGEVIFQTPL	Oryctolagus cuniculus
1	1.00	1	1	0.07	0.07	0.07	0.07	0.069	t	110916	KLDDTENASAYAANA	Mus musculus
1	1.00	1	1	-0.06	-0.058	-0.06	-0.053	-0.06	t	121776	RARARARA	Homo sapiens
1	1.00	1	1	-0.04	-0.037	-0.044	-0.04	-0.04	t	42676	MSTLNTHGAF	Mus musculus
1	1.00	1	1	-0.04	-0.04	-0.044	-0.04	-0.04	t	42676	MSTLNTHGAF	Oryctolagus cuniculus
1	1.00	1	1	-0.04	-0.04	-0.04	-0.04	-0.039	t	115317	KKILDKVGIN	Homo sapiens
1	1.00	1	1	0.03	0.03	0.03	0.03	0.033	t	59714	SMMPQLA	Mus musculus
1	1.00	1	1	0.02	0.022	0.022	0.021	0.02	t	66144	TSAEGDLVGWPSPP	Homo sapiens
1	1.00	1	1	-0.01	-0.01	-0.01	-0.01	-0.012	t	67931	VDEKKM	Mus musculus
1	1.00	1	1	0.04	0.04	0.04	0.04	0.04	t	149084	NPTQHSY	Sus scrofa
1	1.00	1	1	0.05	0.05	0.05	0.05	0.05	t	30151	KDGEGA	Homo sapiens
1	1.00	1	1	0	-0.029	-0.014	0	0.002	t	127731	LSQSPLVLRSSP	Trichosurus vulpecula

1	1.00	1	1	0	0.004	0	0.007	0.002	t	134217	RNVILFSVFDENRSWYLTEN	Homo sapiens
1	1.00	1	1	-0.02	-0.024	-0.017	-0.017	-0.022	t	29379	IVNTLNGFYRSL	Oryctolagus cuniculus
1	1.00	1	1	0	0.003	0	0	-0.001	t	105232	GSNKGAIIGLM	Mus musculus
1	1.00	1	1	0	-0.004	0	-0.001	-0.002	t	61818	STPGIVI	Oryctolagus cuniculus
1	1.00	1	1	0	0	0	0.004	0	t	67299	TWVAGVFVYGGSKTSLYNLR	Homo sapiens
1	1.00	1	1	0	0	0	0.004	0	t	53881	RGGSRERARGRGRGRGEKRP	Homo sapiens
1	1.00	1	1	0	0	0	0.004	0	t	47636	PGEGPSTGPRGQGDGGRRKK	Homo sapiens
1	1.00	1	1	0	0	0	0.004	0	t	53963	RGRGRGEKRPRSPSSQSSSS	Homo sapiens
1	1.00	1	1	0	0	0	0.004	0	t	58193	SGPRHRDGVRRPQKRPSCIG	Homo sapiens
1	1.00	1	1	0	0	0	0.004	0	t	22447	GSPPRRPPPGRRPFFHPVGE	Homo sapiens
1	1.00	1	1	0	0	0	0.004	0	t	18587	GAGGGAGAGGAGAGGGGRGR	Homo sapiens
1	1.00	1	1	0	0	0	0.004	0	t	1433	AGAGGGAGGAGAGGGAGGAG	Homo sapiens
1	1.00	1	1	-0.04	-0.044	-0.045	-0.037	-0.04	t	95308	DGKGYERGHI	Oryctolagus cuniculus
1	1.00	1	1	-0.02	-0.016	-0.013	-0.013	-0.021	t	108212	WPPSFWYHPQ	Homo sapiens
1	1.00	1	1	0.01	0.01	0.003	0.01	0.011	t	73634	YEDRY	Mus musculus

1	1.00	1	1	0.03	0.032	0.035	0.037	0.03	t	113212	VPVLSAKAAQASDLE	Homo sapiens
1	1.00	1	1	0.03	0.03	0.022	0.029	0.03	t	90599	WLQEGKGENKFV	Homo sapiens
1	1.00	1	1	0	-0.005	-0.014	0.002	0.003	t	157091	ANIKGVPTRAET	Canis lupus
1	1.00	1	1	0.03	0.03	0.024	0.03	0.032	t	19375	GENK	Mus musculus
1	1.00	1	1	0	0	0	-0.001	0	t	66509	TSYVG	Homo sapiens
1	1.00	1	1	-0.01	-0.006	-0.017	-0.009	-0.01	t	21497	GNPNRPDGGILT	Homo sapiens
1	1.00	1	1	0	0.002	0	0.007	0	t	107441	HQNWLDG	Homo sapiens
1	1.00	1	1	0.03	0.031	0.019	0.029	0.03	t	19118	GDSSNAGT	Equus caballus
1	1.00	1	1	0.03	0.028	0.019	0.029	0.03	t	19118	GDSSNAGT	Mus musculus
1	1.00	1	1	0.01	0.01	0.003	0.003	0.01	t	119508	QFIGYPI	Homo sapiens
1	1.00	1	1	0.04	0.04	0.04	0.04	0.04	t	6457	CITQYQRESQAYYQRG	Mus musculus
1	1.00	1	1	-0.03	-0.03	-0.03	-0.03	-0.03	t	118557	PPPGMRGP	Homo sapiens
1	1.00	1	1	0.02	0.024	0.02	0.021	0.02	t	36042	LGAALLQS	Homo sapiens
1	1.00	1	1	0.02	0.02	0.009	0.019	0.019	t	28350	IRNVPHPFVALGLKQ	Mus musculus

1	1.00	1	1	-0.02	-0.023	-0.027	-0.02	-0.021	t	108164	TLVCLATG FFPDHVEL	Oryctola gus cuniculu s
1	1.00	1	1	0.03	0.033	0.018	0.03	0.03	t	34927	LAQEAGN FERISGDL	Mus musculu s
1	1.00	1	1	-0.01	-0.012	-0.007	-0.013	-0.014	t	46488	NVSVPSPS STPLLY	Mus musculu s
1	1.00	1	1	-0.01	-0.013	-0.022	-0.011	-0.01	t	51078	QIHTLN	Oryctola gus cuniculu s
1	1.00	1	1	-0.01	-0.008	-0.028	-0.009	-0.008	t	58195	SGPSGPSG TSPSSRS	Homo sapiens
1	1.00	1	1	0.04	0.04	0.028	0.034	0.038	t	57661	SEVEDVIP EGWKADT SYSAK	Homo sapiens
1	1.00	1	1	0	-0.004	0	0.003	-0.001	t	50846	QGDRRCQ SQLERANL	Oryctola gus cuniculu s
1	1.00	1	1	0	-0.004	0	0.003	-0.001	t	50171	PYSPSQDP YSPSPYD	Oryctola gus cuniculu s
1	1.00	1	1	0.02	0.02	0.02	0.02	0.02	t	78397	GLKNDSA VLVLVGS	Mus musculu s
1	1.00	1	1	0.01	0.012	0.01	0.011	0.01	t	7680	DAWREGE EFVVEFDL	Homo sapiens
1	1.00	1	1	0	0	0.002	0	0	t	65678	TPPAYR	Mus musculu s
1	1.00	1	1	0.02	0.02	0.02	0.02	0.02	t	112855	TTPLTNL	Homo sapiens
1	1.00	1	1	-0.01	-0.007	-0.003	-0.01	-0.011	t	133748	YNAELLV	Mus musculu s
1	1.00	1	1	-0.01	-0.008	-0.022	-0.009	-0.01	t	44602	NKSTLQNR RAAVS	Homo sapiens

1	1.00	1	1	0.03	0.03	0.028	0.03	0.028	t	136436	KPVVGMPIVEVW	Gallus gallus
1	1.00	1	1	-0.02	-0.024	-0.018	-0.021	-0.019	t	139500	FHTHISV	Oryctolagus cuniculus
1	1.00	1	1	0.03	0.03	0.03	0.034	0.029	t	131932	KKEKPFSNSKVECQ	Homo sapiens
1	1.00	1	1	-0.02	-0.017	-0.029	-0.02	-0.02	t	78635	EKIERNNKLKQPPPPPNPN	Mus musculus
1	1.00	1	1	-0.03	-0.034	-0.034	-0.033	-0.033	t	49015	PQIPPNWHIP	Oryctolagus cuniculus
1	1.00	1	1	-0.07	-0.077	-0.061	-0.063	-0.07	t	109948	SRYCLSSRLRVSATFW	Homo sapiens
1	1.00	1	1	-0.02	-0.02	-0.021	-0.02	-0.02	t	126331	ISLRPG	Mus musculus
1	1.00	1	1	0.01	0.012	0.013	0.017	0.009	t	125453	KDRLQGMAPAAGAD	Homo sapiens
1	1.00	1	1	-0.02	-0.02	-0.02	-0.02	-0.02	t	66932	TVAEEHV	Homo sapiens
1	1.00	1	1	-0.02	-0.02	-0.02	-0.02	-0.02	t	66932	TVAEEHV	Homo sapiens
1	1.00	1	1	0.04	0.035	0.031	0.04	0.04	t	45603	NQPGVHIQVFEGERAMTKDC	Canis lupus
1	1.00	1	1	0	-0.003	0	0	-0.002	t	45104	NNFVHDCVNI	Oryctolagus cuniculus
1	1.00	1	1	0	0	0	0	-0.002	t	11447	EDTKEPGVLMGGQSE	Mus musculus

1	1.00	1	1	0	0	0	0	-0.002	t	11447	EDTKEPGVLMGGQSE	Mus musculus
1	1.00	1	1	-0.01	-0.01	-0.01	-0.01	-0.012	t	151816	CLTSQAAKQRLE	Sus scrofa
1	1.00	1	1	-0.01	-0.006	-0.013	-0.003	-0.009	t	124068	ELMALHTGQSLEQIER	Homo sapiens
1	1.00	1	1	0.05	0.05	0.047	0.05	0.051	t	20047	GGTEIKYNGE	Mus musculus
1	1.00	1	1	-0.02	-0.018	-0.02	-0.017	-0.022	t	151609	DETPMRGETRKV	Homo sapiens
1	1.00	1	1	0	-0.003	-0.006	0	0	t	33125	KREMAI	Oryctolagus cuniculus
1	1.00	1	1	-0.01	-0.01	-0.02	-0.01	-0.008	t	11313	EDERTLTK	Oryctolagus cuniculus
1	1.00	1	1	0	0.002	0	0.001	-0.002	t	149364	LGGPSGSAALKAAVD	Cavia porcellus
1	1.00	1	1	0.01	0.01	0.01	0.01	0.01	t	79646	SLPTLTL	Oryctolagus cuniculus
1	1.00	1	1	0.01	0.01	0.01	0.01	0.01	t	45004	NMMKYISPPIFL	Oryctolagus cuniculus
1	1.00	1	1	0.01	0.01	0.01	0.01	0.01	t	45004	NMMKYISPPIFL	Oryctolagus cuniculus
1	1.00	1	1	0.01	0.013	0.01	0.01	0.01	t	45004	NMMKYISPPIFL	Mus musculus
1	1.00	1	1	0.01	0.01	0.01	0.01	0.01	t	45004	NMMKYISPPIFL	Mus musculus

1	1.00	1	1	0.01	0.01	0.01	0.01	0.01	t	45004	NMMKYISPPIFL	Oryctolagus cuniculus
1	1.00	1	1	-0.02	-0.016	-0.016	-0.017	-0.02	t	144700	KEWAKRAALP	Homo sapiens
1	1.00	1	1	0.03	0.026	0.028	0.03	0.03	t	20288	GIGVTQNVLYENQKQIA	Mus musculus
1	1.00	1	1	-0.01	-0.007	-0.019	-0.01	-0.009	t	63756	TFTNKLKEKHTDLGK	Mus musculus
1	1.00	1	1	0.05	0.053	0.045	0.05	0.05	t	78703	KKKKTDEAQP	Mus musculus
1	1.00	1	1	0.01	0.008	0.01	0.009	0.008	t	123856	YCDYCDTYLT	Homo sapiens
1	1.00	1	1	-0.01	-0.01	-0.023	-0.008	-0.007	t	98694	ALQTLPAMCNVY	Homo sapiens
1	1.00	1	1	-0.03	-0.034	-0.031	-0.031	-0.03	t	56365	RVMYASSGIG	Oryctolagus cuniculus
1	1.00	1	1	-0.02	-0.023	-0.03	-0.02	-0.018	t	6762	CPFIKPEVKPGTPA	Oryctolagus cuniculus
1	1.00	1	1	0	0	0	0	-0.002	t	151731	SAQSQRAPDR	Mus musculus
1	1.00	1	1	0	0	0	0	-0.002	t	104895	LVEALYLVCGERG	Mus musculus
1	1.00	1	1	0	0	0	0	-0.002	t	79528	CPSKKPYEEVTC	Mus musculus
1	1.00	1	1	0	-0.003	0	0	-0.002	t	79528	CPSKKPYEEVTC	Oryctolagus cuniculus
1	1.00	1	1	0	0	0	0	-0.002	t	79528	CPSKKPYEEVTC	Mus musculus

1	1.00	1	1	0	0	0	0	-0.002	t	79528	CPSKKPYE EVTC	Mus musculu s
1	1.00	1	1	0	0	0	0	-0.002	t	79524	CHTTATIPS SAVTC	Mus musculu s
1	1.00	1	1	0	0	0	0	-0.002	t	79619	NHPPKRQ PG	Mus musculu s
1	1.00	1	1	0	0	0	0	-0.002	t	79619	NHPPKRQ PG	Mus musculu s
1	1.00	1	1	0	-0.003	0	0	-0.002	t	79619	NHPPKRQ PG	Oryctola gus cuniculu s
1	1.00	1	1	0	0	0	0	-0.002	t	79525	CKMWAD AFTSSRGK VVECG	Mus musculu s
1	1.00	1	1	0	0	0	0	-0.002	t	48858	PPPPNPN DPPPPNP ND	Mus musculu s
1	1.00	1	1	0	-0.003	0	0	-0.002	t	58783	SKAFSNCY PYDVPDYA SL	Oryctola gus cuniculu s
1	1.00	1	1	0	-0.003	0	0	-0.002	t	58783	SKAFSNCY PYDVPDYA SL	Oryctola gus cuniculu s
1	1.00	1	1	0	0	0	0	-0.002	t	48858	PPPPNPN DPPPPNP ND	Mus musculu s
1	1.00	1	1	0	0	0	0	-0.002	t	48858	PPPPNPN DPPPPNP ND	Mus musculu s
1	1.00	1	1	0	0	0	0	-0.002	t	19636	GFTTNEER YNVFAE	Mus musculu s
1	1.00	1	1	0	0	0	0	-0.002	t	19636	GFTTNEER YNVFAE	Mus musculu s

1	1.00	1	1	0	0	0	0	-0.002	t	19637	GFTTNEPR YNVFAE	Mus musculu s
1	1.00	1	1	0	0	0	0	-0.002	t	19636	GFTTNEER YNVFAE	Mus musculu s
1	1.00	1	1	0	0	0	0	-0.002	t	9722	DPPPPNP NDPPPPN PN	Mus musculu s
1	1.00	1	1	0	0	0	0	-0.002	t	9722	DPPPPNP NDPPPPN PN	Mus musculu s
1	1.00	1	1	-0.04	-0.042	-0.047	-0.043	-0.04	t	11275	ECWKNAY FDK	Mus musculu s
1	1.00	1	1	0.02	0.022	0.022	0.027	0.021	t	119598	INPESDQP DLSNFMES G	Homo sapiens
1	1.00	1	1	0	0	0.002	0.007	0.001	t	119466	IRGERA	Mus musculu s
1	1.00	1	1	-0.04	-0.04	-0.04	-0.04	-0.038	t	129193	KYYRMM	Homo sapiens
1	1.00	1	1	0	0	0	0	0	t	118536	NITDHGSLI YSFSECAF	Homo sapiens
1	1.00	1	1	0.01	0.01	0.01	0.01	0.01	t	140907	TVSYGEEK PAVL	Oryctola gus cuniculu s
1	1.00	1	1	-0.01	-0.01	-0.01	-0.01	-0.01	t	8566	DHSDLVAE KQRL	Mus musculu s
1	1.00	1	1	0.02	0.022	0.013	0.017	0.02	t	73233	WVVKSTP ASL	Homo sapiens
1	1.00	1	1	-0.07	-0.065	-0.07	-0.07	-0.07	t	49275	PRVVTYKA AA	Homo sapiens
1	1.00	1	1	0	0.002	-0.009	0.003	0.001	t	142141	EIDGPAGQ AEPD	Homo sapiens
1	1.00	1	1	0	0	0	0.004	0	t	135947	KTGHPRYF NQLS	Homo sapiens

1	1.00	1	1	0	0	0	0	0.001	t	61394	SSTKNLIY	Mus musculus
1	1.00	1	1	0	0	0	0	0.001	t	61394	SSTKNLIY	Mus musculus
1	1.00	1	1	0	0	0	0.006	0.001	t	117773	CNSRQTDREDELI	Homo sapiens
1	1.00	1	1	0	0	0	0.006	0.001	t	56335	RVKTWRMH	Homo sapiens
1	1.00	1	1	0	0.002	0	0.006	0.001	t	3274	ANETIYNTTLKYGDV	MD
1	1.00	1	1	0	0	0	0	0.001	t	115497	SSNICNISCD	Homo sapiens
1	1.00	1	1	-0.02	-0.02	-0.02	-0.02	-0.019	t	115530	VAGTWYSLAM	Homo sapiens
1	1.00	1	1	0.01	0.01	0.01	0.01	0.01	t	10782	DVVSTL	Homo sapiens
1	1.00	1	1	0.06	0.06	0.058	0.06	0.06	t	118338	ESEESDDDMGFGLFD	Homo sapiens
1	1.00	1	1	0	0	0	0	-0.001	t	7238	CTTSGFASLFRLGPSQK	Mus musculus
1	1.00	1	1	0.04	0.04	0.043	0.04	0.039	t	125250	SDEFNPCEDIMGYK	Mus musculus
1	1.00	1	1	-0.02	-0.02	-0.023	-0.02	-0.02	t	98729	DIQKVAGTWY	Homo sapiens
1	1.00	1	1	0	-0.007	0.005	0	0	t	74374	YISPYFINTS	Oryctolagus cuniculus
1	1.00	1	1	0	-0.004	0.005	0	0	t	74374	YISPYFINTS	Mus musculus

1	1.00	1	1	0.02	0.02	0.02	0.02	0.019	t	43237	NANPDPN	Mus musculus
1	1.00	1	1	0	-0.003	-0.014	0	0	t	73577	YDQLVTRVVTHEMAHA	Oryctolagus cuniculus
1	1.00	1	1	0.03	0.028	0.027	0.03	0.03	t	120167	RFQSSAVMALQEACEAYL	Mus musculus
1	1.00	1	1	0.02	0.02	0.016	0.02	0.018	t	18994	GDGRQTITNPAYDE	Mus musculus
1	1.00	1	1	0.03	0.03	0.03	0.03	0.03	t	130455	CQGNQDSFTPVVCAAC	Homo sapiens
1	1.00	1	1	0.01	0.01	0.015	0.012	0.01	t	20657	GKPALVPDKE	Homo sapiens
1	1.00	1	1	0.04	0.038	0.035	0.039	0.04	t	72688	WKLQPGDD	Anas platyrhynchos
1	1.00	1	1	0.01	0.008	0.019	0.017	0.01	t	112422	YWASTRE	Homo sapiens
1	1.00	1	1	0.04	0.04	0.04	0.04	0.04	t	13899	EQPTTRAQ	Oryctolagus cuniculus
1	1.00	1	1	0.04	0.04	0.04	0.04	0.04	t	13899	EQPTTRAQ	Cavia porcellus
1	1.00	1	1	0.04	0.04	0.04	0.04	0.04	t	13899	EQPTTRAQ	Oryctolagus cuniculus
1	1.00	1	1	0.04	0.047	0.04	0.04	0.04	t	13899	EQPTTRAQ	Cavia porcellus
1	1.00	1	1	0.01	0.01	0.01	0.01	0.008	t	106646	MHLTQSP	Homo sapiens
1	1.00	1	1	0	-0.002	-0.008	-0.003	0	t	31553	KKGSGGITVK	Homo sapiens

1	1.00	1	1	0	0.004	-0.008	-0.003	0	t	31553	KKGSGGIT VK	Homo sapiens
1	1.00	1	1	-0.03	-0.032	-0.03	-0.023	-0.033	t	134701	KEIRKLQKS THLLIR	Homo sapiens
1	1.00	1	1	-0.03	-0.028	-0.03	-0.023	-0.033	t	134701	KEIRKLQKS THLLIR	Homo sapiens
1	1.00	1	1	-0.04	-0.038	-0.04	-0.033	-0.039	t	144619	DYIWNTL NSG	Homo sapiens
1	1.00	1	1	-0.03	-0.028	-0.032	-0.033	-0.033	t	106089	RRHQSQR PPRRFQG Q	Homo sapiens
1	1.00	1	1	0	0	0	0	0	t	132929	WGENNY M	Homo sapiens
1	1.00	1	1	0.02	0.017	0.011	0.02	0.02	t	49691	PTLDHVLD HVQT	Oryctola gus cuniculu s
1	1.00	1	1	0.02	0.022	0.02	0.027	0.023	t	140582	PWATLVA ES	Homo sapiens
1	1.00	1	1	0.05	0.05	0.047	0.05	0.054	t	61997	STVTEN	Homo sapiens
1	1.00	1	1	0.01	0.008	0.004	0.009	0.01	t	10824	DWSERIRE FM	Mus musculu s
1	1.00	1	1	0	-0.004	0	-0.001	-0.002	t	46859	PALTAVET GATNPL	Oryctola gus cuniculu s
1	1.00	1	1	-0.03	-0.03	-0.05	-0.03	-0.028	t	14678	EVEEIVRD GLVRSHFH	Mus musculu s
1	1.00	1	1	-0.01	-0.01	-0.012	-0.01	-0.01	t	78405	GPGMKND LVPPPSLT	Mus musculu s
1	1.00	1	1	-0.01	-0.01	-0.01	-0.01	-0.01	t	32648	KPAIIPDRE	Homo sapiens

1	1.00	1	1	-0.02	-0.022	-0.028	-0.021	-0.02	t	83781	INPWDSKSDYLG	Mus musculus
1	1.00	1	1	0	-0.004	-0.009	-0.003	-0.001	t	132984	AIQNIRLRHENKDL	Oryctolagus cuniculus
1	1.00	1	1	-0.04	-0.038	-0.025	-0.033	-0.041	t	104286	SRL	Homo sapiens
1	1.00	1	1	0	0	-0.007	0	0	t	128038	KSPKKAKAAKPKKAP	Oryctolagus cuniculus
1	1.00	1	1	0	0	-0.007	0	0	t	128038	KSPKKAKAAKPKKAP	Homo sapiens
1	1.00	1	1	0.01	0.012	0.006	0.007	0.008	t	6314	CGLRAPQRCDLDVES	Homo sapiens
1	1.00	1	1	0.01	0.014	0.01	0.01	0.01	t	57608	SERQPSL	Mus musculus
1	1.00	1	1	-0.02	-0.02	-0.035	-0.02	-0.02	t	111347	GLFFPDKRPKYNLNF	Homo sapiens
1	1.00	1	1	0.02	0.023	0.021	0.02	0.019	t	121471	KIFQDHPLQKTYNY	Mus musculus
1	1.00	1	1	-0.04	-0.038	-0.051	-0.039	-0.04	t	44002	NGIRRPKHLYVSMP	Homo sapiens
1	1.00	1	1	0	0	0	-0.001	-0.003	t	28911	ITGHRMAWDMMMNWS	Homo sapiens
1	1.00	1	1	-0.02	-0.02	-0.02	-0.018	-0.023	t	52121	QQQPFPPQQP	Homo sapiens
1	1.00	1	1	0.04	0.04	0.036	0.04	0.04	t	41057	MAKNEV	Homo sapiens
1	1.00	1	1	-0.02	-0.021	-0.011	-0.019	-0.02	t	42965	MVLLQM	Homo sapiens
1	1.00	1	1	-0.02	-0.018	-0.02	-0.02	-0.021	t	122886	RGTDEYFIRKPP	Rattus norvegicus

1	1.00	1	1	0	0	-0.005	0.002	0	t	113400	FKLGGRDSR	Homo sapiens
1	1.00	1	1	0.02	0.02	0.016	0.02	0.02	t	52106	QQPQQSFPQQ	Mus musculus
1	1.00	1	1	0.02	0.02	0.016	0.02	0.02	t	52106	QQPQQSFPQQ	Mus musculus
1	1.00	1	1	0	0	0	0	0	t	115824	KIAKYIPIQY	Homo sapiens
1	1.00	1	1	0	0.005	0	0.001	-0.001	t	97018	TVTFKATAAT	Homo sapiens(human)
1	1.00	1	1	0.01	0.006	0.01	0.007	0.009	t	134200	PVVNSLDPPLLTRY	Oryctolagus cuniculus
1	1.00	1	1	0.03	0.026	0.033	0.027	0.029	t	15529	FEFPPPPTDE	Oryctolagus cuniculus
1	1.00	1	1	0	-0.001	0.01	-0.003	-0.003	t	30243	KDSKSRGAT	Homo sapiens
1	1.00	1	1	-0.01	-0.01	-0.01	-0.009	-0.01	t	50156	PYQGSGKGVS	Mus musculus
1	1.00	1	1	0.02	0.022	0.02	0.019	0.02	t	149434	QPQPFLPQQP	Homo sapiens
1	1.00	1	1	0.02	0.017	0.019	0.02	0.02	t	63818	TGFADPNTTTG	Oryctolagus cuniculus
1	1.00	1	1	0.01	0.01	0.01	0.01	0.01	t	98999	SGTIK	Homo sapiens
1	1.00	1	1	0	0	-0.012	0	0	t	58928	SKQDIGALKQELAKKDEGNK	Oryctolagus cuniculus

1	1.00	1	1	0.03	0.03	0.03	0.038	0.032	t	110974	QDIVLHLEPQNEIPVDLL	Mus musculus
1	1.00	1	1	0.04	0.036	0.045	0.04	0.04	t	146471	NFDIPEEI	Oryctolagus cuniculus
1	1.00	1	1	-0.01	-0.01	-0.01	-0.01	-0.01	t	110618	GPVGPAGGPGFP	Mus musculus
1	1.00	1	1	0.04	0.038	0.033	0.039	0.042	t	2550	ALGALGRFDEAMQDQ	Mus musculus
1	1.00	1	1	0.04	0.036	0.033	0.039	0.042	t	2550	ALGALGRFDEAMQDQ	Oryctolagus cuniculus
1	1.00	1	1	-0.01	-0.014	-0.015	-0.017	-0.013	t	131104	TGALFKHSENYK	Oryctolagus cuniculus
1	1.00	1	1	0	0	0	0	-0.002	t	52692	QVETEDTKEPGVLMG	Mus musculus
1	1.00	1	1	0	0	0	0	-0.002	t	52692	QVETEDTKEPGVLMG	Mus musculus
1	1.00	1	1	-0.01	-0.01	-0.01	-0.007	-0.01	t	97065	VPKVTKEKPT	Homo sapiens(human)
1	1.00	1	1	0.01	0.012	0.012	0.011	0.01	t	40611	LVTKDIDFD	Homo sapiens
1	1.00	1	1	0.01	0.01	0.01	0.01	0.009	t	47045	PCSICSNNPTCWAICK	Mus musculus
1	1.00	1	1	0.03	0.036	0.033	0.03	0.03	t	141098	VERSESALF	Ovis aries
1	1.00	1	1	0.02	0.02	0.02	0.02	0.018	t	47546	PFPQPQQQLP	Mus musculus
1	1.00	1	1	-0.03	-0.027	-0.039	-0.03	-0.028	t	79815	EVMLRAVFMQQR	Mus musculus

1	1.00	1	1	0.05	0.05	0.05	0.05	0.05	t	104225	RPAVIPDREVLYQEFDEMEE	Homo sapiens
1	1.00	1	1	0.03	0.03	0.03	0.03	0.03	t	134789	RRQGWLKEIRKLQKS	Homo sapiens
1	1.00	1	1	0.03	0.03	0.03	0.03	0.03	t	135899	ASLYHVYEVNLV	Homo sapiens
1	1.00	1	1	0.03	0.03	0.026	0.03	0.03	t	60535	SQQPQQPFPQ	Mus musculus
1	1.00	1	1	0.03	0.03	0.026	0.03	0.03	t	60535	SQQPQQPFPQ	Mus musculus
1	1.00	1	1	0.03	0.028	0.032	0.029	0.03	t	70946	VSGDPRGRYEDI	Mus musculus
1	1.00	1	1	0.01	0.01	0.005	0.01	0.011	t	73634	YEDRY	Mus musculus
1	1.00	1	1	-0.01	-0.01	-0.01	-0.006	-0.01	t	117591	NTHLKAVKMTLKN	Homo sapiens
1	1.00	1	1	-0.01	-0.012	-0.012	-0.007	-0.01	t	51529	QLRRKLEFFTYVRFD	Mus musculus
1	1.00	1	1	0	0	-0.003	0	0	t	35083	LCPFGEVFNATKFPSVY	Homo sapiens
1	1.00	1	1	0.02	0.016	0.017	0.023	0.023	t	100321	NDQIGDSLYSAMTV	Oryctolagus cuniculus
1	1.00	1	1	0	0.002	-0.005	-0.003	-0.001	t	115107	SENSEKTTMPLW	Homo sapiens
1	1.00	1	1	0	-0.012	0	0	0	t	5931	AYSPASKTF	Macaca mulatta
1	1.00	1	1	-0.01	-0.012	-0.019	-0.011	-0.01	t	47136	PDLERYAG	Mus musculus
1	1.00	1	1	-0.02	-0.018	-0.02	-0.021	-0.022	t	17365	FPQPQPFPPQ	Homo sapiens
1	1.00	1	1	0	0	0	0.004	0	t	114941	RQDENYEEAIVHLKVAALGS	Homo sapiens

1	1.00	1	1	0	0	0	0.004	0	t	18588	GAGGGAG GAGAGGG AGGAGA	Homo sapiens
1	1.00	1	1	0	0	0	0.004	0	t	1433	AGAGGGA GGAGAGG GAGGAG	Homo sapiens
1	1.00	1	1	0	0	0	0.004	0	t	1433	AGAGGGA GGAGAGG GAGGAG	Homo sapiens
1	1.00	1	1	0	0	0	0.004	0	t	1433	AGAGGGA GGAGAGG GAGGAG	Homo sapiens
1	1.00	1	1	0.04	0.038	0.053	0.039	0.038	t	138862	KFPED	Mus musculu s
1	1.00	1	1	0.03	0.032	0.023	0.027	0.028	t	106557	KEKEPSKG CCSG	Homo sapiens
1	1.00	1	1	0.01	0.014	0.011	0.011	0.01	t	744	ADPPFS	Homo sapiens
1	1.00	1	1	0.03	0.034	0.029	0.027	0.028	t	98174	KNETPEVT	Homo sapiens
1	1.00	1	1	0.03	0.032	0.029	0.027	0.028	t	98174	KNETPEVT	Homo sapiens
1	1.00	1	1	-0.01	-0.012	-0.012	-0.01	-0.01	t	120172	RKIPKRPG SVHRTPSR Q	Oryctola gus cuniculu s
1	1.00	1	1	0	-0.002	-0.007	-0.001	0	t	70133	VNNKN	Mus musculu s
1	1.00	1	1	-0.01	-0.005	-0.01	-0.01	-0.01	t	11819	EENVEHD A	Aotus
1	1.00	1	1	-0.04	-0.042	-0.042	-0.041	-0.04	t	38923	LQNQGA WP	Anas platyrhy nchos
1	1.00	1	1	0.03	0.032	0.03	0.033	0.028	t	121879	SSQFDPNK QTPSGKK	Homo sapiens
1	1.00	1	1	-0.06	-0.06	-0.065	-0.06	-0.058	t	133676	RIYMRMK HVR	Mus musculu s

1	1.00	1	1	-0.01	-0.008	-0.014	-0.009	-0.008	t	12625	EKAFREL	Homo sapiens
1	1.00	1	1	-0.03	-0.03	-0.037	-0.03	-0.031	t	159026	NLAIHSDL SYWIESRL	Mus musculu s
1	1.00	1	1	-0.06	-0.06	-0.06	-0.056	-0.06	t	115601	DPFYWVS PGVLV	Homo sapiens
1	1.00	1	1	0.01	0.01	0.01	0.01	0.01	t	134077	GYYYPSDP NT	Mus musculu s
1	1.00	1	1	0.02	0.022	0.016	0.017	0.018	t	114918	PNATDKE GKD	Homo sapiens
1	1.00	1	1	0.06	0.06	0.06	0.06	0.06	t	103716	VRERMTT QDVEAITP	Homo sapiens
1	1.00	1	1	0	-0.003	0	0.001	-0.001	t	7491	DAEFRHDS GYEVHHQ K	Oryctola gus cuniculu s
1	1.00	1	1	0.04	0.04	0.04	0.04	0.04	t	106909	TISQLYSSY PSP	Mus musculu s
1	1.00	1	1	-0.02	-0.018	-0.012	-0.019	-0.021	t	29147	ITWIGM	Homo sapiens
1	1.00	1	1	-0.01	-0.012	-0.01	-0.009	-0.01	t	131100	TDSEMDV VRFD	Mus musculu s
1	1.00	1	1	0.05	0.05	0.048	0.049	0.048	t	13984	ERESQAY	Mus musculu s
1	1.00	1	1	0.01	0.01	0.01	0.014	0.01	t	142200	KGLRRDLD ASREAKKQ LEAE	Homo sapiens
1	1.00	1	1	0.03	0.028	0.023	0.023	0.028	t	108202	VVSHFND	Mus musculu s
1	1.00	1	1	0.02	0.013	0.003	0.021	0.022	t	136315	GGGTGPA DGTNATTI	Homo sapiens
1	1.00	1	1	-0.01	-0.022	-0.01	-0.007	-0.01	t	156860	DRWNSM WKESLL	Bos taurus
1	1.00	1	1	0	0.001	-0.001	0.003	-0.001	t	99529	PGQAGQK QILLDAID	Equus caballus

1	1.00	1	1	-0.02	-0.016	-0.02	-0.013	-0.019	t	131904	HCQICHCD VVNLTCE	Homo sapiens
1	1.00	1	1	-0.01	-0.01	-0.008	-0.006	-0.01	t	118432	ISIGYHYPP ICLGRA	Homo sapiens
1	1.00	1	1	-0.05	-0.05	-0.035	-0.044	-0.052	t	33732	KTKIPAVFK I	Homo sapiens
1	1.00	1	1	-0.07	-0.072	-0.077	-0.071	-0.072	t	1165	AESPKKP	Homo sapiens
1	1.00	1	1	0	-0.004	0	0.004	0	t	123565	NVKDYYQ KWM	Oryctolagus cuniculus
1	1.00	1	1	0	-0.015	0	-0.001	0	t	60795	SRSERR	Marmota monax
1	1.00	1	1	-0.03	-0.03	-0.03	-0.03	-0.029	t	115392	NICNISCD KF	Homo sapiens
1	1.00	1	1	0	0.002	-0.018	-0.003	0.002	t	95834	SGPSGTSG PSGT	Homo sapiens
1	1.00	1	1	-0.03	-0.033	-0.03	-0.03	-0.031	t	153415	LPKFHKSP HFYR	Oryctolagus cuniculus
1	1.00	1	1	0.02	0.021	0.023	0.023	0.018	t	31504	KKDGYPVE YDNCAYI	Equus caballus
1	1.00	1	1	0.03	0.029	0.036	0.031	0.03	t	69517	VLGSQE	Homo sapiens
1	1.00	1	1	-0.03	-0.032	-0.03	-0.031	-0.032	t	110385	QLYKTCKQ AGTCPPDII PKV	Mus musculus
1	1.00	1	1	0	0	0.006	0.006	-0.001	t	4912	ATCMQVN LEIMTST	Homo sapiens
1	1.00	1	1	0.04	0.036	0.035	0.033	0.04	t	115135	YSYDVNST EKG	Oryctolagus cuniculus
1	1.00	1	1	0	0	0	0	-0.002	t	72805	WLTEKEGS YP	Mus musculus

1	1.00	1	1	0	0	0	0	-0.002	t	48858	PPPPNPNDPPPPNPND	Mus musculus
1	1.00	1	1	0	0	0	0	-0.002	t	48858	PPPPNPNDPPPPNPND	Mus musculus
1	1.00	1	1	0	0	0	0	-0.002	t	14457	ETLGALASAIKETFEEGTC	Mus musculus
1	1.00	1	1	0	0	0	0	-0.002	t	20838	GLFGAIAGFIE	Mus musculus
1	1.00	1	1	0	0	0	0	-0.002	t	24012	HHPSTNQEQTSLYVQAS	Mus musculus
1	1.00	1	1	0	0	0	0	-0.002	t	20838	GLFGAIAGFIE	Mus musculus
1	1.00	1	1	0	0	0	0	-0.002	t	24012	HHPSTNQEQTSLYVQAS	Mus musculus
1	1.00	1	1	0	0	0	0	-0.002	t	20838	GLFGAIAGFIE	Mus musculus
1	1.00	1	1	0	0	0	0	-0.002	t	22755	GTLVKTITDDQIEV	Mus musculus
1	1.00	1	1	0	0	0	0	-0.002	t	9723	DPPPPNPNDPPPPNPND	Mus musculus
1	1.00	1	1	0	0	0	0	-0.002	t	9723	DPPPPNPNDPPPPNPND	Mus musculus
1	1.00	1	1	0	0	0	0	-0.002	t	9723	DPPPPNPNDPPPPNPND	Mus musculus
1	1.00	1	1	0.05	0.047	0.052	0.05	0.05	t	71006	VSKEEQYYDYEDAT	Oryctolagus cuniculus
1	1.00	1	1	-0.01	-0.01	-0.025	-0.011	-0.01	t	45589	NQMHMLSNKS	Mus musculus
1	1.00	1	1	-0.04	-0.04	-0.045	-0.04	-0.038	t	145832	IPLGTRPP	Mus musculus
1	1.00	1	1	0	-0.012	-0.006	0	0	t	27932	IPLLRTQ	Mus musculus

1	1.00	1	1	0.06	0.06	0.053	0.057	0.059	t	64600	TKKNYSEL DDEEPME LDYP	Homo sapiens
1	1.00	1	1	0.05	0.052	0.049	0.05	0.05	t	34065	KVKEISNQ EPLKI	Myodes glareolu s
1	1.00	1	1	-0.06	-0.058	-0.07	-0.059	-0.061	t	25138	HWRCPPR R	Homo sapiens
1	1.00	1	1	-0.01	-0.012	-0.02	-0.013	-0.01	t	21883	GQHPAKS M	Anas platyrhy nchos
1	1.00	1	1	0.01	0.01	0	0.01	0.01	t	53209	RASLETTN QAIEAIR	Homo sapiens
1	1.00	1	1	0.02	0.02	0.02	0.02	0.021	t	124702	SDDNYDK TED	Homo sapiens
1	1.00	1	1	0.03	0.028	0.027	0.023	0.028	t	122167	PPPITQGE CKQL	Mus musculu s
1	1.00	1	1	0.01	0.01	0.01	0.01	0.01	t	76617	YYTKDTNN NLT	Mus musculu s
1	1.00	1	1	0.01	0.008	0.01	0.01	0.01	t	76617	YYTKDTNN NLT	Mus musculu s
1	1.00	1	1	0	-0.002	0.005	-0.001	-0.002	t	37657	LLPHSNLD HI	Mus musculu s
1	1.00	1	1	0	0	0	0	0	t	112282	QYLSSFT	Homo sapiens
1	1.00	1	1	0.03	0.026	0.03	0.029	0.03	t	46349	NVDENAN AN	Oryctola gus cuniculu s
1	1.00	1	1	0.02	0.02	0.007	0.022	0.023	t	98969	QQPISQQI LQQI	Homo sapiens
1	1.00	1	1	0.01	0.008	0.006	0.009	0.01	t	24918	HTHADGE VHC	Mus musculu s

1	1.00	1	1	-0.01	-0.012	-0.004	-0.009	-0.009	t	78924	AGPEWLL	Mus musculus
1	1.00	1	1	0.01	0.01	0.01	0.01	0.01	t	153474	TLITHIAPYIQT	Mus musculus
1	1.00	1	1	0.03	0.028	0.03	0.023	0.026	t	140014	DSDVGE	Mus musculus
1	1.00	1	1	-0.04	-0.04	-0.03	-0.04	-0.043	t	60032	SPEKPHFPLA	Sus scrofa
1	1.00	1	1	-0.02	-0.02	-0.023	-0.014	-0.02	t	28134	IQALSYALGGDINKV	Homo sapiens
1	1.00	1	1	0	0	-0.002	0	0	t	78416	HPWWTWVSDPNATQP	Mus musculus
1	1.00	1	1	0	0	-0.002	0	0	t	78416	HPWWTWVSDPNATQP	Mus musculus
1	1.00	1	1	-0.01	-0.012	-0.03	-0.017	-0.01	t	128067	NYVADNFFYNVEGRWGSQ	Mus musculus
1	1.00	1	1	0.02	0.018	0.022	0.02	0.019	t	122895	RWLPPVYEDGFS	Oryctolagus cuniculus
1	1.00	1	1	0	-0.002	0	0.001	-0.002	t	12184	EGHRMAWDMMMNWS	Homo sapiens
1	1.00	1	1	0.01	0.01	0.001	0.01	0.011	t	97023	VATRSQTDTSEKDC	Oryctolagus cuniculus
1	1.00	1	1	0.04	0.043	0.021	0.04	0.045	t	34537	KYNDDDTFTVKVGD	Mus musculus
1	1.00	1	1	0	0.004	0	0.001	0	t	51391	QLKLNW	Homo sapiens
1	1.00	1	1	-0.02	-0.02	-0.018	-0.02	-0.02	t	40563	LVRMKRA	Mus musculus

1	1.00	1	1	-0.02	-0.023	-0.018	-0.02	-0.02	t	40563	LVRMKRA	Oryctolagus cuniculus
1	1.00	1	1	0.03	0.03	0.03	0.03	0.028	t	106850	SLQQLYDEYPGP	Mus musculus
1	1.00	1	1	0.01	0.01	0.01	0.013	0.01	t	54431	RKTKRNTN	Homo sapiens
1	1.00	1	1	-0.03	-0.03	-0.032	-0.03	-0.03	t	67916	VDCSRFPNA	Homo sapiens
1	1.00	1	1	0.05	0.053	0.048	0.05	0.047	t	6341	CGRECGIKKGSSGYC	Equus caballus
1	1.00	1	1	0.05	0.053	0.048	0.05	0.047	t	6341	CGRECGIKKGSSGYC	Equus caballus
1	1.00	1	1	0.03	0.032	0.024	0.027	0.028	t	6722	CNRAFNPVCGTDGVTYDN	Homo sapiens
1	1.00	1	1	-0.02	-0.018	-0.017	-0.017	-0.02	t	151713	RGEARK	Homo sapiens
1	1.00	1	1	-0.03	-0.032	-0.041	-0.031	-0.03	t	47491	PFFRKYFGNDTA	Mus musculus
1	1.00	1	1	-0.01	-0.008	-0.007	-0.011	-0.01	t	45602	NQPGHL	Cavia porcellus
1	1.00	1	1	-0.01	-0.003	-0.007	-0.011	-0.01	t	45602	NQPGHL	Cavia porcellus
1	1.00	1	1	-0.01	-0.014	-0.009	-0.013	-0.01	t	25224	IAAISM	Oryctolagus cuniculus
1	1.00	1	1	0.04	0.042	0.033	0.037	0.038	t	106378	EEVKTTEQKR	Homo sapiens
1	1.00	1	1	0.01	0.01	0.01	0.011	0.007	t	153251	AKAFSYWTPFDGKSLYSST	Mus musculus
1	1.00	1	1	-0.04	-0.043	-0.041	-0.04	-0.042	t	78115	DDPPATVYRYDSRPP	Oryctolagus cuniculus

1	1.00	1	1	0.03	0.03	0.03	0.03	0.03	t	158602	PLELSVY	Homo sapiens
1	1.00	1	1	0.03	0.03	0.03	0.03	0.03	t	78225	PFSNLDF M	Homo sapiens
1	1.00	1	1	0.01	0.01	0.01	0.01	0.01	t	99325	IAVPTGVA FWLYNDH	Homo sapiens
1	1.00	1	1	-0.01	-0.013	-0.01	-0.01	-0.009	t	72484	WGNGCGL FG	Mus musculu s
1	1.00	1	1	-0.01	-0.01	-0.01	-0.01	-0.01	t	103002	AAASLAAI AIAFLAG	Homo sapiens
1	1.00	1	1	0.02	0.023	0.02	0.02	0.02	t	101953	PDPLSPGE	Mus musculu s
1	1.00	1	1	0	0	0	0	0	t	59209	SLKAVNPS DPLVYK	Ovis aries
1	1.00	1	1	0	-0.003	0	0	0	t	156907	KVGDPLW	Oryctola gus cuniculu s
1	1.00	1	1	0	0	0	0	0	t	156907	KVGDPLW	Mus musculu s
1	1.00	1	1	-0.04	-0.04	-0.04	-0.04	-0.04	t	111561	MPPCNYK	Homo sapiens
1	1.00	1	1	0.03	0.028	0.024	0.027	0.029	t	48858	PPPPNPN DPPPPNP ND	Mus musculu s
1	1.00	1	1	-0.01	-0.01	-0.025	-0.01	-0.009	t	43226	NANANPN	Mus musculu s
1	1.00	1	1	0	-0.003	0	0	-0.002	t	103839	DAEFRHDS GYEVH	Oryctola gus cuniculu s
1	1.00	1	1	0.02	0.024	0.02	0.021	0.02	t	61112	SSIPNGTY	Homo sapiens
1	1.00	1	1	0.02	0.02	0.017	0.024	0.02	t	33874	KTSVDCN MYICGDST EC	Homo sapiens

1	1.00	1	1	0	-0.002	0	-0.001	0	t	59374	SLNTGWL AGLFY	Mus musculu s
1	1.00	1	1	0	-0.002	0	-0.001	0	t	59374	SLNTGWL AGLFY	Mus musculu s
1	1.00	1	1	-0.02	-0.02	-0.025	-0.018	-0.02	t	118144	SLCQSLRE R	Homo sapiens
1	1.00	1	1	0	0	-0.001	0.004	0	t	30661	KEVPALTA VETGAT	Homo sapiens
1	1.00	1	1	0	0.002	0	0.003	0	t	63086	TCMTTAQ GTSMYPSC	Homo sapiens
1	1.00	1	1	0	0	0	0.003	0	t	7171	CTKPTDG NC	Homo sapiens
1	1.00	1	1	0	0.004	-0.004	-0.003	0.002	t	39083	LRFFVAPF PE	Homo sapiens
1	1.00	1	1	-0.02	-0.017	-0.02	-0.02	-0.019	t	6276	CGAVIDDC	Mus musculu s
1	1.00	1	1	-0.02	-0.02	-0.02	-0.02	-0.019	t	115197	CVKKILDK VG	Homo sapiens
1	1.00	1	1	0.01	0.012	0.01	0.013	0.01	t	119579	FKDYSSVV RPVEDHR Q	Homo sapiens
1	1.00	1	1	-0.04	-0.04	-0.054	-0.04	-0.039	t	13100	ELIRGTG	Mus musculu s
1	1.00	1	1	0.02	0.02	0.02	0.02	0.02	t	78225	PFSNLDF M	Homo sapiens
1	1.00	1	1	0	0	0	0	0	t	106651	MHSPLPI	Homo sapiens
1	1.00	1	1	0	0	0	0	0	t	112392	VTMTCKS	Homo sapiens
1	1.00	1	1	0	0	0	0	0	t	106651	MHSPLPI	Homo sapiens
1	1.00	1	1	0.01	0.01	0.01	0.01	0.01	t	153838	HQKRTAM FQDPQER	Cavia porcellu s

1	1.00	1	1	0.01	0.003	0.01	0.01	0.01	t	153838	HQKRTAMFQDPQER	Oryctolagus cuniculus
1	1.00	1	1	-0.01	-0.008	-0.01	-0.01	-0.01	t	12550	EIPQPQWT	Anas platyrhynchos
1	1.00	1	1	-0.01	-0.01	-0.01	-0.013	-0.013	t	110759	AAHRKESYSIYVYKVLKQVH	Mus musculus
1	1.00	1	1	-0.02	-0.02	-0.02	-0.02	-0.021	t	106685	NLLEFRH	Mus musculus
1	1.00	1	1	0.01	0.013	0.002	0.01	0.008	t	24369	HNDKRADPAFVCRQGVVDRG	Equus caballus
1	1.00	1	1	-0.02	-0.016	-0.018	-0.02	-0.019	t	136441	KRQKRF	Gallus gallus
1	1.00	1	1	-0.01	-0.01	-0.017	-0.01	-0.012	t	133577	FVVDMMERLRISQKWVRVA	Mus musculus
1	1.00	1	1	0.01	0.01	0.008	0.01	0.01	t	110606	GNPGTDGIPGAKG	Rattus norvegicus
1	1.00	1	1	0.02	0.02	0.015	0.018	0.02	t	52439	QSSWPKLEQ	Homo sapiens
1	1.00	1	1	0.01	0.01	0.005	0.01	0.009	t	78687	IRVRKRKGSNKKAEDTLED	Mus musculus
1	1.00	1	1	-0.02	-0.02	-0.021	-0.02	-0.02	t	144804	TVFVKPPPEKSW	Mus musculus
1	1.00	1	1	0	0.001	-0.001	0.003	-0.001	t	99621	RNAMRHLRPEDTLEE	Equus caballus
1	1.00	1	1	0.01	0.01	0.006	0.01	0.01	t	56200	RTTSGLT	Homo sapiens
1	1.00	1	1	0.04	0.042	0.036	0.037	0.038	t	114949	SKEHDGECKE	Homo sapiens
1	1.00	1	1	0.02	0.016	0.007	0.02	0.02	t	149731	GVSDYHKNLINN	Mus musculus
1	1.00	1	1	0.02	0.02	0.015	0.02	0.022	t	3881	APTYSWGE	Mus musculus

1	1.00	1	1	0.03	0.032	0.023	0.029	0.03	t	73590	YDTADII	Felis catus
1	1.00	1	1	0	0	0	0	-0.002	t	13656	EPGVLMG GQSESVEF	Mus musculu s
1	1.00	1	1	-0.01	-0.012	-0.021	-0.013	-0.01	t	638	ADAVSRKK MD	Mus musculu s
1	1.00	1	1	0.02	0.023	0.021	0.02	0.019	t	15385	FDFSGVVE G	Mus musculu s
1	1.00	1	1	0.01	0.01	0.006	0.01	0.01	t	49108	PQQQPFP QPQ	Mus musculu s
1	1.00	1	1	0.01	0.01	0.006	0.01	0.01	t	49108	PQQQPFP QPQ	Mus musculu s
1	1.00	1	1	-0.01	-0.01	-0.01	-0.01	-0.01	t	149345	KDFPILPGE IF	Homo sapiens
1	1.00	1	1	0	0	-0.001	-0.006	-0.002	t	20021	GGSILSGFT LEFL	Homo sapiens
1	1.00	1	1	0.04	0.042	0.046	0.047	0.039	t	2039	AINPESDQ PDLSNF	Homo sapiens
1	1.00	1	1	0.01	0.01	0.002	0.008	0.01	t	2220	AKKQVEK ALEEANSK LAALE	Homo sapiens
1	1.00	1	1	0.02	0.02	0.02	0.02	0.02	t	111556	MPLQNSK	Homo sapiens
1	1.00	1	1	0.02	0.017	0.017	0.02	0.02	t	21501	GNQHVDD RPS	Oryctola gus cuniculu s
1	1.00	1	1	0.01	0.01	0.015	0.016	0.013	t	115912	QAPEY	Homo sapiens
1	1.00	1	1	0.03	0.034	0.03	0.031	0.03	t	19101	GDREIGEK	Homo sapiens
1	1.00	1	1	0	0.002	-0.013	-0.003	-0.001	t	115090	NLLRFFVA PFPE	Homo sapiens

1	1.00	1	1	-0.03	-0.026	-0.026	-0.03	-0.032	t	1463	AGDCAVRQ	Capra hircus
1	1.00	1	1	-0.01	-0.009	-0.011	-0.007	-0.011	t	99090	AFLDVVPGQAGQKQI	Equus caballus
1	1.00	1	1	0	0.002	0.001	-0.002	-0.002	t	106825	SEIYRKYFGN	Homo sapiens
1	1.00	1	1	0.01	0.008	0.006	0.013	0.01	t	35869	LFKEYKLTRPY	Mus musculus
1	1.00	1	1	0.02	0.022	0.008	0.021	0.022	t	7765	DDEHVEEPTVA	Homo sapiens
1	1.00	1	1	0	0.004	0.007	0.003	-0.001	t	55174	RPIDDFDQGWGPITY	Homo sapiens
1	1.00	1	1	-0.01	-0.01	-0.01	-0.01	-0.01	t	79400	SRPTML	Mus musculus
1	1.00	1	1	0.01	0.01	0.008	0.01	0.009	t	65974	TQYERE	Mus musculus
1	1.00	1	1	0.02	0.017	0.018	0.02	0.018	t	79394	SLNAGLAPETTQATLI	Oryctolagus cuniculus
1	1.00	1	1	0	0.015	0	0.007	0.002	t	125478	LHQEYEENLGDSIVGYKEKS	Homo sapiens
1	1.00	1	1	0.06	0.06	0.06	0.06	0.06	t	112146	GSGTDFT	Homo sapiens
1	1.00	1	1	0.03	0.034	0.033	0.029	0.026	t	62289	SVPSSSS	Mus musculus
1	1.00	1	1	-0.02	-0.02	-0.02	-0.014	-0.02	t	158703	CKEADIKTALEARKA	Homo sapiens
1	1.00	1	1	-0.01	-0.012	-0.01	-0.007	-0.01	t	25852	IEKKIAKMEKASSVFNVV	Mus musculus
1	1.00	1	1	-0.04	-0.038	-0.041	-0.033	-0.04	t	144710	KNPWPNVDAH	Homo sapiens

1	1.00	1	1	-0.01	-0.008	-0.01	-0.007	-0.01	t	40578	LVSFGVWIRTPPAYRP	Homo sapiens
1	1.00	1	1	-0.01	-0.008	-0.01	-0.007	-0.01	t	40578	LVSFGVWIRTPPAYRP	Homo sapiens
1	1.00	1	1	0.01	0.01	0.005	0.01	0.012	t	22303	GSDYEDRYYRENM	Mus musculus
1	1.00	1	1	0	0.002	-0.005	0.007	0	t	134410	IIWRSTQG	Homo sapiens
1	1.00	1	1	0	0	0	0	-0.001	t	153284	PTPLSPPLRNTHPQA	Mus musculus
1	1.00	1	1	0	-0.003	0	0	-0.002	t	108382	GGVVIA	Oryctolagus cuniculus
1	1.00	1	1	0.01	0.014	0.01	0.011	0.01	t	57737	SFDASQSTMA	Homo sapiens
1	1.00	1	1	0	0	-0.004	-0.001	-0.001	t	51296	QLCQLL	Mus musculus
1	1.00	1	1	0.01	0.01	0.011	0.007	0.01	t	71049	VSMFSVGPSQ	Homo sapiens
1	1.00	1	1	0.01	0.007	-0.005	0.01	0.01	t	107422	GQIPLRFNGEVKNPGSVV	Oryctolagus cuniculus
1	1.00	1	1	0.04	0.04	0.041	0.04	0.042	t	66387	TSQKETLHSTSP	Homo sapiens
1	1.00	1	1	-0.02	-0.017	-0.014	-0.02	-0.021	t	69462	VLELAA	Mus musculus
1	1.00	1	1	-0.04	-0.038	-0.04	-0.033	-0.04	t	121811	RRARARAR	Homo sapiens
1	1.00	1	1	0	0.004	0	0.003	0	t	108844	DAGVIQSPRHEVTEMG	Homo sapiens

1	1.00	1	1	0	-0.004	-0.007	-0.003	-0.001	t	135446	AKAQLR	Oryctolagus cuniculus
1	1.00	1	1	0.01	0.01	0.01	0.01	0.01	t	100216	KLVFFAEDVG	Homo sapiens(human)
1	1.00	1	1	0.01	0.01	0.01	0.01	0.01	t	111416	IFQLCKITLTADVMT	Mus musculus
1	1.00	1	1	0.01	0.014	0.01	0.017	0.011	t	120066	LKQQWVDYNLKWNPDD	Homo sapiens
1	1.00	1	1	0.03	0.028	0.035	0.029	0.027	t	6361	CGVVNNKVAT	Mus musculus
1	1.00	1	1	0	-0.002	0.001	0.003	-0.001	t	9913	DQTIDWFQPNNKRNQ	Sus scrofa
1	1.00	1	1	-0.01	-0.012	-0.013	-0.007	-0.009	t	116731	GLGESRK	Mus musculus
1	1.00	1	1	-0.02	-0.02	-0.015	-0.02	-0.02	t	108382	GGVVIA	Mus musculus
1	1.00	1	1	0.01	0.006	-0.003	0.007	0.01	t	12932	ELADALLEKG	Oryctolagus cuniculus
1	1.00	1	1	0	0	0	0.002	0	t	47026	PCKPLLRDDITFMV	Homo sapiens
1	1.00	1	1	-0.01	-0.013	-0.008	-0.01	-0.011	t	4885	ATALDTSNKFADFLQI	Oryctolagus cuniculus
1	1.00	1	1	-0.01	-0.01	-0.006	-0.01	-0.012	t	43212	NALSFTNK	Mus musculus
1	1.00	1	1	-0.01	-0.007	-0.006	-0.01	-0.012	t	43212	NALSFTNK	Mus musculus
1	1.00	1	1	0.02	0.02	0.02	0.02	0.02	t	134187	PMSTPSAT	Mus musculus

1	1.00	1	1	0	-0.004	0	-0.003	-0.004	t	97605	RIVRGPANLRNLKEFKE	Oryctolagus cuniculus
1	1.00	1	1	0	-0.002	0	-0.002	-0.003	t	138192	HQTPLST	Mus musculus
1	1.00	1	1	0	-0.002	0	-0.002	-0.003	t	138202	LPTITNT	Mus musculus
1	1.00	1	1	0	-0.002	0	-0.002	-0.003	t	138198	KPTSTLT	Mus musculus
1	1.00	1	1	0	-0.002	0	-0.002	-0.003	t	138166	FHEFWPT	Mus musculus
1	1.00	1	1	0.01	0.01	0.001	0.01	0.01	t	135974	PSNSASVPALTA	Homo sapiens
1	1.00	1	1	0.03	0.028	0.026	0.029	0.03	t	65664	TPNATQPELAPEDPE	Mus musculus
1	1.00	1	1	0.03	0.026	0.026	0.029	0.03	t	65664	TPNATQPELAPEDPE	Oryctolagus cuniculus
1	1.00	1	1	-0.03	-0.03	-0.03	-0.03	-0.032	t	140556	DWRHFMES	Mus musculus
1	1.00	1	1	0.01	0.01	0.01	0.01	0.008	t	117695	YITTIAGVM	Homo sapiens
1	1.00	1	1	0.01	0.01	0.01	0.01	0.008	t	117695	YITTIAGVM	Homo sapiens
1	1.00	1	1	0.03	0.034	0.021	0.027	0.028	t	116834	SPSQDPDRRDPYSPS	Homo sapiens
1	1.00	1	1	0	-0.004	0	-0.001	-0.001	t	49505	PSSKRFQPFQQFGRD	Oryctolagus cuniculus
1	1.00	1	1	-0.05	-0.05	-0.057	-0.05	-0.052	t	95583	LYNSQDHGWD	Homo sapiens
1	1.00	1	1	0	0	0	0	0.001	t	115256	FRELKDLKGY	Homo sapiens

1	1.00	1	1	0.01	0.013	0.01	0.01	0.01	t	21407	GNDYEDRYYREN	Mus musculus
1	1.00	1	1	0.04	0.04	0.04	0.04	0.04	t	112125	GFSLTTY	Homo sapiens
1	1.00	1	1	-0.01	-0.011	-0.002	-0.01	-0.01	t	25649	IDPY	Mus musculus
1	1.00	1	1	-0.05	-0.054	-0.059	-0.051	-0.05	t	74136	YHRVMYASSG	Oryctolagus cuniculus
1	1.00	1	1	0.01	0.012	0.014	0.01	0.008	t	137827	DVKSAIEG	Rattus norvegicus
1	1.00	1	1	0	0	0	0	-0.001	t	41014	MADNGTITVEELKQL	Homo sapiens
1	1.00	1	1	0.01	0.003	0.01	0.011	0.01	t	67514	VAAEEYVEVTRVGDFHYVT	Homo sapiens
1	1.00	1	1	0.05	0.047	0.045	0.05	0.05	t	63242	TDSSSDSQTITN	Oryctolagus cuniculus
1	1.00	1	1	0.01	0.01	0.01	0.01	0.01	t	137858	FTPGTEYT	Oryctolagus cuniculus
1	1.00	1	1	0.01	0.01	0.01	0.01	0.01	t	137858	FTPGTEYT	Homo sapiens
1	1.00	1	1	0.04	0.04	0.04	0.044	0.04	t	135944	KEQFEFALTAVAEEVC	Homo sapiens
1	1.00	1	1	-0.01	-0.01	-0.01	-0.01	-0.011	t	16755	FLLTRILTI	Mus musculus
1	1.00	1	1	0.03	0.028	0.022	0.033	0.032	t	42308	MPPYPTGGPPPV	Sus scrofa
1	1.00	1	1	0.03	0.028	0.022	0.033	0.032	t	42308	MPPYPTGGPPPV	Mus musculus
1	1.00	1	1	0	0	-0.007	0	0	t	158924	KSCTWPETHTLWGDGI	Mus musculus

1	1.00	1	1	0	0	-0.017	0	0.002	t	31539	KKFKAEIR DFFKEMRI QYAK	Homo sapiens
1	1.00	1	1	0.01	0.01	0.002	0.01	0.01	t	52789	QVSFTPDI EGYAELA WGIAS	Oryctola gus cuniculu s
1	1.00	1	1	-0.01	-0.01	-0.014	-0.01	-0.01	t	94445	CCHPACG KHF	Capra hircus
1	1.00	1	1	0.06	0.056	0.053	0.063	0.061	t	97023	VATRSQTD TSEKDC	Oryctola gus cuniculu s
1	1.00	1	1	0	0	0	0	-0.002	t	41581	MGGQSES VEFTKDTQ	Mus musculu s
1	1.00	1	1	-0.01	-0.01	-0.01	-0.006	-0.01	t	115598	ATGMEVG WYRPP	Homo sapiens
1	1.00	1	1	-0.03	-0.028	-0.037	-0.039	-0.032	t	115763	GPVRGPFP II	Homo sapiens
1	1.00	1	1	0.05	0.05	0.05	0.05	0.05	t	110067	VSTLIELPD EIP	Mus musculu s
1	1.00	1	1	0.03	0.03	0.03	0.03	0.03	t	127885	VTKWYSR	Homo sapiens
1	1.00	1	1	0.02	0.022	0.002	0.017	0.022	t	95834	SGPSGTSG PSGT	Homo sapiens
1	1.00	1	1	0.03	0.03	0.03	0.03	0.029	t	133535	CKPGEVQ QC	Mus musculu s
1	1.00	1	1	0.02	0.02	0.02	0.02	0.018	t	99836	QPFPQPQ	Mus musculu s
1	1.00	1	1	0.02	0.034	0.014	0.023	0.02	t	52749	QVMSRSA NEGYD	Homo sapiens
1	1.00	1	1	-0.03	-0.022	-0.03	-0.03	-0.032	t	156868	FHRHPLE	Gallus gallus
1	1.00	1	1	0.02	0.02	0.02	0.02	0.02	t	29751	KAALIEEG Q	Homo sapiens

1	1.00	1	1	0.02	0.02	0.012	0.02	0.02	t	84698	KSDYLGVNKIAE	Homo sapiens
1	1.00	1	1	0	0	0	0.006	0	t	106067	RGGSGGRGRGGSGG	Homo sapiens
1	1.00	1	1	0.02	0.028	0.02	0.02	0.018	t	156846	AKLITCSKTCSKWQ	Gallus gallus
1	1.00	1	1	0	-0.002	0	0.003	0	t	45817	NSGCFRHLDEREECKCLL	Mus musculus
1	1.00	1	1	0	-0.004	0	0.003	0	t	45817	NSGCFRHLDEREECKCLL	Oryctolagus cuniculus
1	1.00	1	1	-0.03	-0.03	-0.037	-0.03	-0.031	t	130543	LLKGKGDKAQIEKRI	Homo sapiens
1	1.00	1	1	0	0	-0.017	-0.004	0.001	t	49324	PSDKHIEQYLKKIQNSLS	Homo sapiens
1	1.00	1	1	0.01	0.012	0.01	0.011	0.01	t	102153	TQYTAYSPVPLL	Homo sapiens
1	1.00	1	1	-0.01	-0.005	-0.003	-0.01	-0.012	t	132864	LQEKLSPL	Mus musculus
1	1.00	1	1	0.02	0.02	0.024	0.02	0.019	t	58060	SGGSWV	Homo sapiens
1	1.00	1	1	-0.03	-0.033	-0.033	-0.03	-0.03	t	97444	LFKLGKLFQ	Oryctolagus cuniculus
1	1.00	1	1	0	-0.003	-0.006	0	0	t	18787	GATFQVEVPGSQHIDSQKKA	Oryctolagus cuniculus
1	1.00	1	1	0.02	0.02	0.018	0.019	0.02	t	69719	VLQELNVTV	Homo sapiens
1	1.00	1	1	0.01	0.012	-0.003	0.011	0.01	t	141734	RADESKTDPQTEA	Homo sapiens

1	1.00	1	1	0.02	0.02	0.013	0.02	0.02	t	120108	NFMESGEWVIKESRGW	Mus musculus
1	1.00	1	1	0	0.004	0	0.001	0	t	137820	DFTPGTEY	Homo sapiens
1	1.00	1	1	0.03	0.03	0.03	0.03	0.029	t	95119	MKKPGENIKPLSCKYSYPKH	Rattus norvegicus
1	1.00	1	1	0.03	0.032	0.029	0.03	0.03	t	56019	RTAPGPGE	Homo sapiens
1	1.00	1	1	0.03	0.032	0.021	0.031	0.029	t	60669	SRGGSQASSRSSSRSR	Homo sapiens
1	1.00	1	1	0.03	0.032	0.021	0.031	0.029	t	60669	SRGGSQASSRSSSRSR	Homo sapiens
1	1.00	1	1	0.01	0.007	0.014	0.01	0.012	t	136053	ALSPTMT	Oryctolagus cuniculus
1	1.00	1	1	0	0.002	0	-0.001	-0.003	t	136595	RHYQQYFQHF	Homo sapiens
1	1.00	1	1	0.04	0.04	0.04	0.04	0.04	t	99558	QEEENQGGKGPLLSI	Homo sapiens
1	1.00	1	1	-0.01	-0.01	-0.01	-0.01	-0.01	t	75409	YPSGAWYYVP	Homo sapiens
1	1.00	1	1	0.03	0.03	0.023	0.037	0.031	t	117015	KEQFLDGDGWTSRWIESK	Homo sapiens
1	1.00	1	1	0.04	0.042	0.042	0.039	0.037	t	96828	RLKEEEEDKK	Homo sapiens
1	1.00	1	1	0.03	0.03	0.028	0.03	0.03	t	66344	TSNQRGVGSTVVILD	Sus scrofa
1	1.00	1	1	0.05	0.048	0.041	0.049	0.05	t	60763	SRPLNDLVSQKTTQLSDITS	Mus musculus
1	1.00	1	1	-0.03	-0.032	-0.027	-0.031	-0.03	t	73739	YEPGRSLW	Mus musculus

1	1.00	1	1	0	-0.002	-0.008	-0.001	0	t	57277	SDSDNDG VCDNVDK	Mus musculus
1	1.00	1	1	0.05	0.052	0.059	0.051	0.052	t	35777	LEYTIV	Homo sapiens
1	1.00	1	1	-0.01	-0.01	-0.015	-0.01	-0.01	t	33761	KTLLSRFL WWLCYL	Homo sapiens
1	1.00	1	1	0.03	0.019	0.03	0.03	0.028	t	106494	GYENPTYK FFEQMQN	Oryctolagus cuniculus
1	1.00	1	1	-0.02	-0.022	-0.018	-0.017	-0.02	t	22881	GTVVAVG PGR	Mus musculus
1	1.00	1	1	0.02	0.023	0.012	0.02	0.022	t	95198	AAPTTS	Mus musculus
1	1.00	1	1	0.01	0.01	0.012	0.01	0.008	t	7230	CTTPAQG NSMFPSC	Mus musculus
1	1.00	1	1	0	0.003	-0.005	0	-0.001	t	106327	DDDKLEQI RRDYTSGA	Mus musculus
1	1.00	1	1	0.03	0.03	0.029	0.031	0.031	t	58683	SIQTKFAP	Sus scrofa
1	1.00	1	1	0	0.003	0.001	0	-0.002	t	43894	NFNGLTGT GVLTPSSK RF	Mus musculus
1	1.00	1	1	0	0	0	0	-0.002	t	70728	VRGLYF	Mus musculus
1	1.00	1	1	0.03	0.03	0.028	0.027	0.028	t	100571	TPATPTPT PKAA	Homo sapiens
1	1.00	1	1	0.03	0.03	0.03	0.03	0.03	t	123526	MVYEALD CI	Ovis aries
1	1.00	1	1	0	0	-0.009	0	-0.001	t	35810	LFDGRDN GAL	Mus musculus

1	1.00	1	1	0.02	0.02	0.02	0.02	0.02	t	67460	TYSFVSLLS PGAKQN	Mus musculu s
1	1.00	1	1	0	0	-0.011	0	0	t	95094	IGFRGLDG VHCTPTTD SGVI	Rattus norvegic us
1	1.00	1	1	-0.01	-0.014	-0.017	-0.007	-0.01	t	95874	SQWGWC GSTD	Oryctola gus cuniculu s
1	1.00	1	1	-0.01	-0.008	-0.01	-0.007	-0.011	t	96521	KVTKEKPT PP	Homo sapiens(human)
1	1.00	1	1	-0.02	-0.02	-0.012	-0.02	-0.019	t	115096	PPSLLFLP	Mus musculu s
1	1.00	1	1	0.01	0.014	0	0.01	0.01	t	88617	SGYDNDA LYASV	Homo sapiens
1	1.00	1	1	-0.05	-0.054	-0.052	-0.047	-0.052	t	95216	AEGLYSML VERD	Oryctola gus cuniculu s
1	1.00	1	1	-0.03	-0.03	-0.039	-0.03	-0.03	t	136003	TRKDINTV TTVA	Homo sapiens
1	1.00	1	1	0.01	0.01	0.014	0.01	0.008	t	151817	CLTSQAAS QRLE	Sus scrofa
1	1.00	1	1	-0.01	-0.012	-0.015	-0.011	-0.01	t	58417	SHSQWNK PSKPKTN MK	Mus musculu s
1	1.00	1	1	-0.01	-0.014	-0.015	-0.011	-0.01	t	58417	SHSQWNK PSKPKTN MK	Oryctola gus cuniculu s
1	1.00	1	1	0.01	0.012	0.002	0.011	0.01	t	14208	ESLTTTSTA LGKLQDVV	Homo sapiens
1	1.00	1	1	-0.01	-0.01	-0.01	-0.01	-0.01	t	5780	AYFYPELF RQ	Homo sapiens
1	1.00	1	1	0.03	0.03	0.039	0.03	0.028	t	12225	EGLTATTF EEDGVAA	Mus musculu s
1	1.00	1	1	-0.01	-0.003	-0.002	-0.007	-0.013	t	68061	VDRQYTIF G	Gallus gallus

1	1.00	1	1	0.01	0.012	0.007	0.011	0.012	t	61125	SSIVYEAD DVILHT	Homo sapiens
1	1.00	1	1	0.01	0.01	0.004	0.01	0.01	t	32415	KMVQVVY D	Homo sapiens
1	1.00	1	1	0.01	0.006	0.004	0.01	0.01	t	32415	KMVQVVY D	Oryctola gus cuniculu s
1	1.00	1	1	0.04	0.038	0.04	0.047	0.041	t	123683	QVPTTEVV GTTPGQA P	Homo sapiens
1	1.00	1	1	-0.01	-0.006	-0.007	-0.009	-0.01	t	7061	CSQHLPYI E	Homo sapiens
1	1.00	1	1	0.01	0.01	0.01	0.01	0.008	t	51908	QPQPFPS QQP	Mus musculu s
1	1.00	1	1	-0.01	-0.012	-0.015	-0.01	-0.009	t	4710	ASQKRPSQ RHGSKY	Rattus norvegic us
1	1.00	1	1	0.01	0.008	0.01	0.007	0.007	t	922	AEGEFDYA WDQTHQ DPAK	Mus musculu s
1	1.00	1	1	0.06	0.06	0.06	0.06	0.06	t	106428	FVEIYSP	Homo sapiens
1	1.00	1	1	0.06	0.06	0.06	0.06	0.06	t	106428	FVEIYSP	Homo sapiens
1	1.00	1	1	0	-0.002	-0.001	0.003	0.002	t	54140	RIEGGEIL	Anas platyrhy nchos
1	1.00	1	1	0.03	0.03	0.024	0.024	0.03	t	52180	QQYPQQQ	Homo sapiens
1	1.00	1	1	0.01	0.01	0.017	0.01	0.007	t	132794	DRLYAQYP VPTP	Mus musculu s
1	1.00	1	1	0.02	0.018	0.009	0.02	0.021	t	39485	LSLLRRQV NEVA	Oryctola gus cuniculu s
1	1.00	1	1	0.02	0.018	0.02	0.013	0.019	t	139618	TGQLVHRF	Mus musculu s

1	1.00	1	1	0.01	0.01	0.002	0.01	0.01	t	80885	DADIGNEYDQVV	Homo sapiens
1	1.00	1	1	-0.01	-0.01	-0.01	-0.008	-0.01	t	135973	PSKFTEPVKDVM	Homo sapiens
1	1.00	1	1	0.03	0.033	0.03	0.03	0.03	t	155333	PADS	Mus musculus
1	1.00	1	1	0	0.004	-0.012	0.001	0.002	t	23048	GVLSVDLSLTVELKI	Homo sapiens
1	1.00	1	1	0	0	0	0	-0.001	t	9754	DPRVRGLYF	Mus musculus
1	1.00	1	1	0	0	0	0	-0.001	t	9754	DPRVRGLYF	Mus musculus
1	1.00	1	1	0	0	0	0	-0.001	t	9754	DPRVRGLYF	Mus musculus
1	1.00	1	1	-0.01	-0.01	0	-0.01	-0.011	t	95161	TYGKFCIKP	Mus musculus
1	1.00	1	1	-0.01	-0.01	-0.008	-0.006	-0.01	t	118433	ISIGYRYPPICLGRA	Homo sapiens
1	1.00	1	1	0.02	0.02	0.022	0.027	0.019	t	104895	LVEALYLVCGERG	Mus musculus
1	1.00	1	1	0	-0.003	-0.012	0	0	t	30604	KESHDQFLQHTILFKGFFTD	Oryctolagus cuniculus
1	1.00	1	1	0	-0.003	-0.011	-0.001	-0.001	t	138175	GAPPNGAPPPPVDP	Oryctolagus cuniculus
1	1.00	1	1	0.07	0.07	0.07	0.07	0.07	t	79504	ADCTTPT	Oryctolagus cuniculus
1	1.00	1	1	0.03	0.03	0.03	0.03	0.03	t	123076	DFMLYETLD	Ovis aries
1	1.00	1	1	0.03	0.03	0.03	0.03	0.03	t	49533	PSTPGDNPLV	Homo sapiens

1	1.00	1	1	0.03	0.03	0.03	0.03	0.03	t	49533	PSTPGDNPLV	Homo sapiens
1	1.00	1	1	0.01	0.01	0.01	0.01	0.01	t	114757	CLLCAYSIEF	Homo sapiens
1	1.00	1	1	0	0	-0.005	0	0	t	10868	DYEDRYYRE	Mus musculus
1	1.00	1	1	0.05	0.053	0.041	0.05	0.052	t	120886	QDNEEFPEKD	Mus musculus
1	1.00	1	1	0.04	0.04	0.045	0.04	0.04	t	28250	IQWDGYARQ	Mus musculus
1	1.00	1	1	-0.01	-0.013	-0.016	-0.01	-0.01	t	159024	NKGAIIGLMVGGVVIA	Oryctolagus cuniculus
1	1.00	1	1	0	-0.004	0	0.003	0	t	105851	GTAPFEIHANRIVGFFSKII	Oryctolagus cuniculus
1	1.00	1	1	0.02	0.02	0.02	0.02	0.02	t	45538	NQELAYFYPE	Homo sapiens
1	1.00	1	1	-0.01	-0.011	-0.019	-0.01	-0.009	t	64752	TLDHVLDH	Anas platyrhynchos
1	1.00	1	1	-0.01	-0.008	0.002	-0.007	-0.01	t	8565	DHSAPL	Homo sapiens
1	1.00	1	1	0.02	0.023	0.02	0.02	0.022	t	7532	DAHHAAD	Mus musculus
1	1.00	1	1	0.04	0.042	0.049	0.047	0.041	t	112348	TEDLAVY	Homo sapiens
1	1.00	1	1	0.01	0.01	0.01	0.007	0.01	t	96755	PTAPTKPTYE	Homo sapiens(human)
1	1.00	1	1	0.03	0.028	0.021	0.029	0.03	t	80408	ANDVDNSNPVVQ	Rattus norvegicus
1	1.00	1	1	0.05	0.052	0.055	0.051	0.05	t	56272	RVEESI	Homo sapiens

1	1.00	1	1	-0.01	-0.01	-0.01	-0.01	-0.01	t	140823	AGKGVDAGKLGT	Oryctolagus cuniculus
1	1.00	1	1	-0.04	-0.04	-0.047	-0.049	-0.043	t	130922	KGMLPDPKNV	Homo sapiens
1	1.00	1	1	0.02	0.02	0.02	0.02	0.02	t	127718	KSTPQFT	Homo sapiens
1	1.00	1	1	0.02	0.02	0.02	0.02	0.02	t	117510	GRGRGMGRGNIFQKR	Homo sapiens
1	1.00	1	1	0	0	0	0	0	t	103378	LPLLEKVIGAGKPLL	Homo sapiens
1	1.00	1	1	-0.03	-0.028	-0.03	-0.033	-0.033	t	106340	DIMPCLHFVKGE	Homo sapiens
1	1.00	1	1	0.01	0.01	0.005	0.01	0.01	t	50442	QDAYNAAGGHNAVFNFPPNG	Homo sapiens
1	1.00	1	1	0	0.003	-0.011	0	0.001	t	79867	INNLIVELIRGT	Mus musculus
1	1.00	1	1	0	-0.003	0	0	-0.002	t	66841	TTTCFRDTDTGK	Oryctolagus cuniculus
1	1.00	1	1	0	-0.008	0	0	-0.002	t	21408	GNDYEDRYYRENMYRYPNQ	Mus musculus
1	1.00	1	1	-0.02	-0.018	-0.015	-0.013	-0.019	t	122179	RQLGRGS	Homo sapiens
1	1.00	1	1	0.03	0.027	0.023	0.03	0.031	t	133862	KLKKIIDDLDAKEN	Oryctolagus cuniculus
1	1.00	1	1	0.06	0.062	0.062	0.059	0.057	t	96823	RKEEEEAEDK	Homo sapiens
1	1.00	1	1	-0.05	-0.048	-0.062	-0.049	-0.05	t	20197	GHWMDATFNTKTA	Homo sapiens

1	1.00	1	1	0.03	0.03	0.03	0.03	0.03	t	79744	AASSPDA	Mus musculus
1	1.00	1	1	0.03	0.03	0.03	0.03	0.03	t	79744	AASSPDA	Mus musculus
1	1.00	1	1	0	-0.004	-0.007	0.003	0	t	96017	YHLYNSQDHG	Oryctolagus cuniculus
1	1.00	1	1	0.04	0.042	0.045	0.04	0.04	t	117376	TGALDVIRNFTLDMI	Homo sapiens
1	1.00	1	1	0.03	0.03	0.03	0.03	0.03	t	7673	DAVTE	Mus musculus
1	1.00	1	1	0.01	0.01	0.01	0.01	0.01	t	58148	SGNLLF	Homo sapiens
1	1.00	1	1	0.1	0.102	0.103	0.101	0.101	t	1157	AESEE	Homo sapiens
1	1.00	1	1	0.03	0.026	0.027	0.03	0.03	t	141053	LTPDTQYEFQVR	Mus musculus
1	1.00	1	1	-0.01	-0.012	-0.008	-0.009	-0.011	t	36372	LHEQYYYF	Mus musculus
1	1.00	1	1	0.08	0.082	0.07	0.077	0.08	t	56145	RTPEVDDEALE	Homo sapiens
1	1.00	1	1	0.01	0.01	0.01	0.011	0.011	t	51233	QKPLK	Bos taurus
1	1.00	1	1	0.02	0.017	0.015	0.02	0.021	t	703	ADLEKALEGAMC	Oryctolagus cuniculus
1	1.00	1	1	0	-0.002	-0.003	0.003	0	t	24425	HPAKSMDV	Anas platyrhynchos
1	1.00	1	1	-0.02	-0.022	-0.034	-0.021	-0.02	t	70489	VPSYSFIRAHDSEVQDLIA	Rattus norvegicus
1	1.00	1	1	-0.01	-0.01	-0.015	-0.007	-0.01	t	62334	SVSRARTRHLLLCL	Homo sapiens

1	1.00	1	1	-0.02	-0.018	-0.02	-0.013	-0.02	t	131766	YCQTWGS	Homo sapiens
1	1.00	1	1	0.05	0.05	0.05	0.05	0.05	t	103731	WISEYKEV EILEADD	Homo sapiens
1	1.00	1	1	0.05	0.05	0.05	0.05	0.05	t	97760	VTLPSGGA V	Oryctola gus cuniculu s
1	1.00	1	1	0.03	0.03	0.03	0.03	0.03	t	121842	SGGVLLPC YSFSWSC	Mus musculu s
1	1.00	1	1	0.03	0.03	0.03	0.03	0.03	t	121842	SGGVLLPC YSFSWSC	Mus musculu s
1	1.00	1	1	0	0	-0.005	0	0	t	67039	TVIYSKYG GT	Mus musculu s
1	1.00	1	1	-0.03	-0.033	-0.041	-0.03	-0.029	t	6540	CLAVLLGA	Oryctola gus cuniculu s
1	1.00	1	1	-0.01	-0.01	-0.023	-0.017	-0.01	t	141933	KIENAAM AIHLCFNN	Homo sapiens
1	1.00	1	1	-0.01	-0.008	-0.025	-0.009	-0.009	t	32862	KPRDTLPP PLNRPPP	Homo sapiens
1	1.00	1	1	0.05	0.047	0.048	0.05	0.049	t	21933	GQPQAQG DGANA	Oryctola gus cuniculu s
1	1.00	1	1	0	0	0	0	0	t	101983	PVYQQQP	Mus musculu s
1	1.00	1	1	0	0	0	0	0	t	101983	PVYQQQP	Mus musculu s
1	1.00	1	1	0	0	0	0	0	t	101983	PVYQQQP	Mus musculu s
1	1.00	1	1	0	0	0	0	0	t	2271	AKQYNICC KFKELLD	Mus musculu s
1	1.00	1	1	0	0.002	0.008	0.001	-0.002	t	29147	ITWIGM	Homo sapiens

1	1.00	1	1	0	0.002	0.008	0.001	-0.002	t	29147	ITWIGM	Homo sapiens
1	1.00	1	1	0	0.002	0.008	0.001	-0.002	t	29147	ITWIGM	Homo sapiens
1	1.00	1	1	0.03	0.032	0.031	0.028	0.028	t	106598	LEAYAFDIAA	Homo sapiens
1	1.00	1	1	-0.01	-0.006	-0.009	-0.013	-0.013	t	114732	AEVDCSRFPN	Homo sapiens
1	1.00	1	1	-0.01	-0.006	-0.009	-0.013	-0.013	t	114732	AEVDCSRFPN	Homo sapiens
1	1.00	1	1	0	0.002	0	0	-0.001	t	118579	RDMLLANKVPAAARA	Homo sapiens
1	1.00	1	1	0	0	0	0	0	t	135003	HWDPFSLSAYFP	Homo sapiens
1	1.00	1	1	0	0	0	0	0	t	135002	HWDPFSLSAYFP	Homo sapiens
1	1.00	1	1	0	0	0	0.004	0	t	144811	VSWLSKEWAK	Homo sapiens
1	1.00	1	1	0	0	0	0.004	0	t	144783	SHVVTMLDNF	Homo sapiens
1	1.00	1	1	0	0	0	0.004	0	t	144594	ARGISRTKYW	Homo sapiens
1	1.00	1	1	0	0	0	0.004	0	t	144697	IYRNLYWEGS	Homo sapiens
1	1.00	1	1	0	0	0	0.004	0	t	144614	DMMYGGMRGM	Homo sapiens
1	1.00	1	1	0	0	0	0.004	0	t	144639	FAQAYARGIS	Homo sapiens
1	1.00	1	1	0	0	0	0.004	0	t	144816	WSHNFTNMLG	Homo sapiens
1	1.00	1	1	0	0	0	0.004	0	t	144619	DYIWNTLNSG	Homo sapiens
1	1.00	1	1	0	0	0	0.004	0	t	144644	FRQQHGKTVV	Homo sapiens
1	1.00	1	1	0	0	0	0.004	0	t	144823	YWEGSGIGAI	Homo sapiens
1	1.00	1	1	0	0.003	0	0	-0.002	t	135501	SQWNKP	Mus musculus

1	1.00	1	1	0	0	0	0	0	t	144804	TVFVKPPP EKSW	Mus musculus
1	1.00	1	1	0.02	0.018	0.02	0.019	0.02	t	66397	TSRFASLF NLGPQQQ	Mus musculus
1	1.00	1	1	0.02	0.02	0.013	0.02	0.02	t	122719	CGVDFEIK AFATHSTD VEED	Rattus norvegicus
1	1.00	1	1	-0.01	-0.01	-0.01	-0.01	-0.01	t	15981	FGGETFTP DWMMEV	Mus musculus
1	1.00	1	1	0.03	0.03	0.03	0.03	0.029	t	107765	CESAQRPF GCC	Mus musculus
1	1.00	1	1	0.01	0.01	0.01	0.01	0.008	t	140610	TQWQGFS VA	Mus musculus
1	1.00	1	1	0.04	0.04	0.04	0.04	0.04	t	79413	TCPLPDPD SQYCE	Mus musculus
1	1.00	1	1	0.02	0.021	0.025	0.019	0.02	t	32680	KPDSMP	Equus caballus
1	1.00	1	1	-0.01	-0.01	-0.011	-0.006	-0.011	t	125029	EIERDTER DRFLSADE AV	Homo sapiens
1	1.00	1	1	-0.01	-0.01	-0.013	-0.012	-0.01	t	60914	SSATK	Homo sapiens
1	1.00	1	1	0.05	0.05	0.039	0.05	0.05	t	42646	MSRIAAG DLESSVDD PRSEE	Homo sapiens
1	1.00	1	1	-0.02	-0.02	-0.02	-0.02	-0.022	t	123010	AGAMIPP PPS	Oryctolagus cuniculus
1	1.00	1	1	-0.03	-0.032	-0.024	-0.029	-0.029	t	79042	PKSKPFA	Mus musculus
1	1.00	1	1	-0.01	-0.008	-0.01	-0.01	-0.01	t	71965	VWVTPVI G	Homo sapiens
1	1.00	1	1	-0.01	-0.014	-0.01	-0.009	-0.01	t	135606	YSSKKLC	Oryctolagus cuniculus

1	1.00	1	1	-0.03	-0.028	-0.03	-0.027	-0.03	t	96489	KPTPPVKPTA	Homo sapiens(human)
1	1.00	1	1	0.03	0.027	0.017	0.03	0.032	t	137939	NVLSADFT	Oryctolagus cuniculus
1	1.00	1	1	-0.02	-0.02	-0.02	-0.02	-0.02	t	156945	QLHTSI	Gallus gallus
1	1.00	1	1	0.07	0.07	0.07	0.07	0.07	t	153815	CYEQLNDSSEEEDEI	Oryctolagus cuniculus
1	1.00	1	1	0.01	0.014	0.005	0.01	0.01	t	57062	SAYTPAYYTKDTNNNLTL	Homo sapiens
1	1.00	1	1	0.03	0.032	0.039	0.037	0.03	t	112338	SVLYSSD	Homo sapiens
1	1.00	1	1	0	0.002	-0.005	0.001	0	t	142055	VKDTPRLIKLIKNS	Homo sapiens
1	1.00	1	1	0	0	0	0	-0.002	t	27998	IPQQPQQPFP	Mus musculus
1	1.00	1	1	0	0.003	0.008	0	0	t	95051	YSQFEKSY	Mus musculus
1	1.00	1	1	-0.04	-0.038	-0.04	-0.034	-0.04	t	138190	HNAKTKL	Homo sapiens
1	1.00	1	1	0.02	0.02	0.02	0.02	0.02	t	31404	KIQRDEDSYE	Homo sapiens
1	1.00	1	1	0	0.002	0.001	-0.002	-0.002	t	106529	ICDRSYTTRR	Homo sapiens
1	1.00	1	1	-0.02	-0.022	-0.018	-0.021	-0.02	t	55270	RPPRYLANQ	Mus musculus
1	1.00	1	1	-0.01	-0.012	-0.015	-0.007	-0.008	t	105090	AIIGLMVGGVVIA	Mus musculus

1	1.00	1	1	0.04	0.042	0.04	0.04	0.04	t	118011	LYKEKALS	Homo sapiens
1	1.00	1	1	-0.03	-0.03	-0.03	-0.03	-0.03	t	145827	IIPKVEGK	Mus musculu s
1	1.00	1	1	-0.01	-0.01	-0.01	-0.01	-0.012	t	35528	LEIVPNSAE	Homo sapiens
1	1.00	1	1	0	0	0	0	-0.002	t	63756	TFTNKLKE KHTDLGK	Mus musculu s
1	1.00	1	1	0.03	0.03	0.032	0.03	0.028	t	118577	RCPAKPEE EGMMINI	Homo sapiens
1	1.00	1	1	0.04	0.037	0.029	0.04	0.04	t	10123	DSGASSTA SSSH	Oryctola gus cuniculu s
1	1.00	1	1	0	0.002	-0.002	0.001	0	t	23404	GYLPISGE MEKVT	Homo sapiens
1	1.00	1	1	-0.01	-0.01	-0.017	-0.01	-0.011	t	106375	EDHAGTY GLGDRK	Mus musculu s
1	1.00	1	1	-0.03	-0.028	-0.03	-0.023	-0.029	t	131075	SQSYPEPA RGSVPMP	Homo sapiens
1	1.00	1	1	0.03	0.03	0.025	0.03	0.03	t	29812	KAGSTVYA VTAVTSIFS VG	Homo sapiens
1	1.00	1	1	0.01	0.01	0.032	0.01	0.006	t	144622	EDVGSNK	Mus musculu s
1	1.00	1	1	-0.01	-0.014	-0.013	-0.011	-0.01	t	39916	LTGISICNP GSSLC	Oryctola gus cuniculu s
1	1.00	1	1	0	0	-0.006	0	0	t	159172	TTDSGKLV TDWCCRS C	Mus musculu s
1	1.00	1	1	0.01	0.01	0.01	0.01	0.01	t	124720	SILPYGDS MDRIEK	Homo sapiens
1	1.00	1	1	0.04	0.043	0.035	0.04	0.04	t	49006	PQGQGKV T	Mus musculu s
1	1.00	1	1	0	0	0	0	0	t	133722	TSKSKYPL VV	Mus musculu s

1	1.00	1	1	0	0	-0.007	0	0	t	158999	LWGDGILE SDLIIPVT	Mus musculu s
1	1.00	1	1	0.01	0.008	0.01	0.003	0.008	t	128612	FDSHY	Mus musculu s
1	1.00	1	1	0	0	-0.009	-0.007	0	t	111013	RTGYIPLG TRPPT	Homo sapiens
1	1.00	1	1	-0.04	-0.04	-0.04	-0.04	-0.04	t	79249	HFQYRM	Mus musculu s
1	1.00	1	1	0.02	0.013	0.013	0.019	0.02	t	91203	DNLAPMS DGAVQPD G	Oryctola gus cuniculu s
1	1.00	1	1	-0.02	-0.018	-0.016	-0.013	-0.019	t	144697	IYRNLYWE GS	Homo sapiens
1	1.00	1	1	0	-0.003	-0.017	0	0.004	t	23252	GWGQPH GGGGWG QPH	Oryctola gus cuniculu s
1	1.00	1	1	0.07	0.07	0.07	0.07	0.07	t	52	AAANTSDS QKE	Homo sapiens
1	1.00	1	1	0.01	0.01	0.01	0.01	0.01	t	131983	NKQEEAW INPFVKQF	Homo sapiens
1	1.00	1	1	0.01	0.01	0.01	0.01	0.01	t	106995	WMTYPDS LIISA	Mus musculu s
1	1.00	1	1	0.01	0.01	-0.001	0.01	0.011	t	15402	FDIAFKFAS NTDWEGK DSKG	Oryctola gus cuniculu s
1	1.00	1	1	0.05	0.046	0.05	0.049	0.05	t	15775	FEYVDTYG DNAG	Oryctola gus cuniculu s
1	1.00	1	1	0.02	0.02	0.015	0.02	0.02	t	54345	RKFNDSVV QSDMKH WPFKVT	Homo sapiens
1	1.00	1	1	-0.01	-0.01	-0.012	-0.01	-0.01	t	45506	NPVIPFKD GIYFAATE K	Homo sapiens
1	1.00	1	1	0.04	0.04	0.038	0.04	0.04	t	6942	CRSACVYF TEYENSG	Mus musculu s

1	1.00	1	1	0.02	0.023	0.013	0.02	0.022	t	150858	ISSLYNLVKSS	Mus musculus
1	1.00	1	1	0.02	0.017	0.021	0.02	0.019	t	5771	AYFLYQQQGRLDKL	Oryctolagus cuniculus
1	1.00	1	1	-0.02	-0.022	-0.013	-0.019	-0.02	t	78994	HQLDPA	Mus musculus
1	1.00	1	1	0.01	0.008	0.003	0.013	0.012	t	106550	IWYINCFGCETHAML	Mus musculus
1	1.00	1	1	0	0	0	0	-0.001	t	119622	NVNHKPSN	Homo sapiens
1	1.00	1	1	0.02	0.02	0.008	0.02	0.023	t	150200	FGEHKQEKDLEYGAC	Mus musculus
1	1.00	1	1	0.03	0.03	0.03	0.03	0.032	t	78637	EKSLTSLSE	Oryctolagus cuniculus
1	1.00	1	1	0.01	0.01	0.004	0.01	0.01	t	22799	GTRIRFKDDTSFED	Homo sapiens
1	1.00	1	1	0	-0.002	0	0.001	0	t	149415	PQQLPQFEEI	Mus musculus
1	1.00	1	1	-0.02	-0.02	-0.02	-0.02	-0.019	t	6524	CKTC	Mus musculus
1	1.00	1	1	0	0	0.002	0	-0.002	t	34329	KWSDKPK	Mus musculus
1	1.00	1	1	-0.01	-0.01	-0.01	-0.01	-0.011	t	131581	PDSRAVTCGMASLSAEKV	Mus musculus
1	1.00	1	1	0.03	0.027	0.023	0.03	0.031	t	76485	YYALDVYAYDVT	Oryctolagus cuniculus
1	1.00	1	1	-0.01	-0.008	-0.01	-0.009	-0.01	t	42007	MLMRTDPFRELDRFA	Homo sapiens

1	1.00	1	1	-0.01	-0.008	-0.01	-0.009	-0.01	t	42007	MLMRTDP FRELDRFA	Homo sapiens
1	1.00	1	1	-0.04	-0.037	-0.039	-0.04	-0.04	t	1402	AFSVAAPV TVTR	Mus musculu s
1	1.00	1	1	0.03	0.03	0.023	0.03	0.031	t	131108	TGVEAAA ASAISVAR	Homo sapiens
1	1.00	1	1	-0.01	-0.01	-0.01	-0.01	-0.012	t	103830	CHAVKVG NPSC	Oryctola gus cuniculu s
1	1.00	1	1	0.01	0.01	0.01	0.01	0.012	t	127856	TDVRYKD	Mus musculu s
1	1.00	1	1	0	0	0	0	0	t	135755	SALVLQYL	Homo sapiens
1	1.00	1	1	0	0	0	0	0	t	123092	DLPTTMK MW	Ovis aries
1	1.00	1	1	-0.03	-0.032	-0.036	-0.027	-0.03	t	58942	SKSSRC	Mus musculu s
1	1.00	1	1	-0.01	-0.01	-0.01	-0.01	-0.01	t	16362	FKEKVLGL LQRATQ	Homo sapiens
1	1.00	1	1	0.01	0.01	-0.002	0.01	0.011	t	104520	IAKEQENK ETIGTLKKI LDE	Rattus norvegic us
1	1.00	1	1	-0.02	-0.024	-0.02	-0.017	-0.021	t	19737	GGGAGAG GAGAGGG GR	Oryctola gus cuniculu s
1	1.00	1	1	0.02	0.018	0.009	0.02	0.021	t	39485	LSLLRRQV NEVA	Oryctola gus cuniculu s
1	1.00	1	1	0.02	0.027	0.02	0.02	0.02	t	39091	LRGDLQVL AQ	Cavia porcellu s
1	1.00	1	1	0.04	0.04	0.042	0.04	0.039	t	119914	GSVVAINP ESDQPDLS	Mus musculu s

1	1.00	1	1	0.01	0.01	0.023	0.018	0.007	t	98824	IIAEKTKIPA	Homo sapiens
1	1.00	1	1	0.03	0.03	0.03	0.03	0.032	t	43746	NESKYSNT	Mus musculus
1	1.00	1	1	0.01	0.013	-0.001	0.01	0.013	t	111309	GDTPTLHEYMLDLQ	Mus musculus
1	1.00	1	1	0.01	0.01	0.01	0.01	0.01	t	72088	VYLLPRRG	Homo sapiens
1	1.00	1	1	0.04	0.04	0.045	0.046	0.043	t	115671	AAMEL	Homo sapiens
1	1.00	1	1	0.02	0.018	0.013	0.02	0.019	t	123016	AIYENEIHALWADLP	Trichosurus vulpecula
1	1.00	1	1	-0.01	-0.012	-0.03	-0.017	-0.01	t	128115	SSHRGNICIGATNYVADN	Mus musculus
1	1.00	1	1	-0.01	-0.008	-0.019	-0.01	-0.009	t	51474	QLPNVGITQGIV	Ovis aries
1	1.00	1	1	0.01	0.012	0.007	0.011	0.008	t	35244	LDNPLENPSSLFDLVARIKN	Homo sapiens
1	1.00	1	1	0	-0.002	0.003	0.003	0	t	52094	QQPP	Mus musculus
1	1.00	1	1	-0.04	-0.038	-0.034	-0.033	-0.038	t	123643	PYMPPPGM	Homo sapiens
1	1.00	1	1	0.01	0.013	0.008	0.01	0.01	t	95132	PYVTYG	Mus musculus
1	1.00	1	1	-0.01	-0.008	-0.01	-0.009	-0.01	t	117018	KIVMFWHEPVYA	Homo sapiens
1	1.00	1	1	0.03	0.032	0.015	0.027	0.03	t	60424	SQEIVQNESGTEEILE	Homo sapiens

1	1.00	1	1	0	0.004	0.008	0.003	-0.002	t	16114	FHISCLTFGRET	Homo sapiens
1	1.00	1	1	0	0.001	0.008	0.003	-0.002	t	16114	FHISCLTFGRET	Homo sapiens
1	1.00	1	1	0	0	0	0	-0.002	t	133722	TSKSKYPLVV	Mus musculus
1	1.00	1	1	0.02	0.022	0.018	0.021	0.02	t	38330	LNRSGGNYTKTDDEA	Homo sapiens
1	1.00	1	1	0.03	0.026	0.028	0.027	0.03	t	120503	ENKIFADDIDIS	Oryctolagus cuniculus
1	1.00	1	1	0.03	0.028	0.028	0.027	0.03	t	120503	ENKIFADDIDIS	Rattus norvegicus
1	1.00	1	1	-0.01	-0.006	-0.01	-0.009	-0.01	t	60662	SRGAPG	Homo sapiens
1	1.00	1	1	-0.01	-0.02	-0.01	-0.009	-0.01	t	60662	SRGAPG	Homo sapiens
1	1.00	1	1	0.04	0.04	0.035	0.037	0.039	t	115728	EPFTESQSLT	Homo sapiens
1	1.00	1	1	0.02	0.028	0.02	0.02	0.018	t	136647	SNNADYKQSLLL	Gallus gallus
1	1.00	1	1	0.01	0.01	0.01	0.01	0.01	t	123125	EFPKLKEFK	Ovis aries
1	1.00	1	1	0.06	0.058	0.048	0.053	0.059	t	108170	TTANPSCPEGT	Rattus norvegicus
1	1.00	1	1	0.03	0.028	0.022	0.029	0.03	t	17993	FTKDTQTGMSGQTTP	Mus musculus
1	1.00	1	1	0	0	0.002	0.001	0.001	t	65678	TPPAYR	Mus musculus
1	1.00	1	1	0.04	0.04	0.043	0.04	0.04	t	125250	SDEFNPCEDIMGYK	Mus musculus
1	1.00	1	1	0.01	0.012	0.003	0.011	0.01	t	56796	SAGGQLFYSRPVVSANGE	Homo sapiens

1	1.00	1	1	0.03	0.032	0.028	0.033	0.029	t	48870	PPPPSSPTHDPPDSDPQIPP	Homo sapiens
1	1.00	1	1	0.02	0.02	0.022	0.02	0.019	t	5962	CAALASLFLSLGPQQK	Mus musculus
1	1.00	1	1	-0.04	-0.038	-0.047	-0.039	-0.04	t	97117	YIQMKRIAVG	Homo sapiens(human)
1	1.00	1	1	0.05	0.046	0.049	0.05	0.05	t	123849	WQDE	Mus musculus
1	1.00	1	1	-0.03	-0.03	-0.03	-0.03	-0.03	t	22127	GRLHLNDRV	Homo sapiens
1	1.00	1	1	0.02	0.018	0.02	0.019	0.018	t	150978	EKNVTVTHSVNLLED	Mus musculus
1	1.00	1	1	0.04	0.043	0.045	0.04	0.04	t	138153	DGDYGA	Mus musculus
1	1.00	1	1	0.03	0.035	0.038	0.027	0.028	t	157280	FSGFDAEFLADA	Homo sapiens
1	1.00	1	1	0	0	0	0	-0.002	t	30262	KDTNNN	Mus musculus
1	1.00	1	1	-0.03	-0.028	-0.03	-0.029	-0.03	t	48005	PIVATNWQK	Homo sapiens
1	1.00	1	1	-0.03	-0.026	-0.03	-0.029	-0.03	t	48005	PIVATNWQK	Homo sapiens
1	1.00	1	1	-0.03	-0.028	-0.03	-0.023	-0.029	t	131075	SQSYPEPARGSVPMP	Homo sapiens
1	1.00	1	1	-0.02	-0.017	-0.02	-0.02	-0.02	t	131346	AIPDRFS	Mus musculus
1	1.00	1	1	0	0	-0.004	0	0	t	6139	CDSSDRPRVYTIT	Mus musculus

1	1.00	1	1	0	0.008	-0.004	0	0	t	6139	CDSSDRPR VYTIT	Gallus gallus
1	1.00	1	1	0.01	0.01	0.01	0.01	0.01	t	99332	IGTTKQ	Equus caballus
1	1.00	1	1	0	0.003	0.003	-0.001	-0.001	t	157189	EFRHDSGY	Mus musculu s
1	1.00	1	1	0	-0.002	0	0.003	0	t	69271	VKMAETC PIFYDVF	Mus musculu s
1	1.00	1	1	0	-0.008	0	0.003	0	t	20064	GGVATY	Macaca
1	1.00	1	1	0	-0.002	0	0.003	0	t	31580	KKIQDCYV ENGLIS	Mus musculu s
1	1.00	1	1	0	-0.002	0	0.003	0	t	12408	EICPAVKR DVDLFLTG	Mus musculu s
1	1.00	1	1	0.01	0.013	0.001	0.01	0.012	t	79963	PGSSPME VMLRA	Mus musculu s
1	1.00	1	1	-0.03	-0.03	-0.035	-0.03	-0.031	t	153366	CFRKHPEA TYSR	Mus musculu s
1	1.00	1	1	0.01	0.01	0.01	0.01	0.009	t	103838	DAEFRHDS GYEV	Mus musculu s
1	1.00	1	1	0	0.004	0	0.001	-0.001	t	23068	GVNLEDP ASRDL	Homo sapiens
1	1.00	1	1	0.04	0.039	0.034	0.039	0.04	t	4228	ARQQTVS ALEDKLA	Myodes glareolu s
1	1.00	1	1	0.03	0.028	0.018	0.039	0.037	t	107242	SQTTANPS CPEGT	Rattus norvegic us
1	1.00	1	1	0.03	0.028	0.018	0.039	0.037	t	107242	SQTTANPS CPEGT	Rattus norvegic us
1	1.00	1	1	0.03	0.032	0.022	0.027	0.028	t	114283	CQIESWD HNDQQFQ C	Homo sapiens
1	1.00	1	1	0.03	0.028	0.026	0.023	0.028	t	14009	ERKIKV	Mus musculu s

1	1.00	1	1	0	0	-0.002	0	-0.003	t	19868	GGGWGQPHGGGWGQPH	Mus musculus
1	1.00	1	1	-0.01	-0.01	-0.01	-0.01	-0.012	t	144663	GREGYPADGGGCKITCFL	Oryctolagus cuniculus
1	1.00	1	1	-0.01	-0.013	-0.01	-0.01	-0.012	t	144663	GREGYPADGGGCKITCFL	Oryctolagus cuniculus
1	1.00	1	1	0	0	0.002	0.002	0	t	50477	QDIQPAVQASWPKV	Homo sapiens
1	1.00	1	1	-0.02	-0.02	-0.018	-0.018	-0.02	t	55625	RRKAMLQDMA	Homo sapiens
1	1.00	1	1	-0.01	-0.01	-0.01	-0.01	-0.01	t	2378	ALAELATKTFGSSG	Homo sapiens
1	1.00	1	1	-0.03	-0.032	-0.03	-0.027	-0.03	t	110468	YYVLHLCLAATKYPLLKLLG	Homo sapiens
1	1.00	1	1	-0.02	-0.02	-0.025	-0.02	-0.019	t	14447	ETKRKDGD	Mus musculus
1	1.00	1	1	-0.02	-0.017	-0.025	-0.02	-0.019	t	14447	ETKRKDGD	Equus caballus
1	1.00	1	1	0.01	0.01	0.01	0.01	0.01	t	25622	IDLTNKNAA	Homo sapiens
1	1.00	1	1	-0.01	-0.01	-0.015	-0.01	-0.01	t	95201	AASAYCSTWD	Homo sapiens
1	1.00	1	1	0.02	0.02	0.02	0.024	0.02	t	118019	MGRGAPPPGMMG	Homo sapiens
1	1.00	1	1	-0.02	-0.024	-0.026	-0.017	-0.018	t	29803	KAFRELADAL	Oryctolagus cuniculus
1	1.00	1	1	0.01	0.01	0.01	0.01	0.01	t	106596	LDLKRTTTTHTS	Mus musculus

1	1.00	1	1	0.01	0.01	0.01	0.01	0.01	t	25623	IDLTNKNAAK	Homo sapiens
1	1.00	1	1	-0.01	-0.01	-0.01	-0.01	-0.01	t	105581	VT	Oryctolagus cuniculus
1	1.00	1	1	0	0	0	0	-0.002	t	103496	PRGYPGQV	Mus musculus
1	1.00	1	1	0	0	0	0	-0.002	t	75950	YTASARGDLAHLTTT	Mus musculus
1	1.00	1	1	0	0	0	0	-0.002	t	68320	VEQHHRRTD	Mus musculus
1	1.00	1	1	0	0	0	0	-0.002	t	75949	YTASARGDLAHL	Mus musculus
1	1.00	1	1	0	0	0	0	-0.002	t	64031	THGQWNKPSK	Mus musculus
1	1.00	1	1	0	0	0	0	-0.002	t	75950	YTASARGDLAHLTTT	Mus musculus
1	1.00	1	1	0	0	0	0	-0.002	t	41290	MDWNMHAA	Mus musculus
1	1.00	1	1	0.03	0.032	0.025	0.033	0.032	t	141604	APPDDGPP	Homo sapiens
1	1.00	1	1	0.02	0.017	0.017	0.02	0.02	t	97760	VTLPSGGAV	Oryctolagus cuniculus
1	1.00	1	1	-0.04	-0.04	-0.045	-0.04	-0.04	t	16305	FISP	Homo sapiens
1	1.00	1	1	0.01	0.01	0.01	0.013	0.01	t	11973	EFDEMEECSQHLPYIEQG	Homo sapiens
1	1.00	1	1	0.04	0.04	0.04	0.044	0.041	t	117700	YSPTSPS	Homo sapiens

1	1.00	1	1	0	-0.004	-0.007	-0.007	-0.001	t	135468	GVGLAP	Oryctolagus cuniculus
1	1.00	1	1	-0.03	-0.032	-0.03	-0.037	-0.033	t	139618	TGQLVHRF	Mus musculus
1	1.00	1	1	-0.01	-0.004	0.005	-0.01	-0.017	t	31063	KGSSGYCAWPACYCY	Equus caballus
1	1.00	1	1	0.04	0.042	0.032	0.037	0.038	t	114283	CQIESWDHNDQQFQC	Homo sapiens
1	1.00	1	1	-0.02	-0.017	-0.026	-0.02	-0.021	t	50814	QFQGQQQPFP	Mus musculus
1	1.00	1	1	-0.01	-0.01	-0.01	-0.01	-0.01	t	51710	QNITDL	Oryctolagus cuniculus
1	1.00	1	1	-0.01	-0.01	0.002	-0.007	-0.011	t	23160	GVSWTM	Homo sapiens
1	1.00	1	1	0.01	0.01	0.01	0.013	0.007	t	146986	GNGPGYALGGGSR	Homo sapiens
1	1.00	1	1	0	0	0	0	0	t	130880	HIDGPSLLIEN	Mus musculus
1	1.00	1	1	0	0	0	0.004	0	t	122445	KQYSIEAALRCRRISKEEYH	Homo sapiens
1	1.00	1	1	0.01	0.007	0.01	0.01	0.009	t	106035	QFAGKDLE	Oryctolagus cuniculus
1	1.00	1	1	0.02	0.02	0.02	0.02	0.02	t	23190	GVTYEIDLTNKNA	Homo sapiens
1	1.00	1	1	-0.05	-0.053	-0.056	-0.05	-0.05	t	105896	KFTQFAGK	Oryctolagus cuniculus
1	1.00	1	1	-0.05	-0.053	-0.056	-0.05	-0.05	t	105896	KFTQFAGK	Oryctolagus cuniculus

1	1.00	1	1	0.03	0.026	0.029	0.03	0.03	t	123838	VWQDE	Mus musculus
1	1.00	1	1	0	0.003	0.009	0	0	t	139227	RGDLQVLTPK	Sus scrofa
1	1.00	1	1	0	0.002	0	0.007	0.002	t	11571	EEEDDDMGFGLFD	Homo sapiens
1	1.00	1	1	-0.05	-0.05	-0.055	-0.05	-0.05	t	67569	VAEKVRNLPA	Homo sapiens
1	1.00	1	1	0.01	0.01	0.01	0.01	0.011	t	79284	LDAVPL	Mus musculus
1	1.00	1	1	0	-0.002	0	-0.001	-0.002	t	69833	VLVLNPSVAATLGFGAYMSK	Mus musculus
1	1.00	1	1	0	-0.002	0	-0.001	-0.002	t	13735	EPQVYTLPPSR	Mus musculus
1	1.00	1	1	0	-0.002	0	-0.001	-0.001	t	4737	ASREAK	Mus musculus
1	1.00	1	1	-0.05	-0.05	-0.05	-0.05	-0.053	t	134333	CIPIPSSWAFARFLW	Homo sapiens
1	1.00	1	1	0.02	0.02	0.022	0.02	0.02	t	110610	GPKGAPGERGPSGLA	Mus musculus
1	1.00	1	1	-0.02	-0.022	-0.02	-0.013	-0.02	t	123636	PVGPAPGMRP	Homo sapiens
1	1.00	1	1	-0.01	-0.01	-0.016	-0.01	-0.01	t	28691	ISQHQCVK	Mus musculus
1	1.00	1	1	0.01	0.005	0.005	0.011	0.01	t	100101	HFYDGFAKVPLKTD	Oryctolagus cuniculus
1	1.00	1	1	0.01	0.01	0.005	0.011	0.01	t	100101	HFYDGFAKVPLKTD	Homo sapiens
1	1.00	1	1	0.01	0.012	0.01	0.011	0.01	t	12951	ELAPEDPEDS	Homo sapiens

1	1.00	1	1	0	-0.007	0	0	0	t	3381	ANPNPYTS RRSV	Oryctola gus cuniculu s
1	1.00	1	1	0	-0.001	-0.004	0	0	t	30596	KERSSLRY GN	Mus musculu s
1	1.00	1	1	0.01	0.013	0.01	0.01	0.008	t	140825	AGKLGTVS YGEEKPAV LGHD	Mus musculu s
1	1.00	1	1	0.01	0.01	0.01	0.01	0.008	t	49085	PQQPFPQ QPE	Mus musculu s
1	1.00	1	1	0.04	0.04	0.04	0.04	0.04	t	141660	GMEKAKE ELLETLQIV	Rattus norvegic us
1	1.00	1	1	0.04	0.042	0.04	0.04	0.04	t	141660	GMEKAKE ELLETLQIV	Rattus norvegic us
1	1.00	1	1	0.04	0.038	0.04	0.04	0.04	t	141660	GMEKAKE ELLETLQIV	Oryctola gus cuniculu s
1	1.00	1	1	0.04	0.04	0.04	0.04	0.04	t	141660	GMEKAKE ELLETLQIV	Oryctola gus cuniculu s
1	1.00	1	1	-0.02	-0.02	-0.013	-0.016	-0.024	t	138249	TFRVVSV	Homo sapiens
1	1.00	1	1	0.02	0.02	0.013	0.02	0.02	t	69210	VKELPTEV QKLKEK	Homo sapiens
1	1.00	1	1	0.03	0.026	0.025	0.029	0.03	t	96	AADQLPN V	Oryctola gus cuniculu s
1	1.00	1	1	-0.01	-0.006	-0.02	-0.009	-0.01	t	8594	DICLGTDIS VKQGDV	Homo sapiens
1	1.00	1	1	-0.01	-0.008	-0.017	-0.013	-0.013	t	106571	KLVSEVPK HCGI	Homo sapiens
1	1.00	1	1	0.05	0.05	0.041	0.05	0.051	t	33206	KRPNDKA G	Mus musculu s

1	1.00	1	1	0	0.003	-0.007	0	0	t	41421	MEQFIRAFIEIDKDNNELID	Mus musculus
1	1.00	1	1	-0.04	-0.04	-0.052	-0.036	-0.038	t	96260	EPNANLSLTTDGKFLKASA	Homo sapiens(human)
1	1.00	1	1	0.03	0.03	0.03	0.03	0.03	t	138027	SADFTPGT	Homo sapiens
1	1.00	1	1	0	0	-0.006	0	0	t	158923	KSCTWPETHTLWGDAV	Mus musculus
1	1.00	1	1	0	0.003	0	-0.001	-0.001	t	79111	VNKGYTGY	Mus musculus
1	1.00	1	1	0.02	0.02	0.009	0.02	0.02	t	75152	YNGEEYLILSARDVL	Homo sapiens
1	1.00	1	1	0.01	0.01	0.01	0.01	0.01	t	104161	NVVLKTITESATSPPT	Oryctolagus cuniculus
1	1.00	1	1	-0.01	-0.01	-0.018	-0.01	-0.008	t	133635	LSRSTKEYNKLK	Mus musculus
1	1.00	1	1	0.03	0.03	0.029	0.03	0.03	t	10550	DVCSDSDNDGVCDNV	Mus musculus
1	1.00	1	1	0	0	0.002	0.003	0	t	67270	TWKKPDYE	Homo sapiens
1	1.00	1	1	0	0	0	0	-0.002	t	21408	GNDYEDRYYRENMYRYPNQ	Mus musculus
1	1.00	1	1	0.02	0.02	0.022	0.021	0.02	t	71446	VTPIDT	Homo sapiens
1	1.00	1	1	0.02	0.02	0.029	0.02	0.019	t	6129	CDPTMDY	Mus musculus
1	1.00	1	1	0.01	0.01	0.01	0.01	0.01	t	124275	HPLAPLNF	Homo sapiens
1	1.00	1	1	0.01	0.01	0.009	0.01	0.009	t	72503	WGQGG	Mus musculus
1	1.00	1	1	0.03	0.03	0.03	0.029	0.033	t	153263	HAGTFLQVA	Mus musculus

1	1.00	1	1	-0.03	-0.032	-0.037	-0.027	-0.03	t	53876	RGGIPSLLKKGKDIKV	Mus musculus
1	1.00	1	1	0.07	0.07	0.07	0.07	0.07	t	123161	EYLGEEYEE	Ovis aries
1	1.00	1	1	0.05	0.05	0.05	0.05	0.05	t	79282	LCEETPPNETTPCI	Mus musculus
1	1.00	1	1	0.03	0.03	0.03	0.03	0.03	t	103929	GKLPIPY	Homo sapiens
1	1.00	1	1	-0.01	-0.01	-0.01	-0.01	-0.01	t	101873	LNYKQEGDK	Homo sapiens
1	1.00	1	1	-0.01	-0.012	-0.022	-0.007	-0.01	t	8381	DGERVPKNNRLSKKYSELSE	Mus musculus
1	1.00	1	1	-0.03	-0.032	-0.028	-0.031	-0.03	t	16123	FHLPYNHNWFAL	Mus musculus
1	1.00	1	1	0	0	0	0	0	t	8636	DIETPRAGKKESTV	Ovis aries
1	1.00	1	1	0	0	0	0	0	t	98981	REREEDWRQP	Homo sapiens
1	1.00	1	1	0.02	0.02	0.011	0.018	0.02	t	135980	QSRRRGDMSTLN	Homo sapiens
1	1.00	1	1	-0.02	-0.022	-0.015	-0.017	-0.02	t	141693	KTWG	Mus musculus
1	1.00	1	1	-0.02	-0.018	-0.02	-0.023	-0.023	t	78143	EKIEKTEEPAP	Homo sapiens
1	1.00	1	1	-0.02	-0.02	-0.02	-0.02	-0.023	t	73080	WSLDPHR	Mus musculus
1	1.00	1	1	0	0	0	0	-0.002	t	21254	GMDGTQLDRW	Mus musculus

1	1.00	1	1	0	0	0	0	-0.002	t	21254	GMDGTQLDRW	Mus musculus
1	1.00	1	1	-0.05	-0.048	-0.055	-0.049	-0.049	t	50494	QDMRNTIMASKTVG	Homo sapiens
1	1.00	1	1	-0.01	-0.015	-0.019	-0.012	-0.01	t	51474	QLPNVGITQGIV	Bos taurus
1	1.00	1	1	-0.01	-0.003	-0.01	-0.011	-0.012	t	67187	TVTTTTKGENFTETDIKI	Gallus gallus
1	1.00	1	1	0.04	0.032	0.045	0.04	0.039	t	125063	GDRAVYENELVA	Mus musculus
1	1.00	1	1	-0.01	-0.01	-0.01	-0.01	-0.012	t	133668	QEGPEYWDRNT	Mus musculus
1	1.00	1	1	0.05	0.051	0.043	0.05	0.051	t	45571	NQITSALSTQ	Mus musculus
1	1.00	1	1	-0.02	-0.02	-0.023	-0.019	-0.018	t	28351	IRNW	Mus musculus
1	1.00	1	1	-0.02	-0.023	-0.023	-0.019	-0.018	t	28351	IRNW	Mus musculus
1	1.00	1	1	-0.02	-0.02	-0.023	-0.019	-0.018	t	28351	IRNW	Mus musculus
1	1.00	1	1	0.01	0.01	0.008	0.01	0.009	t	79928	MDRPTSMKALDH	Mus musculus
1	1.00	1	1	-0.02	-0.02	-0.02	-0.02	-0.022	t	106642	MHLNTPP	Homo sapiens
1	1.00	1	1	0.05	0.046	0.044	0.047	0.052	t	21557	GNYTKTDDEANQLLS	Oryctolagus cuniculus
1	1.00	1	1	-0.01	-0.01	-0.01	-0.01	-0.012	t	28693	ISQQPFPLQP	Mus musculus
1	1.00	1	1	0.01	0.01	0.006	0.01	0.012	t	6093	CDCDRTAA	Mus musculus

1	1.00	1	1	0.01	0.01	0.006	0.01	0.012	t	6093	CDCDRTAA	Mus musculus
1	1.00	1	1	-0.03	-0.032	-0.037	-0.031	-0.03	t	4710	ASQKRPSQRHGSKY	Rattus norvegicus
1	1.00	1	1	0.06	0.06	0.06	0.06	0.06	t	146228	EYSDSGIEV	Oryctolagus cuniculus
1	1.00	1	1	-0.03	-0.024	-0.039	-0.031	-0.03	t	11802	EENKKPAE	Ovis aries
1	1.00	1	1	0.02	0.02	0.02	0.02	0.02	t	156946	QLLYGS	Gallus gallus
1	1.00	1	1	0	0	0	0	0	t	132072	SPFVPYDFMNSSTSP	Homo sapiens
1	1.00	1	1	0.01	0.01	0.006	0.013	0.01	t	67069	TVLGCALPPTPQTP	Homo sapiens
1	1.00	1	1	-0.03	-0.03	-0.03	-0.03	-0.03	t	119844	ERTVLVSMNY	Mus musculus
1	1.00	1	1	-0.03	-0.03	-0.03	-0.03	-0.03	t	119844	ERTVLVSMNY	Mus musculus
1	1.00	1	1	0.02	0.018	0.015	0.023	0.02	t	23694	HEIAWFTERSDKSYEHQT	Mus musculus
1	1.00	1	1	0.02	0.018	0.015	0.023	0.02	t	23694	HEIAWFTERSDKSYEHQT	Mus musculus
1	1.00	1	1	0.06	0.056	0.053	0.053	0.059	t	125330	YVDTYGDNAG	Oryctolagus cuniculus
1	1.00	1	1	0	0.004	-0.005	-0.003	-0.003	t	141619	DLNLIGFGINAKNNQ	Homo sapiens

1	1.00	1	1	0	0.002	0.007	0.007	-0.002	t	113193	TGEIKALA GCDFLTI	Homo sapiens
1	1.00	1	1	0.03	0.033	0.03	0.03	0.03	t	130871	GSESGIFT NTK	Mus musculu s
1	1.00	1	1	0.03	0.03	0.03	0.03	0.03	t	130871	GSESGIFT NTK	Mus musculu s
1	1.00	1	1	-0.01	-0.008	-0.01	-0.009	-0.012	t	146901	ETHGGED VAVFAR	Homo sapiens
1	1.00	1	1	0.06	0.062	0.06	0.067	0.062	t	132210	DGPPGEL GPD	Homo sapiens
1	1.00	1	1	0.02	0.017	0.008	0.02	0.021	t	5728	AYANSTQE ETF	Oryctola gus cuniculu s
1	1.00	1	1	-0.01	-0.013	-0.015	-0.01	-0.01	t	58417	SHSQWNK PSKPKTN MK	Oryctola gus cuniculu s
1	1.00	1	1	0.02	0.024	0.018	0.021	0.02	t	14487	ETMTPSGL VIPENAK	Homo sapiens
1	1.00	1	1	0.01	0.006	-0.003	0.007	0.01	t	5207	ATVSSQSF RR	Oryctola gus cuniculu s
1	1.00	1	1	0.01	0.008	0.001	0.003	0.01	t	120419	AVKYTGYT GG	Mus musculu s
1	1.00	1	1	0.06	0.06	0.06	0.06	0.06	t	118902	LVFEAPNQ EKVSDYE	Oryctola gus cuniculu s
1	1.00	1	1	-0.03	-0.03	-0.011	-0.03	-0.041	t	26849	IKMVSSLK TKIT	Mus musculu s
1	1.00	1	1	-0.03	-0.03	-0.011	-0.03	-0.041	t	26849	IKMVSSLK TKIT	Mus musculu s
1	1.00	1	1	0.04	0.038	0.033	0.043	0.041	t	107127	GEPGDDA PSGAEGPP GPQG	Mus musculu s

1	1.00	1	1	0.01	0.014	0.006	0.013	0.01	t	71839	VVSTLPQA VMGSSY	Homo sapiens
1	1.00	1	1	0.01	0.012	0.006	0.013	0.01	t	71839	VVSTLPQA VMGSSY	Homo sapiens
1	1.00	1	1	0	0.01	0	-0.001	0	t	55070	RNTNRRP QDV	Pan troglody tes
1	1.00	1	1	0.01	0.01	0.01	0.01	0.008	t	78696	KEEGEDDT ARQEIRKA WVK	Oryctola gus cuniculu s
1	1.00	1	1	0.01	0.007	0.01	0.01	0.008	t	78696	KEEGEDDT ARQEIRKA WVK	Oryctola gus cuniculu s
1	1.00	1	1	0.03	0.03	0.037	0.03	0.03	t	71856	VVTPDKEI L	Homo sapiens
1	1.00	1	1	-0.01	-0.01	-0.01	-0.007	-0.01	t	97064	VNVPKVTK EK	Homo sapiens(human)
1	1.00	1	1	0.02	0.023	0.02	0.02	0.018	t	140905	TPAAKVLV EGNTDER GTPEY	Mus musculu s
1	1.00	1	1	-0.01	-0.01	-0.01	-0.01	-0.01	t	158609	SFGTALW	Homo sapiens
1	1.00	1	1	0.01	0.01	0.01	0.01	0.012	t	131865	EPLEVFISA	Homo sapiens
1	1.00	1	1	-0.01	-0.015	-0.018	-0.011	-0.01	t	13678	EPKIGLPG PPSAPVP	Bos taurus
1	1.00	1	1	0	0	0	0.004	0	t	142204	KLEEQNKI SEASRKGL RRDL	Homo sapiens
1	1.00	1	1	0	0	0	0.004	0	t	142288	SEASRKGL RRDLDASR EAKK	Homo sapiens
1	1.00	1	1	0	0	0	0.004	0	t	138910	SEGGDEYV TKGEVVET D	Homo sapiens

1	1.00	1	1	0	0	0	0.004	0	t	142116	ASRKGLRRDLDASREAKKQV	Homo sapiens
1	1.00	1	1	0	0	0	0.004	0	t	142167	GLRRDLDASREAKKQVEKAL	Homo sapiens
1	1.00	1	1	0	0	0	0.004	0	t	142166	GLRRDLDASREAKKQLEAEQ	Homo sapiens
1	1.00	1	1	0	0	0	0.004	0	t	142217	LDASREAKKQLEAEQQKLEE	Homo sapiens
1	1.00	1	1	0	0	0	0.004	0	t	142282	RRDLDASREAKKQLEAEQQK	Homo sapiens
1	1.00	1	1	0	0	0	0.004	0	t	142202	KISEASRKGLRRDLDASREA	Homo sapiens
1	1.00	1	1	0	0	0	0.004	0	t	142125	DLDASREAKKQLEAEQQKLE	Homo sapiens
1	1.00	1	1	0	0	0	0.004	0	t	138884	NKLLIAFGLVILLVTLPCAS	Homo sapiens
1	1.00	1	1	0	0	0	0.004	0	t	142201	KGLRRDLDASREAKKQVEKA	Homo sapiens
1	1.00	1	1	0	0	0	0.004	0	t	110284	HKSAIVTLTYDSEWQRDQ	Homo sapiens
1	1.00	1	1	0	0	0	0.004	0	t	112837	SGYIPANTTIPF	Homo sapiens
1	1.00	1	1	0	0	0	0.004	0	t	110284	HKSAIVTLTYDSEWQRDQ	Homo sapiens
1	1.00	1	1	0	0	0	0.004	0	t	65902	TQNISGTQVYQDPAIVQPK	Homo sapiens

1	1.00	1	1	0	0	0	0.004	0	t	72294	WDEDGEKRIPLDVAE	Homo sapiens
1	1.00	1	1	0	0	0	0.004	0	t	71353	VTHESYQELVKKLEALEDA	Homo sapiens
1	1.00	1	1	0	0	0	0.004	0	t	70991	VSILIIFILVSGLSMFVDQL	Homo sapiens
1	1.00	1	1	0	0	0	0.004	0	t	68087	VDVSEGDIVIYSKYG	Homo sapiens
1	1.00	1	1	0	0	0	0.004	0	t	75152	YNGEEYLILSARDVL	Homo sapiens
1	1.00	1	1	0	0	0	0.004	0	t	71284	VTEEI	Homo sapiens
1	1.00	1	1	0	0	0	0.004	0	t	69344	VKYTS	Homo sapiens
1	1.00	1	1	0	0	0	0.004	0	t	62779	TAADTPTATESISPSPP	Homo sapiens
1	1.00	1	1	0	0	0	0.004	0	t	67969	VDGVHAPDKEVKVHADKS	Homo sapiens
1	1.00	1	1	0	0	0	0.004	0	t	75152	YNGEEYLILSARDVL	Homo sapiens
1	1.00	1	1	0	0	0	0.004	0	t	66933	TVAEEHVEEPTVAEE	Homo sapiens
1	1.00	1	1	0	0	0	0.004	0	t	97170	ADQELGDAPFLDRLRRDQKS	Homo sapiens
1	1.00	1	1	0	0	0	0.004	0	t	67656	VAKVNIKPLEDKILV	Homo sapiens
1	1.00	1	1	0	0	0	0.004	0	t	66933	TVAEEHVEEPTVAEE	Homo sapiens

1	1.00	1	1	0	0	0	0.004	0	t	41069	MAKVKIKPLEDKILV	Homo sapiens
1	1.00	1	1	0	0	0	0.004	0	t	38186	LNDITKEYEKLLNEI	Homo sapiens
1	1.00	1	1	0	0	0	0.004	0	t	44168	NHSQTPRYRRPKFPWFK	Homo sapiens
1	1.00	1	1	0	0	0	0.004	0	t	39183	LRRDLDASREAKKQVEKALE	Homo sapiens
1	1.00	1	1	0	0	0	0.004	0	t	54382	RKLFLVALVLFTIGSLICAI	Homo sapiens
1	1.00	1	1	0	0	0	0.004	0	t	42416	MQTLWDEIMDINKRK	Homo sapiens
1	1.00	1	1	0	0	0	0.004	0	t	55038	RNNHPQNTSDSQKECTDGNK	Homo sapiens
1	1.00	1	1	0	0	0	0.004	0	t	38694	LPRSNTSSGASPPAD	Homo sapiens
1	1.00	1	1	0	0	0	0.004	0	t	43248	NANPNANPNANPNANPNANP	Homo sapiens
1	1.00	1	1	0	0	0	0.004	0	t	43248	NANPNANPNANPNANPNANP	Homo sapiens
1	1.00	1	1	0	0	0	0.004	0	t	46895	PAPQAPYQGYQEPPAPQAPY	Homo sapiens
1	1.00	1	1	0	0	0	0.004	0	t	53152	RALLGLAVCLFLMALCT	Homo sapiens
1	1.00	1	1	0	0	0	0.004	0	t	52411	QSQDNSGDQRQVDLTPKK	Homo sapiens

1	1.00	1	1	0	0	0	0.004	0	t	42416	MQTLWDEIMDINKRK	Homo sapiens
1	1.00	1	1	0	0	0	0.004	0	t	22882	GTVVAVGPGRWDEDG	Homo sapiens
1	1.00	1	1	0	0	0	0.004	0	t	11821	EENVEHDAEENVEHDA	Homo sapiens
1	1.00	1	1	0	0	0	0.004	0	t	11815	EENVEENVEENVEENV	Homo sapiens
1	1.00	1	1	0	0	0	0.004	0	t	20012	GGSGGSVASGGSVAS	Homo sapiens
1	1.00	1	1	0	0	0	0.004	0	t	35316	LDVAEGDTVIYSKYG	Homo sapiens
1	1.00	1	1	0	0	0	0.004	0	t	11821	EENVEHDAEENVEHDA	Homo sapiens
1	1.00	1	1	0	0	0	0.004	0	t	11815	EENVEENVEENVEENV	Homo sapiens
1	1.00	1	1	0	0	0	0.004	0	t	11815	EENVEENVEENVEENV	Homo sapiens
1	1.00	1	1	0	0	0	0.004	0	t	22882	GTVVAVGPGRWDEDG	Homo sapiens
1	1.00	1	1	0	0	0	0.004	0	t	22986	GVGSSAVILDDNFVT	Sus scrofa
1	1.00	1	1	0	0	0	0.004	0	t	11815	EENVEENVEENVEENV	Homo sapiens
1	1.00	1	1	0	0	0	0.004	0	t	35794	LFAKYAEEVGEAVIDDDFGY	Homo sapiens

1	1.00	1	1	0	0	0	0.004	0	t	18761	GASAQSGTSGTSGTS	Homo sapiens
1	1.00	1	1	0	0	0	0.004	0	t	23976	HHAERNGSQSTTSQSN	Homo sapiens
1	1.00	1	1	0	0	0	0.004	0	t	32596	KNQLAGKDEYEAKQKEAE	Homo sapiens
1	1.00	1	1	0	0	0	0.004	0	t	11820	EENVEHDAEENVEENV	Homo sapiens
1	1.00	1	1	0	0	0	0.004	0	t	30799	KFNPDLAPG	Homo sapiens
1	1.00	1	1	0	0	0	0.004	0	t	11815	EENVEENVEENVEENV	Homo sapiens
1	1.00	1	1	0	0	0	0.004	0	t	14016	ERLAQHMTDFDVTHHR	Homo sapiens
1	1.00	1	1	0	0	0	0.004	0	t	11815	EENVEENVEENVEENV	Homo sapiens
1	1.00	1	1	0	0	0	0.004	0	t	12531	EINHVQDHALPKENI	Homo sapiens
1	1.00	1	1	0	0	0	0.004	0	t	4132	ARDVLAVVSK	Homo sapiens
1	1.00	1	1	0	0	0	0.004	0	t	4132	ARDVLAVVSK	Homo sapiens
1	1.00	1	1	0	0	0	0.004	0	t	10098	DSDSESDSDSDSDSDSD	Homo sapiens
1	1.00	1	1	0	0	0	0.004	0	t	10106	DSEPFPRQKHKKVD	Homo sapiens

1	1.00	1	1	0	0	0	0.004	0	t	8900	DKILVQANEAETTTA	Homo sapiens
1	1.00	1	1	0	0	0	0.004	0	t	8942	DKNEKGQHEIVEVEEILPE	Homo sapiens
1	1.00	1	1	0	0	0	0.004	0	t	8360	DGDGAAAMRYTEARMTK	Homo sapiens
1	1.00	1	1	-0.01	-0.01	-0.012	-0.014	-0.01	t	39368	LSGIQLFDRI	Homo sapiens
1	1.00	1	1	0.02	0.018	0.017	0.019	0.02	t	35723	LETHPLYQ	Anas platyrhynchos
1	1.00	1	1	0.05	0.052	0.038	0.047	0.053	t	28907	ITGEYVQILDAHAAY	Homo sapiens
1	1.00	1	1	-0.01	-0.013	-0.022	-0.01	-0.009	t	116971	FAVLKLMGRGTKF	Oryctolagus cuniculus
1	1.00	1	1	0.03	0.026	0.033	0.033	0.03	t	115302	IVTAGEERAVTT	Oryctolagus cuniculus
1	1.00	1	1	0.01	0.003	0.004	0.007	0.01	t	44920	NLVPMVATV	Homo sapiens
1	1.00	1	1	0	0	0	0.006	0.001	t	11813	EENVEENV	Homo sapiens
1	1.00	1	1	0.03	0.03	0.028	0.03	0.033	t	1	AA	Oryctolagus cuniculus
1	1.00	1	1	0.01	0.012	0.005	0.013	0.01	t	33107	KRAASKVSARLLTV	Homo sapiens
1	1.00	1	1	0.01	0.01	0.006	0.01	0.011	t	46858	PALTAAETG	Homo sapiens
1	1.00	1	1	0.02	0.018	0.025	0.023	0.019	t	30307	KEARDQEMN	Mus musculus

1	1.00	1	1	0	-0.002	-0.004	-0.001	0	t	73028	WRGSNHSDQTII	Mus musculus
1	1.00	1	1	-0.02	-0.017	-0.015	-0.019	-0.022	t	65476	TNVGLKIRQLLW	Mus musculus
1	1.00	1	1	0	0.002	-0.002	0.003	0.001	t	75937	YTAADCPGLLYLCP	Homo sapiens
1	1.00	1	1	0.02	0.023	0.019	0.02	0.019	t	140077	VQIINKKLDLSNVQSKC	Mus musculus
1	1.00	1	1	0.02	0.02	0.02	0.023	0.02	t	96126	AVNVPKVTKE	Homo sapiens(human)
1	1.00	1	1	0.01	0.008	-0.004	0.009	0.01	t	75561	YQTELARVQKANADAKATY	Mus musculus
1	1.00	1	1	0	0	0.002	0	0	t	55680	RRPQDVKFPG	Homo sapiens
1	1.00	1	1	0.06	0.06	0.06	0.06	0.06	t	75848	YSPEVG	Mus musculus
1	1.00	1	1	0.03	0.032	0.028	0.037	0.032	t	51852	QPHDLGKVGEVIVTKD	Homo sapiens
1	1.00	1	1	-0.01	-0.008	-0.028	-0.013	-0.008	t	95833	SGPSGPSGPSGT	Homo sapiens
1	1.00	1	1	-0.01	-0.01	-0.01	-0.01	-0.01	t	42928	MVDRGW	Homo sapiens
1	1.00	1	1	0.01	0.006	0.009	0.007	0.01	t	58950	SKTNIFWAGDLL	Oryctolagus cuniculus
1	1.00	1	1	-0.01	-0.014	-0.008	-0.01	-0.01	t	7896	DDYQSFF	Mus musculus
1	1.00	1	1	0.02	0.016	0.02	0.017	0.02	t	119923	GYISKAQEYFN	Oryctolagus cuniculus

1	1.00	1	1	0	0.002	-0.007	0.003	0.004	t	133638	LTSRPA	Homo sapiens
1	1.00	1	1	0.01	0.01	0.01	0.01	0.01	t	98797	GGFGP	Mus musculus
1	1.00	1	1	-0.02	-0.016	-0.023	-0.023	-0.023	t	69399	VLCNRAFNPVCG	Homo sapiens
1	1.00	1	1	-0.01	-0.008	-0.013	-0.007	-0.012	t	47750	PGSPAPPPPEHRGGPEEFEG	Homo sapiens
1	1.00	1	1	-0.01	-0.01	-0.008	-0.01	-0.011	t	119735	AEFHRWSSYM	Mus musculus
1	1.00	1	1	-0.01	-0.013	-0.008	-0.01	-0.011	t	119735	AEFHRWSSYM	Oryctolagus cuniculus
1	1.00	1	1	-0.01	-0.01	-0.008	-0.01	-0.011	t	119735	AEFHRWSSYM	Mus musculus
1	1.00	1	1	0.04	0.04	0.04	0.04	0.04	t	98792	GDTWDDD	Mus musculus
1	1.00	1	1	-0.01	-0.008	-0.01	-0.007	-0.01	t	918	AEGEFATAPPSHYSWDPAK	Homo sapiens
1	1.00	1	1	0	0.008	-0.001	0	-0.003	t	19868	GGGWGQPHGGGWGQPH	Gallus gallus
1	1.00	1	1	0	0	0	0	-0.002	t	36958	LKMADPNRFRGKD	Mus musculus
1	1.00	1	1	0	-0.002	-0.002	0.003	0	t	27690	INNAYNMS	Mus musculus
1	1.00	1	1	0.03	0.028	0.023	0.033	0.032	t	76395	YVRFDLELTFVITST	Mus musculus
1	1.00	1	1	0.01	0.008	0.01	0.013	0.01	t	11438	EDSGSNGKKITCECTKPDS	Mus musculus

1	1.00	1	1	0.01	0.006	0.01	0.013	0.01	t	11438	EDSGSNGKKITCECTKPDS	Oryctolagus cuniculus
1	1.00	1	1	0.01	0.018	0.01	0.013	0.01	t	110912	KATPNDGYFWED	Homo sapiens
1	1.00	1	1	0.01	0.01	0.014	0.01	0.008	t	74476	YKLPDHVRT	Mus musculus
1	1.00	1	1	-0.02	-0.023	-0.035	-0.02	-0.019	t	43224	NANANNAVK	Oryctolagus cuniculus
1	1.00	1	1	0.02	0.02	0.02	0.024	0.022	t	34963	LASLCNPKPSDAPVT	Homo sapiens
1	1.00	1	1	0	0	-0.007	0	0	t	159079	RSNHNRRPGYKTQNQG	Mus musculus
1	1.00	1	1	-0.03	-0.03	-0.038	-0.031	-0.03	t	82608	GENKFVATAASV	Homo sapiens
1	1.00	1	1	0	-0.004	-0.002	0.003	-0.002	t	95354	ELSEFIPGASLR	Oryctolagus cuniculus
1	1.00	1	1	-0.02	-0.02	-0.025	-0.014	-0.018	t	109091	GFPGARGP	Rattus norvegicus
1	1.00	1	1	0.01	0.01	0.01	0.01	0.01	t	72087	VYLLPR	Homo sapiens
1	1.00	1	1	0.01	0.014	0.01	0.017	0.01	t	122874	PPARALKRSLWVEVDLLIGS	Homo sapiens
1	1.00	1	1	0.01	0.014	0.01	0.017	0.01	t	122874	PPARALKRSLWVEVDLLIGS	Homo sapiens
1	1.00	1	1	0.03	0.029	0.03	0.029	0.03	t	42531	MSDLTDIQED	Myodes glareolus
1	1.00	1	1	0.05	0.05	0.052	0.05	0.048	t	18595	GAGKALTGLSTGTSQNTRIS	Homo sapiens

1	1.00	1	1	-0.02	-0.02	-0.022	-0.017	-0.02	t	70648	VQSVASSRRHKRFAG	Homo sapiens
1	1.00	1	1	0.01	0.006	-0.002	0.009	0.01	t	3972	AQGQYSNTLLRIYRDNKTIN	Oryctolagus cuniculus
1	1.00	1	1	-0.01	-0.01	-0.01	-0.001	-0.009	t	135642	CKNKEKKC	Homo sapiens
1	1.00	1	1	0.02	0.02	0.024	0.02	0.02	t	23139	GVSGGS	Homo sapiens
1	1.00	1	1	0	0	0	0	0.001	t	139239	RWPEFSKFGGFDPQG	Oryctolagus cuniculus
1	1.00	1	1	0.01	0.01	0.01	0.01	0.01	t	123612	PPGMIPPP	Oryctolagus cuniculus
1	1.00	1	1	-0.01	-0.01	-0.003	-0.01	-0.013	t	108825	AVLMLPVWACLS	Mus musculus
1	1.00	1	1	-0.03	-0.03	-0.03	-0.03	-0.03	t	115874	NQFLPYPYYA	Homo sapiens
1	1.00	1	1	-0.04	-0.042	-0.034	-0.041	-0.044	t	133539	CYKPGEWAC	Mus musculus
1	1.00	1	1	0	0	0	0	0	t	70269	VPDKEVLYQ	Homo sapiens
1	1.00	1	1	0.03	0.033	0.016	0.03	0.032	t	7134	CTEKFTFSKNPADTG	Mus musculus
1	1.00	1	1	0.03	0.033	0.016	0.03	0.032	t	7134	CTEKFTFSKNPADTG	Mus musculus
1	1.00	1	1	0.01	0.008	0.008	0.013	0.01	t	30194	KDLEDGSKYYFDEDTA	Mus musculus
1	1.00	1	1	0.04	0.037	0.034	0.04	0.042	t	133540	DALVAKAQEVS	Oryctolagus cuniculus
1	1.00	1	1	-0.03	-0.03	-0.03	-0.03	-0.03	t	22127	GRLHLNDRV	Homo sapiens

1	1.00	1	1	0.02	0.023	0.024	0.02	0.018	t	106771	RERMS	Mus musculus
1	1.00	1	1	0.02	0.024	0.015	0.017	0.017	t	78111	CLVRTPEVDDEALEKFDKAL	Homo sapiens
1	1.00	1	1	0.02	0.024	0.02	0.021	0.02	t	70664	VQVVYDYQ	Homo sapiens
1	1.00	1	1	-0.03	-0.028	-0.03	-0.027	-0.031	t	120699	KMFPEVKEKGMAALPRL	Homo sapiens
1	1.00	1	1	0.06	0.065	0.053	0.061	0.061	t	138836	CRTVYPSEEETGE	Homo sapiens
1	1.00	1	1	0	0	0.001	0	-0.001	t	20526	GKDDHYLLEF	Homo sapiens
1	1.00	1	1	-0.01	-0.013	-0.007	-0.01	-0.012	t	18649	GAKYMVLQGEPGAV	Oryctolagus cuniculus
1	1.00	1	1	0.02	0.018	0.012	0.013	0.017	t	122205	VFTLPPPLPETH	Mus musculus
1	1.00	1	1	0	0	0	0	0	t	105583	VTT	Oryctolagus cuniculus
1	1.00	1	1	0	-0.002	0	0.003	0	t	49321	PSDKHIEQYLKKIKNSIS	Mus musculus
1	1.00	1	1	-0.02	-0.02	-0.02	-0.02	-0.02	t	117449	CVP	Homo sapiens
1	1.00	1	1	-0.02	-0.02	-0.02	-0.02	-0.02	t	117449	CVP	Homo sapiens
1	1.00	1	1	-0.02	-0.016	-0.021	-0.023	-0.021	t	39480	LSLILNRLC	Homo sapiens
1	1.00	1	1	0.03	0.03	0.027	0.03	0.03	t	157833	RDPYSPSQDPYSPS	Homo sapiens
1	1.00	1	1	0.01	0.01	0.01	0.01	0.01	t	134151	LRRVIGD	Homo sapiens

1	1.00	1	1	0	0	0.003	0	-0.001	t	1602	AGLLELNG W	Rattus norvegic us
1	1.00	1	1	-0.01	-0.01	-0.01	-0.01	-0.012	t	52138	QQQTAPK APT	Oryctola gus cuniculu s
1	1.00	1	1	0.01	0.01	0.01	0.01	0.011	t	117231	RQIFGDYK TTICGK	Mus musculu s
1	1.00	1	1	0.02	0.017	0.006	0.02	0.024	t	97591	QSATAVLG A	Oryctola gus cuniculu s
1	1.00	1	1	0	0	0	0	0.001	t	42493	MRQLDTN VER	Homo sapiens
1	1.00	1	1	0.03	0.03	0.032	0.03	0.029	t	6948	CRTCTTPA QGTSMY	Mus musculu s
1	1.00	1	1	0.06	0.06	0.06	0.06	0.06	t	103029	AGTVNIGA SDAYLSE	Homo sapiens
1	1.00	1	1	0.04	0.04	0.04	0.04	0.04	t	149082	NPSQGPL	Sus scrofa
1	1.00	1	1	0.04	0.04	0.04	0.04	0.04	t	103745	YEEQMQD AFETGVM F	Homo sapiens
1	1.00	1	1	0.02	0.02	0.02	0.02	0.02	t	157741	PHYSNAP QLVYI	Homo sapiens
1	1.00	1	1	0.02	0.02	0.02	0.02	0.02	t	133659	NPWYNDL SLLTA	Homo sapiens
1	1.00	1	1	0.02	0.02	0.02	0.02	0.02	t	114885	LCAYSIEFG T	Homo sapiens
1	1.00	1	1	-0.05	-0.043	-0.036	-0.05	-0.056	t	4504	ASKLCLG WLWG	Cavia porcellu s
1	1.00	1	1	0.01	0.01	0.01	0.01	0.01	t	136219	FEEYGKIDT IEIIT	Homo sapiens
1	1.00	1	1	-0.01	-0.01	-0.01	-0.01	-0.01	t	54432	RKTKRNTN R	Homo sapiens
1	1.00	1	1	0	0	-0.007	0	0.002	t	44342	NISGTGEK AQQHRKQ	Homo sapiens

1	1.00	1	1	-0.04	-0.04	-0.043	-0.04	-0.041	t	136074	LVKK	Mus musculus
1	1.00	1	1	-0.01	-0.01	-0.01	-0.01	-0.01	t	127665	GIQPTWVPFHST	Trichosurus vulpecula
1	1.00	1	1	0	-0.003	0	0	-0.001	t	48992	PQEQITQHGSPYGRC	Oryctolagus cuniculus
1	1.00	1	1	0.01	0.01	0.01	0.01	0.008	t	39107	LRGSGDLQEYLRHVTR	Oryctolagus cuniculus
1	1.00	1	1	-0.04	-0.04	-0.04	-0.044	-0.04	t	139511	GPM	Homo sapiens
1	1.00	1	1	0.04	0.042	0.04	0.039	0.04	t	149435	QPQYSQPQQP	Homo sapiens
1	1.00	1	1	0	0	-0.013	0.002	0.003	t	98891	LQQQLIPCMDVV	Homo sapiens
1	1.00	1	1	-0.01	-0.008	-0.012	-0.007	-0.01	t	13670	EPIRDALNAMTQNIR	Homo sapiens
1	1.00	1	1	0.01	0.008	-0.002	0.007	0.01	t	95742	QSLACQ	Homo sapiens
1	1.00	1	1	0.05	0.05	0.05	0.05	0.048	t	140996	EEIDYDDTISVEMKK	Mus musculus
1	1.00	1	1	0.04	0.042	0.04	0.037	0.037	t	106315	CSGTKKLSEE	Homo sapiens
1	1.00	1	1	0	-0.003	-0.012	0	0	t	112739	YTTGAVRQIFGDYKTTICGK	Oryctolagus cuniculus
1	1.00	1	1	-0.04	-0.038	-0.04	-0.033	-0.039	t	144619	DYIWNTLNSG	Homo sapiens
1	1.00	1	1	-0.01	-0.01	-0.01	-0.01	-0.011	t	75949	YTASARGDLAHL	Mus musculus
1	1.00	1	1	0.01	0.008	0.015	0.009	0.009	t	146839	DLRTL	Mus musculus

1	1.00	1	1	0.01	0.013	0	0.01	0.01	t	44487	NKENCGAA	Mus musculus
1	1.00	1	1	0.03	0.032	0.039	0.037	0.03	t	112336	SSQSVLY	Homo sapiens
1	1.00	1	1	0.02	0.02	0.014	0.017	0.021	t	67729	VAQYKALPVVLENA	Homo sapiens
1	1.00	1	1	0	0.008	0	0	-0.002	t	156937	PMSKSHL	Gallus gallus
1	1.00	1	1	-0.03	-0.036	-0.044	-0.03	-0.03	t	53338	RDEWWKVIKKDVWNVISWVF	Oryctolagus cuniculus
1	1.00	1	1	-0.03	-0.033	-0.044	-0.03	-0.03	t	53338	RDEWWKVIKKDVWNVISWVF	Oryctolagus cuniculus
1	1.00	1	1	0.03	0.03	0.03	0.03	0.03	t	158613	SQSVAAS	Homo sapiens
1	1.00	1	1	0.01	0.01	0.01	0.01	0.01	t	149067	LNFDPPS	Sus scrofa
1	1.00	1	1	0.01	0.01	0.01	0.01	0.01	t	114752	AYSIEFGTNI	Homo sapiens
1	1.00	1	1	-0.01	-0.01	-0.01	-0.01	-0.01	t	123798	TTLKMWSDF	Ovis aries
1	1.00	1	1	0.04	0.04	0.034	0.04	0.041	t	13524	ENMTPQD	Mus musculus
1	1.00	1	1	-0.02	-0.02	-0.02	-0.02	-0.02	t	96102	APPLLPH	Homo sapiens
1	1.00	1	1	0.01	0.01	0.01	0.01	0.01	t	149934	SGKFYPV	Capra hircus
1	1.00	1	1	0.02	0.016	0.018	0.019	0.019	t	122153	NASGLSRPSPSAH	Oryctolagus cuniculus
1	1.00	1	1	0.03	0.027	0.028	0.03	0.031	t	127185	TCDDPRFQDSS	Oryctolagus cuniculus

1	1.00	1	1	-0.02	-0.018	-0.026	-0.019	-0.02	t	50203	QAAAGDKPS	Homo sapiens
1	1.00	1	1	0.03	0.03	0.021	0.03	0.033	t	110733	VGENVPDDLYIKGSG	Mus musculus
1	1.00	1	1	0	-0.004	0	-0.001	-0.002	t	71353	VTHESYQELVKKLEALEDA	Oryctolagus cuniculus
1	1.00	1	1	0	-0.002	0	-0.001	-0.002	t	62779	TAADTPTATESISPSPP	Mus musculus
1	1.00	1	1	0	0.006	0	-0.001	-0.002	t	43242	NANPNANPNANP	Macaca fascicularis
1	1.00	1	1	0	0	-0.006	0	0.002	t	17328	FPLDLT	Mus musculus
1	1.00	1	1	0.02	0.017	0.027	0.02	0.019	t	67189	TVTYESFHYFGSAPT	Oncorhynchus tshawytscha
1	1.00	1	1	0.01	0.01	0.011	0.01	0.009	t	60594	SQWNKPSK	Mus musculus
1	1.00	1	1	0.03	0.03	0.03	0.03	0.032	t	8355	DGAVQPDGG	Oryctolagus cuniculus
1	1.00	1	1	0.03	0.033	0.03	0.03	0.032	t	8355	DGAVQPDGG	Mus musculus
1	1.00	1	1	-0.03	-0.03	-0.03	-0.03	-0.03	t	79463	WALFRS	Mus musculus
1	1.00	1	1	0.02	0.02	0.018	0.017	0.018	t	100639	WCVPKPGVSDDQ	Homo sapiens
1	1.00	1	1	-0.03	-0.029	-0.041	-0.031	-0.032	t	59853	SNKEGVAADGDALNK	Oryctolagus cuniculus
1	1.00	1	1	0.04	0.04	0.04	0.04	0.04	t	151035	NGTYDYPKYSEEAKL	Homo sapiens

1	1.00	1	1	0.02	0.02	0.02	0.02	0.02	t	99728	TPEFLEQAFQVDDRQ	Homo sapiens
1	1.00	1	1	0.01	0.008	0.007	0.013	0.012	t	52825	QVYYRPVDQ	Mus musculus
1	1.00	1	1	-0.04	-0.04	-0.04	-0.033	-0.041	t	117519	GRGTVAAAAAAT	Mus musculus
1	1.00	1	1	0.05	0.048	0.035	0.053	0.052	t	52357	QSKSTSAASTSDELSGSEGP	Mus musculus
1	1.00	1	1	0.02	0.02	0.012	0.019	0.019	t	65672	TPNSIAAISM	Mus musculus
1	1.00	1	1	0	0	-0.004	-0.003	-0.002	t	32908	KPTPEGDLEILLQK	Homo sapiens
1	1.00	1	1	-0.02	-0.02	-0.023	-0.02	-0.02	t	98974	QTMKGLDIQK	Homo sapiens
1	1.00	1	1	-0.04	-0.04	-0.04	-0.038	-0.04	t	135894	AHGCLFDRRLCS	Homo sapiens
1	1.00	1	1	0	-0.001	-0.001	0	0	t	14749	EVKKKSDS	Mus musculus
1	1.00	1	1	0.02	0.02	0.02	0.02	0.019	t	99836	QPFPQPQ	Mus musculus
1	1.00	1	1	0.02	0.022	0.011	0.02	0.023	t	110733	VGENVPDDLYIKGSG	Homo sapiens
1	1.00	1	1	-0.04	-0.04	-0.04	-0.037	-0.04	t	149025	ANILSRR	Sus scrofa
1	1.00	1	1	-0.02	-0.02	-0.038	-0.02	-0.018	t	13634	EPEGIHYHDKFE	Mus musculus
1	1.00	1	1	0.03	0.028	0.032	0.029	0.03	t	53924	RGLYFPAG	Mus musculus
1	1.00	1	1	-0.01	-0.01	-0.01	-0.007	-0.01	t	22244	GRVVLSGKPAIIPDREVL	Homo sapiens

1	1.00	1	1	0.03	0.033	0.029	0.03	0.028	t	98328	PEVTKAEE	Mus musculus
1	1.00	1	1	0.03	0.03	0.029	0.03	0.028	t	98328	PEVTKAEE	Mus musculus
1	1.00	1	1	0.04	0.042	0.04	0.041	0.039	t	150999	IPSIQSRGLFGAIAG	Homo sapiens
1	1.00	1	1	0	0	0	0	-0.001	t	119528	SRKKTFTGA	Homo sapiens
1	1.00	1	1	-0.02	-0.024	-0.01	-0.02	-0.022	t	74497	YKQKIIAP	Mus musculus
1	1.00	1	1	0.01	0.012	0.003	0.007	0.01	t	73590	YDTADII	Felis catus
1	1.00	1	1	0	0	0	0	-0.002	t	48597	PNANPN	Mus musculus
1	1.00	1	1	0.03	0.03	0.03	0.03	0.03	t	150801	EDDF	Homo sapiens
1	1.00	1	1	0.03	0.03	0.03	0.03	0.03	t	150801	EDDF	Homo sapiens
1	1.00	1	1	0.03	0.034	0.022	0.031	0.032	t	73483	YCADVAAEELMNALV	Homo sapiens
1	1.00	1	1	-0.02	-0.02	-0.02	-0.02	-0.02	t	78628	DQKPPSR	Oryctolagus cuniculus
1	1.00	1	1	-0.02	-0.02	-0.02	-0.02	-0.02	t	78628	DQKPPSR	Oryctolagus cuniculus
1	1.00	1	1	-0.02	-0.018	-0.016	-0.019	-0.02	t	51974	QPTELRYSW	Homo sapiens
1	1.00	1	1	-0.02	-0.022	-0.016	-0.019	-0.02	t	51974	QPTELRYSW	Homo sapiens

1	1.00	1	1	0	0	0	0.004	0	t	48442	PLPHVVDLPQ	Homo sapiens
1	1.00	1	1	0	0	0	0	0	t	120681	KILPEVKEKH	Oryctolagus cuniculus
1	1.00	1	1	-0.01	-0.01	-0.01	-0.006	-0.01	t	118375	GKALCWVCAQSRKHRDHAMV	Homo sapiens
1	1.00	1	1	0.01	0.01	0.01	0.01	0.009	t	153835	HGDTPTLHEYMLDLQP	Cavia porcellus
1	1.00	1	1	-0.02	-0.02	-0.02	-0.02	-0.02	t	47789	PGYPWPLY	Homo sapiens
1	1.00	1	1	0	0	0	0.001	-0.001	t	7765	DDEHVEEPTVA	Homo sapiens
1	1.00	1	1	0.01	0.014	0.01	0.017	0.011	t	122892	RSILPYGDSMDRIE	Homo sapiens
1	1.00	1	1	0	0	0	0	-0.002	t	9753	DPRVRGLY	Mus musculus
1	1.00	1	1	0.01	0.01	0.01	0.014	0.01	t	131250	NHVQAWEQFWDS	Homo sapiens
1	1.00	1	1	0	0	-0.005	0	0.002	t	133608	INTTVCAGY	Mus musculus
1	1.00	1	1	0.02	0.018	0.019	0.02	0.022	t	134471	PKPEQ	Mus musculus
1	1.00	1	1	0.03	0.03	0.023	0.03	0.028	t	95897	TDEYCSPDHN	Homo sapiens
1	1.00	1	1	0.05	0.046	0.041	0.05	0.052	t	20046	GGTEESVVTASRS	Bos taurus
1	1.00	1	1	-0.01	-0.004	-0.011	-0.009	-0.008	t	121074	YDFAEAKSGSSIPAK	Equus caballus
1	1.00	1	1	-0.01	-0.01	-0.01	-0.01	-0.012	t	55305	RPQQPFPLQP	Mus musculus
1	1.00	1	1	-0.01	-0.012	-0.008	-0.011	-0.01	t	73070	WSFADGN	Mus musculus

1	1.00	1	1	0	0	0	0	-0.001	t	61944	STTNK	Mus musculus
1	1.00	1	1	0.02	0.02	0.02	0.02	0.02	t	95930	TTLNPTI	Mus musculus
1	1.00	1	1	-0.01	-0.005	-0.007	-0.014	-0.01	t	25510	ICLAVLWVVK	Homo sapiens
1	1.00	1	1	0.02	0.02	0.017	0.013	0.017	t	115630	NFDKLYIWG	Homo sapiens
1	1.00	1	1	0	-0.003	-0.01	0.001	0	t	4763	ASSAAEFLAAFNVFSEAARC	Canis lupus
1	1.00	1	1	0.01	0.01	0.01	0.01	0.01	t	143845	DLFVTAW	Homo sapiens
1	1.00	1	1	0.01	0.01	0.01	0.01	0.01	t	98797	GGFGP	Mus musculus
1	1.00	1	1	0	0.002	-0.009	0.001	-0.002	t	51793	QPAPATAPSQGGV	Homo sapiens
1	1.00	1	1	0	0.004	-0.009	0.001	-0.002	t	51793	QPAPATAPSQGGV	Homo sapiens
1	1.00	1	1	0.01	0.012	0.01	0.01	0.01	t	117749	APAMIPP	Homo sapiens
1	1.00	1	1	0.02	0.02	0.021	0.017	0.018	t	44227	NIFSGFTPEFLEQAF	Homo sapiens
1	1.00	1	1	0.02	0.021	0.006	0.017	0.02	t	35282	LDRLVESAKREMAQKLA	Equus caballus
1	1.00	1	1	0.04	0.04	0.04	0.04	0.039	t	99775	WELQGDRRCQSQLER	Homo sapiens
1	1.00	1	1	0	0	0	0	-0.001	t	138249	TFRVVSV	Homo sapiens
1	1.00	1	1	0.02	0.02	0.02	0.02	0.02	t	79223	GASHTV	Mus musculus

1	1.00	1	1	-0.02	-0.018	-0.027	-0.017	-0.018	t	49855	PVEIRRGVML	Homo sapiens
1	1.00	1	1	0.05	0.05	0.05	0.05	0.05	t	95928	TSDVAG	Mus musculus
1	1.00	1	1	0.03	0.03	0.03	0.03	0.03	t	132851	KMSESGAGVQFYLPS	Trichosurus vulpecula
1	1.00	1	1	0.03	0.03	0.03	0.03	0.03	t	99546	PSQDPYSPSPYDRRG	Homo sapiens
1	1.00	1	1	-0.02	-0.018	-0.02	-0.019	-0.02	t	4023	AQPGYPWPLY	Homo sapiens
1	1.00	1	1	0.02	0.023	0.02	0.02	0.02	t	38918	LQND	Mus musculus
1	1.00	1	1	0.02	0.023	0.02	0.02	0.02	t	38918	LQND	Mus musculus
1	1.00	1	1	-0.01	-0.01	-0.01	-0.01	-0.01	t	48782	PPFTSAVGGVDHRS	Mus musculus
1	1.00	1	1	-0.02	-0.02	-0.02	-0.02	-0.02	t	134752	MGPKRRQLTF	Homo sapiens
1	1.00	1	1	-0.01	-0.008	-0.007	-0.009	-0.008	t	34278	KVYVGNYDF	Homo sapiens
1	1.00	1	1	-0.01	-0.006	-0.01	-0.007	-0.011	t	110766	AIKSEVSLTGAL	Homo sapiens
1	1.00	1	1	0.07	0.068	0.061	0.063	0.07	t	110884	HEYMLDLQPETTDLYCYE	Mus musculus
1	1.00	1	1	0.07	0.068	0.061	0.063	0.07	t	110884	HEYMLDLQPETTDLYCYE	Mus musculus
1	1.00	1	1	0.07	0.068	0.061	0.063	0.07	t	110884	HEYMLDLQPETTDLYCYE	Mus musculus
1	1.00	1	1	-0.03	-0.026	-0.019	-0.023	-0.03	t	140413	VVLGAHNV	Homo sapiens

1	1.00	1	1	0	0.002	0	0.001	0	t	65932	TQSFLGTTISGVLW	Homo sapiens
1	1.00	1	1	-0.02	-0.02	-0.02	-0.02	-0.021	t	102005	QPDQHHQ	Mus musculus
1	1.00	1	1	0	0.004	-0.015	0.001	0.003	t	60797	SRSESRKNRGGREEIL	Homo sapiens
1	1.00	1	1	0.03	0.03	0.023	0.027	0.03	t	69827	VLVLDTDYK	Homo sapiens
1	1.00	1	1	0.02	0.02	0.02	0.02	0.02	t	293	AALLQSAR	Oryctolagus cuniculus
1	1.00	1	1	0.05	0.05	0.037	0.05	0.053	t	110389	QTISTHLN	Mus musculus
1	1.00	1	1	0.03	0.032	0.028	0.031	0.029	t	28291	IRFKDDTSFEDING	Homo sapiens
1	1.00	1	1	0.02	0.02	0.02	0.02	0.02	t	99053	VVNKGTGNLELVAVR	Homo sapiens
1	1.00	1	1	0.02	0.02	0.02	0.02	0.02	t	79459	VRDGYIV	Oryctolagus cuniculus
1	1.00	1	1	0.02	0.02	0.02	0.02	0.02	t	78942	CTLSVFGYWVC	Mus musculus
1	1.00	1	1	0.02	0.02	0.02	0.02	0.02	t	79459	VRDGYIV	Oryctolagus cuniculus
1	1.00	1	1	0.01	0.012	-0.008	0.007	0.012	t	95833	SGPSGPSGPSGT	Homo sapiens
1	1.00	1	1	0.01	0.012	-0.008	0.007	0.012	t	95833	SGPSGPSGPSGT	Homo sapiens
1	1.00	1	1	0.02	0.009	0.024	0.02	0.018	t	23494	GYVRSGGAVPPACCN	Oryctolagus cuniculus

1	1.00	1	1	0.06	0.058	0.06	0.053	0.058	t	131128	VEMKKED FDIYDEDE	Mus musculus
1	1.00	1	1	0.06	0.056	0.06	0.053	0.058	t	131128	VEMKKED FDIYDEDE	Oryctolagus cuniculus
1	1.00	1	1	0.05	0.05	0.048	0.046	0.05	t	15323	FCETNGLE YI	Homo sapiens
1	1.00	1	1	-0.01	-0.008	-0.005	-0.007	-0.01	t	102563	KKRPKPG	Homo sapiens
1	1.00	1	1	0	0	0	0.006	0.001	t	71284	VTEEI	Homo sapiens
1	1.00	1	1	0	-0.002	-0.006	-0.001	0	t	51161	QIYTINDKI L	Mus musculus
1	1.00	1	1	0.01	0.01	0.01	0.01	0.01	t	1062	AELTGY	Homo sapiens
1	1.00	1	1	0	0	0	-0.003	0	t	96714	NVPKINPK KD	Homo sapiens(human)
1	1.00	1	1	0.06	0.048	0.058	0.06	0.06	t	55924	RSQDACIE FVQQEVD	Mus musculus
1	1.00	1	1	0.06	0.046	0.058	0.06	0.06	t	55924	RSQDACIE FVQQEVD	Oryctolagus cuniculus
1	1.00	1	1	0.01	0.012	0.008	0.007	0.007	t	105997	PFKFFVPP SEQSLRA	Homo sapiens
1	1.00	1	1	0.02	0.02	0.02	0.02	0.018	t	118158	SRNLYDIG E	Homo sapiens
1	1.00	1	1	0	-0.002	0.01	0	0	t	138937	YIGEVLVS V	Homo sapiens
1	1.00	1	1	0	0	0	0	-0.001	t	140510	TTTHFLAT KFYK	Mus musculus
1	1.00	1	1	-0.01	-0.022	-0.008	-0.01	-0.012	t	146185	DAEFGH	Macaca mulatta
1	1.00	1	1	0.02	0.018	0.018	0.017	0.022	t	120609	IFAD	Rattus norvegicus
1	1.00	1	1	0.01	0.01	0.002	0.01	0.013	t	110976	QKSQRSQ DENPVVH F	Rattus norvegicus

1	1.00	1	1	0	0	0	0	-0.002	t	144705	KGTHSLYS QIAG	Mus musculu s
1	1.00	1	1	0	0	0	0	-0.002	t	144654	GPHPTLEV VPMGRGS	Mus musculu s
1	1.00	1	1	0	0	0	0	-0.002	t	138626	SQKDILER KRAAVDR VC	Oryctola gus cuniculu s
1	1.00	1	1	0	0	0	0	-0.002	t	134909	EEKLVF	Mus musculu s
1	1.00	1	1	0	0	0	0	-0.002	t	136381	IKHRKRRL RMIL	Mus musculu s
1	1.00	1	1	0	0	0	0	-0.002	t	144599	CNGTHPSL RLC	Mus musculu s
1	1.00	1	1	0	0	0	0	-0.002	t	138628	SQKEVLER TRAELDTV C	Oryctola gus cuniculu s
1	1.00	1	1	0	0	0	0	-0.002	t	141683	KDWG	Mus musculu s
1	1.00	1	1	0	0	0	0	-0.002	t	142913	MVLNEGT SGTA	Oryctola gus cuniculu s
1	1.00	1	1	0	0	0	0	-0.002	t	136649	SPQRRKRT RQIR	Mus musculu s
1	1.00	1	1	0	0	0	0	-0.002	t	149684	EPAGSNR	Mus musculu s
1	1.00	1	1	0	0	0	0	-0.002	t	144797	TGGCTHG CLAVP	Mus musculu s
1	1.00	1	1	0	0	0	0	-0.002	t	144778	SGTHPFLD KSFS	Mus musculu s
1	1.00	1	1	0	0	0	0	-0.002	t	144676	GTHPSLQF T	Mus musculu s
1	1.00	1	1	0	0	0	0	-0.002	t	144598	CFVGTHP QLRC	Mus musculu s
1	1.00	1	1	0	0	0	0	-0.002	t	141693	KTWG	Mus musculu s

1	1.00	1	1	0	0	0	0	-0.002	t	138862	KFPED	Mus musculus
1	1.00	1	1	0	0	0	0	-0.002	t	144809	VLAPGGTHSRGLLEM	Mus musculus
1	1.00	1	1	0	0	0	0	-0.002	t	144777	SGTHMNLYLHDTAFA	Mus musculus
1	1.00	1	1	0	0	0	0	-0.002	t	138627	SQKEVLEGARASVDRVC	Oryctolagus cuniculus
1	1.00	1	1	0	0	0	0	-0.002	t	144685	HVGTHPMLQPSF	Mus musculus
1	1.00	1	1	0	0	0	0	-0.002	t	135569	KTNMK	Mus musculus
1	1.00	1	1	0	0	0	0	-0.002	t	144793	SYGGRKGTHPGLEMP	Mus musculus
1	1.00	1	1	0	0	0	0	-0.002	t	144601	CSGTHPSISSC	Mus musculus
1	1.00	1	1	0	0	0	0	-0.002	t	136551	PIQRKRRQMPLS	Mus musculus
1	1.00	1	1	0	0	0	0	-0.002	t	144756	QVGTHPGLANDYEYT	Mus musculus
1	1.00	1	1	0	0	0	0	-0.002	t	135799	YGKDVKDLFDYAQE	Mus musculus
1	1.00	1	1	0	0	0	0	-0.002	t	136358	HMRKRTKIKKIR	Mus musculus
1	1.00	1	1	0	0	0	0	-0.002	t	144678	GTQPTLEVVPMGRGS	Mus musculus
1	1.00	1	1	0	-0.003	0	0	-0.002	t	142441	LFSPWLRVQNHF	Oryctolagus cuniculus
1	1.00	1	1	0	0	0	0	-0.002	t	144656	GPTHAMFDSSGP	Mus musculus
1	1.00	1	1	0	0	0	0	-0.002	t	136392	IPKSRKRRIPQR	Mus musculus

1	1.00	1	1	0	0	0	0	-0.002	t	134945	FKAPRP	Mus musculus
1	1.00	1	1	0	0	0	0	-0.002	t	139029	DLQVLT	Mus musculus
1	1.00	1	1	0	0	0	0	-0.002	t	127643	ERPLELRVRVLDI	Homo sapiens
1	1.00	1	1	0	0	0	0	-0.002	t	125085	GTPSHSRRQPHVMS	Macaca fascicularis
1	1.00	1	1	0	0	0	0	-0.002	t	133156	IRLR	Oryctolagus cuniculus
1	1.00	1	1	0	-0.003	0	0	-0.002	t	125602	WMSYPDRTLRTS	Oryctolagus cuniculus
1	1.00	1	1	0	0	0	0	-0.002	t	132233	DSGCVINWKGRELKC	Mus musculus
1	1.00	1	1	0	0	0	0	-0.002	t	127857	TDVRYKDDMYHFFCPAIQAQ	Mus musculus
1	1.00	1	1	0	0	0	0	-0.002	t	122871	PETQPGPLTLELQIAKDK	Oryctolagus cuniculus
1	1.00	1	1	0	0	0	0	-0.002	t	119304	PNQEKVSGIPEQEYSC	Oryctolagus cuniculus
1	1.00	1	1	0	0	0	0	-0.002	t	119048	VSDYEMKLMDLDVEQ	Mus musculus
1	1.00	1	1	0	0	0	0	-0.002	t	132984	AIQNIRLRHENKDL	Oryctolagus cuniculus
1	1.00	1	1	0	0	0	0	-0.002	t	125600	WESYPFRVGTSL	Mus musculus
1	1.00	1	1	0	0	0	0	-0.002	t	122375	GLINRAKAVKQFEESQG	Mus musculus

1	1.00	1	1	0	0	0	0	-0.002	t	132147	AHLDETVWERPHEFW	Mus musculus
1	1.00	1	1	0	0	0	0	-0.002	t	125363	APKTYVSVSGMV	Mus musculus
1	1.00	1	1	0	0	0	0	-0.002	t	125209	QFQIHGPR	Mus musculus
1	1.00	1	1	0	0	0	0	-0.002	t	133157	IRLRHENKDLKARL	Oryctolagus cuniculus
1	1.00	1	1	0	0	0	0	-0.002	t	117940	KARIHPFHILIALETYKTGH	Mus musculus
1	1.00	1	1	0	0	0	0	-0.002	t	119220	ISCAKDGGIPEQEYSC	Oryctolagus cuniculus
1	1.00	1	1	0	0	0	0	-0.002	t	132660	SKAVHADMGYWIESE	Mus musculus
1	1.00	1	1	0	0	0	0	-0.002	t	122512	PEPAKSAPAPKKGSK	Mus musculus
1	1.00	1	1	0	0	0	0	-0.002	t	129732	RERRGIALDGKI	Mus musculus
1	1.00	1	1	0	0	0	0	-0.002	t	132727	VTNEVHTWTEQYKFQ	Mus musculus
1	1.00	1	1	0	0	0	0	-0.002	t	132397	KEENLVKSLVSAGSG	Mus musculus
1	1.00	1	1	0	0	0	0	-0.002	t	125602	WMSYPDRTLRTS	Mus musculus
1	1.00	1	1	0	0	0	0	-0.002	t	125004	DAPDTDWCDSIP	Mus musculus
1	1.00	1	1	0	0	0	0	-0.002	t	131576	NSRQTDREDELI	Rattus norvegicus
1	1.00	1	1	0	0	0	0	-0.002	t	131654	SGFGVVKKPNY	Oryctolagus cuniculus

1	1.00	1	1	0	0	0	0	-0.002	t	127852	TCTIKVVG GYQVNIRV GDTT	Mus musculu s
1	1.00	1	1	0	0	0	0	-0.002	t	117506	GGAHFSVS SLAEG	Mus musculu s
1	1.00	1	1	0	0	0	0	-0.002	t	118877	LDVEQLGI PEQEYSC	Oryctola gus cuniculu s
1	1.00	1	1	0	0	0	0	-0.002	t	127856	TDVRYKD	Mus musculu s
1	1.00	1	1	0	0	0	0	-0.002	t	124713	SGEQPSDE QPSGEHG	Mus musculu s
1	1.00	1	1	0	0	0	0	-0.002	t	120869	PGYRSISG PDPRIRKT	Mus musculu s
1	1.00	1	1	0	0	0	0	-0.002	t	132276	ERIYHFV	Mus musculu s
1	1.00	1	1	0	0	0	0	-0.002	t	122712	ADPEGPLP FPYFERQ	Mus musculu s
1	1.00	1	1	0	0	0	0	-0.002	t	132687	STQTAGP WHLGKLEL	Mus musculu s
1	1.00	1	1	0	0	0	0	-0.002	t	132200	CGNRGPSL RTTTVTG	Mus musculu s
1	1.00	1	1	0	0	0	0	-0.002	t	123849	WQDE	Mus musculu s
1	1.00	1	1	0	0	0	0	-0.002	t	119382	VISCAKDAI EDEEGSC	Oryctola gus cuniculu s
1	1.00	1	1	0	0	0	0	-0.002	t	118599	SAPAPKKG SKKAV	Mus musculu s
1	1.00	1	1	0	0	0	0	-0.002	t	123734	SFSKASNS WFPVEGP ADICQ	marmos ets
1	1.00	1	1	0	0	0	0	-0.002	t	131381	CTYVLDPE NL	Mus musculu s
1	1.00	1	1	0	0	0	0	-0.002	t	125052	FSLRLMEE NW	Mus musculu s

1	1.00	1	1	0	0	0	0	-0.002	t	130524	KFHPKDIP YQVW	Mus musculu s
1	1.00	1	1	0	0	0	0	-0.002	t	118867	KLSQTSNV DKEEEAV	Mus musculu s
1	1.00	1	1	0	0	0	0	-0.002	t	125021	DWC	Mus musculu s
1	1.00	1	1	0	0	0	0	-0.002	t	131374	CKAKAQT DREDLRTL LRY	Rattus norvegic us
1	1.00	1	1	0	0	0	0	-0.002	t	117520	GSFSDADL AD	Mus musculu s
1	1.00	1	1	0	0	0	0	-0.002	t	108382	GGVVIA	Mus musculu s
1	1.00	1	1	0	0	0	0	-0.002	t	103998	IVKWDRD M	Mus musculu s
1	1.00	1	1	0	0	0	0	-0.002	t	104443	DAEFRHDS GYE	Homo sapiens
1	1.00	1	1	0	0	0	0	-0.002	t	108378	GFAGQAG PAGATGA PGRP	Mus musculu s
1	1.00	1	1	0	0	0	0	-0.002	t	105561	VAVVRTPP KSPSSAK	Mus musculu s
1	1.00	1	1	0	0	0	0	-0.002	t	106321	DAEFRH	Mus musculu s
1	1.00	1	1	0	0	0	0	-0.002	t	99922	DAEFRHDS GY	Mus musculu s
1	1.00	1	1	0	0	0	0	-0.002	t	108382	GGVVIA	Mus musculu s
1	1.00	1	1	0	0	0	0	-0.002	t	108382	GGVVIA	Mus musculu s
1	1.00	1	1	0	0	0	0	-0.002	t	105748	EFRHDSGY EVHHQK	Mus musculu s
1	1.00	1	1	0	0	0	0	-0.002	t	103830	CHAVKVG NPSC	Oryctola gus cuniculu s

1	1.00	1	1	0	0	0	0	-0.002	t	115271	GQKGRGSRG	Oryctolagus cuniculus
1	1.00	1	1	0	0	0	0	-0.002	t	102510	HHQKLVFFAEDVGSNK	Mus musculus
1	1.00	1	1	0	0	0	0	-0.002	t	107744	APVDSRTYTRVADHI	Homo sapiens
1	1.00	1	1	0	0	0	0	-0.002	t	100378	PGSGFWSFGSEDGSGDSEN	Mus musculus
1	1.00	1	1	0	0	0	0	-0.002	t	100070	GLMVGGVVIA	Oryctolagus cuniculus
1	1.00	1	1	0	0	0	0	-0.002	t	106321	DAEFRH	Mus musculus
1	1.00	1	1	0	0	0	0	-0.002	t	99922	DAEFRHDSGY	Oryctolagus cuniculus
1	1.00	1	1	0	0	0	0	-0.002	t	100070	GLMVGGVVIA	Oryctolagus cuniculus
1	1.00	1	1	0	0	0	0	-0.002	t	107166	GPPGPQG	Mus musculus
1	1.00	1	1	0	0	0	0	-0.002	t	105562	VAVVRTPPKSPSSAK	Oryctolagus cuniculus
1	1.00	1	1	0	0	0	0	-0.002	t	102564	KKRPKPGGGWNT	Mus musculus
1	1.00	1	1	0	0	0	0	-0.002	t	102423	FEYQD	Mus musculus
1	1.00	1	1	0	0	0	0	-0.002	t	104906	MVGGVVIA	Mus musculus
1	1.00	1	1	0	0	0	0	-0.002	t	99134	DAEFRHDSGYEVHHQ	Mus musculus
1	1.00	1	1	0	0	0	0	-0.002	t	100992	KSLSRHDHIHHH	Ovis aries

1	1.00	1	1	0	0	0	0	-0.002	t	107242	SQTTANPS CPEGT	Rattus norvegic us
1	1.00	1	1	0	0.003	0	0	-0.002	t	105561	VAVVRTPP KSPSSAK	Mus musculu s
1	1.00	1	1	0	0.004	0	0	-0.002	t	100992	KSLSRHDH IHHH	Ovis aries
1	1.00	1	1	0	0	0	0	-0.002	t	110948	MVTSDAQ IFNKPYWL	Mus musculu s
1	1.00	1	1	0	0	0	0	-0.002	t	106092	RSGYSSPG SPGTPGSR SR	Mus musculu s
1	1.00	1	1	0	0	0	0	-0.002	t	79858	IEELQSNH GV	Mus musculu s
1	1.00	1	1	0	-0.008	0	0	-0.002	t	97231	CNTKCQTP	Mus musculu s
1	1.00	1	1	0	0	0	0	-0.002	t	93373	MDKVLNR EESL	Mus musculu s
1	1.00	1	1	0	0	0	0	-0.002	t	79806	EKPAKTYD DAAS	Mus musculu s
1	1.00	1	1	0	0	0	0	-0.002	t	74035	YGLFHKEK MIL	Mus musculu s
1	1.00	1	1	0	0	0	0	-0.002	t	79782	DNDGPVA AGEARPVP EEYLQ	Mus musculu s
1	1.00	1	1	0	0	0	0	-0.002	t	80102	YDDAASES SDDDDIDA LI	Mus musculu s
1	1.00	1	1	0	0	0	0	-0.002	t	71004	VSISPESDR PDLSTF	Mus musculu s
1	1.00	1	1	0	0	0	0	-0.002	t	65409	TNRNPQ	Mus musculu s

1	1.00	1	1	0	0	0	0	-0.002	t	68524	VFMVPQYGYL	Mus musculus
1	1.00	1	1	0	0	0	0	-0.002	t	96526	LAANNPA	Mus musculus
1	1.00	1	1	0	0	0	0	-0.002	t	94836	GSSK	Oryctolagus cuniculus
1	1.00	1	1	0	0	0	0	-0.002	t	74036	YGLFHKEKMLL	Mus musculus
1	1.00	1	1	0	0	0	0	-0.002	t	65409	TNRNPQ	Mus musculus
1	1.00	1	1	0	0	0	0	-0.002	t	63037	TAVSPTTLR	Mus musculus
1	1.00	1	1	0	0	0	0	-0.002	t	41232	MDIDPYKEFG	Mus musculus
1	1.00	1	1	0	0	0	0	-0.002	t	44498	NKGVNPDE	Mus musculus
1	1.00	1	1	0	0.01	0	0	-0.002	t	59967	SNYYSDNEIEQHTAKQPKL	Oryctolagus cuniculus
1	1.00	1	1	0	0	0	0	-0.002	t	46317	NTWTTCQSIAFPSK	Mus musculus
1	1.00	1	1	0	0	0	0	-0.002	t	41537	MFSGGGGPLSPGGKS	Mus musculus
1	1.00	1	1	0	0	0	0	-0.002	t	39164	LRPGPVSQPYHHWDTDK	Mus musculus
1	1.00	1	1	0	0	0	0	-0.002	t	48627	PNEPDDALAMRIIRI	Mus musculus
1	1.00	1	1	0	0	0	0	-0.002	t	39695	LSTFMESGEWVMK	Mus musculus

1	1.00	1	1	0	0	0	0	-0.002	t	48933	PPTAPPLLPHSNL	Homo sapiens
1	1.00	1	1	0	0	0	0	-0.002	t	56699	SAAFYYRV	Mus musculus
1	1.00	1	1	0	0	0	0	-0.002	t	43730	NEREDERTLTKEYEDIVLK	Oryctolagus cuniculus
1	1.00	1	1	0	0	0	0	-0.002	t	55799	RSAPRITFG	Homo sapiens
1	1.00	1	1	0	0	0	0	-0.002	t	51775	QNTRNIVPVSIVSR	Mus musculus
1	1.00	1	1	0	-0.004	0	0	-0.002	t	51233	QKPLK	Mus musculus
1	1.00	1	1	0	0	0	0	-0.002	t	55115	RPAPPKPTPQP	Mus musculus
1	1.00	1	1	0	0	0	0	-0.002	t	50502	QDPDNTDDNGPQDPDNTD	Mus musculus
1	1.00	1	1	0	0	0	0	-0.002	t	58388	SHLVEALYLVCGERG	Mus musculus
1	1.00	1	1	0	0	0	0	-0.002	t	51233	QKPLK	Mus musculus
1	1.00	1	1	0	0	0	0	-0.002	t	50222	QACKGKIQALCEN	Mus musculus
1	1.00	1	1	0	0	0	0	-0.002	t	51827	QPFPQPQLPYPQPQ	Mus musculus
1	1.00	1	1	0	0	0	0	-0.002	t	11463	EDVNGIRKPK	Mus musculus
1	1.00	1	1	0	0	0	0	-0.002	t	12982	ELDGSKNEWGWSKSK	Mus musculus

1	1.00	1	1	0	0	0	0	-0.002	t	16101	FHENWPS	Mus musculus
1	1.00	1	1	0	0	0	0	-0.002	t	23599	HCLGKWLGHPDKF	Mus musculus
1	1.00	1	1	0	0	0	0	-0.002	t	31105	KHFRETEV	Mus musculus
1	1.00	1	1	0	0	0	0	-0.002	t	11815	EENVEENVEENVEENV	Mus musculus
1	1.00	1	1	0	0	0	0	-0.002	t	18036	FTPFYYQSSRTR	Mus musculus
1	1.00	1	1	0	0	0	0	-0.002	t	12632	EKASVPGGGDMGGMDF	Mus musculus
1	1.00	1	1	0	-0.003	0	0	-0.002	t	5967	CADARMYGVLPWNAFPGKVC	Oryctolagus cuniculus
1	1.00	1	1	0	0	0	0	-0.002	t	5865	AYLSPIDLNQLAK	Oryctolagus cuniculus
1	1.00	1	1	0	0	0	0	-0.002	t	10227	DSSAHGTPSTPA	Mus musculus
1	1.00	1	1	0	0	0	0	-0.002	t	1433	AGAGGGAGGAGAGGGAGGAG	Oryctolagus cuniculus
1	1.00	1	1	0	0	0	0	-0.002	t	9723	DPPPPNPNDPPPPNPND	Mus musculus
1	1.00	1	1	0	0	0	0	-0.002	t	9524	DNPASTTNKDK	Mus musculus
1	1.00	1	1	0	0	0	0	-0.002	t	7491	DAEFRHDSGYEVHHQK	Homo sapiens
1	1.00	1	1	0	0	0	0	-0.002	t	9723	DPPPPNPNDPPPPNPND	Mus musculus
1	1.00	1	1	0	0	0	0	-0.002	t	2780	ALNGNGDPNNMDKAVKLY	Mus musculus

1	1.00	1	1	0	0	0	0	-0.002	t	7048	CSNSDKPKCGGS	Mus musculus
1	1.00	1	1	0	0	0	0	-0.002	t	7492	DAEFRHDSGYEVHHQKL	Mus musculus
1	1.00	1	1	0	0	0	0	-0.002	t	3274	ANETIYNTTLKYGDV	MD
1	1.00	1	1	0.03	0.03	0.03	0.03	0.03	t	115796	IQAFLLYQEP	Homo sapiens
1	1.00	1	1	0.01	0.01	0.01	0.01	0.01	t	115675	AFLLYQEPVL	Homo sapiens
1	1.00	1	1	0.01	0.01	0.006	0.007	0.01	t	86504	MSAAYDNIYT	Homo sapiens
1	1.00	1	1	0.04	0.04	0.04	0.04	0.04	t	21548	GNYD	Homo sapiens
1	1.00	1	1	0	0.002	0.008	0.001	-0.002	t	3820	APSQGGVNSPVNV	Homo sapiens
1	1.00	1	1	0.03	0.03	0.015	0.03	0.032	t	95850	SGTSGTSGTSGT	Homo sapiens
1	1.00	1	1	0	0	0	-0.002	0	t	71838	VVSTLP	Homo sapiens
1	1.00	1	1	0.03	0.03	0.03	0.03	0.03	t	135454	DYEDRY	Mus musculus
1	1.00	1	1	0.01	0.01	0.008	0.01	0.009	t	20052	GGTHSQWNKPSKP	Mus musculus
1	1.00	1	1	0	0.001	-0.006	0	-0.002	t	18952	GDDPAKAAFDSLQAS	Mus musculus
1	1.00	1	1	0.02	0.02	0.022	0.02	0.02	t	4701	ASQKIQLI	Mus musculus
1	1.00	1	1	0	0	-0.007	0	-0.002	t	106375	EDHAGTYGLGDRK	Mus musculus
1	1.00	1	1	0.02	0.018	0.017	0.021	0.022	t	5670	AWITEDCPVP	Homo sapiens
1	1.00	1	1	0.01	0.012	-0.003	0.007	0.01	t	115068	KEKVNELSKDIG	Homo sapiens

1	1.00	1	1	-0.03	-0.03	-0.03	-0.026	-0.03	t	75149	YNGANGAMKH	Homo sapiens
1	1.00	1	1	0.07	0.07	0.07	0.07	0.07	t	117547	KLEVQATDREENKQIALGTS	Homo sapiens
1	1.00	1	1	-0.01	-0.025	-0.01	-0.011	-0.01	t	60795	SRSERR	Marmota monax
1	1.00	1	1	-0.02	-0.02	-0.013	-0.02	-0.021	t	122477	LSLSLDLLTFRT	Homo sapiens
1	1.00	1	1	0.02	0.02	0.02	0.02	0.02	t	103516	QDPEGWGKSPGFGTT	Homo sapiens
1	1.00	1	1	0.01	0.01	0.01	0.01	0.01	t	156923	NLHTSF	Gallus gallus
1	1.00	1	1	0	0	-0.005	0	0	t	124925	YYVFFEEQEDEIIGF	Oryctolagus cuniculus
1	1.00	1	1	0	0	0	0.009	0.002	t	131116	TQSYPPPPAWRAASR	Homo sapiens
1	1.00	1	1	-0.01	-0.01	-0.015	-0.01	-0.01	t	59374	SLNTGWLAGLFY	Mus musculus
1	1.00	1	1	-0.01	-0.01	-0.008	-0.01	-0.01	t	125791	CSFWSKYIQTLK	Mus musculus
1	1.00	1	1	-0.02	-0.02	-0.035	-0.02	-0.017	t	64454	TISADSKKTKDLVFL	Mus musculus
1	1.00	1	1	0.02	0.022	0.018	0.017	0.018	t	14639	EVDDEALEKFDKAL	Homo sapiens
1	1.00	1	1	0.01	0.01	0.015	0.01	0.01	t	56888	SALYVGDLCG	Homo sapiens
1	1.00	1	1	0.01	0.01	0.015	0.01	0.009	t	49967	PVSLGGDVPNS	Mus musculus
1	1.00	1	1	0.01	0.013	0.015	0.01	0.009	t	49967	PVSLGGDVPNS	Mus musculus
1	1.00	1	1	0.01	0.01	0.006	0.01	0.01	t	72693	WKSKKN	Homo sapiens

1	1.00	1	1	0	0	0.002	0.001	-0.002	t	153251	AKAFSYWTPFDGKSLYSST	Mus musculus
1	1.00	1	1	-0.01	-0.014	-0.018	-0.013	-0.01	t	70795	VRPATTLLADG	Oryctolagus cuniculus
1	1.00	1	1	0.01	0.01	0.01	0.01	0.01	t	23193	GVTYEIDLTNKNAAKL	Homo sapiens
1	1.00	1	1	0.04	0.037	0.037	0.04	0.04	t	97741	VLDFSTAQQ	Oryctolagus cuniculus
1	1.00	1	1	0	0	-0.016	0	0.004	t	30611	KESQAYYDGRR	Mus musculus
1	1.00	1	1	-0.03	-0.03	-0.03	-0.03	-0.03	t	8918	DKLADYKRRM	Mus musculus
1	1.00	1	1	-0.03	-0.03	-0.03	-0.03	-0.03	t	8918	DKLADYKRRM	Mus musculus
1	1.00	1	1	0	-0.003	-0.011	0	0.002	t	144748	QAVNPNAGN	Oryctolagus cuniculus
1	1.00	1	1	0	-0.002	0.007	0.003	-0.003	t	44021	NGKVNNKVAY	Mus musculus
1	1.00	1	1	0.03	0.03	0.03	0.03	0.031	t	136648	SPLNNFYKTQLR	Mus musculus
1	1.00	1	1	0.03	0.026	0.03	0.03	0.031	t	136648	SPLNNFYKTQLR	Bos taurus
1	1.00	1	1	0.01	0.006	-0.002	0.009	0.01	t	3971	AQGQYSNTLLRIYRDNKSIN	Oryctolagus cuniculus
1	1.00	1	1	-0.02	-0.02	-0.032	-0.02	-0.02	t	120450	DQDLSKKHGQ	Mus musculus
1	1.00	1	1	0.05	0.05	0.05	0.054	0.05	t	115600	DHSYQEEAAMEL	Homo sapiens

1	1.00	1	1	0.02	0.022	0.02	0.021	0.02	t	98796	GGAVP	Homo sapiens
1	1.00	1	1	0.02	0.02	0.02	0.02	0.019	t	139494	ERGSAGH WTSESSVS	Homo sapiens
1	1.00	1	1	0	0	-0.001	0.003	0	t	48494	PLTASPPA TAPGPSAA NVSV	Homo sapiens
1	1.00	1	1	0.02	0.018	0.024	0.017	0.017	t	66833	TTSTGPCK TCTTPAQ	Mus musculu s
1	1.00	1	1	-0.03	-0.03	-0.03	-0.03	-0.03	t	55067	RNTNRRP	Homo sapiens
1	1.00	1	1	0.03	0.03	0.03	0.03	0.03	t	10041	DRRCQSQ LERANLRP	Homo sapiens
1	1.00	1	1	0.01	0.01	0.01	0.01	0.01	t	156425	QQEQQGS GNNVFSG F	Homo sapiens
1	1.00	1	1	0	0	0	0	0	t	140247	KAAESEIEK LTTK	Oryctola gus cuniculu s
1	1.00	1	1	0	0	0	0	0	t	140247	KAAESEIEK LTTK	Oryctola gus cuniculu s
1	1.00	1	1	0.01	0.013	-0.001	0.01	0.012	t	111733	RKATPTTS STSTTAK	Mus musculu s
1	1.00	1	1	-0.01	-0.014	-0.017	-0.017	-0.011	t	108131	SKDWSFY	Oryctola gus cuniculu s
1	1.00	1	1	0	0	0	0	0	t	109996	TRLSRTIGY TVK	Mus musculu s
1	1.00	1	1	0.01	0.008	0.01	0.009	0.011	t	41233	MDIDPYKE FGA	Mus musculu s
1	1.00	1	1	-0.02	-0.02	-0.026	-0.02	-0.02	t	91575	KIVYEKSQL APRKLY	Oryctola gus cuniculu s

1	1.00	1	1	-0.04	-0.038	-0.022	-0.039	-0.049	t	134679	GGSGPQRRGG	Homo sapiens
1	1.00	1	1	0	0.004	0	0.001	0	t	20283	GIGPEKAF	Homo sapiens
1	1.00	1	1	0	0	0	0	0	t	139090	IKRSNSTA	Oryctolagus cuniculus
1	1.00	1	1	-0.02	-0.023	-0.027	-0.02	-0.021	t	79277	KRDGYIV	Oryctolagus cuniculus
1	1.00	1	1	-0.02	-0.023	-0.027	-0.02	-0.021	t	79277	KRDGYIV	Oryctolagus cuniculus
1	1.00	1	1	-0.05	-0.049	-0.051	-0.047	-0.051	t	99138	DDWDNRHPLPNAPLV	Equus caballus
1	1.00	1	1	0	-0.002	0.002	-0.001	0	t	10770	DVVGVGRL	Mus musculus
1	1.00	1	1	0	0	-0.015	0	0.002	t	43222	NANADPN	Mus musculus
1	1.00	1	1	0	-0.002	0	-0.001	0	t	57977	SGAPTYSW	Mus musculus
1	1.00	1	1	-0.02	-0.02	-0.018	-0.02	-0.021	t	60762	SRPLIHFGSDYEDR	Mus musculus
1	1.00	1	1	-0.02	-0.015	-0.027	-0.013	-0.02	t	136634	SINASPGVVVDIAHSPAKK	Homo sapiens
1	1.00	1	1	0.02	0.02	0.02	0.02	0.02	t	79386	SCSPTVYLTSTKCP	Mus musculus
1	1.00	1	1	0	0	0	0	0	t	79086	SLYPGHVSGHRMAWDMMM	Homo sapiens
1	1.00	1	1	0.01	0.014	0.01	0.011	0.01	t	69277	VKNEEEIA	Homo sapiens

1	1.00	1	1	0	0.002	-0.008	-0.003	0.001	t	11615	EEFFFQGPEWRKEKE	Homo sapiens
1	1.00	1	1	-0.03	-0.03	-0.03	-0.03	-0.031	t	9794	DPVAALFFF	Mus musculus
1	1.00	1	1	-0.01	-0.01	-0.01	-0.014	-0.009	t	134257	VKSTP	Homo sapiens
1	1.00	1	1	0.03	0.033	0.031	0.03	0.028	t	117530	IEFRDSDAESSDGEDFIVEE	Mus musculus
1	1.00	1	1	0	-0.002	0	-0.001	-0.002	t	136686	TLHDMGIFSITSSDS	Mus musculus
1	1.00	1	1	0	-0.002	0	-0.001	-0.002	t	136315	GGGTGPADGTNATTI	Mus musculus
1	1.00	1	1	0	-0.002	0	-0.001	-0.002	t	136749	WMLRAAEEYSMNLGF	Mus musculus
1	1.00	1	1	0	-0.002	0	-0.001	-0.002	t	136749	WMLRAAEEYSMNLGF	Mus musculus
1	1.00	1	1	0	-0.002	0	-0.001	-0.002	t	128004	GMIEVQSNYGFVWDVSLKKV	Mus musculus
1	1.00	1	1	0	-0.002	0	-0.001	-0.002	t	130994	PDQRPYCWHYPPKPC	Mus musculus
1	1.00	1	1	0	-0.002	0	-0.001	-0.002	t	127984	FTNLKFKVRKITSSHRGN	Mus musculus
1	1.00	1	1	0	-0.002	0	-0.001	-0.002	t	127919	AFSLPDRSLSAIPLFDVSFE	Mus musculus
1	1.00	1	1	0	-0.002	0	-0.001	-0.002	t	127944	DLVNCNLNTNCVAVAFSLPD	Mus musculus
1	1.00	1	1	0	-0.002	0	-0.001	-0.002	t	110733	VGENVPDDLYIKGSG	Mus musculus

1	1.00	1	1	0	-0.004	0	-0.001	-0.002	t	111013	RTGYIPLG TRPPT	Oryctola gus cuniculu s
1	1.00	1	1	0	-0.002	0	-0.001	-0.002	t	75923	YSWKTWG	Mus musculu s
1	1.00	1	1	0	-0.002	0	-0.001	-0.002	t	67170	TVSTEQNV PDPQVGI	Mus musculu s
1	1.00	1	1	0	-0.004	0	-0.001	-0.002	t	73385	YALADASL KMADPNR FRGKD	Oryctola gus cuniculu s
1	1.00	1	1	0	-0.002	0	-0.001	-0.002	t	66539	TTASGKLIT	Mus musculu s
1	1.00	1	1	0	-0.004	0	-0.001	-0.002	t	73385	YALADASL KMADPNR FRGKD	Oryctola gus cuniculu s
1	1.00	1	1	0	-0.004	0	-0.001	-0.002	t	74430	YKAYVSYK KRKAQEK	Oryctola gus cuniculu s
1	1.00	1	1	0	-0.004	0	-0.001	-0.002	t	70437	VPPTQSKK KNKNET	Oryctola gus cuniculu s
1	1.00	1	1	0	-0.002	0	-0.001	-0.002	t	58416	SHSQLGPP VGGPP	Mus musculu s
1	1.00	1	1	0	-0.002	0	-0.001	-0.002	t	58418	SHSSESHR VKSAGPP	Mus musculu s
1	1.00	1	1	0	-0.004	0	-0.001	-0.002	t	36974	LKNKIFPKK KEDNQAV DT	Oryctola gus cuniculu s
1	1.00	1	1	0	-0.004	0	-0.001	-0.002	t	13460	ENDVLNQ ETEEEMEK	Oryctola gus cuniculu s
1	1.00	1	1	0	-0.004	0	-0.001	-0.002	t	20179	GHRMAW DMM	Oryctola gus cuniculu s
1	1.00	1	1	0	-0.004	0	-0.001	-0.002	t	12951	ELAPEDPE DS	Oryctola gus cuniculu s

1	1.00	1	1	0	-0.004	0	-0.001	-0.002	t	12951	ELAPEDPEDS	Oryctolagus cuniculus
1	1.00	1	1	0	-0.004	0	-0.001	-0.002	t	30349	KEEKEEKEEKEEKEKEKE	Oryctolagus cuniculus
1	1.00	1	1	0	-0.004	0	-0.001	-0.002	t	29899	KANGNGSGDNGDTTW	Oryctolagus cuniculus
1	1.00	1	1	0	-0.002	0	-0.001	-0.002	t	3245	ANDVDNSNPVVQAEQLNWL	Mus musculus
1	1.00	1	1	0	0.001	0	-0.001	-0.002	t	8355	DGAVQPDGG	Oryctolagus cuniculus
1	1.00	1	1	-0.01	-0.007	-0.007	-0.01	-0.012	t	24970	HTRVTGGVQG	Mus musculus
1	1.00	1	1	-0.02	-0.018	-0.02	-0.019	-0.02	t	10813	DWICTVLTDFKTWL	Homo sapiens
1	1.00	1	1	0	0	0.005	0.01	0.003	t	115785	HSYQE	Homo sapiens
1	1.00	1	1	0.02	0.022	0.027	0.023	0.02	t	56448	RVVVTPDKEILYEA	Homo sapiens
1	1.00	1	1	-0.02	-0.018	-0.027	-0.019	-0.018	t	138028	SAGALQLLVVGC	Homo sapiens
1	1.00	1	1	0.02	0.022	0.021	0.021	0.02	t	7778	DDGDKGSDVESYSSMPPLE	Homo sapiens
1	1.00	1	1	0.04	0.034	0.04	0.04	0.04	t	123184	FLTNLPEE	Oryctolagus cuniculus
1	1.00	1	1	-0.02	-0.02	-0.013	-0.02	-0.02	t	119450	FRKADKFPM	Homo sapiens
1	1.00	1	1	-0.02	-0.022	-0.027	-0.023	-0.018	t	65779	TPTAWKPN	Mus musculus

1	1.00	1	1	0	0	0	0	0	t	140914	VLGHDEAAYSKN	Oryctolagus cuniculus
1	1.00	1	1	-0.01	-0.008	-0.003	-0.007	-0.009	t	139202	QIWYERTLPFTF	Homo sapiens
1	1.00	1	1	-0.01	-0.006	-0.003	-0.007	-0.009	t	139202	QIWYERTLPFTF	Homo sapiens
1	1.00	1	1	0	-0.003	0.007	0.004	-0.002	t	130607	SHNLVSSAFPSA	Camelus dromedarius
1	1.00	1	1	0.03	0.03	0.018	0.03	0.033	t	114142	PQKSQRSQDEN	Rattus norvegicus
1	1.00	1	1	0.01	0.018	0.01	0.01	0.009	t	156896	KHTSLAS	Gallus gallus
1	1.00	1	1	-0.04	-0.044	-0.034	-0.037	-0.043	t	49015	PQIPPNWHIP	Oryctolagus cuniculus
1	1.00	1	1	0.03	0.032	0.027	0.031	0.03	t	32912	KPTTFMLKYDENGTITD	Homo sapiens
1	1.00	1	1	0.02	0.013	0.015	0.023	0.019	t	34115	KVLSPLQDNNDSGH	Homo sapiens
1	1.00	1	1	-0.03	-0.027	-0.03	-0.03	-0.032	t	135501	SQWNKP	Mus musculus
1	1.00	1	1	-0.02	-0.022	-0.019	-0.017	-0.02	t	41810	MKDTLRITYL	Mus musculus
1	1.00	1	1	-0.01	-0.008	-0.008	-0.009	-0.01	t	23689	HEHHGVYNQG	Homo sapiens
1	1.00	1	1	0	0.002	-0.006	0.003	0.002	t	133798	DKIRGEGMRYIIILD	Homo sapiens
1	1.00	1	1	-0.03	-0.03	-0.03	-0.027	-0.032	t	134198	PVGPAPGMRPPMGG	Oryctolagus cuniculus
1	1.00	1	1	0.02	0.02	0.02	0.02	0.02	t	53069	RAALIEEGQRIAEM	Homo sapiens

1	1.00	1	1	0	0	0	0	0	t	47494	PFGDSY	Homo sapiens
1	1.00	1	1	0.04	0.044	0.038	0.04	0.04	t	49510	PSSLVDSL	Equus caballus
1	1.00	1	1	0	0	0	0	0	t	132468	LYWFTLY	Homo sapiens
1	1.00	1	1	-0.04	-0.044	-0.04	-0.043	-0.042	t	143962	KIWKAPHVAKYFDDQ	Oryctolagus cuniculus
1	1.00	1	1	0.02	0.02	0.015	0.02	0.018	t	32818	KPPFSGMTGCGNT	Homo sapiens
1	1.00	1	1	0.01	0.006	0.007	0.01	0.01	t	100135	IEVDNEQPTTRAQK	Oryctolagus cuniculus
1	1.00	1	1	0.01	0.012	0.002	0.011	0.01	t	42999	MVTILLCCMTSCCSCLK	Homo sapiens
1	1.00	1	1	0.01	0.012	0.002	0.011	0.01	t	42999	MVTILLCCMTSCCSCLK	Homo sapiens
1	1.00	1	1	0	-0.003	0	0	0	t	135440	AFPAVT	Oryctolagus cuniculus
1	1.00	1	1	-0.02	-0.02	-0.02	-0.02	-0.02	t	136059	EGY	Rattus norvegicus
1	1.00	1	1	0.07	0.068	0.065	0.069	0.07	t	52313	QSESVEFTKDTQTGMS	Mus musculus
1	1.00	1	1	0.01	0.012	0.006	0.007	0.009	t	39742	LSVPDGFKVS	Homo sapiens
1	1.00	1	1	0.05	0.048	0.056	0.049	0.051	t	67967	VDGSDP	Mus musculus
1	1.00	1	1	-0.04	-0.038	-0.04	-0.033	-0.038	t	134764	PTPGPSRRGP	Homo sapiens
1	1.00	1	1	0.02	0.02	0.02	0.02	0.02	t	79344	QCDAFAVPVEGACM	Mus musculus
1	1.00	1	1	0.02	0.02	0.02	0.02	0.019	t	130925	KLLPEDDESRTYHTV	Homo sapiens

1	1.00	1	1	-0.01	-0.01	-0.01	-0.01	-0.01	t	140460	EKLKHSM	Mus musculus
1	1.00	1	1	-0.01	-0.01	-0.01	-0.01	-0.01	t	140460	EKLKHSM	Mus musculus
1	1.00	1	1	-0.01	-0.01	-0.01	-0.01	-0.01	t	111794	SPIAPPR	Homo sapiens
1	1.00	1	1	0.04	0.04	0.04	0.044	0.04	t	134020	CPEEPQLRMKNNEEAEDYDD	Homo sapiens
1	1.00	1	1	0.01	0.01	0.01	0.012	0.01	t	37021	LKSKIQGLLQQASK	Homo sapiens
1	1.00	1	1	0	0	-0.008	0	0.001	t	102942	YGCYCGKGG	Mus musculus
1	1.00	1	1	0	0	-0.006	0	0	t	158836	IHNDVEAWIDRYKYLP	Mus musculus
1	1.00	1	1	0	0	0	0.004	0	t	56918	SANNDAEIGNLI	Homo sapiens
1	1.00	1	1	0	0	0	0.004	0	t	5054	ATLVVNRIRGGF	Homo sapiens
1	1.00	1	1	-0.01	-0.01	-0.01	-0.006	-0.01	t	58859	SKIYQDAAGAAGGMPG	Homo sapiens
1	1.00	1	1	0.01	0.01	0.01	0.01	0.01	t	51483	QLPQGTTLPKGFYAE	Homo sapiens
1	1.00	1	1	-0.01	-0.018	-0.014	-0.01	-0.008	t	29160	ITYGACPRYVK	Mus musculus
1	1.00	1	1	-0.01	-0.018	-0.014	-0.01	-0.008	t	29160	ITYGACPRYVK	Mus musculus
1	1.00	1	1	-0.01	-0.018	-0.014	-0.01	-0.008	t	29160	ITYGACPRYVK	Mus musculus
1	1.00	1	1	0.03	0.032	0.03	0.031	0.03	t	116930	AYKLPQTDDST	Homo sapiens
1	1.00	1	1	0	0	0	0	-0.002	t	144820	YLEDWIKYNNQK	Mus musculus

1	1.00	1	1	0	0	0	0	-0.002	t	103147	EQKLISEEDLN	Mus musculus
1	1.00	1	1	0	0	0	0	-0.002	t	108202	VVSHFND	Mus musculus
1	1.00	1	1	0	0	0	0	-0.002	t	103372	LPGEEDLPG	Mus musculus
1	1.00	1	1	0.01	0.01	0.01	0.01	0.01	t	132103	TQKVSFSGQPQNITT	Homo sapiens
1	1.00	1	1	0.01	0.01	0.01	0.01	0.01	t	104128	MNKVYLP	Homo sapiens
1	1.00	1	1	0.01	0.01	0.01	0.01	0.01	t	79567	HSYTLMF	Oryctolagus cuniculus
1	1.00	1	1	0.01	0.01	0.006	0.01	0.01	t	47545	PFPQPQQQFP	Mus musculus
1	1.00	1	1	0	0.002	-0.009	0.003	0	t	70261	VPCSTCEGNLACLSLCQ	Ovis aries
1	1.00	1	1	0.04	0.04	0.04	0.04	0.04	t	135502	SSSQHL	Oryctolagus cuniculus
1	1.00	1	1	0.03	0.028	0.041	0.029	0.028	t	15383	FDFASGLD	Mus musculus
1	1.00	1	1	0.03	0.032	0.041	0.029	0.028	t	15383	FDFASGLD	MD
1	1.00	1	1	-0.01	-0.01	-0.012	-0.01	-0.01	t	48410	PLLSAGIFGAKPLQSLQ	Mus musculus
1	1.00	1	1	0.02	0.016	0.017	0.019	0.021	t	76577	YYQQTDEANK	Oryctolagus cuniculus
1	1.00	1	1	-0.02	-0.023	-0.021	-0.02	-0.022	t	71199	VSVNDPFIDLEYM	Oryctolagus cuniculus
1	1.00	1	1	0	0	0	0	0	t	1396	AFSKIRDF	Mus musculus

1	1.00	1	1	0.02	0.022	0.004	0.026	0.027	t	134684	GSLPQKSQRDEN	Rattus norvegicus
1	1.00	1	1	0.02	0.024	0.011	0.017	0.018	t	113755	QGQQPGQGQQPGQG	Homo sapiens
1	1.00	1	1	0	0	0.001	0	0.002	t	29703	IYTINDKILS	Mus musculus
1	1.00	1	1	-0.02	-0.02	-0.02	-0.024	-0.019	t	134050	FKPPKY	Homo sapiens
1	1.00	1	1	0.02	0.024	0.02	0.021	0.02	t	12931	ELADALLE	Homo sapiens
1	1.00	1	1	0.01	0.01	0.01	0.01	0.009	t	38304	LNPKTNKWEDK	Homo sapiens
1	1.00	1	1	0.02	0.017	0.012	0.02	0.02	t	84229	KEEGGKETKDADVDRR	Oryctolagus cuniculus
1	1.00	1	1	0.01	0.011	0.009	0.013	0.009	t	99285	GLGVPRERQMEPAFD	Equus caballus
1	1.00	1	1	0	0	0	0	0	t	141979	PKKRVVPNFERKG	Mus musculus
1	1.00	1	1	0	-0.004	0.007	0	0	t	10853	DYAWDQTHQ	Mus musculus
1	1.00	1	1	0	-0.001	-0.005	-0.003	0	t	60319	SPVMGVIGFSFFVK	Myodes glareolus
1	1.00	1	1	-0.01	-0.01	-0.01	-0.01	-0.012	t	153378	EIDLKLEPGTRV	Mus musculus
1	1.00	1	1	0.02	0.02	0.02	0.02	0.02	t	132526	PGSHFCGGTLIHPSF	Homo sapiens
1	1.00	1	1	0.02	0.02	0.02	0.02	0.02	t	132526	PGSHFCGGTLIHPSF	Homo sapiens
1	1.00	1	1	0.02	0.02	0.02	0.02	0.02	t	7515	DAGDSGYLT	Mus musculus

1	1.00	1	1	0	0	0	0	0	t	120448	DPEFLNLIKS	Mus musculus
1	1.00	1	1	0.01	0.01	0.009	0.01	0.01	t	6455	CITQYERESQAYY	Mus musculus
1	1.00	1	1	-0.01	-0.008	-0.007	-0.009	-0.01	t	55678	RRPQDVKF	Homo sapiens
1	1.00	1	1	0.01	0.008	-0.002	0.01	0.011	t	21518	GNSIIHYTNINYYKDAASNS	Homo sapiens
1	1.00	1	1	-0.02	-0.02	-0.022	-0.024	-0.02	t	39368	LSGIQLFDRI	Homo sapiens
1	1.00	1	1	0.01	0.01	0.01	0.01	0.01	t	382	AAPYIEQAQ	Homo sapiens
1	1.00	1	1	0.02	0.019	0.021	0.023	0.02	t	7084	CSSLEKTKHRLQNEIEDL	Homo sapiens
1	1.00	1	1	0	0	-0.003	0	0	t	98974	QTMKGLDIQK	Homo sapiens
1	1.00	1	1	0.05	0.05	0.05	0.05	0.05	t	139619	TGSSTGGRQGSHHE	Homo sapiens
1	1.00	1	1	0.01	0.006	0.005	0.009	0.01	t	97732	VGSAAPAFA	Oryctolagus cuniculus
1	1.00	1	1	0.01	0.006	0.005	0.009	0.01	t	97732	VGSAAPAFA	Oryctolagus cuniculus
1	1.00	1	1	-0.04	-0.042	-0.035	-0.041	-0.04	t	53924	RGLYFPAG	Mus musculus
1	1.00	1	1	-0.01	-0.01	-0.01	-0.01	-0.01	t	56715	SAASCNGFLYFVIF	Homo sapiens
1	1.00	1	1	-0.01	-0.01	-0.01	-0.01	-0.011	t	33794	KTNMKHM	Mus musculus
1	1.00	1	1	0	0	0.006	0	-0.002	t	18955	GDDPLLGNQSLL	Mus musculus
1	1.00	1	1	0.05	0.05	0.05	0.05	0.05	t	104950	QGERSGP	Homo sapiens

1	1.00	1	1	-0.01	-0.009	-0.021	-0.011	-0.01	t	53342	RDFSIRES	Equus caballus
1	1.00	1	1	0.01	0.01	0.006	0.01	0.01	t	51922	QPQQPFPQPQ	Mus musculus
1	1.00	1	1	0.01	0.01	0.006	0.01	0.01	t	51922	QPQQPFPQPQ	Mus musculus
1	1.00	1	1	-0.03	-0.028	-0.031	-0.029	-0.03	t	71262	VTCAMF	Homo sapiens
1	1.00	1	1	-0.02	-0.018	-0.021	-0.019	-0.021	t	40837	LYLGAM	Homo sapiens
1	1.00	1	1	0	0.002	-0.01	0.001	0	t	10581	DVENVDGLSLWEMYK	Homo sapiens
1	1.00	1	1	-0.02	-0.022	-0.026	-0.023	-0.02	t	50255	QAGDQSYFLR	Mus musculus
1	1.00	1	1	0.01	0.012	0.01	0.013	0.012	t	18031	FTPDPYQVPF	Homo sapiens
1	1.00	1	1	-0.02	-0.017	-0.027	-0.02	-0.02	t	33795	KTNMKHMAGA	Mus musculus
1	1.00	1	1	0	-0.003	0	0	0	t	15205	FAKY	Mus musculus
1	1.00	1	1	0	0	0	0	0	t	15205	FAKY	Mus musculus
1	1.00	1	1	0.02	0.02	0.025	0.02	0.018	t	117600	PKKAK	Homo sapiens
1	1.00	1	1	0	0	0	0	0.002	t	65555	TPEMDIPGQV	Mus musculus
1	1.00	1	1	0.05	0.053	0.053	0.05	0.05	t	78938	CAPGQNLCY	Mus musculus

1	1.00	1	1	0.02	0.02	0.02	0.02	0.02	t	136827	DFGSVGG VL	Mus musculu s
1	1.00	1	1	-0.02	-0.012	-0.02	-0.017	-0.02	t	110954	NNKYTVTL KATP	Homo sapiens
1	1.00	1	1	-0.02	-0.02	-0.033	-0.017	-0.017	t	141646	FSKWRKN HMRQKSN K	Homo sapiens
1	1.00	1	1	0.01	0.01	0.01	0.01	0.009	t	138892	REQAPNL VY	Mus musculu s
1	1.00	1	1	0	-0.002	-0.007	0	0	t	45495	NPTLLGEA	Oryctola gus cuniculu s
1	1.00	1	1	0.01	0.01	-0.002	0.004	0.009	t	27725	INQDPDKI LTY	Mus musculu s
1	1.00	1	1	-0.01	-0.009	-0.01	-0.01	-0.012	t	14397	ETHPLYQ	Mus musculu s
1	1.00	1	1	0.01	0.008	0.008	0.009	0.01	t	97431	KTFSMGA K	Mus musculu s
1	1.00	1	1	0.01	0.008	0.008	0.009	0.01	t	97431	KTFSMGA K	Mus musculu s
1	1.00	1	1	0.01	0.022	0.01	0.01	0.008	t	136363	IDPLMFKY WSNM	Gallus gallus
1	1.00	1	1	0.01	0.018	0.01	0.01	0.008	t	136363	IDPLMFKY WSNM	Gallus gallus
1	1.00	1	1	-0.01	-0.002	-0.01	-0.01	-0.012	t	156938	PPEKIHV	Gallus gallus
1	1.00	1	1	-0.01	-0.01	-0.01	-0.01	-0.012	t	47544	PFPQPQQ PFC	Mus musculu s
1	1.00	1	1	0.04	0.04	0.04	0.04	0.04	t	79375	RNYTLT	Mus musculu s
1	1.00	1	1	0	-0.002	-0.007	-0.003	-0.001	t	86999	NQKNVRT LEEWE	Mus musculu s

1	1.00	1	1	0.02	0.02	0.02	0.02	0.02	t	104226	RPQDVKFPGGGQIVGGVYLL	Homo sapiens
1	1.00	1	1	-0.01	-0.01	-0.01	-0.01	-0.01	t	133116	GPSRRGPSLG	Homo sapiens
1	1.00	1	1	0.02	0.02	0.019	0.02	0.02	t	55473	RQQQQQGLRGEEM	Homo sapiens
1	1.00	1	1	0.02	0.02	0.02	0.02	0.021	t	137407	GPEYWDGETRKVKA	Oryctolagus cuniculus
1	1.00	1	1	0.01	0.014	0.01	0.011	0.008	t	23246	GWGLHQPPPQIFLKILPQSG	Homo sapiens
1	1.00	1	1	-0.01	-0.01	-0.01	-0.01	-0.01	t	15981	FGGETFTPDWMMEV	Mus musculus
1	1.00	1	1	-0.03	-0.027	-0.03	-0.03	-0.031	t	105859	HARGNYDAAKR	Mus musculus
1	1.00	1	1	-0.05	-0.052	-0.044	-0.049	-0.049	t	79042	PKSKPFA	Mus musculus
1	1.00	1	1	0	0	0	0	-0.001	t	134411	IPP	Mus musculus
1	1.00	1	1	0	-0.003	0.013	0	-0.001	t	38747	LPVCPL	Oryctolagus cuniculus
1	1.00	1	1	0	-0.004	0	-0.001	-0.001	t	7807	DDKDPQFKDNVILLNK	Oryctolagus cuniculus
1	1.00	1	1	-0.03	-0.03	-0.028	-0.03	-0.031	t	1522	AGGGHNGVFDFPDSG	Homo sapiens
1	1.00	1	1	-0.03	-0.03	-0.028	-0.03	-0.031	t	1522	AGGGHNGVFDFPDSG	Homo sapiens

1	1.00	1	1	0	0.004	0	0	0	t	25645	IDPRGKMV	MD
1	1.00	1	1	-0.03	-0.03	-0.03	-0.03	-0.03	t	126926	RGWNPGFLYNGFP	Oryctolagus cuniculus
1	1.00	1	1	-0.02	-0.023	-0.028	-0.02	-0.02	t	27244	ILPGGGTALIRC	Oryctolagus cuniculus
1	1.00	1	1	0.02	0.02	0.017	0.03	0.022	t	58022	SGETTTAAGTTASPGAASGP	Homo sapiens
1	1.00	1	1	0.03	0.03	0.03	0.038	0.032	t	110909	IVTFCCKCDSTLRLCVQST	Mus musculus
1	1.00	1	1	-0.04	-0.04	-0.044	-0.04	-0.04	t	48864	PPPPPPPPPPPPPPPPPPPP	Homo sapiens
1	1.00	1	1	0.01	0.01	0.019	0.01	0.01	t	20793	GLDFNE	Homo sapiens
1	1.00	1	1	0	0	0	0	-0.002	t	41014	MADNGTITVEELKQL	Homo sapiens
1	1.00	1	1	0	-0.002	0.005	0.003	0	t	115260	GAEGQRPGFGYGG	Mus musculus
1	1.00	1	1	-0.01	-0.01	-0.01	-0.01	-0.009	t	115266	GLFQINNKIW	Homo sapiens
1	1.00	1	1	-0.02	-0.014	-0.02	-0.021	-0.02	t	131294	DWGLPIPSPSP	Homo sapiens
1	1.00	1	1	0.03	0.028	0.03	0.029	0.03	t	116947	DIDYSDYSTR	Mus musculus
1	1.00	1	1	0.02	0.02	0.02	0.02	0.021	t	9609	DPDI	Mus musculus
1	1.00	1	1	-0.03	-0.026	-0.036	-0.03	-0.028	t	123438	LKMWSNFLG	Ovis aries

1	1.00	1	1	0.02	0.024	0.011	0.017	0.018	t	113754	QGQQPGQGQQGQQP	Homo sapiens
1	1.00	1	1	0	0	0	0	0	t	98731	DITNPINLRE	Homo sapiens
1	1.00	1	1	0.03	0.028	0.023	0.023	0.03	t	108131	SKDWSFY	Mus musculus
1	1.00	1	1	0.03	0.028	0.023	0.023	0.03	t	108131	SKDWSFY	Mus musculus
1	1.00	1	1	0.03	0.028	0.023	0.023	0.03	t	108131	SKDWSFY	Mus musculus
1	1.00	1	1	0	0.004	0	0.007	0.001	t	131698	TGVDVSMNTHLKAVKMTLKN	Homo sapiens
1	1.00	1	1	0.01	0.013	-0.004	0.01	0.01	t	94548	HYTQMVWGKTKEIGCGSIKY	Mus musculus
1	1.00	1	1	0.01	-0.004	0.002	0.009	0.01	t	57908	SFSPNNTN	Macaca mulatta
1	1.00	1	1	0	0.004	0	0.001	0	t	595	ACSFSSIP	Homo sapiens
1	1.00	1	1	0	0	0	0	0	t	32783	KPLDKFGNIYDYHYEH	Mus musculus
1	1.00	1	1	0	0	0	0	0	t	32783	KPLDKFGNIYDYHYEH	Mus musculus
1	1.00	1	1	0.02	0.02	0.02	0.022	0.02	t	135991	SEKKISSIQSIV	Homo sapiens
1	1.00	1	1	-0.05	-0.048	-0.05	-0.043	-0.048	t	118361	FRFLALKLYFSFTRP	Homo sapiens
1	1.00	1	1	0	0.004	-0.004	0.001	0	t	18764	GASDLRSS	Homo sapiens

1	1.00	1	1	0.02	0.022	0.018	0.021	0.022	t	28505	ISGDLKTQIDQVEST	Homo sapiens
1	1.00	1	1	0.03	0.035	0.039	0.031	0.03	t	65860	TQGSNW	Homo sapiens
1	1.00	1	1	0	0.004	-0.001	0.007	0.001	t	122732	DRKASPPSGLWSPAY	Homo sapiens
1	1.00	1	1	0	0.002	-0.011	-0.001	0	t	13248	ELRGLAQALIDQKVK	Homo sapiens
1	1.00	1	1	0.01	0.014	-0.007	0.01	0.011	t	51286	QLAKDKVNE	MD
1	1.00	1	1	0.01	0.01	0.012	0.011	0.014	t	45591	NQMIITM	Mus musculus
1	1.00	1	1	0.01	0.012	0.01	0.011	0.01	t	21466	GNLLFT	Homo sapiens
1	1.00	1	1	-0.01	-0.006	-0.014	-0.01	-0.01	t	71182	VSVAFGNPGDTPVTG	Capra hircus
1	1.00	1	1	-0.01	-0.003	-0.014	-0.01	-0.01	t	71182	VSVAFGNPGDTPVTG	Capra hircus
1	1.00	1	1	0.01	0.01	0.017	0.01	0.009	t	105890	IVYVGDGVC	Mus musculus
1	1.00	1	1	0.02	0.018	0.02	0.02	0.02	t	118023	MRPPMGPPIG	Mus musculus
1	1.00	1	1	0.02	0.023	0.021	0.021	0.018	t	99227	ERQMEPAFDQFRQTY	Equus caballus
1	1.00	1	1	0.04	0.04	0.04	0.04	0.04	t	79547	FHQMPDA	Oryctolagus cuniculus
1	1.00	1	1	0.02	0.02	0.02	0.02	0.02	t	119583	GNVFSCSV	Homo sapiens

1	1.00	1	1	0.01	0.01	0.01	0.01	0.01	t	22123	GRLGTYRYVSSGER	Homo sapiens
1	1.00	1	1	-0.03	-0.03	-0.033	-0.03	-0.03	t	50741	QFAPSASAFFGMSRIGM	Homo sapiens
1	1.00	1	1	0.02	0.024	0.024	0.021	0.022	t	24703	HRQWFL	Homo sapiens
1	1.00	1	1	0.08	0.083	0.08	0.08	0.079	t	113679	PEVPEVEDE	Mus musculus
1	1.00	1	1	0.03	0.03	0.035	0.03	0.03	t	53032	QYSPGQ	Homo sapiens
1	1.00	1	1	0.01	0.01	0.01	0.01	0.01	t	156899	KLGDPLA	Oryctolagus cuniculus
1	1.00	1	1	0.01	0.007	0.01	0.01	0.01	t	156899	KLGDPLA	Oryctolagus cuniculus
1	1.00	1	1	0	0	-0.006	0	0	t	1379	AFQNRLESIK	Homo sapiens
1	1.00	1	1	-0.03	-0.028	-0.036	-0.023	-0.028	t	38208	LNENLLRFFVAPFPQVFGKE	Homo sapiens
1	1.00	1	1	-0.01	-0.01	-0.01	-0.01	-0.01	t	153393	GPDAPRIPLSKM	Mus musculus
1	1.00	1	1	-0.03	-0.032	-0.042	-0.033	-0.032	t	89067	SRTRTTSVGDKQVASKV	Mus musculus
1	1.00	1	1	0.03	0.032	0.02	0.027	0.028	t	63287	TEAEDVIPEGWKADTSYESK	Homo sapiens
1	1.00	1	1	-0.08	-0.078	-0.087	-0.081	-0.082	t	131052	RRRMMMQSGR	Homo sapiens
1	1.00	1	1	0	-0.006	0	0	0	t	123226	GGGATPVV	Oryctolagus cuniculus

1	1.00	1	1	0.01	-0.002	0.01	0.013	0.01	t	156980	YTLGHFHGQPQL	Bos taurus
1	1.00	1	1	0.01	0.012	0.008	0.011	0.009	t	53355	RDGQLTIKAERTEQKDFDGR	Homo sapiens
1	1.00	1	1	0.04	0.042	0.04	0.033	0.04	t	123441	LLEYLGEEY	Ovis aries
1	1.00	1	1	0.01	0.012	0.01	0.011	0.01	t	58076	SGHRMAWDM	Homo sapiens
1	1.00	1	1	0.02	0.02	0.02	0.02	0.019	t	118762	CYGSFAKPTN	Mus musculus
1	1.00	1	1	0	0.002	0	0.003	0	t	108874	DPISGHVSLF	Homo sapiens
1	1.00	1	1	0.03	0.03	0.031	0.033	0.03	t	31133	KHLYVSMPTAQSTM	Homo sapiens
1	1.00	1	1	0.01	0.01	0.01	0.01	0.011	t	115304	KCEVFRELKD	Homo sapiens
1	1.00	1	1	0.05	0.05	0.05	0.05	0.05	t	115605	GDEVELPCRISP	Homo sapiens
1	1.00	1	1	0.05	0.05	0.05	0.05	0.05	t	115605	GDEVELPCRISP	Homo sapiens
1	1.00	1	1	0.03	0.03	0.03	0.03	0.03	t	112190	KVTMTCK	Homo sapiens
1	1.00	1	1	0.01	0.008	0.009	0.013	0.01	t	106515	HLPSFLRPDPPL	Mus musculus
1	1.00	1	1	0.01	0.012	0.015	0.013	0.011	t	134031	DEYDEVAMP	Homo sapiens
1	1.00	1	1	0.01	0.01	0.01	0.01	0.01	t	108036	MSVPQTTYSKITELI	Homo sapiens
1	1.00	1	1	-0.01	-0.008	-0.006	-0.009	-0.011	t	35994	LFTGHL	Homo sapiens
1	1.00	1	1	-0.01	-0.01	-0.01	-0.01	-0.01	t	132933	WGQNSFP	Homo sapiens
1	1.00	1	1	0.01	0.013	0.009	0.01	0.008	t	4528	ASLKDALLKYIYDNRGTLI	Equus caballus

1	1.00	1	1	0.01	0.018	0.009	0.01	0.008	t	4528	ASLKDALLKYIYDNRGTLI	Gallus gallus
1	1.00	1	1	-0.02	-0.018	-0.02	-0.019	-0.02	t	51575	QLVNTNGSWHIN	Homo sapiens
1	1.00	1	1	-0.02	-0.018	-0.02	-0.019	-0.02	t	51575	QLVNTNGSWHIN	Homo sapiens
1	1.00	1	1	0.03	0.028	0.016	0.023	0.032	t	120951	SLDPEFLNLI	Mus musculus
1	1.00	1	1	-0.02	-0.023	-0.024	-0.02	-0.021	t	122734	DSVPKWDRLPC	Oryctolagus cuniculus
1	1.00	1	1	0.02	0.02	0.015	0.02	0.02	t	95263	ATYHLYNSQD	Oryctolagus cuniculus
1	1.00	1	1	0.02	0.02	0.015	0.02	0.02	t	95263	ATYHLYNSQD	Homo sapiens
1	1.00	1	1	0.02	0.018	0.02	0.019	0.02	t	23983	HHDSLESRRLREEED	Mus musculus
1	1.00	1	1	0	0.002	-0.003	-0.003	-0.002	t	111822	TEHNATILFLKSDM	Homo sapiens
1	1.00	1	1	-0.03	-0.026	-0.013	-0.023	-0.033	t	117449	CVP	Homo sapiens
1	1.00	1	1	-0.05	-0.046	-0.05	-0.049	-0.05	t	66137	TRYNDALSHDRL	Homo sapiens
1	1.00	1	1	-0.01	-0.014	-0.014	-0.011	-0.01	t	52472	QTAPKAPTEA	Oryctolagus cuniculus
1	1.00	1	1	0.01	0.012	0.01	0.011	0.009	t	19636	GFTTNEERYNVFAE	Homo sapiens
1	1.00	1	1	0.01	0.012	0.01	0.011	0.009	t	19636	GFTTNEERYNVFAE	Homo sapiens

1	1.00	1	1	0	0.003	-0.002	0	0	t	49270	PRVRGLY	Mus musculus
1	1.00	1	1	0.02	0.022	0.019	0.023	0.02	t	1497	AGEPYVIEQGMMDPAK	Homo sapiens
1	1.00	1	1	0	0.002	0.005	0.001	0	t	109908	SFFLYSK	Homo sapiens
1	1.00	1	1	0	0.002	0	0.001	0	t	117103	TYQDFSTRPRSF	Homo sapiens
1	1.00	1	1	0.02	0.02	0.02	0.02	0.02	t	142381	AAEFATEVKKAT	Homo sapiens
1	1.00	1	1	0.01	0.008	0.01	0.009	0.009	t	42697	MSTNPKPQRKTKRNTN	Mus musculus
1	1.00	1	1	-0.01	-0.012	-0.005	-0.011	-0.012	t	27678	INKVVRFD	Mus musculus
1	1.00	1	1	0.02	0.02	0.02	0.02	0.021	t	115277	GYDTQAIVQN	Homo sapiens
1	1.00	1	1	-0.04	-0.037	-0.04	-0.04	-0.039	t	134017	CFHQGKEYAPG	Mus musculus
1	1.00	1	1	-0.01	-0.006	-0.01	-0.007	-0.011	t	110949	NAEKVLEKLGYA	Homo sapiens
1	1.00	1	1	0.01	0.01	0.01	0.01	0.01	t	156876	GLHANLQ	Gallus gallus
1	1.00	1	1	-0.01	-0.007	-0.01	-0.01	-0.012	t	140839	DERGTPEYNIALGQRRADAV	Mus musculus
1	1.00	1	1	-0.01	-0.01	-0.01	-0.01	-0.012	t	131849	DPYVTPGSVINNLSI	Homo sapiens
1	1.00	1	1	0.04	0.04	0.04	0.04	0.04	t	108785	APGPAGEE	Rattus norvegicus
1	1.00	1	1	0.04	0.04	0.04	0.04	0.04	t	103105	DGYPIINYEYAIVNN	Homo sapiens

1	1.00	1	1	0	-0.004	0.004	0	0	t	21841	GPYAGPM ERQKPLK	Bos taurus
1	1.00	1	1	0.01	0.012	0.01	0.013	0.01	t	131628	RGYDSYA	Homo sapiens
1	1.00	1	1	-0.01	-0.012	-0.004	-0.007	-0.009	t	56942	SAPSSKN	Homo sapiens
1	1.00	1	1	-0.01	-0.008	-0.004	-0.007	-0.009	t	56942	SAPSSKN	Homo sapiens
1	1.00	1	1	-0.04	-0.039	-0.051	-0.04	-0.04	t	44002	NGIRRPKH LYVSMP	Myodes glareolu s
1	1.00	1	1	0	0	0	0.004	0	t	136522	NDFFTYHI RHGEVHC GTNVR	Homo sapiens
1	1.00	1	1	0.01	0.01	0.01	0.01	0.01	t	111552	MKVSNSK	Homo sapiens
1	1.00	1	1	-0.01	-0.01	-0.017	-0.01	-0.01	t	40453	LVLNRLKV GLQVVAV KAPGF	Mus musculu s
1	1.00	1	1	0.01	0.014	0.012	0.013	0.012	t	74449	YKFEQPTL TFGAGVN	Homo sapiens
1	1.00	1	1	0.03	0.032	0.025	0.031	0.03	t	22223	GRTWAQP GYPWP	Homo sapiens
1	1.00	1	1	0	0	-0.025	-0.01	0.002	t	69261	VKLQCV	Homo sapiens
1	1.00	1	1	0.01	0.01	0.01	0.01	0.01	t	44555	NKNDNKN D	Mus musculu s
1	1.00	1	1	0.01	0.007	0.01	0.01	0.01	t	44555	NKNDNKN D	Oryctola gus cuniculu s
1	1.00	1	1	0.01	0.01	0.01	0.01	0.01	t	44555	NKNDNKN D	Mus musculu s
1	1.00	1	1	0.01	0.007	0.01	0.01	0.01	t	44555	NKNDNKN D	Oryctola gus cuniculu s

1	1.00	1	1	0.01	0.01	0.01	0.01	0.01	t	11814	EENVEENVEENV	Oryctolagus cuniculus
1	1.00	1	1	0.01	0.01	0.01	0.01	0.01	t	11814	EENVEENVEENV	Oryctolagus cuniculus
1	1.00	1	1	0.01	0.01	0.01	0.01	0.01	t	11814	EENVEENVEENV	Oryctolagus cuniculus
1	1.00	1	1	-0.02	-0.02	-0.02	-0.02	-0.02	t	5674	AWLVHR	Homo sapiens
1	1.00	1	1	0.01	0.016	0.007	0.01	0.01	t	49877	PVGSQGIRLSWEVQ	Ovis aries
1	1.00	1	1	0.02	0.008	0.02	0.023	0.02	t	156872	FSKSSSWESWLP	Bos taurus
1	1.00	1	1	0	-0.006	0	0	0	t	123225	GGATPVVG	Oryctolagus cuniculus
1	1.00	1	1	0	0	0	0	0	t	153867	QLLRREIIKNT	Cavia porcellus
1	1.00	1	1	0	0	0	0	-0.002	t	118185	TSQFTGLKC	Homo sapiens
1	1.00	1	1	0.01	0.01	-0.001	0.01	0.011	t	21807	GPTEESVERAMGRVA	Oryctolagus cuniculus
1	1.00	1	1	0	0	-0.005	0	0.002	t	3880	APTYSWGA	Mus musculus
1	1.00	1	1	0.01	0.01	0.01	0.014	0.01	t	28912	ITGHRMAWDMMMNWSPTT	Homo sapiens
1	1.00	1	1	0.02	0.02	0.02	0.02	0.02	t	4884	ATALDTSN	Oryctolagus cuniculus
1	1.00	1	1	-0.01	-0.01	-0.003	-0.001	-0.01	t	119477	KKTKGLVTT	Homo sapiens
1	1.00	1	1	0.01	0.012	0.015	0.01	0.008	t	94641	PESWHTRWDE	Homo sapiens

1	1.00	1	1	0	0	0	0	-0.002	t	49297	PSAPPLPHVVDLPQLGPRR	Cavia porcellus
1	1.00	1	1	0.01	0.01	0.01	0.01	0.009	t	146624	WNWFDITN	Homo sapiens
1	1.00	1	1	0.05	0.05	0.05	0.05	0.05	t	78562	VYASPNTTGLLNDIL	Mus musculus
1	1.00	1	1	0	0	0.001	0	-0.002	t	137942	PGSEYT	Oryctolagus cuniculus
1	1.00	1	1	0.01	0.01	0.01	0.01	0.01	t	132933	WGQNSFP	Homo sapiens
1	1.00	1	1	0.03	0.033	0.029	0.03	0.029	t	133737	VTPYFSYPVA	Mus musculus
1	1.00	1	1	0.02	0.018	0.023	0.019	0.018	t	51262	QKTTS	Mus musculus
1	1.00	1	1	0	-0.003	0	-0.001	-0.002	t	7491	DAEFRHDSGYEVHHQK	Oryctolagus cuniculus
1	1.00	1	1	0.04	0.038	0.032	0.037	0.04	t	70070	VNGENLVGDDVVLAT	Mus musculus
1	1.00	1	1	0.04	0.038	0.032	0.037	0.04	t	70070	VNGENLVGDDVVLAT	Mus musculus
1	1.00	1	1	0.04	0.038	0.032	0.037	0.04	t	70070	VNGENLVGDDVVLAT	Mus musculus
1	1.00	1	1	0.04	0.038	0.032	0.037	0.04	t	70070	VNGENLVGDDVVLAT	Mus musculus
1	1.00	1	1	0.01	0.006	0.004	0.007	0.008	t	74436	YKDGTLTPLSTKDLTTY	Oryctolagus cuniculus
1	1.00	1	1	0	-0.002	0	0.003	0	t	69271	VKMAETCPIFYDVF	Mus musculus
1	1.00	1	1	0	-0.002	0	0.003	0	t	31580	KKIQDCYVENGLIS	Mus musculus

1	1.00	1	1	0	-0.002	0	0.003	0	t	12408	EICPAVKR DVDLFLTG	Mus musculu s
1	1.00	1	1	-0.02	-0.02	-0.02	-0.02	-0.021	t	151729	RYSPSLNT RL	Mus musculu s
1	1.00	1	1	0.01	0.01	0.01	0.01	0.009	t	153836	HGPKATL QDIVLHLE P	Cavia porcellu s
1	1.00	1	1	-0.03	-0.03	-0.023	-0.03	-0.033	t	30261	KDTLRITYL T	Mus musculu s
1	1.00	1	1	-0.02	-0.023	-0.015	-0.02	-0.022	t	7143	CTGDARH C	Oryctola gus cuniculu s
1	1.00	1	1	-0.03	-0.032	-0.025	-0.033	-0.033	t	109386	KATTVR	Mus musculu s
1	1.00	1	1	-0.02	-0.018	-0.021	-0.019	-0.02	t	63043	TAWDFG	Homo sapiens
1	1.00	1	1	0.05	0.048	0.047	0.047	0.051	t	94606	LTGGFYEP L	Mus musculu s
1	1.00	1	1	0	-0.004	-0.015	-0.007	-0.001	t	103986	IHVLNSES GSAGQMV QD	Oryctola gus cuniculu s
1	1.00	1	1	0	0.002	-0.008	0.003	0	t	110957	PAAVTLAE ADRT	Homo sapiens
1	1.00	1	1	-0.01	0.002	-0.013	-0.01	-0.01	t	68023	VDMIAGA HWG	Pan troglody tes
1	1.00	1	1	0.02	0.018	0.022	0.019	0.02	t	78854	TKDTNNN LKG	Mus musculu s
1	1.00	1	1	0	0	-0.005	0	0	t	60991	SSFFSYGEI	Mus musculu s

1	1.00	1	1	0	-0.003	-0.005	0	0	t	51286	QLAKDKVNE	Oryctolagus cuniculus
1	1.00	1	1	0	0.004	-0.005	0	0	t	51286	QLAKDKVNE	MD
1	1.00	1	1	0	-0.003	-0.005	0	0	t	57766	SFFSYGEI	Oryctolagus cuniculus
1	1.00	1	1	0	0.004	-0.005	0	0	t	57766	SFFSYGEI	MD
1	1.00	1	1	0.01	0.01	0.01	0.01	0.01	t	915	AEGDDPAKA	Mus musculus
1	1.00	1	1	0.02	0.018	0.006	0.013	0.022	t	120460	DYDLFALAPS	Mus musculus
1	1.00	1	1	-0.03	-0.03	-0.03	-0.03	-0.032	t	142485	YNIYSEKEK	Homo sapiens
1	1.00	1	1	-0.03	-0.028	-0.03	-0.023	-0.029	t	131075	SQSYPEPARGSVPMP	Homo sapiens
1	1.00	1	1	0.01	0.01	0.01	0.014	0.01	t	142166	GLRRDLDASREAKKQLEAEQ	Homo sapiens
1	1.00	1	1	-0.02	-0.02	-0.022	-0.024	-0.02	t	39368	LSGIQLFDRI	Homo sapiens
1	1.00	1	1	-0.01	-0.013	-0.014	-0.01	-0.008	t	12625	EKAFREL	Oryctolagus cuniculus
1	1.00	1	1	0.01	0.007	0.01	0.01	0.01	t	30299	KEALDKYELENHD	Oryctolagus cuniculus
1	1.00	1	1	-0.01	-0.01	-0.01	-0.01	-0.01	t	111295	FVAHSPP	Homo sapiens

1	1.00	1	1	-0.03	-0.03	-0.03	-0.03	-0.03	t	111559	MPLVSQK	Homo sapiens
1	1.00	1	1	-0.05	-0.05	-0.05	-0.05	-0.05	t	111560	MPMTPAK	Homo sapiens
1	1.00	1	1	-0.01	-0.01	-0.01	-0.01	-0.013	t	45597	NQNNFVHDCVNITIKQHTVT	Mus musculus
1	1.00	1	1	-0.01	-0.008	-0.01	-0.013	-0.013	t	78142	EKEEPTAAPAE	Homo sapiens
1	1.00	1	1	0.01	0.008	0.002	0.009	0.01	t	97960	DTNNNLTLVP	Mus musculus
1	1.00	1	1	0.03	0.03	0.034	0.033	0.031	t	53494	REEVSFRVGLHEYP	Homo sapiens
1	1.00	1	1	0.01	0.006	0.016	0.011	0.01	t	32382	KMPMYIAGYKTFDGR	Mus musculus
1	1.00	1	1	-0.03	-0.033	-0.03	-0.03	-0.03	t	116855	WPVGGEH	Oryctolagus cuniculus
1	1.00	1	1	0.03	0.026	0.018	0.029	0.03	t	21462	GNLADDTIN	Oryctolagus cuniculus
1	1.00	1	1	-0.03	-0.027	-0.038	-0.03	-0.028	t	38434	LPDKFNAYLANKWV	Mus musculus
1	1.00	1	1	0	0	0	0.001	-0.001	t	48395	PLLGCIGSTCAEDGN	Mus musculus
1	1.00	1	1	0	0	0	0.001	-0.001	t	37316	LLGCIGSTCA	Mus musculus
1	1.00	1	1	-0.01	-0.007	-0.01	-0.01	-0.008	t	139526	ISVRTVYPPE	Mus musculus
1	1.00	1	1	0.07	0.072	0.068	0.073	0.07	t	114207	FLGELTSSEVATEV	Homo sapiens
1	1.00	1	1	0.01	0.01	0.01	0.01	0.009	t	12951	ELAPEDPEDS	Oryctolagus cuniculus

1	1.00	1	1	0.03	0.032	0.024	0.026	0.033	t	24445	HPEYAVSVLL	Homo sapiens
1	1.00	1	1	0.02	0.02	0.02	0.02	0.019	t	140570	KMEMFTYIRCDMELT	Mus musculus
1	1.00	1	1	0	0	-0.013	0	0	t	159078	RSKHNRREGYKVQVQG	Mus musculus
1	1.00	1	1	0.04	0.04	0.031	0.036	0.04	t	135941	ITTQECANVVVG	Homo sapiens
1	1.00	1	1	-0.01	-0.012	-0.005	-0.011	-0.012	t	63885	TGLPSSDRAFLVSVS	Mus musculus
1	1.00	1	1	-0.02	-0.022	-0.02	-0.017	-0.022	t	52121	QQQPFPPQQP	Mus musculus
1	1.00	1	1	0	0	0	0	-0.002	t	51939	QPQQTFPQPQ	Mus musculus
1	1.00	1	1	0.05	0.052	0.041	0.05	0.052	t	20046	GGTEESVVTASRS	Homo sapiens
1	1.00	1	1	0.03	0.03	0.03	0.03	0.03	t	111573	MSSQFYQ	Homo sapiens
1	1.00	1	1	0.03	0.03	0.03	0.03	0.03	t	111738	RPGKEIEAYIFEMFN	Oryctolagus cuniculus
1	1.00	1	1	0.03	0.03	0.03	0.03	0.03	t	112402	WNDNKFY	Homo sapiens
1	1.00	1	1	0.03	0.03	0.037	0.03	0.029	t	120020	LEDPASRDLVVN	Mus musculus
1	1.00	1	1	0.03	0.033	0.037	0.03	0.029	t	120020	LEDPASRDLVVN	Mus musculus
1	1.00	1	1	0.01	0.01	0.01	0.01	0.01	t	79547	FHQMPDA	Oryctolagus cuniculus

1	1.00	1	1	-0.04	-0.038	-0.038	-0.039	-0.041	t	103331	LAALPDFLGGFAGLP	Homo sapiens
1	1.00	1	1	0.04	0.042	0.035	0.037	0.039	t	7902	DEAEEEDENPYVFED	Homo sapiens
1	1.00	1	1	0.02	0.018	0.015	0.021	0.022	t	130905	IRCFKRHGAEVIDTPVFE	Mus musculus
1	1.00	1	1	-0.06	-0.06	-0.065	-0.06	-0.059	t	133667	QDVCTYRDFMYK	Mus musculus
1	1.00	1	1	0	0	0	0	0	t	1115	AEPKPAEPKS	Homo sapiens
1	1.00	1	1	0.02	0.022	0.027	0.02	0.019	t	120679	KIFADDID	Rattus norvegicus
1	1.00	1	1	0	0	-0.002	0	-0.001	t	119052	WNPADYGGIK	Rattus norvegicus
1	1.00	1	1	0	0	-0.002	0	-0.001	t	119052	WNPADYGGIK	Rattus norvegicus
1	1.00	1	1	0.03	0.03	0.03	0.03	0.03	t	109947	SRWQEGN	Homo sapiens
1	1.00	1	1	0	-0.002	0	-0.001	-0.002	t	22755	GTLVKTITDDQIEV	Mus musculus
1	1.00	1	1	0	-0.002	0	-0.001	-0.002	t	24012	HHPSTNQEQTSLYVQAS	Mus musculus
1	1.00	1	1	0.01	0.012	0.01	0.013	0.009	t	56730	SADGTTVTSGSTKDTSLQ	Homo sapiens
1	1.00	1	1	0.01	0.01	0.004	0.01	0.01	t	6479	CKGKIQALCENPEWA	Mus musculus
1	1.00	1	1	0.02	0.022	0.02	0.021	0.02	t	58434	SHVPLATSRTLA	Homo sapiens
1	1.00	1	1	-0.02	-0.018	-0.02	-0.019	-0.02	t	116978	FPSHYWLYPPPT	Homo sapiens
1	1.00	1	1	-0.02	-0.02	-0.02	-0.011	-0.018	t	96463	KIKDPDASKP	Homo sapiens

1	1.00	1	1	-0.03	-0.029	-0.041	-0.03	-0.03	t	51942	QPQRPFG QQP	Mus musculu s
1	1.00	1	1	0.01	0.01	0.007	0.01	0.01	t	73637	YEDRYYRE NM	Mus musculu s
1	1.00	1	1	0.01	0.01	0.007	0.01	0.01	t	73637	YEDRYYRE NM	Mus musculu s
1	1.00	1	1	0.02	0.02	0.02	0.02	0.02	t	112125	GFSLTTY	Homo sapiens
1	1.00	1	1	-0.01	-0.01	-0.022	-0.01	-0.009	t	104521	IGTLKKILD ETVKDKIA KEQ	Rattus norvegic us
1	1.00	1	1	-0.02	-0.016	-0.02	-0.013	-0.018	t	134219	RPYNIYPH GITDVRPL YSRR	Homo sapiens
1	1.00	1	1	0.02	0.02	0.018	0.021	0.021	t	144791	STIDYFQP NNKR	Sus scrofa
1	1.00	1	1	0.03	0.032	0.032	0.033	0.03	t	122892	RSILPYGDS MDRIE	Homo sapiens
1	1.00	1	1	0.01	-0.002	0.016	0.01	0.01	t	35231	LDNAKYQ A	Macaca mulatta
1	1.00	1	1	0.01	0.001	0.016	0.01	0.01	t	35231	LDNAKYQ A	Macaca mulatta
1	1.00	1	1	0.01	0.006	0.001	0.009	0.008	t	63038	TAVSPTTL RTEVVK	Oryctola gus cuniculu s
1	1.00	1	1	-0.01	-0.008	-0.01	-0.003	-0.007	t	107479	KFNWYVD	Homo sapiens
1	1.00	1	1	0	0	0	0	-0.001	t	68018	VDLPQLGL RR	Mus musculu s
1	1.00	1	1	0.02	0.023	0.02	0.02	0.018	t	135458	FGSDYEDR YYR	Mus musculu s

1	1.00	1	1	0.01	0.005	0.01	0.01	0.01	t	119564	EDPSVEEE EIAPPLPP	Canis lupus
1	1.00	1	1	0.01	0.01	0.01	0.01	0.01	t	119564	EDPSVEEE EIAPPLPP	Homo sapiens
1	1.00	1	1	-0.02	-0.019	-0.021	-0.017	-0.021	t	99088	AFDQFRQ TYRQWIIE	Equus caballus
1	1.00	1	1	0.01	0.01	0.01	0.01	0.01	t	42683	MSTNPKP QKKNKRN T	Homo sapiens
1	1.00	1	1	0	0.003	-0.014	-0.003	0	t	142324	VELAILIDN INNTLVNL EWL	Felis catus
1	1.00	1	1	-0.01	-0.01	-0.014	-0.01	-0.01	t	94444	CCHPACG KH	Capra hircus
1	1.00	1	1	0.04	0.042	0.005	0.041	0.05	t	44172	NHVADIDK LIDYAA	Homo sapiens
1	1.00	1	1	0.03	0.034	0.031	0.031	0.03	t	49261	PRTGLD	Homo sapiens
1	1.00	1	1	0	0.002	0	0.001	-0.002	t	149468	SKHPNWT NTQVRSSL	Cavia porcellu s
1	1.00	1	1	0.02	0.024	0.015	0.021	0.019	t	56017	RTALNCN DSLQTGFL AALFY	Homo sapiens
1	1.00	1	1	0	0	0	0	-0.002	t	130289	VSAAPGSA APAAGSA	Homo sapiens
1	1.00	1	1	-0.05	-0.048	-0.05	-0.043	-0.048	t	107607	RVVSVLT	Homo sapiens
1	1.00	1	1	0.02	0.022	0.014	0.023	0.02	t	58381	SHLPYIEQ GMQLAEQ F	Homo sapiens
1	1.00	1	1	0.02	0.018	0.01	0.021	0.022	t	21653	GPGVAGS G	Homo sapiens

1	1.00	1	1	-0.01	-0.01	-0.005	-0.01	-0.011	t	39160	LRPFHQILYGMS	Mus musculus
1	1.00	1	1	0	0.012	0	0	-0.002	t	136364	IDPLMFKYWYNM	Gallus gallus
1	1.00	1	1	0	0.012	0	0	-0.002	t	136162	DAWKMRLSQMYD	Gallus gallus
1	1.00	1	1	0	0	0	0	-0.002	t	136162	DAWKMRLSQMYD	Gallus gallus
1	1.00	1	1	0	-0.003	0	0	-0.002	t	142480	VAAVTEKLRAGT	Oryctolagus cuniculus
1	1.00	1	1	0	0	0	0	-0.002	t	145150	DSSTYQYIYNLT	Mus musculus
1	1.00	1	1	0	0	0	0	-0.002	t	136364	IDPLMFKYWYNM	Gallus gallus
1	1.00	1	1	0	0	0	0	-0.002	t	141683	KDWG	Mus musculus
1	1.00	1	1	0	0	0	0	-0.002	t	136071	LLYGEVGDTLLIIFK	Oryctolagus cuniculus
1	1.00	1	1	0	0	0	0	-0.002	t	134237	SQWN	Mus musculus
1	1.00	1	1	0	0	0	0	-0.002	t	148632	IDSEEELINKKR	Mus musculus
1	1.00	1	1	0	0	0	0	-0.002	t	137747	DTPLTYAGLRLV	Mus musculus
1	1.00	1	1	0	0	0	0	-0.002	t	138840	DREQAPNLVYM	Mus musculus
1	1.00	1	1	0	0	0	0	-0.002	t	136064	HQIALRMEVLGCEAQDLY	Oryctolagus cuniculus
1	1.00	1	1	0	0	0	0	-0.002	t	143959	KFPHLAVGF	Oryctolagus cuniculus
1	1.00	1	1	0	0	0	0	-0.002	t	145643	YDASLEDQKGLVA	Oryctolagus cuniculus

1	1.00	1	1	0	0	0	0	-0.002	t	150978	EKNVTVTH SVNLLED	Mus musculu s
1	1.00	1	1	0	0	0	0	-0.002	t	136082	SDDPSPGA IDSNNS	Oryctola gus cuniculu s
1	1.00	1	1	0	0	0	0	-0.002	t	145539	SSTLCFWF PLAH	Mus musculu s
1	1.00	1	1	0	0	0	0	-0.002	t	142006	SDSAQGS DVSLTA	Oryctola gus cuniculu s
1	1.00	1	1	0	0	0	0	-0.002	t	139618	TGQLVHRF	Mus musculu s
1	1.00	1	1	0	0	0	0	-0.002	t	145655	YQYIYNLTI CEL	Mus musculu s
1	1.00	1	1	0	0	0	0	-0.002	t	141523	ALEPPHNL PVP	Mus musculu s
1	1.00	1	1	0	0	0	0	-0.002	t	153472	TGLRNGIT NKVNSVIE KAA	Mus musculu s
1	1.00	1	1	0	0	0	0	-0.002	t	135076	KNDVVNS QNKTEKTE	Sus scrofa
1	1.00	1	1	0	0	0	0	-0.002	t	145531	SPLLRSPIQ R	Oryctola gus cuniculu s
1	1.00	1	1	0	0	0	0	-0.002	t	134075	GWGQGG THSQWN	Mus musculu s
1	1.00	1	1	0	0	0	0	-0.002	t	150204	FHSVVHTL LEWLMEK ELMV	Mus musculu s
1	1.00	1	1	0	0	0	0	-0.002	t	147083	LCSEYRNT QIYTIN	Oryctola gus cuniculu s
1	1.00	1	1	0	0	0	0	-0.002	t	134860	AGPKAQV EAIQKY	Mus musculu s

1	1.00	1	1	0	0	0	0	-0.002	t	136818	ANFIIFRPVV	Mus musculus
1	1.00	1	1	0	0	0	0	-0.002	t	141693	KTWG	Mus musculus
1	1.00	1	1	0	0	0	0	-0.002	t	134492	QIQRRPSM	Mus musculus
1	1.00	1	1	0	0	0	0	-0.002	t	156373	LTNKITAWKSQQ	Mus musculus
1	1.00	1	1	0	0	0	0	-0.002	t	159296	SFSKPSNSWFPVEGP	Oryctolagus cuniculus
1	1.00	1	1	0	0.012	0	0	-0.002	t	136648	SPLNNFYKTQLR	Gallus gallus
1	1.00	1	1	0	0.012	0	0	-0.002	t	136727	VNWNSWHKTNLS	Gallus gallus
1	1.00	1	1	0	0	0	0	-0.002	t	140674	ESQPDPKPDELHKS	Mus musculus
1	1.00	1	1	0	0	0	0	-0.002	t	145369	MWCKIGHDRCEE	Mus musculus
1	1.00	1	1	0	0	0	0	-0.002	t	136685	TLFTPDKSPAKT	Gallus gallus
1	1.00	1	1	0	0	0	0	-0.002	t	138456	KEQFEFALTAVAEEV	Oryctolagus cuniculus
1	1.00	1	1	0	-0.003	0	0	-0.002	t	138456	KEQFEFALTAVAEEV	Oryctolagus cuniculus
1	1.00	1	1	0	0	0	0	-0.002	t	134200	PVVNSLDPPLLTRY	Oryctolagus cuniculus
1	1.00	1	1	0	0	0	0	-0.002	t	136083	SEGAEYDDQTSQREKEDDK	Oryctolagus cuniculus
1	1.00	1	1	0	0	0	0	-0.002	t	142435	KTDGKGEESE	Homo sapiens

1	1.00	1	1	0	0	0	0	-0.002	t	144467	RPWLVHP RHRY	Mus musculus
1	1.00	1	1	0	0	0	0	-0.002	t	137735	DTALTREA LRLV	Mus musculus
1	1.00	1	1	0	0	0	0	-0.002	t	137827	DVKSAIEG	Rattus norvegicus
1	1.00	1	1	0	0	0	0	-0.002	t	136066	IETDKTSV QV	Rattus norvegicus
1	1.00	1	1	0	0	0	0	-0.002	t	136302	GALIYTPEK YTI	Gallus gallus
1	1.00	1	1	0	0	0	0	-0.002	t	138886	NVGGDNV DIHSIVPV GQDPH	Mus musculus
1	1.00	1	1	0	0	0	0	-0.002	t	144623	EEAEGTTD AQIDANRK RESE	Mus musculus
1	1.00	1	1	0	0	0	0	-0.002	t	145376	NCWKPQV LHC	Homo sapiens
1	1.00	1	1	0	0	0	0	-0.002	t	148701	PAELMRRF ELYF	Mus musculus
1	1.00	1	1	0	0	0	0	-0.002	t	136081	RTTLQSDQ EEIDY	Oryctolagus cuniculus
1	1.00	1	1	0	0	0	0	-0.002	t	156601	ADEPNNL NSK	Oryctolagus cuniculus
1	1.00	1	1	0	0	0	0	-0.002	t	134220	RQGAEPR KNFVKPNE TKTY	Oryctolagus cuniculus
1	1.00	1	1	0	0	0	0	-0.002	t	145239	HHQIDGG NWFHL	Mus musculus
1	1.00	1	1	0	0.002	0	0	-0.002	t	136066	IETDKTSV QV	Rattus norvegicus
1	1.00	1	1	0	0	0	0	-0.002	t	137748	DTQLTLEA LKLV	Mus musculus

1	1.00	1	1	0	0	0	0	-0.002	t	150987	GKCPKYVKSTKLRLA	Mus musculus
1	1.00	1	1	0	0.012	0	0	-0.002	t	136635	SIPTYTPDKVTY	Gallus gallus
1	1.00	1	1	0	-0.003	0	0	-0.002	t	142441	LFSPWLRVQNHF	Oryctolagus cuniculus
1	1.00	1	1	0	0	0	0	-0.002	t	134220	RQGAEPRKNFVKPNETKTY	Oryctolagus cuniculus
1	1.00	1	1	0	0	0	0	-0.002	t	151023	LCKLRGVAPLHLGKC	Mus musculus
1	1.00	1	1	0	0	0	0	-0.002	t	136138	APYDTPWPSPSL	Gallus gallus
1	1.00	1	1	0	0	0	0	-0.002	t	146795	CCELCCNPQCAGCY	Oryctolagus cuniculus
1	1.00	1	1	0	0	0	0	-0.002	t	119410	YKMFPEVKEKGMA	Oryctolagus cuniculus
1	1.00	1	1	0	-0.003	0	0	-0.002	t	125246	RRQPHVMS	Oryctolagus cuniculus
1	1.00	1	1	0	-0.003	0	0	-0.002	t	119331	RTVLVSMNYR	Oryctolagus cuniculus
1	1.00	1	1	0	0	0	0	-0.002	t	119378	VDYNLKWNPDDYGGVK	Rattus norvegicus
1	1.00	1	1	0	0	0	0	-0.002	t	130607	SHNLVSSAFPSA	Camelus dromedarius
1	1.00	1	1	0	0	0	0	-0.002	t	127980	FPVIPGINIEQKNACS	Mus musculus
1	1.00	1	1	0	0	0	0	-0.002	t	131010	PSTRWTELDDVKRLLKGSRS	Homo sapiens
1	1.00	1	1	0	0	0	0	-0.002	t	132849	KKVLGEKTENPKKFK	Oryctolagus cuniculus

1	1.00	1	1	0	-0.003	0	0	-0.002	t	133469	YYYNETKLKESYKVKD	Oryctolagus cuniculus
1	1.00	1	1	0	0	0	0	-0.002	t	123193	FQIHGPR	Rattus norvegicus
1	1.00	1	1	0	0	0	0	-0.002	t	123193	FQIHGPR	Rattus norvegicus
1	1.00	1	1	0	0	0	0	-0.002	t	128066	NSYDVLSALSGNLKL	Mus musculus
1	1.00	1	1	0	0	0	0	-0.002	t	128004	GMIEVQSNYGFVWDVSLKKV	Mus musculus
1	1.00	1	1	0	0	0	0	-0.002	t	128079	PSPDITSTTKTRNFDLVNCN	Mus musculus
1	1.00	1	1	0	0	0	0	-0.002	t	128115	SSHRGNICIGATNYVADN	Mus musculus
1	1.00	1	1	0	0	0	0	-0.002	t	125015	DNESSYSRGFDMTYTE	Mus musculus
1	1.00	1	1	0	0	0	0	-0.002	t	122269	ARQNLKDAGE	Mus musculus
1	1.00	1	1	0	-0.003	0	0	-0.002	t	133041	DHVKDYKESNETKLK	Oryctolagus cuniculus
1	1.00	1	1	0	0	0	0	-0.002	t	131524	KPREE	Mus musculus
1	1.00	1	1	0	0	0	0	-0.002	t	128043	LDAMPAGNPAFPVIPG	Mus musculus
1	1.00	1	1	0	0	0	0	-0.002	t	122932	VCTPKSDEFNPCEDIMGYK	Homo sapiens
1	1.00	1	1	0	-0.003	0	0	-0.002	t	122932	VCTPKSDEFNPCEDIMGYK	Oryctolagus cuniculus

1	1.00	1	1	0	0	0	0	-0.002	t	122932	VCTPKSDE FNPCEDIM GYK	Mus musculu s
1	1.00	1	1	0	0	0	0	-0.002	t	122269	ARQNLKD AGE	Mus musculu s
1	1.00	1	1	0	0	0	0	-0.002	t	122471	LLTEHRMT WDPAQPP RDL	Homo sapiens
1	1.00	1	1	0	0	0	0	-0.002	t	123442	LLKDLRKN	Homo sapiens
1	1.00	1	1	0	0	0	0	-0.002	t	128071	PIDYIIANS QANPEVFI ADS	Mus musculu s
1	1.00	1	1	0	0	0	0	-0.002	t	125055	FVYGGCQ GNN	Mus musculu s
1	1.00	1	1	0	0	0	0	-0.002	t	118077	QEMPLTA LLRNLGK MT	Mus musculu s
1	1.00	1	1	0	0	0	0	-0.002	t	118991	RAEDNAD TLALVFEA	Oryctola gus cuniculu s
1	1.00	1	1	0	0	0	0	-0.002	t	128152	YIFSEEAQ VKDVPVVT	Mus musculu s
1	1.00	1	1	0	0	0	0	-0.002	t	124560	NGVELRD	Mus musculu s
1	1.00	1	1	0	0	0	0	-0.002	t	131818	AKEERRY	Homo sapiens
1	1.00	1	1	0	0	0	0	-0.002	t	118829	GVLAGQA SQLNA	Mus musculu s
1	1.00	1	1	0	0	0	0	-0.002	t	132352	GQELKNP Q	Mus musculu s
1	1.00	1	1	0	0	0	0	-0.002	t	133181	KPEAPRRR SP	Homo sapiens
1	1.00	1	1	0	0	0	0	-0.002	t	133289	PRSPKM	Mus musculu s
1	1.00	1	1	0	0	0	0	-0.002	t	119048	VSDYEMKL MDLDVEQ	Mus musculu s

1	1.00	1	1	0	0	0	0	-0.002	t	118362	FTQLNPTD GGSA	Mus musculu s
1	1.00	1	1	0	0	0	0	-0.002	t	118258	AKSPEKAK	Homo sapiens
1	1.00	1	1	0	0	0	0	-0.002	t	123380	KLPGKF	Homo sapiens
1	1.00	1	1	0	0	0	0	-0.002	t	132269	EPVDGVVL VDPE	Mus musculu s
1	1.00	1	1	0	0	0	0	-0.002	t	122094	FLALDVPTI GLC	Mus musculu s
1	1.00	1	1	0	0	0	0	-0.002	t	133877	LEAKICHQI EYYFGDFN L	Mus musculu s
1	1.00	1	1	0	0	0	0	-0.002	t	128067	NYVADNF FYNVEGR WGSQ	Mus musculu s
1	1.00	1	1	0	0	0	0	-0.002	t	119378	VDYNLKW NPDDYGG VK	Rattus norvegic us
1	1.00	1	1	0	0	0	0	-0.002	t	128146	WSVCVGL TTYVNDYV LPYLA	Mus musculu s
1	1.00	1	1	0	0	0	0	-0.002	t	120649	ITVGLQLIQ LISVDEVN QIV	Mus musculu s
1	1.00	1	1	0	0	0	0	-0.002	t	117509	GQIPPGA M	Mus musculu s
1	1.00	1	1	0	0	0	0	-0.002	t	122778	GMTHMTS RDIEVQGF RI	Homo sapiens
1	1.00	1	1	0	0	0	0	-0.002	t	130614	STLPYLPTY SKH	Camelus dromed arius
1	1.00	1	1	0	0	0	0	-0.002	t	119499	MTFSVPIS	Mus musculu s
1	1.00	1	1	0	0	0	0	-0.002	t	121292	FATFVYAK	Mus musculu s
1	1.00	1	1	0	0	0	0	-0.002	t	118060	PPPGMRP P	Homo sapiens

1	1.00	1	1	0	0	0	0	-0.002	t	132644	SAKEK	Mus musculus
1	1.00	1	1	0	0	0	0	-0.002	t	123758	SSSSQFQIHGPRQ	Mus musculus
1	1.00	1	1	0	0	0	0	-0.002	t	122399	HPQPEDPDTAKE	Mus musculus
1	1.00	1	1	0	0	0	0	-0.002	t	123826	VPRESY	Homo sapiens
1	1.00	1	1	0	0	0	0	-0.002	t	130622	TGLVAQGTDSIG	Camelus dromedarius
1	1.00	1	1	0	0	0	0	-0.002	t	128005	GNLKLCFCGDYIFSEE	Mus musculus
1	1.00	1	1	0	0	0	0	-0.002	t	125250	SDEFNPCEDIMGYK	Mus musculus
1	1.00	1	1	0	0	0	0	-0.002	t	118141	SKGRRFKG	Mus musculus
1	1.00	1	1	0	0	0	0	-0.002	t	124008	DGVVLVD	Mus musculus
1	1.00	1	1	0	0	0	0	-0.002	t	134015	CDKNTGDYYEDSYED	Oryctolagus cuniculus
1	1.00	1	1	0	0	0	0	-0.002	t	119642	SEHETRLVANLLENYNKVIR	Mus musculus
1	1.00	1	1	0	0	0	0	-0.002	t	125250	SDEFNPCEDIMGYK	Mus musculus
1	1.00	1	1	0	0	0	0	-0.002	t	119331	RTVLVSMNYR	Oryctolagus cuniculus
1	1.00	1	1	0	0	0	0	-0.002	t	122138	LKKDV	Mus musculus
1	1.00	1	1	0	0	0	0	-0.002	t	122205	VFTLPPPLPETH	Mus musculus
1	1.00	1	1	0	0	0	0	-0.002	t	122943	WDPAQPPRD	Homo sapiens

1	1.00	1	1	0	0	0	0	-0.002	t	117437	AIALREYRKKMDIPA	Mus musculus
1	1.00	1	1	0	0	0	0	-0.002	t	118867	KLSQTSNVDKEEEAV	Mus musculus
1	1.00	1	1	0	0	0	0	-0.002	t	120946	SISPESDRPDLSTFMESGEW	Mus musculus
1	1.00	1	1	0	0	0	0	-0.002	t	134020	CPEEPQLRMKNNEEAEDYDD	Oryctolagus cuniculus
1	1.00	1	1	0	0	0	0	-0.002	t	123359	KFPEDFESC	Mus musculus
1	1.00	1	1	0	0	0	0	-0.002	t	122748	EHRMTWDPAQPPR	Homo sapiens
1	1.00	1	1	0	0	0	0	-0.002	t	119229	KHARKERNITGGH	Mus musculus
1	1.00	1	1	0	0	0	0	-0.002	t	117399	VMIKSLPALNSPTVEE	Oryctolagus cuniculus
1	1.00	1	1	0	0	0	0	-0.002	t	125230	RERRGIAL	Mus musculus
1	1.00	1	1	0	0.006	0	0	-0.002	t	124588	PQEETLQAFDSHYDYT	Ovis aries
1	1.00	1	1	0	0	0	0	-0.002	t	118499	LQEMPTLALLRNLGKM	Oryctolagus cuniculus
1	1.00	1	1	0	0	0	0	-0.002	t	120920	RLVEHLLDPSRYNKLIR	Oryctolagus cuniculus
1	1.00	1	1	0	0	0	0	-0.002	t	122486	MILHPDVQRRVQQEIDDVI	Homo sapiens
1	1.00	1	1	0	0	0	0	-0.002	t	127986	FVGVGADYRHASCPIDYIIA	Mus musculus

1	1.00	1	1	0	0	0	0	-0.002	t	120436	DGTKVSIS	Rattus norvegicus
1	1.00	1	1	0	0	0	0	-0.002	t	130513	IGYQPSMPLDMP	Camelus dromedarius
1	1.00	1	1	0	0	0	0	-0.002	t	127944	DLVNCNLNTNCVAVAFSLPD	Mus musculus
1	1.00	1	1	0	0	0	0	-0.002	t	119025	SWTDTDGTN	Mus musculus
1	1.00	1	1	0	0	0	0	-0.002	t	122892	RSILPYGDSMDRIE	Homo sapiens
1	1.00	1	1	0	0	0	0	-0.002	t	118678	WPLHDGGAVNRR	Mus musculus
1	1.00	1	1	0	0	0	0	-0.002	t	120416	AVALREYRKKMDIPA	Mus musculus
1	1.00	1	1	0	0	0	0	-0.002	t	118763	CYGSFARPMN	Mus musculus
1	1.00	1	1	0	0	0	0	-0.002	t	121070	WVYYTCCPDTPYL	Rattus norvegicus
1	1.00	1	1	0	0	0	0	-0.002	t	117614	QGFPFYDKPMRI	Mus musculus
1	1.00	1	1	0	0	0	0	-0.002	t	122129	KTVTVKEDQVMQQNPPKF	Mus musculus
1	1.00	1	1	0	0	0	0	-0.002	t	133231	MGPRRRSRKP	Homo sapiens
1	1.00	1	1	0	0	0	0	-0.002	t	125472	LDAVYLNKNKYLTVIDKDAF	Homo sapiens
1	1.00	1	1	0	0	0	0	-0.002	t	119378	VDYNLKWNPDDYGGVK	Rattus norvegicus
1	1.00	1	1	0	0	0	0	-0.002	t	125478	LHQEYEENLGDSIVGYKEKS	Cricetulus migratorius
1	1.00	1	1	0	0	0	0	-0.002	t	129301	LQELEKDEREQ	Homo sapiens

1	1.00	1	1	0	0	0	0	-0.002	t	128010	GRWGSQRAVNVSGGFQF	Mus musculus
1	1.00	1	1	0	0	0	0	-0.002	t	122167	PPPITQGECKQL	Mus musculus
1	1.00	1	1	0	0	0	0	-0.002	t	120528	FIEWLKVKILPEVKEK	Oryctolagus cuniculus
1	1.00	1	1	0	0	0	0	-0.002	t	118633	TNSFNDSGSAPH	Mus musculus
1	1.00	1	1	0	-0.003	0	0	-0.002	t	119410	YKMFPEVKEKGMA	Oryctolagus cuniculus
1	1.00	1	1	0	0	0	0	-0.002	t	118146	SNLDHILEPSIP	Homo sapiens
1	1.00	1	1	0	0	0	0	-0.002	t	128089	QVYYRPVDQYSNQN	Ovis aries
1	1.00	1	1	0	0	0	0	-0.002	t	122430	KGLRRDLDASREAK	Mus musculus
1	1.00	1	1	0	0	0	0	-0.002	t	128084	QKNACSFDLCNSYDVL	Mus musculus
1	1.00	1	1	0	0	0	0	-0.002	t	118762	CYGSFAKPTN	Mus musculus
1	1.00	1	1	0	0	0	0	-0.002	t	128133	TSEPLNSESEVTDGMIEVQS	Mus musculus
1	1.00	1	1	0	0	0	0	-0.002	t	130555	MHNRTQALEDSR	Camelus dromedarius
1	1.00	1	1	0	0	0	0	-0.002	t	126151	GMGCSSPPCECHQEE	Mus musculus
1	1.00	1	1	0	0	0	0	-0.002	t	127933	APDDSFKKLEDRFTNLKF	Mus musculus

1	1.00	1	1	0	0	0	0	-0.002	t	118764	CYGSFARPTN	Mus musculus
1	1.00	1	1	0	0	0	0	-0.002	t	118381	GKTCPKEIPKGSKNT	Homo sapiens
1	1.00	1	1	0	0	0	0	-0.002	t	117613	QEKLQVALGE	Homo sapiens
1	1.00	1	1	0	0	0	0	-0.002	t	123144	ENPTMAGYR	Homo sapiens
1	1.00	1	1	0	0	0	0	-0.002	t	127950	DVPVVTSVTTAGVGPSPDIT	Mus musculus
1	1.00	1	1	0	0	0	0	-0.002	t	117348	PTRAPSGPRPP	Oryctolagus cuniculus
1	1.00	1	1	0	0	0	0	-0.002	t	104367	YSNGGPFLTPIVPTAD	Oryctolagus cuniculus
1	1.00	1	1	0	0	0	0	-0.002	t	105803	GAE	Mus musculus
1	1.00	1	1	0	0	0	0	-0.002	t	107303	AWKYENGNDKHYADSVNG	Oryctolagus cuniculus
1	1.00	1	1	0	0	0	0	-0.002	t	115155	NNQRKKAKNTPFNMLKRERN	Mus musculus
1	1.00	1	1	0	0	0	0	-0.002	t	110920	KPSPWAPKKHRRLS	Mus musculus
1	1.00	1	1	0	0	0	0	-0.002	t	98841	KAMVIVVVNKGTGNLELVAV	Homo sapiens
1	1.00	1	1	0	0	0	0	-0.002	t	110980	QPLGVGISGHPLLNKLDDTE	Mus musculus
1	1.00	1	1	0	0	0	0	-0.002	t	111051	VGPLEVIPEAVDPAGSSIV	Oryctolagus cuniculus
1	1.00	1	1	0	0	0	0	-0.002	t	110824	EDEIDGPAGQAEPDRAHYNI	Mus musculus

1	1.00	1	1	0	0	0	0	-0.002	t	104851	KGAIIGLMVGGVVI	Mus musculus
1	1.00	1	1	0	0	0	0	-0.002	t	104051	LPSDPIGGKSNMHFMNTL	Oryctolagus cuniculus
1	1.00	1	1	0	0	0	0	-0.002	t	110862	GGPTLVSLHELPAETP	Oryctolagus cuniculus
1	1.00	1	1	0	0	0	0	-0.002	t	103965	GYKYLGPGNSLDQGEPT	Oryctolagus cuniculus
1	1.00	1	1	0	0	0	0	-0.002	t	99838	QQRPFI	Mus musculus
1	1.00	1	1	0	0	0	0	-0.002	t	115540	VKNKRTFLSPWISNI	Mus musculus
1	1.00	1	1	0	0	0	0	-0.002	t	110126	YIK	Mus musculus
1	1.00	1	1	0	0	0	0	-0.002	t	105653	AEIVYKSPVVSGD	Mus musculus
1	1.00	1	1	0	0	0	0	-0.002	t	103792	AGTELSATGNESGGG	Oryctolagus cuniculus
1	1.00	1	1	0	0	0	0	-0.002	t	107346	ERMFRK	Rattus norvegicus
1	1.00	1	1	0	-0.004	0	0	-0.002	t	110980	QPLGVGISGHPLLNKLDDTE	Mus musculus
1	1.00	1	1	0	0	0	0	-0.002	t	108551	NDSLNTGFLAFLFYTKK	Capra hircus
1	1.00	1	1	0	0	0	0	-0.002	t	111050	VGPLDIVPEVADPGGPTLV	Oryctolagus cuniculus
1	1.00	1	1	0	0	0	0	-0.002	t	110903	IDGP	Mus musculus
1	1.00	1	1	0	0	0	0	-0.002	t	107448	IPSTVKA	Rattus norvegicus

1	1.00	1	1	0	0	0	0	-0.002	t	110856	FSADLDQFPLGRKFL	Mus musculus
1	1.00	1	1	0	-0.003	0	0	-0.002	t	111050	VGPLDIVPEVADPGGPTLV	Oryctolagus cuniculus
1	1.00	1	1	0	0	0	0	-0.002	t	104850	KEGILQYCQEVYPELQ	Mus musculus
1	1.00	1	1	0	0	0	0	-0.002	t	104049	LPPKLLTEPTTEGDQHPGT	Oryctolagus cuniculus
1	1.00	1	1	0	0	0	0	-0.002	t	101490	ASGEVPVKPSEG	Mus musculus
1	1.00	1	1	0	0	0	0	-0.002	t	103972	HNPINAGTELSATGN	Oryctolagus cuniculus
1	1.00	1	1	0	0	0	0	-0.002	t	101558	DQPDQHQ	Mus musculus
1	1.00	1	1	0	0	0	0	-0.002	t	101689	GNNNDSTGSSDV	Mus musculus
1	1.00	1	1	0	0	0	0	-0.002	t	107852	GEPQSSIIQY	Mus musculus
1	1.00	1	1	0	0	0	0	-0.002	t	116971	FAVLKLMGRGTKF	Rattus norvegicus
1	1.00	1	1	0	0	0	0	-0.002	t	104327	VEQHNPINAGTELSAT	Oryctolagus cuniculus
1	1.00	1	1	0	0	0	0	-0.002	t	107326	DVSDMFSK	Rattus norvegicus
1	1.00	1	1	0	0	0	0	-0.002	t	112584	LPWNAFPGK	Rattus norvegicus
1	1.00	1	1	0	0	0	0	-0.002	t	110916	KLDDTENASAYAANA	Mus musculus
1	1.00	1	1	0	0	0	0	-0.002	t	99833	PPFSQQ	Mus musculus

1	1.00	1	1	0	0	0	0	-0.002	t	108482	IINPSENG DTSTNGIK K	Mus musculu s
1	1.00	1	1	0	0	0	0	-0.002	t	98822	IEAGAIGFK IHEDWG	Mus musculu s
1	1.00	1	1	0	0	0	0	-0.002	t	107668	VSETLK	Rattus norvegic us
1	1.00	1	1	0	0	0	0	-0.002	t	106375	EDHAGTY GLGDRK	Mus musculu s
1	1.00	1	1	0	0	0	0	-0.002	t	99834	QGQQGYY P	Mus musculu s
1	1.00	1	1	0	0	0	0	-0.002	t	108164	TLVCLATG FFPDHVEL	Oryctola gus cuniculu s
1	1.00	1	1	0	0	0	0	-0.002	t	99134	DAEFRHDS GYEVHHQ	Mus musculu s
1	1.00	1	1	0	0	0	0	-0.002	t	107517	LSTNEVA M	Mus musculu s
1	1.00	1	1	0	0	0	0	-0.002	t	102130	TKLYQNPT TYISVGT	Mus musculu s
1	1.00	1	1	0	0	0	0	-0.002	t	113257	ADNKAPQ GLLC	Rattus
1	1.00	1	1	0	0	0	0	-0.002	t	107487	KKPTPSTL	Rattus norvegic us
1	1.00	1	1	0	0	0	0	-0.002	t	110855	FNRAGTV GENVPDD LYIKGS	Mus musculu s
1	1.00	1	1	0	0	0	0	-0.002	t	110385	QLYKTCKQ AGTCPPDII PKV	Mus musculu s
1	1.00	1	1	0	0	0	0	-0.002	t	104362	YNDDEPN GAIRFTMG	Oryctola gus cuniculu s
1	1.00	1	1	0	0	0	0	-0.002	t	110910	KAKPKFTL GKRKATP	Mus musculu s
1	1.00	1	1	0	0	0	0	-0.002	t	110824	EDEIDGPA GQAEPDR AHYNI	Mus musculu s

1	1.00	1	1	0	0	0	0	-0.002	t	104213	QQFNQQAPLNLENTNNGTL	Oryctolagus cuniculus
1	1.00	1	1	0	0	0	0	-0.002	t	101716	HQPTPAA	Mus musculus
1	1.00	1	1	0	0	0	0	-0.002	t	106313	CQQYGSSPQTFG	Oryctolagus cuniculus
1	1.00	1	1	0	0	0	0	-0.002	t	104231	SAADEKFIKETEHAKDYG	Oryctolagus cuniculus
1	1.00	1	1	0	0	0	0	-0.002	t	108897	EIDGPAGQAEP	Mus musculus
1	1.00	1	1	0	0	0	0	-0.002	t	108291	AVGADESVVKEAHREVINS	Mus musculus
1	1.00	1	1	0	0	0	0	-0.002	t	104175	PINAGTELSATGNESG	Oryctolagus cuniculus
1	1.00	1	1	0	0	0	0	-0.002	t	107358	FGEL	Rattus norvegicus
1	1.00	1	1	0	0	0	0	-0.002	t	110824	EDEIDGPAGQAEPDRAHYNI	Mus musculus
1	1.00	1	1	0	0	0	0	-0.002	t	106256	ANYGYTFGSGTRLTVV	Oryctolagus cuniculus
1	1.00	1	1	0	0	0	0	-0.002	t	104057	LTEPTTEGDQHPGTLPAANT	Oryctolagus cuniculus
1	1.00	1	1	0	0	0	0	-0.002	t	98822	IEAGAIGFKIHEDWG	Mus musculus
1	1.00	1	1	0	0	0	0	-0.002	t	101983	PVYQQQP	Mus musculus
1	1.00	1	1	0	0	0	0	-0.002	t	107201	LIVPEGARTSLN	Oryctolagus cuniculus

1	1.00	1	1	0	0	0	0	-0.002	t	117344	PPGMRPP	Oryctolagus cuniculus
1	1.00	1	1	0	0	0	0	-0.002	t	107194	LEQTELSVTRET	Oryctolagus cuniculus
1	1.00	1	1	0	0	0	0	-0.002	t	107486	KKPTPST	Rattus norvegicus
1	1.00	1	1	0	0	0	0	-0.002	t	117366	RRREGPDRSPR	Oryctolagus cuniculus
1	1.00	1	1	0	0	0	0	-0.002	t	101582	EEDLRTR	Mus musculus
1	1.00	1	1	0	0	0	0	-0.002	t	110824	EDEIDGPAGQAEPDRAHYNI	Mus musculus
1	1.00	1	1	0	0	0	0	-0.002	t	103998	IVKWDRDM	Mus musculus
1	1.00	1	1	0	0	0	0	-0.002	t	101962	PEPVQEP	Mus musculus
1	1.00	1	1	0	0	0	0	-0.002	t	115483	SFFDIGGSVSAGFSN	Mus musculus
1	1.00	1	1	0	0	0	0	-0.002	t	107536	PAPDTLKE	Rattus norvegicus
1	1.00	1	1	0	0	0	0	-0.002	t	107324	DTLKAGLIR	Rattus norvegicus
1	1.00	1	1	0	0	0	0	-0.002	t	104216	RAFAPKLSETDSPT	Oryctolagus cuniculus
1	1.00	1	1	0	0	0	0	-0.002	t	105163	EFRHD	Mus musculus
1	1.00	1	1	0	0	0	0	-0.002	t	101953	PDPLSPGE	Mus musculus
1	1.00	1	1	0	0	0	0	-0.002	t	107229	RHEVTEMGQEVT	Oryctolagus cuniculus

1	1.00	1	1	0	0	0	0	-0.002	t	117383	TLHKAFKGSIFVVFDSIESA	Homo sapiens
1	1.00	1	1	0	0	0	0	-0.002	t	107618	SKPQSETL	Rattus norvegicus
1	1.00	1	1	0	0	0	0	-0.002	t	107309	DAGPYVSPTFFAH	Oryctolagus cuniculus
1	1.00	1	1	0	0	0	0	-0.002	t	104327	VEQHNPINAGTELSAT	Sus scrofa
1	1.00	1	1	0	0	0	0	-0.002	t	110975	QIFNKPYWLQRAQGH	Mus musculus
1	1.00	1	1	0	0	0	0	-0.002	t	110845	EYMLD	Mus musculus
1	1.00	1	1	0	0	0	0	-0.002	t	110877	GTVGENVPDDLYIKG	Mus musculus
1	1.00	1	1	0	0	0	0	-0.002	t	103986	IHVLNSESGSAGQMVQD	Oryctolagus cuniculus
1	1.00	1	1	0	0	0	0	-0.002	t	110845	EYMLD	Mus musculus
1	1.00	1	1	0	0	0	0	-0.002	t	99134	DAEFRHDSGYEVHHQ	Mus musculus
1	1.00	1	1	0	0	0	0	-0.002	t	104695	YLLYYTEFTPTEK	Mus musculus
1	1.00	1	1	0	0	0	0	-0.002	t	100129	HYKWLNDPLAAW	Ovis aries
1	1.00	1	1	0	0	0	0	-0.002	t	115351	LKQKSSNSRKKRSTS	Mus musculus
1	1.00	1	1	0	0	0	0	-0.002	t	107378	GELP	Rattus norvegicus
1	1.00	1	1	0	0	0	0	-0.002	t	103940	GLTLPGYKYLGPGNSLD	Oryctolagus cuniculus
1	1.00	1	1	0	0	0	0	-0.002	t	110284	HKSAIVTLTYDSEWQRDQ	Homo sapiens

1	1.00	1	1	0	0	0	0	-0.002	t	110284	HKSAIVTLTYDSEWQRDQ	Homo sapiens
1	1.00	1	1	0	0	0	0	-0.002	t	102093	STGSSDV	Mus musculus
1	1.00	1	1	0	0	0	0	-0.002	t	105971	MVGGVVIAT	Mus musculus
1	1.00	1	1	0	0	0	0	-0.002	t	110762	AGSSIVPLEEYPAEIP	Oryctolagus cuniculus
1	1.00	1	1	0	0	0	0	-0.002	t	104834	HQKLVFFAE	Mus musculus
1	1.00	1	1	0	0	0	0	-0.002	t	101412	WIRNNF	Mus musculus
1	1.00	1	1	0	0	0	0	-0.002	t	110248	GGLGIGTGSGTGGRTGYIPL	Oryctolagus cuniculus
1	1.00	1	1	0	0	0	0	-0.002	t	110248	GGLGIGTGSGTGGRTGYIPL	Oryctolagus cuniculus
1	1.00	1	1	0	-0.004	0	0	-0.002	t	110855	FNRAGTVGENVPDDLYIKGS	Mus musculus
1	1.00	1	1	0	0	0	0	-0.002	t	107455	KAKGQPR	Mus musculus
1	1.00	1	1	0	0	0	0	-0.002	t	107013	YGASSRATGIPDR	Oryctolagus cuniculus
1	1.00	1	1	0	0	0	0	-0.002	t	107368	FSSAP	Rattus norvegicus
1	1.00	1	1	0	0	0	0	-0.002	t	116286	CGKGLSATVTGGQKGRGSR	Oryctolagus cuniculus
1	1.00	1	1	0	0	0	0	-0.002	t	111272	FGAMDFTTLQANKSE	Mus musculus
1	1.00	1	1	0	0	0	0	-0.002	t	111272	FGAMDFTTLQANKSE	Mus musculus

1	1.00	1	1	0	0	0	0	-0.002	t	117246	VTWEVLE GEVEKEAL KKI	Homo sapiens
1	1.00	1	1	0	0	0	0	-0.002	t	102172	VDQQQQP TQGDT	Mus musculu s
1	1.00	1	1	0	0	0	0	-0.002	t	99842	RPQQPYP	Mus musculu s
1	1.00	1	1	0	0	0	0	-0.002	t	105090	AIIGLMVG GVVIA	Mus musculu s
1	1.00	1	1	0	0	0	0	-0.002	t	102390	EHRAW	Sus scrofa
1	1.00	1	1	0	0	0	0	-0.002	t	97484	LVEETSFID AGAP	Mus musculu s
1	1.00	1	1	0	0	0	0	-0.002	t	61801	STNSGINN	Mus musculu s
1	1.00	1	1	0	0	0	0	-0.002	t	97370	GTVLVTRK AL	Mus musculu s
1	1.00	1	1	0	0	0	0	-0.002	t	94553	IFD	Mus musculu s
1	1.00	1	1	0	0	0	0	-0.002	t	75991	YTDAPSFS DIPNPI	Homo sapiens
1	1.00	1	1	0	0	0	0	-0.002	t	96874	SIAPDLSLD FEKLNV	Mus musculu s
1	1.00	1	1	0	0	0	0	-0.002	t	64396	TINKPKGY VGKE	Oryctola gus cuniculu s
1	1.00	1	1	0	0	0	0	-0.002	t	63022	TATRLLSST NSRLC	Mus musculu s
1	1.00	1	1	0	0	0	0	-0.002	t	67253	TWEVLH	Mus musculu s

1	1.00	1	1	0	0	0	0	-0.002	t	67253	TWEVLH	Mus musculus
1	1.00	1	1	0	0	0	0	-0.002	t	79153	ALQREAAERSEDEIK	Oryctolagus cuniculus
1	1.00	1	1	0	0	0	0	-0.002	t	71312	VTGATVS	Oryctolagus cuniculus
1	1.00	1	1	0	0	0	0	-0.002	t	64032	THGQWNKPSKPKTNMK	Oryctolagus cuniculus
1	1.00	1	1	0	0	0	0	-0.002	t	61410	SSTRGGSRVEGIFAGLFMNA	Oryctolagus cuniculus
1	1.00	1	1	0	0	0	0	-0.002	t	64045	THKVGTSFTFES	Oryctolagus cuniculus
1	1.00	1	1	0	0	0	0	-0.002	t	70038	VMYASSG	Oryctolagus cuniculus
1	1.00	1	1	0	0	0	0	-0.002	t	60596	SQWNKPSKPKTN	Oryctolagus cuniculus
1	1.00	1	1	0	0	0	0	-0.002	t	63756	TFTNKLKEKHTDLGK	Mus musculus
1	1.00	1	1	0	0	0	0	-0.002	t	66050	TRLENLMWK	Mus musculus
1	1.00	1	1	0	0	0	0	-0.002	t	60596	SQWNKPSKPKTN	Oryctolagus cuniculus
1	1.00	1	1	0	0	0	0	-0.002	t	67253	TWEVLH	Mus musculus
1	1.00	1	1	0	0	0	0	-0.002	t	75391	YPQPQPFPSQ	Mus musculus

1	1.00	1	1	0	0	0	0	-0.002	t	67564	VAEFIEKVSRR	Oryctolagus cuniculus
1	1.00	1	1	0	0	0	0	-0.002	t	60480	SQKSADQSIDFGQL	Oryctolagus cuniculus
1	1.00	1	1	0	0	0	0	-0.002	t	65000	TLPERVRPPDDWAT	Mus musculus
1	1.00	1	1	0	0	0	0	-0.002	t	75391	YPQPQPFPSQ	Mus musculus
1	1.00	1	1	0	0	0	0	-0.002	t	71354	VTHESYQELVKKLEALEDAV	Mus musculus
1	1.00	1	1	0	0	0	0	-0.002	t	60527	SQPQQPFPQQ	Mus musculus
1	1.00	1	1	0	0	0	0	-0.002	t	66833	TTSTGPCKTCTTPAQ	Mus musculus
1	1.00	1	1	0	0	0	0	-0.002	t	70038	VMYASSG	Oryctolagus cuniculus
1	1.00	1	1	0	0	0	0	-0.002	t	68415	VEYTKYNDDDTFT	Oryctolagus cuniculus
1	1.00	1	1	0	0	0	0	-0.002	t	73430	YAQYMSRAYAEFLGEDPGSG	Mus musculus
1	1.00	1	1	0	0	0	0	-0.002	t	75392	YPQPQPFRPQ	Mus musculus
1	1.00	1	1	0	0	0	0	-0.002	t	82843	GQPAADHYYSKVT	Oryctolagus cuniculus
1	1.00	1	1	0	0	0	0	-0.002	t	78424	IPRSRQIDLQIG	Mus musculus
1	1.00	1	1	0	-0.003	0	0	-0.002	t	64396	TINKPKGYVGKE	Oryctolagus cuniculus

1	1.00	1	1	0	0	0	0	-0.002	t	74068	YGPGVDY	Mus musculus
1	1.00	1	1	0	0	0	0	-0.002	t	76290	YVHDNC	Mus musculus
1	1.00	1	1	0	0	0	0	-0.002	t	69568	VLKLSI	Mus musculus
1	1.00	1	1	0	0	0	0	-0.002	t	61800	STNS	Mus musculus
1	1.00	1	1	0	0	0	0	-0.002	t	78159	GALATYQSEYLAHRRIPP	Oryctolagus cuniculus
1	1.00	1	1	0	0	0	0	-0.002	t	76453	YWERGIPLP	Homo sapiens
1	1.00	1	1	0	0	0	0	-0.002	t	67967	VDGSDP	Mus musculus
1	1.00	1	1	0	0	0	0	-0.002	t	63122	TDAYNQKLSERRAN	Mus musculus
1	1.00	1	1	0	0	0	0	-0.002	t	65421	TNSGINNS	Mus musculus
1	1.00	1	1	0	0	0	0	-0.002	t	67931	VDEKKM	Mus musculus
1	1.00	1	1	0	0	0	0	-0.002	t	66480	TSVENAQQDKGIAIPHDIDL	Cavia porcellus
1	1.00	1	1	0	0	0	0	-0.002	t	75409	YPSGAWYYVP	Homo sapiens
1	1.00	1	1	0	0	0	0	-0.002	t	60596	SQWNKPSKPKTN	Oryctolagus cuniculus
1	1.00	1	1	0	0	0	0	-0.002	t	65790	TPTPVNPSTAPAPAPTPTFA	Homo sapiens
1	1.00	1	1	0	0	0	0	-0.002	t	71312	VTGATVS	Oryctolagus cuniculus

1	1.00	1	1	0	0	0	0	-0.002	t	64057	THNQWNKPSKPKTNLK	Oryctolagus cuniculus
1	1.00	1	1	0	0	0	0	-0.002	t	97348	GLFGAIAGFIEGGW	Oryctolagus cuniculus
1	1.00	1	1	0	0	0	0	-0.002	t	60721	SRKSYKKMLKEMGEVAPEYR	Oryctolagus cuniculus
1	1.00	1	1	0	0	0	0	-0.002	t	64057	THNQWNKPSKPKTNLK	Oryctolagus cuniculus
1	1.00	1	1	0	0	0	0	-0.002	t	72910	WNSTTFHQTL	Mus musculus
1	1.00	1	1	0	0	0	0	-0.002	t	73784	YEVRAELPGVDPDKDVDIMV	Mus musculus
1	1.00	1	1	0	0	0	0	-0.002	t	70437	VPPTQSKKKNKNET	Oryctolagus cuniculus
1	1.00	1	1	0	0	0	0	-0.002	t	64032	THGQWNKPSKPKTNMK	Oryctolagus cuniculus
1	1.00	1	1	0	0	0	0	-0.002	t	64057	THNQWNKPSKPKTNLK	Oryctolagus cuniculus
1	1.00	1	1	0	0	0	0	-0.002	t	75659	YRPLGPGTPPMRARLPA	Oryctolagus cuniculus
1	1.00	1	1	0	0	0	0	-0.002	t	71531	VTTSGGRRQ	Oryctolagus cuniculus
1	1.00	1	1	0	0	0	0	-0.002	t	92303	AKRFKINAI	Mus musculus
1	1.00	1	1	0	0	0	0	-0.002	t	95008	EDVFQNGFTAW	Mus musculus

1	1.00	1	1	0	0	0	0	-0.002	t	67931	VDEKKM	Mus musculus
1	1.00	1	1	0	0	0	0	-0.002	t	61686	STKAAKKPEAKREAI	Mus musculus
1	1.00	1	1	0	0	0	0	-0.002	t	85833	LSIIAEQSNSTVDNQK	Oryctolagus cuniculus
1	1.00	1	1	0	0	0	0	-0.002	t	63102	TCTTPAQGNSMFPS	Mus musculus
1	1.00	1	1	0	0	0	0	-0.002	t	65733	TPRAQATLLR	Mus musculus
1	1.00	1	1	0	-0.003	0	0	-0.002	t	85833	LSIIAEQSNSTVDNQK	Oryctolagus cuniculus
1	1.00	1	1	0	0.003	0	0	-0.002	t	66050	TRLENLMWK	Mus musculus
1	1.00	1	1	0	0	0	0	-0.002	t	63391	TEHRDYHPQQRG	Mus musculus
1	1.00	1	1	0	0	0	0	-0.002	t	60917	SSAVVDNKLKI	Mus musculus
1	1.00	1	1	0	0	0	0	-0.002	t	63038	TAVSPTTLRTEVVK	Oryctolagus cuniculus
1	1.00	1	1	0	0	0	0	-0.002	t	73636	YEDRYYRE	Mus musculus
1	1.00	1	1	0	0	0	0	-0.002	t	64663	TKRRITPKD	Mus musculus
1	1.00	1	1	0	0	0	0	-0.002	t	65556	TPEMDIPGQVNNLMEIAEVD	Mus musculus
1	1.00	1	1	0	0	0	0	-0.002	t	74430	YKAYVSYKKRKAQEK	Oryctolagus cuniculus
1	1.00	1	1	0	0	0	0	-0.002	t	74687	YLGYLEQLLR	Homo sapiens

1	1.00	1	1	0	0	0	0	-0.002	t	64056	THNQWNKPSKPKTNFK	Gallus gallus
1	1.00	1	1	0	0	0	0	-0.002	t	96409	INDMPITNDQKKL	Oryctolagus cuniculus
1	1.00	1	1	0	0	0	0	-0.002	t	68200	VEGMQFDRGYLSPYF	Mus musculus
1	1.00	1	1	0	0	0	0	-0.002	t	72911	WNSTTFHQTLQDP	Mus musculus
1	1.00	1	1	0	0	0	0	-0.002	t	75418	YPTFGEHKQEKDLEY	Mus musculus
1	1.00	1	1	0	0	0	0	-0.002	t	94943	TKVSNGAFVPNM	Oryctolagus cuniculus
1	1.00	1	1	0	0	0	0	-0.002	t	72911	WNSTTFHQTLQDP	Mus musculus
1	1.00	1	1	0	0	0	0	-0.002	t	66072	TRNTGQPATDHYYSNV	Oryctolagus cuniculus
1	1.00	1	1	0	0	0	0	-0.002	t	67931	VDEKKM	Mus musculus
1	1.00	1	1	0	0	0	0	-0.002	t	67615	VAGRKKLEELERDLRKTK	Homo sapiens
1	1.00	1	1	0	0	0	0	-0.002	t	65770	TPSPDRDERGALGWG	Mus musculus
1	1.00	1	1	0	0	0	0	-0.002	t	94832	GLFGNNENQTKV	Oryctolagus cuniculus
1	1.00	1	1	0	0	0	0	-0.002	t	64978	TLNKDQLLSSSKYTIQRSTG	Oryctolagus cuniculus
1	1.00	1	1	0	0	0	0	-0.002	t	65779	TPTAWKPN	Mus musculus

1	1.00	1	1	0	-0.003	0	0	-0.002	t	88388	SAWNSDSEKPFDDHL	Oryctolagus cuniculus
1	1.00	1	1	0	-0.003	0	0	-0.002	t	79622	NYARTGGQVTAG	Oryctolagus cuniculus
1	1.00	1	1	0	0	0	0	-0.002	t	73131	WTGVAQD	Oryctolagus cuniculus
1	1.00	1	1	0	0	0	0	-0.002	t	75318	YPHQPQQQFP	Mus musculus
1	1.00	1	1	0	0	0	0	-0.002	t	60592	SQWGWCGSTDEYCSPDHNCQ	Mus musculus
1	1.00	1	1	0	0	0	0	-0.002	t	95058	AGLQND	Mus musculus
1	1.00	1	1	0	0	0	0	-0.002	t	70729	VRGLYFP	Mus musculus
1	1.00	1	1	0	0	0	0	-0.002	t	63730	TFPQPQQTYP	Mus musculus
1	1.00	1	1	0	0	0	0	-0.002	t	63037	TAVSPTTLR	Oryctolagus cuniculus
1	1.00	1	1	0	0	0	0	-0.002	t	64010	TGYRYDSRTGFFATN	Mus musculus
1	1.00	1	1	0	-0.003	0	0	-0.002	t	65568	TPFHHQHSTGFT	Oryctolagus cuniculus
1	1.00	1	1	0	0	0	0	-0.002	t	72087	VYLLPR	Homo sapiens
1	1.00	1	1	0	0	0	0	-0.002	t	96457	KGTVNLTWSR	Mus musculus

1	1.00	1	1	0	0	0	0	-0.002	t	64010	TGYRYDSR TGFFATN	Mus musculu s
1	1.00	1	1	0	0	0	0	-0.002	t	74488	YKPLGALT H	Mus musculu s
1	1.00	1	1	0	0	0	0	-0.002	t	94580	LDNKYAGK GY	Mus musculu s
1	1.00	1	1	0	0	0	0	-0.002	t	64057	THNQWN KPSKPKTN LK	Oryctola gus cuniculu s
1	1.00	1	1	0	0	0	0	-0.002	t	72504	WGQGGS H	Mus musculu s
1	1.00	1	1	0	0	0	0	-0.002	t	64032	THGQWN KPSKPKTN MK	Oryctola gus cuniculu s
1	1.00	1	1	0	0	0	0	-0.002	t	75372	YPNQVYYR PVDRYSN QNNFV	Oryctola gus cuniculu s
1	1.00	1	1	0	0	0	0	-0.002	t	66767	TTPKNPTK RRITPKDVI	Oryctola gus cuniculu s
1	1.00	1	1	0	0	0	0	-0.002	t	78077	YPYSMWK NLWPS	Mus musculu s
1	1.00	1	1	0	0	0	0	-0.002	t	63604	TEYYLNHG KWPEN	Oryctola gus cuniculu s
1	1.00	1	1	0	0	0	0	-0.002	t	64985	TLNPTI	Mus musculu s
1	1.00	1	1	0	0	0	0	-0.002	t	65555	TPEMDIPG QV	Mus musculu s
1	1.00	1	1	0	0	0	0	-0.002	t	64985	TLNPTI	Mus musculu s

1	1.00	1	1	0	-0.003	0	0	-0.002	t	67187	TVTTTTKGENFTETDIKI	Oryctolagus cuniculus
1	1.00	1	1	0	0	0	0	-0.002	t	68200	VEGMQFDRGYLSPYF	Mus musculus
1	1.00	1	1	0	0	0	0	-0.002	t	79111	VNKGYTGY	Mus musculus
1	1.00	1	1	0	0	0	0	-0.002	t	60753	SRPGSTTPSGNSARYGNNT	Mus musculus
1	1.00	1	1	0	0	0	0	-0.002	t	61843	STPVAPTQEVKK	Oryctolagus cuniculus
1	1.00	1	1	0	0	0	0	-0.002	t	68895	VHTWTEQYK	Mus musculus
1	1.00	1	1	0	0	0	0	-0.002	t	74415	YIWPRNDYDG	Mus musculus
1	1.00	1	1	0	0	0	0	-0.002	t	70391	VPNLRGDLQVLAQKVARTLP	Oryctolagus cuniculus
1	1.00	1	1	0	0	0	0	-0.002	t	67930	VDEKKK	Mus musculus
1	1.00	1	1	0	0	0	0	-0.002	t	67932	VDEKNM	Mus musculus
1	1.00	1	1	0	0	0	0	-0.002	t	70391	VPNLRGDLQVLAQKVARTLP	Oryctolagus cuniculus
1	1.00	1	1	0	0	0	0	-0.002	t	64985	TLNPTI	Mus musculus
1	1.00	1	1	0	0	0	0	-0.002	t	68052	VDQYSNQNNF	Oryctolagus cuniculus

1	1.00	1	1	0	0	0	0	-0.002	t	72961	WPQRVVTKKGRTFL	Mus musculus
1	1.00	1	1	0	0	0	0	-0.002	t	97484	LVEETSFIDAGAP	Oryctolagus cuniculus
1	1.00	1	1	0	0	0	0	-0.002	t	91109	AHFVQQTP	Mus musculus
1	1.00	1	1	0	0	0	0	-0.002	t	97256	EGAKGENKRPNDKAGEV	Mus musculus
1	1.00	1	1	0	0	0	0	-0.002	t	64884	TLKREIEKDG	Mus musculus
1	1.00	1	1	0	0	0	0	-0.002	t	63604	TEYYLNHGKWPEN	Oryctolagus cuniculus
1	1.00	1	1	0	0	0	0	-0.002	t	74009	YGGPANKKNAG	Aotus nancymaae
1	1.00	1	1	0	0	0	0	-0.002	t	94956	TTGNAAAPSTL	Oryctolagus cuniculus
1	1.00	1	1	0	0	0	0	-0.002	t	67187	TVTTTTKGENFTETDIKI	Oryctolagus cuniculus
1	1.00	1	1	0	0	0	0	-0.002	t	78253	QGGCGRGWAFSATGAIEA	Mus musculus
1	1.00	1	1	0	0	0	0	-0.002	t	66539	TTASGKLIT	Mus musculus
1	1.00	1	1	0	0	0	0	-0.002	t	66539	TTASGKLIT	Mus musculus
1	1.00	1	1	0	0	0	0	-0.002	t	79648	SPLLGCIGSTCA	Mus musculus
1	1.00	1	1	0	0	0	0	-0.002	t	60526	SQPNTEQSPAIVLPF	Mus musculus
1	1.00	1	1	0	0	0	0	-0.002	t	65974	TQYERE	Mus musculus

1	1.00	1	1	0	0	0	0	-0.002	t	91100	AFQTPVHS	Mus musculus
1	1.00	1	1	0	0	0	0	-0.002	t	95058	AGLQND	Mus musculus
1	1.00	1	1	0	0	0	0	-0.002	t	94430	AIFD	Mus musculus
1	1.00	1	1	0	0	0	0	-0.002	t	66841	TTTCFRDTDTGK	Mus musculus
1	1.00	1	1	0	0	0	0	-0.002	t	75791	YSLFQKEKMVL	Aotus nancymaae
1	1.00	1	1	0	0	0	0	-0.002	t	73365	YAHHAHHA	Mus musculus
1	1.00	1	1	0	0	0	0	-0.002	t	96701	NQSLAFIRKSDEL	Oryctolagus cuniculus
1	1.00	1	1	0	0	0	0	-0.002	t	63217	TDQMEVDSGPRKRPLRGG	Homo sapiens
1	1.00	1	1	0	0	0	0	-0.002	t	61818	STPGIVI	Oryctolagus cuniculus
1	1.00	1	1	0	0	0	0	-0.002	t	73889	YFRTLEDAAH	Mus musculus
1	1.00	1	1	0	0	0	0	-0.002	t	60663	SRGAPGGGFVPSLQGVP	Homo sapiens
1	1.00	1	1	0	0	0	0	-0.002	t	64587	TKINADNPVPILELE	Mus musculus
1	1.00	1	1	0	0	0	0	-0.002	t	68969	VIGHEITHGFD	Oryctolagus cuniculus

1	1.00	1	1	0	0	0	0	-0.002	t	94884	MRRAHHRRRRASHRRMRGG	Mus musculus
1	1.00	1	1	0	-0.004	0	0	-0.002	t	64698	TKWCPPGQLVNLHDFRSDEI	Oryctolagus cuniculus
1	1.00	1	1	0	0	0	0	-0.002	t	97035	VGINYWLAHK	Homo sapiens
1	1.00	1	1	0	0	0	0	-0.002	t	60595	SQWNKPSKPKT	Mus musculus
1	1.00	1	1	0	0	0	0	-0.002	t	71681	VVGYGVWPAY	Mus musculus
1	1.00	1	1	0	0	0	0	-0.002	t	97075	VSCYGKTKCTASNKNR	Oryctolagus cuniculus
1	1.00	1	1	0	0	0	0	-0.002	t	63758	TFTNPVVSTTQWSAP	Oryctolagus cuniculus
1	1.00	1	1	0	0	0	0	-0.002	t	65920	TQQPFPLQPQ	Mus musculus
1	1.00	1	1	0	0	0	0	-0.002	t	94041	TKRFKINAI	Mus musculus
1	1.00	1	1	0	0	0	0	-0.002	t	63146	TDELNQQRDDITRAI	Oryctolagus cuniculus
1	1.00	1	1	0	0	0	0	-0.002	t	96102	APPLLPH	Homo sapiens
1	1.00	1	1	0	0	0	0	-0.002	t	63760	TFTVKVGDKELFT	Oryctolagus cuniculus
1	1.00	1	1	0	0	0	0	-0.002	t	70937	VSFQYNAVGPYLNINFFDSS	Mus musculus
1	1.00	1	1	0	0	0	0	-0.002	t	65409	TNRNPQ	Oryctolagus cuniculus

1	1.00	1	1	0	0	0	0	-0.002	t	68813	VGVIKSIAP ASKNL	Oryctola gus cuniculu s
1	1.00	1	1	0	0	0	0	-0.002	t	71212	VSVTDHKK WQFNSPF VP	Mus musculu s
1	1.00	1	1	0	0	0	0	-0.002	t	66357	TSPFTEEYL LPPK	Mus musculu s
1	1.00	1	1	0	0	0	0	-0.002	t	97177	AELPF	Mus musculu s
1	1.00	1	1	0	0	0	0	-0.002	t	75532	YQPQQPF PQP	Mus musculu s
1	1.00	1	1	0	0	0	0	-0.002	t	64587	TKINADNP VPILELE	Mus musculu s
1	1.00	1	1	0	0	0	0	-0.002	t	65050	TLRVYK	Mus musculu s
1	1.00	1	1	0	0	0	0	-0.002	t	60535	SQQPQQP FPQ	Mus musculu s
1	1.00	1	1	0	0	0	0	-0.002	t	51906	QPQPFPP QLP	Mus musculu s
1	1.00	1	1	0	-0.003	0	0	-0.002	t	51454	QLNPKDN DVEL	Oryctola gus cuniculu s
1	1.00	1	1	0	0	0	0	-0.002	t	51001	QHGHMH GS	Mus musculu s
1	1.00	1	1	0	0	0	0	-0.002	t	51001	QHGHMH GS	Mus musculu s
1	1.00	1	1	0	0	0	0	-0.002	t	37055	LKVIRK	Mus musculu s
1	1.00	1	1	0	0	0	0	-0.002	t	38313	LNPTIA	Mus musculu s

1	1.00	1	1	0	0	0	0	-0.002	t	60348	SPYQPQQPFP	Mus musculus
1	1.00	1	1	0	0	0	0	-0.002	t	37055	LKVIRK	Homo sapiens
1	1.00	1	1	0	0	0	0	-0.002	t	51906	QPQPFPPQLP	Mus musculus
1	1.00	1	1	0	0	0	0	-0.002	t	39916	LTGISICNPGSSLC	Oryctolagus cuniculus
1	1.00	1	1	0	0	0	0	-0.002	t	44742	NLIESEHSNNNN	Mus musculus
1	1.00	1	1	0	0	0	0	-0.002	t	51912	QPQPFRPQQP	Mus musculus
1	1.00	1	1	0	0	0	0	-0.002	t	56469	RWAPTGAPADVTDQL	Mus musculus
1	1.00	1	1	0	0	0	0	-0.002	t	45420	NPNDPP	Mus musculus
1	1.00	1	1	0	0	0	0	-0.002	t	52086	QQPFPQQPQQ	Mus musculus
1	1.00	1	1	0	0	0	0	-0.002	t	51828	QPFPQPQQTF	Mus musculus
1	1.00	1	1	0	0	0	0	-0.002	t	48715	PNVDPN	Mus musculus
1	1.00	1	1	0	0	0	0	-0.002	t	51463	QLPFPQQPQQ	Mus musculus
1	1.00	1	1	0	0	0	0	-0.002	t	46795	PAFLGCSSRFSGSFSGVEP	Mus musculus
1	1.00	1	1	0	0	0	0	-0.002	t	43247	NANPNANPNANPNANPNA	Oryctolagus cuniculus

1	1.00	1	1	0	0	0	0	-0.002	t	52157	QQTFPQPQQT	Mus musculus
1	1.00	1	1	0	0	0	0	-0.002	t	40922	LYREFD	Mus musculus
1	1.00	1	1	0	0	0	0	-0.002	t	49928	PVPAPRRKVGSDCGS	Mus musculus
1	1.00	1	1	0	0	0	0	-0.002	t	49108	PQQQPFPQPQ	Mus musculus
1	1.00	1	1	0	0	0	0	-0.002	t	48933	PPTAPPLLPHSNL	Homo sapiens
1	1.00	1	1	0	0	0	0	-0.002	t	48912	PPSDIKGKYVKEVEVK	Oryctolagus cuniculus
1	1.00	1	1	0	0	0	0	-0.002	t	58402	SHRELWAK	Mus musculus
1	1.00	1	1	0	0	0	0	-0.002	t	38313	LNPTIA	Mus musculus
1	1.00	1	1	0	0	0	0	-0.002	t	58582	SIKEDVQF	Mus musculus
1	1.00	1	1	0	0	0	0	-0.002	t	38313	LNPTIA	Mus musculus
1	1.00	1	1	0	0	0	0	-0.002	t	51942	QPQRPFGQQP	Mus musculus
1	1.00	1	1	0	0	0	0	-0.002	t	49967	PVSLGGDVPNS	Mus musculus
1	1.00	1	1	0	0	0	0	-0.002	t	49119	PQRPQQPFPQ	Mus musculus
1	1.00	1	1	0	0	0	0	-0.002	t	43246	NANPNANPNANPNANP	Mus musculus

1	1.00	1	1	0	0	0	0	-0.002	t	51939	QPQQTFPQPQ	Mus musculus
1	1.00	1	1	0	0	0	0	-0.002	t	46371	NVESQPGGQPNT	Mus musculus
1	1.00	1	1	0	0	0	0	-0.002	t	52161	QQTQQPFPLQ	Mus musculus
1	1.00	1	1	0	0	0	0	-0.002	t	45422	NPNEPSNPNEPSNPNEPS	Mus musculus
1	1.00	1	1	0	0	0	0	-0.002	t	48690	PNPNDP	Mus musculus
1	1.00	1	1	0	0	0	0	-0.002	t	55261	RPPNAPIL	Mus musculus
1	1.00	1	1	0	0	0	0	-0.002	t	58833	SKGATKEAST	Homo sapiens
1	1.00	1	1	0	0	0	0	-0.002	t	45422	NPNEPSNPNEPSNPNEPS	Mus musculus
1	1.00	1	1	0	0	0	0	-0.002	t	45422	NPNEPSNPNEPSNPNEPS	Mus musculus
1	1.00	1	1	0	0	0	0	-0.002	t	45422	NPNEPSNPNEPSNPNEPS	Mus musculus
1	1.00	1	1	0	0	0	0	-0.002	t	49099	PQQPQQSFPQ	Mus musculus
1	1.00	1	1	0	0	0	0	-0.002	t	55305	RPQQPFPLQP	Mus musculus
1	1.00	1	1	0	0	0	0	-0.002	t	46786	PAEVTQLKGLS	Mus musculus
1	1.00	1	1	0	0	0	0	-0.002	t	52445	QSTVAELQRLKMKVGKTREL	Cavia porcellus
1	1.00	1	1	0	0	0	0	-0.002	t	58863	SKKHKDNEDAESVK	Homo sapiens
1	1.00	1	1	0	0	0	0	-0.002	t	47045	PCSICSNNPTCWAICK	Homo sapiens

1	1.00	1	1	0	0	0	0	-0.002	t	59922	SNTFINNA	Mus musculus
1	1.00	1	1	0	0	0	0	-0.002	t	52088	QQPFPQRPQQ	Mus musculus
1	1.00	1	1	0	0	0	0	-0.002	t	38946	LQPGKTS	Mus musculus
1	1.00	1	1	0	0	0	0	-0.002	t	50814	QFQGQQQPFP	Mus musculus
1	1.00	1	1	0	0	0	0	-0.002	t	50814	QFQGQQQPFP	Mus musculus
1	1.00	1	1	0	0	0	0	-0.002	t	51772	QNTEEMENLSDRVAS	Mus musculus
1	1.00	1	1	0	0	0	0	-0.002	t	51772	QNTEEMENLSDRVAS	Mus musculus
1	1.00	1	1	0	0	0	0	-0.002	t	52085	QQPFPQPQQQ	Mus musculus
1	1.00	1	1	0	0	0	0	-0.002	t	47045	PCSICSNNPTCWAICK	Mus musculus
1	1.00	1	1	0	0	0	0	-0.002	t	46868	PANCPKS	Mus musculus
1	1.00	1	1	0	0	0	0	-0.002	t	40739	LYDSYAYYG	Mus musculus
1	1.00	1	1	0	0	0	0	-0.002	t	47045	PCSICSNNPTCWAICK	Mus musculus
1	1.00	1	1	0	0	0	0	-0.002	t	45443	NPNVDP	Mus musculus

1	1.00	1	1	0	-0.003	0	0	-0.002	t	38919	LQNDPTTNVA	Oryctolagus cuniculus
1	1.00	1	1	0	0	0	0	-0.002	t	53977	RGSGPEP	Mus musculus
1	1.00	1	1	0	0	0	0	-0.002	t	49576	PTAGAQIAA	Mus musculus
1	1.00	1	1	0	0	0	0	-0.002	t	38919	LQNDPTTNVA	Oryctolagus cuniculus
1	1.00	1	1	0	0.012	0	0	-0.002	t	43233	NANP	Mus musculus
1	1.00	1	1	0	0	0	0	-0.002	t	59922	SNTFINNA	Mus musculus
1	1.00	1	1	0	0	0	0	-0.002	t	43233	NANP	Mus musculus
1	1.00	1	1	0	0	0	0	-0.002	t	55307	RPQQPFPQQP	Mus musculus
1	1.00	1	1	0	0	0	0	-0.002	t	49097	PQQPQQPFPQ	Mus musculus
1	1.00	1	1	0	0	0	0	-0.002	t	51772	QNTEEMENLSDRVAS	Mus musculus
1	1.00	1	1	0	0	0	0	-0.002	t	38313	LNPTIA	Mus musculus
1	1.00	1	1	0	0	0	0	-0.002	t	39504	LSN	Mus musculus
1	1.00	1	1	0	0	0	0	-0.002	t	55193	RPKPG	Gallus gallus

1	1.00	1	1	0	0	0	0	-0.002	t	53355	RDGQLTIK AERTEQKD FDGR	Mus musculu s
1	1.00	1	1	0	0	0	0	-0.002	t	41183	MCAVRDP AFSRSRLS	Mus musculu s
1	1.00	1	1	0	0	0	0	-0.002	t	40718	LYATFDEF PPP	Oryctola gus cuniculu s
1	1.00	1	1	0	0	0	0	-0.002	t	53859	RGETPGQL LPEIMQ	Oryctola gus cuniculu s
1	1.00	1	1	0	0	0	0	-0.002	t	52148	QQSFPQQ PQR	Mus musculu s
1	1.00	1	1	0	0	0	0	-0.002	t	41921	MLDMVD GRP	Mus musculu s
1	1.00	1	1	0	0	0	0	-0.002	t	42984	MVQVVYD	Oryctola gus cuniculu s
1	1.00	1	1	0	0	0	0	-0.002	t	52089	QQPFPQS QQP	Mus musculu s
1	1.00	1	1	0	0	0	0	-0.002	t	49110	PQQSFPQ PQH	Mus musculu s
1	1.00	1	1	0	0	0	0	-0.002	t	49107	PQQQFPQ TQQ	Mus musculu s
1	1.00	1	1	0	0	0	0	-0.002	t	44852	NLQKDEP NGRASDT	Oryctola gus cuniculu s
1	1.00	1	1	0	0	0	0	-0.002	t	38918	LQND	Mus musculu s
1	1.00	1	1	0	0	0	0	-0.002	t	50396	QAYQQGV TVD	Oryctola gus cuniculu s

1	1.00	1	1	0	0	0	0	-0.002	t	41870	MKNYLSF GMFALL	Mus musculu s
1	1.00	1	1	0	0	0	0	-0.002	t	47549	PFPQRPQ QPF	Mus musculu s
1	1.00	1	1	0	0	0	0	-0.002	t	38918	LQND	Mus musculu s
1	1.00	1	1	0	0	0	0	-0.002	t	58404	SHRLHNT MPSES	Mus musculu s
1	1.00	1	1	0	0	0	0	-0.002	t	52084	QQPFPQP QQP	Mus musculu s
1	1.00	1	1	0	0	0	0	-0.002	t	38950	LQPQQPF PQQ	Mus musculu s
1	1.00	1	1	0	0	0	0	-0.002	t	39070	LREGVTLG VGASFDT QKL	Oryctola gus cuniculu s
1	1.00	1	1	0	0	0	0	-0.002	t	38953	LQPQQSFP QP	Mus musculu s
1	1.00	1	1	0	0	0	0	-0.002	t	57880	SFRGSAQG IEGS	Homo sapiens
1	1.00	1	1	0	0	0	0	-0.002	t	54393	RKLWCHH FC	Mus musculu s
1	1.00	1	1	0	0	0	0	-0.002	t	60170	SPNVSVPS SSSTPLLY	Oryctola gus cuniculu s
1	1.00	1	1	0	0	0	0	-0.002	t	60170	SPNVSVPS SSSTPLLY	Oryctola gus cuniculu s
1	1.00	1	1	0	0	0	0	-0.002	t	44554	NKND	Mus musculu s

1	1.00	1	1	0	0	0	0	-0.002	t	46758	PADAQSDCKILCFVSACNDF	Mus musculus
1	1.00	1	1	0	0	0	0	-0.002	t	54393	RKLWCHHFC	Mus musculus
1	1.00	1	1	0	0	0	0	-0.002	t	39169	LRPTFDTRLMRLEDEMKEGR	Mus musculus
1	1.00	1	1	0	0.004	0	0	-0.002	t	54393	RKLWCHHFC	Mus musculus
1	1.00	1	1	0	-0.003	0	0	-0.002	t	60170	SPNVSVPSSSSTPLLY	Oryctolagus cuniculus
1	1.00	1	1	0	0	0	0	-0.002	t	58990	SLAELLNAGLGGS	Mus musculus
1	1.00	1	1	0	0	0	0	-0.002	t	48906	PPRRVS	Mus musculus
1	1.00	1	1	0	0	0	0	-0.002	t	39167	LRPTFDTRLM	Mus musculus
1	1.00	1	1	0	0.008	0	0	-0.002	t	53839	RGDLQVLAQK	Cavia porcellus
1	1.00	1	1	0	0.008	0	0	-0.002	t	53839	RGDLQVLAQK	Cavia porcellus
1	1.00	1	1	0	0	0	0	-0.002	t	51929	QPQQPQQSFP	Mus musculus
1	1.00	1	1	0	0	0	0	-0.002	t	57874	SFQQPQQQYP	Mus musculus
1	1.00	1	1	0	0	0	0	-0.002	t	57874	SFQQPQQQYP	Mus musculus

1	1.00	1	1	0	0	0	0	-0.002	t	48670	PNLRGDLQVLAQKVARTLP	Oryctolagus cuniculus
1	1.00	1	1	0	0	0	0	-0.002	t	43242	NANPNANPNANP	Homo sapiens
1	1.00	1	1	0	0	0	0	-0.002	t	52090	QQPFSQPQQP	Mus musculus
1	1.00	1	1	0	0	0	0	-0.002	t	52850	QWNKPSKPKTNM	Oryctolagus cuniculus
1	1.00	1	1	0	0	0	0	-0.002	t	55365	RPVDQYS	Mus musculus
1	1.00	1	1	0	0	0	0	-0.002	t	37755	LLRRQVNEVA	Oryctolagus cuniculus
1	1.00	1	1	0	0	0	0	-0.002	t	40579	LVSFGVWIRTPPAYRPPN	Mus musculus
1	1.00	1	1	0	0	0	0	-0.002	t	52865	QWVMAGLVSAA	Mus musculus
1	1.00	1	1	0	0	0	0	-0.002	t	40579	LVSFGVWIRTPPAYRPPN	Mus musculus
1	1.00	1	1	0	0	0	0	-0.002	t	38314	LNPTIAGAGDV	Oryctolagus cuniculus
1	1.00	1	1	0	0	0	0	-0.002	t	41169	MAVPAFSDIAKSANDLLNKD	Oryctolagus cuniculus
1	1.00	1	1	0	0	0	0	-0.002	t	49629	PTGDRHKQPLVAPAKQLL	Mus musculus
1	1.00	1	1	0	0	0	0	-0.002	t	43242	NANPNANPNANP	Homo sapiens

1	1.00	1	1	0	0	0	0	-0.002	t	51896	QPQLPFPQQP	Mus musculus
1	1.00	1	1	0	0	0	0	-0.002	t	47546	PFPQPQQQLP	Mus musculus
1	1.00	1	1	0	0	0	0	-0.002	t	49629	PTGDRHKQPLVAPAKQLL	Mus musculus
1	1.00	1	1	0	0	0	0	-0.002	t	50929	QGPGAP	Mus musculus
1	1.00	1	1	0	0	0	0	-0.002	t	46276	NTTASDQLLVENAAGHRVA	Cavia porcellus
1	1.00	1	1	0	0	0	0	-0.002	t	49298	PSAPPLPPVADLPQPGLRR	Cavia porcellus
1	1.00	1	1	0	0	0	0	-0.002	t	55106	RPADKTRHKFPTNINKQ	Oryctolagus cuniculus
1	1.00	1	1	0	0	0	0	-0.002	t	47545	PFPQPQQQFP	Mus musculus
1	1.00	1	1	0	0	0	0	-0.002	t	42493	MRQLDTNVER	Homo sapiens
1	1.00	1	1	0	0	0	0	-0.002	t	40074	LTPANLIRGDNA	Oryctolagus cuniculus
1	1.00	1	1	0	0	0	0	-0.002	t	41019	MADPNRFRG	Mus musculus
1	1.00	1	1	0	0	0	0	-0.002	t	56686	RYYRE	Mus musculus
1	1.00	1	1	0	-0.003	0	0	-0.002	t	49005	PQGPGAPQGPGAPQGPGAP	Oryctolagus cuniculus
1	1.00	1	1	0	0	0	0	-0.002	t	47653	PGFTVMAAIL	Mus musculus

1	1.00	1	1	0	0	0	0	-0.002	t	38314	LNPTIAGAGDV	Oryctolagus cuniculus
1	1.00	1	1	0	0	0	0	-0.002	t	51925	QPQQPFPQSQ	Mus musculus
1	1.00	1	1	0	0	0	0	-0.002	t	49092	PQQPFSQQPQ	Mus musculus
1	1.00	1	1	0	0	0	0	-0.002	t	56610	RYPGQGSPGGNRYPPQG	Mus musculus
1	1.00	1	1	0	0	0	0	-0.002	t	59922	SNTFINNA	Mus musculus
1	1.00	1	1	0	0	0	0	-0.002	t	58417	SHSQWNKPSKPKTNMK	Oryctolagus cuniculus
1	1.00	1	1	0	0	0	0	-0.002	t	58916	SKPKTNMKHM	Mus musculus
1	1.00	1	1	0	0	0	0	-0.002	t	49006	PQGQGKVT	Oryctolagus cuniculus
1	1.00	1	1	0	0	0	0	-0.002	t	45252	NNSTNSGI	Mus musculus
1	1.00	1	1	0	-0.003	0	0	-0.002	t	49006	PQGQGKVT	Oryctolagus cuniculus
1	1.00	1	1	0	0	0	0	-0.002	t	45721	NRQDFTQDPG	Mus musculus
1	1.00	1	1	0	0	0	0	-0.002	t	58417	SHSQWNKPSKPKTNMK	Oryctolagus cuniculus
1	1.00	1	1	0	0	0	0	-0.002	t	40104	LTPKELD	Homo sapiens
1	1.00	1	1	0	0	0	0	-0.002	t	52121	QQQPFPPQQP	Mus musculus

1	1.00	1	1	0	0	0	0	-0.002	t	39465	LSKSFLEA GGVSK	Oryctola gus cuniculu s
1	1.00	1	1	0	0	0	0	-0.002	t	56610	RYPGQGS PGGNRYP PQG	Oryctola gus cuniculu s
1	1.00	1	1	0	0	0	0	-0.002	t	39423	LSHNPGAS ALL	Mus musculu s
1	1.00	1	1	0	0	0	0	-0.002	t	51924	QPQQPFP QRP	Mus musculu s
1	1.00	1	1	0	0	0	0	-0.002	t	43481	NDLALPW KHEGAQN WNNAER	Mus musculu s
1	1.00	1	1	0	0	0	0	-0.002	t	41826	MKHM	Mus musculu s
1	1.00	1	1	0	0	0	0	-0.002	t	56807	SAHGTSTG VRGP	Mus musculu s
1	1.00	1	1	0	0	0	0	-0.002	t	38522	LPHSNL	Rattus norvegic us
1	1.00	1	1	0	0	0	0	-0.002	t	51958	QPRKTKPV KSLPERK	Mus musculu s
1	1.00	1	1	0	0	0	0	-0.002	t	45405	NPNANP	Mus musculu s
1	1.00	1	1	0	0	0	0	-0.002	t	47544	PFPQPQQ PFC	Mus musculu s
1	1.00	1	1	0	0	0	0	-0.002	t	49961	PVSGPFGP LPSLAFSA VPAD	Mus musculu s
1	1.00	1	1	0	0	0	0	-0.002	t	36974	LKNKIFPKK KEDNQAV DT	Oryctola gus cuniculu s

1	1.00	1	1	0	0	0	0	-0.002	t	37657	LLPHSNLD HI	Oryctola gus cuniculu s
1	1.00	1	1	0	0	0	0	-0.002	t	41118	MASENKQ RPGSPGPT	Mus musculu s
1	1.00	1	1	0	0	0	0	-0.002	t	43411	NDDASVD FVAEPVK	Mus musculu s
1	1.00	1	1	0	0	0	0	-0.002	t	45104	NNFVHDC VNI	Oryctola gus cuniculu s
1	1.00	1	1	0	0	0	0	-0.002	t	43411	NDDASVD FVAEPVK	Mus musculu s
1	1.00	1	1	0	0	0	0	-0.002	t	46258	NTSDSQKE	Mus musculu s
1	1.00	1	1	0	0	0	0	-0.002	t	59729	SMNRGRG THPSLIW MC	Mus musculu s
1	1.00	1	1	0	0	0	0	-0.002	t	41430	MERVVEQ MCITQYQ RESQAY	Mus musculu s
1	1.00	1	1	0	0	0	0	-0.002	t	51084	QIISQQPF PL	Mus musculu s
1	1.00	1	1	0	0	0	0	-0.002	t	59227	SLKLMATL FSTYASAD	Mus musculu s
1	1.00	1	1	0	0	0	0	-0.002	t	41431	MERVVEQ MCVTQYQ KESQAY	Oryctola gus cuniculu s
1	1.00	1	1	0	0	0	0	-0.002	t	46258	NTSDSQKE	Mus musculu s
1	1.00	1	1	0	0	0	0	-0.002	t	52239	QRPFGQQ PEQ	Mus musculu s

1	1.00	1	1	0	0	0	0	-0.002	t	48854	PPPLSEAAPKPRAAA	Mus musculus
1	1.00	1	1	0	0	0	0	-0.002	t	52891	QYDSNGTKLE	Mus musculus
1	1.00	1	1	0	0	0	0	-0.002	t	38422	LPAYQDYTARAQVSE	Oryctolagus cuniculus
1	1.00	1	1	0	0	0	0	-0.002	t	38304	LNPKTNKWEDK	Homo sapiens
1	1.00	1	1	0	0	0	0	-0.002	t	37361	LLGPYELWELSH	Homo sapiens
1	1.00	1	1	0	0	0	0	-0.002	t	50945	QGQQQPFPPQ	Mus musculus
1	1.00	1	1	0	0	0	0	-0.002	t	50945	QGQQQPFPPQ	Mus musculus
1	1.00	1	1	0	0	0	0	-0.002	t	55048	RNQE	Mus musculus
1	1.00	1	1	0	0	0	0	-0.002	t	52083	QQPFPLQPQQ	Mus musculus
1	1.00	1	1	0	0	0	0	-0.002	t	43411	NDDASVDFVAEPVK	Mus musculus
1	1.00	1	1	0	0	0	0	-0.002	t	38825	LQDPRVRGLYFPAGG	Oryctolagus cuniculus
1	1.00	1	1	0	0	0	0	-0.002	t	43411	NDDASVDFVAEPVK	Mus musculus
1	1.00	1	1	0	0	0	0	-0.002	t	59229	SLKMADPNRFRGKDLP	Mus musculus
1	1.00	1	1	0	0	0	0	-0.002	t	50637	QELDCWHYQHYCVTSTKS	MD

1	1.00	1	1	0	0	0	0	-0.002	t	55671	RRPFNIGINNQQ	Homo sapiens
1	1.00	1	1	0	0	0	0	-0.002	t	48597	PNANPN	Mus musculus
1	1.00	1	1	0	0	0	0	-0.002	t	51922	QPQQPFPQPQ	Mus musculus
1	1.00	1	1	0	0	0	0	-0.002	t	57320	SDYEDRYYREN	Mus musculus
1	1.00	1	1	0	0	0	0	-0.002	t	47969	PIQPQQPFPQ	Mus musculus
1	1.00	1	1	0	0	0	0	-0.002	t	54348	RKGRGGANPVFRPTGGTEVF	Mus musculus
1	1.00	1	1	0	0	0	0	-0.002	t	41754	MINVQAKPAAAAS	Oryctolagus cuniculus
1	1.00	1	1	0	0	0	0	-0.002	t	47821	PHGPADAPPGSPAPPPPEHR	Homo sapiens
1	1.00	1	1	0	0	0	0	-0.002	t	49068	PQPQQPFCQQ	Mus musculus
1	1.00	1	1	0	0	0	0	-0.002	t	57306	SDVEGLQNDP	Oryctolagus cuniculus
1	1.00	1	1	0	-0.003	0	0	-0.002	t	57306	SDVEGLQNDP	Oryctolagus cuniculus
1	1.00	1	1	0	0	0	0	-0.002	t	41728	MIKASFDPTGAFKSPRYKSH	Oryctolagus cuniculus
1	1.00	1	1	0	0	0	0	-0.002	t	38522	LPHSNL	Homo sapiens
1	1.00	1	1	0	0	0	0	-0.002	t	41713	MIHFGND	Mus musculus

1	1.00	1	1	0	0	0	0	-0.002	t	38522	LPHSNL	Rattus norvegicus
1	1.00	1	1	0	0	0	0	-0.002	t	49297	PSAPPLPHVVDLPQLGPRR	Cavia porcellus
1	1.00	1	1	0	0	0	0	-0.002	t	41126	MASLIYRQLL	Mus musculus
1	1.00	1	1	0	0	0	0	-0.002	t	49085	PQQPFPQQPE	Mus musculus
1	1.00	1	1	0	0	0	0	-0.002	t	58802	SKDVKIEVYL	Homo sapiens
1	1.00	1	1	0	0	0	0	-0.002	t	54058	RHKQKIVAPVKQTL	Oryctolagus cuniculus
1	1.00	1	1	0	0	0	0	-0.002	t	45165	NNLMEIAEVD	Mus musculus
1	1.00	1	1	0	0	0	0	-0.002	t	51921	QPQQPFPLQP	Mus musculus
1	1.00	1	1	0	0	0	0	-0.002	t	49070	PQPQQQFPQP	Mus musculus
1	1.00	1	1	0	-0.003	0	0	-0.002	t	50934	QGPHVISRIYQTAT	Oryctolagus cuniculus
1	1.00	1	1	0	0	0	0	-0.002	t	52304	QSCSISNIETVIEFQQ	Mus musculus
1	1.00	1	1	0	0	0	0	-0.002	t	48620	PNDPPP	Mus musculus
1	1.00	1	1	0	0	0	0	-0.002	t	45643	NQWNKPSKPKTN	Oryctolagus cuniculus
1	1.00	1	1	0	0	0	0	-0.002	t	49071	PQPQQQLPLQ	Mus musculus

1	1.00	1	1	0	0	0	0	-0.002	t	39092	LRGDLQVL AQKVARTL	Cavia porcellu s
1	1.00	1	1	0	0	0	0	-0.002	t	48017	PIYGKTIST PSDYM	Mus musculu s
1	1.00	1	1	0	0	0	0	-0.002	t	50934	QGPHVISR IYQTAT	Mus musculu s
1	1.00	1	1	0	0	0	0	-0.002	t	38271	LNLTTASV PMLRWYA ERFCF	Mus musculu s
1	1.00	1	1	0	0	0	0	-0.002	t	48017	PIYGKTIST PSDYM	Mus musculu s
1	1.00	1	1	0	0	0	0	-0.002	t	48017	PIYGKTIST PSDYM	Mus musculu s
1	1.00	1	1	0	0	0	0	-0.002	t	57218	SDIKTTTSS HPVSSLT	Mus musculu s
1	1.00	1	1	0	0	0	0	-0.002	t	39092	LRGDLQVL AQKVARTL	Oryctola gus cuniculu s
1	1.00	1	1	0	0	0	0	-0.002	t	49368	PSFSDIPN PI	Homo sapiens
1	1.00	1	1	0	0	0	0	-0.002	t	43747	NESKYSNT FINNAYN MSIR	Mus musculu s
1	1.00	1	1	0	0	0	0	-0.002	t	37029	LKTAAESA	Mus musculu s
1	1.00	1	1	0	0	0	0	-0.002	t	49079	PQQPFCQ QPQ	Mus musculu s
1	1.00	1	1	0	0	0	0	-0.002	t	37315	LLGCIGSTC	Mus musculu s

1	1.00	1	1	0	0	0	0	-0.002	t	48006	PIVNKQSC SISNIETV	Mus musculu s
1	1.00	1	1	0	0	0	0	-0.002	t	51816	QPFPLQP QQP	Mus musculu s
1	1.00	1	1	0	0	0	0	-0.002	t	46026	NSVGANA PNADTIAS	Mus musculu s
1	1.00	1	1	0	0	0	0	-0.002	t	51908	QPQPFPS QQP	Mus musculu s
1	1.00	1	1	0	0	0	0	-0.002	t	46026	NSVGANA PNADTIAS	Mus musculu s
1	1.00	1	1	0	0	0	0	-0.002	t	43227	NANDPP	Mus musculu s
1	1.00	1	1	0	0	0	0	-0.002	t	49082	PQQPFPQ PQQ	Mus musculu s
1	1.00	1	1	0	0	0	0	-0.002	t	45202	NNNSTNS G	Mus musculu s
1	1.00	1	1	0	0	0	0	-0.002	t	38952	LQPQQPFS QQ	Mus musculu s
1	1.00	1	1	0	0	0	0	-0.002	t	49950	PVRVAWS SPVQNCEP WIPP	Mus musculu s
1	1.00	1	1	0	0	0	0	-0.002	t	52107	QQPQRPF AQQ	Mus musculu s
1	1.00	1	1	0	0	0	0	-0.002	t	51833	QPFRPQQ PYP	Mus musculu s
1	1.00	1	1	0	0	0	0	-0.002	t	45422	NPNEPSN PNEPSNP NEPS	Mus musculu s
1	1.00	1	1	0	0	0	0	-0.002	t	48001	PITNDQKK	Mus musculu s

1	1.00	1	1	0	0	0	0	-0.002	t	53009	QYNLAFSDRQSAQ	Mus musculus
1	1.00	1	1	0	0	0	0	-0.002	t	49291	PSAEECGYSDRVRSITLGNS	Mus musculus
1	1.00	1	1	0	0	0	0	-0.002	t	25649	IDPY	Mus musculus
1	1.00	1	1	0	0	0	0	-0.002	t	12632	EKASVPGGGDMGGMDF	Mus musculus
1	1.00	1	1	0	0	0	0	-0.002	t	11704	EEIVPNSVEQ	Homo sapiens
1	1.00	1	1	0	0	0	0	-0.002	t	24786	HSLGKWLGHPDKF	Mus musculus
1	1.00	1	1	0	0	0	0	-0.002	t	20051	GGTHSQWNKP	Mus musculus
1	1.00	1	1	0	0	0	0	-0.002	t	26372	IHFG	Mus musculus
1	1.00	1	1	0	0	0	0	-0.002	t	24006	HHPITDSDQTRLY	Oryctolagus cuniculus
1	1.00	1	1	0	0	0	0	-0.002	t	15911	FFTEEFKRKNKGKNLASSHR	Canis lupus
1	1.00	1	1	0	0	0	0	-0.002	t	19833	GGGGWGQGGSHSQWNK	Mus musculus
1	1.00	1	1	0	0	0	0	-0.002	t	24786	HSLGKWLGHPDKF	Mus musculus
1	1.00	1	1	0	0	0	0	-0.002	t	32902	KPTKKPTFKTTKKDLKPQ	Oryctolagus cuniculus
1	1.00	1	1	0	0	0	0	-0.002	t	31072	KGTHSLYSQIAGC	Mus musculus
1	1.00	1	1	0	0	0	0	-0.002	t	10868	DYEDRYYRE	Mus musculus

1	1.00	1	1	0	0	0	0	-0.002	t	10868	DYEDRYYRE	Mus musculus
1	1.00	1	1	0	0	0	0	-0.002	t	35530	LEIVPNSAEERL	Homo sapiens
1	1.00	1	1	0	0	0	0	-0.002	t	21791	GPSNSEQIPALTAVE	Rattus norvegicus
1	1.00	1	1	0	0	0	0	-0.002	t	19737	GGGAGAGGAGAGGGGR	Oryctolagus cuniculus
1	1.00	1	1	0	0	0	0	-0.002	t	32571	KNNNSTNSGI	Mus musculus
1	1.00	1	1	0	0	0	0	-0.002	t	35672	LEQWNLVIGFLFL	Mus musculus
1	1.00	1	1	0	0	0	0	-0.002	t	14992	EYEDLLKA	Mus musculus
1	1.00	1	1	0	0	0	0	-0.002	t	30036	KAYSNCYPYDVPDY	Oryctolagus cuniculus
1	1.00	1	1	0	0	0	0	-0.002	t	32783	KPLDKFGNIYDYHYEH	Mus musculus
1	1.00	1	1	0	0	0	0	-0.002	t	15122	EYYLN	Mus musculus
1	1.00	1	1	0	0	0	0	-0.002	t	32783	KPLDKFGNIYDYHYEH	Mus musculus
1	1.00	1	1	0	0	0	0	-0.002	t	24918	HTHADGEVHC	Mus musculus
1	1.00	1	1	0	0	0	0	-0.002	t	13272	ELSKDIGSES	Homo sapiens
1	1.00	1	1	0	0	0	0	-0.002	t	30236	KDRTLIEQK	Mus musculus
1	1.00	1	1	0	0	0	0	-0.002	t	11571	EEEDDDMGFGLFD	Homo sapiens

1	1.00	1	1	0	0	0	0	-0.002	t	35473	LEGAAR	Mus musculus
1	1.00	1	1	0	0	0	0	-0.002	t	11203	EATPAAEAPASEAPAAEAAP	Mus musculus
1	1.00	1	1	0	0	0	0	-0.002	t	19729	GGFSSDPGFLLRPRV	Mus musculus
1	1.00	1	1	0	0	0	0	-0.002	t	32783	KPLDKFGNIYDYHYEH	Mus musculus
1	1.00	1	1	0	0	0	0	-0.002	t	28290	IRFKDDSSYEEVN	Mus musculus
1	1.00	1	1	0	0	0	0	-0.002	t	24786	HSLGKWLGHPDKF	Mus musculus
1	1.00	1	1	0	0	0	0	-0.002	t	34806	LAHLTTTHARHLP	Mus musculus
1	1.00	1	1	0	0	0	0	-0.002	t	28694	ISQQPQQPFP	Mus musculus
1	1.00	1	1	0	0	0	0	-0.002	t	30716	KFDWNTPD	Mus musculus
1	1.00	1	1	0	0	0	0	-0.002	t	29852	KALENKKKQLSAGGKNLS	Homo sapiens
1	1.00	1	1	0	0	0	0	-0.002	t	24014	HHQPQQTFPQ	Mus musculus
1	1.00	1	1	0	0	0	0	-0.002	t	12900	EKVSRRSNFEF	Oryctolagus cuniculus
1	1.00	1	1	0	0	0	0	-0.002	t	30611	KESQAYYDGRR	Mus musculus
1	1.00	1	1	0	0	0	0	-0.002	t	14702	EVGKVA	Homo sapiens

1	1.00	1	1	0	0	0	0	-0.002	t	24694	HRMAWDMMM	Homo sapiens
1	1.00	1	1	0	0	0	0	-0.002	t	35923	LFNLWGPAFHERYPNVTITA	Mus musculus
1	1.00	1	1	0	0	0	0	-0.002	t	36123	LGGKFLE	Mus musculus
1	1.00	1	1	0	0	0	0	-0.002	t	31723	KKTTTKPTKKPTFKTTKK	Oryctolagus cuniculus
1	1.00	1	1	0	0	0	0	-0.002	t	18500	GAAALRAHVSGRRA	Oryctolagus cuniculus
1	1.00	1	1	0	0	0	0	-0.002	t	19868	GGGWGQPHGGGWGQPH	Oryctolagus cuniculus
1	1.00	1	1	0	0	0	0	-0.002	t	17365	FPQPQPFPPQ	Mus musculus
1	1.00	1	1	0	-0.003	0	0	-0.002	t	19868	GGGWGQPHGGGWGQPH	Oryctolagus cuniculus
1	1.00	1	1	0	0	0	0	-0.002	t	18994	GDGRQTITNPAYDE	Mus musculus
1	1.00	1	1	0	0	0	0	-0.002	t	36353	LGWLWG	Mus musculus
1	1.00	1	1	0	0	0	0	-0.002	t	13460	ENDVLNQETEEEMEK	Oryctolagus cuniculus
1	1.00	1	1	0	0	0	0	-0.002	t	19866	GGGWGQGGGTHNQWNK	Gallus gallus
1	1.00	1	1	0	0	0	0	-0.002	t	31160	KHVAGAAAAGAVVGGLGGY	Oryctolagus cuniculus
1	1.00	1	1	0	0	0	0	-0.002	t	14435	ETIYNTTLKY	Homo sapiens

1	1.00	1	1	0	0	0	0	-0.002	t	27999	IPQQTQQPFP	Mus musculus
1	1.00	1	1	0	0.001	0	0	-0.002	t	17642	FRRYQEER	Mus musculus
1	1.00	1	1	0	0	0	0	-0.002	t	19868	GGGWGQPHGGGWGQPH	Mus musculus
1	1.00	1	1	0	0	0	0	-0.002	t	21571	GPARRGAPY	Macaca mulatta
1	1.00	1	1	0	0	0	0	-0.002	t	35247	LDPAFR	Mus musculus
1	1.00	1	1	0	0	0	0	-0.002	t	32844	KPQEEKEKITKEILNGK	Rattus norvegicus
1	1.00	1	1	0	0	0	0	-0.002	t	14561	ETTET	Mus musculus
1	1.00	1	1	0	0	0	0	-0.002	t	30093	KCTSDQDEQFIPKGCSK	Oryctolagus cuniculus
1	1.00	1	1	0	0	0	0	-0.002	t	21868	GQGGSHSQWNKP	Oryctolagus cuniculus
1	1.00	1	1	0	0	0	0	-0.002	t	31555	KKGSSGYS	Mus musculus
1	1.00	1	1	0	0	0	0	-0.002	t	11819	EENVEHDA	Homo sapiens
1	1.00	1	1	0	0	0	0	-0.002	t	11819	EENVEHDA	Homo sapiens
1	1.00	1	1	0	-0.003	0	0	-0.002	t	32844	KPQEEKEKITKEILNGK	Oryctolagus cuniculus
1	1.00	1	1	0	0	0	0	-0.002	t	30863	KFTEPVKDIM	Mus musculus

1	1.00	1	1	0	0	0	0	-0.002	t	15887	FFRDEKEL	Mus musculus
1	1.00	1	1	0	0	0	0	-0.002	t	14013	ERKRR	Homo sapiens
1	1.00	1	1	0	0	0	0	-0.002	t	35420	LEDVRN	Mus musculus
1	1.00	1	1	0	0	0	0	-0.002	t	35413	LEDRSTQASLEEAILR	Mus musculus
1	1.00	1	1	0	0	0	0	-0.002	t	17366	FPQPQQPQQS	Mus musculus
1	1.00	1	1	0	0	0	0	-0.002	t	17333	FPLQPQQPFP	Mus musculus
1	1.00	1	1	0	0	0	0	-0.002	t	22280	GSAMSRPIIHFGSDYEDRYY	Mus musculus
1	1.00	1	1	0	0	0	0	-0.002	t	18588	GAGGGAGGAGAGGGAGGAGA	Homo sapiens
1	1.00	1	1	0	0	0	0	-0.002	t	21815	GPVEDAITAAIGRVA	Rattus norvegicus
1	1.00	1	1	0	0	0	0	-0.002	t	21816	GPVEESVERAMVRVA	Rattus norvegicus
1	1.00	1	1	0	0	0	0	-0.002	t	20854	GLFHKEKMILNEEEITKGA	Mus musculus
1	1.00	1	1	0	0	0	0	-0.002	t	35495	LEGTTNPNG	Homo sapiens
1	1.00	1	1	0	-0.008	0	0	-0.002	t	16551	FLDLMRAVA	Mus musculus
1	1.00	1	1	0	0	0	0	-0.002	t	16569	FLEGAAR	Mus musculus
1	1.00	1	1	0	0	0	0	-0.002	t	22134	GRLITVNPIVT	Mus musculus

1	1.00	1	1	0	0	0	0	-0.002	t	11081	EAIPALTAVETGHTSQV	Oryctolagus cuniculus
1	1.00	1	1	0	0	0	0	-0.002	t	35252	LDPHAFHLLL	Homo sapiens
1	1.00	1	1	0	0	0	0	-0.002	t	18385	FYEPLVRRC	Mus musculus
1	1.00	1	1	0	0	0	0	-0.002	t	10876	DYEKQINKLKN	Mus musculus
1	1.00	1	1	0	0	0	0	-0.002	t	10892	DYHRVMY	Oryctolagus cuniculus
1	1.00	1	1	0	0	0	0	-0.002	t	12843	EKPNLSSKRSE	Oryctolagus cuniculus
1	1.00	1	1	0	0	0	0	-0.002	t	21930	GQPGPSGAAQGQVISN	Mus musculus
1	1.00	1	1	0	0	0	0	-0.002	t	19696	GGDDPDHDPDHPHDL	Mus musculus
1	1.00	1	1	0	0	0	0	-0.002	t	21867	GQGGGTHNQWNKP	Oryctolagus cuniculus
1	1.00	1	1	0	0	0	0	-0.002	t	19833	GGGGWGQGGSHSQWNK	Oryctolagus cuniculus
1	1.00	1	1	0	0	0	0	-0.002	t	10529	DTYRYI	Mus musculus
1	1.00	1	1	0	0	0	0	-0.002	t	21928	GQPAVRNERATGS	Mus musculus
1	1.00	1	1	0	0	0	0	-0.002	t	11571	EEEDDDMGFGLFD	Homo sapiens
1	1.00	1	1	0	0	0	0	-0.002	t	18509	GAAILVAGLSGCSSNKSTTG	Mus musculus
1	1.00	1	1	0	0	0	0	-0.002	t	16101	FHENWPS	Mus musculus

1	1.00	1	1	0	0	0	0	-0.002	t	11497	EEAILRPLRVQGKRAK	Mus musculus
1	1.00	1	1	0	0	0	0	-0.002	t	13871	EQLIEKLIEDDKNSQ	Papio cynocephalus
1	1.00	1	1	0	-0.037	0	0	-0.002	t	13871	EQLIEKLIEDDKNSQ	Papio cynocephalus
1	1.00	1	1	0	0	0	0	-0.002	t	18833	GAVVGGLGGYML	Mus musculus
1	1.00	1	1	0	0	0	0	-0.002	t	24697	HRMQEAVEAERAGRGTGH	Mus musculus
1	1.00	1	1	0	0	0	0	-0.002	t	13984	ERESQAY	Mus musculus
1	1.00	1	1	0	0	0	0	-0.002	t	10639	DVKFPGGGQIVGGVYLLPRR	Mus musculus
1	1.00	1	1	0	-0.003	0	0	-0.002	t	19833	GGGGWGQGGSHSQWNK	Oryctolagus cuniculus
1	1.00	1	1	0	0	0	0	-0.002	t	26046	IFDT	Mus musculus
1	1.00	1	1	0	0	0	0	-0.002	t	27608	INDISHTQSVSSKQRVTGLD	Oryctolagus cuniculus
1	1.00	1	1	0	-0.003	0	0	-0.002	t	33859	KTSERSQPRGRRQPIPKARR	Oryctolagus cuniculus
1	1.00	1	1	0	0	0	0	-0.002	t	24600	HQPQQQFPQT	Mus musculus
1	1.00	1	1	0	0	0	0	-0.002	t	26046	IFDT	Mus musculus
1	1.00	1	1	0	0	0	0	-0.002	t	17376	FPQQPQRPFA	Mus musculus

1	1.00	1	1	0	0	0	0	-0.002	t	32844	KPQEEKEKITKEILNGK	Oryctolagus cuniculus
1	1.00	1	1	0	0.002	0	0	-0.002	t	32844	KPQEEKEKITKEILNGK	Rattus norvegicus
1	1.00	1	1	0	0	0	0	-0.002	t	17408	FPSPYYSPAFTKGEKVDL	Oryctolagus cuniculus
1	1.00	1	1	0	0	0	0	-0.002	t	26046	IFDT	Mus musculus
1	1.00	1	1	0	-0.003	0	0	-0.002	t	17408	FPSPYYSPAFTKGEKVDL	Oryctolagus cuniculus
1	1.00	1	1	0	0	0	0	-0.002	t	24842	HSQWNKPSKPKTNMK	Oryctolagus cuniculus
1	1.00	1	1	0	-0.003	0	0	-0.002	t	31330	KILPEVKEKHEF	Oryctolagus cuniculus
1	1.00	1	1	0	0	0	0	-0.002	t	31330	KILPEVKEKHEF	Oryctolagus cuniculus
1	1.00	1	1	0	0	0	0	-0.002	t	31067	KGTAPFGVAFGNS	Oryctolagus cuniculus
1	1.00	1	1	0	0.001	0	0	-0.002	t	27997	IPQPQWTP	Mus musculus
1	1.00	1	1	0	0	0	0	-0.002	t	30661	KEVPALTAVETGAT	Oryctolagus cuniculus
1	1.00	1	1	0	0	0	0	-0.002	t	27997	IPQPQWTP	Mus musculus
1	1.00	1	1	0	0	0	0	-0.002	t	21715	GPNNSEAIPALTAAE	Rattus norvegicus

1	1.00	1	1	0	0	0	0	-0.002	t	35310	LDTNGGT GTAIV	Oryctolagus cuniculus
1	1.00	1	1	0	0	0	0	-0.002	t	18529	GAATAAA GGYKA	Homo sapiens
1	1.00	1	1	0	0.004	0	0	-0.002	t	11241	ECFPWDR KL	Mus musculus
1	1.00	1	1	0	0	0	0	-0.002	t	21084	GLQN	Mus musculus
1	1.00	1	1	0	-0.003	0	0	-0.002	t	33859	KTSERSQP RGRRQPIP KARR	Oryctolagus cuniculus
1	1.00	1	1	0	0	0	0	-0.002	t	19735	GGFVQFV TSTRNA	Oryctolagus cuniculus
1	1.00	1	1	0	0	0	0	-0.002	t	35662	LEQPVSND LS	Oryctolagus cuniculus
1	1.00	1	1	0	0	0	0	-0.002	t	33163	KRIPNKKP GKKTTT	Oryctolagus cuniculus
1	1.00	1	1	0	0	0	0	-0.002	t	30661	KEVPALTA VETGAT	Oryctolagus cuniculus
1	1.00	1	1	0	0	0	0	-0.002	t	24168	HKYSWK	Mus musculus
1	1.00	1	1	0	0	0	0	-0.002	t	22305	GSDYEDRY YRENMHR YPNQ	Mus musculus
1	1.00	1	1	0	0	0	0	-0.002	t	10801	DWEDRYY RE	Oryctolagus cuniculus
1	1.00	1	1	0	0	0	0	-0.002	t	30661	KEVPALTA VETGAT	Oryctolagus cuniculus

1	1.00	1	1	0	0	0	0	-0.002	t	36307	LGRPDEDS SSSSSSC	Oryctolagus cuniculus
1	1.00	1	1	0	0	0	0	-0.002	t	27998	IPQQPQQ PFP	Mus musculus
1	1.00	1	1	0	0	0	0	-0.002	t	11815	EENVEENV EENVEENV	Homo sapiens
1	1.00	1	1	0	0	0	0	-0.002	t	32914	KPTTKQRQ NKPPNKP N	Oryctolagus cuniculus
1	1.00	1	1	0	0	0	0	-0.002	t	19269	GEGAPPA KRARTDQ ME	Homo sapiens
1	1.00	1	1	0	0.002	0	0	-0.002	t	21715	GPNNSEAI PALTAAE	Rattus norvegicus
1	1.00	1	1	0	0	0	0	-0.002	t	11815	EENVEENV EENVEENV	Homo sapiens
1	1.00	1	1	0	0	0	0	-0.002	t	7112	CTASNKNR GIIKTFSNG	Mus musculus
1	1.00	1	1	0	0	0	0	-0.002	t	9524	DNPASTT NKDK	Mus musculus
1	1.00	1	1	0	0	0	0	-0.002	t	1367	AFNQFGP NAGQR	Mus musculus
1	1.00	1	1	0	0	0	0	-0.002	t	10126	DSGDGRQ TITNP	Mus musculus
1	1.00	1	1	0	0	0	0	-0.002	t	9524	DNPASTT NKDK	Mus musculus
1	1.00	1	1	0	0	0	0	-0.002	t	7665	DAVP	Mus musculus
1	1.00	1	1	0	0	0	0	-0.002	t	4038	AQPVKIDN ASPAST	Oryctolagus cuniculus

1	1.00	1	1	0	0	0	0	-0.002	t	7851	DDPPATVYRYDSRPPEDV	Oryctolagus cuniculus
1	1.00	1	1	0	0	0	0	-0.002	t	8066	DELEAETQNVYAA	Aotus nancymaae
1	1.00	1	1	0	0	0	0	-0.002	t	5261	AVALADLGDFENSA	Oryctolagus cuniculus
1	1.00	1	1	0	0	0	0	-0.002	t	8076	DELKDQMEED	Mus musculus
1	1.00	1	1	0	0	0	0	-0.002	t	9661	DPKSLSKGEIASTFESQNST	Mus musculus
1	1.00	1	1	0	0	0	0	-0.002	t	4331	ASARGDLAHLTTTHARHLP	Mus musculus
1	1.00	1	1	0	0	0	0	-0.002	t	774	ADSVGGKD	Mus musculus
1	1.00	1	1	0	0	0	0	-0.002	t	9847	DQGPPLMTDGGGGDPH	Mus musculus
1	1.00	1	1	0	0	0	0	-0.002	t	7558	DAKDGKEIDTKHLPSTC	Oryctolagus cuniculus
1	1.00	1	1	0	0	0	0	-0.002	t	6338	CGQPVTRTDDDTVA	Oryctolagus cuniculus
1	1.00	1	1	0	0	0	0	-0.002	t	10064	DRVVKEMRRQLEMIDKLTT	Mus musculus
1	1.00	1	1	0	0	0	0	-0.002	t	9352	DMANPMSPVNKSFEIEVTCS	Mus musculus
1	1.00	1	1	0	0	0	0	-0.002	t	7318	CVTQYQKESQAYYD	Oryctolagus cuniculus
1	1.00	1	1	0	0	0	0	-0.002	t	431	AASGDGTITFTGKVIDAPCG	Mus musculus

1	1.00	1	1	0	0	0	0	-0.002	t	7674	DAVTELDKN	Mus musculus
1	1.00	1	1	0	0	0	0	-0.002	t	7532	DAHHAAD	Mus musculus
1	1.00	1	1	0	0	0	0	-0.002	t	709	ADLIAAQKLASKP	Mus musculus
1	1.00	1	1	0	0	0	0	-0.002	t	7675	DAVTPLRIFIVPPPAAEYEQ	Oryctolagus cuniculus
1	1.00	1	1	0	0	0	0	-0.002	t	7535	DAHHAHHA	Mus musculus
1	1.00	1	1	0	0	0	0	-0.002	t	6205	CETEFSKLY	Mus musculus
1	1.00	1	1	0	0	0	0	-0.002	t	2319	AKVNIKPLEDKIL	Oryctolagus cuniculus
1	1.00	1	1	0	0	0	0	-0.002	t	5481	AVNDT	Mus musculus
1	1.00	1	1	0	0	0	0	-0.002	t	145	AAGANPLGLKRGIEKAV	Mus musculus
1	1.00	1	1	0	0	0	0	-0.002	t	5109	ATPYHPPATP	Oryctolagus cuniculus
1	1.00	1	1	0	0	0	0	-0.002	t	5582	AVTRGTINDPQRAKE	Oryctolagus cuniculus
1	1.00	1	1	0	0	0	0	-0.002	t	3242	ANDPPP	Mus musculus
1	1.00	1	1	0	0	0	0	-0.002	t	8119	DEPAGE	Homo sapiens
1	1.00	1	1	0	0	0	0	-0.002	t	7208	CTSPYDGKYWSMYSRL	Oryctolagus cuniculus

1	1.00	1	1	0	0	0	0	-0.002	t	6093	CDCDRTAA	Mus musculus
1	1.00	1	1	0	0	0	0	-0.002	t	7851	DDPPATVYRYDSRPPEDV	Oryctolagus cuniculus
1	1.00	1	1	0	0	0	0	-0.002	t	8475	DGQPAGDRADGQPAGDRA	Mus musculus
1	1.00	1	1	0	0	0	0	-0.002	t	4401	ASFFD	Mus musculus
1	1.00	1	1	0	0	0	0	-0.002	t	5575	AVTPLGPPAAEY	Oryctolagus cuniculus
1	1.00	1	1	0	0	0	0	-0.002	t	9586	DPAFR	Mus musculus
1	1.00	1	1	0	0	0	0	-0.002	t	5597	AVVGGLGGY	Mus musculus
1	1.00	1	1	0	0	0	0	-0.002	t	9611	DPDKDVDIMV	Mus musculus
1	1.00	1	1	0	-0.003	0	0	-0.002	t	6249	CFPYCYPSESA	Oryctolagus cuniculus
1	1.00	1	1	0	0	0	0	-0.002	t	6015	CAPESIEFPVSEARVLED	Homo sapiens
1	1.00	1	1	0	0	0	0	-0.002	t	9516	DNNLVSGP	Oryctolagus cuniculus
1	1.00	1	1	0	0	0	0	-0.002	t	8132	DEQFIPK	Mus musculus
1	1.00	1	1	0	0	0	0	-0.002	t	6883	CQIYPPNANKIREALAQ	Oryctolagus cuniculus
1	1.00	1	1	0	0	0	0	-0.002	t	9585	DPAF	Mus musculus

1	1.00	1	1	0	0	0	0	-0.002	t	6455	CITQYERES QAYY	Mus musculu s
1	1.00	1	1	0	0	0	0	-0.002	t	8388	DGGGGHS HDSGHGG GD	Mus musculu s
1	1.00	1	1	0	0	0	0	-0.002	t	1725	AGWISPG G	Mus musculu s
1	1.00	1	1	0	0	0	0	-0.002	t	6068	CCPDKNKS	Mus musculu s
1	1.00	1	1	0	0	0	0	-0.002	t	7782	DDGKGDR SHKNEDES ALASK	Mus musculu s
1	1.00	1	1	0	0	0	0	-0.002	t	6226	CFGNSEY	Mus musculu s
1	1.00	1	1	0	0	0	0	-0.002	t	10168	DSLPHPQ QATDDSG H	Mus musculu s
1	1.00	1	1	0	0	0	0	-0.002	t	4507	ASKNLDLT NITHVE	Oryctola gus cuniculu s
1	1.00	1	1	0	0	0	0	-0.002	t	7491	DAEFRHDS GYEVHHQ K	Homo sapiens
1	1.00	1	1	0	0	0	0	-0.002	t	435	AASKSCPL PQVRALES LESL	Oryctola gus cuniculu s
1	1.00	1	1	0	0	0	0	-0.002	t	5609	AVVYNNE GTNVELG	Mus musculu s
1	1.00	1	1	0	0	0	0	-0.002	t	8607	DIDPYKEF G	Mus musculu s
1	1.00	1	1	0	0	0	0	-0.002	t	3952	AQEPPRQ	Mus musculu s
1	1.00	1	1	0	0	0	0	-0.002	t	5387	AVIRRANN VLKNEMK RYKGL	Oryctola gus cuniculu s

1	1.00	1	1	0	0	0	0	-0.002	t	7673	DAVTE	Mus musculus
1	1.00	1	1	0	0	0	0	-0.002	t	9754	DPRVRGLYF	Mus musculus
1	1.00	1	1	0	0	0	0	-0.002	t	1045	AELGGQFHHKSENG	Mus musculus
1	1.00	1	1	0	0	0	0	-0.002	t	9754	DPRVRGLYF	Mus musculus
1	1.00	1	1	0	0	0	0	-0.002	t	952	AEGQLG	Mus musculus
1	1.00	1	1	0	0	0	0	-0.002	t	8644	DIFTNSRGKRASKG	Mus musculus
1	1.00	1	1	0	0	0	0	-0.002	t	8218	DEYDDAADAAGDRAP	Mus musculus
1	1.00	1	1	0	0	0	0	-0.002	t	8920	DKLAKEQKSKQNIGALKQ	Mus musculus
1	1.00	1	1	0	0	0	0	-0.002	t	8217	DEYCSPDHNCQSNCKDSGEG	Mus musculus
1	1.00	1	1	0	0	-0.006	0	0	t	149681	ELAEAKVLANRE	Mus musculus
1	1.00	1	1	0.01	0.01	0.01	0.01	0.01	t	134231	SIGAQTDFLSVFFSGYTFKH	Homo sapiens
1	1.00	1	1	0.01	0.01	0.006	0.01	0.01	t	52157	QQTFPQPQQT	Mus musculus
1	1.00	1	1	0.01	0.01	0.006	0.01	0.01	t	52157	QQTFPQPQQT	Mus musculus
1	1.00	1	1	-0.01	-0.01	-0.01	-0.01	-0.012	t	91100	AFQTPVHS	Mus musculus
1	1.00	1	1	0.01	0.01	0.01	0.01	0.011	t	57556	SEMPQLP	Mus musculus

1	1.00	1	1	-0.01	-0.01	-0.01	-0.01	-0.011	t	33794	KTNMKHM	Mus musculus
1	1.00	1	1	0.03	0.03	0.036	0.028	0.03	t	18039	FTPGPSQ	Homo sapiens
1	1.00	1	1	0	-0.002	-0.001	0.003	0	t	14905	EVWREEAYHAADIKD	Mus musculus
1	1.00	1	1	-0.01	-0.01	-0.01	-0.006	-0.01	t	30472	KEKLQERL	Homo sapiens
1	1.00	1	1	-0.04	-0.04	-0.034	-0.04	-0.043	t	79484	YMGAIWAGDW	Mus musculus
1	1.00	1	1	0	0	-0.012	0	0	t	121732	QAPEYRGRTELLKESIGE	Rattus norvegicus
1	1.00	1	1	0	0	0.007	0.003	0	t	47792	PGYPWPLYGNEG	Homo sapiens
1	1.00	1	1	0.02	0.022	0.009	0.011	0.017	t	6985	CSEYPKPDCTAE	Homo sapiens
1	1.00	1	1	0	0	0.009	0.003	-0.003	t	110821	DVRQQYESVAAKNLQEAEEW	Mus musculus
1	1.00	1	1	0	0	0.009	0.003	-0.003	t	110820	DVRQQYESVAAKNLQEAEEW	Mus musculus
1	1.00	1	1	0	0	0	0	-0.002	t	130473	ECIVYGKTALCT	Mus musculus
1	1.00	1	1	0	-0.004	0	0.003	-0.001	t	78411	GTKPTFHVEKGSNPNYLA	Oryctolagus cuniculus
1	1.00	1	1	0.04	0.04	0.04	0.04	0.04	t	153265	ILETLPANPPPASTN	Mus musculus
1	1.00	1	1	0.02	0.02	0.02	0.02	0.02	t	117664	VAARGRGRGMGRGNI	Homo sapiens
1	1.00	1	1	0.02	0.02	0.02	0.02	0.02	t	114970	VDCSRFPNAT	Homo sapiens

1	1.00	1	1	0.02	0.02	0.02	0.02	0.02	t	92973	IFKGSKFNDA	Ovis aries
1	1.00	1	1	0	0	0	0	0	t	110613	GPPGPQGATGPLGPK	Mus musculus
1	1.00	1	1	-0.05	-0.052	-0.06	-0.057	-0.05	t	12992	ELDLQARR	Mus musculus
1	1.00	1	1	0.02	0.02	0.02	0.02	0.019	t	25145	HWYDAEVL	Mus musculus
1	1.00	1	1	0	0.003	0.007	0.007	0	t	119466	IRGERA	Mus musculus
1	1.00	1	1	-0.02	-0.02	-0.015	-0.017	-0.018	t	36947	LKLNWF	Homo sapiens
1	1.00	1	1	0	0	-0.009	-0.004	0	t	135941	ITTQECANVVVG	Homo sapiens
1	1.00	1	1	-0.01	-0.01	-0.003	-0.01	-0.013	t	108825	AVLMLPVWACLS	Mus musculus
1	1.00	1	1	0	0	-0.017	0.002	0.002	t	136749	WMLRAAEEYSMNLGF	Homo sapiens
1	1.00	1	1	0	0	0	0	-0.001	t	61397	SSTKNLIYTSTLCELNV	Sus scrofa
1	1.00	1	1	0.01	0.012	0.02	0.013	0.01	t	140665	DTAAQI	Homo sapiens
1	1.00	1	1	-0.02	-0.022	-0.037	-0.023	-0.017	t	118151	SRFSWGAE	Rattus norvegicus
1	1.00	1	1	0.01	0.014	0.01	0.01	0.012	t	137402	FIDSYICQV	Homo sapiens
1	1.00	1	1	0.01	0.01	0.01	0.01	0.01	t	51473	QLPNVGIT	Oryctolagus cuniculus

1	1.00	1	1	-0.01	-0.01	-0.009	-0.013	-0.012	t	5512	AVPTGVAF WLYNDHD	Homo sapiens
1	1.00	1	1	0.02	0.022	0.018	0.017	0.02	t	96114	ASFAEAPF SN	Homo sapiens
1	1.00	1	1	0	0	-0.003	0	0	t	65296	TNCYKKR WRDHRGY RTE	Oryctola gus cuniculu s
1	1.00	1	1	0.01	0.01	0.01	0.01	0.01	t	42984	MVQVVYD	Oryctola gus cuniculu s
1	1.00	1	1	0	0	0	0.001	-0.001	t	130518	ITQSTQSF NDFTRVV	Mus musculu s
1	1.00	1	1	0	0	0	0.001	-0.001	t	66257	TSHHDSH GLHRV	Mus musculu s
1	1.00	1	1	0	0	0	0.001	-0.001	t	13481	ENHSPVNI AHKL	Mus musculu s
1	1.00	1	1	0	0.003	-0.011	0	0	t	111193	DGPAGQA EPDRAHY NI	Mus musculu s
1	1.00	1	1	0.02	0.02	0.02	0.02	0.019	t	144778	SGTHPFLD KSFS	Mus musculu s
1	1.00	1	1	-0.01	-0.012	-0.017	-0.01	-0.009	t	95029	KNTLRIAYL TEAKVEKL	Oryctola gus cuniculu s
1	1.00	1	1	0	0	0	0	-0.002	t	54860	RMAWDM M	Homo sapiens
1	1.00	1	1	-0.01	-0.01	-0.01	-0.01	-0.01	t	136823	ASIILEFFL	Mus musculu s
1	1.00	1	1	0	-0.004	-0.007	0.001	-0.001	t	131194	ALGAQKE AISPPDAA SAAP	Oryctola gus cuniculu s
1	1.00	1	1	0.05	0.047	0.041	0.05	0.05	t	79292	LKTDLELKY KQTADFS	Oryctola gus cuniculu s

1	1.00	1	1	0	0	0	0	-0.002	t	51925	QPQQPFPQSQ	Mus musculus
1	1.00	1	1	0	0.002	-0.007	-0.003	-0.001	t	118630	TLNLEDVQPHDLG	Homo sapiens
1	1.00	1	1	0.03	0.03	0.03	0.03	0.03	t	51837	QPGHLA	Homo sapiens
1	1.00	1	1	0.01	0.01	0.01	0.01	0.01	t	149087	NPTQSPP	Sus scrofa
1	1.00	1	1	-0.02	-0.022	-0.016	-0.021	-0.02	t	10075	DRYYREN	Mus musculus
1	1.00	1	1	0.05	0.05	0.043	0.05	0.05	t	119480	KQQKTENGAGDQ	Homo sapiens
1	1.00	1	1	0	0.002	0.002	0.003	0.001	t	119641	SEHETRLVAKLFKDYS	Homo sapiens
1	1.00	1	1	0.03	0.027	0.018	0.03	0.031	t	78394	GEAVTVTITDDNEEPIAPY	Oryctolagus cuniculus
1	1.00	1	1	0.01	0.01	0.01	0.01	0.009	t	135477	LGAGLG	Mus musculus
1	1.00	1	1	0.02	0.019	0.018	0.021	0.02	t	49872	PVGGQSSFYSDWYSPACGKA	Homo sapiens
1	1.00	1	1	0	-0.002	-0.001	-0.003	-0.002	t	7065	CSQNPLAELKCSVKSFEI	Mus musculus
1	1.00	1	1	-0.03	-0.025	-0.023	-0.03	-0.032	t	132912	VKAKVQP	Mus musculus
1	1.00	1	1	0	0	0	0	-0.002	t	74036	YGLFHKEKMLL	Mus musculus
1	1.00	1	1	0	0	0	0	-0.002	t	75791	YSLFQKEKMVL	Mus musculus
1	1.00	1	1	0	0	0	0	-0.002	t	75791	YSLFQKEKMVL	Mus musculus

1	1.00	1	1	0	0	0	0	-0.002	t	74035	YGLFHKEKMIL	Mus musculus
1	1.00	1	1	0.04	0.038	0.04	0.037	0.041	t	51147	QIVGGVYLL	Mus musculus
1	1.00	1	1	-0.01	-0.008	-0.01	-0.003	-0.011	t	132552	PSPLPHRDRD	Homo sapiens
1	1.00	1	1	-0.01	-0.01	-0.011	-0.01	-0.01	t	144803	TSAHSKLAVAAL	Mus musculus
1	1.00	1	1	-0.02	-0.02	-0.025	-0.02	-0.019	t	107218	PAGGPGFP	Rattus norvegicus
1	1.00	1	1	-0.01	-0.006	-0.019	-0.007	-0.01	t	136011	VPALTAVETGHT	Homo sapiens
1	1.00	1	1	-0.03	-0.03	-0.039	-0.03	-0.031	t	12852	EKQISDASRQ	Mus musculus
1	1.00	1	1	0.01	0.01	0.001	0.013	0.01	t	65797	TPVGLAVDTPWVEKESALSI	Homo sapiens
1	1.00	1	1	0	0	0	0.007	0.002	t	99134	DAEFRHDSGYEVHHQ	Mus musculus
1	1.00	1	1	0	0.002	0	0.001	0	t	153359	ALLPFKDHLPYPGGGS	Homo sapiens
1	1.00	1	1	0.03	0.03	0.025	0.03	0.03	t	122956	YYVFFEEQEDEIIGFG	Oryctolagus cuniculus
1	1.00	1	1	0.05	0.044	0.05	0.05	0.05	t	123375	KKDELKKSL	Oryctolagus cuniculus
1	1.00	1	1	0.05	0.044	0.05	0.05	0.05	t	123375	KKDELKKSL	Oryctolagus cuniculus
1	1.00	1	1	0.06	0.06	0.053	0.06	0.061	t	133707	STRKEEKQRNGT	Mus musculus

1	1.00	1	1	-0.02	-0.02	-0.02	-0.02	-0.021	t	117829	EKVKRTKDELEVIH	Homo sapiens
1	1.00	1	1	-0.02	-0.016	-0.014	-0.019	-0.02	t	32287	KMALYD	Homo sapiens
1	1.00	1	1	0.03	0.03	0.032	0.03	0.03	t	133634	LSQDVCTY	Mus musculus
1	1.00	1	1	0	-0.002	-0.005	0.003	0.002	t	58832	SKGANPVEIR	Homo sapiens
1	1.00	1	1	0	0.004	-0.005	0.003	0.002	t	58832	SKGANPVEIR	Homo sapiens
1	1.00	1	1	0.02	0.024	0.024	0.021	0.02	t	2237	AKLQDC	Homo sapiens
1	1.00	1	1	0	0	0	0	0	t	130454	CLFPNNSYC	Mus musculus
1	1.00	1	1	0	0	0	0	0	t	130454	CLFPNNSYC	Mus musculus
1	1.00	1	1	0	0	0	0	0	t	108261	ADASVPVMP	Homo sapiens
1	1.00	1	1	0.04	0.033	0.035	0.047	0.04	t	55187	RPKHPIKHQGLPQEVLNENL	Homo sapiens
1	1.00	1	1	-0.01	-0.014	-0.014	-0.011	-0.012	t	111792	SNPSAAGDWIRRNVV	Oryctolagus cuniculus
1	1.00	1	1	0	0.004	-0.002	0	-0.001	t	76195	YTTSTRGDLAHVTAT	Cavia porcellus
1	1.00	1	1	0	0.004	-0.002	0	-0.001	t	76195	YTTSTRGDLAHVTAT	Cavia porcellus
1	1.00	1	1	0.04	0.04	0.04	0.051	0.042	t	118448	KEISQEAREGTQGERCAV	Homo sapiens

1	1.00	1	1	-0.02	-0.016	-0.02	-0.013	-0.019	t	131904	HCQICHCD VVNLTCE	Homo sapiens
1	1.00	1	1	0.03	0.042	0.037	0.03	0.03	t	4702	ASQKIQLV	Pan troglody tes
1	1.00	1	1	0.01	0.008	0.01	0.013	0.01	t	63344	TEEIDEIES VTEEVVE	Mus musculu s
1	1.00	1	1	-0.04	-0.04	-0.041	-0.034	-0.04	t	144710	KNPWPNV DAH	Homo sapiens
1	1.00	1	1	0	0.014	0.005	0.006	0	t	60170	SPNVSVPS SSSTPLLY	Homo sapiens
1	1.00	1	1	0	0	0.005	0.006	0	t	60170	SPNVSVPS SSSTPLLY	Homo sapiens
1	1.00	1	1	-0.04	-0.04	-0.049	-0.04	-0.037	t	141683	KDWG	Mus musculu s
1	1.00	1	1	0	0	0	0	-0.002	t	45540	NQELAYFY PELFRQ	Homo sapiens
1	1.00	1	1	-0.03	-0.031	-0.03	-0.023	-0.03	t	72515	WGRRLMI GTAAAVVL PGLVG	Homo sapiens
1	1.00	1	1	-0.01	-0.01	-0.014	-0.01	-0.01	t	58382	SHLQLIYNL	Sus scrofa
1	1.00	1	1	-0.01	-0.01	-0.014	-0.01	-0.01	t	58382	SHLQLIYNL	Sus scrofa
1	1.00	1	1	0	0	0	0	-0.002	t	49068	PQPQQPF CQQ	Mus musculu s
1	1.00	1	1	0.02	0.022	0.014	0.021	0.02	t	64651	TKPVASDY WDGYSGQ LVCII	Homo sapiens

1	1.00	1	1	0.03	0.03	0.03	0.03	0.03	t	136855	LRPASAWTL	Mus musculus
1	1.00	1	1	0.03	0.03	0.03	0.03	0.03	t	103165	FEETFEVTAAAPVAV	Homo sapiens
1	1.00	1	1	0.03	0.03	0.03	0.03	0.03	t	115467	RFFVAPFPEVFGKEKVNELS	Homo sapiens
1	1.00	1	1	0.03	0.028	0.031	0.029	0.031	t	120436	DGTKVSIS	Rattus norvegicus
1	1.00	1	1	0	0	0	0	-0.002	t	150883	SDDEDLATS	Mus musculus
1	1.00	1	1	0	0	0	0	-0.002	t	135799	YGKDVKDLFDYAQE	Mus musculus
1	1.00	1	1	0	0	0	0	-0.002	t	136353	HGNAEVHASFFDIGGS	Oryctolagus cuniculus
1	1.00	1	1	0	0	0	0	-0.002	t	142913	MVLNEGTSGTA	Oryctolagus cuniculus
1	1.00	1	1	0	0	0	0	-0.002	t	130524	KFHPKDIPYQVW	Mus musculus
1	1.00	1	1	0	0	0	0	-0.002	t	99210	ELFTNRWNLQSLL	Oryctolagus cuniculus
1	1.00	1	1	0	-0.002	0	0	-0.002	t	72805	WLTEKEGSYP	Mus musculus
1	1.00	1	1	0	0	0	0	-0.002	t	97547	PFVPRADEPARKGKVH	Mus musculus
1	1.00	1	1	0	0	0	0	-0.002	t	36958	LKMADPNRFRGKD	Mus musculus
1	1.00	1	1	0	0	0	0	-0.002	t	21254	GMDGTQLDRW	Mus musculus
1	1.00	1	1	0	0	0	0	-0.002	t	11463	EDVNGIRKPK	Mus musculus

1	1.00	1	1	0	0	0	0	-0.002	t	21254	GMDGTQL DRW	Mus musculu s
1	1.00	1	1	0	0	0	0	-0.002	t	7674	DAVTELDK N	Mus musculu s
1	1.00	1	1	0.01	0.006	0.01	0.013	0.01	t	106313	CQQYGSS PQTFG	Oryctola gus cuniculu s
1	1.00	1	1	0	-0.004	0.003	-0.001	0	t	59854	SNKFADFL	Oryctola gus cuniculu s
1	1.00	1	1	0	-0.003	0	0	-0.001	t	43215	NALTQPV DQLLK	Mus musculu s
1	1.00	1	1	0.02	0.02	0.02	0.02	0.02	t	111577	NAMSPPA	Homo sapiens
1	1.00	1	1	0	0	0	0	0	t	108622	SHQESTRG RSRGRS	Homo sapiens
1	1.00	1	1	0	0	0	0	0	t	108622	SHQESTRG RSRGRS	Homo sapiens
1	1.00	1	1	0	-0.002	0	-0.001	0	t	59929	SNTNSVG A	Mus musculu s
1	1.00	1	1	-0.02	-0.018	-0.011	-0.013	-0.019	t	108895	EGNVFSC	Homo sapiens
1	1.00	1	1	0.02	0.02	0.012	0.02	0.02	t	65672	TPNSIAAIS M	Mus musculu s
1	1.00	1	1	-0.01	-0.01	-0.01	-0.01	-0.009	t	6924	CRGDDFP AWYC	Mus musculu s
1	1.00	1	1	-0.01	-0.008	-0.007	-0.009	-0.01	t	46194	NTNRRPQ	Homo sapiens
1	1.00	1	1	0.03	0.032	0.021	0.024	0.029	t	151046	RDQEGRM NYYWTLV E	Homo sapiens
1	1.00	1	1	0.03	0.028	0.019	0.033	0.033	t	108384	GIAGFKGE QGPKGEP	Mus musculu s
1	1.00	1	1	-0.02	-0.02	-0.02	-0.02	-0.018	t	39167	LRPTFDTR LM	Mus musculu s

1	1.00	1	1	0.02	0.02	0.02	0.02	0.02	t	123618	PPPGLAPG	Oryctolagus cuniculus
1	1.00	1	1	-0.01	-0.01	-0.01	-0.01	-0.011	t	153837	HGRLVTLKDIVLDLQP	Cavia porcellus
1	1.00	1	1	-0.01	-0.01	-0.003	-0.01	-0.01	t	30710	KFDRGYISPY	Oryctolagus cuniculus
1	1.00	1	1	0.01	0.01	0.01	0.01	0.009	t	137432	NHNYPPLSLLTF	Mus musculus
1	1.00	1	1	0.01	0.014	0.006	0.011	0.011	t	46858	PALTAAETG	Homo sapiens
1	1.00	1	1	0.01	0.012	0.007	0.011	0.008	t	103493	PPTPISVIFRSDKSG	Homo sapiens
1	1.00	1	1	0.04	0.042	0.04	0.047	0.041	t	141680	ITKHNDDEQYA	Homo sapiens
1	1.00	1	1	0.02	0.024	0.012	0.021	0.019	t	31116	KHIDAYKTFPPTEPKKDKKK	Homo sapiens
1	1.00	1	1	0.03	0.028	0.028	0.029	0.03	t	78752	NKGKWKEHIKEVTEKLKKA	Mus musculus
1	1.00	1	1	-0.03	-0.032	-0.036	-0.027	-0.028	t	38953	LQPQQSFPQP	Mus musculus
1	1.00	1	1	0	-0.004	-0.005	-0.001	0	t	78875	VLHSDYCIVPNKGGTSIGS	Oryctolagus cuniculus
1	1.00	1	1	0	-0.002	-0.005	-0.001	0	t	78875	VLHSDYCIVPNKGGTSIGS	Mus musculus
1	1.00	1	1	0.11	0.11	0.11	0.11	0.112	t	143682	EEIEKE	Mus musculus

1	1.00	1	1	0	0	0	0.003	0	t	117742	AKAQTDR EDLRTLLRY	Homo sapiens
1	1.00	1	1	0	0	0	0.003	0	t	117773	CNSRQTD REDELI	Homo sapiens
1	1.00	1	1	0	0	0	0.003	0	t	124604	QIERDTER DRFLSAPE AV	Homo sapiens
1	1.00	1	1	0.04	0.04	0.04	0.04	0.04	t	20144	GHIELGGK PALVPDKE VLYQ	Homo sapiens
1	1.00	1	1	0.02	0.02	0.02	0.02	0.02	t	78225	PFSNLDF M	Homo sapiens
1	1.00	1	1	0.02	0.02	0.02	0.02	0.02	t	93925	SMLDQSLS SL	Ovis aries
1	1.00	1	1	0	0	-0.005	0	0	t	59939	SNTVKVV GKGGHS	Homo sapiens
1	1.00	1	1	-0.02	-0.018	-0.03	-0.019	-0.018	t	47643	PGFGDRR KAM	Homo sapiens
1	1.00	1	1	0.05	0.05	0.039	0.05	0.05	t	42646	MSRIAAG DLESSVDD PRSEE	Homo sapiens
1	1.00	1	1	0.01	0.01	0.01	0.01	0.008	t	138622	SKDQFEFA LTAV	Oryctola gus cuniculu s
1	1.00	1	1	-0.01	-0.012	-0.002	-0.011	-0.01	t	25649	IDPY	Mus musculu s
1	1.00	1	1	-0.08	-0.082	-0.08	-0.081	-0.079	t	75195	YNMSIRRS	Mus musculu s
1	1.00	1	1	0.02	0.022	0.02	0.021	0.02	t	133553	EDMSYWH EWNP	Homo sapiens
1	1.00	1	1	0	-0.003	0.001	0	-0.003	t	52414	QSQLERA NLRPCEQ H	Oryctola gus cuniculu s

1	1.00	1	1	0.02	0.02	0.02	0.02	0.02	t	95155	TDHYLQWNVSE	Rattus norvegicus
1	1.00	1	1	-0.01	-0.01	-0.005	-0.007	-0.012	t	1465	AGDGEPPEDDDSATGLAFRT	Homo sapiens
1	1.00	1	1	-0.04	-0.04	-0.049	-0.04	-0.04	t	135911	DRVRSITLGNST	Homo sapiens
1	1.00	1	1	0.01	0.006	0.01	0.009	0.01	t	137858	FTPGTEYT	Oryctolagus cuniculus
1	1.00	1	1	0.02	0.022	0.013	0.017	0.02	t	46676	NYNVLMVPKP	Homo sapiens
1	1.00	1	1	0.07	0.066	0.07	0.067	0.068	t	142006	SDSAQGSDVSLTA	Oryctolagus cuniculus
1	1.00	1	1	-0.04	-0.044	-0.054	-0.041	-0.04	t	9154	DLKTNNEGLK	Oryctolagus cuniculus
1	1.00	1	1	0.03	0.026	0.026	0.033	0.03	t	12050	EFMEKECPFIKPEV	Oryctolagus cuniculus
1	1.00	1	1	0.03	0.033	0.029	0.03	0.032	t	34539	KYNGEEYLIL	Mus musculus
1	1.00	1	1	0.02	0.016	0.014	0.019	0.02	t	57505	SEKSEEINEKDLRKKSELQG	Oryctolagus cuniculus
1	1.00	1	1	0	0.003	0	0	0	t	73189	WVEWA	Equus caballus
1	1.00	1	1	-0.04	-0.04	-0.04	-0.04	-0.04	t	104149	NHYFLRS	Homo sapiens
1	1.00	1	1	-0.03	-0.03	-0.031	-0.03	-0.03	t	33818	KTPNSIAAIS	Mus musculus
1	1.00	1	1	-0.01	-0.01	-0.01	-0.01	-0.01	t	149116	SYAAVAS	Sus scrofa
1	1.00	1	1	0.01	0.002	0.009	0.01	0.01	t	24841	HSQWNKPSKP	Mus musculus

1	1.00	1	1	0.01	0.013	0.009	0.01	0.01	t	24841	HSQWNKPSKP	Mus musculus
1	1.00	1	1	-0.05	-0.046	-0.062	-0.053	-0.051	t	104993	SWRSKYGWTA	Homo sapiens
1	1.00	1	1	0.01	0.01	0.01	0.01	0.011	t	52094	QQPP	Mus musculus
1	1.00	1	1	-0.02	-0.023	-0.019	-0.02	-0.023	t	30260	KDTLRIAYLTEAKVEKL	Oryctolagus cuniculus
1	1.00	1	1	0.02	0.02	0.02	0.02	0.02	t	135496	RYPNQVY	Mus musculus
1	1.00	1	1	0.01	0.012	-0.006	0.007	0.011	t	27675	INKRKYDSLKEKL	Homo sapiens
1	1.00	1	1	0	0	-0.005	0	0	t	122730	DPAQPPRD	Oryctolagus cuniculus
1	1.00	1	1	0	0	-0.005	0	0	t	122956	YYVFFEEQEDEIIGFG	Oryctolagus cuniculus
1	1.00	1	1	0	0.011	-0.005	0	0	t	113527	HYGSLPQK	Gallus gallus
1	1.00	1	1	0	0	-0.005	0	0	t	107087	ARGLTGRPGDA	Mus musculus
1	1.00	1	1	0	0	-0.005	0	0	t	107087	ARGLTGRPGDA	Mus musculus
1	1.00	1	1	0.02	0.018	0.009	0.02	0.02	t	39251	LRYGNVLDVN	Mus musculus
1	1.00	1	1	0	0	0.001	0	-0.002	t	60596	SQWNKPSKPKTN	Oryctolagus cuniculus
1	1.00	1	1	0	0.008	0.001	0	-0.002	t	56610	RYPGQGSPGGNRYPPQG	Gallus gallus

1	1.00	1	1	0	0.008	0.001	0	-0.002	t	19868	GGGWGQPHGGGWGQPH	Gallus gallus
1	1.00	1	1	0.03	0.025	0.02	0.029	0.03	t	51791	QPADGSSAGGQQQESS	Bos taurus
1	1.00	1	1	0.03	0.026	0.02	0.029	0.03	t	51791	QPADGSSAGGQQQESS	Oryctolagus cuniculus
1	1.00	1	1	0.01	0.004	0.01	0.01	0.01	t	123209	GATPVVGA	Oryctolagus cuniculus
1	1.00	1	1	0	0	0	0.003	0	t	144467	RPWLVHPRHRY	Sus scrofa
1	1.00	1	1	0	0.003	0	0.003	0	t	142292	SHLQLIYNLTLCELNGTDW	Sus scrofa
1	1.00	1	1	0	0.003	0	0.003	0	t	142325	VIIEKRGKVEVEGHLID	Sus scrofa
1	1.00	1	1	0	0	0	0.003	0	t	140567	KEAWLDSTKAT	Sus scrofa
1	1.00	1	1	0	0.003	0	0.003	0	t	142224	LLDTKGGLYRWRSPV	Sus scrofa
1	1.00	1	1	0	0	0	0.003	0	t	73254	WWYEGLPQQHYIGLVA	Equus caballus
1	1.00	1	1	0	0	0	0.003	0	t	74004	YGGIPGGISTPITQQSEK	Equus caballus
1	1.00	1	1	0	0	0	0.003	0	t	64601	TKKPGGPGKNRAINM	Homo sapiens
1	1.00	1	1	0	0	0	0.003	0	t	72517	WGSFPGCRPFQNYFSYET	Equus caballus

1	1.00	1	1	0	0	0	0.003	0	t	62353	SVTEEIAEE DKSVTEEI AV	Homo sapiens
1	1.00	1	1	0	0.003	0	0.003	0	t	50027	PWFLAHH TGGD	Sus scrofa
1	1.00	1	1	0	0	0	0.003	0	t	42592	MSLLDGR GPVRFVLA	Homo sapiens
1	1.00	1	1	0	0	0	0.003	0	t	37123	LLCNNNNS NNYN	Equus caballus
1	1.00	1	1	0	0	0	0.003	0	t	51372	QLINTNGS WHINSTA	Homo sapiens
1	1.00	1	1	0	0	0	0.003	0	t	51372	QLINTNGS WHINSTA	Homo sapiens
1	1.00	1	1	0	0	0	0.003	0	t	42977	MVNINDT DTWIPKG CNE	Equus caballus
1	1.00	1	1	0	0	0	0.003	0	t	50072	PYCYNNDS KNSMAES KEAR	Equus caballus
1	1.00	1	1	0	-0.002	0	0.003	0	t	51837	QPGHLA	Homo sapiens
1	1.00	1	1	0	0	0	0.003	0	t	46466	NVRRLNIN RKDYTGIY QVP	Equus caballus
1	1.00	1	1	0	-0.002	0	0.003	0	t	48452	PLPPVA	Homo sapiens
1	1.00	1	1	0	-0.002	0	0.003	0	t	48827	PPLPHV	Homo sapiens
1	1.00	1	1	0	0	0	0.003	0	t	35068	LCLAGTTG GILWWYE	Equus caballus
1	1.00	1	1	0	-0.006	0	0.003	0	t	15509	FEDVKGTG GESRDLPS GQG	Papio
1	1.00	1	1	0	0	0	0.003	0	t	20650	GKNRAIN MLKRGLP R	Homo sapiens

1	1.00	1	1	0	-0.006	0	0.003	0	t	6925	CRGFTSFKKPPTPPPEPET	Papio
1	1.00	1	1	0	0	0	0.003	0	t	3528	APEDQSPETRRLLDRLSR	Sus scrofa
1	1.00	1	1	0	-0.008	0	0.003	0	t	3528	APEDQSPETRRLLDRLSR	Sus scrofa
1	1.00	1	1	0	0	0	0.003	0	t	784	ADTSRIGNCGPTIFLGVLED	Equus caballus
1	1.00	1	1	0	0	0	0	-0.002	t	135562	IHAMDIPPPTG	Oryctolagus cuniculus
1	1.00	1	1	0.01	0.01	0.01	0.01	0.011	t	6546	CLDDREC	Mus musculus
1	1.00	1	1	-0.02	-0.017	-0.033	-0.02	-0.017	t	26075	IFGFNALVDR	Oryctolagus cuniculus
1	1.00	1	1	0	0.003	0.001	0.001	-0.002	t	99566	QFRQTYRQWIIEAMS	Equus caballus
1	1.00	1	1	0	0	0	0	0.002	t	128358	CDEFRKIK	Homo sapiens
1	1.00	1	1	0.02	0.017	0.022	0.02	0.019	t	95043	SPHQVYN	Oryctolagus cuniculus
1	1.00	1	1	0.02	0.02	0.022	0.02	0.019	t	95043	SPHQVYN	Mus musculus
1	1.00	1	1	0	-0.002	0.001	-0.001	0	t	96593	LPHSNLDHIL	Rattus norvegicus
1	1.00	1	1	0.02	0.02	0.02	0.02	0.02	t	109944	SREEMTK	Homo sapiens
1	1.00	1	1	-0.01	-0.01	-0.012	-0.01	-0.01	t	156755	SGFSY	Mus musculus
1	1.00	1	1	-0.01	-0.01	-0.012	-0.01	-0.01	t	156755	SGFSY	Mus musculus

1	1.00	1	1	-0.02	-0.018	-0.02	-0.017	-0.02	t	48944	PPVLYLPWLPWGPSV	Homo sapiens
1	1.00	1	1	-0.01	-0.01	-0.014	-0.01	-0.01	t	77968	PPPYAPNPGPPPPYTGA	Sus scrofa
1	1.00	1	1	-0.01	-0.01	-0.014	-0.01	-0.01	t	77968	PPPYAPNPGPPPPYTGA	Mus musculus
1	1.00	1	1	-0.01	-0.01	-0.01	-0.01	-0.013	t	130872	GSFLTKGPSKLNDRA	Mus musculus
1	1.00	1	1	0.01	0.008	0.015	0.013	0.01	t	158999	LWGDGILESDLIIPVT	Mus musculus
1	1.00	1	1	-0.01	-0.01	-0.011	-0.004	-0.01	t	52597	QTPVNGNSPWAPTAPLPGDM	Homo sapiens
1	1.00	1	1	-0.02	-0.012	-0.02	-0.02	-0.022	t	156957	SLHSHLS	Gallus gallus
1	1.00	1	1	0.02	0.018	0.025	0.02	0.019	t	132032	QPPPQAQPLLPQ	Oryctolagus cuniculus
1	1.00	1	1	-0.04	-0.036	-0.043	-0.039	-0.04	t	88259	RTLVVMANTG	Homo sapiens
1	1.00	1	1	-0.02	-0.018	-0.025	-0.019	-0.018	t	63882	TGLIHLHQ	Homo sapiens
1	1.00	1	1	0	-0.004	-0.007	-0.001	0	t	20167	GHNSVDFEED	Oryctolagus cuniculus
1	1.00	1	1	0	-0.012	-0.006	0	0	t	19266	GEFPRHVKLKNEIENLLDQV	Mus musculus
1	1.00	1	1	0.01	0.008	0.01	0.013	0.01	t	95828	SGASAQSGTSAQ	Mus musculus

1	1.00	1	1	0.03	0.03	0.038	0.031	0.029	t	18764	GASDLRSS	Homo sapiens
1	1.00	1	1	0	0	-0.01	0	0	t	67934	VDENANANN	Oryctolagus cuniculus
1	1.00	1	1	-0.04	-0.04	-0.028	-0.03	-0.038	t	138545	PRRKAGI	Homo sapiens
1	1.00	1	1	0	0.004	0	0.007	0.001	t	118058	PPPGIRGP	Homo sapiens
1	1.00	1	1	0	0	0	0	-0.001	t	144598	CFVGTHPQLRC	Mus musculus
1	1.00	1	1	-0.01	-0.01	-0.02	-0.016	-0.012	t	32975	KQEVQRGGRYNQ	Homo sapiens
1	1.00	1	1	-0.01	-0.012	-0.014	-0.007	-0.008	t	30421	KEGYLVDKNT	Mus musculus
1	1.00	1	1	0.01	0.013	0.002	0.01	0.01	t	50296	QAKLEAEAKALKEQLAKQAE	Mus musculus
1	1.00	1	1	0.03	0.03	0.023	0.03	0.03	t	58931	SKQPTIDIDLCDICT	Oryctolagus cuniculus
1	1.00	1	1	0.05	0.05	0.05	0.05	0.05	t	92477	DQSLSSLQFA	Ovis aries
1	1.00	1	1	0.03	0.03	0.03	0.03	0.03	t	104180	PNAPDAS	Mus musculus
1	1.00	1	1	0.01	0.01	0.01	0.01	0.01	t	119619	NNYKTTPP	Homo sapiens
1	1.00	1	1	0.01	0.01	0.01	0.01	0.01	t	78973	FGLGGIGVLTPDNYLITPAL	Homo sapiens
1	1.00	1	1	0	0	-0.007	0	0.001	t	141523	ALEPPHNLPVP	Mus musculus

1	1.00	1	1	0	0	0	0	0	t	70387	VPNLRGDLQV	Cavia porcellus
1	1.00	1	1	0	0	0	0	0	t	70387	VPNLRGDLQV	Cavia porcellus
1	1.00	1	1	0	0	0	0	0	t	23397	GYLEQLLRLK	Homo sapiens
1	1.00	1	1	0.01	0.01	0.01	0.01	0.012	t	131891	GLLETNFTA	Homo sapiens
1	1.00	1	1	-0.01	-0.014	-0.016	-0.011	-0.01	t	416	AARQPDL	Oryctolagus cuniculus
1	1.00	1	1	-0.01	-0.012	-0.016	-0.011	-0.01	t	416	AARQPDL	Mus musculus
1	1.00	1	1	0.03	0.027	0.03	0.03	0.029	t	125414	FSEEKLVFSLRLMEEN	Oryctolagus cuniculus
1	1.00	1	1	-0.04	-0.042	-0.044	-0.041	-0.04	t	42676	MSTLNTHGAF	Mus musculus
1	1.00	1	1	-0.04	-0.044	-0.044	-0.041	-0.04	t	42676	MSTLNTHGAF	Oryctolagus cuniculus
1	1.00	1	1	0.02	0.023	0.015	0.02	0.022	t	33161	KRIPLDVAEG	Mus musculus
1	1.00	1	1	0.03	0.03	0.03	0.041	0.032	t	118539	NNGNLQLRNKEVTWEVLEG	Homo sapiens
1	1.00	1	1	0.07	0.072	0.075	0.077	0.07	t	114207	FLGELTSSEVATEV	Homo sapiens
1	1.00	1	1	-0.05	-0.046	-0.058	-0.049	-0.048	t	20638	GKMVQVVY	Homo sapiens
1	1.00	1	1	0	-0.002	0	-0.001	0	t	45695	NRLGGLFNFGPKQKI	Mus musculus

1	1.00	1	1	0.01	0.018	0.01	0.01	0.008	t	156843	AHSEMGTRATMT	Gallus gallus
1	1.00	1	1	0.02	0.02	0.017	0.021	0.02	t	47399	PERLASCRPL	Homo sapiens
1	1.00	1	1	0	-0.002	-0.011	-0.001	0	t	95094	IGFRGLDGVHCTPTTDSGVI	Rattus norvegicus
1	1.00	1	1	0.05	0.05	0.05	0.05	0.049	t	78455	LPTNLTQ	Mus musculus
1	1.00	1	1	-0.06	-0.062	-0.055	-0.06	-0.063	t	125230	RERRGIAL	Mus musculus
1	1.00	1	1	-0.03	-0.032	-0.04	-0.029	-0.028	t	63850	TGHWRCPP	Homo sapiens
1	1.00	1	1	0.01	0.01	0.01	0.01	0.008	t	38952	LQPQQPFSQQ	Mus musculus
1	1.00	1	1	-0.01	-0.013	-0.01	-0.01	-0.012	t	125052	FSLRLMEENW	Oryctolagus cuniculus
1	1.00	1	1	0	0	0	0	0	t	123858	YDPKFEQLK	Ovis aries
1	1.00	1	1	-0.02	-0.02	-0.02	-0.02	-0.02	t	79515	ALSIIGK	Oryctolagus cuniculus
1	1.00	1	1	0.01	0.01	0.002	0.01	0.01	t	14208	ESLTTTSTALGKLQDVV	Mus musculus
1	1.00	1	1	0.03	0.03	0.023	0.03	0.03	t	124588	PQEETLQAFDSHYDYT	Homo sapiens
1	1.00	1	1	0.07	0.073	0.064	0.07	0.069	t	78429	KEQLPTSYPPERAGW	Mus musculus
1	1.00	1	1	0.02	0.02	0.02	0.026	0.02	t	158669	ADIKTALEARKAADT	Homo sapiens
1	1.00	1	1	-0.01	0.002	-0.008	-0.01	-0.012	t	156975	WSSTVLRVSPTRTVP	Gallus gallus

1	1.00	1	1	0	0.002	-0.003	-0.001	-0.002	t	56885	SALTSAIA GFVFSC	Ovis aries
1	1.00	1	1	-0.04	-0.04	-0.042	-0.044	-0.04	t	69856	VLWVVKS TPA	Homo sapiens
1	1.00	1	1	0	0	-0.004	-0.003	0	t	86551	MTAEPKTI VL	Homo sapiens
1	1.00	1	1	0	0	-0.004	-0.003	0	t	86870	NLFGATLV NA	Homo sapiens
1	1.00	1	1	0.01	0.01	0.01	0.01	0.01	t	24881	HSYSPGEI NRVASC	Homo sapiens
1	1.00	1	1	0.02	0.022	0.018	0.017	0.018	t	58037	SGFTLEFLE HAFSVD	Homo sapiens
1	1.00	1	1	0.02	0.02	0.02	0.02	0.019	t	144778	SGTHPFLD KSFS	Mus musculu s
1	1.00	1	1	0.05	0.05	0.052	0.053	0.052	t	99007	SLQPED	Homo sapiens
1	1.00	1	1	0.01	0.01	0	0.01	0.01	t	9661	DPKSLSKG EIASTFESQ NST	Mus musculu s
1	1.00	1	1	0.01	0.01	0.01	0.01	0.009	t	24243	HLKYATYP PYPQ	Mus musculu s
1	1.00	1	1	-0.02	-0.018	-0.02	-0.017	-0.019	t	7046	CSNKSKR	Homo sapiens
1	1.00	1	1	-0.01	-0.01	-0.01	0.001	-0.01	t	104045	LMEPCYA WGCTGPK	Homo sapiens
1	1.00	1	1	0	0	0.002	0	-0.002	t	23510	GYYYPSDP NTFFYAPP YSA	Sus scrofa
1	1.00	1	1	0	0	0	0	-0.001	t	137743	DTPLTIAGL KLV	Mus musculu s
1	1.00	1	1	0.02	0.022	0.02	0.023	0.021	t	51307	QLDPAFG A	Homo sapiens
1	1.00	1	1	0.03	0.028	0.03	0.029	0.03	t	155333	PADS	Mus musculu s

1	1.00	1	1	0	0	-0.012	0	0.002	t	23048	GVLSVDLS LTVELKI	Oryctolagus cuniculus
1	1.00	1	1	-0.01	-0.01	-0.009	-0.01	-0.011	t	64541	TKDTNNNL	Mus musculus
1	1.00	1	1	0.02	0.02	0.025	0.02	0.02	t	108267	AGQRGIV	Mus musculus
1	1.00	1	1	0.02	0.023	0.02	0.02	0.018	t	140842	EGNTDER GTPEYNIA LGQRR	Mus musculus
1	1.00	1	1	0	0.008	0	0	-0.002	t	156939	PPEKSHL	Gallus gallus
1	1.00	1	1	0.03	0.028	0.032	0.029	0.029	t	49749	PTPSDNPF SKLYKETIE TFD	Mus musculus
1	1.00	1	1	0.05	0.05	0.05	0.05	0.05	t	149083	NPTQGSP	Sus scrofa
1	1.00	1	1	0.05	0.05	0.05	0.05	0.05	t	112115	FSLTTYG	Homo sapiens
1	1.00	1	1	0.03	0.03	0.03	0.03	0.03	t	149079	NLTQTQM	Sus scrofa
1	1.00	1	1	-0.02	-0.022	-0.025	-0.017	-0.018	t	28719	ISSIFSR	Mus musculus
1	1.00	1	1	-0.02	-0.022	-0.025	-0.017	-0.018	t	28719	ISSIFSR	Mus musculus
1	1.00	1	1	-0.04	-0.04	-0.043	-0.04	-0.04	t	50741	QFAPSASA FFGMSRIG M	Homo sapiens
1	1.00	1	1	0.04	0.04	0.037	0.04	0.04	t	98772	FCMENSA EPE	Homo sapiens
1	1.00	1	1	0.03	0.028	0.025	0.031	0.031	t	14774	EVLYEAFD EMEECASK AALI	Homo sapiens
1	1.00	1	1	-0.01	-0.01	-0.01	-0.01	-0.01	t	52476	QTASGLVS MF	Homo sapiens
1	1.00	1	1	-0.02	-0.022	-0.02	-0.021	-0.02	t	46868	PANCPKS	Mus musculus

1	1.00	1	1	0.06	0.057	0.06	0.06	0.06	t	153451	QTAPSPQ DTVVS	Oryctolagus cuniculus
1	1.00	1	1	0.02	0.02	0.02	0.02	0.02	t	104132	MSENVEQ HNPINAGT	Oryctolagus cuniculus
1	1.00	1	1	0.03	0.03	0.023	0.03	0.03	t	131114	TQLDELLT EHRMTW DPAQPP	Homo sapiens
1	1.00	1	1	0.02	0.026	0.02	0.02	0.02	t	123339	ISYDPKFE Q	Ovis aries
1	1.00	1	1	-0.04	-0.042	-0.037	-0.037	-0.043	t	11497	EEAILRPLR VQGKRAK	Mus musculus
1	1.00	1	1	0	-0.008	0	0	0	t	60116	SPLLGCIGS TCAE	Mus musculus
1	1.00	1	1	-0.01	-0.013	-0.022	-0.01	-0.01	t	44602	NKSTLQNR RAAVS	Mus
1	1.00	1	1	-0.03	-0.026	-0.038	-0.029	-0.028	t	98062	GGVAGMK AHK	Homo sapiens
1	1.00	1	1	-0.01	-0.01	-0.01	-0.01	-0.01	t	142022	SRIFRGDN	Mus musculus
1	1.00	1	1	-0.03	-0.03	-0.03	-0.03	-0.03	t	53068	RAAKLQ	Homo sapiens
1	1.00	1	1	-0.01	-0.01	-0.01	-0.01	-0.012	t	52312	QSESVEFT KDTQTGM	Mus musculus
1	1.00	1	1	0.01	0.01	0.01	0.01	0.009	t	125384	DSHYDYTI CGDSEDM VCTPK	Homo sapiens
1	1.00	1	1	0.01	0.006	0	0.01	0.012	t	58440	SIAADQLP	Oryctolagus cuniculus
1	1.00	1	1	0.04	0.037	0.046	0.04	0.04	t	12981	ELDGDQM I	Oryctolagus cuniculus
1	1.00	1	1	0.02	0.02	0.02	0.02	0.02	t	132929	WGENNY M	Homo sapiens

1	1.00	1	1	0	0	0	0	0	t	104344	VTGFTMT	Mus musculus
1	1.00	1	1	-0.03	-0.03	-0.036	-0.033	-0.03	t	66410	TSRVEHAVVYYVYSP	Homo sapiens
1	1.00	1	1	0.02	0.024	0.022	0.023	0.02	t	120168	RGWKHSVTYSGGPDTPY	Homo sapiens
1	1.00	1	1	0	0	0	-0.001	-0.002	t	28911	ITGHRMAWDMMMNWS	Homo sapiens
1	1.00	1	1	0.04	0.04	0.04	0.036	0.04	t	157130	CIIEEN	Homo sapiens
1	1.00	1	1	0.02	0.02	0.01	0.02	0.019	t	48093	PKKPNENKLKQPNE	Mus musculus
1	1.00	1	1	0.02	0.017	0.01	0.02	0.019	t	48093	PKKPNENKLKQPNE	Oryctolagus cuniculus
1	1.00	1	1	-0.03	-0.03	-0.025	-0.032	-0.032	t	41548	MFVPHYTLNANSIIY	Homo sapiens
1	1.00	1	1	0	0	-0.005	-0.001	-0.004	t	138199	KQYLERTRAEVDTV	Mus musculus
1	1.00	1	1	0.02	0.02	0.015	0.018	0.02	t	79047	PSCSPTNMIMDGTASVNIPA	Homo sapiens
1	1.00	1	1	0.02	0.017	0.012	0.02	0.022	t	97025	VDDALIDSTKIYSYFPSV	Oryctolagus cuniculus
1	1.00	1	1	-0.02	-0.026	-0.02	-0.02	-0.02	t	123676	QPPYMPPP	Oryctolagus cuniculus
1	1.00	1	1	0	0	0	0	0	t	78364	DQHGWD	Mus musculus
1	1.00	1	1	0.05	0.052	0.047	0.051	0.05	t	53933	RGPEQTQGNFGDQDLIR	Homo sapiens
1	1.00	1	1	0.01	0.01	0.01	0.01	0.01	t	73663	YEGFWTGVTQNAKGIT	Mus musculus

1	1.00	1	1	0	0	0	0	-0.001	t	110863	GLKAKPKFTLGKRKATPTTS	Homo sapiens
1	1.00	1	1	-0.02	-0.022	-0.025	-0.021	-0.019	t	56699	SAAFYYRV	Mus musculus
1	1.00	1	1	0.01	0.01	0.01	0.01	0.011	t	51233	QKPLK	Bos taurus
1	1.00	1	1	0.01	0.018	0.01	0.01	0.011	t	51233	QKPLK	Capra hircus
1	1.00	1	1	0.01	0.014	0.01	0.01	0.011	t	51233	QKPLK	Mus musculus
1	1.00	1	1	0.04	0.044	0.04	0.043	0.039	t	111036	TESTFTGEAYSV	Homo sapiens
1	1.00	1	1	0.01	0.01	0.01	0.01	0.011	t	115414	PHSSNICNIS	Homo sapiens
1	1.00	1	1	-0.02	-0.006	-0.015	-0.017	-0.021	t	115591	APEYRGRTELLK	Homo sapiens
1	1.00	1	1	0.01	0.01	0.008	0.01	0.011	t	53832	RGD	Homo sapiens
1	1.00	1	1	0.03	0.033	0.03	0.03	0.03	t	119753	AQGARVYAYV	Mus musculus
1	1.00	1	1	0.03	0.03	0.03	0.03	0.03	t	119753	AQGARVYAYV	Mus musculus
1	1.00	1	1	0.03	0.03	0.03	0.03	0.03	t	119753	AQGARVYAYV	Oryctolagus cuniculus
1	1.00	1	1	0.03	0.027	0.03	0.03	0.03	t	119753	AQGARVYAYV	Oryctolagus cuniculus

1	1.00	1	1	0.01	0.01	0.01	0.01	0.01	t	146979	GLAPSKAL DSKSY	Homo sapiens
1	1.00	1	1	0.01	0.01	0.01	0.01	0.01	t	119754	AQRLMRY WAN	Mus musculu s
1	1.00	1	1	0.01	0.01	0.01	0.01	0.01	t	119754	AQRLMRY WAN	Oryctola gus cuniculu s
1	1.00	1	1	0.01	0.01	0.01	0.01	0.01	t	118556	PPPGMIPP	Homo sapiens
1	1.00	1	1	0.01	0.013	0.01	0.01	0.01	t	119754	AQRLMRY WAN	Mus musculu s
1	1.00	1	1	0	0.004	0	0.001	0	t	138080	VSLGQNVL	Homo sapiens
1	1.00	1	1	0.04	0.04	0.035	0.034	0.041	t	115107	SENSEKTT MPLW	Homo sapiens
1	1.00	1	1	0	0	-0.002	0	0.001	t	97718	VAFPLPTD	Mus musculu s
1	1.00	1	1	0	-0.002	0	0.009	0.003	t	38304	LNPKTNK WEDK	Mus musculu s
1	1.00	1	1	0	0	0	-0.002	0	t	3288	ANGAGNQ PG	Homo sapiens
1	1.00	1	1	0	0	0	0	0	t	121870	SPLIKHPEV KSA	Rattus norvegic us
1	1.00	1	1	0	0	0	0	0	t	60432	SQERNFV DSI	Mus musculu s
1	1.00	1	1	0	0.003	0	0	0	t	60432	SQERNFV DSI	Mus musculu s
1	1.00	1	1	-0.03	-0.026	-0.039	-0.029	-0.03	t	60956	SSDSNTKT YADLKHR	Homo sapiens
1	1.00	1	1	0.02	0.024	0.02	0.021	0.02	t	67168	TVSSQSFR	Homo sapiens

1	1.00	1	1	0	0	0	0	0	t	137927	LSADFTPG	Oryctolagus cuniculus
1	1.00	1	1	0	0	0	0	0	t	137927	LSADFTPG	Homo sapiens
1	1.00	1	1	0.02	0.019	0.017	0.021	0.02	t	49872	PVGGQSSFYSDWYSPACGKA	Homo sapiens
1	1.00	1	1	0.01	0.01	0.012	0.01	0.009	t	97177	AELPF	Mus musculus
1	1.00	1	1	-0.03	-0.03	-0.032	-0.03	-0.03	t	104780	FRHDSGY	Mus musculus
1	1.00	1	1	-0.03	-0.03	-0.032	-0.03	-0.03	t	104780	FRHDSGY	Mus musculus
1	1.00	1	1	-0.03	-0.03	-0.032	-0.03	-0.03	t	104780	FRHDSGY	Mus musculus
1	1.00	1	1	-0.03	-0.03	-0.032	-0.03	-0.03	t	104780	FRHDSGY	Mus musculus
1	1.00	1	1	0.02	0.02	0.02	0.02	0.02	t	109252	GSLPQKSQRSQDENPV	Rattus norvegicus
1	1.00	1	1	-0.01	-0.01	-0.013	-0.013	-0.013	t	115652	SSRISIYWTIVKP	Homo sapiens
1	1.00	1	1	0.01	0.008	-0.002	0.003	0.009	t	30300	KEALDKYELENHDLKTKN	Mus musculus
1	1.00	1	1	0.01	0.01	0.01	0.01	0.008	t	48595	PNAN	Mus musculus
1	1.00	1	1	0.02	0.02	0.027	0.02	0.019	t	107744	APVDSRTYTRVADHI	Homo sapiens
1	1.00	1	1	-0.02	-0.02	-0.02	-0.02	-0.022	t	140254	KYEHGGNIID	Mus musculus
1	1.00	1	1	-0.02	-0.02	-0.02	-0.02	-0.02	t	47604	PGACYSRPLV	Homo sapiens

1	1.00	1	1	-0.01	-0.01	-0.01	-0.01	-0.012	t	38950	LQPQQPF PQQ	Mus musculu s
1	1.00	1	1	-0.01	-0.01	-0.01	-0.01	-0.012	t	49087	PQQPFPQ QPQ	Mus musculu s
1	1.00	1	1	0.02	0.02	0.02	0.02	0.02	t	106855	SMFTKYPT QLLY	Mus musculu s
1	1.00	1	1	0	0	0	0	0	t	107659	VKFNWYV	Homo sapiens
1	1.00	1	1	0	0	0	0	0	t	111424	IKMVSEPY GDSLFFY	Mus musculu s
1	1.00	1	1	0	0	-0.004	0	0	t	52158	QQTFPQQ PQL	Mus musculu s
1	1.00	1	1	0	0	-0.004	0	0	t	52158	QQTFPQQ PQL	Mus musculu s
1	1.00	1	1	0	0	-0.005	0	0	t	24786	HSLGKWL GHPDKF	Mus musculu s
1	1.00	1	1	-0.02	-0.018	-0.031	-0.023	-0.02	t	37165	LLDKIYTSP LC	Homo sapiens
1	1.00	1	1	-0.01	-0.01	-0.013	-0.013	-0.01	t	60914	SSATK	Homo sapiens
1	1.00	1	1	0	-0.006	0	0	0	t	123570	PATKKAV Q	Oryctola gus cuniculu s
1	1.00	1	1	0.01	0.008	0.005	0.009	0.01	t	78876	VLLGRKW EDEVDDPE SYF	Mus musculu s
1	1.00	1	1	0.03	0.03	0.03	0.026	0.031	t	134031	DEYDEVA MP	Homo sapiens
1	1.00	1	1	0.01	0.008	0.026	0.017	0.01	t	113886	SSAKSHCY AFCSGLP	Homo sapiens
1	1.00	1	1	0.01	0.012	-0.001	0.013	0.01	t	23797	HFLCTFTF NSNNKEYT FPIT	Homo sapiens

1	1.00	1	1	0.01	0.013	0.011	0.01	0.01	t	59757	SMSYTWTGA	Mus musculus
1	1.00	1	1	0	-0.004	-0.007	-0.007	-0.001	t	135470	KAAQFG	Oryctolagus cuniculus
1	1.00	1	1	-0.03	-0.028	-0.03	-0.023	-0.031	t	139630	VDIDIKIRSCRGSCS	Homo sapiens
1	1.00	1	1	0	0.003	-0.014	0	0.001	t	39788	LTAELKIYSVIQAEINKHL	Mus musculus
1	1.00	1	1	0	0	0	0	-0.002	t	107486	KKPTPST	Rattus norvegicus
1	1.00	1	1	-0.03	-0.03	-0.03	-0.027	-0.03	t	96489	KPTPPVKPTA	Homo sapiens(human)
1	1.00	1	1	0.04	0.034	0.033	0.033	0.039	t	127638	ELQMTPDSLIYS	Trichosurus vulpecula
1	1.00	1	1	0.01	0.01	0.01	0.01	0.01	t	139924	TMPWPFP	Ovis aries
1	1.00	1	1	0.04	0.04	0.036	0.04	0.041	t	115047	ELQFPFLQD	Mus musculus
1	1.00	1	1	0.01	0.012	0.012	0.013	0.01	t	158834	IGFASKSADDVKKAF	Homo sapiens
1	1.00	1	1	0	0	-0.007	0	0.001	t	105696	CAPLKPAKSA	Mus musculus
1	1.00	1	1	-0.03	-0.03	-0.03	-0.025	-0.029	t	110385	QLYKTCKQAGTCPPDIIPKV	Homo sapiens
1	1.00	1	1	0.01	0.008	0.015	0.013	0.01	t	44570	NKPNWDGYA	Mus musculus
1	1.00	1	1	0	0	-0.011	0	0	t	95094	IGFRGLDGVHCTPTTDSGVI	Rattus norvegicus
1	1.00	1	1	-0.01	-0.012	-0.03	-0.017	-0.01	t	141676	IKKLENGRSLEGVYVP	Rattus norvegicus

1	1.00	1	1	-0.01	-0.014	-0.03	-0.017	-0.01	t	141676	IKKLENGRSLEGVYVP	Oryctolagus cuniculus
1	1.00	1	1	0.01	0.01	0.008	0.01	0.009	t	125051	FQSKANCLNT	Mus musculus
1	1.00	1	1	0.02	0.02	0.019	0.014	0.019	t	51613	QMGVSELLIS	Homo sapiens
1	1.00	1	1	-0.03	-0.03	-0.029	-0.03	-0.031	t	76325	YVKRVKTW	Homo sapiens
1	1.00	1	1	-0.03	-0.03	-0.03	-0.03	-0.03	t	103940	GLTLPGYKYLGPGNSLD	Oryctolagus cuniculus
1	1.00	1	1	0.03	0.03	0.025	0.03	0.031	t	19405	GEQQEAIKSAENATKVEDIK	Oryctolagus cuniculus
1	1.00	1	1	0.01	0.012	-0.002	0.007	0.011	t	78412	GYKDVDKAPFNGMTGCG	Homo sapiens
1	1.00	1	1	0.01	0.01	0.01	0.01	0.009	t	78535	TPGYKNDALSHFHLT	Mus musculus
1	1.00	1	1	0.01	0.008	0.006	0.009	0.01	t	59757	SMSYTWTGA	Mus musculus
1	1.00	1	1	-0.04	-0.037	-0.049	-0.04	-0.04	t	79424	TITLKKAPKPK	Mus musculus
1	1.00	1	1	0.03	0.034	0.038	0.033	0.028	t	12119	EGAGDGEPPEDDDSATGLAF	Homo sapiens
1	1.00	1	1	0.03	0.032	0.023	0.029	0.03	t	79010	KVDNAETIKKELGLSLTEP	MD
1	1.00	1	1	0.01	0.014	0.01	0.017	0.011	t	107651	VDGVQVH	Homo sapiens
1	1.00	1	1	-0.05	-0.05	-0.062	-0.048	-0.048	t	96071	AGIRPKGAFQ	Homo sapiens(human)

1	1.00	1	1	0.02	0.02	0.02	0.02	0.02	t	135997	SLEKKMSNYIQF	Homo sapiens
1	1.00	1	1	0.02	0.02	0.02	0.02	0.02	t	123127	EGAAMDLRI	Ovis aries
1	1.00	1	1	0	0	0	0	0	t	156425	QQEQQGSGNNVFSGF	Homo sapiens
1	1.00	1	1	0	0	0	0	0	t	132524	PGMMPVGPAP	Homo sapiens
1	1.00	1	1	0	0	0	0	-0.001	t	69175	VKAETRLN	Mus musculus
1	1.00	1	1	-0.04	-0.036	-0.035	-0.037	-0.041	t	115607	GPRHPIRALVGD	Homo sapiens
1	1.00	1	1	0.01	-0.002	0.01	0.013	0.01	t	156843	AHSEMGTRATMT	Bos taurus
1	1.00	1	1	-0.02	-0.019	-0.014	-0.02	-0.021	t	56089	RTIVCGLFPTQIQV	Myodes glareolus
1	1.00	1	1	0.05	0.05	0.05	0.05	0.051	t	134421	KQIPECENVKTTQCVF	Mus musculus
1	1.00	1	1	0	0	0	0	-0.001	t	144598	CFVGTHPQLRC	Mus musculus
1	1.00	1	1	0	0.004	-0.001	-0.001	-0.002	t	9524	DNPASTTNKDK	Cavia porcellus
1	1.00	1	1	0.01	0.01	0.011	0.007	0.008	t	98790	GDKDNVIDQIEKQAK	Homo sapiens
1	1.00	1	1	0.04	0.036	0.029	0.039	0.04	t	10123	DSGASSTASSSH	Oryctolagus cuniculus
1	1.00	1	1	0.02	0.02	0.015	0.018	0.02	t	78927	ALLEETITAKGVRSPEAIRG	Homo sapiens
1	1.00	1	1	0	0	-0.002	0	0	t	23404	GYLPISGEMEKVT	Homo sapiens

1	1.00	1	1	0	0	0	0	-0.001	t	9754	DPRVRGLYF	Mus musculus
1	1.00	1	1	0	0	0	0	-0.001	t	9754	DPRVRGLYF	Mus musculus
1	1.00	1	1	-0.05	-0.05	-0.057	-0.04	-0.049	t	117345	PRRRSRKPEAPRRRS	Homo sapiens
1	1.00	1	1	0	-0.004	0	0.003	0.002	t	63282	TEAAILTQQAAQFDQ	Oryctolagus cuniculus
1	1.00	1	1	0	-0.002	0	0.003	0.002	t	63282	TEAAILTQQAAQFDQ	Mus musculus
1	1.00	1	1	0.07	0.066	0.07	0.07	0.07	t	110376	QAEPD	Mus musculus
1	1.00	1	1	0.07	0.066	0.07	0.07	0.07	t	110376	QAEPD	Mus musculus
1	1.00	1	1	0.07	0.066	0.07	0.07	0.07	t	110376	QAEPD	Mus musculus
1	1.00	1	1	0.01	0.012	0.002	0.011	0.011	t	60827	SRVKNLNSSEGVPDLLV	Homo sapiens
1	1.00	1	1	0	0	-0.011	0.003	0.002	t	25293	IAGLIAIVMVTILLCCM	Homo sapiens
1	1.00	1	1	-0.02	-0.016	-0.024	-0.02	-0.02	t	14953	EWSPCSVTCGNGIQVRIK	Mus musculus
1	1.00	1	1	0.02	0.021	0.015	0.022	0.022	t	74543	YKYPYVLGQGQDTLAPELPI	Homo sapiens
1	1.00	1	1	0.04	0.04	0.04	0.04	0.039	t	112312	SGSGTDF	Homo sapiens
1	1.00	1	1	0.01	0.01	0	0.016	0.017	t	142810	KEWEQIYR	Rattus norvegicus
1	1.00	1	1	0.03	0.003	0.04	0.03	0.028	t	61824	STPLLYPSLA	Mus musculus

1	1.00	1	1	0.03	0.027	0.03	0.03	0.029	t	125414	FSEEKLVFSLRLMEEN	Oryctolagus cuniculus
1	1.00	1	1	0.02	0.027	0.02	0.02	0.021	t	34585	KYQVIKVRAYTYGV	Capra hircus
1	1.00	1	1	0	0.004	0	0.007	0	t	107773	CSVMHEG	Homo sapiens
1	1.00	1	1	0	0.004	0	0.007	0	t	107773	CSVMHEG	Homo sapiens
1	1.00	1	1	0.01	0.013	0.012	0.01	0.01	t	4024	AQPGYPWPLYGNEG	Mus musculus
1	1.00	1	1	0	-0.003	-0.006	0	0	t	20673	GKREMA	Oryctolagus cuniculus
1	1.00	1	1	0.02	0.02	0.032	0.02	0.018	t	19264	GEFMFDFDGDEIF	Mus musculus
1	1.00	1	1	0.04	0.044	0.037	0.041	0.039	t	103547	RGLGEAQLGNSSGNF	Homo sapiens
1	1.00	1	1	0.03	0.03	0.03	0.03	0.03	t	92807	GGDGVDTIYG	Ovis aries
1	1.00	1	1	0.01	0.01	0.01	0.01	0.01	t	147038	ILYGNGPGYALGG	Homo sapiens
1	1.00	1	1	0.01	0.01	0.01	0.01	0.01	t	132828	FSTTSFPGTVTCYDD	Trichosurus vulpecula
1	1.00	1	1	0.01	0.01	0.01	0.01	0.01	t	79188	DSAVGY	Mus musculus
1	1.00	1	1	0.03	0.03	0.03	0.03	0.028	t	128740	GLGPEKTSFFQALGI	Homo sapiens
1	1.00	1	1	0	0	-0.004	0.003	0.002	t	1385	AFRELADA	Homo sapiens

1	1.00	1	1	0.03	0.032	0.023	0.029	0.03	t	130608	SIDLKDKYKNIGA	Homo sapiens
1	1.00	1	1	-0.01	-0.013	-0.017	-0.01	-0.009	t	126926	RGWNPGFLYNGFP	Oryctolagus cuniculus
1	1.00	1	1	0.02	0.018	0.009	0.019	0.02	t	21791	GPSNSEQIPALTAVE	Rattus norvegicus
1	1.00	1	1	-0.01	-0.01	-0.01	-0.01	-0.011	t	106486	GTQEFRH	Mus musculus
1	1.00	1	1	-0.02	-0.02	-0.02	-0.02	-0.02	t	111558	MPLTQKD	Homo sapiens
1	1.00	1	1	-0.01	-0.008	-0.007	-0.007	-0.012	t	55732	RRRTPSPRRRRS	Homo sapiens
1	1.00	1	1	0.02	0.023	0.02	0.02	0.021	t	43834	NFFSPQIITTDNTFVSG	Mus musculus
1	1.00	1	1	0.02	0.024	0.02	0.02	0.022	t	139839	PAAAQAL	Ovis aries
1	1.00	1	1	0.04	0.036	0.046	0.043	0.04	t	30288	KEAELDGD	Oryctolagus cuniculus
1	1.00	1	1	0	0.002	0	0	0	t	49690	PTLDHVLD	Anas platyrhynchos
1	1.00	1	1	-0.03	-0.03	-0.022	-0.03	-0.03	t	73301	WYWGPSLYSI	Mus musculus
1	1.00	1	1	0.07	0.072	0.07	0.071	0.07	t	133594	GPLYQMNDALLL	Homo sapiens
1	1.00	1	1	0	0	0	0.002	0	t	65473	TNVDQDLVGWPAPP	Homo sapiens
1	1.00	1	1	0.04	0.04	0.038	0.04	0.04	t	47137	PDLGFISEPS	Homo sapiens
1	1.00	1	1	-0.04	-0.044	-0.046	-0.04	-0.04	t	94979	YNKRFPGFVSP	Oryctolagus cuniculus

1	1.00	1	1	0.06	0.063	0.048	0.06	0.06	t	120247	TPEDPTDP TDPQDPSS	Mus musculu s
1	1.00	1	1	0.06	0.063	0.048	0.06	0.06	t	120247	TPEDPTDP TDPQDPSS	Mus musculu s
1	1.00	1	1	-0.04	-0.04	-0.058	-0.042	-0.039	t	98783	FQQPLQQ YPLGQ	Homo sapiens
1	1.00	1	1	0.03	0.03	0.026	0.03	0.03	t	5029	ATLCSALY VG	Homo sapiens
1	1.00	1	1	-0.05	-0.047	-0.042	-0.05	-0.052	t	53716	RFI	Mus musculu s
1	1.00	1	1	0	0	0	0	0	t	91588	KNVSINVE LYFDAT	Oryctola gus cuniculu s
1	1.00	1	1	0.04	0.043	0.042	0.04	0.04	t	142103	ADKKPEEL SVTLVEAY TDVF	Felis catus
1	1.00	1	1	-0.02	-0.018	-0.031	-0.019	-0.021	t	6777	CPKDELRP LEKVLESKT RAI	Homo sapiens
1	1.00	1	1	-0.02	-0.016	-0.013	-0.013	-0.019	t	106491	GWGQLFQ	Homo sapiens
1	1.00	1	1	0.04	0.04	0.04	0.04	0.04	t	111742	RRETQ	Mus musculu s
1	1.00	1	1	-0.03	-0.034	-0.041	-0.031	-0.03	t	16058	FGSKSPFF RKYF	Oryctola gus cuniculu s
1	1.00	1	1	-0.03	-0.032	-0.041	-0.031	-0.03	t	16058	FGSKSPFF RKYF	Mus musculu s
1	1.00	1	1	0.03	0.028	0.035	0.029	0.03	t	4543	ASLQNLLS AS	Mus musculu s
1	1.00	1	1	0.02	0.02	0.02	0.02	0.02	t	77844	CLAAPTGL KLAP	Mus musculu s

1	1.00	1	1	0.02	0.02	0.02	0.02	0.02	t	79469	YCPPPLLNSGEPCS	Mus musculus
1	1.00	1	1	0.01	0.008	0.017	0.007	0.011	t	108107	RYAFGSQIA	Mus musculus
1	1.00	1	1	-0.02	-0.02	-0.02	-0.02	-0.02	t	79537	EHPWPLL	Oryctolagus cuniculus
1	1.00	1	1	0	0.004	0	0.001	0.001	t	19639	GFTWTGVTQNG	Homo sapiens
1	1.00	1	1	0.06	0.06	0.061	0.057	0.06	t	68900	VIAEHQFKE	Homo sapiens
1	1.00	1	1	0.01	0.012	0.014	0.017	0.01	t	122198	TEADVNPKPIPSQMPTSP	Homo sapiens
1	1.00	1	1	-0.01	-0.008	-0.013	-0.003	-0.01	t	120548	GAGAPVYLAAVLEYLTAE	Homo sapiens
1	1.00	1	1	0.04	0.036	0.035	0.033	0.041	t	119538	VEDGECYIQF	Oryctolagus cuniculus
1	1.00	1	1	-0.03	-0.028	-0.035	-0.029	-0.03	t	59984	SPAPSRPFSVLRANDVLWLS	Homo sapiens
1	1.00	1	1	0.01	0.01	0.008	0.01	0.01	t	110606	GNPGTDGIPGAKG	Rattus norvegicus
1	1.00	1	1	-0.01	-0.008	-0.013	-0.007	-0.01	t	48343	PLGLKRGIEK	Homo sapiens
1	1.00	1	1	0.01	0.01	0.01	0.01	0.01	t	62830	TAEGRAIN	Mus musculus
1	1.00	1	1	-0.01	-0.01	-0.005	-0.008	-0.01	t	10834	DWVCTILTDFKNWL	Homo sapiens
1	1.00	1	1	0.05	0.048	0.056	0.049	0.051	t	67967	VDGSDP	Mus musculus

1	1.00	1	1	0.03	0.028	0.025	0.027	0.027	t	70444	VPQLEIVPNSAEERLHSMKE	Homo sapiens
1	1.00	1	1	0.03	0.026	0.038	0.033	0.029	t	137393	EDFESCDSITGMNLEA	Oryctolagus cuniculus
1	1.00	1	1	-0.05	-0.054	-0.062	-0.047	-0.047	t	67200	TVVQRQFSEVWKPSPQVTVR	Oryctolagus cuniculus
1	1.00	1	1	0.01	0.012	0.017	0.013	0.01	t	57679	SEVYCPRPDRCLRAP	Homo sapiens
1	1.00	1	1	0	0	0	0	-0.001	t	120028	LFNNYDPGSRPVREP	Rattus norvegicus
1	1.00	1	1	0.02	0.02	0.02	0.02	0.019	t	130925	KLLPEDDESRTYHTV	Homo sapiens
1	1.00	1	1	-0.01	-0.014	-0.019	-0.011	-0.009	t	51474	QLPNVGITQGIV	Oryctolagus cuniculus
1	1.00	1	1	-0.02	-0.024	-0.029	-0.021	-0.02	t	73612	YDYQHKEGRF	Oryctolagus cuniculus
1	1.00	1	1	0.01	0.006	0.02	0.009	0.01	t	78668	GPNLVLPDQK	Oryctolagus cuniculus
1	1.00	1	1	0.03	0.03	0.03	0.03	0.03	t	115847	LLYQEPVLGP	Homo sapiens
1	1.00	1	1	0.03	0.03	0.03	0.03	0.03	t	114967	TYDNECLLCA	Homo sapiens
1	1.00	1	1	0.03	0.03	0.026	0.03	0.03	t	17366	FPQPQQPQQS	Mus musculus
1	1.00	1	1	-0.01	-0.01	-0.01	-0.01	-0.01	t	117018	KIVMFWHEPVYA	Homo sapiens
1	1.00	1	1	-0.01	-0.01	-0.01	-0.01	-0.01	t	117018	KIVMFWHEPVYA	Mus musculus
1	1.00	1	1	-0.01	-0.01	-0.01	-0.01	-0.01	t	79178	DCEIPKIPMDRKCW	Mus musculus

1	1.00	1	1	0.01	0.011	0.02	0.013	0.007	t	26989	ILCYCYGLPDSEPTK	Equus caballus
1	1.00	1	1	0.01	0.006	0.02	0.013	0.007	t	26989	ILCYCYGLPDSEPTK	Oryctolagus cuniculus
1	1.00	1	1	0	0.003	-0.006	0	0.001	t	28537	ISIKYDPRKDSEVFA	Mus musculus
1	1.00	1	1	-0.02	-0.02	-0.025	-0.02	-0.02	t	120516	EWGWS	Mus musculus
1	1.00	1	1	0.02	0.02	0.018	0.02	0.02	t	38330	LNRSGGNYTKTDDEA	Mus musculus
1	1.00	1	1	-0.02	-0.022	-0.02	-0.02	-0.02	t	120173	RLMRYWANFA	Oryctolagus cuniculus
1	1.00	1	1	-0.02	-0.02	-0.02	-0.02	-0.02	t	120173	RLMRYWANFA	Mus musculus
1	1.00	1	1	0.02	0.014	0.006	0.013	0.019	t	132888	QAYVVDSFGNEIFRC	Trichosurus vulpecula
1	1.00	1	1	0.01	0.01	0.003	0.01	0.01	t	158723	DTGCAIDISRQELRCG	Mus musculus
1	1.00	1	1	-0.02	-0.017	-0.02	-0.02	-0.023	t	21930	GQPGPSGAAQGQVISN	Mus musculus
1	1.00	1	1	0.01	0.023	-0.001	0.013	0.012	t	75571	YQWLTRVGKYRPQDKPNT	Chinchilla lanigera
1	1.00	1	1	0.01	0.006	-0.001	0.013	0.012	t	75571	YQWLTRVGKYRPQDKPNT	Oryctolagus cuniculus
1	1.00	1	1	-0.01	-0.012	-0.012	-0.008	-0.01	t	125462	KLPLSLSFLHLTRADLSYPS	Mus musculus

1	1.00	1	1	0.02	0.015	0.02	0.02	0.02	t	108349	EQEETNPE VLIK	Canis lupus
1	1.00	1	1	0	-0.002	0	-0.001	0	t	10124	DSGCVVS WK	Mus musculu s
1	1.00	1	1	0.03	0.034	0.039	0.037	0.03	t	112421	YSSDHKN	Homo sapiens
1	1.00	1	1	-0.01	-0.01	-0.01	-0.01	-0.012	t	81790	ERGGRKQ RKE	Oryctola gus cuniculu s
1	1.00	1	1	0	0	-0.002	-0.003	-0.002	t	19551	GFKAAVA AAANA	Homo sapiens
1	1.00	1	1	-0.04	-0.04	-0.047	-0.04	-0.04	t	97117	YIQMKRIA VG	Mus musculu s
1	1.00	1	1	0.01	0.01	0.01	0.01	0.01	t	6516	CKRGPDS G	Oryctola gus cuniculu s
1	1.00	1	1	-0.03	-0.033	-0.042	-0.03	-0.03	t	78388	FRRVKCKY PDGTKPTF HV	Oryctola gus cuniculu s
1	1.00	1	1	0.06	0.06	0.059	0.063	0.058	t	47267	PEEFEGAG DGEPPED DDSAT	Homo sapiens
1	1.00	1	1	0	0	0	0	-0.001	t	20051	GGTHSQW NKP	Mus musculu s
1	1.00	1	1	-0.02	-0.02	-0.02	-0.016	-0.02	t	135983	RLRPPEPR PRDR	Homo sapiens
1	1.00	1	1	0.04	0.04	0.04	0.04	0.041	t	115212	DTQAIVQ NND	Homo sapiens
1	1.00	1	1	-0.04	-0.042	-0.038	-0.04	-0.041	t	120598	IARFKMFP EVKEKGM A	Oryctola gus cuniculu s
1	1.00	1	1	-0.03	-0.03	-0.035	-0.03	-0.03	t	44231	NIGFGNRG DANI	Homo sapiens

1	1.00	1	1	-0.01	-0.01	-0.015	-0.013	-0.011	t	115726	ENLHLPLPLL	Homo sapiens
1	1.00	1	1	0.04	0.04	0.04	0.04	0.04	t	99558	QEEENQGGKGPLLSI	Homo sapiens
1	1.00	1	1	0.04	0.04	0.04	0.04	0.04	t	62417	SVYQEL	Homo sapiens
1	1.00	1	1	0	0	0.009	0	0	t	102686	NPKTSQYSY	Mus musculus
1	1.00	1	1	0.02	0.02	0.018	0.02	0.02	t	114531	QGQQEYEQHRRQQQR	Homo sapiens
1	1.00	1	1	0.02	0.02	0.02	0.02	0.02	t	101161	QDLPTPPSDP	Mus musculus
1	1.00	1	1	0	0	0	0	0	t	135993	SGGLRLLLCLLL	Homo sapiens
1	1.00	1	1	0	0	0	0	0	t	123125	EFPKLKEFK	Ovis aries
1	1.00	1	1	0	0	-0.004	0	0	t	52082	QQPFP	Mus musculus
1	1.00	1	1	0	0	-0.004	0	0	t	51929	QPQQPQQSFP	Mus musculus
1	1.00	1	1	0	0	-0.004	0	0	t	51929	QPQQPQQSFP	Mus musculus
1	1.00	1	1	0	0	-0.004	0	0	t	52085	QQPFPQPQQQ	Mus musculus
1	1.00	1	1	0	0	-0.004	0	0	t	52089	QQPFPQSQQP	Mus musculus
1	1.00	1	1	0.03	0.033	0.03	0.03	0.03	t	15122	EYYLN	Mus musculus
1	1.00	1	1	0	0	0.002	0	0	t	96023	YQKFPQYLQY	Homo sapiens
1	1.00	1	1	0.01	0.01	0.007	0.01	0.01	t	98928	PEQSLACQCL	Homo sapiens

1	1.00	1	1	0.01	0.01	0.01	0.01	0.008	t	38314	LNPTIAGA GDV	Oryctola gus cuniculu s
1	1.00	1	1	0.01	0.01	0.01	0.01	0.008	t	38314	LNPTIAGA GDV	Oryctola gus cuniculu s
1	1.00	1	1	0	0	0.016	0.01	0.002	t	116732	GLGFYG	Homo sapiens
1	1.00	1	1	0.02	0.021	0.032	0.026	0.023	t	78200	LQKTSG	Equus caballus
1	1.00	1	1	0.02	0.02	0.024	0.02	0.018	t	99760	VKDGYIVD	Oryctola gus cuniculu s
1	1.00	1	1	-0.02	-0.012	-0.02	-0.02	-0.022	t	156868	FHRHPLE	Gallus gallus
1	1.00	1	1	-0.05	-0.05	-0.057	-0.052	-0.051	t	137907	KGSGPGYY RLTL	Homo sapiens
1	1.00	1	1	0.01	0.014	0.001	0.007	0.008	t	113753	QGQQPG QGQHGQ QP	Homo sapiens
1	1.00	1	1	0.01	0.01	0.01	0.01	0.01	t	98108	HTNSPLGD MLRACQT	Homo sapiens
1	1.00	1	1	0.01	0.01	0.01	0.01	0.01	t	24591	HQLDPAF GANSTNP D	Mus musculu s
1	1.00	1	1	0.01	0.01	0.01	0.01	0.01	t	24591	HQLDPAF GANSTNP D	Mus musculu s
1	1.00	1	1	0.01	0.013	0.01	0.01	0.01	t	24591	HQLDPAF GANSTNP D	Mus musculu s
1	1.00	1	1	0	0.003	0	0	-0.001	t	23068	GVNLEDP ASRDL	Mus musculu s
1	1.00	1	1	-0.02	-0.02	-0.02	-0.02	-0.021	t	153431	NPDHPRV PTFMA	Mus musculu s

1	1.00	1	1	-0.02	-0.017	-0.02	-0.02	-0.021	t	106321	DAEFRH	Mus musculu s
1	1.00	1	1	-0.02	-0.017	-0.02	-0.02	-0.021	t	106321	DAEFRH	Mus musculu s
1	1.00	1	1	0	0	0	0	0.002	t	127668	GKFVLSSG KF	Homo sapiens
1	1.00	1	1	0.02	0.017	0.019	0.02	0.018	t	59853	SNKEGVA ADGDALN K	Oryctola gus cuniculu s
1	1.00	1	1	-0.03	-0.03	-0.027	-0.027	-0.03	t	67552	VADLPQ	Homo sapiens
1	1.00	1	1	-0.02	-0.016	-0.026	-0.02	-0.021	t	32887	KPSTLYKM TVEAVK	Ovis aries
1	1.00	1	1	-0.03	-0.03	-0.046	-0.03	-0.03	t	110308	KTCKQAGT CPPDIIPKV EG	Mus musculu s
1	1.00	1	1	-0.03	-0.028	-0.037	-0.027	-0.03	t	116927	AVCRTSM CSIQSAPP	Homo sapiens
1	1.00	1	1	0.03	0.03	0.03	0.03	0.03	t	4079	AQTASGLV SM	Homo sapiens
1	1.00	1	1	0.01	0.013	0.01	0.01	0.01	t	6457	CITQYQRE SQAYYQR G	Mus musculu s
1	1.00	1	1	0	-0.002	-0.002	-0.001	0	t	74895	YLRHGKLR PFERDISN V	Mus musculu s
1	1.00	1	1	0.01	0.01	-0.002	0.01	0.01	t	51286	QLAKDKV NE	Mus musculu s
1	1.00	1	1	0.04	0.037	0.04	0.04	0.042	t	43575	NEDNEKLR	Oryctola gus cuniculu s
1	1.00	1	1	0	0	0	0.001	-0.001	t	41826	MKHM	Mus musculu s
1	1.00	1	1	-0.01	-0.009	-0.021	-0.01	-0.01	t	39199	LRRTQSM GIQLDQR	Myodes glareolu s

1	1.00	1	1	0.07	0.068	0.069	0.072	0.072	t	106247	ALASQGQ	Mus musculus
1	1.00	1	1	-0.02	-0.022	-0.015	-0.017	-0.02	t	115260	GAEGQRPGFGYGG	Mus musculus
1	1.00	1	1	-0.01	-0.007	-0.01	-0.01	-0.01	t	111176	CTSICKYPDYIKMVS	Mus musculus
1	1.00	1	1	-0.02	-0.027	-0.027	-0.013	-0.018	t	156956	SGIGTLDGFRHRHPD	Homo sapiens
1	1.00	1	1	0.02	0.02	0.02	0.02	0.02	t	55825	RSELSQLS	Mus musculus
1	1.00	1	1	0.04	0.044	0.032	0.04	0.042	t	140604	TDQPLSAD	Cavia porcellus
1	1.00	1	1	-0.03	-0.03	-0.023	-0.03	-0.034	t	133705	SSKTRSFGVHLVGPY	Mus musculus
1	1.00	1	1	0	-0.003	0	0	-0.002	t	144662	GREGYPADGGDSVKIWTS	Oryctolagus cuniculus
1	1.00	1	1	0	0	0	0	-0.002	t	144662	GREGYPADGGDSVKIWTS	Oryctolagus cuniculus
1	1.00	1	1	0.05	0.05	0.05	0.05	0.05	t	99174	EDEYEYDEEDRRRGR	Homo sapiens
1	1.00	1	1	0.05	0.05	0.05	0.05	0.05	t	95997	WCGSTDEYCS	Homo sapiens
1	1.00	1	1	0.03	0.019	0.032	0.03	0.03	t	63215	TDPTGIEPDDHLKE	Myodes glareolus
1	1.00	1	1	0.03	0.016	0.032	0.03	0.03	t	63215	TDPTGIEPDDHLKE	Oryctolagus cuniculus
1	1.00	1	1	0.03	0.034	0.032	0.03	0.03	t	63215	TDPTGIEPDDHLKE	Myodes glareolus
1	1.00	1	1	0.01	0.01	0.01	0.01	0.01	t	135778	VEGTSFLT	Homo sapiens

1	1.00	1	1	0.01	0.01	0.009	0.01	0.01	t	73636	YEDRYYRE	Mus musculus
1	1.00	1	1	0.05	0.044	0.048	0.05	0.05	t	67189	TVTYESFHYFGSAPT	Oncorhynchus tshawytscha
1	1.00	1	1	0	0	0	0	0	t	48001	PITNDQKK	Mus musculus
1	1.00	1	1	-0.02	-0.022	-0.015	-0.017	-0.021	t	141605	APPLLPHSNL	Mus musculus
1	1.00	1	1	0.02	0.022	0.012	0.021	0.022	t	41177	MAYRFNGIGVTQNVLYE	Homo sapiens
1	1.00	1	1	0.02	0.02	0.019	0.02	0.02	t	21722	GPPAAEYWNSQ	Homo sapiens
1	1.00	1	1	-0.02	-0.018	-0.023	-0.019	-0.02	t	7193	CTPPALNCYWPLNDYGF	Homo sapiens
1	1.00	1	1	-0.01	-0.01	-0.007	-0.008	-0.012	t	120426	CVIQGVGVTETPLMK	Homo sapiens
1	1.00	1	1	-0.02	-0.02	-0.02	-0.02	-0.02	t	58769	SIYQCC	Homo sapiens
1	1.00	1	1	-0.01	-0.01	-0.01	-0.01	-0.01	t	153867	QLLRREIIKNT	Cavia porcellus
1	1.00	1	1	0	0	-0.008	0	0	t	39070	LREGVTLGVGASFDTQKL	Oryctolagus cuniculus
1	1.00	1	1	0	-0.004	0	-0.007	-0.005	t	123760	SSYAKVSICLPMDTETPLAL	Oryctolagus cuniculus
1	1.00	1	1	-0.01	-0.01	-0.01	-0.01	-0.01	t	140869	IALGQRRADAVK	Oryctolagus cuniculus
1	1.00	1	1	0.01	0.01	0.009	0.013	0.008	t	11983	EFEGAGDGEPPEDDSATGL	Homo sapiens
1	1.00	1	1	0.02	0.018	0.005	0.023	0.022	t	3493	APAVKYTVFETALKKAITAM	Mus musculus

1	1.00	1	1	0.02	0.018	0.005	0.023	0.022	t	3493	APAVKYTVFETALKKAITAM	Mus musculus
1	1.00	1	1	-0.02	-0.02	-0.02	-0.016	-0.02	t	131412	ERGLRYL	Homo sapiens
1	1.00	1	1	0.04	0.04	0.04	0.04	0.04	t	95014	GRLIELSQEG	Rattus norvegicus
1	1.00	1	1	0	0	0	0	-0.001	t	9942	DRADGQPAGDRAAGQPAG	Mus musculus
1	1.00	1	1	0	0	0	0	-0.001	t	9942	DRADGQPAGDRAAGQPAG	Mus musculus
1	1.00	1	1	0	0	0	0	-0.001	t	9942	DRADGQPAGDRAAGQPAG	Mus musculus
1	1.00	1	1	0.05	0.053	0.039	0.05	0.052	t	111515	LQPETTDLYCYEQLNDSSEE	Mus musculus
1	1.00	1	1	0.05	0.053	0.039	0.05	0.052	t	111515	LQPETTDLYCYEQLNDSSEE	Mus musculus
1	1.00	1	1	0.02	0.02	0.02	0.02	0.02	t	137415	HAPRYHY	Mus musculus
1	1.00	1	1	0.02	0.02	0.02	0.02	0.02	t	123165	FEQLKEGYL	Ovis aries
1	1.00	1	1	0.02	0.02	0.02	0.02	0.02	t	112069	CSFSGFS	Homo sapiens
1	1.00	1	1	0	0	0	0	0	t	104126	MNKTFWF	Homo sapiens
1	1.00	1	1	0.01	0.01	0.015	0.01	0.01	t	127589	ASKPEDWDER	Homo sapiens
1	1.00	1	1	0.01	0.01	0.01	0.01	0.01	t	124560	NGVELRD	Mus musculus
1	1.00	1	1	0.01	0.01	0.01	0.01	0.01	t	43238	NANPN	Mus musculus
1	1.00	1	1	-0.01	-0.01	-0.01	-0.014	-0.009	t	134273	YHPDVP	Homo sapiens

1	1.00	1	1	0.01	0.01	0.011	0.01	0.01	t	97643	SGSQGTVAL	Oryctolagus cuniculus
1	1.00	1	1	-0.02	-0.022	-0.029	-0.017	-0.017	t	115351	LKQKSSNSRKKRSTS	Mus musculus
1	1.00	1	1	0	0.002	0.007	0.007	-0.002	t	112982	FTTVVADTGDFHAID	Homo sapiens
1	1.00	1	1	0	-0.004	-0.012	-0.001	0	t	10486	DTSNKFAD	Oryctolagus cuniculus
1	1.00	1	1	0.07	0.076	0.07	0.07	0.07	t	123034	APQCLEDFP	Ovis aries
1	1.00	1	1	0.06	0.06	0.06	0.06	0.06	t	144812	VTGCIPTEEQ	Homo sapiens
1	1.00	1	1	0.05	0.047	0.043	0.05	0.05	t	137469	YPPEEETGERV	Oryctolagus cuniculus
1	1.00	1	1	-0.03	-0.028	-0.035	-0.029	-0.031	t	74798	YLLPRRGPRL	Homo sapiens
1	1.00	1	1	-0.03	-0.028	-0.035	-0.029	-0.031	t	74798	YLLPRRGPRL	Homo sapiens
1	1.00	1	1	-0.01	-0.008	-0.013	-0.007	-0.01	t	48864	PPPPPPPPPPPPPPPPPPPP	Homo sapiens
1	1.00	1	1	-0.02	-0.023	-0.026	-0.02	-0.02	t	105795	FTQFAGKD	Oryctolagus cuniculus
1	1.00	1	1	0.02	0.017	0.02	0.02	0.017	t	144597	CFLTAAGYGGGLPDSVKI	Oryctolagus cuniculus
1	1.00	1	1	0	0	-0.018	0.007	0.01	t	103944	GNYNLRR	Homo sapiens
1	1.00	1	1	0.06	0.061	0.06	0.06	0.062	t	45059	NNDNPQ	Mus musculus
1	1.00	1	1	0.01	0.008	0.008	0.011	0.009	t	49333	PSDLGGNNLPAKFLE	Homo sapiens

1	1.00	1	1	0	0.008	0	0	-0.002	t	156930	PISKEHV	Gallus gallus
1	1.00	1	1	0.03	0.03	0.03	0.03	0.03	t	103040	ALTGLGDK FGESIVN	Homo sapiens
1	1.00	1	1	0.01	0.01	0.01	0.01	0.01	t	149328	GSFQPQQ LPQ	Mus musculu s
1	1.00	1	1	0.01	0.01	0.01	0.01	0.01	t	114957	SVDCSEYP KP	Homo sapiens
1	1.00	1	1	0.01	0.01	0.01	0.01	0.01	t	116025	VYPFPGPI PN	Homo sapiens
1	1.00	1	1	-0.01	-0.01	-0.01	-0.006	-0.01	t	135710	LAKEQKSK QNIGALKQ ELAK	Homo sapiens
1	1.00	1	1	0.03	0.026	0.027	0.03	0.032	t	63555	TETKIDKLC V	Mus musculu s
1	1.00	1	1	0	0.003	0	0	0	t	31722	KKTTTKPT KKPT	Mus musculu s
1	1.00	1	1	0	0.002	0	0.003	0	t	3289	ANGAGNQ PGANGAG GQAA	Homo sapiens
1	1.00	1	1	0	0.001	0	0.001	0	t	38272	LNLTTWN P	Oryctola gus cuniculu s
1	1.00	1	1	-0.01	-0.01	-0.004	-0.01	-0.012	t	6469	CKALQEQC RFPN	Oryctola gus cuniculu s
1	1.00	1	1	0.03	0.028	0.031	0.023	0.03	t	134786	RPVDQY	Mus musculu s
1	1.00	1	1	0	0	-0.01	0	0	t	44251	NIISCNKN DKNQ	Oryctola gus cuniculu s
1	1.00	1	1	0.02	0.022	0.02	0.023	0.022	t	141272	FNPCEDI	Homo sapiens

1	1.00	1	1	0.03	0.03	0.024	0.03	0.032	t	38599	LPNNTASWFTALTQH	Mus musculus
1	1.00	1	1	0.03	0.03	0.024	0.03	0.032	t	38599	LPNNTASWFTALTQH	Mus musculus
1	1.00	1	1	-0.03	-0.03	-0.03	-0.03	-0.028	t	60751	SRNWLPGPCY	Mus musculus
1	1.00	1	1	0.03	0.027	0.027	0.03	0.03	t	8974	DKQLLPIVNKQSCSIS	Oryctolagus cuniculus
1	1.00	1	1	0	-0.002	0	-0.001	0	t	9690	DPNANPNV	Mus musculus
1	1.00	1	1	-0.01	-0.01	-0.01	-0.014	-0.01	t	124319	IREKIEDAK	Homo sapiens
1	1.00	1	1	0.01	0.01	0.005	0.01	0.009	t	34776	LAGGAATAGAFSRPGLPVEY	Homo sapiens
1	1.00	1	1	-0.02	-0.017	-0.022	-0.02	-0.018	t	120516	EWGWS	Mus musculus
1	1.00	1	1	-0.02	-0.02	-0.022	-0.02	-0.018	t	120516	EWGWS	Oryctolagus cuniculus
1	1.00	1	1	0.07	0.066	0.07	0.063	0.07	t	141884	ESLIA	Oryctolagus cuniculus
1	1.00	1	1	-0.01	-0.01	-0.012	-0.01	-0.01	t	32826	KPPKYHPDVP	Homo sapiens
1	1.00	1	1	0	0.002	0	0.001	-0.001	t	51146	QIVGGVYL	Homo sapiens
1	1.00	1	1	-0.01	-0.012	-0.013	-0.01	-0.01	t	104369	YTIENAVPIHLLRTGDEFS	Oryctolagus cuniculus
1	1.00	1	1	0.02	0.02	0.02	0.018	0.02	t	5367	AVIEPIVAT	Homo sapiens

1	1.00	1	1	0	0	-0.007	0	0	t	122430	KGLRRDLD ASREAK	Mus musculu s
1	1.00	1	1	-0.01	-0.012	-0.03	-0.017	-0.01	t	141633	EELLETLQI VAEREAY	Rattus norvegic us
1	1.00	1	1	-0.01	-0.014	-0.03	-0.017	-0.01	t	141633	EELLETLQI VAEREAY	Oryctola gus cuniculu s
1	1.00	1	1	0.03	0.03	0.036	0.03	0.032	t	14992	EYEDLLKA	Mus musculu s
1	1.00	1	1	0.02	0.02	0.02	0.02	0.02	t	111796	SPLFLDQR SLTP	Homo sapiens
1	1.00	1	1	0.02	0.02	0.02	0.02	0.02	t	79264	KCMDAFVI YETNCQ	Mus musculu s
1	1.00	1	1	0.02	0.02	0.02	0.02	0.02	t	20012	GGSGGSV ASGGSVAS	Homo sapiens
1	1.00	1	1	0	0	0	0	0	t	153280	PLGFFPDH QLDPAFR	Mus musculu s
1	1.00	1	1	0.01	0.013	0.011	0.01	0.01	t	75371	YPNQVYYR PVDQYSN	Mus musculu s
1	1.00	1	1	0.01	0.014	0.01	0.011	0.01	t	70038	VMYASSG	Homo sapiens
1	1.00	1	1	0	0	0.002	0	-0.002	t	134418	KGAIIGLM VGGVV	Homo sapiens
1	1.00	1	1	0	0	0.009	0	0	t	26877	IKPLEDKIL V	Mus musculu s
1	1.00	1	1	0	0	0	0	-0.001	t	153865	PETTDLYC Y	Cavia porcellu s
1	1.00	1	1	0	0	0	0	-0.001	t	153827	EQQYNKPL CDL	Cavia porcellu s
1	1.00	1	1	0	0	0	0	-0.001	t	153885	SEEEDEID GPAGQAE PDRAH	Cavia porcellu s

1	1.00	1	1	0	0	0	0	-0.001	t	153282	PLRNTHPQAMQWNSTTF	Mus musculus
1	1.00	1	1	0	0	0	0	-0.001	t	141693	KTWG	Mus musculus
1	1.00	1	1	0	-0.007	0	0	-0.001	t	153841	KCLKFYSKISEYRHY	Oryctolagus cuniculus
1	1.00	1	1	0	0	0	0	-0.001	t	139154	KWEAAREAEQRR	Oryctolagus cuniculus
1	1.00	1	1	0	0	0	0	-0.001	t	153839	HQKRTAMFQDPQERPRKLPQ	Cavia porcellus
1	1.00	1	1	0	-0.007	0	0	-0.001	t	153815	CYEQLNDSSEEEDEI	Oryctolagus cuniculus
1	1.00	1	1	0	0	0	0	-0.001	t	153820	DTPTLHEYMLDLQP	Oryctolagus cuniculus
1	1.00	1	1	0	0	0	0	-0.001	t	158947	LDIDIDPKVKVATLS	Mus musculus
1	1.00	1	1	0	0	0	0	-0.001	t	158910	KNLDIDPKLQEYLGK	Mus musculus
1	1.00	1	1	0	0	0	0	-0.001	t	139305	TAAQITQRKWEAAREAEQRR	Oryctolagus cuniculus
1	1.00	1	1	0	0	0	0	-0.001	t	153284	PTPLSPPLRNTHPQA	Mus musculus
1	1.00	1	1	0	-0.003	0	0	-0.001	t	139203	QIYKAQAQTDRESLRNLR	Oryctolagus cuniculus
1	1.00	1	1	0	0	0	0	-0.001	t	153815	CYEQLNDSSEEEDEI	Oryctolagus cuniculus
1	1.00	1	1	0	0	0	0	-0.001	t	153836	HGPKATLQDIVLHLEP	Cavia porcellus

1	1.00	1	1	0	0	0	0	-0.001	t	153693	RKIFIDTIPN	Mus musculus
1	1.00	1	1	0	-0.003	0	0	-0.001	t	139305	TAAQITQRKWEAAREAEQRR	Oryctolagus cuniculus
1	1.00	1	1	0	0	0	0	-0.001	t	153856	MFQDPQER	Oryctolagus cuniculus
1	1.00	1	1	0	-0.007	0	0	-0.001	t	153838	HQKRTAMFQDPQER	Oryctolagus cuniculus
1	1.00	1	1	0	0	0	0	-0.001	t	158738	EIVDLDPKLKQINLI	Mus musculus
1	1.00	1	1	0	0	0	0	-0.001	t	139398	YGCDVGPDGRLLRGHDQYAY	Oryctolagus cuniculus
1	1.00	1	1	0	0	0	0	-0.001	t	153837	HGRLVTLKDIVLDLQP	Cavia porcellus
1	1.00	1	1	0	-0.003	0	0	-0.001	t	139398	YGCDVGPDGRLLRGHDQYAY	Oryctolagus cuniculus
1	1.00	1	1	0	0	0	0	-0.001	t	117569	LKYYLAPKIEDEEGS	Oryctolagus cuniculus
1	1.00	1	1	0	0	0	0	-0.001	t	125250	SDEFNPCEDIMGYK	Mus musculus
1	1.00	1	1	0	0	0	0	-0.001	t	122862	NDEYFIRK	Rattus norvegicus
1	1.00	1	1	0	0	0	0	-0.001	t	127643	ERPLELRVRVLDI	Homo sapiens
1	1.00	1	1	0	0	0	0	-0.001	t	122857	MGCSSPPCECHQEE	Mus musculus
1	1.00	1	1	0	0	0	0	-0.001	t	120412	ASKEKQENKIFADDIDISD	Mus musculus

1	1.00	1	1	0	0	0	0	-0.001	t	122150	MSKTLHAQLGFKKT	Oryctolagus cuniculus
1	1.00	1	1	0	0	0	0	-0.001	t	122210	VRVIDPSLGDRKDE	Oryctolagus cuniculus
1	1.00	1	1	0	0	0	0	-0.001	t	122115	IKGANDLAKFHQMLMKIIMK	Oryctolagus cuniculus
1	1.00	1	1	0	0	0	0	-0.001	t	122947	WTQEPQPLEEKWQHRVVE	Homo sapiens
1	1.00	1	1	0	0	0	0	-0.001	t	119943	IGLMVGGVV	Mus musculus
1	1.00	1	1	0	0	0	0	-0.001	t	122886	RGTDEYFIRKPP	Rattus norvegicus
1	1.00	1	1	0	0	0	0	-0.001	t	118058	PPPGIRGP	Oryctolagus cuniculus
1	1.00	1	1	0	0	0	0	-0.001	t	133087	ETKTYFWKVQ	Mus musculus
1	1.00	1	1	0	0	0	0	-0.001	t	131406	EGEGSGFIREDPIGAGA	Oryctolagus cuniculus
1	1.00	1	1	0	0	0	0	-0.001	t	120769	LLVDVEPKVKSKKRE	Mus musculus
1	1.00	1	1	0	0	0	0	-0.001	t	122153	NASGLSRPSPSAH	Oryctolagus cuniculus
1	1.00	1	1	0	0	0	0	-0.001	t	119048	VSDYEMKLMDLDVEQ	Mus musculus
1	1.00	1	1	0	0	0	0	-0.001	t	124588	PQEETLQAFDSHYDYT	Oryctolagus cuniculus
1	1.00	1	1	0	0	0	0	-0.001	t	131701	THHINNMKPSFTRENT	Oryctolagus cuniculus

1	1.00	1	1	0	0	0	0	-0.001	t	122842	LLDLPRDLGGMGCSSPPCE	Oryctolagus cuniculus
1	1.00	1	1	0	0	0	0	-0.001	t	122090	ESPQEISKHQALG	Oryctolagus cuniculus
1	1.00	1	1	0	0	0	0	-0.001	t	127898	YEENLGDSIV	Oryctolagus cuniculus
1	1.00	1	1	0	0	0	0	-0.001	t	131717	TTLVNPNKKNSYPHY	Oryctolagus cuniculus
1	1.00	1	1	0	0	0	0	-0.001	t	131579	PDIITGFSS	Oryctolagus cuniculus
1	1.00	1	1	0	0	0	0	-0.001	t	118902	LVFEAPNQEKVSDYE	Oryctolagus cuniculus
1	1.00	1	1	0	0	0	0	-0.001	t	122073	AEEQARHVKNGLE	Oryctolagus cuniculus
1	1.00	1	1	0	0	0	0	-0.001	t	118783	EIIVTHFPFDEQNCSMK	Rattus norvegicus
1	1.00	1	1	0	0	0	0	-0.001	t	122857	MGCSSPPCECHQEE	Mus musculus
1	1.00	1	1	0	0	0	0	-0.001	t	121288	EYILKKPRS	Rattus norvegicus
1	1.00	1	1	0	0	0	0	-0.001	t	120716	KSAIEGVK	Rattus norvegicus
1	1.00	1	1	0	0	0	0	-0.001	t	118060	PPPGMRPP	Oryctolagus cuniculus
1	1.00	1	1	0	0	0	0	-0.001	t	118877	LDVEQLGIPEQEYSC	Oryctolagus cuniculus

1	1.00	1	1	0	0	0	0	-0.001	t	119496	MFPEVKEK GMAAL	Homo sapiens
1	1.00	1	1	0	0	0	0	-0.001	t	118131	SEESDDD MGFGLFD	Homo sapiens
1	1.00	1	1	0	0	0	0	-0.001	t	118060	PPPGMRP P	Oryctola gus cuniculu s
1	1.00	1	1	0	0	0	0	-0.001	t	120970	STHTMPQ WVRKIFID TIPN	Mus musculu s
1	1.00	1	1	0	0	0	0	-0.001	t	117609	PVQLETLSI RGNNIR	Mus musculu s
1	1.00	1	1	0	0	0	0	-0.001	t	119048	VSDYEMKL MDLDVEQ	Mus musculu s
1	1.00	1	1	0	0	0	0	-0.001	t	122108	GVGVELE NSMGGLN F	Oryctola gus cuniculu s
1	1.00	1	1	0	0	0	0	-0.001	t	125250	SDEFNPCE DIMGYK	Mus musculu s
1	1.00	1	1	0	0	0	0	-0.001	t	132045	RMATPLL MRPMSM	Oryctola gus cuniculu s
1	1.00	1	1	0	0	0	0	-0.001	t	119052	WNPADYG GIK	Rattus norvegic us
1	1.00	1	1	0	0	0	0	-0.001	t	133178	KNFVKPNE TKTYFWK	Mus musculu s
1	1.00	1	1	0	0	0	0	-0.001	t	126201	GYKEKSKF QDTHNNA	Oryctola gus cuniculu s
1	1.00	1	1	0	0	0	0	-0.001	t	118480	KWNPDDY GGVK	Rattus norvegic us
1	1.00	1	1	0	0	0	0	-0.001	t	122746	EEQEDEIIG FGQELKN	Oryctola gus cuniculu s
1	1.00	1	1	0	0	0	0	-0.001	t	118846	ILKKVLEAL KDLI	Oryctola gus cuniculu s

1	1.00	1	1	0	0	0	0	-0.001	t	109905	SEEEND	Mus musculus
1	1.00	1	1	0	0	0	0	-0.001	t	109905	SEEEND	Mus musculus
1	1.00	1	1	0	0	0	0	-0.001	t	111497	LMGTLGIVCPICSQKP	Oryctolagus cuniculus
1	1.00	1	1	0	0	0	0	-0.001	t	107214	MVGGVV	Mus musculus
1	1.00	1	1	0	0	0	0	-0.001	t	104569	MASPGSGFWSFGSEDGS	Mus musculus
1	1.00	1	1	0	0	0	0	-0.001	t	105763	EVHHQK	Mus musculus
1	1.00	1	1	0	0	0	0	-0.001	t	101437	YRSPFSRVVHLYRNGK	Mus musculus
1	1.00	1	1	0	0	0	0	-0.001	t	110761	AENPTNY	Mus musculus
1	1.00	1	1	0	0	0	0	-0.001	t	99833	PPFSQQ	Mus musculus
1	1.00	1	1	0	0	0	0	-0.001	t	110962	PIDVVDP	Mus musculus
1	1.00	1	1	0	0	0	0	-0.001	t	106322	DAEFRHDSG	Mus musculus
1	1.00	1	1	0	0	0	0	-0.001	t	104906	MVGGVVIA	Mus musculus
1	1.00	1	1	0	0	0	0	-0.001	t	99834	QGQQGYYP	Mus musculus
1	1.00	1	1	0	0	0	0	-0.001	t	99836	QPFPQPQ	Mus musculus
1	1.00	1	1	0	-0.003	0	0	-0.001	t	108131	SKDWSFY	Oryctolagus cuniculus

1	1.00	1	1	0	-0.003	0	0	-0.001	t	108019	LSQPKIVKWDR	Oryctolagus cuniculus
1	1.00	1	1	0	0	0	0	-0.001	t	108019	LSQPKIVKWDR	Oryctolagus cuniculus
1	1.00	1	1	0	0	0	0	-0.001	t	108019	LSQPKIVKWDR	Oryctolagus cuniculus
1	1.00	1	1	0	0	0	0	-0.001	t	99842	RPQQPYP	Mus musculus
1	1.00	1	1	0	-0.003	0	0	-0.001	t	108131	SKDWSFY	Oryctolagus cuniculus
1	1.00	1	1	0	0	0	0	-0.001	t	102384	EFRH	Mus musculus
1	1.00	1	1	0	0	0	0	-0.001	t	102384	EFRH	Mus musculus
1	1.00	1	1	0	0	0	0	-0.001	t	108131	SKDWSFY	Oryctolagus cuniculus
1	1.00	1	1	0	0	0	0	-0.001	t	106842	SKDEIDSAVKMLVSLKMSY	Mus musculus
1	1.00	1	1	0	0	0	0	-0.001	t	110376	QAEPD	Mus musculus
1	1.00	1	1	0	0	0	0	-0.001	t	110376	QAEPD	Mus musculus
1	1.00	1	1	0	0	0	0	-0.001	t	63604	TEYYLNHGKWPEN	Oryctolagus cuniculus
1	1.00	1	1	0	0	0	0	-0.001	t	72251	WAPSV	Mus musculus
1	1.00	1	1	0	0	0	0	-0.001	t	95048	VIFQTPLIKNPDVKSAIEG	Rattus norvegicus

1	1.00	1	1	0	0	0	0	-0.001	t	76491	YYDGRRS	Mus musculus
1	1.00	1	1	0	0	0	0	-0.001	t	75075	YMLDLQPETT	Cavia porcellus
1	1.00	1	1	0	0	0	0	-0.001	t	68018	VDLPQLGLRR	Mus musculus
1	1.00	1	1	0	0	0	0	-0.001	t	73430	YAQYMSRAYAEFLGEDPGSG	Mus musculus
1	1.00	1	1	0	0	0	0	-0.001	t	69178	VKAETRLNPDLQPTE	Oryctolagus cuniculus
1	1.00	1	1	0	0	0	0	-0.001	t	63604	TEYYLNHGKWPEN	Oryctolagus cuniculus
1	1.00	1	1	0	0	0	0	-0.001	t	95161	TYGKFCIKP	Mus musculus
1	1.00	1	1	0	0	0	0	-0.001	t	69175	VKAETRLN	Mus musculus
1	1.00	1	1	0	0	0	0	-0.001	t	89661	TQLTSSYEDYML	Mus musculus
1	1.00	1	1	0	0	0	0	-0.001	t	67040	TVKAKLLSVE	Mus musculus
1	1.00	1	1	0	0	0	0	-0.001	t	75203	YNPLYGSRLGVPDTLGGDPK	Homo sapiens
1	1.00	1	1	0	0	0	0	-0.001	t	94606	LTGGFYEPL	Mus musculus
1	1.00	1	1	0	0	0	0	-0.001	t	60663	SRGAPGGGFVPSLQGVP	Oryctolagus cuniculus
1	1.00	1	1	0	0	0	0	-0.001	t	60751	SRNWLPGPCY	Mus musculus

1	1.00	1	1	0	0	0	0	-0.001	t	84423	KKGLQQLQEWEDWVGWIGN	Felis catus
1	1.00	1	1	0	0	0	0	-0.001	t	63217	TDQMEVDSGPRKRPLRGG	Oryctolagus cuniculus
1	1.00	1	1	0	0	0	0	-0.001	t	66060	TRLNPD	Mus musculus
1	1.00	1	1	0	0	0	0	-0.001	t	68548	VFPTKDVAL	Mus musculus
1	1.00	1	1	0	0	0	0	-0.001	t	68019	VDLPQPGLRR	Mus musculus
1	1.00	1	1	0	0	0	0	-0.001	t	95132	PYVTYG	Mus musculus
1	1.00	1	1	0	0	0	0	-0.001	t	73636	YEDRYYRE	Mus musculus
1	1.00	1	1	0	0	0	0	-0.001	t	95058	AGLQND	Mus musculus
1	1.00	1	1	0	0	0	0	-0.001	t	73889	YFRTLEDAAH	Mus musculus
1	1.00	1	1	0	0	0	0	-0.001	t	73433	YARFMDTDPDRYGILPSNF	Mus musculus
1	1.00	1	1	0	0	0	0	-0.001	t	73433	YARFMDTDPDRYGILPSNF	Mus musculus
1	1.00	1	1	0	0	0	0	-0.001	t	65974	TQYERE	Mus musculus
1	1.00	1	1	0	0	0	0	-0.001	t	70391	VPNLRGDLQVLAQKVARTLP	Oryctolagus cuniculus
1	1.00	1	1	0	0	0	0	-0.001	t	97348	GLFGAIAGFIEGGW	Oryctolagus cuniculus

1	1.00	1	1	0	0	0	0	-0.001	t	95058	AGLQND	Mus musculus
1	1.00	1	1	0	0	0	0	-0.001	t	72065	VYKLDISEA	Mus musculus
1	1.00	1	1	0	0	0	0	-0.001	t	70391	VPNLRGDLQVLAQKVARTLP	Oryctolagus cuniculus
1	1.00	1	1	0	0	0	0	-0.001	t	73889	YFRTLEDAAH	Mus musculus
1	1.00	1	1	0	0	0	0	-0.001	t	65684	TPPHSARSKFGYGA	Mus musculus
1	1.00	1	1	0	0	0	0	-0.001	t	64086	THTPLPRGIGY	Mus musculus
1	1.00	1	1	0	0	0	0	-0.001	t	74068	YGPGVDY	Mus musculus
1	1.00	1	1	0	0	0	0	-0.001	t	56962	SARDGIRDAMIGLREEMIEK	Mus musculus
1	1.00	1	1	0	0	0	0	-0.001	t	41537	MFSGGGGPLSPGGKS	Mus musculus
1	1.00	1	1	0	0	0	0	-0.001	t	59229	SLKMADPNRFRGKDLP	Mus musculus
1	1.00	1	1	0	0	0	0	-0.001	t	48332	PLGFFPDHQLDPAFG	Mus musculus
1	1.00	1	1	0	-0.007	0	0	-0.001	t	55951	RSRSES	Oryctolagus cuniculus
1	1.00	1	1	0	-0.003	0	0	-0.001	t	55951	RSRSES	Oryctolagus cuniculus
1	1.00	1	1	0	0	0	0	-0.001	t	55951	RSRSES	Oryctolagus cuniculus

1	1.00	1	1	0	-0.003	0	0	-0.001	t	51393	QLKLNWFKKGSS	Oryctolagus cuniculus
1	1.00	1	1	0	0	0	0	-0.001	t	43215	NALTQPVDQLLK	Mus musculus
1	1.00	1	1	0	0	0	0	-0.001	t	59229	SLKMADPNRFRGKDLP	Mus musculus
1	1.00	1	1	0	0	0	0	-0.001	t	50977	QGVPESPFSRTGEGLD	Oryctolagus cuniculus
1	1.00	1	1	0	0	0	0	-0.001	t	56734	SADNNNSEYSWT	Mus musculus
1	1.00	1	1	0	0	0	0	-0.001	t	50636	QELDCWHYHQYCITS	Capra hircus
1	1.00	1	1	0	0	0	0	-0.001	t	50636	QELDCWHYHQYCITS	Capra hircus
1	1.00	1	1	0	0	0	0	-0.001	t	41826	MKHM	Mus musculus
1	1.00	1	1	0	0	0	0	-0.001	t	58295	SGVMTRGRL	Mus musculus
1	1.00	1	1	0	0	0	0	-0.001	t	39164	LRPGPVSQPYHHWDTDK	Homo sapiens
1	1.00	1	1	0	0	0	0	-0.001	t	53634	RESQAYYQR	Mus musculus
1	1.00	1	1	0	0	0	0	-0.001	t	46859	PALTAVETGATNPL	Oryctolagus cuniculus
1	1.00	1	1	0	0	0	0	-0.001	t	48279	PLATQPPVLAL	Mus musculus
1	1.00	1	1	0	0	0	0	-0.001	t	56622	RYPNQVYYRPV	Mus musculus
1	1.00	1	1	0	0	0	0	-0.001	t	46786	PAEVTQLKGLS	Mus musculus

1	1.00	1	1	0	-0.003	0	0	-0.001	t	55389	RQAESSEAMAAWSERAGEA	Oryctolagus cuniculus
1	1.00	1	1	0	0	0	0	-0.001	t	48912	PPSDIKGKYVKEVEVK	Oryctolagus cuniculus
1	1.00	1	1	0	0	0	0	-0.001	t	51393	QLKLNWFKKGSS	Mus musculus
1	1.00	1	1	0	0	0	0	-0.001	t	48557	PMIHFGNDWEDRYYREN	Mus musculus
1	1.00	1	1	0	0	0	0	-0.001	t	56788	SAFVFPTKD	Mus musculus
1	1.00	1	1	0	0	0	0	-0.001	t	51393	QLKLNWFKKGSS	Oryctolagus cuniculus
1	1.00	1	1	0	0	0	0	-0.001	t	48876	PPPQAPYQGY	Mus musculus
1	1.00	1	1	0	0	0	0	-0.001	t	37660	LLPHSNLDHILEPSIPWKSK	Mus musculus
1	1.00	1	1	0	0	0	0	-0.001	t	54058	RHKQKIVAPVKQTL	Oryctolagus cuniculus
1	1.00	1	1	0	0	0	0	-0.001	t	58077	SGHRMAWDMMMNW	Mus musculus
1	1.00	1	1	0	0	0	0	-0.001	t	45597	NQNNFVHDCVNITIKQHTVT	Mus musculus
1	1.00	1	1	0	0	0	0	-0.001	t	38271	LNLTTASVPMLRWYAERFCF	Mus musculus
1	1.00	1	1	0	0	0	0	-0.001	t	50490	QDLPGNDNNSTATLC	Oryctolagus cuniculus
1	1.00	1	1	0	0	0	0	-0.001	t	38422	LPAYQDYTARAQVSE	Oryctolagus cuniculus

1	1.00	1	1	0	0	0	0	-0.001	t	48906	PPRRVS	Mus musculus
1	1.00	1	1	0	0	0	0	-0.001	t	53122	RAISSWKQRNRWEWRPD	Felis catus
1	1.00	1	1	0	0	0	0	-0.001	t	39664	LSSKAVNHIRSVWKDLLEDT	Mus musculus
1	1.00	1	1	0	0	0	0	-0.001	t	37660	LLPHSNLDHILEPSIPWKSK	Oryctolagus cuniculus
1	1.00	1	1	0	0	0	0	-0.001	t	38918	LQND	Mus musculus
1	1.00	1	1	0	0	0	0	-0.001	t	48912	PPSDIKGKYVKEVEVK	Oryctolagus cuniculus
1	1.00	1	1	0	0	0	0	-0.001	t	56599	RYNLVFSGDSDRICSNRA	Mus musculus
1	1.00	1	1	0	0	0	0	-0.001	t	49749	PTPSDNPFSKLYKETIETFD	Mus musculus
1	1.00	1	1	0	0	0	0	-0.001	t	40332	LVFFAEDV	Mus musculus
1	1.00	1	1	0	0	0	0	-0.001	t	57320	SDYEDRYYREN	Mus musculus
1	1.00	1	1	0	0	0	0	-0.001	t	46894	PAPQAPYQGY	Mus musculus
1	1.00	1	1	0	0	0	0	-0.001	t	13525	ENMYRYPNQVYY	Mus musculus
1	1.00	1	1	0	0	0	0	-0.001	t	19067	GDPEDL	Mus musculus
1	1.00	1	1	0	0	0	0	-0.001	t	19862	GGGTHNQWNKPSKPKTNLKH	Mus musculus
1	1.00	1	1	0	0	0	0	-0.001	t	18706	GANSNNPDWDFNP	Mus musculus

1	1.00	1	1	0	0	0	0	-0.001	t	23973	HGYEKPEELWTAKSRVHPL	Homo sapiens
1	1.00	1	1	0	0	0	0	-0.001	t	11819	EENVEHDA	Oryctolagus cuniculus
1	1.00	1	1	0	0	0	0	-0.001	t	10869	DYEDRYYREN	Mus musculus
1	1.00	1	1	0	0	0	0	-0.001	t	15807	FFFDIDLLL	Mus musculus
1	1.00	1	1	0	0	0	0	-0.001	t	36413	LHQPPPQIFLKILPQSGPIG	Homo sapiens
1	1.00	1	1	0	0	0	0	-0.001	t	24402	HNQWNKPSK	Mus musculus
1	1.00	1	1	0	0	0	0	-0.001	t	20052	GGTHSQWNKPSKP	Mus musculus
1	1.00	1	1	0	0	0	0	-0.001	t	30661	KEVPALTAVETGAT	Oryctolagus cuniculus
1	1.00	1	1	0	0	0	0	-0.001	t	10529	DTYRYI	Mus musculus
1	1.00	1	1	0	0	0	0	-0.001	t	36729	LITNTIAGV	Mus musculus
1	1.00	1	1	0	0	0	0	-0.001	t	24334	HMAGAAAA	Mus musculus
1	1.00	1	1	0	0	0	0	-0.001	t	14435	ETIYNTTLKY	Homo sapiens
1	1.00	1	1	0	0	0	0	-0.001	t	22135	GRLKAESTV	Mus musculus
1	1.00	1	1	0	-0.003	0	0	-0.001	t	28676	ISQAVHAAHAEINEAGR	Oryctolagus cuniculus
1	1.00	1	1	0	0	0	0	-0.001	t	14435	ETIYNTTLKY	Homo sapiens
1	1.00	1	1	0	0	0	0	-0.001	t	10868	DYEDRYYRE	Mus musculus

1	1.00	1	1	0	0	0	0	-0.001	t	19269	GEGAPPAKRARTDQME	Oryctolagus cuniculus
1	1.00	1	1	0	-0.003	0	0	-0.001	t	11813	EENVEENV	Oryctolagus cuniculus
1	1.00	1	1	0	0	0	0	-0.001	t	24841	HSQWNKPSKP	Mus musculus
1	1.00	1	1	0	0	0	0	-0.001	t	11377	EDLDGA	Mus musculus
1	1.00	1	1	0	0	0	0	-0.001	t	13837	EQHHRRTDN	Mus musculus
1	1.00	1	1	0	0	0	0	-0.001	t	17470	FQEPFWLEDG	Mus musculus
1	1.00	1	1	0	0	0	0	-0.001	t	11148	EANNREGVEG	Mus musculus
1	1.00	1	1	0	0	0	0	-0.001	t	17470	FQEPFWLEDG	Mus musculus
1	1.00	1	1	0	0	0	0	-0.001	t	11148	EANNREGVEG	Mus musculus
1	1.00	1	1	0	0	0	0	-0.001	t	9620	DPEDLD	Mus musculus
1	1.00	1	1	0	0	0	0	-0.001	t	6559	CLFKDWEELGEEIRLKV	Mus musculus
1	1.00	1	1	0	0	0	0	-0.001	t	5771	AYFLYQQQGRLDKL	Oryctolagus cuniculus
1	1.00	1	1	0	0	0	0	-0.001	t	9794	DPVAALFFF	Mus musculus
1	1.00	1	1	0	0	0	0	-0.001	t	9524	DNPASTTNKDK	Mus musculus
1	1.00	1	1	0	0	0	0	-0.001	t	9524	DNPASTTNKDK	Mus musculus
1	1.00	1	1	0	0	0	0	-0.001	t	7244	CTWQQWERELQGYD	Capra hircus

1	1.00	1	1	0	0	0	0	-0.001	t	7244	CTWQQWERELQGYD	Capra hircus
1	1.00	1	1	0	0	0	0	-0.001	t	7491	DAEFRHDSGYEVHHQK	Mus musculus
1	1.00	1	1	0	0	0	0	-0.001	t	1450	AGAPSAEQRER	Mus musculus
1	1.00	1	1	0	0	0	0	-0.001	t	7491	DAEFRHDSGYEVHHQK	Mus musculus
1	1.00	1	1	-0.03	-0.03	-0.037	-0.039	-0.033	t	130800	EGAKAFFKGA	Homo sapiens
1	1.00	1	1	0.01	0.01	0.01	0.01	0.01	t	111230	ECISMDYKQTQLCLI	Mus musculus
1	1.00	1	1	-0.01	-0.01	-0.01	-0.01	-0.01	t	131100	TDSEMDVVRFD	Mus musculus
1	1.00	1	1	0.01	0.013	0.011	0.01	0.01	t	149785	KTVEGVKDLQAQ	Mus musculus
1	1.00	1	1	-0.01	-0.007	-0.015	-0.01	-0.01	t	134003	AKTQGPYDVVVLPGG	Mus musculus
1	1.00	1	1	0.03	0.034	0.035	0.031	0.03	t	61824	STPLLYPSLA	Homo sapiens
1	1.00	1	1	0.03	0.027	0.019	0.03	0.03	t	52749	QVMSRSANEGYD	Oryctolagus cuniculus
1	1.00	1	1	-0.02	-0.018	-0.023	-0.019	-0.018	t	138884	NKLLIAFGLVILLVTLPCAS	Homo sapiens
1	1.00	1	1	0	0	-0.012	0	0	t	121795	RISPGKNATGMEVGWYRS	Rattus norvegicus
1	1.00	1	1	-0.03	-0.032	-0.04	-0.031	-0.03	t	13763	EPSGQTADWY	Mus musculus
1	1.00	1	1	0.06	0.064	0.059	0.057	0.058	t	98527	TKTEEPAA	Homo sapiens

1	1.00	1	1	0.06	0.062	0.059	0.057	0.058	t	98527	TKTEEPAA	Homo sapiens
1	1.00	1	1	-0.01	-0.008	-0.005	-0.003	-0.01	t	122943	WDPAQPPRD	Homo sapiens
1	1.00	1	1	0.02	0.018	0.01	0.019	0.02	t	68996	VIITFKSGAT	Mus musculus
1	1.00	1	1	-0.02	-0.018	-0.02	-0.019	-0.02	t	53939	RGPRLGVR	Homo sapiens
1	1.00	1	1	0.04	0.04	0.038	0.038	0.04	t	33987	KVFAGSLFES	Homo sapiens
1	1.00	1	1	-0.01	-0.01	-0.015	-0.01	-0.01	t	66385	TSQGMYGGTYLVEKP	Homo sapiens
1	1.00	1	1	0	0	0	0	-0.001	t	100508	SGYEVHHQKL	Mus musculus
1	1.00	1	1	0	0	0	0	-0.001	t	73070	WSFADGN	Mus musculus
1	1.00	1	1	0	0	0	0	-0.001	t	51949	QPRGGQA	Mus musculus
1	1.00	1	1	0	0	0	0	-0.001	t	51296	QLCQLL	Mus musculus
1	1.00	1	1	0	0	0	0	-0.001	t	6546	CLDDREC	Mus musculus
1	1.00	1	1	0	0	0	0	-0.001	t	1578	AGKNQSQKKKK	Mus musculus
1	1.00	1	1	-0.01	-0.007	-0.01	-0.01	-0.012	t	140853	GEYVQILDAHAAYLNATPAA	Mus musculus
1	1.00	1	1	-0.01	-0.01	-0.01	-0.01	-0.01	t	157277	FRAISTPRTGTMP	Canis lupus
1	1.00	1	1	0.04	0.04	0.034	0.042	0.04	t	68987	VIIEQSWGSP	Homo sapiens
1	1.00	1	1	0	0.003	0.006	0	0	t	18786	GATFQVEVPG	Mus musculus

1	1.00	1	1	0.02	0.02	0.015	0.026	0.023	t	114450	LEIIEEFFKNKSGLK	Homo sapiens
1	1.00	1	1	0.03	0.03	0.03	0.03	0.03	t	123273	GTSVSHVDF	Ovis aries
1	1.00	1	1	0.01	0.01	0.01	0.01	0.01	t	111157	CMPYGSLAC	Homo sapiens
1	1.00	1	1	-0.01	-0.01	-0.01	-0.01	-0.01	t	135682	GKVDAFCG	Homo sapiens
1	1.00	1	1	-0.03	-0.03	-0.03	-0.03	-0.029	t	115239	EVFRELKDLK	Homo sapiens
1	1.00	1	1	0.04	0.042	0.035	0.04	0.038	t	136066	IETDKTSVQV	Rattus norvegicus
1	1.00	1	1	0	0	-0.001	0.003	0	t	50027	PWFLAHHTGGD	Sus scrofa
1	1.00	1	1	0	0	0	0.003	0	t	135571	LFRGRRASQ	Oryctolagus cuniculus
1	1.00	1	1	0	0	0	0.003	0	t	135595	SNKKKRDDFE	Oryctolagus cuniculus
1	1.00	1	1	0	0	0	0.003	0	t	134337	CYCQDPYVKEAENLKKYFNA	Oryctolagus cuniculus
1	1.00	1	1	0	0.002	0	0.003	0	t	157917	RTVKKNIIEEN	Homo sapiens
1	1.00	1	1	0	0	0	0.003	0	t	134337	CYCQDPYVKEAENLKKYFNA	Oryctolagus cuniculus
1	1.00	1	1	0	0.002	0	0.003	0	t	157147	CSRTVKKNIIEEN	Homo sapiens
1	1.00	1	1	0	0	0	0.003	0	t	123016	AIYENEIHALWADLP	Trichosurus vulpecula

1	1.00	1	1	0	0	0	0.003	0	t	125793	CSFWSKYISSLKTSADGAK	Mus musculus
1	1.00	1	1	0	0	0	0.003	0	t	125462	KLPLSLSFLHLTRADLSYPS	Mus musculus
1	1.00	1	1	0	0	0	0.003	0	t	123809	VAVESRNGSRLHFNK	Trichosurus vulpecula
1	1.00	1	1	0	0	0	0.003	0	t	125791	CSFWSKYIQTLK	Mus musculus
1	1.00	1	1	0	0	0	0.003	0	t	123540	NIFEYEVDAEPLRPCELER	Mus musculus
1	1.00	1	1	0	0.015	0	0.003	0	t	125540	QSLRQRKSVNALNSPLHQEY	Homo sapiens
1	1.00	1	1	0	0	0	0.003	0	t	107307	DAEFGHDSGF	Mus musculus
1	1.00	1	1	0	0	0	0.003	0	t	105207	GAIIGLMVGGVVIA	Mus musculus
1	1.00	1	1	0	0	0	0.003	0	t	105207	GAIIGLMVGGVVIA	Mus musculus
1	1.00	1	1	0	-0.002	0	0.003	0	t	111583	NKFGFPDTSFYNPDTQRLVW	Homo sapiens
1	1.00	1	1	0	-0.002	0	0.003	0	t	111450	KGSPCTNVAVNPGDCPPLEL	Homo sapiens
1	1.00	1	1	0	0	0	0.003	0	t	105207	GAIIGLMVGGVVIA	Mus musculus
1	1.00	1	1	0	-0.002	0	0.003	0	t	110685	PMDTFIVSTNPNTVTSSTPI	Homo sapiens
1	1.00	1	1	0	0	0	0.003	0	t	100508	SGYEVHHQKL	Mus musculus
1	1.00	1	1	0	-0.002	0	0.003	0	t	111088	ACQKHTPPAPKEDDPLKKYT	Homo sapiens
1	1.00	1	1	0	-0.002	0	0.003	0	t	110652	LCLIGCKPPIGEHWGKGSPC	Homo sapiens

1	1.00	1	1	0	0	0	0.003	0	t	110884	HEYMLDLQPETTDLYCYE	Mus musculus
1	1.00	1	1	0	0	0	0.003	0	t	107307	DAEFGHDSGF	Mus musculus
1	1.00	1	1	0	-0.002	0	0.003	0	t	111915	VHTGFGAMDFTTLQANKSEV	Homo sapiens
1	1.00	1	1	0	0	0	0.003	0	t	45013	NMPASMKRVRIE	Rattus norvegicus
1	1.00	1	1	0	0	0	0.003	0	t	21254	GMDGTQLDRW	Homo sapiens
1	1.00	1	1	0	0	0	0.003	0	t	30661	KEVPALTAVETGAT	Homo sapiens
1	1.00	1	1	0	0	0	0.003	0	t	35646	LEPGVVAEKVRNLPAG	Rattus norvegicus
1	1.00	1	1	-0.01	-0.013	-0.022	-0.01	-0.009	t	10683	DVNKSAQL	Oryctolagus cuniculus
1	1.00	1	1	0.01	0.01	0.006	0.012	0.01	t	71839	VVSTLPQAVMGSSY	Homo sapiens
1	1.00	1	1	0.01	0.014	0.01	0.017	0.01	t	135498	SCKLPVKKATVVQGERVKIQ	Homo sapiens
1	1.00	1	1	0	0	0	0	0	t	132938	WGSNPAM	Homo sapiens
1	1.00	1	1	-0.01	-0.011	-0.013	-0.01	-0.01	t	12537	EINTLQNQ	Anas platyrhynchos
1	1.00	1	1	-0.06	-0.058	-0.081	-0.063	-0.059	t	53483	REECKCLLNYKQEGDKCVE	Homo sapiens
1	1.00	1	1	-0.01	-0.008	-0.01	-0.003	-0.009	t	108135	SLITAARGAPS	Homo sapiens
1	1.00	1	1	0	-0.002	0	0.003	0	t	142288	SEASRKGLRRDLDASREAKK	Mus musculus

1	1.00	1	1	0	-0.002	0	0.003	0	t	142167	GLRRDLDASREAKKQVEKAL	Mus musculus
1	1.00	1	1	0	-0.002	0	0.003	0	t	142200	KGLRRDLDASREAKKQLEAE	Mus musculus
1	1.00	1	1	0	-0.002	0	0.003	0	t	142201	KGLRRDLDASREAKKQVEKA	Mus musculus
1	1.00	1	1	0	-0.002	0	0.003	0	t	142217	LDASREAKKQLEAEQQKLEE	Mus musculus
1	1.00	1	1	0	-0.002	0	0.003	0	t	142202	KISEASRKGLRRDLDASREA	Mus musculus
1	1.00	1	1	0	-0.002	0	0.003	0	t	142116	ASRKGLRRDLDASREAKKQV	Mus musculus
1	1.00	1	1	0	-0.004	0	0.003	0	t	118490	LKDKYKNIGAKLV	Oryctolagus cuniculus
1	1.00	1	1	0	-0.004	0	0.003	0	t	118275	ATVLARSIAKEGF	Oryctolagus cuniculus
1	1.00	1	1	0	-0.004	0	0.003	0	t	118451	KGIIDPTKVVRTA	Oryctolagus cuniculus
1	1.00	1	1	0	-0.002	0	0.003	0	t	95819	SAQSGTSGTSGT	Mus musculus
1	1.00	1	1	0	-0.002	0	0.003	0	t	70537	VQDGSVTEQIIEELFP	Mus musculus
1	1.00	1	1	0	-0.002	0	0.003	0	t	95818	SAQSGTSGTSAQ	Mus musculus
1	1.00	1	1	0	-0.002	0	0.003	0	t	72476	WGILSRAATNSGWVGLIPHK	Rattus norvegicus

1	1.00	1	1	0	-0.002	0	0.003	0	t	95818	SAQSGTSG TSAQ	Mus musculu s
1	1.00	1	1	0	-0.002	0	0.003	0	t	94738	WAICKRIP NKKP	Mus musculu s
1	1.00	1	1	0	-0.002	0	0.003	0	t	94613	NNPTCWA ICKRI	Mus musculu s
1	1.00	1	1	0	-0.002	0	0.003	0	t	75273	YPEELVEE VIPEELVEE VIP	Mus musculu s
1	1.00	1	1	0	-0.004	0	0.003	0	t	70537	VQDGSVT EQIIEELFP	Oryctola gus cuniculu s
1	1.00	1	1	0	-0.002	0	0.003	0	t	94651	PTCWAICK RIPN	Mus musculu s
1	1.00	1	1	0	-0.002	0	0.003	0	t	62912	TALKKAITA MSQAQKA AKPA	Mus musculu s
1	1.00	1	1	0	-0.002	0	0.003	0	t	66682	TTKPTKKP TFKT	Mus musculu s
1	1.00	1	1	0	-0.002	0	0.003	0	t	65916	TQPSKPTT KQRQ	Mus musculu s
1	1.00	1	1	0	-0.002	0	0.003	0	t	94429	AICKRIPNK KPG	Mus musculu s
1	1.00	1	1	0	-0.002	0	0.003	0	t	69344	VKYTS	Mus musculu s
1	1.00	1	1	0	-0.002	0	0.003	0	t	69271	VKMAETC PIFYDVF	Mus musculu s
1	1.00	1	1	0	-0.004	0	0.003	0	t	62354	SVTEEVVE EEGPVDEE	Oryctola gus cuniculu s
1	1.00	1	1	0	-0.002	0	0.003	0	t	94626	NPTCWAI CKRIP	Mus musculu s
1	1.00	1	1	0	-0.002	0	0.003	0	t	75561	YQTELARV QKANADA KATY	Mus musculu s
1	1.00	1	1	0	-0.002	0	0.003	0	t	94695	SNNPTCW AICKR	Mus musculu s

1	1.00	1	1	0	-0.002	0	0.003	0	t	69324	VKTKNTTT TQTQ	Mus musculus
1	1.00	1	1	0	-0.002	0	0.003	0	t	63344	TEEIDEIES VTEEVVE	Mus musculus
1	1.00	1	1	0	-0.002	0	0.003	0	t	95827	SGASAQS GASAQ	Mus musculus
1	1.00	1	1	0	-0.002	0	0.003	0	t	95827	SGASAQS GASAQ	Mus musculus
1	1.00	1	1	0	-0.002	0	0.003	0	t	95901	TEKPLEPA PVEPSYEA EPTP	Mus musculus
1	1.00	1	1	0	-0.002	0	0.003	0	t	75274	YPEEVVEE VVPEEVVE EVVP	Mus musculus
1	1.00	1	1	0	-0.002	0	0.003	0	t	64653	TKQRQNK PPNKP	Mus musculus
1	1.00	1	1	0	-0.002	0	0.003	0	t	94573	KRIPNKKP GKKT	Mus musculus
1	1.00	1	1	0	-0.002	0	0.003	0	t	95815	SAQSGASA QSGT	Mus musculus
1	1.00	1	1	0	-0.002	0	0.003	0	t	95815	SAQSGASA QSGT	Mus musculus
1	1.00	1	1	0	-0.002	0	0.003	0	t	71285	VTEEIVTEE IVTEEI	Mus musculus
1	1.00	1	1	0	-0.002	0	0.003	0	t	95830	SGASAQS GTSGT	Mus musculus
1	1.00	1	1	0	-0.002	0	0.003	0	t	95815	SAQSGASA QSGT	Mus musculus
1	1.00	1	1	0	-0.002	0	0.003	0	t	71285	VTEEIVTEE IVTEEI	Mus musculus

1	1.00	1	1	0	-0.002	0	0.003	0	t	95830	SGASAQSGTSGT	Mus musculus
1	1.00	1	1	0	-0.002	0	0.003	0	t	75455	YQAKLTAYQTELARVQKAN	Mus musculus
1	1.00	1	1	0	-0.002	0	0.003	0	t	75273	YPEELVEEVIPEELVEEVIP	Mus musculus
1	1.00	1	1	0	-0.002	0	0.003	0	t	64606	TKKPTFKTTKKD	Mus musculus
1	1.00	1	1	0	-0.004	0	0.003	0	t	62354	SVTEEVVEEEGPVDEE	Oryctolagus cuniculus
1	1.00	1	1	0	-0.002	0	0.003	0	t	95842	SGTSAQSGTSGT	Mus musculus
1	1.00	1	1	0	-0.002	0	0.003	0	t	66683	TTKQRQNKPPNK	Mus musculus
1	1.00	1	1	0	-0.002	0	0.003	0	t	95829	SGASAQSGTSGP	Mus musculus
1	1.00	1	1	0	-0.002	0	0.003	0	t	75274	YPEEVVEEVVPEEVVEEVVP	Mus musculus
1	1.00	1	1	0	-0.002	0	0.003	0	t	71285	VTEEIVTEEIVTEEI	Mus musculus
1	1.00	1	1	0	-0.002	0	0.003	0	t	95694	PVKPTAPTKPTYETEKPLKP	Mus musculus
1	1.00	1	1	0	-0.002	0	0.003	0	t	95842	SGTSAQSGTSGT	Mus musculus
1	1.00	1	1	0	-0.002	0	0.003	0	t	63345	TEEIIQGESKVEEVVE	Mus musculus
1	1.00	1	1	0	-0.002	0	0.003	0	t	67054	TVKTKNTTTTQT	Mus musculus

1	1.00	1	1	0	-0.002	0	0.003	0	t	95814	SAQSGASAQSGA	Mus musculus
1	1.00	1	1	0	-0.002	0	0.003	0	t	74475	YKLNFYFDLLRAKL	Mus musculus
1	1.00	1	1	0	-0.002	0	0.003	0	t	95828	SGASAQSGTSAQ	Mus musculus
1	1.00	1	1	0	-0.002	0	0.003	0	t	63345	TEEIIQGESKVEEVVE	Mus musculus
1	1.00	1	1	0	-0.002	0	0.003	0	t	62354	SVTEEVVEEEGPVDEE	Mus musculus
1	1.00	1	1	0	-0.002	0	0.003	0	t	94550	ICKRIPNKKPGK	Mus musculus
1	1.00	1	1	0	-0.002	0	0.003	0	t	71285	VTEEIVTEEIVTEEI	Mus musculus
1	1.00	1	1	0	-0.002	0	0.003	0	t	52245	QRQNKPPNKPNN	Mus musculus
1	1.00	1	1	0	-0.002	0	0.003	0	t	39597	LSQDEIDRMVNDAEKY	Mus musculus
1	1.00	1	1	0	-0.002	0	0.003	0	t	47220	PDVPYVKRVK	Mus musculus
1	1.00	1	1	0	-0.004	0	0.003	0	t	39597	LSQDEIDRMVNDAEKY	Saimiri sciureus
1	1.00	1	1	0	-0.002	0	0.003	0	t	39597	LSQDEIDRMVNDAEKY	Mus musculus
1	1.00	1	1	0	-0.002	0	0.003	0	t	51967	QPSKPTTKQRQN	Mus musculus
1	1.00	1	1	0	-0.002	0	0.003	0	t	40575	LVSEEIVTEEGSVAQE	Mus musculus

1	1.00	1	1	0	-0.004	0	0.003	0	t	40575	LVSEEIVTEEGSVAQE	Oryctolagus cuniculus
1	1.00	1	1	0	-0.004	0	0.003	0	t	40575	LVSEEIVTEEGSVAQE	Oryctolagus cuniculus
1	1.00	1	1	0	-0.002	0	0.003	0	t	43116	NAAGNAAGNAAGNAAG	Mus musculus
1	1.00	1	1	0	-0.002	0	0.003	0	t	48662	PNKKPGKKTTTK	Mus musculus
1	1.00	1	1	0	-0.002	0	0.003	0	t	40110	LTPLEELY	Mus musculus
1	1.00	1	1	0	-0.002	0	0.003	0	t	44571	NKPPNKPNNDFH	Mus musculus
1	1.00	1	1	0	-0.002	0	0.003	0	t	49683	PTKKPTFKTTKK	Mus musculus
1	1.00	1	1	0	-0.004	0	0.003	0	t	58957	SKVEEVVEEQGSENEE	Oryctolagus cuniculus
1	1.00	1	1	0	-0.004	0	0.003	0	t	58957	SKVEEVVEEQGSENEE	Oryctolagus cuniculus
1	1.00	1	1	0	-0.002	0	0.003	0	t	43191	NAKATYEAALKQYEADLAA	Mus musculus
1	1.00	1	1	0	-0.002	0	0.003	0	t	58957	SKVEEVVEEQGSENEE	Mus musculus
1	1.00	1	1	0	-0.004	0	0.003	0	t	58251	SGTEEILEKVSASQEI	Oryctolagus cuniculus
1	1.00	1	1	0	-0.002	0	0.003	0	t	58957	SKVEEVVEEQGSENEE	Mus musculus

1	1.00	1	1	0	-0.014	0	0.003	0	t	43233	NANP	Macaca mulatta
1	1.00	1	1	0	-0.002	0	0.003	0	t	51729	QNKPPNK PNNDF	Mus musculu s
1	1.00	1	1	0	-0.004	0	0.003	0	t	58251	SGTEEILEK VSASQEI	Oryctola gus cuniculu s
1	1.00	1	1	0	-0.002	0	0.003	0	t	55457	RQNKPPN KPNND	Mus musculu s
1	1.00	1	1	0	-0.002	0	0.003	0	t	58251	SGTEEILEK VSASQEI	Mus musculu s
1	1.00	1	1	0	-0.002	0	0.003	0	t	58251	SGTEEILEK VSASQEI	Mus musculu s
1	1.00	1	1	0	-0.002	0	0.003	0	t	58921	SKPTTKQR QNKP	Mus musculu s
1	1.00	1	1	0	-0.002	0	0.003	0	t	45817	NSGCFRHL DEREECKC LL	Mus musculu s
1	1.00	1	1	0	-0.002	0	0.003	0	t	49414	PSKPTTKQ RQNK	Mus musculu s
1	1.00	1	1	0	-0.004	0	0.003	0	t	43730	NEREDERT LTKEYEDIV LK	Oryctola gus cuniculu s
1	1.00	1	1	0	-0.002	0	0.003	0	t	43730	NEREDERT LTKEYEDIV LK	Mus musculu s
1	1.00	1	1	0	-0.002	0	0.003	0	t	43248	NANPNAN PNANPNA NPNANP	Mus musculu s
1	1.00	1	1	0	-0.004	0	0.003	0	t	60424	SQEIVQNE SGTEEILE	Oryctola gus cuniculu s
1	1.00	1	1	0	-0.002	0	0.003	0	t	48681	PNNDFHF EVFNF	Mus musculu s

1	1.00	1	1	0	-0.002	0	0.003	0	t	60424	SQEIVQNE SGTEEILE	Mus musculu s
1	1.00	1	1	0	-0.002	0	0.003	0	t	49137	PQTTKPKE VPTT	Mus musculu s
1	1.00	1	1	0	-0.004	0	0.003	0	t	53195	RARGRGR GRGEKRP	Oryctola gus cuniculu s
1	1.00	1	1	0	-0.004	0	0.003	0	t	48618	PNDPNRN VD	Oryctola gus cuniculu s
1	1.00	1	1	0	-0.002	0	0.003	0	t	48618	PNDPNRN VD	Mus musculu s
1	1.00	1	1	0	-0.004	0	0.003	0	t	60424	SQEIVQNE SGTEEILE	Oryctola gus cuniculu s
1	1.00	1	1	0	-0.002	0	0.003	0	t	43435	NDFHFEVF NFVP	Mus musculu s
1	1.00	1	1	0	-0.002	0	0.003	0	t	60424	SQEIVQNE SGTEEILE	Mus musculu s
1	1.00	1	1	0	-0.004	0	0.003	0	t	45817	NSGCFRHL DEREECKC LL	Oryctola gus cuniculu s
1	1.00	1	1	0	-0.002	0	0.003	0	t	45054	NNDFHFE VFNFV	Mus musculu s
1	1.00	1	1	0	-0.002	0	0.003	0	t	48345	PLGLRSEF PI	Mus musculu s
1	1.00	1	1	0	-0.002	0	0.003	0	t	49786	PTTKQRQ NKPPN	Mus musculu s
1	1.00	1	1	0	-0.002	0	0.003	0	t	11927	EEVVGEEK LVSEEIVT	Mus musculu s
1	1.00	1	1	0	-0.002	0	0.003	0	t	11927	EEVVGEEK LVSEEIVT	Mus musculu s

1	1.00	1	1	0	-0.004	0	0.003	0	t	11927	EEVVGEEKLVSEEIVT	Oryctolagus cuniculus
1	1.00	1	1	0	-0.004	0	0.003	0	t	11927	EEVVGEEKLVSEEIVT	Oryctolagus cuniculus
1	1.00	1	1	0	-0.002	0	0.003	0	t	18536	GACAPYRRLHLCD	Rattus norvegicus
1	1.00	1	1	0	-0.002	0	0.003	0	t	29248	IVEEDAPATEEIDEIE	Mus musculus
1	1.00	1	1	0	-0.002	0	0.003	0	t	29248	IVEEDAPATEEIDEIE	Mus musculus
1	1.00	1	1	0	-0.002	0	0.003	0	t	32827	KPPNKPNNDFHF	Mus musculus
1	1.00	1	1	0	-0.002	0	0.003	0	t	23770	HFEVFNFVPCSI	Mus musculus
1	1.00	1	1	0	-0.002	0	0.003	0	t	32723	KPGKKTTTKPTK	Mus musculus
1	1.00	1	1	0	-0.002	0	0.003	0	t	34197	KVSASQEIVQDGSVTE	Mus musculus
1	1.00	1	1	0	-0.002	0	0.003	0	t	13834	EQGSENEEIFVEEVSA	Mus musculus
1	1.00	1	1	0	-0.002	0	0.003	0	t	13834	EQGSENEEIFVEEVSA	Mus musculus
1	1.00	1	1	0	-0.004	0	0.003	0	t	13834	EQGSENEEIFVEEVSA	Oryctolagus cuniculus
1	1.00	1	1	0	-0.002	0	0.003	0	t	34197	KVSASQEIVQDGSVTE	Mus musculus

1	1.00	1	1	0	-0.002	0	0.003	0	t	11821	EENVEHDAEENVEHDA	Mus musculus
1	1.00	1	1	0	-0.004	0	0.003	0	t	11652	EEGPVDEEIVQEEGTV	Oryctolagus cuniculus
1	1.00	1	1	0	-0.002	0	0.003	0	t	11652	EEGPVDEEIVQEEGTV	Mus musculus
1	1.00	1	1	0	-0.004	0	0.003	0	t	29409	IVQEEGTVTEEIIQGE	Oryctolagus cuniculus
1	1.00	1	1	0	-0.002	0	0.003	0	t	29409	IVQEEGTVTEEIIQGE	Mus musculus
1	1.00	1	1	0	-0.002	0	0.003	0	t	29409	IVQEEGTVTEEIIQGE	Mus musculus
1	1.00	1	1	0	-0.002	0	0.003	0	t	31254	KIFQDHPLQK	Mus musculus
1	1.00	1	1	0	-0.002	0	0.003	0	t	29409	IVQEEGTVTEEIIQGE	Mus musculus
1	1.00	1	1	0	-0.002	0	0.003	0	t	11652	EEGPVDEEIVQEEGTV	Mus musculus
1	1.00	1	1	0	-0.004	0	0.003	0	t	11652	EEGPVDEEIVQEEGTV	Oryctolagus cuniculus
1	1.00	1	1	0	-0.002	0	0.003	0	t	11652	EEGPVDEEIVQEEGTV	Mus musculus
1	1.00	1	1	0	-0.002	0	0.003	0	t	11652	EEGPVDEEIVQEEGTV	Mus musculus
1	1.00	1	1	0	-0.002	0	0.003	0	t	11820	EENVEHDAEENVEENV	Mus musculus

1	1.00	1	1	0	-0.002	0	0.003	0	t	11661	EEGSVAQE IVEEDAPA	Mus musculu s
1	1.00	1	1	0	-0.002	0	0.003	0	t	11661	EEGSVAQE IVEEDAPA	Mus musculu s
1	1.00	1	1	0	-0.004	0	0.003	0	t	11661	EEGSVAQE IVEEDAPA	Oryctola gus cuniculu s
1	1.00	1	1	0	-0.002	0	0.003	0	t	11661	EEGSVAQE IVEEDAPA	Mus musculu s
1	1.00	1	1	0	-0.004	0	0.003	0	t	11661	EEGSVAQE IVEEDAPA	Oryctola gus cuniculu s
1	1.00	1	1	0	-0.002	0	0.003	0	t	17707	FSFCETNG LE	Mus musculu s
1	1.00	1	1	0	-0.002	0	0.003	0	t	26870	IKPGSANK PKDELDYE NDIE	Mus musculu s
1	1.00	1	1	0	-0.004	0	0.003	0	t	26870	IKPGSANK PKDELDYE NDIE	Saimiri sciureus
1	1.00	1	1	0	-0.002	0	0.003	0	t	16103	FHFEVFNF VPCS	Mus musculu s
1	1.00	1	1	0	-0.002	0	0.003	0	t	27395	ILVEGSVTE EVVGEEK	Mus musculu s
1	1.00	1	1	0	-0.004	0	0.003	0	t	27395	ILVEGSVTE EVVGEEK	Oryctola gus cuniculu s
1	1.00	1	1	0	-0.002	0	0.003	0	t	27395	ILVEGSVTE EVVGEEK	Mus musculu s
1	1.00	1	1	0	-0.004	0	0.003	0	t	27395	ILVEGSVTE EVVGEEK	Oryctola gus cuniculu s

1	1.00	1	1	0	-0.002	0	0.003	0	t	15323	FCETNGLEYI	Mus musculus
1	1.00	1	1	0	-0.002	0	0.003	0	t	33892	KTTTKPTKKPTF	Mus musculus
1	1.00	1	1	0	-0.002	0	0.003	0	t	15348	FCSLTSLATSCGAAVFIPFR	Rattus norvegicus
1	1.00	1	1	0	-0.002	0	0.003	0	t	25852	IEKKIAKMEKASSVFNVV	Mus musculus
1	1.00	1	1	0	-0.004	0	0.003	0	t	32769	KPKHKKLKQPGDGNP	Oryctolagus cuniculus
1	1.00	1	1	0	-0.002	0	0.003	0	t	32769	KPKHKKLKQPGDGNP	Mus musculus
1	1.00	1	1	0	-0.002	0	0.003	0	t	32769	KPKHKKLKQPGDGNP	Mus musculus
1	1.00	1	1	0	-0.002	0	0.003	0	t	32901	KPTKKPTFKTTK	Mus musculus
1	1.00	1	1	0	-0.002	0	0.003	0	t	28075	IPSTAFTDIAWVRLPNHY	Rattus norvegicus
1	1.00	1	1	0	-0.004	0	0.003	0	t	28075	IPSTAFTDIAWVRLPNHY	Oryctolagus cuniculus
1	1.00	1	1	0	-0.004	0	0.003	0	t	28075	IPSTAFTDIAWVRLPNHY	Oryctolagus cuniculus
1	1.00	1	1	0	-0.002	0	0.003	0	t	21373	GNAEKYDKMDEPQHYGKS	Mus musculus
1	1.00	1	1	0	-0.004	0	0.003	0	t	21373	GNAEKYDKMDEPQHYGKS	Oryctolagus cuniculus
1	1.00	1	1	0	-0.002	0	0.003	0	t	28074	IPSTACTDIAWVRLPCHYG	Rattus norvegicus

1	1.00	1	1	0	-0.002	0	0.003	0	t	29965	KATYEAAV AANNAKN AALT	Mus musculu s
1	1.00	1	1	0	-0.004	0	0.003	0	t	26178	IFVEEVSAS QEIVQNE	Oryctola gus cuniculu s
1	1.00	1	1	0	-0.004	0	0.003	0	t	26178	IFVEEVSAS QEIVQNE	Oryctola gus cuniculu s
1	1.00	1	1	0	-0.002	0	0.003	0	t	33063	KQRQNKP PNKPN	Mus musculu s
1	1.00	1	1	0	-0.002	0	0.003	0	t	34948	LARSFADI GDIVRGKD LY	Rattus norvegic us
1	1.00	1	1	0	-0.002	0	0.003	0	t	27961	IPNKKPGK KTTT	Mus musculu s
1	1.00	1	1	0	-0.002	0	0.003	0	t	20012	GGSGGSV ASGGSVAS	Mus musculu s
1	1.00	1	1	0	-0.002	0	0.003	0	t	34965	LASLRKAF ADTVPVRP PSNY	Rattus norvegic us
1	1.00	1	1	0	-0.002	0	0.003	0	t	12412	EIDAPRVF DARFSRHY SVQL	Rattus norvegic us
1	1.00	1	1	0	-0.004	0	0.003	0	t	34965	LASLRKAF ADTVPVRP PSNY	Oryctola gus cuniculu s
1	1.00	1	1	0	-0.002	0	0.003	0	t	13644	EPERTAM KKIQDCY	Mus musculu s
1	1.00	1	1	0	-0.004	0	0.003	0	t	34965	LASLRKAF ADTVPVRP PSNY	Oryctola gus cuniculu s
1	1.00	1	1	0	-0.002	0	0.003	0	t	29897	KANADAK AAYEAAV AANNA	Mus musculu s
1	1.00	1	1	0	-0.002	0	0.003	0	t	13438	ENANANN AV	Mus musculu s

1	1.00	1	1	0	-0.002	0	0.003	0	t	12408	EICPAVKRDVDLFLTG	Mus musculus
1	1.00	1	1	0	-0.002	0	0.003	0	t	31580	KKIQDCYVENGLIS	Mus musculus
1	1.00	1	1	0	-0.002	0	0.003	0	t	10069	DRYIPYSP	Mus musculus
1	1.00	1	1	0	-0.002	0	0.003	0	t	9694	DPNANPNVDPNANPNV	Mus musculus
1	1.00	1	1	0	-0.002	0	0.003	0	t	6222	CFDYAPYVSAVDDIC	Mus musculus
1	1.00	1	1	0	-0.002	0	0.003	0	t	751	ADPVTTEGDYVVKISEFYGR	Mus musculus
1	1.00	1	1	0	-0.002	0	0.003	0	t	8448	DGNCEDIPHVNEFSAIDL	Mus musculus
1	1.00	1	1	0	-0.002	0	0.003	0	t	9694	DPNANPNVDPNANPNV	Mus musculus
1	1.00	1	1	0	-0.002	0	0.003	0	t	7971	DEENRKRIEARNSLEN	Mus musculus
1	1.00	1	1	0	-0.002	0	0.003	0	t	3221	ANAANEADYQAKLTAYQTE	Mus musculus
1	1.00	1	1	0	-0.002	0	0.003	0	t	8448	DGNCEDIPHVNEFSAIDL	Mus musculus
1	1.00	1	1	0	-0.004	0	0.003	0	t	8448	DGNCEDIPHVNEFSAIDL	Oryctolagus cuniculus
1	1.00	1	1	0	-0.002	0	0.003	0	t	3221	ANAANEADYQAKLTAYQTE	Mus musculus
1	1.00	1	1	0	-0.002	0	0.003	0	t	8448	DGNCEDIPHVNEFSAIDL	Mus musculus

1	1.00	1	1	0	-0.002	0	0.003	0	t	3221	ANAANEA DYQAKLTA YQTE	Mus musculu s
1	1.00	1	1	0	-0.002	0	0.003	0	t	1975	AIKKRNAD AKADYEAK LAK	Mus musculu s
1	1.00	1	1	0	-0.002	0	0.003	0	t	8448	DGNCEDIP HVNEFSAI DL	Mus musculu s
1	1.00	1	1	0	-0.002	0	0.003	0	t	333	AANNAAN AALTAENT AIKK	Mus musculu s
1	1.00	1	1	0	-0.002	0	0.003	0	t	8275	DFHFEVFN FVPC	Mus musculu s
1	1.00	1	1	-0.02	-0.018	-0.029	-0.023	-0.019	t	113535	IGGRVHFF KDISPIA	Homo sapiens
1	1.00	1	1	-0.01	-0.014	-0.022	-0.011	-0.009	t	116971	FAVLKLM GRGTKF	Oryctola gus cuniculu s
1	1.00	1	1	0.03	0.026	0.03	0.03	0.029	t	123001	AASEDPR	Mus musculu s
1	1.00	1	1	-0.02	-0.02	-0.02	-0.02	-0.021	t	105650	AEFRH	Mus musculu s
1	1.00	1	1	-0.02	-0.02	-0.02	-0.02	-0.021	t	105650	AEFRH	Mus musculu s
1	1.00	1	1	-0.02	-0.02	-0.02	-0.02	-0.021	t	105650	AEFRH	Mus musculu s
1	1.00	1	1	0.04	0.04	0.044	0.04	0.04	t	23207	GVVTLY	Homo sapiens
1	1.00	1	1	0.02	0.016	0.019	0.019	0.018	t	50396	QAYQQGV TVD	Oryctola gus cuniculu s
1	1.00	1	1	0	-0.006	0	0	-0.002	t	123010	AGAMIPP PPS	Oryctola gus cuniculu s
1	1.00	1	1	0	0	-0.005	0	0	t	76396	YVRGGGA VP	Homo sapiens
1	1.00	1	1	0	0	0	0.004	0	t	135945	KFGADAR ALMLQ	Homo sapiens

1	1.00	1	1	0.03	0.03	0.039	0.03	0.03	t	55981	RSTSLS	Homo sapiens
1	1.00	1	1	0.03	0.035	0.039	0.03	0.03	t	55981	RSTSLS	Homo sapiens
1	1.00	1	1	0.02	0.022	0.016	0.023	0.02	t	39253	LRYHNKVYCTTSKSA	Homo sapiens
1	1.00	1	1	-0.02	-0.02	-0.02	-0.02	-0.019	t	115427	PTQLEEQCHI	Homo sapiens
1	1.00	1	1	0.02	0.02	0.018	0.02	0.02	t	95480	ITELSEFIPGAS	Homo sapiens
1	1.00	1	1	-0.01	-0.01	-0.01	-0.01	-0.012	t	5575	AVTPLGPPAAEY	Oryctolagus cuniculus
1	1.00	1	1	0.06	0.06	0.06	0.06	0.06	t	151612	DPLLTGNYKEAS	Mus musculus
1	1.00	1	1	0.04	0.04	0.04	0.04	0.04	t	123278	GYLKDLPTT	Ovis aries
1	1.00	1	1	0.02	0.02	0.02	0.02	0.02	t	102139	TLQPDRS	Mus musculus
1	1.00	1	1	0.02	0.02	0.02	0.02	0.02	t	6292	CGGTPPSLPTC	Homo sapiens
1	1.00	1	1	0.01	0.01	0	0.01	0.012	t	21673	GPKGRNVVLEKKWGAP	Rattus norvegicus
1	1.00	1	1	-0.02	-0.028	-0.022	-0.02	-0.021	t	97223	AWNPSLLG	Mus musculus
1	1.00	1	1	0.04	0.042	0.04	0.047	0.04	t	103993	IPPSLRTLEDNEERMS	Homo sapiens
1	1.00	1	1	-0.01	-0.007	-0.013	-0.01	-0.008	t	52825	QVYYRPVDQ	Mus musculus
1	1.00	1	1	0.01	0.01	0.01	0.01	0.01	t	11699	EEITQVATISANSDH	Homo sapiens

1	1.00	1	1	0	0.001	-0.004	0.001	0	t	61192	SSLRYGNV LDVNAI	Homo sapiens
1	1.00	1	1	0	0.001	-0.004	0.001	0	t	30597	KERSSLRY GNVLDV	Homo sapiens
1	1.00	1	1	0	0.001	-0.004	0.001	0	t	22799	GTRIRFKD DTSFED	Homo sapiens
1	1.00	1	1	0	0.001	-0.004	0.001	0	t	11354	EDINGIRR PKHLYV	Homo sapiens
1	1.00	1	1	0.02	0.022	0.016	0.014	0.02	t	112837	SGYIPANT TIPF	Homo sapiens
1	1.00	1	1	0.01	0.01	0.01	0.01	0.008	t	119573	EWEDTYGI VL	Homo sapiens
1	1.00	1	1	0.01	0.01	0.006	0.01	0.009	t	105684	ASFDKVKG DPVGILYA VFKA	Homo sapiens
1	1.00	1	1	0.01	0.01	0.01	0.01	0.01	t	23191	GVTYEIDL TNKNAA	Homo sapiens
1	1.00	1	1	-0.03	-0.032	-0.024	-0.029	-0.029	t	79042	PKSKPFA	Mus musculu s
1	1.00	1	1	0	0.002	-0.01	0.001	0.001	t	24012	HHPSTNQ EQTSLYVQ AS	Homo sapiens
1	1.00	1	1	0.03	0.029	0.022	0.029	0.03	t	52477	QTASQVET EDTKEPG	Mus musculu s
1	1.00	1	1	0.01	0.01	0.01	0.01	0.01	t	128540	EKTSFFQA LGITTKI	Homo sapiens
1	1.00	1	1	0	0	0	0	0	t	33746	KTKRNTNR	Homo sapiens
1	1.00	1	1	0.05	0.05	0.05	0.05	0.05	t	115825	KIEKFQSEE Q	Homo sapiens
1	1.00	1	1	0.03	0.03	0.03	0.03	0.03	t	99602	RDQSSYLQ GFSRNTL	Homo sapiens
1	1.00	1	1	0.01	0.01	0.01	0.01	0.01	t	156927	NTFQAFY	Gallus gallus
1	1.00	1	1	0.01	0.012	0.01	0.01	0.01	t	117749	APAMIPP	Homo sapiens
1	1.00	1	1	0.01	0.01	0.01	0.01	0.01	t	117749	APAMIPP	Homo sapiens

1	1.00	1	1	0.01	0.01	0.01	0.01	0.01	t	16123	FHLPYNHNWFAL	Mus musculus
1	1.00	1	1	0.03	0.028	0.025	0.031	0.031	t	14774	EVLYEAFDEMEECASKAALI	Homo sapiens
1	1.00	1	1	-0.02	-0.02	-0.022	-0.017	-0.02	t	70648	VQSVASSRRHKRFAG	Homo sapiens
1	1.00	1	1	-0.03	-0.03	-0.036	-0.03	-0.032	t	59300	SLLRRRRNGEDVSV	Oryctolagus cuniculus
1	1.00	1	1	-0.03	-0.027	-0.036	-0.03	-0.032	t	59300	SLLRRRRNGEDVSV	Mus musculus
1	1.00	1	1	-0.01	-0.008	-0.008	-0.007	-0.01	t	13728	EPPVVHGC	Homo sapiens
1	1.00	1	1	0.02	0.02	0.015	0.02	0.022	t	12182	EGGTWRIG	Homo sapiens
1	1.00	1	1	0.01	0.012	0.007	0.011	0.012	t	47200	PDSDAESYSSMPPL	Homo sapiens
1	1.00	1	1	0.01	0.012	0.007	0.011	0.012	t	47200	PDSDAESYSSMPPL	Homo sapiens
1	1.00	1	1	-0.04	-0.036	-0.04	-0.043	-0.04	t	34180	KVQYDMPK	Homo sapiens
1	1.00	1	1	0	0.002	0	0.007	0.003	t	140606	TMWDALEAP	Homo sapiens
1	1.00	1	1	0	-0.003	0	0	-0.001	t	130872	GSFLTKGPSKLNDRA	Oryctolagus cuniculus
1	1.00	1	1	0	-0.003	0	0	-0.001	t	66841	TTTCFRDTDTGK	Oryctolagus cuniculus
1	1.00	1	1	0	0	0	0	-0.001	t	48819	PPLGNWFG	Rattus norvegicus

1	1.00	1	1	0.03	0.03	0.017	0.032	0.033	t	98968	QQNPQAQGSVQP	Homo sapiens
1	1.00	1	1	0.01	-0.002	0.01	0.013	0.01	t	156871	FQNIASMPHLYV	Bos taurus
1	1.00	1	1	0.01	0.01	0.007	0.01	0.011	t	62715	SYSYNKDNNELSHFA	Mus musculus
1	1.00	1	1	-0.03	-0.03	-0.028	-0.029	-0.028	t	98839	KALLYR	Homo sapiens
1	1.00	1	1	-0.02	-0.018	-0.03	-0.023	-0.021	t	1740	AHAAYLNATPAAKVL	Homo sapiens
1	1.00	1	1	0.01	0.014	0.012	0.013	0.008	t	59983	SPAPPPPEHRGGPEEFEGAG	Homo sapiens
1	1.00	1	1	0.03	0.032	0.029	0.031	0.03	t	40018	LTLNLITEMG	Homo sapiens
1	1.00	1	1	0.02	0.024	0.02	0.021	0.02	t	137844	EYETDPSVTKMV	Homo sapiens
1	1.00	1	1	-0.02	-0.02	-0.025	-0.026	-0.022	t	115574	YWLAHKALCS	Homo sapiens
1	1.00	1	1	0.03	0.032	0.03	0.031	0.029	t	151005	KEVLVLWGIHHPSTS	Homo sapiens
1	1.00	1	1	0.02	0.02	0.02	0.02	0.02	t	70664	VQVVYDYQ	Oryctolagus cuniculus
1	1.00	1	1	0.02	0.018	0.019	0.022	0.022	t	106599	LEFRHSL	Mus musculus
1	1.00	1	1	0	0	-0.012	-0.004	0	t	30799	KFNPDLAPG	Homo sapiens
1	1.00	1	1	-0.02	-0.022	-0.017	-0.017	-0.02	t	5597	AVVGGLGGY	Mus musculus
1	1.00	1	1	0.02	0.02	0.02	0.02	0.02	t	135837	DMYGDIRLTGLI	Mus musculus

1	1.00	1	1	0.02	0.02	0.02	0.02	0.02	t	135837	DMYGDIRL TGLI	Mus musculu s
1	1.00	1	1	0.02	0.02	0.02	0.02	0.02	t	135837	DMYGDIRL TGLI	Mus musculu s
1	1.00	1	1	-0.01	-0.01	-0.01	-0.01	-0.01	t	118217	VTKYITKG WKEVH	Homo sapiens
1	1.00	1	1	0.03	0.03	0.03	0.03	0.033	t	9588	DPAFSL	Mus musculu s
1	1.00	1	1	0.02	0.009	0.016	0.02	0.02	t	147083	LCSEYRNT QIYTIN	Oryctola gus cuniculu s
1	1.00	1	1	-0.01	-0.008	-0.008	-0.003	-0.009	t	122793	HTQNT	Homo sapiens
1	1.00	1	1	0.01	0.01	0.01	0.01	0.011	t	130598	RQFECYTT CVG	Mus musculu s
1	1.00	1	1	0.04	0.04	0.04	0.04	0.04	t	107861	GLEGQDT KTITFDVS	Homo sapiens
1	1.00	1	1	0.02	0.02	0.02	0.02	0.02	t	119592	HTFPAVLQ	Homo sapiens
1	1.00	1	1	0.02	0.02	0.02	0.02	0.02	t	133956	WIMSNF	Mus musculu s
1	1.00	1	1	0	0	0	0	0	t	141862	CIKTLMTN VETYFK	Homo sapiens
1	1.00	1	1	0	0	0	0	0	t	140834	DAVKGYLA GKGV	Oryctola gus cuniculu s
1	1.00	1	1	0.01	0.01	0.01	0.01	0.01	t	98953	QEPDDLK QKA	Homo sapiens
1	1.00	1	1	0.02	0.02	0.021	0.02	0.02	t	35139	LDEGKQSL TKLAAAW G	Homo sapiens
1	1.00	1	1	0	0	0.002	0	-0.001	t	119900	GKTINKIDI DTEAYT	Rattus norvegic us

1	1.00	1	1	0	0	0.002	0	-0.001	t	119900	GKTINKIDI DTEAYT	Rattus norvegic us
1	1.00	1	1	0	0.002	0.002	0	-0.001	t	119900	GKTINKIDI DTEAYT	Rattus norvegic us
1	1.00	1	1	0	0	0.002	0	-0.001	t	119900	GKTINKIDI DTEAYT	Rattus norvegic us
1	1.00	1	1	0	0	0.002	0	-0.001	t	119900	GKTINKIDI DTEAYT	Rattus norvegic us
1	1.00	1	1	0	0	0.002	0	-0.001	t	119900	GKTINKIDI DTEAYT	Rattus norvegic us
1	1.00	1	1	0	0.002	0.002	0	-0.001	t	119900	GKTINKIDI DTEAYT	Rattus norvegic us
1	1.00	1	1	0	-0.003	0.007	0.001	0	t	5029	ATLCSALY VG	Homo sapiens
1	1.00	1	1	-0.01	-0.01	-0.024	-0.013	-0.01	t	17795	FSNVYLFA KDKSGPLQ PGV	Homo sapiens
1	1.00	1	1	-0.02	-0.018	-0.02	-0.02	-0.021	t	122886	RGTDEYFI RKPP	Rattus norvegic us
1	1.00	1	1	0.05	0.049	0.059	0.051	0.05	t	783	ADTQGS	Homo sapiens
1	1.00	1	1	-0.02	-0.016	-0.018	-0.021	-0.022	t	51830	QPFPSQQ PYL	Homo sapiens
1	1.00	1	1	0.01	0.012	0.01	0.01	0.01	t	118052	PPGAMPP	Homo sapiens
1	1.00	1	1	-0.02	-0.02	-0.02	-0.02	-0.022	t	20523	GKCGVSIP YK	Homo sapiens

1	1.00	1	1	0.01	0.018	0.01	0.013	0.01	t	110957	PAAVTLAE ADRT	Homo sapiens
1	1.00	1	1	0.01	0.01	0.01	0.014	0.011	t	135930	GDKVSFFC KNKEKKC	Homo sapiens
1	1.00	1	1	0	0.003	0	0	-0.002	t	135501	SQWNKP	Mus musculu s
1	1.00	1	1	0	0	0.006	0	0	t	37029	LKTAAESA	Mus musculu s
1	1.00	1	1	0.01	0.007	0.01	0.01	0.012	t	140934	YGEEKPAV LGHD	Oryctola gus cuniculu s
1	1.00	1	1	0.06	0.06	0.06	0.06	0.062	t	27557	IMSGEVPS TEDLVN	Homo sapiens
1	1.00	1	1	0.01	0.01	0.01	0.01	0.01	t	156923	NLHTSF	Gallus gallus
1	1.00	1	1	0.01	0.01	0.01	0.01	0.01	t	136126	AKYNQLLR IEEELGSKA	Homo sapiens
1	1.00	1	1	-0.01	-0.01	-0.01	-0.01	-0.009	t	115392	NICNISCD KF	Homo sapiens
1	1.00	1	1	0	0.003	0.002	0	0	t	119641	SEHETRLV AKLFKDYS	Mus musculu s
1	1.00	1	1	0.07	0.07	0.07	0.07	0.07	t	96444	KDEDEEDE ED	Homo sapiens
1	1.00	1	1	0.03	0.03	0.03	0.03	0.03	t	123278	GYLKDLPT T	Ovis aries
1	1.00	1	1	-0.01	-0.01	-0.01	-0.01	-0.01	t	113480	GQQPGQG QPGYYPT	Homo sapiens
1	1.00	1	1	-0.01	-0.01	-0.01	-0.01	-0.01	t	79586	KIPIALS	Oryctola gus cuniculu s
1	1.00	1	1	-0.02	-0.02	-0.02	-0.02	-0.02	t	45637	NQVYYRP	Mus musculu s

1	1.00	1	1	-0.01	-0.007	-0.003	-0.01	-0.01	t	21469	GNLLLSCL	Sus scrofa
1	1.00	1	1	-0.04	-0.042	-0.056	-0.041	-0.04	t	17376	FPQQPQR PFA	Mus musculu s
1	1.00	1	1	0	0.002	0.007	0.007	0	t	65050	TLRVYK	Homo sapiens
1	1.00	1	1	0.02	0.022	0.022	0.02	0.02	t	52844	QWKLQPG D	Anas platyrhy nchos
1	1.00	1	1	-0.01	-0.01	-0.01	-0.01	-0.011	t	47160	PDPPQPD FPQLN	Oryctola gus cuniculu s
1	1.00	1	1	-0.01	-0.012	-0.005	-0.011	-0.011	t	48332	PLGFFPDH QLDPAFG	Mus musculu s
1	1.00	1	1	0.02	0.017	0.01	0.02	0.02	t	140938	YLNATPAA KVLVEGNT DERG	Oryctola gus cuniculu s
1	1.00	1	1	-0.02	-0.02	-0.026	-0.02	-0.02	t	50203	QAAAGDK PS	Homo sapiens
1	1.00	1	1	-0.01	-0.01	-0.017	-0.01	-0.012	t	95543	LCPNNLCC SQ	Homo sapiens
1	1.00	1	1	-0.03	-0.027	-0.039	-0.03	-0.028	t	79995	RAVFMQQ RPLRM	Mus musculu s
1	1.00	1	1	-0.03	-0.032	-0.031	-0.031	-0.03	t	48869	PPPPRVQP RKTKSV	Sus scrofa
1	1.00	1	1	-0.01	-0.013	-0.01	-0.01	-0.012	t	133468	YYYNEIKLK ESYKLKD	Oryctola gus cuniculu s
1	1.00	1	1	0.04	0.04	0.04	0.04	0.04	t	109476	LDSDGSF	Homo sapiens
1	1.00	1	1	0.04	0.04	0.04	0.04	0.04	t	75847	YSPELE	Mus musculu s

1	1.00	1	1	0.04	0.04	0.04	0.04	0.04	t	75847	YSPELE	Mus musculus
1	1.00	1	1	0	0	0	0	0	t	139317	TLLELPAAE	Oryctolagus cuniculus
1	1.00	1	1	-0.01	-0.015	-0.015	-0.01	-0.01	t	41294	MDYVSCTPGLRVELSTVSNA	Canis lupus
1	1.00	1	1	0.03	0.029	0.032	0.033	0.03	t	5112	ATQAATVAVTPEETAVASPP	Homo sapiens
1	1.00	1	1	-0.04	-0.038	-0.045	-0.037	-0.04	t	62326	SVSHARPRWFWFCL	Homo sapiens
1	1.00	1	1	0.02	0.023	0.011	0.02	0.019	t	78627	DPDPPDANDPAPPNAN	Mus musculus
1	1.00	1	1	0.01	0.01	0.002	0.01	0.011	t	65492	TPADPAASSSERG	Mus musculus
1	1.00	1	1	0.02	0.018	0.017	0.023	0.022	t	66751	TTPAQGTSM	Mus musculus
1	1.00	1	1	0.01	0.012	0.01	0.013	0.01	t	51145	QIVGGV	Homo sapiens
1	1.00	1	1	0.01	0.008	-0.002	0.011	0.012	t	96494	KQMGQTGGSY	Mus musculus
1	1.00	1	1	0.01	0.01	0.01	0.01	0.01	t	32635	KNVVTGEVQVLYTA	Homo sapiens
1	1.00	1	1	-0.03	-0.027	-0.039	-0.03	-0.028	t	80090	VTSVAMKPYEVT	Mus musculus
1	1.00	1	1	-0.03	-0.027	-0.039	-0.03	-0.028	t	80090	VTSVAMKPYEVT	Mus musculus
1	1.00	1	1	0.01	0.013	0.011	0.01	0.01	t	149785	KTVEGVKDLQAQ	Mus musculus

1	1.00	1	1	0	-0.004	0	0.001	-0.001	t	131303	PVPSTPPT PSPSTPPT PSPS	Oryctolagus cuniculus
1	1.00	1	1	0	-0.002	0	0.001	-0.001	t	131101	TDSEMDV VRFDDDN S	Mus musculus
1	1.00	1	1	-0.03	-0.03	-0.03	-0.03	-0.031	t	119605	KSRWQQG N	Homo sapiens
1	1.00	1	1	-0.04	-0.036	-0.04	-0.04	-0.041	t	122998	AAMDLRI GF	Ovis aries
1	1.00	1	1	0.03	0.03	0.028	0.033	0.03	t	54335	RKDRVED A	Homo sapiens
1	1.00	1	1	0.02	0.02	0.018	0.02	0.02	t	7155	CTIAALGSS DRDTVV	Homo sapiens
1	1.00	1	1	-0.01	-0.01	-0.01	-0.01	-0.01	t	88981	SPTSVTVG KGAFSFKR E	Aotus
1	1.00	1	1	0	-0.002	-0.02	-0.007	0	t	141778	VYVPPRRQ QLCLYELF	Rattus norvegicus
1	1.00	1	1	0	-0.004	0	0.003	0	t	76396	YVRGGGA VP	Oryctolagus cuniculus
1	1.00	1	1	0	0	0	0	-0.002	t	63756	TFTNKLKE KHTDLGK	Mus musculus
1	1.00	1	1	0.01	0.01	0.01	0.01	0.01	t	142408	DQVTKKEK KTIKKA	Homo sapiens
1	1.00	1	1	0.01	0.01	0.01	0.01	0.01	t	131883	FVKQFSNI SFSRDSP	Homo sapiens
1	1.00	1	1	0.02	0.024	0.02	0.021	0.02	t	137882	GQNVLSA D	Homo sapiens
1	1.00	1	1	0.01	0.007	0.008	0.01	0.01	t	141622	DPNGWTG TDNNFSI	Oryctolagus cuniculus
1	1.00	1	1	-0.04	-0.04	-0.043	-0.04	-0.04	t	8239	DFCGKGY HLMSFPQ AAP	Homo sapiens

1	1.00	1	1	0.02	0.02	0.02	0.02	0.021	t	144676	GTHPSLQFT	Mus musculus
1	1.00	1	1	-0.02	-0.022	-0.011	-0.017	-0.021	t	106192	YCHTFHGAG	Mus musculus
1	1.00	1	1	0	-0.01	0	0.001	0	t	51371	QLINTNGSWHIN	Homo sapiens
1	1.00	1	1	0.04	0.043	0.04	0.04	0.04	t	126897	QSQQFSVSENLLKEA	Mus musculus
1	1.00	1	1	0.02	0.023	0.02	0.02	0.02	t	43506	NDPN	Mus musculus
1	1.00	1	1	-0.01	-0.014	-0.016	-0.011	-0.01	t	18512	GAALLQSARR	Oryctolagus cuniculus
1	1.00	1	1	-0.02	-0.024	-0.016	-0.02	-0.02	t	51974	QPTELRYSW	Mus musculus
1	1.00	1	1	-0.02	-0.02	-0.016	-0.02	-0.02	t	51974	QPTELRYSW	Mus musculus
1	1.00	1	1	-0.02	-0.02	-0.016	-0.02	-0.02	t	51974	QPTELRYSW	Mus musculus
1	1.00	1	1	0	-0.004	0	-0.001	0	t	120681	KILPEVKEKH	Oryctolagus cuniculus
1	1.00	1	1	-0.02	-0.023	-0.028	-0.02	-0.021	t	153388	GKFKKIVKGTHHWMLLTF	Oryctolagus cuniculus
1	1.00	1	1	0.02	0.018	0.02	0.023	0.02	t	95842	SGTSAQSGTSGT	Mus musculus
1	1.00	1	1	-0.01	-0.012	-0.012	-0.007	-0.009	t	99288	GLSERRAQ	Mus musculus
1	1.00	1	1	0	-0.002	0.004	-0.003	0	t	115053	GGSYFMISR	Mus musculus
1	1.00	1	1	0	-0.002	0.004	-0.003	0	t	115053	GGSYFMISR	Mus musculus

1	1.00	1	1	-0.02	-0.018	-0.023	-0.023	-0.022	t	50648	QELQEVD RRCRC	Homo sapiens
1	1.00	1	1	-0.02	-0.02	-0.025	-0.02	-0.02	t	55826	RSELSQLS MYRVFEV	Homo sapiens
1	1.00	1	1	0	0.002	0.005	0.001	0	t	66435	TSSLASL	Homo sapiens
1	1.00	1	1	-0.03	-0.03	-0.038	-0.03	-0.03	t	106739	QKREACKC IVRA	Homo sapiens
1	1.00	1	1	0.03	0.03	0.03	0.03	0.029	t	75849	YSPEVR	Mus musculu s
1	1.00	1	1	0	-0.002	0	0	-0.001	t	7765	DDEHVEEP TVA	Mus musculu s
1	1.00	1	1	-0.01	-0.014	-0.008	-0.011	-0.011	t	4885	ATALDTSN KFADFLQI	Bos taurus
1	1.00	1	1	0	0	0	0	0.002	t	107479	KFNWYVD	Mus musculu s
1	1.00	1	1	-0.03	-0.029	-0.011	-0.024	-0.03	t	78322	YKGHLH	Equus caballus
1	1.00	1	1	-0.01	-0.008	-0.01	-0.009	-0.01	t	22132	GRLITV	Homo sapiens
1	1.00	1	1	0	-0.001	-0.004	0	0	t	11353	EDINGIRR PK	Mus musculu s
1	1.00	1	1	0	0.002	0.002	-0.003	-0.002	t	52600	QTQQPQQ PFP	Homo sapiens
1	1.00	1	1	0.02	0.016	0.02	0.017	0.02	t	119923	GYISKAQE YFN	Oryctola gus cuniculu s
1	1.00	1	1	0.02	0.02	0.02	0.02	0.02	t	123186	FMVYEAL DA	Ovis aries
1	1.00	1	1	0.02	0.02	0.02	0.02	0.02	t	114823	FNPVCGT DGV	Homo sapiens

1	1.00	1	1	-0.01	-0.024	-0.01	-0.01	-0.01	t	137866	GDTRPRFL	Oryctolagus cuniculus
1	1.00	1	1	0.03	0.033	0.038	0.03	0.032	t	30986	KGIYPLSYE	Mus musculus
1	1.00	1	1	0	0	-0.01	0	0	t	50957	QGSGKGVSFQLVKLG	Homo sapiens
1	1.00	1	1	0.01	0.01	0.001	0.01	0.01	t	7809	DDKEKDPQYRALMGENQDLR	Mus musculus
1	1.00	1	1	0.01	0.008	0.005	0.003	0.01	t	129661	PVDGVVLVD	Mus musculus
1	1.00	1	1	0	0	-0.015	0	0.001	t	48618	PNDPNRNVD	Mus musculus
1	1.00	1	1	0.01	0.007	-0.002	0.01	0.01	t	116169	TPRTPPPSQGKG	Oryctolagus cuniculus
1	1.00	1	1	0.01	0.018	-0.002	0.01	0.01	t	116169	TPRTPPPSQGKG	Gallus gallus
1	1.00	1	1	0.02	0.018	0.02	0.027	0.02	t	116434	PGFGYGGRASDYKSAHKG	Homo sapiens
1	1.00	1	1	-0.02	-0.024	-0.013	-0.021	-0.022	t	44598	NKSLGACPIR	Oryctolagus cuniculus
1	1.00	1	1	0.01	0.008	0.011	0.013	0.011	t	66474	TSTVQVFTDSEYQLPYVLGS	Homo sapiens
1	1.00	1	1	0.02	0.02	0.008	0.02	0.023	t	150200	FGEHKQEKDLEYGAC	Mus musculus
1	1.00	1	1	0.04	0.042	0.039	0.043	0.042	t	47858	PHVVDL	Homo sapiens
1	1.00	1	1	-0.01	-0.012	-0.015	-0.011	-0.01	t	78735	LRVKIERDDLSRETIIQII	Mus musculus

1	1.00	1	1	-0.01	-0.01	-0.01	-0.01	-0.01	t	133543	DKAFAV	Mus musculus
1	1.00	1	1	-0.04	-0.042	-0.049	-0.037	-0.037	t	96170	DEDSYERDP	Mus musculus
1	1.00	1	1	0	0.002	0.005	-0.003	-0.002	t	95356	ENGDVTFTEVGY	Homo sapiens
1	1.00	1	1	-0.04	-0.038	-0.038	-0.037	-0.04	t	134418	KGAIIGLMVGGVV	Homo sapiens
1	1.00	1	1	0.05	0.05	0.05	0.05	0.05	t	92811	GGNGDDFIDG	Ovis aries
1	1.00	1	1	0.03	0.03	0.03	0.03	0.03	t	111791	SNIQVLLDVPRSDVQ	Oryctolagus cuniculus
1	1.00	1	1	0.04	0.042	0.04	0.043	0.04	t	151609	DETPMRGETRKV	Homo sapiens
1	1.00	1	1	-0.04	-0.04	-0.043	-0.04	-0.04	t	50741	QFAPSASAFFGMSRIGM	Homo sapiens
1	1.00	1	1	0	0.002	0	0.001	0	t	128066	NSYDVLSALSGNLKL	Homo sapiens
1	1.00	1	1	0	0	0	0	0	t	48597	PNANPN	Mus musculus
1	1.00	1	1	-0.01	-0.01	-0.01	-0.01	-0.009	t	65409	TNRNPQ	Mus musculus
1	1.00	1	1	-0.02	-0.02	-0.029	-0.022	-0.02	t	135913	EEAIERAVVHVA	Homo sapiens
1	1.00	1	1	0.03	0.032	0.023	0.027	0.031	t	13908	EQQKEQCVKECEKYY	Homo sapiens
1	1.00	1	1	0	0	0	0	0	t	38681	LPRRGPRL	Homo sapiens
1	1.00	1	1	0	0	0	0	0	t	25624	IDLTNKNAAKL	Homo sapiens

1	1.00	1	1	0	-0.002	-0.003	0.003	0.002	t	18954	GDDPLLG NQ	Anas platyrhy nchos
1	1.00	1	1	0	-0.002	0	-0.001	0	t	45695	NRLGGLFN FGPKQKI	Mus musculu s
1	1.00	1	1	0.03	0.034	0.035	0.027	0.03	t	70542	VQDLMKS SGV	Homo sapiens
1	1.00	1	1	0	0	0	0	0	t	60116	SPLLGCIGS TCAE	Mus musculu s
1	1.00	1	1	-0.02	-0.018	-0.015	-0.019	-0.02	t	67449	TYRFVSL	Homo sapiens
1	1.00	1	1	0.06	0.06	0.06	0.06	0.06	t	78872	VIPDREVL YQEFDEM EECAS	Homo sapiens
1	1.00	1	1	0.06	0.06	0.06	0.06	0.06	t	78872	VIPDREVL YQEFDEM EECAS	Homo sapiens
1	1.00	1	1	0.02	0.02	0.02	0.02	0.02	t	153274	NLSTSNPL GFFPDHQ	Mus musculu s
1	1.00	1	1	0.02	0.02	0.02	0.02	0.02	t	115796	IQAFLLYQ EP	Homo sapiens
1	1.00	1	1	0	0	0	0	0	t	130637	WSSCVFSV SYCL	Mus musculu s
1	1.00	1	1	0	0	0	0	0	t	108416	HAKKTPPA S	Homo sapiens
1	1.00	1	1	-0.02	-0.02	-0.02	-0.02	-0.02	t	114902	NDCLLCAY SI	Homo sapiens
1	1.00	1	1	-0.02	-0.02	-0.02	-0.02	-0.02	t	115865	MPFPKYP VEP	Homo sapiens
1	1.00	1	1	0.01	0.011	0.009	0.011	0.01	t	39198	LRRTQSM GIQLDQ	Homo sapiens
1	1.00	1	1	0.01	0.012	0.01	0.013	0.01	t	119925	HFPFDEQ NGSMKLG TWT	Homo sapiens
1	1.00	1	1	0.01	0.012	0.01	0.013	0.01	t	119925	HFPFDEQ NGSMKLG TWT	Homo sapiens

1	1.00	1	1	0.01	0.014	0.01	0.013	0.01	t	119925	HFPFDEQNGSMKLGTWT	Homo sapiens
1	1.00	1	1	0.02	0.018	0.018	0.023	0.02	t	158944	KVQVQGPWDEEDIKLD	Mus musculus
1	1.00	1	1	0.01	0.008	0.007	0.009	0.01	t	43839	NFGDQDLIRQGTDYK	Macaca cyclopis
1	1.00	1	1	0	-0.003	-0.007	0	0	t	79329	PDHVRTK	Oryctolagus cuniculus
1	1.00	1	1	0	0	0.006	0	0	t	54838	RLYIGG	Homo sapiens
1	1.00	1	1	0	0	0	0	-0.001	t	108095	RFTPEIAK	Mus musculus
1	1.00	1	1	0.03	0.031	0.028	0.03	0.029	t	28291	IRFKDDTSFEDING	Myodes glareolus
1	1.00	1	1	-0.01	-0.01	-0.01	-0.01	-0.01	t	98698	APKPAPQPGP	Mus musculus
1	1.00	1	1	-0.04	-0.04	-0.043	-0.04	-0.04	t	98885	LPMHIRLSFN	Homo sapiens
1	1.00	1	1	0	0.006	0	0	0	t	123227	GGGDAPPSH	Ovis aries
1	1.00	1	1	0.03	0.034	0.039	0.037	0.03	t	117214	KGEQGEPGA	Homo sapiens
1	1.00	1	1	0.01	0.01	0	0.01	0.011	t	119575	EWESTYSILG	Homo sapiens
1	1.00	1	1	-0.05	-0.048	-0.06	-0.049	-0.051	t	97117	YIQMKRIAVG	Homo sapiens(human)
1	1.00	1	1	0	0.003	-0.007	0	0	t	72889	WNK	Mus musculus
1	1.00	1	1	0.01	0.01	0.01	0.01	0.01	t	65101	TLTLSHF	Homo sapiens

1	1.00	1	1	0.01	0.013	0.01	0.01	0.01	t	79285	LDPEYWNFRNGDLT	Mus musculus
1	1.00	1	1	0.02	0.023	0.02	0.02	0.018	t	135465	GSDYEDRYYREN	Mus musculus
1	1.00	1	1	-0.01	-0.008	-0.01	-0.009	-0.01	t	116978	FPSHYWLYPPPT	Homo sapiens
1	1.00	1	1	0.01	0.01	0.011	0.016	0.008	t	8379	DGEPPEDDDSATGLAFRTPN	Homo sapiens
1	1.00	1	1	0.03	0.03	0.03	0.03	0.03	t	158290	SQQAPAVPPLPQ	Canis lupus
1	1.00	1	1	-0.04	-0.038	-0.041	-0.039	-0.04	t	120410	ARLVVLASGTGSLLR	Homo sapiens
1	1.00	1	1	0.01	0.01	-0.002	0.01	0.014	t	133537	CLTINTTVCA	Mus musculus
1	1.00	1	1	0.03	0.033	0.035	0.03	0.03	t	61409	SSTQVQHTLLQT	Mus musculus
1	1.00	1	1	0.03	0.03	0.03	0.03	0.03	t	138088	WLDLSKT	Mus musculus
1	1.00	1	1	0.03	0.03	0.03	0.03	0.03	t	138088	WLDLSKT	Mus musculus
1	1.00	1	1	0.03	0.03	0.03	0.03	0.03	t	138088	WLDLSKT	Mus musculus
1	1.00	1	1	0.03	0.03	0.03	0.03	0.03	t	138088	WLDLSKT	Mus musculus
1	1.00	1	1	0.01	0.01	0.01	0.01	0.01	t	107511	LPAPIEK	Homo sapiens
1	1.00	1	1	0.01	0.01	0.01	0.01	0.01	t	110616	GPSGLAGPKGANGDP	Mus musculus
1	1.00	1	1	0.06	0.058	0.058	0.059	0.06	t	55924	RSQDACIEFVQQEVD	Mus musculus
1	1.00	1	1	-0.01	-0.01	-0.005	-0.007	-0.008	t	41541	MFTCKK	Homo sapiens

1	1.00	1	1	-0.02	-0.021	-0.02	-0.017	-0.022	t	156860	DRWNSMWKESLL	Bos taurus
1	1.00	1	1	0.03	0.028	0.025	0.029	0.03	t	22252	GRWDEDGEKRIPLDVA	Mus musculus
1	1.00	1	1	0	-0.002	0	-0.001	-0.002	t	97231	CNTKCQTP	Mus musculus
1	1.00	1	1	0	-0.002	0	-0.001	-0.002	t	39164	LRPGPVSQPYHHWDTDK	Mus musculus
1	1.00	1	1	0	0	0	0	0	t	106441	GDLKRTT	Mus musculus
1	1.00	1	1	0	0	0	0	0	t	130805	ELNPEPDTEVFPMTF	Homo sapiens
1	1.00	1	1	-0.04	-0.042	-0.051	-0.041	-0.041	t	37698	LLQAHRQLEER	Mus musculus
1	1.00	1	1	0.03	0.032	0.021	0.024	0.029	t	151045	PVHDCNTTCQTPKGA	Homo sapiens
1	1.00	1	1	0.06	0.057	0.062	0.06	0.06	t	132709	TTTTGESADPVT	Oryctolagus cuniculus
1	1.00	1	1	-0.04	-0.04	-0.036	-0.04	-0.042	t	138757	AHWSGHCCL	Mus musculus
1	1.00	1	1	0.02	0.018	0.015	0.019	0.018	t	73784	YEVRAELPGVDPDKDVDIMV	Mus musculus
1	1.00	1	1	0.06	0.058	0.057	0.063	0.06	t	94572	KQWEDEVTEFNYKVGLQNSN	Mus musculus
1	1.00	1	1	0.01	0.01	0.01	0.01	0.011	t	99209	ELFTNR	Oryctolagus cuniculus
1	1.00	1	1	-0.07	-0.066	-0.074	-0.069	-0.07	t	73020	WRCPPRRR	Homo sapiens

1	1.00	1	1	-0.01	-0.012	-0.011	-0.011	-0.01	t	60433	SQERNFVDSIGQSFQ	Mus musculus
1	1.00	1	1	0	-0.002	0	0.003	0	t	95818	SAQSGTSGTSAQ	Mus musculus
1	1.00	1	1	0.01	0.004	0.01	0.01	0.01	t	123210	GAVQGPVP	Oryctolagus cuniculus
1	1.00	1	1	0	0	0.012	0	-0.004	t	20089	GGWNTGGSR	Mus musculus
1	1.00	1	1	-0.03	-0.03	-0.031	-0.027	-0.03	t	65521	TPCSK	Homo sapiens
1	1.00	1	1	0.02	0.02	0.014	0.014	0.02	t	23078	GVNQELAYFY	Homo sapiens
1	1.00	1	1	0	0.002	0	0.007	0.003	t	11571	EEEDDDMGFGLFD	Homo sapiens
1	1.00	1	1	0.03	0.035	0.039	0.031	0.03	t	65861	TQGSNWIQ	Homo sapiens
1	1.00	1	1	0.02	0.02	0.02	0.02	0.02	t	94451	CCNPACG	Oryctolagus cuniculus
1	1.00	1	1	0.02	0.02	0.02	0.02	0.02	t	94451	CCNPACG	Capra hircus
1	1.00	1	1	0.02	0.027	0.02	0.02	0.02	t	94451	CCNPACG	Capra hircus
1	1.00	1	1	-0.09	-0.086	-0.088	-0.083	-0.092	t	121503	KRLKISKKPKDPEPP	Homo sapiens
1	1.00	1	1	-0.01	-0.01	-0.01	-0.01	-0.012	t	49106	PQQQFPQPQQ	Mus musculus

1	1.00	1	1	0.04	0.04	0.04	0.04	0.04	t	137555	QEWQQQDECQIDRLD	Homo sapiens
1	1.00	1	1	0.02	0.02	0.02	0.02	0.02	t	134687	GWLKEIRKLQKSTHL	Homo sapiens
1	1.00	1	1	0.02	0.02	0.02	0.02	0.02	t	79384	SCNSFMIPAQPSCT	Mus musculus
1	1.00	1	1	0	0	0	0	0	t	79357	RCDLRYDAFVPWCP	Mus musculus
1	1.00	1	1	-0.02	-0.02	-0.024	-0.02	-0.02	t	47544	PFPQPQQPFC	Mus musculus
1	1.00	1	1	-0.02	-0.02	-0.024	-0.02	-0.02	t	47544	PFPQPQQPFC	Mus musculus
1	1.00	1	1	0	0.003	-0.01	0	0	t	139404	YIDINKTIADMLEQYNPNYT	Felis catus
1	1.00	1	1	-0.02	-0.02	-0.025	-0.022	-0.021	t	70010	VMPVGGQSSFYSDWY	Homo sapiens
1	1.00	1	1	0.03	0.03	0.03	0.03	0.03	t	111907	VEVGRGQPLGVGISG	Mus musculus
1	1.00	1	1	0.02	0.023	0.02	0.02	0.018	t	140854	GFDKYDITGEYVQILDAHAA	Mus musculus
1	1.00	1	1	0	0	0.005	0.01	0.003	t	115785	HSYQE	Homo sapiens
1	1.00	1	1	0.01	0.012	0.01	0.009	0.008	t	51908	QPQPFPSQQP	Homo sapiens
1	1.00	1	1	0.05	0.055	0.043	0.051	0.05	t	49890	PVIVPDREVLYREFDEMEEC	Homo sapiens
1	1.00	1	1	0.05	0.055	0.043	0.051	0.05	t	49890	PVIVPDREVLYREFDEMEEC	Homo sapiens
1	1.00	1	1	-0.03	-0.03	-0.03	-0.034	-0.029	t	134233	SKLIKIF	Homo sapiens

1	1.00	1	1	0.01	0.007	0.011	0.01	0.01	t	50864	QGGGGWGQPHGGG	Oryctolagus cuniculus
1	1.00	1	1	0.01	0.01	-0.001	0.01	0.012	t	144701	KFGNYNHTQLLGQLQAV	Oryctolagus cuniculus
1	1.00	1	1	0.02	0.022	0.011	0.017	0.018	t	105700	CPSTYEEPAQQGRRH	Homo sapiens
1	1.00	1	1	0.01	0.007	0.01	0.01	0.01	t	45948	NSQLRLNYEKSL	Mus musculus
1	1.00	1	1	-0.01	-0.01	-0.01	-0.01	-0.01	t	120609	IFAD	Rattus norvegicus
1	1.00	1	1	-0.03	-0.032	-0.031	-0.027	-0.028	t	55460	RQPDLIP	Mus musculus
1	1.00	1	1	0.02	0.02	0.018	0.017	0.018	t	14639	EVDDEALEKFDKAL	Homo sapiens
1	1.00	1	1	0.05	0.052	0.05	0.053	0.05	t	134015	CDKNTGDYYEDSYED	Homo sapiens
1	1.00	1	1	0.01	0.008	0.015	0.009	0.009	t	49967	PVSLGGDVPNS	Mus musculus
1	1.00	1	1	-0.02	-0.02	-0.025	-0.023	-0.021	t	49454	PSPAPSRPFSVLRANDV	Homo sapiens
1	1.00	1	1	-0.01	-0.007	-0.019	-0.01	-0.008	t	63757	TFTNKLKSKHTDLGK	Mus musculus
1	1.00	1	1	-0.02	-0.02	-0.027	-0.02	-0.022	t	95543	LCPNNLCCSQ	Homo sapiens
1	1.00	1	1	0.04	0.04	0.045	0.043	0.042	t	23349	GYGTVT	Homo sapiens
1	1.00	1	1	-0.01	-0.012	-0.002	-0.011	-0.012	t	73529	YDDVNGIRKP	Mus musculus
1	1.00	1	1	0.05	0.05	0.05	0.05	0.05	t	97476	LSTTDSFS	Mus musculus

1	1.00	1	1	-0.01	-0.01	-0.014	-0.01	-0.01	t	49097	PQQPQQPFPQ	Mus musculus
1	1.00	1	1	-0.01	-0.01	-0.014	-0.01	-0.01	t	49097	PQQPQQPFPQ	Mus musculus
1	1.00	1	1	0.01	0.012	0.01	0.017	0.01	t	139614	SSHHPGIAEFPSRGK	Homo sapiens
1	1.00	1	1	0.02	0.022	0.02	0.021	0.02	t	133553	EDMSYWHEWNP	Homo sapiens
1	1.00	1	1	-0.05	-0.05	-0.05	-0.05	-0.05	t	158610	SFGTFLW	Homo sapiens
1	1.00	1	1	-0.04	-0.04	-0.04	-0.04	-0.04	t	117546	KIRRSPSKPLPEVT	Homo sapiens
1	1.00	1	1	0.01	0.007	0.008	0.01	0.01	t	45693	NRKNQLKDMA	Oryctolagus cuniculus
1	1.00	1	1	0.02	0.02	0.02	0.02	0.02	t	132940	WGSNVTS	Homo sapiens
1	1.00	1	1	0	0	0	0	0	t	475	AATVLQLPQGTTLPK	Homo sapiens
1	1.00	1	1	-0.02	-0.02	-0.02	-0.02	-0.02	t	131853	DVDEPLEVFISAPRS	Homo sapiens
1	1.00	1	1	0	0	0	0.001	-0.001	t	3245	ANDVDNSNPVVQAEQLNWL	Homo sapiens
1	1.00	1	1	-0.01	-0.007	-0.019	-0.01	-0.01	t	100283	LPNIAEQYTH	Mus musculus
1	1.00	1	1	0	0.002	-0.003	0.001	0	t	21812	GPTRTIGGSQ	Homo sapiens
1	1.00	1	1	0.02	0.02	0.01	0.02	0.02	t	33488	KSQQARQVKNNNPFS	Homo sapiens
1	1.00	1	1	0.04	0.04	0.047	0.04	0.04	t	68171	VEESIY	Homo sapiens
1	1.00	1	1	-0.01	-0.012	-0.01	-0.017	-0.008	t	107784	DGSFFLY	Mus musculus

1	1.00	1	1	0	-0.003	0	0	-0.001	t	69183	VKAVTTLLKA	Oryctolagus cuniculus
1	1.00	1	1	0	-0.003	0	0	-0.001	t	26849	IKMVSSLKTKIT	Oryctolagus cuniculus
1	1.00	1	1	0.01	0.006	0.005	0.01	0.011	t	73784	YEVRAELPGVDPDKDVDIMV	Mus musculus
1	1.00	1	1	0.04	0.042	0.054	0.041	0.04	t	11877	EESIYQ	Homo sapiens
1	1.00	1	1	-0.02	-0.018	-0.02	-0.013	-0.021	t	139631	VIQNRQDGSVDFGRK	Homo sapiens
1	1.00	1	1	-0.04	-0.038	-0.034	-0.039	-0.042	t	1165	AESPKKP	Homo sapiens
1	1.00	1	1	0.02	0.02	0.02	0.024	0.02	t	115657	TLRIRNVRFSDE	Homo sapiens
1	1.00	1	1	0.01	0.014	0.007	0.01	0.008	t	110762	AGSSIVPLEEYPAEIP	Oryctolagus cuniculus
1	1.00	1	1	-0.02	-0.02	-0.023	-0.02	-0.02	t	6233	CFKKPPKYPPALPI	Homo sapiens
1	1.00	1	1	0	-0.014	0	0.001	-0.001	t	7491	DAEFRHDSGYEVHHQK	Macaca mulatta
1	1.00	1	1	0.06	0.06	0.06	0.06	0.06	t	115464	QYTDAPSFSDIPNPIGSENS	Homo sapiens
1	1.00	1	1	0.02	0.02	0.02	0.02	0.02	t	108518	LGLHALQSP	Homo sapiens
1	1.00	1	1	-0.02	-0.016	-0.023	-0.019	-0.019	t	55436	RQKLKDAERAVEVD	Homo sapiens
1	1.00	1	1	-0.02	-0.02	-0.02	-0.02	-0.02	t	135755	SALVLQYL	Homo sapiens
1	1.00	1	1	-0.01	-0.006	-0.01	-0.009	-0.01	t	137927	LSADFTPG	Homo sapiens

1	1.00	1	1	0.02	0.027	0.027	0.021	0.02	t	136302	GALIYTPEKYTI	Gallus gallus
1	1.00	1	1	0.02	0.022	0.017	0.021	0.02	t	13421	EMVDVVTG	Homo sapiens
1	1.00	1	1	0.02	0.022	0.017	0.021	0.02	t	13421	EMVDVVTG	Homo sapiens
1	1.00	1	1	0.02	0.02	0.013	0.02	0.02	t	23594	HCHTHADGEVHC	Mus musculus
1	1.00	1	1	0.01	0.014	0.001	0.007	0.008	t	113709	PQQPGQGQQPGQLQ	Homo sapiens
1	1.00	1	1	-0.01	-0.01	-0.01	-0.01	-0.01	t	132938	WGSNPAM	Homo sapiens
1	1.00	1	1	-0.01	-0.01	-0.01	-0.01	-0.01	t	70269	VPDKEVLYQ	Homo sapiens
1	1.00	1	1	0.01	0.012	0.01	0.017	0.013	t	140582	PWATLVAES	Homo sapiens
1	1.00	1	1	-0.01	-0.008	-0.01	-0.003	-0.01	t	108136	SLNTADSKTTTFPAR	Homo sapiens
1	1.00	1	1	0.01	0.014	0.005	0.011	0.01	t	82831	GPTHSKEIPALTAVE	Homo sapiens
1	1.00	1	1	0.02	0.018	0.02	0.012	0.018	t	144385	KPLEEVLNL	Mus musculus
1	1.00	1	1	-0.03	-0.03	-0.035	-0.03	-0.03	t	57509	SEKTTMPLW	Homo sapiens
1	1.00	1	1	-0.03	-0.034	-0.035	-0.031	-0.03	t	97732	VGSAAPAFA	Oryctolagus cuniculus
1	1.00	1	1	0.06	0.06	0.053	0.051	0.06	t	119457	GLELPE	Homo sapiens
1	1.00	1	1	0	0.001	-0.002	0.003	0	t	72517	WGSFPGCRPFQNYFSYET	Equus caballus
1	1.00	1	1	0.02	0.017	0.02	0.02	0.017	t	144795	TAAGYCNTGGGLPDSVKI	Oryctolagus cuniculus
1	1.00	1	1	0.02	0.02	0.02	0.02	0.021	t	144676	GTHPSLQFT	Mus musculus

1	1.00	1	1	0.03	0.03	0.03	0.03	0.03	t	155334	PADSS	Oryctolagus cuniculus
1	1.00	1	1	-0.03	-0.032	-0.03	-0.029	-0.03	t	149414	PQLPYPQPQP	Mus musculus
1	1.00	1	1	-0.03	-0.034	-0.033	-0.031	-0.03	t	68970	VIGHEYFHNWT	Oryctolagus cuniculus
1	1.00	1	1	0	0	0	0	0	t	65411	TNRRPQD	Homo sapiens
1	1.00	1	1	-0.02	-0.024	-0.022	-0.021	-0.022	t	57733	SFCIMPPSISEG	Oryctolagus cuniculus
1	1.00	1	1	-0.02	-0.023	-0.027	-0.02	-0.018	t	138028	SAGALQLLVVGC	Oryctolagus cuniculus
1	1.00	1	1	0.05	0.048	0.05	0.053	0.053	t	106509	HGLTTE	Mus musculus
1	1.00	1	1	0.02	0.02	0.018	0.017	0.018	t	24099	HITDDNEEPIAPYHF	Homo sapiens
1	1.00	1	1	-0.01	-0.012	-0.015	-0.011	-0.01	t	78647	ETIIQIIEYGKKFNSSAGD	Mus musculus
1	1.00	1	1	-0.03	-0.033	-0.036	-0.03	-0.03	t	44598	NKSLGACPIR	Oryctolagus cuniculus
1	1.00	1	1	-0.01	-0.008	-0.01	-0.003	-0.008	t	124703	SDDNYDKTEDVDIEEMALKL	Homo sapiens
1	1.00	1	1	-0.03	-0.03	-0.03	-0.03	-0.029	t	115317	KKILDKVGIN	Homo sapiens
1	1.00	1	1	0.03	0.03	0.03	0.03	0.03	t	119535	TQDLGYSRV	Homo sapiens
1	1.00	1	1	0.03	0.03	0.03	0.03	0.03	t	130760	CSFHYQNRC	Homo sapiens

1	1.00	1	1	0.01	0.012	0.01	0.01	0.01	t	141697	LEGVYVPP RRQQLCLY	Rattus norvegicus
1	1.00	1	1	0.01	0.008	0.01	0.01	0.01	t	141697	LEGVYVPP RRQQLCLY	Oryctolagus cuniculus
1	1.00	1	1	0.01	0.01	0.01	0.01	0.01	t	141697	LEGVYVPP RRQQLCLY	Oryctolagus cuniculus
1	1.00	1	1	0.01	0.01	0.01	0.01	0.01	t	141697	LEGVYVPP RRQQLCLY	Rattus norvegicus
1	1.00	1	1	0.01	0.01	0.01	0.01	0.01	t	123086	DKFNMGL DL	Ovis aries
1	1.00	1	1	0.01	0.01	0.01	0.01	0.01	t	119510	QKKMVAL SG	Homo sapiens
1	1.00	1	1	0.01	0.01	0.01	0.01	0.01	t	131275	WHDDNPF FFEAP	Homo sapiens
1	1.00	1	1	0.01	0.01	0.01	0.01	0.01	t	97691	TKQPVLDF S	Oryctolagus cuniculus
1	1.00	1	1	-0.01	-0.01	-0.01	-0.01	-0.01	t	135661	EDAKPGQ F	Homo sapiens
1	1.00	1	1	0.04	0.043	0.04	0.04	0.042	t	59946	SNVQQDS Q	Mus musculus
1	1.00	1	1	-0.01	-0.01	-0.011	-0.01	-0.01	t	60249	SPSAVPTD HGAHLSL	Homo sapiens
1	1.00	1	1	0	0.003	-0.009	0	0.002	t	79964	PGSSPME VMLRAVF	Mus musculus
1	1.00	1	1	0.01	0.01	0.01	0.01	0.009	t	55477	RQQWELQ GDRRCQS Q	Homo sapiens
1	1.00	1	1	-0.03	-0.024	-0.039	-0.031	-0.03	t	11802	EENKKPAE	Ovis aries

1	1.00	1	1	0	0.002	-0.01	0.001	0	t	14380	ETFTNKLKEKHTD	Homo sapiens
1	1.00	1	1	-0.04	-0.04	-0.04	-0.04	-0.04	t	101716	HQPTPAA	Mus musculus
1	1.00	1	1	-0.04	-0.04	-0.04	-0.04	-0.04	t	101716	HQPTPAA	Mus musculus
1	1.00	1	1	0.03	0.03	0.025	0.03	0.03	t	47398	PERIKLDYDQYCADV	Homo sapiens
1	1.00	1	1	0	0.004	0	0.001	0	t	800	ADYHRVMY	Homo sapiens
1	1.00	1	1	-0.01	-0.012	-0.016	-0.007	-0.008	t	98653	YYTKNTNNNL	Mus musculus
1	1.00	1	1	-0.01	-0.014	-0.016	-0.007	-0.008	t	98653	YYTKNTNNNL	Oryctolagus cuniculus
1	1.00	1	1	-0.01	-0.012	-0.007	-0.011	-0.012	t	46488	NVSVPSPSSTPLLY	Mus musculus
1	1.00	1	1	0.03	0.027	0.035	0.03	0.03	t	107086	ANYGYTFGSG	Oryctolagus cuniculus
1	1.00	1	1	0.01	0.008	0.01	0.007	0.009	t	74488	YKPLGALTH	Mus musculus
1	1.00	1	1	-0.04	-0.04	-0.045	-0.04	-0.04	t	78117	DIQKVAGTWYSLAMAA	Mus musculus
1	1.00	1	1	0.03	0.034	0.027	0.037	0.03	t	105976	NGVTLGYTSGTCQGNASATK	Homo sapiens
1	1.00	1	1	0.01	0.008	0.009	0.009	0.011	t	21763	GPRLQPY	Mus musculus
1	1.00	1	1	-0.06	-0.063	-0.065	-0.06	-0.06	t	132846	KAKRLHLSPVRLAE	Oryctolagus cuniculus

1	1.00	1	1	0	0	0	-0.006	-0.004	t	24786	HSLGKWL GHPDKF	Mus musculu s
1	1.00	1	1	0.02	0.017	0.02	0.02	0.022	t	140843	EKPAVLGH DEAA	Oryctola gus cuniculu s
1	1.00	1	1	-0.03	-0.028	-0.031	-0.029	-0.028	t	25061	HVLGRL	Homo sapiens
1	1.00	1	1	0.04	0.04	0.04	0.04	0.04	t	103359	LILTSYTSD EAMLDA	Homo sapiens
1	1.00	1	1	0.02	0.02	0.02	0.02	0.02	t	153349	ACTDFEG MLCGGGS	Mus musculu s
1	1.00	1	1	0	0	0	0	0	t	135440	AFPAVT	Oryctola gus cuniculu s
1	1.00	1	1	-0.02	-0.02	-0.02	-0.02	-0.02	t	104149	NHYFLRS	Homo sapiens
1	1.00	1	1	0	0	0	0	0	t	132098	TLLLEGVM AA	Homo sapiens
1	1.00	1	1	0	-0.004	-0.012	0.003	0	t	106779	RKPLSPG MTGR	Oryctola gus cuniculu s
1	1.00	1	1	0.03	0.032	0.027	0.027	0.028	t	95766	RNANEEEY SI	Homo sapiens
1	1.00	1	1	0.03	0.032	0.027	0.027	0.028	t	95766	RNANEEEY SI	Homo sapiens
1	1.00	1	1	0.04	0.04	0.039	0.04	0.037	t	124367	KLKARVDE LERIRRS	Oryctola gus cuniculu s
1	1.00	1	1	0	0	0.011	0	-0.002	t	47006	PAYEKL	Mus musculu s
1	1.00	1	1	-0.04	-0.043	-0.042	-0.04	-0.041	t	30116	KDATGIDN HREAKL	Oryctola gus cuniculu s
1	1.00	1	1	-0.03	-0.027	-0.03	-0.03	-0.03	t	131346	AIPDRFS	Mus musculu s

1	1.00	1	1	-0.01	-0.008	-0.003	-0.003	-0.009	t	108052	NWLSLITAAR	Homo sapiens
1	1.00	1	1	0.03	0.03	0.03	0.03	0.03	t	129024	KADQLYK	Homo sapiens
1	1.00	1	1	0.01	0.01	0.01	0.01	0.01	t	140559	FPYELLMQG	Mus musculus
1	1.00	1	1	0.02	0.02	0.015	0.02	0.019	t	107083	AKDVKFGADARALML	Rattus norvegicus
1	1.00	1	1	0.03	0.026	0.02	0.033	0.031	t	63736	TFQVEVPGSQHIDS	Oryctolagus cuniculus
1	1.00	1	1	-0.02	-0.02	-0.02	-0.02	-0.022	t	137747	DTPLTYAGLRLV	Mus musculus
1	1.00	1	1	0.04	0.04	0.044	0.04	0.04	t	25262	IAETQHGTI	Mus musculus
1	1.00	1	1	0.01	0.01	0.01	0.01	0.01	t	115233	EPMIGVNQELAYFYPELFRQ	Homo sapiens
1	1.00	1	1	-0.01	-0.01	-0.01	-0.01	-0.01	t	138203	MGEFFMPYPGTRIN	Oryctolagus cuniculus
1	1.00	1	1	0.01	0.007	0.005	0.01	0.01	t	138215	PPVANDTRIVMGRL	Oryctolagus cuniculus
1	1.00	1	1	0	0.002	0	0.007	0.003	t	110995	RARPAKAAATQKKVERKA	Homo sapiens
1	1.00	1	1	0.03	0.03	0.03	0.03	0.03	t	116930	AYKLPQTDDST	Homo sapiens
1	1.00	1	1	0.03	0.03	0.03	0.03	0.03	t	116930	AYKLPQTDDST	Mus musculus
1	1.00	1	1	0.02	0.02	0.014	0.02	0.02	t	22685	GTHNGQIGNDPNRDI	Mus musculus
1	1.00	1	1	0.04	0.04	0.04	0.04	0.042	t	45639	NQVYYRPMDEYSN	Mus musculus
1	1.00	1	1	0.02	0.02	0.011	0.02	0.02	t	15991	FGHQVGDG	Homo sapiens

1	1.00	1	1	0.02	0.024	0.02	0.021	0.02	t	73611	YDYQHKEG	Homo sapiens
1	1.00	1	1	0.02	0.024	0.02	0.021	0.02	t	4770	ASSGIGPE	Homo sapiens
1	1.00	1	1	0	0.004	0	0.001	0	t	38583	LPLPWL	Homo sapiens
1	1.00	1	1	0.01	0.011	0.01	0.007	0.008	t	8803	DITPQEFSKKYLQAP	Homo sapiens
1	1.00	1	1	0.02	0.023	0.011	0.02	0.021	t	75691	YRYNIYSEKEKSNINIDFN	Equus caballus
1	1.00	1	1	0	0.003	0	0	0	t	32913	KPTTKQRQNKPP	Mus musculus
1	1.00	1	1	0.03	0.032	0.036	0.033	0.029	t	13641	EPEQVELQPPPQGG	Homo sapiens
1	1.00	1	1	0.04	0.042	0.04	0.042	0.038	t	48851	PPPEHRGGPEEFEGAGDGEP	Homo sapiens
1	1.00	1	1	0	0	0	0.002	0	t	22121	GRLGIYRYVSTGER	Homo sapiens
1	1.00	1	1	0.01	0.01	0.004	0.012	0.01	t	53267	RCEPVVCYLYGKRGGGKSLT	Homo sapiens
1	1.00	1	1	0	0.003	0.003	0	0	t	97181	AFPLPTDA	Mus musculus
1	1.00	1	1	0	0.003	0.003	0	0	t	97181	AFPLPTDA	Mus musculus
1	1.00	1	1	0	0.002	-0.007	-0.003	0.002	t	49621	PTFKTTKKDHKP	Homo sapiens

1	1.00	1	1	0.03	0.026	0.029	0.029	0.03	t	110862	GGPTLVSL HELPAETP	Oryctolagus cuniculus
1	1.00	1	1	-0.01	-0.007	-0.008	-0.01	-0.011	t	7165	CTKKKSS	Mus musculus
1	1.00	1	1	-0.02	-0.017	-0.02	-0.02	-0.021	t	113345	DTPTLH	Mus musculus
1	1.00	1	1	0	0.002	-0.006	0.001	0	t	25054	HVIWDVG LQSS	Homo sapiens
1	1.00	1	1	0.02	0.02	0.02	0.02	0.02	t	12931	ELADALLE	Oryctolagus cuniculus
1	1.00	1	1	-0.05	-0.05	-0.05	-0.05	-0.051	t	114223	PFSRVVHL	Rattus norvegicus
1	1.00	1	1	0.02	0.02	0.02	0.02	0.02	t	43159	NAGVLTCS LSK	Mus musculus
1	1.00	1	1	0	0.002	0.005	0.007	0	t	127981	FRRAVLM GGSALSPA AVISH	Homo sapiens
1	1.00	1	1	-0.02	-0.016	-0.015	-0.013	-0.021	t	116434	PGFGYGG RASDYKSA HKG	Homo sapiens
1	1.00	1	1	-0.06	-0.056	-0.06	-0.053	-0.061	t	134792	RSPSPTPT PGPSRRG	Homo sapiens
1	1.00	1	1	-0.05	-0.052	-0.042	-0.049	-0.053	t	149384	MSLPGRW KPK	Mus musculus
1	1.00	1	1	0.02	0.02	0.02	0.02	0.02	t	123299	HVDFMLY ET	Ovis aries
1	1.00	1	1	0.02	0.02	0.02	0.02	0.02	t	131867	EPSPESIRR KMEIDD	Homo sapiens
1	1.00	1	1	0.02	0.02	0.02	0.02	0.02	t	123300	HVDFMVY EA	Ovis aries

1	1.00	1	1	0	0	0	0	0	t	128101	SFSRALENATRDYFIICPII	Homo sapiens
1	1.00	1	1	0	0	0	0	0	t	102245	YNMPALL	Homo sapiens
1	1.00	1	1	0	0	0	0	0	t	79586	KIPIALS	Oryctolagus cuniculus
1	1.00	1	1	-0.04	-0.04	-0.048	-0.04	-0.04	t	125454	KDYKTMVKFCN	Mus musculus
1	1.00	1	1	-0.05	-0.05	-0.05	-0.04	-0.048	t	108608	RGGIRRQSL	Homo sapiens
1	1.00	1	1	-0.04	-0.038	-0.04	-0.033	-0.039	t	138218	PTPAPGPGRR	Homo sapiens
1	1.00	1	1	-0.01	-0.01	-0.01	-0.01	-0.01	t	98746	EDWRRPSHQQ	Homo sapiens
1	1.00	1	1	-0.01	-0.01	-0.012	-0.013	-0.012	t	23372	GYIWIQRNTG	Homo sapiens
1	1.00	1	1	-0.01	-0.006	-0.01	-0.009	-0.01	t	69595	VLLGACSF	Homo sapiens
1	1.00	1	1	-0.01	-0.008	-0.01	-0.013	-0.01	t	24812	HSMKEGIHAQ	Homo sapiens
1	1.00	1	1	0.04	0.038	0.035	0.037	0.04	t	133634	LSQDVCTY	Mus musculus
1	1.00	1	1	-0.03	-0.03	-0.034	-0.03	-0.03	t	120014	LAAQGARVYA	Mus musculus
1	1.00	1	1	0	0	0	-0.003	-0.003	t	14435	ETIYNTTLKY	Homo sapiens
1	1.00	1	1	0	0	-0.015	0	0.001	t	48618	PNDPNRNVD	Mus musculus
1	1.00	1	1	0	-0.002	0	0.003	0	t	21254	GMDGTQLDRW	Mus musculus
1	1.00	1	1	0	-0.002	0	0.003	0	t	21254	GMDGTQLDRW	Mus musculus

1	1.00	1	1	0.01	0.014	0.01	0.011	0.01	t	137883	GQNVLSADFTPG	Homo sapiens
1	1.00	1	1	-0.04	-0.04	-0.04	-0.04	-0.041	t	112328	SPKLLIY	Homo sapiens
1	1.00	1	1	-0.03	-0.032	-0.033	-0.031	-0.03	t	31779	KLCVWNNKTP	Mus musculus
1	1.00	1	1	-0.02	-0.027	-0.027	-0.02	-0.019	t	159284	FSLRLMEENWNAEKRS	Oryctolagus cuniculus
1	1.00	1	1	0	0	-0.007	0	0.001	t	122375	GLINRAKAVKQFEESQG	Rattus norvegicus
1	1.00	1	1	0	0	-0.007	0	0.001	t	122375	GLINRAKAVKQFEESQG	Mus musculus
1	1.00	1	1	0	0	-0.007	0	0.001	t	122375	GLINRAKAVKQFEESQG	Mus musculus
1	1.00	1	1	0	0	0	-0.009	-0.002	t	130800	EGAKAFFKGA	Homo sapiens
1	1.00	1	1	-0.03	-0.026	-0.033	-0.029	-0.03	t	66163	TSARPAVMPMDAWRE	Homo sapiens
1	1.00	1	1	0.01	0.01	0.01	0.01	0.01	t	77872	FGTSPTGLKLAP	Mus musculus
1	1.00	1	1	-0.01	-0.01	-0.01	-0.01	-0.01	t	116944	DFRYSTRPFAL	Mus musculus
1	1.00	1	1	-0.01	-0.01	0	-0.01	-0.011	t	95161	TYGKFCIKP	Mus musculus
1	1.00	1	1	0.03	0.03	0.03	0.03	0.03	t	96104	APTKPTYETE	Homo sapiens(human)
1	1.00	1	1	-0.01	-0.01	-0.01	-0.01	-0.01	t	151031	LVKKGNSYPKLSKSY	Homo sapiens
1	1.00	1	1	-0.01	-0.01	-0.01	-0.01	-0.01	t	105889	IVGFFSKI	Oryctolagus cuniculus

1	1.00	1	1	-0.01	-0.01	-0.01	-0.01	-0.01	t	23313	GYCAWPA CYCYGLPN	Equus caballus
1	1.00	1	1	0.04	0.036	0.044	0.039	0.038	t	155760	SATQLYKT CKQAGT	Oryctola gus cuniculu s
1	1.00	1	1	0	-0.002	-0.004	-0.008	0	t	67297	TWSGKFP T	Anas platyrhy nchos
1	1.00	1	1	-0.04	-0.04	-0.027	-0.032	-0.043	t	98691	ALKALPM HIR	Homo sapiens
1	1.00	1	1	0.01	0.01	0.007	0.01	0.01	t	99005	SLAMAAS DIS	Homo sapiens
1	1.00	1	1	0.01	0.012	0.017	0.017	0.008	t	113212	VPVLSAKA AQASDLE	Homo sapiens
1	1.00	1	1	0	0.004	0	0.001	0	t	6540	CLAVLLGA	Homo sapiens
1	1.00	1	1	0	-0.002	0.006	0.001	0.001	t	78924	AGPEWLL	Mus musculu s
1	1.00	1	1	-0.01	-0.01	-0.016	-0.01	-0.01	t	159083	RYKYLPET PKQLAKVV	Mus musculu s
1	1.00	1	1	0	0	0.005	0.002	0	t	57279	SDSESCSS MPPLEG	Homo sapiens
1	1.00	1	1	0.01	0.008	0.008	0.013	0.011	t	99661	SERRAQSV	Mus musculu s
1	1.00	1	1	0.06	0.06	0.065	0.06	0.06	t	97596	QTPLTMA ALELL	Mus musculu s
1	1.00	1	1	0	-0.002	0	-0.003	-0.001	t	49191	PRGRRQPI	Mus musculu s
1	1.00	1	1	0.03	0.032	0.029	0.031	0.03	t	40018	LTLNLITE MG	Homo sapiens
1	1.00	1	1	0.03	0.032	0.024	0.031	0.03	t	75920	YSVLYNST FFSTFKCY G	Homo sapiens

1	1.00	1	1	0	0	-0.011	0	0.001	t	106842	SKDEIDSAVKMLVSLKMSY	Mus musculus
1	1.00	1	1	0	0.003	-0.011	0	0.002	t	144735	NSSNYCCELCCNPACTGCY	Mus musculus
1	1.00	1	1	0	0	0	0	0	t	9755	DPRVRGLYFPA	Mus musculus
1	1.00	1	1	-0.02	-0.022	-0.033	-0.017	-0.017	t	141646	FSKWRKNHMRQKSNK	Mus musculus
1	1.00	1	1	0	0.003	0.002	0.007	0.004	t	104895	LVEALYLVCGERG	Mus musculus
1	1.00	1	1	-0.02	-0.02	-0.032	-0.02	-0.018	t	109534	LVGPRGERGFP	Mus musculus
1	1.00	1	1	0	0	0	0	-0.001	t	149298	ELDKWAS	Homo sapiens
1	1.00	1	1	0.06	0.06	0.06	0.06	0.06	t	103013	AEGDTVIYSKYGGTE	Homo sapiens
1	1.00	1	1	0.04	0.04	0.04	0.04	0.04	t	113861	SISTTNFTNP	Oryctolagus cuniculus
1	1.00	1	1	0.02	0.02	0.02	0.02	0.02	t	137891	HETHYGYATLSY	Homo sapiens
1	1.00	1	1	0	0	0	0	0	t	149415	PQQLPQFEEI	Mus musculus
1	1.00	1	1	0	0	0	0	0	t	149415	PQQLPQFEEI	Homo sapiens
1	1.00	1	1	-0.02	-0.02	-0.02	-0.02	-0.02	t	127719	KWSFTPP	Homo sapiens

1	1.00	1	1	0.01	0.014	0.007	0.007	0.007	t	115486	SISSSEEIVPNSVEQKHIQK	Homo sapiens
1	1.00	1	1	-0.01	-0.011	-0.003	-0.01	-0.008	t	40236	LTWSGKFP	Anas platyrhynchos
1	1.00	1	1	-0.03	-0.027	-0.03	-0.03	-0.03	t	119988	KIFAQRLMRY	Mus musculus
1	1.00	1	1	-0.01	-0.007	-0.012	-0.01	-0.01	t	17642	FRRYQEER	Mus musculus
1	1.00	1	1	-0.01	-0.008	-0.012	-0.01	-0.01	t	17642	FRRYQEER	Anas platyrhynchos
1	1.00	1	1	0.02	0.023	0.016	0.02	0.02	t	75318	YPHQPQQQFP	Mus musculus
1	1.00	1	1	0	0	0.006	0.002	0	t	67732	VARDDKGRRYYYLT	Homo sapiens
1	1.00	1	1	0	-0.004	0	-0.003	-0.003	t	71143	VSSYHSKPCNPAQPV	Oryctolagus cuniculus
1	1.00	1	1	0	-0.004	0	-0.003	-0.003	t	38345	LNTEPSQLPPTAPP	Oryctolagus cuniculus
1	1.00	1	1	0	-0.004	0	-0.003	-0.003	t	56614	RYPHYSLIKPESSL	Oryctolagus cuniculus
1	1.00	1	1	0	-0.004	0	-0.003	-0.003	t	6918	CRFPNITNSHVPILQE	Oryctolagus cuniculus

1	1.00	1	1	0.03	0.034	0.041	0.031	0.029	t	44700	NLEYTIVITP	Homo sapiens
1	1.00	1	1	-0.01	-0.01	0.001	-0.007	-0.01	t	11029	EAELTGYG	Homo sapiens
1	1.00	1	1	0	0	-0.012	0	0.003	t	10126	DSGDGRQTITNP	Mus musculus
1	1.00	1	1	0.01	0.01	0.01	0.012	0.01	t	25496	ICESAGTQEDAASL	Homo sapiens
1	1.00	1	1	-0.01	-0.014	-0.011	-0.011	-0.01	t	78615	CPGHANRKQCGG	Oryctolagus cuniculus
1	1.00	1	1	0.05	0.054	0.054	0.05	0.05	t	19034	GDLQVLAQKV	Cavia porcellus
1	1.00	1	1	0	0	0	0.003	0	t	117773	CNSRQTDREDELI	Homo sapiens
1	1.00	1	1	0	0	0	0.003	0	t	44920	NLVPMVATV	Homo sapiens
1	1.00	1	1	0.01	0.01	0.01	0.01	0.01	t	14475	ETLVTF	Homo sapiens
1	1.00	1	1	-0.02	-0.017	-0.014	-0.02	-0.021	t	69462	VLELAA	Mus musculus
1	1.00	1	1	0.02	0.016	0.023	0.023	0.02	t	115406	NRKVKPTTEGGVETE	Oryctolagus cuniculus
1	1.00	1	1	0.01	0.008	-0.006	0.009	0.011	t	149247	AEQQWALYADGSIRPQ	Mus musculus
1	1.00	1	1	0.04	0.038	0.032	0.033	0.038	t	122167	PPPITQGECKQL	Mus musculus
1	1.00	1	1	0.01	0.006	0.001	0.01	0.012	t	52441	QSTHVDIRTLEDLLMGTLGI	Mus musculus

1	1.00	1	1	0	-0.002	-0.002	-0.001	0	t	97683	TFSMGAKP	Mus musculus
1	1.00	1	1	0.03	0.03	0.03	0.03	0.03	t	41052	MAILGD	Homo sapiens
1	1.00	1	1	-0.01	-0.013	-0.013	-0.01	-0.01	t	7208	CTSPYDGKYWSMYSRL	Oryctolagus cuniculus
1	1.00	1	1	0.01	0.01	0.01	0.01	0.01	t	119618	NHYTQKSL	Homo sapiens
1	1.00	1	1	-0.01	-0.01	-0.014	-0.01	-0.01	t	47545	PFPQPQQQFP	Mus musculus
1	1.00	1	1	-0.04	-0.04	-0.04	-0.04	-0.042	t	141621	DNWHGSNRP	Oryctolagus cuniculus
1	1.00	1	1	0	0	0	0	0	t	4732	ASRAALIEEGQRIA	Homo sapiens
1	1.00	1	1	0.01	0	0.01	0.017	0.01	t	134194	PTKSDPRCLTRYYSSFVNME	Homo sapiens
1	1.00	1	1	0.02	0.02	0.02	0.02	0.02	t	144873	ATWVDLVLEGDSCLTI	Mus musculus
1	1.00	1	1	-0.01	-0.002	-0.01	-0.01	-0.012	t	156959	SLHTTQR	Gallus gallus
1	1.00	1	1	-0.02	-0.02	-0.02	-0.02	-0.02	t	111109	AMGYQHP	Homo sapiens
1	1.00	1	1	-0.02	-0.012	-0.02	-0.02	-0.022	t	156840	AAPKMCSTWCQMWQ	Gallus gallus
1	1.00	1	1	-0.01	-0.008	-0.011	-0.009	-0.012	t	63973	TGTDLELIQILKDHYNISLD	Homo sapiens
1	1.00	1	1	0	0.002	0	0.003	0.001	t	60780	SRRFAPAL	Homo sapiens
1	1.00	1	1	0	0	0	0	0	t	595	ACSFSSIP	Oryctolagus cuniculus
1	1.00	1	1	0.01	0.01	0.01	0.01	0.01	t	10040	DRRCQSQLER	Homo sapiens

1	1.00	1	1	0.02	0.018	0.02	0.02	0.02	t	40359	LVGATPE	Mus musculus
1	1.00	1	1	0.03	0.03	0.039	0.03	0.03	t	65860	TQGSNW	Homo sapiens
1	1.00	1	1	0.03	0.035	0.039	0.03	0.03	t	65860	TQGSNW	Homo sapiens
1	1.00	1	1	0.02	0.02	0.02	0.02	0.02	t	135899	ASLYHVYEVNLV	Homo sapiens
1	1.00	1	1	0.02	0.02	0.02	0.02	0.02	t	133598	HLNSLYYDWTEP	Homo sapiens
1	1.00	1	1	0.02	0.02	0.02	0.02	0.02	t	123165	FEQLKEGYL	Ovis aries
1	1.00	1	1	0.02	0.02	0.02	0.02	0.02	t	78357	DFPAGYLNDSTLFPP	Mus musculus
1	1.00	1	1	0.02	0.02	0.02	0.02	0.02	t	41948	MLGNAPSVVPNTTLGM	Homo sapiens
1	1.00	1	1	0.02	0.02	0.02	0.02	0.02	t	41948	MLGNAPSVVPNTTLGM	Homo sapiens
1	1.00	1	1	0	0	0	0	0	t	132933	WGQNSFP	Homo sapiens
1	1.00	1	1	0	0	0	0	0	t	112360	TMTCKSS	Homo sapiens
1	1.00	1	1	-0.01	-0.01	-0.01	-0.01	-0.01	t	22122	GRLGTYRYVASGER	Homo sapiens
1	1.00	1	1	0.01	0.007	-0.003	0.01	0.01	t	105942	LSADQISTVQASFDKVKGD	Oryctolagus cuniculus
1	1.00	1	1	0.02	0.026	0.023	0.02	0.02	t	141098	VERSESALF	Ovis aries
1	1.00	1	1	0.02	0.024	0.023	0.02	0.02	t	141098	VERSESALF	Ovis aries
1	1.00	1	1	0.01	0.01	0.002	0.008	0.01	t	48052	PKEITVATSRTLSYYKL	Homo sapiens

1	1.00	1	1	0.01	0.007	0.008	0.01	0.01	t	125772	CAPEPGIP GETRG	Oryctolagus cuniculus
1	1.00	1	1	0.06	0.06	0.062	0.06	0.06	t	114193	AEYYNKQY LEQTRAEL DT	Mus musculus
1	1.00	1	1	0.01	0.01	0.01	0.01	0.01	t	14638	EVDDEALE KF	Homo sapiens
1	1.00	1	1	0	0	0	0	-0.001	t	56686	RYYRE	Mus musculus
1	1.00	1	1	0.02	0.02	0.02	0.02	0.018	t	64770	TLEEWESG KNPYEPRE	Oryctolagus cuniculus
1	1.00	1	1	0.02	0.016	0.015	0.021	0.023	t	134021	CQSIAAPS KTS	Oryctolagus cuniculus
1	1.00	1	1	0.01	0.01	0.01	0.012	0.01	t	4911	ATCMQAD LEVMTST	Homo sapiens
1	1.00	1	1	-0.01	-0.01	-0.026	-0.008	-0.007	t	99014	SQCQAIH NVVHA	Homo sapiens
1	1.00	1	1	0.03	0.027	0.028	0.03	0.03	t	8526	DGYPVEYD NCAYICW	Oryctolagus cuniculus
1	1.00	1	1	-0.02	-0.018	-0.027	-0.024	-0.021	t	138807	RLQGISPKI	Homo sapiens
1	1.00	1	1	-0.01	-0.008	-0.012	-0.009	-0.01	t	22882	GTVVAVG PGRWDED G	Homo sapiens
1	1.00	1	1	0.03	0.034	0.025	0.03	0.03	t	94037	TIQNWFR EAD	Ovis aries
1	1.00	1	1	-0.03	-0.03	-0.038	-0.031	-0.03	t	82909	GTDSGWG NRQSF	Homo sapiens
1	1.00	1	1	0.01	0.01	0.009	0.006	0.01	t	55050	RNQMGVS ELL	Homo sapiens

1	1.00	1	1	0.01	0.01	0.01	0.01	0.01	t	153824	EKEKYKSITDELDQTFSE	Homo sapiens
1	1.00	1	1	0.04	0.048	0.024	0.04	0.042	t	110138	YMLDLQPETTDLYCYEQLND	Gallus gallus
1	1.00	1	1	0.04	0.037	0.024	0.04	0.042	t	110138	YMLDLQPETTDLYCYEQLND	Oryctolagus cuniculus
1	1.00	1	1	0.01	0.012	0.002	0.011	0.012	t	79279	LADLVLIAVIDHVTDLDK	Homo sapiens
1	1.00	1	1	-0.05	-0.05	-0.05	-0.05	-0.05	t	115384	MKPWIQPKTKVIPYVRYL	Homo sapiens
1	1.00	1	1	0.02	0.02	0.02	0.02	0.019	t	77802	TEYSASVKGRFI	Mus
1	1.00	1	1	-0.03	-0.032	-0.03	-0.029	-0.03	t	149371	LPYPQPQPFL	Mus musculus
1	1.00	1	1	-0.01	-0.01	-0.013	-0.01	-0.012	t	41601	MGLIAGAVGGSLLAALV	Mus musculus
1	1.00	1	1	0.01	0.012	0.015	0.017	0.01	t	122655	YKRGIIDRGQYLQLQELECD	Homo sapiens
1	1.00	1	1	0	0	0	0	-0.002	t	49270	PRVRGLY	Mus musculus
1	1.00	1	1	-0.02	-0.02	-0.019	-0.026	-0.022	t	114939	RPICGTDGVT	Homo sapiens
1	1.00	1	1	0.02	0.017	0.011	0.02	0.021	t	49691	PTLDHVLDHVQT	Oryctolagus cuniculus
1	1.00	1	1	0.01	0.01	0.01	0.01	0.01	t	136081	RTTLQSDQEEIDY	Oryctolagus cuniculus
1	1.00	1	1	0.01	0.01	0.01	0.01	0.01	t	136081	RTTLQSDQEEIDY	Oryctolagus cuniculus
1	1.00	1	1	0.01	0.01	0.01	0.01	0.01	t	136081	RTTLQSDQEEIDY	Homo sapiens

1	1.00	1	1	-0.01	-0.01	-0.014	-0.007	-0.01	t	86674	NDVFREAQRSYL	Homo sapiens
1	1.00	1	1	0	0	0	0	0	t	133623	KPGEMR	Mus musculus
1	1.00	1	1	0.03	0.028	0.023	0.023	0.029	t	108202	VVSHFND	Mus musculus
1	1.00	1	1	-0.03	-0.032	-0.031	-0.031	-0.031	t	51958	QPRKTKPVKSLPERK	Mus musculus
1	1.00	1	1	0.07	0.07	0.07	0.07	0.07	t	123628	PQQLMPGQ	Oryctolagus cuniculus
1	1.00	1	1	-0.02	-0.02	-0.02	-0.02	-0.022	t	138626	SQKDILERKRAAVDRVC	Oryctolagus cuniculus
1	1.00	1	1	-0.01	-0.01	-0.01	-0.01	-0.01	t	115931	QTPVVVPPFL	Homo sapiens
1	1.00	1	1	-0.01	-0.012	-0.01	-0.007	-0.011	t	70541	VQDKYGRVCYGD	Mus musculus
1	1.00	1	1	0.02	0.02	0.02	0.016	0.018	t	122406	IEIGLEGKGFEPTLEALFGK	Homo sapiens
1	1.00	1	1	-0.03	-0.04	-0.03	-0.023	-0.03	t	134147	LPKGVKHLKDFPILPGEIFK	Homo sapiens
1	1.00	1	1	0	-0.005	0.002	0.003	0	t	20939	GLIVDTRDVEERVHV	Bos taurus
1	1.00	1	1	0.03	0.028	0.017	0.033	0.032	t	5244	AVAASNNPELTTLTAALSGQ	Mus musculus
1	1.00	1	1	0	-0.005	0	0	0	t	108349	EQEETNPEVLIK	Canis lupus
1	1.00	1	1	0	0	0	0	0	t	108349	EQEETNPEVLIK	Canis lupus

1	1.00	1	1	-0.01	-0.01	-0.01	-0.01	-0.01	t	72910	WNSTTFHQTL	Mus musculus
1	1.00	1	1	-0.06	-0.06	-0.076	-0.058	-0.057	t	98924	PCMDVVLQQHNI	Homo sapiens
1	1.00	1	1	0.02	0.016	0.008	0.013	0.018	t	132886	PVRLAELHSDLKIQE	Oryctolagus cuniculus
1	1.00	1	1	-0.03	-0.03	-0.028	-0.03	-0.032	t	119467	ISGKPGPPPMGFHSPLIK	Oryctolagus cuniculus
1	1.00	1	1	-0.04	-0.044	-0.04	-0.047	-0.041	t	122320	DVTVGPLIFL	Oryctolagus cuniculus
1	1.00	1	1	0.04	0.042	0.045	0.037	0.039	t	95077	ETFTETWNRFITHTE	Homo sapiens
1	1.00	1	1	-0.02	-0.02	-0.02	-0.02	-0.02	t	111166	CPVYIKPC	Homo sapiens
1	1.00	1	1	-0.01	-0.01	-0.006	-0.007	-0.01	t	24508	HPSVRYVCLPPERD	Homo sapiens
1	1.00	1	1	0.06	0.048	0.055	0.06	0.06	t	50160	PYQPVSNLTATTQGQ	Mus musculus
1	1.00	1	1	0.02	0.02	0.02	0.02	0.02	t	122209	VQKDVLL	Homo sapiens
1	1.00	1	1	0	0	0	0	0	t	147368	VFSFGGYTLRGTS	Homo sapiens
1	1.00	1	1	0	0	0	0	0	t	117103	TYQDFSTRPRSF	Homo sapiens
1	1.00	1	1	0	0	0	0	0	t	80178	ADAPQGFYVL	Homo sapiens
1	1.00	1	1	0	0	0	0	0	t	97555	PLPTDAGV	Mus musculus
1	1.00	1	1	0	0	0	0	0	t	97555	PLPTDAGV	Mus musculus

1	1.00	1	1	0	-0.002	-0.007	0.003	-0.001	t	5558	AVTFASFDVPSKQPT	Mus musculus
1	1.00	1	1	0	-0.004	-0.007	0.003	-0.001	t	5558	AVTFASFDVPSKQPT	Oryctolagus cuniculus
1	1.00	1	1	0	0.005	-0.002	-0.003	-0.002	t	99383	KKNPQLQDLDMMLTC	Homo sapiens
1	1.00	1	1	0.01	0.01	0.005	0.008	0.01	t	5057	ATMILAYAMRVPEVI	Homo sapiens
1	1.00	1	1	-0.01	-0.01	-0.01	-0.006	-0.01	t	142202	KISEASRKGLRRDLDASREA	Homo sapiens
1	1.00	1	1	-0.01	-0.006	-0.01	-0.003	-0.01	t	123636	PVGPAPGMRP	Homo sapiens
1	1.00	1	1	0.01	0.01	0.008	0.01	0.01	t	110606	GNPGTDGIPGAKG	Rattus norvegicus
1	1.00	1	1	0	0.004	0.004	0.007	0	t	133672	RGGMR	Homo sapiens
1	1.00	1	1	-0.01	-0.01	-0.001	-0.01	-0.012	t	46055	NTANPDWDF	Mus musculus
1	1.00	1	1	-0.04	-0.04	-0.04	-0.04	-0.041	t	135722	NGKVDAFC	Homo sapiens
1	1.00	1	1	0	-0.002	0.008	-0.001	-0.002	t	23913	HGISEMWYLTPK	Mus musculus
1	1.00	1	1	-0.01	-0.01	-0.026	-0.008	-0.007	t	98814	HNVVHAIILHQQ	Homo sapiens
1	1.00	1	1	0	0	0	0	-0.002	t	774	ADSVGGKD	Mus musculus
1	1.00	1	1	-0.01	-0.008	-0.01	-0.009	-0.01	t	65931	TQSFLATCVNGVCW	Homo sapiens
1	1.00	1	1	-0.01	-0.01	-0.01	-0.01	-0.01	t	71312	VTGATVS	Oryctolagus cuniculus

1	1.00	1	1	-0.01	-0.01	-0.01	-0.01	-0.01	t	71312	VTGATVS	Oryctolagus cuniculus
1	1.00	1	1	-0.02	-0.018	-0.029	-0.023	-0.022	t	105940	LRYQQQSRRRSLPYS	Homo sapiens
1	1.00	1	1	-0.01	-0.002	-0.01	-0.007	-0.01	t	110839	ETTSQMIVKDIK	Homo sapiens
1	1.00	1	1	0	0.003	0	0	-0.002	t	135458	FGSDYEDRYYR	Mus musculus
1	1.00	1	1	0.01	0.005	-0.004	0.01	0.012	t	132789	DGFMDFEVYSHQTKP	Trichosurus vulpecula
1	1.00	1	1	0.01	0.012	0.015	0.011	0.01	t	65325	TNGLASL	Homo sapiens
1	1.00	1	1	0.01	0.01	0.01	0.01	0.01	t	48382	PLKPTKRSFIEDLLF	Homo sapiens
1	1.00	1	1	0.03	0.028	0.024	0.027	0.03	t	151711	RATHQE	Mus musculus
1	1.00	1	1	-0.06	-0.058	-0.081	-0.063	-0.058	t	53483	REECKCLLNYKQEGDKCVE	Homo sapiens
1	1.00	1	1	0.02	0.025	0.023	0.016	0.02	t	48129	PKQGPLPN	Homo sapiens
1	1.00	1	1	-0.01	-0.008	-0.018	-0.013	-0.01	t	13911	EQQRFRNCQRYV	Homo sapiens
1	1.00	1	1	-0.04	-0.04	-0.032	-0.043	-0.046	t	30984	KGITTKWMA	Homo sapiens
1	1.00	1	1	0.01	0.008	0.01	0.009	0.009	t	51147	QIVGGVYLL	Mus musculus
1	1.00	1	1	0	0	0	0	0	t	24651	HQTLQDPRVRG	Mus musculus

1	1.00	1	1	0	0	0	0	0	t	24651	HQTLQDPRVRG	Mus musculus
1	1.00	1	1	0	0	0	0	0	t	24651	HQTLQDPRVRG	Mus musculus
1	1.00	1	1	0.03	0.03	0.03	0.03	0.03	t	98986	RKIRPEGREGEQEWG	Homo sapiens
1	1.00	1	1	0.03	0.03	0.03	0.03	0.03	t	78299	WHLAYSP	Homo sapiens
1	1.00	1	1	0	0	-0.014	0	0	t	10799	DWDFNPNKDTWPDAN	Mus musculus
1	1.00	1	1	0.01	0.01	0.01	0.01	0.01	t	149078	NITQEAF	Sus scrofa
1	1.00	1	1	0.01	0.01	0.01	0.01	0.01	t	105975	NGIEETICTASFKKN	Homo sapiens
1	1.00	1	1	0.01	0.01	0.01	0.01	0.01	t	103687	VCAVDMPYLTVELIE	Homo sapiens
1	1.00	1	1	0.01	0.01	0.01	0.01	0.01	t	78356	DFLIRYLNDSTPFPF	Mus musculus
1	1.00	1	1	0	0.002	0.005	0.003	0.002	t	138238	SMLDTPT	Homo sapiens
1	1.00	1	1	-0.01	-0.01	-0.01	-0.01	-0.01	t	119578	FAIVKFTKVLLQYTGH	Mus musculus
1	1.00	1	1	-0.01	-0.01	-0.01	-0.01	-0.01	t	119578	FAIVKFTKVLLQYTGH	Homo sapiens
1	1.00	1	1	-0.01	-0.01	-0.01	-0.01	-0.01	t	119578	FAIVKFTKVLLQYTGH	Homo sapiens
1	1.00	1	1	0	0	0	0	0	t	140510	TTTHFLATKFYK	Mus musculus
1	1.00	1	1	0.02	0.014	0.02	0.019	0.02	t	4884	ATALDTSN	Oryctolagus cuniculus
1	1.00	1	1	0.03	0.028	0.025	0.033	0.031	t	16396	FKLEKEVAE	Mus musculus
1	1.00	1	1	0	0	0	0	0	t	149979	TKLSHKN	Capra hircus

1	1.00	1	1	0.02	0.02	0.018	0.02	0.019	t	22015	GREGYPA DSKGCKIT	Mus musculu s
1	1.00	1	1	0	0.004	0	0.001	0	t	73449	YASSGIGP	Homo sapiens
1	1.00	1	1	0	0.002	0	0.001	0	t	54861	RMAWDM MM	Homo sapiens
1	1.00	1	1	0	0	-0.01	-0.001	0.002	t	57875	SFQSFNLT EPHIT	Homo sapiens
1	1.00	1	1	-0.02	-0.02	-0.01	-0.02	-0.021	t	45420	NPNDPP	Mus musculu s
1	1.00	1	1	-0.01	-0.008	-0.019	-0.013	-0.012	t	106196	YGLPREQA RQLKNNN	Homo sapiens
1	1.00	1	1	0.08	0.074	0.074	0.073	0.082	t	127778	PSEFEADL MLGP	Trichosu rus vulpecul a
1	1.00	1	1	0	0	0	-0.003	-0.003	t	72087	VYLLPR	Homo sapiens
1	1.00	1	1	0.02	0.023	0.02	0.02	0.022	t	7532	DAHHAAD	Mus musculu s
1	1.00	1	1	0	-0.003	-0.014	0	0.004	t	97590	QRDKVNL TL	Oryctola gus cuniculu s
1	1.00	1	1	0.01	0.008	-0.005	0.009	0.011	t	36729	LITNTIAGV	Mus musculu s
1	1.00	1	1	-0.01	-0.01	0	-0.009	-0.012	t	75286	YPFMWG GAYCF	Mus musculu s
1	1.00	1	1	0.01	0.01	0.008	0.017	0.01	t	120805	MSGRGKQ GGKARAK A	Homo sapiens
1	1.00	1	1	-0.03	-0.032	-0.022	-0.029	-0.033	t	149384	MSLPGRW KPK	Mus musculu s

1	1.00	1	1	0.01	0.012	0.005	0.01	0.01	t	74162	YIAGDPALAYGYAQDQEPDA	Homo sapiens
1	1.00	1	1	0.02	0.02	0.02	0.02	0.02	t	92845	GNGKITQDEL	Ovis aries
1	1.00	1	1	0	0	0	0	0	t	1666	AGRMIPKG	Anas platyrhynchos
1	1.00	1	1	-0.01	-0.022	-0.008	-0.01	-0.012	t	146185	DAEFGH	Macaca mulatta
1	1.00	1	1	0.01	0.013	0.005	0.01	0.009	t	96216	EEEGLLKKSADTLWNMQK	Mus musculus
1	1.00	1	1	0.01	0.012	0.015	0.007	0.008	t	18963	GDEGRSVFDDNV	Homo sapiens
1	1.00	1	1	-0.01	-0.01	-0.01	-0.006	-0.01	t	18759	GASAQSGASAQSGAS	Homo sapiens
1	1.00	1	1	0.01	0.013	0.012	0.01	0.01	t	135845	WTEIHDWEAAME	Mus musculus
1	1.00	1	1	0.02	0.02	0.02	0.02	0.019	t	49998	PVVEVNGVTIQVGSR	Mus musculus
1	1.00	1	1	-0.01	-0.013	-0.01	-0.01	-0.01	t	50156	PYQGSGKGVS	Oryctolagus cuniculus
1	1.00	1	1	0.01	0.014	0.01	0.011	0.01	t	56364	RVMYASSG	Homo sapiens
1	1.00	1	1	0.03	0.03	0.03	0.03	0.03	t	145145	DPPERPDSP	Mus musculus
1	1.00	1	1	0.01	0.01	0.01	0.01	0.01	t	8514	DGVMSTRCPCGAAI	Homo sapiens
1	1.00	1	1	-0.01	-0.01	-0.01	-0.01	-0.01	t	107773	CSVMHEG	Homo sapiens

1	1.00	1	1	-0.01	-0.014	-0.021	-0.01	-0.01	t	28331	IRLRHENKDLKARLENAMEV	Mus musculus
1	1.00	1	1	-0.02	-0.02	-0.011	-0.02	-0.023	t	134492	QIQRRPSM	Mus musculus
1	1.00	1	1	-0.04	-0.043	-0.043	-0.04	-0.037	t	124708	SFERFEIF	Oryctolagus cuniculus
1	1.00	1	1	-0.03	-0.03	-0.031	-0.03	-0.03	t	127963	EPKPKKAPAKKGEKV	Homo sapiens
1	1.00	1	1	-0.03	-0.027	-0.044	-0.03	-0.028	t	100295	LVDGNGRFVF	Mus musculus
1	1.00	1	1	0	0.002	0	0.001	0	t	47260	PEDTLNKV	Homo sapiens
1	1.00	1	1	0	0	-0.016	0	0.004	t	76491	YYDGRRS	Mus musculus
1	1.00	1	1	0	0	0	0	-0.001	t	102510	HHQKLVFFAEDVGSNK	Rattus norvegicus
1	1.00	1	1	0	-0.003	0	0	-0.001	t	102510	HHQKLVFFAEDVGSNK	Oryctolagus cuniculus
1	1.00	1	1	0	-0.003	0	0	-0.001	t	68401	VEVPGSQHIDSQKKA	Oryctolagus cuniculus
1	1.00	1	1	0	0.004	0	0	-0.001	t	72805	WLTEKEGSYP	Ovis aries
1	1.00	1	1	0	-0.003	0	0	-0.001	t	48670	PNLRGDLQVLAQKVARTLP	Oryctolagus cuniculus
1	1.00	1	1	0	-0.003	0	0	-0.001	t	48670	PNLRGDLQVLAQKVARTLP	Oryctolagus cuniculus

1	1.00	1	1	0	-0.003	0	0	-0.001	t	11815	EENVEENV EENVEENV	Oryctolagus cuniculus
1	1.00	1	1	0	0	0	0	-0.001	t	32844	KPQEEKEK ITKEILNGK	Rattus norvegicus
1	1.00	1	1	0	-0.003	0	0	-0.001	t	32844	KPQEEKEK ITKEILNGK	Oryctolagus cuniculus
1	1.00	1	1	0	-0.003	0	0	-0.001	t	9106	DLGSIA	Oryctolagus cuniculus
1	1.00	1	1	0	-0.002	0	0	-0.001	t	7171	CTKPTDG NC	Mus musculus
1	1.00	1	1	0	0.012	0	0	-0.001	t	9757	DPRVRGLY FPAGGSSS GTV	Pan troglodytes
1	1.00	1	1	0.01	0.01	0.005	0.01	0.011	t	140674	ESQPDPKP DELHKS	Mus musculus
1	1.00	1	1	0.04	0.038	0.053	0.043	0.041	t	138862	KFPED	Mus musculus
1	1.00	1	1	0.01	0.01	0.01	0.013	0.01	t	96450	KEKPTPPV KP	Homo sapiens(human)
1	1.00	1	1	-0.02	-0.02	-0.02	-0.02	-0.022	t	144661	GREGYPA DGGCAWP ACYC	Oryctolagus cuniculus
1	1.00	1	1	0.03	0.03	0.03	0.03	0.03	t	141623	DSKLNEIF GSSDTNDI	Rattus norvegicus
1	1.00	1	1	0.03	0.032	0.03	0.03	0.03	t	141623	DSKLNEIF GSSDTNDI	Rattus norvegicus
1	1.00	1	1	0.03	0.03	0.03	0.03	0.03	t	75848	YSPEVG	Mus musculus
1	1.00	1	1	0.01	0.01	0.01	0.01	0.01	t	111156	CMNKPAY AC	Homo sapiens

1	1.00	1	1	0	0	-0.005	0	0.001	t	10868	DYEDRYYRE	Mus musculus
1	1.00	1	1	-0.03	-0.03	-0.03	-0.03	-0.03	t	156423	QNQLDQVPRRFYLAG	Homo sapiens
1	1.00	1	1	-0.03	-0.032	-0.05	-0.037	-0.03	t	128043	LDAMPAGNPAFPVIPG	Mus musculus
1	1.00	1	1	-0.01	-0.01	-0.023	-0.008	-0.008	t	14426	ETIGTLKKILDETVK	Homo sapiens
1	1.00	1	1	0.01	0.013	0.015	0.01	0.008	t	124924	YYVFFEEQEDE	Mus musculus
1	1.00	1	1	0.01	0.009	0.012	0.011	0.009	t	24362	HNASGKEAKVCTISPIMGYS	Homo sapiens
1	1.00	1	1	0.02	0.02	0.013	0.02	0.022	t	7005	CSGPTTIRGQFS	Mus musculus
1	1.00	1	1	0.04	0.04	0.04	0.04	0.04	t	156866	FGSSSAG	Gallus gallus
1	1.00	1	1	0	-0.002	0.002	-0.001	0	t	52021	QQFGRDVSDF	Mus musculus
1	1.00	1	1	0.02	0.028	0.02	0.02	0.018	t	156848	ALTVRCTAPCPQRN	Gallus gallus
1	1.00	1	1	-0.03	-0.026	-0.027	-0.029	-0.03	t	150874	NWFDISKWLWYI	Homo sapiens
1	1.00	1	1	0.02	0.022	0.01	0.017	0.02	t	158728	DVKKAFKVIDQDASG	Homo sapiens
1	1.00	1	1	0	0	0.002	0.001	0.001	t	65678	TPPAYR	Mus musculus
1	1.00	1	1	0.01	0.01	0.005	0.01	0.01	t	102946	YGCYCGVGG	Mus musculus
1	1.00	1	1	0.03	0.026	0.03	0.03	0.029	t	123001	AASEDPR	Mus musculus

1	1.00	1	1	-0.01	-0.01	-0.014	-0.01	-0.013	t	114666	VRSRPSRS RSSRSER	Homo sapiens
1	1.00	1	1	-0.01	-0.01	-0.015	-0.011	-0.014	t	138242	SQPEILERT RAEVDTA	Mus musculu s
1	1.00	1	1	0.02	0.02	0.02	0.02	0.02	t	65798	TPVIGSQA	Homo sapiens
1	1.00	1	1	0	0	0	0	0	t	73635	YEDRYY	Mus musculu s
1	1.00	1	1	-0.01	-0.008	-0.01	-0.009	-0.009	t	46316	NTWKSK	Homo sapiens
1	1.00	1	1	-0.03	-0.03	-0.028	-0.03	-0.029	t	16123	FHLPYNHN WFAL	Homo sapiens
1	1.00	1	1	0.02	0.018	0.019	0.023	0.02	t	106690	NSDHFLQP DPPL	Mus musculu s
1	1.00	1	1	0.02	0.02	0.02	0.02	0.02	t	106921	TPYTNNPD LLFT	Mus musculu s
1	1.00	1	1	0.02	0.02	0.02	0.02	0.02	t	78260	RIVSIT	Equus caballus
1	1.00	1	1	0	0	0	0	0	t	148591	FAQPQQL FPE	Homo sapiens
1	1.00	1	1	0.01	0.008	0.009	0.009	0.01	t	39349	LSFNPTQL EEQCHI	Mus musculu s
1	1.00	1	1	0.01	0.01	0.007	0.01	0.009	t	52910	QYGLEKRD NGHQGE	Mus musculu s
1	1.00	1	1	-0.01	-0.01	-0.017	-0.019	-0.013	t	131038	RKIAKDEG AK	Homo sapiens
1	1.00	1	1	-0.01	-0.006	-0.013	-0.01	-0.008	t	123754	SRISYDPKF	Ovis aries
1	1.00	1	1	0.01	0.006	0.013	0.009	0.008	t	47863	PHYSLIKPE SSL	Oryctola gus cuniculu s

1	1.00	1	1	0.02	0.014	0.018	0.02	0.02	t	32623	KNTTTTQTQPSKPTT	Oryctolagus cuniculus
1	1.00	1	1	-0.06	-0.058	-0.059	-0.059	-0.061	t	3687	APPLRTWKSRARAV	Homo sapiens
1	1.00	1	1	0.03	0.032	0.03	0.031	0.03	t	62993	TASITSPLTTQNTL	Homo sapiens
1	1.00	1	1	-0.02	-0.02	-0.022	-0.02	-0.021	t	120846	NREPVQLETLSIRGN	Mus musculus
1	1.00	1	1	0.01	0.01	0.01	0.01	0.01	t	78141	EKAEEVEK	Homo sapiens
1	1.00	1	1	0.01	0.01	0.01	0.01	0.01	t	55036	RNNHPQNTSDSQKEATD	Oryctolagus cuniculus
1	1.00	1	1	0.02	0.02	0.018	0.02	0.021	t	144791	STIDYFQPNNKR	Sus scrofa
1	1.00	1	1	-0.01	-0.01	-0.01	-0.01	-0.01	t	79552	GEDVGAFPPGKFVTAAL	Homo sapiens
1	1.00	1	1	-0.01	-0.012	-0.01	-0.007	-0.01	t	95829	SGASAQSGTSGP	Mus musculus
1	1.00	1	1	0.03	0.038	0.023	0.03	0.031	t	106495	GYEVHHQKLVF	Gallus gallus
1	1.00	1	1	0	0	-0.006	0.002	0	t	25004	HTYARFGIEIQVQINPTPFQ	Homo sapiens
1	1.00	1	1	-0.02	-0.02	-0.018	-0.014	-0.018	t	6233	CFKKPPKYPPALPI	Homo sapiens
1	1.00	1	1	-0.01	-0.011	-0.02	-0.007	-0.008	t	31502	KKDGKKAEPKNPRENKLDQP	Homo sapiens
1	1.00	1	1	0.03	0.03	0.033	0.037	0.032	t	115649	SPGKNATGMEVG	Homo sapiens
1	1.00	1	1	0	-0.004	-0.007	-0.001	-0.001	t	46859	PALTAVETGATNPL	Oryctolagus cuniculus

1	1.00	1	1	0.02	0.023	0.02	0.02	0.021	t	43839	NFGDQDLIRQGTDYK	Macaca cyclopis
1	1.00	1	1	0.04	0.037	0.005	0.04	0.05	t	44172	NHVADIDKLIDYAA	Oryctolagus cuniculus
1	1.00	1	1	0.03	0.03	0.03	0.03	0.03	t	102248	YNPTYLL	Homo sapiens
1	1.00	1	1	0.03	0.03	0.03	0.03	0.03	t	7514	DAGDSAYSQ	Mus musculus
1	1.00	1	1	0.01	0.01	0.01	0.01	0.01	t	134757	PEAPRRRSPSPTPTP	Homo sapiens
1	1.00	1	1	0.01	0.01	0.01	0.01	0.009	t	7003	CSGLSSLFTPGPKQQI	Mus musculus
1	1.00	1	1	0.07	0.07	0.07	0.07	0.07	t	110916	KLDDTENASAYAANA	Mus musculus
1	1.00	1	1	0.07	0.07	0.07	0.07	0.07	t	110916	KLDDTENASAYAANA	Mus musculus
1	1.00	1	1	0.04	0.044	0.031	0.04	0.039	t	112581	LKKYTFWEVNLKEKFSADLD	Cavia porcellus
1	1.00	1	1	-0.02	-0.018	-0.025	-0.019	-0.022	t	56856	SALEWIDLDSSL	Homo sapiens
1	1.00	1	1	0.01	0.01	0.005	0.01	0.01	t	134131	LEEAKTQGPYDVVVL	Mus musculus
1	1.00	1	1	-0.04	-0.04	-0.04	-0.033	-0.04	t	135994	SGLAPKRPGPAG	Homo sapiens
1	1.00	1	1	0.01	0.007	0.004	0.01	0.008	t	21911	GQLQAVNPNAGNRGQ	Oryctolagus cuniculus
1	1.00	1	1	0	-0.002	0	-0.001	-0.002	t	150987	GKCPKYVKSTKLRLA	Mus musculus
1	1.00	1	1	0	-0.002	0	-0.001	-0.002	t	150978	EKNVTVTHSVNLLED	Mus musculus
1	1.00	1	1	0	-0.002	0	-0.001	-0.002	t	137290	SEIEKLKNKYEVAGIP	Mus musculus

1	1.00	1	1	0	-0.002	0	-0.001	-0.002	t	151023	LCKLRGVA PLHLGKC	Mus musculu s
1	1.00	1	1	0	-0.002	0	-0.001	-0.002	t	128004	GMIEVQS NYGFVWD VSLKKV	Mus musculu s
1	1.00	1	1	0	-0.004	0	-0.001	-0.002	t	133518	ASDVVKK MVGETVE SA	Oryctola gus cuniculu s
1	1.00	1	1	0	-0.004	0	-0.001	-0.002	t	133733	VKTLLPYG SVINYYGY VKPG	Oryctola gus cuniculu s
1	1.00	1	1	0	-0.002	0	-0.001	-0.002	t	130994	PDQRPYC WHYPPKP C	Mus musculu s
1	1.00	1	1	0	-0.002	0	-0.001	-0.002	t	128066	NSYDVLSA LSGNLKL	Mus musculu s
1	1.00	1	1	0	-0.004	0	-0.001	-0.002	t	133540	DALVAKA QEVS	Oryctola gus cuniculu s
1	1.00	1	1	0	-0.002	0	-0.001	-0.002	t	128084	QKNACSF DLCNSYDV L	Mus musculu s
1	1.00	1	1	0	-0.002	0	-0.001	-0.002	t	127944	DLVNCNL NTNCVAV AFSLPD	Mus musculu s
1	1.00	1	1	0	-0.002	0	-0.001	-0.002	t	127984	FTNLKFKV RKITSSHR GN	Mus musculu s
1	1.00	1	1	0	-0.002	0	-0.001	-0.002	t	103545	RGIHVDIII DRSHSKLT FKQ	Mus musculu s
1	1.00	1	1	0	-0.002	0	-0.001	-0.002	t	110920	KPSPWAP KKHRRLS	Mus musculu s
1	1.00	1	1	0	-0.002	0	-0.001	-0.002	t	103049	ANYTNLSL RLDNNL	Mus musculu s

1	1.00	1	1	0	-0.002	0	-0.001	-0.002	t	110284	HKSAIVTLTYDSEWQRDQ	Mus musculus
1	1.00	1	1	0	-0.004	0	-0.001	-0.002	t	117348	PTRAPSGPRPP	Oryctolagus cuniculus
1	1.00	1	1	0	-0.004	0	-0.001	-0.002	t	111013	RTGYIPLGTRPPT	Oryctolagus cuniculus
1	1.00	1	1	0	-0.002	0	-0.001	-0.002	t	103188	FVLPQDRKIAIQAVLEKIQT	Mus musculus
1	1.00	1	1	0	-0.004	0	-0.001	-0.002	t	110284	HKSAIVTLTYDSEWQRDQ	Oryctolagus cuniculus
1	1.00	1	1	0	-0.002	0	-0.001	-0.002	t	107852	GEPQSSIIQY	Mus musculus
1	1.00	1	1	0	-0.002	0	-0.001	-0.002	t	110284	HKSAIVTLTYDSEWQRDQ	Mus musculus
1	1.00	1	1	0	-0.004	0	-0.001	-0.002	t	71312	VTGATVS	Oryctolagus cuniculus
1	1.00	1	1	0	-0.002	0	-0.001	-0.002	t	65360	TNLEDPASRDLVVSY	Mus musculus
1	1.00	1	1	0	-0.004	0	-0.001	-0.002	t	85833	LSIIAEQSNSTVDNQK	Oryctolagus cuniculus
1	1.00	1	1	0	-0.004	0	-0.001	-0.002	t	64690	TKVAEAEKQKAAEA	Oryctolagus cuniculus
1	1.00	1	1	0	-0.002	0	-0.001	-0.002	t	68895	VHTWTEQYK	Mus musculus
1	1.00	1	1	0	-0.004	0	-0.001	-0.002	t	64978	TLNKDQLLSSSKYTIQRSTG	Oryctolagus cuniculus

1	1.00	1	1	0	-0.004	0	-0.001	-0.002	t	70437	VPPTQSKKKNKNET	Oryctolagus cuniculus
1	1.00	1	1	0	-0.004	0	-0.001	-0.002	t	61410	SSTRGGSRVEGIFAGLFMNA	Oryctolagus cuniculus
1	1.00	1	1	0	-0.004	0	-0.001	-0.002	t	65298	TNDEEDTNDEEDTNDEED	Oryctolagus cuniculus
1	1.00	1	1	0	-0.004	0	-0.001	-0.002	t	67558	VAEAEKQKAAEATK	Oryctolagus cuniculus
1	1.00	1	1	0	-0.002	0	-0.001	-0.002	t	79303	LVAAGVDYEDERISFQDWPK	Rattus norvegicus
1	1.00	1	1	0	-0.003	0	-0.001	-0.002	t	70937	VSFQYNAVGPYLNINFFDSS	Mus musculus
1	1.00	1	1	0	-0.002	0	-0.001	-0.002	t	66539	TTASGKLIT	Mus musculus
1	1.00	1	1	0	-0.002	0	-0.001	-0.002	t	46258	NTSDSQKE	Mus musculus
1	1.00	1	1	0	-0.004	0	-0.001	-0.002	t	51163	QKAAEATKVAEAEK	Oryctolagus cuniculus
1	1.00	1	1	0	-0.006	0	-0.001	-0.002	t	44852	NLQKDEPNGRASDT	Oryctolagus cuniculus
1	1.00	1	1	0	-0.004	0	-0.001	-0.002	t	51454	QLNPKDNDVEL	Oryctolagus cuniculus
1	1.00	1	1	0	0.004	0	-0.001	-0.002	t	46371	NVESQPGGQPNT	Mus musculus
1	1.00	1	1	0	-0.004	0	-0.001	-0.002	t	36974	LKNKIFPKKKEDNQAVDT	Oryctolagus cuniculus

1	1.00	1	1	0	-0.004	0	-0.001	-0.002	t	38345	LNTEPSQL PPTAPP	Oryctolagus cuniculus
1	1.00	1	1	0	-0.002	0	-0.001	-0.002	t	39164	LRPGPVSQ PYHHWDT DK	Mus musculus
1	1.00	1	1	0	-0.004	0	-0.001	-0.002	t	46895	PAPQAPY QGYQEPP APQAPY	Oryctolagus cuniculus
1	1.00	1	1	0	-0.004	0	-0.001	-0.002	t	56614	RYPHYSLIK PESSL	Oryctolagus cuniculus
1	1.00	1	1	0	-0.002	0	-0.001	-0.002	t	37055	LKVIRK	Mus musculus
1	1.00	1	1	0	-0.004	0	-0.001	-0.002	t	44251	NIISCNKN DKNQ	Oryctolagus cuniculus
1	1.00	1	1	0	-0.004	0	-0.001	-0.002	t	30349	KEEKEEKE EKEEKEKE KE	Oryctolagus cuniculus
1	1.00	1	1	0	-0.004	0	-0.001	-0.002	t	30988	KGIYQTSN FRVVPSGD VVRF	Oryctolagus cuniculus
1	1.00	1	1	0	-0.004	0	-0.001	-0.002	t	33859	KTSERSQP RGRRQPIP KARR	Oryctolagus cuniculus
1	1.00	1	1	0	-0.004	0	-0.001	-0.002	t	10892	DYHRVMY	Oryctolagus cuniculus
1	1.00	1	1	0	-0.004	0	-0.001	-0.002	t	13460	ENDVLNQ ETEEEMEK	Oryctolagus cuniculus
1	1.00	1	1	0	-0.002	0	-0.001	-0.002	t	35252	LDPHAFHL LL	Mus musculus
1	1.00	1	1	0	0.009	0	-0.001	-0.002	t	21571	GPARRGA PY	Oryctolagus cuniculus

1	1.00	1	1	0	-0.004	0	-0.001	-0.002	t	12625	EKAFREL	Oryctolagus cuniculus
1	1.00	1	1	0	-0.002	0	-0.001	-0.002	t	8119	DEPAGE	Mus musculus
1	1.00	1	1	0	-0.004	0	-0.001	-0.002	t	8942	DKNEKGQHEIVEVEEILPE	Oryctolagus cuniculus
1	1.00	1	1	0	-0.002	0	-0.001	-0.002	t	6632	CLTFGRETVIEY	Mus musculus
1	1.00	1	1	0	-0.004	0	-0.001	-0.002	t	5387	AVIRRANNVLKNEMKRYKGL	Oryctolagus cuniculus
1	1.00	1	1	0	-0.004	0	-0.001	-0.002	t	843	AEATKVAEAEKQKA	Oryctolagus cuniculus
1	1.00	1	1	-0.01	-0.01	-0.014	-0.01	-0.01	t	157811	QRQCQQRCER	Homo sapiens
1	1.00	1	1	0	0.002	0.001	0.007	0	t	122655	YKRGIIDRGQYLQLQELECD	Homo sapiens
1	1.00	1	1	0	0	0	0	0	t	117140	DWEYSVWLSN	Homo sapiens
1	1.00	1	1	0	0	0	0	0	t	117140	DWEYSVWLSN	Homo sapiens
1	1.00	1	1	0	0.004	0	0.001	0	t	22133	GRLITVNP	MD
1	1.00	1	1	0.02	0.02	0.027	0.02	0.02	t	4701	ASQKIQLI	Mus musculus
1	1.00	1	1	0.01	0.008	0.001	0.009	0.01	t	89	AADKPTLDIRMMNIEA	Mus musculus
1	1.00	1	1	0.01	0.008	0.001	0.009	0.01	t	89	AADKPTLDIRMMNIEA	Mus musculus
1	1.00	1	1	0.02	0.02	0.02	0.02	0.02	t	56862	SALLED	Mus musculus
1	1.00	1	1	0.02	0.02	0.02	0.02	0.02	t	56862	SALLED	Mus musculus

1	1.00	1	1	0	0	0	0	0	t	57705	SEYPSQPLS	Homo sapiens
1	1.00	1	1	0	0	0	0	0	t	109996	TRLSRTIGYTVK	Mus musculus
1	1.00	1	1	-0.02	-0.02	-0.024	-0.02	-0.02	t	94445	CCHPACGKHF	Capra hircus
1	1.00	1	1	-0.02	-0.013	-0.024	-0.02	-0.02	t	94445	CCHPACGKHF	Capra hircus
1	1.00	1	1	0.02	0.028	0.02	0.023	0.02	t	110922	KQMDIKSINDLQ	Homo sapiens
1	1.00	1	1	-0.02	-0.02	-0.021	-0.02	-0.022	t	16043	FGQIFRPDNFVFGQS	Mus musculus
1	1.00	1	1	0.02	0.022	0.02	0.021	0.02	t	117097	TLSTDQSTTRNW	Homo sapiens
1	1.00	1	1	0.02	0.02	0.02	0.02	0.02	t	40292	LVDADDPL	Homo sapiens
1	1.00	1	1	-0.02	-0.02	-0.02	-0.02	-0.02	t	130453	CLFFQNGKVKCAAC	Homo sapiens
1	1.00	1	1	-0.04	-0.04	-0.04	-0.036	-0.04	t	115784	HRTFE	Homo sapiens
1	1.00	1	1	-0.01	-0.002	-0.01	-0.01	-0.012	t	156840	AAPKMCSTWCQMWQ	Gallus gallus
1	1.00	1	1	-0.03	-0.027	-0.03	-0.03	-0.032	t	135501	SQWNKP	Mus musculus
1	1.00	1	1	0.02	0.02	0.02	0.02	0.02	t	139930	TYDQIQKRFEGATPSYLS	Oryctolagus cuniculus
1	1.00	1	1	0.02	0.02	0.02	0.02	0.02	t	135442	AGLGGL	Oryctolagus cuniculus
1	1.00	1	1	0.02	0.02	0.02	0.02	0.02	t	133955	WIMNNF	Mus musculus

1	1.00	1	1	0.02	0.02	0.02	0.02	0.02	t	103002	AAASLAAIAIAFLAG	Homo sapiens
1	1.00	1	1	0.02	0.018	0.018	0.019	0.02	t	97565	PTGSAAAN	Mus musculus
1	1.00	1	1	0.03	0.034	0.042	0.031	0.03	t	17402	FPSDEF	Homo sapiens
1	1.00	1	1	-0.05	-0.05	-0.05	-0.053	-0.053	t	110997	RCHAANPNGRYYWGGQYTWD	Mus musculus
1	1.00	1	1	-0.03	-0.034	-0.039	-0.031	-0.031	t	71851	VVTHEMAHALG	Oryctolagus cuniculus
1	1.00	1	1	0	0	0	0	0.001	t	45410	NPNANPNANPNA	Mus musculus
1	1.00	1	1	-0.01	-0.01	-0.01	-0.01	-0.01	t	136082	SDDPSPGAIDSNNS	Oryctolagus cuniculus
1	1.00	1	1	-0.01	-0.01	-0.01	-0.01	-0.01	t	136082	SDDPSPGAIDSNNS	Oryctolagus cuniculus
1	1.00	1	1	0	0	-0.007	0	0	t	158938	KTQNQGPWDEGRVEID	Mus musculus
1	1.00	1	1	-0.01	-0.01	-0.01	-0.01	-0.01	t	54224	RIQLLEEDLER	Homo sapiens
1	1.00	1	1	-0.02	-0.016	-0.02	-0.02	-0.023	t	75951	YTASARGDLAHLTTTHAR	Cavia porcellus
1	1.00	1	1	-0.02	-0.016	-0.02	-0.02	-0.023	t	75951	YTASARGDLAHLTTTHAR	Cavia porcellus

1	1.00	1	1	0.01	0.01	0.01	0.012	0.01	t	6060	CCDLAPEARQAIKS	Homo sapiens
1	1.00	1	1	0.03	0.028	0.023	0.023	0.03	t	130916	KEDFDIYDEDE	Mus musculus
1	1.00	1	1	0	-0.002	-0.017	0.001	0.002	t	136749	WMLRAAEEYSMNLGF	Mus musculus
1	1.00	1	1	0.01	0.007	0.01	0.01	0.01	t	122289	CSNSSSSQFQIHGP	Oryctolagus cuniculus
1	1.00	1	1	0.01	0.007	0.01	0.01	0.01	t	122289	CSNSSSSQFQIHGP	Oryctolagus cuniculus
1	1.00	1	1	0	0.002	-0.005	-0.003	-0.002	t	13233	ELQGDRRCQSQLERA	Homo sapiens
1	1.00	1	1	-0.02	-0.022	-0.03	-0.021	-0.02	t	13425	EMVIITFKSG	Mus musculus
1	1.00	1	1	-0.02	-0.022	-0.03	-0.021	-0.02	t	13425	EMVIITFKSG	Mus musculus
1	1.00	1	1	0.01	0.01	0.01	0.01	0.01	t	135959	LTRAYAKDVKFG	Homo sapiens
1	1.00	1	1	-0.03	-0.03	-0.03	-0.03	-0.03	t	149067	LNFDPPS	Sus scrofa
1	1.00	1	1	0.02	0.022	0.016	0.021	0.02	t	137919	LGQNVLSA	Homo sapiens
1	1.00	1	1	0.01	0.009	0.007	0.01	0.01	t	28290	IRFKDDSSYEEVN	Mus musculus
1	1.00	1	1	0.01	0.01	0.01	0.01	0.009	t	58127	SGLSSLFTPGPKQQI	Mus musculus
1	1.00	1	1	0	-0.002	0	0.003	0	t	137395	EESEDEKRRWGQRDN	Mus musculus
1	1.00	1	1	0	-0.002	0	0.003	0	t	148758	QEQKQQLQQQ	Mus musculus
1	1.00	1	1	0	-0.004	0	0.003	0	t	105851	GTAPFEIHANRIVGFFSKII	Oryctolagus cuniculus

1	1.00	1	1	0	-0.004	0	0.003	0	t	105684	ASFDKVKGDPVGILYAVFKA	Oryctolagus cuniculus
1	1.00	1	1	0	-0.004	0	0.003	0	t	105814	GELPNIEADVNTFVASHKPR	Oryctolagus cuniculus
1	1.00	1	1	0	-0.004	0	0.003	0	t	105718	DPSIMAKFTQFAGKDLESIK	Oryctolagus cuniculus
1	1.00	1	1	0	-0.004	0	0.003	0	t	105942	LSADQISTVQASFDKVKGD	Oryctolagus cuniculus
1	1.00	1	1	0	-0.002	0	0.003	0	t	98155	KEEPTAAP	Mus musculus
1	1.00	1	1	0	-0.004	0	0.003	0	t	111752	SAAGDWIRRNVVAYW	Oryctolagus cuniculus
1	1.00	1	1	0	-0.002	0	0.003	0	t	98186	KTEEPAAP	Mus musculus
1	1.00	1	1	0	-0.004	0	0.003	0	t	105854	GVTHDQLNNFRAGFVSYMKA	Oryctolagus cuniculus
1	1.00	1	1	0	-0.004	0	0.003	0	t	95268	CAQVYNKLGMYP	Oryctolagus cuniculus
1	1.00	1	1	0	-0.004	0	0.003	0	t	95201	AASAYCSTWD	Oryctolagus cuniculus
1	1.00	1	1	0	-0.002	0	0.003	0	t	65565	TPEVDDEALEK	Mus musculus
1	1.00	1	1	0	-0.002	0	0.003	0	t	72178	VYVEELKPTPEGDLEILLQK	Mus musculus
1	1.00	1	1	0	-0.004	0	0.003	0	t	95686	PSLKFILNTVNG	Oryctolagus cuniculus

1	1.00	1	1	0	-0.004	0	0.003	0	t	95987	VTFTEVGY TRAE	Oryctolagus cuniculus
1	1.00	1	1	0	-0.004	0	0.003	0	t	64391	TINGLART TADRQTA	Oryctolagus cuniculus
1	1.00	1	1	0	-0.004	0	0.003	0	t	70153	VNPNNAA AL	Oryctolagus cuniculus
1	1.00	1	1	0	-0.004	0	0.003	0	t	96017	YHLYNSQD HG	Oryctolagus cuniculus
1	1.00	1	1	0	-0.004	0	0.003	0	t	95752	RAGFITELS EFI	Oryctolagus cuniculus
1	1.00	1	1	0	-0.004	0	0.003	0	t	70370	VPLYNRFS YIPNGALK FVD	Oryctolagus cuniculus
1	1.00	1	1	0	-0.002	0	0.003	0	t	62912	TALKKAITA MSQAQKA AKPA	Mus musculus
1	1.00	1	1	0	-0.002	0	0.003	0	t	74689	YLGYLEQL LRLKKYKV PQLE	Mus musculus
1	1.00	1	1	0	-0.004	0	0.003	0	t	95383	FILNTVNG TTRT	Oryctolagus cuniculus
1	1.00	1	1	0	-0.004	0	0.003	0	t	95696	PVSQFHIQ GQVY	Oryctolagus cuniculus
1	1.00	1	1	0	-0.004	0	0.003	0	t	66759	TTPDRQA AC	Oryctolagus cuniculus
1	1.00	1	1	0	-0.004	0	0.003	0	t	96025	YSMLVER DHKNE	Oryctolagus cuniculus
1	1.00	1	1	0	-0.004	0	0.003	0	t	76396	YVRGGGA VP	Oryctolagus cuniculus

1	1.00	1	1	0	-0.002	0	0.003	0	t	62912	TALKKAITA MSQAQKA AKPA	Mus musculu s
1	1.00	1	1	0	-0.004	0	0.003	0	t	95334	EDVPQPP VSQFH	Oryctola gus cuniculu s
1	1.00	1	1	0	-0.004	0	0.003	0	t	74075	YGRDPYSP SQDPYSP	Oryctola gus cuniculu s
1	1.00	1	1	0	-0.004	0	0.003	0	t	95216	AEGLYSML VERD	Oryctola gus cuniculu s
1	1.00	1	1	0	-0.004	0	0.003	0	t	95354	ELSEFIPGA SLR	Oryctola gus cuniculu s
1	1.00	1	1	0	-0.004	0	0.003	0	t	95728	QPPVSQF HIQGQ	Oryctola gus cuniculu s
1	1.00	1	1	0	-0.002	0	0.003	0	t	69271	VKMAETC PIFYDVF	Mus musculu s
1	1.00	1	1	0	-0.004	0	0.003	0	t	95417	GLYSMLVE RDHK	Oryctola gus cuniculu s
1	1.00	1	1	0	-0.004	0	0.003	0	t	95480	ITELSEFIP GAS	Oryctola gus cuniculu s
1	1.00	1	1	0	-0.002	0	0.003	0	t	60722	SRLGRSSA WV	Mus musculu s
1	1.00	1	1	0	-0.004	0	0.003	0	t	95979	VPQPPVS QFHIQ	Oryctola gus cuniculu s
1	1.00	1	1	0	-0.002	0	0.003	0	t	70444	VPQLEIVP NSAEERLH SMKE	Mus musculu s
1	1.00	1	1	0	-0.002	0	0.003	0	t	62912	TALKKAITA MSQAQKA AKPA	Mus musculu s
1	1.00	1	1	0	-0.004	0	0.003	0	t	95624	NLCCSQW GWC	Oryctola gus cuniculu s

1	1.00	1	1	0	-0.004	0	0.003	0	t	95406	GFITELSEFIPG	Oryctolagus cuniculus
1	1.00	1	1	0	-0.002	0	0.003	0	t	68322	VEQKHIQKEDVPSERYLGYL	Mus musculus
1	1.00	1	1	0	-0.004	0	0.003	0	t	95263	ATYHLYNSQD	Oryctolagus cuniculus
1	1.00	1	1	0	-0.004	0	0.003	0	t	95583	LYNSQDHGWD	Oryctolagus cuniculus
1	1.00	1	1	0	-0.004	0	0.003	0	t	95272	CCSQWGWCGS	Oryctolagus cuniculus
1	1.00	1	1	0	-0.004	0	0.003	0	t	67324	TYDNECLLCAHKVE	Oryctolagus cuniculus
1	1.00	1	1	0	-0.002	0	0.003	0	t	64783	TLEVHGVKPAAEEVKATPAG	Mus musculus
1	1.00	1	1	0	-0.002	0	0.003	0	t	64783	TLEVHGVKPAAEEVKATPAG	Mus musculus
1	1.00	1	1	0	-0.004	0	0.003	0	t	95370	EVGYTRAEGLYS	Oryctolagus cuniculus
1	1.00	1	1	0	-0.002	0	0.003	0	t	55187	RPKHPIKHQGLPQEVLNENL	Mus musculus
1	1.00	1	1	0	-0.004	0	0.003	0	t	50172	PYSPSQDPYSPSQDP	Oryctolagus cuniculus
1	1.00	1	1	0	-0.002	0	0.003	0	t	50795	QFKTTDVLWFNFTTG	Mus musculus
1	1.00	1	1	0	-0.002	0	0.003	0	t	53247	RAYALWSARQQFKTT	Mus musculus

1	1.00	1	1	0	-0.002	0	0.003	0	t	47669	PGGPDRFTLLTPGSH	Mus musculus
1	1.00	1	1	0	-0.002	0	0.003	0	t	37165	LLDKIYTSPLC	Mus musculus
1	1.00	1	1	0	-0.004	0	0.003	0	t	38505	LPGKCGVNVPYKIST	Oryctolagus cuniculus
1	1.00	1	1	0	-0.002	0	0.003	0	t	52106	QQPQQSFPQQ	Mus musculus
1	1.00	1	1	0	-0.004	0	0.003	0	t	53247	RAYALWSARQQFKTT	Oryctolagus cuniculus
1	1.00	1	1	0	-0.002	0	0.003	0	t	50795	QFKTTDVLWFNFTTG	Mus musculus
1	1.00	1	1	0	-0.004	0	0.003	0	t	48680	PNNAAGLPGKCGVNV	Oryctolagus cuniculus
1	1.00	1	1	0	-0.004	0	0.003	0	t	56195	RTTPDRQAA	Oryctolagus cuniculus
1	1.00	1	1	0	-0.002	0	0.003	0	t	52082	QQPFP	Mus musculus
1	1.00	1	1	0	-0.004	0	0.003	0	t	60267	SPSQDPYSPSQDPDR	Oryctolagus cuniculus
1	1.00	1	1	0	-0.002	0	0.003	0	t	41204	MCQCVQKYGTEFCKKRLA	Mus musculus
1	1.00	1	1	0	-0.002	0	0.003	0	t	38208	LNENLLRFFVAPFPQVFGKE	Mus musculus
1	1.00	1	1	0	-0.004	0	0.003	0	t	50121	PYKISTSTNCATVK	Oryctolagus cuniculus

1	1.00	1	1	0	-0.002	0	0.003	0	t	50934	QGPHVISRIYQTAT	Mus musculus
1	1.00	1	1	0	-0.004	0	0.003	0	t	50846	QGDRRCQSQLERANL	Oryctolagus cuniculus
1	1.00	1	1	0	-0.002	0	0.003	0	t	60374	SQAQKAAKPAAAATGTATAA	Mus musculus
1	1.00	1	1	0	-0.004	0	0.003	0	t	60414	SQDPYSPSQDPDRRD	Oryctolagus cuniculus
1	1.00	1	1	0	-0.004	0	0.003	0	t	50934	QGPHVISRIYQTAT	Oryctolagus cuniculus
1	1.00	1	1	0	-0.004	0	0.003	0	t	52194	QRDEDSYGRDPYSPS	Oryctolagus cuniculus
1	1.00	1	1	0	-0.004	0	0.003	0	t	59387	SLPGQTKILAKVFYGEN	Oryctolagus cuniculus
1	1.00	1	1	0	-0.004	0	0.003	0	t	53409	RDPYSPSQDPYSPSQ	Oryctolagus cuniculus
1	1.00	1	1	0	-0.004	0	0.003	0	t	56185	RTTADRQTA	Oryctolagus cuniculus
1	1.00	1	1	0	-0.002	0	0.003	0	t	52600	QTQQPQQPFP	Mus musculus
1	1.00	1	1	0	-0.004	0	0.003	0	t	47669	PGGPDRFTLLTPGSH	Oryctolagus cuniculus
1	1.00	1	1	0	-0.004	0	0.003	0	t	55889	RSLPPIVKDASIQVVSAIR	Oryctolagus cuniculus
1	1.00	1	1	0	-0.004	0	0.003	0	t	47770	PGVDIIEGPVKNV	Oryctolagus cuniculus

1	1.00	1	1	0	-0.004	0	0.003	0	t	58703	SISGVNPNNAAGLPG	Oryctolagus cuniculus
1	1.00	1	1	0	-0.002	0	0.003	0	t	60374	SQAQKAAKPAAAATGTATAA	Mus musculus
1	1.00	1	1	0	-0.004	0	0.003	0	t	47024	PCIGYVRSGGAVPPA	Oryctolagus cuniculus
1	1.00	1	1	0	-0.002	0	0.003	0	t	47669	PGGPDRFTLLTPGSH	Mus musculus
1	1.00	1	1	0	-0.004	0	0.003	0	t	20770	GLARTTADRQTACNC	Oryctolagus cuniculus
1	1.00	1	1	0	-0.004	0	0.003	0	t	23169	GVTHDQLNNFR	Oryctolagus cuniculus
1	1.00	1	1	0	-0.021	0	0.003	0	t	21851	GQCKWVNGREICNDRDRPTA	Mus musculus
1	1.00	1	1	0	-0.002	0	0.003	0	t	28676	ISQAVHAAHAEINEAGR	Mus musculus
1	1.00	1	1	0	-0.004	0	0.003	0	t	22771	GTNISKEHDGECKE	Oryctolagus cuniculus
1	1.00	1	1	0	-0.004	0	0.003	0	t	17943	FTEVGYTRAEGL	Oryctolagus cuniculus
1	1.00	1	1	0	-0.004	0	0.003	0	t	17795	FSNVYLFAKDKSGPLQPGV	Oryctolagus cuniculus
1	1.00	1	1	0	-0.002	0	0.003	0	t	14906	EVWREEAYHACDIKD	Mus musculus
1	1.00	1	1	0	-0.004	0	0.003	0	t	36137	LGGWKLQSDPRAYAL	Oryctolagus cuniculus

1	1.00	1	1	0	-0.002	0	0.003	0	t	23169	GVTHDQLNNFR	Mus musculus
1	1.00	1	1	0	-0.004	0	0.003	0	t	28676	ISQAVHAAHAEINEAGR	Oryctolagus cuniculus
1	1.00	1	1	0	-0.004	0	0.003	0	t	14905	EVWREEAYHAADIKD	Oryctolagus cuniculus
1	1.00	1	1	0	-0.002	0	0.003	0	t	12408	EICPAVKRDVDLFLTG	Mus musculus
1	1.00	1	1	0	-0.002	0	0.003	0	t	34335	KWVNGREI	Mus musculus
1	1.00	1	1	0	-0.004	0	0.003	0	t	23077	GVNPNNAAGLPGKCG	Oryctolagus cuniculus
1	1.00	1	1	0	-0.002	0	0.003	0	t	26167	IFSQNMNIKLKMP	Mus musculus
1	1.00	1	1	0	-0.002	0	0.003	0	t	34335	KWVNGREI	Mus musculus
1	1.00	1	1	0	-0.004	0	0.003	0	t	23494	GYVRSGGAVPPACCN	Oryctolagus cuniculus
1	1.00	1	1	0	-0.002	0	0.003	0	t	36137	LGGWKLQSDPRAYAL	Mus musculus
1	1.00	1	1	0	-0.002	0	0.003	0	t	20726	GKVYLVGGPELGGWK	Mus musculus
1	1.00	1	1	0	-0.004	0	0.003	0	t	20726	GKVYLVGGPELGGWK	Oryctolagus cuniculus
1	1.00	1	1	0	-0.002	0	0.003	0	t	31580	KKIQDCYVENGLIS	Mus musculus

1	1.00	1	1	0	-0.021	0	0.003	0	t	34335	KWVNGRE I	Mus musculu s
1	1.00	1	1	0	-0.002	0	0.003	0	t	24814	HSMKEGI HAQQKEP MIGVNQ	Mus musculu s
1	1.00	1	1	0	-0.002	0	0.003	0	t	14905	EVWREEA YHAADIKD	Mus musculu s
1	1.00	1	1	0	-0.002	0	0.003	0	t	26167	IFSQNMNI KLKMP	Mus musculu s
1	1.00	1	1	0	-0.002	0	0.003	0	t	11035	EAESISSSE EIVPNSVE QKH	Mus musculu s
1	1.00	1	1	0	-0.004	0	0.003	0	t	22372	GSIGAAS MEF	Oryctola gus cuniculu s
1	1.00	1	1	0	-0.004	0	0.003	0	t	13233	ELQGDRR CQSQLERA	Oryctola gus cuniculu s
1	1.00	1	1	0	-0.002	0	0.003	0	t	21851	GQCKWV NGREICND RDRPTA	Mus musculu s
1	1.00	1	1	0	-0.004	0	0.003	0	t	30872	KFVDSTVV ASVTIIDRS LP	Oryctola gus cuniculu s
1	1.00	1	1	0	-0.004	0	0.003	0	t	22621	GTDGVTYT NDCLLC	Oryctola gus cuniculu s
1	1.00	1	1	0	-0.002	0	0.003	0	t	26167	IFSQNMNI KLKMP	Mus musculu s
1	1.00	1	1	0	-0.002	0	0.003	0	t	346	AAPANDK FTVFEAAF NDAIK	Mus musculu s
1	1.00	1	1	0	-0.002	0	0.003	0	t	481	AAVDSSKA ALTSKLDA AYKL	Mus musculu s
1	1.00	1	1	0	-0.002	0	0.003	0	t	6895	CQQTLSVR ALC	Mus musculu s
1	1.00	1	1	0	-0.002	0	0.003	0	t	481	AAVDSSKA ALTSKLDA AYKL	Mus musculu s

1	1.00	1	1	0	-0.004	0	0.003	0	t	751	ADPVTTEGDYVVKISEFYGR	Oryctolagus cuniculus
1	1.00	1	1	0	-0.004	0	0.003	0	t	10277	DSYGRDPYSPSQDPY	Oryctolagus cuniculus
1	1.00	1	1	0	-0.002	0	0.003	0	t	751	ADPVTTEGDYVVKISEFYGR	Mus musculus
1	1.00	1	1	0	-0.002	0	0.003	0	t	346	AAPANDKFTVFEAAFNDAIK	Mus musculus
1	1.00	1	1	0	-0.004	0	0.003	0	t	7947	DEDSYGRDPYSPSQD	Oryctolagus cuniculus
1	1.00	1	1	0	-0.002	0	0.003	0	t	895	AEEVKATPAGELQVIDKVDA	Mus musculus
1	1.00	1	1	0	-0.002	0	0.003	0	t	4792	ASTGGAYQSYKFIPALEAAV	Mus musculus
1	1.00	1	1	0	-0.002	0	0.003	0	t	346	AAPANDKFTVFEAAFNDAIK	Mus musculus
1	1.00	1	1	0	-0.002	0	0.003	0	t	6986	CSEYREPLLC	Mus musculus
1	1.00	1	1	0	-0.002	0	0.003	0	t	4792	ASTGGAYQSYKFIPALEAAV	Mus musculus
1	1.00	1	1	0	-0.002	0	0.003	0	t	895	AEEVKATPAGELQVIDKVDA	Mus musculus
1	1.00	1	1	0	-0.002	0	0.003	0	t	3493	APAVKYTVFETALKKAITAM	Mus musculus
1	1.00	1	1	0	-0.002	0	0.003	0	t	895	AEEVKATPAGELQVIDKVDA	Mus musculus
1	1.00	1	1	0	-0.004	0	0.003	0	t	1206	AEVDCSRFPNATDK	Oryctolagus cuniculus
1	1.00	1	1	0	-0.002	0	0.003	0	t	3493	APAVKYTVFETALKKAITAM	Mus musculus

1	1.00	1	1	0	-0.002	0	0.003	0	t	8756	DIPNPIGSENSEKTTMPLW	Mus musculus
1	1.00	1	1	0	-0.004	0	0.003	0	t	10041	DRRCQSQLERANLRP	Oryctolagus cuniculus
1	1.00	1	1	0	-0.004	0	0.003	0	t	4923	ATDKEGKDVLVCNK	Oryctolagus cuniculus
1	1.00	1	1	0.01	0.017	0.018	0.01	0.009	t	110962	PIDVVDP	Mus musculus
1	1.00	1	1	0	-0.003	-0.004	0	0	t	122941	VVKLLRDPIYVEV	Oryctolagus cuniculus
1	1.00	1	1	-0.01	-0.012	-0.01	-0.013	-0.009	t	96526	LAANNPA	Mus musculus
1	1.00	1	1	0.02	0.02	0.02	0.02	0.021	t	122569	SFQDENFVFEEF	Rattus norvegicus
1	1.00	1	1	-0.03	-0.033	-0.036	-0.03	-0.03	t	105895	KDLESIKG	Oryctolagus cuniculus
1	1.00	1	1	0.01	0.008	0.015	0.009	0.01	t	24950	HTNLPQALWGMQ	Mus musculus
1	1.00	1	1	0	0	0	0	0	t	48598	PNANPNA	Mus musculus
1	1.00	1	1	0	0.01	0	-0.001	0	t	68023	VDMIAGAHWG	Pan troglodytes
1	1.00	1	1	0.02	0.018	0.022	0.021	0.021	t	78854	TKDTNNNLKG	Mus musculus
1	1.00	1	1	-0.03	-0.028	-0.03	-0.023	-0.03	t	137399	FCGLPRLE	Homo sapiens
1	1.00	1	1	0	0	0	0.004	0.001	t	135930	GDKVSFFCKNKEKKC	Homo sapiens

1	1.00	1	1	0	-0.003	0.008	0	0	t	125211	QHWSYGL RPG	Canis lupus
1	1.00	1	1	0	-0.003	-0.002	0	0	t	135835	CWAFSGV AATESAY	Oryctola gus cuniculu s
1	1.00	1	1	0.03	0.03	0.023	0.03	0.03	t	131652	SFDSSNPE YFDGYWG YS	Oryctola gus cuniculu s
1	1.00	1	1	0.03	0.028	0.026	0.027	0.03	t	1476	AGDQSYFL	Mus musculu s
1	1.00	1	1	-0.06	-0.06	-0.062	-0.06	-0.062	t	45610	NQQIAGIG AALIE	Oryctola gus cuniculu s
1	1.00	1	1	0.02	0.02	0.02	0.02	0.02	t	114920	PVCGTDG VTY	Homo sapiens
1	1.00	1	1	0	0	0	0	0	t	127961	ELLADVQF ALGLPFYP AYEG	Homo sapiens
1	1.00	1	1	-0.02	-0.021	-0.035	-0.02	-0.018	t	13897	EQPFPSRL GPLVTLE	Bos taurus
1	1.00	1	1	0	-0.002	0	-0.007	-0.005	t	119673	WNPDDYG GVK	Rattus norvegic us
1	1.00	1	1	0.03	0.032	0.03	0.031	0.03	t	34935	LAQMYQA NDNSLED V	Homo sapiens
1	1.00	1	1	0.05	0.05	0.047	0.05	0.052	t	20047	GGTEIKYN GE	Mus musculu s
1	1.00	1	1	0.01	0.01	0.004	0.01	0.01	t	74942	YLTIGKNP DFKSTFAV LWEP	Oryctola gus cuniculu s
1	1.00	1	1	0	0.004	-0.005	0.001	0	t	113400	FKLGGRDS R	Homo sapiens

1	1.00	1	1	0.03	0.03	0.032	0.027	0.03	t	9118	DLIAYKQ	Homo sapiens
1	1.00	1	1	0.02	0.025	0.034	0.02	0.018	t	132850	KLDLYLSYPQPY	Mus musculus
1	1.00	1	1	0.01	0.016	0	0.01	0.011	t	139100	ITGRLAALNAYVSQTLTQY	Felis catus
1	1.00	1	1	0	0	0	0.001	-0.001	t	82078	FHQNGQVTEVTT	Homo sapiens
1	1.00	1	1	0	0	0	0.001	-0.001	t	70817	VRRRVNAANKKPEDLTLNDL	Homo sapiens
1	1.00	1	1	0	0	0	0.001	-0.001	t	46792	PAFGANSNNPDWDFNP	Homo sapiens
1	1.00	1	1	0	0	0	0.001	-0.001	t	18933	GDAKKKKDGKKAEPKNPREN	Homo sapiens
1	1.00	1	1	-0.04	-0.04	-0.038	-0.04	-0.04	t	125011	DFHFEVFNFVP	Mus musculus
1	1.00	1	1	0.01	0.007	0.003	0.01	0.012	t	118385	GMTSNGFIADPDDRGML	Oryctolagus cuniculus
1	1.00	1	1	-0.01	-0.014	-0.013	-0.011	-0.01	t	37755	LLRRQVNEVA	Oryctolagus cuniculus
1	1.00	1	1	0.01	0.01	0.01	0.01	0.012	t	9941	DRADGQPAG	Mus musculus
1	1.00	1	1	-0.03	-0.03	-0.03	-0.027	-0.03	t	118540	NPVEIRRGVMLAV	Homo sapiens
1	1.00	1	1	0.02	0.017	0.018	0.02	0.02	t	105867	HTDFAGAE	Oryctolagus cuniculus
1	1.00	1	1	0.01	0.01	0.01	0.01	0.01	t	23193	GVTYEIDLTNKNAAKL	Homo sapiens

1	1.00	1	1	0.02	0.02	0.015	0.02	0.02	t	120403	AMGSKGN RTDSC	Oryctola gus cuniculu s
1	1.00	1	1	0.02	0.023	0.015	0.02	0.02	t	120403	AMGSKGN RTDSC	Mus musculu s
1	1.00	1	1	0.02	0.022	0.032	0.027	0.021	t	104569	MASPGSG FWSFGSE DGS	Homo sapiens
1	1.00	1	1	0.01	0.012	0.01	0.01	0.01	t	117924	IIAKMKGT F	Homo sapiens
1	1.00	1	1	0	-0.002	0.005	-0.001	-0.002	t	24249	HLLRLSEI	Mus musculu s
1	1.00	1	1	0	-0.001	-0.006	-0.001	0	t	25786	IEEPSGQT ADWYTI	Myodes glareolu s
1	1.00	1	1	0.07	0.07	0.07	0.07	0.07	t	115514	TLEDSPEVI ESPPEINTV QV	Homo sapiens
1	1.00	1	1	0.03	0.03	0.03	0.03	0.03	t	112119	FYNTVLK	Homo sapiens
1	1.00	1	1	0.03	0.03	0.03	0.03	0.03	t	104870	LATYHLYN SQ	Homo sapiens
1	1.00	1	1	0.03	0.03	0.03	0.03	0.03	t	79254	ICADDEE WYSAMCA	Mus musculu s
1	1.00	1	1	-0.03	-0.03	-0.03	-0.03	-0.03	t	99571	QGRGYFG LIFPGCPR	Homo sapiens
1	1.00	1	1	-0.01	-0.01	-0.005	-0.013	-0.012	t	66979	TVDSNNLP ILEF	Homo sapiens
1	1.00	1	1	-0.01	-0.008	-0.005	-0.009	-0.01	t	19491	GFADLMG YIP	Homo sapiens
1	1.00	1	1	0.02	0.023	0.02	0.02	0.02	t	110275	GPVGPSD PSIVSLVEE TSFI	Mus musculu s
1	1.00	1	1	0.02	0.02	0.02	0.02	0.02	t	110275	GPVGPSD PSIVSLVEE TSFI	Mus musculu s

1	1.00	1	1	0	0.002	0	0.007	0	t	108072	PPGPTGASG	Homo sapiens
1	1.00	1	1	0	0.004	0	0.007	0	t	108072	PPGPTGASG	Homo sapiens
1	1.00	1	1	0	-0.006	0	0	0	t	42795	MTEQQWNFAGIEAAAS	Homo sapiens
1	1.00	1	1	0.01	0.01	0.01	0.01	0.009	t	55457	RQNKPPNKPNND	Mus musculus
1	1.00	1	1	-0.01	-0.007	-0.01	-0.01	-0.01	t	53634	RESQAYYQR	Mus musculus
1	1.00	1	1	0.02	0.018	0.022	0.019	0.02	t	75151	YNGEEYLILS	Mus musculus
1	1.00	1	1	-0.01	-0.006	-0.005	-0.009	-0.01	t	66105	TRTFTSL	Homo sapiens
1	1.00	1	1	0	0.004	0	0.001	0	t	141566	SSIVIPAAVGIKLNVTEDA	Homo sapiens
1	1.00	1	1	0	0	0	0	0	t	112841	SSKRGRQTPVLYAMLDH	Rattus norvegicus
1	1.00	1	1	-0.03	-0.03	-0.026	-0.03	-0.032	t	105940	LRYQQQSRRRSLPYS	Homo sapiens
1	1.00	1	1	0.04	0.04	0.043	0.04	0.04	t	125250	SDEFNPCEDIMGYK	Mus musculus
1	1.00	1	1	0.03	0.03	0.013	0.03	0.032	t	96098	ANEEIRKRNA	Homo sapiens(human)
1	1.00	1	1	0.04	0.042	0.04	0.037	0.041	t	5990	CAISGGYPVC	Homo sapiens
1	1.00	1	1	-0.01	-0.007	-0.01	-0.01	-0.011	t	21617	GPFFLSPTS	Mus musculus

1	1.00	1	1	0.03	0.026	0.03	0.033	0.033	t	115328	KTSASIGSL CADA	Oryctolagus cuniculus
1	1.00	1	1	0	0	0	0	-0.001	t	134411	IPP	Mus musculus
1	1.00	1	1	0	0.004	-0.002	-0.003	-0.002	t	100398	PTTPATPT PTPK	Homo sapiens
1	1.00	1	1	-0.01	-0.017	-0.017	-0.003	-0.008	t	156956	SGIGTLDG FRHRHPD	Homo sapiens
1	1.00	1	1	0	0.004	-0.002	-0.003	0	t	21860	GQFEHFRE CC	Homo sapiens
1	1.00	1	1	-0.01	-0.014	-0.003	-0.01	-0.01	t	10860	DYDGYGW RE	Mus musculus
1	1.00	1	1	-0.01	-0.01	-0.01	-0.01	-0.012	t	49082	PQQPFPQ PQQ	Mus musculus
1	1.00	1	1	0.08	0.08	0.08	0.08	0.08	t	79504	ADCTTPT	Oryctolagus cuniculus
1	1.00	1	1	0.04	0.04	0.04	0.04	0.04	t	153349	ACTDFEG MLCGGGS	Mus musculus
1	1.00	1	1	0.04	0.04	0.04	0.04	0.04	t	115536	VEELKPTP EGDLEILLQ KWE	Homo sapiens
1	1.00	1	1	0.02	0.02	0.02	0.02	0.02	t	103431	MWELYLA YSEAGFRS	Homo sapiens
1	1.00	1	1	-0.02	-0.016	-0.02	-0.019	-0.019	t	96102	APPLLPH	Homo sapiens
1	1.00	1	1	-0.02	-0.018	-0.02	-0.019	-0.019	t	96102	APPLLPH	Homo sapiens
1	1.00	1	1	-0.02	-0.02	-0.02	-0.02	-0.02	t	158596	KFGTALW	Homo sapiens

1	1.00	1	1	0	-0.002	0	0.003	0	t	38304	LNPKTNK WEDK	Mus musculu s
1	1.00	1	1	0	0	0.001	0	0	t	73635	YEDRYY	Mus musculu s
1	1.00	1	1	0	0	0.001	0	0	t	73635	YEDRYY	Mus musculu s
1	1.00	1	1	0.04	0.04	0.04	0.042	0.042	t	27494	IMGGECPT AEDMVN	Homo sapiens
1	1.00	1	1	0	0.002	-0.002	-0.001	0	t	130990	NVLRGMG GAF	Homo sapiens
1	1.00	1	1	0	0.004	0	0	0	t	25645	IDPRGKM V	MD
1	1.00	1	1	0.03	0.03	0.03	0.03	0.03	t	79969	PKQLTFEG K	Mus musculu s
1	1.00	1	1	0.05	0.05	0.048	0.05	0.05	t	20197	GHWMDA TFNTKTA	Homo sapiens
1	1.00	1	1	-0.02	-0.022	-0.021	-0.019	-0.02	t	133909	PEPAKKVP APK	Mus musculu s
1	1.00	1	1	-0.02	-0.024	-0.021	-0.019	-0.02	t	133909	PEPAKKVP APK	Oryctola gus cuniculu s
1	1.00	1	1	0.05	0.053	0.051	0.051	0.048	t	99741	TWVNTIQ TNGLLNEA	Equus caballus
1	1.00	1	1	0.07	0.07	0.07	0.07	0.07	t	115745	FTESQSLTL T	Homo sapiens
1	1.00	1	1	0	0.002	-0.007	-0.002	-0.002	t	106822	SDPAHKA RLERP	Homo sapiens
1	1.00	1	1	0.05	0.05	0.05	0.05	0.05	t	100291	LSTEEKEFL K	Mus musculu s
1	1.00	1	1	0.03	0.03	0.03	0.03	0.03	t	77781	GLVGRPKS KL	Homo sapiens
1	1.00	1	1	0.01	0.01	0.01	0.01	0.01	t	99122	CESRCTKL EYDPRCV	Homo sapiens

1	1.00	1	1	0.01	0.01	0.01	0.01	0.01	t	79828	FLGPKQLTFEGK	Mus musculus
1	1.00	1	1	0.01	0.01	0.01	0.01	0.01	t	92477	DQSLSSLQFA	Ovis aries
1	1.00	1	1	-0.05	-0.05	-0.05	-0.05	-0.05	t	139892	SKLPLAL	Ovis aries
1	1.00	1	1	0.01	0.002	0.008	0.01	0.01	t	95744	QTLPPSVP	Bos taurus
1	1.00	1	1	0	0.002	-0.012	-0.003	0.001	t	51847	QPGVDIIEGPVKNVAVPLY	Homo sapiens
1	1.00	1	1	0	0.003	-0.007	0	0	t	110810	DNASLARLDLERKVESLQEE	Mus musculus
1	1.00	1	1	0.02	0.017	0.02	0.02	0.02	t	23587	HCDGFQNE	Oryctolagus cuniculus
1	1.00	1	1	0	0.003	0	0	0	t	141074	RHLEFEARTLSP	Mus musculus
1	1.00	1	1	0	0	0	0	0	t	60333	SPWAPTAPLPGDMNP	Homo sapiens
1	1.00	1	1	0.01	0.009	0.003	0.01	0.012	t	56768	SAEYEKSVNKYLKTIMPF	Equus caballus
1	1.00	1	1	-0.04	-0.042	-0.045	-0.047	-0.043	t	124008	DGVVLVD	Mus musculus
1	1.00	1	1	-0.03	-0.029	-0.031	-0.027	-0.031	t	99405	KVMIGKPKAQNIRQG	Equus caballus
1	1.00	1	1	0.04	0.04	0.04	0.04	0.04	t	142427	KAEKDAEAAQ	Homo sapiens
1	1.00	1	1	0	0	0	0	-0.001	t	144742	PFYGYRE	Mus musculus

1	1.00	1	1	0	0	0	0	-0.001	t	150858	ISSLYNLVKSS	Mus musculus
1	1.00	1	1	0	0	0	0	-0.001	t	141786	YGEESSRRGDLAALARRV	Mus musculus
1	1.00	1	1	0	0	0	0	-0.001	t	138194	IIPKSSWS	Homo sapiens
1	1.00	1	1	0	0	0	0	-0.001	t	141963	MVDRGWG	Mus musculus
1	1.00	1	1	0	0	0	0	-0.001	t	139029	DLQVLT	Mus musculus
1	1.00	1	1	0	0	0	0	-0.001	t	134860	AGPKAQVEAIQKY	Mus musculus
1	1.00	1	1	0	0	0	0	-0.001	t	142913	MVLNEGTSGTA	Oryctolagus cuniculus
1	1.00	1	1	0	0	0	0	-0.001	t	141523	ALEPPHNLPVP	Mus musculus
1	1.00	1	1	0	0	0	0	-0.001	t	150204	FHSVVHTLLEWLMEKELMV	Mus musculus
1	1.00	1	1	0	0	0	0	-0.001	t	125004	DAPDTDWCDSIP	Mus musculus
1	1.00	1	1	0	0	0	0	-0.001	t	125021	DWC	Mus musculus
1	1.00	1	1	0	0	0	0	-0.001	t	122292	CTYILDPEKLTLRVPYKA	Oryctolagus cuniculus
1	1.00	1	1	0	0	0	0	-0.001	t	122291	CTYILDPEKLTLRATYDN	Oryctolagus cuniculus
1	1.00	1	1	0	0	0	0	-0.001	t	124069	ELQIAKDERYGS	Oryctolagus cuniculus

1	1.00	1	1	0	0	0	0	-0.001	t	125608	YYKKHIS	Mus musculus
1	1.00	1	1	0	0	0	0	-0.001	t	122083	DPNIKLVL DDCWATS	Oryctolagus cuniculus
1	1.00	1	1	0	0	0	0	-0.001	t	122202	TSPPLLWD SVHL	Mus musculus
1	1.00	1	1	0	0	0	0	-0.001	t	125454	KDYKTMV KFCN	Mus musculus
1	1.00	1	1	0	0	0	0	-0.001	t	122083	DPNIKLVL DDCWATS	Mus musculus
1	1.00	1	1	0	0	0	0	-0.001	t	124232	GSITRDSIF RLR	Oryctolagus cuniculus
1	1.00	1	1	0	0	0	0	-0.001	t	123849	WQDE	Mus musculus
1	1.00	1	1	0	0	0	0	-0.001	t	124713	SGEQPSDE QPSGEHG	Mus musculus
1	1.00	1	1	0	0	0	0	-0.001	t	122094	FLALDVPTI GLC	Mus musculus
1	1.00	1	1	0	0	0	0	-0.001	t	103998	IVKWDRD M	Mus musculus
1	1.00	1	1	0	0	0	0	-0.001	t	102510	HHQKLVFF AEDVGSN K	Mus musculus
1	1.00	1	1	0	0	0	0	-0.001	t	107233	RSFTLASSE TG	Mus musculus
1	1.00	1	1	0	0	0	0	-0.001	t	105207	GAIIGLMV GGVVIA	Mus musculus
1	1.00	1	1	0	0	0	0	-0.001	t	104443	DAEFRHDS GYE	Homo sapiens
1	1.00	1	1	0	0	0	0	-0.001	t	112352	TGNKYPGY P	Mus musculus
1	1.00	1	1	0	0	0	0	-0.001	t	115630	NFDKLYIW G	Homo sapiens

1	1.00	1	1	0	0	0	0	-0.001	t	112316	SIKNPTNAK	Mus musculus
1	1.00	1	1	0	0	0	0	-0.001	t	106322	DAEFRHDSG	Mus musculus
1	1.00	1	1	0	0	0	0	-0.001	t	99922	DAEFRHDSGY	Mus musculus
1	1.00	1	1	0	0	0	0	-0.001	t	104443	DAEFRHDSGYE	Mus musculus
1	1.00	1	1	0	0	0	0	-0.001	t	108600	QLINTNGS	Capra hircus
1	1.00	1	1	0	0	0	0	-0.001	t	104443	DAEFRHDSGYE	Mus musculus
1	1.00	1	1	0	0	0	0	-0.001	t	99922	DAEFRHDSGY	Mus musculus
1	1.00	1	1	0	0	0	0	-0.001	t	99922	DAEFRHDSGY	Mus musculus
1	1.00	1	1	0	0	0	0	-0.001	t	106322	DAEFRHDSG	Mus musculus
1	1.00	1	1	0	0	0	0	-0.001	t	115652	SSRISIYWTIVKP	Homo sapiens
1	1.00	1	1	0	0	0	0	-0.001	t	102344	DAEFR	Mus musculus
1	1.00	1	1	0	0	0	0	-0.001	t	61800	STNS	Mus musculus
1	1.00	1	1	0	0	0	0	-0.001	t	63756	TFTNKLKEKHTDLGK	Mus musculus
1	1.00	1	1	0	0	0	0	-0.001	t	78635	EKIERNNKLKQPPPPPNPN	Mus musculus
1	1.00	1	1	0	0	0	0	-0.001	t	71284	VTEEI	Homo sapiens
1	1.00	1	1	0	0	0	0	-0.001	t	67875	VCRQGVVDRGWGNGCGLFGK	Equus caballus

1	1.00	1	1	0	0	0	0	-0.001	t	62353	SVTEEIAEEDKSVTEEIAV	Homo sapiens
1	1.00	1	1	0	0	0	0	-0.001	t	78627	DPDPPDANDPAPPNAN	Mus musculus
1	1.00	1	1	0	0	0	0	-0.001	t	68524	VFMVPQYGYL	Mus musculus
1	1.00	1	1	0	0	0	0	-0.001	t	65421	TNSGINNS	Mus musculus
1	1.00	1	1	0	0	0	0	-0.001	t	67214	TVWRNRETLMEFEEPHATKQ	Equus caballus
1	1.00	1	1	0	0	0	0	-0.001	t	97484	LVEETSFIDAGAP	Oryctolagus cuniculus
1	1.00	1	1	0	0	0	0	-0.001	t	74871	YLPVGGLRRIGG	Mus musculus
1	1.00	1	1	0	0	0	0	-0.001	t	78687	IRVRKRKGSNKKAEDTLED	Mus musculus
1	1.00	1	1	0	0	0	0	-0.001	t	61801	STNSGINN	Mus musculus
1	1.00	1	1	0	0	0	0	-0.001	t	72485	WGNGCGLFGKGSIDTCAKFA	Equus caballus
1	1.00	1	1	0	0	0	0	-0.001	t	74008	YGGL	Mus musculus
1	1.00	1	1	0	0	0	0	-0.001	t	71284	VTEEI	Oryctolagus cuniculus
1	1.00	1	1	0	0	0	0	-0.001	t	74009	YGGPANKKNAG	Mus musculus
1	1.00	1	1	0	0	0	0	-0.001	t	45420	NPNDPP	Mus musculus

1	1.00	1	1	0	0	0	0	-0.001	t	53447	REACR	Mus musculus
1	1.00	1	1	0	0	0	0	-0.001	t	60024	SPDVDLG DISGINAS	Mus musculus
1	1.00	1	1	0	0	0	0	-0.001	t	43242	NANPNAN PNANP	Mus musculus
1	1.00	1	1	0	0	0	0	-0.001	t	39689	LSSY	Mus musculus
1	1.00	1	1	0	0	0	0	-0.001	t	46008	NSTNSGIN	Mus musculus
1	1.00	1	1	0	0	0	0	-0.001	t	43246	NANPNAN PNANPNA NP	Mus musculus
1	1.00	1	1	0	0	0	0	-0.001	t	43242	NANPNAN PNANP	Mus musculus
1	1.00	1	1	0	0	0	0	-0.001	t	59967	SNYYSDNE IEQHTAKQ PKL	Oryctolagus cuniculus
1	1.00	1	1	0	0	0	0	-0.001	t	43242	NANPNAN PNANP	Mus musculus
1	1.00	1	1	0	0	0	0	-0.001	t	45416	NPNANPN ANPNANP NENPNA	Homo sapiens
1	1.00	1	1	0	0	0	0	-0.001	t	43242	NANPNAN PNANP	Mus musculus
1	1.00	1	1	0	0	0	0	-0.001	t	43152	NAGGNAG GNAGGNA GGNAGG	Mus musculus
1	1.00	1	1	0	0	0	0	-0.001	t	43730	NEREDERT LTKEYEDIV LK	Oryctolagus cuniculus

1	1.00	1	1	0	0	0	0	-0.001	t	43248	NANPNANPNANPNANPNANP	Mus musculus
1	1.00	1	1	0	0	0	0	-0.001	t	57644	SETFTNKLKEKHTDLGKEG	Mus musculus
1	1.00	1	1	0	0	0	0	-0.001	t	56699	SAAFYYRV	Mus musculus
1	1.00	1	1	0	0	0	0	-0.001	t	60024	SPDVDLGDISGINAS	Mus musculus
1	1.00	1	1	0	0	0	0	-0.001	t	43248	NANPNANPNANPNANPNANP	Mus musculus
1	1.00	1	1	0	0	0	0	-0.001	t	50931	QGPGAPQGPGAPQGPGAP	Mus musculus
1	1.00	1	1	0	0	0	0	-0.001	t	49450	PSNQDSFQWQ	Mus musculus
1	1.00	1	1	0	0	0	0	-0.001	t	41019	MADPNRFRG	Mus musculus
1	1.00	1	1	0	0	0	0	-0.001	t	57569	SENHGNYSAQVGASQ	Mus musculus
1	1.00	1	1	0	0	0	0	-0.001	t	51775	QNTRNIVPVSIVSR	Mus musculus
1	1.00	1	1	0	0	0	0	-0.001	t	49450	PSNQDSFQWQ	Mus musculus
1	1.00	1	1	0	0	0	0	-0.001	t	51775	QNTRNIVPVSIVSR	Oryctolagus cuniculus
1	1.00	1	1	0	0	0	0	-0.001	t	37660	LLPHSNLDHILEPSIPWKSK	Mus musculus
1	1.00	1	1	0	0	0	0	-0.001	t	48690	PNPNDP	Mus musculus

1	1.00	1	1	0	0	0	0	-0.001	t	45252	NNSTNSGI	Mus musculus
1	1.00	1	1	0	0	0	0	-0.001	t	37660	LLPHSNLDHILEPSIPWKSK	Mus musculus
1	1.00	1	1	0	0	0	0	-0.001	t	40739	LYDSYAYYG	Mus musculus
1	1.00	1	1	0	0	0	0	-0.001	t	45276	NNVARCSYGADSTLGPV	Mus musculus
1	1.00	1	1	0	0	0	0	-0.001	t	48620	PNDPPP	Mus musculus
1	1.00	1	1	0	0	0	0	-0.001	t	30716	KFDWNTPD	Mus musculus
1	1.00	1	1	0	0	0	0	-0.001	t	11813	EENVEENV	Oryctolagus cuniculus
1	1.00	1	1	0	0	0	0	-0.001	t	11463	EDVNGIRKPK	Mus musculus
1	1.00	1	1	0	0	0	0	-0.001	t	24369	HNDKRADPAFVCRQGVVDRG	Equus caballus
1	1.00	1	1	0	0	0	0	-0.001	t	11813	EENVEENV	Homo sapiens
1	1.00	1	1	0	0	0	0	-0.001	t	11819	EENVEHDA	Mus musculus
1	1.00	1	1	0	0	0	0	-0.001	t	19481	GEYGEVTVDCEPRSGIDTNA	Equus caballus
1	1.00	1	1	0	0	0	0	-0.001	t	11819	EENVEHDA	Mus musculus

1	1.00	1	1	0	0	0	0	-0.001	t	11819	EENVEHDA	Mus musculus
1	1.00	1	1	0	0	0	0	-0.001	t	31105	KHFRETEV	Mus musculus
1	1.00	1	1	0	0	0	0	-0.001	t	11814	EENVEENVEENV	Oryctolagus cuniculus
1	1.00	1	1	0	0	0	0	-0.001	t	11815	EENVEENVEENVEENV	Oryctolagus cuniculus
1	1.00	1	1	0	0	0	0	-0.001	t	15205	FAKY	Mus musculus
1	1.00	1	1	0	0	0	0	-0.001	t	27675	INKRKYDSLKEKL	Homo sapiens
1	1.00	1	1	0	0	0	0	-0.001	t	32570	KNNNSTNS	Mus musculus
1	1.00	1	1	0	0	0	0	-0.001	t	10801	DWEDRYYRE	Oryctolagus cuniculus
1	1.00	1	1	0	0	0	0	-0.001	t	11819	EENVEHDA	Oryctolagus cuniculus
1	1.00	1	1	0	0	0	0	-0.001	t	31018	KGNCSGPTTIR	Oryctolagus cuniculus
1	1.00	1	1	0	0	0	0	-0.001	t	27463	IMDINKRKYDSLK	Homo sapiens
1	1.00	1	1	0	0	0	0	-0.001	t	17135	FNCLGMSNRDFLEGVSGATW	Equus caballus

1	1.00	1	1	0	0	0	0	-0.001	t	1478	AGDRAGDR	Mus musculus
1	1.00	1	1	0	0	0	0	-0.001	t	3242	ANDPPP	Mus musculus
1	1.00	1	1	0	0	0	0	-0.001	t	9723	DPPPPNPNDPPPPNPND	Mus musculus
1	1.00	1	1	0	0	0	0	-0.001	t	7491	DAEFRHDSGYEVHHQK	Mus musculus
1	1.00	1	1	0	0	0	0	-0.001	t	9516	DNNLVSGP	Mus musculus
1	1.00	1	1	0	0	0	0	-0.001	t	9516	DNNLVSGP	Mus musculus
1	1.00	1	1	0	0	0	0	-0.001	t	3044	ALYTDEDLLFDLEKQK	Oryctolagus cuniculus
1	1.00	1	1	0	0	0	0	-0.001	t	7492	DAEFRHDSGYEVHHQKL	Mus musculus
1	1.00	1	1	0	0	0	0	-0.001	t	7492	DAEFRHDSGYEVHHQKL	Mus musculus
1	1.00	1	1	0	0	0	0	-0.001	t	6402	CHPSDGKCN	Mus musculus
1	1.00	1	1	0	0	0	0	-0.001	t	3274	ANETIYNTTLKYGDV	MD
1	1.00	1	1	0	0	0	0	-0.001	t	9524	DNPASTTNKDK	Mus musculus
1	1.00	1	1	0.01	0.013	0.01	0.01	0.008	t	135458	FGSDYEDRYYR	Mus musculus
1	1.00	1	1	0.07	0.072	0.075	0.069	0.07	t	135786	VNSTEAET	Homo sapiens
1	1.00	1	1	0	0	0	0	0	t	135451	CLGKAC	Oryctolagus cuniculus

1	1.00	1	1	0.03	0.033	0.029	0.03	0.03	t	133611	IVSYLCDLAPEAPPPTLPP	Mus musculus
1	1.00	1	1	0.05	0.05	0.05	0.05	0.052	t	27555	IMSGEKPTVEDVVN	Homo sapiens
1	1.00	1	1	0.02	0.018	0.009	0.019	0.02	t	39251	LRYGNVLDVN	Mus musculus
1	1.00	1	1	0.03	0.026	0.027	0.03	0.028	t	140996	EEIDYDDTISVEMKK	Mus musculus
1	1.00	1	1	0.01	0.008	0.015	0.007	0.009	t	53894	RGKDLPVLDQLTD	Mus musculus
1	1.00	1	1	0.04	0.04	0.04	0.04	0.04	t	140596	SLDELDWDSM	Mus musculus
1	1.00	1	1	0.02	0.02	0.02	0.02	0.02	t	123424	LGDKFNMGL	Ovis aries
1	1.00	1	1	0.02	0.02	0.02	0.02	0.02	t	123425	LGEEYEEHL	Ovis aries
1	1.00	1	1	-0.02	-0.02	-0.02	-0.02	-0.02	t	112308	SFSGFSL	Homo sapiens
1	1.00	1	1	-0.04	-0.04	-0.04	-0.04	-0.04	t	149430	QPFLPQQPYP	Homo sapiens
1	1.00	1	1	-0.01	-0.008	-0.015	-0.013	-0.009	t	115256	FRELKDLKGY	Homo sapiens
1	1.00	1	1	0.01	0.013	0.01	0.01	0.008	t	140853	GEYVQILDAHAAYLNATPAA	Mus musculus
1	1.00	1	1	0	-0.002	0	-0.002	-0.002	t	122406	IEIGLEGKGFEPTLEALFGK	Mus musculus
1	1.00	1	1	0	-0.004	-0.012	0	0.001	t	42008	MLMRTDPFRELDRFAEQVLG	Mus musculus
1	1.00	1	1	0	-0.002	-0.011	-0.007	0	t	98434	RYMDQPSRD	Mus musculus

1	1.00	1	1	-0.01	-0.01	-0.01	-0.008	-0.01	t	27598	INCYTEGP CVPKPP	Homo sapiens
1	1.00	1	1	-0.02	-0.018	-0.013	-0.013	-0.019	t	122179	RQLGRGS	Homo sapiens
1	1.00	1	1	-0.03	-0.03	-0.03	-0.03	-0.03	t	132430	LGFA	Mus musculu s
1	1.00	1	1	0.03	0.03	0.023	0.027	0.032	t	50721	QEVLNENL LR	Homo sapiens
1	1.00	1	1	0.01	0.012	0.012	0.009	0.01	t	56885	SALTSAIA GFVFSC	Ovis aries
1	1.00	1	1	0.04	0.038	0.053	0.039	0.039	t	138862	KFPED	Mus musculu s
1	1.00	1	1	0.02	0.02	0.02	0.02	0.019	t	131437	GASVKVT	Homo sapiens
1	1.00	1	1	0	0	0	0	0	t	79223	GASHTV	Mus musculu s
1	1.00	1	1	0.01	0.012	0.008	0.007	0.009	t	105746	EETICTASF KKNIGR	Homo sapiens
1	1.00	1	1	-0.03	-0.028	-0.032	-0.031	-0.03	t	25015	HVAGAAA LILSKHPN	Cavia porcellu s
1	1.00	1	1	0.04	0.035	0.044	0.039	0.04	t	34920	LAPTVAHG VFNPEFG	Bos taurus
1	1.00	1	1	0.02	0.02	0.02	0.02	0.018	t	78073	WWSPTM LTQSKE	Mus musculu s
1	1.00	1	1	0	0.004	0.002	0.007	0.002	t	433	AASGPKVV IDGKDQN VTGSV	Homo sapiens
1	1.00	1	1	0.03	0.03	0.03	0.03	0.03	t	123791	TPVVGAV Q	Oryctola gus cuniculu s

1	1.00	1	1	0.01	0.01	0.01	0.01	0.01	t	103457	PAEFLENFVRSSNLK	Homo sapiens
1	1.00	1	1	-0.01	-0.01	-0.01	-0.01	-0.01	t	158597	KWPIIDT	Homo sapiens
1	1.00	1	1	-0.01	-0.01	-0.01	-0.01	-0.01	t	135643	CPFGFEGK	Homo sapiens
1	1.00	1	1	-0.01	-0.01	-0.01	-0.01	-0.01	t	117438	AIVTKYITKGWKEVHELYKE	Homo sapiens
1	1.00	1	1	-0.01	-0.01	-0.014	-0.01	-0.01	t	49070	PQPQQQFPQP	Mus musculus
1	1.00	1	1	-0.03	-0.03	-0.03	-0.03	-0.03	t	108555	NKALTFFLP	Homo sapiens
1	1.00	1	1	-0.04	-0.038	-0.037	-0.039	-0.04	t	24696	HRMAWNMMMNWSPT	Homo sapiens
1	1.00	1	1	0.01	0.013	0.005	0.01	0.01	t	139866	QGAFASPIQLQRRNGSKF	Mus musculus
1	1.00	1	1	0	0	0	0.004	0.001	t	138608	RVGAHDP	Homo sapiens
1	1.00	1	1	0	0	0	0.004	0.001	t	135650	DKVSFFCKNKEKKC	Homo sapiens
1	1.00	1	1	0	0	0	0.004	0.001	t	135711	LDETVKDKLAKEQKSKQNI	Homo sapiens
1	1.00	1	1	0	0	0	0.004	0.001	t	138304	ATVQLPQ	Homo sapiens
1	1.00	1	1	0	0	0	0.004	0.001	t	136420	KIHAREIFDSRGNPTVE	Homo sapiens
1	1.00	1	1	0	0	0	0.004	0.001	t	131762	YAASSYL	Homo sapiens
1	1.00	1	1	0	0	0	0.004	0.001	t	131878	FLMLVGGSTL	Homo sapiens
1	1.00	1	1	0	0	0	0.004	0.001	t	131438	GAVTVAW	Homo sapiens
1	1.00	1	1	0	0	0	0.004	0.001	t	118383	GLKKMLRTCAVHITLD	Homo sapiens
1	1.00	1	1	0	0	0	0.004	0.001	t	117742	AKAQTDREDLRTLLRY	Homo sapiens
1	1.00	1	1	0	0	0	0.004	0.001	t	133372	SPSPTPTPGPSRRG	Homo sapiens

1	1.00	1	1	0	0	0	0.004	0.001	t	133900	NEHPVFAYLKDKLP	Homo sapiens
1	1.00	1	1	0	0	0	0.004	0.001	t	131365	ATLVCLI	Homo sapiens
1	1.00	1	1	0	0	0	0.004	0.001	t	121112	AACDPRHGRYLTVA	Homo sapiens
1	1.00	1	1	0	0	0	0.004	0.001	t	117907	HKAFKGSI	Homo sapiens
1	1.00	1	1	0	0	0	0.004	0.001	t	133776	AGDLSKVDAKQPGDY	Homo sapiens
1	1.00	1	1	0	0	0	0.004	0.001	t	122874	PPARALKRSLWVEVDLLIGS	Homo sapiens
1	1.00	1	1	0	0	0	0.004	0.001	t	125229	RDSEGDLVAEKLGPI	Homo sapiens
1	1.00	1	1	0	0	0	0.004	0.001	t	131344	AIGDRSKTDRENSVSIG	Homo sapiens
1	1.00	1	1	0	0	0	0.004	0.001	t	132029	QLGPTCLSSL	Homo sapiens
1	1.00	1	1	0	0	0	0.004	0.001	t	130987	NPVENMMDRDSQ	Homo sapiens
1	1.00	1	1	0	0	0	0.004	0.001	t	131978	MLVGGSTLCV	Homo sapiens
1	1.00	1	1	0	0	0	0.004	0.001	t	118105	RKGHFLLSSKSGFWTIWL	Homo sapiens
1	1.00	1	1	0	0	0	0.004	0.001	t	117773	CNSRQTDREDELI	Homo sapiens
1	1.00	1	1	0	0	0	0.004	0.001	t	117333	MVTSTITEKLLKNLVKI	Homo sapiens
1	1.00	1	1	0	-0.002	0	0.004	0.001	t	108834	CKPISGHNSLFWYRQT	Mus musculus
1	1.00	1	1	0	0	0	0.004	0.001	t	106675	NEEGFFSARGHRPLDKK	Homo sapiens
1	1.00	1	1	0	0	0	0.004	0.001	t	117300	GSGKGKVQFQGKKTKF	Homo sapiens
1	1.00	1	1	0	0	0	0.004	0.001	t	117015	KEQFLDGDGWTSRWIESK	Homo sapiens

1	1.00	1	1	0	0	0	0.004	0.001	t	117246	VTWEVLEGEVEKEALKKI	Homo sapiens
1	1.00	1	1	0	0	0	0.004	0.001	t	63098	TCRRMLAFLKDKENR	Homo sapiens
1	1.00	1	1	0	0	0	0.004	0.001	t	32159	KLSEGDLLAEIETDK	Homo sapiens
1	1.00	1	1	0.04	0.038	0.032	0.04	0.042	t	141748	SLLTEVETP	Oryctolagus cuniculus
1	1.00	1	1	0.04	0.04	0.032	0.04	0.042	t	141748	SLLTEVETP	Mus musculus
1	1.00	1	1	0	-0.003	0	0	-0.002	t	118733	ADVPLVVEYKIADMG	Oryctolagus cuniculus
1	1.00	1	1	-0.02	-0.02	-0.016	-0.02	-0.023	t	157808	QQDRHQKIRHFR	Homo sapiens
1	1.00	1	1	0	0.004	0	0.001	0	t	52814	QVVYDYQH	Homo sapiens
1	1.00	1	1	-0.06	-0.056	-0.056	-0.059	-0.06	t	36304	LGRLIT	Homo sapiens
1	1.00	1	1	0.02	0.02	0.02	0.016	0.021	t	134250	TYNYNVL	Homo sapiens
1	1.00	1	1	0.05	0.05	0.048	0.049	0.049	t	13984	ERESQAY	Mus musculus
1	1.00	1	1	0.04	0.04	0.034	0.04	0.041	t	13524	ENMTPQD	Mus musculus
1	1.00	1	1	-0.02	-0.02	-0.017	-0.02	-0.019	t	56995	SASPIPR	Homo sapiens
1	1.00	1	1	0.02	0.022	0.02	0.023	0.02	t	16087	FGYRKPLDNIKDNVGKMEDY	Aotus nancymaae

1	1.00	1	1	-0.03	-0.032	-0.042	-0.033	-0.032	t	89067	SRTRTTSVGDKQVASKV	Mus musculus
1	1.00	1	1	0.01	0.01	0.01	0.01	0.009	t	3514	APDTR	Mus musculus
1	1.00	1	1	-0.03	-0.033	-0.033	-0.03	-0.03	t	97443	LFILTRDGM	Oryctolagus cuniculus
1	1.00	1	1	0.04	0.04	0.04	0.036	0.038	t	118563	PVTTPEEIAQVAT	Homo sapiens
1	1.00	1	1	-0.04	-0.04	-0.049	-0.04	-0.04	t	93546	PDMAEEIAALVLAKLGKDA	Mus musculus
1	1.00	1	1	0.02	0.02	0.013	0.02	0.02	t	133551	EAPPPTLPPDMAQVTVGPG	Mus musculus
1	1.00	1	1	0.03	0.03	0.03	0.03	0.029	t	104225	RPAVIPDREVLYQEFDEMEE	Homo sapiens
1	1.00	1	1	-0.01	-0.01	-0.01	-0.01	-0.011	t	140458	EHNDFPMYTWRP	Mus musculus
1	1.00	1	1	-0.03	-0.026	-0.025	-0.03	-0.03	t	44015	NGKKKNWKKIM	Mus musculus
1	1.00	1	1	0.03	0.03	0.03	0.03	0.03	t	132608	RKAGICFGDSGGPLI	Homo sapiens
1	1.00	1	1	0	0	0	0	-0.002	t	135576	LRYSTGTVSG	Oryctolagus cuniculus
1	1.00	1	1	-0.01	-0.008	-0.01	-0.009	-0.01	t	71862	VVTVRAERPGVDPDR	Homo sapiens
1	1.00	1	1	-0.05	-0.05	-0.058	-0.05	-0.048	t	20638	GKMVQVVY	Oryctolagus cuniculus
1	1.00	1	1	-0.01	-0.01	-0.014	-0.01	-0.01	t	157091	ANIKGVPTRAET	Canis lupus
1	1.00	1	1	0.05	0.054	0.038	0.047	0.052	t	70355	VPLGTQYTDA	Homo sapiens

1	1.00	1	1	0	0.003	0.001	0.001	-0.002	t	99636	RQWIIEAMSEGIKVM	Equus caballus
1	1.00	1	1	0	0	0.003	0	-0.001	t	133697	SITTANPSSFNKY	Rattus norvegicus
1	1.00	1	1	0	0	0	0	0	t	111172	CTHPEILFC	Homo sapiens
1	1.00	1	1	0	0.002	0.002	0.003	-0.001	t	6623	CLSATCDCTLCGP	Homo sapiens
1	1.00	1	1	0.02	0.017	0.028	0.02	0.018	t	23049	GVLSVVAAGNE	Oryctolagus cuniculus
1	1.00	1	1	0	-0.002	0	-0.001	-0.001	t	56687	RYYRENM	Mus musculus
1	1.00	1	1	0	0	0	0	-0.001	t	7000	CSGFVGLFSPGPSQRI	Mus musculus
1	1.00	1	1	0.02	0.018	0.022	0.02	0.019	t	122512	PEPAKSAPAPKKGSK	Mus musculus
1	1.00	1	1	0.02	0.018	0.02	0.017	0.018	t	922	AEGEFDYAWDQTHQDPAK	Mus musculus
1	1.00	1	1	0.01	0.012	0.011	0.017	0.013	t	118776	EADYFEYHQEGGPDGEPDVP	Homo sapiens
1	1.00	1	1	-0.01	-0.006	-0.01	-0.009	-0.01	t	71848	VVTGATVS	Homo sapiens
1	1.00	1	1	-0.01	-0.006	-0.017	-0.013	-0.011	t	78341	AALTAENTAIKKRNADAKA	Homo sapiens
1	1.00	1	1	0.01	0.01	0.01	0.01	0.01	t	32907	KPTPEGDLEI	Homo sapiens
1	1.00	1	1	0.01	0.01	0.01	0.01	0.01	t	22562	GSVNSFFSR	Mus musculus
1	1.00	1	1	0.01	0.01	0.01	0.01	0.01	t	22562	GSVNSFFSR	Mus musculus
1	1.00	1	1	0.01	0.01	0.01	0.01	0.01	t	22562	GSVNSFFSR	Mus musculus

1	1.00	1	1	0.01	0.01	0.01	0.012	0.01	t	10812	DWICEVLS DFKTWL	Homo sapiens
1	1.00	1	1	0.01	0.008	0.004	0.009	0.01	t	20722	GKVTNGS	Homo sapiens
1	1.00	1	1	0.03	0.03	0.03	0.03	0.03	t	31064	KGSSIAAD	Oryctola gus cuniculu s
1	1.00	1	1	0.03	0.03	0.026	0.03	0.032	t	1476	AGDQSYFL	Mus musculu s
1	1.00	1	1	-0.01	-0.013	-0.003	-0.01	-0.012	t	130622	TGLVAQG TDSIG	Camelus dromed arius
1	1.00	1	1	-0.03	-0.034	-0.031	-0.033	-0.03	t	36659	LIPLLRT	Oryctola gus cuniculu s
1	1.00	1	1	-0.03	-0.032	-0.031	-0.033	-0.03	t	36659	LIPLLRT	Mus musculu s
1	1.00	1	1	0.01	0.012	0.007	0.01	0.01	t	7846	DDPLLGN Q	Anas platyrhy nchos
1	1.00	1	1	0	0.002	-0.005	0.001	0	t	19093	GDRAAGQ PAGDRAA GQPA	Homo sapiens
1	1.00	1	1	0	0.002	-0.005	0.001	0	t	19093	GDRAAGQ PAGDRAA GQPA	Homo sapiens
1	1.00	1	1	0	0.002	-0.005	0.001	0	t	19093	GDRAAGQ PAGDRAA GQPA	Homo sapiens
1	1.00	1	1	0.02	0.023	0.02	0.02	0.021	t	107517	LSTNEVA M	Mus musculu s
1	1.00	1	1	0.03	0.028	0.04	0.029	0.026	t	97596	QTPLTMA ALELL	Mus musculu s
1	1.00	1	1	0.03	0.028	0.04	0.029	0.026	t	97596	QTPLTMA ALELL	Mus musculu s
1	1.00	1	1	-0.03	-0.028	-0.027	-0.029	-0.03	t	26600	IIMYAPTL WARMIL	Homo sapiens

1	1.00	1	1	0	0	-0.005	-0.003	-0.002	t	6238	CFLLFSGCFALREQA	Homo sapiens
1	1.00	1	1	0.01	0.024	-0.006	0.007	0.01	t	21373	GNAEKYDKMDEPQHYGKS	Homo sapiens
1	1.00	1	1	0.05	0.05	0.056	0.054	0.05	t	45965	NSRSTS	Homo sapiens
1	1.00	1	1	0.01	0.009	0.007	0.013	0.01	t	8834	DKAAEAIINELKKASKKVGG	Homo sapiens
1	1.00	1	1	0	0	-0.003	0.006	0	t	26801	IKGVIVHRLEGVSYN	Homo sapiens
1	1.00	1	1	-0.02	-0.018	-0.019	-0.019	-0.023	t	79575	IPKARRPEGRTWAQPGY	Homo sapiens
1	1.00	1	1	0.02	0.018	0.022	0.023	0.022	t	23354	GYHGSSLY	Mus musculus
1	1.00	1	1	-0.04	-0.04	-0.04	-0.04	-0.04	t	137391	DTHKRDTY	Oryctolagus cuniculus
1	1.00	1	1	0	0.004	0.003	0.001	0.002	t	64697	TKVTLDGRPL	Homo sapiens
1	1.00	1	1	-0.01	-0.01	-0.01	-0.007	-0.01	t	61124	SSIVYEAADMIMHT	Homo sapiens
1	1.00	1	1	0.03	0.03	0.035	0.033	0.03	t	18527	GAASSYFEYVDTYG	Homo sapiens
1	1.00	1	1	0.05	0.053	0.056	0.05	0.05	t	53194	RARGIYETETRA	Mus musculus
1	1.00	1	1	0.03	0.03	0.03	0.03	0.03	t	123872	YLKELPTTL	Ovis aries
1	1.00	1	1	0.03	0.03	0.03	0.03	0.03	t	115465	RCEKDERFFSDKIAKYIPIQ	Homo sapiens
1	1.00	1	1	0.01	0.01	0.01	0.01	0.01	t	135755	SALVLQYL	Homo sapiens

1	1.00	1	1	0.01	0.01	0.01	0.01	0.01	t	137733	DTALTIQA LKLV	Mus musculus
1	1.00	1	1	0.01	0.01	0.01	0.01	0.01	t	140586	PWQLPLN FQ	Mus musculus
1	1.00	1	1	0.01	0.01	0.01	0.01	0.01	t	140586	PWQLPLN FQ	Mus musculus
1	1.00	1	1	0.01	0.01	0.01	0.01	0.01	t	121778	RDDPQLFA EAYAFCF	Mus musculus
1	1.00	1	1	0.01	0.01	0.01	0.01	0.01	t	121778	RDDPQLFA EAYAFCF	Mus musculus
1	1.00	1	1	0.01	0.01	0.01	0.01	0.01	t	109700	PLGPKGQT	Rattus norvegicus
1	1.00	1	1	0	0.002	0.005	0.003	0.002	t	138238	SMLDTPT	Homo sapiens
1	1.00	1	1	-0.01	-0.01	-0.01	-0.01	-0.01	t	135720	MKGKYGIY	Homo sapiens
1	1.00	1	1	-0.01	-0.01	-0.01	-0.01	-0.01	t	98958	QILQQILQ QQLI	Homo sapiens
1	1.00	1	1	-0.01	-0.007	-0.011	-0.01	-0.012	t	45646	NQYGRIRV LQRFNQR	Sus scrofa
1	1.00	1	1	-0.02	-0.019	-0.022	-0.017	-0.023	t	64876	TLKKGSSG YCAWPAC	Equus caballus
1	1.00	1	1	0.03	0.028	0.027	0.023	0.029	t	122167	PPPITQGE CKQL	Mus musculus
1	1.00	1	1	0	0	0	-0.008	-0.005	t	23179	GVTSAPDT RPAPGSTA	Mus musculus
1	1.00	1	1	0.01	0.008	0.017	0.013	0.01	t	97445	LGNATALS	Mus musculus
1	1.00	1	1	0	-0.002	0	-0.002	-0.002	t	122406	IEIGLEGKG FEPTLEALF GK	Mus musculus
1	1.00	1	1	0	0	0	0	0	t	137448	SHKLHKV	Mus musculus

1	1.00	1	1	0	0	0	0	0	t	132814	FGENRPA	Homo sapiens
1	1.00	1	1	0	0.003	0	0	0	t	72505	WGQGGS HSQW	Mus musculu s
1	1.00	1	1	-0.01	-0.012	-0.021	-0.007	-0.008	t	43317	NATAEGR AINRRVE	Mus musculu s
1	1.00	1	1	0	0.002	-0.007	-0.003	-0.001	t	57523	SELLISTAV Q	Homo sapiens
1	1.00	1	1	0.01	0.013	0.01	0.01	0.011	t	144705	KGTHSLYS QIAG	Mus musculu s
1	1.00	1	1	0	0	0	0	0	t	21990	GRAIN	Mus musculu s
1	1.00	1	1	-0.02	-0.02	-0.02	-0.02	-0.02	t	97020	TVTRGTIS DP	Oryctola gus cuniculu s
1	1.00	1	1	-0.02	-0.02	-0.02	-0.02	-0.02	t	97020	TVTRGTIS DP	Oryctola gus cuniculu s
1	1.00	1	1	0.03	0.032	0.03	0.031	0.03	t	133661	NYSPLHNS DITV	Homo sapiens
1	1.00	1	1	-0.01	-0.014	-0.01	-0.011	-0.01	t	52476	QTASGLVS MF	Oryctola gus cuniculu s
1	1.00	1	1	0.03	0.033	0.014	0.036	0.039	t	136062	FGISETQA EAILE	Mus musculu s
1	1.00	1	1	-0.04	-0.04	-0.036	-0.034	-0.038	t	53682	RFAPALPV	Homo sapiens
1	1.00	1	1	0.01	0.013	0.01	0.01	0.012	t	134702	KEKKYS	Mus musculu s

1	1.00	1	1	0.01	0.008	0.007	0.013	0.009	t	23690	HEHHGVYNQGRGIDSGERLM	Mus musculus
1	1.00	1	1	0.01	0.008	0.007	0.013	0.009	t	23690	HEHHGVYNQGRGIDSGERLM	Mus musculus
1	1.00	1	1	0.02	0.02	0.02	0.02	0.02	t	106965	VKGEEKEPSK	Homo sapiens
1	1.00	1	1	-0.01	-0.012	-0.014	-0.013	-0.01	t	122197	TDVVFNSKGIGN	Mus musculus
1	1.00	1	1	0.02	0.018	0.015	0.013	0.017	t	108607	RGAQGPPGATGF	Mus musculus
1	1.00	1	1	0.02	0.02	0.02	0.02	0.02	t	122101	GKGAGDL	Homo sapiens
1	1.00	1	1	0	0	0	0	0	t	153458	SELSPAMLHFMFGGGS	Mus musculus
1	1.00	1	1	0	0.003	0	0	0	t	105232	GSNKGAIIGLM	Mus musculus
1	1.00	1	1	0	0.003	0	0	0	t	105232	GSNKGAIIGLM	Mus musculus
1	1.00	1	1	-0.01	-0.008	-0.015	-0.009	-0.01	t	56804	SAGRSTGSLVRLFAPG	Homo sapiens
1	1.00	1	1	0.07	0.071	0.067	0.07	0.071	t	19405	GEQQEAIKSAENATKVEDIK	Oryctolagus cuniculus
1	1.00	1	1	0	0	-0.007	0	0	t	124601	QGPGLTDWLFPRR	Mus musculus
1	1.00	1	1	-0.01	-0.01	-0.01	-0.01	-0.01	t	9399	DMRNTIMASK	Homo sapiens
1	1.00	1	1	0.03	0.036	0.03	0.03	0.03	t	123520	MSEKFNMGL	Ovis aries

1	1.00	1	1	0.04	0.04	0.045	0.033	0.036	t	140665	DTAAQI	Mus musculus
1	1.00	1	1	-0.03	-0.03	-0.037	-0.036	-0.03	t	49368	PSFSDIPNPI	Homo sapiens
1	1.00	1	1	0.03	0.028	0.03	0.037	0.03	t	123760	SSYAKVSICLPMDTETPLAL	Homo sapiens
1	1.00	1	1	-0.05	-0.056	-0.057	-0.05	-0.05	t	123035	APRIMHHM	Oryctolagus cuniculus
1	1.00	1	1	0	-0.003	-0.006	-0.001	-0.001	t	105901	KGTAPFEI	Oryctolagus cuniculus
1	1.00	1	1	0	0	0.007	0	0	t	119451	FRKTSISGA	Homo sapiens
1	1.00	1	1	0.01	0.012	0.005	0.011	0.009	t	25149	HYAPRPCGI	Homo sapiens
1	1.00	1	1	0.02	0.02	0.021	0.02	0.02	t	57638	SESVEFTKDTQTGMSG	Mus musculus
1	1.00	1	1	-0.01	-0.01	-0.017	-0.01	-0.01	t	106375	EDHAGTYGLGDRK	Mus musculus
1	1.00	1	1	0.05	0.045	0.043	0.047	0.05	t	96229	EGQLGD	Oryctolagus cuniculus
1	1.00	1	1	0.01	0.01	0.01	0.01	0.009	t	149298	ELDKWAS	Homo sapiens
1	1.00	1	1	0.02	0.022	0.015	0.019	0.02	t	100120	HQKLVFFAED	Homo sapiens(human)
1	1.00	1	1	0.01	0.01	0.01	0.01	0.01	t	135572	LLCEDGCLAL	Oryctolagus cuniculus
1	1.00	1	1	0.01	0.01	0.01	0.01	0.01	t	135572	LLCEDGCLAL	Oryctolagus cuniculus

1	1.00	1	1	0.01	0.01	0.01	0.01	0.01	t	97564	PTDAGVA T	Mus musculu s
1	1.00	1	1	0.01	0.01	0.01	0.01	0.01	t	49533	PSTPGDNP LV	Homo sapiens
1	1.00	1	1	-0.01	-0.01	-0.014	-0.01	-0.01	t	49068	PQPQQPF CQQ	Mus musculu s
1	1.00	1	1	0.02	0.02	0.026	0.02	0.02	t	86153	MANTGA MELV	Homo sapiens
1	1.00	1	1	-0.01	-0.008	-0.003	-0.01	-0.01	t	95003	CTKPSDGN C	Homo sapiens
1	1.00	1	1	-0.01	-0.008	-0.003	-0.01	-0.01	t	95003	CTKPSDGN C	Homo sapiens
1	1.00	1	1	0	0.001	-0.001	0.001	0	t	13064	ELGAFFSIL QDMR	Homo sapiens
1	1.00	1	1	-0.03	-0.03	-0.04	-0.027	-0.03	t	55987	RSVHDVVL VGGSTRIP KVQS	Homo sapiens
1	1.00	1	1	-0.03	-0.035	-0.04	-0.027	-0.03	t	55987	RSVHDVVL VGGSTRIP KVQS	Canis lupus
1	1.00	1	1	0.02	0.016	0.016	0.02	0.02	t	140761	TYRVDVD GYRND	Bos taurus
1	1.00	1	1	0.02	0.016	0.016	0.02	0.02	t	140761	TYRVDVD GYRND	Bos taurus
1	1.00	1	1	0	0	-0.005	0	0	t	60991	SSFFSYGEI	Mus musculu s
1	1.00	1	1	0.01	0.01	0.015	0.013	0.01	t	22658	GTGGTAS MSYKFPPV PPENL	Homo sapiens

1	1.00	1	1	0	0.002	0	0.001	0	t	56958	SAQSGTSG TSAQSGT	Homo sapiens
1	1.00	1	1	0.02	0.024	0.022	0.021	0.02	t	74201	YIEQGMQ LAEQFKQ	Homo sapiens
1	1.00	1	1	0	-0.001	0.002	0.003	0	t	29005	ITNDQKKL MS	Bos taurus
1	1.00	1	1	0	0.004	0.011	0.007	0.002	t	122209	VQKDVLL	Homo sapiens
1	1.00	1	1	-0.02	-0.02	-0.02	-0.02	-0.02	t	141760	TAPPLLPH	Rattus norvegic us
1	1.00	1	1	-0.02	-0.02	-0.02	-0.02	-0.02	t	141760	TAPPLLPH	Rattus norvegic us
1	1.00	1	1	0.03	0.03	0.034	0.03	0.032	t	136057	DISAFAD	Rattus norvegic us
1	1.00	1	1	-0.02	-0.025	-0.03	-0.02	-0.019	t	132875	NCTKDFM SVSFSQIL	Trichosu rus vulpecul a
1	1.00	1	1	-0.04	-0.038	-0.048	-0.043	-0.04	t	12531	EINHVQD HALPKENI	Homo sapiens
1	1.00	1	1	0.02	0.02	0.025	0.02	0.021	t	122194	STVQDCV	Homo sapiens
1	1.00	1	1	-0.02	-0.016	-0.018	-0.02	-0.018	t	136441	KRQKRF	Gallus gallus
1	1.00	1	1	0.04	0.04	0.028	0.036	0.041	t	117773	CNSRQTD REDELI	Homo sapiens

1	1.00	1	1	-0.01	-0.01	-0.012	-0.011	-0.01	t	83781	INPWDSKS DYLG	Homo sapiens
1	1.00	1	1	0	0	0	0	0	t	34709	LADALLEK	Oryctola gus cuniculu s
1	1.00	1	1	-0.05	-0.05	-0.04	-0.043	-0.049	t	122136	LGRGGSK	Homo sapiens
1	1.00	1	1	0.02	0.03	0.02	0.019	0.02	t	65930	TQSCNCSI YP	Pan troglody tes
1	1.00	1	1	0.02	0.02	0.02	0.02	0.02	t	79932	MKPYEVT PTRM	Mus musculu s
1	1.00	1	1	0.01	0.01	0.011	0.003	0.01	t	55293	RPQDVKFP	Homo sapiens
1	1.00	1	1	-0.01	-0.01	-0.002	-0.008	-0.013	t	149256	ALDKWQN	Homo sapiens
1	1.00	1	1	0	0	0	0	-0.001	t	7001	CSGFVSLL APGAKQN	Mus musculu s
1	1.00	1	1	0.02	0.02	0.013	0.02	0.02	t	105835	GLKGEQG EPGA	Mus musculu s
1	1.00	1	1	0.02	0.021	0.021	0.017	0.02	t	57506	SEKSKCEE NTMFQPY	Equus caballus
1	1.00	1	1	0.04	0.04	0.04	0.04	0.04	t	156339	KDDPTNKF YQSVIQ	Homo sapiens
1	1.00	1	1	0.04	0.04	0.04	0.04	0.04	t	133954	WIKENF	Mus musculu s
1	1.00	1	1	0	0	0	0	0	t	130805	ELNPEPDT EVFPMTF	Homo sapiens
1	1.00	1	1	0	0	0	0	0	t	130805	ELNPEPDT EVFPMTF	Homo sapiens
1	1.00	1	1	0	0	0	0	0	t	130805	ELNPEPDT EVFPMTF	Homo sapiens

1	1.00	1	1	-0.02	-0.02	-0.02	-0.02	-0.02	t	135762	SYVTPICI	Homo sapiens
1	1.00	1	1	-0.04	-0.038	-0.04	-0.033	-0.04	t	107859	GHRPLDKKREEAPSLRPA	Homo sapiens
1	1.00	1	1	-0.01	-0.008	-0.007	-0.013	-0.012	t	41056	MAKLVLSLCFLLFSG	Homo sapiens
1	1.00	1	1	0.01	0.011	0.009	0.011	0.009	t	7092	CSTKAIGRTILKENIKYEVA	Equus caballus
1	1.00	1	1	-0.01	-0.01	-0.012	-0.01	-0.013	t	19504	GFCDGYCKTHLKASS	Mus musculus
1	1.00	1	1	-0.02	-0.02	-0.023	-0.027	-0.023	t	115630	NFDKLYIWG	Homo sapiens
1	1.00	1	1	0	-0.003	0.003	0	-0.003	t	111120	ARMRLYDPNQAALQA	Oryctolagus cuniculus
1	1.00	1	1	0.02	0.02	0.018	0.02	0.02	t	73092	WSPQAQGILTTV	Mus musculus
1	1.00	1	1	0	0	0	0.003	0	t	29937	KARRPEGRTWAQPGY	Homo sapiens
1	1.00	1	1	-0.01	-0.008	-0.005	-0.007	-0.01	t	57679	SEVYCPRPDRCLRAP	Homo sapiens
1	1.00	1	1	0.05	0.05	0.05	0.05	0.05	t	158418	VEGERVETT	Canis lupus
1	1.00	1	1	-0.03	-0.03	-0.03	-0.03	-0.03	t	110618	GPVGPAGGPGFP	Mus musculus
1	1.00	1	1	0	0	0	0	-0.002	t	117625	REAVAGRGRGRGR	Homo sapiens
1	1.00	1	1	-0.03	-0.032	-0.05	-0.037	-0.028	t	44556	NKNDNKNDNKND	Mus musculus
1	1.00	1	1	-0.03	-0.032	-0.05	-0.037	-0.028	t	44556	NKNDNKNDNKND	Mus musculus
1	1.00	1	1	0.05	0.05	0.05	0.05	0.05	t	108262	AENGPRLLVEAEQAKVFS	Mus musculus

1	1.00	1	1	0.01	0.008	0.005	0.009	0.01	t	78752	NKGKWKEHIKEVTEKLKKA	Mus musculus
1	1.00	1	1	0.01	0.006	0.005	0.009	0.01	t	78752	NKGKWKEHIKEVTEKLKKA	Oryctolagus cuniculus
1	1.00	1	1	-0.01	-0.007	-0.019	-0.01	-0.008	t	63756	TFTNKLKEKHTDLGK	Mus musculus
1	1.00	1	1	0.03	0.03	0.03	0.03	0.03	t	142415	EVAKAEVLNAEVK	Homo sapiens
1	1.00	1	1	-0.01	-0.01	-0.01	-0.01	-0.01	t	135464	GLAPGV	Oryctolagus cuniculus
1	1.00	1	1	0.01	0.012	0.01	0.011	0.009	t	58458	SICGNNQLCKSICKT	Homo sapiens
1	1.00	1	1	0	0	-0.004	0	0	t	15510	FEDVNGIRKP	Mus musculus
1	1.00	1	1	0	0	-0.004	0	0	t	15510	FEDVNGIRKP	Mus musculus
1	1.00	1	1	-0.02	-0.02	-0.019	-0.02	-0.02	t	70025	VMTRSSHQSLVIKLM	Homo sapiens
1	1.00	1	1	0.05	0.05	0.05	0.05	0.05	t	79603	LLTTDEL	Oryctolagus cuniculus
1	1.00	1	1	0.03	0.03	0.03	0.03	0.03	t	98842	KDLAFPGSGE	Homo sapiens
1	1.00	1	1	0.01	0.01	0.01	0.01	0.01	t	103348	LGDVGEAFVDSLTSQ	Homo sapiens
1	1.00	1	1	-0.01	-0.01	-0.01	-0.01	-0.01	t	119583	GNVFSCSV	Homo sapiens
1	1.00	1	1	-0.01	-0.01	-0.01	-0.01	-0.01	t	51084	QIISQQPFPL	Mus musculus

1	1.00	1	1	-0.01	-0.01	-0.01	-0.01	-0.01	t	51084	QIISQQPFPL	Mus musculus
1	1.00	1	1	0	-0.002	0	0.003	0	t	57631	SESTEDQAMEDIKQMEAESI	Mus musculus
1	1.00	1	1	0	0.002	0.008	0.001	-0.001	t	146417	LELDKWASL	Homo sapiens
1	1.00	1	1	0.02	0.018	0.021	0.017	0.017	t	55785	RRYQKSTEL	Mus musculus
1	1.00	1	1	0	-0.003	0	0	0	t	19927	GGMPGGMPGGMPG	Oryctolagus cuniculus
1	1.00	1	1	-0.01	-0.01	-0.01	-0.007	-0.01	t	24284	HLPYIEQGMMLAEQFK	Homo sapiens
1	1.00	1	1	-0.01	-0.01	-0.01	-0.007	-0.01	t	24284	HLPYIEQGMMLAEQFK	Homo sapiens
1	1.00	1	1	0	0.004	0.002	0.003	0	t	78643	EQGMQLAEQFKQKALGL	Homo sapiens
1	1.00	1	1	0.02	0.02	0.02	0.022	0.02	t	44232	NIGGEGNHNESPL	Homo sapiens
1	1.00	1	1	0.02	0.02	0.02	0.02	0.018	t	123287	HDSPSVRKTH	Oryctolagus cuniculus
1	1.00	1	1	0	0	0	0.002	0	t	3507	APDKEILYEAFDEM	Homo sapiens
1	1.00	1	1	0.02	0.023	0.02	0.02	0.018	t	135458	FGSDYEDRYYR	Mus musculus
1	1.00	1	1	0.01	0.01	0.011	0.01	0.01	t	43411	NDDASVDFVAEPVK	Mus musculus
1	1.00	1	1	0.01	0.01	0.011	0.01	0.01	t	43411	NDDASVDFVAEPVK	Mus musculus
1	1.00	1	1	0.01	0.01	0.01	0.01	0.01	t	144781	SGVLLQYYGM	Homo sapiens

1	1.00	1	1	-0.06	-0.056	-0.06	-0.053	-0.058	t	145639	WTPGVKRNGF	Homo sapiens
1	1.00	1	1	0	-0.002	-0.011	-0.001	0	t	95094	IGFRGLDGVHCTPTTDSGVI	Rattus norvegicus
1	1.00	1	1	-0.03	-0.027	-0.037	-0.03	-0.028	t	100318	NAPKTFQFIN	Mus musculus
1	1.00	1	1	0	-0.004	0	-0.003	-0.003	t	71143	VSSYHSKPCNPAQPV	Oryctolagus cuniculus
1	1.00	1	1	0	-0.004	0	-0.003	-0.003	t	48779	PPFSLSPVPTLGSRSRR	Oryctolagus cuniculus
1	1.00	1	1	0.02	0.02	0.02	0.02	0.02	t	68631	VGATPERP	Homo sapiens
1	1.00	1	1	0.01	0.01	0.01	0.01	0.009	t	124736	SPWSIGA	Homo sapiens
1	1.00	1	1	-0.01	-0.01	-0.006	-0.01	-0.008	t	136054	APAPTP	Rattus norvegicus
1	1.00	1	1	-0.02	-0.022	-0.032	-0.027	-0.021	t	144604	CYAIRN	Mus musculus
1	1.00	1	1	0.01	0.014	0.017	0.007	0.009	t	106336	DHFADGYDEV	Homo sapiens
1	1.00	1	1	0.02	0.023	0.013	0.02	0.02	t	53633	RESNGETK	Equus caballus
1	1.00	1	1	0.02	0.02	0.013	0.02	0.02	t	53633	RESNGETK	Mus musculus
1	1.00	1	1	-0.03	-0.028	-0.03	-0.023	-0.03	t	117432	AARGRGRGMGRGNIF	Homo sapiens

1	1.00	1	1	0.04	0.038	0.042	0.037	0.04	t	139029	DLQVLT	Mus musculus
1	1.00	1	1	0.04	0.04	0.04	0.04	0.04	t	35127	LDDSYS	Homo sapiens
1	1.00	1	1	0	0	0	0	0	t	141052	LRDIFAWEP	Ovis aries
1	1.00	1	1	0	0	0	0	0	t	115403	NQELAYFYPELFRQFYQLDA	Homo sapiens
1	1.00	1	1	0	0	0	0	0	t	109189	GPLGPKGQ	Rattus norvegicus
1	1.00	1	1	-0.02	-0.018	-0.033	-0.019	-0.018	t	137946	PGYYRLTLIANG	Homo sapiens
1	1.00	1	1	-0.02	-0.02	-0.019	-0.023	-0.02	t	55577	RRGPRLGV	Homo sapiens
1	1.00	1	1	0.03	0.032	0.03	0.033	0.028	t	107799	DSGHRGYSGSQASDNEGH	Homo sapiens
1	1.00	1	1	0	0.003	-0.007	0	-0.001	t	55106	RPADKTRHKFPTNINKQ	Equus caballus
1	1.00	1	1	-0.01	-0.01	-0.01	-0.01	-0.011	t	91100	AFQTPVHS	Mus musculus
1	1.00	1	1	-0.02	-0.02	-0.023	-0.02	-0.02	t	98858	LACQCLVRTP	Homo sapiens
1	1.00	1	1	0.01	0.012	0.01	0.011	0.01	t	98938	PQP	Homo sapiens
1	1.00	1	1	-0.03	-0.03	-0.033	-0.032	-0.03	t	98840	KALPMHIRLS	Homo sapiens
1	1.00	1	1	0	0	0	0	-0.001	t	3516	APDTRP	Mus musculus
1	1.00	1	1	-0.02	-0.02	-0.024	-0.02	-0.018	t	36890	LKIKIASGFGPLITH	Homo sapiens
1	1.00	1	1	0	0	0	0	0.001	t	52082	QQPFP	Mus musculus

1	1.00	1	1	0	0	0.005	0	-0.003	t	22305	GSDYEDRYYRENMHRYPNQ	Mus musculus
1	1.00	1	1	0	0	-0.003	0.004	0	t	39732	LSVDLSLTVELKIKI	Homo sapiens
1	1.00	1	1	0.04	0.038	0.032	0.033	0.039	t	122167	PPPITQGECKQL	Mus musculus
1	1.00	1	1	-0.01	-0.008	-0.017	-0.007	-0.006	t	135896	APHLTRAYAKDV	Homo sapiens
1	1.00	1	1	-0.01	-0.01	-0.01	-0.01	-0.01	t	156874	GHFPPTPIKTLA	Gallus gallus
1	1.00	1	1	-0.02	-0.02	-0.025	-0.02	-0.018	t	44793	NLLRFFVAPF	Homo sapiens
1	1.00	1	1	0	0.003	-0.006	0	0.002	t	133724	TVCAGYCMTRDVN	Mus musculus
1	1.00	1	1	-0.07	-0.066	-0.071	-0.063	-0.07	t	127963	EPKPKKAPAKKGEKV	Homo sapiens
1	1.00	1	1	0.04	0.037	0.04	0.041	0.04	t	49147	PQVNLVDT	Bos taurus
1	1.00	1	1	0	0	-0.005	0	0.001	t	10801	DWEDRYYRE	Mus musculus
1	1.00	1	1	0.01	0.007	0.002	0.01	0.011	t	60827	SRVKNLNSSEGVPDLLV	Oryctolagus cuniculus
1	1.00	1	1	-0.01	-0.012	-0.002	-0.005	-0.012	t	138841	EASPQAWWDESQ	Mus musculus
1	1.00	1	1	-0.01	-0.01	-0.01	-0.007	-0.01	t	32109	KLQKTYSQYKVQYDM	Homo sapiens
1	1.00	1	1	0.04	0.042	0.04	0.042	0.04	t	13986	EREVSVPA	Homo sapiens
1	1.00	1	1	0.05	0.05	0.05	0.05	0.05	t	122799	IKYIAEQLES	Rattus norvegicus

1	1.00	1	1	0.03	0.03	0.03	0.03	0.03	t	149041	FLHTRLFVSDWYNTP	Rattus norvegicus
1	1.00	1	1	0.01	0.01	0.01	0.01	0.01	t	123128	EGAAMDLRM	Ovis aries
1	1.00	1	1	0.01	0.01	0.01	0.01	0.01	t	109027	GAPGPAGE	Rattus norvegicus
1	1.00	1	1	0.01	0.01	0.01	0.01	0.01	t	103246	GYIPILGAPWLADLV	Homo sapiens
1	1.00	1	1	-0.01	-0.01	-0.01	-0.01	-0.01	t	115931	QTPVVVPPFL	Homo sapiens
1	1.00	1	1	-0.01	-0.01	-0.01	-0.01	-0.01	t	99267	GEPDLSNNFGKLFEV	Homo sapiens
1	1.00	1	1	0	0.004	-0.003	-0.003	-0.003	t	115404	NQEQPIRCEKDERFFSDKIA	Homo sapiens
1	1.00	1	1	0.02	0.017	0.016	0.02	0.021	t	153286	QKLVFFAEDVGSNKGA	Oryctolagus cuniculus
1	1.00	1	1	0.01	0.006	0.01	0.013	0.011	t	57630	SESTEDQAMEDIKQMEAE	Oryctolagus cuniculus
1	1.00	1	1	-0.01	-0.01	-0.01	-0.01	-0.011	t	133536	CKPVENRAC	Mus musculus
1	1.00	1	1	0.01	0.011	0.003	0.007	0.013	t	130553	MDPNCSC	Mus musculus
1	1.00	1	1	0.01	0.013	0.003	0.01	0.008	t	45003	NMLYKDDFVGALIFSGAVI	Mus musculus
1	1.00	1	1	0.01	0.021	0.003	0.01	0.008	t	45003	NMLYKDDFVGALIFSGAVI	Gallus gallus
1	1.00	1	1	0.01	0.013	0.003	0.01	0.008	t	45003	NMLYKDDFVGALIFSGAVI	Mus musculus

1	1.00	1	1	0.02	0.02	0.014	0.02	0.021	t	76185	YTTGGAQSHTLR	Mus musculus
1	1.00	1	1	0	0	0	0	0.001	t	153381	ETPLTIAGLKLK	Mus musculus
1	1.00	1	1	-0.02	-0.022	-0.025	-0.02	-0.02	t	117295	GITKPAIRRLARRGG	Mus musculus
1	1.00	1	1	0	0	0	0.002	0	t	27554	IMSGEKPSMEDVVN	Homo sapiens
1	1.00	1	1	0	-0.004	-0.004	0.003	0.002	t	1385	AFRELADA	Oryctolagus cuniculus
1	1.00	1	1	-0.01	-0.008	-0.012	-0.009	-0.009	t	49748	PTPSAPARGE	Homo sapiens
1	1.00	1	1	0.03	0.032	0.04	0.033	0.03	t	60649	SREQLNKLFGIE	Homo sapiens
1	1.00	1	1	-0.06	-0.06	-0.06	-0.06	-0.061	t	105701	CVVGCGGGV	Mus musculus
1	1.00	1	1	0	-0.002	-0.005	-0.007	0.001	t	124008	DGVVLVD	Mus musculus
1	1.00	1	1	0	0	-0.005	0	0	t	132687	STQTAGPWHLGKLEL	Mus musculus
1	1.00	1	1	-0.02	-0.02	-0.033	-0.02	-0.019	t	69323	VKTIGDKRTLTLNTTANYT	Mus musculus
1	1.00	1	1	0	0	-0.005	0	-0.001	t	66683	TTKQRQNKPPNK	Mus musculus
1	1.00	1	1	0.02	0.02	0.012	0.019	0.02	t	80395	ALYASVAVQQQD	Homo sapiens
1	1.00	1	1	0.02	0.02	0.017	0.02	0.022	t	133531	CDTLKPGEC	Mus musculus
1	1.00	1	1	-0.05	-0.05	-0.05	-0.05	-0.05	t	145831	IPLGTRP	Mus musculus
1	1.00	1	1	0	0	0	0	0	t	66938	TVAGCALPPPKKTP	Homo sapiens

1	1.00	1	1	0.01	0.01	0.011	0.016	0.01	t	71055	VSNAPPVASPSILKPGASAA	Homo sapiens
1	1.00	1	1	0.01	0.01	0.003	0.001	0.01	t	119508	QFIGYPI	Homo sapiens
1	1.00	1	1	-0.02	-0.018	-0.018	-0.02	-0.022	t	9595	DPALLLMHELIHVLHGLYG	Homo sapiens
1	1.00	1	1	-0.05	-0.05	-0.043	-0.05	-0.054	t	137866	GDTRPRFL	Oryctolagus cuniculus
1	1.00	1	1	-0.01	-0.006	-0.009	-0.013	-0.01	t	36576	LIKIFQDHPL	Homo sapiens
1	1.00	1	1	-0.01	-0.008	-0.009	-0.013	-0.01	t	36576	LIKIFQDHPL	Homo sapiens
1	1.00	1	1	0.01	0.01	0.01	0.01	0.011	t	50929	QGPGAP	Mus musculus
1	1.00	1	1	0.01	0.01	0.01	0.01	0.011	t	50929	QGPGAP	Mus musculus
1	1.00	1	1	0.02	0.018	0.015	0.019	0.02	t	78875	VLHSDYCIVPNKGGTSIGS	Mus musculus
1	1.00	1	1	0	0.002	0.009	0.007	0	t	110868	GNVSPGTRREYHTEKLVT	Homo sapiens
1	1.00	1	1	0	0.002	0.009	0.007	0	t	110868	GNVSPGTRREYHTEKLVT	Homo sapiens
1	1.00	1	1	0.04	0.04	0.04	0.04	0.04	t	112396	WASTRES	Homo sapiens
1	1.00	1	1	0.02	0.02	0.02	0.02	0.02	t	140585	PWNTFATNA	Mus musculus
1	1.00	1	1	0.02	0.02	0.02	0.02	0.02	t	140585	PWNTFATNA	Mus musculus
1	1.00	1	1	0.02	0.02	0.02	0.02	0.02	t	122458	LEPETGKDMSPYEAYKRGII	Homo sapiens
1	1.00	1	1	0.02	0.02	0.02	0.02	0.02	t	92346	APTSMLDQSL	Ovis aries

1	1.00	1	1	0	0	0	0	0	t	151070	WTLVEPGDKITFEAT	Homo sapiens
1	1.00	1	1	0.01	0.012	0.011	0.013	0.01	t	31152	KHSEWLNKARGIESW	Homo sapiens
1	1.00	1	1	-0.04	-0.04	-0.04	-0.033	-0.04	t	117519	GRGTVAAAAAAAT	Mus musculus
1	1.00	1	1	0.02	0.022	0.022	0.021	0.019	t	103544	RGEHRDEHTQDAGDK	Homo sapiens
1	1.00	1	1	0.03	0.034	0.03	0.037	0.032	t	117363	RPDAEYWNSQKDLLEQKRGR	Homo sapiens
1	1.00	1	1	0.01	0.01	0.01	0.01	0.01	t	30421	KEGYLVDKNT	Mus musculus
1	1.00	1	1	0.03	0.029	0.03	0.03	0.03	t	48771	PPETTTIP	Anas platyrhynchos
1	1.00	1	1	0.04	0.034	0.04	0.04	0.04	t	123395	KSQETPAT	Oryctolagus cuniculus
1	1.00	1	1	0.06	0.062	0.072	0.063	0.062	t	40254	LVAEEDER	Homo sapiens
1	1.00	1	1	0.01	0.014	0.013	0.007	0.01	t	71140	VSSSLAPCIP	Homo sapiens
1	1.00	1	1	-0.01	-0.012	-0.01	-0.009	-0.01	t	149439	QQQPLSQVSF	Mus musculus
1	1.00	1	1	-0.01	-0.006	-0.008	-0.009	-0.01	t	120904	RALKRRNARTKAR	Homo sapiens
1	1.00	1	1	-0.02	-0.02	-0.017	-0.021	-0.02	t	49883	PVIGSQAR	Homo sapiens
1	1.00	1	1	0	0	0.005	0	-0.001	t	107214	MVGGVV	Mus musculus

1	1.00	1	1	0	0.003	0.006	0	-0.001	t	119322	QRVFKEKVDTKAPEPP	Mus musculus
1	1.00	1	1	0.01	0.014	0.016	0.011	0.01	t	53905	RGLAQALIDQ	Homo sapiens
1	1.00	1	1	0.01	0.014	0.016	0.011	0.01	t	53905	RGLAQALIDQ	Homo sapiens
1	1.00	1	1	0.03	0.03	0.03	0.03	0.03	t	115605	GDEVELPCRISP	Homo sapiens
1	1.00	1	1	0.03	0.03	0.03	0.03	0.03	t	115605	GDEVELPCRISP	Homo sapiens
1	1.00	1	1	0.03	0.03	0.03	0.03	0.03	t	79649	SPSAPTH	Oryctolagus cuniculus
1	1.00	1	1	0.01	0.01	0.01	0.01	0.01	t	115653	SSYAGQFRVIGP	Homo sapiens
1	1.00	1	1	0.01	0.01	0.01	0.01	0.01	t	115653	SSYAGQFRVIGP	Homo sapiens
1	1.00	1	1	0.01	0.01	0.01	0.01	0.01	t	103693	VFGGAASCAAPIQAD	Homo sapiens
1	1.00	1	1	0.01	0.012	0.01	0.01	0.01	t	82034	FFVQYGGAYKRH	Homo sapiens
1	1.00	1	1	0	0	0	0	0.002	t	78558	VSIFPPA	Mus musculus
1	1.00	1	1	0.01	0.009	0.007	0.011	0.011	t	17129	FNASDSVGQQIKVIPVDPYF	Homo sapiens
1	1.00	1	1	-0.02	-0.022	-0.02	-0.023	-0.02	t	24749	HSHYHKF	Mus musculus
1	1.00	1	1	0.01	0.007	0.006	0.01	0.01	t	4074	AQSTMKAEELTPGR	Myodes glareolus
1	1.00	1	1	0.01	0.014	0.017	0.017	0.011	t	135547	DWEYSVWSLN	Homo sapiens

1	1.00	1	1	0	0	-0.002	0	0	t	110596	GIAGFKGD<br>QGPKG	Rattus<br>norvegic<br>us
1	1.00	1	1	-0.08	-0.076	-0.08	-0.073	-0.081	t	134147	LPKGVKHL<br>KDFPILPGE<br>IFK	Homo<br>sapiens
1	1.00	1	1	0	0.003	0	0	0	t	60594	SQWNKPS<br>K	Mus<br>musculu<br>s
1	1.00	1	1	0	-0.001	0	0	0	t	57770	SFFVKDW<br>SER	Mus<br>musculu<br>s
1	1.00	1	1	0.02	0.02	0.02	0.02	0.02	t	109252	GSLPQKSQ<br>RSQDENP<br>V	Rattus<br>norvegic<br>us
1	1.00	1	1	0.02	0.02	0.02	0.02	0.02	t	109252	GSLPQKSQ<br>RSQDENP<br>V	Rattus<br>norvegic<br>us
1	1.00	1	1	-0.03	-0.03	-0.037	-0.03	-0.03	t	159026	NLAIHSDL<br>SYWIESRL	Mus<br>musculu<br>s
1	1.00	1	1	0.03	0.033	0.034	0.03	0.03	t	122857	MGCSSPP<br>CECHQEE	Mus<br>musculu<br>s
1	1.00	1	1	0.02	0.02	0.02	0.02	0.02	t	17843	FSSIPNGT	Oryctola<br>gus<br>cuniculu<br>s
1	1.00	1	1	0	0	0	0	0	t	73661	YEGFSYGV<br>TNKAKGIT	Mus<br>musculu<br>s
1	1.00	1	1	-0.02	-0.018	-0.013	-0.013	-0.02	t	108212	WPPSFWY<br>HPQ	Homo<br>sapiens
1	1.00	1	1	-0.02	-0.016	-0.013	-0.013	-0.02	t	108212	WPPSFWY<br>HPQ	Homo<br>sapiens
1	1.00	1	1	0.05	0.05	0.041	0.046	0.05	t	135990	SASVPALT<br>AVET	Homo<br>sapiens
1	1.00	1	1	0.04	0.042	0.043	0.04	0.038	t	58914	SKPFATDS<br>GA	Homo<br>sapiens
1	1.00	1	1	0	0	0.004	0.003	0	t	7868	DDSEPVLK<br>GVKLHYT	Homo<br>sapiens

1	1.00	1	1	0.02	0.018	0.025	0.023	0.019	t	159043	NTFVVDGPETKECPTQ	Mus musculus
1	1.00	1	1	-0.03	-0.026	-0.02	-0.03	-0.032	t	68757	VGPKASKK	MD
1	1.00	1	1	0.01	0.01	0.007	0.01	0.01	t	25975	IESPPEINTVQVTS	Homo sapiens
1	1.00	1	1	0.02	0.02	0.02	0.02	0.02	t	153257	FNPNKDTWPDANKVG	Mus musculus
1	1.00	1	1	0	0	0	0	0	t	108074	PQVYTLP	Oryctolagus cuniculus
1	1.00	1	1	0	0	0	0	0	t	108074	PQVYTLP	Mus musculus
1	1.00	1	1	-0.02	-0.02	-0.02	-0.02	-0.02	t	127778	PSEFEADLMLGP	Trichosurus vulpecula
1	1.00	1	1	-0.01	-0.014	-0.01	-0.007	-0.01	t	95749	QVYNKLGMYPPN	Oryctolagus cuniculus
1	1.00	1	1	-0.01	-0.01	-0.01	-0.01	-0.01	t	63037	TAVSPTTLR	Mus musculus
1	1.00	1	1	-0.01	-0.01	-0.01	-0.01	-0.01	t	117437	AIALREYRKKMDIPA	Homo sapiens
1	1.00	1	1	0.01	0.01	0.003	0.01	0.01	t	50172	PYSPSQDPYSPSQDP	Oryctolagus cuniculus
1	1.00	1	1	0	0	-0.006	0	-0.002	t	916	AEGDDPAKAAFDSLQ	Mus musculus
1	1.00	1	1	0.01	0.012	0.01	0.011	0.01	t	117112	WPMNDDSTRR	Homo sapiens
1	1.00	1	1	-0.02	-0.024	-0.026	-0.021	-0.018	t	12900	EKVSRRSNFEF	Oryctolagus cuniculus

1	1.00	1	1	-0.02	-0.022	-0.025	-0.017	-0.02	t	14487	ETMTPSGL VIPENAK	Mus musculu s
1	1.00	1	1	0.01	0.01	0.002	0.009	0.01	t	83781	INPWDSKS DYLG	Homo sapiens
1	1.00	1	1	0.01	0.008	0.002	0.009	0.01	t	83781	INPWDSKS DYLG	Mus musculu s
1	1.00	1	1	0.02	0.016	0.019	0.019	0.018	t	59853	SNKEGVA ADGDALN K	Oryctola gus cuniculu s
1	1.00	1	1	0.02	0.02	0.009	0.019	0.02	t	28350	IRNVPHPF VALGLKQ	Mus musculu s
1	1.00	1	1	-0.03	-0.03	-0.037	-0.028	-0.03	t	116927	AVCRTSM CSIQSAPP	Homo sapiens
1	1.00	1	1	-0.02	-0.023	-0.027	-0.02	-0.02	t	108164	TLVCLATG FFPDHVEL	Oryctola gus cuniculu s
1	1.00	1	1	0.02	0.022	0.021	0.017	0.017	t	106512	HILRWGN ESE	Homo sapiens
1	1.00	1	1	0.03	0.029	0.036	0.031	0.031	t	69517	VLGSQE	Homo sapiens
1	1.00	1	1	0.02	0.016	0.013	0.021	0.019	t	131194	ALGAQKE AISPPDAA SAAP	Oryctola gus cuniculu s
1	1.00	1	1	0.02	0.017	0.017	0.02	0.02	t	52139	QQQTAPK APTE	Oryctola gus cuniculu s
1	1.00	1	1	0.01	0.01	0.01	0.01	0.01	t	103245	GVYKVCK GLEKIPLL	Homo sapiens
1	1.00	1	1	0.01	0.01	0.01	0.01	0.01	t	79179	DCFNSKN WEHAQCT	Mus musculu s
1	1.00	1	1	-0.01	-0.01	-0.01	-0.01	-0.01	t	123859	YEALDCIRY	Ovis aries

1	1.00	1	1	-0.01	-0.01	0	-0.009	-0.011	t	75286	YPFMWG GAYCF	Mus musculu s
1	1.00	1	1	-0.01	-0.01	0	-0.009	-0.011	t	75286	YPFMWG GAYCF	Mus musculu s
1	1.00	1	1	0	0	0	0	0	t	137450	SHRLPLK	Mus musculu s
1	1.00	1	1	0.03	0.034	0.022	0.027	0.03	t	47020	PCEQHLM QKIQRDED	Homo sapiens
1	1.00	1	1	0.02	0.018	0.02	0.019	0.02	t	55825	RSELSQLS	Mus musculu s
1	1.00	1	1	0	0.004	0	0.001	0	t	32415	KMVQVVY D	Homo sapiens
1	1.00	1	1	0.04	0.036	0.033	0.039	0.04	t	84644	KQLLTYIEG AYARTRTT	Oryctola gus cuniculu s
1	1.00	1	1	0.03	0.029	0.026	0.03	0.032	t	48900	PPQWKLQ P	Anas platyrhy nchos
1	1.00	1	1	0.02	0.023	0.023	0.02	0.02	t	97313	FSMGAKP T	Mus musculu s
1	1.00	1	1	-0.01	-0.015	-0.021	-0.007	-0.01	t	70261	VPCSTCEG NLACLSLC Q	Bos taurus
1	1.00	1	1	0	0	0	0	0	t	36425	LHSTSPEG N	Homo sapiens
1	1.00	1	1	0	-0.002	0	-0.007	-0.002	t	132668	SNYRAYA	Mus musculu s
1	1.00	1	1	0.02	0.02	0.012	0.02	0.022	t	41177	MAYRFNG IGVTQNVL YE	Mus musculu s
1	1.00	1	1	0	0.002	-0.004	-0.003	-0.001	t	52082	QQPFP	Homo sapiens
1	1.00	1	1	-0.02	-0.02	-0.02	-0.02	-0.018	t	49656	PTIAGA	Mus musculu s

1	1.00	1	1	0.04	0.043	0.028	0.04	0.042	t	43215	NALTQPVDQLLK	Equus caballus
1	1.00	1	1	0	0	0	0	0	t	50535	QDVVVL	Homo sapiens
1	1.00	1	1	-0.02	-0.023	-0.023	-0.02	-0.02	t	7193	CTPPALNCYWPLNDYGF	Oryctolagus cuniculus
1	1.00	1	1	0.01	0.01	0.01	0.01	0.009	t	138189	HILGSTA	Mus musculus
1	1.00	1	1	-0.01	-0.01	-0.007	-0.016	-0.016	t	70792	VRNSWDTNWGDNGY	Homo sapiens
1	1.00	1	1	-0.01	-0.01	-0.01	-0.01	-0.012	t	106770	RER	Mus musculus
1	1.00	1	1	0.02	0.02	0.02	0.02	0.02	t	137923	LMDAAVDVFADG	Homo sapiens
1	1.00	1	1	0.02	0.02	0.02	0.02	0.02	t	133953	WIINNF	Mus musculus
1	1.00	1	1	0	0	0	0	0	t	123859	YEALDCIRY	Ovis aries
1	1.00	1	1	0	0	0	0	0	t	124284	HPLPPAATF	Homo sapiens
1	1.00	1	1	0	0	0	0	0	t	125515	PDLTKVYSTDIFFILEITDN	Homo sapiens
1	1.00	1	1	0	0	0	0	0	t	114885	LCAYSIEFGT	Homo sapiens
1	1.00	1	1	0.02	0.024	0.02	0.027	0.02	t	131867	EPSPESIRRKMEIDD	Homo sapiens
1	1.00	1	1	-0.01	-0.01	-0.015	-0.01	-0.01	t	10803	DWEDRYYRENMYR	Mus musculus
1	1.00	1	1	0	-0.003	-0.001	-0.001	0.002	t	99763	VLGYTDRI	Mus musculus

1	1.00	1	1	0	-0.003	0	0	-0.001	t	69178	VKAETRLNPDLQPTE	Oryctolagus cuniculus
1	1.00	1	1	0	-0.003	0	0	-0.001	t	9524	DNPASTTNKDK	Oryctolagus cuniculus
1	1.00	1	1	0	-0.003	0	0	-0.001	t	9524	DNPASTTNKDK	Oryctolagus cuniculus
1	1.00	1	1	0.01	0.006	0.004	0.01	0.01	t	32416	KMVQVVYDYQ	Oryctolagus cuniculus
1	1.00	1	1	0.01	0.008	0.01	0.009	0.01	t	12280	EGSNTNSV	Mus musculus
1	1.00	1	1	0.02	0.02	0.016	0.014	0.018	t	53409	RDPYSPSQDPYSPSQ	Homo sapiens
1	1.00	1	1	0	0	0	0	0.001	t	122895	RWLPPVYEDGFS	Oryctolagus cuniculus
1	1.00	1	1	0	0.002	0	0.001	-0.001	t	51370	QLINSNGSWHIN	Homo sapiens
1	1.00	1	1	0.02	0.02	0.02	0.02	0.02	t	135496	RYPNQVY	Mus musculus
1	1.00	1	1	0.02	0.014	0.02	0.02	0.02	t	123779	TMELMLSM	Oryctolagus cuniculus
1	1.00	1	1	0	0	-0.005	0	0	t	124925	YYVFFEEQEDEIIGF	Oryctolagus cuniculus
1	1.00	1	1	0.02	0.02	0.02	0.02	0.019	t	56718	SAAYATSSFSSLFTRG	Homo sapiens
1	1.00	1	1	-0.02	-0.02	-0.02	-0.02	-0.021	t	100634	VTNSKKFIDI	Mus musculus
1	1.00	1	1	-0.06	-0.063	-0.06	-0.06	-0.061	t	136058	DKATV	Oryctolagus cuniculus

1	1.00	1	1	-0.02	-0.02	-0.02	-0.02	-0.022	t	19737	GGGAGAGGAGAGGGGR	Oryctolagus cuniculus
1	1.00	1	1	-0.03	-0.028	-0.032	-0.031	-0.03	t	25015	HVAGAAALILSKHPN	Cavia porcellus
1	1.00	1	1	-0.05	-0.047	-0.051	-0.05	-0.052	t	94741	WEQWW	Mus musculus
1	1.00	1	1	0.03	0.028	0.024	0.027	0.03	t	48858	PPPPNPNDPPPPNPND	Mus musculus
1	1.00	1	1	0.03	0.028	0.024	0.027	0.03	t	48858	PPPPNPNDPPPPNPND	Mus musculus
1	1.00	1	1	0.05	0.05	0.05	0.05	0.05	t	115906	PVEPFTESQS	Homo sapiens
1	1.00	1	1	0.03	0.03	0.03	0.03	0.03	t	149077	NATQERY	Sus scrofa
1	1.00	1	1	0	0.002	0	0	-0.001	t	113879	SQRSQDEN	Rattus norvegicus
1	1.00	1	1	0	0.002	0	0	-0.001	t	113628	LRLIHYSYDVN	Rattus norvegicus
1	1.00	1	1	0	0	0	0	-0.001	t	102510	HHQKLVFFAEDVGSNK	Rattus norvegicus
1	1.00	1	1	0	-0.003	0	0	-0.001	t	102510	HHQKLVFFAEDVGSNK	Oryctolagus cuniculus
1	1.00	1	1	0.01	0.01	0.01	0.01	0.01	t	117085	SQMPEYLLKADN	Homo sapiens
1	1.00	1	1	-0.01	-0.01	-0.01	-0.01	-0.01	t	97920	AYTPAYYTKN	Oryctolagus cuniculus
1	1.00	1	1	-0.01	-0.007	-0.01	-0.01	-0.01	t	97920	AYTPAYYTKN	Mus musculus
1	1.00	1	1	-0.01	-0.01	-0.01	-0.01	-0.01	t	97920	AYTPAYYTKN	Mus musculus

1	1.00	1	1	-0.03	-0.03	-0.03	-0.03	-0.03	t	97371	GVAFPLPT	Mus musculus
1	1.00	1	1	-0.05	-0.05	-0.05	-0.05	-0.05	t	107570	PSVFLFP	Homo sapiens
1	1.00	1	1	0.03	0.03	0.03	0.03	0.032	t	108897	EIDGPAGQAEP	Mus musculus
1	1.00	1	1	0.01	0.007	0.01	0.01	0.007	t	107422	GQIPLRFNGEVKNPGSVV	Oryctolagus cuniculus
1	1.00	1	1	0	0.003	-0.009	0	0.001	t	78627	DPDPPDANDPAPPNAN	Mus musculus
1	1.00	1	1	-0.02	-0.02	-0.02	-0.02	-0.02	t	132927	WGANVPS	Homo sapiens
1	1.00	1	1	0.01	0.014	0.01	0.017	0.009	t	107774	DAGPQGKVGPS	Homo sapiens
1	1.00	1	1	0.03	0.023	0.027	0.031	0.028	t	73171	WTWETKGDLAEN	Homo sapiens
1	1.00	1	1	-0.01	-0.01	-0.017	-0.006	-0.01	t	122471	LLTEHRMTWDPAQPPRDL	Homo sapiens
1	1.00	1	1	-0.01	-0.01	-0.017	-0.006	-0.01	t	122471	LLTEHRMTWDPAQPPRDL	Homo sapiens
1	1.00	1	1	-0.01	-0.014	-0.004	-0.011	-0.012	t	6469	CKALQEQCRFPN	Oryctolagus cuniculus
1	1.00	1	1	-0.01	-0.013	-0.003	-0.006	-0.01	t	130622	TGLVAQGTDSIG	Camelus dromedarius
1	1.00	1	1	0	0	0	0	0	t	108651	TGPSTRGRQGSRHE	Homo sapiens
1	1.00	1	1	0	0	0	0	0	t	108651	TGPSTRGRQGSRHE	Homo sapiens
1	1.00	1	1	0.01	0.013	0.002	0.01	0.01	t	12415	EIDGPAGQAEPDRAHYNIVT	Mus musculus

1	1.00	1	1	0.02	0.02	0.021	0.02	0.02	t	57637	SESVEFTK DTQTGMS	Mus musculus
1	1.00	1	1	-0.02	-0.022	-0.02	-0.019	-0.02	t	149519	VRVPVPQL QL	Mus musculus
1	1.00	1	1	0.06	0.06	0.047	0.06	0.063	t	57716	SEYSSQPS S	Homo sapiens
1	1.00	1	1	-0.05	-0.045	-0.045	-0.042	-0.048	t	144190	ADGLAVIG VLMK	Homo sapiens
1	1.00	1	1	-0.05	-0.053	-0.045	-0.042	-0.048	t	144190	ADGLAVIG VLMK	Homo sapiens
1	1.00	1	1	0	0	0	0	-0.001	t	51146	QIVGGVYL	Mus musculus
1	1.00	1	1	0.02	0.02	0.02	0.02	0.02	t	140607	TMYQAIT QT	Mus musculus
1	1.00	1	1	0.02	0.02	0.02	0.02	0.02	t	140607	TMYQAIT QT	Mus musculus
1	1.00	1	1	0.02	0.023	0.02	0.02	0.02	t	120146	QGARVYA YVF	Mus musculus
1	1.00	1	1	0.02	0.02	0.02	0.02	0.02	t	100153	INDQIKFII N	Mus musculus
1	1.00	1	1	0.02	0.02	0.02	0.02	0.02	t	77801	SVKKCLFE KC	Homo sapiens
1	1.00	1	1	0	0	0	0	0	t	115554	VVVPPFLQ PEVMGVS KVKEA	Homo sapiens
1	1.00	1	1	0	0	0	0	0	t	80130	AAGLIMTA EP	Homo sapiens
1	1.00	1	1	-0.01	-0.01	-0.01	-0.01	-0.01	t	22123	GRLGTYRY VSSGER	Homo sapiens
1	1.00	1	1	0	0.003	0.001	0.003	0	t	78139	EGPRAG	Equus caballus

1	1.00	1	1	0.02	0.018	0.02	0.019	0.018	t	123346	KDYYQKWMEE	Homo sapiens
1	1.00	1	1	0.01	0.013	0.005	0.01	0.01	t	78687	IRVRKRKGSNKKAEDTLED	Mus musculus
1	1.00	1	1	0.01	0.01	0.005	0.01	0.01	t	78687	IRVRKRKGSNKKAEDTLED	Mus musculus
1	1.00	1	1	0.05	0.05	0.05	0.05	0.05	t	132377	IERRQQEVET	Homo sapiens
1	1.00	1	1	0	0.003	0.004	0	0	t	94825	GGQFNP	Mus musculus
1	1.00	1	1	0.06	0.058	0.059	0.063	0.062	t	70720	VRFQEAANKQKQELD	Mus musculus
1	1.00	1	1	-0.02	-0.024	-0.028	-0.021	-0.019	t	66902	TTVQQQFSEVWKPFPQSTVR	Oryctolagus cuniculus
1	1.00	1	1	-0.03	-0.03	-0.011	-0.03	-0.04	t	26849	IKMVSSLKTKIT	Mus musculus
1	1.00	1	1	0.02	0.02	0.018	0.02	0.02	t	16057	FGSKLELVKK	Mus musculus
1	1.00	1	1	0.02	0.023	0.018	0.02	0.02	t	16057	FGSKLELVKK	Mus musculus
1	1.00	1	1	-0.03	-0.028	-0.03	-0.023	-0.032	t	134701	KEIRKLQKSTHLLIR	Homo sapiens
1	1.00	1	1	-0.01	-0.012	-0.01	-0.011	-0.012	t	46488	NVSVPSPSSTPLLY	Mus musculus
1	1.00	1	1	0.03	0.036	0.02	0.03	0.03	t	139214	QTKLNLTAEIDQLEQRADNL	Felis catus
1	1.00	1	1	0.04	0.037	0.04	0.039	0.041	t	156601	ADEPNNLNSK	Oryctolagus cuniculus

1	1.00	1	1	0.02	0.018	0.02	0.019	0.02	t	20012	GGSGGSVASGGSVAS	Mus musculus
1	1.00	1	1	0.03	0.03	0.03	0.03	0.03	t	126903	QVINVREG	Homo sapiens
1	1.00	1	1	-0.01	-0.012	-0.011	-0.007	-0.008	t	106862	SPEFRRD	Mus musculus
1	1.00	1	1	-0.02	-0.02	-0.02	-0.02	-0.02	t	30472	KEKLQERL	Homo sapiens
1	1.00	1	1	0.04	0.04	0.04	0.044	0.039	t	134051	FPFSGETVFMSMENPGLWIL	Homo sapiens
1	1.00	1	1	0.03	0.036	0.03	0.03	0.03	t	123491	MGLDLPNLP	Ovis aries
1	1.00	1	1	-0.01	-0.008	-0.025	-0.009	-0.008	t	32862	KPRDTLPPPLNRPPP	Homo sapiens
1	1.00	1	1	-0.02	-0.017	-0.013	-0.02	-0.02	t	106380	EFFRHNS	Mus musculus
1	1.00	1	1	0.02	0.023	0.015	0.02	0.022	t	33161	KRIPLDVAEG	Mus musculus
1	1.00	1	1	0.01	0.01	0.006	0.01	0.01	t	36071	LGDDTGIHVI	Homo sapiens
1	1.00	1	1	0.01	0.01	0.01	0.01	0.01	t	70038	VMYASSG	Oryctolagus cuniculus
1	1.00	1	1	0.01	0.01	0.01	0.01	0.01	t	70038	VMYASSG	Oryctolagus cuniculus
1	1.00	1	1	-0.02	-0.017	-0.027	-0.02	-0.018	t	113183	SYGAPATPAA	Mus musculus
1	1.00	1	1	0	0	0	-0.003	-0.002	t	29810	KAGNEGLEWVEL	Homo sapiens

1	1.00	1	1	0	-0.002	0	-0.001	-0.001	t	136554	PLDLPQIIER	Mus musculus
1	1.00	1	1	0	-0.002	0	-0.001	-0.001	t	136573	QLEKALDCQIYG	Mus musculus
1	1.00	1	1	0	0.011	0	-0.001	-0.001	t	63217	TDQMEVDSGPRKRPLRGG	Oryctolagus cuniculus
1	1.00	1	1	0	-0.002	0	-0.001	-0.001	t	61124	SSIVYEAADMIMHT	Mus musculus
1	1.00	1	1	0	-0.004	0	-0.001	-0.001	t	60663	SRGAPGGGFVPSLQGVP	Oryctolagus cuniculus
1	1.00	1	1	0	0.011	0	-0.001	-0.001	t	60663	SRGAPGGGFVPSLQGVP	Oryctolagus cuniculus
1	1.00	1	1	0	-0.004	0	-0.001	-0.001	t	38345	LNTEPSQLPPTAPP	Oryctolagus cuniculus
1	1.00	1	1	0	-0.004	0	-0.001	-0.001	t	48779	PPFSLSPVPTLGSRSRR	Oryctolagus cuniculus
1	1.00	1	1	0	-0.004	0	-0.001	-0.001	t	56614	RYPHYSLIKPESSL	Oryctolagus cuniculus
1	1.00	1	1	0	-0.002	0	-0.001	-0.001	t	58077	SGHRMAWDMMMNW	Mus musculus
1	1.00	1	1	0	-0.004	0	-0.001	-0.001	t	50977	QGVPESPFSRTGEGLD	Oryctolagus cuniculus
1	1.00	1	1	0	-0.002	0	-0.001	-0.001	t	56962	SARDGIRDAMIGLREEMIEK	Mus musculus
1	1.00	1	1	0	-0.004	0	-0.001	-0.001	t	46859	PALTAVETGATNPL	Oryctolagus cuniculus

1	1.00	1	1	0	0.011	0	-0.001	-0.001	t	29852	KALENKKKQLSAGGKNLS	Oryctolagus cuniculus
1	1.00	1	1	0	-0.004	0	-0.001	-0.001	t	29852	KALENKKKQLSAGGKNLS	Oryctolagus cuniculus
1	1.00	1	1	0	0.011	0	-0.001	-0.001	t	19269	GEGAPPAKRARTDQME	Oryctolagus cuniculus
1	1.00	1	1	0	-0.004	0	-0.001	-0.001	t	6429	CILRQLRHLPSRVRYPHYS	Oryctolagus cuniculus
1	1.00	1	1	-0.05	-0.048	-0.063	-0.05	-0.05	t	18536	GACAPYRRLHLCD	Rattus norvegicus
1	1.00	1	1	0.01	0.01	0.01	0.01	0.01	t	43190	NAKATYEAALKQYEADL	Mus musculus
1	1.00	1	1	-0.01	-0.012	-0.01	-0.011	-0.01	t	45695	NRLGGLFNFGPKQKI	Mus musculus
1	1.00	1	1	-0.01	-0.019	-0.009	-0.01	-0.01	t	109030	GAQGPRGE	Macaca mulatta
1	1.00	1	1	0	0.002	-0.002	-0.003	-0.002	t	45550	NQFLPYPYYAKPAA	Homo sapiens
1	1.00	1	1	0.04	0.042	0.046	0.047	0.04	t	2039	AINPESDQPDLSNF	Homo sapiens
1	1.00	1	1	-0.04	-0.044	-0.04	-0.043	-0.04	t	5109	ATPYHPPATP	Oryctolagus cuniculus
1	1.00	1	1	0.05	0.05	0.05	0.05	0.05	t	103260	HRSDGSGDTFLFTQY	Homo sapiens
1	1.00	1	1	0.01	0.01	0.008	0.01	0.01	t	114555	QSENDHRRSIVRVEG	Homo sapiens

1	1.00	1	1	0.01	0.01	0.01	0.01	0.01	t	133786	CMTSPWRC	Mus musculus
1	1.00	1	1	0.01	0.01	0.01	0.01	0.01	t	99042	VLQQSTYQLLQE	Homo sapiens
1	1.00	1	1	0.01	0.01	0.01	0.01	0.01	t	47650	PGFMFSRPSDPAFAA	Mus musculus
1	1.00	1	1	0.01	0.01	0.01	0.01	0.01	t	47650	PGFMFSRPSDPAFAA	Mus musculus
1	1.00	1	1	-0.01	-0.01	-0.01	-0.01	-0.01	t	132691	TCSADGRTLFYNLCL	Mus musculus
1	1.00	1	1	-0.01	-0.01	-0.01	-0.01	-0.01	t	99752	VEYTKY	Oryctolagus cuniculus
1	1.00	1	1	-0.01	-0.01	-0.01	-0.01	-0.01	t	99436	LPHFNSKAMVIVVVN	Homo sapiens
1	1.00	1	1	-0.01	-0.007	-0.01	-0.01	-0.01	t	97919	AYTPAYYTKD	Mus musculus
1	1.00	1	1	-0.01	-0.01	-0.01	-0.01	-0.01	t	97919	AYTPAYYTKD	Mus musculus
1	1.00	1	1	-0.04	-0.034	-0.033	-0.033	-0.042	t	127671	GPDRSPRQPPVLPAA	Homo sapiens
1	1.00	1	1	0.01	0.01	0.01	0.01	0.009	t	137741	DTPLTHAALTLV	Mus musculus
1	1.00	1	1	-0.02	-0.02	-0.02	-0.018	-0.022	t	52121	QQQPFPPQQP	Homo sapiens
1	1.00	1	1	-0.02	-0.016	-0.022	-0.02	-0.021	t	61589	STEQNVPDPQVG	Cavia porcellus
1	1.00	1	1	0.01	0.012	0.011	0.011	0.01	t	68541	VFPDLG	Homo sapiens
1	1.00	1	1	0.01	0.01	0.007	0.01	0.01	t	67059	TVLAQALVRE	Homo sapiens

1	1.00	1	1	-0.04	-0.037	-0.045	-0.04	-0.038	t	51799	QPDLIPL	Mus musculus
1	1.00	1	1	-0.04	-0.04	-0.045	-0.04	-0.038	t	51799	QPDLIPL	Oryctolagus cuniculus
1	1.00	1	1	0	0.005	0	0.001	0	t	97018	TVTFKATAAT	Homo sapiens(human)
1	1.00	1	1	0.01	0.01	0.01	0.01	0.011	t	50929	QGPGAP	Mus musculus
1	1.00	1	1	0	0	-0.005	-0.002	-0.003	t	142472	SATAAETAKNNAVS	Homo sapiens
1	1.00	1	1	-0.01	-0.007	-0.01	-0.01	-0.011	t	72889	WNK	Mus musculus
1	1.00	1	1	0	-0.003	-0.013	0	0	t	106009	PRGVTHDQ	Oryctolagus cuniculus
1	1.00	1	1	0.02	0.02	0.02	0.02	0.023	t	126068	FVFEEF	Rattus norvegicus
1	1.00	1	1	0	-0.002	-0.001	0.003	0	t	50027	PWFLAHHTGGD	Sus scrofa
1	1.00	1	1	0	-0.002	0	0.002	0	t	110460	YEQLNDSSEEEDEIDG	Mus musculus
1	1.00	1	1	0	0	0	0.002	0	t	18172	FVGAGIAGAAVGSI	Homo sapiens
1	1.00	1	1	0	0.002	-0.007	0.001	0	t	43084	MYRVFEVSVIRNPGL	Homo sapiens
1	1.00	1	1	0	0.002	-0.01	0.007	0.002	t	138886	NVGGDNVDIHSIVPVGQDPH	Homo sapiens
1	1.00	1	1	0	0	0	0	0	t	133623	KPGEMR	Mus musculus
1	1.00	1	1	0	0	0	0	0	t	133623	KPGEMR	Mus musculus

1	1.00	1	1	0	0	0	0	0	t	133623	KPGEMR	Mus musculus
1	1.00	1	1	0	0	0	0	0	t	133623	KPGEMR	Mus musculus
1	1.00	1	1	-0.01	-0.01	-0.01	-0.01	-0.011	t	72890	WNKP	Oryctolagus cuniculus
1	1.00	1	1	-0.02	-0.02	-0.02	-0.02	-0.02	t	132793	DLQEAKYETFS	Mus musculus
1	1.00	1	1	0.03	0.027	0.027	0.03	0.03	t	97536	NVTTNQTIQ	Oryctolagus cuniculus
1	1.00	1	1	-0.01	-0.013	-0.008	-0.01	-0.012	t	57184	SDEVRKHTYNLTIA	Oryctolagus cuniculus
1	1.00	1	1	0.01	0.007	0.01	0.01	0.007	t	144671	GREGYPADGGYCNTECTL	Oryctolagus cuniculus
1	1.00	1	1	0	-0.011	0	0	0.002	t	156862	DVGDPLR	Oryctolagus cuniculus
1	1.00	1	1	0.06	0.062	0.044	0.054	0.06	t	142217	LDASREAKKQLEAEQQKLEE	Homo sapiens
1	1.00	1	1	0	0	0	0	0	t	10800	DWEDRYY	Mus musculus
1	1.00	1	1	-0.01	-0.01	-0.022	-0.01	-0.008	t	120984	TESDIKRNHK	Mus musculus
1	1.00	1	1	0	-0.003	0	0	0.002	t	140834	DAVKGYLAGKGV	Oryctolagus cuniculus
1	1.00	1	1	0.1	0.1	0.094	0.1	0.103	t	1157	AESEE	Homo sapiens
1	1.00	1	1	-0.02	-0.02	-0.02	-0.02	-0.02	t	142431	KKAEEAKKIVDKI	Homo sapiens

1	1.00	1	1	0	0	0	0.004	0	t	135947	KTGHPRYFNQLS	Homo sapiens
1	1.00	1	1	-0.01	-0.002	-0.01	-0.01	-0.012	t	156911	LGMSTNPGPRIL	Gallus gallus
1	1.00	1	1	-0.04	-0.038	-0.041	-0.033	-0.039	t	144710	KNPWPNVDAH	Homo sapiens
1	1.00	1	1	-0.01	-0.006	-0.013	-0.009	-0.011	t	103549	RHGLSSLGWNLCRIF	Homo sapiens
1	1.00	1	1	0	0	0	0	0	t	123078	DFPKLKEFK	Ovis aries
1	1.00	1	1	0	0	0	0	0	t	103286	ISMIDGPAPDGYPII	Homo sapiens
1	1.00	1	1	0	0	0	0	0	t	115874	NQFLPYPYYA	Homo sapiens
1	1.00	1	1	-0.02	-0.02	-0.02	-0.02	-0.02	t	115935	QYVLSRYPSY	Homo sapiens
1	1.00	1	1	-0.04	-0.04	-0.04	-0.04	-0.04	t	135766	TIYNNMFC	Homo sapiens
1	1.00	1	1	0	0	0	0.001	0	t	32890	KPTDGN	Mus musculus
1	1.00	1	1	0.01	0.01	0.01	0.01	0.01	t	125384	DSHYDYTICGDSEDMVCTPK	Homo sapiens
1	1.00	1	1	0	0.003	0	0	0.001	t	72504	WGQGGSH	Mus musculus
1	1.00	1	1	0	-0.003	0	0	-0.001	t	137759	NQEDAEAITHLF	Oryctolagus cuniculus
1	1.00	1	1	0	0	0	0	-0.001	t	75441	YPYDVPDYA	Rattus norvegicus
1	1.00	1	1	0	-0.003	0	0	-0.001	t	79775	DDATVAIRSAINNLIVELIR	Oryctolagus cuniculus
1	1.00	1	1	0	-0.003	0	0	-0.001	t	64628	TKNTNNNLTL	Oryctolagus cuniculus

1	1.00	1	1	0	-0.003	0	0	-0.001	t	75791	YSLFQKEKMVL	Oryctolagus cuniculus
1	1.00	1	1	0	-0.017	0	0	-0.001	t	55818	RSDYKFYEDANGTRDHKKG	Oryctolagus cuniculus
1	1.00	1	1	0	-0.003	0	0	-0.001	t	43242	NANPNANPNANP	Oryctolagus cuniculus
1	1.00	1	1	0	0	0	0	-0.001	t	36958	LKMADPNRFRGKD	Mus musculus
1	1.00	1	1	0	0	0	0	-0.001	t	51371	QLINTNGSWHIN	Rattus norvegicus
1	1.00	1	1	0	-0.003	0	0	-0.001	t	52929	QYIKANSKFIGITE	Oryctolagus cuniculus
1	1.00	1	1	0	-0.003	0	0	-0.001	t	43242	NANPNANPNANP	Oryctolagus cuniculus
1	1.00	1	1	0	0	0	0	-0.001	t	36958	LKMADPNRFRGKD	Mus musculus
1	1.00	1	1	0	0.003	-0.007	0	0.001	t	41430	MERVVEQMCITQYQRESQAY	Mus musculus
1	1.00	1	1	-0.01	-0.006	-0.01	-0.009	-0.01	t	12625	EKAFREL	Homo sapiens
1	1.00	1	1	-0.01	-0.01	-0.01	-0.01	-0.01	t	66060	TRLNPD	Mus musculus
1	1.00	1	1	-0.01	-0.008	-0.01	-0.01	-0.01	t	28911	ITGHRMAWDMMMNWS	Homo sapiens
1	1.00	1	1	-0.01	-0.01	-0.01	-0.01	-0.009	t	125529	PVEGPAVICRCC	Oryctolagus cuniculus

1	1.00	1	1	-0.02	-0.022	-0.04	-0.027	-0.02	t	141663	GRSLEGVYVPPRRQQL	Rattus norvegicus
1	1.00	1	1	0.03	0.034	0.019	0.03	0.03	t	114011	VTTVTTHNNPTFTDPSVLQP	Cavia porcellus
1	1.00	1	1	-0.02	-0.022	-0.022	-0.021	-0.021	t	97223	AWNPSLLG	Mus musculus
1	1.00	1	1	0.02	0.02	0.02	0.023	0.02	t	34910	LAPNTPRPPAQAPAKDMPSG	Homo sapiens
1	1.00	1	1	0.01	0.01	0.01	0.012	0.01	t	73614	YEAADAILHTP	Homo sapiens
1	1.00	1	1	0	0.012	0.001	-0.001	0	t	6139	CDSSDRPRVYTIT	Gallus gallus
1	1.00	1	1	0.03	0.027	0.018	0.031	0.032	t	1460	AGCLIGAEHVDTSYECD	Homo sapiens
1	1.00	1	1	0	-0.012	0	0	-0.001	t	71506	VTSTLTSLFRPGASQKIQL	Mus musculus
1	1.00	1	1	0	0	-0.004	0	0	t	61862	STRGRQTVKENKGT	Myodes glareolus
1	1.00	1	1	0	-0.004	-0.004	0	0	t	63215	TDPTGIEPDDHLKE	Oryctolagus cuniculus
1	1.00	1	1	0	-0.004	-0.004	0	0	t	64645	TKPTDPTGIEPDDH	Oryctolagus cuniculus
1	1.00	1	1	0	0	-0.004	0	0	t	64645	TKPTDPTGIEPDDH	Myodes glareolus
1	1.00	1	1	0	0	-0.004	0	0	t	66194	TSFEDINGIRRPKH	Myodes glareolus
1	1.00	1	1	0	-0.004	-0.004	0	0	t	61192	SSLRYGNVLDVNAI	Oryctolagus cuniculus
1	1.00	1	1	0	0	-0.004	0	0	t	63215	TDPTGIEPDDHLKE	Myodes glareolus

1	1.00	1	1	0	0	-0.004	0	0	t	72183	VYVIGFTLP IILKA	Myodes glareolu s
1	1.00	1	1	0	0	-0.004	0	0	t	41979	MLKRNKIY FMQRQD	Myodes glareolu s
1	1.00	1	1	0	0	-0.004	0	0	t	52660	QTVSALED KLADYK	Myodes glareolu s
1	1.00	1	1	0	0	-0.004	0	0	t	55007	RNKIYFMQ RQDVLD	Myodes glareolu s
1	1.00	1	1	0	0	-0.004	0	0	t	28144	IQEDITRHE QQLIV	Myodes glareolu s
1	1.00	1	1	0	-0.004	-0.004	0	0	t	14751	EVKPGTPA QEIEML	Oryctola gus cuniculu s
1	1.00	1	1	0	0	-0.004	0	0	t	11370	EDKLADYK RRMADA	Myodes glareolu s
1	1.00	1	1	0	0	-0.004	0	0	t	11354	EDINGIRR PKHLYV	Myodes glareolu s
1	1.00	1	1	0	0	-0.004	0	0	t	26575	IILKALYML STRGR	Myodes glareolu s
1	1.00	1	1	0	0	-0.004	0	0	t	29578	IYFMQRQ DVLDKNH	Myodes glareolu s
1	1.00	1	1	0	0	-0.004	0	0	t	22178	GRQTVKE NKGTRIR	Myodes glareolu s
1	1.00	1	1	0	0	-0.004	0	0	t	30597	KERSSLRY GNVLDV	Myodes glareolu s
1	1.00	1	1	0	0	-0.004	0	0	t	22799	GTRIRFKD DTSFED	Myodes glareolu s
1	1.00	1	1	0	0	-0.004	0	0	t	26227	IGFTLPIILK ALYM	Myodes glareolu s
1	1.00	1	1	0	0	-0.004	0	0	t	7885	DDVNKNT LQARQQT	Myodes glareolu s
1	1.00	1	1	0	0	-0.004	0	0	t	8804	DITRHEQQ LIVARQ	Myodes glareolu s
1	1.00	1	1	0	0	0	0	0.001	t	108075	PREPQVY	Homo sapiens

1	1.00	1	1	0.01	0.01	0.01	0.01	0.01	t	76325	YVKRVKTW	Homo sapiens
1	1.00	1	1	0.03	0.028	0.03	0.031	0.03	t	149516	VPVPQLQLQN	Mus musculus
1	1.00	1	1	-0.01	-0.01	-0.01	-0.01	-0.01	t	145272	KDLLPSRDRQHLPLI	Homo sapiens
1	1.00	1	1	0.02	0.02	0.026	0.02	0.021	t	46443	NVPEKQT	Mus musculus
1	1.00	1	1	0	0.004	0.004	0.001	0	t	23249	GWGNGC	Homo sapiens
1	1.00	1	1	0	0	-0.01	0	0.001	t	24012	HHPSTNQEQTSLYVQAS	Mus musculus
1	1.00	1	1	0.01	0.01	0.01	0.01	0.01	t	149090	NSTQGPV	Sus scrofa
1	1.00	1	1	0.01	0.01	0.01	0.01	0.01	t	132934	WGQNTSM	Homo sapiens
1	1.00	1	1	0.01	0.01	0.01	0.01	0.01	t	112407	YFCHQYL	Homo sapiens
1	1.00	1	1	0.01	0.01	0.01	0.01	0.01	t	113467	GQGQQPGQLQQPAQ	Homo sapiens
1	1.00	1	1	0	0.003	0	0	0	t	38824	LQDPRVRGL	Mus musculus
1	1.00	1	1	-0.01	-0.01	-0.01	-0.01	-0.013	t	51827	QPFPQPQLPYPQPQ	Mus musculus
1	1.00	1	1	-0.01	-0.01	-0.018	-0.01	-0.008	t	46050	NTAEYWGFK	Mus musculus
1	1.00	1	1	0.02	0.018	0.013	0.013	0.017	t	130816	ESKAISDAQITASSYFTNM	Mus musculus
1	1.00	1	1	0	0	-0.008	0	0	t	4220	ARQKLKDAER	Mus musculus
1	1.00	1	1	0	0.003	-0.008	0	0	t	4220	ARQKLKDAER	Mus musculus

1	1.00	1	1	0.02	0.024	0.02	0.021	0.02	t	58082	SGIGPEKA	Homo sapiens
1	1.00	1	1	0.04	0.04	0.04	0.04	0.04	t	142427	KAEKDAEA AQ	Homo sapiens
1	1.00	1	1	0.02	0.02	0.02	0.02	0.021	t	29285	IVGGVYLL	Homo sapiens
1	1.00	1	1	-0.04	-0.038	-0.049	-0.037	-0.038	t	63098	TCRRMLAF LKDKENR	Homo sapiens
1	1.00	1	1	0	0	0	0.002	0	t	33365	KSKFGYGA KDVRNL	Homo sapiens
1	1.00	1	1	-0.02	-0.02	-0.02	-0.018	-0.02	t	136003	TRKDINTV TTVA	Homo sapiens
1	1.00	1	1	-0.03	-0.03	-0.046	-0.03	-0.03	t	16394	FKKYNDKL PLYISNPN YKVN	Mus musculu s
1	1.00	1	1	-0.03	-0.03	-0.046	-0.03	-0.03	t	16394	FKKYNDKL PLYISNPN YKVN	Mus musculu s
1	1.00	1	1	0.05	0.054	0.05	0.053	0.051	t	117315	KALSVETE KLLKYLEAV	Homo sapiens
1	1.00	1	1	0.02	0.02	0.02	0.02	0.02	t	78141	EKAEEVEK	Homo sapiens
1	1.00	1	1	0	0	0	0	0	t	151019	LCKLRGVA PL	Mus musculu s
1	1.00	1	1	0	0	-0.008	-0.001	0	t	90217	VNKIAEPE ARLI	Homo sapiens
1	1.00	1	1	0.01	0.009	0.015	0.011	0.01	t	819	AEAEGGT W	Homo sapiens
1	1.00	1	1	0	0	0	0	0	t	42755	MTDVIRYR YSNIYEAS DK	Aotus nancyma ae
1	1.00	1	1	-0.02	-0.02	-0.02	-0.02	-0.02	t	142381	AAEFATEV KKAT	Homo sapiens
1	1.00	1	1	0.01	0.01	0.01	0.01	0.009	t	137733	DTALTIQA LKLV	Mus musculu s

1	1.00	1	1	0.02	0.02	0.02	0.02	0.02	t	117408	YLQSASIFSK	Homo sapiens
1	1.00	1	1	-0.02	-0.022	-0.02	-0.023	-0.019	t	72249	WAP	Mus musculus
1	1.00	1	1	0.04	0.04	0.04	0.04	0.04	t	114847	GTNISKEHDG	Homo sapiens
1	1.00	1	1	0.02	0.02	0.02	0.02	0.02	t	72553	WHTTIPPVFDEP	Rattus norvegicus
1	1.00	1	1	0.02	0.02	0.02	0.02	0.02	t	41329	MEECASQLPYIEQGMQLAEQ	Homo sapiens
1	1.00	1	1	0.03	0.027	0.034	0.03	0.028	t	95031	NAYANEEATKL	Oryctolagus cuniculus
1	1.00	1	1	-0.02	-0.02	-0.02	-0.02	-0.02	t	101079	NKPVEPTYEV	Mus musculus
1	1.00	1	1	-0.01	-0.006	-0.01	-0.009	-0.01	t	137811	AVSLGQNV	Homo sapiens
1	1.00	1	1	-0.02	-0.018	-0.037	-0.019	-0.016	t	20084	GGVYLLPRRGPRLGV	Homo sapiens
1	1.00	1	1	-0.04	-0.038	-0.023	-0.039	-0.049	t	134682	GPSRRGPSLGASSHQ	Homo sapiens
1	1.00	1	1	0.02	0.02	0.018	0.017	0.018	t	139881	RGDKSRPPVWYVEGE	Mus musculus
1	1.00	1	1	0.01	0.013	0.009	0.01	0.01	t	97902	ATYEAALKQY	Mus musculus
1	1.00	1	1	0.05	0.052	0.045	0.051	0.052	t	15439	FDPLVAEEDEREIS	Homo sapiens
1	1.00	1	1	0.03	0.034	0.03	0.037	0.032	t	117364	RPDAEYWNSQKDLLEQRRAA	Homo sapiens
1	1.00	1	1	-0.03	-0.033	-0.03	-0.03	-0.03	t	153415	LPKFHKSPHFYR	Oryctolagus cuniculus

1	1.00	1	1	-0.02	-0.016	-0.015	-0.013	-0.02	t	133900	NEHPVFAYLKDKLP	Homo sapiens
1	1.00	1	1	0.03	0.033	0.036	0.031	0.031	t	105904	KLVFFAEDV	Mus musculus
1	1.00	1	1	0.01	0.013	0.01	0.01	0.01	t	26046	IFDT	Mus musculus
1	1.00	1	1	0.01	0.013	0.01	0.01	0.01	t	26046	IFDT	Mus musculus
1	1.00	1	1	0.01	0.018	0.012	0.01	0.012	t	74045	YGLLAVDPLFKP	Gallus gallus
1	1.00	1	1	0	0	0.001	0	0	t	29696	IYSYFPSVI	Mus musculus
1	1.00	1	1	0.03	0.03	0.03	0.03	0.03	t	149087	NPTQSPP	Sus scrofa
1	1.00	1	1	0.03	0.03	0.03	0.03	0.03	t	114818	FCNAVVESNG	Homo sapiens
1	1.00	1	1	0.01	0.01	0.01	0.01	0.011	t	43729	NEREDERT	Oryctolagus cuniculus
1	1.00	1	1	0.01	0.01	0.01	0.01	0.01	t	119516	RKLAHPGTS	Homo sapiens
1	1.00	1	1	0.01	0.01	0.01	0.01	0.01	t	111966	YKSNNIARMRLYDPN	Oryctolagus cuniculus
1	1.00	1	1	0.01	0.01	0.01	0.01	0.01	t	25452	IATQEQPAFD	Mus musculus
1	1.00	1	1	-0.01	-0.01	-0.01	-0.01	-0.01	t	110585	GAPGIAGAPGFPGPR	Mus musculus
1	1.00	1	1	-0.01	-0.007	-0.021	-0.01	-0.008	t	111634	PNNNKILVPKVSGLQ	Mus musculus
1	1.00	1	1	0.01	0.012	0.017	0.017	0.012	t	145235	GTWICDFKSCSW	Homo sapiens

1	1.00	1	1	0.01	0.008	0.002	0.007	0.01	t	9039	DLDASREAKKQLEAEHQK	Mus musculus
1	1.00	1	1	0	0	0	0	-0.001	t	28299	IRGDTFATR	Mus musculus
1	1.00	1	1	0	0	0	0	-0.001	t	7492	DAEFRHDSGYEVHHQKL	Mus musculus
1	1.00	1	1	-0.01	-0.01	-0.01	-0.01	-0.011	t	103830	CHAVKVGNPSC	Oryctolagus cuniculus
1	1.00	1	1	0	0.002	0.003	0.001	0	t	5344	AVGGYAISIS	Homo sapiens
1	1.00	1	1	-0.02	-0.018	-0.02	-0.013	-0.02	t	108141	SPSDAKSHVRVADLI	Homo sapiens
1	1.00	1	1	-0.02	-0.016	-0.02	-0.017	-0.02	t	19737	GGGAGAGGAGAGGGGR	Homo sapiens
1	1.00	1	1	-0.01	-0.006	-0.015	-0.01	-0.01	t	3383	ANPPDHSAPLGVTRPSAPPL	Cavia porcellus
1	1.00	1	1	0.01	0.014	0.019	0.017	0.01	t	112422	YWASTRE	Homo sapiens
1	1.00	1	1	0	0	0.007	0.004	0	t	63096	TCRNMWSGT	Homo sapiens
1	1.00	1	1	0.01	0.01	0.008	0.01	0.01	t	125051	FQSKANCLNT	Mus musculus
1	1.00	1	1	0	0.003	0	0	0	t	123063	CPTMTRVLQGVLPALPQ	Mus musculus
1	1.00	1	1	0	0	0	0	0	t	123063	CPTMTRVLQGVLPALPQ	Mus musculus
1	1.00	1	1	0	-0.002	0.005	-0.001	-0.003	t	18363	FWRGDLVFDFQV	Mus musculus

1	1.00	1	1	-0.03	-0.026	-0.031	-0.029	-0.032	t	147074	KRKQNLVQAWQAK	Homo sapiens
1	1.00	1	1	0	0	0.005	0	0	t	124925	YYVFFEEQEDEIIGF	Homo sapiens
1	1.00	1	1	-0.02	-0.018	-0.02	-0.019	-0.02	t	117018	KIVMFWHEPVYA	Homo sapiens
1	1.00	1	1	0.02	0.018	0.022	0.02	0.02	t	122895	RWLPPVYEDGFS	Oryctolagus cuniculus
1	1.00	1	1	0.01	0.008	0.008	0.009	0.011	t	103658	TIYYQKFKIPQILK	Mus musculus
1	1.00	1	1	-0.01	-0.01	-0.012	-0.01	-0.011	t	27961	IPNKKPGKKTTT	Mus musculus
1	1.00	1	1	-0.02	-0.02	-0.02	-0.02	-0.019	t	115255	FQINNKIWCK	Homo sapiens
1	1.00	1	1	0.02	0.023	0.01	0.023	0.02	t	74004	YGGIPGGISTPITQQSEK	Equus caballus
1	1.00	1	1	-0.03	-0.028	-0.031	-0.029	-0.028	t	120810	MVNKSRMMPAV	Homo sapiens
1	1.00	1	1	0.04	0.04	0.04	0.04	0.04	t	115839	KYPVEPFTES	Homo sapiens
1	1.00	1	1	0.04	0.04	0.04	0.04	0.04	t	112222	LYSSDHK	Homo sapiens
1	1.00	1	1	0.02	0.02	0.02	0.02	0.02	t	123455	LPNLPYYID	Ovis aries
1	1.00	1	1	0.02	0.02	0.02	0.02	0.02	t	114529	QETTLVRSSRSSSER	Homo sapiens
1	1.00	1	1	0.02	0.02	0.02	0.02	0.02	t	112402	WNDNKFY	Homo sapiens
1	1.00	1	1	-0.01	-0.004	-0.003	-0.003	-0.012	t	127729	LRWAERQVGALGAES	Homo sapiens
1	1.00	1	1	0	0	0	0	0	t	123858	YDPKFEQLK	Ovis aries

1	1.00	1	1	0	0	0	0	0	t	107026	YQLPPKF WFISP	Mus musculus
1	1.00	1	1	-0.02	-0.02	-0.018	-0.02	-0.019	t	40563	LVRMKRA	Mus musculus
1	1.00	1	1	-0.04	-0.04	-0.04	-0.04	-0.04	t	149431	QPFPPQQ PYP	Homo sapiens
1	1.00	1	1	0	0	0.002	0	-0.001	t	6479	CKGKIQAL CENPEWA	Mus musculus
1	1.00	1	1	0.03	0.033	0.031	0.03	0.03	t	22894	GTYETGNK	Mus musculus
1	1.00	1	1	0	0.002	0	-0.003	0	t	49078	PQQPF	Homo sapiens
1	1.00	1	1	0.03	0.028	0.028	0.033	0.033	t	105869	IAGFKGEQ GPK	Mus musculus
1	1.00	1	1	-0.01	-0.008	-0.012	-0.003	-0.01	t	98784	GAKGKAA LLRELSDV VPNLN	Homo sapiens
1	1.00	1	1	0.01	0.01	0.017	0.01	0.007	t	140567	KEAWLDS TKAT	Mus musculus
1	1.00	1	1	0.02	0.02	0.02	0.02	0.019	t	59377	SLNWSDR TI	Rattus norvegicus
1	1.00	1	1	0.01	0.008	0	0.013	0.012	t	8217	DEYCSPDH NCQSNCK DSGEG	Mus musculus
1	1.00	1	1	-0.01	-0.008	-0.007	-0.009	-0.01	t	55680	RRPQDVK FPG	Homo sapiens
1	1.00	1	1	0	0	0	0	-0.001	t	30093	KCTSDQDE QFIPKGCS K	Mus musculus

1	1.00	1	1	-0.01	-0.007	-0.01	-0.01	-0.01	t	49656	PTIAGA	Mus musculus
1	1.00	1	1	-0.01	-0.007	-0.01	-0.01	-0.01	t	49656	PTIAGA	Mus musculus
1	1.00	1	1	0.05	0.056	0.05	0.05	0.05	t	123705	RIEDLPKIK	Ovis aries
1	1.00	1	1	0	-0.004	0	-0.003	-0.003	t	138304	ATVQLPQ	Oryctolagus cuniculus
1	1.00	1	1	0	-0.002	0	-0.003	-0.003	t	109917	SHQESTRGRSRGRSGRSGS	Mus musculus
1	1.00	1	1	0	0.004	-0.003	0.001	-0.001	t	103170	FGDVDAHGAMIRAQA	Homo sapiens
1	1.00	1	1	0.01	0.01	0.01	0.01	0.01	t	36428	LHSTSSEGN	Homo sapiens
1	1.00	1	1	0.03	0.03	0.028	0.03	0.029	t	153265	ILETLPANPPPASTN	Mus musculus
1	1.00	1	1	0	0	0	0	-0.001	t	36956	LKMADPNRFRG	Mus musculus
1	1.00	1	1	0	0	0	0	-0.001	t	36956	LKMADPNRFRG	Mus musculus
1	1.00	1	1	0.01	0.01	0.01	0.013	0.01	t	51145	QIVGGV	Homo sapiens
1	1.00	1	1	0.01	0.01	0.01	0.01	0.011	t	119658	TYGIVLLSGV	Homo sapiens
1	1.00	1	1	0.04	0.04	0.033	0.036	0.04	t	33842	KTRNQMGVSE	Homo sapiens
1	1.00	1	1	0	0	0	0.004	0	t	136010	VMIKSLPALNSPTVEEC	Homo sapiens
1	1.00	1	1	-0.03	-0.032	-0.001	-0.033	-0.04	t	44174	NHVRIPIGYWAV	Mus musculus
1	1.00	1	1	-0.02	-0.02	-0.02	-0.02	-0.022	t	118129	SEEELKTHISKG	Homo sapiens

1	1.00	1	1	0.02	0.017	0.018	0.02	0.02	t	73450	YASSGIGP EK	Oryctola gus cuniculu s
1	1.00	1	1	0.02	0.02	0.018	0.02	0.02	t	73450	YASSGIGP EK	Oryctola gus cuniculu s
1	1.00	1	1	0	0	0	0	-0.001	t	141559	SDEALPLG SPR	Mus musculu s
1	1.00	1	1	0	0	0	0	-0.001	t	118116	RSGKPRGY AF	Homo sapiens
1	1.00	1	1	-0.04	-0.04	-0.04	-0.04	-0.04	t	55185	RPKHPIKH QG	Homo sapiens
1	1.00	1	1	0.02	0.025	0.03	0.022	0.017	t	157463	KKETLICS	Homo sapiens
1	1.00	1	1	-0.01	-0.01	-0.005	-0.011	-0.01	t	54736	RLRLVDAD	Homo sapiens
1	1.00	1	1	0.06	0.064	0.061	0.061	0.061	t	59350	SLNEEQ	Homo sapiens
1	1.00	1	1	0	-0.005	0.001	0	0	t	108632	SKEENTPE VKA	Canis lupus
1	1.00	1	1	0	0	0.001	0	0	t	108632	SKEENTPE VKA	Canis lupus
1	1.00	1	1	0.05	0.05	0.05	0.05	0.05	t	96209	EDKDEDEE DE	Homo sapiens
1	1.00	1	1	0.01	0.01	0.01	0.01	0.008	t	10024	DRNMYLN T	Mus musculu s
1	1.00	1	1	0.01	0.01	0.01	0.01	0.01	t	141754	SSSKTWG APLYY	Mus musculu s
1	1.00	1	1	-0.01	-0.01	-0.01	-0.01	-0.01	t	112389	VPDRFTG	Homo sapiens
1	1.00	1	1	0.01	0.01	0.01	0.01	0.009	t	130924	KLFPEEDE MRTETQR	Homo sapiens

1	1.00	1	1	0.01	0.012	0.007	0.011	0.009	t	103493	PPTPISVIF RSDKSG	Homo sapiens
1	1.00	1	1	0	0	-0.007	0	0	t	95543	LCPNNLCC SQ	Homo sapiens
1	1.00	1	1	0.04	0.04	0.04	0.04	0.041	t	72548	WHQTTPQ PYLPG	Rattus norvegic us
1	1.00	1	1	-0.01	-0.01	-0.01	-0.01	-0.01	t	129828	RSGKG	Homo sapiens
1	1.00	1	1	0.02	0.02	0.02	0.02	0.02	t	137882	GQNVLSA D	Homo sapiens
1	1.00	1	1	0.06	0.06	0.069	0.06	0.058	t	79851	GVINE	Mus musculu s
1	1.00	1	1	0	0	0	0	0	t	51371	QLINTNGS WHIN	Homo sapiens
1	1.00	1	1	-0.02	-0.023	-0.03	-0.02	-0.017	t	6762	CPFIKPEVK PGTPA	Oryctola gus cuniculu s
1	1.00	1	1	0	0.002	0.004	0.003	0.002	t	18508	GAAILTPT PVNPSTA	Homo sapiens
1	1.00	1	1	0.04	0.036	0.032	0.037	0.04	t	2816	ALPKNTVG GTIL	Oryctola gus cuniculu s
1	1.00	1	1	0	-0.002	-0.001	-0.003	-0.002	t	122269	ARQNLKD AGE	Mus musculu s
1	1.00	1	1	-0.02	-0.017	-0.029	-0.02	-0.018	t	54749	RLRVTHQT	Mus musculu s
1	1.00	1	1	-0.01	-0.007	-0.007	-0.01	-0.009	t	142291	SHLQIYNL	Sus scrofa
1	1.00	1	1	-0.02	-0.02	-0.02	-0.02	-0.02	t	103664	TPPAPAPP PFRLPLL	Homo sapiens

1	1.00	1	1	0.01	0.013	0.009	0.01	0.01	t	6456	CITQYERESQAYYQRGS	Mus musculus
1	1.00	1	1	0.03	0.028	0.033	0.033	0.03	t	14019	ERLGDY	Mus musculus
1	1.00	1	1	-0.01	-0.011	-0.02	-0.007	-0.01	t	32677	KPDLTGTSKSYVRSL	Homo sapiens
1	1.00	1	1	0.01	0.006	0.003	0.009	0.01	t	61337	SSSDSQTITNPA	Oryctolagus cuniculus
1	1.00	1	1	-0.01	-0.013	-0.01	-0.01	-0.01	t	66629	TTGYRFTNF	Oryctolagus cuniculus
1	1.00	1	1	0	0	0	0	-0.001	t	104443	DAEFRHDSGYE	Mus musculus
1	1.00	1	1	0	0	0	0	-0.001	t	104443	DAEFRHDSGYE	Mus musculus
1	1.00	1	1	0.02	0.02	0.024	0.026	0.021	t	108857	DGQPGPAG	Rattus norvegicus
1	1.00	1	1	-0.01	-0.022	-0.01	-0.007	-0.01	t	156911	LGMSTNPGPRIL	Bos taurus
1	1.00	1	1	0.03	0.032	0.023	0.033	0.03	t	117015	KEQFLDGDGWTSRWIESK	Homo sapiens
1	1.00	1	1	0.01	0.01	0.01	0.01	0.01	t	130456	CRPQVNNPKEWSCAAC	Homo sapiens
1	1.00	1	1	0	0.002	-0.013	-0.003	0	t	115090	NLLRFFVAPFPE	Homo sapiens
1	1.00	1	1	0.03	0.025	0.026	0.027	0.03	t	49790	PTTNTGEGTSSPVTPTYT	Mesocricetus auratus
1	1.00	1	1	0.03	0.027	0.019	0.03	0.032	t	154	AAGGSYSSGDLGKKG	Oncorhynchus tshawytscha
1	1.00	1	1	0.01	0.015	0.014	0.007	0.008	t	157230	FDAEFLADAFNV	Homo sapiens

1	1.00	1	1	-0.01	-0.01	-0.01	-0.01	-0.008	t	131935	KPFSNSKV	Homo sapiens
1	1.00	1	1	0.03	0.025	0.025	0.03	0.03	t	10144	DSILFVPTTVDPASFSDDNS	Canis lupus
1	1.00	1	1	-0.02	-0.02	-0.02	-0.024	-0.022	t	118681	YAKDVKFGADARA	Homo sapiens
1	1.00	1	1	0.01	0.01	0.011	0.013	0.011	t	66474	TSTVQVFTDSEYQLPYVLGS	Homo sapiens
1	1.00	1	1	0.03	0.03	0.03	0.03	0.03	t	153351	ACTYSPRDMCGGGS	Mus musculus
1	1.00	1	1	0.01	0.01	0.01	0.01	0.01	t	121736	QCFLAITNIQLILPY	Mus musculus
1	1.00	1	1	0.01	0.01	0.01	0.01	0.01	t	121736	QCFLAITNIQLILPY	Mus musculus
1	1.00	1	1	0.01	0.01	0.01	0.01	0.01	t	115895	PPFLQPEVMG	Homo sapiens
1	1.00	1	1	0.01	0.01	0.01	0.01	0.01	t	79760	ASSPDAHVPFCF	Mus musculus
1	1.00	1	1	0.01	0.01	0.01	0.01	0.01	t	79345	QCDSFVISPPRTCG	Mus musculus
1	1.00	1	1	-0.01	-0.01	-0.01	-0.01	-0.01	t	138140	ALLSKVTGPPPMPD	Oryctolagus cuniculus
1	1.00	1	1	-0.03	-0.03	-0.03	-0.03	-0.03	t	80178	ADAPQGFYVL	Homo sapiens
1	1.00	1	1	0.02	0.013	0.021	0.017	0.017	t	24084	HIPYKISASTNCATVK	Homo sapiens
1	1.00	1	1	-0.01	-0.01	-0.01	-0.01	-0.011	t	16018	FGNDYEDRYYR	Mus musculus
1	1.00	1	1	-0.01	-0.01	-0.01	-0.01	-0.01	t	122150	MSKTLHAQLGFKKT	Oryctolagus cuniculus

1	1.00	1	1	-0.01	-0.01	-0.01	-0.01	-0.01	t	122150	MSKTLHAQLGFKKT	Oryctolagus cuniculus
1	1.00	1	1	-0.02	-0.022	-0.021	-0.017	-0.018	t	24080	HIPAYATHV	Mus musculus
1	1.00	1	1	0.04	0.04	0.04	0.036	0.041	t	134216	RNVELQ	Homo sapiens
1	1.00	1	1	0.05	0.054	0.043	0.051	0.054	t	137858	FTPGTEYT	Homo sapiens
1	1.00	1	1	0.01	0.014	0.01	0.013	0.012	t	33858	KTSERSQPRGRRQPIPKA	Homo sapiens
1	1.00	1	1	-0.05	-0.048	-0.057	-0.043	-0.049	t	105887	ISRCQVCMKKRH	Homo sapiens
1	1.00	1	1	0	0	-0.005	0	0	t	44569	NKPNNDFHFEVF	Mus musculus
1	1.00	1	1	0	0	-0.005	0	0	t	44569	NKPNNDFHFEVF	Mus musculus
1	1.00	1	1	0	0	0	0	0	t	57210	SDGYPPNRQDPRFTD	Homo sapiens
1	1.00	1	1	-0.05	-0.046	-0.05	-0.05	-0.051	t	122999	AAMDLRMGF	Ovis aries
1	1.00	1	1	0.01	0.01	0.015	0.01	0.01	t	6020	CAQQWPEWYPC	Mus musculus
1	1.00	1	1	0.01	0.01	0.015	0.01	0.01	t	6020	CAQQWPEWYPC	Mus musculus
1	1.00	1	1	0.01	0.01	0.015	0.01	0.01	t	6020	CAQQWPEWYPC	Mus musculus
1	1.00	1	1	-0.01	-0.011	-0.01	-0.007	-0.011	t	130469	DQPPTSRVPSLR	Camelus dromedarius
1	1.00	1	1	0	0	0	0	0	t	122950	YGHGSLELLADVQ	Mus musculus
1	1.00	1	1	0	0	0	0	0	t	110618	GPVGPAGGPGFP	Mus musculus

1	1.00	1	1	0	0	0	0	0	t	113778	QQPGQ	Homo sapiens
1	1.00	1	1	0.01	0.007	0.01	0.01	0.01	t	741	ADPNRFR GKD	Oryctolagus cuniculus
1	1.00	1	1	0.01	0.007	0.01	0.01	0.01	t	741	ADPNRFR GKD	Oryctolagus cuniculus
1	1.00	1	1	0.01	0.01	0.01	0.01	0.01	t	741	ADPNRFR GKD	Oryctolagus cuniculus
1	1.00	1	1	0.04	0.044	0.035	0.04	0.04	t	94038	TIYGNDGN DR	Ovis aries
1	1.00	1	1	-0.03	-0.03	-0.023	-0.03	-0.03	t	119492	LRKKGYDP G	Homo sapiens
1	1.00	1	1	0.02	0.02	0.019	0.02	0.02	t	70810	VRRDSAW DVRPLTET	Homo sapiens
1	1.00	1	1	0.03	0.03	0.03	0.03	0.031	t	61800	STNS	Mus musculus
1	1.00	1	1	0.04	0.04	0.04	0.04	0.041	t	115108	SHGRTQD ENP	Mus musculus
1	1.00	1	1	0	-0.002	0	0.003	0	t	146990	GPGTIKKIS F	Mus musculus
1	1.00	1	1	0.01	0.006	0.013	0.009	0.01	t	80699	CITTIYKTG	Oryctolagus cuniculus
1	1.00	1	1	0.03	0.03	0.033	0.03	0.03	t	69699	VLPPLLTD DMIAAYT AA	Homo sapiens
1	1.00	1	1	-0.01	-0.01	-0.01	-0.009	-0.012	t	156664	GGTHSQW NKPSKPKT NM	Mus musculus
1	1.00	1	1	-0.01	-0.012	-0.006	-0.007	-0.007	t	141706	MLEPFQIL	Mus musculus

1	1.00	1	1	-0.02	-0.018	-0.021	-0.023	-0.023	t	113876	SPQQSGQGQPGYYP	Homo sapiens
1	1.00	1	1	0.04	0.04	0.04	0.04	0.04	t	127712	KNWSTPF	Homo sapiens
1	1.00	1	1	0.04	0.04	0.04	0.04	0.04	t	114956	SSYANTTSED	Homo sapiens
1	1.00	1	1	0	-0.002	-0.008	0.003	0.002	t	111634	PNNNKILVPKVSGLQ	Mus musculus
1	1.00	1	1	0.02	0.02	0.02	0.02	0.02	t	151032	LYQNADTYVFVGSSR	Homo sapiens
1	1.00	1	1	0.02	0.02	0.02	0.02	0.02	t	104176	PKPQRKTKRNTNRRPQDVKF	Homo sapiens
1	1.00	1	1	0.02	0.02	0.02	0.02	0.02	t	92470	DLTFEKVKHN	Ovis aries
1	1.00	1	1	-0.03	-0.03	-0.032	-0.03	-0.03	t	97479	LTAWNPSL	Mus musculus
1	1.00	1	1	-0.02	-0.02	-0.02	-0.02	-0.02	t	139895	SLFASAQ	Ovis aries
1	1.00	1	1	0	0	-0.01	-0.006	-0.002	t	60327	SPVTKRASLKIDSKK	Homo sapiens
1	1.00	1	1	0	-0.003	-0.012	-0.001	0.001	t	10683	DVNKSAQL	Oryctolagus cuniculus
1	1.00	1	1	0	0	0.002	0	0	t	52600	QTQQPQQPFP	Mus musculus
1	1.00	1	1	0	0	0	0	-0.002	t	73660	YEGFSWTGVTQNKAKGIT	Mus musculus
1	1.00	1	1	0.02	0.02	0.02	0.02	0.02	t	135445	AKAAQFG	Oryctolagus cuniculus
1	1.00	1	1	0.01	0.01	0.009	0.01	0.01	t	72503	WGQGG	Mus musculus
1	1.00	1	1	0.01	0.01	0.012	0.011	0.01	t	71437	VTPDKEILY	Homo sapiens

1	1.00	1	1	0.01	0.006	0.008	0.013	0.008	t	51327	QLERANLR PCEQHLM	Oryctola gus cuniculu s
1	1.00	1	1	0.01	0.012	0.01	0.007	0.01	t	8754	DIPNPIGSE N	Homo sapiens
1	1.00	1	1	0.01	0.01	0.01	0.01	0.01	t	23191	GVTYEIDL TNKNAA	Homo sapiens
1	1.00	1	1	0	0	0	0	-0.001	t	48597	PNANPN	Mus musculu s
1	1.00	1	1	-0.02	-0.02	-0.015	-0.02	-0.019	t	108382	GGVVIA	Mus musculu s
1	1.00	1	1	-0.02	-0.02	-0.015	-0.02	-0.019	t	108382	GGVVIA	Mus musculu s
1	1.00	1	1	-0.01	-0.012	-0.007	-0.011	-0.012	t	150982	FEDINGIRR P	Mus musculu s
1	1.00	1	1	0	0	0	0	-0.001	t	64638	TKPSDGNC T	Oryctola gus cuniculu s
1	1.00	1	1	0	0	0	0	-0.001	t	64638	TKPSDGNC T	Oryctola gus cuniculu s
1	1.00	1	1	0.05	0.05	0.05	0.05	0.05	t	69235	VKIDNASP AS	Mus musculu s
1	1.00	1	1	0.05	0.05	0.05	0.05	0.05	t	69235	VKIDNASP AS	Homo sapiens
1	1.00	1	1	0.01	0.014	0.022	0.013	0.01	t	48663	PNKPNND FHFEV	Homo sapiens
1	1.00	1	1	0.01	0.014	0.022	0.013	0.01	t	48663	PNKPNND FHFEV	Homo sapiens
1	1.00	1	1	0.01	0.01	0.009	0.013	0.011	t	56501	RYAHPS	Homo sapiens
1	1.00	1	1	-0.02	-0.023	-0.023	-0.02	-0.02	t	81798	ERLRDLRR KEKSE	Oryctola gus cuniculu s

1	1.00	1	1	-0.02	-0.024	-0.023	-0.02	-0.02	t	81798	ERLRDLRR KEKSE	Oryctolagus cuniculus
1	1.00	1	1	0	0.003	0.002	0	0	t	119641	SEHETRLV AKLFKDYS	Mus musculus
1	1.00	1	1	0	-0.003	0.001	0	0	t	6478	CKFTLVLTK CGSQVL	Oryctolagus cuniculus
1	1.00	1	1	-0.05	-0.05	-0.06	-0.05	-0.051	t	97117	YIQMKRIA VG	Mus musculus
1	1.00	1	1	-0.01	-0.011	-0.021	-0.01	-0.008	t	49757	PTPVSEEII TPTLQA	Bos taurus
1	1.00	1	1	-0.01	-0.008	-0.018	-0.007	-0.01	t	110841	EVTPAIKSE VSL	Homo sapiens
1	1.00	1	1	0.05	0.046	0.05	0.043	0.05	t	131106	TGLE	Oryctolagus cuniculus
1	1.00	1	1	0.05	0.05	0.05	0.05	0.05	t	111161	CNSSSMN SC	Homo sapiens
1	1.00	1	1	0.03	0.03	0.03	0.03	0.03	t	114859	ICGTDGVT YT	Homo sapiens
1	1.00	1	1	0.03	0.03	0.03	0.03	0.03	t	79384	SCNSFMIP AQPSCT	Mus musculus
1	1.00	1	1	0.01	0.01	0.01	0.01	0.01	t	103260	HRSDGSG DTFLFTQY	Homo sapiens
1	1.00	1	1	0.01	0.01	0.01	0.01	0.01	t	115980	SPEVIESPP E	Homo sapiens
1	1.00	1	1	0.01	0.01	0.01	0.01	0.01	t	78226	PFTNLAHV	Homo sapiens
1	1.00	1	1	-0.01	-0.01	-0.01	-0.01	-0.01	t	116978	FPSHYWLY PPPT	Homo sapiens
1	1.00	1	1	0.01	0.012	0.01	0.017	0.01	t	104253	SNVECDGL NCDWII	Homo sapiens
1	1.00	1	1	0.02	0.02	0.015	0.02	0.022	t	73636	YEDRYYRE	Mus musculus

1	1.00	1	1	-0.01	-0.01	-0.01	-0.01	-0.011	t	116682	CHGRGTC NYYANAYS	Oryctolagus cuniculus
1	1.00	1	1	0.04	0.05	0.04	0.037	0.039	t	134149	LRENQLLP GFEL	Rattus rattus
1	1.00	1	1	0.08	0.077	0.079	0.08	0.08	t	145119	CLPKPLPK GTEDNDQ RAT	Oryctolagus cuniculus
1	1.00	1	1	0.03	0.028	0.035	0.029	0.03	t	61409	SSTQVQHT LLQT	Mus musculus
1	1.00	1	1	0.02	0.02	0.013	0.02	0.021	t	122719	CGVDFEIK AFATHSTD VEED	Rattus norvegicus
1	1.00	1	1	0	0.004	0	0.001	0	t	37310	LLGACSFS	Homo sapiens
1	1.00	1	1	0.06	0.064	0.069	0.063	0.06	t	50354	QASDDGD KGSDVES	Homo sapiens
1	1.00	1	1	-0.02	-0.02	-0.02	-0.02	-0.02	t	78562	VYASPNTT GLLNDIL	Mus musculus
1	1.00	1	1	0	0	-0.004	0	0.003	t	52082	QQPFP	Mus musculus
1	1.00	1	1	-0.01	-0.01	-0.01	-0.01	-0.011	t	145776	FDTGAFDP DWPY	Mus musculus
1	1.00	1	1	0	0	0	0	-0.002	t	104202	QHGHLTTS SQELERYT FNP	Oryctolagus cuniculus
1	1.00	1	1	0.02	0.022	0.027	0.023	0.021	t	151609	DETPMRG ETRKV	Homo sapiens
1	1.00	1	1	0.04	0.026	0.027	0.039	0.047	t	61502	SSYTQMY Q	Macaca mulatta
1	1.00	1	1	0.02	0.018	0.02	0.019	0.022	t	146990	GPGTIKKIS F	Mus musculus
1	1.00	1	1	0.01	0.01	0.01	0.01	0.009	t	75847	YSPELE	Mus musculus

1	1.00	1	1	0.03	0.026	0.026	0.029	0.03	t	67972	VDHFHLGDDMDPEL	Oryctolagus cuniculus
1	1.00	1	1	0	-0.002	-0.007	0	0.001	t	75544	YQRESQAYYQRGA	Oryctolagus cuniculus
1	1.00	1	1	0.02	0.02	0.008	0.02	0.02	t	60592	SQWGWCGSTDEYCSPDHNCQ	Mus musculus
1	1.00	1	1	0.02	0.02	0.008	0.02	0.02	t	60592	SQWGWCGSTDEYCSPDHNCQ	Mus musculus
1	1.00	1	1	0	-0.002	-0.005	-0.001	0	t	78752	NKGKWKEHIKEVTEKLKKA	Mus musculus
1	1.00	1	1	-0.01	-0.012	-0.015	-0.007	-0.009	t	51775	QNTRNIVPVSIVSR	Mus musculus
1	1.00	1	1	-0.02	-0.016	-0.011	-0.013	-0.02	t	110852	FFSARGHRPLDKKREEAP	Homo sapiens
1	1.00	1	1	-0.02	-0.018	-0.011	-0.013	-0.02	t	110854	FFSARGHRPLDKKREEAP	Homo sapiens
1	1.00	1	1	0.03	0.027	0.028	0.03	0.031	t	38408	LPAMSTTDLEAYFKDC	Oryctolagus cuniculus
1	1.00	1	1	0.01	0.006	0.01	0.013	0.01	t	111208	DPDVNRFIDKYGQ	Oryctolagus cuniculus
1	1.00	1	1	0.02	0.02	0.022	0.02	0.019	t	78923	AFGANS	Mus musculus
1	1.00	1	1	0	-0.002	0.012	-0.001	-0.004	t	20089	GGWNTGGSR	Mus musculus
1	1.00	1	1	-0.02	-0.02	-0.02	-0.02	-0.018	t	128088	QVYYRPV	Ovis aries
1	1.00	1	1	0.02	0.022	0.017	0.021	0.02	t	21831	GPVYCFTPSPVVVGTTDR	Homo sapiens

1	1.00	1	1	0.04	0.04	0.04	0.04	0.04	t	92780	GDDFIDG GKG	Ovis aries
1	1.00	1	1	0.02	0.02	0.02	0.02	0.02	t	135682	GKVDAFC G	Homo sapiens
1	1.00	1	1	0.02	0.02	0.02	0.02	0.02	t	123425	LGEEYEEH L	Ovis aries
1	1.00	1	1	0.02	0.02	0.02	0.02	0.02	t	103932	GKLPPPI	Homo sapiens
1	1.00	1	1	0.02	0.02	0.02	0.02	0.02	t	70799	VRPLTETR GDLFSGD	Homo sapiens
1	1.00	1	1	-0.02	-0.02	-0.02	-0.02	-0.02	t	96476	KLFPNSLD QT	Homo sapiens
1	1.00	1	1	0.01	0.01	0.01	0.01	0.01	t	3524	APEARQ	Homo sapiens
1	1.00	1	1	0.03	0.033	0.024	0.031	0.032	t	41868	MKNNPVY SESL	Equus caballus
1	1.00	1	1	0	0.002	0	-0.003	-0.002	t	28075	IPSTAFTDI AWVRLPN HY	Homo sapiens
1	1.00	1	1	0.01	0.01	0.009	0.01	0.01	t	73636	YEDRYYRE	Mus musculu s
1	1.00	1	1	0.03	0.035	0.039	0.03	0.03	t	65861	TQGSNWI Q	Homo sapiens
1	1.00	1	1	0.03	0.03	0.039	0.03	0.03	t	65861	TQGSNWI Q	Homo sapiens
1	1.00	1	1	0.02	0.008	0.02	0.023	0.02	t	156918	MAITDHP GSAMV	Bos taurus
1	1.00	1	1	0.03	0.03	0.03	0.03	0.03	t	79672	VNLEDPAS RDL	Homo sapiens
1	1.00	1	1	0.03	0.03	0.03	0.03	0.03	t	133535	CKPGEVQ QC	Mus musculu s
1	1.00	1	1	0	-0.003	0.005	0	-0.001	t	146795	CCELCCNP QCAGCY	Oryctola gus cuniculu s

1	1.00	1	1	-0.02	-0.022	-0.02	-0.021	-0.022	t	70472	VPSDTVQT RHVV	Mus musculu s
1	1.00	1	1	-0.03	-0.028	-0.03	-0.033	-0.032	t	115887	PHPHLSF MAI	Homo sapiens
1	1.00	1	1	0.01	0.01	0.01	0.01	0.01	t	33888	KTTLLK	Homo sapiens
1	1.00	1	1	0	0	0.011	0.007	0	t	102381	EEIAQVATI SANGDKDI GNI	Mus musculu s
1	1.00	1	1	0.01	0.01	0.01	0.01	0.01	t	94449	CCNPA	Capra hircus
1	1.00	1	1	0	-0.002	0	0.003	0	t	27728	INQQLNPK TNKWEDK	Mus musculu s
1	1.00	1	1	0	-0.002	0	0.003	0	t	27727	INQQLNPK	Mus musculu s
1	1.00	1	1	0.03	0.03	0.03	0.03	0.03	t	7532	DAHHAAD	Mus musculu s
1	1.00	1	1	0.03	0.03	0.03	0.03	0.03	t	7532	DAHHAAD	Mus musculu s
1	1.00	1	1	-0.03	-0.03	-0.03	-0.03	-0.029	t	115297	INYWLAHK AL	Homo sapiens
1	1.00	1	1	0.03	0.027	0.033	0.036	0.027	t	73159	WTTCQSIA FPSKTSASI GSL	Mus musculu s
1	1.00	1	1	0.03	0.03	0.03	0.03	0.03	t	123875	YMESEKFI K	Ovis aries
1	1.00	1	1	0.03	0.03	0.03	0.03	0.03	t	123149	ESEKFIKW P	Ovis aries
1	1.00	1	1	0.03	0.03	0.03	0.03	0.03	t	84512	KLQKNGA DLA	Homo sapiens

1	1.00	1	1	0.01	0.01	0.01	0.01	0.01	t	121847	SGVFLSVP ESYFGSC	Mus musculu s
1	1.00	1	1	0.01	0.01	0.01	0.01	0.01	t	121847	SGVFLSVP ESYFGSC	Mus musculu s
1	1.00	1	1	0.01	0.01	0.01	0.01	0.01	t	103929	GKLPIPY	Homo sapiens
1	1.00	1	1	-0.01	-0.01	-0.01	-0.01	-0.01	t	105850	GTAPFEIH	Oryctola gus cuniculu s
1	1.00	1	1	-0.03	-0.03	-0.03	-0.03	-0.03	t	123626	PPYMPPP G	Oryctola gus cuniculu s
1	1.00	1	1	-0.03	-0.03	-0.03	-0.03	-0.03	t	115824	KIAKYIPIQ Y	Homo sapiens
1	1.00	1	1	0.02	0.017	0.018	0.02	0.02	t	68858	VHILCYCY GLPDSEP	Oryctola gus cuniculu s
1	1.00	1	1	0.02	0.023	0.018	0.02	0.02	t	68858	VHILCYCY GLPDSEP	Equus caballus
1	1.00	1	1	0.03	0.03	0.03	0.03	0.03	t	95119	MKKPGENI KPLSCKYSY PKH	Rattus norvegic us
1	1.00	1	1	0.03	0.03	0.03	0.03	0.03	t	142424	GDEFDTYD DIKKVT	Homo sapiens
1	1.00	1	1	0.03	0.032	0.021	0.031	0.03	t	60669	SRGGSQAS SRSSSRSR	Homo sapiens
1	1.00	1	1	0.03	0.03	0.03	0.03	0.032	t	152053	PYCLGSPS QDGY	Sus scrofa
1	1.00	1	1	-0.02	-0.02	-0.027	-0.026	-0.022	t	60082	SPIAGKTS VLRA	Homo sapiens
1	1.00	1	1	-0.02	-0.018	-0.02	-0.013	-0.017	t	140546	CNLWEVM HGTC	Homo sapiens
1	1.00	1	1	-0.03	-0.032	-0.022	-0.027	-0.031	t	27067	ILGGKFL	Mus musculu s
1	1.00	1	1	-0.02	-0.022	-0.02	-0.027	-0.022	t	135497	RYPNQVYY RPM	Mus musculu s

1	1.00	1	1	0	-0.002	0	-0.001	0	t	45695	NRLGGLFNFGPKQKI	Mus musculus
1	1.00	1	1	0.02	0.016	0.023	0.019	0.018	t	33090	KQVTIRIGCKTSSS	Oryctolagus cuniculus
1	1.00	1	1	0	-0.004	0	0	-0.001	t	124604	QIERDTERDRFLSAPEAV	Oryctolagus cuniculus
1	1.00	1	1	0	-0.002	0	0	-0.001	t	3245	ANDVDNSNPVVQAEQLNWL	Mus musculus
1	1.00	1	1	0	-0.003	-0.003	0	0	t	21812	GPTRTIGGSQ	Oryctolagus cuniculus
1	1.00	1	1	0	-0.002	-0.007	-0.001	-0.002	t	20480	GIVRTPLAELLDG	Mus musculus
1	1.00	1	1	0	-0.004	-0.012	0.003	0.003	t	106779	RKPLSPGMTGR	Oryctolagus cuniculus
1	1.00	1	1	-0.01	-0.01	-0.01	-0.01	-0.011	t	133694	SHMNALLRQQAF	Homo sapiens
1	1.00	1	1	0.07	0.07	0.068	0.067	0.068	t	60470	SQKGKQQEEEENEGS	Homo sapiens
1	1.00	1	1	0.02	0.02	0.014	0.02	0.02	t	64032	THGQWNKPSKPKTNMK	Oryctolagus cuniculus
1	1.00	1	1	0.02	0.02	0.014	0.02	0.02	t	64032	THGQWNKPSKPKTNMK	Oryctolagus cuniculus
1	1.00	1	1	-0.03	-0.03	-0.047	-0.03	-0.026	t	145725	CKQAGTCPP	Mus musculus
1	1.00	1	1	-0.04	-0.04	-0.04	-0.04	-0.04	t	111166	CPVYIKPC	Homo sapiens
1	1.00	1	1	0	0.002	-0.004	0.001	0.001	t	19639	GFTWTGVTQNG	Homo sapiens

1	1.00	1	1	0.06	0.06	0.06	0.06	0.06	t	115730	EPTSTPTTE A	Homo sapiens
1	1.00	1	1	0.02	0.02	0.02	0.02	0.02	t	92809	GGKGDDIL DG	Ovis aries
1	1.00	1	1	0.02	0.02	0.02	0.02	0.02	t	95318	DLQVLAQ K	Bos taurus
1	1.00	1	1	0.02	0.028	0.02	0.02	0.02	t	95318	DLQVLAQ K	Cavia porcellu s
1	1.00	1	1	0	0	0	0	0	t	100352	NQLKEHPT DF	Mus musculu s
1	1.00	1	1	0	0	0	0	0	t	79357	RCDLRYDA FVPWCP	Mus musculu s
1	1.00	1	1	0	0.004	0	0	0	t	51123	QIPYNLKIF AIMLDTHK MLV	Aotus nancyma ae
1	1.00	1	1	0.02	0.018	0.028	0.021	0.02	t	86712	NFKQGQG	Homo sapiens
1	1.00	1	1	0.01	0.01	0.032	0.01	0.007	t	144622	EDVGSNK	Mus musculu s
1	1.00	1	1	-0.03	-0.028	-0.037	-0.027	-0.029	t	118348	FHSGKHY WEVDVTG KE	Homo sapiens
1	1.00	1	1	-0.01	-0.007	-0.008	-0.01	-0.01	t	19577	GFLMHAP AFETAGTY	Mus musculu s
1	1.00	1	1	0	0	-0.006	0	0.001	t	159172	TTDSGKLV TDWCCRS C	Mus musculu s
1	1.00	1	1	-0.02	-0.018	-0.02	-0.013	-0.02	t	134274	YKWTVTV EDGPTKSD PRCLT	Homo sapiens
1	1.00	1	1	0	0.004	0	0.001	0.001	t	49505	PSSKRFQP FQQFGRD	Homo sapiens

1	1.00	1	1	0.02	0.018	0.013	0.023	0.022	t	106550	IWYINCFG CETHAML	Mus musculu s
1	1.00	1	1	0	0	-0.01	0	0.001	t	44251	NIISCNKN DKNQ	Oryctola gus cuniculu s
1	1.00	1	1	0.05	0.052	0.043	0.053	0.051	t	125357	AEIETDKA TIGFEVQE EG	Homo sapiens
1	1.00	1	1	-0.01	-0.01	-0.01	-0.01	-0.01	t	137927	LSADFTPG	Homo sapiens
1	1.00	1	1	-0.03	-0.028	-0.034	-0.029	-0.03	t	138028	SAGALQLL VVGC	Homo sapiens
1	1.00	1	1	0.01	0.012	0.01	0.011	0.01	t	117111	WPMNDD STRPR	Homo sapiens
1	1.00	1	1	-0.01	-0.008	-0.013	-0.003	-0.01	t	120548	GAGAPVYL AAVLEYLT AE	Homo sapiens
1	1.00	1	1	0.02	0.017	0.017	0.02	0.02	t	13421	EMVDVVT G	Oryctola gus cuniculu s
1	1.00	1	1	-0.04	-0.04	-0.04	-0.04	-0.042	t	134670	FIDEPRRR PI	Homo sapiens
1	1.00	1	1	0.03	0.03	0.03	0.03	0.03	t	109425	KNQVSLT	Homo sapiens
1	1.00	1	1	-0.02	-0.018	-0.024	-0.012	-0.02	t	55299	RPQKRPSC IGCKGTHG GTGA	Homo sapiens
1	1.00	1	1	0.04	0.043	0.04	0.04	0.04	t	105702	CVYDQYG TEFEDYSY ES	Mus musculu s
1	1.00	1	1	0	0	0	0	-0.001	t	39183	LRRDLDAS REAKKQVE KALE	Homo sapiens
1	1.00	1	1	-0.02	-0.02	-0.02	-0.02	-0.021	t	144647	GDNGKGF FELPMKD	Oryctola gus cuniculu s

1	1.00	1	1	0.02	0.016	0.02	0.02	0.021	t	75950	YTASARGD LAHLTTT	Mus musculus
1	1.00	1	1	0.02	0.016	0.02	0.02	0.021	t	75950	YTASARGD LAHLTTT	Mus musculus
1	1.00	1	1	-0.01	-0.012	-0.015	-0.011	-0.01	t	78735	LRVKIERD DLSRETIIQ II	Mus musculus
1	1.00	1	1	0.01	0.007	0.014	0.01	0.01	t	99785	YIVDDVN	Oryctolagus cuniculus
1	1.00	1	1	-0.03	-0.032	-0.03	-0.03	-0.03	t	111211	DPVGPSD PSIVSLVEE TSF	Oryctolagus cuniculus
1	1.00	1	1	-0.03	-0.033	-0.03	-0.03	-0.03	t	111211	DPVGPSD PSIVSLVEE TSF	Oryctolagus cuniculus
1	1.00	1	1	0.05	0.052	0.047	0.051	0.05	t	46895	PAPQAPY QGYQEPP APQAPY	Homo sapiens
1	1.00	1	1	0	0.004	-0.003	0.001	0	t	752	ADQAQYN QMH	Homo sapiens
1	1.00	1	1	-0.01	-0.01	-0.01	-0.01	-0.012	t	113371	EPARSRSR	Homo sapiens
1	1.00	1	1	0.04	0.04	0.041	0.043	0.042	t	11451	EDTLNKVD	Homo sapiens
1	1.00	1	1	0.02	0.017	0.021	0.02	0.02	t	5771	AYFLYQQ QGRLDKL	Oryctolagus cuniculus
1	1.00	1	1	0.01	0.01	0.01	0.01	0.01	t	151110	AEKA	Homo sapiens
1	1.00	1	1	0.03	0.03	0.03	0.03	0.03	t	158587	EFGTFLW	Homo sapiens

1	1.00	1	1	0.03	0.03	0.03	0.03	0.03	t	138219	PTPETGPLQR	Homo sapiens
1	1.00	1	1	0.03	0.03	0.03	0.03	0.03	t	123343	KAYMESEKF	Ovis aries
1	1.00	1	1	0.01	0.01	0.01	0.01	0.01	t	132907	TCYDDEMKIGFPEDL	Trichosurus vulpecula
1	1.00	1	1	0.01	0.01	0.01	0.01	0.01	t	114761	DCSEYPKPDC	Homo sapiens
1	1.00	1	1	0.01	0.01	0.01	0.01	0.01	t	102091	SSTWNTWQYFPL	Homo sapiens
1	1.00	1	1	-0.01	-0.01	-0.01	-0.01	-0.01	t	100405	PYYGPGVDYKNNLD	Oryctolagus cuniculus
1	1.00	1	1	-0.03	-0.03	-0.03	-0.03	-0.03	t	110585	GAPGIAGAPGFPGPR	Mus musculus
1	1.00	1	1	-0.03	-0.03	-0.03	-0.03	-0.03	t	115259	FYQKFPQYLQYLYQGPIVLN	Homo sapiens
1	1.00	1	1	-0.03	-0.03	-0.03	-0.03	-0.03	t	111157	CMPYGSLAC	Homo sapiens
1	1.00	1	1	-0.03	-0.03	-0.03	-0.03	-0.03	t	81550	EIKVQMSAAY	Homo sapiens
1	1.00	1	1	-0.01	-0.022	-0.015	-0.009	-0.01	t	64050	THLRALFGYSEVRKVILTKR	Canis lupus
1	1.00	1	1	-0.05	-0.048	-0.05	-0.043	-0.05	t	140648	ALREYRKKMDIPA	Homo sapiens
1	1.00	1	1	0.01	0.01	0.01	0.01	0.012	t	132100	TLPTPVVQL	Homo sapiens
1	1.00	1	1	-0.01	-0.01	-0.01	-0.01	-0.011	t	108860	DGVNH	Mus musculus
1	1.00	1	1	0.01	0.011	0.006	0.011	0.008	t	48395	PLLGCIGSTCAEDGN	Mus musculus
1	1.00	1	1	0.01	0.007	0.01	0.01	0.012	t	135495	RITLYL	Oryctolagus cuniculus
1	1.00	1	1	0.03	0.03	0.03	0.034	0.03	t	117448	CTLAEK	Homo sapiens

1	1.00	1	1	0	-0.003	0.002	0	-0.002	t	56610	RYPGQGSPGGNRYPPQG	Oryctolagus cuniculus
1	1.00	1	1	0	0	0	0	0	t	44554	NKND	Mus musculus
1	1.00	1	1	0	0.003	0	0	0	t	44554	NKND	Mus musculus
1	1.00	1	1	-0.02	-0.02	-0.021	-0.023	-0.018	t	48849	PPPAPDPSPT	Homo sapiens
1	1.00	1	1	0	0	0	0.002	0	t	25009	HTYSPHELSRVAAT	Homo sapiens
1	1.00	1	1	0.02	0.022	0.014	0.021	0.022	t	58903	SKNFNFEV	Homo sapiens
1	1.00	1	1	-0.01	-0.01	-0.012	-0.01	-0.01	t	69833	VLVLNPSVAATLGFGAYMSK	Mus musculus
1	1.00	1	1	-0.01	-0.012	-0.004	-0.009	-0.011	t	98804	GLVWTSGPAT	Mus musculus
1	1.00	1	1	-0.03	-0.03	-0.03	-0.027	-0.028	t	70661	VQVRI	Homo sapiens
1	1.00	1	1	0.03	0.026	0.03	0.024	0.031	t	142443	LSTTEVAM	Oryctolagus cuniculus
1	1.00	1	1	0.03	0.026	0.03	0.024	0.031	t	142443	LSTTEVAM	Oryctolagus cuniculus
1	1.00	1	1	0.02	0.02	0.02	0.02	0.02	t	108152	TCPPCPAPELLGG	Homo sapiens
1	1.00	1	1	0.02	0.02	0.02	0.02	0.02	t	106315	CSGTKKLSEE	Homo sapiens
1	1.00	1	1	0	-0.002	0.005	-0.001	0.002	t	52332	QSHFLLQGT	Mus musculus
1	1.00	1	1	0.02	0.022	0	0.021	0.024	t	46479	NVSINNNNNNNSNT	Homo sapiens

1	1.00	1	1	0.02	0.022	0.013	0.017	0.02	t	20548	GKEKVNELSK	Homo sapiens
1	1.00	1	1	-0.08	-0.083	-0.072	-0.08	-0.082	t	58956	SKVALAALPA	Oryctolagus cuniculus
1	1.00	1	1	-0.03	-0.032	-0.027	-0.031	-0.032	t	19504	GFCDGYCKTHLKASS	Mus musculus
1	1.00	1	1	-0.03	-0.032	-0.027	-0.031	-0.032	t	19504	GFCDGYCKTHLKASS	Mus musculus
1	1.00	1	1	0.01	0.01	0.008	0.014	0.012	t	134415	IWRSTQGV	Homo sapiens
1	1.00	1	1	-0.03	-0.03	-0.03	-0.03	-0.03	t	142022	SRIFRGDN	Mus musculus
1	1.00	1	1	-0.03	-0.03	-0.03	-0.03	-0.03	t	142022	SRIFRGDN	Mus musculus
1	1.00	1	1	-0.03	-0.03	-0.03	-0.03	-0.03	t	142022	SRIFRGDN	Sus scrofa
1	1.00	1	1	-0.01	-0.007	-0.01	-0.01	-0.012	t	140853	GEYVQILDAHAAYLNATPAA	Mus musculus
1	1.00	1	1	0.08	0.08	0.08	0.08	0.08	t	115884	PGEIVESLSS	Homo sapiens
1	1.00	1	1	0.04	0.04	0.04	0.04	0.04	t	79478	YFWNYSYEVF	Mus musculus
1	1.00	1	1	0.02	0.012	0.02	0.02	0.02	t	144195	AGGGSGSADDVVDA	Bos taurus
1	1.00	1	1	0.02	0.02	0.02	0.02	0.02	t	144195	AGGGSGSADDVVDA	Bos taurus
1	1.00	1	1	0.02	0.02	0.02	0.02	0.02	t	99445	LRAPQRCDLDVESGG	Homo sapiens
1	1.00	1	1	0.02	0.02	0.02	0.02	0.02	t	79142	ACDPSTVMVPWPCS	Mus musculus
1	1.00	1	1	0.02	0.02	0.02	0.02	0.02	t	93328	LSKVVDNYEL	Ovis aries

1	1.00	1	1	0	0	0	0	0	t	151032	LYQNADTY VFVGSSR	Homo sapiens
1	1.00	1	1	0	0	0	0	0	t	137734	DTALTKAA LKLV	Mus musculu s
1	1.00	1	1	0	0	0	0	0	t	123653	QCLEDFPK L	Ovis aries
1	1.00	1	1	0	0	0	0	0	t	118610	SPGFNDG GKNTAPLT LC	Homo sapiens
1	1.00	1	1	0	0	0	0	0	t	123307	IEALPSIKA	Ovis aries
1	1.00	1	1	0	0	0	0	0	t	106921	TPYTNNPD LLFT	Mus musculu s
1	1.00	1	1	-0.02	-0.02	-0.02	-0.02	-0.02	t	107363	FLFPPKP	Homo sapiens
1	1.00	1	1	-0.04	-0.04	-0.04	-0.04	-0.04	t	142131	DTPTLHEY MLDL	Homo sapiens
1	1.00	1	1	0.01	0.007	0.008	0.01	0.01	t	22884	GTVVAVG PGRWDED GE	Mus musculu s
1	1.00	1	1	0.01	0.012	0.012	0.007	0.008	t	44858	NLRGDLQ VLA	Cavia porcellu s
1	1.00	1	1	0.01	0.012	0.012	0.007	0.008	t	44858	NLRGDLQ VLA	Cavia porcellu s
1	1.00	1	1	-0.01	-0.012	-0.016	-0.013	-0.01	t	54238	RIRFKDDT SF	Mus musculu s
1	1.00	1	1	0.02	0.018	0.013	0.02	0.02	t	123016	AIYENEIHA LWADLP	Trichosu rus vulpecul a
1	1.00	1	1	-0.04	-0.037	-0.038	-0.04	-0.042	t	51833	QPFRPQQ PYP	Mus musculu s
1	1.00	1	1	0.01	0.01	0.01	0.01	0.01	t	134781	RGDGPFRL GG	Homo sapiens

1	1.00	1	1	-0.06	-0.058	-0.067	-0.061	-0.061	t	107828	ERGLKGHR GFT	Homo sapiens
1	1.00	1	1	0	-0.003	-0.011	-0.001	0	t	138175	GAPPNGA PPPPVDP	Oryctola gus cuniculu s
1	1.00	1	1	0.03	0.03	0.03	0.03	0.031	t	49533	PSTPGDNP LV	Homo sapiens
1	1.00	1	1	0.05	0.054	0.012	0.051	0.059	t	133561	ESTWYM WQSRSE	Homo sapiens
1	1.00	1	1	0.02	0.008	0.02	0.023	0.02	t	156872	FSKSSSWE SWLP	Bos taurus
1	1.00	1	1	0.09	0.09	0.09	0.09	0.09	t	110460	YEQLNDSS EEEDEIDG	Homo sapiens
1	1.00	1	1	-0.02	-0.018	-0.016	-0.019	-0.02	t	47726	PGPSQKI	Homo sapiens
1	1.00	1	1	-0.02	-0.018	-0.016	-0.019	-0.02	t	47726	PGPSQKI	Homo sapiens
1	1.00	1	1	0.01	0.016	0.01	0.01	0.01	t	123133	EKFNMGL DL	Ovis aries
1	1.00	1	1	-0.02	-0.02	-0.018	-0.02	-0.019	t	153288	SDPRKMCI Y	Mus musculu s
1	1.00	1	1	0.02	0.018	0.028	0.019	0.02	t	79040	PGQNLCYT K	Mus musculu s
1	1.00	1	1	-0.02	-0.021	-0.018	-0.023	-0.022	t	3986	AQKKIIAEK TKI	Homo sapiens
1	1.00	1	1	0	0	0	0	-0.001	t	926	AEGEFKTR RNTNYQD PAK	Homo sapiens
1	1.00	1	1	0.02	0.02	0.015	0.023	0.02	t	24286	HLPYIEQG MQLAEQ	Homo sapiens
1	1.00	1	1	0.01	0.01	0.01	0.01	0.01	t	72503	WGQGG	Mus musculu s
1	1.00	1	1	0.01	0.01	0.01	0.01	0.01	t	72503	WGQGG	Mus musculu s
1	1.00	1	1	0.02	0.02	0.02	0.02	0.019	t	151792	AINEGQNK PEI	Homo sapiens

1	1.00	1	1	0	0	0	0	-0.001	t	130986	NPIENMMDRDSQ	Homo sapiens
1	1.00	1	1	-0.01	-0.01	-0.01	-0.014	-0.013	t	28911	ITGHRMAWDMMMNWS	Homo sapiens
1	1.00	1	1	0.05	0.052	0.043	0.047	0.052	t	14574	ETTTASGLVIPDTAK	Homo sapiens
1	1.00	1	1	0.01	0.008	0	0.013	0.012	t	8475	DGQPAGDRADGQPAGDRA	Mus musculus
1	1.00	1	1	0	0.004	0.007	-0.003	-0.001	t	106411	FALEAYAFDI	Homo sapiens
1	1.00	1	1	0.03	0.03	0.03	0.03	0.03	t	92809	GGKGDDILDG	Ovis aries
1	1.00	1	1	0.01	0.01	0.01	0.01	0.01	t	140605	TLYDALGAV	Mus musculus
1	1.00	1	1	0.01	0.01	0.01	0.01	0.01	t	140605	TLYDALGAV	Mus musculus
1	1.00	1	1	0.01	0.01	0.01	0.01	0.01	t	105697	CCAVLNGAY	Mus musculus
1	1.00	1	1	0.02	0.02	0.02	0.021	0.021	t	51306	QLDPAFG	Mus musculus
1	1.00	1	1	-0.01	-0.01	-0.01	-0.01	-0.01	t	135698	IYTKVSRY	Homo sapiens
1	1.00	1	1	-0.01	-0.01	-0.01	-0.01	-0.01	t	78299	WHLAYSP	Homo sapiens
1	1.00	1	1	0.02	0.016	0.013	0.023	0.021	t	106418	FIFPPSDEQLKSGTASVV	Oryctolagus cuniculus
1	1.00	1	1	0.01	0.01	0.011	0.01	0.01	t	60594	SQWNKPSK	Mus musculus
1	1.00	1	1	0.02	0.022	0.016	0.021	0.02	t	73068	WSEVDAVSSP	Homo sapiens
1	1.00	1	1	0.03	0.034	0.03	0.03	0.028	t	79369	RGDLRGDLRGDL	Cavia porcellus

1	1.00	1	1	0.03	0.027	0.03	0.03	0.028	t	79369	RGDLRGDLRGDL	Oryctolagus cuniculus
1	1.00	1	1	0.01	0.01	0.009	0.007	0.011	t	22372	GSIGAASMEF	Homo sapiens
1	1.00	1	1	0	0	0	0	0	t	137451	SHRLPVK	Mus musculus
1	1.00	1	1	-0.04	-0.04	-0.047	-0.049	-0.042	t	130922	KGMLPDPKNV	Homo sapiens
1	1.00	1	1	0.02	0.016	0.003	0.013	0.021	t	115640	QKSQRSQDEN	Oryctolagus cuniculus
1	1.00	1	1	-0.01	-0.007	-0.01	-0.01	-0.007	t	108075	PREPQVY	Mus musculus
1	1.00	1	1	-0.01	-0.006	-0.021	-0.013	-0.01	t	29897	KANADAKAAYEAAVAANNA	Homo sapiens
1	1.00	1	1	-0.02	-0.02	-0.013	-0.023	-0.02	t	25296	IAGMDWAVK	Homo sapiens
1	1.00	1	1	-0.06	-0.058	-0.059	-0.063	-0.062	t	114997	YTNDCLLCAY	Homo sapiens
1	1.00	1	1	0.03	0.03	0.018	0.03	0.03	t	42074	MLTESTFKNLSVTFKFGEEF	Mus musculus
1	1.00	1	1	0.03	0.03	0.018	0.03	0.03	t	42074	MLTESTFKNLSVTFKFGEEF	Mus musculus
1	1.00	1	1	-0.01	-0.01	-0.013	-0.01	-0.01	t	45877	NSKKIICTCKEPTPNAYYEG	Mus musculus
1	1.00	1	1	0.03	0.028	0.03	0.029	0.03	t	116930	AYKLPQTDDST	Mus musculus
1	1.00	1	1	0.07	0.07	0.07	0.07	0.07	t	103792	AGTELSATGNESGGG	Oryctolagus cuniculus

1	1.00	1	1	-0.01	-0.01	-0.008	-0.01	-0.011	t	60024	SPDVDLG DISGINAS	Mus musculu s
1	1.00	1	1	0.03	0.028	0.033	0.033	0.031	t	33222	KRTISIWG T	Mus musculu s
1	1.00	1	1	0.01	0.01	0.005	0.01	0.012	t	145838	IVSLVEETS FIDAGAP	Mus musculu s
1	1.00	1	1	-0.02	-0.02	-0.025	-0.02	-0.019	t	107219	PAGGPGF PG	Rattus norvegic us
1	1.00	1	1	0.03	0.03	0.03	0.03	0.031	t	7673	DAVTE	Mus musculu s
1	1.00	1	1	-0.01	-0.013	-0.002	-0.01	-0.013	t	42885	MTTAQGT SMYPSC	Oryctola gus cuniculu s
1	1.00	1	1	0.02	0.02	0.02	0.02	0.02	t	73611	YDYQHKE G	Oryctola gus cuniculu s
1	1.00	1	1	0.02	0.02	0.02	0.02	0.02	t	4770	ASSGIGPE	Oryctola gus cuniculu s
1	1.00	1	1	-0.03	-0.026	-0.031	-0.029	-0.032	t	147064	KHQGAQY VWNRTA	Homo sapiens
1	1.00	1	1	0.02	0.017	0.02	0.02	0.022	t	140889	PAVLGHD EAAYS	Oryctola gus cuniculu s
1	1.00	1	1	-0.04	-0.036	-0.053	-0.043	-0.04	t	18536	GACAPYRR LHLCD	Homo sapiens
1	1.00	1	1	0.04	0.043	0.028	0.04	0.042	t	75418	YPTFGEHK QEKDLEY	Mus musculu s
1	1.00	1	1	-0.04	-0.046	-0.04	-0.04	-0.042	t	123030	APPPAGA MIP	Oryctola gus cuniculu s

1	1.00	1	1	0	0	-0.004	0	0	t	71577	VTYEIDLTNKNAAKLRGD	Homo sapiens
1	1.00	1	1	0.01	0.01	0.01	0.01	0.008	t	17366	FPQPQQPQQS	Mus musculus
1	1.00	1	1	0.06	0.06	0.06	0.06	0.06	t	135728	NSTEAETI	Homo sapiens
1	1.00	1	1	0.04	0.04	0.04	0.04	0.04	t	135719	MEEKCSFE	Homo sapiens
1	1.00	1	1	0.02	0.02	0.02	0.02	0.02	t	135995	SIQSIVPALEIA	Homo sapiens
1	1.00	1	1	0.02	0.02	0.02	0.02	0.02	t	144036	PSIIGGSSVDLV	Homo sapiens
1	1.00	1	1	0.02	0.02	0.02	0.02	0.02	t	114772	DGVTYTNDCL	Homo sapiens
1	1.00	1	1	0.02	0.02	0.02	0.02	0.02	t	103121	DVGSLNGTYVNREPV	Homo sapiens
1	1.00	1	1	0	0.003	0	0	0	t	98330	PGSDVYYAGL	Mus musculus
1	1.00	1	1	0	0	0	0	0	t	97283	ETINFADAE	Oryctolagus cuniculus
1	1.00	1	1	-0.01	-0.01	-0.009	-0.013	-0.01	t	42633	MSPVNKSFEIEVTCS	Homo sapiens
1	1.00	1	1	-0.01	-0.004	-0.01	-0.009	-0.01	t	44851	NLQKDEPNGRA	Equus caballus
1	1.00	1	1	-0.01	-0.01	-0.01	-0.01	-0.01	t	63037	TAVSPTTLR	Mus musculus
1	1.00	1	1	-0.01	-0.01	-0.01	-0.01	-0.01	t	27462	IMDINKRK	Homo sapiens
1	1.00	1	1	0.01	0.013	0.012	0.01	0.011	t	62016	STYQYIYNLT	Sus scrofa
1	1.00	1	1	-0.01	-0.01	-0.01	-0.008	-0.01	t	70813	VRRKDDRRGALLSP	Homo sapiens

1	1.00	1	1	0	0	-0.005	0	0.002	t	127725	LPACNTPG	Homo sapiens
1	1.00	1	1	0.01	0.01	0.003	0.007	0.01	t	54206	RIPADVID AFHAAT	Homo sapiens
1	1.00	1	1	0.01	0.007	-0.001	0.01	0.012	t	36043	LGAALLQS AR	Oryctolagus cuniculus
1	1.00	1	1	0.02	0.02	0.024	0.02	0.02	t	157276	FQSQPPP GVPQG	Canis lupus
1	1.00	1	1	0	-0.002	-0.001	-0.001	0	t	2609	ALHQALQ DPRVRGL	Mus musculus
1	1.00	1	1	0	-0.002	-0.001	-0.001	0	t	2609	ALHQALQ DPRVRGL	Mus musculus
1	1.00	1	1	0.01	0.01	0.01	0.01	0.01	t	23190	GVTYEIDL TNKNA	Homo sapiens
1	1.00	1	1	-0.01	-0.012	-0.01	-0.007	-0.01	t	95814	SAQSGASA QSGA	Mus musculus
1	1.00	1	1	-0.01	-0.007	-0.011	-0.01	-0.011	t	60595	SQWNKPS KPKT	Mus musculus
1	1.00	1	1	-0.01	-0.01	-0.01	-0.01	-0.01	t	69595	VLLGACSF	Oryctolagus cuniculus
1	1.00	1	1	-0.02	-0.02	-0.01	-0.013	-0.019	t	122136	LGRGGSK	Homo sapiens
1	1.00	1	1	0	0.002	0.004	0.003	0.002	t	136015	YHVYEVNL VSEH	Homo sapiens
1	1.00	1	1	0	-0.003	0	0	0	t	50507	QDPRVR	Oryctolagus cuniculus
1	1.00	1	1	0	-0.003	0	0	0	t	50507	QDPRVR	Oryctolagus cuniculus

1	1.00	1	1	-0.02	-0.023	-0.029	-0.02	-0.02	t	113862	SITSGPMA RP	Oryctola gus cuniculu s
1	1.00	1	1	-0.03	-0.037	-0.03	-0.027	-0.031	t	32145	KLRRSTN WG	Homo sapiens
1	1.00	1	1	-0.01	-0.01	-0.005	-0.01	-0.013	t	30290	KEAFRLKN GRQE	Homo sapiens
1	1.00	1	1	-0.06	-0.056	-0.056	-0.06	-0.06	t	123281	GYWKLRG LA	Ovis aries
1	1.00	1	1	-0.06	-0.054	-0.056	-0.06	-0.06	t	123281	GYWKLRG LA	Ovis aries
1	1.00	1	1	0	0.002	0.006	0.003	0	t	65697	TPPRCSDQ MYCSLSR	Homo sapiens
1	1.00	1	1	0	0.002	0.006	0.003	0	t	65697	TPPRCSDQ MYCSLSR	Homo sapiens
1	1.00	1	1	0.02	0.017	0.018	0.02	0.021	t	126227	HNNAHYY VFFEEQED	Oryctola gus cuniculu s
1	1.00	1	1	0.03	0.026	0.033	0.033	0.03	t	115265	GKDITADN K	Oryctola gus cuniculu s
1	1.00	1	1	0.01	0.01	-0.004	0.004	0.01	t	55889	RSLPPIVKD ASIQVVSAI R	Homo sapiens
1	1.00	1	1	0.07	0.07	0.07	0.07	0.07	t	115906	PVEPFTES QS	Homo sapiens
1	1.00	1	1	0.05	0.05	0.05	0.05	0.05	t	96208	EDEEDEED KE	Homo sapiens
1	1.00	1	1	0.03	0.03	0.03	0.03	0.03	t	106850	SLQQLYDE YPGP	Mus musculu s
1	1.00	1	1	0.03	0.03	0.03	0.03	0.03	t	103578	SAAIGLSM AGSSAMI	Homo sapiens
1	1.00	1	1	0.03	0.03	0.03	0.03	0.03	t	107765	CESAQRPF GCC	Mus musculu s

1	1.00	1	1	0.03	0.03	0.03	0.03	0.03	t	107765	CESAQRPFGCC	Mus musculus
1	1.00	1	1	0.03	0.03	0.03	0.03	0.03	t	112398	WFAYWGQ	Homo sapiens
1	1.00	1	1	0.01	0.013	0.005	0.002	0.008	t	144208	AQAGGPDGAAAGGG	Capra hircus
1	1.00	1	1	0.01	0.005	0.005	0.002	0.008	t	144208	AQAGGPDGAAAGGG	Bos taurus
1	1.00	1	1	0.01	0.01	0.01	0.01	0.01	t	137883	GQNVLSADFTPG	Homo sapiens
1	1.00	1	1	0.01	0.01	0.01	0.01	0.01	t	120920	RLVEHLLDPSRYNKLIR	Oryctolagus cuniculus
1	1.00	1	1	-0.01	-0.01	-0.01	-0.01	-0.01	t	115907	PVVVPPFLQP	Homo sapiens
1	1.00	1	1	0.01	0.012	0.01	0.017	0.01	t	104155	NPEDCFKTGCNSPT	Homo sapiens
1	1.00	1	1	0.01	0.008	0.009	0.007	0.01	t	33126	KREMVIITFK	Mus musculus
1	1.00	1	1	-0.02	-0.027	-0.017	-0.019	-0.02	t	968	AEHQFKEKV	Homo sapiens
1	1.00	1	1	0	0	-0.001	0	-0.001	t	121474	KKAPAKKGEKVPKGK	Homo sapiens
1	1.00	1	1	-0.02	-0.016	-0.013	-0.013	-0.018	t	112860	YAQPSER	Homo sapiens
1	1.00	1	1	0.02	0.009	0.016	0.02	0.02	t	147083	LCSEYRNTQIYTIN	Oryctolagus cuniculus
1	1.00	1	1	0.02	0.009	0.016	0.02	0.02	t	147083	LCSEYRNTQIYTIN	Oryctolagus cuniculus
1	1.00	1	1	0	0.003	0.008	0.001	-0.002	t	16114	FHISCLTFGRET	Mus musculus

1	1.00	1	1	-0.01	-0.01	-0.012	-0.01	-0.01	t	8898	DKILVQAG EAETMTP	Homo sapiens
1	1.00	1	1	-0.01	-0.01	-0.012	-0.01	-0.01	t	8898	DKILVQAG EAETMTP	Homo sapiens
1	1.00	1	1	-0.02	-0.02	-0.03	-0.02	-0.02	t	140938	YLNATPAA KVLVEGNT DERG	Mus musculu s
1	1.00	1	1	0.03	0.03	0.03	0.032	0.03	t	22123	GRLGTYRY VSSGER	Homo sapiens
1	1.00	1	1	0.01	0.012	0.01	0.013	0.011	t	119837	ELIPSTSSA VPLIGK	Homo sapiens
1	1.00	1	1	0.05	0.048	0.057	0.051	0.049	t	103083	CGGVYDD KTGCA	Mus musculu s
1	1.00	1	1	-0.03	-0.028	-0.03	-0.023	-0.028	t	107366	FPPKPKD	Homo sapiens
1	1.00	1	1	-0.01	-0.01	0.007	-0.01	-0.014	t	145921	NPEIVIYQY	Mus musculu s
1	1.00	1	1	0	0.004	0.003	0.001	0	t	51374	QLINTNGS WHVN	Homo sapiens
1	1.00	1	1	0	0.004	0	0.001	0	t	75498	YQHKEGRF	Homo sapiens
1	1.00	1	1	0	0	0	0.003	0	t	42795	MTEQQW NFAGIEAA AS	Homo sapiens
1	1.00	1	1	0	0.005	0	0.003	0	t	42795	MTEQQW NFAGIEAA AS	Homo sapiens
1	1.00	1	1	-0.01	-0.012	-0.01	-0.011	-0.01	t	151031	LVKKGNSY PKLSKSY	Mus musculu s
1	1.00	1	1	-0.01	-0.01	-0.011	-0.006	-0.01	t	125029	EIERDTER DRFLSADE AV	Homo sapiens

1	1.00	1	1	0	-0.002	0	0.001	0	t	28911	ITGHRMAWDMMMNWS	Mus musculus
1	1.00	1	1	0	-0.002	0	-0.001	0	t	4737	ASREAK	Mus musculus
1	1.00	1	1	0	0	0	0.002	0	t	65515	TPCAAEEQKLPINA	Homo sapiens
1	1.00	1	1	-0.01	-0.008	-0.015	-0.007	-0.01	t	114941	RQDENYEEAIVHLKVAALGS	Homo sapiens
1	1.00	1	1	0.04	0.04	0.04	0.04	0.042	t	22783	GTPLDIDTASESS	Mus musculus
1	1.00	1	1	0	0	-0.006	0	0	t	91410	GQVKRDNLAPMSDGA	Oryctolagus cuniculus
1	1.00	1	1	-0.04	-0.036	-0.043	-0.039	-0.04	t	51758	QNRLESIKDS	Homo sapiens
1	1.00	1	1	0.06	0.06	0.06	0.06	0.061	t	6816	CPNSLTPADPTMDY	Mus musculus
1	1.00	1	1	-0.01	-0.01	-0.01	-0.01	-0.011	t	131295	ECQEATLMVMVSKDLFGTGK	Callithrix pygmaea
1	1.00	1	1	-0.02	-0.02	-0.02	-0.016	-0.018	t	118575	RATWKSNY	Homo sapiens
1	1.00	1	1	0	0	0	0	0	t	6540	CLAVLLGA	Oryctolagus cuniculus
1	1.00	1	1	-0.05	-0.05	-0.05	-0.05	-0.05	t	145832	IPLGTRPP	Mus musculus
1	1.00	1	1	-0.01	-0.01	-0.01	-0.01	-0.012	t	131913	IGGGWGNSDARYTPG	Homo sapiens
1	1.00	1	1	0.05	0.052	0.05	0.051	0.049	t	151026	LREQLSSVSSFERFE	Homo sapiens
1	1.00	1	1	0.02	0.016	0.02	0.023	0.02	t	76350	YVLLSEKKIS	Oryctolagus cuniculus

1	1.00	1	1	-0.01	-0.01	-0.01	-0.01	-0.011	t	131130	VIASLTCGRRFEYDDPRFLR	Homo sapiens
1	1.00	1	1	0.02	0.02	0.016	0.02	0.02	t	28413	IRVEES	Homo sapiens
1	1.00	1	1	0.02	0.02	0.021	0.017	0.019	t	44227	NIFSGFTPEFLEQAF	Homo sapiens
1	1.00	1	1	0.02	0.02	0.022	0.02	0.016	t	55924	RSQDACIEFVQQEVD	Mus musculus
1	1.00	1	1	0.03	0.03	0.024	0.03	0.03	t	75920	YSVLYNSTFFSTFKCYG	Mus musculus
1	1.00	1	1	0.03	0.027	0.024	0.03	0.03	t	75920	YSVLYNSTFFSTFKCYG	Oryctolagus cuniculus
1	1.00	1	1	0.03	0.03	0.024	0.03	0.03	t	75920	YSVLYNSTFFSTFKCYG	Homo sapiens
1	1.00	1	1	0.04	0.04	0.04	0.04	0.04	t	99775	WELQGDRRCQSQLER	Homo sapiens
1	1.00	1	1	0.04	0.04	0.04	0.04	0.04	t	78001	SEGLKEET	Mus musculus
1	1.00	1	1	0.02	0.02	0.02	0.02	0.02	t	149042	FLHTRLLFRIVSYSG	Rattus norvegicus
1	1.00	1	1	0.02	0.02	0.02	0.02	0.02	t	120939	SEQSKEVYYE	Mus musculus
1	1.00	1	1	0	0	0	0	0	t	141581	YVYRWVEAECVA	Mus musculus
1	1.00	1	1	0	0	0	0	0	t	153423	MPKDMLPYANPNGGGS	Mus musculus
1	1.00	1	1	0	0	0	0	0	t	119654	TSGVHTFP	Homo sapiens
1	1.00	1	1	0	0	0	0	0	t	123075	DFLGKNPYL	Ovis aries
1	1.00	1	1	0	0	0	0	0	t	108037	MTQMPTNHAMQLILE	Homo sapiens

1	1.00	1	1	-0.02	-0.02	-0.02	-0.02	-0.02	t	135900	ATCRFYTLDSIK	Homo sapiens
1	1.00	1	1	0.01	0.012	0.004	0.007	0.009	t	95093	HQQGGWSTLIEDNIP	Homo sapiens
1	1.00	1	1	0.01	0.006	0.008	0.013	0.01	t	95728	QPPVSQFHIQGQ	Oryctolagus cuniculus
1	1.00	1	1	0.03	0.03	0.03	0.03	0.031	t	61800	STNS	Mus musculus
1	1.00	1	1	0.01	0.01	0.01	0.01	0.007	t	151731	SAQSQRAPDR	Mus musculus
1	1.00	1	1	0	0	-0.004	0.003	0	t	38764	LPVWARPD	Homo sapiens
1	1.00	1	1	0	0.003	0	0	-0.001	t	135477	LGAGLG	Mus musculus
1	1.00	1	1	0.01	0.01	0.01	0.01	0.01	t	107034	YTTRR	Homo sapiens
1	1.00	1	1	0.06	0.058	0.058	0.057	0.06	t	46849	PALDKLKLTGDEATGA	Mus musculus
1	1.00	1	1	0.06	0.058	0.058	0.057	0.06	t	46849	PALDKLKLTGDEATGA	Mus musculus
1	1.00	1	1	-0.03	-0.028	-0.023	-0.033	-0.03	t	95898	TDYKKY	Homo sapiens
1	1.00	1	1	-0.04	-0.04	-0.04	-0.04	-0.042	t	2558	ALGGWGLHQPPPQIFLKILP	Homo sapiens
1	1.00	1	1	-0.01	-0.012	-0.012	-0.011	-0.01	t	17642	FRRYQEER	Anas platyrhynchos
1	1.00	1	1	-0.01	-0.01	-0.02	-0.01	-0.008	t	104231	SAADEKFIKETEHAKDYG	Oryctolagus cuniculus
1	1.00	1	1	0	0.004	0.006	0.001	0	t	28637	ISNRDF	Homo sapiens

1	1.00	1	1	-0.01	-0.012	-0.01	-0.007	-0.01	t	95827	SGASAQSGASAQ	Mus musculus
1	1.00	1	1	0.01	0.008	0.021	0.013	0.013	t	78194	LITVNPI	Mus musculus
1	1.00	1	1	0	0	0.011	0	-0.002	t	47006	PAYEKL	Mus musculus
1	1.00	1	1	0.03	0.034	0.037	0.037	0.033	t	135546	DWEYS	Homo sapiens
1	1.00	1	1	0	-0.003	0	-0.001	-0.001	t	32889	KPTDG	Oryctolagus cuniculus
1	1.00	1	1	0.01	0.01	0.01	0.01	0.01	t	94442	CCHPACG	Capra hircus
1	1.00	1	1	0	0.002	0	-0.003	-0.003	t	106317	CSKIQSTIFRGY	Homo sapiens
1	1.00	1	1	-0.01	-0.008	-0.005	-0.007	-0.01	t	12571	EIRQVGDFHYVTGM	Homo sapiens
1	1.00	1	1	-0.02	-0.027	-0.027	-0.013	-0.018	t	136137	APDRKGFRLLLASPRSCYKL	Homo sapiens
1	1.00	1	1	0.02	0.018	0.013	0.017	0.018	t	29732	IYYHGEPIPVTVAVTNSTEK	Mus musculus
1	1.00	1	1	-0.02	-0.017	-0.026	-0.02	-0.018	t	41126	MASLIYRQLL	Mus musculus
1	1.00	1	1	0	0.002	0	0.003	-0.002	t	121925	TPFLPKIFIKPNAQK	Homo sapiens
1	1.00	1	1	-0.02	-0.017	-0.031	-0.02	-0.018	t	111219	DVNVYHIFFQMSLWLPSEAT	Mus musculus
1	1.00	1	1	0.03	0.03	0.03	0.03	0.03	t	149073	MTSSETQ	Sus scrofa
1	1.00	1	1	0.03	0.03	0.03	0.03	0.03	t	134788	RRGPSLGASSHQHSR	Homo sapiens

1	1.00	1	1	0.03	0.03	0.03	0.03	0.03	t	132813	FGENNNM	Homo sapiens
1	1.00	1	1	0.03	0.03	0.03	0.03	0.03	t	108282	AQAYDLSPS	Homo sapiens
1	1.00	1	1	0.03	0.03	0.03	0.03	0.03	t	115827	KKNQDKTEIP	Homo sapiens
1	1.00	1	1	0.03	0.03	0.03	0.03	0.03	t	79889	KQLTFEGKPALE	Mus musculus
1	1.00	1	1	0.03	0.03	0.03	0.03	0.03	t	79889	KQLTFEGKPALE	Mus musculus
1	1.00	1	1	0.01	0.01	0.01	0.01	0.01	t	149512	VLQESTYQLV	Homo sapiens
1	1.00	1	1	0.01	0.01	0.01	0.01	0.01	t	149512	VLQESTYQLV	Mus musculus
1	1.00	1	1	0.01	0.01	0.01	0.01	0.01	t	117103	TYQDFSTRPRSF	Homo sapiens
1	1.00	1	1	0.01	0.01	0.01	0.01	0.01	t	117103	TYQDFSTRPRSF	Mus musculus
1	1.00	1	1	0.01	0.01	0.01	0.01	0.01	t	79638	RLVTDEI	Oryctolagus cuniculus
1	1.00	1	1	-0.01	-0.01	-0.01	-0.01	-0.01	t	151295	LVLAKLGKDAGQPK	Mus musculus
1	1.00	1	1	0	-0.001	0	0.003	0	t	130612	SPNTAHITSKSL	Camelus dromedarius
1	1.00	1	1	0	-0.001	0	0.003	0	t	130612	SPNTAHITSKSL	Camelus dromedarius
1	1.00	1	1	0	0	0.002	0.004	0	t	75152	YNGEEYLILSARDVL	Homo sapiens
1	1.00	1	1	0	0	0.002	0.004	0	t	67656	VAKVNIKPLEDKILV	Homo sapiens

1	1.00	1	1	0	0	0.002	0.004	0	t	22882	GTVVAVGPGRWDEDG	Homo sapiens
1	1.00	1	1	0	0	0.002	0.004	0	t	4132	ARDVLAVVSK	Homo sapiens
1	1.00	1	1	-0.01	-0.015	-0.019	-0.012	-0.009	t	51474	QLPNVGITQGIV	Bos taurus
1	1.00	1	1	0	0.002	-0.008	0.001	0	t	23296	GWVFGSTMNNKSQSVII	Homo sapiens
1	1.00	1	1	0	-0.002	-0.002	-0.007	0	t	122295	DDVWLP	Mus musculus
1	1.00	1	1	-0.02	-0.018	-0.015	-0.013	-0.022	t	131898	GRPLSVQQQMMQQSR	Homo sapiens
1	1.00	1	1	0	-0.002	-0.012	-0.003	0.001	t	79867	INNLIVELIRGT	Mus musculus
1	1.00	1	1	-0.01	-0.015	-0.014	-0.007	-0.008	t	157771	QGDRYTGEWYQGKK	Canis lupus
1	1.00	1	1	0.03	0.027	0.027	0.03	0.029	t	55449	RQLQNSMSGASADSTQA	Oryctolagus cuniculus
1	1.00	1	1	0.03	0.03	0.025	0.03	0.031	t	29812	KAGSTVYAVTAVTSIFSVG	Homo sapiens
1	1.00	1	1	-0.05	-0.053	-0.065	-0.05	-0.05	t	97605	RIVRGPANLRNLKEFKE	Oryctolagus cuniculus
1	1.00	1	1	-0.05	-0.053	-0.065	-0.05	-0.05	t	97605	RIVRGPANLRNLKEFKE	Oryctolagus cuniculus
1	1.00	1	1	0.02	0.024	0.02	0.021	0.02	t	58458	SICGNNQLCKSICKT	Homo sapiens
1	1.00	1	1	-0.02	-0.022	-0.02	-0.019	-0.02	t	149431	QPFPPQQPYP	Mus musculus

1	1.00	1	1	0.01	0.01	0.005	0.01	0.01	t	44869	NLSGKAIN HIRS	Homo sapiens
1	1.00	1	1	-0.01	-0.01	-0.01	-0.01	-0.01	t	156875	GHTSLR	Gallus gallus
1	1.00	1	1	0.03	0.028	0.027	0.033	0.032	t	32692	KPEKEKAS VPGGGD M	Rattus norvegic us
1	1.00	1	1	-0.01	-0.006	0.001	-0.009	-0.011	t	11029	EAELTGYG	Homo sapiens
1	1.00	1	1	-0.07	-0.033	-0.08	-0.07	-0.07	t	12792	EKLCVWN NKTPHAIA	Oryctola gus cuniculu s
1	1.00	1	1	-0.07	-0.07	-0.08	-0.07	-0.07	t	12792	EKLCVWN NKTPHAIA	Oryctola gus cuniculu s
1	1.00	1	1	-0.07	-0.033	-0.08	-0.07	-0.07	t	12792	EKLCVWN NKTPHAIA	Oryctola gus cuniculu s
1	1.00	1	1	0.02	0.02	0.02	0.02	0.02	t	133795	DFTYDQV AFNGLPQF	Homo sapiens
1	1.00	1	1	0.02	0.02	0.02	0.02	0.02	t	133795	DFTYDQV AFNGLPQF	Homo sapiens
1	1.00	1	1	0.02	0.02	0.02	0.02	0.02	t	133795	DFTYDQV AFNGLPQF	Homo sapiens
1	1.00	1	1	0.02	0.02	0.02	0.02	0.02	t	114775	DKEGKDVL VC	Homo sapiens
1	1.00	1	1	0	0	0	0	0	t	78230	PHMNNYH L	Homo sapiens
1	1.00	1	1	-0.02	-0.02	-0.02	-0.02	-0.02	t	151729	RYSPSLNT RL	Mus musculu s
1	1.00	1	1	-0.04	-0.04	-0.04	-0.04	-0.04	t	111157	CMPYGSL AC	Homo sapiens
1	1.00	1	1	-0.02	-0.018	-0.019	-0.018	-0.02	t	41115	MASCRPID KFDQGW	Homo sapiens
1	1.00	1	1	-0.05	-0.049	-0.055	-0.05	-0.053	t	56852	SALEDKLA DYKRRM	Myodes glareolu s
1	1.00	1	1	0.05	0.048	0.057	0.047	0.048	t	142442	LKGDDSA GEQEL	Mus musculu s

1	1.00	1	1	-0.02	-0.018	-0.037	-0.023	-0.02	t	8127	DEPTLLYVLFEVFDV	Homo sapiens
1	1.00	1	1	0.01	0.012	0.015	0.013	0.01	t	5274	AVASPPATASVESSPLPAAA	Homo sapiens
1	1.00	1	1	-0.01	-0.006	-0.01	-0.009	-0.01	t	43039	MYASSGIG	Homo sapiens
1	1.00	1	1	0.03	0.032	0.03	0.027	0.028	t	115869	NKKIEKFQSE	Homo sapiens
1	1.00	1	1	-0.04	-0.043	-0.035	-0.037	-0.042	t	134198	PVGPAPGMRPPMGG	Oryctolagus cuniculus
1	1.00	1	1	-0.01	-0.006	0.001	-0.003	-0.009	t	122101	GKGAGDL	Homo sapiens
1	1.00	1	1	0.02	0.014	0.02	0.02	0.018	t	123323	ILNKPLITVSNSKIL	Oryctolagus cuniculus
1	1.00	1	1	0.02	0.02	0.02	0.02	0.021	t	107814	EGLHNHY	Homo sapiens
1	1.00	1	1	0.02	0.018	0.019	0.019	0.02	t	61573	STEELFKEY	Mus musculus
1	1.00	1	1	0.01	0.01	-0.001	0.01	0.012	t	153469	STETGNQHHYQTRVVSNANK	Mus musculus
1	1.00	1	1	-0.03	-0.03	-0.021	-0.023	-0.03	t	136007	VKEKGMAALPRL	Homo sapiens
1	1.00	1	1	0.01	0.011	-0.009	0.003	0.012	t	120653	KAAIPEDKEYDVSGR	Equus caballus
1	1.00	1	1	0	0	0	0.004	0.001	t	11813	EENVEENV	Homo sapiens
1	1.00	1	1	0.03	0.032	0.023	0.021	0.028	t	115745	FTESQSLTLT	Homo sapiens
1	1.00	1	1	-0.04	-0.04	-0.033	-0.04	-0.044	t	133554	EGLYSPWWPRSLPVL	Mus musculus

1	1.00	1	1	0.05	0.05	0.05	0.05	0.05	t	98883	LNENKVLVLD	Homo sapiens
1	1.00	1	1	0	-0.003	0	0	0.001	t	24009	HHPSTDKEQTNLY	Oryctolagus cuniculus
1	1.00	1	1	0.03	0.03	0.03	0.03	0.03	t	137816	DAETLMDAAVDV	Oryctolagus cuniculus
1	1.00	1	1	0.01	0.01	0.01	0.01	0.01	t	149062	IPPNPSQ	Sus scrofa
1	1.00	1	1	0.01	0.01	0.01	0.01	0.01	t	119463	HKKSLSSPS	Homo sapiens
1	1.00	1	1	0.01	0.01	0.01	0.01	0.01	t	121849	SGVPLYSHISYVISC	Mus musculus
1	1.00	1	1	0.01	0.01	0.01	0.01	0.01	t	121849	SGVPLYSHISYVISC	Mus musculus
1	1.00	1	1	0.01	0.01	0.01	0.01	0.01	t	122488	MSAKAALEPRLLAPG	Mus musculus
1	1.00	1	1	-0.01	-0.01	-0.01	-0.01	-0.01	t	115258	FYPELFRQFYQLDAYPSGAW	Homo sapiens
1	1.00	1	1	-0.01	-0.01	-0.01	-0.01	-0.01	t	77924	IYTSPTFPPTAH	Mus musculus
1	1.00	1	1	-0.03	-0.03	-0.03	-0.03	-0.03	t	112853	TPTFMYF	Homo sapiens
1	1.00	1	1	-0.02	-0.02	-0.027	-0.02	-0.02	t	100032	FEPNTVKAHAAY	Oryctolagus cuniculus
1	1.00	1	1	-0.03	-0.028	-0.039	-0.033	-0.031	t	45563	NQGSLKLKSFDEHLK	Homo sapiens
1	1.00	1	1	0.03	0.028	0.031	0.023	0.03	t	134786	RPVDQY	Mus musculus
1	1.00	1	1	0.03	0.027	0.028	0.03	0.029	t	64085	THTMIFDNAFNCTFEYI	Oryctolagus cuniculus
1	1.00	1	1	0.03	0.026	0.028	0.029	0.03	t	78871	VDVDPNKGKWKEHIKEVTE	Oryctolagus cuniculus

1	1.00	1	1	0.03	0.028	0.028	0.029	0.03	t	78871	VDVDPNKGKWKEHIKEVTE	Mus musculus
1	1.00	1	1	-0.05	-0.05	-0.052	-0.054	-0.05	t	69856	VLWVVKSTPA	Homo sapiens
1	1.00	1	1	0	0	0	0	0	t	5582	AVTRGTINDPQRAKE	Mus musculus
1	1.00	1	1	0	0	0	0	0	t	5582	AVTRGTINDPQRAKE	Mus musculus
1	1.00	1	1	0.03	0.03	0.024	0.03	0.028	t	15509	FEDVKGTGGESRDLPSGQG	Mus musculus
1	1.00	1	1	0	0	0	0.006	0.002	t	27463	IMDINKRKYDSLK	Homo sapiens
1	1.00	1	1	0	0	-0.015	0	0.001	t	48618	PNDPNRNVD	Mus musculus
1	1.00	1	1	0.01	0.008	0.01	0.009	0.01	t	65684	TPPHSARSKFGYGA	Mus musculus
1	1.00	1	1	-0.02	-0.024	-0.02	-0.017	-0.02	t	19737	GGGAGAGGAGAGGGGR	Oryctolagus cuniculus
1	1.00	1	1	0.02	0.016	0.017	0.019	0.02	t	127935	ARLSAKPAPPKPEPK	Oryctolagus cuniculus
1	1.00	1	1	0.01	0.012	0.009	0.007	0.012	t	8383	DGFKVSNSSA	Homo sapiens
1	1.00	1	1	0.01	0.009	-0.006	0.009	0.012	t	10358	DTIASGSQRSTNSAS	Mus musculus
1	1.00	1	1	0.03	0.03	0.019	0.03	0.03	t	31794	KLDVVKLLYNEQFAVQGLLR	Homo sapiens
1	1.00	1	1	0	-0.003	-0.003	0	0.002	t	12626	EKAFRELADA	Oryctolagus cuniculus

1	1.00	1	1	0	0	0	0.004	-0.002	t	132027	QCSTIGQMPNNQSIN	Homo sapiens
1	1.00	1	1	0	0	0	-0.002	0	t	7061	CSQHLPYIE	Homo sapiens
1	1.00	1	1	-0.05	-0.05	-0.05	-0.05	-0.051	t	114223	PFSRVVHL	Rattus norvegicus
1	1.00	1	1	-0.02	-0.02	-0.02	-0.02	-0.02	t	95016	IRLPSDDVWLPDLVL	Rattus norvegicus
1	1.00	1	1	-0.02	-0.02	-0.02	-0.02	-0.02	t	24052	HIHSVW	Homo sapiens
1	1.00	1	1	-0.01	-0.01	-0.017	-0.01	-0.008	t	108998	FVCGDRGFYFNK	Mus musculus
1	1.00	1	1	-0.01	-0.01	-0.01	-0.01	-0.011	t	118763	CYGSFARPMN	Mus musculus
1	1.00	1	1	-0.06	-0.06	-0.065	-0.06	-0.063	t	131104	TGALFKHSENYK	Oryctolagus cuniculus
1	1.00	1	1	0	-0.002	0.003	0.003	0.001	t	52094	QQPP	Mus musculus
1	1.00	1	1	0	0	-0.008	-0.003	0.001	t	33538	KSTWDLALNNM	Homo sapiens
1	1.00	1	1	0.04	0.04	0.04	0.04	0.04	t	79413	TCPLPDPDSQYCE	Mus musculus
1	1.00	1	1	-0.01	-0.003	-0.016	-0.01	-0.011	t	43547	NDYDGFLENAHEHHGVYNQG	Cavia porcellus
1	1.00	1	1	0.01	0.01	0.01	0.007	0.01	t	33745	KTKRNTN	Homo sapiens
1	1.00	1	1	0.02	0.02	0.014	0.02	0.021	t	76185	YTTGGAQSHTLR	Mus musculus
1	1.00	1	1	-0.02	-0.022	-0.015	-0.017	-0.021	t	159085	RYKYYPETPQGLAKII	Mus musculus

1	1.00	1	1	0.01	0.01	0.01	0.01	0.008	t	144660	GREGYPADGGASASASAS	Oryctolagus cuniculus
1	1.00	1	1	0.04	0.04	0.04	0.04	0.04	t	145371	NALTESKYVKLL	Homo sapiens
1	1.00	1	1	0.04	0.04	0.04	0.04	0.04	t	103000	AAAGDLVGPGCAEYA	Homo sapiens
1	1.00	1	1	0.02	0.02	0.02	0.02	0.02	t	143675	DRVDLGTLRGYY	Mus musculus
1	1.00	1	1	0.02	0.02	0.02	0.02	0.02	t	153337	ACNVYEAYWCGGGS	Mus musculus
1	1.00	1	1	0.02	0.02	0.02	0.02	0.02	t	135778	VEGTSFLT	Homo sapiens
1	1.00	1	1	0.02	0.02	0.02	0.02	0.02	t	153339	ACPPYETWWCGGGS	Mus musculus
1	1.00	1	1	0.02	0.02	0.02	0.02	0.02	t	115280	HKEMPFPKYPVEPFTESQSL	Homo sapiens
1	1.00	1	1	0.02	0.02	0.02	0.02	0.02	t	79640	SASIRPQ	Oryctolagus cuniculus
1	1.00	1	1	0.02	0.02	0.02	0.02	0.02	t	88060	RMAFTEIKVQ	Homo sapiens
1	1.00	1	1	0.02	0.02	0.02	0.02	0.02	t	51830	QPFPSQQPYL	Mus musculus
1	1.00	1	1	0	0	0	0	0	t	149059	HLYNPTQ	Sus scrofa
1	1.00	1	1	0.07	0.072	0.065	0.071	0.069	t	23985	HHFQSHGQLSDHPHALSSSS	Homo sapiens
1	1.00	1	1	-0.01	-0.01	-0.003	-0.01	-0.014	t	107380	GEPGIAGFKGDQGPK	Mus musculus
1	1.00	1	1	0.01	0.012	0.01	0.017	0.015	t	119673	WNPDDYGGVK	Homo sapiens
1	1.00	1	1	0.03	0.03	0.028	0.03	0.029	t	51852	QPHDLGKVGEVIVTKD	Rattus norvegicus

1	1.00	1	1	0.02	0.02	0.02	0.02	0.02	t	136234	FPGLITLTISLLDTSNLELP	Homo sapiens
1	1.00	1	1	0.05	0.051	0.043	0.049	0.051	t	29278	IVGAGDSS	Equus caballus
1	1.00	1	1	-0.01	-0.006	-0.007	-0.007	-0.01	t	7063	CSQHLPYIEQGMQLA	Homo sapiens
1	1.00	1	1	-0.01	-0.008	-0.007	-0.007	-0.01	t	7063	CSQHLPYIEQGMQLA	Homo sapiens
1	1.00	1	1	-0.01	-0.01	-0.01	-0.008	-0.01	t	110879	GYRGRSSIRTGVKRPAVSK	Homo sapiens
1	1.00	1	1	0.06	0.058	0.048	0.053	0.06	t	108170	TTANPSCPEGT	Rattus norvegicus
1	1.00	1	1	-0.03	-0.028	-0.032	-0.027	-0.03	t	9586	DPAFR	Homo sapiens
1	1.00	1	1	0.03	0.034	0.03	0.031	0.031	t	71848	VVTGATVS	Homo sapiens
1	1.00	1	1	0	-0.002	0	-0.001	0	t	117103	TYQDFSTRPRSF	Mus musculus
1	1.00	1	1	-0.02	-0.02	-0.027	-0.02	-0.021	t	4681	ASPTGYDWRADW	Mus musculus
1	1.00	1	1	-0.05	-0.048	-0.041	-0.05	-0.052	t	100129	HYKWLNDPLAAW	Ovis aries
1	1.00	1	1	-0.03	-0.03	-0.03	-0.03	-0.03	t	136415	KFAGRNFRNPLAK	Homo sapiens
1	1.00	1	1	-0.03	-0.03	-0.03	-0.03	-0.03	t	136415	KFAGRNFRNPLAK	Homo sapiens
1	1.00	1	1	-0.01	-0.01	-0.01	-0.008	-0.01	t	27554	IMSGEKPSMEDVVN	Homo sapiens
1	1.00	1	1	0.02	0.023	0.022	0.02	0.02	t	5962	CAALASLFLSLGPQQK	Mus musculus

1	1.00	1	1	0	0	-0.004	-0.006	-0.002	t	106057	REFSPRGQHGRRERA	Homo sapiens
1	1.00	1	1	0.03	0.032	0.03	0.031	0.03	t	116930	AYKLPQTDDST	Homo sapiens
1	1.00	1	1	0.02	0.028	0.019	0.02	0.018	t	4528	ASLKDALLKYIYDNRGTLI	Gallus gallus
1	1.00	1	1	0.02	0.022	0.021	0.017	0.017	t	106539	ILRWGNESEI	Homo sapiens
1	1.00	1	1	0.01	0.008	0.01	0.009	0.01	t	50609	QEGVQQEGA	Mus musculus
1	1.00	1	1	-0.04	-0.041	-0.039	-0.039	-0.04	t	54104	RHVLGR	Homo sapiens
1	1.00	1	1	-0.03	-0.03	-0.05	-0.03	-0.027	t	141737	REAYYLWKQYNPTGKG	Rattus norvegicus
1	1.00	1	1	0.03	0.028	0.03	0.031	0.03	t	149428	QLQLQNPSQQ	Mus musculus
1	1.00	1	1	0.02	0.024	0.024	0.027	0.022	t	122730	DPAQPPRD	Homo sapiens
1	1.00	1	1	0	0.003	0.005	0	0.003	t	116284	CAYSVAHLC	Mus musculus
1	1.00	1	1	-0.03	-0.026	-0.03	-0.03	-0.031	t	146496	PPGAQPPTSRKEAM	Homo sapiens
1	1.00	1	1	0.02	0.02	0.022	0.02	0.021	t	106965	VKGEEKEPSK	Homo sapiens
1	1.00	1	1	-0.01	-0.002	-0.006	-0.01	-0.012	t	58049	SGGKLVGILTSRNET	Gallus gallus
1	1.00	1	1	-0.01	-0.002	-0.006	-0.01	-0.012	t	58049	SGGKLVGILTSRNET	Gallus gallus

1	1.00	1	1	0.01	0.01	0.01	0.01	0.01	t	151019	LCKLRGVAPL	Mus musculus
1	1.00	1	1	0	0	-0.004	0	0.001	t	6139	CDSSDRPRVYTIT	Mus musculus
1	1.00	1	1	0.07	0.07	0.061	0.07	0.072	t	78573	YTTEGGTKSEVEDVIPEG	Oryctolagus cuniculus
1	1.00	1	1	-0.01	-0.013	-0.017	-0.01	-0.009	t	114149	YGSLPQKAQGHRPQDE	Oryctolagus cuniculus
1	1.00	1	1	0.03	0.03	0.03	0.03	0.03	t	108579	PPLLLTDVT	Homo sapiens
1	1.00	1	1	0.03	0.03	0.03	0.03	0.03	t	105023	VIQGSQL	Homo sapiens
1	1.00	1	1	0.03	0.03	0.03	0.03	0.03	t	109854	RQMVEKESLDQA	Mus musculus
1	1.00	1	1	0.03	0.03	0.03	0.03	0.03	t	93326	LSFSDSNLKD	Ovis aries
1	1.00	1	1	0.01	0.01	0.01	0.01	0.01	t	124285	HPLSPHPSY	Homo sapiens
1	1.00	1	1	0.01	0.01	0.01	0.01	0.01	t	112149	GVGWIRQ	Homo sapiens
1	1.00	1	1	0	0	-0.002	0	-0.001	t	50900	QGLPQEVLNE	Homo sapiens
1	1.00	1	1	0	0.004	0	0.001	0	t	23068	GVNLEDPASRDL	Homo sapiens
1	1.00	1	1	0	0.004	0	0.001	0	t	23068	GVNLEDPASRDL	Homo sapiens
1	1.00	1	1	0	0	-0.012	-0.002	0	t	45464	NPRENKLKQPGDRADGQPAG	Homo sapiens
1	1.00	1	1	0	0	-0.012	-0.002	0	t	45464	NPRENKLKQPGDRADGQPAG	Homo sapiens
1	1.00	1	1	0	0	-0.005	-0.007	0	t	146248	FLFCGSMMATGKIL	Homo sapiens

1	1.00	1	1	0.02	0.02	0.018	0.02	0.02	t	110606	GNPGTDGI PGAKG	Rattus norvegic us
1	1.00	1	1	0	-0.003	0	0	0	t	135472	KDRHDF	Oryctola gus cuniculu s
1	1.00	1	1	0	0	0	0	0	t	118374	GKALCWV CAQSRKH RD	Homo sapiens
1	1.00	1	1	0	0	0	0	0	t	38272	LNLTTWN P	Oryctola gus cuniculu s
1	1.00	1	1	0.01	0.01	0.011	0.006	0.011	t	48129	PKQGPLP N	Homo sapiens
1	1.00	1	1	-0.02	-0.02	-0.035	-0.014	-0.018	t	7705	DCHLLGTE ELSGIPPPP RGV	Homo sapiens
1	1.00	1	1	-0.01	-0.01	-0.003	-0.003	-0.01	t	119476	KKKKYAEA V	Homo sapiens
1	1.00	1	1	0.03	0.033	0.031	0.03	0.029	t	117530	IEFRDSDA ESSDGEDF IVEE	Mus musculu s
1	1.00	1	1	0.02	0.02	0.02	0.024	0.02	t	118645	VAVSAAP GSAAPAA G	Homo sapiens
1	1.00	1	1	0.02	0.02	0.02	0.02	0.02	t	130581	QAFDSH	Mus musculu s
1	1.00	1	1	0	0	-0.005	-0.004	0	t	133870	LAKLAGGV AVICAGA	Homo sapiens
1	1.00	1	1	0.02	0.02	0.02	0.024	0.021	t	118019	MGRGAPP PGMMG	Homo sapiens
1	1.00	1	1	0.02	0.02	0.015	0.02	0.02	t	136087	VETDKATV GF	Oryctola gus cuniculu s
1	1.00	1	1	-0.01	-0.008	-0.011	-0.009	-0.01	t	40018	LTLNLITE MG	Homo sapiens
1	1.00	1	1	0	0	-0.02	-0.006	0	t	48319	PLFDGIFCS SSNFLGISF L	Homo sapiens
1	1.00	1	1	0.01	0.01	0.01	0.01	0.009	t	98955	QFEDFFPA SSRDQSS	Homo sapiens

1	1.00	1	1	0	-0.004	0	-0.003	0	t	117909	HKGFKGVD	Oryctolagus cuniculus
1	1.00	1	1	0	0	0	0	0	t	73449	YASSGIGP	Oryctolagus cuniculus
1	1.00	1	1	0	0	0	0	0	t	9755	DPRVRGLYFPA	Mus musculus
1	1.00	1	1	0	0	0	0	-0.001	t	144385	KPLEEVLNL	Mus musculus
1	1.00	1	1	0	0	0	0	-0.001	t	146625	WNWFDITNW	Homo sapiens
1	1.00	1	1	0	0	0	0	-0.001	t	146624	WNWFDITN	Homo sapiens
1	1.00	1	1	0	0	0	0	-0.001	t	134989	GPPGARGLTGRPGDAGPP	Mus musculus
1	1.00	1	1	0	0	0	0	-0.001	t	103643	TGALQERSK	Mus musculus
1	1.00	1	1	0	0	0	0	-0.001	t	69178	VKAETRLNPDLQPTE	Mus musculus
1	1.00	1	1	0	0	0	0	-0.001	t	69178	VKAETRLNPDLQPTE	Mus musculus
1	1.00	1	1	0	0	0	0	-0.001	t	51147	QIVGGVYLL	Mus musculus
1	1.00	1	1	0	0	0	0	-0.001	t	51147	QIVGGVYLL	Mus musculus
1	1.00	1	1	0	0	0	0	-0.001	t	23876	HFVQQTPKSQPTLV	Mus musculus
1	1.00	1	1	0	0	0	0	-0.001	t	32990	KQHTVTTTTKGE	Mus musculus

1	1.00	1	1	0	0	0	0	-0.001	t	24591	HQLDPAF GANSTNP D	Mus musculu s
1	1.00	1	1	0	0	0	0	-0.001	t	10692	DVPDYASL	Mus musculu s
1	1.00	1	1	0.02	0.023	0.02	0.02	0.018	t	135465	GSDYEDRY YREN	Mus musculu s
1	1.00	1	1	-0.04	-0.038	-0.05	-0.039	-0.036	t	55781	RRYPF	Homo sapiens
1	1.00	1	1	0	0	-0.002	0	-0.001	t	21469	GNLLLSCL	Mus musculu s
1	1.00	1	1	-0.02	-0.02	-0.021	-0.02	-0.02	t	108634	SKVEQEET NPEVLIKD L	Canis lupus
1	1.00	1	1	0	0	0	0	0	t	40076	LTPCD	Homo sapiens
1	1.00	1	1	0	0	0	0	0	t	40076	LTPCD	Mus musculu s
1	1.00	1	1	0	0	-0.003	0.003	0	t	48414	PLLVETWK	Homo sapiens
1	1.00	1	1	0.04	0.04	0.04	0.04	0.041	t	115212	DTQAIVQ NND	Homo sapiens
1	1.00	1	1	-0.04	-0.038	-0.035	-0.037	-0.04	t	33259	KRYISWCL WWLQYF	Homo sapiens
1	1.00	1	1	-0.04	-0.038	-0.021	-0.037	-0.043	t	33448	KSMRSAN KP	Homo sapiens
1	1.00	1	1	0.04	0.038	0.033	0.037	0.039	t	56329	RVKMEKLK LKGTTYG MC	Mus musculu s
1	1.00	1	1	0.01	0.017	0.017	0.007	0.008	t	136728	VNYNGKE HLLIP	Gallus gallus
1	1.00	1	1	0.04	0.04	0.04	0.04	0.04	t	78295	WADLYSP	Homo sapiens

1	1.00	1	1	0	0	0	0	0	t	144665	GREGYPADGGITCFLTAA	Oryctolagus cuniculus
1	1.00	1	1	0	0	0	0	0	t	146920	FGGYTLRGTSIFG	Homo sapiens
1	1.00	1	1	-0.01	-0.006	-0.014	-0.01	-0.01	t	21838	GPWSPIDIHHLS	Mus musculus
1	1.00	1	1	-0.01	-0.01	-0.014	-0.01	-0.01	t	21838	GPWSPIDIHHLS	Canis lupus
1	1.00	1	1	-0.01	-0.01	-0.014	-0.01	-0.01	t	21838	GPWSPIDIHHLS	Canis lupus
1	1.00	1	1	-0.03	-0.03	-0.03	-0.03	-0.03	t	123230	GGPGIGRAA	Oryctolagus cuniculus
1	1.00	1	1	0.05	0.05	0.05	0.05	0.051	t	63236	TDSPGG	Oryctolagus cuniculus
1	1.00	1	1	-0.02	-0.018	-0.008	-0.013	-0.02	t	122564	RTAGFDIKLIDT	Homo sapiens
1	1.00	1	1	0	-0.002	0	-0.003	-0.003	t	41870	MKNYLSFGMFALL	Mus musculus
1	1.00	1	1	-0.01	-0.012	0.005	-0.007	-0.01	t	48690	PNPNDP	Mus musculus
1	1.00	1	1	0	0	-0.005	0	-0.002	t	56583	RYLQEKYNLYNSDVFDGKVQ	Oryctolagus cuniculus
1	1.00	1	1	-0.02	-0.018	-0.02	-0.013	-0.018	t	107364	FNWYVDG	Homo sapiens
1	1.00	1	1	0	0	0	0	-0.001	t	151731	SAQSQRAPDR	Mus musculus

1	1.00	1	1	0	0	0	0	-0.001	t	142007	SDWRFLRGYHQYA	Homo sapiens
1	1.00	1	1	0.01	0.01	0.005	0.009	0.01	t	74162	YIAGDPALAYGYAQDQEPDA	Homo sapiens
1	1.00	1	1	0.01	0.008	0.018	0.013	0.01	t	137394	EDHRQVVE	Rattus norvegicus
1	1.00	1	1	0.02	0.019	0.018	0.021	0.02	t	48613	PNDGEERQSYWF	Bos taurus
1	1.00	1	1	0.02	0.022	0.017	0.023	0.022	t	49954	PVSATAMAPSVDPSAEPTAP	Homo sapiens
1	1.00	1	1	0.01	0.012	0.01	0.011	0.01	t	150994	GVTAACPHAGAKSFY	Homo sapiens
1	1.00	1	1	0.01	0.008	0.008	0.013	0.01	t	61686	STKAAKKPEAKREAI	Mus musculus
1	1.00	1	1	0.03	0.026	0.029	0.03	0.031	t	123838	VWQDE	Mus musculus
1	1.00	1	1	0.01	0.01	0.01	0.01	0.01	t	56364	RVMYASSG	Oryctolagus cuniculus
1	1.00	1	1	0	0	-0.01	0.006	0.002	t	50119	PYKELRLEVGKQRLKYAQEE	Homo sapiens
1	1.00	1	1	0.05	0.05	0.041	0.05	0.05	t	139901	SPFGEVPPKLTSEEIAAN	Ovis aries
1	1.00	1	1	-0.01	-0.008	-0.005	-0.009	-0.01	t	67450	TYRGFTSL	Homo sapiens
1	1.00	1	1	0.01	0.01	0.01	0.01	0.01	t	141932	KDKKDDKYTKILAA	Homo sapiens
1	1.00	1	1	0.01	0.01	0.01	0.01	0.01	t	77874	FLPTSPTGLRIE	Mus musculus
1	1.00	1	1	0	0.002	0	0.007	0.003	t	11571	EEEDDDMGFGLFD	Homo sapiens

1	1.00	1	1	0.01	0.01	0.003	0.01	0.008	t	128620	FFQALGITTKISRGT	Homo sapiens
1	1.00	1	1	0	0	-0.005	0	0.002	t	123805	TVGKSSKMLQ	Oryctolagus cuniculus
1	1.00	1	1	0.01	0.01	0.01	0.01	0.008	t	145725	CKQAGTCPP	Mus musculus
1	1.00	1	1	0.01	0.01	0.01	0.01	0.01	t	132928	WGENFIQVPVSE	Homo sapiens
1	1.00	1	1	0.01	0.01	0.01	0.01	0.01	t	107446	IEKTISK	Homo sapiens
1	1.00	1	1	0.01	0.01	0.01	0.01	0.01	t	43237	NANPDPN	Mus musculus
1	1.00	1	1	-0.01	-0.01	-0.01	-0.01	-0.01	t	112196	LAVYFCH	Homo sapiens
1	1.00	1	1	-0.04	-0.04	-0.051	-0.043	-0.04	t	39136	LRLKKYKVPQ	Homo sapiens
1	1.00	1	1	-0.03	-0.027	-0.023	-0.03	-0.031	t	102860	TPYTSLYTW	Mus musculus
1	1.00	1	1	0.03	0.03	0.037	0.03	0.027	t	79040	PGQNLCYTK	Mus musculus
1	1.00	1	1	0.01	0.008	0.01	0.009	0.008	t	123346	KDYYQKWMEE	Homo sapiens
1	1.00	1	1	-0.03	-0.026	-0.024	-0.029	-0.03	t	35062	LCKHSM	Homo sapiens
1	1.00	1	1	0	-0.004	0	-0.001	-0.001	t	71353	VTHESYQELVKKLEALEDA	Oryctolagus cuniculus
1	1.00	1	1	0	0.006	0	-0.001	-0.001	t	43242	NANPNANPNANP	Macaca fascicularis
1	1.00	1	1	0	-0.002	0	-0.001	-0.001	t	7171	CTKPTDGNC	Mus musculus
1	1.00	1	1	0	-0.002	0	-0.001	-0.001	t	7171	CTKPTDGNC	Mus musculus

1	1.00	1	1	0	-0.007	0	0	0	t	112442	ACQKH	Oryctola gus cuniculu s
1	1.00	1	1	-0.04	-0.04	-0.036	-0.034	-0.039	t	138757	AHWSGHC CL	Mus musculu s
1	1.00	1	1	0.01	0.01	0.014	0.014	0.009	t	134001	AIQHESGIL GPLLYGEV GDT	Homo sapiens
1	1.00	1	1	0	0.002	0	0.001	0	t	75923	YSWKTWG	Homo sapiens
1	1.00	1	1	0	0.002	0	0.001	0	t	39255	LRYSWKT WG	Homo sapiens
1	1.00	1	1	0.01	0.014	0.012	0.011	0.007	t	17998	FTKWTWE TKGDL	Homo sapiens
1	1.00	1	1	0.01	0.01	0.01	0.01	0.011	t	138255	TTLTLRH	Homo sapiens
1	1.00	1	1	0.01	0.01	0.015	0.01	0.011	t	5232	ATYTTGGA QS	Mus musculu s
1	1.00	1	1	0.02	0.02	0.018	0.02	0.018	t	95356	ENGDVTFT EVGY	Homo sapiens
1	1.00	1	1	0	0.002	0.009	-0.003	-0.002	t	115349	LKGYGGVS LP	Homo sapiens
1	1.00	1	1	0	0	0	0	0	t	153860	NLQKRMQ QLENDLD Q	Homo sapiens
1	1.00	1	1	0	0	0	0	0	t	6995	CSFSSIPN	Oryctola gus cuniculu s
1	1.00	1	1	0.05	0.05	0.059	0.052	0.05	t	136004	TTPEEIAQ VATI	Homo sapiens
1	1.00	1	1	0.01	0.013	0.007	0.01	0.011	t	31580	KKIQDCYV ENGLIS	Mus musculu s
1	1.00	1	1	-0.03	-0.032	-0.036	-0.031	-0.03	t	82816	GNVTPRVS YAHG	Mus musculu s

1	1.00	1	1	-0.03	-0.03	-0.036	-0.031	-0.03	t	82816	GNVTPRVSYAHG	Homo sapiens
1	1.00	1	1	-0.01	-0.01	-0.013	-0.01	-0.011	t	49950	PVRVAWSSPVQNCEPWIPP	Mus musculus
1	1.00	1	1	0.05	0.052	0.05	0.053	0.05	t	134015	CDKNTGDYYEDSYED	Homo sapiens
1	1.00	1	1	-0.02	-0.022	-0.025	-0.021	-0.02	t	78752	NKGKWKEHIKEVTEKLKKA	Mus musculus
1	1.00	1	1	-0.02	-0.024	-0.025	-0.021	-0.02	t	78752	NKGKWKEHIKEVTEKLKKA	Oryctolagus cuniculus
1	1.00	1	1	0.01	0.01	0.01	0.007	0.01	t	10636	DVKFPGGGQI	Homo sapiens
1	1.00	1	1	-0.01	-0.006	-0.01	-0.007	-0.011	t	110766	AIKSEVSLTGAL	Homo sapiens
1	1.00	1	1	0.03	0.03	0.03	0.03	0.031	t	108360	FEDMAKSDKARYDRE	Homo sapiens
1	1.00	1	1	0.02	0.02	0.02	0.02	0.02	t	112769	EPLYGPY	Homo sapiens
1	1.00	1	1	0	0	0	0	0	t	111725	RHGEEYDLQFIFQLC	Mus musculus
1	1.00	1	1	0	0	0	0	0	t	115474	RVYVEELKPTPEGDLEILLQ	Homo sapiens
1	1.00	1	1	0	0	-0.004	-0.006	0	t	76352	YVLMVPKQ	Homo sapiens
1	1.00	1	1	0	0	-0.012	0	0	t	97605	RIVRGPANLRNLKEFKE	Oryctolagus cuniculus
1	1.00	1	1	0.03	0.028	0.037	0.029	0.03	t	20204	GIADLNDAS	Rattus norvegicus

1	1.00	1	1	0	0.004	0.006	0.001	0	t	86748	NHIENDPLKI	Homo sapiens
1	1.00	1	1	0.01	0.012	0.008	0.011	0.01	t	4132	ARDVLAVVSK	Homo sapiens
1	1.00	1	1	0.03	0.024	0.03	0.03	0.027	t	123482	MALLHQIQGNELEPL	Oryctolagus cuniculus
1	1.00	1	1	0	0	0	0.007	0	t	67730	VARALAHGVRVLEDGVNFAT	Homo sapiens
1	1.00	1	1	-0.02	-0.018	-0.011	-0.013	-0.02	t	38582	LPLPLLQSWM	Homo sapiens
1	1.00	1	1	0.01	0.015	0.015	0.01	0.01	t	4754	ASRQAEVIA	Homo sapiens
1	1.00	1	1	0	0	0	0	-0.001	t	58405	SHRLLQTYWSSA	Homo sapiens
1	1.00	1	1	-0.01	-0.01	-0.01	-0.008	-0.01	t	3290	ANGAGNQPGANGAGNQPG	Homo sapiens
1	1.00	1	1	-0.03	-0.032	-0.028	-0.031	-0.029	t	16123	FHLPYNHNWFAL	Mus musculus
1	1.00	1	1	0	-0.003	-0.001	0	0	t	35628	LENLMWKQI	Oryctolagus cuniculus
1	1.00	1	1	0	0	-0.005	0	-0.002	t	32808	KPNLSSKRSELSQLSMYRVF	Mus musculus
1	1.00	1	1	-0.01	-0.014	-0.015	-0.011	-0.01	t	97430	KRLRLALAS	Oryctolagus cuniculus
1	1.00	1	1	-0.04	-0.039	-0.041	-0.037	-0.041	t	99380	KIADDWDNRHPLPNA	Equus caballus
1	1.00	1	1	-0.06	-0.059	-0.061	-0.057	-0.061	t	99594	QTYRQWIIEAMSEGI	Equus caballus

1	1.00	1	1	0	0	0	0	-0.001	t	150858	ISSLYNLVKSS	Mus musculus
1	1.00	1	1	0	0	0	0	-0.001	t	142913	MVLNEGTSGTA	Oryctolagus cuniculus
1	1.00	1	1	0	0	0	0	-0.001	t	150883	SDDEDLATS	Mus musculus
1	1.00	1	1	0	0	0	0	-0.001	t	102004	QPD	Mus musculus
1	1.00	1	1	0	0	0	0	-0.001	t	108482	IINPSENGDTSTNGIKK	Mus musculus
1	1.00	1	1	0	0	0	0	-0.001	t	63038	TAVSPTTLRTEVVK	Sus scrofa
1	1.00	1	1	0	0	0	0	-0.001	t	97771	WYGYHHSN	Mus musculus
1	1.00	1	1	0	0	0	0	-0.001	t	63122	TDAYNQKLSERRAN	Mus musculus
1	1.00	1	1	0	0	0	0	-0.001	t	43317	NATAEGRAINRRVE	Mus musculus
1	1.00	1	1	0	0	0	0	-0.001	t	59229	SLKMADPNRFRGKDLP	Mus musculus
1	1.00	1	1	0	-0.003	0	0	-0.001	t	59229	SLKMADPNRFRGKDLP	Oryctolagus cuniculus
1	1.00	1	1	0	-0.003	0	0	-0.001	t	53894	RGKDLPVLDQLTD	Oryctolagus cuniculus
1	1.00	1	1	0	0	0	0	-0.001	t	59229	SLKMADPNRFRGKDLP	Mus musculus
1	1.00	1	1	0	0	0	0	-0.001	t	21059	GLPDSVKIWTSETNK	Mus musculus
1	1.00	1	1	0	0.004	0	0	-0.001	t	22015	GREGYPADSKGCKIT	Ovis aries

1	1.00	1	1	0	0	0	0	-0.001	t	11463	EDVNGIRKPK	Mus musculus
1	1.00	1	1	0	0	0	0	-0.001	t	11575	EEEEEEDGFMGFGMFD	Mus musculus
1	1.00	1	1	0	0	0	0	-0.001	t	22015	GREGYPADSKGCKIT	Mus musculus
1	1.00	1	1	0	-0.003	0	0	-0.001	t	21059	GLPDSVKIWTSETNK	Oryctolagus cuniculus
1	1.00	1	1	0	0	0	0	-0.001	t	7674	DAVTELDKN	Mus musculus
1	1.00	1	1	0.01	0.008	0.015	0.009	0.01	t	63885	TGLPSSDRAFLVSVS	Mus musculus
1	1.00	1	1	0.01	0.01	0.01	0.01	0.01	t	23192	GVTYEIDLTNKNAAK	Homo sapiens
1	1.00	1	1	-0.02	-0.02	-0.02	-0.02	-0.021	t	112323	SLTTYGI	Homo sapiens
1	1.00	1	1	0.03	0.04	0.03	0.027	0.03	t	66044	TRKTSERSQP	Pan troglodytes
1	1.00	1	1	-0.06	-0.06	-0.06	-0.06	-0.064	t	138227	QKRAA	Mus musculus
1	1.00	1	1	0.01	0.01	0.01	0.01	0.01	t	67933	VDENANAN	Oryctolagus cuniculus
1	1.00	1	1	0	-0.003	-0.001	0	-0.001	t	50218	QAASAALGALGRFDE	Oryctolagus cuniculus
1	1.00	1	1	-0.02	-0.023	-0.02	-0.02	-0.022	t	133467	YYTYFGRNAYNETKLK	Oryctolagus cuniculus
1	1.00	1	1	0.02	0.02	0.021	0.02	0.019	t	49998	PVVEVNGVTIQVGSR	Mus musculus
1	1.00	1	1	0.01	0.01	0.01	0.01	0.01	t	78225	PFSNLDFM	Homo sapiens

1	1.00	1	1	-0.01	-0.01	-0.01	-0.01	-0.01	t	158873	KAFKVIDQDASGFIE	Homo sapiens
1	1.00	1	1	-0.02	-0.018	-0.001	-0.019	-0.023	t	57723	SFAIKWEYVL	Homo sapiens
1	1.00	1	1	0.01	0.01	0.006	0.01	0.013	t	6093	CDCDRTAA	Mus musculus
1	1.00	1	1	0.01	0.01	0.006	0.01	0.013	t	6093	CDCDRTAA	Mus musculus
1	1.00	1	1	0.01	0.007	0.006	0.01	0.013	t	6093	CDCDRTAA	Mus musculus
1	1.00	1	1	0	-0.001	0.006	0	0	t	18953	GDDPLLGN	Anas platyrhynchos
1	1.00	1	1	0.01	0.012	0.01	0.01	0.01	t	117747	AMKISFAKK	Homo sapiens
1	1.00	1	1	-0.03	-0.03	-0.029	-0.033	-0.031	t	63082	TCKKNM	Homo sapiens
1	1.00	1	1	0.02	0.018	0.013	0.02	0.02	t	38422	LPAYQDYTARAQVSE	Oryctolagus cuniculus
1	1.00	1	1	0	-0.004	-0.007	-0.001	0	t	2242	AKLTLVLTKCGSQIL	Oryctolagus cuniculus
1	1.00	1	1	-0.03	-0.03	-0.032	-0.03	-0.03	t	119562	DYTGKIMWTPPAIFKS	Mus musculus
1	1.00	1	1	-0.03	-0.033	-0.032	-0.03	-0.03	t	119562	DYTGKIMWTPPAIFKS	Oryctolagus cuniculus
1	1.00	1	1	-0.03	-0.03	-0.032	-0.03	-0.03	t	119562	DYTGKIMWTPPAIFKS	Mus musculus
1	1.00	1	1	-0.03	-0.03	-0.032	-0.03	-0.03	t	119562	DYTGKIMWTPPAIFKS	Mus musculus
1	1.00	1	1	0	0	0	0	0	t	117745	ALLRNLGKMTA	Homo sapiens

1	1.00	1	1	-0.04	-0.04	-0.033	-0.04	-0.04	t	60758	SRPIIHFGS	Mus musculus
1	1.00	1	1	-0.02	-0.02	-0.02	-0.02	-0.02	t	73594	YDTRCF	Homo sapiens
1	1.00	1	1	0.02	0.02	0.019	0.017	0.02	t	79672	VNLEDPASRDL	Homo sapiens
1	1.00	1	1	0.05	0.048	0.045	0.043	0.05	t	132269	EPVDGVVLVDPE	Mus musculus
1	1.00	1	1	-0.02	-0.018	-0.02	-0.019	-0.02	t	116954	DPTDYSTRPHMW	Homo sapiens
1	1.00	1	1	-0.02	-0.02	-0.027	-0.017	-0.02	t	125553	RRPEMGDQAHMPYTTAVIHE	Homo sapiens
1	1.00	1	1	0	0	0	0	0	t	106256	ANYGYTFGSGTRLTVV	Oryctolagus cuniculus
1	1.00	1	1	0	0	0	0	0	t	54430	RKTKRNT	Homo sapiens
1	1.00	1	1	0	0	0	0	0	t	48596	PNANP	Mus musculus
1	1.00	1	1	0	0	-0.003	0	0	t	50058	PWYVWLGFIAGLIAIVM	Homo sapiens
1	1.00	1	1	0	0	-0.003	0	0	t	50058	PWYVWLGFIAGLIAIVM	Homo sapiens
1	1.00	1	1	-0.04	-0.04	-0.04	-0.04	-0.04	t	156943	QFTPDKHMLMLP	Gallus gallus
1	1.00	1	1	0.01	0.01	0.01	0.01	0.009	t	108215	WVAMHPQGDL	Homo sapiens
1	1.00	1	1	-0.03	-0.026	-0.032	-0.023	-0.026	t	116292	DPVVHFFKNIVTP	Homo sapiens
1	1.00	1	1	-0.01	-0.013	-0.017	-0.01	-0.01	t	108164	TLVCLATGFFPDHVEL	Oryctolagus cuniculus
1	1.00	1	1	0.03	0.026	0.023	0.033	0.032	t	106811	SALTQPASVSGSPGQSIT	Oryctolagus cuniculus

1	1.00	1	1	0.03	0.027	0.026	0.03	0.028	t	7558	DAKDGKEIDTKHLPSTC	Oryctolagus cuniculus
1	1.00	1	1	0	-0.002	0	0.001	0	t	73554	YDKPYYMLNLYDPNKYVDV	Mus musculus
1	1.00	1	1	0	-0.002	0	0.001	0	t	45824	NSGILKDFWGDYLQYDKPY	Mus musculus
1	1.00	1	1	0	-0.002	0	0.001	0	t	45824	NSGILKDFWGDYLQYDKPY	Mus musculus
1	1.00	1	1	0	-0.002	0	0.001	0	t	31365	KINIGSKVNFDPIDKNQIQ	Mus musculus
1	1.00	1	1	0	0	0	0	0.001	t	115357	LLQKWENGEC	Homo sapiens
1	1.00	1	1	0.02	0.02	0.017	0.026	0.022	t	33493	KSREQLSKLHGVDTS	Homo sapiens
1	1.00	1	1	0.01	0.01	0.003	0.01	0.011	t	141534	DRMNLQTLRGYY	Mus musculus
1	1.00	1	1	0.05	0.05	0.047	0.05	0.05	t	35356	LEALADQTDALQSEEAAVVK	Mus musculus
1	1.00	1	1	0.02	0.02	0.02	0.02	0.02	t	39392	LSGRPAIIPDREALYQEFDE	Homo sapiens
1	1.00	1	1	0	0	0	0	0	t	135950	LLCLLLLSSRPG	Homo sapiens
1	1.00	1	1	0	0	0	0	0	t	121843	SGLGLFPYSSFIWSC	Mus musculus
1	1.00	1	1	0	0	0	0	0	t	121843	SGLGLFPYSSFIWSC	Mus musculus
1	1.00	1	1	0	0	0	0	0	t	7238	CTTSGFASLFRLGPSQK	Mus musculus

1	1.00	1	1	0.01	0.014	0.017	0.017	0.011	t	108126	SIFFAVVLE L	Homo sapiens
1	1.00	1	1	-0.02	-0.016	-0.025	-0.02	-0.02	t	15868	FFPDHQLD PAFRANT	Mus musculu s
1	1.00	1	1	0.01	0.01	0.008	0.006	0.01	t	139570	QEWGTPG S	Homo sapiens
1	1.00	1	1	0.01	0.009	0.01	0.01	0.01	t	38428	LPDDFFPKI DDLVR	Anas platyrhy nchos
1	1.00	1	1	0.06	0.06	0.06	0.056	0.06	t	117661	TVTSAHPE DSSFYICS	Homo sapiens
1	1.00	1	1	0.02	0.023	0.02	0.02	0.02	t	135441	AGLGAGIP	Mus musculu s
1	1.00	1	1	0	-0.002	-0.012	-0.001	0	t	14280	ESSVDRRP METVSKD S	Mus musculu s
1	1.00	1	1	0.01	0.007	0.009	0.01	0.009	t	45667	NRDQGRK PTPPTPP	Oryctola gus cuniculu s
1	1.00	1	1	0.01	0.01	0.006	0.016	0.012	t	55670	RRPFFHPV GEADYFEY HQEG	Homo sapiens
1	1.00	1	1	-0.03	-0.032	-0.037	-0.027	-0.029	t	106947	VATWLNP DPSQK	Mus musculu s
1	1.00	1	1	0	0	0	0	-0.001	t	106374	EDHAGTY GLGDRK	Mus musculu s
1	1.00	1	1	-0.02	-0.022	-0.02	-0.017	-0.021	t	52121	QQQPFPP QQP	Mus musculu s
1	1.00	1	1	-0.02	-0.017	-0.013	-0.02	-0.02	t	106876	STLFRHD	Mus musculu s
1	1.00	1	1	0.01	0.01	0.01	0.01	0.01	t	23190	GVTYEIDL TNKNA	Homo sapiens

1	1.00	1	1	-0.01	-0.01	-0.01	-0.01	-0.01	t	142381	AAEFATEVKKAT	Homo sapiens
1	1.00	1	1	-0.01	-0.012	-0.007	-0.011	-0.008	t	61636	STGPCRTCMT	Mus musculus
1	1.00	1	1	-0.01	-0.014	-0.007	-0.007	-0.012	t	9819	DPYSPSQDPDRRDPY	Oryctolagus cuniculus
1	1.00	1	1	0	0	0	0	0	t	24651	HQTLQDPRVRG	Mus musculus
1	1.00	1	1	0.01	0.007	0.01	0.01	0.012	t	140916	VSYGEEKPAVLG	Oryctolagus cuniculus
1	1.00	1	1	0.02	0.024	0.022	0.027	0.023	t	120168	RGWKHSVTYSGGPDTPY	Homo sapiens
1	1.00	1	1	-0.05	-0.052	-0.051	-0.053	-0.052	t	139725	EMRTRIRYNRRS	Mus musculus
1	1.00	1	1	-0.03	-0.03	-0.03	-0.03	-0.03	t	156936	PMPRFF	Gallus gallus
1	1.00	1	1	0.02	0.008	0.02	0.02	0.019	t	61391	SSTISSCT	Macaca mulatta
1	1.00	1	1	0.03	0.03	0.03	0.03	0.03	t	149113	STPNVTQ	Sus scrofa
1	1.00	1	1	0.03	0.03	0.03	0.03	0.03	t	93510	NSLDKLISSV	Ovis aries
1	1.00	1	1	-0.01	-0.01	-0.025	-0.01	-0.01	t	102810	SLNGKIRAVNVPKVTKEKPT	Mus musculus
1	1.00	1	1	0.01	0.01	0.01	0.01	0.01	t	138892	REQAPNLVY	Mus musculus
1	1.00	1	1	0.01	0.01	0.01	0.01	0.01	t	119625	PENNYKTT	Homo sapiens
1	1.00	1	1	0.01	0.01	0.01	0.01	0.01	t	132035	QSLTPSPGWQSLGSS	Homo sapiens

1	1.00	1	1	-0.01	-0.01	-0.01	-0.01	-0.01	t	135833	CDGWML GECILV	Mus musculus
1	1.00	1	1	-0.01	-0.01	-0.01	-0.01	-0.01	t	104747	DCGDSFN PLF	Homo sapiens
1	1.00	1	1	-0.01	-0.01	-0.01	-0.01	-0.01	t	105280	LDVTT	Oryctolagus cuniculus
1	1.00	1	1	-0.03	-0.03	-0.03	-0.03	-0.03	t	115189	AWYYVPL GTQYTDA PSFSDI	Homo sapiens
1	1.00	1	1	-0.03	-0.03	-0.03	-0.03	-0.03	t	115874	NQFLPYPY YA	Homo sapiens
1	1.00	1	1	-0.05	-0.05	-0.05	-0.05	-0.05	t	99008	SMPVNTP GQFEDFFP	Homo sapiens
1	1.00	1	1	-0.01	-0.014	-0.008	-0.013	-0.012	t	78836	SPKRIGTG DR	Oryctolagus cuniculus
1	1.00	1	1	0	0	-0.011	0	0	t	35982	LFSLMPDV AHSLA	Homo sapiens
1	1.00	1	1	-0.04	-0.04	-0.048	-0.04	-0.038	t	20639	GKMVQVV YDY	Oryctolagus cuniculus
1	1.00	1	1	-0.01	-0.008	-0.023	-0.007	-0.008	t	8356	DGAVQPD GGQPAVR NERA	Cavia porcellus
1	1.00	1	1	-0.01	-0.014	-0.023	-0.007	-0.008	t	8356	DGAVQPD GGQPAVR NERA	Oryctolagus cuniculus
1	1.00	1	1	-0.02	-0.02	-0.02	-0.02	-0.02	t	102005	QPDQHHQ	Mus musculus
1	1.00	1	1	0	0	0	-0.003	-0.002	t	71284	VTEEI	Homo sapiens
1	1.00	1	1	0.01	0.01	0.015	0.01	0.01	t	20657	GKPALVPD KE	Homo sapiens

1	1.00	1	1	0	0.004	0	0.001	0	t	42932	MVDVVTGA	Homo sapiens
1	1.00	1	1	0.01	0.013	0.022	0.01	0.01	t	79052	QLDPAF	Mus musculus
1	1.00	1	1	-0.02	-0.02	-0.02	-0.02	-0.02	t	79475	YDWLMF	Mus musculus
1	1.00	1	1	0.01	0.012	0.006	0.007	0.008	t	6223	CFEIKCTKPEACSGEPVVV	Homo sapiens
1	1.00	1	1	-0.03	-0.033	-0.041	-0.03	-0.029	t	6541	CLAVLLGACS	Oryctolagus cuniculus
1	1.00	1	1	0.01	0.008	0.007	0.009	0.01	t	28290	IRFKDDSSYEEVN	Mus musculus
1	1.00	1	1	0.01	0.01	0.003	0.01	0.011	t	102696	NTKWDIYGY	Mus musculus
1	1.00	1	1	0.01	0.01	0.012	0.01	0.011	t	153254	ERHTKFPSV	Mus musculus
1	1.00	1	1	0	-0.002	0	-0.001	0	t	56686	RYYRE	Mus musculus
1	1.00	1	1	0	0.002	0	0.001	0	t	68664	VGGPLTNSKGQNCGYRRCR	Homo sapiens
1	1.00	1	1	0.02	0.024	-0.001	0.017	0.022	t	153819	DTLEQQNKEANNRAEKSE	Homo sapiens
1	1.00	1	1	-0.03	-0.026	-0.03	-0.033	-0.032	t	106311	CLPFVQGKEK	Homo sapiens
1	1.00	1	1	-0.01	-0.01	-0.01	-0.01	-0.011	t	9597	DPAPPNANDPAPPNAN	Mus musculus
1	1.00	1	1	0	0.003	-0.006	0	0	t	98433	RVQKANADAK	Mus musculus

1	1.00	1	1	0	0	-0.006	0	0	t	98433	RVQKANADAK	Mus musculus
1	1.00	1	1	-0.03	-0.028	-0.035	-0.029	-0.028	t	54863	RMAWDMMMNW	Homo sapiens
1	1.00	1	1	0.02	0.017	0.021	0.02	0.018	t	65996	TRAPRREELEWDDEDH	Oryctolagus cuniculus
1	1.00	1	1	-0.02	-0.018	-0.003	-0.013	-0.023	t	113010	GRVSTEVDARLSFDK	Homo sapiens
1	1.00	1	1	0.02	0.018	0.008	0.019	0.02	t	112841	SSKRGRQTPVLYAMLDH	Rattus norvegicus
1	1.00	1	1	-0.01	-0.008	-0.01	-0.007	-0.009	t	918	AEGEFATAPPSHYSWDPAK	Homo sapiens
1	1.00	1	1	-0.08	-0.08	-0.08	-0.08	-0.08	t	115384	MKPWIQPKTKVIPYVRYL	Homo sapiens
1	1.00	1	1	-0.02	-0.02	-0.016	-0.02	-0.022	t	124061	ELERIRRSILPYGD	Homo sapiens
1	1.00	1	1	-0.02	-0.018	-0.02	-0.013	-0.018	t	107533	NWLDGKE	Homo sapiens
1	1.00	1	1	-0.01	-0.008	-0.005	-0.007	-0.009	t	102563	KKRPKPG	Homo sapiens
1	1.00	1	1	0.01	0.008	0.004	0.007	0.01	t	10824	DWSERIREFM	Mus musculus
1	1.00	1	1	0.01	0.012	0.01	0.011	0.011	t	16123	FHLPYNHNWFAL	Homo sapiens
1	1.00	1	1	0.01	0.014	0.01	0.017	0.011	t	122874	PPARALKRSLWVEVDLLIGS	Homo sapiens
1	1.00	1	1	0.02	0.02	0.02	0.02	0.02	t	127619	DEYKRFMNYWTASNE	Homo sapiens
1	1.00	1	1	0.02	0.02	0.02	0.02	0.02	t	133956	WIMSNF	Mus musculus

1	1.00	1	1	0	0	0	0	0	t	135842	NTYGDFMVKSPN	Mus musculus
1	1.00	1	1	0	0	0	0	0	t	150970	AVPADHGAHLSLRGLPVCAF	Mus musculus
1	1.00	1	1	0	0	0	0	0	t	112074	DGAWFAY	Homo sapiens
1	1.00	1	1	0	0	0	0	0	t	115309	KFPQYLQYLYQGPIVLNPWD	Homo sapiens
1	1.00	1	1	-0.02	-0.02	-0.01	-0.02	-0.02	t	45420	NPNDPP	Mus musculus
1	1.00	1	1	-0.02	-0.02	-0.01	-0.02	-0.02	t	45420	NPNDPP	Mus musculus
1	1.00	1	1	0.05	0.05	0.045	0.047	0.05	t	49022	PQLEIVPNSA	Homo sapiens
1	1.00	1	1	-0.02	-0.02	-0.02	-0.02	-0.02	t	115305	KDERFFSDKIAKYIPIQYVL	Homo sapiens
1	1.00	1	1	-0.02	-0.02	-0.02	-0.02	-0.02	t	79507	AHIPWLL	Oryctolagus cuniculus
1	1.00	1	1	-0.02	-0.02	-0.02	-0.02	-0.02	t	17365	FPQPQPFPPQ	Mus musculus
1	1.00	1	1	-0.02	-0.02	-0.02	-0.02	-0.02	t	17365	FPQPQPFPPQ	Mus musculus
1	1.00	1	1	-0.01	-0.01	-0.01	-0.012	-0.01	t	98975	QVEKLIKNQKESHFV	Homo sapiens
1	1.00	1	1	0.01	0.01	0	0.01	0.01	t	50160	PYQPVSNLTATTQGQ	Mus musculus
1	1.00	1	1	-0.03	-0.042	-0.03	-0.027	-0.03	t	156850	APHNKWPHLNPP	Bos taurus
1	1.00	1	1	0.03	0.03	0.032	0.03	0.032	t	52397	QSPHLTYPEPMS	Mus musculus
1	1.00	1	1	-0.04	-0.04	-0.047	-0.04	-0.041	t	68019	VDLPQPGLRR	Mus musculus

1	1.00	1	1	-0.03	-0.033	-0.035	-0.03	-0.029	t	97478	LTAGALWNA	Oryctolagus cuniculus
1	1.00	1	1	-0.01	-0.01	-0.01	-0.01	-0.01	t	132816	FGQNAPR	Homo sapiens
1	1.00	1	1	0.01	0.012	0.003	0.007	0.012	t	78068	VYDYNCHVDL	Homo sapiens
1	1.00	1	1	-0.01	-0.01	-0.022	-0.017	-0.01	t	96453	KFKEAFLRSV	Homo sapiens(human)
1	1.00	1	1	-0.02	-0.017	-0.015	-0.02	-0.022	t	122083	DPNIKLVLDDCWATS	Mus musculus
1	1.00	1	1	0.03	0.028	0.025	0.031	0.03	t	135799	YGKDVKDLFDYAQE	Mus musculus
1	1.00	1	1	0.07	0.072	0.078	0.067	0.071	t	18808	GAVDSI	Homo sapiens
1	1.00	1	1	0.04	0.04	0.042	0.049	0.039	t	102381	EEIAQVATISANGDKDIGNI	Mus musculus
1	1.00	1	1	0.04	0.04	0.042	0.049	0.039	t	102381	EEIAQVATISANGDKDIGNI	Mus musculus
1	1.00	1	1	0.01	0.01	0.01	0.01	0.01	t	43239	NANPNAN	Mus musculus
1	1.00	1	1	0	0	-0.005	0	0	t	113400	FKLGGRDSR	Oryctolagus cuniculus
1	1.00	1	1	0	0	-0.005	0	0	t	113400	FKLGGRDSR	Homo sapiens
1	1.00	1	1	0	0	-0.005	0	0	t	113400	FKLGGRDSR	Homo sapiens
1	1.00	1	1	0.03	0.032	0.024	0.033	0.03	t	10817	DWLEDTLSEGIRQWWKLKPG	Homo sapiens

1	1.00	1	1	0	0	0.007	0	0	t	119452	FRKTSKGGS	Homo sapiens
1	1.00	1	1	0	-0.002	-0.005	-0.001	0	t	53342	RDFSIRES	Mus musculus
1	1.00	1	1	0	-0.004	-0.005	-0.007	-0.002	t	130537	LCENIANHLK	Oryctolagus cuniculus
1	1.00	1	1	0.03	0.03	0.032	0.027	0.03	t	9118	DLIAYKQ	Homo sapiens
1	1.00	1	1	-0.01	-0.01	-0.01	-0.01	-0.01	t	118846	ILKKVLEALKDLI	Oryctolagus cuniculus
1	1.00	1	1	0	0	0	0	-0.001	t	135711	LDETVKDKLAKEQKSKQNI	Homo sapiens
1	1.00	1	1	0	0	0	0	-0.001	t	135837	DMYGDIRLTGLI	Mus musculus
1	1.00	1	1	0	0	0	0	-0.001	t	144678	GTQPTLEVVPMGRGS	Mus musculus
1	1.00	1	1	0	0	0	0	-0.001	t	141693	KTWG	Mus musculus
1	1.00	1	1	0	0	0	0	-0.001	t	144820	YLEDWIKYNNQK	Mus musculus
1	1.00	1	1	0	0	0	0	-0.001	t	153480	TPSWRNPFPTFYGGGS	Mus musculus
1	1.00	1	1	0	0	0	0	-0.001	t	153333	ACLDAKLQQCGGGS	Mus musculus
1	1.00	1	1	0	0	0	0	-0.001	t	153420	MDWINPFPNFNSGGGS	Mus musculus
1	1.00	1	1	0	0	0	0	-0.001	t	150883	SDDEDLATS	Mus musculus
1	1.00	1	1	0	0	0	0	-0.001	t	135660	ECPEGYILDDGFICTDIDE	Mus musculus

1	1.00	1	1	0	0	0	0	-0.001	t	135836	DLFGNVLLGQRLDQG	Mus musculus
1	1.00	1	1	0	0	0	0	-0.001	t	144599	CNGTHPSLRLC	Mus musculus
1	1.00	1	1	0	0	0	0	-0.001	t	138628	SQKEVLERTRAELDTVC	Oryctolagus cuniculus
1	1.00	1	1	0	0	0	0	-0.001	t	143328	DEGDTDLYDYYPEE	Mus musculus
1	1.00	1	1	0	0	0	0	-0.001	t	151101	AAA	Homo sapiens
1	1.00	1	1	0	0	0	0	-0.001	t	144779	SGTHWFLAGCIS	Mus musculus
1	1.00	1	1	0	0	0	0	-0.001	t	136542	PDGSESPTDQET	Mus musculus
1	1.00	1	1	0	0	0	0	-0.001	t	135841	HVTDMMELYTLG	Mus musculus
1	1.00	1	1	0	0	0	0	-0.001	t	136544	PEEYVSPDAEVIG	Mus musculus
1	1.00	1	1	0	0	0	0	-0.001	t	134784	RKRSQMLFRGRRASQ	Mus musculus
1	1.00	1	1	0	0	0	0	-0.001	t	153341	ACQVPHFFGCGGGS	Mus musculus
1	1.00	1	1	0	0	0	0	-0.001	t	138812	SSVLYGGPPSAA	Mus musculus
1	1.00	1	1	0	0	0	0	-0.001	t	141559	SDEALPLGSPR	Mus musculus
1	1.00	1	1	0	0	0	0	-0.001	t	139860	PVQRGAWRWPVVTNE	Mus musculus
1	1.00	1	1	0	0	0	0	-0.001	t	153362	APWSLRDHLAVTGGGS	Mus musculus
1	1.00	1	1	0	0	0	0	-0.001	t	135650	DKVSFFCKNKEKKC	Homo sapiens

1	1.00	1	1	0	0	0	0	-0.001	t	139846	PEWWLEE PPFISSGK	Mus musculus
1	1.00	1	1	0	0	0	0	-0.001	t	137743	DTPLTIAGL KLV	Mus musculus
1	1.00	1	1	0	0	0	0	-0.001	t	146813	DAEFGHD SGFEVRH QK	Mus musculus
1	1.00	1	1	0	0	0	0	-0.001	t	141560	SDEALPPG SPR	Mus musculus
1	1.00	1	1	0	0	0	0	-0.001	t	153357	ACWYSTR DHCGGGS	Mus musculus
1	1.00	1	1	0	0	0	0	-0.001	t	145144	DPGQLRL WRLAS	Homo sapiens
1	1.00	1	1	0	0	0	0	-0.001	t	135191	NTLKTPRV GGC	Homo sapiens
1	1.00	1	1	0	0	0	0	-0.001	t	139602	SDSMLSW	Oryctolagus cuniculus
1	1.00	1	1	0	0	0	0	-0.001	t	135841	HVTDMM ELYTLG	Mus musculus
1	1.00	1	1	0	0	0	0	-0.001	t	137469	YPPEEETG ERV	Oryctolagus cuniculus
1	1.00	1	1	0	0	0	0	-0.001	t	135471	KDKATFGC HDGC	Homo sapiens
1	1.00	1	1	0	0	0	0	-0.001	t	144654	GPHPTLEV VPMGRGS	Mus musculus
1	1.00	1	1	0	0	0	0	-0.001	t	135834	CEFRDGA GSICG	Mus musculus
1	1.00	1	1	0	0	0	0	-0.001	t	134421	KQIPECEN VKTTQCVF	Mus musculus
1	1.00	1	1	0	0	0	0	-0.001	t	134926	ENPVVHFF KNIVTPRT P	Homo sapiens
1	1.00	1	1	0	0	0	0	-0.001	t	145777	FDTGAFDP DWPYD	Mus musculus

1	1.00	1	1	0	0	0	0	-0.001	t	140695	KGRFTISRDNAKSTLYL	Mus musculus
1	1.00	1	1	0	0	0	0	-0.001	t	144809	VLAPGGTHSRGLLEM	Mus musculus
1	1.00	1	1	0	0	0	0	-0.001	t	139305	TAAQITQRKWEAAREAEQRR	Oryctolagus cuniculus
1	1.00	1	1	0	0	0	0	-0.001	t	138232	RLTSSLRYNP	Oryctolagus cuniculus
1	1.00	1	1	0	0	0	0	-0.001	t	134702	KEKKYS	Mus musculus
1	1.00	1	1	0	0	0	0	-0.001	t	137866	GDTRPRFL	Oryctolagus cuniculus
1	1.00	1	1	0	0	0	0	-0.001	t	153334	ACLEILSWLCGGGS	Mus musculus
1	1.00	1	1	0	0	0	0	-0.001	t	153331	ACISPNQWLCGGGS	Mus musculus
1	1.00	1	1	0	0	0	0	-0.001	t	138456	KEQFEFALTAVAEEV	Oryctolagus cuniculus
1	1.00	1	1	0	0	0	0	-0.001	t	137944	PGTEYT	Oryctolagus cuniculus
1	1.00	1	1	0	0	0	0	-0.001	t	153447	QPAIWINPFPAWGGGS	Mus musculus
1	1.00	1	1	0	0	0	0	-0.001	t	153346	ACRDHYPLSCGGGS	Mus musculus
1	1.00	1	1	0	0	0	0	-0.001	t	135842	NTYGDFMVKSPN	Mus musculus
1	1.00	1	1	0	0	0	0	-0.001	t	136061	ETQAE	Mus musculus
1	1.00	1	1	0	0	0	0	-0.001	t	151609	DETPMRGETRKV	Homo sapiens

1	1.00	1	1	0	0	0	0	-0.001	t	135842	NTYGDFMVKSPN	Mus musculus
1	1.00	1	1	0	0	0	0	-0.001	t	145376	NCWKPQVLHC	Homo sapiens
1	1.00	1	1	0	0	0	0	-0.001	t	139225	REAEQRR	Oryctolagus cuniculus
1	1.00	1	1	0	0	0	0	-0.001	t	138198	KPTSTLT	Mus musculus
1	1.00	1	1	0	0	0	0	-0.001	t	138189	HILGSTA	Mus musculus
1	1.00	1	1	0	0	0	0	-0.001	t	135837	DMYGDIRLTGLI	Mus musculus
1	1.00	1	1	0	0	0	0	-0.001	t	135839	DYMGNIIQDFDT	Mus musculus
1	1.00	1	1	0	0	0	0	-0.001	t	144777	SGTHMNLYLHDTAFA	Mus musculus
1	1.00	1	1	0	0	0	0	-0.001	t	146189	DIPEEIKQ	Mus musculus
1	1.00	1	1	0	0	0	0	-0.001	t	144656	GPTHAMFDSSGP	Mus musculus
1	1.00	1	1	0	0	0	0	-0.001	t	142480	VAAVTEKLRAGT	Mus musculus
1	1.00	1	1	0	0	0	0	-0.001	t	138192	HQTPLST	Mus musculus
1	1.00	1	1	0	0	0	0	-0.001	t	139500	FHTHISV	Oryctolagus cuniculus
1	1.00	1	1	0	0	0	0	-0.001	t	134422	KRKRSQMLFRGRRASQ	Mus musculus
1	1.00	1	1	0	0	0	0	-0.001	t	137136	DQQTQIAKDTAQT	Mus musculus

1	1.00	1	1	0	0	0	0	-0.001	t	151713	RGEARK	Homo sapiens
1	1.00	1	1	0	0	0	0	-0.001	t	144786	SMNRGRGTHPSLIWM	Mus musculus
1	1.00	1	1	0	0	0	0	-0.001	t	135642	CKNKEKKC	Homo sapiens
1	1.00	1	1	0	0	0	0	-0.001	t	140459	EHWSHDMFSPGD	Mus musculus
1	1.00	1	1	0	0	0	0	-0.001	t	135545	DVFYPYPYASGS	Mus musculus
1	1.00	1	1	0	0	0	0	-0.001	t	153359	ALLPFKDHLPYPGGGS	Mus musculus
1	1.00	1	1	0	0	0	0	-0.001	t	138283	AFSFEAQGGLA	Oryctolagus cuniculus
1	1.00	1	1	0	0	0	0	-0.001	t	134804	TIFILFCCSKEKRKKKQAAT	Mus musculus
1	1.00	1	1	0	0	0	0	-0.001	t	134767	QDPYVKEAENLKKYFNAG	Mus musculus
1	1.00	1	1	0	0	0	0	-0.001	t	138264	YSNKTPHLQWRL	Homo sapiens
1	1.00	1	1	0	0	0	0	-0.001	t	158947	LDIDIDPKVKVATLS	Mus musculus
1	1.00	1	1	0	0	0	0	-0.001	t	136419	KIHAREIFDSRGNPTVE	Homo sapiens
1	1.00	1	1	0	0	0	0	-0.001	t	144705	KGTHSLYSQIAG	Mus musculus
1	1.00	1	1	0	0	0	0	-0.001	t	136420	KIHAREIFDSRGNPTVE	Homo sapiens
1	1.00	1	1	0	0	0	0	-0.001	t	136418	KIHAREIFDSRGNPTVE	Homo sapiens
1	1.00	1	1	0	0	0	0	-0.001	t	153498	WSPSKLYSARDHGGGS	Mus musculus

1	1.00	1	1	0	0	0	0	-0.001	t	136353	HGNAEVHASFFDIGGS	Oryctolagus cuniculus
1	1.00	1	1	0	0	0	0	-0.001	t	135845	WTEIHDWEAAME	Mus musculus
1	1.00	1	1	0	0	0	0	-0.001	t	140460	EKLKHSM	Mus musculus
1	1.00	1	1	0	0	0	0	-0.001	t	138141	ANKTSPFLMWRL	Homo sapiens
1	1.00	1	1	0	0	0	0	-0.001	t	144673	GTFCGGEECMRG	Mus musculus
1	1.00	1	1	0	0	0	0	-0.001	t	153382	FAENKKDHFSPGGGGS	Mus musculus
1	1.00	1	1	0	0	0	0	-0.001	t	144675	GTHPFLSQW	Mus musculus
1	1.00	1	1	0	0	0	0	-0.001	t	135845	WTEIHDWEAAME	Mus musculus
1	1.00	1	1	0	0	0	0	-0.001	t	150200	FGEHKQEKDLEYGAC	Mus musculus
1	1.00	1	1	0	0	0	0	-0.001	t	138255	TTLTLRH	Homo sapiens
1	1.00	1	1	0	0	0	0	-0.001	t	138238	SMLDTPT	Mus musculus
1	1.00	1	1	0	0	0	0	-0.001	t	144756	QVGTHPGLANDYEYT	Mus musculus
1	1.00	1	1	0	0	0	0	-0.001	t	153434	NSPFMLHSALSGGGS	Mus musculus
1	1.00	1	1	0	0	0	0	-0.001	t	135839	DYMGNIIQDFDT	Mus musculus
1	1.00	1	1	0	0	0	0	-0.001	t	142441	LFSPWLRVQNHF	Mus musculus
1	1.00	1	1	0	0	0	0	-0.001	t	153481	TPTWPLLTIFPSGGGS	Mus musculus

1	1.00	1	1	0	0	0	0	-0.001	t	141764	TLNFNAEGEPELLMLANWR	Mus musculus
1	1.00	1	1	0	0	0	0	-0.001	t	138202	LPTITNT	Mus musculus
1	1.00	1	1	0	0	0	0	-0.001	t	151023	LCKLRGVAPLHLGKC	Mus musculus
1	1.00	1	1	0	0	0	0	-0.001	t	135350	VGVTETPL	Oryctolagus cuniculus
1	1.00	1	1	0	0	0	0	-0.001	t	137962	RDSPEDFV	Oryctolagus cuniculus
1	1.00	1	1	0	0	0	0	-0.001	t	151763	WYRATHQE	Mus musculus
1	1.00	1	1	0	0	0	0	-0.001	t	153396	HSFEPWSARDMLGGGS	Mus musculus
1	1.00	1	1	0	0	0	0	-0.001	t	144623	EEAEGTTDAQIDANRKRESE	Mus musculus
1	1.00	1	1	0	0	0	0	-0.001	t	137432	NHNYPPLSLLTF	Mus musculus
1	1.00	1	1	0	0	0	0	-0.001	t	149867	PESFDGDPASNTAPLQPE	Homo sapiens
1	1.00	1	1	0	0	0	0	-0.001	t	144701	KFGNYNHTQLLGQLQAV	Oryctolagus cuniculus
1	1.00	1	1	0	0	0	0	-0.001	t	135799	YGKDVKDLFDYAQE	Mus musculus
1	1.00	1	1	0	0	0	0	-0.001	t	144598	CFVGTHPQLRC	Mus musculus
1	1.00	1	1	0	0	0	0	-0.001	t	146337	IPEEIKQL	Mus musculus
1	1.00	1	1	0	0	0	0	-0.001	t	135847	YPAIVETGL	Mus musculus
1	1.00	1	1	0	0	0	0	-0.001	t	134492	QIQRRPSM	Mus musculus

1	1.00	1	1	0	0	0	0	-0.001	t	144676	GTHPSLQFT	Mus musculus
1	1.00	1	1	0	0	0	0	-0.001	t	135930	GDKVSFFCKNKEKKC	Homo sapiens
1	1.00	1	1	0	0	0	0	-0.001	t	135833	CDGWMLGECILV	Mus musculus
1	1.00	1	1	0	0	0	0	-0.001	t	142561	CKVMVHDPHSLA	Mus musculus
1	1.00	1	1	0	0	0	0	-0.001	t	141683	KDWG	Mus musculus
1	1.00	1	1	0	0	0	0	-0.001	t	149867	PESFDGDPASNTAPLQPE	Mus musculus
1	1.00	1	1	0	0	0	0	-0.001	t	141105	WEISQASHYFERHLE	Oryctolagus cuniculus
1	1.00	1	1	0	0	0	0	-0.001	t	138608	RVGAHDP	Homo sapiens
1	1.00	1	1	0	0	0	0	-0.001	t	149684	EPAGSNR	Mus musculus
1	1.00	1	1	0	0	0	0	-0.001	t	135449	CATLRVYKGG	Homo sapiens
1	1.00	1	1	0	0	0	0	-0.001	t	119025	SWTDTDGTN	Mus musculus
1	1.00	1	1	0	0	0	0	-0.001	t	121112	AACDPRHGRYLTVA	Homo sapiens
1	1.00	1	1	0	0	0	0	-0.001	t	131406	EGEGSGFIREDPIGAGA	Oryctolagus cuniculus
1	1.00	1	1	0	0	0	0	-0.001	t	125209	QFQIHGPR	Mus musculus
1	1.00	1	1	0	0	0	0	-0.001	t	122917	TKVYSTDIFFILEITDNPY	Mus musculus
1	1.00	1	1	0	0	0	0	-0.001	t	122375	GLINRAKAVKQFEESQG	Mus musculus

1	1.00	1	1	0	0	0	0	-0.001	t	118810	GHGKGVG FGEGVGS GK	Mus musculu s
1	1.00	1	1	0	0	0	0	-0.001	t	130925	KLLPEDDE SRTYHTV	Homo sapiens
1	1.00	1	1	0	0	0	0	-0.001	t	122895	RWLPPVY EDGFS	Oryctola gus cuniculu s
1	1.00	1	1	0	0	0	0	-0.001	t	133067	EELSELREE	Mus musculu s
1	1.00	1	1	0	0	0	0	-0.001	t	131576	NSRQTDR EDELI	Rattus norvegic us
1	1.00	1	1	0	0	0	0	-0.001	t	132727	VTNEVHT WTEQYKF Q	Mus musculu s
1	1.00	1	1	0	0	0	0	-0.001	t	119823	EDDDMGF GLFD	Homo sapiens
1	1.00	1	1	0	0	0	0	-0.001	t	120769	LLVDVEPK VKSKKRE	Mus musculu s
1	1.00	1	1	0	0	0	0	-0.001	t	131101	TDSEMDV VRFDDDN S	Mus musculu s
1	1.00	1	1	0	0	0	0	-0.001	t	122551	RGQYLQL QELECDW EEVTTS	Homo sapiens
1	1.00	1	1	0	0	0	0	-0.001	t	133622	KPGDPSAL HVVRCWI C	Mus musculu s
1	1.00	1	1	0	0	0	0	-0.001	t	125015	DNESSYSR GFDMTYT E	Mus musculu s
1	1.00	1	1	0	0	0	0	-0.001	t	122399	HPQPEDP DTAKE	Mus musculu s
1	1.00	1	1	0	0	0	0	-0.001	t	123193	FQIHGPR	Mus musculu s
1	1.00	1	1	0	0	0	0	-0.001	t	132754	YER	Mus musculu s
1	1.00	1	1	0	0	0	0	-0.001	t	122878	PYEFSRKV PTFATPW PDFVP	Oryctola gus cuniculu s

1	1.00	1	1	0	0	0	0	-0.001	t	124925	YYVFFEEQEDEIIGF	Oryctolagus cuniculus
1	1.00	1	1	0	0	0	0	-0.001	t	123193	FQIHGPR	Mus musculus
1	1.00	1	1	0	0	0	0	-0.001	t	118762	CYGSFAKPTN	Mus musculus
1	1.00	1	1	0	-0.003	0	0	-0.001	t	118877	LDVEQLGIPEQEYSC	Oryctolagus cuniculus
1	1.00	1	1	0	0	0	0	-0.001	t	132890	QSPWQTWFTRAL	Homo sapiens
1	1.00	1	1	0	0	0	0	-0.001	t	120699	KMFPEVKEKGMAALPRL	Oryctolagus cuniculus
1	1.00	1	1	0	0	0	0	-0.001	t	122875	PRGWNPGFLYNGFPL	Oryctolagus cuniculus
1	1.00	1	1	0	0	0	0	-0.001	t	118877	LDVEQLGIPEQEYSC	Oryctolagus cuniculus
1	1.00	1	1	0	0	0	0	-0.001	t	118383	GLKKMLRTCAVHITLD	Homo sapiens
1	1.00	1	1	0	0	0	0	-0.001	t	125415	FSPDGKPCTPPALNCYW	Mus musculus
1	1.00	1	1	0	0	0	0	-0.001	t	125230	RERRGIAL	Mus musculus
1	1.00	1	1	0	0	0	0	-0.001	t	130805	ELNPEPDTEVFPMTF	Homo sapiens
1	1.00	1	1	0	0	0	0	-0.001	t	130524	KFHPKDIPYQVW	Mus musculus
1	1.00	1	1	0	0	0	0	-0.001	t	118036	NIVTPRTPPP	Rattus norvegicus

1	1.00	1	1	0	0	0	0	-0.001	t	122915	THKANPSPEPNMHEC	Oryctolagus cuniculus
1	1.00	1	1	0	0	0	0	-0.001	t	130987	NPVENMMDRDSQ	Homo sapiens
1	1.00	1	1	0	0	0	0	-0.001	t	121154	ASAARLTMMW	Homo sapiens
1	1.00	1	1	0	0	0	0	-0.001	t	132948	YNCEPANPSEKNSPSTQYCY	Mus musculus
1	1.00	1	1	0	0	0	0	-0.001	t	132200	CGNRGPSLRTTTVTG	Mus musculus
1	1.00	1	1	0	0	0	0	-0.001	t	119673	WNPDDYGGVK	Rattus norvegicus
1	1.00	1	1	0	0	0	0	-0.001	t	118581	RDRDRDRE	Mus musculus
1	1.00	1	1	0	0	0	0	-0.001	t	131658	SGLRLLEKSQH	Oryctolagus cuniculus
1	1.00	1	1	0	0	0	0	-0.001	t	133623	KPGEMR	Mus musculus
1	1.00	1	1	0	0	0	0	-0.001	t	125021	DWC	Mus musculus
1	1.00	1	1	0	0	0	0	-0.001	t	122749	EIIGFGQELKNPQEE	Oryctolagus cuniculus
1	1.00	1	1	0	0	0	0	-0.001	t	122889	RLDASFQEHPDLPG	Oryctolagus cuniculus
1	1.00	1	1	0	0	0	0	-0.001	t	117400	VSKDDSIISSLDVTD	Homo sapiens
1	1.00	1	1	0	0	0	0	-0.001	t	130787	EDFDIYDEDE	Mus musculus
1	1.00	1	1	0	0	0	0	-0.001	t	125384	DSHYDYTICGDSEDMVCTPK	Oryctolagus cuniculus

1	1.00	1	1	0	0	0	0	-0.001	t	133536	CKPVENRAC	Mus musculus
1	1.00	1	1	0	0	0	0	-0.001	t	132843	ITPWPHWLERSS	Mus musculus
1	1.00	1	1	0	0	0	0	-0.001	t	118105	RKGHFLLSSKSGFWTIWL	Homo sapiens
1	1.00	1	1	0	-0.003	0	0	-0.001	t	120347	YRGWKHWVYYTCCPDTPYLD	Oryctolagus cuniculus
1	1.00	1	1	0	0	0	0	-0.001	t	120347	YRGWKHWVYYTCCPDTPYLD	Mus musculus
1	1.00	1	1	0	0	0	0	-0.001	t	122328	EEYHLYKDGHLPISEFALLV	Homo sapiens
1	1.00	1	1	0	0	0	0	-0.001	t	118019	MGRGAPPPGMMG	Homo sapiens
1	1.00	1	1	0	0	0	0	-0.001	t	130808	ENEPPVWTTESKLQS	Homo sapiens
1	1.00	1	1	0	0	0	0	-0.001	t	120347	YRGWKHWVYYTCCPDTPYLD	Mus musculus
1	1.00	1	1	0	0	0	0	-0.001	t	122861	NALNSPLHQEYEENL	Oryctolagus cuniculus
1	1.00	1	1	0	0	0	0	-0.001	t	122327	EEVTTSGPCGEESVLLDRKS	Homo sapiens
1	1.00	1	1	0	0	0	0	-0.001	t	131075	SQSYPEPARGSVPMP	Homo sapiens
1	1.00	1	1	0	0	0	0	-0.001	t	122508	PCGEESVLLDRKSGKQYSIE	Homo sapiens
1	1.00	1	1	0	0	0	0	-0.001	t	119187	GNAEVHASFFDIGG	Oryctolagus cuniculus
1	1.00	1	1	0	0	0	0	-0.001	t	122775	GIFNTGLKMFPDLTKVYST	Mus musculus

1	1.00	1	1	0	0	0	0	-0.001	t	120109	NIHPKAPIWGIITSNF	Rattus norvegicus
1	1.00	1	1	0	0	0	0	-0.001	t	125375	DHTLFAASHNHR	Sus scrofa
1	1.00	1	1	0	0	0	0	-0.001	t	125375	DHTLFAASHNHR	Mus musculus
1	1.00	1	1	0	0	0	0	-0.001	t	123734	SFSKASNSWFPVEGPADICQ	marmosets
1	1.00	1	1	0	0	0	0	-0.001	t	118763	CYGSFARPMN	Mus musculus
1	1.00	1	1	0	0	0	0	-0.001	t	122915	THKANPSPEPNMHEC	Oryctolagus cuniculus
1	1.00	1	1	0	0	0	0	-0.001	t	129301	LQELEKDEREQ	Homo sapiens
1	1.00	1	1	0	0	0	0	-0.001	t	119499	MTFSVPIS	Mus musculus
1	1.00	1	1	0	0	0	0	-0.001	t	118346	FEYFPSHFFVPELEVTIIC	Mus musculus
1	1.00	1	1	0	0	0	0	-0.001	t	131701	THHINNMKPSFTRENT	Oryctolagus cuniculus
1	1.00	1	1	0	0	0	0	-0.001	t	131579	PDIITGFSS	Oryctolagus cuniculus
1	1.00	1	1	0	0	0	0	-0.001	t	125229	RDSEGDLVAEKLGPI	Homo sapiens
1	1.00	1	1	0	0	0	0	-0.001	t	125441	HPLTCDDPRFQ	Oryctolagus cuniculus
1	1.00	1	1	0	0	0	0	-0.001	t	118151	SRFSWGAE	Rattus norvegicus
1	1.00	1	1	0	0	0	0	-0.001	t	133542	DGRRDVVVRSATFYL	Mus musculus

1	1.00	1	1	0	0	0	0	-0.001	t	125236	RIEKDTDRDNFMSAEEAQ	Homo sapiens
1	1.00	1	1	0	0	0	0	-0.001	t	133580	GFLSSCNFRFCELVR	Mus musculus
1	1.00	1	1	0	0	0	0	-0.001	t	117598	PEPAKSAPAPKKG	Mus musculus
1	1.00	1	1	0	0	0	0	-0.001	t	122430	KGLRRDLDASREAK	Mus musculus
1	1.00	1	1	0	0	0	0	-0.001	t	118060	PPPGMRPP	Mus musculus
1	1.00	1	1	0	0	0	0	-0.001	t	120924	RPEPKKPWSGVWNASTY	Mus musculus
1	1.00	1	1	0	0	0	0	-0.001	t	130492	FKEQFLDGDGWTSR	Oryctolagus cuniculus
1	1.00	1	1	0	0	0	0	-0.001	t	131657	SGLQAEDEADYYCSSY	Homo sapiens
1	1.00	1	1	0	0.002	0	0	-0.001	t	123282	HACQKKLLKFEALQQEEGEE	Rattus norvegicus
1	1.00	1	1	0	0	0	0	-0.001	t	123282	HACQKKLLKFEALQQEEGEE	Oryctolagus cuniculus
1	1.00	1	1	0	-0.003	0	0	-0.001	t	117544	KFRFLALKLYFSFTRP	Oryctolagus cuniculus
1	1.00	1	1	0	0	0	0	-0.001	t	131116	TQSYPPPPAWRAASR	Homo sapiens
1	1.00	1	1	0	0	0	0	-0.001	t	122892	RSILPYGDSMDRIE	Homo sapiens
1	1.00	1	1	0	0	0	0	-0.001	t	124588	PQEETLQAFDSHYDYT	Homo sapiens

1	1.00	1	1	0	0	0	0	-0.001	t	133705	SSKTRSFGVHLVGPY	Mus musculus
1	1.00	1	1	0	0	0	0	-0.001	t	118374	GKALCWVCAQSRKHRD	Homo sapiens
1	1.00	1	1	0	0	0	0	-0.001	t	123283	HAEQKKLLKFEALEQEKGKE	Oryctolagus cuniculus
1	1.00	1	1	0	0	0	0	-0.001	t	118798	GAGFGHGSGYGVGHGK	Mus musculus
1	1.00	1	1	0	0	0	0	-0.001	t	117640	SFEYFPSHFFVPELEVTIIC	Mus musculus
1	1.00	1	1	0	0	0	0	-0.001	t	119135	EGVKYVLNETTNWLWAFLAR	Mus musculus
1	1.00	1	1	0	0	0	0	-0.001	t	121542	LFKPPKYHPDVPYVKR	Homo sapiens
1	1.00	1	1	0	0	0	0	-0.001	t	119143	EPTITFGTAI	Oryctolagus cuniculus
1	1.00	1	1	0	0	0	0	-0.001	t	125029	EIERDTERDRFLSADEAV	Homo sapiens
1	1.00	1	1	0	0	0	0	-0.001	t	130729	APHKHKASLSIY	Homo sapiens
1	1.00	1	1	0	0	0	0	-0.001	t	122538	QELECDWEEVTTSGPCGEES	Homo sapiens
1	1.00	1	1	0	0	0	0	-0.001	t	130815	ESDPPVASYQWRLIN	Homo sapiens
1	1.00	1	1	0	0	0	0	-0.001	t	122445	KQYSIEAALRCRRISKEEYH	Homo sapiens
1	1.00	1	1	0	0	0	0	-0.001	t	124008	DGVVLVD	Mus musculus
1	1.00	1	1	0	0	0	0	-0.001	t	117495	ERDRKREKRKPKS	Mus musculus
1	1.00	1	1	0	0	0	0	-0.001	t	117399	VMIKSLPALNSPTVEE	Oryctolagus cuniculus

1	1.00	1	1	0	0	0	0	-0.001	t	130488	FDIYDEDE	Mus musculus
1	1.00	1	1	0	0	0	0	-0.001	t	127643	ERPLELRVRVLDI	Homo sapiens
1	1.00	1	1	0	0	0	0	-0.001	t	133372	SPSPTPTPGPSRRG	Homo sapiens
1	1.00	1	1	0	-0.003	0	0	-0.001	t	121064	WKHWVYYTCCPDTPYLDITY	Oryctolagus cuniculus
1	1.00	1	1	0	0	0	0	-0.001	t	133707	STRKEEKQRNGT	Mus musculus
1	1.00	1	1	0	0	0	0	-0.001	t	133531	CDTLKPGEC	Mus musculus
1	1.00	1	1	0	0	0	0	-0.001	t	133630	LPPGLHVFPLASNRS	Mus musculus
1	1.00	1	1	0	0	0	0	-0.001	t	131303	PVPSTPPTPSPSTPPTPSPS	Homo sapiens
1	1.00	1	1	0	0	0	0	-0.001	t	117742	AKAQTDREDLRTLLRY	Homo sapiens
1	1.00	1	1	0	0	0	0	-0.001	t	117742	AKAQTDREDLRTLLRY	Homo sapiens
1	1.00	1	1	0	0	0	0	-0.001	t	130817	ESVMQSYPPHLQIPG	Homo sapiens
1	1.00	1	1	0	0	0	0	-0.001	t	131686	SYLSLTP	Homo sapiens
1	1.00	1	1	0	0	0	0	-0.001	t	118867	KLSQTSNVDKEEEAV	Mus musculus
1	1.00	1	1	0	0	0	0	-0.001	t	131438	GAVTVAW	Homo sapiens
1	1.00	1	1	0	0	0	0	-0.001	t	131374	CKAKAQTDREDLRTLLRY	Rattus norvegicus
1	1.00	1	1	0	0	0	0	-0.001	t	122377	GLPRLETPADLSTAIASRS	Mus musculus

1	1.00	1	1	0	0	0	0	-0.001	t	119048	VSDYEMKLMDLDVEQ	Mus musculus
1	1.00	1	1	0	0	0	0	-0.001	t	122377	GLPRLETPADLSTAIASRS	Mus musculus
1	1.00	1	1	0	0	0	0	-0.001	t	122377	GLPRLETPADLSTAIASRS	Oryctolagus cuniculus
1	1.00	1	1	0	0	0	0	-0.001	t	118336	ERADLIAYLKKATNE	Mus musculus
1	1.00	1	1	0	0	0	0	-0.001	t	119331	RTVLVSMNYR	Oryctolagus cuniculus
1	1.00	1	1	0	0	0	0	-0.001	t	118764	CYGSFARPTN	Mus musculus
1	1.00	1	1	0	0	0	0	-0.001	t	133900	NEHPVFAYLKDKLP	Homo sapiens
1	1.00	1	1	0	0	0	0	-0.001	t	118581	RDRDRDRE	Mus musculus
1	1.00	1	1	0	0	0	0	-0.001	t	124604	QIERDTERDRFLSAPEAV	Homo sapiens
1	1.00	1	1	0	-0.003	0	0	-0.001	t	122377	GLPRLETPADLSTAIASRS	Oryctolagus cuniculus
1	1.00	1	1	0	0	0	0	-0.001	t	122728	DLSTAIASRSVADKILDLY	Oryctolagus cuniculus
1	1.00	1	1	0	0	0	0	-0.001	t	131040	RLAPEPDDPITPMTK	Homo sapiens
1	1.00	1	1	0	0	0	0	-0.001	t	132687	STQTAGPWHLGKLEL	Mus musculus
1	1.00	1	1	0	0	0	0	-0.001	t	118060	PPPGMRPP	Homo sapiens
1	1.00	1	1	0	0	0	0	-0.001	t	120436	DGTKVSIS	Rattus norvegicus

1	1.00	1	1	0	0	0	0	-0.001	t	130837	FTRHSQTTANPSCPE	Rattus norvegicus
1	1.00	1	1	0	0	0	0	-0.001	t	122780	GQELKNPQEETLQAFDSHYD	Oryctolagus cuniculus
1	1.00	1	1	0	0	0	0	-0.001	t	132390	ISNELNHILLENDMK	Mus musculus
1	1.00	1	1	0	0	0	0	-0.001	t	130868	GQSYPPRPDTSLHVT	Homo sapiens
1	1.00	1	1	0	0	0	0	-0.001	t	133665	PVLLFCFLAGRCVSV	Mus musculus
1	1.00	1	1	0	0	0	0	-0.001	t	132397	KEENLVKSLVSAGSG	Mus musculus
1	1.00	1	1	0	0	0	0	-0.001	t	131628	RGYDSYA	Homo sapiens
1	1.00	1	1	0	0	0	0	-0.001	t	130844	GDQSTDYGIFQINSR	Mus musculus
1	1.00	1	1	0	0	0	0	-0.001	t	121932	TRGYLERRL	Mus musculus
1	1.00	1	1	0	0	0	0	-0.001	t	132365	HKYSWKSWGKAKIIG	Mus musculus
1	1.00	1	1	0	0	0	0	-0.001	t	127185	TCDDPRFQDSS	Oryctolagus cuniculus
1	1.00	1	1	0	0	0	0	-0.001	t	119048	VSDYEMKLMDLDVEQ	Mus musculus
1	1.00	1	1	0	0	0	0	-0.001	t	119052	WNPADYGGIK	Rattus norvegicus
1	1.00	1	1	0	0	0	0	-0.001	t	122842	LLDLPRDLGGMGCSSPPCE	Oryctolagus cuniculus
1	1.00	1	1	0	0	0	0	-0.001	t	122746	EEQEDEIIGFGQELKN	Oryctolagus cuniculus

1	1.00	1	1	0	0	0	0	-0.001	t	125403	ETTKLQRF QAML	Mus musculu s
1	1.00	1	1	0	-0.003	0	0	-0.001	t	118060	PPPGMRP P	Oryctola gus cuniculu s
1	1.00	1	1	0	0	0	0	-0.001	t	119052	WNPADYG GIK	Rattus norvegic us
1	1.00	1	1	0	0	0	0	-0.001	t	119673	WNPDDYG GVK	Rattus norvegic us
1	1.00	1	1	0	0	0	0	-0.001	t	118766	DGEGRGD GGGEGHG QG	Mus musculu s
1	1.00	1	1	0	0	0	0	-0.001	t	117773	CNSRQTD REDELI	Homo sapiens
1	1.00	1	1	0	0	0	0	-0.001	t	132504	NNAIEPRS FSQNPPV	Mus musculu s
1	1.00	1	1	0	0	0	0	-0.001	t	120020	LEDPASRD LVVN	Mus musculu s
1	1.00	1	1	0	0	0	0	-0.001	t	118538	NNGNLQL RKEVTWE VLEG	Mus musculu s
1	1.00	1	1	0	0	0	0	-0.001	t	117773	CNSRQTD REDELI	Homo sapiens
1	1.00	1	1	0	0	0	0	-0.001	t	133535	CKPGEVQ QC	Mus musculu s
1	1.00	1	1	0	0	0	0	-0.001	t	119673	WNPDDYG GVK	Rattus norvegic us
1	1.00	1	1	0	0	0	0	-0.001	t	131717	TTLVNPNK KNSYPHY	Oryctola gus cuniculu s
1	1.00	1	1	0	0	0	0	-0.001	t	133776	AGDLSKVD AKQPGDY	Homo sapiens
1	1.00	1	1	0	0	0	0	-0.001	t	117558	LDGPTYKR RLDECEE	Homo sapiens

1	1.00	1	1	0	0	0	0	-0.001	t	119599	KAEEYILKKPRSELMFEE	Mus musculus
1	1.00	1	1	0	0	0	0	-0.001	t	119673	WNPDDYGGVK	Rattus norvegicus
1	1.00	1	1	0	0	0	0	-0.001	t	131365	ATLVCLI	Homo sapiens
1	1.00	1	1	0	0	0	0	-0.001	t	133528	CADQKPGEC	Mus musculus
1	1.00	1	1	0	0	0	0	-0.001	t	122269	ARQNLKDAGE	Mus musculus
1	1.00	1	1	0	0	0	0	-0.001	t	131338	AASSYLSLTPEQWKSH	Homo sapiens
1	1.00	1	1	0	0	0	0	-0.001	t	119601	KGNNVHGFQKQIIRSFY	Rattus norvegicus
1	1.00	1	1	0	0	0	0	-0.001	t	122262	ALRCRRISKEEYHLYKDGHL	Homo sapiens
1	1.00	1	1	0	0	0	0	-0.001	t	125127	LDPENLTL	Mus musculus
1	1.00	1	1	0	0	0	0	-0.001	t	122468	LLDRKSGKQYSIEAALRCRR	Homo sapiens
1	1.00	1	1	0	0	0	0	-0.001	t	122254	AGETKPSSSLSIGSIISKSP	Homo sapiens
1	1.00	1	1	0	0	0	0	-0.001	t	123849	WQDE	Mus musculus
1	1.00	1	1	0	-0.003	0	0	-0.001	t	122377	GLPRLETPADLSTAIASRS	Oryctolagus cuniculus
1	1.00	1	1	0	0	0	0	-0.001	t	118548	PAPGMRPP	Homo sapiens
1	1.00	1	1	0	0	0	0	-0.001	t	118127	SDDDMGFGLFD	Homo sapiens
1	1.00	1	1	0	0	0	0	-0.001	t	131344	AIGDRSKTDRENSVSIG	Homo sapiens

1	1.00	1	1	0	0	0	0	-0.001	t	131652	SFDSSNPE YFDGYWG YS	Oryctola gus cuniculu s
1	1.00	1	1	0	0	0	0	-0.001	t	126201	GYKEKSKF QDTHNNA	Oryctola gus cuniculu s
1	1.00	1	1	0	0	0	0	-0.001	t	125403	ETTKLQRF QAML	Sus scrofa
1	1.00	1	1	0	0	0	0	-0.001	t	123838	VWQDE	Mus musculu s
1	1.00	1	1	0	0	0	0	-0.001	t	118127	SDDDMGF GLFD	Homo sapiens
1	1.00	1	1	0	0	0	0	-0.001	t	133539	CYKPGEW AC	Mus musculu s
1	1.00	1	1	0	0	0	0	-0.001	t	131697	TGTSSDVG GYNYVSW Y	Homo sapiens
1	1.00	1	1	0	0	0	0	-0.001	t	117907	HKAFKGSI	Oryctola gus cuniculu s
1	1.00	1	1	0	0	0	0	-0.001	t	117907	HKAFKGSI	Homo sapiens
1	1.00	1	1	0	0	0	0	-0.001	t	117909	HKGFKGV D	Oryctola gus cuniculu s
1	1.00	1	1	0	0	0	0	-0.001	t	118826	GSGKGAGI GLGVGGG VGA	Mus musculu s
1	1.00	1	1	0	0	0	0	-0.001	t	124713	SGEQPSDE QPSGEHG	Mus musculu s
1	1.00	1	1	0	0	0	0	-0.001	t	118060	PPPGMRP P	Homo sapiens
1	1.00	1	1	0	0	0	0	-0.001	t	133554	EGLYSPW WPRSLPVL	Mus musculu s
1	1.00	1	1	0	0	0	0	-0.001	t	118829	GVLAGQA SQLNA	Mus musculu s

1	1.00	1	1	0	0	0	0	-0.001	t	131020	QLSPESDYDDHGMRY	Homo sapiens
1	1.00	1	1	0	0	0	0	-0.001	t	119823	EDDDMGFGLFD	Homo sapiens
1	1.00	1	1	0	0	0	0	-0.001	t	133538	CNKPGERTC	Mus musculus
1	1.00	1	1	0	0	0	0	-0.001	t	131504	KATLVCLISDFYPGAV	Homo sapiens
1	1.00	1	1	0	0	0	0	-0.001	t	117614	QGFPFYDKPMRI	Mus musculus
1	1.00	1	1	0	0	0	0	-0.001	t	120682	KILPEVKEKHEFLSRL	Oryctolagus cuniculus
1	1.00	1	1	0	0	0	0	-0.001	t	122377	GLPRLETPADLSTAIASRS	Oryctolagus cuniculus
1	1.00	1	1	0	0	0	0	-0.001	t	122790	HQEEDFRVTCKDIQR	Oryctolagus cuniculus
1	1.00	1	1	0	0	0	0	-0.001	t	108019	LSQPKIVKWDR	Mus musculus
1	1.00	1	1	0	0	0	0	-0.001	t	112453	ASQKRPSQR	Mus musculus
1	1.00	1	1	0	0	0	0	-0.001	t	115268	GLQYTPSWLVG	Mus musculus
1	1.00	1	1	0	0	0	0	-0.001	t	115096	PPSLLFLP	Mus musculus
1	1.00	1	1	0	0	0	0	-0.001	t	112603	MIAATYNFAVLKLMGRGTKF	Rattus norvegicus
1	1.00	1	1	0	0	0	0	-0.001	t	101412	WIRNNF	Mus musculus
1	1.00	1	1	0	0	0	0	-0.001	t	117383	TLHKAFKGSIFVVFDSIESA	Oryctolagus cuniculus

1	1.00	1	1	0	0	0	0	-0.001	t	108834	CKPISGHNSLFWYRQT	Homo sapiens
1	1.00	1	1	0	0	0	0	-0.001	t	102384	EFRH	Cavia porcellus
1	1.00	1	1	0	0	0	0	-0.001	t	102384	EFRH	Mus musculus
1	1.00	1	1	0	0	0	0	-0.001	t	104226	RPQDVKFPGGGQIVGGVYLL	Homo sapiens
1	1.00	1	1	0	-0.004	0	0	-0.001	t	102384	EFRH	Mus musculus
1	1.00	1	1	0	0	0	0	-0.001	t	102384	EFRH	Mus musculus
1	1.00	1	1	0	0	0	0	-0.001	t	99134	DAEFRHDSGYEVHHQ	Mus musculus
1	1.00	1	1	0	0	0	0	-0.001	t	112453	ASQKRPSQR	Mus musculus
1	1.00	1	1	0	0	0	0	-0.001	t	105570	VGGVVIA	Mus musculus
1	1.00	1	1	0	0	0	0	-0.001	t	98822	IEAGAIGFKIHEDWG	Mus musculus
1	1.00	1	1	0	0	0	0	-0.001	t	108217	WWNMFQMGVV	Homo sapiens
1	1.00	1	1	0	0	0	0	-0.001	t	108216	WWATFFEATR	Homo sapiens
1	1.00	1	1	0	0	0	0	-0.001	t	102384	EFRH	Mus musculus
1	1.00	1	1	0	0	0	0	-0.001	t	108629	SHQESTRGRSRGRSGRSGS	Homo sapiens
1	1.00	1	1	0	0	0	0	-0.001	t	112453	ASQKRPSQR	Mus musculus
1	1.00	1	1	0	0	0	0	-0.001	t	107891	HRLAFGQYT	Mus musculus
1	1.00	1	1	0	0	0	0	-0.001	t	99040	VLDTDYKKYLLFCMENSAEP	Homo sapiens

1	1.00	1	1	0	0	0	0	-0.001	t	100635	VTNYLVDGNG	Mus musculus
1	1.00	1	1	0	0	0	0	-0.001	t	100635	VTNYLVDGNG	Mus musculus
1	1.00	1	1	0	0	0	0	-0.001	t	104443	DAEFRHDSGYE	Mus musculus
1	1.00	1	1	0	0	0	0	-0.001	t	105127	DAEFRHD	Mus musculus
1	1.00	1	1	0	0	0	0	-0.001	t	115069	KEVDRLMS	Mus musculus
1	1.00	1	1	0	0	0	0	-0.001	t	117015	KEQFLDGDGWTSRWIESK	Homo sapiens
1	1.00	1	1	0	0	0	0	-0.001	t	105127	DAEFRHD	Mus musculus
1	1.00	1	1	0	0	0	0	-0.001	t	107013	YGASSRATGIPDR	Oryctolagus cuniculus
1	1.00	1	1	0	0	0	0	-0.001	t	108599	QGDAKKGANLFKTRC	Homo sapiens
1	1.00	1	1	0	0	0	0	-0.001	t	105127	DAEFRHD	Mus musculus
1	1.00	1	1	0	0	0	0	-0.001	t	100070	GLMVGGVVIA	Oryctolagus cuniculus
1	1.00	1	1	0	0	0	0	-0.001	t	100070	GLMVGGVVIA	Oryctolagus cuniculus
1	1.00	1	1	0	0	0	0	-0.001	t	100070	GLMVGGVVIA	Oryctolagus cuniculus
1	1.00	1	1	0	0	0	0	-0.001	t	99134	DAEFRHDSGYEVHHQ	Mus musculus
1	1.00	1	1	0	0	0	0	-0.001	t	112149	GVGWIRQ	Homo sapiens

1	1.00	1	1	0	0	0	0	-0.001	t	107166	GPPGPQG	Mus musculus
1	1.00	1	1	0	0	0	0	-0.001	t	108215	WVAMHPQGDL	Homo sapiens
1	1.00	1	1	0	0	0	0	-0.001	t	117140	DWEYSVWLSN	Mus musculus
1	1.00	1	1	0	0	0	0	-0.001	t	117140	DWEYSVWLSN	Mus musculus
1	1.00	1	1	0	0	0	0	-0.001	t	99134	DAEFRHDSGYEVHHQ	Mus musculus
1	1.00	1	1	0	0	0	0	-0.001	t	100070	GLMVGGVVIA	Oryctolagus cuniculus
1	1.00	1	1	0	0	0	0	-0.001	t	108052	NWLSLITAAR	Homo sapiens
1	1.00	1	1	0	0	0	0	-0.001	t	105127	DAEFRHD	Mus musculus
1	1.00	1	1	0	0	0	0	-0.001	t	113879	SQRSQDEN	Rattus norvegicus
1	1.00	1	1	0	0	0	0	-0.001	t	108213	WQQGNVF	Mus musculus
1	1.00	1	1	0	0	0	0	-0.001	t	108064	PGPPGPP	Mus musculus
1	1.00	1	1	0	-0.002	0	0	-0.001	t	99921	DAEFRHDS	Mus musculus
1	1.00	1	1	0	0	0	0	-0.001	t	107882	HEHTFGRQW	Mus musculus
1	1.00	1	1	0	0	0	0	-0.001	t	117246	VTWEVLEGEVEKEALKKI	Oryctolagus cuniculus
1	1.00	1	1	0	0	0	0	-0.001	t	104569	MASPGSGFWSFGSEDGS	Mus musculus
1	1.00	1	1	0	0	0	0	-0.001	t	112672	TAEFQMTFHLFI	Oryctolagus cuniculus

1	1.00	1	1	0	0	0	0	-0.001	t	99134	DAEFRHDSGYEVHHQ	Mus musculus
1	1.00	1	1	0	-0.003	0	0	-0.001	t	111193	DGPAGQAEPDRAHYNI	Oryctolagus cuniculus
1	1.00	1	1	0	0	0	0	-0.001	t	99134	DAEFRHDSGYEVHHQ	Mus musculus
1	1.00	1	1	0	0	0	0	-0.001	t	108345	ENPKKYIPGTK	Homo sapiens
1	1.00	1	1	0	0	0	0	-0.001	t	99134	DAEFRHDSGYEVHHQ	Mus musculus
1	1.00	1	1	0	0	0	0	-0.001	t	104443	DAEFRHDSGYE	Mus musculus
1	1.00	1	1	0	0	0	0	-0.001	t	110863	GLKAKPKFTLGKRKATPTTS	Homo sapiens
1	1.00	1	1	0	0	0	0	-0.001	t	104443	DAEFRHDSGYE	Mus musculus
1	1.00	1	1	0	0	0	0	-0.001	t	108212	WPPSFWYHPQ	Homo sapiens
1	1.00	1	1	0	0	0	0	-0.001	t	115047	ELQFPFLQD	Mus musculus
1	1.00	1	1	0	0	0	0	-0.001	t	106131	TAIPSCPEGTVPLYS	Homo sapiens
1	1.00	1	1	0	0	0	0	-0.001	t	108210	WFTSWERDWW	Homo sapiens
1	1.00	1	1	0	0	0	0	-0.001	t	109253	GSLPQKSQRSQDENPVVHF	Rattus norvegicus
1	1.00	1	1	0	0	0	0	-0.001	t	117345	PRRRSRKPEAPRRRS	Homo sapiens
1	1.00	1	1	0	-0.003	0	0	-0.001	t	117260	ANNGNLQLRNKEVTWEVLEG	Oryctolagus cuniculus
1	1.00	1	1	0	0	0	0	-0.001	t	99134	DAEFRHDSGYEVHHQ	Mus musculus

1	1.00	1	1	0	0	0	0	-0.001	t	103998	IVKWDRDM	Mus musculus
1	1.00	1	1	0	0	0	0	-0.001	t	102435	FPKLTLYSW	Mus musculus
1	1.00	1	1	0	-0.002	0	0	-0.001	t	108295	CKRDSTWC	Mus musculus
1	1.00	1	1	0	0	0	0	-0.001	t	112453	ASQKRPSQR	Mus musculus
1	1.00	1	1	0	-0.003	0	0	-0.001	t	116677	ASATQLYKTCKQAGTCPPD	Oryctolagus cuniculus
1	1.00	1	1	0	0	0	0	-0.001	t	111125	ASQKRPSQR	Mus musculus
1	1.00	1	1	0	0	0	0	-0.001	t	108074	PQVYTLP	Homo sapiens
1	1.00	1	1	0	0	0	0	-0.001	t	114226	QKSHGRTQDENPVVHF	Mus musculus
1	1.00	1	1	0	0	0	0	-0.001	t	104443	DAEFRHDSGYE	Mus musculus
1	1.00	1	1	0	0	0	0	-0.001	t	107814	EGLHNHY	Mus musculus
1	1.00	1	1	0	0	0	0	-0.001	t	107814	EGLHNHY	Homo sapiens
1	1.00	1	1	0	-0.003	0	0	-0.001	t	108522	LHEYMLDLQPETTDL	Oryctolagus cuniculus
1	1.00	1	1	0	0	0	0	-0.001	t	108382	GGVVIA	Mus musculus
1	1.00	1	1	0	-0.003	0	0	-0.001	t	108075	PREPQVY	Oryctolagus cuniculus
1	1.00	1	1	0	0	0	0	-0.001	t	108075	PREPQVY	Homo sapiens

1	1.00	1	1	0	0	0	0	-0.001	t	108200	VSLGKWL GHPDKF	Mus musculu s
1	1.00	1	1	0	0	0	0	-0.001	t	108382	GGVVIA	Mus musculu s
1	1.00	1	1	0	0	0	0	-0.001	t	99922	DAEFRHDS GY	Mus musculu s
1	1.00	1	1	0	0	0	0	-0.001	t	104906	MVGGVVI A	Mus musculu s
1	1.00	1	1	0	0	0	0	-0.001	t	99922	DAEFRHDS GY	Mus musculu s
1	1.00	1	1	0	0	0	0	-0.001	t	99922	DAEFRHDS GY	Mus musculu s
1	1.00	1	1	0	0	0	0	-0.001	t	100153	INDQIKFII N	Mus musculu s
1	1.00	1	1	0	0	0	0	-0.001	t	107949	KSLSLSP	Oryctola gus cuniculu s
1	1.00	1	1	0	0	0	0	-0.001	t	102860	TPYTSLYT W	Mus musculu s
1	1.00	1	1	0	0	0	0	-0.001	t	104906	MVGGVVI A	Mus musculu s
1	1.00	1	1	0	0	0	0	-0.001	t	112453	ASQKRPSQ R	Mus musculu s
1	1.00	1	1	0	0	0	0	-0.001	t	110037	VIASEGDS VNITCST	Rattus norvegic us
1	1.00	1	1	0	0	0	0	-0.001	t	99134	DAEFRHDS GYEVHHQ	Mus musculu s
1	1.00	1	1	0	0	0	0	-0.001	t	106675	NEEGFFSA RGHRPLDK K	Homo sapiens
1	1.00	1	1	0	0	0	0	-0.001	t	113013	GSLPQKSQ RSQDEN	Rattus norvegic us
1	1.00	1	1	0	0	0	0	-0.001	t	110385	QLYKTCKQ AGTCPPDII PKV	Oryctola gus cuniculu s

1	1.00	1	1	0	0	0	0	-0.001	t	99134	DAEFRHDSGYEVHHQ	Mus musculus
1	1.00	1	1	0	0	0	0	-0.001	t	114414	IPKVPPGPNITA	Homo sapiens
1	1.00	1	1	0	0	0	0	-0.001	t	107662	VLTVLHQNWL	Oryctolagus cuniculus
1	1.00	1	1	0	0	0	0	-0.001	t	108088	RAAPFGNQW	Mus musculus
1	1.00	1	1	0	0	0	0	-0.001	t	102880	VDELD	Mus musculus
1	1.00	1	1	0	0	0	0	-0.001	t	113942	TQDENPVVHF	Mus musculus
1	1.00	1	1	0	0	0	0	-0.001	t	113365	EHPVTGCGERTEGRCLHYTV	Mus musculus
1	1.00	1	1	0	0	0	0	-0.001	t	108182	VDLMTIRSCR	Homo sapiens
1	1.00	1	1	0	0	0	0	-0.001	t	117383	TLHKAFKGSIFVVFDSIESA	Homo sapiens
1	1.00	1	1	0	0	0	0	-0.001	t	105021	VHHQKLVFFAEDVGSNK	Oryctolagus cuniculus
1	1.00	1	1	0	0	0	0	-0.001	t	113942	TQDENPVVHF	Mus musculus
1	1.00	1	1	0	0	0	0	-0.001	t	104443	DAEFRHDSGYE	Mus musculus
1	1.00	1	1	0	0.002	0	0	-0.001	t	113879	SQRSQDEN	Rattus norvegicus
1	1.00	1	1	0	0	0	0	-0.001	t	108104	RRLPFGSQM	Mus musculus
1	1.00	1	1	0	0	0	0	-0.001	t	108105	RRLPFGSSL	Mus musculus

1	1.00	1	1	0	0	0	0	-0.001	t	104850	KEGILQYCQEVYPELQ	Mus musculus
1	1.00	1	1	0	0	0	0	-0.001	t	117260	ANNGNLQLRNKEVTWEVLEG	Homo sapiens
1	1.00	1	1	0	0	0	0	-0.001	t	109406	KIQPSEPRDSAVYFCA	Homo sapiens
1	1.00	1	1	0	0	0	0	-0.001	t	102944	YGCYCGPGG	Mus musculus
1	1.00	1	1	0	0	0	0	-0.001	t	108168	TRSFGIQAT	Mus musculus
1	1.00	1	1	0	0	0	0	-0.001	t	115127	WTQSLR	Mus musculus
1	1.00	1	1	0	0	0	0	-0.001	t	108167	TRAFGNEAT	Mus musculus
1	1.00	1	1	0	0	0	0	-0.001	t	108525	LKKPKDRNDLI	Homo sapiens
1	1.00	1	1	0	0	0	0	-0.001	t	104443	DAEFRHDSGYE	Mus musculus
1	1.00	1	1	0	0	0	0	-0.001	t	117315	KALSVETEKLLKYLEAV	Homo sapiens
1	1.00	1	1	0	0	0	0	-0.001	t	108107	RYAFGSQIA	Mus musculus
1	1.00	1	1	0	0	0	0	-0.001	t	113365	EHPVTGCGERTEGRCLHYTV	Mus musculus
1	1.00	1	1	0	0	0	0	-0.001	t	113365	EHPVTGCGERTEGRCLHYTV	Mus musculus
1	1.00	1	1	0	0	0	0	-0.001	t	108666	TRCAQCHTLK	Homo sapiens
1	1.00	1	1	0	0	0	0	-0.001	t	116745	GSNKGAIIGLMVGGVVIA	Mus musculus
1	1.00	1	1	0	-0.003	0	0	-0.001	t	111309	GDTPTLHEYMLDLQ	Oryctolagus cuniculus

1	1.00	1	1	0	0	0	0	-0.001	t	107951	KTTICGKG LSATVT	Mus musculus
1	1.00	1	1	0	0	0	0	-0.001	t	117074	RSLLSGGL P	Mus musculus
1	1.00	1	1	0	0	0	0	-0.001	t	116677	ASATQLYK TCKQAGT CPPD	Mus musculus
1	1.00	1	1	0	0.002	0	0	-0.001	t	116971	FAVLKLM GRGTKF	Rattus norvegicus
1	1.00	1	1	0	0	0	0	-0.001	t	104443	DAEFRHDS GYE	Mus musculus
1	1.00	1	1	0	-0.003	0	0	-0.001	t	107773	CSVMHEG	Oryctolagus cuniculus
1	1.00	1	1	0	0	0	0	-0.001	t	107773	CSVMHEG	Homo sapiens
1	1.00	1	1	0	0	0	0	-0.001	t	117333	MVTSTITE KLLKNLVKI	Homo sapiens
1	1.00	1	1	0	0	0	0	-0.001	t	108156	TDIPPCPH GWISLWK	Homo sapiens
1	1.00	1	1	0	0	0	0	-0.001	t	106842	SKDEIDSA VKMLVSLK MSY	Mus musculus
1	1.00	1	1	0	-0.002	0	0	-0.001	t	108297	CKYLWSKC	Mus musculus
1	1.00	1	1	0	0	0	0	-0.001	t	108019	LSQPKIVK WDR	Mus musculus
1	1.00	1	1	0	0	0	0	-0.001	t	107768	CIAPKRHN SAC	Mus musculus
1	1.00	1	1	0	0	0	0	-0.001	t	106036	QFAGKDLE SIK	Mus musculus
1	1.00	1	1	0	0	0	0	-0.001	t	102563	KKRPKPG	Homo sapiens
1	1.00	1	1	0	0	0	0	-0.001	t	108156	TDIPPCPH GWISLWK	Homo sapiens

1	1.00	1	1	0	0	0	0	-0.001	t	112508	FFKNIVTPRTPPPSQQK	Mus musculus
1	1.00	1	1	0	0	0	0	-0.001	t	108126	SIFFAVVLEL	Homo sapiens
1	1.00	1	1	0	0	0	0	-0.001	t	108628	SHQESTRGRSRGRSGRSGS	Homo sapiens
1	1.00	1	1	0	-0.003	0	0	-0.001	t	108019	LSQPKIVKWDR	Oryctolagus cuniculus
1	1.00	1	1	0	0	0	0	-0.001	t	108378	GFAGQAGPAGATGAPGRP	Mus musculus
1	1.00	1	1	0	0	0	0	-0.001	t	102942	YGCYCGKGG	Mus musculus
1	1.00	1	1	0	0	0	0	-0.001	t	99945	DIRDSLSEEE	Mus musculus
1	1.00	1	1	0	0	0	0	-0.001	t	105127	DAEFRHD	Mus musculus
1	1.00	1	1	0	0	0	0	-0.001	t	99975	EEKELLKRIQ	Mus musculus
1	1.00	1	1	0	0	0	0	-0.001	t	99134	DAEFRHDSGYEVHHQ	Mus musculus
1	1.00	1	1	0	0	0	0	-0.001	t	108152	TCPPCPAPELLGG	Homo sapiens
1	1.00	1	1	0	0	0	0	-0.001	t	99975	EEKELLKRIQ	Mus musculus
1	1.00	1	1	0	0	0	0	-0.001	t	115058	GNAEVHASFFDIGGS	Oryctolagus cuniculus
1	1.00	1	1	0	0	0	0	-0.001	t	117260	ANNGNLQLRNKEVTWEVLEG	Mus musculus
1	1.00	1	1	0	-0.003	0	0	-0.001	t	108131	SKDWSFY	Oryctolagus cuniculus

1	1.00	1	1	0	0	0	0	-0.001	t	107479	KFNWYVD	Homo sapiens
1	1.00	1	1	0	-0.003	0	0	-0.001	t	108131	SKDWSFY	Oryctolagus cuniculus
1	1.00	1	1	0	0	0	0	-0.001	t	106321	DAEFRH	Mus musculus
1	1.00	1	1	0	0	0	0	-0.001	t	108131	SKDWSFY	Mus musculus
1	1.00	1	1	0	0	0	0	-0.001	t	108131	SKDWSFY	Mus musculus
1	1.00	1	1	0	0	0	0	-0.001	t	107758	AWNCCVKQTP	Homo sapiens
1	1.00	1	1	0	0	0	0	-0.001	t	117300	GSGKGKVQFQGKKTKF	Oryctolagus cuniculus
1	1.00	1	1	0	0	0	0	-0.001	t	112453	ASQKRPSQR	Mus musculus
1	1.00	1	1	0	0	0	0	-0.001	t	108007	LLPWLDGHPM	Homo sapiens
1	1.00	1	1	0	0	0	0	-0.001	t	108134	SLCINLRAAR	Homo sapiens
1	1.00	1	1	0	0	0	0	-0.001	t	107951	KTTICGKGLSATVT	Mus musculus
1	1.00	1	1	0	0	0	0	-0.001	t	108135	SLITAARGAPS	Homo sapiens
1	1.00	1	1	0	-0.003	0	0	-0.001	t	107479	KFNWYVD	Oryctolagus cuniculus
1	1.00	1	1	0	0	0	0	-0.001	t	107479	KFNWYVD	Oryctolagus cuniculus
1	1.00	1	1	0	0	0	0	-0.001	t	99134	DAEFRHDSGYEVHHQ	Mus musculus

1	1.00	1	1	0	0	0	0	-0.001	t	107479	KFNWYVD	Mus musculus
1	1.00	1	1	0	0	0	0	-0.001	t	107242	SQTTANPSCPEGT	Rattus norvegicus
1	1.00	1	1	0	0	0	0	-0.001	t	78457	LRLPPAP	Mus musculus
1	1.00	1	1	0	0	0	0	-0.001	t	70391	VPNLRGDLQVLAQKVARTLP	Mus musculus
1	1.00	1	1	0	0	0	0	-0.001	t	79535	EDDDDDFGMGALF	Mus musculus
1	1.00	1	1	0	0	0	0	-0.001	t	95163	VLPGHQPSNGNMIYSYHTME	Rattus norvegicus
1	1.00	1	1	0	0	0	0	-0.001	t	78536	TPLCDYAAARVGACG	Mus musculus
1	1.00	1	1	0	0	0	0	-0.001	t	97020	TVTRGTISDP	Oryctolagus cuniculus
1	1.00	1	1	0	0	0	0	-0.001	t	64086	THTPLPRGIGY	Mus musculus
1	1.00	1	1	0	0	0	0	-0.001	t	96457	KGTVNLTWSR	Mus musculus
1	1.00	1	1	0	0	0	0	-0.001	t	79284	LDAVPL	Mus musculus
1	1.00	1	1	0	0	0	0	-0.001	t	62715	SYSYNKDNNELSHFA	Mus musculus
1	1.00	1	1	0	0	0	0	-0.001	t	64099	THWQLGERPD	Homo sapiens
1	1.00	1	1	0	0	0	0	-0.001	t	62715	SYSYNKDNNELSHFA	Mus musculus
1	1.00	1	1	0	0	0	0	-0.001	t	65614	TPIRHRS	Homo sapiens

1	1.00	1	1	0	-0.003	0	0	-0.001	t	95142	RIKNTDGSIS	Oryctolagus cuniculus
1	1.00	1	1	0	0	0	0	-0.001	t	77975	QACQNNDTCPRL	Mus musculus
1	1.00	1	1	0	0	0	0	-0.001	t	96846	RVFPRGTVENP	Oryctolagus cuniculus
1	1.00	1	1	0	0	0	0	-0.001	t	70384	VPNLQGVPESPFSRT	Homo sapiens
1	1.00	1	1	0	0	0	0	-0.001	t	69942	VMENFVAF	Homo sapiens
1	1.00	1	1	0	0	0	0	-0.001	t	94483	CYKKVWRDHRGTIIERGC	Mus musculus
1	1.00	1	1	0	0	0	0	-0.001	t	72087	VYLLPR	Homo sapiens
1	1.00	1	1	0	0	0	0	-0.001	t	79266	KDAVPN	Mus musculus
1	1.00	1	1	0	0	0	0	-0.001	t	63391	TEHRDYHPQQRG	Mus musculus
1	1.00	1	1	0	0	0	0	-0.001	t	75847	YSPELE	Mus musculus
1	1.00	1	1	0	0	0	0	-0.001	t	63037	TAVSPTTLR	Oryctolagus cuniculus
1	1.00	1	1	0	0	0	0	-0.001	t	66749	TTPAQGNSMFPSC	Oryctolagus cuniculus
1	1.00	1	1	0	0	0	0	-0.001	t	70554	VQESYYRYVAREQSCR	Oryctolagus cuniculus
1	1.00	1	1	0	0	0	0	-0.001	t	67994	VDKGHNYCGYPENLLI	Homo sapiens

1	1.00	1	1	0	0	0	0	-0.001	t	63037	TAVSPTTLR	Sus scrofa
1	1.00	1	1	0	0	0	0	-0.001	t	62932	TANGFYRLPSG	Mus musculus
1	1.00	1	1	0	0	0	0	-0.001	t	97023	VATRSQTDTSEKDC	Oryctolagus cuniculus
1	1.00	1	1	0	0	0	0	-0.001	t	75849	YSPEVR	Mus musculus
1	1.00	1	1	0	0	0	0	-0.001	t	71507	VTSTLTSLFRPGASQKIQLV	Pan troglodytes
1	1.00	1	1	0	0	0	0	-0.001	t	60751	SRNWLPGPCY	Mus musculus
1	1.00	1	1	0	0	0	0	-0.001	t	62328	SVSLPYANLATH	Mus musculus
1	1.00	1	1	0	-0.002	0	0	-0.001	t	76618	YYTKDTNNNLTLVPAVVG	Mus musculus
1	1.00	1	1	0	-0.003	0	0	-0.001	t	66108	TRTNPWPALAEGEF	unidentified
1	1.00	1	1	0	0	0	0	-0.001	t	62141	SVGHMTGRFTSLFSVG	Homo sapiens
1	1.00	1	1	0	0	0	0	-0.001	t	74506	YKSDNIR	Homo sapiens
1	1.00	1	1	0	0	0	0	-0.001	t	72113	VYNKTISGSGP	Oryctolagus cuniculus
1	1.00	1	1	0	0	0	0	-0.001	t	63098	TCRRMLAFLKDKENR	Homo sapiens
1	1.00	1	1	0	0	0	0	-0.001	t	72113	VYNKTISGSGP	Mus musculus
1	1.00	1	1	0	0	0	0	-0.001	t	65820	TQAHTTMRLANLFTSG	Homo sapiens

1	1.00	1	1	0	0	0	0	-0.001	t	79535	EDDDDDF GMGALF	Mus musculus
1	1.00	1	1	0	0	0	0	-0.001	t	79474	YDAVPY	Mus musculus
1	1.00	1	1	0	0	0	0	-0.001	t	70391	VPNLRGDL QVLAQKV ARTLP	Mus musculus
1	1.00	1	1	0	-0.002	0	0	-0.001	t	66347	TSNTHACR TCSNPCR	Mus musculus
1	1.00	1	1	0	0	0	0	-0.001	t	70817	VRRRVNA ANKKPEDL TLNDL	Aotus lemurinus
1	1.00	1	1	0	0	0	0	-0.001	t	73081	WSLFLNH AE	Mus musculus
1	1.00	1	1	0	0	0	0	-0.001	t	97348	GLFGAIAG FIEGGW	Oryctolagus cuniculus
1	1.00	1	1	0	0	0	0	-0.001	t	77802	TEYSASVK GRFI	Mus
1	1.00	1	1	0	0	0	0	-0.001	t	64045	THKVGTSF TFES	Oryctolagus cuniculus
1	1.00	1	1	0	-0.003	0	0	-0.001	t	72113	VYNKTISG SGP	Oryctolagus cuniculus
1	1.00	1	1	0	0	0	0	-0.001	t	66751	TTPAQGTS M	Mus musculus
1	1.00	1	1	0	0	0	0	-0.001	t	79389	SDVAGLQ NDPTT	Mus musculus
1	1.00	1	1	0	0	0	0	-0.001	t	60722	SRLGRSSA WV	Homo sapiens
1	1.00	1	1	0	0	0	0	-0.001	t	65105	TLTPENTL	Mus musculus
1	1.00	1	1	0	0	0	0	-0.001	t	97547	PFVPRADE PARKGKV H	Mus musculus

1	1.00	1	1	0	0	0	0	-0.001	t	78176	HLP	MD
1	1.00	1	1	0	0	0	0	-0.001	t	64411	TIPAQGTSM	Mus musculus
1	1.00	1	1	0	0	0	0	-0.001	t	69323	VKTIGDKRTLTLNTTANYT	Mus musculus
1	1.00	1	1	0	0	0	0	-0.001	t	74036	YGLFHKEKMLL	Mus musculus
1	1.00	1	1	0	0	0	0	-0.001	t	79153	ALQREAAERSEDEIK	Oryctolagus cuniculus
1	1.00	1	1	0	0	0	0	-0.001	t	73784	YEVRAELPGVDPDKDVDIMV	Mus musculus
1	1.00	1	1	0	0	0	0	-0.001	t	65678	TPPAYR	Mus musculus
1	1.00	1	1	0	0	0	0	-0.001	t	66257	TSHHDSHGLHRV	Mus musculus
1	1.00	1	1	0	0	0	0	-0.001	t	95119	MKKPGENIKPLSCKYSYPKH	Rattus norvegicus
1	1.00	1	1	0	0	0	0	-0.001	t	72065	VYKLDISEA	Mus musculus
1	1.00	1	1	0	0	0	0	-0.001	t	78424	IPRSRQIDLQIG	Mus musculus
1	1.00	1	1	0	0	0	0	-0.001	t	62852	TAGHGAYGITSLFTRG	Homo sapiens
1	1.00	1	1	0	0	0	0	-0.001	t	97596	QTPLTMAALELL	Mus musculus
1	1.00	1	1	0	0	0	0	-0.001	t	66347	TSNTHACRTCSNPCR	Mus musculus
1	1.00	1	1	0	0	0	0	-0.001	t	70460	VPQYLRWFEEWAEDFCRK	Homo sapiens

1	1.00	1	1	0	0	0	0	-0.001	t	71263	VTCDTPPTY	Oryctolagus cuniculus
1	1.00	1	1	0	0	0	0	-0.001	t	70391	VPNLRGDLQVLAQKVARTLP	Mus musculus
1	1.00	1	1	0	0	0	0	-0.001	t	80002	RKADLEKALEGAM	Oryctolagus cuniculus
1	1.00	1	1	0	0	0	0	-0.001	t	77810	YSASVKGRFIVS	Mus
1	1.00	1	1	0	0	0	0	-0.001	t	97288	ETQLTTAGLRLL	Mus musculus
1	1.00	1	1	0	0	0	0	-0.001	t	63736	TFQVEVPGSQHIDS	Oryctolagus cuniculus
1	1.00	1	1	0	0	0	0	-0.001	t	79278	KSAVGY	Mus musculus
1	1.00	1	1	0	0	0	0	-0.001	t	79867	INNLIVELIRGT	Mus musculus
1	1.00	1	1	0	0	0	0	-0.001	t	62932	TANGFYRLPSG	Homo sapiens
1	1.00	1	1	0	0	0	0	-0.001	t	79602	LLPDSVI	Mus musculus
1	1.00	1	1	0	0	0	0	-0.001	t	70542	VQDLMKSSGV	Homo sapiens
1	1.00	1	1	0	0	0	0	-0.001	t	74462	YKIGDNW	Homo sapiens
1	1.00	1	1	0	0	0	0	-0.001	t	62345	SVTCPQQV	Mus musculus
1	1.00	1	1	0	0	0	0	-0.001	t	78873	VKFPGGGQIVGGVYLLPRRG	Homo sapiens

1	1.00	1	1	0	0	0	0	-0.001	t	74970	YLVKYLCKIARSQDA	Oryctolagus cuniculus
1	1.00	1	1	0	0	0	0	-0.001	t	72251	WAPSV	Mus musculus
1	1.00	1	1	0	0	0	0	-0.001	t	70817	VRRRVNAANKKPEDLTLNDL	Homo sapiens
1	1.00	1	1	0	0	0	0	-0.001	t	74821	YLMEVQK	Homo sapiens
1	1.00	1	1	0	0	0	0	-0.001	t	61612	STFYGKPTGAGPK	Mus musculus
1	1.00	1	1	0	-0.003	0	0	-0.001	t	72178	VYVEELKPTPEGDLEILQK	Oryctolagus cuniculus
1	1.00	1	1	0	0	0	0	-0.001	t	72178	VYVEELKPTPEGDLEILQK	Mus musculus
1	1.00	1	1	0	-0.002	0	0	-0.001	t	72805	WLTEKEGSYP	Mus musculus
1	1.00	1	1	0	0	0	0	-0.001	t	92900	HEALREPRKLERTISK	Oryctolagus cuniculus
1	1.00	1	1	0	0	0	0	-0.001	t	78286	TTAETLDATR	Mus musculus
1	1.00	1	1	0	0	0	0	-0.001	t	78286	TTAETLDATR	Mus musculus
1	1.00	1	1	0	0	0	0	-0.001	t	60797	SRSESRKNRGGREEIL	Homo sapiens
1	1.00	1	1	0	0	0	0	-0.001	t	94380	YVFLPPEEEKDKR	Oryctolagus cuniculus
1	1.00	1	1	0	0	0	0	-0.001	t	74009	YGGPANKKNAG	Mus musculus
1	1.00	1	1	0	0	0	0	-0.001	t	60526	SQPNTEQSPAIVLPF	Mus musculus

1	1.00	1	1	0	0	0	0	-0.001	t	79806	EKPAKTYDDAAS	Mus musculus
1	1.00	1	1	0	0	0	0	-0.001	t	67325	TYEAALKQYEADL	Mus musculus
1	1.00	1	1	0	0	0	0	-0.001	t	97595	QTPLTMAALELF	Mus musculus
1	1.00	1	1	0	0	0	0	-0.001	t	66304	TSLNDRQGNATK	Oryctolagus cuniculus
1	1.00	1	1	0	0	0	0	-0.001	t	67967	VDGSDP	Mus musculus
1	1.00	1	1	0	0	0	0	-0.001	t	76618	YYTKDTNNNLTLVPAVVG	Homo sapiens
1	1.00	1	1	0	0	0	0	-0.001	t	94606	LTGGFYEPL	Mus musculus
1	1.00	1	1	0	0	0	0	-0.001	t	79852	HFFKNIVTPRT	Rattus norvegicus
1	1.00	1	1	0	0	0	0	-0.001	t	68041	VDPQ	Mus musculus
1	1.00	1	1	0	0	0	0	-0.001	t	65409	TNRNPQ	Mus musculus
1	1.00	1	1	0	0	0	0	-0.001	t	74045	YGLLAVDPLFKP	Gallus gallus
1	1.00	1	1	0	0	0	0	-0.001	t	63122	TDAYNQKLSERRAN	Mus musculus
1	1.00	1	1	0	0	0	0	-0.001	t	63122	TDAYNQKLSERRAN	Mus musculus
1	1.00	1	1	0	0	0	0	-0.001	t	63758	TFTNPVVSTTQWSAP	Oryctolagus cuniculus

1	1.00	1	1	0	0	0	0	-0.001	t	63122	TDAYNQKLSERRAN	Mus musculus
1	1.00	1	1	0	0	0	0	-0.001	t	61708	STLAKHAPAGYR	Oryctolagus cuniculus
1	1.00	1	1	0	0	0	0	-0.001	t	67325	TYEAALKQYEADL	Mus musculus
1	1.00	1	1	0	0	0	0	-0.001	t	67325	TYEAALKQYEADL	Mus musculus
1	1.00	1	1	0	0	0	0	-0.001	t	67325	TYEAALKQYEADL	Mus musculus
1	1.00	1	1	0	0	0	0	-0.001	t	74006	YGGIYMNG	Mus musculus
1	1.00	1	1	0	0	0	0	-0.001	t	79188	DSAVGY	Mus musculus
1	1.00	1	1	0	0	0	0	-0.001	t	70391	VPNLRGDLQVLAQKVARTLP	Mus musculus
1	1.00	1	1	0	0	0	0	-0.001	t	74497	YKQKIIAP	Mus musculus
1	1.00	1	1	0	0	0	0	-0.001	t	62489	SWPLYDASGLG	Mus musculus
1	1.00	1	1	0	0	0	0	-0.001	t	97256	EGAKGENKRPNDKAGEV	Mus musculus
1	1.00	1	1	0	0	0	0	-0.001	t	69178	VKAETRLNPDLQPTE	Mus musculus
1	1.00	1	1	0	0	0	0	-0.001	t	96102	APPLLPH	Homo sapiens
1	1.00	1	1	0	0	0	0	-0.001	t	96085	ALCSEKLDQWLCEKL	Homo sapiens

1	1.00	1	1	0	0	0	0	-0.001	t	74226	YIIDHVDQHNATEIC	Oryctolagus cuniculus
1	1.00	1	1	0	0	0	0	-0.001	t	76290	YVHDNC	Mus musculus
1	1.00	1	1	0	0	0	0	-0.001	t	97252	DTPLTTAALRLV	Mus musculus
1	1.00	1	1	0	0	0	0	-0.001	t	60671	SRGKGPGKKNK	Sus scrofa
1	1.00	1	1	0	0	0	0	-0.001	t	60671	SRGKGPGKKNK	Sus scrofa
1	1.00	1	1	0	0	0	0	-0.001	t	69303	VKQSRGEA	Mus musculus
1	1.00	1	1	0	0	0	0	-0.001	t	95051	YSQFEKSY	Oryctolagus cuniculus
1	1.00	1	1	0	0	0	0	-0.001	t	67076	TVLRDPVFYQ	Homo sapiens
1	1.00	1	1	0	0	0	0	-0.001	t	77955	MCSPTALRCQAH	Mus musculus
1	1.00	1	1	0	0	0	0	-0.001	t	65050	TLRVYK	Mus musculus
1	1.00	1	1	0	0	0	0	-0.001	t	79176	DAVPNM	Mus musculus
1	1.00	1	1	0	0	0	0	-0.001	t	79176	DAVPNM	Mus musculus
1	1.00	1	1	0	0	0	0	-0.001	t	94513	FHPDK	Mus musculus
1	1.00	1	1	0	0	0	0	-0.001	t	61686	STKAAKKPEAKREAI	Mus musculus
1	1.00	1	1	0	0	0	0	-0.001	t	62885	TAIHAVW	Homo sapiens

1	1.00	1	1	0	0	0	0	-0.001	t	95043	SPHQVYN	Oryctolagus cuniculus
1	1.00	1	1	0	0	0	0	-0.001	t	72940	WPKDTDT	Homo sapiens
1	1.00	1	1	0	0	0	0	-0.001	t	53023	QYPWRLA	Homo sapiens
1	1.00	1	1	0	0	0	0	-0.001	t	41233	MDIDPYKEFGA	Mus musculus
1	1.00	1	1	0	0	0	0	-0.001	t	41608	MGPNLVLPDQKPPS	Mus musculus
1	1.00	1	1	0	0	0	0	-0.001	t	41571	MGFGLFD	Homo sapiens
1	1.00	1	1	0	0	0	0	-0.001	t	50502	QDPDNTDDNGPQDPDNTD	Mus musculus
1	1.00	1	1	0	0	0	0	-0.001	t	58416	SHSQLGPPVGGPP	Homo sapiens
1	1.00	1	1	0	0	0	0	-0.001	t	39092	LRGDLQVLAQKVARTL	Mus musculus
1	1.00	1	1	0	0	0	0	-0.001	t	56195	RTTPDRQAA	Oryctolagus cuniculus
1	1.00	1	1	0	-0.003	0	0	-0.001	t	39092	LRGDLQVLAQKVARTL	Oryctolagus cuniculus
1	1.00	1	1	0	0	0	0	-0.001	t	56807	SAHGTSTGVRGP	Mus musculus
1	1.00	1	1	0	0	0	0	-0.001	t	38522	LPHSNL	Homo sapiens
1	1.00	1	1	0	0	0	0	-0.001	t	56734	SADNNNSEYSWT	Mus musculus

1	1.00	1	1	0	-0.003	0	0	-0.001	t	40563	LVRMKRA	Oryctolagus cuniculus
1	1.00	1	1	0	0	0	0	-0.001	t	43317	NATAEGRAINRRVE	Mus musculus
1	1.00	1	1	0	0	0	0	-0.001	t	49455	PSPEAHVPPPYVEPTTTQCF	Homo sapiens
1	1.00	1	1	0	0	0	0	-0.001	t	40563	LVRMKRA	Mus musculus
1	1.00	1	1	0	0	0	0	-0.001	t	56397	RVRGLYLPAGGS	Mus musculus
1	1.00	1	1	0	0	0	0	-0.001	t	39808	LTANTKSMALSG	Oryctolagus cuniculus
1	1.00	1	1	0	0	0	0	-0.001	t	59387	SLPGQTKILAKVFYGEN	Homo sapiens
1	1.00	1	1	0	0	0	0	-0.001	t	54393	RKLWCHHFC	Capra hircus
1	1.00	1	1	0	0	0	0	-0.001	t	56022	RTCAHPGEH	Oryctolagus cuniculus
1	1.00	1	1	0	0	0	0	-0.001	t	57880	SFRGSAQGIEGS	Homo sapiens
1	1.00	1	1	0	0	0	0	-0.001	t	50930	QGPGAPQGPGAP	Mus musculus
1	1.00	1	1	0	0	0	0	-0.001	t	41232	MDIDPYKEFG	Mus musculus
1	1.00	1	1	0	0	0	0	-0.001	t	53195	RARGRGRGRGEKRP	Homo sapiens

1	1.00	1	1	0	0	0	0	-0.001	t	51258	QKTAASY	Homo sapiens
1	1.00	1	1	0	0	0	0	-0.001	t	56022	RTCAHPGEH	Mus musculus
1	1.00	1	1	0	0	0	0	-0.001	t	37372	LLGTTLL	Mus musculus
1	1.00	1	1	0	0	0	0	-0.001	t	58409	SHSAFNMFDFGVGPP	Homo sapiens
1	1.00	1	1	0	0.004	0	0	-0.001	t	49680	PTKEFFREA	Mus musculus
1	1.00	1	1	0	0	0	0	-0.001	t	43160	NAGVLTCSLSKR	Mus musculus
1	1.00	1	1	0	0	0	0	-0.001	t	55726	RRRSQSPRRRR	Mus musculus
1	1.00	1	1	0	0	0	0	-0.001	t	43191	NAKATYEAALKQYEADLAA	Mus musculus
1	1.00	1	1	0	0	0	0	-0.001	t	48876	PPPQAPYQGY	Mus musculus
1	1.00	1	1	0	-0.003	0	0	-0.001	t	45990	NSSSFNTTQGWHHPSQRLLF	Oryctolagus cuniculus
1	1.00	1	1	0	0	0	0	-0.001	t	57557	SEMPQLPSEMPQLP	Mus musculus
1	1.00	1	1	0	0	0	0	-0.001	t	38522	LPHSNL	Rattus norvegicus
1	1.00	1	1	0	0	0	0	-0.001	t	56937	SAPPTRVRR	Oryctolagus cuniculus
1	1.00	1	1	0	0.002	0	0	-0.001	t	38522	LPHSNL	Rattus norvegicus

1	1.00	1	1	0	0	0	0	-0.001	t	50930	QGPGAPQGPGAP	Mus musculus
1	1.00	1	1	0	0	0	0	-0.001	t	45990	NSSSFNTTQGWHHPSQRLLF	Mus musculus
1	1.00	1	1	0	0	0	0	-0.001	t	38522	LPHSNL	Oryctolagus cuniculus
1	1.00	1	1	0	0	0	0	-0.001	t	45990	NSSSFNTTQGWHHPSQRLLF	Oryctolagus cuniculus
1	1.00	1	1	0	0	0	0	-0.001	t	41571	MGFGLFD	Homo sapiens
1	1.00	1	1	0	0	0	0	-0.001	t	39788	LTAELKIYSVIQAEINKHL	Mus musculus
1	1.00	1	1	0	0	0	0	-0.001	t	57743	SFDVPSKQPTIDIDL	Oryctolagus cuniculus
1	1.00	1	1	0	0	0	0	-0.001	t	41571	MGFGLFD	Homo sapiens
1	1.00	1	1	0	0	0	0	-0.001	t	53166	RANDKGSVISLARLTEELKT	Mus musculus
1	1.00	1	1	0	0	0	0	-0.001	t	41169	MAVPAFSDIAKSANDLLNKD	Oryctolagus cuniculus
1	1.00	1	1	0	0	0	0	-0.001	t	46317	NTWTTCQSIAFPSK	Mus musculus
1	1.00	1	1	0	0	0	0	-0.001	t	48732	PPA	Mus musculus
1	1.00	1	1	0	0	0	0	-0.001	t	43317	NATAEGRAINRRVE	Mus musculus
1	1.00	1	1	0	0	0	0	-0.001	t	53878	RGGPDDLTAL	Homo sapiens
1	1.00	1	1	0	0	0	0	-0.001	t	57299	SDTRGDPVFNLPFQ	Mus musculus

1	1.00	1	1	0	0	0	0	-0.001	t	48933	PPTAPPLL PHSNL	Homo sapiens
1	1.00	1	1	0	0	0	0	-0.001	t	58422	SHSVTKSL RVFGGPP	Homo sapiens
1	1.00	1	1	0	0	0	0	-0.001	t	50929	QGPGAP	Mus musculu s
1	1.00	1	1	0	0	0	0	-0.001	t	43191	NAKATYEA ALKQYEAD LAA	Mus musculu s
1	1.00	1	1	0	0	0	0	-0.001	t	37660	LLPHSNLD HILEPSIPW KSK	Mus musculu s
1	1.00	1	1	0	0	0	0	-0.001	t	52016	QQEVDYII DHVDQHN	Oryctola gus cuniculu s
1	1.00	1	1	0	0	0	0	-0.001	t	56719	SAAYTAFG IANLFRVG	Homo sapiens
1	1.00	1	1	0	0	0	0	-0.001	t	57654	SETQKSQN Y	Mus musculu s
1	1.00	1	1	0	0	0	0	-0.001	t	43246	NANPNAN PNANPNA NP	Mus musculu s
1	1.00	1	1	0	0	0	0	-0.001	t	43246	NANPNAN PNANPNA NP	Mus musculu s
1	1.00	1	1	0	0	0	0	-0.001	t	43246	NANPNAN PNANPNA NP	Mus musculu s
1	1.00	1	1	0	0	0	0	-0.001	t	55073	RNTNRRP QDVKFPG GGQIVG	Homo sapiens
1	1.00	1	1	0	0	0	0	-0.001	t	58388	SHLVEALY LVCGERG	Mus musculu s
1	1.00	1	1	0	0	0	0	-0.001	t	58207	SGQARVLY SEFINAL	Mus musculu s

1	1.00	1	1	0	0	0	0	-0.001	t	54786	RLTENTEPLL	Homo sapiens
1	1.00	1	1	0	0	0	0	-0.001	t	50929	QGPGAP	Mus musculus
1	1.00	1	1	0	0	0	0	-0.001	t	55994	RSVSKEEPGM	Homo sapiens
1	1.00	1	1	0	0	0	0	-0.001	t	39024	LQYTPSWMLV	Mus musculus
1	1.00	1	1	0	0	0	0	-0.001	t	55799	RSAPRITFG	Homo sapiens
1	1.00	1	1	0	0	0	0	-0.001	t	41183	MCAVRDPAFSRSRLS	Mus musculus
1	1.00	1	1	0	0	0	0	-0.001	t	41537	MFSGGGGPLSPGGKS	Mus musculus
1	1.00	1	1	0	0	0	0	-0.001	t	57542	SELSQLS	Mus musculus
1	1.00	1	1	0	0	0	0	-0.001	t	57542	SELSQLS	Mus musculus
1	1.00	1	1	0	0	0	0	-0.001	t	58931	SKQPTIDIDLCDICT	Oryctolagus cuniculus
1	1.00	1	1	0	0	0	0	-0.001	t	50930	QGPGAPQGPGAP	Mus musculus
1	1.00	1	1	0	0	0	0	-0.001	t	51775	QNTRNIVPVSIVSR	Mus musculus
1	1.00	1	1	0	0	0	0	-0.001	t	56306	RVHVSKEEQYYDYEDATFE	Mus musculus
1	1.00	1	1	0	-0.003	0	0	-0.001	t	56306	RVHVSKEEQYYDYEDATFE	Oryctolagus cuniculus
1	1.00	1	1	0	0	0	0	-0.001	t	45390	NPLGLKRGIEKAVEK	Rattus norvegicus
1	1.00	1	1	0	0	0	0	-0.001	t	47986	PISLVNLSTASSAPPTRVRR	Mus musculus

1	1.00	1	1	0	0	0	0	-0.001	t	45571	NQITSALS TQ	Mus musculu s
1	1.00	1	1	0	0	0	0	-0.001	t	46551	NWNFDFG	Mus musculu s
1	1.00	1	1	0	0	0	0	-0.001	t	53014	QYNTGNF SDGFYPFI N	Gallus gallus
1	1.00	1	1	0	0	0	0	-0.001	t	49629	PTGDRHK QPLVAPAK QLL	Mus musculu s
1	1.00	1	1	0	-0.003	0	0	-0.001	t	47986	PISLVNLST ASSAPPTR VRR	Oryctola gus cuniculu s
1	1.00	1	1	0	0	0	0	-0.001	t	56699	SAAFYYRV	Mus musculu s
1	1.00	1	1	0	0	0	0	-0.001	t	43317	NATAEGR AINRRVE	Mus musculu s
1	1.00	1	1	0	0	0	0	-0.001	t	46550	NWNFD	Mus musculu s
1	1.00	1	1	0	0	0	0	-0.001	t	46795	PAFLGCSS RFSGSFSG VEP	Mus musculu s
1	1.00	1	1	0	0	0	0	-0.001	t	45667	NRDQGRK PTPPTPP	Oryctola gus cuniculu s
1	1.00	1	1	0	0	0	0	-0.001	t	46792	PAFGANS NNPDWDF NP	Mus musculu s
1	1.00	1	1	0	0	0	0	-0.001	t	46792	PAFGANS NNPDWDF NP	Homo sapiens
1	1.00	1	1	0	0	0	0	-0.001	t	47984	PISLLFNEK EADDNDH EPQI	Homo sapiens

1	1.00	1	1	0	0	0	0	-0.001	t	39070	LREGVTLG VGASFDT QKL	Oryctola gus cuniculu s
1	1.00	1	1	0	0	0	0	-0.001	t	44616	NKVIWDR DWMYP	Mus musculu s
1	1.00	1	1	0	-0.003	0	0	-0.001	t	53357	RDGQSVIG ACASPYEG RYR	Oryctola gus cuniculu s
1	1.00	1	1	0	0	0	0	-0.001	t	54553	RLGDTDLI AEVEHDY	Homo sapiens
1	1.00	1	1	0	0	0	0	-0.001	t	53357	RDGQSVIG ACASPYEG RYR	Mus musculu s
1	1.00	1	1	0	-0.004	0	0	-0.001	t	51233	QKPLK	Mus musculu s
1	1.00	1	1	0	0	0	0	-0.001	t	41870	MKNYLSF GMFALL	Mus musculu s
1	1.00	1	1	0	0.004	0	0	-0.001	t	51233	QKPLK	Mus musculu s
1	1.00	1	1	0	0	0	0	-0.001	t	37660	LLPHSNLD HILEPSIPW KSK	Homo sapiens
1	1.00	1	1	0	0	0	0	-0.001	t	48598	PNANPNA	Mus musculu s
1	1.00	1	1	0	0	0	0	-0.001	t	42698	MSTNPKP QRKTKRNT NRR	Homo sapiens
1	1.00	1	1	0	0	0	0	-0.001	t	59593	SLYELTH	Homo sapiens
1	1.00	1	1	0	0	0	0	-0.001	t	55260	RPPNAP	Mus musculu s

1	1.00	1	1	0	0	0	0	-0.001	t	51847	QPGVDIIEGPVKNVAVPLY	Homo sapiens
1	1.00	1	1	0	0	0	0	-0.001	t	39092	LRGDLQVLAQKVARTL	Mus musculus
1	1.00	1	1	0	0	0	0	-0.001	t	48331	PLGFFPDHQLDP	Homo sapiens
1	1.00	1	1	0	0	0	0	-0.001	t	59982	SPAPERRGYSGYDVPDY	Mus musculus
1	1.00	1	1	0	0	0	0	-0.001	t	46443	NVPEKQT	Mus musculus
1	1.00	1	1	0	0	0	0	-0.001	t	38304	LNPKTNKWEDK	Homo sapiens
1	1.00	1	1	0	0	0	0	-0.001	t	43481	NDLALPWKHEGAQNWNNAER	Mus musculus
1	1.00	1	1	0	0	0	0	-0.001	t	57062	SAYTPAYYTKDTNNNLTL	Homo sapiens
1	1.00	1	1	0	-0.002	0	0	-0.001	t	57062	SAYTPAYYTKDTNNNLTL	Mus musculus
1	1.00	1	1	0	0	0	0	-0.001	t	47289	PEGRTWAQ	Homo sapiens
1	1.00	1	1	0	0	0	0	-0.001	t	50508	QDPRVRGLYFPAGG	Mus musculus
1	1.00	1	1	0	0	0	0	-0.001	t	43576	NEDNEKLRKPKHKKLKQPG	Mus musculus
1	1.00	1	1	0	0	0	0	-0.001	t	55671	RRPFNIGINNQQ	Homo sapiens
1	1.00	1	1	0	0	0	0	-0.001	t	56701	SAAHTAFGIANLFRMG	Homo sapiens

1	1.00	1	1	0	0	0	0	-0.001	t	50508	QDPRVRG LYFPAGG	Mus musculu s
1	1.00	1	1	0	0	0	0	-0.001	t	47565	PFSPDGKP CTPP	Homo sapiens
1	1.00	1	1	0	0	0	0	-0.001	t	52124	QQQPP	Homo sapiens
1	1.00	1	1	0	0	0	0	-0.001	t	38810	LQAMRKY SPFRNGY MEPTLG	Homo sapiens
1	1.00	1	1	0	0	0	0	-0.001	t	48993	PQEQITQH GSPYGRCA NK	Mus musculu s
1	1.00	1	1	0	-0.003	0	0	-0.001	t	48993	PQEQITQH GSPYGRCA NK	Oryctola gus cuniculu s
1	1.00	1	1	0	0	0	0	-0.001	t	39169	LRPTFDTR LMRLEDE MKEGR	Mus musculu s
1	1.00	1	1	0	0	0	0	-0.001	t	37171	LLDPSFDY FQLYTT	Mus musculu s
1	1.00	1	1	0	0	0	0	-0.001	t	47201	PDSDPQIP PPYVEPTA PQVL	Homo sapiens
1	1.00	1	1	0	0	0	0	-0.001	t	38325	LNRADIL	Homo sapiens
1	1.00	1	1	0	0	0	0	-0.001	t	41375	MEILGIAIN RYNGLTLR G	Mus musculu s
1	1.00	1	1	0	0	0	0	-0.001	t	54058	RHKQKIVA PVKQTL	Ovis aries
1	1.00	1	1	0	0	0	0	-0.001	t	41375	MEILGIAIN RYNGLTLR G	Mus musculu s

1	1.00	1	1	0	0	0	0	-0.001	t	39167	LRPTFDTRLM	Mus musculus
1	1.00	1	1	0	-0.003	0	0	-0.001	t	54058	RHKQKIVAPVKQTL	Oryctolagus cuniculus
1	1.00	1	1	0	0	0	0	-0.001	t	55185	RPKHPIKHQG	Homo sapiens
1	1.00	1	1	0	0	0	0	-0.001	t	51371	QLINTNGSWHIN	Rattus norvegicus
1	1.00	1	1	0	0	0	0	-0.001	t	51371	QLINTNGSWHIN	Mus musculus
1	1.00	1	1	0	0	0	0	-0.001	t	42475	MRKTKLAPTVAHGVF	Bos taurus
1	1.00	1	1	0	0	0	0	-0.001	t	52420	QSRGEAR	Mus musculus
1	1.00	1	1	0	0	0	0	-0.001	t	58944	SKTDNLT	Homo sapiens
1	1.00	1	1	0	0	0	0	-0.001	t	51371	QLINTNGSWHIN	Mus musculus
1	1.00	1	1	0	0	0	0	-0.001	t	42475	MRKTKLAPTVAHGVF	Mus musculus
1	1.00	1	1	0	0	0	0	-0.001	t	56185	RTTADRQTA	Oryctolagus cuniculus
1	1.00	1	1	0	0	0	0	-0.001	t	38730	LPTLARSFIICIQMI	Homo sapiens
1	1.00	1	1	0	-0.003	0	0	-0.001	t	46960	PASTTNKDKL	Oryctolagus cuniculus
1	1.00	1	1	0	0	0	0	-0.001	t	36958	LKMADPNRFRGKD	Mus musculus

1	1.00	1	1	0	0	0	0	-0.001	t	46443	NVPEKQT	Mus musculus
1	1.00	1	1	0	0	0	0	-0.001	t	51371	QLINTNGSWHIN	Mus musculus
1	1.00	1	1	0	0	0	0	-0.001	t	36958	LKMADPNRFRGKD	Mus musculus
1	1.00	1	1	0	0	0	0	-0.001	t	39164	LRPGPVSQPYHHWDTDK	Homo sapiens
1	1.00	1	1	0	0	0	0	-0.001	t	58371	SHITSYPAYFW	Mus musculus
1	1.00	1	1	0	0	0	0	-0.001	t	48627	PNEPDDALAMRIIRI	Mus musculus
1	1.00	1	1	0	0	0	0	-0.001	t	46894	PAPQAPYQGY	Mus musculus
1	1.00	1	1	0	0	0	0	-0.001	t	55174	RPIDDFDQGWGPITY	Homo sapiens
1	1.00	1	1	0	0	0	0	-0.001	t	51371	QLINTNGSWHIN	Mus musculus
1	1.00	1	1	0	0	0	0	-0.001	t	53355	RDGQLTIKAERTEQKDFDGR	Mus musculus
1	1.00	1	1	0	0	0	0	-0.001	t	51371	QLINTNGSWHIN	Mus musculus
1	1.00	1	1	0	0	0	0	-0.001	t	42493	MRQLDTNVER	Homo sapiens
1	1.00	1	1	0	0	0	0	-0.001	t	50508	QDPRVRGLYFPAGG	Mus musculus
1	1.00	1	1	0	-0.002	0	0	-0.001	t	45190	NNNLTLVPAVVGKPGSDV	Mus musculus
1	1.00	1	1	0	0	0	0	-0.001	t	50508	QDPRVRGLYFPAGG	Mus musculus
1	1.00	1	1	0	0	0	0	-0.001	t	41204	MCQCVQKYGTEFCKKRLA	Mus musculus

1	1.00	1	1	0	0	0	0	-0.001	t	46859	PALTAVET GATNPL	Homo sapiens
1	1.00	1	1	0	0	0	0	-0.001	t	50508	QDPRVRG LYFPAGG	Mus musculu s
1	1.00	1	1	0	0	0	0	-0.001	t	50222	QACKGKIQ ALCEN	Mus musculu s
1	1.00	1	1	0	0	0	0	-0.001	t	37627	LLNQTARA FPDCA	Oryctola gus cuniculu s
1	1.00	1	1	0	0	0	0	-0.001	t	43242	NANPNAN PNANP	Mus musculu s
1	1.00	1	1	0	0	0	0	-0.001	t	43242	NANPNAN PNANP	Mus musculu s
1	1.00	1	1	0	0	0	0	-0.001	t	55641	RRLGRQTY DNES	Mus musculu s
1	1.00	1	1	0	0	0	0	-0.001	t	46443	NVPEKQT	Mus musculu s
1	1.00	1	1	0	0	0	0	-0.001	t	43242	NANPNAN PNANP	Mus musculu s
1	1.00	1	1	0	0	0	0	-0.001	t	43242	NANPNAN PNANP	Mus musculu s
1	1.00	1	1	0	0	0	0	-0.001	t	43242	NANPNAN PNANP	Mus musculu s
1	1.00	1	1	0	0	0	0	-0.001	t	43242	NANPNAN PNANP	Mus musculu s
1	1.00	1	1	0	0	0	0	-0.001	t	43242	NANPNAN PNANP	Oryctola gus cuniculu s
1	1.00	1	1	0	0	0	0	-0.001	t	43242	NANPNAN PNANP	Mus musculu s

1	1.00	1	1	0	0	0	0	-0.001	t	46443	NVPEKQT	Mus musculus
1	1.00	1	1	0	0	0	0	-0.001	t	47598	PFWVRGTTW	Homo sapiens
1	1.00	1	1	0	0	0	0	-0.001	t	52094	QQPP	Mus musculus
1	1.00	1	1	0	0	0	0	-0.001	t	45279	NNVDQI	Homo sapiens
1	1.00	1	1	0	0	0	0	-0.001	t	48906	PPRRVS	Mus musculus
1	1.00	1	1	0	0	0	0	-0.001	t	50930	QGPGAPQGPGAP	Mus musculus
1	1.00	1	1	0	0	0	0	-0.001	t	49534	PSTPGERVRH	Homo sapiens
1	1.00	1	1	0	0	0	0	-0.001	t	42475	MRKTKLAPTVAHGVF	Bos taurus
1	1.00	1	1	0	0	0	0	-0.001	t	43730	NEREDERTLTKEYEDIVLK	Oryctolagus cuniculus
1	1.00	1	1	0	0	0	0	-0.001	t	58388	SHLVEALYLVCGERG	Mus musculus
1	1.00	1	1	0	0	0	0	-0.001	t	56718	SAAYATSSFSSLFTRG	Homo sapiens
1	1.00	1	1	0	0	0	0	-0.001	t	52082	QQPFP	Mus musculus
1	1.00	1	1	0	0	0	0	-0.001	t	49629	PTGDRHKQPLVAPAKQLL	Mus musculus
1	1.00	1	1	0	0	0	0	-0.001	t	55389	RQAESSEAMAAWSERAGEA	Mus musculus

1	1.00	1	1	0	0	0	0	-0.001	t	21763	GPRLQPY	Mus musculus
1	1.00	1	1	0	0	0	0	-0.001	t	21763	GPRLQPY	Mus musculus
1	1.00	1	1	0	0	0	0	-0.001	t	11055	EAGKHSG	Homo sapiens
1	1.00	1	1	0	0	0	0	-0.001	t	10908	DYKDDDDK	Mus musculus
1	1.00	1	1	0	0	0	0	-0.001	t	15468	FDTGAFDPDWPA	Mus musculus
1	1.00	1	1	0	0.002	0	0	-0.001	t	21815	GPVEDAITAAIGRVA	Rattus norvegicus
1	1.00	1	1	0	0	0	0	-0.001	t	28351	IRNW	Mus musculus
1	1.00	1	1	0	0	0	0	-0.001	t	14000	ERHLTFT	Homo sapiens
1	1.00	1	1	0	0	0	0	-0.001	t	13961	ERAGAMERAN	Homo sapiens
1	1.00	1	1	0	0	0	0	-0.001	t	18363	FWRGDLVFDFQV	Homo sapiens
1	1.00	1	1	0	0	0	0	-0.001	t	31383	KIPDPPQPDFPQLNSDWVPS	Oryctolagus cuniculus
1	1.00	1	1	0	0	0	0	-0.001	t	11309	EDENTLQHAYPID	Mus musculus
1	1.00	1	1	0	-0.003	0	0	-0.001	t	31383	KIPDPPQPDFPQLNSDWVPS	Oryctolagus cuniculus
1	1.00	1	1	0	0	0	0	-0.001	t	31885	KLGKFGAARV	Homo sapiens
1	1.00	1	1	0	0	0	0	-0.001	t	25146	HWYISKPQ	Mus musculus

1	1.00	1	1	0	0	0	0	-0.001	t	31383	KIPDPPQPDFPQLNSDWVPS	Mus musculus
1	1.00	1	1	0	0	0	0	-0.001	t	24037	HIDSQKKAIERMK	Oryctolagus cuniculus
1	1.00	1	1	0	0	0	0	-0.001	t	23599	HCLGKWLGHPDKF	Mus musculus
1	1.00	1	1	0	0	0	0	-0.001	t	20667	GKQPASD	Homo sapiens
1	1.00	1	1	0	0	0	0	-0.001	t	11208	EAVEAERAGRGTG	Mus musculus
1	1.00	1	1	0	0	0	0	-0.001	t	30780	KFLVNGRWID	Homo sapiens
1	1.00	1	1	0	0	0	0	-0.001	t	11208	EAVEAERAGRGTG	Mus musculus
1	1.00	1	1	0	0.002	0	0	-0.001	t	21816	GPVEESVERAMVRVA	Rattus norvegicus
1	1.00	1	1	0	0	0	0	-0.001	t	13624	EPDKSII	Homo sapiens
1	1.00	1	1	0	0	0	0	-0.001	t	20064	GGVATY	Macaca
1	1.00	1	1	0	0	0	0	-0.001	t	25140	HWSYGLRPG	Rattus norvegicus
1	1.00	1	1	0	0	0	0	-0.001	t	15430	FDMATNP	Homo sapiens
1	1.00	1	1	0	0	0	0	-0.001	t	29812	KAGSTVYAVTAVTSIFSVG	Homo sapiens
1	1.00	1	1	0	0	0	0	-0.001	t	13623	EPDKMTT	Homo sapiens
1	1.00	1	1	0	0	0	0	-0.001	t	36931	LKLAT	Mus musculus

1	1.00	1	1	0	0	0	0	-0.001	t	36729	LITNTIAGV	Mus musculus
1	1.00	1	1	0	0	0	0	-0.001	t	32841	KPPTPPPEPET	Mus musculus
1	1.00	1	1	0	0	0	0	-0.001	t	14844	EVPLGVAADANK	Oryctolagus cuniculus
1	1.00	1	1	0	0	0	0	-0.001	t	12345	EHKYSWKS	Mus musculus
1	1.00	1	1	0	0	0	0	-0.001	t	21823	GPVLRR	Mus musculus
1	1.00	1	1	0	0	0	0	-0.001	t	12632	EKASVPGGGDMGGMDF	Mus musculus
1	1.00	1	1	0	0	0	0	-0.001	t	30093	KCTSDQDEQFIPKGCSK	Mus musculus
1	1.00	1	1	0	0	0	0	-0.001	t	27910	IPKVPPGPNITA	Homo sapiens
1	1.00	1	1	0	0	0	0	-0.001	t	30093	KCTSDQDEQFIPKGCSK	Mus musculus
1	1.00	1	1	0	0	0	0	-0.001	t	15417	FDKVK	Mus musculus
1	1.00	1	1	0	0	0	0	-0.001	t	23616	HDEGRQIIQFEE	Mus musculus
1	1.00	1	1	0	0	0	0	-0.001	t	16123	FHLPYNHNWFAL	Mus musculus
1	1.00	1	1	0	0	0	0	-0.001	t	36931	LKLAT	Mus musculus
1	1.00	1	1	0	0	0	0	-0.001	t	36931	LKLAT	Mus musculus
1	1.00	1	1	0	0	0	0	-0.001	t	15417	FDKVK	Mus musculus

1	1.00	1	1	0	0	0	0	-0.001	t	11081	EAIPALTAVETGHTSQV	Oryctolagus cuniculus
1	1.00	1	1	0	0	0	0	-0.001	t	36931	LKLAT	Mus musculus
1	1.00	1	1	0	0	0	0	-0.001	t	34498	KYLEEPV	Homo sapiens
1	1.00	1	1	0	0	0	0	-0.001	t	28537	ISIKYDPRKDSEVFA	Mus musculus
1	1.00	1	1	0	0	0	0	-0.001	t	28537	ISIKYDPRKDSEVFA	Mus musculus
1	1.00	1	1	0	0	0	0	-0.001	t	28075	IPSTAFTDIAWVRLPNHY	Homo sapiens
1	1.00	1	1	0	0	0	0	-0.001	t	12307	EGVQQEGAQQPA	Mus musculus
1	1.00	1	1	0	0	0	0	-0.001	t	18177	FVGLFTP	Homo sapiens
1	1.00	1	1	0	0	0	0	-0.001	t	12542	EIPDPVEAGEEV	Mus musculus
1	1.00	1	1	0	0	0	0	-0.001	t	11744	EELFKEYK	Mus musculus
1	1.00	1	1	0	0	0	0	-0.001	t	25587	IDIDLCDICTNTMDV	Oryctolagus cuniculus
1	1.00	1	1	0	-0.002	0	0	-0.001	t	12375	EHWSYGLRPG	Mus musculus
1	1.00	1	1	0	0	0	0	-0.001	t	35063	LCKIARSQDACIEFV	Oryctolagus cuniculus
1	1.00	1	1	0	0	0	0	-0.001	t	11081	EAIPALTAVETGHTSQV	Oryctolagus cuniculus
1	1.00	1	1	0	0	0	0	-0.001	t	23068	GVNLEDPASRDL	Homo sapiens

1	1.00	1	1	0	0	0	0	-0.001	t	28804	ITADMYT	Homo sapiens
1	1.00	1	1	0	0	0	0	-0.001	t	21723	GPPAAGPPAAGPPAA	Homo sapiens
1	1.00	1	1	0	0	0	0	-0.001	t	24168	HKYSWK	Mus musculus
1	1.00	1	1	0	-0.003	0	0	-0.001	t	11815	EENVEENVEENVEENV	Oryctolagus cuniculus
1	1.00	1	1	0	0	0	0	-0.001	t	16396	FKLEKEVAE	Mus musculus
1	1.00	1	1	0	0	0	0	-0.001	t	22230	GRVCLR	Mus musculus
1	1.00	1	1	0	-0.004	0	0	-0.001	t	13095	ELIHVLHGLYGMQVSS	Oryctolagus cuniculus
1	1.00	1	1	0	0	0	0	-0.001	t	32769	KPKHKKLKQPGDGNP	Mus musculus
1	1.00	1	1	0	0	0	0	-0.001	t	13572	ENPVVHFFKNIVTPR	Mus musculus
1	1.00	1	1	0	0	0	0	-0.001	t	22312	GSENKRTGALGNLKN	Mus musculus
1	1.00	1	1	0	-0.006	0	0	-0.001	t	12375	EHWSYGLRPG	Canis lupus
1	1.00	1	1	0	-0.003	0	0	-0.001	t	23975	HHAEMQNPVYLIPETVPYIK	Oryctolagus cuniculus
1	1.00	1	1	0	0	0	0	-0.001	t	16397	FKLKAIE	Homo sapiens
1	1.00	1	1	0	0	0	0	-0.001	t	20097	GGYGDGGAHGGGYGG	Homo sapiens
1	1.00	1	1	0	0	0	0	-0.001	t	30200	KDLLEQKRAAVDTYC	Oryctolagus cuniculus

1	1.00	1	1	0	0	0	0	-0.001	t	11377	EDLDGA	Mus musculus
1	1.00	1	1	0	0	0	0	-0.001	t	11571	EEEDDDMGFGLFD	Mus musculus
1	1.00	1	1	0	0	0	0	-0.001	t	11571	EEEDDDMGFGLFD	Mus musculus
1	1.00	1	1	0	0	0	0	-0.001	t	13481	ENHSPVNIAHKL	Mus musculus
1	1.00	1	1	0	0	0	0	-0.001	t	23975	HHAEMQNPVYLIPETVPYIK	Mus musculus
1	1.00	1	1	0	0	0	0	-0.001	t	21293	GMLATVAFNTFTDNVDQR	Mus musculus
1	1.00	1	1	0	0	0	0	-0.001	t	30093	KCTSDQDEQFIPKGCSK	Mus musculus
1	1.00	1	1	0	0	0	0	-0.001	t	34645	KYYGNGVTC	Oryctolagus cuniculus
1	1.00	1	1	0	0	0	0	-0.001	t	21254	GMDGTQLDRW	Mus musculus
1	1.00	1	1	0	0	0	0	-0.001	t	15981	FGGETFTPDWMMEV	Mus musculus
1	1.00	1	1	0	0	0	0	-0.001	t	11571	EEEDDDMGFGLFD	Mus musculus
1	1.00	1	1	0	0	0	0	-0.001	t	35484	LEGGSRGAPGGGFVPNLQ	Homo sapiens
1	1.00	1	1	0	0	0	0	-0.001	t	11571	EEEDDDMGFGLFD	Homo sapiens
1	1.00	1	1	0	0	0	0	-0.001	t	11571	EEEDDDMGFGLFD	Homo sapiens
1	1.00	1	1	0	0	0	0	-0.001	t	23069	GVNLEDPASRDLVVS	Oryctolagus cuniculus

1	1.00	1	1	0	0	0	0	-0.001	t	13743	EPRCPYVG ADHFDCP	Oryctolagus cuniculus
1	1.00	1	1	0	0	0	0	-0.001	t	11571	EEEDDDM GFGLFD	Mus musculus
1	1.00	1	1	0	0	0	0	-0.001	t	11571	EEEDDDM GFGLFD	Homo sapiens
1	1.00	1	1	0	0	0	0	-0.001	t	11571	EEEDDDM GFGLFD	Homo sapiens
1	1.00	1	1	0	0	0	0	-0.001	t	23032	GVLAKDV RFSQV	Homo sapiens
1	1.00	1	1	0	0	0	0	-0.001	t	23685	HEGKAYFP REGV	Homo sapiens
1	1.00	1	1	0	0	0	0	-0.001	t	12982	ELDGSKNE WGWSKSK	Mus musculus
1	1.00	1	1	0	0	0	0	-0.001	t	23694	HEIAWFTE RSDKSYEH QT	Mus musculus
1	1.00	1	1	0	0	0	0	-0.001	t	33294	KSDNNVT	Homo sapiens
1	1.00	1	1	0	0	0	0	-0.001	t	11411	EDPASR	Mus musculus
1	1.00	1	1	0	0	0	0	-0.001	t	21625	GPFYTSAP Q	Oryctolagus cuniculus
1	1.00	1	1	0	0	0	0	-0.001	t	12856	EKQT	Mus musculus
1	1.00	1	1	0	0.002	0	0	-0.001	t	21807	GPTEESVE RAMGRVA	Rattus norvegicus
1	1.00	1	1	0	0	0	0	-0.001	t	32159	KLSEGDLL AEIETDK	Homo sapiens
1	1.00	1	1	0	0	0	0	-0.001	t	19067	GDPEDL	Mus musculus

1	1.00	1	1	0	0	0	0	-0.001	t	11872	EESDDDM GFGLFD	Homo sapiens
1	1.00	1	1	0	0	0	0	-0.001	t	34165	KVPPWAR TA	Mus musculu s
1	1.00	1	1	0	0	0	0	-0.001	t	11872	EESDDDM GFGLFD	Homo sapiens
1	1.00	1	1	0	0	0	0	-0.001	t	36940	LKLKSIVS WAKKVL	Oryctola gus cuniculu s
1	1.00	1	1	0	0	0	0	-0.001	t	24315	HLVEKGY	Homo sapiens
1	1.00	1	1	0	0	0	0	-0.001	t	11872	EESDDDM GFGLFD	Homo sapiens
1	1.00	1	1	0	0	0	0	-0.001	t	36353	LGWLWG	Mus musculu s
1	1.00	1	1	0	0	0	0	-0.001	t	14223	ESNPVDG AHLSL	Mus musculu s
1	1.00	1	1	0	0	0	0	-0.001	t	30262	KDTNNN	Mus musculu s
1	1.00	1	1	0	0	0	0	-0.001	t	16101	FHENWPS	Mus musculu s
1	1.00	1	1	0	0	0	0	-0.001	t	26844	IKLPALF	Homo sapiens
1	1.00	1	1	0	0	0	0	-0.001	t	11872	EESDDDM GFGLFD	Homo sapiens
1	1.00	1	1	0	0.004	0	0	-0.001	t	25807	IEFQQKNN RLLEITREF	Mus musculu s
1	1.00	1	1	0	0	0	0	-0.001	t	11872	EESDDDM GFGLFD	Homo sapiens
1	1.00	1	1	0	0	0	0	-0.001	t	15982	FGGETFTP DWMMEV AIDNE	Mus musculu s
1	1.00	1	1	0	0	0	0	-0.001	t	26829	IKKMLAD QTVEEHIG	Oryctola gus cuniculu s

1	1.00	1	1	0	0	0	0	-0.001	t	32548	KNLEEPK	Homo sapiens
1	1.00	1	1	0	0	0	0	-0.001	t	13524	ENMTPQD	Mus musculus
1	1.00	1	1	0	0	0	0	-0.001	t	28676	ISQAVHAAHAEINEAGR	Mus musculus
1	1.00	1	1	0	0	0	0	-0.001	t	25807	IEFQQKNNRLLEITREF	Mus musculus
1	1.00	1	1	0	0	0	0	-0.001	t	24445	HPEYAVSVLL	Homo sapiens
1	1.00	1	1	0	0	0	0	-0.001	t	14273	ESSPNTQWELRAFMA	Mus musculus
1	1.00	1	1	0	0	0	0	-0.001	t	15468	FDTGAFDPDWPA	Mus musculus
1	1.00	1	1	0	0	0	0	-0.001	t	18441	FYNQKFKGKATL	Mus musculus
1	1.00	1	1	0	0	0	0	-0.001	t	31002	KGLNGLFDLGPKQKI	Mus musculus
1	1.00	1	1	0	0	0	0	-0.001	t	30661	KEVPALTAVETGAT	Oryctolagus cuniculus
1	1.00	1	1	0	0	0	0	-0.001	t	32159	KLSEGDLLAEIETDK	Homo sapiens
1	1.00	1	1	0	0	0	0	-0.001	t	34779	LAGHGAFQITSLFTRG	Homo sapiens
1	1.00	1	1	0	0	0	0	-0.001	t	26317	IGRGNFGEVFSG	Mus musculus
1	1.00	1	1	0	0	0	0	-0.001	t	30661	KEVPALTAVETGAT	Oryctolagus cuniculus
1	1.00	1	1	0	0	0	0	-0.001	t	20586	GKIGMYP	Homo sapiens

1	1.00	1	1	0	0	0	0	-0.001	t	22360	GSGVRGDFGSLAPRVARQL	Cavia porcellus
1	1.00	1	1	0	0	0	0	-0.001	t	22360	GSGVRGDFGSLAPRVARQL	Cavia porcellus
1	1.00	1	1	0	0	0	0	-0.001	t	33151	KRHFLSQRQ	Mus musculus
1	1.00	1	1	0	0	0	0	-0.001	t	23947	HGRVGIYFGMK	Mus musculus
1	1.00	1	1	0	0	0	0	-0.001	t	4331	ASARGDLAHLTTTHARHLP	Mus musculus
1	1.00	1	1	0	0	0	0	-0.001	t	9044	DLDGAA	Mus musculus
1	1.00	1	1	0	0	0	0	-0.001	t	7674	DAVTELDKN	Mus musculus
1	1.00	1	1	0	0	0	0	-0.001	t	9722	DPPPPNPNDPPPPNPN	Mus musculus
1	1.00	1	1	0	0	0	0	-0.001	t	934	AEGEFPQDARFPGGGDPAK	Homo sapiens
1	1.00	1	1	0	0	0	0	-0.001	t	1373	AFPL	Mus musculus
1	1.00	1	1	0	0	0	0	-0.001	t	4330	ASARGDLAHLTTT	Mus musculus
1	1.00	1	1	0	0	0	0	-0.001	t	9611	DPDKDVDIMV	Mus musculus
1	1.00	1	1	0	0	0	0	-0.001	t	7756	DDDMGFGLFD	Homo sapiens
1	1.00	1	1	0	0	0	0	-0.001	t	7673	DAVTE	Mus musculus

1	1.00	1	1	0	0	0	0	-0.001	t	9722	DPPPPNPNDPPPPNPN	Mus musculus
1	1.00	1	1	0	0	0	0	-0.001	t	7209	CTSRFASLFNLGPQQQ	Mus musculus
1	1.00	1	1	0	0	0	0	-0.001	t	6501	CKLSQGAPDSC	Mus musculus
1	1.00	1	1	0	0	0	0	-0.001	t	1746	AHAYSGYDVPDY	Mus musculus
1	1.00	1	1	0	0	0	0	-0.001	t	1022	AEKQKAAEATKVAE	Homo sapiens
1	1.00	1	1	0	0	0	0	-0.001	t	4071	AQSRNVRTFTGLFTSG	Homo sapiens
1	1.00	1	1	0	0	0	0	-0.001	t	6501	CKLSQGAPDSC	Mus musculus
1	1.00	1	1	0	0	0	0	-0.001	t	921	AEGEFCSPPDSPGVCGDPAK	Mus musculus
1	1.00	1	1	0	0	0	0	-0.001	t	4331	ASARGDLAHLTTTHARHLP	Mus musculus
1	1.00	1	1	0	0	0	0	-0.001	t	9722	DPPPPNPNDPPPPNPN	Mus musculus
1	1.00	1	1	0	0	0	0	-0.001	t	6139	CDSSDRPRVYTIT	Mus musculus
1	1.00	1	1	0	0	0	0	-0.001	t	9586	DPAFR	Mus musculus
1	1.00	1	1	0	0	0	0	-0.001	t	247	AAKGTPW	Homo sapiens
1	1.00	1	1	0	0	0	0	-0.001	t	5994	CAKSPRYTLD	Oryctolagus cuniculus
1	1.00	1	1	0	0	0	0	-0.001	t	7756	DDDMGFGLFD	Homo sapiens
1	1.00	1	1	0	0	0	0	-0.001	t	4149	ARGSLAVTI	Mus musculus

1	1.00	1	1	0	0	0	0	-0.001	t	1254	AFAQAPTHQLSL	Mus musculus
1	1.00	1	1	0	0	0	0	-0.001	t	5559	AVTFSKG	Homo sapiens
1	1.00	1	1	0	0	0	0	-0.001	t	5563	AVTKGTINDPQAAKEALDKY	Oryctolagus cuniculus
1	1.00	1	1	0	0	0	0	-0.001	t	6343	CGRVCLRC	Mus musculus
1	1.00	1	1	0	0	0	0	-0.001	t	9524	DNPASTTNKDK	Mus musculus
1	1.00	1	1	0	0	0	0	-0.001	t	939	AEGEFRLGVRALRKAPDPAK	Homo sapiens
1	1.00	1	1	0	0	0	0	-0.001	t	2780	ALNGNGDPNNMDKAVKLY	Mus musculus
1	1.00	1	1	0	0	0	0	-0.001	t	918	AEGEFATAPPSHYSWDPAK	Homo sapiens
1	1.00	1	1	0	-0.003	0	0	-0.001	t	7851	DDPPATVYRYDSRPPEDV	Oryctolagus cuniculus
1	1.00	1	1	0	0	0	0	-0.001	t	757	ADQTVEEHIGYLVKY	Oryctolagus cuniculus
1	1.00	1	1	0	0	0	0	-0.001	t	7187	CTNGLTALFTPGAKQN	Mus musculus
1	1.00	1	1	0	0	0	0	-0.001	t	3933	AQAANGGASGQVKVTKVT	Homo sapiens
1	1.00	1	1	0	0	0	0	-0.001	t	793	ADVTITVNGKVVAKPC	Oryctolagus cuniculus
1	1.00	1	1	0	0	0	0	-0.001	t	4941	ATEKSNVVRGWV	Homo sapiens
1	1.00	1	1	0	0	0	0	-0.001	t	1406	AFTPDWMMEVAIDNE	Mus musculus

1	1.00	1	1	0	0	0	0	-0.001	t	6632	CLTFGRET VIEY	Mus musculu s
1	1.00	1	1	0	0	0	0	-0.001	t	8133	DEQFIPK	Mus musculu s
1	1.00	1	1	0	0	0	0	-0.001	t	9723	DPPPPNP NDPPPPN PND	Mus musculu s
1	1.00	1	1	0	0	0	0	-0.001	t	9723	DPPPPNP NDPPPPN PND	Mus musculu s
1	1.00	1	1	0	0	0	0	-0.001	t	7491	DAEFRHDS GYEVHHQ K	Mus musculu s
1	1.00	1	1	0	0	0	0	-0.001	t	8756	DIPNPIGSE NSEKTTM PLW	Homo sapiens
1	1.00	1	1	0	0	0	0	-0.001	t	7491	DAEFRHDS GYEVHHQ K	Mus musculu s
1	1.00	1	1	0	0	0	0	-0.001	t	2273	AKREAIVK ADEDDNE ET	Mus musculu s
1	1.00	1	1	0	-0.003	0	0	-0.001	t	5967	CADARMY GVLPWNA FPGKVC	Oryctola gus cuniculu s
1	1.00	1	1	0	0	0	0	-0.001	t	5147	ATSRSTM QLASFFTP G	Homo sapiens
1	1.00	1	1	0	0	0	0	-0.001	t	7491	DAEFRHDS GYEVHHQ K	Mus musculu s
1	1.00	1	1	0	0	0	0	-0.001	t	7491	DAEFRHDS GYEVHHQ K	Mus musculu s
1	1.00	1	1	0	0	0	0	-0.001	t	4710	ASQKRPSQ RHGSKY	Rattus norvegic us
1	1.00	1	1	0	0	0	0	-0.001	t	9134	DLIQEGNI GLMKAV	Oryctola gus cuniculu s
1	1.00	1	1	0	0	0	0	-0.001	t	7491	DAEFRHDS GYEVHHQ K	Mus musculu s

1	1.00	1	1	0	0	0	0	-0.001	t	7491	DAEFRHDSGYEVHHQK	Homo sapiens
1	1.00	1	1	0	0	0	0	-0.001	t	7756	DDDMGFGLFD	Homo sapiens
1	1.00	1	1	0	-0.004	0	0	-0.001	t	9595	DPALLLMHELIHVLHGLYG	Oryctolagus cuniculus
1	1.00	1	1	0	0	0	0	-0.001	t	610	ACYLPFQLSCGGKS	Mus musculus
1	1.00	1	1	0	0	0	0	-0.001	t	6020	CAQQWPEWYPC	Mus musculus
1	1.00	1	1	0	0	0	0	-0.001	t	10227	DSSAHGTPSTPA	Oryctolagus cuniculus
1	1.00	1	1	0	0	0	0	-0.001	t	6632	CLTFGRETVIEY	Homo sapiens
1	1.00	1	1	0	0	0	0	-0.001	t	9613	DPDKDVDIMVRDGQLTIKAE	Mus musculus
1	1.00	1	1	0	0	0	0	-0.001	t	6210	CEYNVFHNKTFELPRA	Mus musculus
1	1.00	1	1	0	0	0	0	-0.001	t	6226	CFGNSEY	Mus musculus
1	1.00	1	1	0	0	0	0	-0.001	t	3938	AQAHTTRGLTNLFSSG	Homo sapiens
1	1.00	1	1	0	0	0	0	-0.001	t	9232	DLPDYGDYGEDLFDY	Mus musculus
1	1.00	1	1	0	0	0	0	-0.001	t	1536	AGGSGRP	Mus musculus
1	1.00	1	1	0	0	0	0	-0.001	t	3245	ANDVDNSNPVVQAEQLNWL	Homo sapiens

1	1.00	1	1	0	0	0	0	-0.001	t	6412	CIEFVQQEVDYIIDH	Oryctolagus cuniculus
1	1.00	1	1	0	0	0	0	-0.001	t	7756	DDDMGFGLFD	Homo sapiens
1	1.00	1	1	0	0	0	0	-0.001	t	5558	AVTFASFDVPSKQPT	Oryctolagus cuniculus
1	1.00	1	1	0	-0.003	0	0	-0.001	t	7851	DDPPATVYRYDSRPPEDV	Oryctolagus cuniculus
1	1.00	1	1	0	0	0	0	-0.001	t	4429	ASGQ	Mus musculus
1	1.00	1	1	0	0	0	0	-0.001	t	9278	DLSLLGKARKVVVTKD	Rattus norvegicus
1	1.00	1	1	0	0	0	0	-0.001	t	8544	DHILEPSIPWKSKLLTLVQL	Homo sapiens
1	1.00	1	1	0	0	0	0	-0.001	t	3517	APDTRPAP	Mus musculus
1	1.00	1	1	0	0	0	0	-0.001	t	1157	AESEE	Homo sapiens
1	1.00	1	1	0	0	0	0	-0.001	t	6276	CGAVIDDC	Mus musculus
1	1.00	1	1	0	0	0	0	-0.001	t	6883	CQIYPPNANKIREALAQ	Oryctolagus cuniculus
1	1.00	1	1	0	0	0	0	-0.001	t	3516	APDTRP	Mus musculus
1	1.00	1	1	0	0	0	0	-0.001	t	6249	CFPYCYPSESA	Mus musculus
1	1.00	1	1	0	0	0	0	-0.001	t	8552	DHLPSPW	Homo sapiens

1	1.00	1	1	0	0	0	0	-0.001	t	6524	CKTC	Mus musculus
1	1.00	1	1	0	0	0	0	-0.001	t	9524	DNPASTTNKDK	Mus musculus
1	1.00	1	1	0	0	0	0	-0.001	t	868	AEEAFDLWNECAKACV	Homo sapiens
1	1.00	1	1	0	0	0	0	-0.001	t	1433	AGAGGGAGGAGAGGGAGGAG	Oryctolagus cuniculus
1	1.00	1	1	0	0	0	0	-0.001	t	1497	AGEPYVIEQGMMDPAK	Homo sapiens
1	1.00	1	1	0	0	0	0	-0.001	t	3514	APDTR	Mus musculus
1	1.00	1	1	0.03	0.028	0.028	0.029	0.03	t	26317	IGRGNFGEVFSG	Mus musculus
1	1.00	1	1	0.03	0.027	0.03	0.03	0.03	t	133843	GTKAVTKYTSSK	Oryctolagus cuniculus
1	1.00	1	1	0.01	0.01	0.01	0.01	0.01	t	142413	EIAAEVAKAKVAK	Homo sapiens
1	1.00	1	1	0.02	0.02	0.02	0.02	0.019	t	108498	KIHAREIFDSRGNPTV	Homo sapiens
1	1.00	1	1	0.01	0.006	0.006	0.01	0.008	t	42583	MSKKSGKWWESDDKFAKAVY	Oryctolagus cuniculus
1	1.00	1	1	0.03	0.028	0.031	0.029	0.028	t	68053	VDRAFSFEGIYVYSS	Mus musculus
1	1.00	1	1	-0.04	-0.04	-0.045	-0.042	-0.04	t	53067	RAAIRAS	Homo sapiens
1	1.00	1	1	-0.03	-0.029	-0.027	-0.029	-0.03	t	50304	QALGALK	Homo sapiens

1	1.00	1	1	0.03	0.03	0.03	0.03	0.03	t	142436	KVAATSLE RWQQ	Mus musculu s
1	1.00	1	1	0.03	0.03	0.03	0.03	0.03	t	142424	GDEFDTYD DIKKVT	Homo sapiens
1	1.00	1	1	0.03	0.03	0.03	0.03	0.03	t	158602	PLELSVY	Homo sapiens
1	1.00	1	1	0.01	0.01	0.01	0.01	0.01	t	149107	SLAPRFPW LNPLDGW	Rattus norvegic us
1	1.00	1	1	0.01	0.01	0.01	0.01	0.01	t	78298	WHHMYV P	Homo sapiens
1	1.00	1	1	0.01	0.01	0.01	0.01	0.01	t	45434	NPNPDPN	Mus musculu s
1	1.00	1	1	-0.01	-0.01	-0.01	-0.01	-0.01	t	113774	QQGQQP GQGQPGY Y	Homo sapiens
1	1.00	1	1	-0.01	-0.01	-0.01	-0.01	-0.01	t	106026	QARQLKN NNPFKFFV	Homo sapiens
1	1.00	1	1	-0.01	-0.01	-0.01	-0.01	-0.01	t	107650	TYRVVSV	Homo sapiens
1	1.00	1	1	-0.01	-0.01	-0.01	-0.01	-0.01	t	79386	SCSPTVYLT STKCP	Mus musculu s
1	1.00	1	1	-0.01	-0.01	-0.01	-0.01	-0.01	t	79293	LLDYGWL WMW	Mus musculu s
1	1.00	1	1	0.04	0.04	0.034	0.04	0.04	t	72816	WLTYHGAI KLDDKDP QF	Homo sapiens
1	1.00	1	1	0.01	0.012	-0.004	0.007	0.01	t	346	AAPANDK FTVFEAAF NDAIK	Homo sapiens
1	1.00	1	1	-0.04	-0.04	-0.048	-0.04	-0.04	t	125454	KDYKTMV KFCN	Mus musculu s
1	1.00	1	1	0	0	0.005	0	0	t	130474	ECLLLSKYC MPS	Mus musculu s
1	1.00	1	1	-0.04	-0.042	-0.05	-0.039	-0.039	t	42790	MTDVSRKI RAWGRRL MIGTA	Homo sapiens

1	1.00	1	1	-0.04	-0.04	-0.052	-0.04	-0.038	t	121023	VATKGLNEHG	Mus musculus
1	1.00	1	1	0	0.002	0.007	0.003	0	t	29752	KAALIEEGQRMAEM	Homo sapiens
1	1.00	1	1	0	0.002	0.007	0.003	0	t	29752	KAALIEEGQRMAEM	Homo sapiens
1	1.00	1	1	0.01	0.012	0.013	0.007	0.007	t	39985	LTKFTTQKQVSSLFQ	Homo sapiens
1	1.00	1	1	0.03	0.028	0.006	0.033	0.037	t	20521	GKAVTKEQL	Mus musculus
1	1.00	1	1	0	-0.001	0	0	0	t	41258	MDPELRGLAQ	Mus musculus
1	1.00	1	1	0.01	0.013	0.018	0.01	0.008	t	60013	SPDDPAFRFAPYFTP	Mus musculus
1	1.00	1	1	-0.01	0.002	-0.005	-0.01	-0.01	t	19491	GFADLMGYIP	Pan troglodytes
1	1.00	1	1	0.02	0.018	0.02	0.019	0.02	t	110275	GPVGPSDPSIVSLVEETSFI	Mus musculus
1	1.00	1	1	-0.01	-0.012	-0.013	-0.009	-0.01	t	138935	YDQREPQAWCRPNENQSWT	Homo sapiens
1	1.00	1	1	0.02	0.02	0.02	0.02	0.02	t	105505	SAE	Oryctolagus cuniculus
1	1.00	1	1	0.02	0.02	0.02	0.02	0.02	t	105505	SAE	Oryctolagus cuniculus
1	1.00	1	1	0.02	0.02	0.02	0.02	0.02	t	105505	SAE	Oryctolagus cuniculus
1	1.00	1	1	0.02	0.02	0.02	0.02	0.02	t	105505	SAE	Oryctolagus cuniculus

1	1.00	1	1	0	-0.003	0	0	0	t	48992	PQEQITQHGSPYGRC	Oryctolagus cuniculus
1	1.00	1	1	0	0	0	0	0	t	48992	PQEQITQHGSPYGRC	Oryctolagus cuniculus
1	1.00	1	1	-0.05	-0.047	-0.046	-0.05	-0.051	t	22756	GTLVRMAPVIG	Mus musculus
1	1.00	1	1	0.04	0.038	0.047	0.043	0.04	t	106690	NSDHFLQPDPPL	Mus musculus
1	1.00	1	1	-0.03	-0.03	-0.02	-0.024	-0.03	t	76368	YVNTNMGLKIRQ	Homo sapiens
1	1.00	1	1	0	-0.003	-0.004	0	0.002	t	12626	EKAFRELADA	Oryctolagus cuniculus
1	1.00	1	1	0.01	0.014	0.014	0.01	0.01	t	134335	CTKPSDGNCT	Homo sapiens
1	1.00	1	1	0.01	0.014	0.014	0.01	0.01	t	134335	CTKPSDGNCT	Homo sapiens
1	1.00	1	1	0.01	0.014	0.014	0.01	0.01	t	134335	CTKPSDGNCT	Homo sapiens
1	1.00	1	1	0.01	0.01	0.009	0.013	0.01	t	71431	VTNNTP	Homo sapiens
1	1.00	1	1	0.01	0.006	0.007	0.009	0.01	t	57257	SDPDTSFLEGLDLGVDVRTY	Oryctolagus cuniculus
1	1.00	1	1	0.02	0.02	0.015	0.02	0.02	t	136087	VETDKATVGF	Oryctolagus cuniculus
1	1.00	1	1	0.02	0.022	0.015	0.02	0.02	t	136087	VETDKATVGF	Rattus norvegicus

1	1.00	1	1	0	-0.003	0	0	0.002	t	140894	QRRADAVKGYLA	Oryctolagus cuniculus
1	1.00	1	1	-0.01	-0.01	-0.005	-0.01	-0.01	t	66105	TRTFTSL	Homo sapiens
1	1.00	1	1	0.01	0.01	0.014	0.01	0.01	t	37301	LLFTGH	Homo sapiens
1	1.00	1	1	0.01	0.01	0.01	0.011	0.01	t	107008	YEFRHSS	Mus musculus
1	1.00	1	1	0	0	0	0	0.002	t	121713	PLTALLRN	Homo sapiens
1	1.00	1	1	0.02	0.02	0.02	0.02	0.02	t	149061	INPTQTT	Sus scrofa
1	1.00	1	1	0.02	0.02	0.02	0.02	0.02	t	135742	QKSCEPAV	Homo sapiens
1	1.00	1	1	0.02	0.02	0.02	0.02	0.02	t	156339	KDDPTNKFYQSVIQ	Homo sapiens
1	1.00	1	1	0.02	0.02	0.02	0.02	0.02	t	156958	SLHSSVQ	Gallus gallus
1	1.00	1	1	0.02	0.02	0.02	0.02	0.02	t	132929	WGENNYM	Homo sapiens
1	1.00	1	1	0.02	0.02	0.02	0.02	0.02	t	127680	HFHRPPT	Homo sapiens
1	1.00	1	1	0	0	0	0	0	t	133950	WIFANF	Mus musculus
1	1.00	1	1	-0.02	-0.02	-0.02	-0.02	-0.02	t	89581	TLVNADANYL	Homo sapiens
1	1.00	1	1	-0.04	-0.04	-0.04	-0.04	-0.04	t	135674	FNDFTRVV	Homo sapiens
1	1.00	1	1	-0.02	-0.018	-0.021	-0.023	-0.023	t	113876	SPQQSGQGQPGYYP	Homo sapiens
1	1.00	1	1	0.01	0.01	0.01	0.01	0.009	t	24870	HSVRGFTSLFS	Homo sapiens
1	1.00	1	1	0.01	0.013	0.015	0.01	0.01	t	49967	PVSLGGDVPNS	Sus scrofa
1	1.00	1	1	0	0	-0.007	0	0.002	t	8469	DGPQKSVSQGGVSLDLYSKL	Mus musculus

1	1.00	1	1	0.01	0.012	-0.002	0.011	0.012	t	141933	KIENAAMAIHLCFNN	Homo sapiens
1	1.00	1	1	0.02	0.02	0.015	0.02	0.018	t	23985	HHFQSHGQLSDHPHALSSSS	Homo sapiens
1	1.00	1	1	-0.02	-0.02	-0.022	-0.02	-0.02	t	97075	VSCYGKTKCTASNKNR	Oryctolagus cuniculus
1	1.00	1	1	0.02	0.022	0.02	0.023	0.02	t	104443	DAEFRHDSGYE	Homo sapiens
1	1.00	1	1	-0.01	-0.008	-0.005	-0.007	-0.012	t	25309	IAHQFKEKVLGLLQRATQQQ	Homo sapiens
1	1.00	1	1	0.01	0.013	-0.007	0.01	0.012	t	7852	DDPPKDGNKDDLPKEEKKDD	Mus musculus
1	1.00	1	1	-0.01	-0.006	0.001	-0.003	-0.008	t	122071	AALGSAI	Homo sapiens
1	1.00	1	1	0	0	-0.002	-0.006	-0.002	t	13661	EPIAPYHFDLSGH	Homo sapiens
1	1.00	1	1	0.02	0.018	0.02	0.013	0.02	t	117614	QGFPFYDKPMRI	Mus musculus
1	1.00	1	1	0.01	0.012	0.01	0.011	0.01	t	66240	TSGTSGTSGPSGPSG	Homo sapiens
1	1.00	1	1	-0.03	-0.031	-0.038	-0.03	-0.028	t	46801	PAGAGRRV	Anas platyrhynchos
1	1.00	1	1	-0.01	-0.01	-0.01	-0.011	-0.009	t	153288	SDPRKMCIY	Mus musculus
1	1.00	1	1	0.03	0.03	0.021	0.03	0.033	t	110733	VGENVPDDLYIKGSG	Mus musculus
1	1.00	1	1	0.01	0.014	0.016	0.011	0.01	t	12048	EFLVNT	Homo sapiens

1	1.00	1	1	0.06	0.058	0.057	0.059	0.06	t	98093	HGFTEQNSG	Mus musculus
1	1.00	1	1	-0.01	-0.01	-0.01	-0.007	-0.01	t	49193	PRGRRQPIPKARRPE	Homo sapiens
1	1.00	1	1	0	0.002	0.006	0.001	0	t	9951	DRASLST	Homo sapiens
1	1.00	1	1	0	0.004	0.006	0.001	0	t	9951	DRASLST	Homo sapiens
1	1.00	1	1	0.03	0.03	0.028	0.03	0.03	t	117605	PNHPSSSGSFSDADLAD	Mus musculus
1	1.00	1	1	0.03	0.03	0.03	0.03	0.03	t	153336	ACNQVLSWLCGGGS	Mus musculus
1	1.00	1	1	0.01	0.01	0.01	0.01	0.01	t	123405	KWLGDKFNM	Ovis aries
1	1.00	1	1	0.01	0.01	0.01	0.01	0.01	t	124274	HPLAPAGTF	Homo sapiens
1	1.00	1	1	0.01	0.01	0.01	0.01	0.01	t	117053	PHPSTMFDRQED	Homo sapiens
1	1.00	1	1	-0.01	-0.01	-0.01	-0.01	-0.01	t	110615	GPRGPPGPQGATGPL	Mus musculus
1	1.00	1	1	-0.03	-0.03	-0.03	-0.03	-0.03	t	92667	FEKVKHNLVI	Ovis aries
1	1.00	1	1	0	0	-0.009	0	0	t	29965	KATYEAAVAANNAKNAALT	Mus musculus
1	1.00	1	1	0.01	0.008	-0.004	0.009	0.01	t	4013	AQNFRNIMHGSDSFFYTFTS	Mus musculus
1	1.00	1	1	0	-0.006	0	0.003	0.001	t	6925	CRGFTSFKKPPTPPPEPET	Papio

1	1.00	1	1	-0.02	-0.02	-0.02	-0.02	-0.02	t	6712	CNNPHRIL	Oryctolagus cuniculus
1	1.00	1	1	-0.03	-0.034	-0.038	-0.031	-0.029	t	66901	TTVQQQFADAWKPSPVMTVR	Oryctolagus cuniculus
1	1.00	1	1	0.07	0.07	0.072	0.076	0.07	t	25497	ICESAGVQEDAASL	Homo sapiens
1	1.00	1	1	0.02	0.02	0.021	0.02	0.022	t	68041	VDPQ	Mus musculus
1	1.00	1	1	-0.04	-0.044	-0.043	-0.041	-0.042	t	75659	YRPLGPGTPPMRARLPA	Oryctolagus cuniculus
1	1.00	1	1	0.02	0.022	0.015	0.021	0.02	t	36264	LGPLYLIQATITTT	Homo sapiens
1	1.00	1	1	0	0	0	0.002	0	t	119505	NSTMGY	Homo sapiens
1	1.00	1	1	0	0	0	0.002	0	t	119546	WTAN	Homo sapiens
1	1.00	1	1	0	0	0	0.002	0	t	119475	KKIK	Homo sapiens
1	1.00	1	1	0	0	0	0.002	0	t	119473	KILKVIRK	Homo sapiens
1	1.00	1	1	0	0	0	0.002	0	t	119478	KNDK	Homo sapiens
1	1.00	1	1	0	0	0	0.002	0	t	119484	LDKK	Homo sapiens
1	1.00	1	1	0	0	0	0.002	0	t	66312	TSLNLQKDEPNGRASDTAGQ	Equus caballus
1	1.00	1	1	0	0.007	0	0.002	0	t	38273	LNLTTWNPTLLG	Ovis aries
1	1.00	1	1	0	0.007	0	0.002	0	t	51474	QLPNVGITQGIV	Ovis aries
1	1.00	1	1	0.03	0.031	0.032	0.029	0.03	t	28548	ISITTEVMPVSMAKTSV	Oryctolagus cuniculus
1	1.00	1	1	-0.07	-0.07	-0.068	-0.067	-0.069	t	99009	SNALSKRL	Homo sapiens

1	1.00	1	1	-0.01	-0.008	-0.002	-0.003	-0.012	t	113101	NPVMERFAAHAGDLV	Homo sapiens
1	1.00	1	1	0.02	0.02	0.02	0.017	0.022	t	60358	SQAAPYIEQ	Homo sapiens
1	1.00	1	1	0	0	0	0.002	0	t	10832	DWVCSILTDFKNWL	Homo sapiens
1	1.00	1	1	0.04	0.037	0.038	0.04	0.04	t	35662	LEQPVSNDLS	Oryctolagus cuniculus
1	1.00	1	1	0.04	0.037	0.038	0.04	0.04	t	35662	LEQPVSNDLS	Oryctolagus cuniculus
1	1.00	1	1	-0.03	-0.03	-0.035	-0.024	-0.03	t	11700	EEITQVATISANSDHNIGKL	Homo sapiens
1	1.00	1	1	0.01	0.01	0.009	0.01	0.01	t	73636	YEDRYYRE	Mus musculus
1	1.00	1	1	0.03	0.026	0.032	0.03	0.029	t	94521	GDLQVLAQK	Oryctolagus cuniculus
1	1.00	1	1	-0.02	-0.02	-0.027	-0.014	-0.018	t	106990	VYATRSSAVRLRSSVP	Homo sapiens
1	1.00	1	1	0	0	0	0	0	t	9982	DRHFLN	Homo sapiens
1	1.00	1	1	-0.02	-0.02	-0.02	-0.02	-0.02	t	132765	YRAYA	Mus musculus
1	1.00	1	1	0	-0.001	-0.003	0.001	0.002	t	12134	EGCGWAGWLL	Homo sapiens
1	1.00	1	1	0	0	-0.008	0	0.002	t	48672	PNLSAYPKSHGKTAK	Oryctolagus cuniculus
1	1.00	1	1	0.02	0.022	0.02	0.021	0.02	t	66241	TSGTSGTSGTSGTSG	Homo sapiens
1	1.00	1	1	0.04	0.04	0.04	0.04	0.04	t	119506	PIVET	Homo sapiens

1	1.00	1	1	-0.02	-0.022	-0.02	-0.021	-0.021	t	70472	VPSDTVQTRHVV	Mus musculus
1	1.00	1	1	-0.03	-0.032	-0.03	-0.033	-0.032	t	110385	QLYKTCKQAGTCPPDIIPKV	Mus musculus
1	1.00	1	1	-0.03	-0.028	-0.029	-0.033	-0.03	t	70522	VPYVKRVKTW	Homo sapiens
1	1.00	1	1	0	-0.004	-0.005	-0.001	0	t	78657	FVHAKKKEEGEDDTARQEI	Oryctolagus cuniculus
1	1.00	1	1	0	-0.002	-0.005	-0.001	0	t	78657	FVHAKKKEEGEDDTARQEI	Mus musculus
1	1.00	1	1	0	0.004	0	0.007	0	t	128016	HLLTARATNSQLFRRAVLMG	Homo sapiens
1	1.00	1	1	0.01	0.012	0.015	0.013	0.01	t	48555	PMHECLSAPSVCADNY	Homo sapiens
1	1.00	1	1	-0.03	-0.022	-0.03	-0.03	-0.032	t	156850	APHNKWPHLNPP	Gallus gallus
1	1.00	1	1	0.04	0.04	0.04	0.04	0.04	t	79462	VTSTYH	Mus musculus
1	1.00	1	1	0.02	0.02	0.02	0.02	0.02	t	149054	GSPNFTQ	Sus scrofa
1	1.00	1	1	0.02	0.02	0.02	0.02	0.02	t	119602	KGQPREPQ	Homo sapiens
1	1.00	1	1	0.02	0.02	0.02	0.02	0.02	t	39391	LSGQPAVIPDREVLYQQFDE	Homo sapiens
1	1.00	1	1	0	0	0	0	0	t	134761	PRRRSPSPTPTPGPS	Homo sapiens
1	1.00	1	1	0	0	0	0	0	t	153423	MPKDMLPYANPNGGGS	Mus musculus
1	1.00	1	1	0	0	0	0	0	t	115904	PTVMFPPQSV	Homo sapiens
1	1.00	1	1	-0.02	-0.02	-0.02	-0.02	-0.02	t	114785	ECLLCAHKVE	Homo sapiens

1	1.00	1	1	0.03	0.027	0.041	0.03	0.028	t	111927	VSTAVDTG VLGTSYP	Oryctolagus cuniculus
1	1.00	1	1	-0.01	-0.006	-0.007	-0.013	-0.013	t	116695	DRRDPYSP SPYDRRG	Homo sapiens
1	1.00	1	1	0.02	0.038	0.02	0.019	0.02	t	111238	EGEAAQV EEVRIY	Oryctolagus cuniculus
1	1.00	1	1	0.01	0.012	0.003	0.009	0.01	t	133787	CQALVRM LAKKPGW K	Homo sapiens
1	1.00	1	1	0.02	0.022	0.018	0.02	0.017	t	131990	NSPLRIGG GWGNSD A	Homo sapiens
1	1.00	1	1	0	0	0	0	0	t	86742	NGVWVM MDGNEQ VEYPLK	Oryctolagus cuniculus
1	1.00	1	1	-0.02	-0.02	-0.022	-0.02	-0.022	t	608	ACYCYGLP NWVKVW D	Mus musculus
1	1.00	1	1	-0.08	-0.077	-0.09	-0.08	-0.081	t	111232	EDTYRFVT SQAIACQ	Mus musculus
1	1.00	1	1	-0.04	-0.04	-0.035	-0.03	-0.037	t	115784	HRTFE	Homo sapiens
1	1.00	1	1	0	0	0.01	0	-0.003	t	131836	CAPGSCFP GV	Mus musculus
1	1.00	1	1	-0.01	-0.006	-0.011	-0.009	-0.01	t	6742	CNYSKFW YLEHAK	Homo sapiens
1	1.00	1	1	0.02	0.017	0.02	0.02	0.02	t	53555	RELADALL EK	Oryctolagus cuniculus
1	1.00	1	1	0	-0.004	-0.005	-0.007	-0.002	t	130536	LCENIAGH LK	Oryctolagus cuniculus
1	1.00	1	1	0	-0.002	-0.005	-0.007	-0.002	t	130536	LCENIAGH LK	Rattus norvegicus

1	1.00	1	1	0.03	0.028	0.025	0.027	0.03	t	32535	KNKLEEKA NKAMININ	Mus musculu s
1	1.00	1	1	-0.01	-0.007	-0.031	-0.01	-0.007	t	79775	DDATVAIR SAINNLIVE LIR	Mus musculu s
1	1.00	1	1	-0.03	-0.03	-0.03	-0.03	-0.03	t	117074	RSLLSGGL P	Mus musculu s
1	1.00	1	1	0	0	0	0.001	0	t	10868	DYEDRYYR E	Mus musculu s
1	1.00	1	1	-0.02	-0.022	-0.02	-0.019	-0.021	t	72551	WHRPPW WPSSSL	Oryctola gus cuniculu s
1	1.00	1	1	0	0.002	-0.007	0.007	0	t	128144	WGPVIDG HFLREPPA RALKR	Homo sapiens
1	1.00	1	1	0	0	0.007	0	0	t	119463	HKKSLSSP S	Homo sapiens
1	1.00	1	1	-0.03	-0.032	-0.033	-0.033	-0.03	t	75661	YRPPNAPI L	Mus musculu s
1	1.00	1	1	0.01	0.017	0.012	0.01	0.011	t	95521	KQTLPPSV	Cavia porcellu s
1	1.00	1	1	-0.01	-0.01	-0.01	-0.01	-0.01	t	44095	NGTYRATY	Oryctola gus cuniculu s
1	1.00	1	1	0.01	0.01	0.01	0.01	0.01	t	123522	MTQAPRI M	Oryctola gus cuniculu s
1	1.00	1	1	0.03	0.033	0.031	0.031	0.028	t	99416	LFGILSVDC TSEEMN	Equus caballus
1	1.00	1	1	0.01	0.01	0.01	0.01	0.01	t	151071	YAADLKST QNAIDEI	Homo sapiens
1	1.00	1	1	0.01	0.01	0.01	0.01	0.01	t	133558	EKVTSTWT DWQY	Homo sapiens
1	1.00	1	1	0.01	0.01	0.01	0.01	0.01	t	108297	CKYLWSKC	Mus musculu s

1	1.00	1	1	0.01	0.01	0.01	0.01	0.01	t	104745	CSTWDANKPY	Homo sapiens
1	1.00	1	1	0.01	0.01	0.01	0.01	0.01	t	110586	GAPGNRGFPGQDGLA	Mus musculus
1	1.00	1	1	0.01	0.01	0.01	0.01	0.01	t	108297	CKYLWSKC	Homo sapiens
1	1.00	1	1	0.01	0.01	0.01	0.01	0.01	t	101921	NGGCDADAT	Homo sapiens
1	1.00	1	1	-0.01	-0.01	-0.01	-0.01	-0.01	t	96566	LKIVSPMVVK	Homo sapiens(human)
1	1.00	1	1	0	0.002	-0.01	-0.003	-0.002	t	56675	RYVKQEVQRGGR	Homo sapiens
1	1.00	1	1	-0.01	-0.012	-0.004	-0.004	-0.009	t	27728	INQQLNPKTNKWEDK	Mus musculus
1	1.00	1	1	-0.02	-0.024	-0.02	-0.017	-0.02	t	95354	ELSEFIPGASLR	Oryctolagus cuniculus
1	1.00	1	1	-0.01	-0.01	-0.005	0	-0.007	t	116024	VTLRI	Homo sapiens
1	1.00	1	1	-0.02	-0.023	-0.019	-0.02	-0.021	t	10431	DTNKDKPSYQ	Oryctolagus cuniculus
1	1.00	1	1	-0.02	-0.02	-0.02	-0.02	-0.02	t	117300	GSGKGKVQFQGKKTKF	Homo sapiens
1	1.00	1	1	-0.04	-0.028	-0.037	-0.04	-0.04	t	24696	HRMAWNMMMNWSPT	Pan troglodytes
1	1.00	1	1	0	-0.003	0	0	0.001	t	135440	AFPAVT	Oryctolagus cuniculus
1	1.00	1	1	-0.01	-0.01	-0.01	-0.01	-0.011	t	119382	VISCAKDAIEDEEGSC	Oryctolagus cuniculus

1	1.00	1	1	0.02	0.02	0.02	0.02	0.02	t	132400	KGKAGKD GGAGQ	Mus musculu s
1	1.00	1	1	0	0	0	0	0	t	127989	GGAGAGG GAGAGG	Homo sapiens
1	1.00	1	1	0.02	0.022	0.018	0.021	0.019	t	53923	RGLTSLFS AFSAQ	Homo sapiens
1	1.00	1	1	-0.01	-0.01	-0.005	-0.007	-0.01	t	65519	TPCILSGSA S	Homo sapiens
1	1.00	1	1	0	0	-0.002	0	0	t	70153	VNPNNAA AL	Homo sapiens
1	1.00	1	1	0	0.002	0	0.001	0	t	22145	GRLVLSGK PAIIPDREV LYR	Homo sapiens
1	1.00	1	1	0	0	0	-0.001	0	t	106441	GDLKRTT	Mus musculu s
1	1.00	1	1	0	0	0	0	0	t	50931	QGPGAPQ GPGAPQG PGAP	Mus musculu s
1	1.00	1	1	0	0	0	0	0	t	52814	QVVYDYQ H	Oryctola gus cuniculu s
1	1.00	1	1	0	0	0	0	0	t	36546	LIHFGND	Mus musculu s
1	1.00	1	1	0.01	0.01	0.01	0.01	0.009	t	130924	KLFPEEDE MRTETQR	Homo sapiens
1	1.00	1	1	0.01	0.01	0.005	0.01	0.01	t	44870	NLSGKAV NHIRS	Homo sapiens
1	1.00	1	1	0	-0.004	0	-0.001	0	t	137964	RFKEFGSV	Oryctola gus cuniculu s
1	1.00	1	1	0.01	0.012	-0.004	0.011	0.01	t	44634	NKYLKVQL KKGETVEN TNS	Homo sapiens
1	1.00	1	1	0.01	0.01	0.01	0.007	0.01	t	21016	GLLQRATQ QQ	Homo sapiens
1	1.00	1	1	0	0	0	0.004	0	t	115645	RNGKDQD GDQAP	Homo sapiens

1	1.00	1	1	-0.01	-0.01	-0.01	-0.01	-0.01	t	75949	YTASARGD LAHL	Mus musculu s
1	1.00	1	1	-0.01	-0.01	-0.01	-0.01	-0.01	t	75949	YTASARGD LAHL	Mus musculu s
1	1.00	1	1	0.04	0.04	0.04	0.04	0.04	t	135702	KLTRAEAV	Homo sapiens
1	1.00	1	1	0.02	0.02	0.02	0.02	0.02	t	115626	LKDAIGEG KVTL	Homo sapiens
1	1.00	1	1	0.02	0.02	0.02	0.02	0.02	t	103426	MTLLELSD FVKKFEE	Homo sapiens
1	1.00	1	1	0.02	0.02	0.02	0.02	0.02	t	115626	LKDAIGEG KVTL	Homo sapiens
1	1.00	1	1	0	0	0	0	0	t	142391	DAANIAH QKWLKAT	Homo sapiens
1	1.00	1	1	0	0	0	0	0	t	138249	TFRVVSV	Homo sapiens
1	1.00	1	1	0	0	0	0	0	t	140878	LAGKGVD AGKLG	Oryctola gus cuniculu s
1	1.00	1	1	0	0	0	0	0	t	127738	MHIPTPD	Homo sapiens
1	1.00	1	1	0	0	0	0	0	t	131823	ALVRYSSK EEVVKAQ	Homo sapiens
1	1.00	1	1	-0.02	-0.02	-0.02	-0.02	-0.02	t	99975	EEKELLKRI Q	Mus musculu s
1	1.00	1	1	-0.02	-0.02	-0.03	-0.02	-0.019	t	104165	PAANTRK GYHQTMN NSYTE	Oryctola gus cuniculu s
1	1.00	1	1	0	0	-0.006	0	0.001	t	158836	IHNDVEA WIDRYKYL P	Mus musculu s
1	1.00	1	1	0	-0.003	0.003	0	-0.003	t	111123	ARTYNQN LIRHVGGG	Oryctola gus cuniculu s

1	1.00	1	1	-0.02	-0.016	-0.023	-0.019	-0.021	t	103462	PDRSAERCGSPAWDL	Homo sapiens
1	1.00	1	1	-0.01	-0.01	-0.01	-0.008	-0.01	t	63540	TESTLSTALAELAT	Homo sapiens
1	1.00	1	1	-0.01	-0.01	-0.01	-0.008	-0.01	t	62924	TALVVAQLLRIPQAI	Homo sapiens
1	1.00	1	1	-0.01	-0.01	-0.026	-0.008	-0.007	t	98807	GSFRPSQQNPQA	Homo sapiens
1	1.00	1	1	0.05	0.05	0.05	0.044	0.048	t	21102	GLRGEEMEEMVQS	Homo sapiens
1	1.00	1	1	-0.04	-0.04	-0.04	-0.04	-0.043	t	129296	LPYGDSMDRIEKDRLQ	Homo sapiens
1	1.00	1	1	-0.01	-0.013	-0.013	-0.01	-0.008	t	67168	TVSSQSFR	Oryctolagus cuniculus
1	1.00	1	1	-0.01	-0.01	-0.01	-0.01	-0.01	t	72910	WNSTTFHQTL	Mus musculus
1	1.00	1	1	-0.01	-0.01	-0.01	-0.01	-0.01	t	72910	WNSTTFHQTL	Mus musculus
1	1.00	1	1	-0.01	-0.01	-0.01	-0.01	-0.01	t	72910	WNSTTFHQTL	Mus musculus
1	1.00	1	1	-0.01	-0.01	-0.01	-0.01	-0.01	t	72910	WNSTTFHQTL	Mus musculus
1	1.00	1	1	0.02	0.022	0.02	0.023	0.019	t	108989	FRCQVQFYGLSENDEW	Homo sapiens
1	1.00	1	1	-0.06	-0.06	-0.06	-0.06	-0.06	t	107124	GAPGLRGGAG	Mus musculus
1	1.00	1	1	-0.03	-0.03	-0.038	-0.031	-0.03	t	87165	NYKNGGFFVQYG	Homo sapiens
1	1.00	1	1	0.01	0.008	0.01	0.011	0.01	t	149425	QGFFQPSQQN	Mus musculus
1	1.00	1	1	0.02	0.02	0.025	0.02	0.018	t	50235	QAELDDKYAGKGYKLGSK	Mus musculus

1	1.00	1	1	0.04	0.026	0.039	0.04	0.04	t	63818	TGFADPNT TTG	Oryctola gus cuniculu s
1	1.00	1	1	0	-0.002	-0.001	-0.001	0	t	72251	WAPSV	Mus musculu s
1	1.00	1	1	0	0	0	0	0	t	36278	LGQGSFRP SQQN	Mus musculu s
1	1.00	1	1	0	0	-0.005	0	0	t	19093	GDRAAGQ PAGDRAA GQPA	Mus musculu s
1	1.00	1	1	0	0	-0.005	0	0	t	19093	GDRAAGQ PAGDRAA GQPA	Mus musculu s
1	1.00	1	1	0.03	0.03	0.03	0.03	0.03	t	103359	LILTSYTSD EAMLDA	Homo sapiens
1	1.00	1	1	0.05	0.05	0.057	0.05	0.05	t	63571	TEVEDDRE PSVPSEY	Homo sapiens
1	1.00	1	1	0.01	0.01	0.01	0.01	0.01	t	149073	MTSSETQ	Sus scrofa
1	1.00	1	1	0.01	0.01	0.01	0.01	0.01	t	137965	RFLPKYEG NIDI	Homo sapiens
1	1.00	1	1	0.01	0.01	0.01	0.01	0.01	t	132170	ASKAPAN GGNIR	Mus musculu s
1	1.00	1	1	0.01	0.01	0.01	0.01	0.01	t	118047	PGIMAPPP GM	Mus musculu s
1	1.00	1	1	0.01	0.01	0.01	0.01	0.01	t	112395	VYFCHQY	Homo sapiens
1	1.00	1	1	0.01	0.01	0.01	0.01	0.01	t	115907	PVVVPPFL QP	Homo sapiens
1	1.00	1	1	0.01	0.01	0.01	0.01	0.01	t	112218	LTCSFSG	Homo sapiens
1	1.00	1	1	0.01	0.01	0.01	0.01	0.01	t	114845	GTDGVTYT ND	Homo sapiens
1	1.00	1	1	0.01	0.01	0.01	0.01	0.01	t	113193	TGEIKALA GCDFLTI	Homo sapiens
1	1.00	1	1	0.01	0.006	0.01	0.01	0.01	t	9478	DNIHVKM FKVIMNN DKSELI	Aotus sp.
1	1.00	1	1	-0.01	-0.01	-0.01	-0.01	-0.01	t	114784	ECKETVP MNC	Homo sapiens

1	1.00	1	1	-0.05	-0.05	-0.05	-0.05	-0.05	t	110795	CDLIPNLKLNNG	Homo sapiens
1	1.00	1	1	0	0.003	0	0	0	t	73606	YDWRADW	Mus musculus
1	1.00	1	1	0.02	0.02	0.012	0.02	0.02	t	88944	SNSHNSQTEVAA	Homo sapiens
1	1.00	1	1	0.01	0.012	0.012	0.011	0.01	t	32971	KQELDEISTNIRQAG	Homo sapiens
1	1.00	1	1	0.02	0.008	0.02	0.02	0.02	t	108790	AQGPRGEP	Macaca mulatta
1	1.00	1	1	0.09	0.092	0.091	0.088	0.088	t	106879	STSTSSSSSG	Homo sapiens
1	1.00	1	1	-0.01	-0.01	-0.014	-0.01	-0.011	t	122659	YTSLHGYFVFGPTGCNLEGF	Rattus norvegicus
1	1.00	1	1	0	0.004	0	0.001	0	t	24712	HRVMYASS	Homo sapiens
1	1.00	1	1	0	0.004	0	0.001	0	t	35006	LAVLLGAC	Homo sapiens
1	1.00	1	1	0.06	0.06	0.058	0.06	0.058	t	95563	LNTVNGTTRTVN	Homo sapiens
1	1.00	1	1	-0.03	-0.03	-0.023	-0.03	-0.03	t	119493	LSRKKTLTT	Homo sapiens
1	1.00	1	1	0.05	0.05	0.052	0.05	0.05	t	97650	SLLTEVET	Mus musculus
1	1.00	1	1	0	0	0	0	0	t	48463	PLQKTYNY	Homo sapiens
1	1.00	1	1	-0.01	-0.01	-0.01	-0.01	-0.011	t	153378	EIDLKLEPGTRV	Mus musculus

1	1.00	1	1	0	0.003	-0.008	0	-0.002	t	72485	WGNGCGL FGKGSIDT CAKFA	Equus caballus
1	1.00	1	1	0	0	-0.006	0	0	t	55675	RRPKHLYV SMPTAQ	Homo sapiens
1	1.00	1	1	0	0.003	0	0	-0.002	t	135458	FGSDYEDR YYR	Mus musculu s
1	1.00	1	1	0.06	0.064	0.054	0.06	0.062	t	92285	AFNGGDG VDT	Ovis aries
1	1.00	1	1	0.01	0.008	0.011	0.009	0.01	t	79166	CEGNLACL S	Mus musculu s
1	1.00	1	1	0	0	0	0	0	t	70667	VQWMNR LIAFAFAG NHVSP	Homo sapiens
1	1.00	1	1	0.01	0.012	0.012	0.011	0.009	t	61420	SSTTSKVP PSPGGE	Homo sapiens
1	1.00	1	1	0.03	0.026	0.038	0.03	0.028	t	140593	PYQFFTAS E	Mus musculu s
1	1.00	1	1	0.03	0.033	0.03	0.03	0.029	t	133737	VTPYFSYP VA	Mus musculu s
1	1.00	1	1	0.06	0.046	0.058	0.06	0.061	t	55924	RSQDACIE FVQQEVD	Oryctola gus cuniculu s
1	1.00	1	1	0.02	0.02	0.02	0.02	0.02	t	133955	WIMNNF	Mus musculu s
1	1.00	1	1	0.02	0.02	0.02	0.02	0.02	t	79409	TCCLCSGK LALACE	Mus musculu s
1	1.00	1	1	0.02	0.02	0.02	0.02	0.02	t	95158	TNVLVNSS GHCQYLPP GIFK	Rattus norvegic us
1	1.00	1	1	0.02	0.02	0.02	0.02	0.02	t	22083	GRIILSGKP AVVPDRE VLYQ	Homo sapiens

1	1.00	1	1	0	0	0	0	0	t	123580	PGMPPMTQ	Oryctolagus cuniculus
1	1.00	1	1	0	0	0	0	0	t	92808	GGKGDDIFVH	Ovis aries
1	1.00	1	1	-0.02	-0.02	-0.02	-0.02	-0.02	t	112165	IGVGWIR	Homo sapiens
1	1.00	1	1	-0.04	-0.04	-0.04	-0.04	-0.04	t	100174	KDALLHEHYV	Mus musculus
1	1.00	1	1	-0.01	-0.012	-0.012	-0.01	-0.011	t	59353	SLNFLGGTTVCLGQN	Oryctolagus cuniculus
1	1.00	1	1	-0.02	-0.02	-0.02	-0.02	-0.02	t	95119	MKKPGENIKPLSCKYSYPKH	Rattus norvegicus
1	1.00	1	1	0.02	0.014	0.013	0.017	0.02	t	78213	MVLVYYESIAYSF	Oryctolagus cuniculus
1	1.00	1	1	0	0	0	0	-0.001	t	135441	AGLGAGIP	Mus musculus
1	1.00	1	1	0	0	0	0	-0.001	t	135477	LGAGLG	Mus musculus
1	1.00	1	1	0	0	0	0	-0.001	t	158723	DTGCAIDISRQELRCG	Mus musculus
1	1.00	1	1	0	0	0	0	-0.001	t	158938	KTQNQGPWDEGRVEID	Mus musculus
1	1.00	1	1	0	0	0	0	-0.001	t	139632	VKPLPSPDTD	Mus musculus
1	1.00	1	1	0	0	0	0	-0.001	t	159043	NTFVVDGPETKECPTQ	Mus musculus
1	1.00	1	1	0	0	0	0	-0.001	t	149684	EPAGSNR	Mus musculus
1	1.00	1	1	0	0	0	0	-0.001	t	159287	KTQGHSYHLRRKNGVKHPVY	Aotus
1	1.00	1	1	0	0	0	0	-0.001	t	144678	GTQPTLEVVPMGRGS	Mus musculus

1	1.00	1	1	0	0	0	0	-0.001	t	141693	KTWG	Mus musculus
1	1.00	1	1	0	0	0	0	-0.001	t	141955	LTYLRGISTKVTVGNIV	Mus musculus
1	1.00	1	1	0	0	0	0	-0.001	t	144656	GPTHAMFDSSGP	Mus musculus
1	1.00	1	1	0	0	0	0	-0.001	t	138628	SQKEVLERTRAELDTVC	Oryctolagus cuniculus
1	1.00	1	1	0	0	0	0	-0.001	t	144793	SYGGRKGTHPGLEMP	Mus musculus
1	1.00	1	1	0	0	0	0	-0.001	t	148701	PAELMRRFELYF	Mus musculus
1	1.00	1	1	0	-0.003	0	0	-0.001	t	142480	VAAVTEKLRAGT	Oryctolagus cuniculus
1	1.00	1	1	0	0	0	0	-0.001	t	144654	GPHPTLEVVPMGRGS	Mus musculus
1	1.00	1	1	0	0	0	0	-0.001	t	144675	GTHPFLSQW	Mus musculus
1	1.00	1	1	0	0	0	0	-0.001	t	139475	ATPRAHEVSE	Mus musculus
1	1.00	1	1	0	0	0	0	-0.001	t	153379	EQEIFTNITDRV	Mus musculus
1	1.00	1	1	0	0	0	0	-0.001	t	144673	GTFCGGEECMRG	Mus musculus
1	1.00	1	1	0	0	0	0	-0.001	t	144763	RIEDPPFNSLL	Mus musculus
1	1.00	1	1	0	0	0	0	-0.001	t	144712	KSLSSTEFIPN	Mus musculus
1	1.00	1	1	0	0	0	0	-0.001	t	159296	SFSKPSNSWFPVEGP	Oryctolagus cuniculus
1	1.00	1	1	0	0	0	0	-0.001	t	153379	EQEIFTNITDRV	Mus musculus

1	1.00	1	1	0	0	0	0	-0.001	t	141786	YGEESSRRGDLAALARRV	Mus musculus
1	1.00	1	1	0	0	0	0	-0.001	t	144598	CFVGTHPQLRC	Mus musculus
1	1.00	1	1	0	0	0	0	-0.001	t	158838	IHNDVEAWMDRYKYYP	Mus musculus
1	1.00	1	1	0	0	0	0	-0.001	t	135414	AAAYKAA	Mus musculus
1	1.00	1	1	0	0	0	0	-0.001	t	144779	SGTHWFLAGCIS	Mus musculus
1	1.00	1	1	0	0	0	0	-0.001	t	159026	NLAIHSDLSYWIESRL	Mus musculus
1	1.00	1	1	0	0	0	0	-0.001	t	141963	MVDRGWG	Mus musculus
1	1.00	1	1	0	0	0	0	-0.001	t	134411	IPP	Mus musculus
1	1.00	1	1	0	0	0	0	-0.001	t	138886	NVGGDNVDIHSIVPVGQDPH	Mus musculus
1	1.00	1	1	0	0	0	0	-0.001	t	144705	KGTHSLYSQIAG	Mus musculus
1	1.00	1	1	0	0	0	0	-0.001	t	144809	VLAPGGTHSRGLLEM	Mus musculus
1	1.00	1	1	0	0	0	0	-0.001	t	144676	GTHPSLQFT	Mus musculus
1	1.00	1	1	0	0	0	0	-0.001	t	150204	FHSVVHTLLEWLMEKELMV	Mus musculus
1	1.00	1	1	0	0	0	0	-0.001	t	159173	TTESGKLITDWCCRSC	Mus musculus
1	1.00	1	1	0	0	0	0	-0.001	t	159288	LLHYSTISQDNLDISFGELQ	Aotus

1	1.00	1	1	0	0	0	0	-0.001	t	144685	HVGTHPM LQPSF	Mus musculu s
1	1.00	1	1	0	0	0	0	-0.001	t	141683	KDWG	Mus musculu s
1	1.00	1	1	0	0	0	0	-0.001	t	148632	IDSEEELIN KKR	Mus musculu s
1	1.00	1	1	0	0	0	0	-0.001	t	144873	ATWVDLV LEGDSCLTI	Mus musculu s
1	1.00	1	1	0	0	0	0	-0.001	t	135799	YGKDVKDL FDYAQE	Mus musculu s
1	1.00	1	1	0	-0.003	0	0	-0.001	t	142441	LFSPWLRV QNHF	Oryctola gus cuniculu s
1	1.00	1	1	0	0	0	0	-0.001	t	144622	EDVGSNK	Mus musculu s
1	1.00	1	1	0	0	0	0	-0.001	t	159085	RYKYYPET PQGLAKII	Mus musculu s
1	1.00	1	1	0	0	0	0	-0.001	t	141523	ALEPPHNL PVP	Mus musculu s
1	1.00	1	1	0	0	0	0	-0.001	t	138765	EEIKMDIS KLNA	Mus musculu s
1	1.00	1	1	0	0	0	0	-0.001	t	159284	FSLRLMEE NWNAEKR S	Oryctola gus cuniculu s
1	1.00	1	1	0	0	0	0	-0.001	t	138893	RHDSGYEV H	Mus musculu s
1	1.00	1	1	0	0	0	0	-0.001	t	156601	ADEPNNL NSK	Oryctola gus cuniculu s
1	1.00	1	1	0	0	0	0	-0.001	t	157189	EFRHDSGY	Mus musculu s
1	1.00	1	1	0	0	0	0	-0.001	t	139525	ISSQTNDT HKRDTYA ATPRA	Mus musculu s

1	1.00	1	1	0	0	0	0	-0.001	t	144799	TLNLT	Mus musculus
1	1.00	1	1	0	0	0	0	-0.001	t	144797	TGGCTHGCLAVP	Mus musculus
1	1.00	1	1	0	0	0	0	-0.001	t	144756	QVGTHPGLANDYEYT	Mus musculus
1	1.00	1	1	0	0	0	0	-0.001	t	142435	KTDGKGEESE	Homo sapiens
1	1.00	1	1	0	0	0	0	-0.001	t	134860	AGPKAQVEAIQKY	Mus musculus
1	1.00	1	1	0	0	0	0	-0.001	t	144601	CSGTHPSISSC	Mus musculus
1	1.00	1	1	0	0	0	0	-0.001	t	159302	YVLNSKLHNSRSFDKFKWIQ	Aotus
1	1.00	1	1	0	0	0	0	-0.001	t	139935	VIAIALFGTIATANAAD	Homo sapiens
1	1.00	1	1	0	0	0	0	-0.001	t	138527	NYEVAYRGILQR	Oryctolagus cuniculus
1	1.00	1	1	0	0	0	0	-0.001	t	150858	ISSLYNLVKSS	Mus musculus
1	1.00	1	1	0	0	0	0	-0.001	t	138194	IIPKSSWS	Homo sapiens
1	1.00	1	1	0	0	0	0	-0.001	t	122205	VFTLPPPLPETH	Mus musculus
1	1.00	1	1	0	0	0	0	-0.001	t	122293	CTYVLDPENLTLKAPYEA	Oryctolagus cuniculus
1	1.00	1	1	0	0	0	0	-0.001	t	132849	KKVLGEKTENPKKFK	Oryctolagus cuniculus
1	1.00	1	1	0	0	0	0	-0.001	t	123849	WQDE	Mus musculus

1	1.00	1	1	0	0	0	0	-0.001	t	118877	LDVEQLGI PEQEYSC	Oryctolagus cuniculus
1	1.00	1	1	0	0	0	0	-0.001	t	132200	CGNRGPSL RTTTVTG	Mus musculus
1	1.00	1	1	0	0	0	0	-0.001	t	118362	FTQLNPTD GGSA	Mus musculus
1	1.00	1	1	0	0	0	0	-0.001	t	123540	NIFEYEVD AEPLRPCE LER	Mus musculus
1	1.00	1	1	0	0	0	0	-0.001	t	117773	CNSRQTD REDELI	Rattus norvegicus
1	1.00	1	1	0	0	0	0	-0.001	t	123562	NSSSSQFQ IHGPR	Mus musculus
1	1.00	1	1	0	0	0	0	-0.001	t	118391	GVDQHDN GSRAR	Mus musculus
1	1.00	1	1	0	0	0	0	-0.001	t	122167	PPPITQGE CKQL	Mus musculus
1	1.00	1	1	0	0	0	0	-0.001	t	131297	FSLRLMEE NWNAEKR SPTF	Callithrix pygmaea
1	1.00	1	1	0	0	0	0	-0.001	t	118740	AKEASSYD YILGWEFG GG	Mus musculus
1	1.00	1	1	0	0	0	0	-0.001	t	125400	ELLKLLIEA	Mus musculus
1	1.00	1	1	0	0	0	0	-0.001	t	122406	IEIGLEGKG FEPTLEALF GK	Homo sapiens
1	1.00	1	1	0	0	0	0	-0.001	t	127643	ERPLELRV RVLDI	Homo sapiens
1	1.00	1	1	0	0	0	0	-0.001	t	123562	NSSSSQFQ IHGPR	Mus musculus
1	1.00	1	1	0	0	0	0	-0.001	t	119383	VISCAKDA SGEEDEI	Oryctolagus cuniculus

1	1.00	1	1	0	0	0	0	-0.001	t	132660	SKAVHADMGYWIESE	Mus musculus
1	1.00	1	1	0	0	0	0	-0.001	t	118141	SKGRRFKG	Mus musculus
1	1.00	1	1	0	0	0	0	-0.001	t	125063	GDRAVYENELVA	Mus musculus
1	1.00	1	1	0	0	0	0	-0.001	t	131576	NSRQTDREDELI	Rattus norvegicus
1	1.00	1	1	0	0	0	0	-0.001	t	131654	SGFGVVKKPNY	Oryctolagus cuniculus
1	1.00	1	1	0	0	0	0	-0.001	t	118867	KLSQTSNVDKEEEAV	Mus musculus
1	1.00	1	1	0	0	0	0	-0.001	t	124232	GSITRDSIFRLR	Oryctolagus cuniculus
1	1.00	1	1	0	0	0	0	-0.001	t	122721	CSNSSSSQFQIHGPR	Mus musculus
1	1.00	1	1	0	0	0	0	-0.001	t	131500	KAKAQTDREDLRTLLR	Rattus norvegicus
1	1.00	1	1	0	0	0	0	-0.001	t	125454	KDYKTMVKFCN	Mus musculus
1	1.00	1	1	0	0	0	0	-0.001	t	125529	PVEGPAVICRCC	Oryctolagus cuniculus
1	1.00	1	1	0	0.002	0	0	-0.001	t	131576	NSRQTDREDELI	Rattus norvegicus
1	1.00	1	1	0	0	0	0	-0.001	t	122083	DPNIKLVLDDCWATS	Oryctolagus cuniculus
1	1.00	1	1	0	0	0	0	-0.001	t	122291	CTYILDPEKLTLRATYDN	Oryctolagus cuniculus

1	1.00	1	1	0	0	0	0	-0.001	t	125870	EEEEEEEEE	Mus musculus
1	1.00	1	1	0	0	0	0	-0.001	t	125367	ASSAFKAPRPGPETLQFT	Oryctolagus cuniculus
1	1.00	1	1	0	0	0	0	-0.001	t	118898	LSHIGDAVVISCAKD	Oryctolagus cuniculus
1	1.00	1	1	0	0	0	0	-0.001	t	118678	WPLHDGGAVNRR	Mus musculus
1	1.00	1	1	0	0	0	0	-0.001	t	125870	EEEEEEEEE	Mus musculus
1	1.00	1	1	0	0	0	0	-0.001	t	123809	VAVESRNGSRLHFNK	Trichosurus vulpecula
1	1.00	1	1	0	0	0	0	-0.001	t	125608	YYKKHIS	Mus musculus
1	1.00	1	1	0	-0.008	0	0	-0.001	t	125246	RRQPHVMS	Mus musculus
1	1.00	1	1	0	0	0	0	-0.001	t	125246	RRQPHVMS	Mus musculus
1	1.00	1	1	0	0	0	0	-0.001	t	123880	YRVNLKFLPNETTSN	Trichosurus vulpecula
1	1.00	1	1	0	0	0	0	-0.001	t	125209	QFQIHGPR	Mus musculus
1	1.00	1	1	0	0	0	0	-0.001	t	118991	RAEDNADTLALVFEA	Oryctolagus cuniculus
1	1.00	1	1	0	0	0	0	-0.001	t	122375	GLINRAKAVKQFEESQG	Mus musculus
1	1.00	1	1	0	0	0	0	-0.001	t	122292	CTYILDPEKLTLRVPYKA	Oryctolagus cuniculus
1	1.00	1	1	0	0	0	0	-0.001	t	122375	GLINRAKAVKQFEESQG	Mus musculus

1	1.00	1	1	0	0	0	0	-0.001	t	125246	RRQPHVMS	Macaca fascicularis
1	1.00	1	1	0	0	0	0	-0.001	t	125575	THDALKAVKQLIKT	Mus musculus
1	1.00	1	1	0	0	0	0	-0.001	t	132233	DSGCVINWKGRELKC	Mus musculus
1	1.00	1	1	0	0	0	0	-0.001	t	122734	DSVPKWDRLPC	Oryctolagus cuniculus
1	1.00	1	1	0	0	0	0	-0.001	t	122721	CSNSSSSQFQIHGPR	Mus musculus
1	1.00	1	1	0	0	0	0	-0.001	t	125414	FSEEKLVFSLRLMEEN	Oryctolagus cuniculus
1	1.00	1	1	0	0	0	0	-0.001	t	120598	IARFKMFPEVKEKGMA	Oryctolagus cuniculus
1	1.00	1	1	0	0	0	0	-0.001	t	125021	DWC	Mus musculus
1	1.00	1	1	0	0	0	0	-0.001	t	124069	ELQIAKDERYGS	Oryctolagus cuniculus
1	1.00	1	1	0	0	0	0	-0.001	t	122293	CTYVLDPENLTLKAPYEA	Mus musculus
1	1.00	1	1	0	0	0	0	-0.001	t	132390	ISNELNHILLENDMK	Mus musculus
1	1.00	1	1	0	0	0	0	-0.001	t	131295	ECQEATLMVMVSKDLFGTGK	Callithrix pygmaea
1	1.00	1	1	0	0	0	0	-0.001	t	130524	KFHPKDIPYQVW	Mus musculus
1	1.00	1	1	0	0	0	0	-0.001	t	119048	VSDYEMKLMDLDVEQ	Mus musculus
1	1.00	1	1	0	0	0	0	-0.001	t	124713	SGEQPSDEQPSGEHG	Mus musculus

1	1.00	1	1	0	0	0	0	-0.001	t	121065	WLKVKILPEVKEKHEF	Oryctolagus cuniculus
1	1.00	1	1	0	0	0	0	-0.001	t	132365	HKYSWKSWGKAKIIG	Mus musculus
1	1.00	1	1	0	0	0	0	-0.001	t	119208	IEDEEGSGIPEQEYSC	Oryctolagus cuniculus
1	1.00	1	1	0	0	0	0	-0.001	t	117569	LKYYLAPKIEDEEGS	Oryctolagus cuniculus
1	1.00	1	1	0	0	0	0	-0.001	t	122202	TSPPLLWDSVHL	Mus musculus
1	1.00	1	1	0	0	0	0	-0.001	t	132727	VTNEVHTWTEQYKFQ	Mus musculus
1	1.00	1	1	0	0	0	0	-0.001	t	118830	GWEFGGG	Mus musculus
1	1.00	1	1	0	0	0	0	-0.001	t	123016	AIYENEIHALWADLP	Trichosurus vulpecula
1	1.00	1	1	0	0	0	0	-0.001	t	118902	LVFEAPNQEKVSDYE	Oryctolagus cuniculus
1	1.00	1	1	0	0	0	0	-0.001	t	131374	CKAKAQTDREDLRTLLRY	Rattus norvegicus
1	1.00	1	1	0	0	0	0	-0.001	t	122947	WTQEPQPLEEKWQHRVVE	Homo sapiens
1	1.00	1	1	0	0	0	0	-0.001	t	131687	SYPDNSYQQPYGE	Oryctolagus cuniculus
1	1.00	1	1	0	0	0	0	-0.001	t	122452	KTTKQSFDLSVKAQYKKNKH	Homo sapiens
1	1.00	1	1	0	0	0	0	-0.001	t	123734	SFSKASNSWFPVEGPADICQ	marmosets

1	1.00	1	1	0	0	0	0	-0.001	t	125263	SSSSQFQIHG	Mus musculus
1	1.00	1	1	0	0	0	0	-0.001	t	125185	NSSSSQFQIHG	Mus musculus
1	1.00	1	1	0	0	0	0	-0.001	t	130524	KFHPKDIPYQVW	Mus musculus
1	1.00	1	1	0	0	0	0	-0.001	t	121529	LDAPGGMELSDTNRALQL	Mus musculus
1	1.00	1	1	0	0	0	0	-0.001	t	122293	CTYVLDPENLTLKAPYEA	Oryctolagus cuniculus
1	1.00	1	1	0	0	0	0	-0.001	t	122293	CTYVLDPENLTLKAPYEA	Oryctolagus cuniculus
1	1.00	1	1	0	0	0	0	-0.001	t	102344	DAEFR	Mus musculus
1	1.00	1	1	0	0	0	0	-0.001	t	102344	DAEFR	Mus musculus
1	1.00	1	1	0	0	0	0	-0.001	t	112816	PGKVCGSNLLSICKTAEF	Mus musculus
1	1.00	1	1	0	0	0	0	-0.001	t	101983	PVYQQQP	Mus musculus
1	1.00	1	1	0	0	0	0	-0.001	t	102344	DAEFR	Mus musculus
1	1.00	1	1	0	0	0	0	-0.001	t	102510	HHQKLVFFAEDVGSNK	Mus musculus
1	1.00	1	1	0	0	0	0	-0.001	t	102344	DAEFR	Mus musculus
1	1.00	1	1	0	0	0	0	-0.001	t	111915	VHTGFGAMDFTTLQANKSEV	Cavia porcellus
1	1.00	1	1	0	0	0	0	-0.001	t	110980	QPLGVGISGHPLLNKLDDTE	Cavia porcellus
1	1.00	1	1	0	0	0	0	-0.001	t	100437	QPNNKRNQLWLRLQTAGN	Mus musculus

1	1.00	1	1	0	0	0	0	-0.001	t	99134	DAEFRHDS GYEVHHQ	Mus musculu s
1	1.00	1	1	0	0	0	0	-0.001	t	99134	DAEFRHDS GYEVHHQ	Mus musculu s
1	1.00	1	1	0	0	0	0	-0.001	t	109277	GVNH	Mus musculu s
1	1.00	1	1	0	0	0	0	-0.001	t	105232	GSNKGAII GLM	Mus musculu s
1	1.00	1	1	0	0	0	0	-0.001	t	104851	KGAIIGLM VGGVVI	Mus musculu s
1	1.00	1	1	0	0	0	0	-0.001	t	105859	HARGNYD AAKR	Mus musculu s
1	1.00	1	1	0	0	0	0	-0.001	t	110920	KPSPWAP KKHRRLS	Mus musculu s
1	1.00	1	1	0	0	0	0	-0.001	t	108848	DEIDGVN H	Mus musculu s
1	1.00	1	1	0	0	0	0	-0.001	t	99134	DAEFRHDS GYEVHHQ	Mus musculu s
1	1.00	1	1	0	0	0	0	-0.001	t	111051	VGPLEVIP EAVDPAG SSIV	Oryctola gus cuniculu s
1	1.00	1	1	0	0	0	0	-0.001	t	112739	YTTGAVR QIFGDYKT TICGK	Oryctola gus cuniculu s
1	1.00	1	1	0	0	0	0	-0.001	t	102069	SKSSITITN KRLTRKC	Mus musculu s
1	1.00	1	1	0	0	0	0	-0.001	t	99134	DAEFRHDS GYEVHHQ	Mus musculu s
1	1.00	1	1	0	0	0	0	-0.001	t	101554	DPLAQQS	Mus musculu s
1	1.00	1	1	0	0	0	0	-0.001	t	106331	DFMLTQP HSVSESPG KTVT	Oryctola gus cuniculu s
1	1.00	1	1	0	0	0	0	-0.001	t	101490	ASGEVPVK PSEG	Mus musculu s

1	1.00	1	1	0	0	0	0	-0.001	t	105232	GSNKGAII GLM	Mus musculus
1	1.00	1	1	0	-0.003	0	0	-0.001	t	103839	DAEFRHDS GYEVH	Oryctolagus cuniculus
1	1.00	1	1	0	0	0	0	-0.001	t	99134	DAEFRHDS GYEVHHQ	Mus musculus
1	1.00	1	1	0	0	0	0	-0.001	t	104443	DAEFRHDS GYE	Mus musculus
1	1.00	1	1	0	0	0	0	-0.001	t	112603	MIAATYNF AVLKLMG RGTKF	Oryctolagus cuniculus
1	1.00	1	1	0	0	0	0	-0.001	t	112352	TGNKYPGY P	Mus musculus
1	1.00	1	1	0	0	0	0	-0.001	t	110652	LCLIGCKPP IGEHWGK GSPC	Cavia porcellus
1	1.00	1	1	0	0	0	0	-0.001	t	105971	MVGGVVI AT	Mus musculus
1	1.00	1	1	0	0	0	0	-0.001	t	109325	IDGVNH	Mus musculus
1	1.00	1	1	0	0	0	0	-0.001	t	104443	DAEFRHDS GYE	Mus musculus
1	1.00	1	1	0	0	0	0	-0.001	t	108382	GGVVIA	Mus musculus
1	1.00	1	1	0	0	0	0	-0.001	t	99134	DAEFRHDS GYEVHHQ	Mus musculus
1	1.00	1	1	0	0	0	0	-0.001	t	108382	GGVVIA	Mus musculus
1	1.00	1	1	0	0	0	0	-0.001	t	108382	GGVVIA	Mus musculus
1	1.00	1	1	0	0	0	0	-0.001	t	108378	GFAGQAG PAGATGA PGRP	Mus musculus
1	1.00	1	1	0	0	0	0	-0.001	t	99134	DAEFRHDS GYEVHHQ	Mus musculus

1	1.00	1	1	0	0	0	0	-0.001	t	103839	DAEFRHDSGYEVH	Mus musculus
1	1.00	1	1	0	0	0	0	-0.001	t	113539	IIPKVEGKTIAEQILQYGSM	Cavia porcellus
1	1.00	1	1	0	0	0	0	-0.001	t	111050	VGPLDIVPEVADPGGPTLV	Oryctolagus cuniculus
1	1.00	1	1	0	0	0	0	-0.001	t	104443	DAEFRHDSGYE	Mus musculus
1	1.00	1	1	0	0	0	0	-0.001	t	112625	PSEATVYLPPVPVSKVVSTD	Cavia porcellus
1	1.00	1	1	0	0	0	0	-0.001	t	108897	EIDGPAGQAEP	Mus musculus
1	1.00	1	1	0	0	0	0	-0.001	t	104906	MVGGVVIA	Mus musculus
1	1.00	1	1	0	0	0	0	-0.001	t	110126	YIK	Mus musculus
1	1.00	1	1	0	0	0	0	-0.001	t	107107	EAIQP	Rattus norvegicus
1	1.00	1	1	0	0	0	0	-0.001	t	110855	FNRAGTVGENVPDDLYIKGS	Cavia porcellus
1	1.00	1	1	0	0	0	0	-0.001	t	104906	MVGGVVIA	Mus musculus
1	1.00	1	1	0	0	0	0	-0.001	t	102093	STGSSDV	Mus musculus
1	1.00	1	1	0	0	0	0	-0.001	t	108898	EIDGPAGQAEPDRAHYN	Mus musculus
1	1.00	1	1	0	0	0	0	-0.001	t	105763	EVHHQK	Mus musculus
1	1.00	1	1	0	0	0	0	-0.001	t	112316	SIKNPTNAK	Mus musculus
1	1.00	1	1	0	0	0	0	-0.001	t	108900	EIPVDLL	Mus musculus

1	1.00	1	1	0	0	0	0	-0.001	t	107852	GEPQSSIIQY	Mus musculus
1	1.00	1	1	0	-0.003	0	0	-0.001	t	105232	GSNKGAIIGLM	Oryctolagus cuniculus
1	1.00	1	1	0	0	0	0	-0.001	t	105497	RHDSGYE	Mus musculus
1	1.00	1	1	0	0	0	0	-0.001	t	103998	IVKWDRDM	Mus musculus
1	1.00	1	1	0	0	0	0	-0.001	t	99922	DAEFRHDSGY	Mus musculus
1	1.00	1	1	0	0	0	0	-0.001	t	112726	YFPTPSGSMVTSDAQIFNKP	Cavia porcellus
1	1.00	1	1	0	0	0	0	-0.001	t	99922	DAEFRHDSGY	Mus musculus
1	1.00	1	1	0	0	0	0	-0.001	t	101716	HQPTPAA	Mus musculus
1	1.00	1	1	0	-0.003	0	0	-0.001	t	105232	GSNKGAIIGLM	Oryctolagus cuniculus
1	1.00	1	1	0	0	0	0	-0.001	t	102005	QPDQHHQ	Mus musculus
1	1.00	1	1	0	0	0	0	-0.001	t	108860	DGVNH	Mus musculus
1	1.00	1	1	0	0	0	0	-0.001	t	99922	DAEFRHDSGY	Mus musculus
1	1.00	1	1	0	0	0	0	-0.001	t	109348	IPVDL	Mus musculus
1	1.00	1	1	0	0	0	0	-0.001	t	100279	LNP	Mus musculus
1	1.00	1	1	0	0	0	0	-0.001	t	114011	VTTVTTHNNPTFTDPSVLQP	Cavia porcellus
1	1.00	1	1	0	0	0	0	-0.001	t	100655	YHSRYFT	Mus musculus

1	1.00	1	1	0	0	0	0	-0.001	t	99922	DAEFRHDS GY	Mus musculu s
1	1.00	1	1	0	0	0	0	-0.001	t	99134	DAEFRHDS GYEVHHQ	Mus musculu s
1	1.00	1	1	0	0	0	0	-0.001	t	109624	NHQH	Mus musculu s
1	1.00	1	1	0	0	0	0	-0.001	t	101962	PEPVQEP	Mus musculu s
1	1.00	1	1	0	0	0	0	-0.001	t	106341	DIQMTQS PSSLSASV GDRV	Oryctola gus cuniculu s
1	1.00	1	1	0	0	0	0	-0.001	t	104443	DAEFRHDS GYE	Mus musculu s
1	1.00	1	1	0	0	0	0	-0.001	t	107166	GPPGPQG	Mus musculu s
1	1.00	1	1	0	0	0	0	-0.001	t	104443	DAEFRHDS GYE	Mus musculu s
1	1.00	1	1	0	0	0	0	-0.001	t	104586	NRVAPNV RQ	Homo sapiens
1	1.00	1	1	0	0	0	0	-0.001	t	105127	DAEFRHD	Mus musculu s
1	1.00	1	1	0	0	0	0	-0.001	t	106321	DAEFRH	Mus musculu s
1	1.00	1	1	0	0	0	0	-0.001	t	107181	IDVEF	Rattus norvegic us
1	1.00	1	1	0	0	0	0	-0.001	t	101546	DLNPLDPL AQQS	Mus musculu s
1	1.00	1	1	0	0	0	0	-0.001	t	107269	YKVIHAWL I	Mus musculu s
1	1.00	1	1	0	0	0	0	-0.001	t	108861	DGVNHQ	Mus musculu s
1	1.00	1	1	0	0	0	0	-0.001	t	105207	GAIIGLMV GGVVIA	Mus musculu s
1	1.00	1	1	0	0	0	0	-0.001	t	112702	VHTGFGA MDFTTLQ A	Cavia porcellu s

1	1.00	1	1	0	0	0	0	-0.001	t	105570	VGGVVIA	Mus musculus
1	1.00	1	1	0	0	0	0	-0.001	t	100070	GLMVGGVVIA	Oryctolagus cuniculus
1	1.00	1	1	0	0	0	0	-0.001	t	108862	DGVNHQHL	Mus musculus
1	1.00	1	1	0	0	0	0	-0.001	t	105127	DAEFRHD	Mus musculus
1	1.00	1	1	0	0	0	0	-0.001	t	111585	NKSEVPLDICTSICKYPDYI	Cavia porcellus
1	1.00	1	1	0	-0.003	0	0	-0.001	t	111050	VGPLDIVPEVADPGGPTLV	Oryctolagus cuniculus
1	1.00	1	1	0	0	0	0	-0.001	t	110762	AGSSIVPLEEYPAEIP	Oryctolagus cuniculus
1	1.00	1	1	0	0	0	0	-0.001	t	106321	DAEFRH	Mus musculus
1	1.00	1	1	0	0	0	0	-0.001	t	105127	DAEFRHD	Mus musculus
1	1.00	1	1	0	0	0	0	-0.001	t	109905	SEEEND	Mus musculus
1	1.00	1	1	0	0	0	0	-0.001	t	106321	DAEFRH	Mus musculus
1	1.00	1	1	0	0	0	0	-0.001	t	110862	GGPTLVSLHELPAETP	Oryctolagus cuniculus
1	1.00	1	1	0	0	0	0	-0.001	t	104995	SYSTTAVVTN	Oryctolagus cuniculus
1	1.00	1	1	0	0	0	0	-0.001	t	104443	DAEFRHDSGYE	Mus musculus
1	1.00	1	1	0	0	0	0	-0.001	t	107242	SQTTANPSCPEGT	Rattus norvegicus

1	1.00	1	1	0	0	0	0	-0.001	t	106322	DAEFRHDSG	Mus musculus
1	1.00	1	1	0	0	0	0	-0.001	t	107242	SQTTANPSCPEGT	Rattus norvegicus
1	1.00	1	1	0	0	0	0	-0.001	t	65409	TNRNPQ	Mus musculus
1	1.00	1	1	0	0	0	0	-0.001	t	79806	EKPAKTYDDAAS	Mus musculus
1	1.00	1	1	0	0	0	0	-0.001	t	71285	VTEEIVTEEIVTEEI	Mus musculus
1	1.00	1	1	0	0	0	0	-0.001	t	72006	VYEAADMIM	Homo sapiens
1	1.00	1	1	0	0	0	0	-0.001	t	72485	WGNGCGLFGKGSIDTCAKFA	Equus caballus
1	1.00	1	1	0	0	0	0	-0.001	t	74488	YKPLGALTH	Mus musculus
1	1.00	1	1	0	0	0	0	-0.001	t	71851	VVTHEMAHALG	Oryctolagus cuniculus
1	1.00	1	1	0	0	0	0	-0.001	t	63756	TFTNKLKEKHTDLGK	Mus musculus
1	1.00	1	1	0	0	0	0	-0.001	t	65409	TNRNPQ	Mus musculus
1	1.00	1	1	0	0	0	0	-0.001	t	97177	AELPF	Mus musculus
1	1.00	1	1	0	0	0	0	-0.001	t	67214	TVWRNRETLMEFEEPHATKQ	Equus caballus
1	1.00	1	1	0	0	0	0	-0.001	t	63165	TDHAGSNDNIALLVQ	Homo sapiens
1	1.00	1	1	0	0	0	0	-0.001	t	79356	RAVIWAWMWE	Mus musculus
1	1.00	1	1	0	0	0	0	-0.001	t	69244	VKIPQHMHPLPI	Mus musculus

1	1.00	1	1	0	0	0	0	-0.001	t	74037	YGLFQKEKMVL	Mus musculus
1	1.00	1	1	0	0	0	0	-0.001	t	71284	VTEEI	Homo sapiens
1	1.00	1	1	0	0	0	0	-0.001	t	78627	DPDPPDANDPAPPNAN	Mus musculus
1	1.00	1	1	0	0	0	0	-0.001	t	65409	TNRNPQ	Oryctolagus cuniculus
1	1.00	1	1	0	0	0	0	-0.001	t	61124	SSIVYEAADMIMHT	Mus musculus
1	1.00	1	1	0	0	0	0	-0.001	t	60595	SQWNKPSKPKT	Mus musculus
1	1.00	1	1	0	0	0	0	-0.001	t	62779	TAADTPTATESISPSPP	Mus musculus
1	1.00	1	1	0	0	0	0	-0.001	t	72961	WPQRVVTKKGRTFL	Mus musculus
1	1.00	1	1	0	0	0	0	-0.001	t	72039	VYHGAGSKTL	Homo sapiens
1	1.00	1	1	0	0	0	0	-0.001	t	65421	TNSGINNS	Mus musculus
1	1.00	1	1	0	0	0	0	-0.001	t	71285	VTEEIVTEEIVTEEI	Mus musculus
1	1.00	1	1	0	0	0	0	-0.001	t	65409	TNRNPQ	Mus musculus
1	1.00	1	1	0	0	0	0	-0.001	t	78687	IRVRKRKGSNKKAEDTLED	Mus musculus
1	1.00	1	1	0	0	0	0	-0.001	t	74036	YGLFHKEKMLL	Mus musculus
1	1.00	1	1	0	0	0	0	-0.001	t	62779	TAADTPTATESISPSPP	Mus musculus

1	1.00	1	1	0	0	0	0	-0.001	t	71285	VTEEIVTEEIVTEEI	Mus musculus
1	1.00	1	1	0	0	0	0	-0.001	t	65205	TMGIYFATPEWVALN	Mus musculus
1	1.00	1	1	0	0	0	0	-0.001	t	75441	YPYDVPDYA	Mus musculus
1	1.00	1	1	0	0	0	0	-0.001	t	67875	VCRQGVVDRGWGNGCGLFGK	Equus caballus
1	1.00	1	1	0	0	0	0	-0.001	t	60596	SQWNKPSKPKTN	Oryctolagus cuniculus
1	1.00	1	1	0	0	0	0	-0.001	t	65409	TNRNPQ	Mus musculus
1	1.00	1	1	0	0	0	0	-0.001	t	74009	YGGPANKKNAG	Mus musculus
1	1.00	1	1	0	0	0	0	-0.001	t	71284	VTEEI	Oryctolagus cuniculus
1	1.00	1	1	0	0	0	0	-0.001	t	65205	TMGIYFATPEWVALN	Mus musculus
1	1.00	1	1	0	0	0	0	-0.001	t	61256	SSNYCCELCCNPACTGCY	Mus musculus
1	1.00	1	1	0	0	0	0	-0.001	t	71285	VTEEIVTEEIVTEEI	Mus musculus
1	1.00	1	1	0	0	0	0	-0.001	t	62353	SVTEEIAEEDKSVTEEIAV	Homo sapiens
1	1.00	1	1	0	0	0	0	-0.001	t	61800	STNS	Mus musculus
1	1.00	1	1	0	0	0	0	-0.001	t	65000	TLPERVRPPDDWAT	Mus musculus
1	1.00	1	1	0	-0.008	0	0	-0.001	t	65205	TMGIYFATPEWVALN	Mus musculus
1	1.00	1	1	0	0	0	0	-0.001	t	71285	VTEEIVTEEIVTEEI	Mus musculus

1	1.00	1	1	0	0	0	0	-0.001	t	63975	TGTEKLIETYFSKNYQDYEY	Mus musculus
1	1.00	1	1	0	0	0	0	-0.001	t	66075	TRPHGR	Mus musculus
1	1.00	1	1	0	0	0	0	-0.001	t	63077	TCGVGVRVRRRVNAANKKPE	Homo sapiens
1	1.00	1	1	0	0	0	0	-0.001	t	65050	TLRVYK	Homo sapiens
1	1.00	1	1	0	0	0	0	-0.001	t	97484	LVEETSFIDAGAP	Oryctolagus cuniculus
1	1.00	1	1	0	0	0	0	-0.001	t	63077	TCGVGVRVRRRVNAANKKPE	Homo sapiens
1	1.00	1	1	0	0	0	0	-0.001	t	75371	YPNQVYYRPVDQYSN	Mus musculus
1	1.00	1	1	0	0	0	0	-0.001	t	78635	EKIERNNKLKQPPPPPNPN	Mus musculus
1	1.00	1	1	0	0	0	0	-0.001	t	61801	STNSGINN	Mus musculus
1	1.00	1	1	0	0	0	0	-0.001	t	69883	VLYQEFDE	Mus musculus
1	1.00	1	1	0	0	0	0	-0.001	t	68969	VIGHEITHGFD	Oryctolagus cuniculus
1	1.00	1	1	0	0	0	0	-0.001	t	71285	VTEEIVTEEIVTEEI	Mus musculus
1	1.00	1	1	0	0	0	0	-0.001	t	62676	SYPPP	Mus musculus
1	1.00	1	1	0	0	0	0	-0.001	t	62150	SVIALGSQEGALHQALAGAI	Equus caballus
1	1.00	1	1	0	0	0	0	-0.001	t	75791	YSLFQKEKMVL	Mus musculus

1	1.00	1	1	0	0	0	0	-0.001	t	71353	VTHESYQELVKKLEALEDA	Oryctolagus cuniculus
1	1.00	1	1	0	0	0	0	-0.001	t	68042	VDPTGLEPDDHLK	Mus musculus
1	1.00	1	1	0	0	0	0	-0.001	t	64074	THSQWNKPSKPKTNMK	Mus musculus
1	1.00	1	1	0	0	0	0	-0.001	t	72504	WGQGGSH	Mus musculus
1	1.00	1	1	0	0	0	0	-0.001	t	52825	QVYYRPVDQ	Mus musculus
1	1.00	1	1	0	0	0	0	-0.001	t	49928	PVPAPRRKVGSDCGS	Mus musculus
1	1.00	1	1	0	0	0	0	-0.001	t	41537	MFSGGGGPLSPGGKS	Mus musculus
1	1.00	1	1	0	0	0	0	-0.001	t	40739	LYDSYAYYG	Mus musculus
1	1.00	1	1	0	0	0	0	-0.001	t	40729	LYDAAKAYAEQLKENGVINY	Mus musculus
1	1.00	1	1	0	0	0	0	-0.001	t	43246	NANPNANPNANPNANP	Mus musculus
1	1.00	1	1	0	0	0	0	-0.001	t	43246	NANPNANPNANPNANP	Mus musculus
1	1.00	1	1	0	0	0	0	-0.001	t	43246	NANPNANPNANPNANP	Mus musculus
1	1.00	1	1	0	0	0	0	-0.001	t	56699	SAAFYYRV	Mus musculus
1	1.00	1	1	0	0	0	0	-0.001	t	48933	PPTAPPLLPHSNL	Homo sapiens

1	1.00	1	1	0	0	0	0	-0.001	t	50637	QELDCWHYQHYCVTSTKS	MD
1	1.00	1	1	0	0	0	0	-0.001	t	58077	SGHRMAWDMMMNW	Mus musculus
1	1.00	1	1	0	0	0	0	-0.001	t	46317	NTWTTCQSIAFPSK	Mus musculus
1	1.00	1	1	0	0	0	0	-0.001	t	60024	SPDVDLGDISGINAS	Mus musculus
1	1.00	1	1	0	0	0	0	-0.001	t	45420	NPNDPP	Mus musculus
1	1.00	1	1	0	-0.008	0	0	-0.001	t	47730	PGQAEPPQAFIDEV	Mus musculus
1	1.00	1	1	0	0	0	0	-0.001	t	57569	SENHGNYSAQVGASQ	Mus musculus
1	1.00	1	1	0	0	0	0	-0.001	t	57569	SENHGNYSAQVGASQ	Mus musculus
1	1.00	1	1	0	0	0	0	-0.001	t	45416	NPNANPNANPNANPNENPNA	Homo sapiens
1	1.00	1	1	0	0	0	0	-0.001	t	50502	QDPDNTDDNGPQDPDNTD	Mus musculus
1	1.00	1	1	0	0	0	0	-0.001	t	48690	PNPNDP	Mus musculus
1	1.00	1	1	0	0	0	0	-0.001	t	47730	PGQAEPPQAFIDEV	Mus musculus
1	1.00	1	1	0	0	0	0	-0.001	t	40167	LTSGHLKCRVKMEKLQLKGT	Equus caballus
1	1.00	1	1	0	0	0	0	-0.001	t	51233	QKPLK	Mus musculus

1	1.00	1	1	0	0.004	0	0	-0.001	t	54393	RKLWCHHFC	Mus musculus
1	1.00	1	1	0	0	0	0	-0.001	t	54393	RKLWCHHFC	Mus musculus
1	1.00	1	1	0	0	0	0	-0.001	t	60024	SPDVDLGDISGINAS	Mus musculus
1	1.00	1	1	0	0	0	0	-0.001	t	43248	NANPNANPNANPNANPNANP	Mus musculus
1	1.00	1	1	0	0	0	0	-0.001	t	43248	NANPNANPNANPNANPNANP	Mus musculus
1	1.00	1	1	0	0	0	0	-0.001	t	43248	NANPNANPNANPNANPNANP	Mus musculus
1	1.00	1	1	0	0	0	0	-0.001	t	49795	PTTRTDDKLR	Oryctolagus cuniculus
1	1.00	1	1	0	0	0	0	-0.001	t	49450	PSNQDSFQWQ	Mus musculus
1	1.00	1	1	0	0	0	0	-0.001	t	41019	MADPNRFRG	Mus musculus
1	1.00	1	1	0	0	0	0	-0.001	t	41019	MADPNRFRG	Mus musculus
1	1.00	1	1	0	0	0	0	-0.001	t	58388	SHLVEALYLVCGERG	Mus musculus
1	1.00	1	1	0	0	0	0	-0.001	t	51438	QLLSSSKYTIQRSTGDNIDT	Mus musculus
1	1.00	1	1	0	0	0	0	-0.001	t	56610	RYPGQGSPGGNRYPPQG	Mus musculus
1	1.00	1	1	0	0	0	0	-0.001	t	58381	SHLPYIEQGMQLAEQF	Mus musculus

1	1.00	1	1	0	0	0	0	-0.001	t	51775	QNTRNIVPVSIVSR	Oryctolagus cuniculus
1	1.00	1	1	0	0	0	0	-0.001	t	50171	PYSPSQDPYSPSPYD	Homo sapiens
1	1.00	1	1	0	0	0	0	-0.001	t	55799	RSAPRITFG	Homo sapiens
1	1.00	1	1	0	0	0	0	-0.001	t	55036	RNNHPQNTSDSQKEATD	Oryctolagus cuniculus
1	1.00	1	1	0	0	0	0	-0.001	t	49878	PVGTPE	Mus musculus
1	1.00	1	1	0	0	0	0	-0.001	t	59922	SNTFINNA	Oryctolagus cuniculus
1	1.00	1	1	0	0	0	0	-0.001	t	45327	NPDREYDFRD	Oryctolagus cuniculus
1	1.00	1	1	0	0	0	0	-0.001	t	37660	LLPHSNLDHILEPSIPWKSK	Mus musculus
1	1.00	1	1	0	0	0	0	-0.001	t	40332	LVFFAEDV	Mus musculus
1	1.00	1	1	0	0	0	0	-0.001	t	41826	MKHM	Mus musculus
1	1.00	1	1	0	0	0	0	-0.001	t	37660	LLPHSNLDHILEPSIPWKSK	Mus musculus
1	1.00	1	1	0	0	0	0	-0.001	t	55193	RPKPG	Gallus gallus
1	1.00	1	1	0	0	0	0	-0.001	t	47028	PCKT	Mus musculus
1	1.00	1	1	0	0	0	0	-0.001	t	50932	QGPGAPQGPGAPQGPGAPQG	Mus musculus

1	1.00	1	1	0	0	0	0	-0.001	t	43730	NEREDERTLTKEYEDIVLK	Oryctolagus cuniculus
1	1.00	1	1	0	-0.003	0	0	-0.001	t	43730	NEREDERTLTKEYEDIVLK	Oryctolagus cuniculus
1	1.00	1	1	0	0	0	0	-0.001	t	50931	QGPGAPQGPGAPQGPGAP	Mus musculus
1	1.00	1	1	0	0	0	0	-0.001	t	50931	QGPGAPQGPGAPQGPGAP	Mus musculus
1	1.00	1	1	0	0	0	0	-0.001	t	48627	PNEPDDALAMRIIRI	Mus musculus
1	1.00	1	1	0	0	0	0	-0.001	t	50931	QGPGAPQGPGAPQGPGAP	Mus musculus
1	1.00	1	1	0	-0.008	0	0	-0.001	t	60117	SPLLGCIGSTCAEDGN	Mus musculus
1	1.00	1	1	0	0	0	0	-0.001	t	43242	NANPNANPNANP	Mus musculus
1	1.00	1	1	0	0	0	0	-0.001	t	43242	NANPNANPNANP	Mus musculus
1	1.00	1	1	0	0	0	0	-0.001	t	52042	QQINHHWHKSGSSIGKAFTT	Equus caballus
1	1.00	1	1	0	0	0	0	-0.001	t	50930	QGPGAPQGPGAP	Mus musculus
1	1.00	1	1	0	0	0	0	-0.001	t	50930	QGPGAPQGPGAP	Mus musculus
1	1.00	1	1	0	0	0	0	-0.001	t	50930	QGPGAPQGPGAP	Mus musculus
1	1.00	1	1	0	0	0	0	-0.001	t	46899	PAPRRKFQQVKRL	Mus musculus
1	1.00	1	1	0	0	0	0	-0.001	t	43242	NANPNANPNANP	Mus musculus

1	1.00	1	1	0	0	0	0	-0.001	t	53702	RFFRDEKELQ	Mus musculus
1	1.00	1	1	0	0	0	0	-0.001	t	50930	QGPGAPQGPGAP	Mus musculus
1	1.00	1	1	0	0	0	0	-0.001	t	41232	MDIDPYKEFG	Mus musculus
1	1.00	1	1	0	0	0	0	-0.001	t	50930	QGPGAPQGPGAP	Mus musculus
1	1.00	1	1	0	0	0	0	-0.001	t	48620	PNDPPP	Mus musculus
1	1.00	1	1	0	0	0	0	-0.001	t	45252	NNSTNSGI	Mus musculus
1	1.00	1	1	0	0	0	0	-0.001	t	48382	PLKPTKRSFIEDLLF	Homo sapiens
1	1.00	1	1	0	0	0	0	-0.001	t	57644	SETFTNKLKEKHTDLGKEG	Mus musculus
1	1.00	1	1	0	0	0	0	-0.001	t	43242	NANPNANPNANP	Aotus nancymaae
1	1.00	1	1	0	0	0	0	-0.001	t	43242	NANPNANPNANP	Mus musculus
1	1.00	1	1	0	0	0	0	-0.001	t	56502	RYAPACKPL	Homo sapiens
1	1.00	1	1	0	0	0	0	-0.001	t	48858	PPPPNPNDPPPPNPND	Mus musculus
1	1.00	1	1	0	0	0	0	-0.001	t	51483	QLPQGTTLPKGFYAE	Homo sapiens
1	1.00	1	1	0	0	0	0	-0.001	t	50222	QACKGKIQALCEN	Mus musculus
1	1.00	1	1	0	0	0	0	-0.001	t	43242	NANPNANPNANP	Mus musculus

1	1.00	1	1	0	0	0	0	-0.001	t	43242	NANPNANPNANP	Macaca fascicularis
1	1.00	1	1	0	0	0	0	-0.001	t	53681	RFAKYQQQGRLEAR	Oryctolagus cuniculus
1	1.00	1	1	0	0	0	0	-0.001	t	51775	QNTRNIVPVSIVSR	Mus musculus
1	1.00	1	1	0	0	0	0	-0.001	t	43152	NAGGNAGGNAGGNAGGNAGG	Mus musculus
1	1.00	1	1	0	0	0	0	-0.001	t	48618	PNDPNRNVD	Mus musculus
1	1.00	1	1	0	0	0	0	-0.001	t	49961	PVSGPFGPLPSLAFSAVPAD	Mus musculus
1	1.00	1	1	0	0	0	0	-0.001	t	43227	NANDPP	Mus musculus
1	1.00	1	1	0	0	0	0	-0.001	t	51958	QPRKTKPVKSLPERK	Mus musculus
1	1.00	1	1	0	0	0	0	-0.001	t	46026	NSVGANAPNADTIAS	Mus musculus
1	1.00	1	1	0	0	0	0	-0.001	t	46026	NSVGANAPNADTIAS	Mus musculus
1	1.00	1	1	0	0	0	0	-0.001	t	60024	SPDVDLGDISGINAS	Mus musculus
1	1.00	1	1	0	0	0	0	-0.001	t	45202	NNNSTNSG	Mus musculus
1	1.00	1	1	0	0	0	0	-0.001	t	53009	QYNLAFSDRQSAQ	Mus musculus

1	1.00	1	1	0	0	0	0	-0.001	t	43246	NANPNANPNANPNANP	Mus musculus
1	1.00	1	1	0	0	0	0	-0.001	t	56599	RYNLVFSGDSDRICSNRA	Mus musculus
1	1.00	1	1	0	0	0	0	-0.001	t	10868	DYEDRYYRE	Mus musculus
1	1.00	1	1	0	0	0	0	-0.001	t	10868	DYEDRYYRE	Mus musculus
1	1.00	1	1	0	0	0	0	-0.001	t	10868	DYEDRYYRE	Mus musculus
1	1.00	1	1	0	0	0	0	-0.001	t	26372	IHFG	Mus musculus
1	1.00	1	1	0	0	0	0	-0.001	t	11819	EENVEHDA	Mus musculus
1	1.00	1	1	0	0	0	0	-0.001	t	23511	GYYYPSDPNTFYAPPYS	Mus musculus
1	1.00	1	1	0	0	0	0	-0.001	t	23511	GYYYPSDPNTFYAPPYS	Mus musculus
1	1.00	1	1	0	0	0	0	-0.001	t	32719	KPGKIS	Homo sapiens
1	1.00	1	1	0	0	0	0	-0.001	t	10801	DWEDRYYRE	Oryctolagus cuniculus
1	1.00	1	1	0	0	0	0	-0.001	t	18766	GASEDVRKQP	Oryctolagus cuniculus
1	1.00	1	1	0	0	0	0	-0.001	t	19096	GDRADGQPA	Aotus griseimembra
1	1.00	1	1	0	0	0	0	-0.001	t	10639	DVKFPGGGQIVGGVYLLPRR	Mus musculus
1	1.00	1	1	0	0	0	0	-0.001	t	24369	HNDKRADPAFVCRQGVVDRG	Equus caballus

1	1.00	1	1	0	0	0	0	-0.001	t	26375	IHFGSDYE DRYY	Mus musculu s
1	1.00	1	1	0	0	0	0	-0.001	t	10639	DVKFPGG GQIVGGV YLLPRR	Mus musculu s
1	1.00	1	1	0	0	0	0	-0.001	t	11815	EENVEENV EENVEENV	Oryctola gus cuniculu s
1	1.00	1	1	0	0	0	0	-0.001	t	32570	KNNNSTN S	Mus musculu s
1	1.00	1	1	0	0	0	0	-0.001	t	11814	EENVEENV EENV	Oryctola gus cuniculu s
1	1.00	1	1	0	0	0	0	-0.001	t	32571	KNNNSTN SGI	Mus musculu s
1	1.00	1	1	0	0	0	0	-0.001	t	10496	DTTIP	Mus musculu s
1	1.00	1	1	0	0	0	0	-0.001	t	17524	FQQFGRD VSDFTDSV RDPKT	Mus musculu s
1	1.00	1	1	0	0	0	0	-0.001	t	11819	EENVEHD A	Mus musculu s
1	1.00	1	1	0	0	0	0	-0.001	t	20051	GGTHSQW NKP	Mus musculu s
1	1.00	1	1	0	0	0	0	-0.001	t	17135	FNCLGMS NRDFLEGV SGATW	Equus caballus
1	1.00	1	1	0	0	0	0	-0.001	t	18833	GAVVGGL GGYML	Mus musculu s
1	1.00	1	1	0	0	0	0	-0.001	t	14869	EVSEKII	Oryctola gus cuniculu s
1	1.00	1	1	0	0	0	0	-0.001	t	24500	HPSDGKC	Mus musculu s

1	1.00	1	1	0	0	0	0	-0.001	t	19868	GGGWGQPHGGGWGQPH	Mus musculus
1	1.00	1	1	0	0	0	0	-0.001	t	15205	FAKY	Mus musculus
1	1.00	1	1	0	0	0	0	-0.001	t	19097	GDRADGQPAGDRAAGQPA	Mus musculus
1	1.00	1	1	0	-0.003	0	0	-0.001	t	11813	EENVEENV	Oryctolagus cuniculus
1	1.00	1	1	0	-0.003	0	0	-0.001	t	11813	EENVEENV	Oryctolagus cuniculus
1	1.00	1	1	0	0	0	0	-0.001	t	32841	KPPTPPPEPET	Mus musculus
1	1.00	1	1	0	0	0	0	-0.001	t	30180	KDKDKDNTDE	Mus musculus
1	1.00	1	1	0	-0.003	0	0	-0.001	t	11813	EENVEENV	Oryctolagus cuniculus
1	1.00	1	1	0	0	0	0	-0.001	t	21867	GQGGGTHNQWNKP	Oryctolagus cuniculus
1	1.00	1	1	0	0	0	0	-0.001	t	31046	KGQKPNLLDRLRHRKNGYRH	Mus musculus
1	1.00	1	1	0	0	0	0	-0.001	t	16018	FGNDYEDRYYR	Mus musculus
1	1.00	1	1	0	0	0	0	-0.001	t	36166	LGIMGEYRGTPRNQDLYDAA	Mus musculus
1	1.00	1	1	0	0	0	0	-0.001	t	30716	KFDWNTPD	Mus musculus

1	1.00	1	1	0	0	0	0	-0.001	t	11819	EENVEHD A	Mus musculu s
1	1.00	1	1	0	0	0	0	-0.001	t	22305	GSDYEDRY YRENMHR YPNQ	Mus musculu s
1	1.00	1	1	0	0	0	0	-0.001	t	16101	FHENWPS	Mus musculu s
1	1.00	1	1	0	0	0	0	-0.001	t	31018	KGNCSGPT TIR	Oryctola gus cuniculu s
1	1.00	1	1	0	-0.003	0	0	-0.001	t	30716	KFDWNTP D	Mus musculu s
1	1.00	1	1	0	-0.008	0	0	-0.001	t	16551	FLDLMRA VA	Mus musculu s
1	1.00	1	1	0	0	0	0	-0.001	t	11813	EENVEENV	Homo sapiens
1	1.00	1	1	0	0	0	0	-0.001	t	30716	KFDWNTP D	Mus musculu s
1	1.00	1	1	0	0	0	0	-0.001	t	11819	EENVEHD A	Oryctola gus cuniculu s
1	1.00	1	1	0	0	0	0	-0.001	t	23599	HCLGKWL GHPDKF	Mus musculu s
1	1.00	1	1	0	0	0	0	-0.001	t	27463	IMDINKRK YDSLK	Homo sapiens
1	1.00	1	1	0	0	0	0	-0.001	t	12982	ELDGSKNE WGWSKSK	Mus musculu s
1	1.00	1	1	0	0	0	0	-0.001	t	30186	KDKKKKTD EAQPLPQR QKKQ	Homo sapiens

1	1.00	1	1	0	0	0	0	-0.001	t	11241	ECFPWDRKL	Mus musculus
1	1.00	1	1	0	0.004	0	0	-0.001	t	11241	ECFPWDRKL	Mus musculus
1	1.00	1	1	0	0	0	0	-0.001	t	27675	INKRKYDSLKEKL	Homo sapiens
1	1.00	1	1	0	0	0	0	-0.001	t	14952	EWSPCSVTCGKGTRSRKR	Oryctolagus cuniculus
1	1.00	1	1	0	0	0	0	-0.001	t	11819	EENVEHDA	Mus musculus
1	1.00	1	1	0	0	0	0	-0.001	t	10358	DTIASGSQRSTNSAS	Mus musculus
1	1.00	1	1	0	0	0	0	-0.001	t	19097	GDRADGQPAGDRAAGQPA	Mus musculus
1	1.00	1	1	0	0	0	0	-0.001	t	19093	GDRAAGQPAGDRAAGQPA	Mus musculus
1	1.00	1	1	0	0	0	0	-0.001	t	26075	IFGFNALVDR	Oryctolagus cuniculus
1	1.00	1	1	0	0	0	0	-0.001	t	13984	ERESQAY	Mus musculus
1	1.00	1	1	0	0	0	0	-0.001	t	25149	HYAPRPCGI	Homo sapiens
1	1.00	1	1	0	0	0	0	-0.001	t	19093	GDRAAGQPAGDRAAGQPA	Mus musculus
1	1.00	1	1	0	0	0	0	-0.001	t	19093	GDRAAGQPAGDRAAGQPA	Mus musculus
1	1.00	1	1	0	0	0	0	-0.001	t	11819	EENVEHDA	Mus musculus

1	1.00	1	1	0	0	0	0	-0.001	t	19093	GDRAAGQPAGDRAAGQPA	Mus musculus
1	1.00	1	1	0	0	0	0	-0.001	t	21469	GNLLLSCL	Mus musculus
1	1.00	1	1	0	0	0	0	-0.001	t	10639	DVKFPGGGQIVGGVYLLPRR	Mus musculus
1	1.00	1	1	0	0	0	0	-0.001	t	21469	GNLLLSCL	Mus musculus
1	1.00	1	1	0	0	0	0	-0.001	t	33795	KTNMKHMAGA	Mus musculus
1	1.00	1	1	0	0	0	0	-0.001	t	4737	ASREAK	Mus musculus
1	1.00	1	1	0	0	0	0	-0.001	t	9723	DPPPPNPNDPPPPNPND	Mus musculus
1	1.00	1	1	0	0	0	0	-0.001	t	4789	ASTAAPPYTNEQAYQMLLAL	Mus musculus
1	1.00	1	1	0	0	0	0	-0.001	t	9610	DPDIGQSK	Mus musculus
1	1.00	1	1	0	0	0	0	-0.001	t	9609	DPDI	Mus musculus
1	1.00	1	1	0	0	0	0	-0.001	t	7491	DAEFRHDSGYEVHHQK	Macaca mulatta
1	1.00	1	1	0	0	0	0	-0.001	t	3290	ANGAGNQPGANGAGNQPG	Mus musculus
1	1.00	1	1	0	0	0	0	-0.001	t	3290	ANGAGNQPGANGAGNQPG	Mus musculus
1	1.00	1	1	0	0	0	0	-0.001	t	9516	DNNLVSGP	Mus musculus
1	1.00	1	1	0	0	0	0	-0.001	t	9516	DNNLVSGP	Mus musculus

1	1.00	1	1	0	0	0	0	-0.001	t	9516	DNNLVSGP	Mus musculus
1	1.00	1	1	0	0	0	0	-0.001	t	8916	DKKTEETTLLEDRIL	Mus musculus
1	1.00	1	1	0	0	0	0	-0.001	t	9524	DNPASTTNKDK	Mus musculus
1	1.00	1	1	0	0	0	0	-0.001	t	9757	DPRVRGLYFPAGGSSSGTV	Pan troglodytes
1	1.00	1	1	0	0	0	0	-0.001	t	9694	DPNANPNVDPNANPNV	Mus musculus
1	1.00	1	1	0	0	0	0	-0.001	t	9694	DPNANPNVDPNANPNV	Mus musculus
1	1.00	1	1	0	0	0	0	-0.001	t	5771	AYFLYQQQGRLDKL	Oryctolagus cuniculus
1	1.00	1	1	0	0	0	0	-0.001	t	9694	DPNANPNVDPNANPNV	Mus musculus
1	1.00	1	1	0	0	0	0	-0.001	t	9524	DNPASTTNKDK	Mus musculus
1	1.00	1	1	0	0	0	0	-0.001	t	7491	DAEFRHDSGYEVHHQK	Mus musculus
1	1.00	1	1	0	0	0	0	-0.001	t	9694	DPNANPNVDPNANPNV	Mus musculus
1	1.00	1	1	0	0	0	0	-0.001	t	9621	DPEDSALL	Mus musculus
1	1.00	1	1	0	0	0	0	-0.001	t	9722	DPPPPNPNDPPPPNPN	Mus musculus
1	1.00	1	1	0	0	0	0	-0.001	t	9694	DPNANPNVDPNANPNV	Mus musculus

1	1.00	1	1	0	0	0	0	-0.001	t	8226	DEYSNQN NFVHDC	Mus musculu s
1	1.00	1	1	0	0	0	0	-0.001	t	5967	CADARMY GVLPWNA FPGKVC	Oryctola gus cuniculu s
1	1.00	1	1	0	0	0	0	-0.001	t	8748	DIPFP	Mus musculu s
1	1.00	1	1	0	0	0	0	-0.001	t	5352	AVGPN	Mus musculu s
1	1.00	1	1	0	0	0	0	-0.001	t	9722	DPPPPNP NDPPPPN PN	Mus musculu s
1	1.00	1	1	0	0	0	0	-0.001	t	6455	CITQYERES QAYY	Mus musculu s
1	1.00	1	1	0	0	0	0	-0.001	t	4397	ASFEAQG ALANIAVD KA	Mus musculu s
1	1.00	1	1	0	0	0	0	-0.001	t	2039	AINPESDQ PDLSNF	Homo sapiens
1	1.00	1	1	0	0	0	0	-0.001	t	2780	ALNGNGD PNNMDKA VKLY	Mus musculu s
1	1.00	1	1	0	0	0	0	-0.001	t	6402	CHPSDGK CN	Mus musculu s
1	1.00	1	1	0	0	0	0	-0.001	t	8076	DELKDQM EED	Mus musculu s
1	1.00	1	1	0	0	0	0	-0.001	t	9722	DPPPPNP NDPPPPN PN	Mus musculu s
1	1.00	1	1	0	0	0	0	-0.001	t	8916	DKKTEETT LLEDRIL	Mus musculu s
1	1.00	1	1	0	0	0	0	-0.001	t	5597	AVVGGLG GY	Mus musculu s
1	1.00	1	1	0	0	0	0	-0.001	t	8916	DKKTEETT LLEDRIL	Mus musculu s

1	1.00	1	1	0	0	0	0	-0.001	t	8644	DIFTNSRG KRASKG	Mus musculu s
1	1.00	1	1	0	0	0	0	-0.001	t	709	ADLIAAQK LASKP	Mus musculu s
1	1.00	1	1	0	0	0	0	-0.001	t	3656	APMSTPS ATSVR	Mus musculu s
1	1.00	1	1	0	0	0	0	-0.001	t	7492	DAEFRHDS GYEVHHQ KL	Mus musculu s
1	1.00	1	1	0	0	0	0	-0.001	t	4397	ASFEAQG ALANIAVD KA	Mus musculu s
1	1.00	1	1	0	0	0	0	-0.001	t	9694	DPNANPN VDPNANP NV	Mus musculu s
1	1.00	1	1	0	0	0	0	-0.001	t	9694	DPNANPN VDPNANP NV	Mus musculu s
1	1.00	1	1	0	0	0	0	-0.001	t	7754	DDDIPF	Mus musculu s
1	1.00	1	1	0	0	0	0	-0.001	t	3242	ANDPPP	Mus musculu s
1	1.00	1	1	0	0	0	0	-0.001	t	9722	DPPPPNP NDPPPPN PN	Mus musculu s
[truncated: 566,242 more chars]
